# PESI - a taxonomic backbone for Europe

**DOI:** 10.3897/BDJ.3.e5848

**Published:** 2015-09-28

**Authors:** Yde de Jong, Juliana Kouwenberg, Louis Boumans, Charles Hussey, Roger Hyam, Nicola Nicolson, Paul Kirk, Alan Paton, Ellinor Michel, Michael D. Guiry, Phillip S. Boegh, Henrik Ærenlund Pedersen, Henrik Enghoff, Eckhard von Raab-Straube, Anton Güntsch, Marc Geoffroy, Andreas Müller, Andreas Kohlbecker, Walter Berendsohn, Ward Appeltans, Christos Arvanitidis, Bart Vanhoorne, Joram Declerck, Leen Vandepitte, Francisco Hernandez, Róisín Nash, Mark John Costello, David Ouvrard, Pascale Bezard-Falgas, Thierry Bourgoin, Florian Tobias Wetzel, Falko Glöckler, Günther Korb, Caroline Ring, Gregor Hagedorn, Christoph Häuser, Nihat Aktaç, Ahmet Asan, Adorian Ardelean, Paulo Alexandre Vieira Borges, Dhimiter Dhora, Hasmik Khachatryan, Michael Malicky, Shaig Ibrahimov, Alexander Tuzikov, Aaike De Wever, Snejana Moncheva, Nikolai Spassov, Karel Chobot, Alexi Popov, Igor Boršić, Spyros Sfenthourakis, Urmas Kõljalg, Pertti Uotila, Gargominy Olivier, Jean-Claude Dauvin, David Tarkhnishvili, Giorgi Chaladze, Michael Tuerkay, Anastasios Legakis, László Peregovits, Gudmundur Gudmundsson, Erling Ólafsson, Liam Lysaght, Bella Sarah Galil, Francesco M. Raimondo, Gianniantonio Domina, Fabio Stoch, Alessandro Minelli, Voldermars Spungis, Eduardas Budrys, Sergej Olenin, Armand Turpel, Tania Walisch, Vladimir Krpach, Marie Therese Gambin, Laurentia Ungureanu, Gordan Karaman, Roy M.J.C. Kleukers, Elisabeth Stur, Kaare Aagaard, Nils Valland, Toril Loennechen Moen, Wieslaw Bogdanowicz, Piotr Tykarski, Jan Marcin Węsławski, Monika Kędra, Antonio M. de Frias Martins, António Domingos Abreu, Ricardo Silva, Sergei Medvedev, Alexander Ryss, Smiljka Šimić, Karol Marhold, Eduard Stloukal, Davorin Tome, Marian A. Ramos, Benito Valdés, Francisco Pina, Sven Kullander, Anders Telenius, Yves Gonseth, Pascal Tschudin, Oleksandra Sergeyeva, Volodymyr Vladymyrov, Volodymyr Bohdanovych Rizun, Chris Raper, Dan Lear, Pavel Stoev, Lyubomir Penev, Ana Casino Rubio, Thierry Backeljau, Hannu Saarenmaa, Sandrine Ulenberg

**Affiliations:** ‡ University of Amsterdam - Faculty of Science, Amsterdam, Netherlands; § University of Eastern Finland, Joensuu, Finland; | Royal Belgian Institute of Natural Sciences, Brussels, Belgium; ¶ Museum für Naturkunde, Leibniz Institute for Evolution and Biodiversity Science, Berlin, Germany; # University of Oslo - Natural History Museum, Oslo, Norway; ¤ The Natural History Museum, London, United Kingdom; « Royal Botanic Garden Edinburgh, Edinburgh, United Kingdom; » Royal Botanic Gardens, Kew, London, United Kingdom; ˄ Natural History Museum, London, United Kingdom; ˅ AlgaeBase c/o Ryan Institute, National University of Ireland, Galway, Ireland; ¦ Danish Meteorological Institute, Copenhagen, Denmark; ˀ Zoological Museum Copenhagen, University of Copenhagen, Copenhagen, Denmark; ˁ Botanic Garden and Botanical Museum Berlin-Dahlem, Freie Universität Berlin, Berlin, Germany; ₵ OBIS, Oostende, Belgium; ℓ Hellenic Centre for Marine Research, Heraklion, Greece; ₰ Flanders Marine Institute (VLIZ), Oostende, Belgium; ₱ Marine and Freshwater Research Centre (MFRC), Galway Mayo Institute of Technology (GMIT), Galway, Ireland; ₳ Ecological Consultancy Services Ltd, Dublin, Ireland; ₴ University of Auckland, Auckland, New Zealand; ₣ Muséum national d’Histoire naturelle, Paris, France; ₮ Muséum national d’Histoire naturelle, Département Systématique & Evolution, UMR 7205 MNHN-CNRS-UPMC-EPHE, (ISyEB), Paris, France; ₦ Trakya University, Edirne, Turkey; ₭ myNature Association, Timișoara, Romania; ₲ CE3C – Centre for Ecology, Evolution and Environmental Changes / Azorean Biodiversity Group and Universidade dos Açores, Angra do Heroísmo, Azores, Portugal; ‽ University of Shkodra, Faculty of Natural Sciences, Shkodra, Albania; ₩ National Academy of Sciences of Armenia, Institute of Zoology, Yerevan, Armenia; ₸ Oberösterreichisches Landesmuseum, Biologiezentrum, Linz, Austria; ‡‡ Institute of Zoology, Baku, Azerbaijan; §§ United Institute of Informatics Problems, National Academy of Sciences of Belarus, Minsk, Belarus; || Institute of Oceanology, Varna, Bulgaria; ¶¶ National Museum of Natural History, Sofia, Bulgaria; ## Nature Conservation Agency of the Czech Republic, Prague, Czech Republic; ¤¤ State Institute for Nature Protection, Zagreb, Croatia; «« University of Cyprus, Nicosia, Cyprus; »» University of Tartu, Tartu, Estonia; ˄˄ University of Helsinki, Helsinki, Finland; ˅˅ Muséum national d'Histoire naturelle, Paris, France; ¦¦ University of Sciences and Technology of Lille, Lille, France; ˀˀ Ilia Chavchavadze State University, Tbilisi, Georgia; ˁˁ Senckenberg Museum, Frankfurt, Germany; ₵₵ National and Kapodistrian University of Athens, Athens, Greece; ℓℓ Hungarian Natural History Museum, Budapest, Hungary; ₰₰ Icelandic Institute of Natural History, Reykjavik, Iceland; ₱₱ National Biodiversity Data Center, Waterford, Ireland; ₳₳ National Institute of Oceanography, Israel Oceanographic and Limnological Research, Haifa, Israel; ₴₴ University Palermo, Botanical Garden and Herbarium Mediterraneum, Palermo, Italy; ₣₣ University of L'Aquila, L'Aquila, Italy; ₮₮ University of Padova, Padova, Italy; ₦₦ University of Latvia, Riga, Latvia; ₭₭ Nature Research Centre, Vilnius, Lithuania; ₲₲ Klaipeda University, Klaipeda, Lithuania; ‽‽ Musée national d'histoire naturelle Luxembourg, Luxembourg, Luxembourg; ₩₩ Macedonian Museum of Natural History, Skopje, Macedonia; ₸₸ Malta Environment & Planning Authority, Floriana, Malta; ‡‡‡ Institute of Zoology of the Academy of Sciences of Moldova, Chişinău, Moldova; §§§ Montenegrin Academy of Sciences and Arts, Podgorica, Montenegro; ||| Naturalis Biodiversity Center, Leiden, Netherlands; ¶¶¶ NTNU University Museum, Norwegian University of Science and Technology, Trondheim, Norway; ### Norwegian Biodiversity Information Centre (Artsdatabanken), Trondheim, Norway; ¤¤¤ Museum and Institute of Zoology, Polish Academy of Sciences, Warsaw, Poland; ««« University of Warsaw, Faculty of Biology, Dept. of Ecology, Biological and Chemical Research Centre, Warsaw, Poland; »»» Institute of Oceanology of Polish Academy of Sciences, Sopot, Poland; ˄˄˄ University of the Azores, Ponta Delgada, Portugal; ˅˅˅ Estação de Biologia Marinha do Funchal, Madeira, Portugal; ¦¦¦ Naturdata, Lisboa, Portugal; ˀˀˀ Zoological Institute of Russian Academy of Sciences, St Petersburg, Russia; ˁˁˁ Centre for the Balkan Biodiversity Conservation, Novi Sad, Serbia; ₵₵₵ Institute of Botany, Slovak Academy of Sciences, Bratislava, Slovakia; ℓℓℓ Department of Botany, Faculty of Science, Charles University, Praha, Czech Republic; ₰₰₰ Comenius University, Bratislava, Slovakia; ₱₱₱ Slovenian National Institute of Biology, Ljubljana, Slovenia; ₳₳₳ Museo Nacional de Ciencias Naturales (CSIC), Madrid, Spain; ₴₴₴ University of Seville, Seville, Spain; ₣₣₣ Swedish Museum of Natural History, Stockholm, Sweden; ₮₮₮ Centre Suisse de Cartographie de la Faune, Neuchâtel, Switzerland; ₦₦₦ Institute of Biology of the Southern Seas, Sevastopol, Ukraine; ₭₭₭ State Museum of Natural History, National Academy of Sciences of Ukraine, Lviv, Ukraine; ₲₲₲ Marine Biological Association of the United Kingdom, Plymouth, United Kingdom; ‽‽‽ Pensoft Publishers, Sofia, Bulgaria; ₩₩₩ Institute of Biodiversity and Ecosystem Research, Sofia, Bulgaria; ₸₸₸ CETAF c/o Royal Belgian Institute of Natural Sciences, Brussels, Belgium

**Keywords:** PESI, Euro+Med PlantBase, Fauna Europaea, European Register of Marine Species, Index Fungorum, International Plant Names Index (IPNI), AlgaeBase, ZooBank, European taxonomic backbone, EU-nomen, Taxonomic indexing, Authority File, Taxonomy, Nomenclature, Global Names Architecture, INSPIRE, LifeWatch, EUBON

## Abstract

**Background:**

Reliable taxonomy underpins communication in all of biology, not least nature conservation and sustainable use of ecosystem resources. The flexibility of taxonomic interpretations, however, presents a serious challenge for end-users of taxonomic concepts. Users need standardised and continuously harmonised taxonomic reference systems, as well as high-quality and complete taxonomic data sets, but these are generally lacking for non-specialists. The solution is in dynamic, expertly curated web-based taxonomic tools.

The Pan-European Species-directories Infrastructure (PESI) worked to solve this key issue by providing a taxonomic e-infrastructure for Europe. It strengthened the relevant social (expertise) and information (standards, data and technical) capacities of five major community networks on taxonomic indexing in Europe, which is essential for proper biodiversity assessment and monitoring activities. The key objectives of PESI were: 1) standardisation in taxonomic reference systems, 2) enhancement of the quality and completeness of taxonomic data sets and 3) creation of integrated access to taxonomic information.

**New information:**

This paper describes the results of PESI and its future prospects, including the involvement in major European biodiversity informatics initiatives and programs.

## Project description

**Figure 1.**
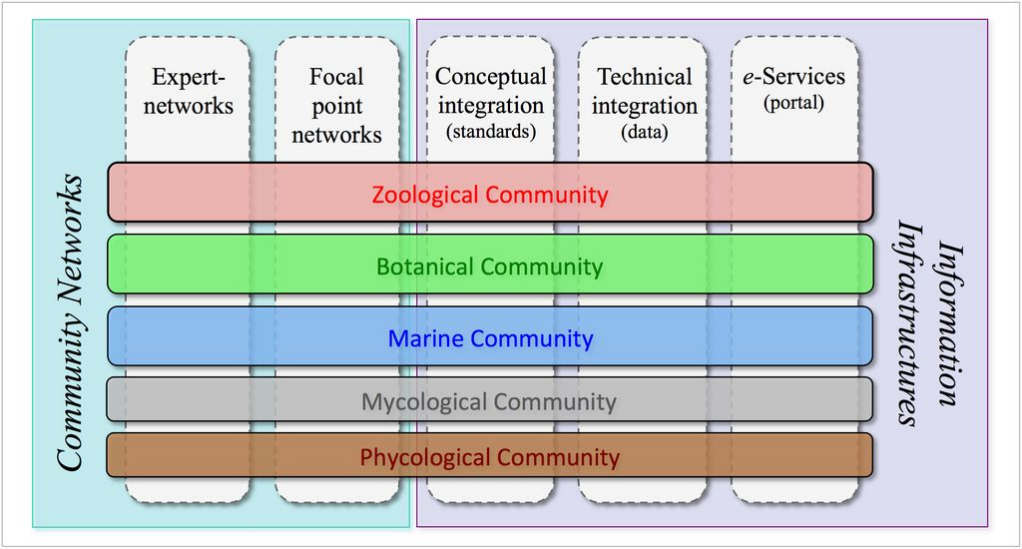
Five community networks (horizontal) are integrated in five categories of coordination effort (vertical) in PESI. Community networks represent the FP4 and FP5 key programs on European taxonomic indexing: Fauna Europaea, ERMS, Euro+Med PlantBase, supplemented by Index Fungorum and AlgaeBase.

The Pan-European Species-directories Infrastructure (PESI) provides a mechanism to deliver an integrated, annotated checklist of the species occurring in 'geographic Europe', aiming to cover the Western Paleartic biogeographic region. The PESI checklist (also called EU-nomen) serves as a taxonomic standard and backbone for Europe. At the core of *EU-nomen* are five community networks, with common nomenclatures or systems designations: Zoology, Botany, Marine Biota, Mycology and Phycology. These five community networks are integrated in five infrastructural components (Fig. 1): expert networks (knowledge), focal point networks (consensus), conceptual integration (standards), technical integration (data) and e-Services (dissemination). The databases from the community networks Euro+Med PlantBase (E+M); Fauna Europaea (FaEu); the European Register of Marine Species (ERMS), and Species Fungorum Europe (SF-EU) - feed into a single data warehouse that supports the PESI Portal, a service webportal developed during the project phase of PESI (May 2008 to May 2011). Surrounding this core, PESI includes the interactions with the geographic focal point networks, a network of taxonomic experts and global species databases.

PESI merges data from multiple sources and publishes it online. This requires a mapping between the different schemas used by the different data sources and/or an implementation of standards within those data sources. A pragmatic approach was taken for the main databases. A bespoke procedure was developed at the Botanic Garden and Botanical Museum Berlin-Dahlem (BGBM) using the EDIT Common Data Model-based Platform for Cybertaxonomy to merge Euro+Med PlantBase and Fauna Europaea. These data are merged with ERMS and additional data provided by these checklists (such as distribution details) in the PESI Data Warehouse. This procedure is repeated periodically to keep the data warehouse synchronised with the source databases. The Flanders Marine Institute (VLIZ), that manages the portal, adds further information (such as images, vernacular names and priority status) and gathers information from other portals, such as the World Register of Marine Species.

In order for the data sets to be merged in this way they need to share common vocabularies for some fields – these include: taxon status, nomenclatural status and occurrence status. PESI provides species lists based on geographic regions and European legislation (e.g. the Habitat Directive, Birds Directive, CITES and IUCN (conservation status) directives). The resulting dataset includes a total of nearly 450,000 scientific names (which include 240,000 valid species and infraspecific names) and 190,800 vernacular names in 117 languages. In the PESI portal, each species has its own result page including a map of its geographical distribution, and many have deep links to additional data (photos, videos, literature, global prioritised species databases). The portal further includes: a Taxon Search Tool, a Taxon Match Tool (for users to match their species names against the names in PESI to check spelling, synonymy and classificaiton), a metadata database on taxonomic resources and expertise (provided by focal point partners all over Europe and adjacent countries), Web Services and links to existing nomenclators. The Project website provides information about the PESI project and the available communication tools.

PESI builds upon previous European taxonomic projects (like EDIT, ENBI and EuroCAT) and strengthens the taxonomic networks to ensure that data are updated and that relevant social networks and research communities are maintained and developed. PESI contributes to building the capacity necessary to support a growing number of international initiatives such as the Global Biodiversity Information Facility (GBIF), LifeWatch (LW), the Catalogue of Life (CoL), the Encyclopedia of Life (EoL), the Global Names Architecture (GNA), GÉANT, and Research Data Alliance (RDA). The project also provides key contributions to relevant ensuing EC-FP7 projects like ViBRANT (on cybertaxonomy), OpenUp! (opening up the natural history heritage for Europeana), BioVeL (on developing virtual labs for biodiversity research), iMarine (on developing an e-Infrastructure for fisheries management and marine conservation), EU BON (on building the European Biodiversity Observation Network, as a European contribution to GEO BON) and BiodiversityKnowledge (on developing a recommended design for a scientific biodiversity Network of Knowledge). At the same time, it provides a robust infrastructure to support the nomenclatural needs of European users and stakeholders on biodiversity management and research. PESI has specifically addressed the issues of pooling resources, expertise networks, standardisation, sustainability, accessibility and international cooperation (Jong 2011).

PESI was initiated during the EC-FP6 European Distributed Institute of Taxonomy Network (EDIT) and Marine Biodiversity and Ecosystem Functioning (MarBEF) EU Networks of Excellence and funded as a project by the European Commission, contributing to the EC-FP7-Infrastructures 'Capacities' subprogram under the Coordination and Support Actions (CSA) funding scheme (RI-223806), starting in May 2008 for a period of three years. PESI is formally accepted as an INSPIRE standard for Europe and adopted in the workplans of various major EU initiatives on biodiversity research, including LifeWatch and EU BON, as the taxonomic backbone for Europe.

### Coordination and integration of European expert networks

The strengthening and integration of European taxonomic communities has been progressing since the start of the taxonomic indexing EU framework programmes Fauna Europaea (Jong et al. 2014), European Register of Marine Species (ERMS) (Costello 2000, Costello 2004, Costello et al. 2001, Cuvelier et al. 2006), and Euro+Med PlantBase (Greuter and Raab-Straube 2005, Raab-Straube and Raus 2013). These initiatives built up expert networks to fulfil the project objectives and played an important role in helping to identify and consolidate the European taxonomic expert community. Other sources of taxonomic expertise are also included, such as the community of the EDIT Network of Excellence (EU FP6). PESI has reviewed and developed long-term strategies and plans for the sustainability of the contributing communities systems in terms of ownership and support of individual experts and institutions.

PESI makes a significant addition to the species registers through the expansion of the network of expertise towards Eastern Europe. In addition to providing more comprehensive data for the pan-European species registers, this expansion has overcome the separation of knowledge and taxonomic practice over decades. Another achievement is the closer collaboration of the taxonomic societies, especially with respect to improving taxonomic coverage and addressing long-term maintenance and upgrading of the taxonomy.

### Coordination and integration of European focal point networks

In addition to creating a network of taxonomic experts, PESI has developed a network of regional (often national) focal points. These focal points (either individuals or organisations) complement the taxonomic network through: (1) liaising with national governmental bodies on the implementation of European standards relevant to, for instance, national and European regulations and environmental monitoring, (2) collecting and transferring local expertise and applied tools, (3) lobbying and public policy assistance at national and European level, and (4) supporting closer collaboration of scientific contributor and user communities across Europe. Focal points contribute country-specific information about species, relevant databases, local literature, experts, professional societies and major users such as government organisations.

### Coordination of taxonomic meta-data standard assessment

In biology, taxon names provide anchors that allow information about organisms to be linked. A taxonomic name, typically a species name, is attached to every primary data object (field observation, specimen, genetic data, etc.). Therefore names, together with their organisation into taxonomic classifications, are understood as core (meta-)data for biological information systems. There are many challenges in integrating data sources that contain taxonomic names and classifications, particularly where the sources extend over different biological kingdoms or national boundaries. Names may be erroneously assigned or incomplete and so searches based on exact character matching against names in current use may fail. Names that are synonyms or old combinations no longer in current use may occur in museum and herbarium specimen catalogues or in legislative lists. Names with orthographical errors occur in legislative lists on national or international level and some of them are often in use by certain taxonomists. There may also be disagreement amongst experts on the identity of specimens and on the taxonomic constituents of genera and the arrangement of classifications. The partners involved in PESI have extensive experience with handling such problems and PESI has produced practical solutions for many of these issues. The availability of authoritative taxonomic metadata standards is of particular relevance where species are directly linked to societal issues such as conservation and environmental control. PESI promotes harmonisation and certification of taxonomic metadata standards of prioritised taxa that are listed in various EU regulations and legislative lists. To address these issues, PESI had the following objectives:

- To prepare a roadmap (conceptual development and strategic plan) for the application of taxonomic standards within Europe, with the purpose of overcoming the instability and inconsistency of taxon names (and concepts) and attached data. This work addressed technical, linguistic, educational and legal barriers to progress in defining and implementing appropriate standards.
- To promote co-operation between PESI and other networks and organisations. This optimised the cross-linking of European biodiversity resources using approved taxonomic data standards, and improved data quality and consistency. This facilitated discovery and exchange of biodiversity data, both within Europe, and between Europe and globally.
- To work closely with relevant standards organisations to identify appropriate authoritative standards and schemes and to ensure their adoption within the European biodiversity community. Work included development of a management classification scheme, utilisation of globally unique identifiers for names (GUIDs) and support for nomenclators (such as ZooBank, International Plants Names Index, Index Fungorum and AlgaeBase) to help implement a practical name resolution service and support the definition of a future architecture for common names, proceeding from existing surveys (e.g. Suppl. material 1).

### Coordination and integration of information e-infrastructures

PESI has technically integrated the pan-European species registers into an e-infrastructure by creating a joint access (middle) layer, the PESI data warehouse. This includes an index of species names associated with a number of attributes, such as synonyms, their place in the management classification and their geographical distribution. This data content results from the integration of the earlier pan-European checklists into a unified directory, following advanced routines on data verification and harmonisation. These routines are laid down in the PESI Common Data Model store (PESI CDM-store), an instance of the EDIT Platform for Cybertaxonomy. The PESI data warehouse not only hosts the integrated Taxonomic Backbone for Europe, but also allows unambiguous cross-links to, and compatibility with, other biodiversity information services, such as persistent identifiers, standardised vocabularies and exchange formats. Therefore this component is crucial to secure the stability of the European Taxonomic Backbone and to link PESI with global e-gateways.

### e-Services for users and dissemination

PESI has built an interactive, multilingual web portal to carry out the dissemination of the developed species names service and to support the use of the pan-European species data in the e-science domain. This includes relevant supplementary data, such as (region-based) occurrence details, literature and DNA sequence information, and applies dynamic links to other pertinent data services. Additionally, web services allow users to link PESI-functions into desktop applications as well as service-oriented information infrastructures and hereby establish enhanced access to species names, GUIDs, occurrence details and the hierarchical classification. The PESI web portal provides the interface to the European Taxonomic Backbone.

## Project results

PESI resulted in an integrated overview regarding the taxonomy and occurrence of European species, including their current legislation status and other important metadata annotations (like vernacular or common names). The PESI Taxonomic Backbone serves as a taxonomic data standard resource, facilitating and optimising the integration and sharing of European biodiversity data, supporting a wide range of European services, major biodiversity programs and stakeholders on nature conservation and biodiversity management.

### Towards a European taxonomic workforce (ETW)

**Figure 2.**
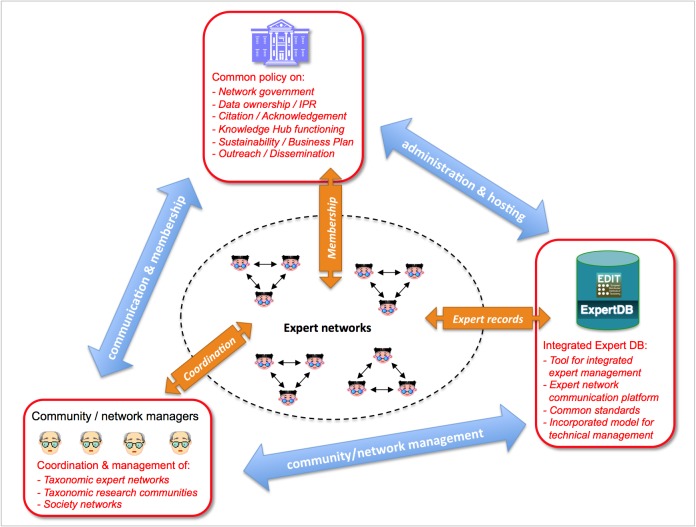
PESI Expert Communities Common Infrastructure outline, showing a common governance organisation, including relevant expert(ise) network management tools.

**Figure 3.**
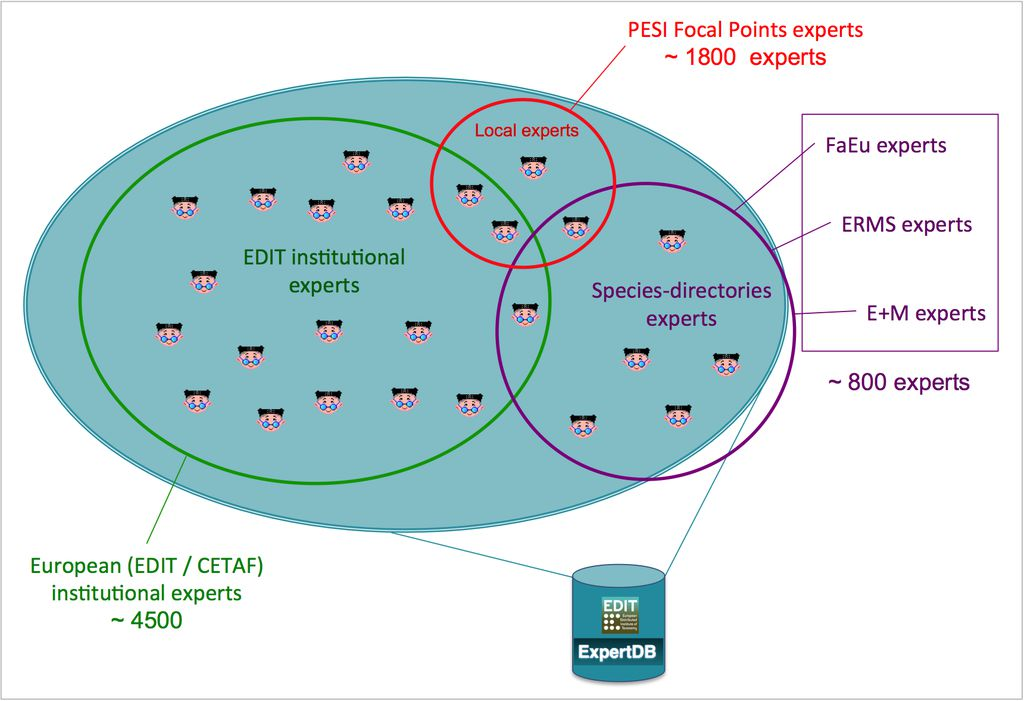
Europe's integrated taxonomic workforce as established in EDIT and PESI, brought together (as a pilot) into a shared expert system.

**Figure 4.**
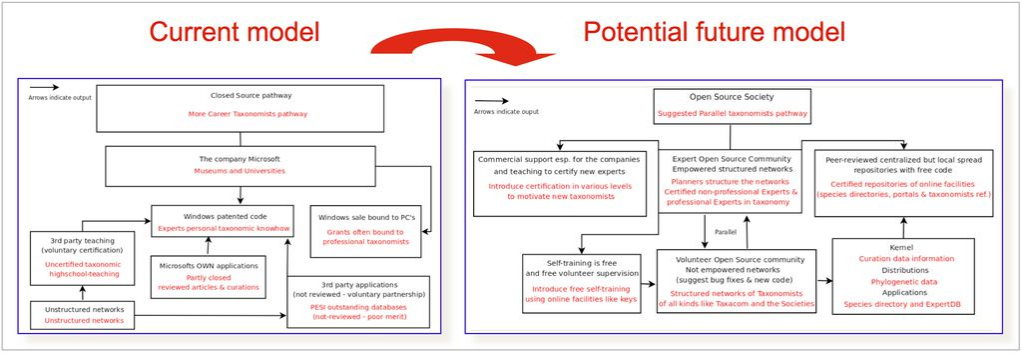
The *Open Source Society* network as an analogy for modelling the future *Taxonomic Community*.

*A pan-European Taxonomic Workforce (ETW)*, consisting of all people who carry out taxonomic work in Europe, was identified as pivotal for the integration and coordination of the current and future expert networks within Europe (Fig. 2). This task force integrates the already existing expert networks supporting the pan-European checklists, extended by the collaborative capacity of the EDIT institutional staff previously assembled within the EDIT Expert Database (Fig. 3). The additional inclusion of the Focal Point network was an important step forward carried out by PESI. A report on the tasks, activities and operational standards necessary for a functional ETW was drafted (Suppl. material 2), taking into account the concepts of an Open Source Society model (Fig. 4) and discussing the need for an *accreditation system* for taxonomists, clarifying expert competency through certification, a citation system for online data, and a method for recognising the contribution of non-career taxonomists and other citizen scientists groups. Also the proper acknowledgement of (online) taxonomic databases curation by museum staff was evaluated and approved by CETAF.

*Quality control and standards*

PESI has set out a working Intellectual Property Rights (IPR) model, based on the SMEBD model, under which PESI can proceed with its aim to integrate taxonomic databases in Europe, ensuring the free and wide dissemination of data, and promoting the further virtualisation efforts (e-Taxonomy; e-Science; e-Publishing) in this field. The SMEBD model encourages scientists to input their data without being burdened with its upkeep. This allows scientists to retain full ownership of their work, knowing that data stewards are managing their data accordingly. This IPR model proposes a Creative Commons Attribution (cc-BY) license that allows for the wide dissemination of data to other scientists and interested parties, giving recognition to the creator of the data (but see Hagedorn et al. 2011 for pitfalls of the -by clause-). In relation to citation of data, PESI recommends (Suppl. material 3) that contributing databases adopt a standard citation system to simplify the citation system in PESI and that the date accessed be automatically added, as already done in ERMS.

*Gap analyses and Continuity*

To secure the continuity of electronic biodiversity data, PESI has reviewed gaps in taxonomic expertise, species registers and informatics resources throughout Europe and surveyed potential ways to complete these gaps (Suppl. material 4). From the data presented in the report it was found that Kingdom Animalia is the closest to completion, followed by the Kingdoms Chromista, Plantae, Fungi and Bacteria. However, it is clear that the completion of each of the Kingdoms will not be without problems and will require further funding.

The functional and successful operation of ETW involves a series of informatics resources, which will be addressed within the gap analysis, particularly in relation to successful developments in this area, like Scratchpads, the Platform for Cybertaxonomy and tools for e-Publishing.

Recently the hosting of the EDIT Expert Database (including the ETW) at the Zoological Museum Copenhagen has ended in favour of a less centralised model, hosting the relevant (zoological, botanical, marine, terrestrial, etc.) expert data in the respective pan-European checklists databases. A collaboration with CETAF on the governance of taxonomic knowledge networks is under consideration (see section 'Progressions').

The continuity of relevant electronic taxonomic biodiversity data resources and expertise networks were examined (Suppl. material 6), also as an input to the PESI business plan (Suppl. material 7). This shows a close collaboration with other initiatives like GBIF, LifeWatch, EU BON and Global Names (see also the sections 'Progressions' and 'Outlooks') and the PESI Focal Points network (see below).

### National Focal Point network set up

**Figure 5.**
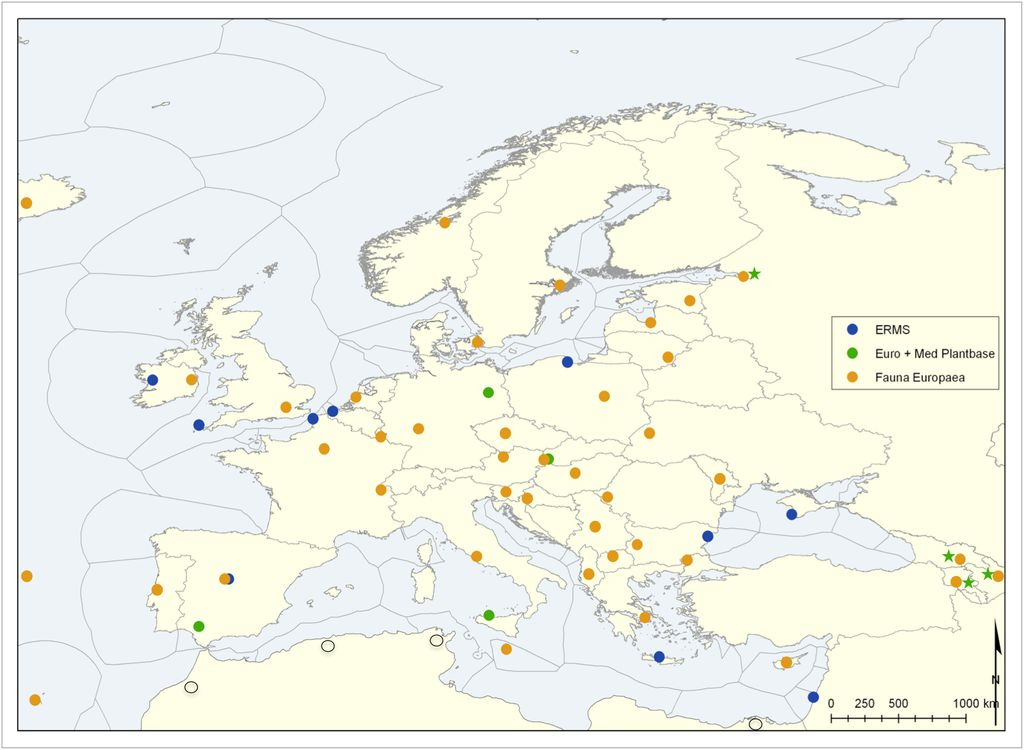
PESI Focal Points Network, showing the respective Fauna Europaea, Euro+PlantBase and ERMS focal point partners. Northern African 'proto focal points' are indicated with open circles. Pan-Caucasian Plant Biodiversity Initiative partners are indicated with green asterisks. Focal Points network details are described in Suppl. material 34.

**Figure 6.**
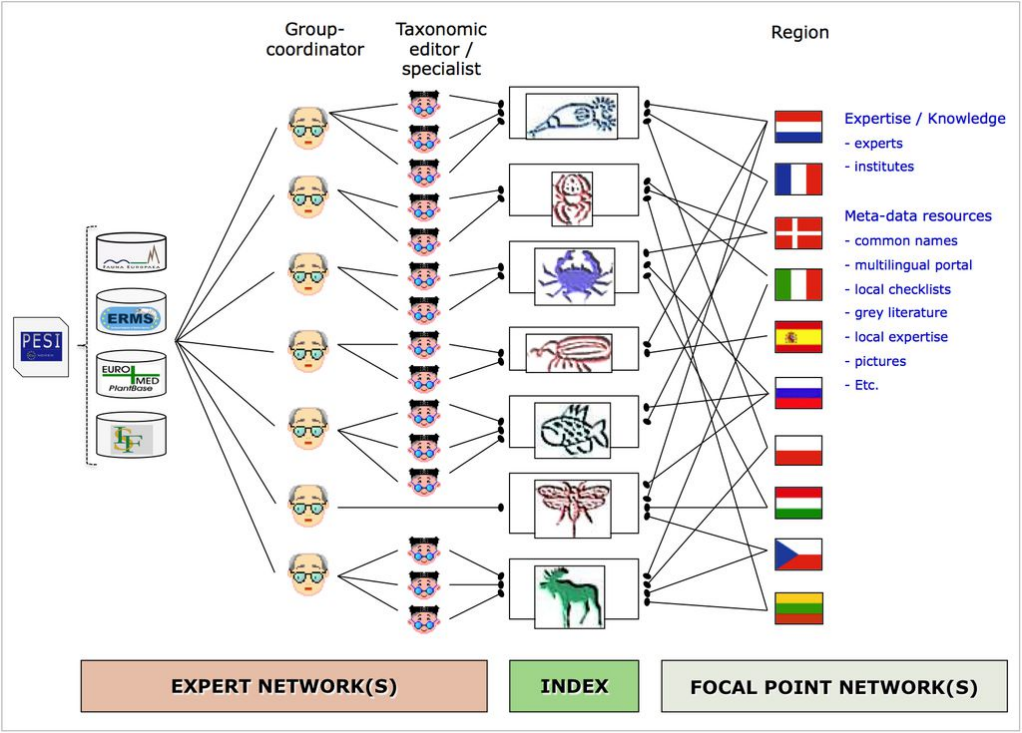
The role of PESI Focal Points as an addition to the expert network.

The terrestrial and freshwater Focal Point Networks in PESI originate from focal point partners within Fauna Europaea (Jong et al. 2014) and Euro+Med PlantBase. This integrated Focal Point network proceeded from groundwork carried out in the pan-European checklist programmes (like Fauna Europaea NAS) and was further explored in the FP6 EDIT Network of Excellence (Suppl. material 8) as a vehicle to annotate taxonomic data more generally. The marine focal points originate from marine networks constructed during FP6 Networks of Excellence (MarBEF/ERMS) and other marine projects. PESI comprises a total of 34 contracted Focal Point partners and 24 associated, non-contracted, Focal Points being members of the consortium from 47 countries (Fig. 5). In addition to the expert networks (organised around a taxonomic group), the Focal Point networks in PESI provide a complementary organisation of taxonomic expertise according to their geographical, national and regional focus (Fig. 6). A Focal Point Working Group committee was created in the initial project year to define the Focal Point working programme, set up the tasks, assist the Focal Points in networking processes and in preparing the activities/deliverables. The committee also prepared a plan for sharing and distribution of the reserved seed money budget among the Focal Points to ensure their ability to perform the activities described in the work programme (Suppl. material 9) further detailed in a Focal Points Handbook (Suppl. material 10).

For the botanical community, the PESI Focal Point network built on the existing infrastructure of a regional advisory network for Euro+Med Plantbase. The former Euro+Med PlantBase editorial centres, each of them responsible for the coordination of a share of vascular plant families, became the PESI Botanical Focal Points: Berlin (Euro+Med PlantBase secretariat), Bratislava, Palermo and Sevilla, plus Helsinki as a non-contracting partner. The Berlin Botanical Focal Point established and maintained the connection with the regional advisory network, consisting of 85 floristic and taxonomic botanical experts, mostly based at botanical institutions all over Europe, Northern Africa, the Levant and the Caucasian countries. The regional advisers are responsible for 64 major areas (countries) and 48 minor subdivisions (mostly individual islands). Euro+Med PlantBase now has 190 vascular plant families online, corresponding to 95% of the European Flora of Vascular Plants. The Helsinki FP took care of linking grid-map data from the Atlas Flora Europaeae with the map services of the PESI web Portal.

A selection of the Fauna Europaea Focal Points agreed to become PESI Focal Point partners, others became associate, non-contracted Focal Points. The network of Marine National Focal Points aims at the continuous communication, information exchange and harmonisation of taxonomic data and practices followed by the systematists involved. Only through this process it was possible for the European taxonomic experts on marine taxa to join forces and avoid further fragmentation after the end of the Networks of Excellence, within the 6th European Framework Programme.

Over three years the entire process resulted in two major achievements: a) it created the largest network of taxonomists in Europe through the National Focal Points, and b) produced a massive amount of information on all kinds of existing nomenclators, museum catalogues, existing taxonomic expertise, societies and systematists assemblages, taxonomic information publishing gates, etc., which are now available through the single multilingual portal of PESI, which can, in turn, be used by any member of the National Focal Point network. However, the overall achievement is that the defragmentation of the European taxonomic community was encountered and that the process paved the way for this community towards a cohesive and challenging framework in the future. The next successful steps are already visible through a number of projects and initiatives (e.g. LifeWatch, EU BON, and COST action applications).

At this moment the public PESI Portal includes a large number of taxonomic-related data provided by the Focal Points. These data comprise contact and expertise details of nearly 2,000 experts and more than 500 organizations (from professional institutions to amateur societies) in Europe, in addition to the European Taxonomic Workforce (ETW) and EDIT Expert Database (see above, Fig. 3). It also contains almost 2,000 scientific publications in 700 journals and more than 100 URLs of websites with information on local fauna and flora. In addition there are 190,800 vernacular names of species and higher taxa in 117 languages. To keep the Focal Point Networks active, a Focal Point sustainability plan was drawn (Suppl. material 7) from a questionnaire that was completed and returned by 23 Focal Point partners. Regarding future coordination and management ambitions for grouped Focal Point networks, we derived a draft plan based on their answers (Suppl. material 37).

PESI has a significant outreach to eastern European countries and beyond, connecting local knowledge networks to pan-European expertise and supporting the implementation of relevant e-Infrastructures for information management. For example, the Ukraine PESI Focal Point was selected as incrEAST "Project of the Month - March 2012" as well as contributing to the GÉANT Eastern Partnership Event Program in Moldova.

### Conceptual integration: standards and internationalisation

PESI is about integration and presentation of data. To do this effectively a common framework is needed to support the establishment and promotion of controlled vocabularies and metadata standards that enable effective integration of data and best practices. PESI sets out to provide a taxonomic backbone for Europe, at the heart of which is an annotated synonymised checklist of species. The prime focus has been to identify appropriate standards to support this task. In addition to ensuring that data held within PESI are successfully integrated, it is important that our data are accessible and interoperable with other initiatives. We have therefore looked at protocols, data models, controlled vocabularies and ontologiesthat will facilitate this interoperability, working closely with external organisations involved with biodiversity informatics to assist in the process of developing standards and resources for data exchange and data validation.

More specifically PESI focused on:

- Defining PESI as an annotated checklist, listing the range of different taxonomic products, differentiating between standard taxonomies and standards used to exchange taxonomic information and build consensus biological classifications (Suppl. material 11).
- Examining the rationale, logistics and challenges to coordinate a set of independent initiatives that collectively catalogue nomenclatural acts according to the different codes of nomenclature, allowing a differentiation between nomenclature and taxonomy and proposing a strategy for linking nomenclators to PESI (Suppl. material 12). For this purpose a series of workshops was organised in collaboration with EDIT, including a "GNOMA" workshop, to develop a common terminology and define common services and formats for a 'Global Nomenclators Architecture' (Suppl. material 13), and workshops focused on identifying strategies for populating ZooBank, the developing registry for animal names (Pyle and Michel 2008), inviting ICZN commissioners, data managers from a number of large zoological databases, representatives from Zoological Record, and representatives from other nomenclatural databases regarding common issues such as the registration of new species names (Suppl. material 14) (see also section 'Outlooks').
- Outlining the success in reaching agreements between key players in the global community and specifically detailing how standards would be used to exchange taxonomic data, in particular by using the Darwin Core Archive format. This has resulted in The Montpellier Declaration (Suppl. material 15).
- Identifying the opportunities for PESI to contribute to the setting up of a global system for managing scientific names of organisms and potential pitfalls (Suppl. material 16).

PESI researched and proposed standards for working with data associated with biological organisms, including:

- A management classification integrating the schemes used in the component datasets.
- An informal classification using informal names for higher groupings familiar to the non-expert (terms such as: snails, butterflies, dragonflies).
- A scheme for Globally Unique Identifiers (GUIDs) that can be applied to biological names (and other entities) to allow machine-level matching of equivalents.
- A vocabulary of approved terms to cover occurrence status of organisms.
- A controlled set of terms for geographical areas, including European marine regions.
- Adoption of the Darwin Core Archive standard (Taxon & Occurence terms) for data exchange.

The latter has been developed by the Biodiversity Information Standards organisation (TDWG) in association with GBIF and provides a simple flat-file structure, such as may be used in a spreadsheet or delimited text file, together with two XML metadata files that describe the resource and the data structure. It is important to use a simple solution to make it easy for data providers to submit their data to PESI. It also provides the mechanism for PESI data to be passed to third parties, such as GBIF or other European taxonomy and biodiversity projects.

Controlled vocabularies developed and recommended for occurrence status, taxon status, nomenclatural status and geographical regions in use in the PESI data warehouse and PESI Portal are listed as an appendix in the PESI Focal Point Handbook (Suppl. material 10).

PESI partners organised sessions and presented papers at the annual conferences of the Biodiversity Standards Organisation (TDWG) and, most importantly, made use of these occasions to further the development of PESI objectives through networking with the leading participants in the development of biodiversity informatics standards. A significant outcome from these activities is the ‘Montpellier Declaration’. This is an agreement, proposed by PESI, between major biodiversity informatics projects to use a standard approach to sharing data (discussed in Suppl. material 15). More recently, at the TDWG 2013 meeting in Florence, PESI partners took the lead on establishing the Research Data Alliance (RDA) Biodiversity Data Integration (BDI) interest group, also discussing the potential of a so-called RDA 'Global Names Architecture' working group on defining a next generation names infrastructure supporting biodiversity research as a linked open-data science (see also the section 'Outlooks'). In addition to the RDA charter, the importance of developing a next generation names infrastructure, provisionally called "Global Names Europe" (GN-EU), was put forward by PESI partners (i) as participants of the ‘Nomina meetings' (organised by GBIF and EoL), (ii) as a ViBRANT milestone (Suppl. material 17), (iii) as input to EUDAT Data Pilot surveys, (iv) as part of the LifeWatch roadmap, (v) as a contribution to COOPEUS (enhancing US and European collaboration) and (vi) as a EC DG CONNECT consultation bid (Suppl. material 18).

### Technical integration: advances in the Platform for Cybertaxonomy

International infrastructures for the production, maintenance, and publication of taxonomic checklists are highly heterogeneous with regard to scope, information models, workflows, and implementation. The PESI project recognised from the beginning that an effort for integrating European checklist information into a single unified system would need an “information broker” responsible for merging disparate and potentially conflicting information, performing data quality measures, and streamlining the process of data publication from the individual checklist to the common European information portal and service layer.

**Figure 7.**
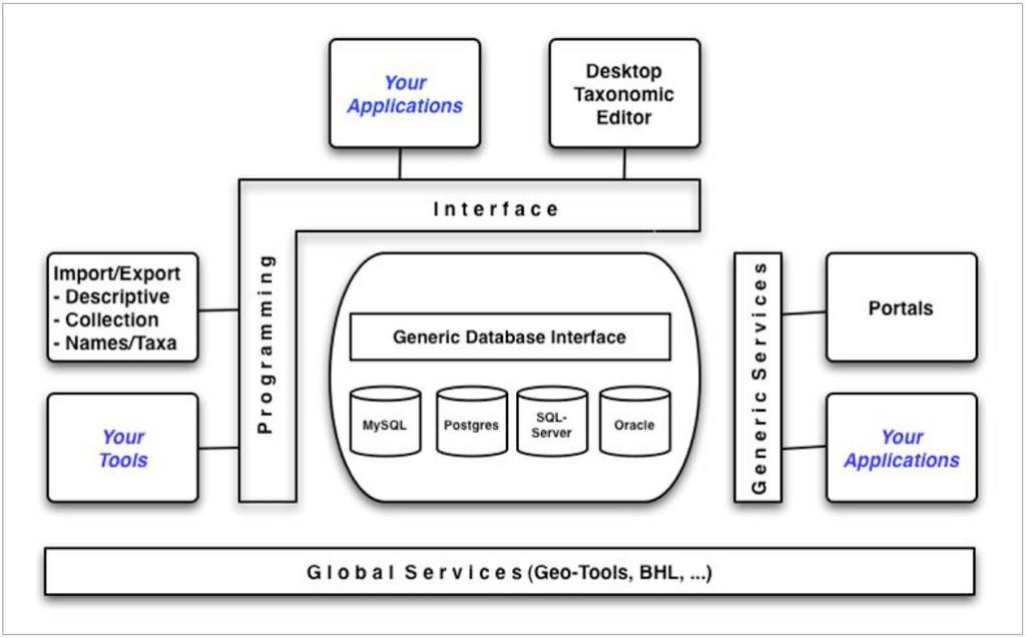
Simplified architecture of the EDIT Platform for Cybertaxonomy. Internal data stores are encapsulated by Java and web service APIs.

The EDIT Platform for Cybertaxonomy (Ciardelli et al. 2009, Berendsohn 2010, wp5.e-taxonomy.eu), implemented in the framework of the EU Network of Excellence EDIT, provides the technological basis for the mediation of taxonomic data and is used for merging processes, quality control, and pre-processing for data publication in PESI. The Platform is based on the EDIT Common Data Model (CDM), which is an agreed and comprehensive object-oriented information model covering the taxonomic workflow from fieldwork to data publication in electronic form and on paper (Fig. 7).

The model can be deployed using almost any Database Management System (DBMS). Application programmers can develop all kinds of systems (e.g. portals, editor software, import and export functions) using well defined APIs (Application Programming Interfaces) exposed as a Java Library (wp5.e-taxonomy.eu/cdmlib) and as generic web-services (wp5.e-taxonomy.eu/cdmlib/rest-api.html).

*Data merging and publication*

For its deployment as the central merging facility in PESI, the BGBM-team extended the platform and its library (Suppl. material 19) with methods for:

- importing data from the existing pan-European checklists,
- merging them into a single taxonomy across organism groups,
- quality control at different levels,
- creation of detailed reports for feedback to the checklist managers,
- mapping of the vocabularies used by the individual checklists to the agreed PESI vocabularies (e.g. status values, geographic regions),
- export of the consolidated checklist into the PESI data warehouse.

**Figure 8.**
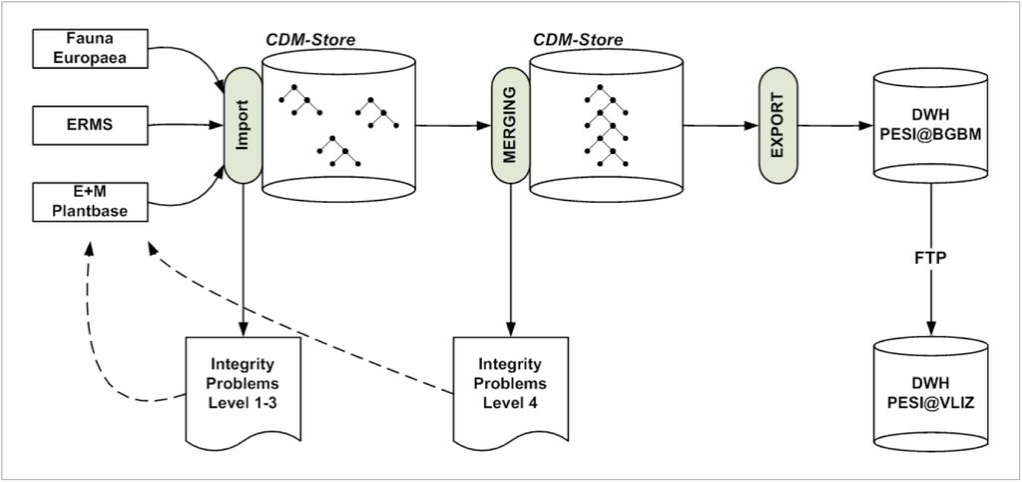
Information flow for merging and publication of checklist data in PESI (after Suppl. material 20).

With these methods, a data publication cycle in PESI is performed in three basic phases (Fig. 8):

1. Import. The source checklists are parsed and transformed into the internal CDM data structure using the import layer of the EDIT platform. This step also involves data quality control at three levels. Level 1 (syntax of terms) checks the syntactical correctness of individual terms. Level 2 (structural integrity) checks the completeness and appropriateness of data belonging to individual objects. Level 3 (referential integrity) checks the correctness of relations between objects. The different quality levels and rules have been jointly developed on a common EDIT/PESI wiki site.
2. Merging. The individual taxonomic trees are merged into a single taxonomy, which is later used for data publication. This merging involves a final data quality assessment (level 4) for the detection of overlaps and conflicts between the different checklists. Again, a transcript is produced and fed back to the checklists managers responsible for resolving the conflicts or defining priority rules.
3. Export. All data are exported into a Data Warehouse Structure optimized for data publication purposes. From the FTPserver hosted at the BGBM the data are harvested for publication at VLIZ.

*The PESI GUID strategy*

The publication of taxonomic data in web portals (for human consumption) is an important aspect of the PESI infrastructure. However, providing information in machine-readable form is becoming increasingly important, because this makes the information reusable in a wide range of potential applications (e.g. as part of the taxonomic backbone in species information systems). PESI addresses this aspect by including a SOAP-compliant web-service interface into its portal implementation. In addition, REST-full services have been implemented, providing light-weight interfaces to the PESI backbone, like EU BON taxonomic backbone UTIS. As prerequisite, the technical partners had to agree on a common approach for handling identifiers (Suppl. material 21). The agreed system consists of UUIDs implemented by the contributing checklists in parallel to their local identifier system. These UUIDs are propagated through the merging process and finally published using the OMG LSID protocol. Aside from its LSID, each taxon (also) has a persistent HTTP-URI identifier using the LSID as part of its syntax and redirecting to a web-representation of this taxon. With this architectural approach, PESI can adopt additional identifier-protocols in the future with comparably little effort by adding functionality at portal level.

The agreement on a common identifier system had to include clear rules as to when changes to an object imply issuing a new identifier. To ensure consistent application, these rules have to work at a machine level - the operations that turn an existing taxonomic object into a new one had to be defined. The PESI approach has been recognised by the “Beginner’s Guide to Persistent Identifiers” recently published by GBIF and is fully compliant with the persistent identifier strategies developed by the organisation for Biodiversity Information Standards (TDWG).

*Sustainability of the platform development*

The EDIT Platform for Cybertaxonomy is continuously improved by an international team of developers, coordinated by the Biodiversity Informatics Research Group of the BGBM. It is the basis for an increasing number of checklists on different scales as well as geographic and taxonomic scopes. In addition, several new EU e-infrastructure projects have the EDIT-Platform integrated as an important technological component:

- i4Life provided tools for the comparison and harmonisation of the various species catalogues used by six global biodiversity programmes using the Catalogue of Life as a yardstick, including the creation of an EDIT Platform instance of the Catalogue of Life, which can be integrated in the emerging LifeWatch infrastructure.
- ViBRANT supported the development of virtual research communities involved in biodiversity sciences. Interfaces between the EDIT platform and Scratchpads (the primary technological platform for ViBRANT) were defined and implemented (see section 'Progression').
- BioVeL developed tools for pipelining data and analysis in Biodiversity Sciences into efficient workflows. One aspect of BioVeL is to expose EDIT platform services in a way that makes them efficiently usable in workflow environments (see section 'Progression').
- EU BON is building the European Biodiversity Observation Network, as a European contribution to GEO BON (see 'Progression' and 'Outlooks' sections).

With new projects related to EDIT platform developments in progress (like EU BON) and the increasing number of users of the technology we expect to maintain the Platform as an integral part of the European and world-wide biodiversity informatics landscape.

*Sustainability of European GSDs*

**Figure 9.**
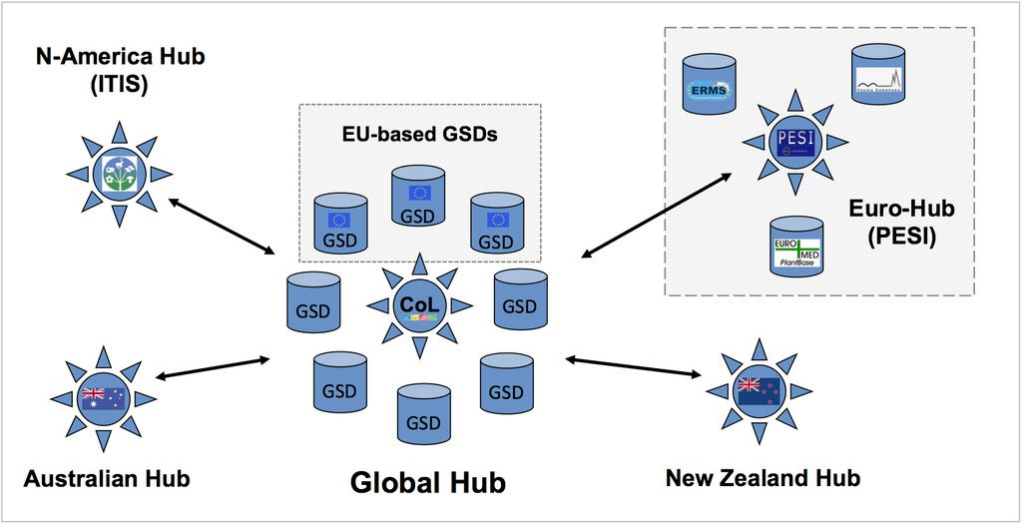
Simplyfied *Catalogue of Life* Architecture, showing two PESI contributions: Euro-Hub development and governance of EU-based GSD.

PESI supported the development of governance plans for EU-based Global or Regional Species Databases (GSDs/RSDs) in the following ways: (a) as a European taxonomic infrastructural component, (b) as a contribution to global efforts like the Catalogue of Life (Fig. 9), (c) by evaluating the costs for future maintenance (maintenance and updating) and (d) by providing recommendations to optimise the collation and integration of taxonomic data in terms of expert network management, data hosting, and data interoperability (Suppl. materials 22, 23).

### PESI e-Services

*PESI portal*

**Figure 10.**
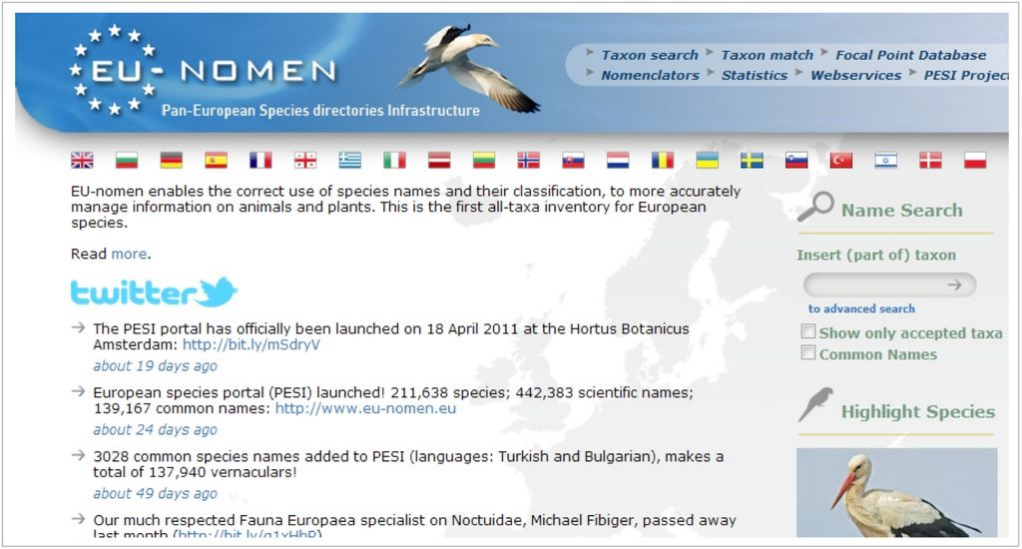
PESI web-portal homepage (screenshot).

The PESI web portal is the interface to the European Taxonomic Backbone (Fig. 10) developed and hosted by the Flanders Marine Institute in Belgium (Suppl. material 24). The portal provides an integrated view of the pan-European checklists.

Still operating separately, the register’s data are merged about every year in the PESI Data Warehouse and are made available through this single portal. In addition to taxonomic information, PESI harvests information on species (images, literature, conservation status) and provides links to other portals (e.g. national checklists, red species lists and other bioinformatics databases such as the Biodiversity Heritage Library for literature and the DNA databases of the Barcode of Life and GenBank). So far three editions of the European Taxonomic Backbone have been released (see section 'Dissemination').

The portal is an important instrument for standardisation of species names. The search interface is the main public access point to information on species living in Europe. However, the portal also provides services for those building their own species applications. The PESI-website can be consulted in 21 European languages.

*Web statistics*

**Figure 11.**
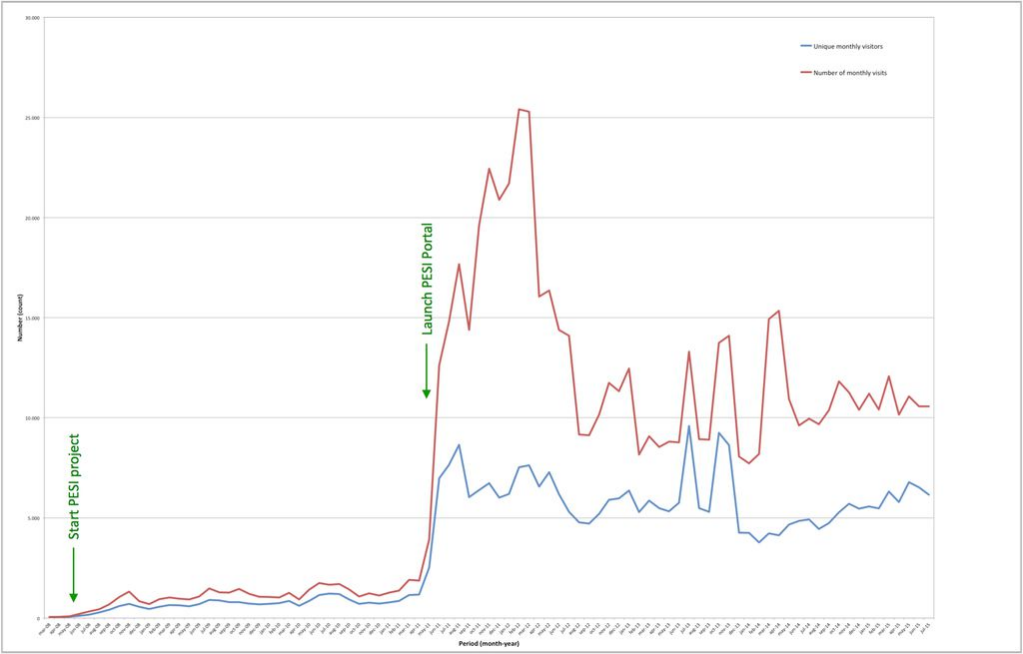
PESI web-portal statistics showing the number of unique (monthly) visitors and the number of (monthly) visits since the start of the project (see: Suppl. material 35).

The website attracts around 5,000 unique visitors per month (Fig. 11). The majority of the portal visitors enters the PESI web site via the direct address; only a small number of the visitors are directed to the site by internet search engines, of which Google is the most important.

*Search parameters*

**Figure 12.**
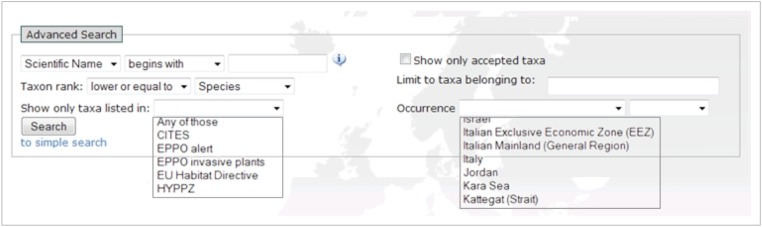
PESI web-portal advanced search interface (screenshot).

**Figure 13.**
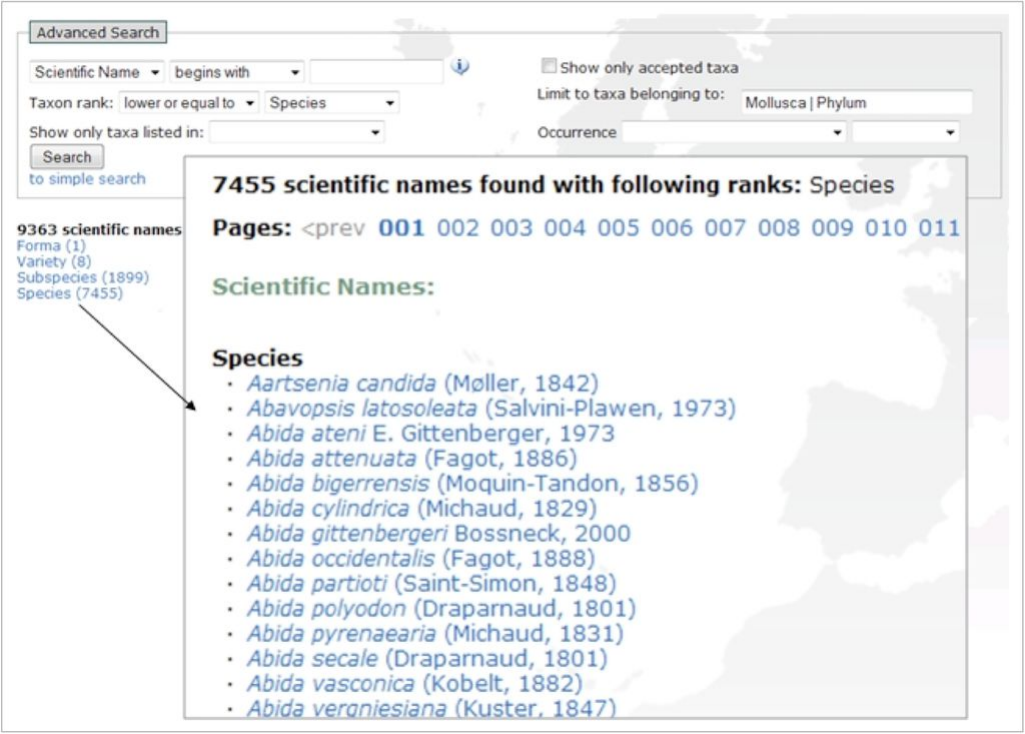
PESI web-portal example of a search and output of all species and infraspecies of molluscs

The advanced search interface provides a number of fields to generate output based on selected parameters (Fig. 12). This can be seen as applying filters on a database. The parameters can be combined (Fig. 13), and none of the fields are mandatory, which means that a search without setting a parameter will output the entire database.

*Taxonomic parameters*

- (part of) Scientific name, Common name, Authority
- Equals/above/below taxon rank (e.g., species, family, class, …)
- Belonging to a group (a higher rank e.g., Mollusca)

*Additional parameters*

- Priority lists + Priority status (e.g. IUCN-endangered, EU Bird directive, HYPPZ,... ). For a common agenda on prioritised species see: Suppl. material 25.
- Occurrence + Occurrence status (Absent, Present, Introduced,... )

The occurrence status “present” includes all other statuses except for “absent”. The list of areas is linked to the Marine Regions gazetteer. This hierarchical gazetteer of place names makes it possible to add relationships to areas. The users can, for example, generate a list of species from France and the system automatically includes the species that are recorded as present in Corsica.

Besides creating lists of species, the user can search for a particular taxon by entering (part of) the scientific name, name authority or common name. However, if there is no exact match, the search tool performs a number of ‘intelligent’ consecutive queries until matches are found:

1. fuzzy match (Tony Rees’ TAXAMATCH algorithm), which checks for a number of spelling errors,
2. checks if the name is present in the World Register of Marine Species (WoRMS),
3. checks if the name is present in the Catalogue of Life (CoL),
4. checks if the name is present in the Global Names Index (GNI),
5. checks other potential genus-species combinations:

- FaEu model: it checks for reverse synonyms, e.g. when the species epithet occurs in a current combination and you enter a synonymous genus or species name or both in the search box. For example, if you enter *Avesaonchotheca blomei* the portal will not find an exact match, but will suggest *Aonchotheca caudinflata* (Molin, 1858), because *Avesaonchotheca* is a generic synonym of *Aonchotheca*, and the species epithet *blomei* occurs in *Capillaria blomei*, which is a synonym of *A. caudinflata*.
- WoRMS model: checks if the species epithet occurs in other genera within the same *Classis*. For example, if you enter *Parus merula*, then the portal will not find an exact match, but will suggest *Turdus merula*, because it knows the genus *Parus* belongs to the Class Aves (=birds) and the species epithet *merula* occurs in the bird genus *Turdus*.

*E-services for taxonomic standardisation*

The PESI web portal also provides a number of tools for quality control and to standardise a user's own species names. The ‘taxon match’ is an intelligent name validation service to cross-match external species lists against names in PESI. In addition, if an external species list is restricted to a particular area, you can also check if this corresponds to the occurrences in the PESI database. Once you have run the match, the tool exports the results (exact and suggested spellings, the higher classification, occurrence status and the checklist’s Globally Unique Identifiers) as a spreadsheet file.

The PESI taxon match tool has been promoted as an important tool within the PESI focal points network and at various other meetings (e.g. GBIF EU-nodes meetings). During the project phase, as part of the PESI Focal Points validation process, 357 files have been uploaded and matched.

In contrast to the taxon match, where users have to upload a species list, the portal also provides a platform-independent SOAP/WSDL web service. This web service allows users to dynamically link their own applications to the PESI database and will allow them to match a locally stored species list and add taxonomic and additional information derived from PESI.

A few examples of possible applications:

- getGUID: Get the first exact matching GUID for a given name.
- getPESIRecords: Get one or more matching (max. 50) PESIRecords for a given name.
- getPESINameByGUID: Get the correct name for a given GUID.
- getPESIRecordByGUID: Get the complete PESI Record for a given GUID.

*Additional (other than taxonomic) information on the web portal*

The PESI portal shows species distribution maps, if occurrence details are available. There are over 6 million distribution records in the PESI database. The maps are built on OpenLayers. The backend of both occurrence types is GeoServer, an open source implementation of WMS that implements the Open Geospatial Consortium (OGC) standards.

There are two types of occurrence data on the portal:

1. The first type is provided by the component databases and directly included in the PESI Data Warehouse. The areas used have been standardised to TDWG areas for terrestrial areas and for marine areas to the IHO/EEZ Intersect, which is a standard proposed by VLIZ. These occurrences are shown on the map as polygons and coloured according to the Occurrence Status.
2. The second type of occurrence data are those provided by Atlas Florae Europaeae (AFE). AFE provides its occurrence data via a Web Mapping Service (WMS) server set up at the University of Helsinki. The data have been extracted from the AFE volumes and displayed as a grid, and colored according to the PESI Occurrence Statuses. These occurrences are restricted to a number of vascular plants described in AFE.

*Deep links to other biodiversity information systems*

The PESI portal provides links to other portals (see above). On name matching, more specifically:

The Biodiversity Heritage Library (BHL) API is queried for scientific names (redirecting to the Global Names recognition and discovery tools and services). If matches are found, the information is cached in our database for one month. The next time someone loads a particular taxon page within the same month, the cached information is displayed. If the request time compared to the last update in the database is above one month, re-querying the BHL web service refreshes the information.

GenBank provides a standard method for linking to their pages. We retrieve the information from their Taxon Browser tool. If the link exists, it is displayed on the species page.

The Barcode of Life database (BOLD) results are fetched from the BOLD Taxonomy Browser. Only when the 'specimens with barcodes' parameter is set, a link is visible on the species page.

### e-Publications

The PESI project recognises the need to link the conventional scientific publications to the species databases to provide users with a more comprehensive resource. To that end the project engaged with major publishers of scientific journals to understand how their online information systems were developing, and to explain how the species databases were evolving. A first workshop was held on 16th July 2009 in Amsterdam inviting key publishers, including PLoS, InterResearch, Allen Press, CRC Press (Taylor & Francis), Oxford University Press, Scopus, Science Direct, Elsevier, ISI Web of Science of Thomson Reuters, OvidSP (Biological Abstracts), Wiley, ProQuest (part of Cambridge), and JSTOR (Suppl. material 5).

The process of contacting publishers revealed that most publishers were very limited in the functionality they had on their websites, and constrained by the limitations of the commercially provided software they used to provide added functionality. A first step in that regard has been the initiation of a special PLoS ONE Collection of scientific papers arising from the World Register of Marine Species (WoRMS). WoRMS is a superset of the European Register of Marine Species, which is one of the three primary European species databases that form the core of the PESI project. At present, more than 19 papers have been published (Costello et al. 2013b). Each paper should be directly linked to the WoRMS database so readers will get openaccess to the primary data and information.

A similar approach was followed later on by Fauna Europaea in collaboration with Pensoft, supported by the FP-7 ViBRANT project, starting the publishing of so-called 'Contributions on Fauna Europaea' as a special series of the Biological Data Journal (BDJ), with 10 data papers published so far. Euro+Med from the beginning took a distinctive approach towards updating the database. For instance, all occurrence records for a species in Euro+Med must have been published in a peer-reviewed paper. To accommodate miscellaneous new data, a series of "Euro+Med Notulae" was initiated in 2005 (Greuter and Raab-Straube 2005).

Another conclusion of this publishers workshop was that an important, practical, option to link to a wider range of journals would be the use of RSS feeds, because this would provide no or little action on the side of the journal. Such a system would need the feeds to be aware of what species (or higher taxonomic) names to search and match to the published papers. The Global Biodiversity Information Facility (GBIF) communicated their advances on developing a relevant tool. The usefulness of such a tool should be tested. For example, if species or taxonomic names are not apparent in the titles, abstracts and keywords of journal articles, they may be overlooked. Alternatively, papers of very peripheral relevance may be fed to the species database and overwhelm the editors and readers. In the latter case additional filters could be placed to constrain the RSS feed. The PESI partners intend to explore these options further on when the GBIF tool becomes available, a collaboration highlighted at the EDIT second publishers workshop in Bratislava (Suppl. material 26).

The highest priority journals for species databases to be linked are those describing new species, and rationalising species nomenclatures (e.g. identifying synonyms or reclassifying species). One of the leading taxonomic journals in this field is Zootaxa, which publishes almost five times more new species than the next ranked journals (http://www.organismnames.com/metrics.htm?page=tsj). The PESI project started a regular dialogue with this journal, which led to the invitation of the Zootaxa Chief Editor (Dr Zhi-Qiang Zhang), to a follow-up EDIT third publishers workshop in Copenhagen on 7-8 October 2010 (Suppl. material 27), jointly organised by EDIT and PESI. EDIT had proposed a new European Journal of Taxonomy (EJT) that would integrate existing small journals in Europe, and thus have the resources to modernise their publication processes. This is now established (Bénichou L et al. 2012). However, only a few journals appeared willing to lose their identity by joining EJT. Zootaxa's Chief Editor proposed a complementary approach to journal ‘integration’. In this ‘aggregation’ approach, a common portal would be established using open-source Open Journal Systems software. This would manage editorial processes (i.e., paper submission, assignment of referees, editorial decisions), and publication (e.g., when online, when open access) and archiving. Importantly, each paper, past and future, would be indexed by species names and classification, and geographic area studied, so as to directly link papers with species in the PESI databases. This portal would be called Biotaxa and is now operational and serving 30 journals, including those that describe 30% of all new species each year. We envisage that moving the editorial process of taxonomic publishing online by providing a permanent low-cost portal for publication and by archiving an intimately linked with the expert-edited species databases will revolutionise the taxonomic publications process (see also 'Outlooks' section).

In relation to this subject, the 'Environmental and Natural Science Publishing in Europe' (ENSI) proposal was drafted to the European Commission FP7-SCIENCE-IN-SOCIETY-2011-1 call for funding entitled ‘Improved dissemination and preservation of natural history publications’ (FP7 289063).

## Project dissemimation

The project results have been dissemimated in various ways. Some main public communication tools are mentioned below.

### Data Resources

The PESI project homepage can be found here: http://www.eu-nomen.eu/pesi

An interface to the European Taxonomic Backbone is provided by the PESI webportal: www.eu-nomen.eu

**Figure 14.**
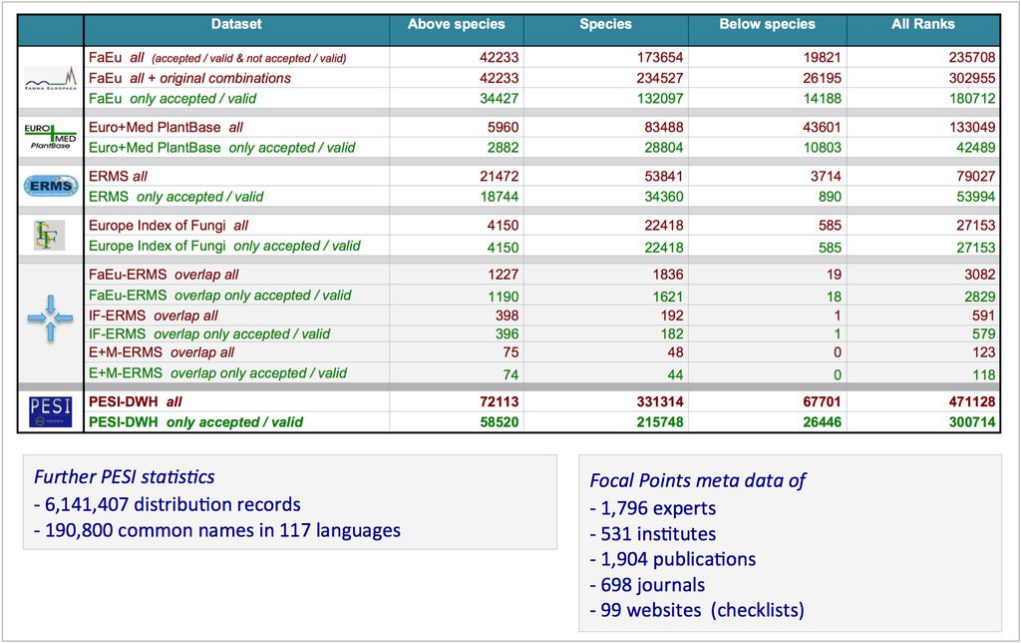
PESI Portal and PESI Datawarehouse (version 3) statistics. Source: http://www.eu-nomen.eu/portal/stats.php.

PESI statistics for version 3 are summarised in Fig. 14.

### Project Information & Promotion

An introduction to PESI is available as a videoclip: http://www.eu-nomen.eu/portal/introvid (Suppl. material 36)

A PESI brochure is available here: Suppl. material 32

A PESI Flyer is available here: Suppl. material 33

## Project progressions

PESI is well situated within the EC infrastructural and policy development. This is partly due to the fact that many end-users, stakeholders and EC directives (like INSPIRE) adopted PESI as a European standard. Therefore major European biodiversity programs, like LifeWatch and EU BON, incorporate PESI components in their respective roadmaps and work plans to further the developments of a Taxonomic Backbone for Europe.

For establishing standards and sharing resources, PESI makes use of a huge network of taxonomic specialists in all European countries. Together with principal partners (like CETAF), PESI contributes to the objective on establishing an integrated taxonomic (working) community for Europe, operating as one virtual workforce representing a shared knowledge network.

In addition, PESI is selected by diverse EC bodies (like GÉANT) to outreach European biodiversity e-Infrastructures for the Eastern Partnership countries, thus extending the current geographic scope of the pan-European checklists, finally covering the whole Palearctica ("Flora/Fauna Palearctica").

PESI contributes to ongoing e-infrastructural developments, like VIBRANT and BioVeL, supporting the development of a shared and open virtual infrastructure to provide a more efficient interface between the existing biodiversity information infrastructures and stakeholders (policymakers, researchers, biodiversity managers).

Some instances of the above synergies and developments are highlighted below.

### PESI & INSPIRE

The INSPIRE directive establishes an infrastructure for spatial information in Europe to support community environmental policies and activities, which may have an impact on the environment, operated by the Member States of the European Union. As part of the INSPIRE Data Specification for the spatial data theme Species Distribution (Commission Regulation (EU) No 1253/2013), PESI is selected as a formal taxonomic standard for Europe, meaning that it is the first prioritised taxonomic reference classification to be used for data connected to species names.

### Toward a European Clearing House on taxonomic expertise

Part of the PESI future progress lies in its potential to further engage and organise the taxonomic community participation as a vital, virtual workforce including (i) the instalment of proper expert network governance, (ii) the application of proper ownership licences, (iii) the use of appropriate mechanisms for acknowledging expert contributions, (iv) the ability to function as an efficient 'knowledge hub', supporting biodiversity research and decision making more generally, (v) the successful involvement of more open and dynamically organised social networks and communities, like non-professional taxonomists and citizen scientists.

*Network governance*

Considering the overall decline of taxonomy as a scientific discipline, the maintenance of a basic (taxonomic) expertise capacity will be essential to satisfy the knowledge needs for a wide range of biodiversity-related information services in the near future, including the pan-European checklists. In PESI we moved forward from the efforts of EDIT on establishing a collaborative workforce, adding the integrated PESI expert networks into a single EDIT Expert Database and by defining more sophisticated social networking standards. To support the process of expert network integration, the SMEBD licensing model was applied. Further, the role of taxonomic institutes on the sustainable hosting of the taxonomic databases was studied (Costello et al. 2014) as well as the need for a federal facility, offering secretarial support on the expert network functioning.

A network of European leading taxonomic institutions forms the Consortium of European Taxonomic Facilities (CETAF), holding the majority of the worlds' biodiversity collections and their data. CETAF's mission contains the enhancement of Europe's knowledgebase capacity on taxonomy for a wide range of scientific and popular users (Price 2014). CETAF incorporates a legacy of past EC projects as working commissions and interest groups. CETAF has recognised the importance of data curation (CETAF 2004, CETAF 2008) and is interested to consider the set up of a special body to take care of the integrated management of the PESI taxonomic workforce, including the continuing hosting of the EDIT Expert Database, as a European Clearing House on taxonomic expertise.

*Accreditation and data papers*

An important vehicle for authoring metadata provenance, providing a clear recognition of all contributors and enabling the receipt of credits as a formal scientific publication by means of citation, are 'data papers'. Data papers also play an important role in the publication of small bits and pieces of information, like new distributions, which would otherwise be difficult to publish (termed 'micro-publication'). Data papers allow a more flexible/dynamic way of expert involvement within the process of data collation and reviewing, because contributions are more easily acknowledgeable than is currently the case. Finally, by using novel e-publishing tools, the process of manuscript drafting is highly automated, being more convenient for the editors, but also enabling a direct cross-indexing with other relevant metadata resources, supporting feedback mechanisms in foreseen name annotation workflows (see also 'next generation name infrastructure' subsection, below).

PESI already supports the publishing of data papers accompanying the WoRMS, Fauna Europaea and Euro+Med updating process (see 'Results'). More emphasis will be put on this work within the EU BON project, to trigger expert participation, to automate the dissemination and back-linking (tagging) of taxon names (within data papers) to external resources (Penev et al. 2010, Catapano 2010), and to support the evaluation of gaps in taxonomic information and knowledge (see 'e-Publishing' subsection, below).

*Networks of Knowledge*

As part of the Intergovernmental Platform on Biodiversity and Ecosystem Services (IPBES) developments and the Mapping and Assessing Ecosystems and their Services (MAES) process, the European Commission is exploring mechanisms to build so-called Networks of Knowledge (NoKs), to interact more broadly with the whole community of knowledge holders on biodiversity and ecosystem services, to inform decision making and strengthen the knowledge-policy interface. BiodiversityKnowledge (KNEU) is proposed as a governance structure for this NoK. PESI is involved in the development and testing of the BiodiversityKnowledge prototype, improving the knowledge flow between biodiversity knowledge holders and users in Europe, a process recently concluded (Suppl. material 28) and waiting for further H2020 application. A challenging issue in a Network of Knowledge involvement would be the implementation of the applicable sociology, motivating the actual experts participation in cross-disciplinary inventories and assuring a proper scientific reward system.

*Non-professional taxonomists and citizen scientists*

Currently around 50% of the experts contributing to the updating of the pan-European checklists includes professional taxonomists. However, the contributions of non-professional taxonomists will significantly increase in the near future. PESI should effectively anticipate this development by implementing relevant social networking mechanisms, including mentoring, educational and accreditation systems, in close collaboration with taxonomic institutes and societies.

An associated exercise is the adequate integration of efforts of voluntary biodiversity recorders, who provide a major contribution to the continuing monitoring of Europe's biodiversity. During PESI an ESF networking proposal was submitted entitled “Citizens Monitoring Biodiversity (CMD)” to optimise the involvement of the volunteer biodiversity observation community into the European biodiversity programs (Boumans and Jong 2009). This ESF proposal was awarded, but constrained by the formal country convergence criteria and needs a follow-up.

### Geographic extensions

Indexing biodiversity is a global challenge. PESI contributes to worldwide efforts on preparing global catalogues (like CoL) and supports relevant name services (like GBIF-ECAT) on increasing the resolving power for integrating biodiversity data.

More particularly, PESI has extended the pan-European checklists geographic scope by involving Focal Points from outer European Union territories, with the ultimate intention of covering the whole Palearctic (see 'Outlooks' section). The Palearctic is the largest biogeographic area of the world, containing a very rich and unique flora and fauna within a vastly diverse environment of unique habitats, especially in biogeographic transition and refuge zones regions, like the Caucasus. Integrating the available taxonomic expertise, data and resources into shared research infrastructures is crucial for globalising biodiversity assessments.

PESI partners from Eastern European countries, up to Russia and the Caucasus, are actively involved in carrying out aspects of the PESI work plan. To progress the participation of Caucasus partners a proposal was drafted to the European Commission FP7-INCO-2010-6 call for funding entitled "Network for Biodiversity Research in the Caucasus (NBRC): Developing the Biodiversity Research Centre in Tbilisi in a regional and international context" further building local capacities and integrating the scientific excellence and facilities for exploring the Caucasus Biodiversity Hotspot into the European Research Area. Unfortunately this proposal was not funded in this round.

Similarly, for the Mediterranean, a connection was made with national partners (Morocco, Algeria, Tunisia, Egypt) and biodiversity networks (BioNET-NAFRINET / ATUTAX) in Northern Africa (see: BioNET-NAFRINET 2010 LCC Meeting). Northern Africa is an area of great ecological importance as a conversion zone in between several biogeographical regions and as a passageway for migrating species. A proposal written to the JRS Biodiversity Foundation entitled "Networks for Biodiversity Indexing in Northern Africa contributing to EU-nomen" to extend the PESI work program toward Northern Africa was rejected for full funding, although travel grants have been applied for North African scientists to attend TDWG meetings, to become familiar with biodiversity informatics best practises and to share their experiences in doing this kind of work at home.

### Vernacular names and *Europeana*

Vernacular names are the most important search terms for non-professional users to retrieve biodiversity information. By means of the network of Focal Points (see 'Results' section), PESI collected substantial additional information on European species, including non-scientific names.

As part of the OpenUp! project, Opening Up the Natural History Heritage for Europeana (Berendsohn and Güntsch 2012), PESI participated in the development of an information infrastructure, harvesting vernacular names from various resources, to deliver suitable search terms and controlled vocabularies to advance the *Europeana* portal functioning, thus PESI serving as a meta-data repository for local biodiversity information, serving other initiatives.

### Virtualisation and Automation

PESI is participating in innovative biodiversity-informatics projects, developing and staging the ongoing virtualisation of the biodiversity research domain, building virtual tools and workbenches, including the collective use of data from multiple sources, and the automation of workflows of various tasks and processes.

The pan-European checklists have been applying automation for around 15 years since the initial versions of their data management systems. This has included implementing advanced virtual workbenches, including largely automated data-entry and data cleaning routines, which has eliminated a lot of manual processing. PESI continued this practice by installing automated routines for, among others, assembling the PESI Data Warehouse and for supporting the validation of checklists by means of the automated mapping tools (see Results).

**Figure 15.**
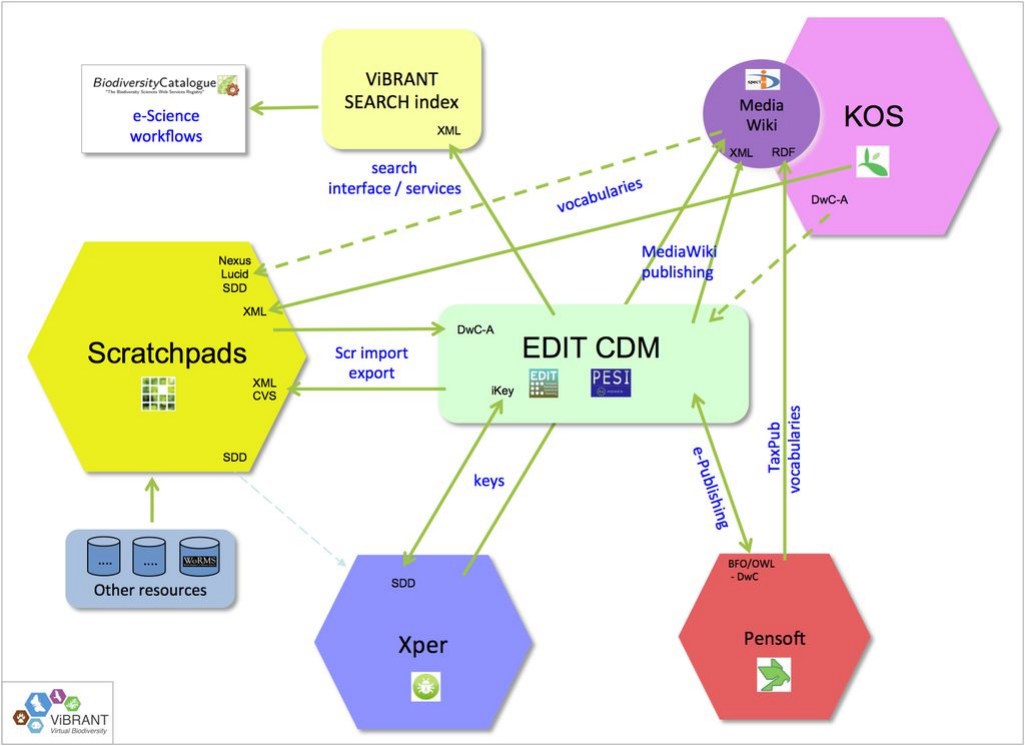
Aspects of increased biodiversity platform interoperability as established in the ViBRANT project (after: Suppl. material 29).

More efficiency in workflow automation is obtained when restrictions of distributed architectures are further reduced, enhancing cross-platform operationability. This requires a broad set of infrastructural adaptations, including the harmonisation and standardisation of APIs, data exchange formats and ontologies. As part of the ViBRANT project, PESI contributed to the development of a common publishing platform for taxonomists, driving the (virtual) integration of some major biodiversity information delivery platforms (see: Fig. 15). As a consequence, the reciprocal uptake of metadata standards and controlled vocabularies has been enhanced, as well as the use of other platform-specific services on running certain tasks, like e-Publishing and taxonomic key generation.

### PESI infrastructure potentials on a larger scale

In its original stage, the PESI infrastructure implemented two gateways to European biodiversity based on the taxon-level information provided by the participating checklists:

- a feature-rich web-portal offering convenient human-readable access
- a webservice layer, which has a small and effective set of methods for retrieving XML-encoded information that can be further processed by machines on any platform using any programming language.

Both webportal and webservices are optimised for a usage scenario with requests on individual objects (e.g. a particular name or taxon, information related to a particular identifier, etc.). However, we believe that PESI services will play an increasingly important role in workflow-driven systems. In this context, PESI will, for example, be used to expand a taxon name query to include its synonyms when combining independent scientific services that use different taxonomies. For this purpose several measures should be taken, including: (i) the extension of existing service layers supporting the retrieval of massive amounts of data, (ii) the optimisation of services for performance and reliability to ensure their usefulness in a workflow environment, and (iii) the optimisation of the PESI data warehouse structure for efficient output-oriented queries.

With these extensions, new workflow-oriented scientific applications can be realised and add further value to the PESI infrastructure. Both the EC-FP7 BioVeL and iMarine projects, in which PESI was represented, followed this approach towards a virtual biodiversity e-Library enabling pipelining of data and analysis into efficient integrated workflows (see subsection 'VREs', bellow).

Further European Taxonomic Backbone advancements are scheduled as part of the EU BON and LifeWatch projects:

In the EU BON project, *Building the European Biodiversity Observation Network*, the PESI Backbone will be advanced to satisfy the needs of the GEO BON / GEOSS system, also serving as a taxonomic backbone for the projected EU BON Biodiversity Portal. The European Biodiversity Portal will be developed to serve as a main information hub by providing integrated biodiversity data from different fields. The linked data will come both from in-situ as well as from earth observation data and the taxonomic backbone will be essential to link the data from different disciplines and locations. This process (so far) includes: (i) the completion of the Fauna Europaea and Euro+Med PlantBase migration and integration to the EDIT Platform for Cybertaxonomy (a process kicked-off in PESI), (ii) the registration of the third PESI Backbone version, as a EU BON taxonomic backbone prototype, to the respective GEOSS CRS and BiodiversityCatalogue services, (iii) the enhancement of relevant associated EDIT Platform functions, like the validation tools, and (iv) the harmonisation with other checklists, like the Catalogue of Life (CoL), FADA and WoRMS, to allow for federated (multiple checklist) searches and a unified data-model mapping (Suppl. materials 30, 31).

A complementary effort is foreseen in LifeWatch where, as part of the Flemish contribution to LifeWatch, the LifeWatch Taxonomic Backbone will be established, especially focussed on the marine environment. This LifeWatch Taxonomic Backbone will integrate various taxonomic checklists, including EU-nomen, and a range of associated biogeographical, ecological, genomic and literature data resources, supporting the LifeWatch infrastructure developments (Dekeyzer et al. 2014).

### Interactive Virtual Research Environments, portals and labs

Special cases of automated workflows are Virtual Research Environments (VREs), which will provide the next generation of shared research environments. Virtual labs provide an interactive environment in which researchers can access, collect, integrate and explore large amounts of data from multiple resources for analysis, data mining, and visualisation, supported by automated workflows and standardised web services.

PESI is involved in e-Science projects developing virtual labs for scientists (as part of BioVeL) and fishery agencies (as part of iMarine), guiding the accurate application of taxonomy and connecting the related web services, to accelerate the access, integration and application of taxonomic names as critical meta-data (Güntsch et al. 2014, Vanden Berghe et al. 2015). Virtual labs components are proposed to advance biodiversity web portal functions, allowing users a more direct and interactive environment for examining biodiversity data. This could include the extension of the species information, showing additional species features or occurence predictions obtained by executing customised analytical pipelines, which could be performed 'live' or in pre-analysis.

**Figure 16.**
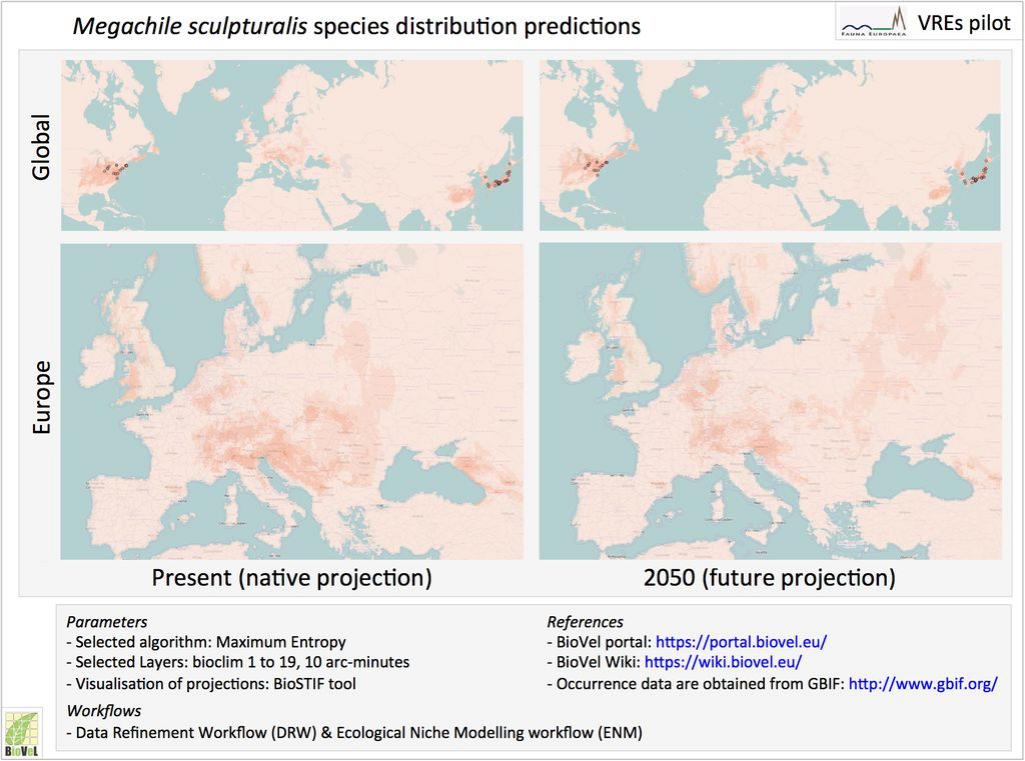
Example of virtual lab application (Fauna Europaea VRE pilot), showing the species distribution predictions of *Megachile sculpturalis*, using the BioVel Portal.

An example of how virtual labs could increase the analytical value of biodiversity portals is given in Fig. 16, showing the potential distributional capacity of *Megachile sculpturalis*, an invasive species in Piemonte (Italy), now also captured in Liguria (Quaranta et al. 2014). The species distribution predictions can be obtained very rapidly by operating the BioVel virtual labs. The results firstly shows that in general, compared to the source areas in the US and Japan, the circumstances are suboptimal for *Megachile sculpturalis* in Europe (which would show as deep red areas). Secondly it seems that the preferred habitat for *Megachile sculpturalis* in Europe is becoming smaller due to climate change over the next decennia. At least in Piemonte the species will probably disappear. Such insights, easily obtained from applying VREs, could be very important for all sorts of biodiversity managers and environmental controllers.

**Figure 17.**
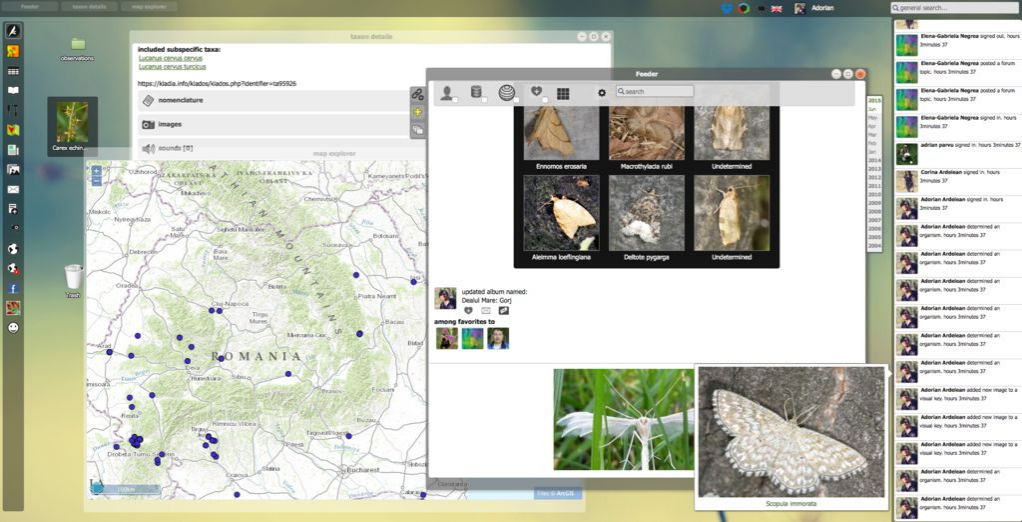
Screen capture of a biodiversity dedicated user interface in myBiOSis environment.

**Figure 18.**
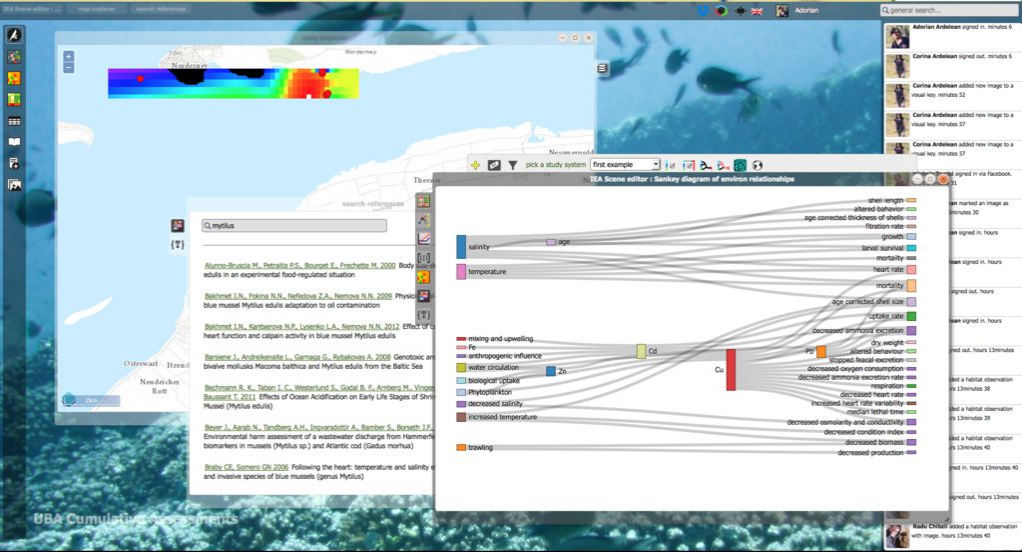
Screen capture of the Assessment toolkit in myBiOSis environment. This figure demonstrates flexibility of the system to accommodate distinct projects and research scopes.

Other instances of interactive environments can be found in advanced biodiversity data portals. As part of the Focal Points workplans, PESI stimulates the sharing of best practices on portal development, especially regarding taxonomic checklist governance, data exchange and sophisticated web tools. As an example, the Romanian PESI Focal Point receives dedicated support from the myBiOSis portal. The myBiOSis system allows an integrated view of a flexible selection of frequently-used web applications within a single user interface (template), thus easily accommodating the requirements of various projects and usertypes. Although the original myBiOSis modules are developed for recording species occurrences and phenotypic features (Fig. 17), a next step forward is the development of research toolkits (as Virtual Labs) that could take advantage of the accumulative body of biodiversity data, integrating local applications, as well as remotely distributed automated workflows, and supercomputing facilities. A prototype of such an interactive research environment, examining the cumulative impact of anthropogenic stressors on the ecosystems, including both environmental data as well as biotic interactions, is scheduled as part of a recently started UBA project (Fig. 18) (Eilers and Raabe 2015). Since cumulative impact assessments deal with present and near future scenarios, there is a good potential, not only in opening new research paths, but also in helping decision-makers to take effective measures in protection and conservation.

Similarly the Azores Bioportal (ABP) provides a regional e-infrastructure for the Azores Islands (Borges et al. 2010). ABP was the first Biodiversity Portal in Portugal, starting in 2008, offering easy access to island biodiversity data and inspiring the creation of a national equivalent e-infrastructure named PORBIOTA that will be funded between 2015-2020 (also in association with LifeWatch Europe). The ABP is a key e-infrastructure for the integrated management of biodiversity data of the Azores, delivering a large number of specialised services and tools supporting research, policy, education and nature conservation. International collaboration is well supported. As an example, data collated by the ABP project are relevant in contributing to the EU BEST Indicator Essential Biodiversity Variables for Islands for the novel IPBES platform and the Azorean and Madeira islands taxonomic checklists are validated against the pan-European checklists.

**Figure 19.**
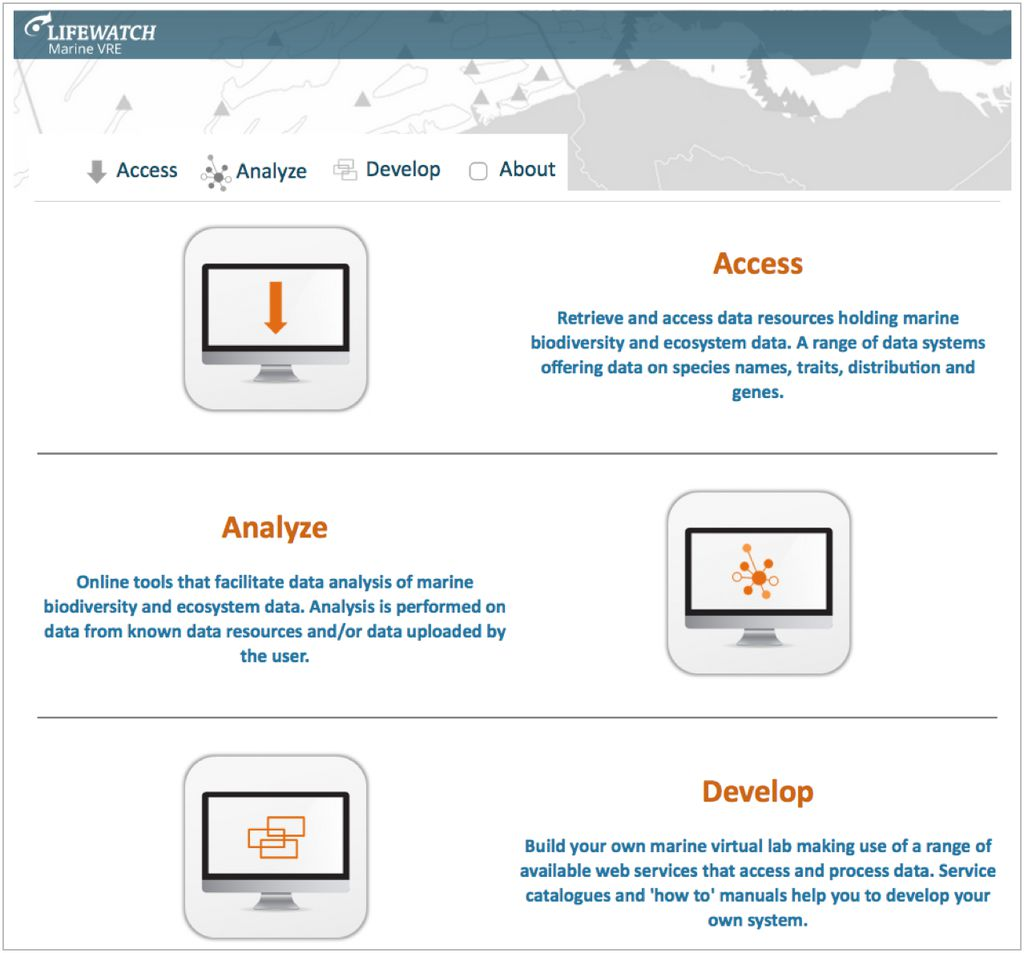
LifeWatch marine virtual research environment (VRE) (source: http://marine.lifewatch.eu).

A marine virtual research environment (VRE) has been created as the first operating component of the LifeWatch Research Infrastructure (ESFRI). It has been organised through a bottom-up approach from the participating states. The main services designed are: (1) VRE entry page & VRE components (virtual laboratories); (2) VRE calculation tools and biotic indices; (3) VRE derived products as base layers; (4) Biosensor data collaborative platform; (5) Taxonomic Backbone & species traits; 6) VRE training event & marine LW follow up meetings. The components that are strictly related to PESI are (2) and (5). The later is based on the taxonomic backbone developed in the course of WoRMS (World Register of Marine Species) and PESI, while the former on PESI and ViBRANT projects. Another innovative virtual laboratory under development is the one on the 3D representations of micro-CT scanning's of macro-organisms. This laboratory will offer a suit of galleries of interactive 3D representations allowing the user to have access to both morphological and anatomical representations. In its current version the Marine LW VRE is offered through a single portal: http://marine.lifewatch.eu. It offers three modules for its exploration and use: (1) Access; (2) Analyze; (3) Develop (see: Fig. 19).

## Project outlooks

Environments are changing rapidly all over the planet, making it imperative that we have our systems for communicating biological information working efficiently and reliably. Data based on proper identification of taxa are essential to monitor changes in nature; information needs to be integrated on the dynamics on species existence (migration, extinction, intrusion) and on instability of the associated ecosystems. Ongoing, critical environmental assessments are important to document and control critical disorders, like the decline of (native) pollinator species, the impact of algal blooms, the effect of overexploitation and the invasion of pest species. Taxonomy is a foundational science, but its reliable application is hindered by the limited knowledge of many aspects of biodiversity and the relative disorganisation and inaccessibility of taxonomic information. PESI contributes to the synthesis and access to existing taxonomic knowledge by maintaining a network of outstanding experts and by taking care about the delivery of persistent standards and data integration routines, securing a high-level access to biodiversity data.

The biodiversity community, in anticipation of the European Commission H2020 call for larger, more integrated networks, is: (i) pushing biodiversity research as an innovative information science (Hardisty 2013), by profiling biodiversity informatics as a joint movement (e.g. BIH2013, BIHorizons) and (ii) pushing the foundation of LifeWatch as an European Research Infrastructure Consortium (ERIC), and (iii) establishing large community networks contributing to the monitoring of biodiversity and the sharing of data, like GBIF and EU BON. PESI is on this roadmap (e.g., as "PESI Plus"), stressing the need for a sustainable, long-term initiative, taking care of the delivery of sustained taxonomic indexes and reference files, both globally and for Europe (Hussey et al. 2008). In addition, PESI supports the development and implementation of key informatics advancements, making scattered biodiversity data more readily available and useful, and transforming the current knowledge and understanding of biodiversity to a next level. The subsections below highlight some outlooks on trends and practices addressing the relevance and involvment of 'EU-nomen' components.

### Towards a next generation names infrastructure

*Annotation workflows*

**Figure 20.**
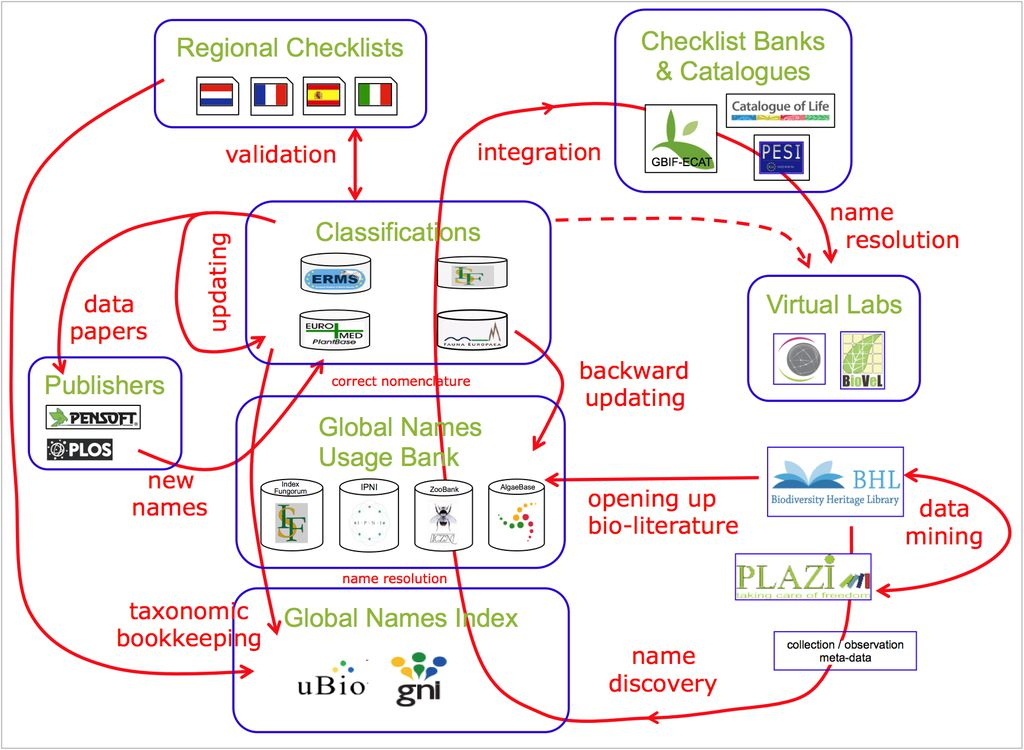
Potential workflows in a next generation linked open-data names architecture.

PESI contributes to the development of a next generation linked open-data names architecture, expanding the inter-platform operability and making the workflow orchestration and task automation more efficient between associated name services (Fig. 20). In the short term, this can result in: (i) the implementation of a common nomenclatural reference system (Pyle and Michel 2008), supporting the disambiguation of taxonomic information and advancing the common access to important resources, like taxonomic literature, and in (ii) the set up of an open taxonomic indexing system, supporting a communal book-keeping of taxon names, increasing the integration and resolution of taxonomic information (Patterson et al. 2010). PESI could profit from such achievements to re-organise some of its internal workflows, such as the management of data from distributed taxonomic resources (e.g., regional checklists), by increasing the portal functions, stretching out to additional external resources and information types, and by optimising the uptake of new taxa names (provided by publishers) as an integrated routine of the data editing process.

In the longer term a next generation names architecture is developing feedback mechanisms to automate the cross-annotation of different workflows using taxonomic information. This further virtualisation provides a number of advantages. Firstly, because most biological information (observations and knowledge) is linked to names, this will significantly increase the shared use and discovery of biological information. Secondly, because every virtual interaction provides a virtual documentation, this will enable the generation and accumulation of new 'information facts', finally resulting in a novel data ecosystem, also called 'big data' science.

However, virtualisation and automation aren't self-evident processes. They need a careful monitoring of the existing information environment and an exact knowledge on the amount of virtualisation and automation required, together with a defined strategy on the relevant infrastructural changes to be made. PESI is considering these steps, provisionally called *Global Names Architecture* (GNA), in close collaboration with important international contributors in this domain, like GBIF, CoL and Global Names.

*Global Names Achitecture & Global Names Usage Bank*

**Figure 21.**
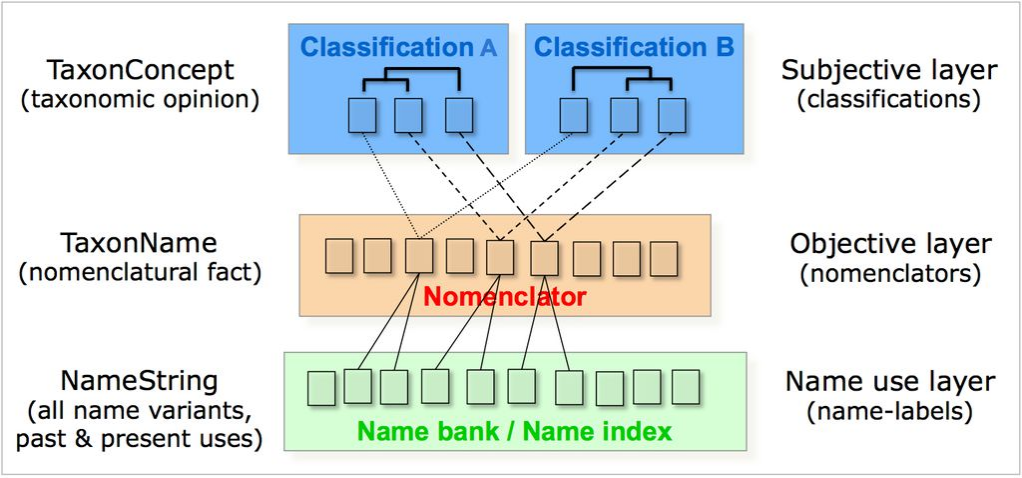
Global Names Architecture schematic representation of three cross-reference layers model.

Taxonomic decisions are based on consideration of data in an interpretive framework. The selection of what data are used, how it is weighted, and the philosophical framework for interpretation, all are individual choices made by the taxonomist. The choice is based on a taxonomist's skills, past experience, data availability, educational background, and the structure of diversity in the organisms under study. Thus, many taxonomists have a natural scepticism towards 'big data' attempts, as they see this a limiting their ability to make taxonomic judgements. However, in the case of a *Global Names Architecture* (GNA), taxonomy would benefit greatly from an infrastructure handling nomenclature (name description), science (postulating species concepts) and taxonomic practice (all names in use) as discrete systems (Fig. 21). Indeed, such a synthesis is the main hope for creating a more unified, testable taxonomic framework. EU-nomen will provide continuing support on establishing GNA, especially on developing a *Global Names Usage Bank* (GNUB), securing the further International Plant Names Index (IPNI), Index Fungorum (IF), AlgaeBase, and ZooBank implementations.

As an example, ZooBank is the official online nomenclatural registry for zoology, under the auspices of the International Commission for Zoological Nomenclature (ICZN). It is a registration system for new and legacy scientific names for animals, which comprise by far the greatest number of described taxa of any organismal group. PESI and ZooBank have had a closely paralleled, collaborative development (see also 'Results') and it is envisioned that they will become tightly linked in future perspectives.

After beginning in 2008 as a stand-alone system, like many other current nomenclatural and taxonomic sources, it was seen that ZooBank’s effectiveness would be exponentially increased if it were developed as a service within the *Global Names Architecture* (GNA), operating on top of the *Global Names Usage Bank* (GNUB, Pyle and Michel 2009). The new GNUB-based ZooBank was publicly launched on September 4, 2012 (coinciding with the amendment to the ICZN Code supporting electronic publication). It has been highly successful, increasing its growth ten-fold, with a current rate of 5000 entries/month. It is the most visible representation of the GNUB system.

**Figure 22.**
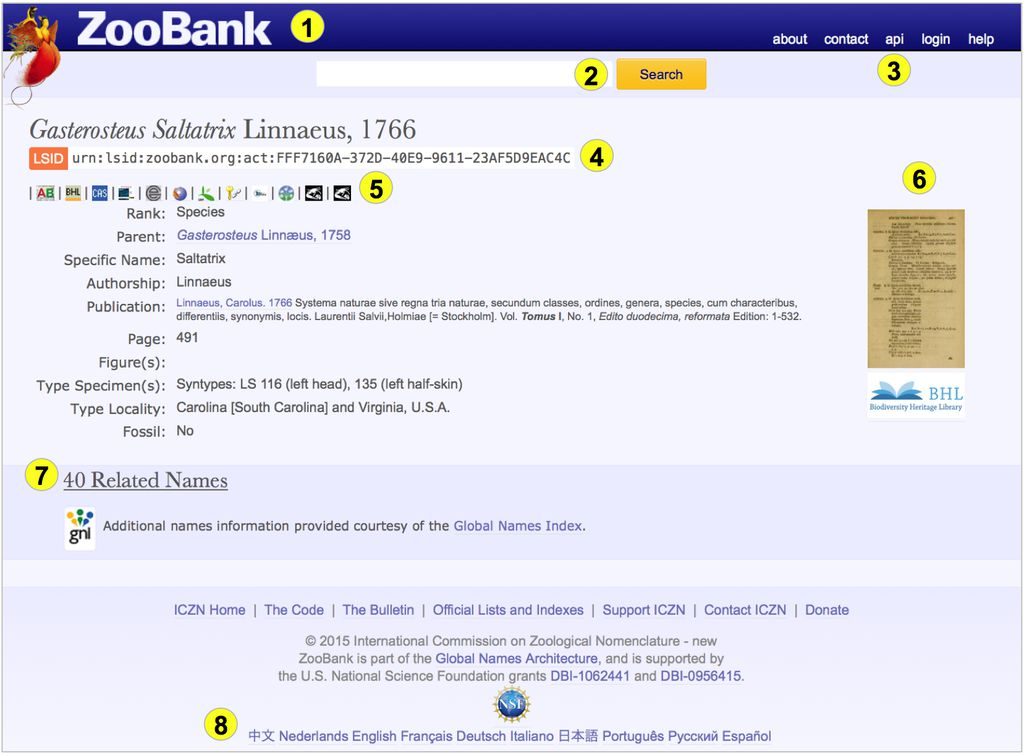
An example ZooBank page (*Gasterosteus Saltatrix* Linnaeus, 1766), illustrating several GNUB services: (1) user authentication; (2) “fuzzy” searching of GNUB content; (3) APIs and services; (4) ZooBank registration; (5) External Identifier cross-linking; (6) BHL page linking; (7) similar/related name discovery (via GNI’s name searching service); and (8) multi-lingual support. Not shown are services to manage user accounts, de-duplicate records, prototype reconciliation tools, services for journal publishers, and visualization tools for author publication history and other statistics.

ZooBank is the forerunner model for GNUB-based registration systems that can be developed in other nomenclatural domains. In addition there are many other services that GNUB can facilitate, leveraging the power of a robust code-based nomenclatural ‘skeleton’ and a usage-based ‘body’. Some of the GNUB services are indicated in the ZooBank interface shown in Fig. 22.

GNUB/GNA provides a single shared platform for all cross-links, such that anytime a record is indexed in GNA, it is automatically cross-linked to all other data systems that are indexed in GNA. Unlike most existing biodiversity data initiatives, the components of GNA (particularly GNUB) are not intended to provide novel information; rather, GNUB is an index of core facts that are shared across all of biology. Nothing in GNUB is original or novel content; it merely represents a structured way of organizing information to facilitate broader data integration among other databases that do contain original information. Thus, the GNUB index does not compete with other data resources; but rather serves as a core infrastructure for cross-linking (and thereby empowering) other biological data sources.

### e-Publishing perspectives

 Despite the traditional role of scholarly publications to serve as the primary vehicle for disseminating and reusing peer-reviewed scientific findings, it was recognised in PESI that scholarly publishing cannot persist anymore as just a method of communicating final results, because of the obstacle it is for efficient data sharing, reproducibility and reuse (see section e-Publications). Rather, scholarly publishing should become a part of the scientific process itself (Costello 2009, Costello et al. 2013, Smith et al. 2013, Piwowar and Vision 2013).

EU-nomen will contribute to the further integration of scholarly publishing services as an integral and interlinked part of the whole data gathering, data mobilisation and research process, synchronised with data sources, data aggregators, attribution and annotation services, and ontology frameworks. Technically this integration of the publishing and research processes will partly be achieved through a commonly developed and shared API library, based on community-agreed data exchange formats.

On expert engagement, a special role in the process will be played by the "data paper" concept as an important instrument of data mobilisation, publication and community involvement (Costello et al. 2013a,Chavan and Penev 2011). Next-generation data papers will be generated and submitted from metadata registries of data repositories to scholarly journals "at the click of a button", via APIs. Currently the only workflow of this kind is piloted by the Biodiversity Data Journal (BDJ), which has the option to submit complex manuscripts in XML through the API of the associated Pensoft Writing Tool (PWT).

### EU BON advancements

Because taxonomic information provides the primary identification of an object and is a prerequisite to make other biodiversity data discoverable and available, the evaluation of the state and trends of biodiversity is only possible with help of an elaborated taxonomy. Accordingly the EU BON project, by developing a webportal for enabling an integrated access to European biodiversity data (Hoffmann et al. 2014), recognise the importance of proper taxonomic metadata standards to evaluate common issues in biological research and conservation, for instance to generate Essential Biodiversity Variables (EBV) (Pereira et al. 2013), also supporting the improvement of policy reporting (Geijzendorffer et al. 2015), and therefore includes PESI as an essential part of its taxonomic backbone for the projects' portal services.

EU BON supports advances on the PESI work program, focussing on improving the pan-European terrestrial (Fauna Europaea and Euro+Med PlantBase) checklists quality and completeness. For this purpose the gaps in taxonomic data and knowledge will be further analysed with help of the connected expert networks, for instance as part of the Fauna Europaea data papers preparations. More fundamentally, technical solutions will be needed to advance the experts' data management tools (or 'virtual workbenches') with improved annotation functions, allowing easy review and uptake of discrepancies that might be found to occur between the pan-European checklists and other resources (like regional checklists, observation/monitor data or newly published names). This feature will increase the quality of the pan-European checklists data, speedingup the harvesting of all 'names in use' and close the 'delay gap' between the publication of new names and the actual integration into the pan-European checklists. Presumably, implementing such advanced taxonomic workbenches and publishing tools will also motivate experts to become more engaged in working on taxonomic databases and on editing, updating and annotating existing datasets.

### Towards a comprehensive taxonomic Palearctic survey

Already from the earliest stage, because of the extended geographic scope of the pan-European checklists, experts and institutes of European neighbourhood countries (including Russia) have been involved in the checklist work programs. Consequently, enhancing the range and collaboration towards a full Palearctic coverage follows the footprints of earlier projects, like Fauna Europaea, Euro+Med PlantBase, WoRMS, EDIT, PESI and other (international or local) initiatives. Such an extension seems inevitable, considering the practical necessity of assessing biodiversity as a borderless science and taking into account the European Union economic and political arguments, aiming a further exploitation of the potentials on cooperation in science, technology and innovation with outer-EU partners (Sonnenburg et al. 2012).

For two centuries, the Natural History Institutes in Eastern European and Central Asian countries have accumulated an extensive knowledge and huge collections on the flora and fauna of this wide region. The integration and implementation of this information into existing global or European biodiversity databasing initiatives is, however, inadequate because of a suboptimal use of shared virtual and social infrastructures. The proposed "Flora/Fauna/Mycota Palearctica" project will solve this impediment by intensifying the existing collaboration with *EU-nomen* partners, by extending the partnership towards Northern Africa and Central Asian countries, and by effecting a common work program, integrating available taxonomic resources into a uniform system and the associated experts into a corresponding network. The accumulated information, freely accessible for the world community, will significantly advance the global and regional capacity on (future) biodiversity assessments.

## Supplementary Material

Supplementary material 1 Contribution to the Global Name Architecture (GBIF/ECAT white paper) Data type: pdf
File: oo_44321.pdf Yde de Jong & David Remsen

Supplementary material 2 The European Taxonomic Work force (ETW), its tasks, activities and operational standards inspiration by the Open Source Society Data type: pdf
File: oo_43994.pdf Phillip Bøgh, Henrik Enghoff, Roisin Nash, Louis Boumans & Yde de Jong

Supplementary material 3 The Government of IPR of Electronic Biodiversity Data Data type: pdf
File: oo_43998.pdf Mark Costello, Roisin Nash, John Brophy, Louis Boumans, Henrik Ærenlund Pedersen, Javier Atalah, Ward Appeltans & Yde de Jong

Supplementary material 4 How to complete taxonomic gaps in the pan-­European species registers, including experts and informatics resources Data type: pdf
File: oo_44000.pdf Roisin Nash, Charles Hussey, Mark Costello, Ward Appeltans, Juliana Kouwenberg & Yde de Jong

Supplementary material 5 PESI workshop on linking taxonomic databases with online science journals Data type: pdf
File: oo_44002.pdf Mark Costello, Ward Appeltans, Bart Vanhoorne, Vishwas Chavan, David Remsen, Catriona MacCallum & Yde de Jong

Supplementary material 6 Design of a mechanism to keep control of the continuity of European electronic (taxonomic) biodiversity data resources and expertise networks Data type: pdf
File: oo_44001.pdf Roisin Nash, Julia Kouwenberg & Yde de Jong

Supplementary material 7 PESI Business Plan Data type: pdf
File: oo_44003.pdf Yde de Jong, Julia Kouwenberg & Olaf Banki

Supplementary material 8 The future of taxonomy – the role of national focal points networks in taxonomic information infrastructure networks Data type: pdf
File: oo_44012.pdf Yde de Jong & Eduard Stloukal

Supplementary material 9 PESI Focal Points Working Plan Data type: pdf
File: oo_44007.pdf Nihat Aktaç, Julia Kouwenberg, Louis Boumans & Yde de Jong

Supplementary material 10 PESI Focal Points Handbook Data type: pdf
File: oo_44008.pdf Nihat Aktaç, Julia Kouwenberg, Louis Boumans & Yde de Jong

Supplementary material 11 Report on authoritative taxonomic standards from multiple sources suitable for deployment within European Research Area. Data type: pdf
File: oo_44049.pdf Roger Hyam, Charles Hussey, Ward Appeltans, Julia Kouwenberg & Yde de Jong

Supplementary material 12 Report on Procedures and Mechanisms for the functioning of Nomenclators within the e-Infrastructure Data type: pdf
File: oo_44050.pdf Roger Hyam, Charles Hussey, Julia Kouwenberg & Yde de Jong

Supplementary material 13 The future of taxonomy – the role of GSD-networks and nomenclators in taxonomic information infrastructure networks (GNOMA). Data type: pdf
Brief description: Global Nomenclator Architecture (GNOMA) meeting on the contribution of nomenclators to the Global Names Architecture (GNA). ZooBank provisioning meeting on defining strategies for the uploading of ZooBank, especially on the contribution of taxonomic (zoological) key-resources.
File: oo_44047.pdf Yde de Jong, David Remsen, Elinor Michel, Nicola Nicholson & Paul Kirk

Supplementary material 14 The future of taxonomy – the role of GSD-networks and nomenclators in taxonomic information infrastructure networks (ZooBank). Data type: pdf
Brief description: Initial scoping meeting on GSDs and nomenclators involvement
File: oo_44048.pdf Yde de Jong, David Remsen, Elinor Michel, Nicola Nicholson & Paul Kirk

Supplementary material 15 Application and Adoption of Taxonomic Standards Data type: pdf
File: oo_44063.pdf Roger Hyam

Supplementary material 16 Report on the contributions to the set up of a Global Name Architecture Data type: pdf
File: oo_44064.pdf Roger Hyam, Charles Hussey & Yde de Jong

Supplementary material 17 Global Names Europe (GN-­EU) – a names based cyber-­‐infrastructure Data type: pdf
File: oo_44068.pdf Yde de Jong, Nicola Nicolson, Alan Paton, Paul Kirk, Ellinor Michel, Dauvit King, Donat Agosti & Lyubo Penev

Supplementary material 18 GN-EU – a names based cyberinfrastructure contributing to the Global Names Architecture developments as a necessary component of Research Data e-Infrastructures : Framework for Action in H2020 Data type: pdf
File: oo_44069.pdf Yde de Jong, Paddy Patterson, Rich Pyle, Nicola Nicolson & Paul Kirk

Supplementary material 19 PESI Report on the criteria, procedures and mechanisms for quality control Data type: pdf
File: oo_44209.pdf Anton Güntsch

Supplementary material 20 PESI joint e-infrastructure disseminating pan-European checklists Data type: pdf
File: oo_44210.pdf Anton Güntsch

Supplementary material 21 Versioning and the use of GUIDs for PESI Data type: pdf
File: oo_44211.pdf Anton Güntsch, Walter Berendsohn & Marc Geoffroy

Supplementary material 22 Working plan to support European GSDs maintenance and updating Data type: pdf
File: oo_45976.pdf Pascale Bezard-Falgas, David Ouvrard, Thierry Bourgoin & Yde de Jong

Supplementary material 23 Sustainability of European GSDs: Quantify financial and other resources to ensure long-term maintenance of European GSDs database systems Data type: pdf
File: oo_45977.pdf Pascale Bezard-Falgas, Thierry Bourgoin & Yde de Jong

Supplementary material 24 PESI web portal Data type: pdf
File: oo_44220.pdf Ward Appeltans, Bart Vanhoorne, Joram Declerck, Julia Kouwenberg & Yde de Jong

Supplementary material 25 Towards a Common Agenda on Prioritised Taxa Data type: pdf
File: oo_44318.pdf Louis Boumans, Julia Kouwenberg, Ward Appeltans & Yde de Jong

Supplementary material 26 Applying taxonomy to organise and deliver publications to biologists Data type: pdf
File: oo_44186.pdf David Remsen

Supplementary material 27 EDIT Scientific Publishing in Natural History Institutions 3nd meeting Data type: pdf
Brief description: Joint EDIT - PESI meeting on Scientific Publishing in NHIs
File: oo_44009.pdf Laurence Bénichou & Daphne Duin

Supplementary material 28 A recommended design for “BiodiversityKnowledge”, a Network of Knowledge to support decision making on biodiversity and ecosystem services in Europe Data type: pdf
File: oo_44375.pdf The consortium of the KNEU project, based on a broad European consultation

Supplementary material 29 ViBRANT: Design of robust services Data type: pdf
Brief description: Also available at: http://vbrant.eu/content/d43-design-robust-services
File: oo_44570.pdf Yde de Jong (ed.)

Supplementary material 30 Taxonomic backbone databases integrated with EDIT platform and EU BON portal [EU BON MS121] Data type: pdf
File: oo_45086.pdf Andreas Kohlbecker

Supplementary material 31 EU BON taxonomic backbone and services prototype integrated in EU BON portal [EU BON MS122] Data type: pdf
File: oo_45087.pdf Andreas Kohlbecker

Supplementary material 32 PESI Brochure Data type: pdf
File: oo_45625.pdf Roisin Nash, Louis Boumans, Juliana Kouwenberg, Ward Appeltans, Mark Costello, Yde de Jong

Supplementary material 33 PESI Flyer Data type: pdf
File: oo_45626.pdf Roisin Nash, Juliana Kouwenberg, Ward Appeltans, Yde de Jong

Supplementary material 34 PESI Focal Point Network description Data type: pdf
File: oo_57883.pdf Yde de Jong

Supplementary material 35 PESI Webstatistics Data type: xlsx
File: oo_49019.xlsx Yde de Jong & Bart Verhoorne

Supplementary material 36 PESI videoclip Data type: wmv
File: oo_57459.wmv VLIZ team (video) & Anton Güntsch (music)

Supplementary material 37 Coordination framework for grouped PESI focal points Data type: png
Brief description: Eight institutes commited themseleves to a coordinating role for Focal Point activities covering larger regions in the EU and adjacent countries if funding would become available. This figure gives a schematic presentation of this future effort to elaborate.
File: oo_57933.png Yde de Jong & Juliana Kouwenberg

## References

[B1640686] Bénichou L, Martens K, Higley G, Gérard I, Dessein S, Duin D, Costello MJ (2012). European Journal of Taxonomy: A public collaborative project in Open Access scholarly communication. Scholarly and Research Communication.

[B1648525] Berendsohn Walter G. (2010). Devising the EDIT Platform for Cybertaxonomy. Nimis P.L. & Vignes-Lebbe R. (ed.): Tools for identifying Biodiversity: Progress and Problems..

[B1648618] Berendsohn Walter, Güntsch Anton (2012). OpenUp! Creating a cross-domain pipeline for natural history data. ZooKeys.

[B1637121] Borges P. A.V., Gabriel R., Arroz A., Costa A, Cunha R., Silva L., Mendonça E., Martins A. F., Reis F., Cardoso P. (2010). The Azorean Biodiversity Portal: An internet database for regional biodiversity outreach. Systematics and Biodiversity.

[B1613428] Boumans L., Jong Y. de (2009). ESF proposal: “Citizens Monitoring Biodiversity” recommended for funding. EDIT Newsletter.

[B1632908] Catapano T (2010). TaxPub: An Extension of the NLM / NCBI Journal Publishing DTD for Taxonomic Descriptions..

[B1642943] CETAF (2004). Support for European Directory of Species Names (Memorandum of Understanding).

[B1642952] CETAF (2008). Position Paper on Biodiversity and Europe: the Contribution of Taxonomy and the European Taxonomic Facilities.

[B1633377] Chavan Vishwas, Penev Lyubomir (2011). The data paper: a mechanism to incentivize data publishing in biodiversity science. BMC Bioinformatics.

[B1648540] Ciardelli P., Kelbert P., Kohlbecker A., Hoffmann N., Güntsch A., Berendsohn W. G. (2009). The EDIT Platform for Cybertaxonomy and the taxonomic workflow: selected Components. Lecture Notes in Informatics (LNI) 154: 625-638.

[B1549543] Costello Mark (2000). Developing Species Information Systems: The European Register of Marine Species (ERMS). Oceanography.

[B1640845] Costello Mark J. (2009). Motivation of online data publication. BioScience.

[B1633348] Costello Mark J., Michener William K., Gahegan Mark, Zhang Zhi-Qiang, Bourne Philip E. (2013). Biodiversity data should be published, cited, and peer reviewed. Trends in Ecology & Evolution.

[B1607713] Costello Mark J., Appeltans Ward, Bailly Nicolas, Berendsohn Walter G., Jong Yde de, Edwards Martin, Froese Rainer, Huettmann Falk, Los Wouter, Mees Jan, Segers Hendrik, Bisby Frank A. (2014). Strategies for the sustainability of online open-access biodiversity databases. Biological Conservation.

[B1640657] Costello Mark J., Bouchet Philippe, Boxshall Geoff, Fauchald Kristian, Gordon Dennis, Hoeksema Bert W., Poore Gary C. B., van Soest Rob W. M., Stöhr Sabine, Walter T. Chad, Vanhoorne Bart, Decock Wim, Appeltans Ward (2013). Global Coordination and Standardisation in Marine Biodiversity through the World Register of Marine Species (WoRMS) and Related Databases. PLoS ONE.

[B1549563] Costello M. J. (2004). A new infrastructure for marine biology in Europe: marine biodiversity informatics. MarBEF Newsletter.

[B1549534] Costello M. J., Emblow C. S., White R. J. (2001). European register of marine species: a check-list of the marine species in Europe and a bibliography of guides to their identification.

[B1549573] Cuvelier D., Claus S., Appeltans W., Vanhoorne B., Vanden Berghe E., Costello M. J. (2006). European Register of Marine Species (ERMS) - plans turning into reality !. MarBEF Newsletter.

[B1629589] Dekeyzer S., Vandepitte L., Claus S., Deneudt K., Hernandez F., Naumov A., Sukhotin A., Martynova D., Berezina N., Berger N., Khalaman V. (2014). The LifeWatch taxonomic backbone: supporting the marine biodiversity and ecosystem functioning research community. A variety of interactions in marine environment.

[B1635961] Eilers S., Raabe T., Eilers S., Ardelean A., Raabe T., Duerselen C. -D., Burkhard B., Hertz-Kleptow C., Dierschke V., Hill K., Hill R. (2015). Developing a concept for the cumulative assessment of anthropogenic impacts with regard to the Marine Strategy Frame Directive (MSFD). Report on behalf of the German Federal Environment Agency (Umweltbundesamt), Project No. FKZ 3711 25 216.

[B1637885] Geijzendorffer Ilse R., Regan Eugenie C., Pereira Henrique M., Brotons Lluis, Brummitt Neil, Gavish Yoni, Haase Peter, Martin Corinne S., Mihoub Jean-Baptiste, Secades Cristina, Schmeller Dirk S., Stoll Stefan, Wetzel Florian T., Walters Michele (2015). Bridging the gap between biodiversity data and policy reporting needs: An Essential Biodiversity Variables perspective. Journal of Applied Ecology.

[B1635989] Greuter W., Raab-Straube E. von (2005). Euro+Med Notulae, 1. Willdenowia.

[B1623348] Güntsch Anton, Mathew Cherian, Obst Matthias, Vicario Saverio, Haines Robert, Williams Alan, Jong Yde de, Goble Carole (2014). A semi-automated workflow for biodiversity data retrieval, cleaning, and quality control. Biodiversity Data Journal.

[B1648605] Hagedorn Gregor, Mietchen Daniel, Morris Robert, Agosti Donat, Penev Lyubomir, Berendsohn Walter, Hobern Donald (2011). Creative Commons licenses and the non-commercial condition: Implications for the re-use of biodiversity information. ZooKeys.

[B1630995] Hardisty Alex (2013). Horizon 2020: A call to forge biodiversity links. Nature.

[B1637397] Hoffmann Anke, Penner Johannes, Vohland Katrin, Cramer Wolfgang, Doubleday Robert, Henle Klaus, Kõljalg Urmas, Kühn Ingolf, Kunin William, Negro Juan José, Penev Lyubomir, Rodríguez Carlos, Saarenmaa Hannu, Schmeller Dirk, Stoev Pavel, Sutherland William, Tuama Éamonn Ó, Wetzel Florian, Häuser Christoph L. (2014). The need for an integrated biodiversity policy support process – Building the European contribution to a global Biodiversity Observation Network (EU BON). Nature Conservation.

[B1607288] Hussey C., Jong Y. de, Remsen D. (2008). Actual usage of biological nomenclature and its implications for data integrators;a national, regional and global perspective. Zootaxa.

[B1546377] Jong Y. de (2011). Standardising Taxonomic Data – The backbone of Europe's biodiversity management. International Innovation - Environment.

[B1549410] Jong Yde de, Verbeek Melina, Michelsen Verner, Per Place Bjørn de, Los Wouter, Steeman Fedor, Bailly Nicolas, Basire Claire, Chylarecki Przemek, Stloukal Eduard, Hagedorn Gregor, Wetzel Florian, Glöckler Falko, Kroupa Alexander, Korb Günther, Hoffmann Anke, Häuser Christoph, Kohlbecker Andreas, Müller Andreas, Güntsch Anton, Stoev Pavel, Penev Lyubomir (2014). Fauna Europaea – all European animal species on the web. Biodiversity Data Journal.

[B1623808] Patterson D. J., Cooper J., Kirk P. M., Pyle R. L., Remsen D. P. (2010). Names are key to the big new biology. Trends in Ecology & Evolution.

[B1632879] Penev Lyubomir, Agosti Donat, Georgiev Teodor, Catapano Terry, Miller Jeremy, Blagoderov Vladimir, Roberts David, Smith Vincent S, Brake Irina, Ryrcroft Simon, Scott Ben, Johnson Norman F, Morris Robert A, Sautter Guido, Chavan Vishwas, Robertson Tim, Remsen David, Stoev Pavel, Parr Cynthia, Knapp Sandra, Kress W. John, Thompson F. Chris, Erwin Terry (2010). Semantic tagging of and semantic enhancements to systematics papers: ZooKeys working examples. ZooKeys.

[B1637942] Pereira HM, Ferrier S, Walters M, Geller GN, Jongman RHG, Scholes RJ, Bruford MW, Brummitt N, Butchart SHM, Cardosa AC, Coops NC, Dullloo E, Faith DP, Freyhof J, Gregory RD, Heip C, Hoeft R, Hurtt G, Jetz W, Karp DS, McGeoch MA, Obura D, Onoda Y, Pettorelli N, Reyers B, Sayre R, Scharlemann JPW, Stuart SN, Turak E, Walpole M, Wegmann M (2013). Essential biodiversity variables.. Science.

[B1633338] Piwowar Heather A., Vision Todd J. (2013). Data reuse and the open data citation advantage. PeerJ.

[B1642923] Price Michelle J. (2014). The Consortium of European Taxonomic Facilities (CETAF): Exploring and Documenting Diversity in Nature. Taxon.

[B1605059] Pyle RL, Michel E (2008). ZooBank: Developing a nomenclatural tool for unifying 250 years of biological information.. Zootaxa.

[B1642899] Pyle R., Michel E. (2009). Unifying nomenclature: ZooBank and Global Names Usage Bank. Bulletin of Zoological Nomenclature.

[B1623363] Quaranta Marino, Sommaruga Angelo, Balzarini Patrizia, Felicioli Antonio (2014). A new species for the bee fauna of Italy: *Megachile
sculpturalis* continues its colonization of Europe. Bulletin of Insectology.

[B1635999] Raab-Straube E. von, Raus T. (2013). Euro+Med-Checklist Notulae, 1. Willdenowia.

[B1633316] Smith Vincent, Georgiev Teodor, Stoev Pavel, Biserkov Jordan, Miller Jeremy, Livermore Laurence, Baker Edward, Mietchen Daniel, Couvreur Thomas, Mueller Gregory, Dikow Torsten, Helgen Kristofer M., Frank Jiři, Agosti Donat, Roberts David, Penev Lyubomir (2013). Beyond dead trees: integrating the scientific process in the Biodiversity Data Journal. Biodiversity Data Journal.

[B1637685] Sonnenburg J., Bonas G., Schuch K. (2012). White Paper on opportunities and challenges in view of enhancing the EU cooperation with Eastern Europe, Central Asia and South Caucasus in Science, Research and Innovation..

[B1640469] Vanden Berghe Edward, Coro Gianpaolo, Bailly Nicolas, Fiorellato Fabio, Aldemita Caselyn, Ellenbroek Anton, Pagano Pasquale (2015). Retrieving taxa names from large biodiversity data collections using a flexible matching workflow. Ecological Informatics.

