# Supplementary material for: PESI - a taxonomic backbone for Europe
Source: Biodivers Data J. 2015 Sep 28;(3):e5848. doi: 10.3897/BDJ.3.e5848 (PMC4609752; doi:10.3897/BDJ.3.e5848)
Supplement: Supplementary material 1 — Contribution to the Global Name Architecture (GBIF/ECAT white paper) [file biodiversity_data_journal-3-e5848-s001.pdf]

Project no. 018340

Project acronym: EDIT

Project title: Toward the European Distributed Institute of Taxonomy

Instrument: Network of Excellence

Thematic Priority: Sub-Priority 1.1.6.3: "Global Change and Ecosystems"

C3.2.1 Contribution to the Global Name Architecture (GBIF/ECAT white paper)

Due date of component: Month 25
Actual submission date: Month 25

Start date of project: 01/03/2006 Duration: 5 years

Organisation name of lead contractor for this component: 5 UvA

Revision 1

**Project co-funded by the European Commission within the Sixth Framework Programme (2002-2006)**		
**Dissemination Level ("X" in the relevant box)**		
**PU**	Public	X
**PP**	Restricted to other programme participants (including the Commission Services)	
**RE**	Restricted to a group specified by the consortium (including the Commission Services)	
**CO**	Confidential, only for members of the consortium (including the Commission Services)	

C3.2.1 Contribution to the Global Name Architecture (GBIF/ECAT white paper)

Authors
Revision 1: Yde de Jong & David Remsen

Introduction

The GBIF Strategic Plan calls for the creation of a federated ECAT 'catalogue' implying a dedicated and discreet centrally available electronic list of names applied to the organisms of the Earth. Significant proportions of these names remain unavailable to the GBIF network yet and must be made available via a distributed provider network. Since ECAT resources are limited, to establish global and regional capacity, context and value, the decentralized GBIF network requires a close collaboration with other (global and regional) biodiversity initiatives. The ECAT-supported infrastructure that facilitates the inventorying, maintenance, creation, integration, and serving of taxonomic resources for regional and global access is not only mobilized within the GBIF Nodes, but also available through liaison with regional (e.g. EC or NSF funded) programs.

Additionally, when both a metadata registry of data providers and an integrated ECAT catalogue exists, a key-functionality of the ECAT Global Name Architecture should be the creation of a name resolving facility to mediate for queries across interoperable biological data sources, addressing the taxonomic "names problem" by unambiguous cross-linking all sorts of "uses of names". This service application for outreach beyond the GBIF core within the cyber-taxonomy domain, like EoL, SpeciesBase, EDIT and LifeWatch.

By addressing relevant activities and elements within the ECAT Work Programme[1], this document would like to explore the possibilities for collaboration between GBIF/ECAT and EDIT on the discovery, integration, and sharing of taxonomic (meta-)data for all European organisms and expertise resources, including the contribution to the set up of a common name resolving facility that could serve as a taxonomic backbone, cross-referencing taxonomic names and concepts, to mediate in cyber-taxonomic processes that draw upon the collaborative potential of the web. Preferably this will result in a Memorandum of Understanding (MoU) between EDIT and GBIF.

1 GBIF Work Programme 2009-2010, version 3 April 2008.

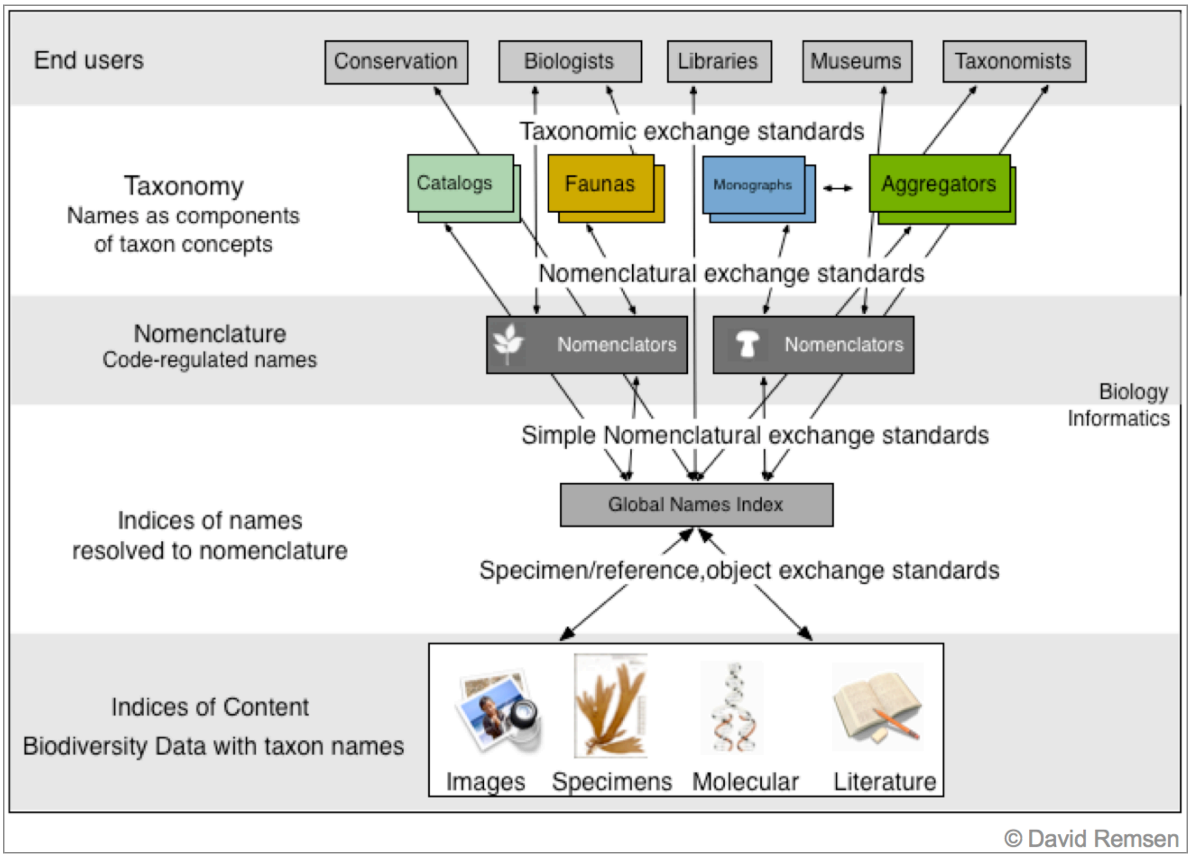

Figure 1: Outline of the GBIF/ECAT Global Name Architecture (GNA) conceptual layers.

Activity 1: Develop standard interfaces/methodologies and tools for data sharing

The foundation of the ECAT programme is dependant upon standardized tools and methodologies being deployed and adopted by existing and potential data providers. This capacity is in its infancy as compared to parallel developments focus on specimen data.

With partners, ECAT will develop and design methods and tools to enable the discovery, mobilization, and access to taxonomic names data. This includes the identification and promotion of interfaces with existing content providers and the development of toolsets to engage new data providers.

- EDIT will contribute to the coordination and promotion of standard methods and exchange formats, tools and infrastructures, by supporting the involvement of EU-based key taxonomic data providers and attributed project staff at relevant workshops to define the Global Nomenclature Architecture (GNA) requirements. [liaison: *EDIT WP3.2*]
- Moreover, supplementary to the GBIF nodes program and other associated networks, EDIT will contribute to the set up of action plans to ensure the sustainable engagement of EU-based key taxonomic databases to the developed GNA data sharing interfaces and to identify and target additional, major classes of potential European taxonomic names providers. [liaison: *EDIT WP3.2*]

Activity 2: Implement standard interfaces against key targeted databases and explore the use of standards within cybertaxonomic processes

Considering the availability of relevant tools and methods developed in Activity 1, this Activity focus on the implementation of standard data sharing interfaces among important taxonomic and nomenclatural aggregators and databases and to support the development of tools and methods that embed these standards into cybertaxonomic practices.

- EDIT will contribute to the commission of an inventory identifying and prioritizing primary nomenclatural zoological data resources to facilitate the uploading (nomenclatural content development) of ZooBank. [liaison: *EDIT WP3.2*]
- EDIT will contribute to the coordination of the implementation of standards-based interfaces with targeted EU-based taxonomic names providers. [liaison: *EDIT WP3.2*]
- EDIT will explore the use of standards-based interfaces within EU-based cybertaxonomy initiatives (like web-based taxonomy) and cybertaxonomy platform developments. [liaison: *EDIT WP5 & W6*]

Activity 3: Address social barriers to sharing taxonomic names data

The mobilisation, liberation and (re-)use of taxonomic data sources by initiatives developing federated, data exchange networks is increasingly hindered by non-technical aspects. This includes lack of (proper) attribution and other IPR violating issues. This activity will address the concerns of providers on sharing data resources and will develop a set of recommendation for the GBIF roadmap to solve this impediment that will be used for the design of a technical attribution resolution model for implementation.

- EDIT will contribute to the development of a set of recommendations by involving the *Society for the Management of Electronic Biodiversity Data* (SMEBD) efforts and by linking to the EDIT web-based taxonomy IPR work program. [liaison: *EDIT WP2 & W6*]

Activity 4: Implement an ECAT metadata repository

A critical component of a federated network of data resources mobilized to assemble a new, collective resource, is a registry that identifies the existing or potential contributing parts. Therefore a dynamic and standards-based metadata registry will be needed to accurately describe the different classes of resources that serve the priorities of ECAT in various ways. This registry would serve as the collective access point for a dynamic ECAT catalogue of names (Activity 5) as well as the basis for resource discovery for partnering institutions and initiatives.

GBIF will develop a scalable plan, including a consensus metadata profile and standardised LSID assignment, incorporated into a shared technical implementation that could ultimately be mirrored and federated for use by other initiatives sharing the resource load.

- EDIT will contribute to the population of metadata of European based taxonomic resources. [liaison: *EDIT WP3.2*]
- EDIT will contribute to the development of a consensus metadata profile for taxonomic names resources and the design of a LSID implementation plan. [liaison: *EDIT WP5*]

Activity 5: Index resources within an integrated ECAT data catalogue

Based on the established data sharing tools and interfaces (Activities 1 & 2) and the metadata registry (Activity 4), this activity will deal with the actual harvesting of taxonomic data into the ECAT index representing a centrally available electronic list of names applied to the organisms of the Earth that represents "as much scientific opinion on taxonomy as possible and implement and display this in a form that presents all extant, presumably valid, opinions in a clear and useful framework[2]". This includes the development of intuitive and effective human and machine accessible interfaces to this index and the enabling of annotation and caching of persistently identifiable concepts and access to annotations by providers.

- In addition to the taxonomic resource mobilisation efforts of Activities 1 and 2, EDIT will contribute to the coordination of the harvesting of targeted EU-based taxonomic names providers. [liaison: *EDIT WP3.2*]
- EDIT will contribute to the engagement of a wider sector of the European expert taxonomic community to support the scoping of the user needs and to address and help with the filling of potential taxonomic gaps when relevant. [liaison: *EDIT WP2*]

Activity 6: Develop software that enables interchange among indexed resources

A collective index of taxonomic and nomenclatural data will reveal overlaps, conflicts, and quality differences that can only be revealed through a collective assessment of resources. Validating and cross-referencing names originating from one source data against other known sources requires the development of (algorithmic and data management) tools, methods, and

---

2 GBIF Third-Year Review, 46.

services that enable the effective integration of indexed names content in the electronic catalogue. This resolution function includes the identification and reconciliation of name variants (e.g. orthographs) to an authoritative (nomenclatural) taxonomic identifier.

Furthermore, to ensure the established consensus taxonomic backbone serves as a potential organizational framework for data mediating, a consensus management classification as well as standardized thesauri (authority files) on for instance author names, and citations formats needs to be developed.

- EDIT will contribute to the effective integration of indexed names content in the ECAT electronic catalogue, by supporting the integration and cross-referencing of relevant prioritised European taxonomic authority files, in particular the pan-European checklists, to the consensus global checklist. [liaison: *EDIT WP3.2*]
- EDIT will contribute to the engagement of a wider sector of the European expert taxonomic community to assist with orthographic and nomenclatural assertions, and to support the set up of a consensus management classification. [liaison: *EDIT WP2*]

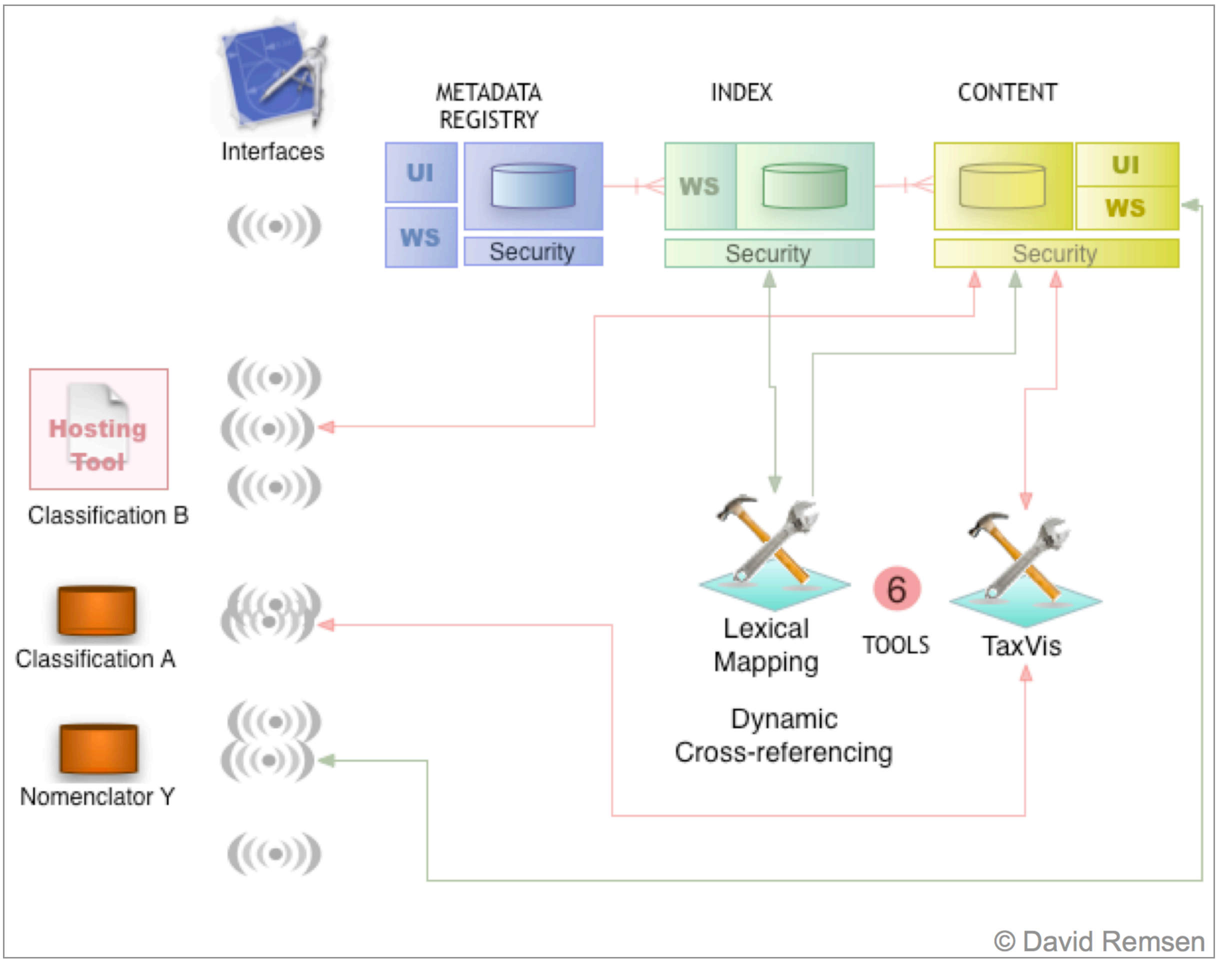

Figure 2: Outline of the Global Name Architecture showing the placement of ECAT Work Plan Activity 6. The mentioned 'content' component includes a service mapping taxon concepts.

Activity 7: Develop tools and interfaces to construct integrated catalogue(s) based on ECAT-mediated indexed resources

Since social networking capacity is growing rapidly on the web and is serving as a framework for building collaborative workbench *e*-environments, this activity will utilize the collaborative powers of web-enabled taxonomy to allow a network of expert curators to maintain and refine a web-based taxonomic checklist. The supporting infrastructure will ensure that the resultant data is accessible through open standards.

- EDIT could contribute to this effort, by promoting the use of the developed web-based workbench environments for the continued maintenance and curation of checklist data by one or more expert(s) within their social networks [liaison: *EDIT WP2*] and by ensuring a proper implementation within the cybertaxonomy platform [liaison: *EDIT WP5 & W6*]
- EDIT will study the relevance of the developed web-based workbench environment for the long-term maintenance of the pan-European checklists databases. [liaison: *EDIT WP3.2*]

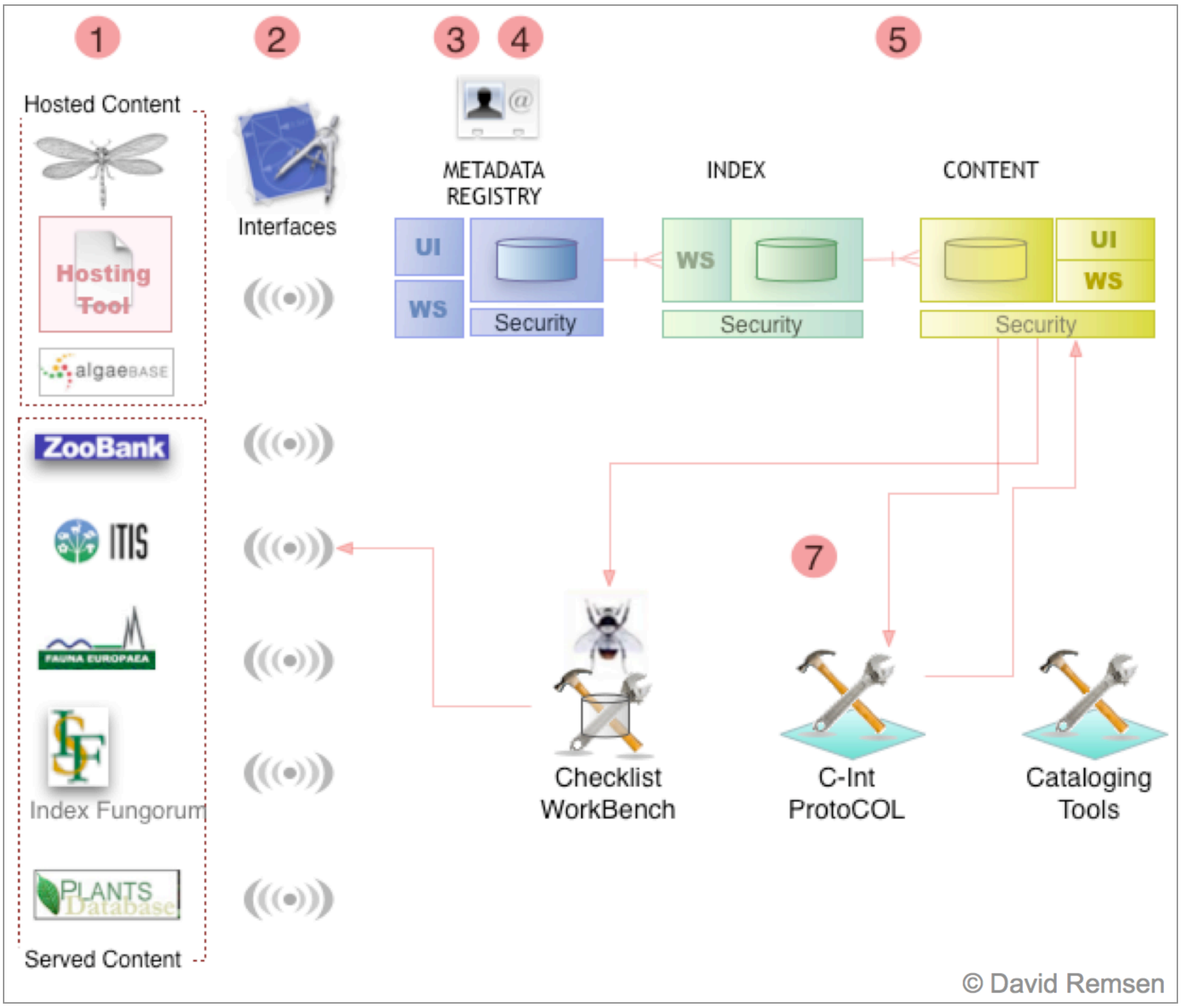

Figure 3: Outline of the Global Name Architecture showing the placement of several ECAT Work Plan Activities.

Activity 8: Develop tools and methods for web sites to exploit constructed catalogue(s) and ECAT-mediated resources for their own organizations and uses.

GBIF has a vision[3] to be "the primary Web-wide source for all data and information about biodiversity through building the registries, indices and mapping tools to allow searching across interoperable sources of information from molecules to ecology". The electronic catalogue is a key to this. This activity would like to demonstrate the utility of the electronic catalogue and refine methods that enable external organizations to utilise the derived value within their own organisations. This goal will be facilitated through the use of common services and intelligent collation of sources within the catalogue that enable catalogue resources to be incorporated and embedded into remote organizations and sites. This requires the assembly of services and tools that are directed toward this level of integration.

- EDIT will study the outreach of the ECAT potential to their own domain of envisioned portal services and associated expertise communities. [liaison: *All EDIT WPs*]

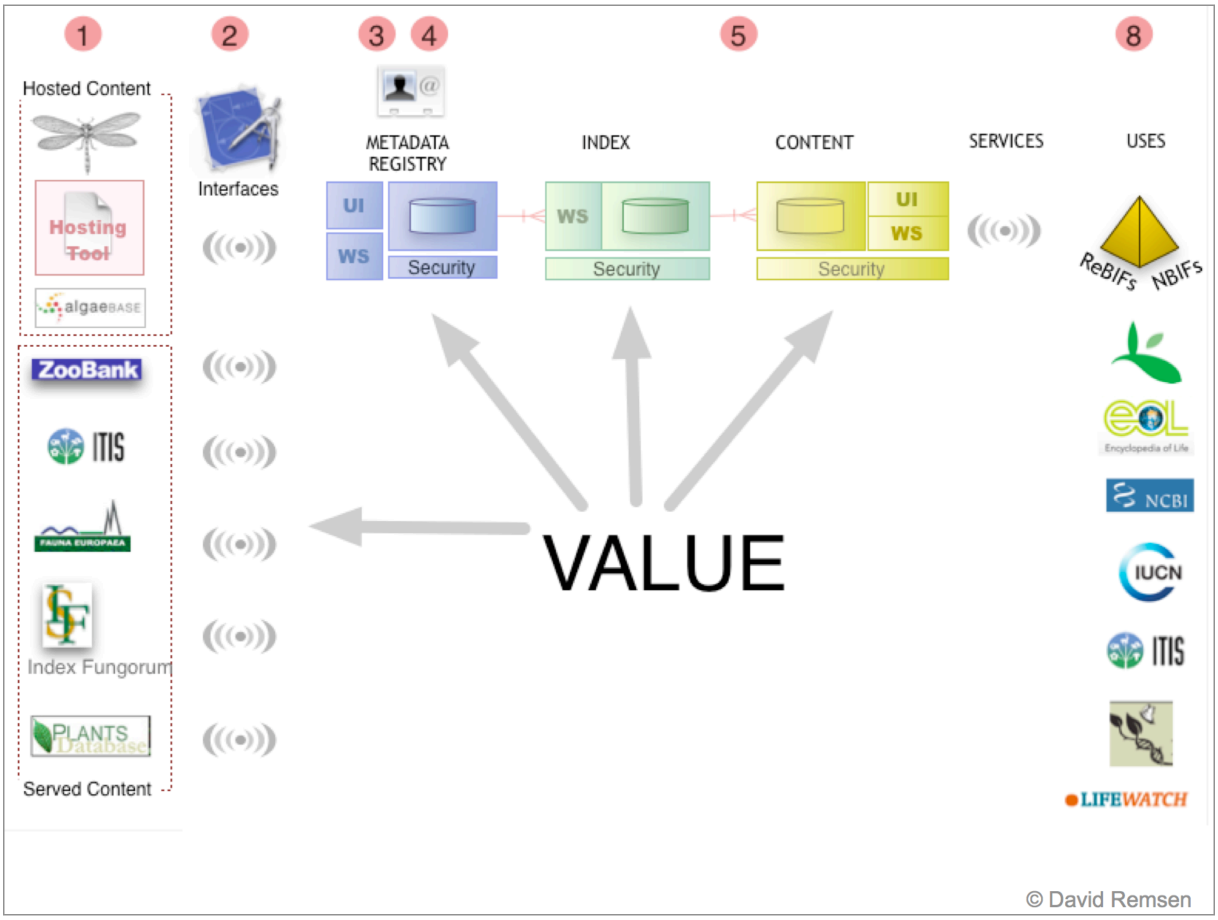

Figure 4: Outline of the Global Name Architecture showing the possible outreach of the ECAT catalogue (Activity 8).

3 Strategic Plan 2007-2001/Content/Goal A/Activity 4.
