# Supplementary material for: PESI - a taxonomic backbone for Europe
Source: Biodivers Data J. 2015 Sep 28;(3):e5848. doi: 10.3897/BDJ.3.e5848 (PMC4609752; doi:10.3897/BDJ.3.e5848)
Supplement: Supplementary material 2 — The European Taxonomic Work force (ETW), its tasks, activities and operational standards inspiration by the Open Source Society [file biodiversity_data_journal-3-e5848-s002.pdf]

The European Taxonomic Work force (ETW), its tasks, activities and operational standards inspiration by the Open Source Society

Contents

1 The European taxonomic workforce 2
1.1 What is the European Taxonomic Workforce (ETW)? 2
1.2 How is the ETW organised? 3
1.2.1 Organisation of career taxonomists in their institutions 5
1.2.2 Taxonomic projects and their organisation 6
1.2.3 Taxonomic societies and other networks 6
1.3 Why should the ETW be better organised? 7
1.4 How should the ETW be better organised? 9
1.4.1 The present Taxonomic community 9
1.4.2 Open Source Taxonomy (OST) procedures 10
1.4.3 European expert recruiting and networking methods 13
1.5 What is the PESI taxonomic taskforce (PESI-TT) 27
1.6 What are the tasks of the PESI-TT? 27
1.7 How is the PESI-TT organised? 27
1.7.1 Fauna Europaea 28
1.7.2 Euro+Med PlantBase 30
1.7.3 European and World Registers of Marine Species 30
1.8 How should the PESI-TT be better organised? 31
1.8.1 Challenges of the PESI-TT 31
1.8.2 Suggested work flow for input to checklists 32
1.8.3 Expansion of the network 34
1.8.4 An Integrated Virtual Expert Network system 35
2 Accreditation system for PESI-ETW 39
2.1 Status, Merit and Inclusiveness 39
2.2 PESI citation system for online data 40
2.3 Expert competency clarification through certification 41
3 Action plan 42
3.1 Management of the PESI ETW 42
3.2 Motivating contributors 43

1 The European taxonomic workforce

1.1 What is the European Taxonomic Workforce (ETW)?

Shortly and broadly, the European taxonomic workforce (ETW) consists of all people who do taxonomic work in Europe. i.e. the scientific classification of all living organisms on earth and their relationships. The term *taxonomy* is used in a wide variety of contexts; here only taxonomy applied to living and extinct organisms is considered.

Taxonomists can be grouped into five categories according to their activities (Enghoff & Seberg 2006):

i. Alpha taxonomists: recognise, describe, name, revise and synonymise taxa.
ii. Beta taxonomists: compare and classify taxa, construct phylogenetic relationships, based on shared characters
iii. Gamma taxonomists: study intraspecific variation.
iv. Tool makers: construct identification keys, morphological or DNA barcodes, and other taxonomy related tools.
v. Tool users: identify specimens, make inventories, monographs

Most taxonomists engage in several taxonomic activities and thus fall into more than one of the five listed categories. Furthermore, because species descriptions may include distinguishing features, morphology, habitat, geographic distribution, physiology, and genetics, taxonomists may become involved in ecology and biogeography. An individual taxonomist can be described by at least two sets of parameters. One set has to do with their work:
→ the types of activities she performs (see above),
→ the group(s) of organisms she is interested in,
→ the geographical area(s) she focuses on,
→ the ecosystems she focuses on (marine, freshwater, forest, caves ....),
→ the methods s(he) employs

A quite different set of parameters has to do with the person: Is she doing her taxonomic work as part of her career or not? Has she received professional training in taxonomy, e.g., does she have a relevant degree in biology? For how many years to come can she be expected to be part of the ETW?

A very large part of the taxonomic work done in Europe is done by persons whose job it is to do something else than taxonomy. This group of taxonomists includes true amateurs, but also retired professionals and professional biologists who do some taxonomy although their main professional task is different. The diversity of taxonomists' personal backgrounds was addressed at a recent workshop on "Professionals and non-professionals as producers and users of taxonomic knowledge – synergies, conflicts and the role of taxonomic societies". In the workshop report (EDIT 2008a), a discussion is summarized which "developed about the terminology professionals and non-professionals which the workshop organisers had applied in the workshop handouts. Alternatively, the terminology *amateur taxonomists* versus *expert taxonomists*, *scientists* versus *citizen scientists*, *taxonomists* versus *para-taxonomists*, or single terminologies like *naturalists*, *private taxonomist* or the wording *biological recorder* were mentioned". After the workshop, a simple terminology, viz., *career taxonomists* and

non-career taxonomists, inspired by an unpublished essay by R. Mesibov[9] will be employed throughout this report to avoids value statements.
Non-career taxonomists play a very large and probably increasing role in the ETW. This is evident from the statistics presented by Fontaine (no date) that showed that more than half of the new European non-marine animal species described during the period 1998-2003 were described by non-career taxonomists.

Because of the loose organisation of the ETW (see below), its tasks are defined in a mainly bottom-up way. Non-career taxonomists do what they like (as implicit in the word *amateur*), and although the work of career taxonomists is to a variable degree governed by local research policies, these are rarely coordinated at a national level, and certainly not at a European level. If we look at the five taxonomic activities listed above, there is a strong difference between the extent to which career and non-career taxonomists engage in them. Many more of the latter are involved in using identification guides and inventorying species, whereas beta- and gamma-taxonomy are dominated by career taxonomists. The often enormous effort by non-career taxonomists in biological recording, contributing to atlas projects, censuses etcetera deserves particular mention. One might argue that this type of activity is not a proper taxonomic activity but even if a large part of the biological recording is disregarded, non-career taxonomists still contribute very much to inventories of specific areas.
There is also a marked difference between career and non-career taxonomists with regard to the geographical area they focus on. Non-career taxonomists focus on the fauna and flora of their own country to a much higher degree than career taxonomists. Especially career taxonomists employed at the major natural history museums of Europe tend to work on organisms from other regions. The historical background is that these museums often hold large collections from, and frequently have maintained collaboration with, former colonies. Table 1 summarises publications from a museum in a well-studied country with a poor biota (Denmark) and a relatively poorly studied country with a rich biota (Spain). The great importance of papers dealing with non-European biota is shared between the two exemplar museums although the tendency unsurprisingly is much stronger in the Danish one.

Table 1. Geographical focus of taxonomic publications from two European Natural History Museums based on lists of publications in Ramos (2006) and Fink-Jensen & Hansen (2007).

	year(s)	own country	rest of Europe	rest of World
Natural History Museum of Denmark (zoology & palaeontology)	2006	7	9	37
Museo Nacional de Ciencias Naturales, Spain (Dept. of Biodiversity and Evolutionary Biology)	2004-2005	39	14	55

1.2 How is the ETW organised?

The European Taxonomic Workforce is not organized in a simple way. It may be compared with an early Medieval army which could include well-organized regiments under the command of a nobleman, as well as more loosely organised satellite groups of mercenaries, peasants etc. The latter groups would join forces with the core group as long as they were

rewarded. And for that we have found numerous ways to provide involvement, acknowledgement and fringe benefit for the non-career taxonomists.

The historical way to collaborate still influences the present taxonomic workforce and is the largest challenge for involvement of the non-career taxonomists, students, emeriti and even other career taxonomists.

Three levels of network accessibility is up for election. “The top-down organizations with little networking”, “The collaboratories with ad-hoc networking” and the “The network organized community”.

→ “The top-down organizations with little networking”. Before the 17th century all collections were closed and in private hands. Career experts that continued that pathway died out due to their isolation. They received no public goodwill, and the contacts for funding & recruiting and for insight were limited. The managers spend all their energy to take the initiatives and they regarded networking to be redundant except for a few in personal relations.

→ “The collaboratories with ad-hoc networking”. Many museums opened to the public back to the 17th century and became “Institutions without walls”. This is the working condition for many career taxonomists. The most open-minded became associated different public societies that included non-career taxonomists. However, such conditions may still be in contrast to the modern inclusiveness and common goal achievement of the Open Source Society: Star & Griesemer (1989)A pointed out the barrier “interessement”. That is “In order to create scientific authority, entrepreneurs gradually enlist participants from a range of locations, re-interpret [the participants] concerns to fit their own programmatic goals and then establish themselves as gatekeepers”. Even though some career taxonomists chooses its environment, it is not a one way obligatory point of passage; The non-career participants works with enthusiasm -only- as long as they find it interesting - and they commonly have other ways and agendas than the researchers. Getting too far from their original concerns decrease their motivation to collaborate. The history of the heterogeneity in the collaboration is exemplified in California 1907-39B: Biological research was increasingly conducted in academic institutions rather than in societies formed by amateurs. Professional biologists sought international credibility from other professional biological communities “by distinguish themselves from amateurs, establishing advanced degrees as credentials, establishing specialized journals for the dissemination of results and by increasingly eschewing the public's eclectic interests in science”. In the last 25 years, many career taxonomists have chosen this inter-institutional working method, where their projects are institutional managed: A “Collaboratorium”. The taxonomic societies, non-career- and new taxonomists have little or no attendance.

Network in the collaboratorium is unstructured due to a primary focus on institutional ad-hoc collaborations rather than more permanent open networking. The top-down organization is partly changed to decentralized collegial self-governance or even Open Source Governance. In many aspects the taxonomic community resemble the collaboratories. Open walls are partly present: Specimens are shared across museums, some publications are published under Open Access, Synthesys exchange scientists, and EDIT& PESI are already working with networking & pooling information online. These online efforts are steps forward towards dealing with distant networking and aspects of an online organized community:

→ “The network organized community”. As mentioned in the introduction, the time is ripe for Open Source Taxonomy. Furthermore, the OSS “bazaar – cathedral model" is suggested

by Tsiavos, P. (2007)D: “builds on the notion of the audience as curator (Bourriaud, 2000; 2002)D and increasingly demands its participation in the production of taxonomies (or Folksonomies)”. Mesibov (2008) suggested a OSS pathway to be the way forward, as well. Any Open Source Taxonomy will ideally be managed by a structured network online, but still grounded as all taxonomists are encouraged to attach to local societies. Wherever possible the societies is further attached to biological institutions for equipment access & training. The network organized community encourages pleasurable tasks and drive the present organization towards the OSS pathway. That will embrace a mixture of motivated career taxonomists and qualifying non-career enthusiasts to share resources online but also meet socially in person. Beside inclusiveness, transparency motivate to participate: To-do lists, common projects and milestones make clear the common goals. The cost is some interference in the planning, but useless suggestions are discarded already by the community. Many eyes may review the databases, helps to promote the results to the public and may lobby for you. In such network organization the companies and scientific institutions share resources with their volunteer community. The quality of their contributions is in competition with the contributions of the volunteer people. 40% of the descriptions are already contributed by the non-career taxonomists. Due to the huge number of unorganised, non-career taxonomists, an untapped potential of experts is liable if better facilitated. The most critical drawbacks of the present taxonomic community is insufficient open networking and the closed scientific resources; Such motivation killers decrease the activity level and recruiting of experts. If the changes implemented are too small we may fall back into the business as usual or to the “collaboratorium” structure – even we improve the networking between the career-taxonomists.

Three main aspects play a role in the current organisation of the ETW:

1. employment relationships of the career taxonomists
2. participation in projects (mainly career taxonomists)
3. taxonomic societies and other networks (both groups)

1.2.1 Organisation of career taxonomists in their institutions

There are three types of institutions that employ taxonomists namely 1) Collection institutes such as Natural history museums and Botanical gardens, 2) Universities and 3) Research Institutes e.g. Baillarguet and Centre d'Estudis Avançats de Blanes. Research by employees in such institutions is in principle governed by each institution’s research strategy. In the case if collection institutes this will largely be determined by the collections they hold which essentially reflects the expertise of the career taxonomists employed who work with these.

Table 2. Excerpts of research strategies involving taxonomy. Statements directly influencing taxonomic research in boldface.

Please extend this table listing important institutes in Europe with taxonomic research strategies, like EDIT, PESI and CETAF institutes

Centre de Biologie et de Gestion des Populations<br>Baillarguet, France	The CBGP ..... carries out research in the fields of **systematics, genetics and ecology relevant to the management of populations and communities of organisms for the purposes of agriculture, public health and biodiversity**.

Royal Botanic Garden<br>Edinburgh, UK	Five cross cutting topics have been identified which reflect the research required to meet the strategic objectives:<br>**Describing the biodiversity of species and habitats within important ecosystems and contributing towards completion of the inventory of life on earth**
Forschungsmuseum Alexander König<br>Bonn, Germany	Research fields exercised at a worldwide scale are biodiversity in terrestrial habitats, **taxonomy and systematics of terrestrial vertebrates and arthropods (including the limnetic fauna)** and evolutionary biology.

1.2.2 Taxonomic projects and their organisation

Institutional taxonomic research to an increasing degree needs funding in addition to that provided through the core funds of the institutions. Therefore, externally funded projects play an important and increasing role in the structuring of taxonomic research.

Many taxonomists have projects together with other taxonomists, funded in various ways. When it comes to the organising effect of projects, it is, however, the large-scale, international projects that are important. Large-scale taxonomic projects are of different kinds:

- compiling and structuring *existing taxonomic knowledge*, e.g.
    - Species 2000
    - European Register of Marine Species (ERMS) and World Register of Marine Species (WoRMS)
    - Fauna Europaea
    - Euro+Med PlantBase
    - Global Biodiversity Information Facility (GBIF)
    - Encyclopedia of Life (EoL)
    - Creating a Taxonomic e-Science (CATE)
    - CBD
- organising *taxonomic institutions at directors' level*, notably,
    - Toward the European Distributed Institute of Taxonomy (EDIT, see above)
    - CETAF
- generation of *new taxonomic knowledge*, e.g.,
    - Assembling the Tree of Life (ToL)
    - Planetary Biodiversity Inventories (PBI), e.g., on spiders and slime moulds
    - All-Taxa Biodiversity Inventories (ATBI), e.g., those organised by EDIT,

Among the large-scale project types, PBI merit special attention, because they are among the few large-scale projects that include generation of new *basic* taxonomic knowledge (recognition and description of species). PBI aside, this important part of taxonomic research is so far neither supported by large-scale projects (Bourgoin & Silvain 2008), organised nor funded like the PBI.

1.2.3 Taxonomic societies and other networks

Non-career taxonomists often have access to some resources of the natural history museums and other taxonomic institutions, and sometimes are partners in large-scale taxonomic projects such as Fauna Europaea. Nevertheless, it is first and foremost the taxonomic societies that organise the non-career taxonomists throughout Europe. Taxonomic societies range from local groups over national societies, to regional or international ones e.g. Societas Europaea Lepidopterolociga and Centre International de Myriapodologie. Many societies play a crucial

role as a meeting point between career and non-career taxonomists. EDIT and PESI have integrated the societies and other networks into the EditExpertNet.

1.3 Why should the ETW be better organised?

Challenges of PESI-ETW

Perhaps the greatest challenge in taxonomy is trying to discover the 10 million species estimated to live on earth before many go extinct due to habitat loss and climate change. However, the most important reason is to know more about species, their standards, and their relationships to environmental dynamics so that environmental policies can be developed and become more effective, so that habitats can be preserved and species DON'T go extinct. Only one-fifth of these species are presently named. A recent EDIT-sponsored workshop on the future of taxonomy concluded that the present efforts must be better coordinated so as to increase the rate of description of new species through more time-efficient taxonomic methods and tools. Other main reasons why the ETW should be better organised are:

1. we need a better understanding of relationships between biological diversity and environmental factors in various EU habitat types
2. to construct common European base lines to which we can measure the relationships between changes in habitats and changes in biological diversity
3. to better collaborate on the level of "ecoregions taxonomy" comprising several EU countries and neighbouring countries.

The pathway to do so, we call The Open Source Taxonomy pathway.

Table 3 A SWOT analysis of current taxonomy in Europe.

**Strengths**	**Weaknesses**
• Current taxonomic expertise<br>• Taxonomic institutions<br>• Specimen collections<br>• Public interest in discovering life on earth<br>• Taxonomic digital infrastructures (e.g. Fauna Europaea, ERMS, Euro+Med PlantBase, Species 2000, EditExpertNet)	• Little routine in and time to learn networking beside using Emails<br>• Dispersed nature of physical taxonomic communities<br>• Inhomogeneous community of highly specialised career and non-career taxonomists make organisation difficult<br>• Large number of journals publishing new species descriptions without central registration and under restricted access<br>• Slow adoption of internet for mainstream taxonomic data management
**Opportunities**	**Threats**
• Open Source Taxonomy (OST)<br>• Network facilities for communication<br>• Internet for data publication<br>• Institutional willingness to collaborate with the amateur community<br>• International initiatives in biodiversity informatics	• Reducing funding<br>• Limited leadership<br>• Failure to take decisions or act<br>• Lack common goal of taxonomic community<br>• The databases are not being updated and their reviewing editors are too few

Our SWOT analysis of taxonomy in Europe supports these conclusions (See table 3).
We consider that two approaches are essential to improve taxonomic efficiency:

1. increased coordination amongst taxonomists and their organisations
2. the use of digital technologies for data capture (e.g. images), management and publication on the World Wide Web. PESI is a coordination project building a digital infrastructure. As such we address both the technological side and the organisational mechanisms and tools to improve coordination amongst taxonomists.

1.4 How should the ETW be better organised?

The present taxonomic workforce will be developed from a very long tradition of inter-institutional organizations. However, business as usual is no longer an option[1] and time has changed since the Internet appeared. Focus of this chapter is to have a more efficient taxonomic workforce it is firstly necessary to address a number of issues. Therefore the common issues for both the career and the non-career taxonomists. Issues, that are especially relevant to the PESI Taxonomic Taskforce are discussed in the next section 1.5.

The Open Source Society community (OSS) has provided several new ideas in how to develop and organise the PESI Taxonomic workforce. It is envisaged that the global economy will continue to be knowledge based into the future and that an open source society will be leading the way[20C]. Therefore, the chosen OST model here reproduces selected procedures and methods from the OSS. The capacity of which, reaches beyond that witnessed by the Linux software development & organization.

This has ong been implemented in deviating branches, such as CERN which incorporates 1000 participants[5], have already been witnessed. Similarly Richard Jefferson has successfully pushed the Open Source software movement to biological sciences, namely agriculture, creating the Biological Innovation for Open Society at BIOS.net[6] in 2004. In 2007, Red Hat[7] followed up within the biomedical research area and finally in 2008, the zoologist Bob Mesibov[9] suggested an Open Source pathway as the alternative to the present 'More Career Taxonomists' (MCT) pathway for the Taxonomic community. To widen his point of view, we suggest that the MCT strategy is applied in addition to an OSS Open Strategy.

In order to provide here theories which are clear and concise, links are provided to allow the reader to delve further for more extensive information. From the present situation over OSS inspired procedures and networks, we'll present examples [Ex:] that are traceable to the same number of the given suggestions [Rx:].

1.4.1 The present Taxonomic community.

A. review of the literature on the history of the taxonomic organizations and consultations with taxonomic stakeholders and experts has indicated that the starting point appear to be an inter-institutional collaboration strategy called Collaboratorium (see 1.2) that use the MCT strategy. A number of issues were prioritisedby the taxonomists:

1. Budgets (for project development or continuation of biodiversity projects)
2. Accreditation (for the produced resources, including the on-line ones)
3. Relief (of the working procedures)
4. Easy access to exchange information and resources
5. Their life-work to be continued.

One solution to the budget issue maybe to initiate online donations. Currently 47% of taxonomic institutes do not accept online donations, Brovo, B. 2007[27]. In line, we

suggest (R1) that some projects can immediately be financed through online donations. This can be extended by creating an EU-fund for Biodiversity to handle the many small investments deriving from the societies, funds on deposit with the banks from EU-projects and handle inheritances. Great cost reductions are possible such as exchanging computer-

licensed software with free software. The fund-raising issue will be deepened in the Continuity deliverable D2.3. Beside funding, the other points (2.-5.) are integrated below.

A 'full-blown' Open Source Society collaboration does not *need* to follow a stepwise development from the present Collaboratorium situation of restricted resources over Open access to view all resources, Open Peer Review as the sole review process, Open Law to share all sources, Open Science for the whole public and to Open Strategy to design the projects. Some taxonomic networks have already chosen to follow a sole procedure form the OSS and not yet selected the others. Well functional procedures within the present Collaboratorium may persist: Most notable are the management of the career taxonomists, the headhunting of experts for special tasks, such as the handling of collections within museums, traditional peer-reviewing and citation processes (MLA or APA Citation[20Ø]). However, due to the reduced number of taxonomic experts, competition between settled career taxonomists in Europe is less interesting. Contrary, competition reduces collaboration and fast building on each others results. Therefore, we welcome the OST changes like suggested (Rx) below:

1.4.2 Open Source Taxonomy (OST) procedures

---transformed from theories of the Open Source Society[3,4].

B. Using the resources developed within the Open Source Society, it is currently legal to profit by offering services, e.g. Google. However, a major reason for the initial success in the community of the Open Source Society has been the 'non-monetary incentives'[12]

Like the gift economy of the OSS, the taxonomic community today is essentially providing free taxonomic resources and classifications. The institutions while they may initially get money to develop these resources through for example research funds once they are developed the institutes receive no further payments for use of the developed resources in the form of royalties. So, therefore career taxonomists would have little to loose to choose the OSS pathway. Taxonomists need to increase the public's perception and appreciation of taxonomy. Through OST there are more networking opportunities and openness creates further activity and visibility for the ambassadors of taxonomy. Transforming selected OSS procedures to the OST community will allow taxonomists at different levels to help creating and verifying resources the more available they are (of both physical and virtual resources). There is the potential in an OSS, to create a smooth transition from an amateur status to an expert status which would include a hierarchy of educational steps.

When the encouragement is not salary, OST may motivate through acknowledgement and inclusiveness at succeeding levels – Open Access (B1) to view the resources, collaborate editing and reviewing (B2), share projects (B3), collaborate development of new projects (B4) or even common decision making of designing the projects (B5):

B.1. Open Access in taxonomy[15]

(Publications incl. physical & digital collections – made accessible to the public)

As part of the Collaboratorium openness, most museum experts have already offered physical collections on loan for a longer periods, while a researcher is working with one taxon. An extended version is the Global ownership collection[28] that covers the global ownership to all collections for all researchers. It is currently available (Ex:) for biological collections of *data* within BioCASE[49] and for physical collections during certain periods at NMA[29]. The increased access to physical collections can be correlated to a "shift from expert curators to facilitators of participation"[48a] like the "Collosal Squid Project"[48b].

How to open up the collections may be a matter of deployment (R2):
A. Unica specimens or the crown jewels are presented in protected exhibition cases for local presentation, only,
B. Research collections through Global ownership by scientists, where a duplicate collection could be primary lent out to meetings or for teaching before it is open to the public.
C. Duplicate collection may be primary lent to the society meetings or teaching before it is open to the public.
D. Combining local physical collections with data of the virtual collections

Open Access of images and text is in principle straight forward and relatively easy to access online. The development of open access via 3D imagery of collections will provide online access to museum collections. Such virtual 3D-collection is useful for self-training, especially when combining with hands-on focus on physical collections. Besides, scientific descriptions of new species need full physical access to the specimens.

In order to keep paper-publications alive in the future the ever updated "Print on Demand[64]" must be highlighted (R3). Where the publishers are only paid for services in connection with the press and distribution.

The main motivation to openly publish online is increased global visibility, although online virtual publishing provides less merit. PESI could take the initiative (R4) with EDIT and CETAF to request EU politicians to take up the U.S. Public access policy[31]. However until such time as this has been implemented agreements with selected online publishers will help to encourage authors to prioritize publication resources with Open Access and Open Peer Review.

As the public research is already paid for by the public (as now in USA[31]), free and open access is the logical way to publish. Open Access initiatives are recently rewarded as a big advantage for Bioinformatics[32] and new Open Access journals are in fast development[33] like the EDITs 'European journal in e-Taxonomy'.

B.2. Open Peer Review in taxonomy[14]
(Collaborate review process – made editable to registered experts).

Open Peer Review is based on collaborate analysing and publishing. Using Open Peer Review in OST may *start* as an extra validating layer of online journals, checklists and even projects[20H]. It will relieve the traditional peer-reviewers and encourage more interested taxonomists to participate. The principle is "Given enough eyeballs, all bugs are shallow" like Raymond[10] said in his Bazaar review model - in contrast to the cathedral model[53,71] that utilize (only) traditional & closed peer review.

Risks: Lorenz (2008)[35]described the risk of spreading errors in "Open Review as a Wiki" where no gate-keepers (or traditional peer-review) were present. Aside form this there is still a risk of an experts shortage if there is not a sufficient number of experts participating in the Open Review layer, as Spies (2005)[36] from the ZooBank observed. The more expert activity the better Open Peer Review. Such Open Review will provide much needed relief to the current experts (as used in the Algaebase.org). Thus, for taxonomic purposes a high activity level of competent people is needed or the organisation will have to remain a traditional peer review backup-layer that removes the final errors.

Advantages: The best academic basis for Open Peer Review is found in the debate of Open intelligence[36,44]. The weakness of keeping information closed is described by Steel (2008)[45]

and the advantages of Open Peer Review are evident in e.g. Wikipedia: "to collectively create an intellectual product without centralized control and with almost all of those people being volunteers"[46]. Wikipedia is now inserting "experienced users" as gatekeepers. In Collumbia non-career taxonomists are included for their collaborating networks: "People interested in Colombian birds, will be able to review, in an easy and fast way, all of the information that hundreds of collectors, researchers, naturalists and amateurs have been collecting for decades. This information is a testimony to Colombia's natural heritage", Rodriguez[20A]. A similar example can be seen in a project in Costa Rica[20D]. An option to Open Peer Review (R5) may be inserted in all publishing resources (journals as well as databases), and as a backup, two responsible experts to finally confirm the review quality. For the checklists, we invite a number of experts for the Open Review layer, which also act as a waiting list of experts to be headhunted for the final (traditional) Peer Review layer. A waiting list of stand-in group-coordinators will relieve the first appointed group-coordinators, as well.

Open Peer Review is also about collaborative publishing, and the reviewers get their names on the articles (R6). Those who participate with what text is measurable using the "Stream management" method of the WikiGenes[50] "where authorship matters". This method of open review will also allow a recommendable (R7) citation where each version will have a time-stamped and can be recognized as Repository versions like time-stamped versions of the OSS applications[20z]. XML and RSS feeds can filter according to relevance using selected keywords, and referring to the latest repositories this will allow authors to follow the publication as it is being updated. Kelley, M. 2007[52] describes the Web2.0 as a facilitator of "ongoing accumulation of information through the participation of multiple users over an indefinite time-span.". More about Accreditation in section 2.2.

B.3. Open Law in taxonomy[12,20Æ]
(Share projects and secure IPR to registered experts)

Copyleft[20V] or the Creative Common Share Alike counterpart licensing is an important issue of what open source is all about. Open Law legislation intends to secure the permanent openness of any scientific resource offered under e.g., Open Access. Using Creative Common Share Alike in taxonomy entails that you can share & exchange resources and still avoid the risk of having to compete with a proprietary modified and licensed version of your own work.

Open Law avoids the fear of patent litigation, email spamming and IPL violation. Such clear arrangements allow, in practice, the possibility to further update outdated articles and share databases. The latter has great implications as shared code implies; easy future integration, shared effort to improve and advance any development, maintenance and the shared code serves as a backup (R8). Code developed within PESI could through open law be shared with all relevant stakeholders e.g. The North-African equivalent to the EditExpertNet. More about IPR in the deliverable D2.2.

B.4. Open Science in taxonomy[13]
(Collaborate project development by registered stakeholders)

The well-founded professional inductive & deductive research[20q] method is part of open science[13] where an elaborated pool of taxonomists at different levels can participate especially in the inductive reasoning work. Open science[20T] includes Citicen science[20U]. A typical way from OSS is to send an unfinished project suggestion to the community members and let the stakeholders be involved in the project management through open discussions about how to structure and implement the project you have chosen. A certain guaranty exists against immoral competitors as Star & Griesemer[6] pointed out, due to building "collections of

scientific value, which are not easily duplicated elsewhere”. An example of Open Science is given in the “Learning together” section at the IPY open science conference[20R]. Especially projects that are funded can be better implemented (R9) through common and transparent discussions at e.g., Sci-Mate.org[20H] instead of using email-collaboration.

B.5. Open Strategy in taxonomy [12]
(Common goal and a meritocratic decisions by registered stakeholders).

Open Strategy is a collaborate leadership and decision principle to create the whole framework around the initiation of a project. It may concern which project to choose, where to search funds or other ways to collaborate in project development. Non-career taxonomists started the BAP[20G] project on conservation e.g., the conservation of butterflies and plants. It is highly interdisciplinary, and the motivating and implicative aspects are being studied[20I]**.** More projects can start (R10) completing LFA templates[20P] that are common for career as well as for registered non-career taxonomists. An increased network will help achieving the nessesary funding, as well.

1.4.3 European expert recruiting and networking methods

C. Expert recruiting is a matter of training new taxonomists to become experts, discover and head-hunt potential experts, verify that they are experts (section 2.3) and use the networks to guide them into active parts of the taxonomic community (like the checklists).

C.1.European biodiversity expert recruiting system

The identification of experts not currently involved in Open Peer Review layer of the checklists or other online resources is important in order to fill any gaps that are identified and to potentially assist those experts currently working. In return the new experts identified will achieve acknowledgement and access to resources according to their level of competence (e.g., collecting permits).

C.1.1. Discover & head-hunting acknowledged experts from institutions & networks.

The EditExpertNet has acknowledged experts from the EDIT institutions and from SMEBD members as they are either educated taxonomists or accredited by the SMEBD partners such as participants in updating the checklists. The societies and other networks of EditExpertNet may be requested, too. However, PESI needs to take decisions (R11) about any other institutions or networks that may offer reliable experts and criteria of who to include. Finally, lists of or requests need to be send to invite the experts and attempt to include them within the European expert network/ community service (See section 1.4.3).

C.1.2. Discover & head-hunting experts from publications.

Potential experts of specified taxa discovered from published articles may be spotted from computers connected to comprehensive library journals. GBIF, according to Dave Remsen, is developing a RSS-feed service that automize the process. However, an evaluation of publishing experts may be verified (R12) by an internal Review layer in the EditExpertNet by the existing experts. A criteria may be that consensus or no opponent is found to invite a new expert. Such validation is also fruitful in order to create an activity in a taxon specific area. A lot of valuable non-career taxonomists will never publish a taxonomic article, but they may be a valuable source of expertise and help towards the classification of a species and updating of the checklists. These may be discovered through the Taxonomic Certification Program:

C.1.3. Talent development, spotting & clarification: The Taxonomic Certification Program.

The transfer of taxonomic knowledge and enthusiasm to the next generation is vital for biodiversity in general, but it is also highly motivating for current experts as it one way to insure that their life work will be continued and methids and knowledge will be used by these new taxonomists.

The development of a self-training facility supported by taxonomists via online networks based on animated images and real physical collections would be the initial step. Action will start on the collection of images for animations and online keys by the Swedish NRM.se, Georgian iliauni.edu.ge, Danish SNM.dk & didactics Ind.ku.dk as soon as such a facility is funded.

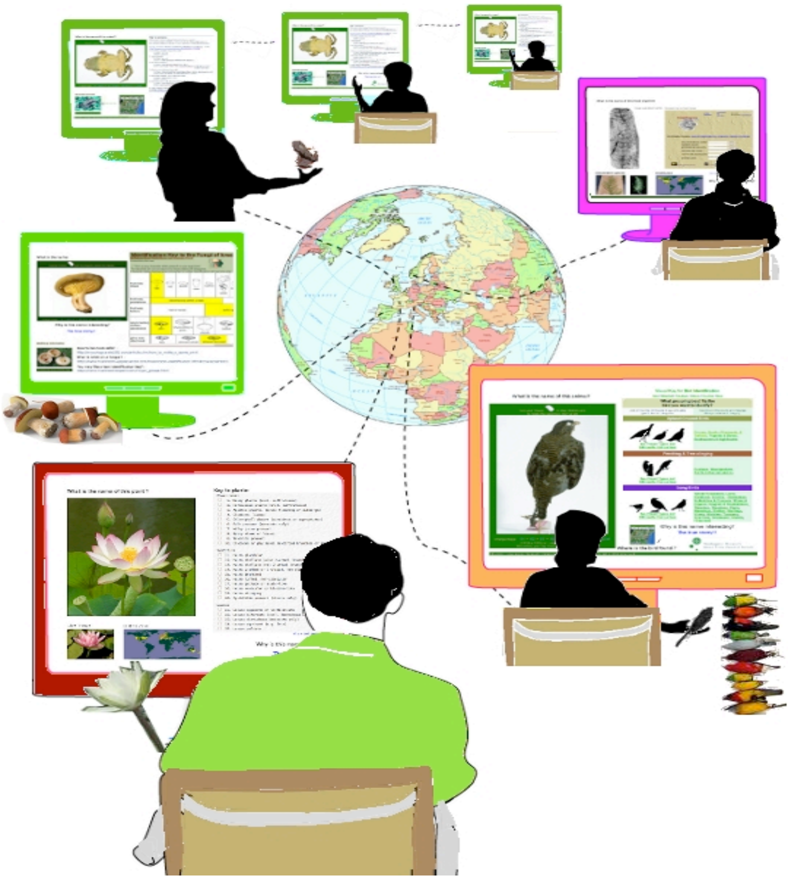

Figure.1. All levels of the taxonomic certification program will have access to the identification program and once it is widespread help can be provided online.

Such taxonomic certification program[48] will serve two main purposes: 1. Promote & train taxonomy for fun in the "Taxonomic Driving Licence", where the talent gradually will develop and eventually specialize and become a serious expert and 2. Competences clarification as these unknown non-career experts or students will achieve an accredited "Expert certification"(see 3.3).

Promote & train taxonomy for fun is well-known in botanic workshops that encourage the participants to identify the collections. The Boise State University has monthly events[50a], as well as promotions where there are active 'demonstrations'[51a]. Mycologists[52a],

ornithologists[53], entomologists[54a] or the marine 'Fun activities' of MarBEF invite newcomers to come and learn about the different realms online. Many identification keys and the rotation techniques are already freely available online.

With very few exceptions, like the Perkins-Tryon Public School, taxonomic syllabus are no longer available in schools and universities. The taxonomic certification scheme, however, can offer integrated modules utilizing the Exemplary principle[17] to supply taxonomic certifications in various levels. Such modules may provide scientific literacy in the aspect of immersion, IT-technique, networking, and systematic learning. For pre-recruiting and talent spotting, we suggest two “Taxonomic driving-licenses” at general taxonomic levels such as order or family/genus depending on the taxonomic group. E.g., in fungus it would be the Amanita genus to exclude genera that hold dangerous species to eat. However, we suggest a smooth transition from novice to expert. From the level of secondary school with biology as specialisation, the first certification level can be motivating to step further and such taxonomic involvement may in time improve the recruitment of career taxonomists. The self-training aspect of the Expert certification can be useful even for experts with narrow expertises, as it will ease their effort, if they consider including a second and deviating taxonomic group.

Outside institutions, the preparation for certifications of non-career experts will strengthen their scientific systematic thinking, too. This may be supplemented with local initiatives like the societies to eventually increase awareness of the species directories to input data.

Using the same technique for competence clarification (see 3.3), we suggest (R13) the Taxonomic Driving-license for training, to make taxonomy popular and for talent spotting. As a pay-back for the effort the institutions may help with sharing resources: collecting permits, access to protected collections with these new experts.

C.2 European expert network/community service - to insure that all taxonomists are part of active and competent networks.

Linné started a taxonomic wave which currently needs a minor motivation boost to increase the activity of the taxonomic community and to rebuild the past popularity of taxonomy. It may help if taxonomists are working towards a common goal such as 'Biodiversity conservation' (R14). The US president Obama showed the way of creating a movement using the power of OSS and as his network organizer, Mr. Hughes, commented: “You can have the best technology in the world, but if you don't have a community who wants to use it and who is excited about it, then it has no purpose”. Hughes points out that the online network has to “be in sync” with it's purpose and “keep it real, keep it local”. These experiences highlight two points: Reaching a high activity is crucial to motivate and the facilities need to be local based to be relevant and applied. “Reaching Critical Mass”[20W] of users is needed to achieve stable and high activity. Firstly, we did build the robust expert infrastructure that encourages options for recruiting and networking (www.EditExpertNet.org). This need to be strongly promoted internally among the experts and external through stakeholders. Secondly, many motivation procedures and methods must be utilized, as they are the key to the needed (critical mass) activity (R15).

PESI has set up the present OST model for communication and management of the expert network. That is a practical OST working method from the OSS. These methods are important to motivate all taxonomists to deliver more quality work into the taxonomic community. For practical usage, the information-service, EditExpertNet, is developed on taxonomic experts, their expertise, their networks and ongoing projects by EDIT (WP2) in close collaboration

with PESI (WP2). The most important role of the EditExpertNet is to help experts to be channelled to more permanent members within the taxonomic networks especially to the European checklists.

C.2.1.Which networks to involve in the OST?

To meet the results of the workshop in Spain[56], the career and non-career taxonomists need to work together in virtual and in personal networks to enhance the inclusiveness.

Career taxonomists will also benefit if they are invited to online network activity and they need to boost the *local* societies, which have become formalized members of the European expert network/community service.

The OST network is intended to supplement the current institutional communities established. EDIT partners should provide up till 20% of the European experts and as many as possible of the rest by PESI. That is the focal point networks, SMEBD, and the self-made networks created by the experts, themselves (see below). The workforce will benefit from establishing new taxonomic networks that interact with each other in a national and in an even more local way (Keep it relevant, and keep it local, as previously mentioned). Collaborating networking presupposed inclusiveness when an approximately 200 taxonomists from 10 countries worked with botanical biodiversity in the southern Africa[20A]. Taxonomists handling Bacteria classification have recently established such online collaborating network[20E]. Emails could be sent to all possible networks to request experts to register their expertises (R16), followed by a request to create new networks and to update the OST each year. The request would highlight the options the experts would enjoy as a quid pro quo for their involvement.

In the present Collaboratorium situation, the career-taxonomists have some institutional networks based on personal email lists. The upside is that it is very easy to operate. However, as it ends in the personal e-mail boxes, the account will soon be overloaded and the experts spend all time to keep-up answering many the same questions over and over again. It leaves little or no time to improving their capability to learn the efficient networking facilities. Besides, ordinary email lists provide little attachment or inclusiveness for the experts or potential funding stakeholders to the taxonomic checklists. Taxacom[20F] is a well-known Open Source mailinglist – an involvement success, although it lacks organisation in the taxonomic discussions. The EditExpertNet offers the same options but for registered users only, and offers filtering due to your personal choice like your selected countries, selected taxa, methods used or even your selected societies. This personalized choice with dynamic *ad-hoc* networking may be the answer to efficiently filter people and information. Figure 8 shows examples of the technical possible ad-hoc networking, permanent networks and their possible interactions. Ad-Hoc-networking expires after 8 weeks. Figure 2-4 shows examples of how to create such Ad-Hoc networking.

Many of the old local societies have recently gone online and may be nicely presented in EDITs ScratchPads. The checklists networks are under integration with the rest of the taxonomic workforce and intend to be further developed under the continuity, described in the deliverable D2.3.

Due to undecided privacy restrictions in PESI and EDIT, the EditExpertNet can offer limited networking. EDIT will decide their role on the 4th of October 2010. Untill then, bulk invitations, except for updating, is no longer allowed. However, the technique in this base provides networking between networks, between experts and networks, and of cause between the experts (R17). The experts of e.g., PESIs Focal Points and the NHM partner of EDIT can

get special limitations and be addressed as a network, only. Open Source networks may accept that they can have direct access to their members. Decisions of the status of each single network need to be taken.

The checklists of PESI will benefit of the most possible open structure to recruit experts to update the databases and group coordinators: When the restrictions are clarified (R18), PESI can request the experts to go from traditional Peer Review and include a waiting lists, over an Open Peer Review layer safeguarded by a traditional Peer Review layer and ending with only an Open Peer Review layer performed by all expert members (including the waiting lists) – a clear relief for the peer-reviewers and database administrators. The point is, that it works dynamically going towards traditional Peer Review when few experts works in a taxon and towards Open Review when numerous experts work within a taxon. The group-coordinators can then focus on the more rare groups. Headhunting experts from e.g., the societies to supplement with experts and coordinators will strengthen the review process of the checklists.

In the EditExpertNet, you can join or create "Direct communication" in self-made ad-hoc networks, "Networking communication" in more permanent contact creating fora, "Specific network communication" in selfmade networks, connection to "Established networks – not reviewed by PESI*" that may include mixed network of networks and single experts. An example flow may be: A female expert enters the PESI portal. If her interest is focused on information of a species, she may go directly to the checklists, but if her interest concerns taxonomic experts she will go to the EditExpertNet. She can choose just to view "Simple search" information or the menu points of "Advanced search" experts from their expertises, institutions, country etc. From the advanced search results she can choose to create her self-made network. Her selection and network criteria can be according to any search criteria.

Figure. 2: Advanced search of of a taxon, and the option to export a file of experts (in .csv format) for e.g., a checklist leader. A normal expert may only search a taxon and create an Ad-hoc network (and invite them for a specific subject).

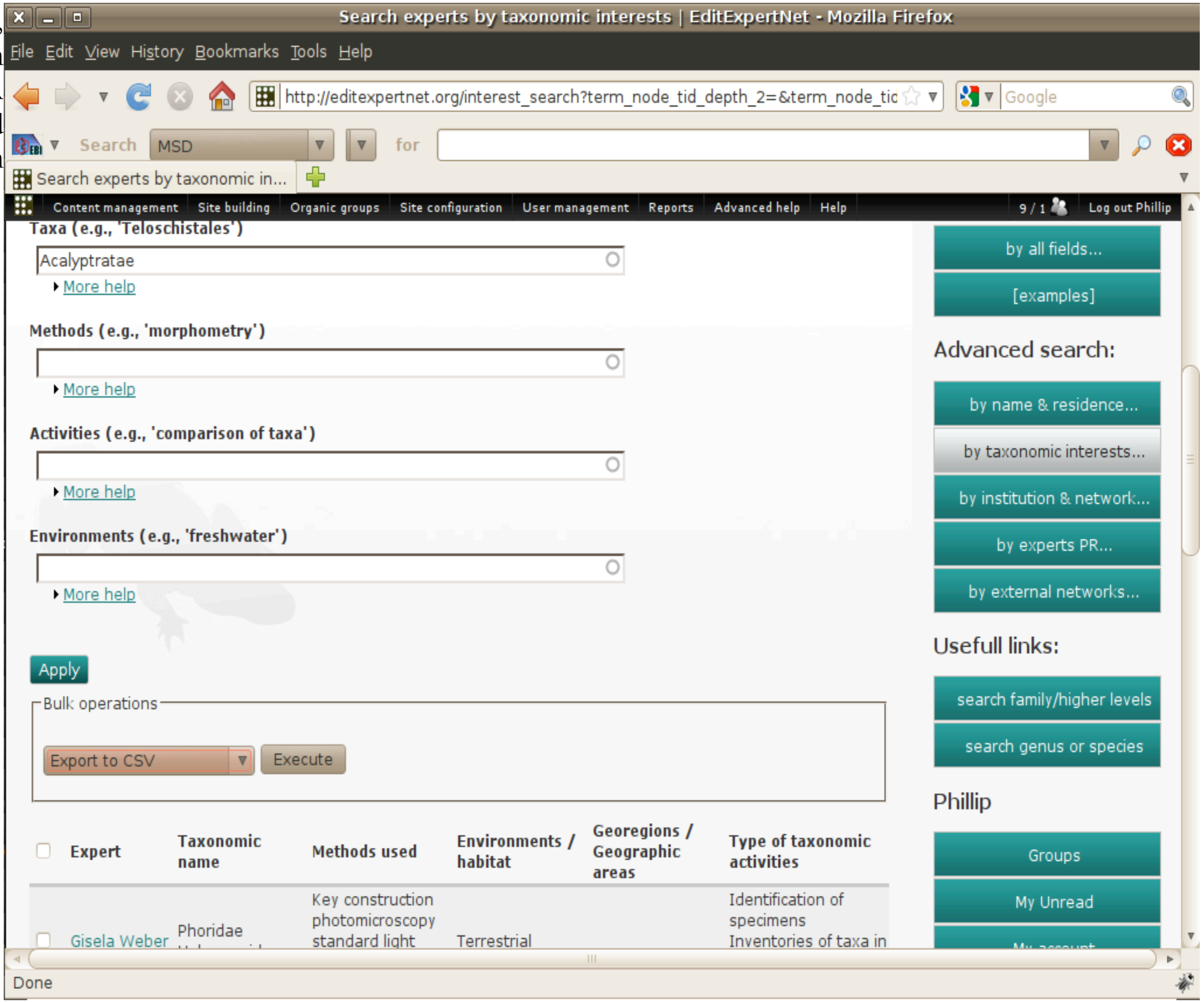

Figure 3: Illustration of a Gap-analyses created for one country. Group of countries can be created, like the Nordic country shown in the next figure.

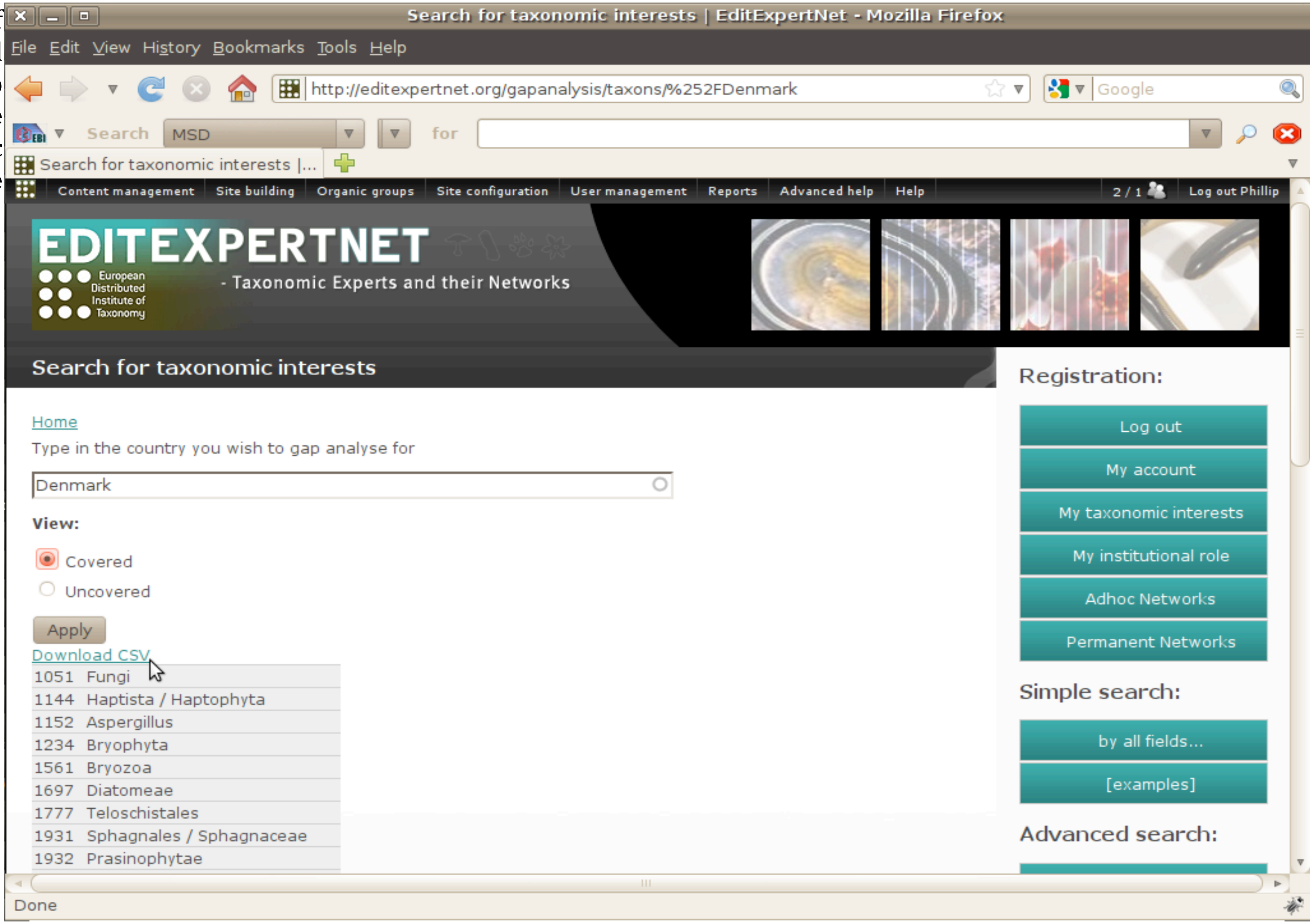

Figure 4: A list of groups established.

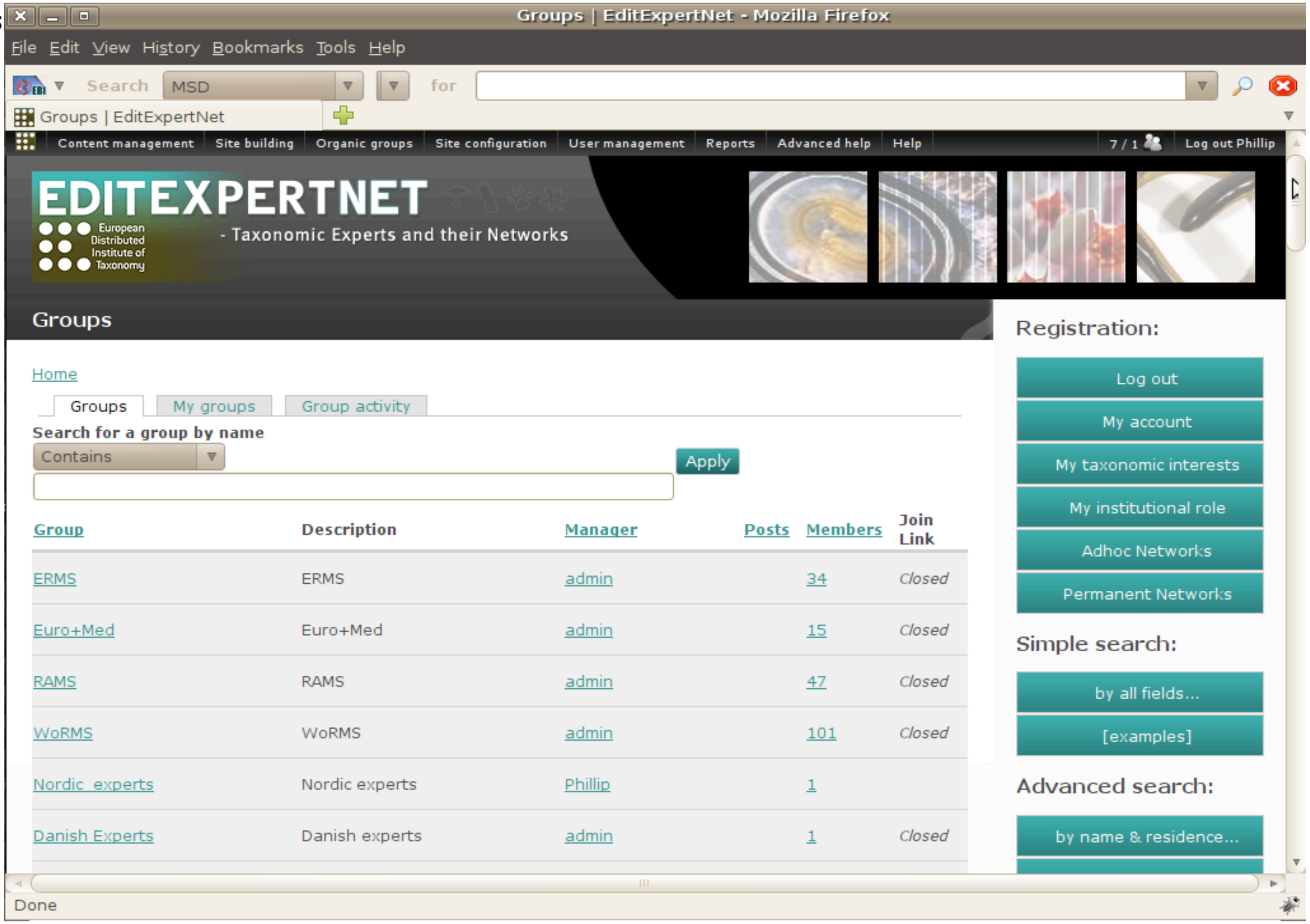

A special interest may be creating the "Specific network communication" from her special taxon of expertise. These will correspond to the same taxon that are handled in the checklists. For example if you wish to attract experts in the Netherlands to join a network on Coleoptera: All she will need to do is to search Coleoptera AND Netherlands, write a message to explain what is her immediate request, click 'create network' and the national experts will be invited to join this network, e.g., to work on red listing of Coleoptera species. Such created "specific network" may serve as recruiting to the waiting list/Open Peer Review layer of a relevant checklist (like Fauna Europaea). If she had chosen 'Networking communication' – like the female network - or the 'Established networks', the connection to the checklists are indirect, but being active networking to this recruiting facility (EditExpertNet), she'll probably be encouraged to participate in other narrow networks to finally find her way to the checklist open review layer. If the expert is also registered as a checklist or focal point administrator or an institutional editor, she can make a gap-analysis and download lists of selected networks and expert information.

PESI may also organize new presumable useful 'permanent' social network fora, like female network, meeting coordinators network, but also specific networks like methodology networks, courators network, taxa specific networks and a tree-improvement network (Mesibov, 2008[57]). An expert may be visible online, and if she sends out a question the recipient can see the request, and may reject the question by not responding or simply reply instantly. Few and highly appealing networks are needed as all new networks and the renovated society networks have to accelerate high activity of communication. A way is that a 'cheerleader' invite a lot of relevant participants and let them ask relevant and even provoking questions to boost activity. When interesting activity is ongoing the 'direct marketing' between experts will drive more to register and hopefully the activity will finally increase the recruiting of experts to update the checklists.

* Linking to the checklists are under preparation and direct access to other EDIT networks are created if you login to EditExpertNet using the CSSO that provide a secure and facilitated way for registered experts to login only once, but be able to surf all facilities (R19). The CSSO is suggested in all expert facilities of taxonomy to ease the login .

The next step is the long time recruiting of taxonomists from the training resources network where small and locally based physical societies connect to the internet – streamlining the habits for online participation. (See section C.3)

C.2.2. How to bring authority to the network community?

Due to the declining number of career taxonomists, more responsibility and tasks *need to* be carried out by the non-career taxonomists. The OSS has proved that it is possible to split a professional job into minor manageable tasks using "Ground mapping"[65a]. These tasks can be scientifically performed by 'an army of largely unpaid' enthusiasts (Mesibov, 2008[9].) This is in line with the ideas behind "Citizen Science"[53], too, which "involves the enlistment of large numbers of relatively untrained individuals in the collection of scientific data" like in the Audubon Society's Christmas Bird Count. The career taxonomists can regard it as a possibility to 'outsource' some identification and, for them, routine tasks. Attentiveness, however, is needed that it will backfire if too many boring tasks are given to voluntary people. On the other hand, the non-career taxonomists can regard this as an opportunity to achieve inclusiveness and acknowledgement, when they produce high quality work. Create good quality science together is highly motivating [i.e. Scientific immersion].

Any sharing tasks may create conflicts. To prevent common disagreements, an organization theory called the Computer-Supported Collaboration (CSC), is developed to deal with both content processing and with context processing. That is to address how to minimize human relation problems that may occur when no body language is available in distant collaboration. The CSC include “bid and ask” relationships and provide a hard-core agreement tools, as we'll suggest (R20) usage of contracts, system integration, collaborative content management, agree on delegated assignments and produce co-authored output, inspired from Wong, I. 2007[20]. For network users, a warranty contract is obvious. CSC workshop at University of Maryland (2008) addressed “Social technology for biodiversity”[70] Ambitious projects like Encyclopedia of Life and Tree of Life were emphasized as examples of successful computer supported collaboration.

Working in networks, the career and non-career taxonomists need to co-exist and collaborate with respect to their skills. In stead of using the University position as the original setting of the hierarchy, the OSS way is that the most competent is taking in charge the top-hierarchy of the Internet network according to the skills present. The hierarchy is run by the most competent organizer or researcher in their respective areas. Some scientists may regret that they may not be on top status of the managers or information officers network, as they are less skilled in this area. The point is to empower the network community using meritocracy[16]. In the Open Source Software, the hierarchy of the career- and non-career taxonomists establishes fast in most networks. That is, even it is regarded bad habit to highlight ones formal educational level. Naturally, few of the non-career taxonomists will outmatch the career taxonomist in science due to the educational head start, but anybody who does not use your skills will eventually loose status.

To help the hierarchy to be established fast and acceptable for most network users, we'll suggest (R21) a OSS rewarding method of the meritocracy called “Stratification mapping” (Goodman, S. 2006[23]), that include all assets that brings authority to a network participant: 1) Human Assets: Accurate information of specialized knowledge. 2) Honorific Assets: Network members may be given a score or rank on a website that is displayed by their name as an honour or award. 3) Reputation system: rated up or down by other user. 4) Social Assets: a person can be considered central in a network by accumulating a large quantity and specific quality of social ties. 5) Political Assets: Many forum governments enforce policies that give differential control over the website content. Finally, the moderator and administrator usually have unchecked power to delete and change all messages, while readers are given limited authorization. Poorly rated messages may even be hidden from view by the moderators of Slashdoc.org. Finally, to avoid loosing your status you need to follow the networks 'netiquettes'[62] .About providing merit, citations, and grants please go to the PESI-TT.

C.2.3. Ways to make the taxonomic networks more active?

As mentioned, high activity is crucial for recruiting experts and for the future of taxonomy. Promotion is valuable both internal and external. We'll suggest (R22) to call for debaters to start networks, illustrate interesting networking in practice, e.g., show off the value of registered expertises, and the close connection from debate on taxa to keep upgrading the checklists. Externally, we need further integration between the PESI-facilities, cross-linking to large sites, press coverage of our features in TaxaCom, in publications and in highlight out facilities to our collaboration partners.

C.2.4. Who should maintain the new taxonomic networks?

In principle no-one appointed – that is, no extra staff is needed. The ad-hoc networks should open and close automatically according to the usage. 8 weeks without activity prompts the network to close, while the 'permanent' networks or meeting rooms will close according to 1 year inactivity when the technical database-administrator updates the software code, anyway.

In this document we have utilized the definitions given at: http://www.sci-mate.org/item.php?id=21

References for the PESI ETW

1. Fabian Haas and Christoph L. Häuser How many taxonomists are there? www.gti-kontaktstelle.de/taxonomy_E.html *or* Fabian Haas and Christoph L. Häuser, 2007. Taxonomists: an endangered species?: http://www.gti-kontaktstelle.de/downloads/Poster_2_Klein.pdf
3. Boegh, P.S. 2009. Sci-Mate Open Peer Review publishing. http://www.sci-mate.org/item.php?id=21
4. Matteo Turchetto and Andreas Viklund, 2008. The Open Source Society: What Community Software Development Can Teach Business, Government, and Media. Unpublished. http://dailyrevolution.net/?p=1000
5. Matteo Turchetto and Andreas Viklund, 2008. The Open Source Society: What Community Software Development Can Teach Business, Government, and Media. Unpublished. http://dailyrevolution.net/?p=1000
6. Star,L.S. & Griesemer, J.R. (1989). Institutional Ecology, `Translations' and Boundary Objects: Amateurs and Professionals in Berkeley's Museum of Vertebrate Zoology, 1907-39 http://sss.sagepub.com/cgi/content/abstract/19/3/387. Social Studies of Science, Vol. 19, No. 3, 387-420 (1989). DOI: 10.1177/030631289019003001.
7. Thomsen, 2000. Virtual Museums: Inter-Museum Collaboration. http://www.ischool.utexas.edu/~vmuseum/thomsen.html
8. Nathan, Bos et al. 2007. From Shared Databases to Communities of Practice: A Taxonomy of Collaboratories. http://www3.interscience.wiley.com/journal/117979323/abstract?CRETRY=1&SRETRY=0
9.Mesibov, Bob. 2008A, TAXONOMY SHOULD BE DISTRIBUTED: www.scientific.dk/pesi/ProgressivTAXtext.pdf

10. Wikipedia 2009,http://en.wikipedia.org/wiki/Open_access
12. Wide Open . Open source methods and their future potential Geoff Mulgan Tom Steinberg with Omar Salem , 2005. p41-43(Open Peer Review) + p66(OpenStrategy) ISBN 1841801429. http://www.bioteams.com/WideOpen%5B1%5D.pdf
13. Wikipedia 2009,http://en.wikipedia.org/wiki/Open_science
14. Wikipedia 2009,http://en.wikipedia.org/wiki/Open_peer_review
15. Wikipedia 2009,http://en.wikipedia.org/wiki/Open_access
16. Wikipedia 2009,http://en.wikipedia.org/wiki/Meritocracy
20. Amateurs as Experts: methods http://www.lancs.ac.uk/fss/projects/ieppp/amateurs/methods.htm
20A. Siebert & Smith (2005). Lessons learnt from the SABONET Project whilst building capacity to document the botanical diversity of southern Africa. http://www.bgci.org/africa/sabonet
20B. BioBite (2003) – Collumbia. Editorial Importance of the shared effort in conservation initiatives. http://www.biomap.net/English/biobyte4english.htm
20D. Mata & Gámez, InBio, 2004. Biodiversity Information and Policy-making in the light of Costa Rica's Experience. http://ipts.jrc.ec.europa.eu/home/report/english/articles/vol84/ENV1E846.htm

20E. Physorg, 2009. Online collaboration identifies bacteria.
http://www.physorg.com/news154265533.html
20C. *Rolfes,R.,Oliveri,A., McNulty,C.2010.*Visions for the Future of Associations. Associations now. http://www.asaecenter.org/PublicationsResources/ANowDetail.cfm?ItemNumber=46915
20F. Taxacom -- Biological Systematics Discussion List.http://mailman.nhm.ku.edu/mailman/listinfo/taxacom
20G. 20. Amateurs as Experts: methods
http://www.lancs.ac.uk/fss/projects/ieppp/amateurs/methods.htm
20I. Amateurs as Experts: Harnessing New Networks for Biodiversity.
http://csec.lancs.ac.uk/docs/Amateurs%20as%20Experts%20Final%20Report.pdf
20H. Sci-mate open access publishing. http://www.sci-mate.org/wiki/index.php/About_Sci-Mate
20P. Bayón, A.; Santos, O.C.; Boticario, J.G., 2008.A component to carry out the Logical Framework Approach in dotLRN. 7th OPENACS / .LRN Conference, Valencia (Spain) (2008).http://aulavirtual.uv.es/ficheros/view/openacs/Paper_LFA.pdf
20T. Wikipedia 2009,http://en.wikipedia.org/wiki/Open_science
20U. The New York Times. The Wild Side. Olivia Judson, 2009. Guest Column: A New Kind of Big Science: http://judson.blogs.nytimes.com/2009/01/13/guest-column-a-new-kind-of-big-science/?th&emc=th
20V. Copyleft, wikipedia. http://en.wikipedia.org/wiki/Copyleft
20W. Annual Conference; University of Massachusetts – Amherst; Year 2010; Conference Proceedings of JITP 2010: The Politics of Open Source. The Journal of Information Technology and Politics. http://scholarworks.umass.edu/cgi/viewcontent.cgi?article=1000&context=jitpc2010
20Æ.Open Law Project, 2009. Promoting open access to the law. http://openlawproject.org/
20z. Time-stamped repository example of a OSS Linux drawing-application.
ftp://ftp.gimp.org/pub/gimp
20Ø. Ashlock, J. M. , 2007-08-11 "Open Source Society: Can Production Outside of the Market Survive?" Paper presented at the annual meeting of the American Sociological Association, TBA, New York, New York City Online. 2010-06-04 from
http://www.allacademic.com/meta/p184744_index.html
http://www.allacademic.com/meta/p_mla_apa_research_citation/1/8/4/7/4/p184744_index.html
20q.Deduction & Induction.2006, Trochim,W.M.K.
http://www.socialresearchmethods.net/kb/dedind.php
20y. Tezcan,S., Tezcan,F.,Gülperçin, N. 2010. İzmir'den 4000 böcek türü = 4000 insect species from Izmir, Turkey.253 p.ISBN/ISSN9786058912580.
http://libris.bahcesehir.edu.tr/fmi/xsl/yordam/y1_detay.xsl?-db=YordamBT&Ba%C4%9F%C4%B1%C5%9F%23=B172&-token.aranankelime=B172&-token.tk=&-token.gt=0&-token.d=1&-skip=1&-find&-token.yzyzm=yzm
20R. T6-1. Learning together: The impacts of integrating education, outreach and research in IPY
http://ipy-osc.no/session/t6-1
20S The Open Archives Initiative Protocol for Metadata Harvesting. 2008.
http://www.openarchives.org/OAI/openarchivesprotocol.htm
21a. National Association of Mold Professionals. http://www.moldpro.org
21b. Field Identification Skills Certificate. http://www.bsbi.org.uk/field_skills.html
23. Goodman, Sasha. "Stratification Mapping: The Distribution of Status in an Online Organization" Paper presented at the annual meeting of the American Sociological Association, Montreal Convention Center, Montreal, Quebec, Canada, Aug 11, 2006
http://www.allacademic.com//meta/p_mla_apa_research_citation/1/0/4/5/6/pages104560/p104560-1.php
27. The Struggles and Strategies of Online Organizing, Britt Brovo. 2007.
http://www.techsoup.org/learningcenter/techplan/archives/page9820.cfm
28. California Native Plant Society.

www.cnps.org/cnps/chapters/pdf/chapter_annual_reports2007.pdf
29. National Museum of Australia. The audience is cast as sailors on Captain .... landing, at Botany Bay.
www.nma.gov.au/libraries/attachments/review/review_report_20030715/files/552/ReviewReport20030715.pdf
31. First U.S. Public access policy made permanent. Consolidated Appropriations Act ensures NIH public access policy will persist. 2009. http://www.taxpayeraccess.org/media/Release09-0312.html
32. First U.S. Public access policy made permanent. Consolidated Appropriations Act ensures NIH public access policy will persist. March 12, 2009.http://www.taxpayeraccess.org/media/Release09-0312.html
33. The Bourne Commendation: Open Access Evangelist Wins 2009 Benjamin Franklin Award:http://www.bio-itworld.com/news/04/06/09/philip-bourne-benjamin-franklin-award-2009.html
35. Lorenz, 2008. Open Peer Review as a wiki.
http://mailman.nhm.ku.edu/pipermail/taxacom/2008-April/026902.html
36. Spies, 2005. Zoobank-list. Quality control, "the real challenge".
http://list.afriherp.org/pipermail/zoobank-list/2005-October/000067.html
44. BLOg Source INTelligence - just about open intelligence. A conversation about collective intelligence.http://blosint.wordpress.com/2008/08/14/a-conversation-about-collective-intelligence
45. BLOg Source INTelligence - just about open intelligence. The New Craft of Intelligence.
http://blosint.wordpress.com/2008/08
46. BLOg Source INTelligence - just about open intelligence. A conversation about collective intelligence.http://blosint.wordpress.com/2008/08/14/a-conversation-about-collective-intelligence
47. IDENTIFICATION LEVELS, Graham,A. 2009. CSIRO Australia:
www.scientific.dk/pesi/id_levels_experts.pdf
48. Boegh,P.S.Taxonomy needs scientific driving licenses and certifications.
http://www.wikigenes.org/e/art/e/32.html

48a. The future of museums: the shift from expert curators to facilitators of participation. Ross Dawson, 2009. http://rossdawsonblog.com/weblog/archives/2009/03/the_future_of_m_2.html
48b. Collosal Squid Project of the Museum of New Zealand Te Papa Tongarewa.
http://squid.tepapa.govt.nz
49. Museum 2.0 Hackerspaces: DIY Science Centers for Adults (2009).
http://museumtwo.blogspot.com.
50. The Department of Biological Sciences of Boise State University hosted the first Idaho Botanical Foray in August 2008. www.idahonativeplants.org/archives/cat_announcements.aspx
50a. The Department of Biological Sciences of Boise State University hosted the first Idaho Botanical Foray in August 2008. www.idahonativeplants.org/archives/cat_announcements.aspx
51a. Action Demo Classic.
http://www.ces.purdue.edu/ces/vanderburgh/4h/school/schoolenrichment.htm#_Action_Demo_Classic
52a. Minnesota Mycological Society.
http://www.minnesotamushrooms.org/index.php?option=com_content&view=category&layout=blog&id=1&Itemid=3&91234362fce806469b7d05506d0fa5a3=60bb7ad52a2e2cbc6c8c1129bd15c7ef
52. Minnesota Mycological Society.
http://www.minnesotamushrooms.org/index.php?option=com_content&view=category&layout=blog&id=1&Itemid=3&91234362fce806469b7d05506d0fa5a3=60bb7ad52a2e2cbc6c8c1129bd15c7ef

53. Scoping study on Integrating Countryside Bird Survey and Breeding Bird Survey Data to Generate All-Ireland Trends.
http://www.bto.org/research/reports/researchrpt_abstracts/2005/rpt_376.htm
54a. BENHS Indoor Workshops 2008 - 2009. http://www.benhs.org.uk/works.html
50. A wiki for the life sciences where authorship matters. Hoffmann, R.,Axton, M. 2008
http://www.wikigenes.org/e/art/e/A_wiki_for_the_life_sciences_where_authorship_matters.html
53. Wikipedia 2009,http://en.wikipedia.org/wiki/The_Cathedral_and_the_Bazaar
56. Workshop report Professionals and non-professionals as producers and users of taxonomic knowledge – synergies, conflicts and the role of taxonomic societies . An EDIT activity of WP2 and WP4 : http://www.e-taxonomy.eu/files/Workshop_ALMERIA_report_080324.pdf
57. Mesibov, Bob. 2008B, Taxacom. Systematists as holists:
http://markmail.org/message/wivlvhspec6ey4qw
62. Wikipedia 2009,http://en.wikipedia.org/wiki/Netiquette
64. Wikipedia 2009, http://en.wikipedia.org/wiki/Print_on_demand
65a. Ecological Census Techniques: a handbook, Second Edition.
http://ecologia.ib.usp.br/lepac/milton/Livros/Sutherland%202006%20Ecological%20census%20techniques.pdf
66. Societas Europaea Lepidopterologica http://www.soceurlep.org
70. Cynthia, S.P. 2008. Social technology for biodiversity.
http://mailman.nhm.ku.edu/pipermail/taxacom/2008-April/027046.html
71. Tsiavos, P. (2007) The Museum as a Cathedral and a Bazaar: Tracing flows of rights in the UK cultural, memory and education sector.
http://icommons.pentabarf.org/programme/iSummit08/attachments/8_ISUMMIT%202008_05_04_08_CURATION.pdf

1.5 What is the PESI taxonomic taskforce (PESI-TT)

The European Taxonomic Workforce described above is a pool from which subsets, taskforces, can emerge/be organized to tackle a concrete task into the PESI taxonomic taskforce. Taxonomic taskforces may consist of just a few people, or they may be very large, like the PESI taxonomic taskforce. In the Description of work for PESI (PESI 2008) one of the coordinating activities of the project is "the formation of European taxonomic taskforces to upgrade the pan-European checklists". The core of the PESI-TT comprises the expert networks which were created for the pan-European checklist projects: ERMS, Fauna Europaea, Euro+Med PlantBase.

1.6 What are the tasks of the PESI-TT?

It is PESI's ambition to expand the PESI-TT to also include specialists from the mycological, phycological and other communities and, in the long run, to include also experts on the pan-Palaearctic biota. See Annex 2 for further background information.

The most urgent task of the PESI-TT is to secure that the existing pan-European checklists ERMS, Fauna Europaea, Euro+Med PlantBase are updated and that information on groups of organisms not covered by these projects is made similarly available.

The information that needs to be updated and made available includes:

- taxonomic and nomenclatural information: newly described taxa, new synonyms, new concepts of species, genera, changes in names, status, or rank, etc.
- faunistic and floristic information: new area records, removal/correction of erroneous records, etc.
- bibliographic information: new references for names, taxa, and area records
- all kind of corrections of errors in spelling of names, in authorship of names, in references etc.

The PESI-TT therefore includes taxonomists specialised in a particular group at the global or regional level, as well as local experts in the flora and fauna of particular countries and regions. As the scope of PESI widens to include other groups of organisms (fungi, algae etc.) and geographically, the entire western Palaearctic subregion ("Flora-Fauna Palaearctica"), the PESI-TT will grow accordingly. The first step in PESI is the inclusion of specialists covering European fungi, algae etc., and specialists with a geographical focus on Turkey, Ukraine, Georgia, and Russia.

1.7 How is the PESI-TT organised?

The existing PESI-TT consists of contributors to Fauna Europaea, Euro+Med PlantBase and European Register of Marine species (ERMS). These circa 700 individuals are organised in the Society for the Management of Electronic Biodiversity Data (SMEBD), which owns and governs the copyright of their contributions. Membership is for lifetime; even if members no longer contribute they continue to have a voice in how the databases are managed. The SMEBD Council is elected by the members and appoints committees to manage its databases. This management includes appointing host institutes and taxonomic editors and distribution of the database.

1.7.1 Fauna Europaea

No less than 476 experts contributed taxonomic and/or faunistic information for Fauna Europaea. The vast majority of the experts are from Europe but a few are from other countries like Australia, Canada, South Africa, and the USA. In most cases this was because there are no European experts available on certain animal groups, even if these animals occur in Europe. Fifty-nine of the experts were group coordinators, in addition to providing their own data. Group coordinators were responsible for data on larger or smaller groups of animals and coordinated the work of the taxonomic experts within that group. This is illustrated in Figure 5.

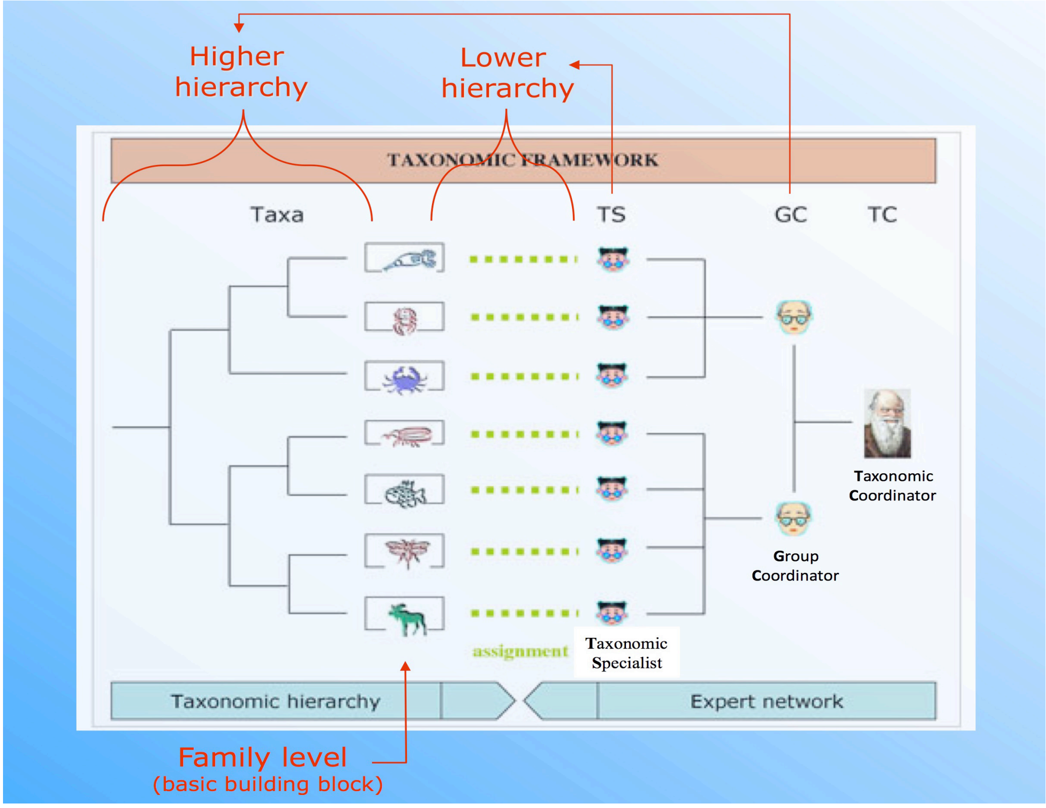

Figure 5. Diagram of the work flow in Fauna Europaea project.

- Community Committee (Committee with 1-7 participants - working with possible complains. Overall coordination of the network)
- Executive Manager (running the secretariat - working with call for group-coordinators of the checklist and possible hiring staff – overlooking the gap-analysis)
- Data Manager & web master (high level content management and web master, should have access to the Expert DB to change records)
- Technical Manager (technical management and system support, should have access to the Expert DB to change records)
- Group coordinator (**GC**) version X (supervising the editing on a certain group)
- Taxonomic Editor (**TC**) version X (equals Taxonomic specialist in FaEu)
- Associate Specialist (**TS**) version X (additional specialists being permanent associated, local or society experts)
- Reviewer (Ad-hoc specialists being invited from the registered participants)
- Advisor specialist (Ad-hoc specialists open for registered and non-registered members - a future OSS role)

Several methods have been used within Fauna Europaea to increase to quality level of the data. These include: checks on technical and logical correctness (so-called Business rules) within the data import tools, comparisons between checklists, calculations and visual checks and regional and thematic validation workshops using a European network of National Focal Points.

Figure 6. Data validation process in the Fauna Europaea project.

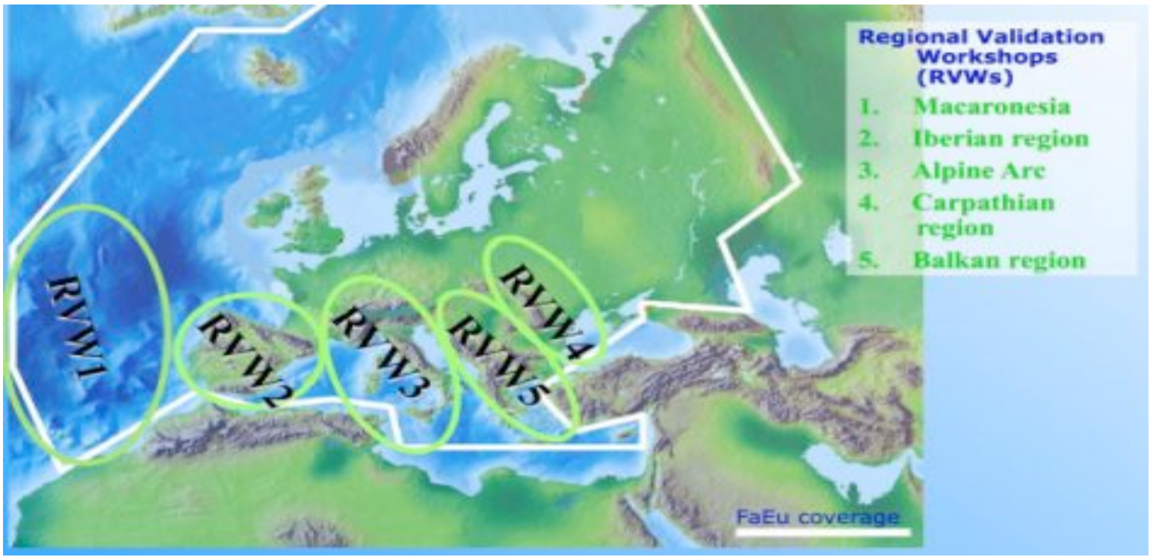

Figure 7. Regional validation workshops in the Fauna Europaea project.

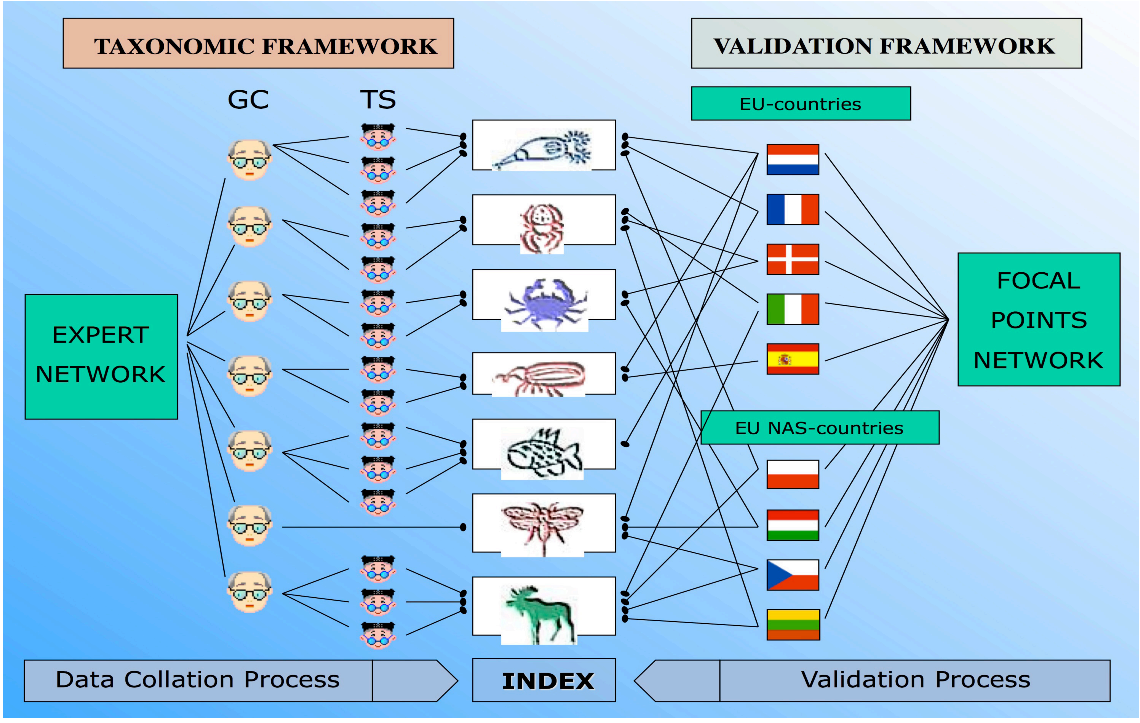

1.7.2 Euro+Med PlantBase

The PESI-TT dealing with vascular plants (ferns and flowering plants) is primarily based on the existing export network of Euro+Med PlantBase. This network was originally set up from 2000-2003 and continues its work up to the present. Seven Editorial Centres, each of them responsible for a certain share of the vascular plant families, and the Euro+Med PlantBase Secretariat are coordinating the activities of the taxonomic experts and the network of the regional advisers all over Europe and the Mediterranean. Some of the Editorial Centres also have the role of PESI botanical Focal Points. The Editorial Centres, each one for its set of plant families, are mainly responsible for updating taxonomic and nomenclatural information for Euro+Med PlantBase. They are doing this in close collaboration with the taxonomic experts for each group. The Euro+Med PlantBase Secretariat assembles the updates and takes care about data standardization and correction issues, so finalizing the taxonomic backbone of all European vascular plant taxa. The Secretariat distributes the updated plant families as they become available from the Editorial Centres into the network of regional advisers. The regional advisers, with their local expertise, mainly have the task to add floristic information and to correct erroneous entries. They receive taxonomically and regionally filtered slices of the database (e.g. all Rosacae occurring in Denmark and adjacent countries), and will check this against their local information. The feedback of regional advisers serves to update the information in Euro+Med PlantBase.

1.7.3 European and World Registers of Marine Species

The European Register of Marine Species (ERMS) was developed to provide a common open-access quality-controlled infrastructure to help coordinate marine ecological research and taxonomy in Europe (Costello 2000). Most of the 170 scientists that contributed to its establishment were career taxonomists, but database experts and data compilers were also involved. SMEBD was founded to own the database on behalf of these contributors, but with the long-term option of SMEBD having a role for overseeing other biodiversity databases. In 2002, SMEBD approved the Flanders Marine Institute (VLIZ) to host ERMS. VLIZ's nationally funded data centre provides a team of support staff that provide 24/7 access, archiving and IT support; and permanent funding to maintain the infrastructure as part of its mission. VLIZ integrated ERMS with other databases that provided for economies of scale and technology cost-efficiencies (Cuvelier et al. 2006). Further projects built and expanded this infrastructure in terms of content and functionality, such that it was planned to expand it to become a World Register of Marine Species (WoRMS) with regional (e.g. Europe, Antarctic) and taxonomic views (Costello and Appeltans 2008).

WoRMS is a collaboration between nearly 200 peer-selected taxonomic experts to publish information on all marine species online by 2010 (www.marinespecies.org). A single common database, called Aphia, hosts a growing number of Global Species Databases, several Regional Species Databases, and its first thematic databases (on harmful algae). Several GSD also include freshwater and terrestrial species. GSD on Crinoidea, Holothuroidea, Mollusca, Amphipoda, Trematoda, Echiura, Chaetognatha, Invasive Species, are in preparation, as are closer links with the GSD for algae, AlgaeBase. The editors are life-members of SMEBD. To date WoRMS contains over 142,743 valid species, 209,858 species names including synonyms, and 10,108 images. SMEBD has signed data supply agreements with Species 2000 for the Catalogue of Life, and the Encyclopedia of Life, and supplies content to GenBank and copies of the databases to 23 Institutions to add their quality control of species names.

1.8 How should the PESI-TT be better organised?

1.8.1 Challenges of the PESI-TT

Valuable databases need updating. Taxonomic checklists are invaluable reference databases for researchers and other users who need specific knowledge about a certain species. This also holds for the Expert Database developed by EDIT which will be advanced to facilitate direct communication However, to be useful in the long run, these databases need technical maintenance and regular update of content. In this chapter, focus is pointed at suggestions that meet these challenges.

Long-term leadership is currently lacking. The number of overlapping and fractioned facilities within the taxonomic community has decreased considerably the last years, resulting in fewer web-pages to keep updated – partly thanks to EDIT and PESI. At the moment the experts are well-motivated to update their data in e.g., Fauna Europaea. So the general needs for having a budget to update databases are decreasing. However, for larger rounds of upgrading, coordinated initiatives may still be needed every 5-10 years. It is a serious problem that no continuous leadership of the PESI-TT exists. Numerous backbone facilities are developed or are developing, but no single institution has the responsibility to assure that the maintenance is actually done. One of the problems is that the present budget for maintaining leadership is small and is limited by short period of time.

Monthly updates:
* create one website/interface for all experts to include their monthly updates by groupe
* send an automatic message 1x per month to these experts to invite them for registration of updates in their group
* send an automatic email to group coordinators (login, passw) for updates

Barriers for future updates of the checklists are connected to the institutional structure and to the decreasing number of career taxonomists. There is a lack of incentives for updating: Low merit for updating virtual collections for the career taxonomists, limited access to scientific resources for the non-career experts due to e.g. copyrights and the competitive structure leaves little incitement for open collaboration. In some countries, an institution can order the career experts to retire at a certain age, even though they may still be scientifically productive and are able to verify the updates in the species databases.

The number of experts has decreased due to priority given to other branches of science. It takes many years to become an expert within taxonomy, which means they are difficult to replace. The challenge is to find a pathway to safeguard that the experts effectively can and want to upgrade the databases in the future. A forecast analysis is valuable in order to show whether updating the checklists now, as opposed to updating in ten years from now, is recommended or not from an economic point of view.

Below are given several options for carrying out the updating labour in a way that keeps the costs down and at the same time strengthens the scientific level. PESI suggests delegating some parts of the work to non-experts so that the experts can concentrate more on the tasks only they can do. The career taxonomists can profit from the assistance of non-career taxonomists and the latter gain access to resources and user-friendly online facilities.

Even though many taxonomic online facilities exist, especially facilities that motivate the experts to update the various species directories still need to be developed. Information

harvesting facilities can be used by non-expert staff; community-driven networking and open review can be established to filter irrelevant information. Such an open structure depends on an open collaboration, which is a challenge to some traditional working experts. However, that leaves method development, biotope control, the final species verification and α-taxonomy in the hands of the experts – which in fact are high priority tasks for the traditional career-experts.

Provided the more open structure becomes available to the non-career experts, they can fill important gaps of scientific expertise as well. The development of such open collaborate networking has just begun and everybody needs to grow accustomed to changes.

1.8.2 Suggested work flow for input to checklists

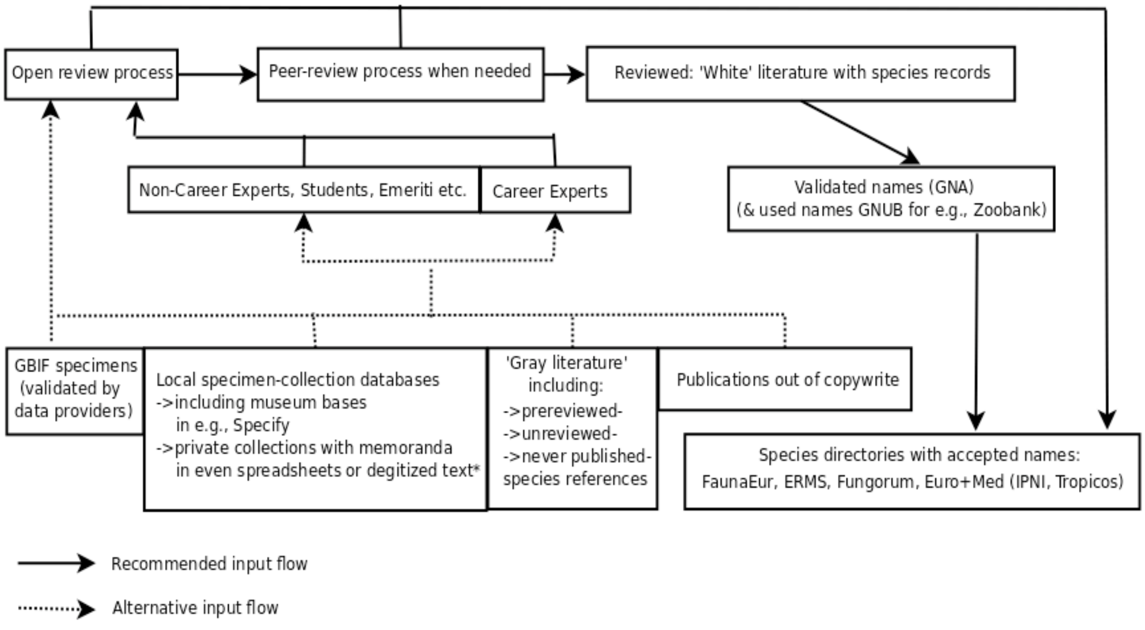

Figure 8. Outline of the suggested future work flow to keep updating the European taxonomic online resources (journals, checklists, and other databases) .

The present way to input data into the databases may change as more career experts retire. Open review (described in Annex 5 paragraph 4a) may take over as the first step to relief the reduced number of career reviewers. Open review may even be the only step depending on activity level of experts in the network. All qualified taxonomists are invited to participate in the reviewing process. The labour of the experts needs to concentrate on the tasks that require most taxonomic expertise, such as validating names in GNA, being gate-keepers of the species directories and filter incorrect information. The last five years experiences have given birth to different approaches to the upgrading of the species directories: the involvement of taxonomic societies, the Open review approach, more traditional institutional support and combinations of these possibilities. All of these are described below. This document focuses on the organisation model of Fauna Europaea. The organization structure of Fauna Europaea may be useful for other checklists to mirror, but any structure is acceptable providing the lists will be maintained.

The role of taxonomic networks incuding the societies

Some Fauna Europaea group coordinators are moving towards a model inviting international or European taxonomic societies to take over the governance of a particular taxonomic group. The Societas Europaea Lepidopterologica (rf. Ole Karsholt) could be an example in point. Such a model will delegate the maintenance of a Fauna Europaea taxonomic partition to a particular task group, which will mainly exist of non professional taxonomists. An important next step for Fauna Europaea in this context will be to advance the model into the virtual expert society. This should ease the addition of additional skilled taxonomists as Associate Specialist to the network.

It needs to be worked out in detail which taxonomic societies could be involved in the maintenance of the taxonomic checklists, and what tasks they could perform. Supplementing the society solution with the open review solution may also be an option.

The Open review approach in PESI-TT

Fauna Europaea is already working on an open forum that debates new species and their inclusion in the list (like the new Open Review process of Fauna Europaea). The group coordinators of Fauna Europaea act as gate-keepers who control the liability of the new species records or the revisions made. After a period of Open Review, the group coordinators (career or non-career experts) close the subject and upgrade the Fauna Europaea themselves or through the main leader. In case of serious doubt about the planned upgrade, any complaint about the group coordinators decision is forwarded to an advisor group. This group can accept or deny the decision. In extreme occasions the advisor group can even replace the group coordinator. Fauna Europaea considers mediating another layer of national reviewers of Associated Specialists before the group coordinators receive the review. That will bring further input to the checklists.

In Fauna Europaea, the involvement of regional experts, put forward by the National Focal Points, is progressing. They could take over the validation of the regional components of Fauna Europaea or participate as the Associated Specialist. This may be true for the overlap of Fauna Europaea and ERMS, as well. In a transition period VLIZ, helped by the GBIF automatic tools, may need to perform such revision of the checklists. This appears to be a labour-efficient procedure for quality control.

Another consequence of the retiring experts may be a need of a new way to actualize the existing knowledge. Many unpublished species records are left unused and may be difficult to revitalise in ten years from now. PESI can consider establishing an alternative input flow deriving from harvesting digitized publications out of copyright, from 'Gray literature', and finally from the specimen-registrations and collections. It is very time consuming sniffing useful species records from poorly registered collections and interviewing emiriti that did not manage to publish their latest scientific tasks. . Automated sniffing facilities (e.g. Google books and Google schools) can be combined with standardizing mapping facilities (using e.g., GoldenGate with Plazi). However, such detective work may be suitable for an online community, too. An online facility may be developed to gather the effort of the data providers of specimens to GBIF and the experts that upgrade the EU species directories can do both registrations in the same working process.

Updating checklists is a matter of motivation that leads to commitment in the networks. The career taxonomists have little time left for reviewing, which is why an efficient filtering using the open review process is important. Elaborating the geographic area and motivating experts from the focal points may be a way forward to recruit more experts and cover the taxonomic expertise gaps. Such Open review procedures are using the unlimited networking of the internet.

Institutional support
Support from institutions cannot replace the involvement of experts, but it is important with regard to the technical and organizational aspects. To provide a sustainable structure, current parts of the Fauna Europaea data management system are distributed to different partners:

- SNM (Copenhagen) takes on the maintenance of the expert database of Fauna Europaea
- The maintenance of the Fauna Europaea taxonomic database will go from ZMA (Amsterdam) to the EDIT Cybertaxonomy Platform maintained by BGBM (Berlin).
- The Fauna Europaea web portal will be merged with the PESI portal maintained by VLIZ (Oostende). A new specific FaEu web portal may be produced with a portal tool of the Cybertaxonomy Platform.

A combination of solutions
Eventually the validation and maintenance of the databases will require a combination of the aforementioned solutions. In some cases a database curator will trust an international society to fully take over the maintenance of the data on a group of organisms. This would work as long as it does not destroy an existing open review layer. Open review will assist in delegating part of the labour of career experts and a relief of incongruence on basic levels. An extra layer of non-career experts as Associate Specialists could be considered. The Society and the Open review solutions can easily supplement each other as long as a clear division of the labour is established.
Finally, the expert management, the checklists and the web-interface, already taken on by several institutions, would be supervised by SMEBD or even the planned research infrastructure LifeWatch. Ideally, no supervising or update session initiatives would be needed as a continuous update would be carried out by the Open review process of the community-driven network.
Some of the botanic, mycological and marine checklists may be inspired by the validation and updating structure of Fauna Europaea.

1.8.3 Expansion of the network

The PESI-TT builds on the existing networks of taxonomic experts of the Pan-European checklists (FaEur, ERMS and Euro+Med). A far more elaborate network is required to accomplish complementary expertise for the extensive checklists. One solution to this is a Virtual Expert Network system described below. Expanded networking is important to entice the recruitment of new experts and motivate both career & non-career experts to collaborate and contribute to cybertaxonomy and research initiatives.

Expanding network communication
As a first step, PESI would set up a model for communication and management of the expert network. This would build on the information-service on taxonomic experts, their expertise and ongoing projects developed by EDIT (WP2) in close collaboration with PESI (WP2). From this, a direct communication network is developed and involve integrating the database of taxonomic societies and other externa networks into the expert network (www.EditExpertNet.org). Finally, this is part of the cybergate being developed by EDIT WP5 and the species directory network of the PESI portal of PESI WP6.

The EDIT Taxonomic Experts & Networks (EditExpertNet) database provides searching facilities within expertise both among experts invited by EDIT and experts from outside EDIT

(e.g. DAISIE invasive species experts and from the database of ETI) both by name and by expertise. This is useful to find new experts and to discover existing expertise gaps. Further gap-analyses are conducted by ERMS and Fauna Europaea. These will be implemented in the EditExpertNet as well, to ease discovering gaps within expertise and promote inviting the missing experts.

PESI may consider organising networking services for experts and staff that support the taxonomic experts. This would then involve establishing new categories, which are straight forward to create, into the EditExpertNet. A certain overlap is inevitable for experts, organizers, informatics staff etc. A personal homepage with CV, literature lists is available for every expert in the EditExpertNet. When fully developed, the EditExpertNet will provide easy access to identify participants for cooperation purposes (e.g. meeting organizers).

1.8.4 An Integrated Virtual Expert Network system

Virtual collaborative organisations

Besides direct networking, the most important role of the EditExpertNet is to help experts to become more permanent members within the taxonomic networks: The EditExpertNet experts can be channelled to relevant virtual collaborative organisation, such as any of the Scratchpads.eu projects currently developed by EDIT WP6. In return, the presentation of already existing and upcoming societies/networks in the Scratchpads will add groups with a permanent link to the homepage of the EditExpertNet. The Scratchpads networks are already online with varying user-interfaces and engaging both new and present experts of the societies in networking. The personal touch of these networks appeals to some users and may remain as interesting references. The Scratchpads facility is scheduled to continue until at least 2016 and is planned to be maintained into the future. Retention and further development of such personalized webpage references are important to keep the motivation of the involved experts. In these Scratchpads advanced taxonomic facilities are possible and local checklists from these may be reviewed and exported into the PESI checklists.

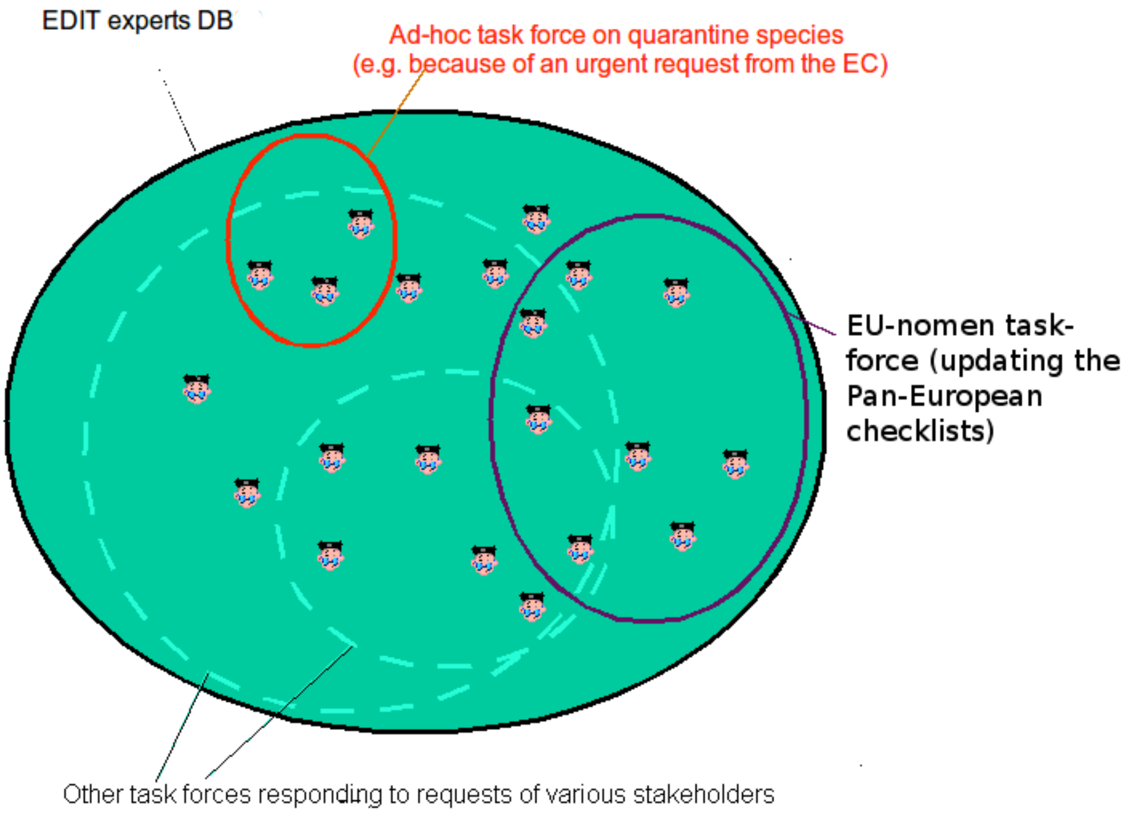

Figure 9. A single communication network (EditExpertNet) which will encompass the current EDIT expert DB and the PESI-TT.

Networking functionalities

EDIT WP5 and the PESI portal WP6 are developing a one-way entrance option to get access to all taxonomic facilities – a so-called "cybergate". From this gate, each expert will access the cyberplatform containing facilities like the integrated Pan-European checklist, the experts and an elaborated network. An integration of all these facilities is being carried out by PESI WP6 and will be accessible through the PESI Portal .

Links

Taxon records from each expert will be linked to the same taxon in the Species directories and in turn integrating this into the SMEBD web pages. Scratchpads developers should also consider this linkage as a priority to develop within scratchpads. Mechanisms to automatically monitor taxonomic expertise coverage, and therefore exposing gaps, will become part of the database – like previously done in Fauna Europaea, which EcoServe and PESI WP2 is working on today. Scratchpads offer aesthetic and personalized homepages for the societies and recruiting of participants between Scratchpads and EditExpertNet will simply be a matter of following a link. The two types supplement each other to catch all persons interested in taxonomy – the target group – and will be a foundation to update the checklists. Every member of the integrated Virtual Expert Network system would have the possibility to join the Open review layer of the European Checklists directly from the networks and vice versa. The final collaboration and integration will be considered in the different networks (initiative is taken by PESI WP2).

Searches

The EditExpertNet aims to fully cover the experts' contact information, their expertise and the desired networks they participate in. Like in the Expert database, the networks can be searched according to taxonomic interests. The experts are able to search for existing networks like the older societies, and EditExpertNet direct the experts towards these more permanent networks. This is also true for searching experts, choose the experts and invite them to an ad-hoc network or a permanent one. From here experts for a waiting list or a supplementing group coordinator can easily be headhunted. The goal is to sustain the experts in the Open review networks of the checklists.

Structure

The EditExpertNet will be structured according to Direct networking, Taxonomic specific networking and Networking communication including social contacts:

Direct network communication: As in the Listserv networks (like TaxaCom) the EditExpertNet offers Email networking. But the new facility will be elaborated to show status of availability, possible rejections and auto-logging of your conversation to your Email (like in the Google instant messenger/Chat). An expert may be visible online, and if (s)he sends out a question the recipient can see the request, and may reject the question by not responding, click hold-on which means I want to discuss this later or simply reply instantly. Visualized by a special icon, even non-registered guests from outside the database may be considered as respondents.

Taxonomic specific networks: These motivating networks could cover all specific aspects such as taxa interests, geographic area, taxonomic activities, methodological approaches and focal habitats. For instance, once beetle experts have inserted their interests in the database, any group leader or network moderator can search experts by interests and invite them to take part in an automatically generated network. The next logical step would be to participate in updating checklists or to Open Review a checklist.

Networking communication: The experts will be able to search for useful organisational and social networks: A fundraising network, a meeting organizers network, a recruits discussion network, a taxonomists abroad network, a female expert network, etc. (see Fig. 10). "You can have the best (IT) technology in the world, but if you don't have a community who want to use it and who is excited about it, it has no purpose" (Mr. Hughes, Obama's campaign leader). Mr. Hughes also pointed out as a basic recipe for success that all networks are more stable when they are physical and socially founded and they have a common goal (like biodiversity conservation) – particularly important for non-career taxonomists, who has no colleagues in the taxonomy realm. To involve themselves in cyber-taxonomy, the feeling of inclusiveness is needed in a combination of online facilities and further local involvement.

A standardized simple interface and dynamic networking

Standardized for all purposes: To reduce time wastage, it is essential that the experts use the same (standardized) interface independent of their chosen network. A user-friendly standard is particularly important as the experts will sign-up for their preferred networks allowing irrelevant messages not to enter their inboxes (see section 1.4.3).

Keep-It-Simple Stupid (KISS) interface: As soon as more experts have completed their registrations in the EditExpertNet, it will be easy to set up networks. For example if you wish to attract experts in the Netherlands to join a network on Coleoptera: All (s)he will need to do is to search Coleoptera AND Netherlands, write a message to explain her requests, click 'auto-

generate network' and the national experts will be invited to join this network, e.g., to work on red listing of Coleoptera species. Should an expert not conform to the netiquette then a ban system can easily be placed on this expert. The facility aims to use standard networking modules that are simple to upgrade.

Dynamic networking: Ad-hoc research projects, institutionally or individually based can easily be opened and closed depending on temporary needs. Active networks may become permanent while some may die out after few months, if the expected or immediate need of a certain network turns out to be of no interest. This opening and closing of relevant or redundant networks is the mainstream procedure in the Open Source communities and needs little labour to maintain.

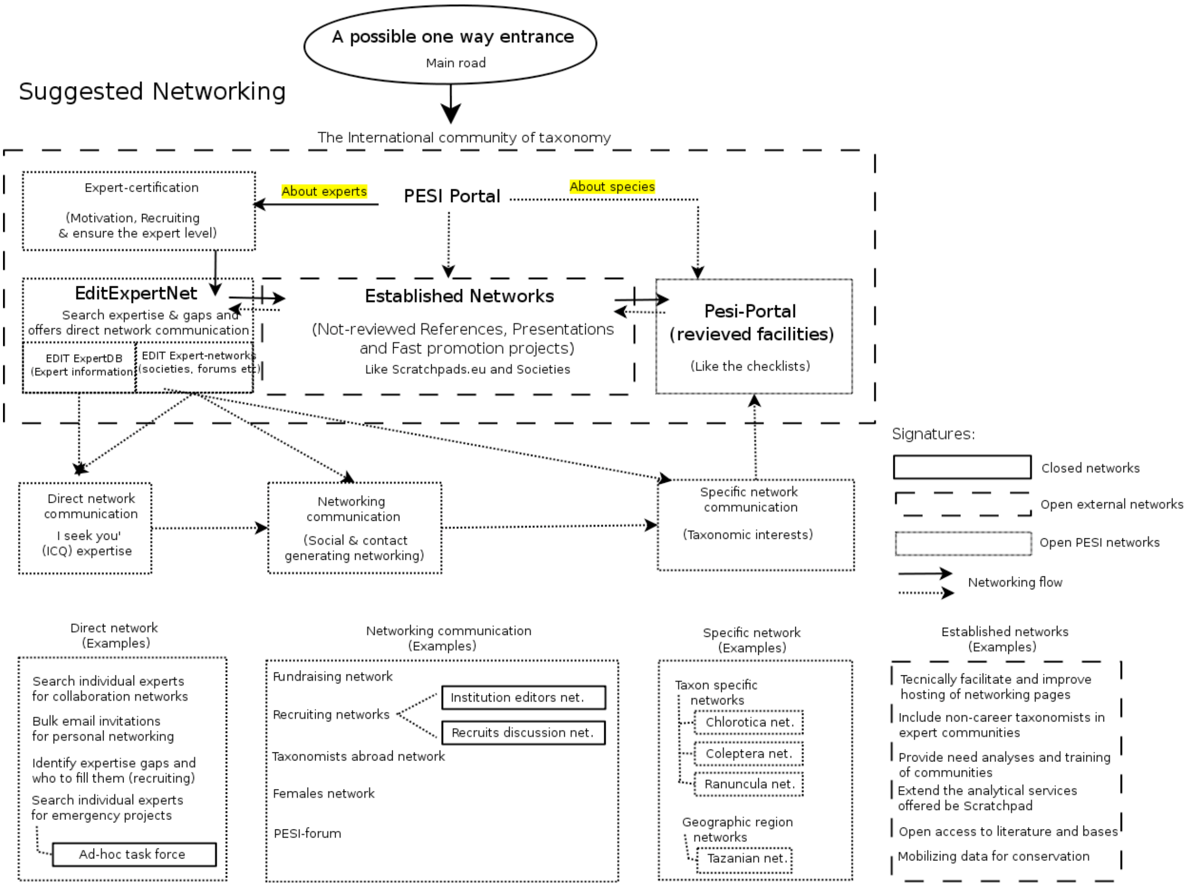

Figure 10. Outline of the envisaged taxonomic cybergate.

2 Accreditation system for PESI-ETW

2.1 Status, Merit and Inclusiveness

Status is clearly a factor for the non-career taxonomist when they are group coordinators in Fauna Europaea. This may not be that important for the career taxonomists. Many online tasks are added to the work of the career experts with less supporting staff. However, no employers can seriously oppose the fact that there is no substantial difference between updating an online database and a physical collection. An obvious way to get merit for updating serious checklists is to ask for the same merit for maintaining checklists & databases as for maintaining physical collections.

The Consortium of European Taxonomy Facilities (CETAF) took an important step in this direction in 2004, when this organisation decided to express "its support for European Directories of Species Names" (http://www.cetaf.org/Spec.database.pdf). Specifically, CETAF strongly recommended that

"*the employees in its member institutes, if they are invited to act as selected taxonomic specialists for European taxonomic databases, are allowed to perform this scientific work as part of their institutional tasks on the basis of the normal project decision structure of the institute, a policy to give proper credit and citation of the taxonomic specialist contributions (as established in the procedures of the database services).*"

Given that CETAF represents the major natural history collections, and thereby most of the largest taxonomic research institutions in Europe, this decision was crucial. It might be used as a model for a more inclusive agreement among institutions employing taxonomists.

Curation of electronic databases could be written directly into the contracts of the career experts of the museums. This may pose a problem, however, for experts without any curation responsibilities. Negotiation may be needed to convince the Universities to rank checklist updates alongside updating teaching resources and updating online presentations. Finally, for the most active group coordinators and database leaders, long-term effort has to be done if the taxonomic community wants these researchers to keep the virtual facilities in a scientific level. If so, an electronic database publication needs to be considered equivalent to paper publications.

At the short term, taxonomy needs to acknowledge gate-keepers of databases as being equivalent to museum scientific curators. Ten random interviews with experts revealed that merit is highlighted by all as the most important motivating issue to long-term update the databases as universities typically earmark no budget for this task. PESI advocates the introduction of merits in taxonomy within the university systems: updating of the species directories is a contract obligation likewise the collections and must be met accordingly.

At the long term, PESI may suggest a preceding science strengthening plan of the species directories that include reviewing and certification (which PESI may carry out anyway). PESI could ask one of the universities to be a pioneer in charge of a project "Acquire merit in Taxonomy". After such experiences, we would have the final conclusive argument for the Universities to rank the online papers & databases alongside the hardcopy papers or the merit given for the museum collections.

Finally PESI may consider monitoring the degree of expert engagement and success: Better citations of experts by improving transparency of who inputs the species and moderates the

scientific basis. Better merit for the experts through measuring the number of online unique searches of the bases. Monitoring of each database throughout a period of 4-5 years may act as a measurement and may provide arguments for further funding.

For non-career taxonomists (amateurs, emeriti etc.), credit is important, too. In their case, credit may involve recognition of their status as taxonomic experts - as for co-authorship to papers for which they have delivered substantial data (see EDIT, 2008a). Besides, the classic community driven networking of the OSSp provides status according to meritocracy: The status inside each network belongs to those who points out the weightiest arguments based on the highest scientific references. This opens up more options to the best non-career taxonomists. In open networks the non-career taxonomists are not prejudged as they enter a discussion, as it is regarded bad etiquette to justify your arguments based solely on someone's title. The non-career experts can prepare themselves better and give more qualified input if they had Open Access to the scientific resources. For them easy access to high quality data and scientific literature is an important incentive in itself.
The non-career experts will be motivated to do their very best as they are invited to Open Review, and many eyes may increase the accountability of the checklists. That is an immediate advantage. Besides, for all taxonomists, time is a limiting factor, and time-reducing tools are always high on the wish list. Efficient collaboration is an obvious way to increase efficiency and saving time. A way of networking that intends to help all taxonomists is given in Figure 10.

2.2 PESI citation system for online data

In times where impact factors and citation indices have become the most important indicators of scientific research activity, PESI needs to find an answer to the question of many career taxonomists: why should I invest my time in an activity which is not rewarded at all by my department? Many departments and institutions, who in turn have to report their scientific output to the university they are part of, sometimes even strongly oppose engagement of its staff in networks which do not yield measurable output in terms of highly valued publications in peer-reviewed journals with a high impact factor. To partly overcome this difficulty, PESI will implement a credit system to all contributors, similar to existing citation recommendations in Fauna Europaea, E+M PlantBase and ERMS. This should be an easy system as in ERMS, where users can immediately copy and paste the correct citation. PESI strongly recommends to editors of scientific journals to make it their editorial policy to cite in their articles the online databases not only anonymously, as a whole, but always with the respective authorship of certain taxonomic groups. Such a credit system will raise the citation index and impact of the Pan-European Checklist, and thus will in turn motivate more career taxonomists to contribute to its treatments and so strengthen the PESI taxonomic taskforce. PESI will also seek to measure the impact of its product by counting the access to the online database regularly.

A citation of the updating experts may motivate more experts to update (Costello, 2009). How to measure online database contribution and evolution of a PESI credit system for online publishing online data can be worked out by PESI partner SMEBD who has currently delivered the preliminary report on the government of copyright of electronic biodiversity data (M2.3) in preparation of the final report (D2.2).

2.3 *Expert* competency clarification through *certification*

Expert-certification procedure(s), we advise to be developed to safely let more non-career taxonomists, students etc. be included in the EDITexpertNet.org and finally the checklists networks.

In C.1.2. "Discover & head-hunting experts from publications", taxonomic publishing scientists are straight forward to be invited. In C.1.3. "Discover & head-hunting acknowledged experts from institutions & networks" we mentioned that EDIT and SMEBD invited experts have recorded their names, taxon, projects, references etc. into the database, and other authorities may be advisable.

However, a more applied scientific approach for the experts may be suitable for especially non-career taxonomists: That is a "*Competency clarification*". It is a natural continuity as upgrading hobby taxonomists to experts (see C.1.1). "What is an expert, and how do we secure that maintenance is done by an appropriate level of the experts?". Unclear competences to degrade experts or permissions to manage input of the repositories. This will lead to dispute among the taxonomic experts (as in the Open Source society). Passing the highest "Expert certification" level will help the community to let an expert be part of the Open Peer Review layer.

Registrations of physical collections may be classified according to the two levels of driving licenses, and five levels of certifications to be used in the databases & collections. An existing example is the Slime Molds Certificates, the Field Identification Skills Certificate (FISC) for plants or the fish-expert identification levels of the Hobart University[47]. Then all can participate which is highly motivating and the non-career taxonomists requested an option to obtain status[56]. Different levels of university courses may be regarded as certifications – University-course in eg. bachelor level basic botany may be equivalent to certification level 4, Higher botanic taxonomy to level 2 and a supervised specialization in palms provide an expert-level 1, in palms only. For scientific articles the researchers will now be able to choose search-results of identifications performed by the highest classified experts, only.

Certification may be exposed to many branches: a certain EU-standardized certification level may grant permission to sell fungus for restaurants, certifications to spread pesticides, to cut rainforest trees, to fish commercially etc. PESI can justify the inclusion of these certified experts into expert networks and as reviewers of checklists, filling some of the current gaps in expertise. Using the same technique as for the Taxonomic Driving-license for training (see C.1.1), R13: an Expert certification is valuable for competency clarification of esp. non-career taxonomists and for sharing collecting permits, access protected collections and other resources with these new experts. Invitations from the EditExpertNet database will automatically be send to new high level certified experts.

References (PESI ETW is above)

Bourgoin, T. & Silvain, J.-F. 2008. Systématique et taxinomie. – Commision scientifique de l'IFB. Réflexion stratégique: 41-50. http://www.gis-ifb.org/content/download/2067/10761/version/1/file/IFB+FR+BD.pdf.

Costello, M.J. 2000. Developing species information systems: the European Register of Marine Species. –Oceanography 13 (3), 48-55.

Costello, M.J. & Appeltans, W. 2008. Taxonomic editors plan a World Register of Marine Species (WoRMS). –MarBEF Newsletter No. 8, 36-38. ISSN 1649-5519.

Costello, Mark (2009). Motivating Online Publication of Data. BioScience 59(5):418-427. 2009.doi: 10.1525/bio.2009.59.5.9 http://www.bioone.org/doi/abs/10.1525/bio.2009.59.5.9
Cuvelier, D., Claus, S., Appeltans, W., Vanhoorne, B., Vanden Berghe, E. & Costello, M.J. 2006. European Register of Marine Species (ERMS) – plans turning into reality! – MarBEF Newsletter No. 4, 14-15.
EDIT 2008a. Professionals and non-professionals as producers and users of taxonomic knowledge – synergies, conflicts and the role of taxonomic societies. – Report from a workshop of EDIT (Toward the European Distributed Institute of Taxonomy), Rodalquilar, Almería, Spain, April 23 – 24, 2008. – http://www.e-taxonomy.eu/files/C2.3.3_Workshop_ALMERIA_report.pdf
EDIT 2008b. Taxonomy in Europe in the 21st century. Report to the Board of Directors. - http://ww2.bgbm.org/EditDocumentRepository/Taxonomy21report.pdf
Enghoff, H. & Seberg, O. 2006. A taxonomy of taxonomy and taxonomists. – The Systematist, Newsletter of the Systematics Association 27: 13-15. – http://www.systass.org/newsletter/TheSystematist27.pdf
Fink-Jensen, J. & Hansen, W.H. (eds.) 2007. Københavns Universitet Årbog 2006. - http://www.ku.dk/publikationer/pdf/arkiv/aarbog/aarbog2006.pdf
Fontaine, B. <no date>. Fauna Europaea. What remains to be discovered, where and by whom? - http://zoology.fns.uniba.sk/edit/meetings/documents/Fontain%20-%20FaEu.ppt Godfray, H. C. J. 2002. Challenges for taxonomy. – Nature 417: 17-19.
PESI 2008. Grant agreement, annex 1, “Description of work”. Available from http://www.eu-nomen.eu/pesi/index.php?option=com_remository&Itemid=56&func=select&id=7
Ramos, M.A. (ed.) 2006. Museo Nacional de Ciencias Naturales, Consejo Superior de Investigaciones científicas. Memoria 2005-2005. – Museo Nacional de Ciencias Naturales, Madrid, 271 pp.
Wheeler, Q. D. (ed.) 2008. The new taxonomy. – The Systematics Association Special Volume Series 76, CSC Press.
A. StarL.S. & Griesemer, J.R. (1989). Institutional Ecology, `Translations' and Boundary Objects: Amateurs and Professionals in Berkeley's Museum of Vertebrate Zoology, 1907-39 http://sss.sagepub.com/cgi/content/abstract/19/3/387. Social Studies of Science, Vol. 19, No. 3, 387-420 (1989). DOI: 10.1177/030631289019003001.
B. Thomsen, 2000. Virtual Museums: Inter-Museum Collaboration. http://www.ischool.utexas.edu/~vmuseum/thomsen.html
C. Nathan, Bos et al. 2007. From Shared Databases to Communities of Practice: A Taxonomy of Collaboratories. http://www3.interscience.wiley.com/journal/117979323/abstract?CRETRY=1&SRETRY=0
D. Tsiavos, P. (2007) The Museum as a Cathedral and a Bazaar: Tracing flows of rights in the UK cultural, memory and education sector. http://icommons.pentabarf.org/programme/iSummit08/attachments/8_ISUMMIT%202008_05_04_08_CURATION.pdf

3 Action plan

3.1 Management of the PESI ETW

The PESI European Taxonomic Workforce or its its Taxonomic Taskforce (PESI-TT) is not limited in time to the currently EC-funded PESI project but it needs to be maintained and managed for many years.

Especially the many recommendations (*R1-R25*) concerning both career and non-career taxonomists in the section 1.4 (PESI-ETW) need leadership and act upon the delegation.

There are established networks for taxonomic institutions in Europe, notably CETAF for major museums and EDIT for a slightly different range of organisations. EDIT plans to network with the many taxon-based societies which include both career and non-career taxonomists. SMEBD provides a legal platform for individual taxonomists that are contributing to online taxonomic infrastructures. SMEBD was founded in order to manage,

through the transferral of ownership, the ERMS database on behalf of the contributors, but with the long-term option of having a role for overseeing other biodiversity databases. It currently also includes to the Fauna Europaea and Euro+Med PlantBase databases. Thus, SMEBD is well positioned to hold Intellectual Property Rights (IPR) associated with the Pan-European Species-directories.

In view of the future management of the PESI TT the following action items were formulated.

1) In the long term, the executive management of the PESI-TT needs to be handed over to a (semi-) permanent body. At present, SMEBD seems to be the most realistic option, since SMEBD exists as a legal body, however with very limited resources.
Action item: Take steps to help SMEBD to come in a position to take on future management of the Pan-European species databases, including the PESI-TT (UvA, SNM, SMEBD, EcoServe, others?).

2) SMEBD may need to generate its own finances to cover administrative costs, including annual audits and some coordinator time. SMEBD is currently discussing possible ways to generate revenues. Another option is for SMEBD to apply for a major FP7 coordination action beginning in 2010.
Action item: Identify possibilities for future revenue generation for SMEBD (SMEBD, EcoServe,others?)

3) For Euro+Med in collaboration with Kew, further funding beyond the lifetime of PESI is very important. A high need of data completion (e.g. for countries of former Yugoslavia) and data cleaning (ensure data consistency) exists for this base.
Action item: Identify possibilities for future funding of Euro+Med (FUB-BGBM, others?)

4) If the SMEBD option fails for whatever reason, contingency plans should be in place. These could consist in individual institutions taking responsibility for (part of) the Pan-European species databases.
Action item: Contact potential alternative future managers of the Pan-European species databases, including the PESI-TT, e.g., larger natural history museums, botanic gardens, major taxonomic societies (FUB-BGBM).

3.2 Motivating contributors

1) Since some parts of the work of the PESI-ETW will remain unpaid, motivating taxonomists to join and stay in the PESI-ETW is an important task. PESI should take political action to motivate scientists to contribute to the Pan-European species databases.
Action item: Liaise with the EDIT Board of Directors' working group on evaluation metrics for taxonomic work (UCPH-SNM, others?).
Action item: Develop online networking facilities in the framework of the expert and society database being developed by EDIT (UCPH-SNM, a new milestone is planned for 06-2010).

2) PESI should explore the possibilities offered by the Open Source Society given in section 1.4, and collected in Appendix 1. After each suggestion an ***L*** or an ***M*** is marked.
Action item: Two small PESI groups need to be established. A committee and a manager group. The committee group need fast to select suggestions, write some requests to authorities and delegate who of the manager group that will implement each suggestion. ***L*** marked needs decisions.

Appendix 1. Open Source Taxonomy suggestions

The suggestions, below, comes from section 1.4 and the Action Plan is given in 3.2.

R1: Some projects can immediately be financed through online donations. This can be extended by *creating an EU-fund for Biodiversity and by cost reductions such as exchanging computer-licensed software with free software.* ***L***

R2: How to open up the collections may be a matter of deployment: A.Unica specimens or the crown jewels are presented in protected exhibition cases for local presentation, only, B. research collection may be Global ownership to scientists, secondarily, in long time loan, C. the duplicate collection may be primary lent to the society meetings or teaching before it is open to the public, Combining local physical collections with data of the virtual collections. ***L***

R3: The ever updated "Print on Demand[64]" must be highlighted. ***L***

R4: PESI may take initiative with EDIT and CETAF to request the EU politicians to take up the U.S. Public access policy[31]. ***M***

R5: Until settled, an option to Open Peer Review is inserted in all publishing resources (journals as well as databases), and as a backup, two responsible experts to finally confirm the review quality. For the checklists, we invite a number of experts for the Open Review layer, which also act as a waiting list of experts to be headhunted for the final (traditional) Peer Review layer. A waiting list of stand-in group-coordinators will relief the first appointed group-coordinators, as well. ***M***

R6: Who participate with what text is measurable using the "Stream management" method of WikiGenes[50] "where authorship matters". ***L***

R7: to provide citable time-stamped publications, different versions can be recognized as Repository versions like time-stamped versions of the OSS applications[20z]. XML and RSS feeds can filter according to relevance using selected keywords, and referring to the latest repositories will keep the experts attention to the ever updated version. ***L***

R8: The PESI databases code, we advise to spread to any serious partner to ease integration, maintenance and further development. ***M***

R9: Especially projects that are funded can be better implemented through common and transparent discussions at e.g., Sci-Mate.org[20H] in stead of using Email-collaboration. ***M***

R10: More projects can start completing LFA templates[20P] that are common for career as we as for registered non-career taxonomists. ***M***

R11: PESI needs to take decisions about any other institutions or networks that may offer reliable experts and criteria of who to include. (Is a recommendation of an EditExpertNet expert sufficient?). *Finally, lists of or requests need to be send to invite the experts and attempt to canalize them to the European expert network/ community service* (see C.2). ***L***

R12: an evaluation of publishing experts may be verified by an internal Review layer in the EditExpertNet by the existing experts. ***M***

R13: Taxonomic Driving-license for training, to make taxonomy popular and to spot talents. An Expert certification is valuable for competency clarification of esp. non-career taxonomists. As a pay-back for the effort, we'll share resources with these new experts: such as collecting permits, access to protected collections and costly journals. ***L*** *and* ***M*** *for fund-raising.*

R14: all are aware of working for an easy understandable common goal such as 'Biodiversity conservation' ***L***

R15: many motivation procedures and methods must be utilized, as they are the key to the needed (critical mass) activity. ***M***

R16: Emails need to be send to all possible networks to request experts to register their expertises, followed by a request to create networks and update each year. The request must highlight the options the experts will enjoy as a quid pro quo for their involvement. ***L*** *and* ***M***

R17: The experts of e.g., PESIs Focal Points and the NHM partner of EDIT can get special limitations and be addressed as a network, only. Open Source networks may accept that they can have direct access to their members. Decisions of the status of each single network need to be taken. ***L***

R18: PESI can request the experts to go from traditional Peer Review and include a waiting lists, over an Open Peer Review layer safeguarded by a traditional Peer Review layer and ending with only an Open Peer Review layer performed by all expert members of the waiting list – a clear relief for peer-reviewers and database administrators. ***L***

R19:The CSSO is suggested in all expert facilities of taxonomy to ease the login. ***L***

R20: usage of contracts, system integration, collaborative content management, agree on delegated assignments and produce co-authored output, inspired from Wong, I. 2007[20]. For network users, a warranty contract is obvious. ***L***

R21: an OSS rewarding method of the meritocracy called "Stratification mapping" (Goodman, S. 2006[23]), that include all assets that brings authority to a network participant: 1) Human Assets: Accurate information of specialized knowledge. 2) Honorific Assets: Network members may be given a score or rank on a website that is displayed by their name as an honor or award. 3) Reputation system: rated up or down by other user. 4) Social Assets: a person can be considered central in a network by accumulating a large quantity and specific quality of social ties. 5) Political Assets: Many forum governments enforce policies that give differential control over the website content. Finally, the moderator and administrator usually have unchecked power to delete and change all messages, while readers are given limited authorization. Poorly rated messages may even be hidden from view by the moderators of Slashdoc.org*. Finally, to avoid loosing your status you need to follow the networks 'netiquettes'[62] .* ***M***

R22: To fire up the networks we may call for debaters, illustrate interesting networking in practice, e.g., show off the value of registered expertises, and the close connection from debate on taxa to keep upgrading the checklists. Externally, we need further integration between the PESI-facilities, cross-linking to large sites, press coverage of our features in TaxaCom, in publications and in highlight out facilities to our collaboration partners. ***M***

Table 4. Action items aimed at the log-term maintenance of the taxonomic work force. Institutional dependent Principles in bold, Institutional facilitated Open source society (OSS) Principles *<u>in underlined italic</u>* and independent OSS principles *in italic.* The combinations below are our suggestions using institutional, institutional facilitated and independent solutions. It mirrors the OSS success of today.

Principles	Practical 'tools'	Intermediate aims	Budget available	Tool(s) available	SuggestionWhen	Suggestion Who
**Institutional semi-permanent leadership**	Staff to overview maintenance	Taking initiatives to updating rounds	(No) Admin OK	Staff exists	2010 → .... (semi-perm.)	SMEBD, UvA, SNM,EcoServe
Community driven networking leadership	A well structured & empowered networking system (an OSS net) Scratchpads+EditExpertNet+Portal	Create an integrated Virtual Expert Network system	(Yes), but needs integration	(No, but developing)	2009 → .... (semi-perm.)	UvA, SNM, SMEBD,BGBM, EcoServe
**Provide a survey**	A forecast analysis	To prevent costly or too late action	No	No	2011	Impartial agent
<u>Society leadership</u>	An empowered society network	Societies take initiative to upgrade	No	No	2010 – 2011	Fauna Eur. ?
**Give status to headhunted experts**	Inviting experts into the networks	Get experts from GSD, Focal points, Associated Specialist (& outside EU)	Yes	Yes	2010 – 2011	SNM, BGBM
<u>Give status + include non-career experts</u>	Inviting non-career experts into Open review & EditExpertNet	Urge non-career experts to participate	Yes	(No, but developing)	2009 → .... (semi-perm.)	SNM, NHM, SMEBD
<u>Give status to non-career experts</u>	Co-authorship in articles and references in the databases	Urge non-career experts to participate	Yes	No	2009 → .... (semi-perm.)	EcoServe
<u>Retain the expertise</u>	Sniffing species info, harvesting and mapping facilities	Systematically find unused or disappearing info for checklists	No	Yes	2009 → .... (semi-perm.)	No partners but staff available
**Retain the expertise**	Sniffing species info., harvest and mapping facilities	Systematic find unused or disappearing info. for the checklists	No	Yes	2011→ .... (semi-perm.)	No partners but staff available
**Retain the expertise**	Political change retirement age to unlimited	Urge career-experts to continue	(Yes)	Staff exists	2010 – 2011	Steering Com.
**Relieve the experts**	Political change University merit	Urge career-experts to participate	Yes	No	2010 – 2011	Steering Com.
**Relieve the experts**	Hire new taxonomic support staff	Urge career-experts to participate	No	Staff exists	2010 → ... (semi-perm.)	A fund-raiser, donor, sponsor
<u>Keeo experts</u>	Direct, Social & taxonomic networks	Urge the experts to participate	Yes	Yes	SNM	
Open Access to taxonomic resources	Open online and physical facilities	Urge esp. non-career experts to participate	(Yes) more are needed	(Yes) more are needed	2009 – 2016	SNM, VLIZ, BGBM
<u>Exemplary principle</u>	Expert certification	Recruiting experts to participate	No	Staff exists	2011-2016	SNM

List of acronyms

ATBI	All-Taxa Biodiversity Inventory
CATE	Creating a Taxonomic e-Science
CETAF	Consortium of European Taxonomy Facilities
CoL	Catalogue of Life
EDIT	European Distributed Institute of Taxonomy
ERMS	European Register of Marine Species
ETW	European Taxonomic Workforce
GBIF	Global Biodiversity Information Facility
GC	Group Coordinator
GSD	Global Species Database
IPR	Intellectual Property Rights
MarBEF	Marine Biodiversity and Ecosystem Functioning
MCT	More Career Taxonomists
OS	Open Source
OSS	Open Source Society
OST	Open Source Taxonomy
PBI	Planetary Biodiversity Inventory
PESI	A Pan-European Species-Directories Infrastructure
PESI-ETW	PESI European Taxonomic Workforce
PESI-TT	PESI Taxonomic Taskforce
SMEBD	Society for Management of Electronic Biodiversity Data
TC	Taxonomic Coordinator
TS	Taxonomic Specialist
WoRMS	World Register of Marine Species

Configuration History

Version No.	Date	Changes made	Author
0.1	30th April 09	First draft for circulation within PESI	Henrik Enghoff & Phillip Bøgh
0.2	2nd June 2009	Updated version (modified after partners' comments)	do.
1.0	8th June 2009	Updated after second round of partners' comments	do.
1.1	22nd Sept. 09	Updated version (modified after partners' comments)	do.
2.0<br>2.1	4th Oct. 2009<br>7th Oct. 2009	Updated version (modified after partners' comments)<br>Rephrasing and editing of main document	do.<br>Roisin Nash & Louis Boumans
3.0	31th Jul 1010	Updated version	do.
3.1	24th Aug 1010	Updated version	Phillip Bøgh & Roisin Nash
