# Supplementary material for: PESI - a taxonomic backbone for Europe
Source: Biodivers Data J. 2015 Sep 28;(3):e5848. doi: 10.3897/BDJ.3.e5848 (PMC4609752; doi:10.3897/BDJ.3.e5848)
Supplement: Supplementary material 3 — The Government of IPR of Electronic Biodiversity Data [file biodiversity_data_journal-3-e5848-s003.pdf]

The Government of IPR of Electronic Biodiversity Data

[PESI Deliverable D 2.2 & PESI Milestone M 2.3]

Prepared by Society of the Management of Electronic Biodiversity Data Ltd (SMEBD), B23 KCR Industrial Estate, Ravensdale Park, Kimmage, Dublin 12. www.smebd.eu .

This report constitutes Deliverable 2.2 of Work Package 2 on the coordination and integration of European expert networks. The value of the taxonomic databases is highly dependant on the input of taxonomic experts who compile, revise and validate the data. A major concern is that in the current academic setting the work on reference databases receives little acknowledgement. In its breadth this issue and potential solutions are the topic of D2.1 "*The European Taxonomic Workforce*".

The current Deliverable 2.2 focuses on one part of the problem, namely the management of the intellectual property of the contributors to the database: how use of this property is licensed and how proper attribution and citation are promoted.

This report lays out the possible database identification systems and licensing schemes. It serves as a background document which was used for discussion among the contributors of the Pan-European Species-directories Infrastructure in order to decide on the government of copyright of electronic biodiversity data within PESI.

Contents

Contents 2

1 Introduction 3

2 Background 3

2.1 Relevant copyright legislation 3

2.2 IPR and natural science 4

2.3 Identifying databases 6

2.4 Licensing 8

2.5 Alternative licences 8

3 PESI and IPR 9

3.1 SMEBD IPR model 10

a. Data ownership statement 10
b. Terms of use of data 10
c. Termination of licence and rights of use 11
d. Disclaimer 11

3.2 Identifying databases in PESI 12

4 Advantages and disadvantages of SMEBD IPR model 12

5 Advantages and disadvantages of Creative Commons Attribution-ShareAlike 12

6 Citation 13

7 Data submission protocol 14

8 Software & tools developed within the PESI framework 15

9 Conclusion 16

10 References 17

Appendix 1: Glossary of terms with respect to IPR in databases 19

Appendix 2: SMEBD - standard agreement with a contributor 21

Appendix 3: Potential alternative IPR model 22

1 Introduction

The PESI portal[1] will provide improved access to, at minimum, the three main taxon registers in Europe namely: the European Register of Marine Species (ERMS), Fauna Europaea and Euro+Med PlantBase, extended by at least two Phyco-Myco databases on European fungi[2] and European desmids.
While SMEBD will continue to manage these databases an important question is raised - how will PESI manage the intellectual property of the contributors now available through the PESI portal? Following this it should be made clear how the use of this property will be licensed and how proper attribution and citation will be promoted into the future.

2 Background

Databases containing large amounts of biological data, from a local to a global scale, are currently available via the World Wide Web. This data, which in most cases can be downloaded with the click of a mouse, has allowed scientists to carryout in-depth analysis of critical issues affecting society today. Current topics including the impacts of climate change and the decline of marine biodiversity worldwide through to problems in relation to public health are now being assessed for patterns and elucidation over scales never before thought possible. However, this new source of information through online 'data publication' is highly susceptible to unauthorised access and sharing by users. Ideally a balance would be decided between the protection of data and the level of access to data, particularly where it has been found that data is being inappropriately applied by users.

Intellectual property rights or licensing are both tools which could be applied to digital online works and databases to provide a form of control as opposed to the outright sale of data as happens in some online journals. Licensing is a very flexible tool that provides a range of means of sharing and access to digital works. In order to efficiently implement licensing tools there is a need for relevant copyright legislation, appropriate identification methods, technological protection methods, secure networks and fast enforcement instruments (Koskinen-Olsson, 2008).

Intellectual property laws protect a variety of intellectual creations including copyrights which protect expressions, trademarks which protect names and patents, which protect ideas. For example, ERMS is an authoritative taxonomic list of species occurring in the European marine environment where the authors are a group of scientists who compiled and created the database. In this case, the actual database would constitute an expression, and would be protected under copyright. The name ERMS would indicate the authors as the source of the database, and could be protected by trademark. The actual idea of how to create an authoritative taxonomic list of species occurring in the European marine environment as described in the database could be protected by patent (Costello *et al.*, 2008).

It is important to distinguish that names, ideas, facts and short statements are not covered by copyright. As most online biodiversity databases are classified as a form of expression, the access and sharing of these 'expressions' are governed by copyright. Therefore the present discussion document focuses on the applications of copyright in the use and sharing of online databases rather than the issues of trademark and patents.

2.1 Relevant copyright legislation

---

1 EU-Nomen portal: http://www.eu-nomen.eu/portal/
2 http://pesi.indexfungorum.org/

Copyright for online material is subject to international conventions, namely the World Intellectual Property Organisation (WIPO) Internet Treaties of 1996, the WIPO Copyright Treaty (WCT), and the WIPO Performance and Phonograms Treaty (WPPT). These international copyright standards have been updated specifically to address the internet era and to lay the basis for an appropriate expansion of electronic databases (WIPO, 1996). However, it is not reasonable to accurately generalise about online copyright legislation since there are considerable differences among countries around the world.

The U.S. Copyright Act of 1976 (94th Congress, 1976) states that the items of expression can include literary, scientific and artistic works; audio-visual works, sound recordings; and architectural works. Online expressions include text and data on webpages, contents of emails, software, sound files, pictures, etc. From the moment an original creation is in an appropriate form it qualifies for copyright protection.

The EU Directive 96/9/EC on the legal protection of databases (EC, 1996) is a European Directive concerning copyright law. It complements the copyright law on the treatment of databases, and provides new and unique rights for the creators of databases which do not qualify under copyright laws. The Directive defines a database as "a collection of independent works, data or other materials arranged in a systematic or methodical way and individually accessible by electronic or other means". Non-electronic databases are also covered, however the computer program used to create the database is not included in this directive. Under article 3 of this directive, databases which, "by reason of the selection or arrangement of their contents, constitute the author's own intellectual creation" are protected by copyright as collections and no other criterion may be used by Member States. The restricted actions by the database copyright directive are analogous to those for other kind of creations (EC, 1996) including:

- The complete or partial reproduction by any means and in any form.
- Any alteration of the database, such as translation, adaptation or rearrangement.
- The public distribution of the database.
- Any display or performance to the public.

However, the database directive allows for the lawful use of databases by any user including the:

- Reproduction for the purposes of private use of a non-electronic database.
- Use for educational or scientific research purposes, considering that the original source is cited.
- Use of the databases for public security or an administrative or judicial procedure purposes.

Copyright protection generally ends seventy years after the death of the last publicly identified author.

2.2 IPR and natural science

Biodiversity and IPR, by their very nature as established disciplines, involve issues that can be quite intricate to grasp; so it is no surprise that when these two concepts meet, a degree of ambiguity accompanies the associated discussion. Achieving an equitable balance between intellectual property rights to access and use scientific knowledge, including that of natural science and biologically derived products, is notoriously difficult to achieve.

IPRs such as copyrights, patents and trademarks are centuries old. It wasn't until the 1930s that IPRs began to extend to living entities and scientific discoveries, the first being asexually producing plant varieties. Until recently, the majority of countries across the globe allowed farmers and other plant breeders to be exempt from such rights as "limit right regulatory powers over the marketing of 'their' varieties" as long as they did not indulge in branded commercial transactions (Kothari & Anuradha,

1999). However, over the past two decades certain amendments, such as that of the UPOV (Union for the Protection of New Varieties of Plants), has practically eradicated that exception for farmers and breeders. These sorts of IPR amendments have become further finalised, especially over the course of the last decade, in various Trade Related Intellectual Property Rights agreements (TRIPs) and through the Convention on Biological Diversity (CBD). In their fundamental state they represent two international treaties within the World Trade Organisation (WTO) that have "significant implications for the nexus" (CBD, 1992) of intellectual property rights (IPRs), biodiversity and connected Nature Science knowledgeable advancements.

- The CBD requires international parties to protect biodiversity and the traditions associated with biodiversity whether it is from indigenous communities or other local communities associated with biodiversity of any sort. The CBD paves basic elements and guidelines for those wishing to access biodiversity resources; databases or others (CBD, 1992).
- The TRIPs Agreement compels those concerned parties to amend their national IPR regimes to meet international standards, which could have momentous inferences for biodiversity, and those in charge of managing the biodiversity resources; database and others (TRIPs, 1994).

Right across the spectrum of national and international agreements two broad philosophical approaches motivate the decision to grant IPRs on the products of human intellectual effort and ingenuity. The first of these approaches can be seen predominating in many civil legal systems across continental Europe. This approach believes that the products of the human mind are stamped with the personality of their creator, inventor or author, thus, bestowing him or her with a moral as well as an economic claim to exploit those products to the exclusion of third parties. The second approach and the one which has mostly been attributed to the partnership of natural science and IPR takes an active view of intellectual property. Legal protection for the products of human intellectual effort and ingenuity is granted not because of a moral commitment to reimburse creators or innovators, but more so because the products they create enrich a society's culture and knowledge and thus increase its welfare. This incremental approach used in many international intellectual property systems was originally expressed with the intention to provide an "adequate incentive" for creators, inventors and natural scientists to invest "time, resources and the intellectual capital needed to create intellectual property discoveries and property" (Helfer, 2002).

An example of this Instrumental approach to practice in the world of Natural Science can be seen through the discovery of new plant varieties being afforded legal protection to encourage commercial plant breeders to invest resources, labour and time needed to improve existing plant varieties. IPRs in plant varieties thus provide some guarantee to breeders alike that they will be able to recoup the risks and costs of a value-added innovation that is based upon the underlying biological resource (Lesser, 1997).

However, granting of IPRs to natural scientists has in the past and potentially may in the future have certain consequences for overall scientific development. Referring back to the example outlined above, the granting of IPRs for plant breeders has had uncertain consequences for preserving genetic diversity. The incentives that IPRs create for private parties to invest in research and breeding techniques relating to new plant varieties is believed by some to benefit and increase plant genetic diversity over time. Conversely, a considerable proportion of the scientific community has argued that IPRs play more of a detrimental role upon biodiversity. It is postulated that the increase in genetic diversity created by *ex situ* seed collection and distribution, and *in situ* conservation by indigenous farming communities has ultimately diminished over that past few decades following the introduction of IPRs to the field of natural science, as farmers began to rely on seeds with uniform genetic characteristics from commercial third parties (Fowler, 1994).

Furthermore, commercialization has introduced market considerations into the manner in which scientific research is carried out (Chapman, 1999). Commercialization has also changed intellectual property from a means to provide incentives to natural science researchers and inventors to a means to entice investment and protect the overall resources of investors. It is also evident and freely commented upon by many in the natural science community that corporate investment in scientific research and development has forced unwilling restriction on sciences' tradition of open publication, particularly in Life Science publications. Some scientist are feeling the need to delay publications and withhold data from being uploaded on to scientific databases so as to secure intellectual property rights first (Marshall, 1997). Needless to say there is a prevalent concern worldwide in the scientific community that privatization, accompanied by legal restrictions and high prices, will restrict scientists' access to data needed for research (Committee on Issues in the Transborder Flow of Scientific Data, 1997).

A study by the Committee on Issues in the Transborder Flow of Scientific Data (1997) comments as follows on these trends:

"Science operates according to a "market" of its own, one that has rules and values different from those of commercial markets. While protection of intellectual property may concern a scientist who is writing a textbook, that same scientist, publishing a paper in a scientific journal, is motivated by the desire to propagate ideas, with the expectation of full and open access to results. To commercial publishers (including many professional societies), protection of intellectual property means protection of the right to reproduce and distribute printed material. To scientists, protection of intellectual property usually signifies assurance of proper attribution and credit for ideas and achievements. Generally, scientists are more concerned that their work be read and used rather than that it be protected against unauthorized copying. These conflicting viewpoints pose challenging problems for science and the rest of society."

The commercialization of scientific research and the protection of data through non-disclosure or the enforcement of IPR may help to encourage the flow of private investment into funding scientific discovery. However, it is far from certain that this investment, should it emerge due to greater application of IPR to scientific data, would result in the progress of science, particularly those areas that are not so well suited to generating financial gain. Scientific discoveries have always come about by building on the work of others and attempts to curtail the ability of scientists to use existing data may well impact on future scientific discoveries. The scientific community has long depended more on reputation, etiquette and peer-review to ensure the proper recognition of someone's work than on enforcement of any legal instruments and it is likely to be for this reason that there are so few (if any) examples of IPR cases in the field of natural science outside of biotechnology.

2.3 Identifying databases

It is crucial for the copyright and licensing process of online databases that the databases are correctly identified. Identification systems are applied in order to provide information on the database, rather than to protect it from unlawful use. Identification systems can be divided into two main categories (Koskinen-Olsson, 2008), namely identifiers and digital signatures.

There are several methods of identifying the origin of any data; for example, print media successfully use unique International Standard Book Numbers (ISBN) and International Standard Serial Numbers (ISSN) to track and find publications in bookshops and libraries. These two identifiers were designed for use in the physical or printed world. Many identifiers have now been invented for online use. One of the most widely used within the digital area is the "Digital Object Identifier" (DOI). The DOI can be attached to any scientific or literacy work, such as an article, chapter or illustration. It was originally

developed for publishing, but in principal it can be applied to any type of content and it is currently being considered for scientific databases (e.g. the Encyclopedia of Life). The DOI system *"…identifies objects with content in the digital environment. DOI names are assigned to any entity used on digital networks. They are used to provide current information, including where they can be found on the web. Information about a digital object may change over time, including where to find it, but its DOI name will not change"* (DOI, 2010). The DOI offers a system for persistent identification, managing content and metadata, and enabling the adequate citation and sharing of a database. However, it is costly to subscribe to and thus academic libraries tend to use the Handle system for archive material digitally (Costello, 2009).

Life Science Identifiers (LSIDs) is another relevant identification system, which is currently being used by biodiversity databases such as, ERMS, FaEu, WoRMS, Index Fungorum and uBio. LSIDs are *"persistent, location-independent, resource identifiers for uniquely naming biologically significant resources including species names, occurrences, genes or proteins, or data objects that encode information about them. LSIDs are a way to identify and locate pieces of biological information on the web*" (LSID, 2010). Every LSID consists of up to five parts, each separated by a colon.

The way biodiversity databases are electronically identified will be also reflected in the way databases will be cited. For example, an important aspect to consider is the taxonomic resolution at which biodiversity databases are electronically identified, i.e. DOI names assigned to individual species records, higher taxonomical level such as Order or to the entire database. The analogous situation in the print era would be to decide whether to individually cite pages, chapters, the book or the library where a book is deposited. For example, the World Register of Marine Species (WoRMS) (Appeltans et al., 2010) has a unique referencing system, so each record has a unique identification, called AphiaID. While they encourage users to cite the individual global or regional species lists (often the source of the data), they also give the option to cite the whole database. This is an important aspect that needs to be considered as currently many biodiversity database webpages provide unclear or no recommended citation.

Digital watermarks, signatures and fingerprints are other useful systems for identifying digital objects. The presence of digital signatures can be used to indicate the legitimacy of a digital expression. These identifiers are embedded in the digital material, and the author can track them over the web using specific software. They constitute an effective measure against unlawful use and are most commonly used on images (Koskinen-Olsson, 2008).

Another means of protecting against the unauthorised use of online content are the so-called technological protection measures (TPM). These measures make it impossible to access or use online contents without authorization. This can be done either by limiting the access to the source by, for example, using passwords, or by encrypting the material itself. Limiting access to an online database is a common practice and can be seen if you visit the webpage Mapping European Seabed Habitats (MESH) (JNCC, 2009) where the maps and data produced by the project are stored and you can only gain access if you are a legitimate user and have the password. Controlled access does not necessarily involve payment, but it merely identifies the potential user of the data. Delivery of copyright license material can also take place in a digital envelope or wrapper, in which case the recipient needs to have a key to open the file.

It is important to make sure that when the content of a database is delivered, it is accompanied by information about the relevant identifiers, licensing conditions and other information, i.e. the terms of use.

2.4 Licensing

While the benefit of making data publicly available is appreciated by governments, funding agencies and the scientific community collectively, the benefits to individual scientists are less evident (Costello, 2009). This is because individual scientists' concerns regarding making data openly available and introducing measures to motivate online publication have not been addressed (Klump *et al.*, 2006, Parr, 2006, Heidorn, 2008). The main obstacle in making more primary scientific data available is not policy or money, but misunderstandings and inertia within parts of the scientific community. Copyright issues have been identified as one of the main reason mentioned by scientists for not making data available (Costello, 2009).

Due to the absence of printed copies of scientific work or databases in the online world, licensing is a tool that can provide authors with the opportunity to share and disseminate their work in a legitimate way. The license would define the terms and conditions under which the data was granted to users and their obligations.

The EU Directive 96/9/EC on the legal protection of databases (EC, 1996) states that any original expression that is fixed in a tangible form is protected as soon as it is expressed. For example, a biodiversity database webpage is protected as soon as the author stops typing and saves the .html file. Given that the copyright protection by this Directive is quite restrictive and considering the benefits associated with open access resources in science there is a need for more flexible licensing options that would provide a compromise between the protection of the author and legitimate interest of users. In defence of the Directive, plagiarism and breaches of copyright are likely to happen as is witnessed with print media today. While scientific databases seem unlikely to generate sufficient revenue to enable taking legal action to enforce copyright and prosecute plagiarism, this action potentially exists.

2.5 Alternative licences

One significant alternative licensing option that has been recommended for scientific publications (Schlögl & Velden, 2004, Clarke, 2005) is the Creative Commons (CC) (Creative Commons, 2001) suite of licenses. The CC provides free legal tools that give any author, from individual creators to major institutions, a simple, standardized way to adapt copyrights to their work. CC licenses allow authors to easily change their copyright terms from the default of "all rights reserved" to "some rights reserved." However, a CC license is not an alternative to copyright, they are applied on top of copyright, so authors can modify their copyright terms to best suit their needs. CC ensures that their licenses work globally by collaborating with an international network of intellectual property experts. For example, if a scientist has created a database and wants other colleagues to share, use, and build upon it, he/she can consider publishing the database under a CC licence. Creative Commons licences are flexible tools that also protect users from copyright infringement, as long as they abide by the specified terms.

In order to obtain a CC license for a database or any scientific work, authors first need to choose the conditions within the following four major categories: *Attribution* (by), *Share Alike* (sa), *Non-commercial* (nc) and *No Derivative Works* (nd) (Creative Commons, 2001). By creating different combinations of these conditions, there are six main licenses offered to authors who choose to publish their work with a CC license. These licenses are listed below starting with the most permissive and ending with the most restrictive type (Creative Commons, 2001).

Attribution (cc by)

This license lets users distribute, modify and build upon the author's creation, even commercially, as long as they cite the author. This is the most accommodating of the licenses offered.

Attribution Share Alike (cc by-sa)
Users can modify and build upon creations even for commercial purposes, as long as authors are cited and license their new creations under identical terms. This license is often compared to open source licenses. All new works based on the original will be licensed in the same way, so any derivatives will also allow commercial use.

Attribution No Derivatives (cc by-nd)
This license allows for redistribution, commercial and non-commercial, as long as it is passed along unaltered and in whole, with credit to the author.

Attribution Non-Commercial (cc by-nc)
This license lets others modify and build upon an author's work non-commercially, and although their new works must also acknowledge the author and be non-commercial, they don't have to license their derivative works on the same terms. This license is currently being used for some important online biodiversity datasets, for example FishBase (Froese & Pauly, 2009).

Attribution Non-Commercial Share Alike (cc by-nc-sa)
Users can modify and build upon an author's creation only for non-commercial purposes, conditional to users citing the author and licensing their new creations under the identical terms.

Attribution Non-Commercial No Derivatives (cc by-nc-nd)
This is the most restrictive as it only allows redistribution. This license is often called the "free advertising" license because it allows users to download a given work and distribute it as long as they cite the author, but without changing the original work or using it commercially. This kind of license has been suggested as the most appropriated for open access scientific publications (Clarke, 2005).

It is important to bear in mind that behind biodiversity databases there is an enormous amount of time and effort contributed by the creative scientists, in which funding agencies and institutions have made huge investments. Biodiversity research in the digital era relies heavily on material protected by copyright. Licensing is one answer to the demands for access to biodiversity information and new and innovative ways to do science are constantly evolving.

3 PESI and IPR

The scientists that created the three main European taxonomic databases (ERMS, Fauna Europaea and Euro + Med PlantBase) are all members of the Society for the Management of Electronic Biodiversity Data (SMEBD), an independent legal organisation and a partner in the PESI project.

SMEBD effectively democratises ownership of the three databases to the community that created it under the Creative Common *Attribution-ShareAlike* license (see below). Individual contributors assign copyright to SMEBD, but retain collective control by electing a Council that governs the databases (cf. Appendix 2). This is implemented by each database having a committee derived from its contributing experts who handle its day-to-day management. Their remit involves the appointment of new data editors, who host the database, and the provision of copies of the database to users under certain approved conditions.

At this point it should be made clear that while potential providers of data to the PESI portal will be required to sign an agreement to follow SMEBD's IPR policy model in relation to their submitted data they will in fact retain ownership of their databases and are free to continue with the copyright, T&C etc that they have established when they are contacted for use of their data. The ownership of databases contributed outside the SMEBD model will remain with the original author or owner of the data within the database. The owners of such databases assign the right to the SMEBD committee to access, display and share the data through the PESI portal. PESI does not claim any intellectual property rights on the data that is made available through the database. Therefore the agreement is in relation only to data which will be extracted/available through the PESI portal. By contributing data they in turn become members of SMEBD . However, the authors of this document are aware that issues may arise in the future and thus, it may be necessary to provide data contributors with an alternative IPR policy under which the datasets are made available through the PESI portal. It is important to facilitate all potential data providers with sufficient certainty and transparency in relation to their rights to encourage a wide participation. Therefore, the authors present here the SMEBD IPR Model, while the outline of an alternative model that may be considered for data contributors who wish to contribute data outside of the SMEBD model in the future is presented in Appendix 3.

3.1 SMEBD IPR model

a. Data ownership statement

The content of the PESI databases is vested in the Society for the Management of Electronic Biodiversity Data Ltd (www.smebd.eu). All scientists contributing to the databases are eligible for SMEBD membership, and thus share collective responsibility for ensuring the data are quality controlled, maintained, and hosted by appropriate institutions. Upon completion of the PESI project, SMEBD will continue to develop the databases in collaboration with their host organisations. Decisions on the management of the databases, such as appointing and replacing experts to edit their content, and providing copies to third parties, are made by specific database committees under the authority of the SMEBD Council.

For new databases, contributors interested in becoming involved and providing information to PESI will have the option of allowing SMEBD to take over the management of the selection of data they provide and signing a SMEBD agreement form (Appendix 2).

The EU-Nomen portal will have a common approach to citation and Creative Commons licensing for all databases, including databases outside the SMBED committee. The copyright used will follow the *Attribution-ShareAlike* scheme (for more information, view http://creativecommons.org/licenses/by-sa/3.0/). Ideally, all data providers should abide by the same licence requirements to avoid conflicting policy interactions if combined datasets from different sources are downloaded through the PESI facilities.

b. Terms of use of data

By downloading or consulting data from this website, the visitor acknowledges that he/she agrees with the PESI data policy, and agrees to the following:

- If data is extracted from the PESI website for secondary analysis resulting in a publication, the PESI website should be cited following the citation scheme given below in Section 6 (Citation).

Users must acknowledge the contribution of the relevant Data Providers in any derived information product or publication, whether printed, electronic or broadcast, that is based wholly or in part on the material, data and/or information they make available to them. Where users make specific use of or reference to a particular biological dataset they must acknowledge the original data provider (where their name is made available) using the referencing format provided within the PESI database.

c. Termination of licence and rights of use

This License and the rights granted hereunder will terminate automatically upon any breach by the user of the terms of this License. Individuals or entities who have received Adaptations or Collections from the user under this License, however, will not have their licenses terminated provided such individuals or entities remain in full compliance with those licenses.

Subject to the above terms and conditions, the license granted here is perpetual (for the duration of the applicable copyright in the Work). Notwithstanding the above, PESI reserves the right to release the Work under different license terms or to stop distributing the Work at any time; provided, however that any such election will not serve to withdraw this License (or any other license that has been, or is required to be, granted under the terms of this License), and this License will continue in full force and effect unless terminated as stated above.

d. Disclaimer

General disclaimer

The PESI Data Administrators reserves their exclusive right in its sole discretion to alter, limit or discontinue the Site or any Materials in any respect. The PESI Data Administrators shall have no obligation to take the needs of any User into consideration in connection therewith. The PESI Data Administrators reserves the right to deny in their sole discretion any user access to this Site or any portion thereof without notice. No waiver by the Data Administrators of any provision of these Terms and Conditions shall be binding except as set forth in writing and signed by its duly authorized representative.

No Warranty

PESI and its data suppliers provide this database free of charge for the benefit of the public in an “as is” condition. Neither PESI nor its data suppliers warrants, guarantees, or makes any representation regarding the accuracy, completeness, correctness, reliability, currency or otherwise, of the databases or the use or results to be obtained from using the databases or the information contained therein, or any related documentation or written materials. Neither PESI nor its data suppliers makes any representations or warranties whatsoever, express or implied, with respect to the database, and, in particular, PESI and its data suppliers disclaim all implied warranties including without limitation any warranties of merchantability, non-interference, non-infringement, informational content, or fitness for a particular purpose with regard to the database.

Limitations on liability

Except to the extent required by applicable law, in no event will PESI or its data suppliers be liable to you on any legal theory for any special, incidental, consequential, punitive or exemplary damages arising out of this license or the use of the work, even if licensor has been advised of the possibility of such damages.

3.2 Identifying databases in PESI

As discussed in Section 2, the identification of databases, and the data held within them, is a crucial tool in dealing with issues of IPR. Which unique, persistent identifier would be appropriate for PESI was investigated under D5.5 of WP5. It is proposed that the unique identifier, which is non-actionable, will be generated by the checklist source (e.g AphiaID in ERMS and WoRMS) and that they will be converted into a Life Science Identifier (LSID), which is actionable, at the PESI portal level. LSIDs are also among the two persistent identifiers recommended for use by GBIF, the other being HTTP URIs (Cryer *et al*, 2009), which should lend weight to their selection by PESI should that be the formal decision taken.

4 Advantages and disadvantages of SMEBD IPR model

The SMEBD IPR models allow for an open access database in which there is no restriction for data users to obtain data. The model will promote data usage and encourage data creators to share relevant data with one another without the fear of them breaching specific IPR laws. Furthermore, it will aid the development and expansion of research amongst the scientific community.

By following the SMEBD model, a data contributor facilitates the more ready dissemination of data as decisions can be made by the SMEBD Council and committees in relation to data storage, modification and dissemination. The contributor retains control of the data via the election of the SMEBD Council and the committees created to manage each database and by the express right to use and publish the data.

The SMEBD model will encourage scientist to input their data whilst not being burdened with the upkeep of their data. It will allow scientist to retain full ownership of their work and have confidence in knowing that their data is being managed accordingly by data stewards. This will encourage scientists to input data, hence, aiding the development and expansion of research amongst the scientific community. PESI and the relevant data contributors to the database will bear no financial burden as the ‘Data Stewardship Program’ will be run voluntarily within participating taxonomic experts.

While the ready accessibility of data through the PESI portal is a surely an advantage to the scientific community as a whole and in-keeping with principles of making scientific publicly available, in some cases projects are funded by third party organisations who have invested a lot of capital to carry out scientific research. These organisations may not be comfortable allowing commercial use of data that they paid to obtain by third parties free of charge. As stated in the SMEBD standard agreement with a contributor (Appendix 2) “*SMEBD owns, authorises dissemination and revision of its databases…”*. Scientists may not feel comfortable handing over the management of their submitted data in which they have invested so much time and effort to a data management committee. Where this situation arises, the application of a more restrictive IPR model, similar to that presented in Appendix 3 may be appropriate. However, there is also the option for the data provider to become involved in the ‘Data Stewardship Programme’ and in this way play an active part in the management of the data within PESI.

5 Advantages and disadvantages of Creative Commons Attribution-ShareAlike

As it is proposed that the PESI portal will follow a Creative Commons Attribute-ShareAlike (cc by-sa) licence, it is important to consider some of the advantages and disadvantages that may be relevant to this choice.

The main advantage of the cc-by-sa model is that it allows for the wide dissemination of data to other scientists and interested parties, but still provides recognition of the work done by the creator of the data. The *ShareAlike* clause will ensure further dissemination of derivative works, thereby promoting the idea of Creative Commons and the sharing of data.

The use of a Creative Commons licence provides users with a simple to understand "human-readable" summary of the licence, which makes it clear what is and isn't permitted under the licence. The legal version is also accessible if more detail is required.

The differing conditions that may be applied through a CC licence (*Attribution, ShareAlike,* etc) can be easily incorporated where data with different CC conditions are combined. The CC icon used can show what conditions apply in a clear and concise manner.

One disadvantage of the Creative Commons model is that it may not be widely know among researchers and therefore may create uncertainty for people providing or using the data. However, this situation is likely to change over time as the system becomes more widespread; Creative Commons licences are already used by WoRMS, Fishbase, Tree of Life (www.tolweb.org) and the Biodiversity Heritage Library (www.biodiversitylibrary.org).

Another disadvantage of the cc-by-sa is the potential issue of 'attribution stacking'. The need to acknowledge the original source of the data, as well as the source that was actually utilised (for example a derivative work) may result in numerous sources being cited for the same data. This may not become an issue in practise, as users are likely to follow the current scientific etiquette in relating to citation and cite the original source.

6 Citation

The citation systems currently in use by ERMS, Fauna Europaea and Euro+Med Plantbase differ from each other and the system adopted by PESI should consider the advantages and disadvantages of the systems chosen by these three databases in formulating its own citation system. The following is a summary of the citation model followed the each of the three databases:

- The MarBEF data policy sets out how data downloaded from the site (including ERMS data) should be cited and the citation for individual records is generated at the bottom of the record page in ERMS.

- The system adopted by Fauna Europaea is explicit on how to cite the database as a whole or groups within the database. However, the method of citation varies depending on the taxonomic level in question and whether the Taxonomic Specialist is also the Group Coordinator, making it more complicated. The user must acquire the information on the record page and insert it into the citation template.

- Euro+Med Plantbase provides a template for citing the database in its entirety and also individual taxon pages. The template for individual taxon pages is populated by reference to the source provided at the top of the taxon page being referenced and the date accessed. This information is then inserted into the citation template.

The citation system proposed for PESI generally follows that used in citing works published in hard-copy and so should be straightforward for researchers to use.

- [Author/s][Year]. [Chapter] *In* [Book]. [Publisher]. [Page]

The template to be used for PESI will be as follows when verified by expert/contributed by European Species Database (ESD) or other database outside SMEBD:

- Editors (Year). Taxon + Authority. In: ESD title or other Database or data manager. Accessed through the PESI at [URL] on [date].

For example:

- Reid, David G., Gofas, S. (2010). *Littorina littorea* (Linnaeus, 1758). *In*: Bouchet, P.; Gofas, S.; Rosenberg, G. (2010) World Marine Mollusca database. Accessed through the World Register of Marine Species at http://www. eu-nomen.eu/pesi/aphia.php?p=taxdetails&id=140262 on 2010-07-28.

Where the record has not been verified the database is the author, in which case the following template is used.

- PESI (Year). Taxon + Authority. Accessed through PESI at [URL] on [date].

For example:

- PESI (2010). *Salmo salar* Linnaeus, 1758. Accessed through PESI at http:// eu-nomen.eu/pesi/aphia.php?p=taxdetails&id=127186 on 2010-07-28.

Databases will be cited over a range of taxonomical resolution, from individual records to the whole database using the unique referencing system as discussed in Section 3.2, where each record or higher level of database organisation has a unique identification. This unique, persistent identifier will form part of the URL as is the case with the AphiaID in ERMS and WoRMS.

The citation system on ERMS (and WoRMS) is facilitated by the generation of the citation at the bottom of the record page. This facility removes any confusion for the user in relation to the correct citation of records as it is merely a case of 'copy and pasting' the citation from the record page. It is recommended that this system be adopted by the PESI portal to ensure the correct attribution of credit is straightforward for users.

PESI should encourage the citation of the original author of the data in addition to the PESI portal as emphasised in D2.1 of WP2 (WP2, 2010).

7 Data submission protocol

In order to publish taxonomic checklist databases in the EU-Nomen portal it is necessary to adopt a standard and effective data submission protocol. This will allow for data provider contribution in a more simplistic and easy to use manner. It is desirable that species records are also accompanied by other information about the taxon itself. In this context, the use of the Darwin Core Archive (DwC-A) has been suggested as the preferred exchange format for taxonomical data (WP4, 2010). The DwC-A format

is a simple and efficient way to publish checklist data, and it is supported by other online taxonomical databases, such as GBIF ECAT project. Appendix 2 of the PESI WP4 report "Application and Adoption of Taxonomic Standards" provides a detailed applicability statement specifying how DwC-A should be used. Additionally, the documentation available from GBIF (http://code.google.com/p/gbif-ecat/wiki/publishingChecklists) should be sufficient for many data suppliers to adopt this data format.

To publish checklist data into the PESI portal data providers will need to:

- Identify the specific data elements in their dataset that they would like to publish.
- Determine whether the DwC-A data format suits the specific dataset they are willing to publish. There are some good examples provided at the GBIF ECAT project webpage. (http://code.google.com/p/gbif-ecat/wiki/publishingClassifications) that may be helpful to review.
- Follow the best practices for publishing checklists provided by GBIF ECAT project (http://code.google.com/p/gbif-ecat/wiki/BestPractices).
- Export the data into the DwC-A format using the Integrated Publishing ToolKit (http://code.google.com/p/gbif-providertoolkit/).
- Clearly label the data with the PESI portal license (Attribution -Share alike) or any other terms of use constraints attached, author and publication date.
- Submit the data to the PESI portal.

8 Software & tools developed within the PESI framework

Similar to electronic databases licensing, software and tools development IPR licenses fall into two main categories:

a) Conventional copyright licenses, where all right are reserved by the author of the software/tools;

or

b) Open sources licences which are more flexible allowing users open access to and distribution of the softwares.

There are licences specifically designed for use with software that allow open access. Some examples of these free software licence are the GNU General Public License and the GNU Lesser General Public License and the BSD licences (http://creativecommons.org/choose/cc-lgpl) or the Mozilla Public License Version 1.1 (http://www.mozilla.org/MPL/MPL-1.1.html).

The free software licences grant user permission to: run the software for any purpose, study how the software works and adjust it to the user's needs, redistribute copies, to build upon the software and publicly release the improvements.

There are several software/tools developments that have been developed with the PESI portal and the licences that apply to them fall in the two main categories of software IPR as shown in Table 1.

Table 1. List of software/tools developed with the PESI portal.

Software/tool	Description	Licence type
**PESI website**	Joomla!	Open source
**PESI species portal**	Php code	Copyright/IPR VLIZ

**Mapping software**	OpenLayers and GeoServer	Open source
**PESI focal point database structure**	Php code	Copyright/IPR VLIZ
**Webservices**	SOAP	Open source
**Webservices: taxon match tool**		Open source
**PESI data warehouse structure**		Joint copyright between VLIZ and BGBM
**EDIT Software**	Drupal	Open source

It is clear from Table 1 that the use of Open Source software has been of great importance in the development of the PESI portal and the provision of biodiversity database webservices. The use of open source licences should be considered for any future software created for the development of the PESI portal.

9 Conclusion

The issue of IPR in relation to biodiversity data and databases is not one that can be resolved in a straight-forward manner. The legal issues relating to IPR in so many fields can be seen frequently in the media with organisations suing and countersuing in an attempt to protect their actual or perceived IPR. This report has attempted to set out a working model under which PESI can proceed with its aim to integrate taxonomic databases in Europe with an IPR model that will ensure the free and wide dissemination of data, promoting the furthering of science in this field. "*The value of data is in its use"* (Committee on Issues in the Transborder Flow of Scientific Data, 1997), or as Isaac Newton once wrote "*If I have seen a little further it is by standing on the shoulders of Giants"* (Though this is more correctly attributed to Bernard of Chartres in the 12th century (Wikipedia, 2010).

10 References

94th Congress (1976). An Act for the general revision of the Copyright Law, title 17 of the United States Code, and for other purposes.

Appeltans, W., Bouchet, P., Boxshall, G. A., Fauchald, K., Gordon, D. P., Hoeksema, B. W., Poore, G. C. B., van Soest, R. W. M., Stöhr, S., Walter, T. C. & M. J. Costello (eds) (2010). World Register of Marine Species. Accessed at http://www.marinespecies.org on [2010-06-22].

CBD (1992). Convention on Biological Diversity, adopted June 5, 1992, A/CONF. 151/26, 31 ILM 818 (1992).

Clarke, R. (2005). A proposal for an open content licence for research paper (Pr)ePrints.. http://www.rogerclarke.com/EC/PrePrLic.html

Chapman, A. R. (1999). A human rights perspective on intellectual property, scientific progress, and access to the benefits of science. *In*: Intellectual Property and Human Rights (Geneva: World Intellectual Property Organization), pp.127-68.

Committee on Issues in the Transborder Flow of Scientific Data, National Research Council (1997). *Bits of Power: Issues in Global Access to Scientific Data*. National Academic Press, Washington D.C. 250 pp.

Costello, M. J., Bouchet, P., Boxshall, G., Arvantidis, C. & Appeltans, W. (2008). European Register of Marine Species. . http://www.marbef.org/data/erms.php . Consulted on 2009-11-19

Costello, M. J. (2009). Motivating Online Publication of Data. *BioScience* **59**: 418–427.

Creative Commons (2001). "Some Rights Reserved": Building a Layer of Reasonable Copyright. http://creativecommons.org

Cryer, P., Hyam, R., Miller, C., Nicolson, N., Ó Tuama, É., Page, R., Rees, J., Riccardi, G., Richards, K., White, R. (2009). Adoption of Persistent Identifiers for Biodiversity Informatics: Recommendations of the GBIF LSID GUID Task Group 6 November 2009.

DOI (2010). The Digital Object Identifier. Accessed at www.doi.org on16/08/10

EC (1996). Directive 96/9/EC of the European Parliament and of the Council of 11 March 1996 on the legal protection of databases. Oficial Journal of the European community.

Fowler, C. (1994).*Unnatural selection: Technology, Politics, and Plant Evolution*. International Studies in Global Change Vol. 6. Gordon and Breach. New York. 317 pp.

Froese, R. & Pauly, D. (2009). FishBase.www.fishbase.org. version (08/2009).

Heidorn, P. B. (2008). Shedding light on the dark data in the long tail of science. In: Shreeves S. and Cragin M. (Eds). Institutional repositories: practice, current research, data collection, future. *Library Trends* **57**: in press. http://hdl.handle.net/2142/9127

Helfer, L.R. (2002). Intellectual Property Rights In plant varieties: An Overview with options for National Government. FAO Legal Paper Online 31. Food and Agriculture Organisation, Rome. 62 pp.

JNCC (2009). Mapping European Seabed Habitats (MESH). www.searchmesh.net

Klump, J., Bertelmann, R., Brase, J., Diepenbroek, M., Grobe, H., Hock, H., Lautenschlager, M., Schindler, U., Sens I. & Wachter, J. (2006). Data publication in the open access initiative. *Data Science Journal* **5**: 79-83.

Kothari, A. & Anuradha, R V. (1999). Biodiversity and Intellectual Property Rights: Can the Two Co-Exist. *Journal of International Wildlife Law and Policy* **2**: 204-223.

Koskinen-Olsson, T. (2008). Access to knowledge in the digital era. *Learned Publishing* **21**: 93-102.

Lesser, W. H. (1997). The Role of Intellectual Property Rights in Biotechnology

Transfer under the Convention on Biological Diversity. IASAAA Briefs No. 3-1997. International Service for the Acquisition of Agri-biotech Applications. Ithaca, NY. 22 pp.

LSID (2010). Life Science Identifiers. Accessed at www.lsids.sourceforge.net on 16/08/10.

Marshall, E. (1997). Secretiveness Found Widespread in Life Sciences. *Science* 276: 525.

Parr, C. S. (2006). Open sourcing ecological data. *BioScience* **57**: 309-310.

Schlögl, R. & Velden, T. (2004). Berlin 2 Open Access - Roadmap Proposal. Berlin 2 Open Access: Steps Toward Implementation of the Berlin Declaration on Open Access to Knowledge in the Sciences and Humanities, CERN, Geneva.

SMEBD (2009). World Register of Marine Species. Accessed at http://www.marinespecies.org on [2009-09-25].

TRIPs (1994). Trade Related Aspects of Intellectual Property Rights, Marrakesh Agreement Establishing the World Trade Organisation, Annex IC, 33 I.L.M.81 (1994)

Wikipedia (2010). Standing on the Shoulders of Giants. Accessed at http://en.wikipedia.org/wiki/Standing_on_the_shoulders_of_giants on 01/09/10.

WIPO (1996). The WIPO Copyright Treaty (WCT) and the WIPO performances and phonograms treaty (WPPT). 25 pp.

WP2 (2010). The European Taxonomic Work force (ETW), its tasks, activities and operational standards inspiration by the Open Source Society. A Pan-European Species-directories Infrastructure (PESI). Report of Work Package 2 on Deliverable 2.1: Version 3.1. 50 pp.

WP4 (2010). Application and Adoption of Taxonomic Standards. A Pan-European Species-directories Infrastructure (PESI). Third Report of Work Package 4. 15 pp.

Appendix 1: Glossary of terms with respect to IPR in databases

AphiaID: AphiaIDs are unique identifying numbers generated by the unique referencing system used by WoRMS and ERMS to identify individual records. Existing records may be updated, but never overwritten with a complete new record.

Authorship: the person who creates or gives existence to a database, work or information. Authorship cannot be sold or transferred, although future editions may develop from earlier versions with different authors.

CC: Creative Commons a licensing setup which allows creators to release works on the Word Wide Web under specific terms governing their attribution, commercialization, and derivation.

Commons: refers to online resources that are collectively owned. This can include everything from images to software. The process by which the commons are transformed into private property is often termed enclosure.

Copyright: gives the author of an original work exclusive right for a certain time period in relation to that work, including its publication, distribution and adaptation, after which time the work is said to enter the public domain.

Custodian: refers to the person and/or organisation that is in possession of, and responsible for, a database.

DOI: Digital Object Identifier. DOI is a system for identifying content objects in the digital environment. DOI® names are assigned to any entity for use on digital networks. They are used to provide current information, including where they (or information about them) can be found on the Internet. Information about a digital object may change over time, including where to find it, but its DOI name will not change. http://www.doi.org/

ERMS: European Register of Marine Species. ERMS is an authoritative taxonomic list of species occurring in the European marine environment.
http://www.marbef.org/data/erms.php

Euro+Med Plantbase: Euro+Med Plantbase is a database and information system for the vascular plants of Europe and the Mediterranean region.
http://www.emplantbase.org

Fauna Europaea: Fauna Europaea is a database of the scientific names and distribution of all living multicellular European land and fresh-water animals.
http://www.faunaeur.org/

HTTP: Hypertext Transfer Protocol is an application layer protocol for distributed, collaborative, hypermedia information systems. It is the key technology that turns the Internet into the World Wide Web.

HTTP URI: An HTTP URI is a web address or name starting with 'http://' See also HTTP and URI.

IPR: **Intellectual property rights.** IPR usually refers to four major categories of intellectual property: patents, copyrights, trademarks, and trade secrets and any other similar rights whether registered or unregistered existing anywhere in the world.

LSID: Life Science Identifier: LSIDs are persistent, location-independent, resource identifiers for uniquely naming biologically significant resources including species names, concepts, occurrences, genes or proteins, or data objects that encode information about them. To put it simply, LSIDs are a way to identify and locate pieces of biological information on the web. http://lsids.sourceforge.net/

Ownership: the person or organisation that is the legal owner of a database. Ownership may be transferred or sold. They may or may not be the custodian or host of the database.

Public domain: is a range of abstract materials—commonly referred to as intellectual property—which are not owned or controlled by anyone. The term indicates that these materials are therefore "public property", and available for anyone to use for any purpose. However, the principle of attribution of source still applies, as is the custom in professional practice.

SMEBD: The Society for the Management of Electronic Biodiversity Data. SMEBD is a society aiming to make biodiversity data available for the benefit of science and environmental management. http://www.smebd.eu/

URI: Uniform Resource Identifier. A string of characters used to identify a name or a resource on the Internet.

WoRMS: World Register of Marine Species. WoRMS aims to provide an authoritative and comprehensive list of names of marine organisms, including information on synonymy. http://www.marinespecies.org

Appendix 2: SMEBD - standard agreement with a contributor

AGREEMENT WITH:

This document establishes the basis on which data and/or intellectual property is provided to databases for which the Society for the Management of Electronic Biodiversity Data (SMEBD) takes responsibility. Such databases may be defined geographically, taxonomically, or by other themes. By contributing to such databases you will be benefiting the scientific community in general by assisting the production of good quality information of use to scientists, regulators, students and society. All contributors to the databases are life-members of SMEBD (www.smebd.eu)*. SMEBD owns, and authorises dissemination and revisions of its databases, and nominates the database host institutions. It is legally incorporated in Ireland as a limited company with no shareholders. Should SMEBD be wound up members' financial liability is limited to €2. Signatories to these Agreements with Contributors become members of SMEBD and thus have a say in the management of the databases by electing its Council. The Council establishes committees for the management of the databases.

The contributor hereby

1. agrees to voluntarily provide data, information, opinion, or other expert assistance to the database,
2. retains the right to use and publish any data and intellectual property created by the contributor,
3. authorises the project to store, compile, modify, and disseminate data provided and derived by any means (e.g. electronic, World Wide Web, book),
4. recognises that products of the database are the copyright of SMEBD.

SMEBD hereby agrees to

1. make the database publicly accessible online,
2. acknowledge the contribution of the contributor in such publication and derivatives of it,
3. provide the contributor with a copy of such publications,
4. record that the contributor is a member of the SMEBD and as such involve them in the affairs of the SMEBD, including the right to elect individuals to the SMEBD governing Council.

The agreement shall remain in force until either party notifies the other in writing that it wishes to discontinue it. Such notification would not be retrospective. This agreement will come into force when the contributor has provided data or other documented expertise or assistance to the database.

Authorised signature on behalf of SMEBD	Authorised signature on behalf of the Contributor named above
---	---

* Formerly known as the Society for the Management of European Biodiversity Data

Appendix 3: Potential alternative IPR model

For data contributors that have issues with the current SMEBD agreement the authors provide an alternative IPR model which may be considered in the future.

One of the major constraints for scientist managing their own data input and upkeep would be the scientist spending more time on quality control of data on the database rather than their own scientific work. What is not wanted is other scientist taking ownership/management, without accepting any real responsibility for the data. However, most would agree that those who have created and own the original data have a greater deal of care for that data than those people who don't own it. It is less likely that the quality of data control would be high when it is entrusted in the management of those who have no stake in the data. Therefore, the second model presented is a model in which should ensure that the quality of data is upheld, achieved through a proposed external stewardship of data whereby there is an assignment of some degree of ownership to those entrusted with the data quality, without permitting full ownership/managment of the data in question. This model should allow for both effective quality control and management, whilst still allowing data creators to maintain full/majority ownership/management of the data.

As in the SMEBD model the data available through the eu-nomen portal will have a common approach to citation and creative commons licensing. The copyright used will follow the *Attribution-ShareAlike* scheme (view http://creativecommons.org/licenses/by-sa/3.0/ for more information). Ideally, all data providers should abide the same licence requirements to avoid conflicting policy interactions if combined datasets from different sources are downloaded through the PESI facilities.

Under the PESI model data providers may wish to add additional terms of use for the data provided that will apply on top of the SMEBD model terms of use, for example a commercial use constraint. These constraints will broaden the spectrum of scientist wishing to input their data as they will not feel like they are offending previous commercial contracts with whom they obtained the data for.

If a commercial constraint is attached it may operate under an *Attribution-NonCommercial-ShareAlike* Creative Commons license. This license prevents database users from obtaining data for commercial use without obtaining prior consent from the Database Operator, Data Steward and/or the Data Creator.

Data providers not members of the SMEBD include:

- Other biodiversity databases
- Universities
- Museums
- Botanical Gardens
- Other research institutions
- Environmental consultancies
- Career and non-career taxonomists
- NGOs
- Government bodies

a. Data ownership statement

As in the SMEBD IPR model the ownership of databases contributed outside the SMEBD model will remain with the original author or owner of the data within the database. The owners of such databases assign the right to the SMEBD committee to access, display and share the data through the PESI portal.

PESI does not claim any intellectual property rights on the data that is made available through the database.

b. Terms of use of data

By downloading or consulting data from this website, the visitor acknowledges that he/she agrees with the PESI data policy, and agrees to the following:

- If data is extracted from the PESI website for secondary analysis resulting in a publication, the PESI website should be cited following the citation scheme given below in section 4 (Citation).

- If any individual data source of PESI constitutes a significant proportion of the records used in the secondary analysis (e.g., more than 10% of the data are derived from this source), the individual data source should also be cited.

- If any individual data source of PESI constitutes a substantial proportion of the records used in the secondary analysis (i.e. more than 25% of the data are derived from this source, or the data are essential to arrive at the conclusion of the analysis), the manager/custodian of this dataset should be contacted.

Under *Attribution-NonCommercial-ShareAlike* Creative Commons license a user downloading or consulting data from the website acknowledges that he/she agrees to the following:

- Under these *Terms & Conditions* users are permitted to view and download any *material, data and/or information* that *Data Providers* allow you to access on this website for your own private use or for the use of educational purposes.

- Under no circumstances may the material, data and/or information held on the PESI on behalf of *Data Providers* be copied, reproduced, republished, uploaded, posted, transmitted, or distributed for commercial use without prior written consent from the Data Provider and/or the Database Author.

- Users may not make any financial profit from use of the material, data and/or information on this website or from any products you derive without first obtaining written permission from the relevant data provider.

- Users may not republish wholesale the material, data and/or information made available to you, or exploit it for commercial or academic research purposes without first obtaining written permission from the relevant data provider (the contact details of which are provided within the metadata of the relevant dataset).

If combined datasets from different sources are downloaded through the PESI facilities have different licence requirements (e.g. under the SMEBD model and the PESI model) the following will apply:

- The *material, data and/or information* you access may be subject to *Specific Terms of Use* imposed by the Data Provider. Where *Specific Terms of Use* have been identified for a dataset they may be provided within 'A Use Constraint' located alongside the provided metadata for that dataset. If there is any conflict or inconsistency between these *Terms & Conditions* and the *Specific Terms Use*, the latter will prevail, but only in relation to the particular *material, data*

and/or information restricted by those *Specific Terms of Use*, and only to the extent necessary to resolve that conflict or inconsistency.

Other SMEBD terms of use, such as citation requirements, termination and disclaimer (see Section 3.1. c and d) apply to the PESI terms of use model.

**Configuration History**			
**Version No.**	**Date**	**Changes made**	**Author**
1.0	16 December 2009	First draft for circulation.	MC & EcoServ staff
1.1	24 December 2009	Revision of first draft	LB
1.2	28 December 2009	Report first version	YdJ
2.0	22 Februari 2010	Improved draft send to SMEBD council	HAP
2.1	19 April 2010	Minor corrections	RN
2.2	9 August 2010	Major revision	JA & EcoServ staff
2.3	6 September 2010	Minor corrections	JB
2.4	8 September 2010	Minor corrections	WA
2.5	17 September 2010	Minor corrections	YdJ
