# Supplementary material for: PESI - a taxonomic backbone for Europe
Source: Biodivers Data J. 2015 Sep 28;(3):e5848. doi: 10.3897/BDJ.3.e5848 (PMC4609752; doi:10.3897/BDJ.3.e5848)
Supplement: Supplementary material 5 — PESI workshop on linking taxonomic databases with online science journals [file biodiversity_data_journal-3-e5848-s005.pdf]

Report on the PESI workshop on linking taxonomic databases with online science journals

This PESI workshop vision is to plan how to use automated tools to improve quality control and interoperability between online science resources, including taxonomic databases and journals, so as to make authoritative data and information more easily accessible to the scientific community.

Participants introduced themselves. The only change to the agenda (Appendix 1) was that the presentation by Dave Remsen (by video conference call from USA) was moved to the afternoon.

The PESI project, workshops goals, opportunities for mutual benefits to the taxonomic databases and publishers, and structure and services of the PESI databases were outlined by Dr Yde de Jong, Dr Mark Costello, Ward Appeltans and Bart Vanhorne (Appendix 2).

Dr Vishwas Chavan described the new Integrated Publishing Toolkit recently launched by GBIF, and the proposed Data Usage Index to track data use on GBIF, so that data providers will have a metric of how much their data is being used (analogous to a paper citation index).

Catriona MacCallum described the publication strategy and methods of the Public Library of Science (PLoS). PLoS wished to know the best current advice for where to publish species distribution data. Those present agreed that GBIF was the leading global facility, and that authors could publish data through any one of the many current GBIF data providers. Online data appendices should follow the GBIF data schema where appropriate.

It was agreed to adapt existing tools, rather than develop new tools or resources de novo; and to work in a step-wise fashion to judge the success of initial measures, and facilitate adoption of new opportunities as they arose.

Desirable outcomes of the activities would be to

- Enrich the content on species pages in the taxonomic databases (e.g. live links to recent journal publications);
- Increase the visibility of published papers by exposing them on species pages;
- Easier and more direct access to published papers for database taxonomic editors;
- Improved quality control services for authors and publishers of journal papers through the taxonomic databases;
- Adoption of standards for online data appendices.

In addition to this report and appendices, additional information is provided on previous meetings of relevance, namely a meeting with the Editor of ZooBank journal (Appendix 3) and the EDIT workshop with taxonomic publishers in Europe, primarily museums (Appendix 4).

Appendix 1
Agenda, Background Notes, Publishers contacted, and briefing notes from participants.

Appendix 2
Participants' presentations as individual pdf files.

Appendix 3
Report on previous meeting with Zhi-Qiang Zhang, Editor of ZooBank (which publishes more animal taxonomy papers per year than any other journal).

Appendix 4
Notes from the EDIT workshop on scientific publishing by natural history institutions.

AGENDA

Wednesday 15th July 2009

Location
University of Amsterdam, Roeterstraat 11, Building E, Room 331.E E1.17.

GoogleMaps: http://maps.google.nl/maps?f=q&source=s_q&hl=nl&q=Roetersstraat+11,+Amsterdam+1018+Amsterdam,+Noord-Holland&sll=52.516221,5.515137&sspn=8.924815,23.939209&ie=UTF8&cd=1&geocode=FecBHwMd6O5KAA&split=0&z=17

Confirmed attendance
Yde de Jong (PESI coordinator), Mark Costello (PESI and SMEBD), Ward Appeltans (WoRMS Data Manager), Bart Vanhoorne (WoRMS Data Programmer), Vishwas Chavan and David Remsen (GBIF), Catriona MacCallum (PLoS).

Apologies for not being able to attend: Markus Döring (GBIF), Cathy Kennedy (Oxford), Joep Verheggen (Elsevier), Daniela Bone (Allen Press). John Austin & Matthias Seaman (Inter-Research), Marian Boletta (Thomson), John Sulzycki (CRC Press, Taylor and Francis Group), Laurence Bénichou (EDIT).

10:00 Refreshments and introductions
Opening remarks (Yde de Jong, PESI coordinator)

The biodiversity databases

10:20 Potential synergies between science publishers and taxonomic databases (Mark Costello)
10:30 PESI taxonomic databases: the World Register of Marine Species example (Ward Appeltans, Bart Vanhoorne)
11:20 GBIF Integrated (data) Publishing Toolkit and Zookeys case study (Vishwas Chavan GBIF)
11:40 GBIF plans for taxonomic data management; experience in using RSS feeds (Dave Remsen GBIF by video-call)

12:00 Lunch

Online functionality of the science publishers

13:00 PLoS online publications (Catriona MacCallum)
Others?

14:30 Break

15:00 Open discussion on way forward

17:30 End of meeting

19:30 Complimentary evening meal (Mark, Ward, Bart, Yde, ... others very welcome)

PESI publishers workshop proposal v.2

Linking science publishing with expert-edited scientific databases

Background

PESI is the Pan European Species directories Infrastructure, a project funded by the European Commission FP7 to support scientific infrastructures (www.eu-nomen.eu). It is bringing together, updating and improving databases on marine, land and freshwater animal and plant species hosted in Europe. Some databases are regional and some global. All are directly supported by hundreds of leading scientific experts who validate their content, and are open-access and online.

PESI wishes to develop automated interactions between its expert-edited databases and scientific publishers so as to improve services for the wider scientific community. The PESI database and publishers share the same community of editors, referees and authors; and reply on their cooperation for their content and quality control (peer-review, etc.). The aim of this discussion paper is to see how we can improve our mutual services for the scientific community through our collaboration, and provide mutual benefits to our own organisations. At first, we envisage using existing technologies (e.g. RSS feeds) so as the work involved is not time consuming. The ideas below are proposed to be a starting point, and new ideas are welcomed. We plan to hold a workshop between PESI project leaders and database managers, and publishers representatives, in Ostend in July 2009 to discuss the plans in more detail. Initiatives may be common to several publishers or bilateral.

Possibilities include:

(a) RSS feeds to PESI editors that alert them to a paper published that mentions their taxon of interest. We can provide the publisher with the full taxon list and contact for respective editors. [The publisher may wish to use this database for other purposes as well.] Then the editor may have privileged access to the paper regardless of their institutional licence (a benefit to the editor) and with a click of a button they can add the paper link to the species page that they manage (a benefit to the publisher).

(b) Provide standardized web-based and/or offline template for authors to create data appendices that they can then publish their species distribution data through the Global Biodiversity Information Facility (www.gbif.org) and similar biodiversity initiatives (e.g. Ocean Biogeographic Information System). This could be an appendix (excel sheet or similar) to a journal paper, and include the paper citation as the source (thus promoting the authors and journal). In this way the authors work is more widely disseminated (publicised) and can be automatically integrated with larger datasets which enables other scientists to reproduce the study findings, and repeat the study with new data. It is widely accepted that authors should make data available, and this is normal practice in some fields (e.g. genetics data goes to GenBank), but does not happen routinely for species distribution data.

(c) Provide online quality control tools that authors can get automatic feedback on the correctness of their species names (including synonyms and classification), and perhaps existing distribution by matching them against one or more PESI databases. It should be good practice that the methods state that they have checked species names and perhaps distribution information, against such authoritative online database. They may then analyse their data with this additional data, and know if their data repeats other findings or is novel in any way.

(d) Other ideas......................

Publisher contacts

1.	PLoS	Peter Binfield<br><br>Catriona MacCallum	pbinfield@plos.org<br><br>cjmaccallum@plos.org	Managing Editor, PLoS ONE<br><br>Senior editor PLoS Biology, former editor TREE
2.	InterResearch	John Austin, Mathias Seaman	john@int-res.com, matthias@int-res.com	Production and Associate Editor-in-chief
3.	Allen Press	Daniela Boone	dbone@allenpress.com	Publishing coordinator
4.	CRC Press (Taylor & Francis)	John Sulzycki	John.sulzycki@taylorandfrancis.com	Senior Editor
5.	Oxford University Press	Dr Cathy Kennedy	Cathy.kennedy@oxfordjournals.org	Senior Editor
6.	Scopus, Science Direct, Elsevier	Joep Verheggen	J.Verheggen@elsevier.com	Director of Product Management
7.	Web of Science, ISI, Thomson Reuters	Mariana Boletta	mariana.boletta@thomsonreuters.com	Senior Editor, Science
8.	OvidSP (Biological Abstracts)	Gino Erispé	G.Erispe@wolterskluwer.com	Regional Sales Manager – ANZ Wolters Kluwer Ovid Technologies
9.	Wiley	Dr Karen Chambers	kchambers@wiley.com	Editor
10.	ProQuest (part of Cambridge)	Dr Craig Emerson	Craig.Emerson@proquest.com	VP Editorial Operations
11.	JSTOR	Sherry Aschenbrenner or John Burns		Directors of User Services or Advanced Technology Planning

Notes

From Dave Remsem 16 June 2009

The current uBioRSS application does the following:

1. Every 30 minutes it checks a (still growing) list of RSS feeds and based on specific publication schedules it checks to see if any new feed data is available
2. When new feed data is published it is run through a taxonomic name find process that identifies the taxon names within the feed. This information and the source feed data are then stored.
3. The index of names tied to the feeds is then cross-referenced to accessible taxonomic lists and species list profiles.
4. The feeds can be browsed via some of these taxonomies. For instance you can navigate through them using the Catalog of Life or the NCBI taxonomy or the IUCN redlist.
5. Users can create profiles and submit their own lists of feeds or lists of taxa to track.
6. Any node in a taxonomy is a RSS feed so you could, for example, track any publications related to redlisted mammals for instance.

This is the state of the current application that we made in 2005. We are now at GBIF working with uBio to extend the capacity of this sort of application with a faster and more refined indexing model. We would like to make it easier to add additional taxonomies for example to be able to browse via WoRMS. We are refining the index of names. First we had the Nomina IV meeting to consolidate name recognition, discovery and reconciliation to make the tools better. Second, we will refine the indexing process that will allow us to, for instance, distinguish names found in the title, abstract and headers, from names found in the body of the document. We are expanding the data types we can index to include any text-bearing data object including PDF, DOC, PPT, etc.

Lots more but all of this becomes really exciting when we partner with guys like you to add the authority files and to take these data feeds and integrate them with other data in your portals.

More details to come.

Appendix 3

A list of taxonomic journals

ACTA BOTANICA GALLICA
ACTA PARASITOLOGICA
ACTA PHYTOTAXONOMICA SINICA
ACTA PROTOZOOLOGICA
ACTA ZOOLOGICA ACADEMIAE SCIENTIARUM HUNGARICAE
ADANSONIA
AMEGHINIANA
AMERICAN JOURNAL OF BOTANY
AMERICAN MUSEUM NOVITATES
ANNALES BOTANICI FENNICI
ANNALES DE LA SOCIETE ENTOMOLOGIQUE DE FRANCE
ANNALES ZOOLOGICI
ANNALS OF CARNEGIE MUSEUM
ANNALS OF THE ENTOMOLOGICAL SOCIETY OF AMERICA
ANNALS OF THE MISSOURI BOTANICAL GARDEN
ANTONIE VAN LEEUWENHOEK INTERNATIONAL JOURNAL OF GENERAL AND MOLECULAR MICROBIOLOGY
APPLIED AND ENVIRONMENTAL MICROBIOLOGY
AQUATIC INSECTS
AUSTRALIAN JOURNAL OF ENTOMOLOGY
AUSTRALIAN SYSTEMATIC BOTANY
BELGIAN JOURNAL OF BOTANY
BIOLOGIA
BIOLOGICAL JOURNAL OF THE LINNEAN SOCIETY
BLUMEA
BOTANICAL JOURNAL OF THE LINNEAN SOCIETY
BOTANICAL REVIEW
BOTHALIA
BRITTONIA
BRYOLOGIST
BULLETIN OF MARINE SCIENCE
BULLETIN OF THE AMERICAN MUSEUM OF NATURAL HISTORY
CAHIERS DE BIOLOGIE MARINE
CANADIAN ENTOMOLOGIST
CANDOLLEA
COLEOPTERISTS BULLETIN
COMPARATIVE PARASITOLOGY
COPEIA
CRETACEOUS RESEARCH
CRUSTACEANA
CYBIUM
DEUTSCHE ENTOMOLOGISCHE ZEITSCHRIFT
ENTOMOLOGICA FENNICA
ENTOMOLOGICAL NEWS
EUROPEAN JOURNAL OF ENTOMOLOGY
EUROPEAN JOURNAL OF PHYCOLOGY
EUROPEAN JOURNAL OF PROTISTOLOGY
FLORIDA ENTOMOLOGIST
FOLIA PARASITOLOGICA
FUNGAL DIVERSITY
GEOBIOS
HERPETOLOGICA
HYDROBIOLOGIA
ICHTHYOLOGICAL EXPLORATION OF FRESHWATERS
ICHTHYOLOGICAL RESEARCH
INSECT SYSTEMATICS & EVOLUTION
INTERNATIONAL JOURNAL OF ACAROLOGY
INTERNATIONAL JOURNAL OF SYSTEMATIC AND EVOLUTIONARY MICROBIOLOGY
INVERTEBRATE SYSTEMATICS
ISRAEL JOURNAL OF PLANT SCIENCES
JOURNAL OF ARACHNOLOGY
JOURNAL OF CLINICAL MICROBIOLOGY
JOURNAL OF CRUSTACEAN BIOLOGY
JOURNAL OF EUKARYOTIC MICROBIOLOGY
JOURNAL OF HERPETOLOGY
JOURNAL OF NATURAL HISTORY
JOURNAL OF PALEONTOLOGY
JOURNAL OF PARASITOLOGY
JOURNAL OF PHYCOLOGY
JOURNAL OF THE KANSAS ENTOMOLOGICAL SOCIETY
JOURNAL OF THE MARINE BIOLOGICAL ASSOCIATION OF THE UNITED KINGDOM
JOURNAL OF THE NEW YORK ENTOMOLOGICAL SOCIETY
JOURNAL OF VERTEBRATE PALEONTOLOGY
JOURNAL OF ZOOLOGICAL SYSTEMATICS AND EVOLUTIONARY RESEARCH
LICHENOLOGIST
MEMORIAS DO INSTITUTO OSWALDO CRUZ
MICROPALEONTOLOGY
MYCOLOGIA
MYCOLOGICAL RESEARCH
MYCOTAXON
NAUTILUS
NEMATOLOGY
NEW ZEALAND JOURNAL OF BOTANY
NORDIC JOURNAL OF BOTANY
NOVA HEDWIGIA
NOVON
ORIENTAL INSECTS
PALAEONTOLOGY
PALEONTOLOGICAL JOURNAL

PAN-PACIFIC ENTOMOLOGIST
PARASITE-JOURNAL DE LA SOCIETE FRANCAISE DE PARASITOLOGIE
PHYCOLOGIA
PLANT SYSTEMATICS AND EVOLUTION
PROCEEDINGS OF THE BIOLOGICAL SOCIETY OF WASHINGTON
PROCEEDINGS OF THE ENTOMOLOGICAL SOCIETY OF WASHINGTON
PROTIST
RAFFLES BULLETIN OF ZOOLOGY
REVIEW OF PALAEOBOTANY AND PALYNOLOGY
REVISTA BRASILEIRA DE ENTOMOLOGIA
REVISTA BRASILEIRA DE ZOOLOGIA
REVISTA DE BIOLOGIA TROPICAL
REVUE SUISSE DE ZOOLOGIE
SCIENTIA MARINA
SOCIOBIOLOGY
STUDIES IN MYCOLOGY
STUDIES ON NEOTROPICAL FAUNA AND ENVIRONMENT
SYDOWIA
SYSTEMATIC AND APPLIED MICROBIOLOGY
SYSTEMATIC BIOLOGY
SYSTEMATIC BOTANY
SYSTEMATIC ENTOMOLOGY
SYSTEMATIC PARASITOLOGY
TAXON
TRANSACTIONS OF THE AMERICAN ENTOMOLOGICAL SOCIETY
VELIGER
ZOOLOGICAL JOURNAL OF THE LINNEAN SOCIETY
ZOOLOGICAL SCIENCE
ZOOLOGICAL STUDIES
ZOOLOGICHESKY ZHURNAL
ZOOSYSTEMA
ZOOTAXA
ZOOLOGICAL SCIENCE
ZOOLOGICAL STUDIES
ZOOLOGICHESKY ZHURNAL
ZOOSYSTEMA
ZOOTAXA

Appendix 4

Report on meeting with ZooTaxa Editor and proposals arising

Dear PESI colleagues,

Arising from the meeting described below (Annex), I suggest PESI consider two initiatives:

1. A demonstration project that links a taxonomic journal to several taxonomic databases. This could involve providing a metadata entry form for use by ZooTaxa authors to upload their MS, and require them to entry the usual author details, abstract, and document, but also species names, other species described or mentioned (perhaps as a list in one file), distribution (using drop down menu of countries and geographic regions), the option of including a GBIF/OBIS compatible species distribution table /(that could be sent to GBIF/OBIS upon publication), Taxonomic group (order, family) and perhaps major environment (e.g. marine, Land, freshwater, parasite). Once published the species name could be registered in ZooBank, LSID applied, distribution data sent to GBIF/OBIS, and all with associated metadata already captured.

After a trail period, PESI could make this system available for other taxonomic journals (that do not have such facilities) as well, and in the long-term perhaps the journals would pay a modest fee for the service. It could also be used for conference proceedings, etc..

2. A simpler and compatible activity would be an email alert that would go to Taxonomic Editors of WoRMS/FaEU or others notifying them of the publication of papers by a journal that mention the taxon they are responsible for. The journal should give free access to the editor to download and study the paper (regardless of whether it is open access or not) so they can judge its suitability to cite on the taxonomic database. This will promote the journal and its publications. I believe this is a task under WP6 for VLIZ.

Future phases of this system might see authors submitting their paper with the images, legends, tables, and parts of the text in several parts such that they could be re-assembled by organisations such as Plazi or EoL with all associated metadata and attribution. However, I suggest the above steps would be a pre-requisite for this and should be established first.

I cannot recall where all responsibilities for these infrastructure developments lie, but perhaps VLIZ can take the lead in establishing one or both of the above mechanisms for linking WoRMS to ZooTaxa. Then this could be expanded to FaEu and PlantBase later on. No doubt some improvements on the above can be made in due course.

Mark Costello 25 Nov. 2008

ANNEX

Meeting Zhi-Qiang Zhang (Editor and founder of ZooTaxa) and Mark Costello, Auckland, 28th October 2008

Zootaxa is a grass-roots journal that far exceeds other taxonomic journals in the number of papers it publishes each year. It produced about 24,000 pages in 2007 and described 15% of all new taxa described in that year. It draws its revenue from subscriptions and open-access page-charges of $20/page (there is no charge to authors for papers that are not open-access). This pays for the editing, typesetting, printing and online publishing. Although established as a commercial journal it is not aimed to make a profit but provide

a service. Its editor is a full-time taxonomist at Landcare Research, New Zealand. He is an ICZN Commissioner and supports the new names registration proposal.

Zhi-Qiang is keen to advance Zootaxa further to serve the needs of taxonomy and has taken several steps recently:

- With Richard Pyle has indexed Zootaxa papers, authors and new taxa in ZooBank. This has created an LSID for each author and paper, and also for many new taxa (project still on-going with new tax added to ZooBank regularly).
- With Donat Agosti (GBIF grant) provided ants and fish content to Plazi.org.
- With Vincent Smith (EDIT scratchpads) to create an online collaborative work-space for taxonomists to produce publications (i.e. www.Zootaxa.info hosted at NHM London).

The first two tasks are very time consuming to do retroactively. Ideally, authors would create the key metadata when submitting a paper for publication. However, this must be simple, like filling out a web form with ones' name, address, abstract, etc. as most authors do not know XML. Ideally, this would allow images and other content to be re-assembled to provide species identification tools as Plazi.org is demonstrating. The third demonstration project has a very poor response time; it may benefit from being hosted on larger and faster servers with greater bandwidth.

Zootaxa lacks adequate IT support, and plans to adopt or develop an electronic manuscript submission and management system. It would like to provide RSS feeds for its readers and digital object identifiers for its papers. It does not use DOI at the moment but is considering the use of LSID.

Zhi-Qiang proposed at the recent Systema Naturae 250 Symposium in Paris that an international taxonomic society (mainly using the cyber-infrastructure as a means of communication and collaboration) was required. The 5,000 authors and 150 editors of Zootaxa could provide the basis for this. This could be at least complementary to SMEBD (whose members contribute to databases), and probably there is significant overlap in membership already.

Zootaxa has been asked to develop an agreement to supply content to EoL. While positive about this, it will require funding to provide the IT support services. Cynthia Parr is considering this. Zootaxa is aware that EoL is an aggregator and not a creator of content, and holds a strong view of the need for the taxonomists to validate taxonomic content.

I had sent Zhi-Qiang a copy of my article on 'Motivating online data publication'. He agreed with this approach, as it fits well with what Zootaxa is trying to develop through Plazi and ZooBank. For example, Zootaxa could publish papers from which standardised data content could be made available through integrated databases like GBIF or Plazi.

There seem to be three avenues to explore in order of ease of implementation:

1. Developing live links (e.g. RSS feeds) between Zootaxa and WoRMS as a demonstration project for PESI, and associated services.
2. Connecting the world taxonomic community so they can work more efficiently and collaboratively. This is also what WoRMS, EDIT and PESI want to happen.
3. Integrating Zootaxa with the major online taxonomic initiatives like ZooBank, GBIF, EoL.

I suggest that as part of PESI (and/or EDIT?) we explore some activities to link Zootaxa with WoRMS and Fauna Europaea so as to produce mutual benefits to the taxonomic authors and editors, and the wider user community.

Might VLIZ be interested in creating an online document publication system that could be used for ZooTaxa, but also Publication of datasets for OBIS/GBIF, conference proceedings that VLIZ and/or IODE organise? This could be similar to those for many journals but would allow additional keywords and metadata that are important for ZooBank, OBIS/GBIF, IODE etc. to be created by the authors. Would this be an appropriate use of PESI and/or EDIT funding?

Should PESI discuss closer collaboration with EDIT Scratchpads and Donat Agosti's Plazi to create some synergy of effort?

Appendix 4

Notes from the EDIT workshop on scientific publishing, Bratislava, June 2009

Objectives of the meeting:
Bring together technical staff and decision-maker about scientific publishing within Natural History Institutions in Europe in order to help them with the impact of electronic revolution in our field. The meeting aim to inform and bring together these teams.

Presentations of the Publications and Publishers in the EDIT Network:
• 124 titles (of which 79 journals) most of them devoted to systematics and taxonomy, 25 EDIT's institutions are publishers (own at least one a title)
12 representatives met in Paris in December 2008 as a steering committee.

We noticed some similarities and some differences between the Scientific Publishing departments:

Similarities: they share a core mission: dissemination of original scientific results, mainly devoted to taxonomy.

- most of them have the same editorial process: but sometimes with different structures. Some (such as London) chose to outsource their publishing model and entrust commercial publishers with their publications whereas others (such as Paris, Kew, Tervuren…) chose to control the whole process in-house.
- Editorial policy: most of the Journals support the in-house material (authors have to deposit the taxonomic material of the species described in the journal)
- link with the library: aim to sustain the exchange
- low print-run: between 50 and 500
- long standing-journals (1/3 of the journals are at least 60-year old)

Differences: choice of the economic model (pay-per-article *versus* free-open access), 38% of the journals are available online, 28% are covered by IF
According to unpublished data by Benoit Fontaine and Philippe Bouchet who studied the increment of marine biodiversity inventory between 2002 and 2006, it appears that most of the species are still nowadays described in journals published by, or at least on behalf of, natural history institutions.

In the 8th December meeting we identified some issues that needed to be discussed on a second meeting: how to enhance the dissemination and accessibility of our journals, the specific barriers for us being the exchange programmes, the nomenclature and the sustainability of the support. In the Bratislava meeting, useful tools for electronic publishing and dissemination were presented to the audience and the last session was devoted to exchange about technical practice such as print-on-demand, copyrights issues and a proposal to reduce the fragmentation by launching an European joint journal on zoology.

The outputs of the Bratislava meeting will be presented soon (probably in August) in a report and all the presentations will be available on the website of Edit.

Laurence Bénichou
Responsable d'édition / Editorial manager, Publications scientifiques,
du Muséum national d'Histoire naturelle, DICAP, CP 39 - 57 rue Cuvier, F-75231 PARIS cedex 5.
T. 00 33 (0)1 40 79 48 38; F. 00 33 (0)1 40 79 38 40; Website: http://www.mnhn.fr/pubsci
