# Supplementary material for: PESI - a taxonomic backbone for Europe
Source: Biodivers Data J. 2015 Sep 28;(3):e5848. doi: 10.3897/BDJ.3.e5848 (PMC4609752; doi:10.3897/BDJ.3.e5848)
Supplement: Supplementary material 6 — Design of a mechanism to keep control of the continuity of European electronic (taxonomic) biodiversity data resources and expertise networks [file biodiversity_data_journal-3-e5848-s006.pdf]

A Pan-European Species-directories Infrastructure

PESI WP2 — Deliverable D 2.3

Design of a mechanism to keep control of the continuity of European electronic (taxonomic) biodiversity data resources and expertise networks

Table of Contents

Aim ... 3

Introduction ... 3

European electronic taxonomic biodiversity data, resource and expertise ... 3

Continuity of Taxonomy into the future ... 7

Current situation ... 7

- Vision ... 7
- Mission ... 8
- Organisation ... 8
- Products and services ... 11

External environment ... 13

- The economy ... 13
- Market analysis ... 13
- Competitor analysis ... 14
- Competitive advantages ... 14

Strategy and plans ... 15

- Objectives ... 15
- Taxonomic strategy ... 15
- Marketing strategy ... 15
- Operating plan ... 17

Financial analysis ... 20

- Operating costs ... 20

Risk analysis ... 21

- Critical success factors ... 21

Conclusion/Discussion ... 22

Aim

This deliverable aims to design and implement a mechanism to keep control of the quality and updating of taxonomic biodiversity data i.e. the continuity of European electronic biodiversity data. In delivering such an aim this report includes the continuity of taxonomic resources and expertise networks as being vital elements in the long-term accomplishment of continuity.

Introduction

The long-term maintenance of taxonomic knowledge infrastructures is a major concern throughout the world. It cannot be predicted how technology will develop in the long term. Therefore, PESI and the taxonomic realm must develop a survival mechanism which is not only flexible but also strong enough to persist beyond the short term goals of many projects.

The strengthening and integration of European taxonomic communities initially began with taxonomic indexing EU framework programmes Fauna Europaea, ERMS, and Euro+Med PlantBase which developed expert networks who contributed to the defragmentation of the European taxonomic expertise. In addition, their expert resources were organised in the Society for the Management of Electronic Biodiversity Data (SMEBD). One of the key elements involved in driving this initiative forward is SMEBD with PESI providing the necessary impetus and resources to realise these tasks and to move them forward. SMEBD was originally established to develop sustainable approaches to the databases themselves, the expert networks and the intellectual property rights of electronic taxonomic resources.

This document helps to outline how the PESI partners envisage the control and continuity of European electronic (taxonomic) biodiversity data resources and expertise networks into the future. This document was not written in isolation, but in conjunction and parallel with some key deliverables such as the business plan (D1.3) and the working plan (D2.1). The results from the work packages and the development of new projects along with the key players in the field of taxonomy and electronic databases also played a part in the designing of an effective mechanism.

European electronic taxonomic biodiversity data, resource and expertise

It may be useful to distinguish the resource into three components, namely (1) the electronic taxonomic biodiversity data, (2) the experts who contribute and validate its content, (3a) the

software, including the web interface and services, and (3b) the hardware, including archiving, back-ups, 24-7 online access, and response time.

Typically electronic biodiversity data is held within a taxonomic database developed by one or a few scientists who work for a university, museum or other scientific organisation, however there are exceptions where personal databases have been created. Most of these online taxonomic resources are created by short-term project funding and/or the time contributed by one or more scientists. The first issue that arises with continuity of databases is **funding**. It may have been initially created to satisfy the needs of a funding agency for research results to be made publicly available, and its creators may or may not wish to continue to develop it once the funds have depleted. The resource will become out-of-date and may go off-line when the project funding ends, the host institute changes its web site, or the scientists move positions or retire. Funding involves all three components i.e. the development of the database and operational costs which include software, hardware and personnel time e.g. data encoders and IT people.

There is the potential that each of these components may be managed by different people and have different funding streams. If the resource is a stand-alone facility, with its own hardware, software, IT support and scientific staff, it will have a significant budget, probably in hundreds of thousands of euro per year. However, most taxonomic databases are modest in their demands for resources and may be more cost-effectively maintained if they are integrated into larger computer systems. If this is planned at an early stage, and the database follows common standards, the difficulties in extracting data due to idiosyncratic formats will be minimised.

The funding sources may be grouped into host organisations, funding agencies, individual scientists, and users. Ideally, it is desirable to have portfolio of funds from several sources in case some are unavailable at some time. For example, the European Register of Marine Species was initiated by a €385,000 research project in 1997 (Costello 2000), moved to a new host institution in 2000 which had €250,000 of projects that built upon it, received six small grants from projects funded by EU and USA sources totalling €110,000, and then €300,000 and €400,000 projects in 2004 and 2008 respectively to develop the content, editorial board, and infrastructure further. The incremental extensions of the content resulted in a new goal to produce a World Register of Marine Species which doubled the size of its editorial board while making it a more prestigious and valuable resource (Appeltans et al. 2009). Between projects, the host institution can maintain the online services and address user needs, while the editors can keep it updated with modest time input.

However, the scientist(s) may be very successful in winning regular funding to develop the database over some years, and perhaps a decade or more for example as is the case with AlgaeBase. However, ultimately, somebody else will need to take over the organisation; unfortunately no one lives for ever so **succession** i.e. a new chair/co-ordinator to maintain the development of the database. In relation to the larger databases which have several collaborators involved it will often facilitate a smooth transition to a new chair/co-ordinator.

The PESI project developed an expert network-based process to build and maintain an authorities list of the European species. Two kinds of networks have a specific role in the process. Firstly, the expert network, with a structure of Group Coordinators (GCs) collaborating with Taxonomic Specialists (TSs) on their taxonomic groups. Secondly, the national Focal Points (FP), which are the institutes that maintain national checklists, or can be regarded as the primary source of information about the national fauna, and the country's taxonomists and literature resources. In summary, the roles of the two networks are related to either the data collation process (the focal points), or the validation process (the expert network).

A survey conducted by the Fauna Europea project concluded that, whereas the compilation of a complete database of European species is a huge task aptly deserving some form of financial compensation, updating a completed database will expectedly take much less efforts, and most Group Coordinators seem to be happy with minimal benefits. However, it should be kept in mind that when there is a high workload involved it is likely that they will expect compensation for their efforts. This would especially be reflected with those groups that have incomplete data sets. A report on what gaps are present and how to complete these taxonomic gaps in Europe will be addressed in D2.4.

The valuable input from the Group Coordinators and Taxonomic Specialists must be acknowledged by giving them proper credit with every (on-line or off-line) publication. This is crucial for these experts to maintain their involvement in the continuity within their networks and as a result the continuity of the electronic taxonomic databases (See D2.1 for further information on the topic of accreditation).

In general, the tasks related to the continuity of the electronic data resources can be divided into: maintenance, updating, validation of the European species lists and global species databases (GSDs) and as a result the **long term hosting** of the databases.

To provide an appropriate IT- infrastructure for future **maintenance**, the hosting of the servers, such as the PESI server, and related technical support has to be considered. This includes the survey of possible database platforms for the final release of PESI (e.g. Oracle, MySQL, SQL Server) and an inventory of the expected technical services on system management (hardware continuance, performance, security), server support (system upgrades, bug elimination), and application development (configuration changes, additional developments).

To provide an efficient **updating** system improvements are required for both the on-line and off-line data entry tools and associated data management system. This will guarantee the ease of future data importing and updating of existing data. This also includes the implementation of the so-called business rules to secure the input of validated ('cleaned') data into the database for both on-line and off-line data entry tools, advanced tools for changing parent taxa for lower taxa within the on-line tool, improvements on the user-role access system if relevant, and the delivery of a prototype of the off-line tool allowing data export and import routines through internet by use of XML format.

However, prerequisites for ensuring it lives on include:

1. The resource must
    a. Have a significant amount of unique content;
    b. Be regarded by scientific peers as being of top quality;
    c. Have scientists willing to spend time maintaining or developing its content;
    d. Have a good track record in its development;
    e. Be clear on its Intellectual Property Rights, copyright and ownership.
2. If the above is the case the resource should seek recognition by:
    a. Having its value to science endorsed by large scientific and governmental organisations
    b. Having a large user community, especially amongst scientists, government organisations, and appropriate industry, but also public (e.g. student, teacher) use.
    c. Documenting use of the resource, such as pages viewed and content downloaded over time, citation in scientific papers, links to it from other websites, and uses in research, management or policy arenas.

If these conditions are satisfied then it has become part of the science infrastructure, and other scientists or organisations may feel proud to continue it. The factors that motivate scientists and

institutions to support such resources are similar, and include its prestigious nature, direct benefits to their work, and relevance to their mission.

For these reasons, there should be a succession plan for the resource to be continued. This may involve integration into another larger database that has long-term support, additional project funding, finding a sponsor, transferring control to other scientists, and any combination of these options (see D2.1 for further information on the European Taxonomic Work Force).

Continuity of Taxonomy into the future

Essentially all of the information presented until now has set the scene on the continuity issues and how problems have and are being tackled and how potentially we could continue to solve them and develop a new 'mechanism' as it were to incorporate all of these issues of control and continuity. Taxonomists need to re-examine not only what has to be achieved in order to survive into the future but also how consensus decisions about this will be achieved. There also is the case of the resources required and how to engage taxonomists to collaborate and organise the continuity into the future.

The use of the term 'Mechanism' within the title deflects from what is required for the control and continuity of taxonomy. This task needs an objective viewpoint and a business rather than a scientific perspective. This task is more comparable to how a company can survive in recessionary times than the survival of the kakapo, a flightless and nocturnal parrot on the edge of extinction.

Here we examine the 'company' as having three elements, all of which are essential for the survival of the company, and devise a plan of action/mechanism to keep the control and continuity of European electronic (taxonomic) biodiversity data resources and expertise networks into the future.

While SMEBD has devised a business plan on how it intends to survive into the future and manage electronic databases here the plan needs to be more specific and directed at the databases, resources and expert networks themselves so that SMEBD will have something to manage!!

Current situation

Vision

The vision for the future is for the European Taxonomists to continue to operate a dedicated taxonomic knowledge e-infrastructure but to incorporate the new organizational structure and working procedures of the taxonomic workforce outlined in D2.1. This would include validated high-end electronic taxonomic data, resources and expertise which would be accessible to all.

Mission

To keep control of the continuity of European electronic taxonomic biodiversity data resources and expertise networks.

Organisation

Below is a proposed taxonomic organisational structure where the lines of communication involve a number of the key taxonomic players in Europe with a vested interest in electronic biodiversity data and databases. While it may not be obvious why some partners are involved, it is that they envisage digitising their collections in the future and therefore it is important that they are involved from the start.

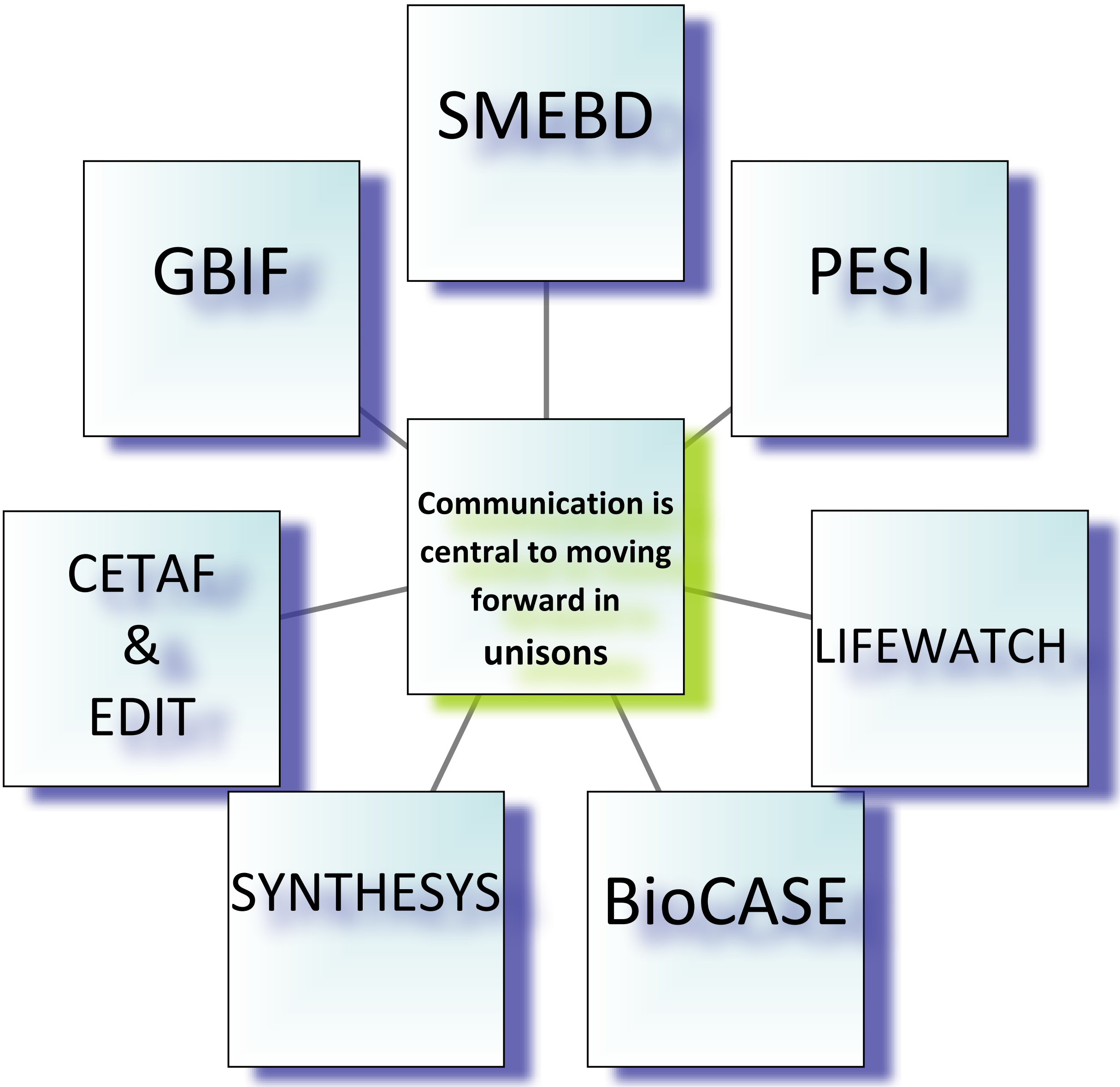

Fig. 1 An overview of the key taxonomic players in Europe

While the figure above provides an overview of the broader picture the organisation of the expert networks needs to be considered. The Taxonomic Expert Database (EditExpertNet) developed by EDIT (see figure below) depicts how potentially all taxonomic networks could be linked together under one 'umbrella'. EditExpertNet is an information service whose objective is to provide an efficient online information service on European Taxonomic experts, their expertise and ongoing and planned taxonomic research projects, building on existing services and content from previous efforts. This robust expert infrastructure has the potential to be the back-end networking tool to co-ordinate the taxonomic community. A front-end facility TaxNet is under development to ease the networking. EditExpertNet also has the means to encourage recruitment of new experts with the aim to achieve the needed critical mass activity for sustainability. These methods are important to motivate all taxonomists to deliver more quality work into the taxonomic community. However, the most important role of the EditExpertNet and TaxNet is to help channel current experts to become more permanent members within the taxonomic networks prioritising the European Checklists. Active collaboration in a test-community, TaxNet for Dipterists, encourages experts among its 1300 members to take responsibility in Fauna Europaea (v.f. D2.1). New networks of experts would be encouraged in other taxa.

Career and non-career taxonomists would work together in virtual and in personal networks to enhance the inclusiveness of the Open Source Taxonomy (OST) model. The hierarchy of the members would be based on the way an Open Source Society (OSS) operates in that the most competent member of each role would take up a position and it would not be a reflection on their work status i.e. whether they are a career or non-career taxonomist. This hierarchy would be aided by an OSS rewarding method of meritocracy called stratification mapping (See D2.1, C2.2 for more information).

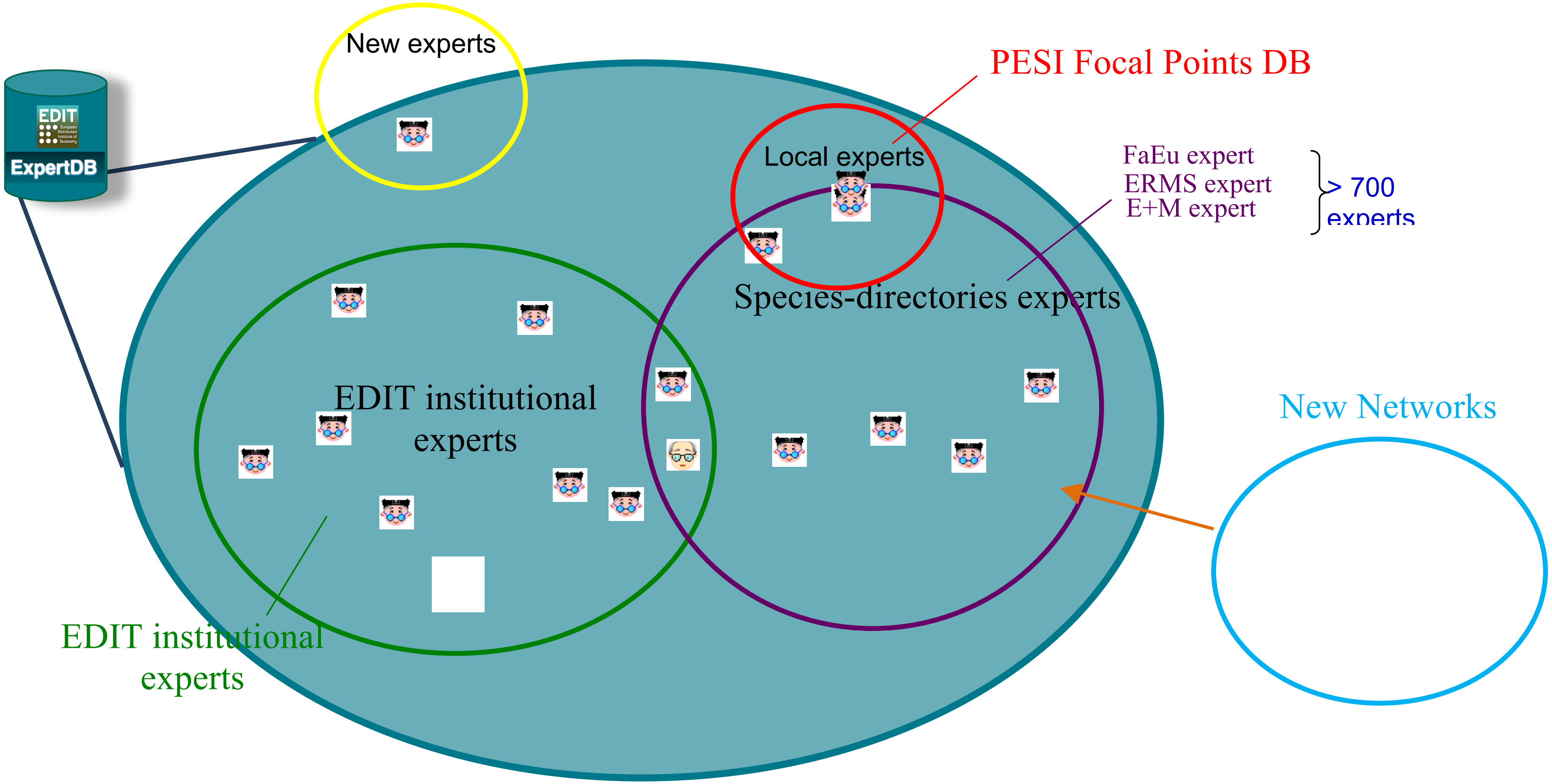

Fig 2. The EditExpertNet involving different networks and individuals.

Within the umbrella of the EditExpertNet & TaxNet, each individual network has its own organisation. The organisations of a number of networks and suggested improvements going forward, particularly those involved in the PESI taxonomic taskforce (PESI TT) are outlined in detail in D2.1. For example the PESI TT currently consists of contributors to Fauna Europaea, Euro + Med PlantBase and the Eruropean register of Marine Species (ERMS). These *c.* 700 individuals are organised within SMEBD, which owns and governs the copyright of their contributions. Membership is for lifetime; even if a member no longer contributes they continue to have a voice in how the databases are managed. The SMEBD council is elected by the members and appoints committees to manage its databases. This management includes appointed host institutes and taxonomic editors and distribution of the database. D2.1 provides a suggested workflow (see figure 3) for input to checklists however as more career experts retire open review may take over as the first step to relieve the reduction in numbers of reviewers. This is only one example of the many suggested improvements to the organisation of networks with electronic biodiversity data described in D2.1.

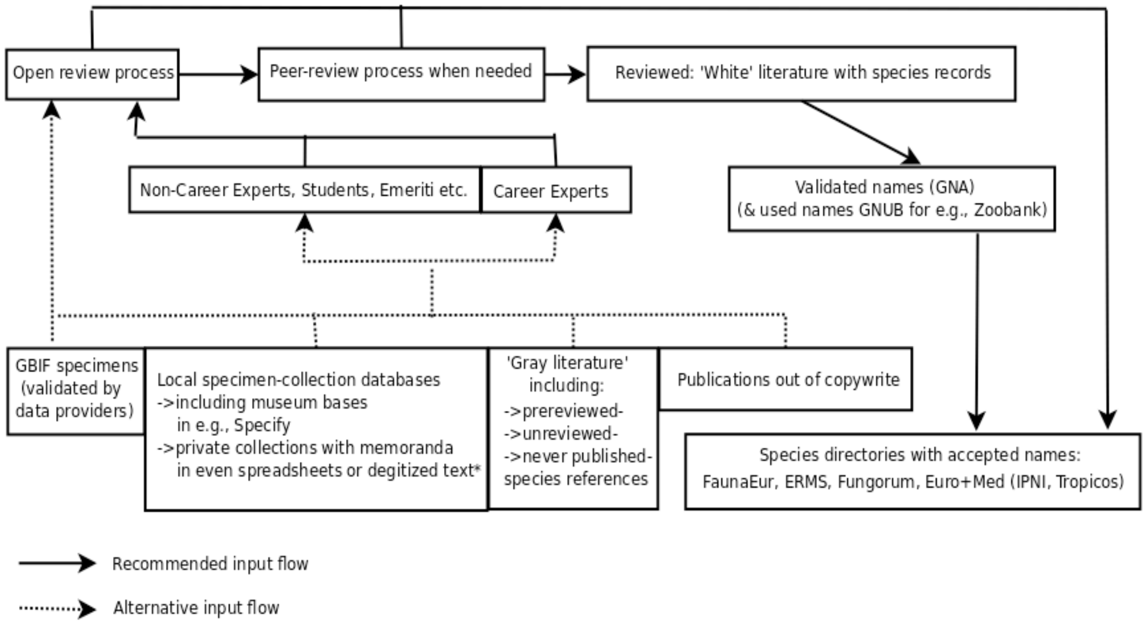

Fig. 3. Outline of the suggested future work flow to keep updating the European taxonomic online resources (journals, checklists, and other databases).

Products and services

The expertise networks and projects have produced a number of significant taxonomic products and services that are currently free to access on line:

The European registers have created European Species Databases which include **lists of accepted species names** and synonyms. The PESI project has expanded this to include a range of information in relation to species recorded in Europe for example:

a. Classification

b. Country lists
c. Distribution lists
d. Endangered/protected species
e. Common names

The Taxon matching tool provides online quality control tools that authors can get automatic feedback on the correctness of their species names (including synonyms and classification), and existing distribution by matching them against one or more PESI databases. It should be best practice that as part of methodology of a project producing a species list states that they have checked species names and perhaps distribution information, against such authoritative online database. They may then analyse their data with this additional data, and know if their data repeats other findings or is novel in any way.

The **platform for Cybertaxonomy** covers nomenclature management, extraction of data from literature, geographical information, computer-aided description and identification, publication support, specimen data access, and other fields.

Scratch pad websites provide users and their community of researchers a space to work on the web. They are easy to use, adaptable to reflect users needs, and provide the user with powerful tools for managing data.

An online **communication facility**. A platform, similar to facebook, but aimed specifically at taxonomists as the usergroup. This facility provides users with the support for efficient planning and collaborative management going forward (v.f. D2.1). It is an active and user-friendly facility that creates structured and selective networking.

Future planned products and services include

a) **Mapping** the distribution of biodiversity data
b) **Data analyses** on biodiversity and community ecology data analysis a link can be made with community ecology softwares packages or demo versions that users can use following instructions to analyses their data, e.g. diversity indexes, ordination techniques, univariate and multivariate statistical test.
c) Digitized database of **Europe's unique collections** for potential multidisciplinary use.
d) Expert transparency along with an education tool to both train experts and to allow transparency within the taxonomic workforce (vf. D2.1).

External environment

The economy

Hot topics for debate and on the political agenda are biodiversity loss and global change both of which require a quality list of species and their distributions in order to detect changes.

Market analysis

Technology does not work in isolation, and requires parallel development in contributor and user practices and user needs were explored. This is not a simple task because users are often not good at explaining what they want, and appreciating how new tools can make their work easier or faster.

In the case of digital taxonomic data it is the end users who are to be the primary market. An end-users forum and a user feedback system will allow the user to comment and provide feedback on the system performance, and will also allow other experts to communicate on the quality of the taxonomic data and their needs moving forward.

Provide standardized web-based and/or offline template for authors to create data appendices that they can then publish their species distribution data through the Global Biodiversity Information Facility (www.gbif.org) and similar biodiversity initiatives (e.g. Ocean Biogeographic Information System). This could be an appendix (excel sheet or similar) to a journal paper, and include the paper citation as the source (thus promoting the authors and journal). In this way the authors work is more widely disseminated (publicised) and can be automatically integrated with larger datasets which enables other scientists to reproduce the study findings, and repeat the study with new data. It is widely accepted that authors should make data available, and this is normal practice in some fields (e.g. genetics data goes to GenBank), but does not happen routinely for species distribution data.

The products need to be freely available for a number of users outlined below but there is also a market for the major users who will pay a financial contribution for the complete product along with regular updates. One of the obvious options available to ensure the continuity of the biodiversity databases is to raise funds through special services and products however this is not the only one (see marketing strategy below). The following categories of small scale typical End-users for the PESI portal and the taxonomic registers have been identified

Table 1: A selection of the variety of End-Users of taxonomic biodiversity data resources and expertise networks in Europe

Science	Education / Health	Environment/ Conservation	Agriculture / Game	Trade	Policy and administration
Universities	Students	Government Agencies	Farmers & coops	Customs	Policy makers
Professional societies	Journalists & Writers	Consultancies	Agricultural services	Forensics	Legal Authorities
Biotechnology	Public	NGO's	Pest / Invasive control agencies	Service industry	
research organisations	Eco-tourism	Engineers	Land-owners and land-use managers		
Applied sciences			Hunters, game-keepers		

Competitor analysis

There is no other competitor providing the same resources with or without the level of quality control that is offered here.

Competitive advantages

Taxonomic expertise and Networks are currently established which operate, provide and maintain a Checklist for Europe which provides the 'Taxonomic Backbone' from Europe feeding into to the Global Names architecture as the European contribution.

This active taxonomic expert networks have been transferred into virtual research communities and are networking via PESI, SMEBD etc. and have established partnerships with international biodiversity research programmes like Lifewatch, GBIF, EoL, and CoL.

Data resources and Quality Control

The data validation that has been established in European taxonomic databases such as the European register ensures a very high level of quality control. Measures are underway to ease the challenge of keeping up to date on the latest publications in species taxonomy and distribution via the development of tools such as RSS feeds that will automatically notify taxon editors and data managers of who has published on certain taxa in Europe (T6.2. E-science applications). The database can then provide the publisher with the full taxon list and contact for respective editors.

Strategy and plans

Objectives

To achieve such critical-mass of users as a result of the quality and useful content of the taxonomic databases and as a consequent raise interest from potential long-term national funding sources and backing.

Taxonomic strategy

The value of European Species Databases are currently underestimated and the dependence of a number of services on this data needs to become more transparent. The ground work has initiated with the networking and partnerships that have been established both within Europe and internationally by SMEBD and during the PESI project and these will continue beyond the lifetime of PESI. The suggested 'vehicle' to keep control of the continuity of European electronic (taxonomic) biodiversity data resources and expertise networks is SMEBD.

SMEBD – the way forward…

SMEBD is already making strives to become this 'vehicle' with the recent establishment of an official Board of Directors from around Europe. The development for a business plan for the continuity of SMEBD is underway (D1.3: A business plan to guarantee the long-term continuity and exploitation of the Pan-European Species directives Infrastructure) and is due for completion in early 2011 and will be presented at their annual meeting. In the interim of presenting the business plan SMEBD's current chair, treasurer and secretary have been actively and successfully implementing the proposed plan to generate revenue to offset the overheads incorporated from banking, audits etc. and to allow SMEBD become self-sufficient.

Action

The next strategic move would be the joint promotion to highlight to 'all' how taxonomic data is a vital source to be maintained into for the future and the core for everything that is biodiversity! In developing and promoting the benefits of the current resources (electronic taxonomic biodiversity data) to new and a broader range of users this will increase the visibility of the potential of the data and increase the chances of backing from national and international resources. Therefore, a marketing strategy is required.

Marketing strategy

The main focus of any marketing strategy is to improve communication with customers or in the case of SMEBD and GSDs the users. Communication can be successfully achieved orally (person to person or via telephone or internet telecommunications), written (brochures or letters) or via online

websites. While projects such as PESI frequently have an outreach package once the project is finished the marketing of its products cease.

Promotional marketing

1. Awareness for EU-nomen as a brand is important as is a catchphrase that depicts the database e.g. EU-nomen: "The site that offers you the Accepted name every time".

2. Target audiences which will not only make a difference to the number of users currently employing the service but also raise the bar for the level of quality data out there for example:

    a. Environmental policy makers who commission work such as NATURA 2000 site monitoring
    b. Councils that sanction planning applications, where a site assessment of the biodiversity present is required.

Each of these civil servants could make 'taxon matching' obligatory as part of the results presented in the tenders, ensuring that the results are a true depiction of the state of the biodiversity and not incorporating a series of synonyms. This not only increases awareness to the civil servants but also to the consultants and the general public whom have an interest in the wildlife in their area. Having the electronic databases accessed in this way makes them apart of everyday life and their importance soars and increases the likelihood of their continuation through sponsorship.

Sales marketing

It all comes down to sales in the end! To get that elusive deal of long-term national sponsorship from governments around Europe to guarantee the continuation of electronic biodiversity databases and their associated expertise networks. This however is work in progress and will not happen overnight. In the interim there are a number of ways to attain funding:

1. There are a number of key players (ICES, EoL, CITES, IUCN, Research Universities both in Europe and outside, to mention a few) where for example a complete copy of for example the EU-nomen database would be appealing. One approach could be to offer the Database for free but charge a yearly subscription for updates.

2. Allow website advertising on the portal which is specifically related to the product and audience so as not to downgrade the site – for example the advertisement of scientific books, events for museums or consultancies offering services.

3. SMEBD is exploring the potential to charge a nominal financial contribution entitled 'database usage financial contribution' for WoRMS. This is more aptly charged to the larger

institutes and universities world-wide and to date has received three successful contributions. This is another potential avenue to explore for funding databases.

4. Not to overlook the importance of EU funding to date, particularly from the Framework programme, this has been vital in the success of achieving the high level of resources and networks available today. The securing of EU funding will be important in the interim of securing annual patronage. These projects will not only allow the databases to be maintained into the near future but will also continue to build, filling the current gaps which will be highlighted in D2.4 (Detailed plan of how to compete taxonomic gaps in the pan-European species registers, including experts and informatics resources).

Collaborative Marketing

Probably not a technically correct area of marketing, however it is apt for this situation. The integration of taxonomic databases with other online biodiversity data resources, for example advanced linkages and integration of with other projects working in the domain of biodiversity information could be realized by setting up sophisticated portal services (which includes the development of a meta database for elaborated linking), and through a Euro-Hub, which is an effort of the EU-funded EuroCat project. Furthermore, as an open network, the European Network for Biodiversity Information (ENBI) will harmonize these European activities towards the Global Biodiversity Information Facility (GBIF).

Operating plan

The Open Source Society (OSS) community has provided several new ideas on how to develop and organise the Taxonomic workforce in Europe. It has envisaged that the global economy will continue to be knowledge based into the future and that an open source society will be leading the way (see D2.1 for more information on this). Therefore the way forward for the operation of the taxonomic community is the Open Source Taxonomic (OST) model outlined in D2.1 where a number of procedures and methods from the OSS have been adapted and some of which are described in brief below and illustrated in Figure 3.

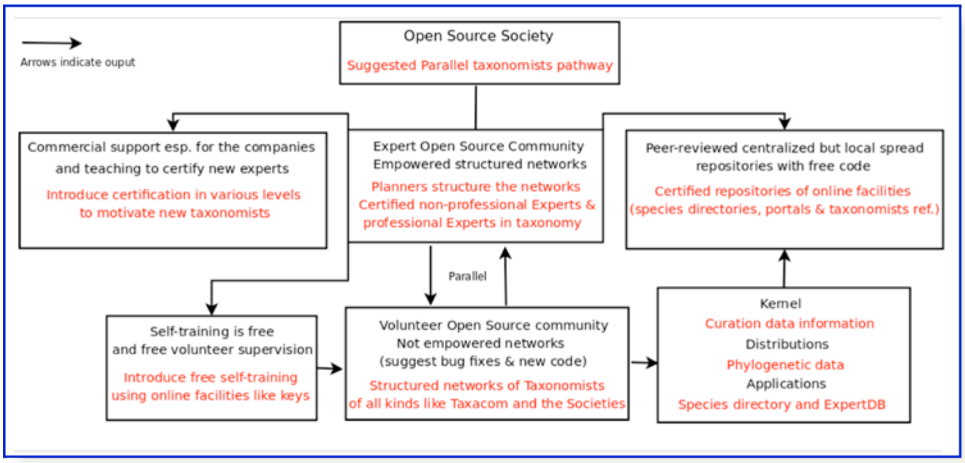

Fig. 3. The Open Source Society network as an example for modelling the future Taxonomic Community

The present and future OST model for communication, management and network co-ordination of the taxonomic community within the community involves the Taxonomic Expert Database (EditExpertNet) developed by EDIT. While D2.1 (C.2 European expert network/community service) provides a more in-depth look at the network services here only a synopsis is provided to illustrate again how the networks potentially can be sustained.

Communication between networks would involve the establishment of an Ad-Hoc or specific networks. EditExpertNet & TaxNet currently allows members to join or create a line of "Direct" communication in self-made adhoc networks. For more permanent contact the creation of a, "Specific" new line of network communication allows a member to connect to already established networks creating a new forum where more than one network is involved. Already established networks can easily be searched for within the database.

The information service was designed for both internal as well as external use. The Expert Database is currently being advanced to facilitate direct communication; however, to be useful in the long run, these databases and the frontend networking facility need technical maintenance and regular update of content.

The incorporation of SMEBD experts within the EDIT Expert DB, not only improves communication within the taxonomic community but it also allows for the integrated management of a pan-European network of taxonomic experts that could potentially create a common agenda for grant applications and research funding, including an integrated cost model for the maintenance of the relevant e-services.

D2.2 has set out a working IPR model, based on the current SMEBD model, under which PESI can proceed with its aim to integrate taxonomic databases in Europe with an IPR model that will ensure the free and wide dissemination of data, promoting the furthering of science in this field. The SMEBD model encourages scientists to input their data whilst not being burdened with the upkeep of their data. This allows scientist to retain full ownership of their work and have confidence in knowing that their data is being managed accordingly by data stewards.

This IPR model suggests a Creative Commons Attribute-ShareAlike (cc by-sa) licence that allows for the wide dissemination of data to other scientists and interested parties, but still provides recognition of the work done by the creator of the data. The ShareAlike clause will ensure further dissemination of derivative works, thereby promoting the idea of Creative Commons and the sharing of data.

In relation to citation of data, D2.2 recommended that contributing databases adopt a standard citation system to simplify the citation system in PESI and that the date accessed be automatically added as in ERMS.

The use of Open Source software has been of great importance in the development of not only the PESI portal but also the provision of biodiversity database webservices and more. The use of open source licences should be considered for any future software created for the development of taxonomic resources.

Should SMEBD be successful in driving this initiative forward then it would be apt that a member and perhaps even one of the Directors would take on the role of the Marketing Director.

GenBank could be used as comparative to the way forward for the operation of electronic taxonomic resources essentially a 'TaxBank'! GenBank is now an integral part of the world science e-infrastructure with host institutions in three different countries, and a large global network of scientists who use it daily in their research. This has been aided by the large resources for human, animal, plant and microbial genetic sciences, and the fact that genetic data is more amenable to data management than text based information.

Opportunities

Task 6.2 (E-science applications report) revealed that in the past three decades there has been a decline in the number of opportunities available to publish new records for the distribution of species. This is especially the case where the novelty is limited to new national or regional locations for species, or where the data only confirms the continued presence of a species. However, this data has increasing value over time, and biodiversity informatics enables international collaboration and data management to be fast at low cost. Global change is now a part of everyone's life. Changes in the distribution of marine species due to sea temperature rise is very apparent today none more so than in the Arctic, where an increase in biodiversity is threatening the native species. The use of the accepted names of these species is the platform to illustrate their distributions. Such location data could be published electronically such that it can be integrated with other data through GBIF (T6.2, E-science applications report).

However, at present this approach lacks the prestige associated with the peer-review and quality control systems in scientific journals. There is an opportunity to develop mechanisms with scientific journals for them to publish such data as electronic appendices in standard formats that will enable automatic publication and integration of the data in GBIF, OBIS, *Atlas Florae Europaeae* and similar systems. This will increase visibility for the journals and their authors, and thus their citation rates. More importantly it will help change science culture to embrace the role of online data publication. In this way, scientists will be motivated to contribute content to online databases (T6.2, E-science applications report).

Financial analysis

Operating costs

Storing and managing a large and ever-growing amount of data is a challenge for smaller institutes as they typically lack the resources to handle it. Some Institutes have developed expertise and logistical support for the development of data and information systems along with the archiving of datasets the services which they outsource to projects and smaller institutes e.g. Flanders Marine Institute (VLIZ). The cost structure of the core service in relation to European electronic biodiversity data consists of 4 components:

- Data (additions, updates, validation)
- Information technology (server hosting, software maintenance, updating)
- Management (coordination, project development, administration)
- Overheads (housing, equipment, travel)

WP5 in PESI has carried out a detailed evaluation on the costs of the preservation and the maintenance of existing GSDs (D5.4 Pan-European checklists and GSDs sustainability plans as components of business plan). Once this report has been finalised and the figure calculated for each GSD then a realistic figure can be calculated for the continuity of electronic biodiversity data.

Risk analysis

Risks to be considered in relation to the success of the continuity of European electronic (taxonomic) biodiversity data resources and expertise networks include:

Resource constraints - In recent year's taxonomists and their associated skills have been seen as a dying breed. However, with the resurgence of new career taxonomists through the efforts of EDIT this risk has been reduced, but, by no means eliminated.

Quality Assurance – The expertise of taxonomists has been acquired over several years of practice and this will take time to build in the new generation of taxonomists. Is there time to pass on these valuable skill sets to ensure a high level of quality assurance in future years?

Investment – Insufficient investment in training, career development and research to feed into a continuous feed of a new generation of taxonomists. EDIT is currently tackling this problem so for now the risk is reduced.

SMEBD management and marketing – Insufficient investment in marketing and sales to secure cash flow for the maintenance of resources including a secretariat is a potential risk to success.

Natural disasters – the storage of backups of GSDs at an offsite/online facility so as to avoid the complete loss of data due to unforeseen circumstances such as earthquakes, flooding etc.

Critical success factors

One of the most vital elements for the successful continuity of European electronic (taxonomic) biodiversity data resources and the expert networks moving forward is the accreditation of those involved in order for them to continue to carry out the most vital work as gatekeepers, group co-ordinators, editors, authors etc. This is described in detail in the working plan (D2.1: Chapter. 2 - accreditation system for PESI-ETW).

Open collaboration to allow the integration of taxonomic databases with other online biodiversity data resources should not be overlooked going forward.

The securing of funds to finance the maintenance of data resources and expertise networks is fundamental to continuity into the future.

Conclusion/Discussion

The working plan (D2.1) has examined the taxonomic workforce in Europe and beyond how improve its organisation and the challenges it faces moving into the future. This document while on the surface it may, in places, appear in contradiction to the movement towards open source taxonomy, if you look deeper you will see that currently all the services developed are free to use online. However, the need for capital to maintain these resources including the data encoders cannot be overlooked. This funding/sponsorship needs to be long-term and not per project basis to allow the continuity of electronic data resources and expertise networks. Taxonomy in its own right needs to be campaigned to be on the political agenda and not under the veil of biodiversity loss and climate change. Policy makers throughout Europe need to be lobbied in unisons for the plight of taxonomy and the maintenance of electronic resources in Europe.

Had the world listened to Thomas Watson, the Chairman of IBM in 1943 when he said “I think there is a world market for maybe five computers” where would electronic data resources be now? The directors of SMEBD and GSDs worldwide need to be aware of how quickly technology is moving and be prepared to be ready for a fight for the survival and continuity of European (taxonomic) biodiversity data resources and expertise networks.

Configuration History			
**Version No.**	**Date**	**Changes made**	**Author**
0.5	15 December 2010	First draft for circulation	RN / EcoServ staff
0.6	21 December 2010	Minor corrections	YdJ
0.7	14 January 2010	Second draft for circulation	RN
1.0	4 Februari 2011	Final first version	JK & YdJ
