# Supplementary material for: PESI - a taxonomic backbone for Europe
Source: Biodivers Data J. 2015 Sep 28;(3):e5848. doi: 10.3897/BDJ.3.e5848 (PMC4609752; doi:10.3897/BDJ.3.e5848)
Supplement: Supplementary material 7 — PESI Business Plan [file biodiversity_data_journal-3-e5848-s007.pdf]

PESI Business Plan

____

Yde de Jong (ed.)

Index

INDEX 2

Context of the report 4

PESI SUSTAINABLE COMPONENTS 6

EUROPEAN EXPERT NETWORKS MANAGEMENT 6

Introduction 6

Fauna Europaea (FaEu) 7

Euro+Med PlantBase (E+M) 7

European Register of Marine Species (ERMS) 8

Europe Index of Fungi (IF-EU) 8

AlgaeBase Europe 8

Society for the Management of Electronic Biodiversity Data SMEBD) 9

EDIT Expert DB 9

EUROPEAN FOCAL POINTS NETWORKS 11

Introduction 11

Focal Points continuity plan and LifeWatch connection 11

Further outreach (in space and time) 12

EURO-BASED NOMENCLATORS AND GSDS/RSDS 13

Introduction 13

International Plant Names Index (IPNI) 14

ZooBank 14

European-based GSDs/RSDs 14

PAN-EUROPEAN CHECKLISTS DATA MANAGEMENT SYSTEMS 16

Introduction 16

Fauna Europaea (FaEu) 16

Euro+Med PlantBase (E+M) 17

European Register of Marine Species (ERMS) 17

Europe Index of Fungi (IF-EU) 17

AlgaeBase Europe 18

TAXONOMIC BACKBONE E-INFRASTRUCTURE AND E-SERVICES 19

PESI CDM-store and PESI Data Warehouse 19

PESI Portal 20

PESI Portal service & LifeWatch 21

PESI general outreach and Global Names Architecture contributions 21

APPENDICES 25

Appendix 1 — Letter of UvA to MfN on migrating the hosting of FaEu 25

Appendix 2 — Individual Focal Point Sustainability Plan — NHM — Partner 4 26

Appendix 3 — Individual Focal Point Sustainability Plan — UNIPA — Partner 15 28

Appendix 4 — Individual Focal Point Sustainability Plan — CSFI — Partner 21 30

Appendix 5 — Individual Focal Point Sustainability Plan — NIB — Partner 30 32

Appendix 6 — Individual Focal Point Sustainability Plan — RBINS - Partner 00 33

Appendix 7 — Individual Focal Point Sustainability Plan — NTNU - Partner 24 35

Appendix 8 — Individual Focal Point Sustainability Plan — NRC - Partner 20 37

Appendix 9 — Individual Focal Point Sustainability Plan — SMNH — Partner 25 39

Appendix 10 — Individual Focal Point Sustainability Plan — NRM — Partner 22 42
Appendix 11 — Individual Focal Point Sustainability Plan — LU — Partner 33 44
Appendix 12 — Individual Focal Point Sustainability Plan — IBSAS — Partner 17 46
Appendix 13 — Individual Focal Point Sustainability Plan — NNM/NCB — Partner 19 48
Appendix 14 — Individual Focal Point Sustainability Plan — MBA — Partner 39 50
Appendix 15 — Individual Focal Point Sustainability Plan — TU — Partner 3 52
Appendix 16 — Individual Focal Point Sustainability Plan — NMNHS — Partner 31 54
Appendix 17 — Individual Focal Point Sustainability Plan — UCPH — Partner 2 56
Appendix 18 — Individual Focal Point Sustainability Plan — IBSS — Partner 4 58
Appendix 19 — Individual Focal Point Sustainability Plan — NKUA — Partner 18 60
Appendix 20 — Individual Focal Point Sustainability Plan — ABG — Partner 00 62
Appendix 21 — Individual Focal Point Sustainability Plan — HCMR — Partner 34 65
Appendix 22 — Individual Focal Point Sustainability Plan — CUB — Partner 23 68
Appendix 23 — Individual Focal Point Sustainability Plan — CSIC — Partner 29 70

CONFIGURATION HISTORY **72**

Introduction

Context of the report

The urgency of global problems related to conservation and sustainable use of biological resources is generally acknowledged. Obstacles to the proper development and implementation of environmental management systems include poor access to reliable biodiversity information. Part of this problem lays in the lack of standardisation in taxonomic reference systems. Other parts of the problem concern the quality and completeness of taxonomic data sets, and the absence of an integrated access to taxonomic information.

PESI will contribute to the solution of this impediment by improving the European e-infrastructure through the strengthening of the respective scientific, social, political, technological, and information capacities in Europe, needed for a proper biodiversity assessment.

PESI is building a common web-based European biodiversity information infrastructure, by providing standardised and authoritative taxonomic information. For this purpose PESI defines and coordinates strategies to enhance the quality and reliability of European biodiversity information by integrating the infrastructural components of five major community networks on taxonomic indexing into a joint work programme and by developing an infrastructure of regional/national focal points (Figure 1).

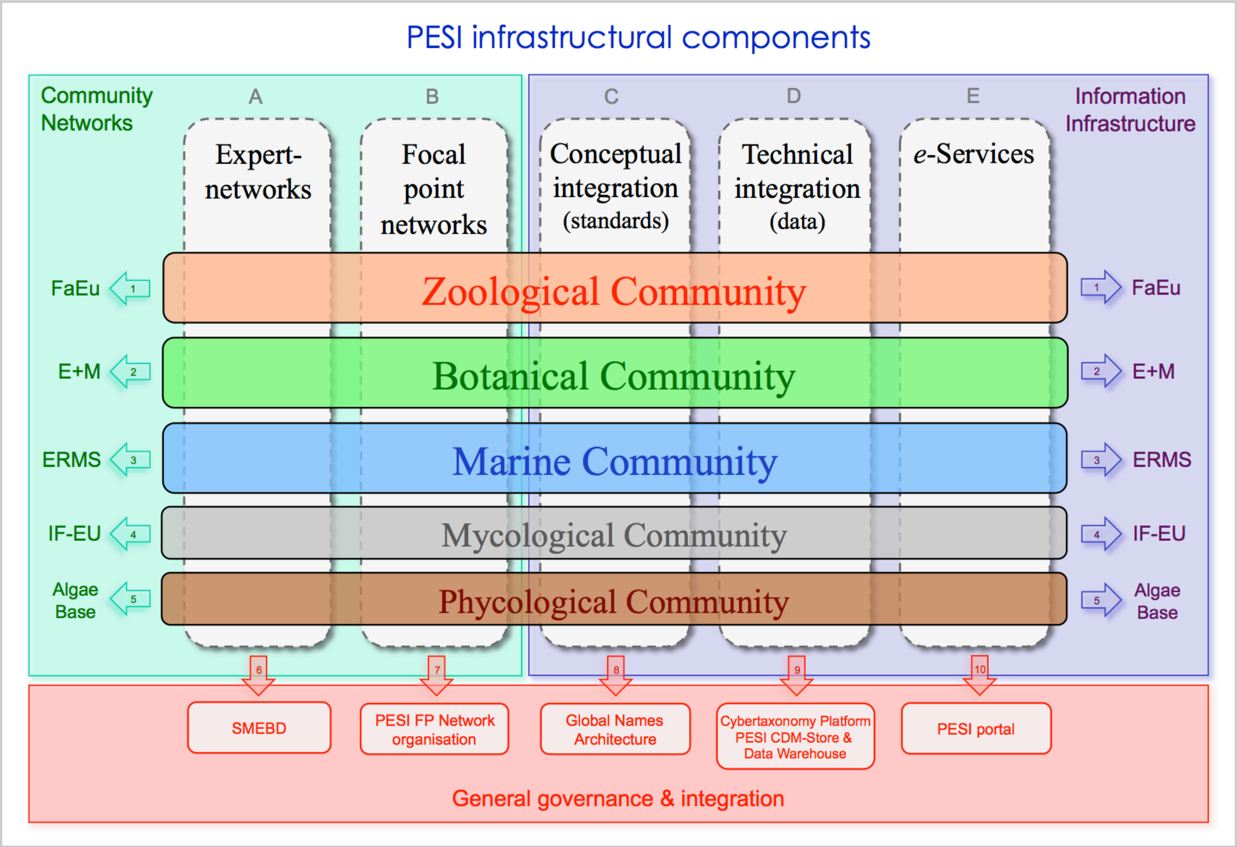

Figure 1: Five community networks (horizontal) integrated in five infrastructural components (vertical) in PESI. The numbering in the report will follow the listing in the above figure.

The durable objective of PESI will be the implementation of an authoritative taxonomic standard for Europe functioning as a Taxonomic Backbone. PESI contains several sustainable (social, conceptual, organisational, and technical) infrastructural components to support this objective.

This report will provide a roadmap on how the stability and continuity in the performance of these pan-European taxonomic services will or could be secured for a post PESI period.

This report will not aim to design mechanisms to keep control of the continuity of European electronic (taxonomic) biodiversity data resources and expertise networks, because this has already been surveyed in an earlier PESI deliverable (PESI D2.3)[1], however, it describes the concrete sustainability of the PESI associated and initiated infrastructural components.

As a preliminary conclusion one can say that at the moment a firm basis exists supporting the stability and continuity of the pan-European Taxonomic Backbone services as described in the below report. The issue of the long-term sustainability of the pan-European Taxonomic Backbone services will be further explored in the context of the LifeWatch construction plan developments continuing 2011.

LifeWatch is at this moment active with start-up activities to facilitate the beginning of the LifeWatch Construction Phase. In order to find solutions for complex scientific and societal problems, LifeWatch is in need for services that can tie correct taxonomic names to species or higher taxa attributes and ecosystem parameters. This will enable more profound insight and knowledge into the relation between diversity, composition, and the functioning of ecosystems as well as the services these systems can provide. Because of the standardization efforts within PESI comparative studies at European scales will be within reach. LifeWatch will thus build further upon PESI's services and use of data. Currently the PESI infrastructure plays a role in several of the National LifeWatch Programme proposals. If funding is acquired the National LifeWatch Programmes will be managed through distributed LifeWatch Centers. In several countries the PESI focal points are likely to take part in these LifeWatch Centers. Next to the actual PESI infrastructure, the large community that is actively involved in PESI is of interest for LifeWatch. PESI could ensure a strong role for taxonomists into the LifeWatch infrastructure. It is therefore very likely that through the LifeWatch infrastructure the functionality of PESI can be maintained and further developed in the years to come.

---

1 PESI D2.3 Continuity Plan

PESI sustainable components

European Expert Networks management

Introduction

A crucial part of the PESI project will be the involvement of the expert community to work collaboratively on the PESI tasks following common work formats. The PESI Expert Communities Common Infrastructure contains several sustainable components and coordination activities (see Fig. 2) of which the sustainability will be discussed below.

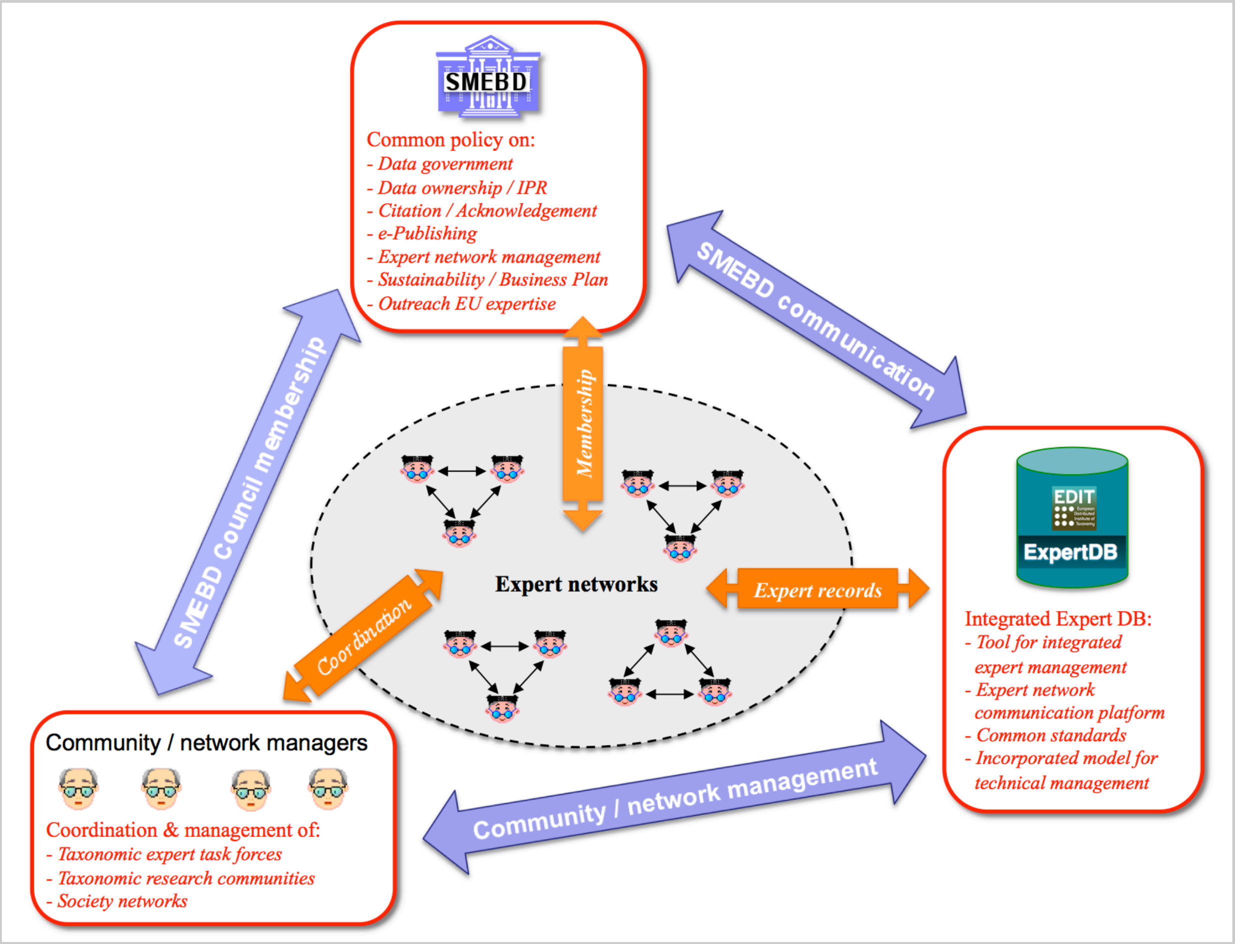

Figure 2: PESI Expert Communities Common Infrastructure outline.

Especially the supervision of the taxonomic communities contributing to the maintenance of the pan-European checklists is a vital component for the continuation of the updating process, taking care about keeping alive the pan-European checklists expert networks. Securing these positions in most cases requires an in-kind institutional support for a considerable period, occasionally funded by external resources.

Fauna Europaea (FaEu)

All ***Fauna Europaea***[2] (Fig.1 component A1) coordinating and management tasks are currently by the head of the Department of Biodiversity Informatics of the Zoological Museum Amsterdam (ZMA)[3] (Dr Yde de Jong), mostly as an in-kind (institutional) contribution, partly supported by external funds (like EDIT & PESI). This includes the management of the huge (around 500 people) expert community on updating their respective data sets.

Within 2011 all Fauna Europaea management tasks will gradually be handed over to the Museum für Naturkunde (MfN)[4] in Berlin (see Appendix 1). MfN will offer Fauna Europaea a sustainable position for an appropriate period taking care about the expert network coordination and data management, partly based on in-kind institutional commitment and partly by attracting additional funding.

Euro+Med PlantBase (E+M)

The scientific data management and coordination of ***Euro+Med PlantBase***[5] (Fig.1 component A2) is undertaken by the Euro+Med PlantBase Secretariat, currently located at the Botanischer Garten / Botanisches Museum Berlin-Dahlem (BGBM)[6]. All activities to maintain, update and expand the data in E+M are coordinated by the secretariat and its managing editor. These tasks comprise the coordination of the data contributions (by taxonomic editors, who are responsible for editing and updating of taxonomic groups; and by regional advisers, who are critically reviewing, correcting and filling the gaps in the database), supervision of data entries, data cleaning, contacting and helping new editors and regional advisors and integrating their contributions into the database. The most important task is to fill the taxonomic gaps, to reach full coverage of the European taxa in Euro+Med PlantBase as soon as possible. To fulfil this task, the secretariat is actively seeking for new data contributors and closely working together with present partners. It is equally important to update the existing families in E+M PlantBase by regular screening of the relevant literature and integrating new data. Only some of the present taxonomic editors can do that regularly, so that the E+M secretariat is helping them with this task as well.

To maintain and expand Euro+Med PlantBase as the standard information source for Euro-Mediterranean Plant diversity within PESI, the Euro+Med PlantBase secretariat will need to continue its activity on a regular basis. Until the end of 2011, in-house funds of the BGBM have already been made available. The BGBM will keep the engagement in maintaining the scientific data coordination and management of Euro+Med PlantBase at a basic level. For more substantial tasks (e.g. incorporation of additional large data sets), the BGBM will continue to seek for external funds and will actively contribute to the preparation of international and national project proposals.

---

2 http://www.faunaeur.org

3 http://www.science.uva.nl/zma

4 http://www.naturkundemuseum-berlin.de

5 http://www.emplantbase.org

6 http://www.bgbm.de

European Register of Marine Species (ERMS)

The ***European Register of Marine Species*** (ERMS)[7] (Fig.1 component A3) is an authoritative taxonomic list of species occurring in the European marine environment, defined as up to the strandline or splash zone above the high tide mark and down to 0.5 (psu, ppt) salinity in estuaries. The register was actively maintained and daily updated in the framework of the MarBEF EU Network of Excellence by a board of taxonomic editors, which are world experts on the taxonomy of their relevant taxa. The register is now part of the World register of marine species WoRMS[8], together with about 35 global or regional species lists, which are all maintained by specialized taxonomic experts or groups of experts. The world register now has more than 200.000 species and involves more than 170 taxonomic editors.

The activities on the management of the network of marine taxonomists supporting the continuing updating of the ERMS is secured by VLIZ[9], connected to their ongoing involvement on maintaining the marine taxonomic and biogeographic data services, including WoRMS, VLIMAR[10], and EUROBIS[11], supported by the Flemish government and some running and anticipated EC FP7 projects (like 4D4Life[12] and EcoBOS). VLIZ and SMEBD (see below) work constantly with the taxonomic experts to further complete and keep the content of the ERMS database up to date.

WoRMS has been proposed as the taxonomic data standard for IOC's IODE network[13] of ocean data centres.

Europe Index of Fungi (IF-EU)

The short time management of the ***Europe Index of Fungi*** (IF-EU)[14] (Fig.1 component A4) is secured by CABI[15], but it was recently agreed in the CABI management team that a proposal will be written within the next nine months to coordinate national checklist managers to publish their fungal data in a way it can be harvested centrally (perhaps using the IPT[16]) to further populate the European checklist portal.

AlgaeBase Europe

For ***AlgaeBase Europe*** and AlgaeBase[17] (Fig.1 component A5) professor M.D. Guiry will act as data manager and co-ordinator until 27 July 2014 on a volunteer base. No funding is available for programming, so no changes can be made to the data management system during this period. The

7 http://www.marbef.org/data/erms.php
8 http://www.marinespecies.org
9 http://www.vliz.be
10 http://www.vliz.be/vmdcdata/vlimar
11 http://www.marbef.org/data/eurobis.php
12 http://www.4d4life.eu
13 http://www.iode.org
14 http://pesi.indexfungorum.org
15 http://www.cabi.org
16 http://www.gbif.org/informatics/infrastructure/publishing
17 http://www.algaebase.org

database data will rapidly become useless unless they are maintained; therefore some mechanism to protect the AlgaeBase investment to date is required urgently.

Society for the Management of Electronic Biodiversity Data SMEBD)

The ***Society for the Management of Electronic Biodiversity Data*** (SMEBD)[18] (Fig.1 component A6), as an independent organisation, covers the organisation of the European taxonomic work force into a common management structure (Fig. 3). SMEBD is an active organisation having yearly meetings and a secretariat, last years funded by EDIT and PESI. To support the yearly cost, to keep the secretarial function in place, an amount of around 7k Euro is needed, which will be obtained via the donations of users requesting for data licences. Sponsoring for the annual meeting costs will very likely be received via the recently started EC FP7 BioFresh[19] project, currently providing SMEBD's chair position (Hendrik Segers). In addition, some funding is expected via the OpenUp![20] project, which links parts of its activities, especially those related to zoology, through SMEBD.

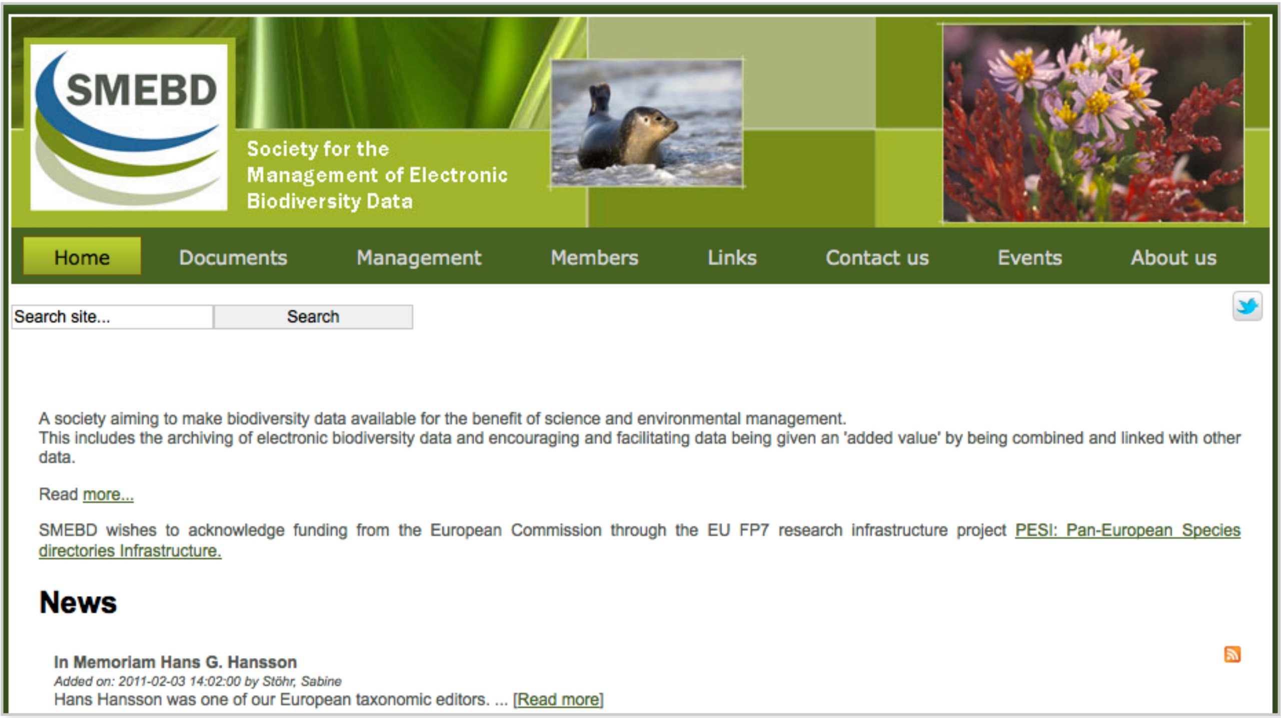

Figure 3: Fragment of the SMEBD home page.

EDIT Expert DB

The common European taxonomic experts register system and expert networks management tool, build in collaboration between EDIT WP2 and PESI, known as the ***EDIT Expert NET***[21] (Fig. 4) is supposed to be maintained as an expertise service after the EDIT project life span. Currently 4516 experts are included in the in the backend-database.

The University of Copenhagen / Natural History Museum of Denmark will host the EditExpertNet

18 http://www.smebd.eu
19 http://www.freshwaterbiodiversity.eu
20 http://open-up.eu
21 http://www.editexpertnet.org

database and its front-end functions (TaxNet) for at least the rest of 2011. Security updates are not necessary in the first two years after the project because of the selected, sustainable (Linux & Drupal) systems. So far no network coordinator and/or data manager will be available to take care after the PESI ending, however, active lobbying towards the new CETAF consortium exists to highlight the relevance of EditExpertNet as a common expert community service for Europe.

Successful pilots like the 'TaxNet for Dipterists' to use the social networking for discovering and recruitment of experts as described in PESI D2.1 will end with the termination of PESI.

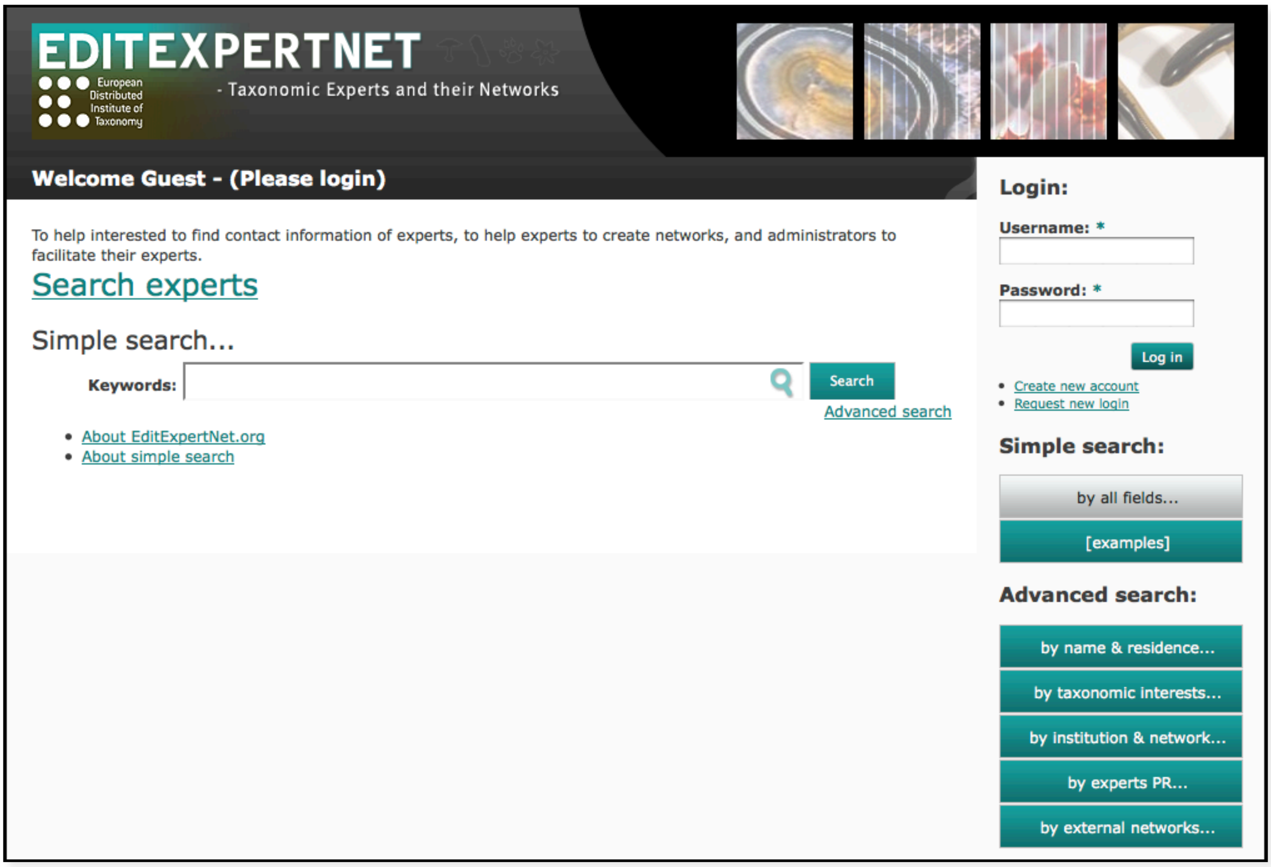

Figure 4: EditExpertNet search interface.

European Focal Points Networks

Introduction

The network of national and regional Focal Point networks, not only assures the efficient access to local taxonomic expertise and resources, but also takes care about the synergistic promotions of taxonomic standards throughout Europe (see Fig. 5).

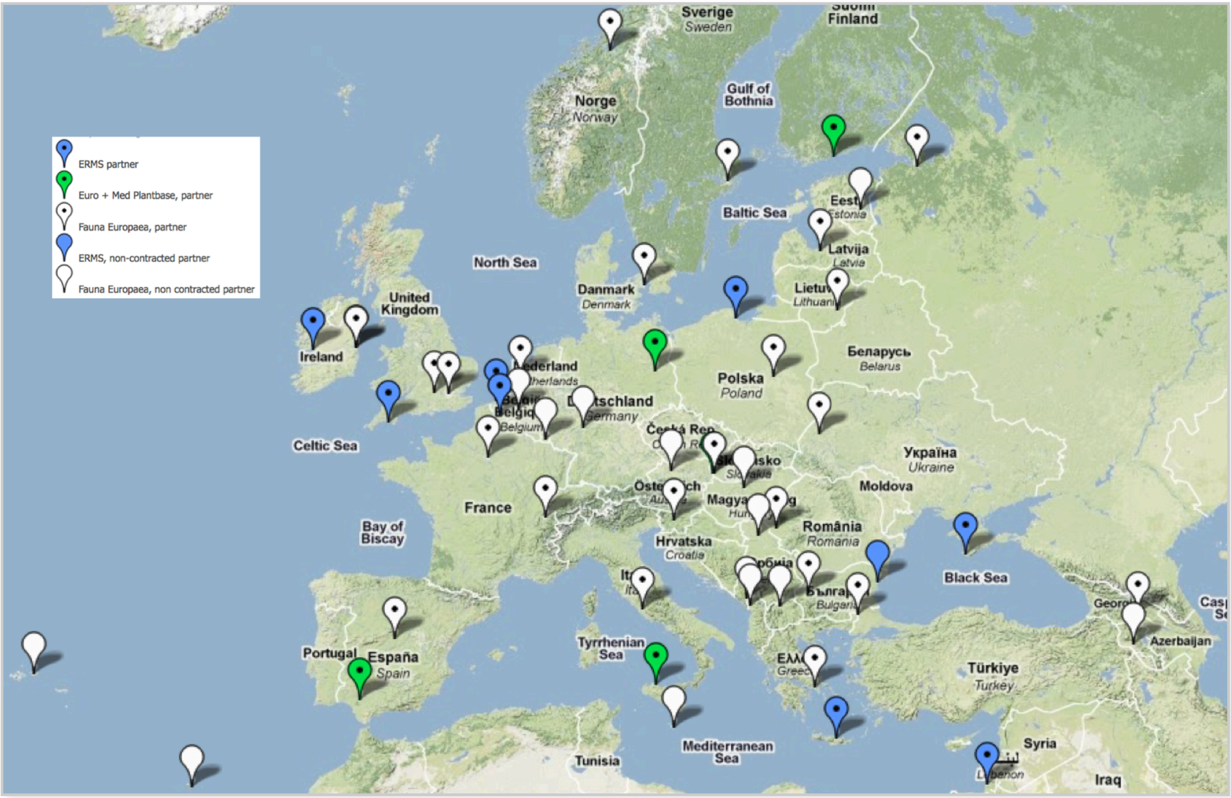

Figure 5: PESI Focal Points Networks.

Focal Points continuity plan and LifeWatch connection

The drafting of a continuity plan, to support the future Focal Point activities and the establishment of a Focal Point organisation (Fig.1 component B7), is part of the PESI Focal Point Work Plan[22] and will not be further addressed here. The results of this assessment will become available via the PESI Focal Points Wiki[23]. This includes guidelines to support National Focal Points as 'Clearing Houses' on establishing national taxonomic standards for collaboration in international (global or regional) and thematic programs.

In this context, collaboratively with the EC FP7 LifeWatch[24] management, a concerted approach is drawn to include National Focal Points into their subsequent National LifeWatch Programs, as a contribution to the LifeWatch construction phase, which will be further exploited continuing 2011. These National LifeWatch Programs are managed by distributed (national) LifeWatch Centres. Integrating the PESI National Focal Points into the (national) LifeWatch Centres will ensure a strong link between the PESI functionality and services and the further development and use

---

22 PESI D3.1 Focal Point Work Plan
23 http://pesifocalpointhandbook.wikispaces.com
24 http://www.lifewatch.eu

thereof in the LifeWatch Construction Phase. From a LifeWatch point of view it would be a valuable contribution to the LifeWatch Construction Phase if a country would propose to coordinate the European PESI Focal Points. In turn, from a PESI point of view this would ensure some coordination into the activities and contributions of the European PESI Focal Points. Several countries are considering including such a proposal in their National LifeWatch Program for the LifeWatch Construction.

As an active community, the contributors to the checklists within PESI are an important user community for LifeWatch. The PESI National Focal Point Network is a potential structure to communicate between LifeWatch and this community. LifeWatch will have a Service Center with a front office that has an objective to engage with the user communities and adjust the infrastructure and services to the user needs. It is envisioned that the LifeWatch Service Center hosted by Italy will engage with the PESI National Focal Point Network. Some reasons for this engagement are that LifeWatch should be able to locate and engage with specialist in Biodiversity throughout Europe. An easy engagement would also mean that contributors within PESI would obtain ample opportunities to contribute in creating European wide research strategies for the role of taxonomy within ecosystem research.

Further outreach (in space and time)

PESI started the geographic expansion of the European expertise networks to eventually cover the entire Western Palaearctic biogeographic region. As an important first step, the cooperation with partners from the Caucasus and Balkan was intensified to draft proposals to set up regional Focal Point networks in the Caucasus and at the Balkan, to cover taxonomic gaps in the pan-European checklists.

As an example the context of the 'Pan-Caucasian Plant Biodiversity Initiative' meeting at Berlin (26-30 January 2009) was used to initiate the set up of networks of botanical and zoological focal points in the Caucasus and to discuss a draft work plan. After both networks have been established (Russia, Georgia, Armenia, Azerbaijan) the proposal 'Network for Biodiversity Research in the Caucasus (NBRC)' was drafted and submitted as part of the first INCO call.

Analogous to this Caucasus proposal, next INCO calls are planned to trigger the drafting of a proposal for the Balkan; either focusing on a certain topic (like sub terrestrial organisms) or on a more common subject related to taxonomic capacity building and/or taxonomic indexing in general.

In addition, the liaisons towards North African taxonomists, who are strengthening their regional coordination and cooperation networks, was intensified by participating into the first conference of the *Association Tunisienne de Taxonomie* (ATUTAX), as well as the meeting of the North African taxonomic network BioNET-NAFRINET. Both meetings were held in Tunis on April 23 and April 24-25, 2010, respectively. We hope these participations will ease potential future collaborations on extending the pan-European checklists scope.

Last but not least, as part of this PESI Business Plan all individual Focal Points have been requested to contribute following a standard questionnaire, highlighting their basic PESI project experiences and future expectations. The results (so far received) are given as Appendices 2-20.

Euro-based nomenclators and GSDs/RSDs

Introduction

Because the correct use of names and names relationships is essential for biodiversity management, the availability of taxonomically validated standardised nomenclatures are fundamental for biological *e*-infrastructures. PESI is the next step in integrating and securing taxonomically authoritative species name registers that underpin the management of biodiversity in Europe, providing the essential taxonomic meta-data standards for easy access to, and interoperability of, all kinds of biodiversity data (Fig.6).

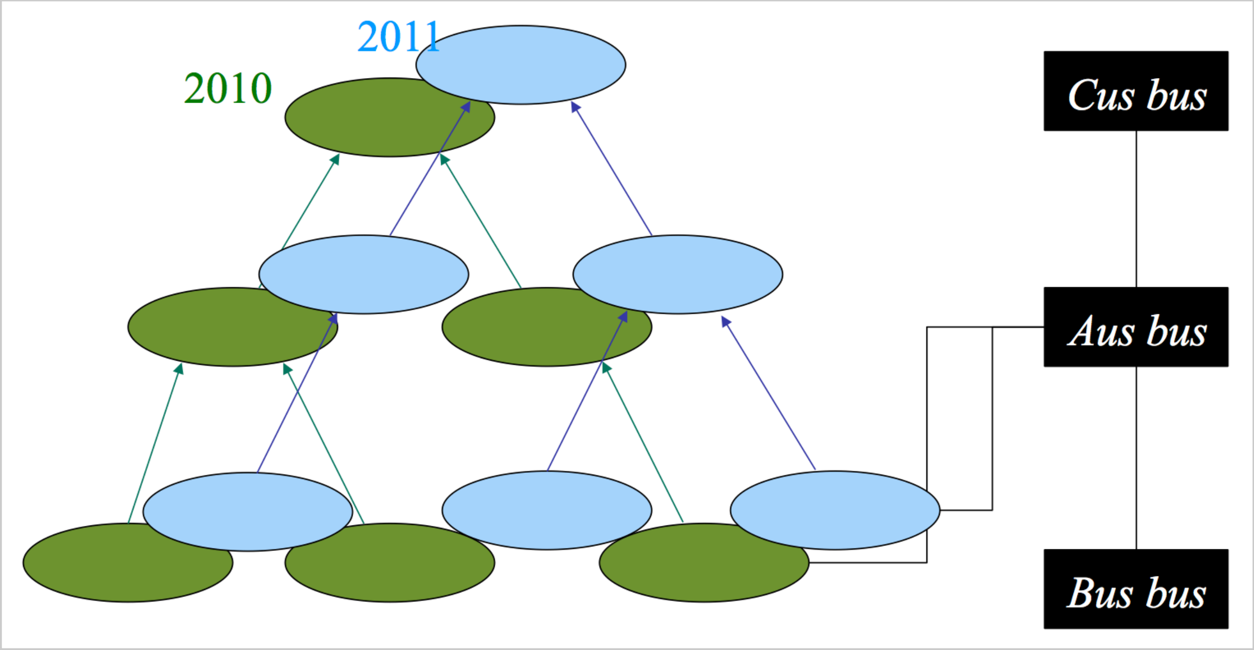

Figure 6: Two important features of a taxonomic backbone: (1) connecting different uses of the same name for multiple classifications and (2) providing name-name relationships of species names (homotypic or objective synonymy).

PESI supports the implementation of taxonomic standards in several ways. Firstly by expanding the network of involved end-users adopting the pan-European checklists as their taxonomic point of reference, which addresses scientific communities, applied biodiversity researchers, scientific institutes, and national biodiversity networks and services as well as the general public. Secondly by actively scrutinising important species lists, like the European prioritised species lists, by comparing these to the PESI taxonomic standard and then disseminating the outcomes through the PESI portal. Thirdly by linking PESI with relevant global information infrastructures, developing common solutions for creating a complete and integrated taxonomic framework for all names. For this purpose PESI will not only deliver the integrated four main all-taxon registers, representing the highest level of expert-validated taxonomic reference lists in Europe (FaEu, E+M, ERMS and IF-EU) in one integrated backbone structure, but also work in close collaboration with EU based nomenclators (IPNI, ZooBank, Index Fungorum and AlgaeBase) and the network of EU-based Global Species Databases (GSDs) to refine the concept of a taxonomic backbone, providing an efficient access and interlinking of biological information into an integrated, global, virtual (bench) architecture. In this respect PESI supports international efforts on the development of a

Global Names Architecture (GNA)[25] (Fig.1 component C8) and exploits the connectivity of the 'PESI data warehouse' to the *Catalogue of Life* as the so-called 'European Hub', for instance as part of the EC FP7 i4Life project[26].

Technical and conceptual contributions to the Global Names Architecture developments have been discussed in subsequent PESI WP4 reports, like PESI D4.4[27], and will not be recapitulated here. Likewise the roles of nomenclators and Global species Databases (GSDs) in the global framework of taxonomic information services have already been considered in earlier PESI WP4 and WP5 reports (PESI D4.2[28] & PESI D5.1[29]). This PESI D1.3 report will focus on the sustainability of the involved information infrastructures and services.

The sustainability of the EU-based nomenclators Index Fungorum and AlgaeBase, will be addressed in other chapters, because they also contribute to the establishment of European lists of fungi and freshwater algae (particular desmids) respectively.

International Plant Names Index (IPNI)

RBG Kew has maintained IPNI with partners (The Harvard University Herbaria and the Australian National Herbarium) since its inception (1996) and continues to employ a dedicated team of five editors and between half and full time person across the partnership addressing technical issues. The partnership is actively planning future developments congruent with the hotly anticipated Global Names Architecture though additional funding will be required. IPNI will seek to develop IPNI in-line with the proposed GNA and therefore be available to projects such as LifeWatch.

IPNI provides the raw factual nomenclatural data for many online taxonomic projects. Provision of IPNI LSIDs in products such as The Plant List (TPL) helps to embed IPNI data in such projects and their derivatives. IPNI is therefore a fundamental building block of many plant e-taxonomy projects. Recent workflows have been adopted to support electronic publication of nomenclatural acts.

ZooBank

The ZooBank Committee, which runs under the supervision of the International Commission for Zoological Nomenclature (ICZN), determines the business plan and policies for Zoobank. Zoobank operates within a component of the Global Names Architecture (GNA) called Global Names Usage Bank (GNUB). The development of GNA is largely secured by GBIF, funding for developing the ZooBank part of GNUB is being actively sought and is likely to be forthcoming. This could partly be covered by asking a registration fee for submitting new zoological names ($20), an additional $2M grant from NSF to further develop GNA (especially GNUB) is pending.

European-based GSDs/RSDs

A concise plan addressing the possible issues for GSD sustainability and evaluating the cost of maintaining Global or a Regional Systematic Databases (GSDs/RSDs) on the long-term has been

25 http://www.gbif.org/informatics/name-services/global-names-architecture
26 http://www.i4life.eu
27 PESI D4.4 Contributions to the Global Names Architecture
28 PESI D4.2 Nomenclators Role Report
29 PESI D5.1 Sustainability Plan EU-based GSDs

drafted as PESI Deliverable D5.4[30]. Apart from new ideas about feedback mechanisms between GSDs and major Taxonomic database initiatives, PESI D5.4 also provides an easy-to-use tool allowing evaluating the financial costs of maintenance for any individual database through a formula to fill in with a few parameters. For further discussion GSD sustainability we refer to that document.

30 PESI D5.4 GSD maintenance Cost

Pan-European checklists data management systems

Introduction

To allow for the proper maintenance and updating of the pan-European checklists data, the hosting of the associated data management systems needs to be secured, as well as competent staff, to carry out relevant data management tasks, being employed.

Recently, checklists custodians of the pan-European checklists have delivered the last versions for integration into the PESI Taxonomic Backbone. The contributions from the involved databases into the PESI Data Warehouse so far are shown in Fig. 7.

Dataset	Above species	Species	Below species	All Ranks
FaEu *all (accepted / valid & not accepted / valid)*	42018	172466	19807	234291
FaEu *all + orginal combinations*	42018	233192	26184	301394
FaEu *only accepted / valid*	34300	131672	14234	180206
Euro+Med PlantBase *all*	5173	75934	41450	122557
Euro+Med PlantBase *only accepted / valid*	2371	25830	10116	38317
ERMS *all*	19058	43236	1322	63616
ERMS *only accepted / valid*	17565	33685	769	52019
Europe Index of Fungi *all*	3969	22384	0	26353
Europe Index of Fungi *only accepted / valid*	3969	22384	0	26353
FaEu-ERMS *overlap all*	1923	1806	20	3749
FaEu-ERMS *overlap only accepted / valid*	1859	1664	20	3543
IF-ERMS *overlap all*	389	217	0	606
IF-ERMS *overlap only accepted / valid*	386	207	0	593
E+M-ERMS *overlap all*	45	32	2	79
E+M-ERMS *overlap only accepted / valid*	43	27	1	71
**PESI-DWH *all***	**67861**	**311965**	**62557**	**442383**
**PESI-DWH *only accepted / valid***	**55885**	**211638**	**25097**	**292620**

Figure 7: Statistics of the pan-European checklist's and PESI Data Warehouse integration efforts

Fauna Europaea (FaEu)

The ***Fauna Europaea*** data management tools and web portal (Fig. 1 component D1) have been maintained based on ZMA Biodiversity Informatics Department commitment, financially supported by the University of Amsterdam. Hosting of the databases is secured in Amsterdam at SARA[31] until at least the end of this year.

A migration of the Fauna Europaea database to a new data management environment linked to the PESI CDM-Store[32] and the EDIT Taxonomic Editor Tool[33] is supposed to be ready by the end of 2011. This move is already anticipated in PESI (meaning that FaEu is already implemented into the CDM-store of the Cybertaxonomy Platform), but firstly the relevant data management tools need to be adapted according to the zoological standards and practise. For this purpose a few pilots will be set up for testing using a selection of Fauna Europaea taxonomic sectors.

Efforts on further developing the Fauna Europaea data management system towards an advanced virtual workbench is planned in close collaboration between MfN and BGBM.

31 http://www.sara.nl
32 http://wp5.e-taxonomy.eu/blog/node/218
33 http://wp5.e-taxonomy.eu/taxeditor

Euro+Med PlantBase (E+M)

Europe and the Mediterranean area is one of two regional focal areas of research of the BGBM. The technical hosting of ***Euro+Med PlantBase*** (Fig. 1 component D2) is therefore part of the institutional science strategy and will be maintained beyond funded project phases. Technical hosting includes:

- Providing the necessary storage and computing capacities for running both the SQL-Server and CDM-Store instances of Euro+Med.
- Regular updates of server-software components.
- Running the Euro+Med portal and minor bugfixes.
- Creating data exports for related scientific initiatives.
- Participating in international standardisation efforts to ensure interoperability of the Euro+Med data services.

The BGBM will perform this function for at least 5 years. However, more substantial modifications of the software platform (e.g. due to significant changes of underlying data standards) will probably require additional funding. If necessary, the BGBM will continue to support the development of new project proposals at both international and national level.

Euro+Med PlantBase provides the taxonomic backbone for the Euro-Mediterranean Biodiversity of Vascular Plants. This is an excellent starting point for a wide range of expansion possibilities:

- taxonomic widening: integration of mosses and liverworts into Euro+Med PlantBase;
- geographic widening: completion of Caucasus , expansion into Middle East;
- data type widening: integration of plant images, descriptions, keys, specimen images (towards an E+M - eFlora)
- service widening: integration with national biodiversity portals in Europe (FloraWeb, TelaBotanica, Anthos, Flora Croatica Database etc.), so that PESI/Euro+Med will serve as a gateway to the smaller databases with higher resolution (e.g. Grid maps – FloraWeb[Germany]; BSBI [Great Britain and Ireland], departements – TelaBotanica[France]; dotmaps/provinces – Anthos (Iberian Peninsula)); integration with BHL, collection services.
- Integration with services provided by the emerging international biodiversity informatics infrastructures (e.g. BHL, BioCASE, etc.)
- Implementation of an advance annotation system allowing for enrichment of taxonomic information at all levels of granularity. Version control for annotated content.
- Processing of taxonomic concepts and transmission of factual data between concepts.

European Register of Marine Species (ERMS)

The maintenances of the ***European Register of Marine Species*** database system (Fig. 1 component D3) will secured at VLIZ. VLIZ developed and hosts the World register databases, it's editing interfaces and it's web services. VLIZ can guarantee to further maintain and update the WORMS system, and thus also the ERMS system.

Europe Index of Fungi (IF-EU)

For the ***Europe Index of Fungi*** the existing database and web site (Fig. 1 component D4) is secure and managed until 27th August 2012 (when the current custodian retires). Also after that date at least the hosting of the service will be secured for a certain period when no alternative hosting could be arranged, although new data entering could be reduced.

AlgaeBase Europe

The future of ***AlgaeBase*** is rather uncertain due to complications that have arisen recently with the University of Galway (NUI Galway)[34] and with the company developing the software and hosting the database (Fig. 1 component D5). In view of this an Advisory Board is set up to consider proposals for the continuance of *AlgaeBase*.

NUI Galway has undertaken to host AlgaeBase and AlgaeBase Europe until 27 July 2014, when funding will be required. No funding is available to replace the server and no funding is available to enhance or accelerate data entry or updating at the moment. A system of on-line identification keys has been programmed into AlgaeBase as well, but currently no funds exist to enter the data the implement on-line identification keys.

[34] http://www.nuigalway.ie

Taxonomic Backbone e-Infrastructure and e-Services

PESI CDM-store and PESI Data Warehouse

Hosting and technical availability of the data system

The PESI technical platform is based and depending on the availability of the EDIT Platform for Cybertaxonomy and in particular on its core CDM store infrastructure and interface layers (Fig. 1 component D9). The BGBM will continue at least for a period of 5 years to maintain and develop this infrastructure. Platform-related activities include i) the hosting of the central PESI data store and data warehouse structures, ii) coordinating international development efforts for the EDIT platform, iii) convening and chairing the EDIT ISTC (Information Science and Technology Committee), iv) promotion of the platform for developer and user communities, v) documentation of the core platform components, and vi) fundraising for developments which can not be covered by the basic maintenance activities of the BGBM. In a similar way, other European institutions take responsibility for other aspects of the EDIT platform such as descriptive components (LIS, Paris), the Geo-Platform (RMCA, Tervuren).

The Platform and the CDM technology are a central component of the BGBM's biodiversity informatics strategy, and new projects and project applications are in many cases strongly related to this base technology. New platform-related projects and initiatives include for example ViBRANT (lead NHML, with several EDIT and PESI partners), i4Life (with several EDIT partners), GBIF and several pending and forthcoming applications to the German research council (DFG).

Sustainability of the management

Management of the merging process and creation of the PESI DWH will be carried out by the BGBM. We are assuming that agreed structures (e.g. data models of participating checklists and export structure of the PESI data warehouse) are kept stable. If changes occur (e.g. through additional or changed concepts in the source checklists), additional resources for the adaption of the merging process have to be found. The BGBM will actively support this process to ensure that the participating checklists are appropriately represented in the PESI portal.

Potential follow-up projects and initiatives

The EDIT/PESI infrastructure offers for the first time a technical framework for taxonomic data processing, which is based on a common European information model for this purpose. As a consequence, a growing number of European taxonomic institutions joined the software development and are taking responsibility for well-defined aspects of the platform. This approach led already to a powerful software suite including tools for data storage (used by PESI for example), advanced data editing tools, and modules for data publication in the web and on paper. However, significant additional developments will have to be addressed in new projects and initiatives. Such developments include

- Implementation of a fully featured web-editing environment for CDM-stores to be used in the context of distributed editing of PESI data. Editing software will include specialized data management tools for efficient maintenance of large numbers of homogeneous data records in tabular form (e.g. name lists, reference lists, author lists, distribution records, etc.).

- Annotation workflows: The EDIT/PESI platform has interfaces for adding annotations to every object in the system, which could be used for all kinds of content enrichment (e.g. Portal User comments and corrections, field observations, etc.). In the context of a new project, this layer could be equipped with configurable User-interfaces supporting the generation of annotations for different User communities and use cases.
- Workflows: The EDIT/PESI data has a rich service layer allowing for machine access to all classes in the CDM. This layer is used for example to connect the EDIT portals software to an underlying instance of the CDM store. Assuming that the platform will play a prominent role in the context of workflow environments in LifeWatch, the EDIT service layer has to be hardened and appropriately described in a machine readable way. This will open up PESI to a completely new range of applications requiring access to a stable and agreed taxonomic backbone.
- Integration and Ontologies: we foresee that the PESI portal and services will increasingly serve as an information broker for a range of related services and data repositories and an integrated access layer to be implemented. This integrated view on distributed biodiversity information infrastructures require a machine-readable representation of terminologies and relations between concepts used in different knowledge domains. The setup of a framework for the development and maintenance of an ontology will be the basis for the integration of PESI services with for example specimen and observation searches (e.g. BioCASE), access to literature references (e.g. BHL and BHL-Europe), DNA (e.g. DNA-Bank-Network).

PESI Portal

A common user web-interface for searching the PESI Data Warehouse is developed as part of the ***PESI Portal***[35] (Fig. 1 component E10). hosted by VLIZ. Apart from the PESI Data Warehouse, also additional information files, like the Focal Points taxonomic expertise and resources database and the commons names are linked to the PESI Portal. The PESI portal not only includes user services searching for taxonomic names, but also web-services facilitating names validation and advanced searches, like those on Europe's prioritised species and the Taxon Match Tool.

We not only need to keep the PESI Portal running (housing of the data systems), and available to it's users, but also need to maintain the system up-to-date, to keep performing up to the expectations of it's users. Without such an updating program, data hosted in the PESI portal will become outdated rather fast. In addition, it is realistic to state that the community will soon have additional requests requiring further PESI Portal improvements.

The PESI Portal is developed and hosted by VLIZ at the moment and is therefore the best candidate to keep maintaining the system. VLIZ is prepared to do so for a five years period after the project. VLIZ offers such web services to many other organizations, especially for marine taxonomy (WoRMS) and geographic (VLIMAR) and biogeographic databases (EUROBIS / EMODNET), therefore taking care about the PESI Portal fits within the VLIZ core activities and strategic plan. This implies keeping the database server and the web server running, making backups, installing mandatory operating system and middleware updates, protecting the data portal from hacks, moving to new hardware as needed, and so on.

[35] http://www.eu-nomen.eu/portal

PESI Portal service & LifeWatch

Even more guarantees on a long-term PESI Portal functioning can be offered when the LifeWatch-Flanders proposal will be granted. The LifeWatch Service Center is the front office for LifeWatch. It shall be developed and hosted by Italy. One of the objectives of the LifeWatch Service Center is to ensure and steer the infrastructure to the user's needs and desires. LifeWatch has the vision to construct a user centric high-end services infrastructure. To reach this goal, close contact with end-users is therefore crucial. When a community that goes well beyond the PESI infrastructure itself uses extended services, it is essential that a constant flow of information is ensured. Integration of the PESI portal into LifeWatch is a logical step from this point of view. Maintaining and developing the PESI Portal should hence be done in close cooperation with the LifeWatch Service Center.

PESI general outreach and Global Names Architecture contributions

Several roadmap reports have been drafted in collaboration with for instance EDIT[36] and GBIF[37] to show how PESI c/would be linked with global information infrastructures to contribute to a sustainable outreach of the PESI information services. Below a follow-up of these reports evaluating the state of affairs at the end of the PESI project, considering some recent developments on cross-linking the PESI (European) Taxonomic Backbone towards other biodiversity information systems as part of some newly funded EC FP7 projects.

PESI Taxonomic Backbone preparations

At the end of January 2011, last versions of the pan-European checklists are delivered by the checklists custodians for import into EDIT's Cybertaxonomy Platform[38] – Common Data Model[39] store (PESI-CDM store) (see statistics Fig. 7 and ❶ in Fig. 8). After import into the CDM store these checklists will be integration into the PESI Data Warehouse (see ❷ in Fig. 8). This process was completed around the mid of March and delivered the final version of the PESI European Taxonomic Backbone, as a formal output of the PESI project, which also will be implemented into the PESI portal (see ❸ in Fig. 8).

GNA cross-linking progress

The PESI Data Warehouse will be exported as a *Darwin Core Archive* (DwC-A) file[40] and registered at GBIF's metadata registry (GBRDS)[41] (see ❹ in Fig. 8). The EDIT Platform for Cybertaxonomy will install the appropriate services to allow harvesting. Also an extended meta-data document, describing the respective checklists meta-data profile, will be included in the package with the DwC-A file for incorporation into GBIF's meta-data repositories.

So far the only deviation from the described process is that the integration of the projected AlgaeBase[42] contribution will be postponed until later this year, because the provisioned checklist of European Desmids could not be finished on time. In addition, it was decided during the last

36 http://www.e-taxonomy.eu/node/728
37 http://www.e-taxonomy.eu/node/452
38 http://wp5.e-taxonomy.eu
39 http://dev.e-taxonomy.eu/trac/wiki/CommonDataModel
40 http://code.google.com/p/gbif-ecat/wiki/DwCArchive
41 http://www.gbif.org/informatics/infrastructure/discovering
42 http://www.algaebase.org

SMEBD council and PESI SC meetings in Paris that a separate MoU between GBIF and PESI on sharing the integrated PESI data set should be drafted.

Individual internationalisation of checklists

Apart from the above described cross-linking of the PESI Taxonomic Backbone, also the individual pan-European checklists continue their own dissemination services with information infrastructures of which some are outside Europe. Fauna Europaea -for instance- issued around 70 licences for use and shares data with outer European information services like *iBol*[43] and *uBio*[44] (see ❺ in Fig. 8). Similarly ERMS issued around 30 licences for use and has a significant international outreach via *WoRMS*.

In addition, for their international outreach, both WoRMS and Index Fungorum are also partners within the *Catalogue of Life*[45] / *4D4Life*[46] initiatives (not shown in Fig. 8). In this context Fauna Europaea will contribute to the EC FP7 freshwater monitoring project *BioFresh*[47] by providing the taxonomic reference list for animals (not shown in Fig. 8).

Dissemination of PESI into new FP7 projects

PESI provides key contributions to the recently started EC FP7 projects *ViBRANT*[48] and *i4Life*[49]. In ViBRANT, PESI will contribute to the set-up of an architecture supporting the access to, and exchange of, associated vocabulary services, like the PESI CDM-store and GBIF's Vocabulary Service (see ❻ in Fig. 8). This facilitates the management and dissemination of standardised ontologies for Scratchpads users. In i4Life the connectivity of the 'PESI data warehouse' to the Catalogue of Life will be further exploited as the so-called 'European Hub' of this global taxonomic indexing initiative (see ❼ in Fig. 8).

More extended integration of the PESI Taxonomic Backbone into the European scientific e-domain developments is scheduled when the negotiations on funding the *BioVel* project will successfully end. For BioVel, establishing a virtual biodiversity laboratory, PESI will provide a liaison to the taxonomic research community, including support on the definition of the relevant taxonomic work flows, as well as a connection to the PESI information services (see ❽ in Fig. 8).

As a contribution to the *OpenUp!*[50] project, *Opening Up the Natural History Heritage for Europeana*, PESI will provide the vernaculars names of animal species (see ❾ in Fig. 8). The results of OpenUp! will be distributed through the *Europeana*[51] portal using the BioCASE[52] facilities (wrappers, etc.).

Some PESI contributions to recently submitted proposals are pending depending the results of the evaluation process. PESI is for instance supporting some of the LifeWatch-initiated projects, like

43 http://ibol.org
44 http://www.ubio.org
45 http://www.catalogueoflife.org
46 http://www.4d4life.eu
47 http://www.freshwaterbiodiversity.eu
48 http://vbrant.eu
49 http://www.i4life.eu
50 http://open-up.eu
51 http://www.europeana.eu/portal
52 http://www.biocase.org

EcoBOS and *iMarine*. In EcoBOS, which builds a network of marine research stations for biodiversity monitoring and study, PESI will provide a Clearing House to support the continuing efforts on building a taxonomic reference list on marine species. PESI will participate in the iMarine project by exploiting the implementation of components of the Virtual Research Environments developed by the *D4Science-II* project. This participation will focus on the application of AquaMaps, especially within the marine domain (through VLIZ).

All above-mentioned projects have an (mixed) outreach inside and outside Europe (shown by block arrows in Fig. 8).

Advanced PESI portal services

A special case of cross-linking to be established by PESI, advancing the PESI web-portal services, will be the implementation of the Global Names Index (GNI)[53] into the name search routines to optimise the name matching for users and to provide relevant forwarding to other taxonomic information services in case names are absent in PESI (see ❿ in Fig. 18).

[53] http://gni.globalnames.org

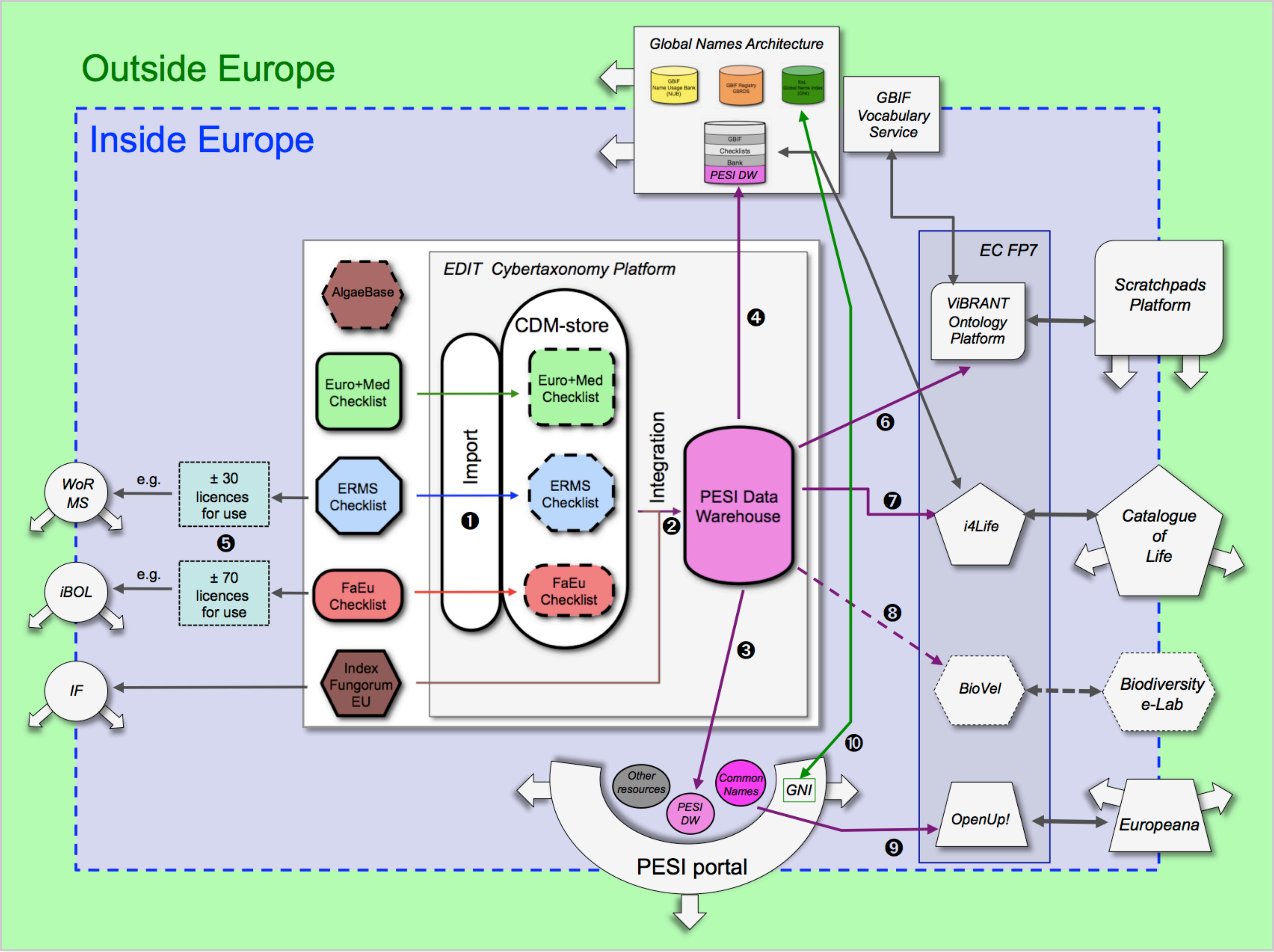

Figure 8: Overview of the PESI cross-linking with major global information infrastructures and EC FP7 programs having a dissemination inside and outside Europe

Appendices

Appendix 1 — Letter of UvA to MfN on migrating the hosting of FaEu

FNWI Algemeen UIT - 1850

UNIVERSITEIT VAN AMSTERDAM

Faculty of Science
Faculty Administration

Science Park 904
1098 XH Amsterdam
P.O. Box 94216
1090 GE Amsterdam

T 020 525 7678
F 020 525 7675
www.science.uva.nl

Museum für Naturkunde
Leibniz Institute for Research on Evolution and Biodiversity
Dr. Cristoph L. Häuser
The Directorate
Invalidenstr. 43
10115 Berlin
Germany

Date
15 maart 2011

Subject
Fauna Europaea

Enclosed

Your reference

Our reference
FNWI_U11 / 1850

Dear Dr. Häuser,

I am pleased that your university is willing to take over and to continue the services of Fauna Europaea (http://www.faunaeur.org/). Dr. Yde de Jong is preparing the transition to your institute. To this end he will continue to operate the service and the transition up to the end of this year. The transition also implies that we will secure costs for hosting the database and portal services in 2011. We expect that your institute will cover the costs from 2012 on.

In the spirit of this European cooperation I hope that your institute will be in the position to continue and expand the Fauna Europaea services.

Yours sincerely,

R. Rust

Drs. R. Rust,
Director of Finance

Appendix 2 — Individual Focal Point Sustainability Plan — NHM — Partner 4

Institute: Natural History Museum

Contact person: Charles Hussey (until 31 December 2011)

1. Please give a short description of your sustainability plan in view of your local/national situation

The Natural History maintains a Species Dictionary which is a compilation of over 200 checklist which together represent a master list of species recorded in the UK. The NHM is committed to maintaining this service, to fill outstanding gaps, and keep existing checklists up-to-date through liaison with the individuals and recording schemes, who are the data providers.

2. Please describe in what way your Focal Point activities (see above key functions and roles) will be sustained at national level in the near future.

The Natural History Museum is a founder member of the National Biodiversity Network. The NBN, which includes a number of government agencies (and is also the UK node for GBIF) enables us to feed into, and respond to, the UK biodiversity agenda.

3. How has PESI contributed to:
– the sustainability of your national Focal Point status.

Sustainability was assured, even without PESI.

– the connection with other European or national biodiversity initiatives.

Focal Point meetings have provided useful networking opportunities.

– the quality and use of biodiversity information both at a national and European scale.

The Taxon match tool has proved invaluable. Existing data in PESI has been used to improve quality in the Species Dictionary, principally by supplying missing name authorities and uncovering undetected synonymy.

4. Which specific support from PESI would in your view improve the contacts with European and/or national biodiversity initiatives, including liaison with LifeWatch?

A Wiki and electronic newsletter.

5. What support would you require from PESI (in cooperation with LifeWatch) to ensure that your FP activities gain priority on the national political Agenda?

Encourage 'citizen science' activities.

6. Would you like to continue the national Focal Point activities after the project timespan?

It is anticipated that our role will continue, although money for foreign travel will be limited for the next few years.

7. Would you like to coordinate/manage on a more general basis the activities of grouped European Focal Points?

This would have to be negotiated with senior management but, as one of the leading museums in Europe, we would be well-placed to become involved.

8. What would in your view a continuity plan of the Focal Point Network comprise beyond PESI (establishment of a formal Focal Point Organisation, involvement in LifeWatch, association with GBIF, other)?

The key issue is that you are asking already busy people take on additional duties for no payment. Unless focal point activities align with the mission of the host institution, it is unreasonable to expect the employer to sanction these activities. Effort is likely to vary widely between countries, making the network patchy. The best way forward would be to build some funding for Focal Points into future large-scale funding initiatives (such as Lifewatch).

9. Please feel free to communicate any other ideas, suggestions, comments.

The PESI data warehouse is now an excellent resource, but to keep its relevance it must be regularly updated. This must be impressed on all stakeholders and funding agencies. I believe that a centralised facility has a greater chance of survival than separate national endeavours. PESI needs an institutional home that can maintain it independent of external funding. Whilst it might physically reside in one location, I wonder whether management could rotate between partners, in the same way that the EU presidency rotates. This might provide impetus for national governments to support the project/service for a defined period.

Appendix 3 — Individual Focal Point Sustainability Plan — UNIPA — Partner 15

Institute:
Dipartimento di Scienze Ambientali e Biodiversità, Università di Palermo (formerly Dipartimento di Scienze Botaniche, Università di Palermo)

Contact person: Prof. Francesco M. Raimondo

1. Please give a short description of your sustainability plan in view of your local/national situation

The Dipartimento di Scienze Ambientali e Biodiversità, Università di Palermo (Formerly Dipartimento di Scienze botaniche) works since several years to the assessment of biodiversity at Regional, National and Mediterranean level by field studies, review of old collections, databasing, and checklists preparation. This was done mainly with the financial support of the Regional Administration (Regione Siciliana), European projects gave new boost to these researches and allowed the sharing of information with the rest of the European and Mediterranean institution involved in Biodiversity studies.

2. Please describe in what way your Focal Point activities (see above key functions and roles) will be sustained at national level in the near future

The members of the Dipartimento di Biologia ambientale e biodiversità in the bosom of the Società Botanica Italiana participate to national projects aiming to: Unlock local and regional species inventories, Compilation of national species checklists including aliens, update and quality check of national species checklists.

These data will be make available to the PESI user community, the organization of the Annual meetings of the Società Botanica Italiana are good opportunities to Build awareness of PESI at national level and to demonstrate PESI tools to national users.

New opportunities (e.g. festivities and National and International days) has to be stimulated to communicate with policy makers regarding country-specific prioritised species and to advise them in environmental policy-making regarding threatened species and pests.

3. How has PESI contributed to:
– the sustainability of your national Focal Point status
– the connection with other European or national biodiversity initiatives
– the quality and use of biodiversity information both at a national and European scale

PESI contributed to the sustainability of our national Focal Point status giving us the possibility to hire ad hoc personnel to find, verify and update the Euro+Med Cheklist data. The connection with other European and national Biodiversity initiatives was supported by the organization of meetings that started cooperatives studies. The PESI portal and the PESI taxonomic tools developed improved the availability and the accessibility of the data collected

4. Which specific support from PESI would in your view improve the contacts with European and/or national biodiversity initiatives, including liaison with LifeWatch?

Organization of meetings and workshops

5. What support would you require from PESI (in cooperation with LifeWatch) to ensure that your FP activities gain priority on the national political Agenda?

Organization of high visibility activities of popular science to awaken our politicians and the public opinion to biodiversity studies

6. Would you like to continue the national Focal Point activities after the project timespan?

YES

7. Would you like to coordinate/manage on a more general basis the activities of grouped European Focal Points?

YES

8. What would in your view a continuity plan of the Focal Point Network comprise beyond PESI (establishment of a formal Focal Point Organisation, involvement in LifeWatch, association with GBIF, other)?

Association with GBIF, in this moment Italy does not belong to GBIF

9. Please feel free to communicate any other ideas, suggestions, comments.

A part of the efforts has to be addressed to the field collection of Primary data on Biodiversity, in this moment projects like PESI worked on literature data.

Appendix 4 — Individual Focal Point Sustainability Plan — CSFI — Partner 21

Institute: COMITATO SCIENTIFICO PER LA FAUNA D'ITALIA

Contact person: Fabio Stoch

1. Please give a short description of your sustainability plan in view of your local/national situation

CSFI as Focal Point is participating to the Italian Network of Biodiversity, a three-year project financially supported by the Italian Ministry for Environment. This will allow to take care of the website, to support the updating of the Checklist of Italian Fauna which will act as a nomenclator for the entire network, which will be connected with BioCase and GBIF

2. Please describe in what way your Focal Point activities (see above key functions and roles) will be sustained at national level in the near future

The key functions will be supported by the Network of Biodiversity; the amount of resources is very low due to the economic chrisis in Italy, and further support will be searched through EU projects

3. How has PESI contributed to:
– the sustainability of your national Focal Point status
– the connection with other European or national biodiversity initiatives
– the quality and use of biodiversity information both at a national and European scale

As a member of PESI, and following the PESI strategy on the role of nomenclators, CSFI obtained an important position within the Italian Network and as a wrapper of data to GBIF, BioCase and other EU and international initiatives; moreover the Checklist of CSFI will be used to validate all the biodiversity datasets including Nature 2000 monitoring programmes

4. Which specific support from PESI would in your view improve the contacts with European and/or national biodiversity initiatives, including liaison with LifeWatch?

We have currently only indirect contacts with the Italian representatives of LifeWatch; we are preparing a seminar to illustrate PESI results at the national level, inviting the PESI co-ordinator and other operators to improve our image and contacts

5. What support would you require from PESI (in cooperation with LifeWatch) to ensure that your FP activities gain priority on the national political Agenda?

The above mentioned symposium is the first step. The effective implementation of a working network of Focal Points will help to throw light on our activity at the national, political level

6. Would you like to continue the national Focal Point activities after the project timespan?

Yes

7. Would you like to coordinate/manage on a more general basis the activities of grouped European Focal Points?

Yes, if a small amount of resources will be available we have the skills to co-ordinate general activities of FPs

8. What would in your view a continuity plan of the Focal Point Network comprise beyond PESI (establishment of a formal Focal Point Organisation, involvement in LifeWatch, association with GBIF, other)?

FP Network must continue in the effort to offer a validation set of nomenclators which is the basis for LifeWatch and GBIF; more important contacts (for our faunal aspect) with ICZN and his Zoobank is needed to validate the assembly rules of the checklists

9. Please feel free to communicate any other ideas, suggestions, comments.

FP Network participants may put together taxonomic skills to proceed with the implementation of distributional atlases of important groups, following the example of the Italian Network, offering to EU (and indirectly to GBIF) a sound basis to build an atlas, not only offering datasets, more or less validated; this means a) to create capacity building, i.e. taxonomic training; b) to improve literature screening and field sampling (in connection with the heredity of EDIT, LifeWatch, BioFresh, etc.) offering the skills; c) to maintain updated the national (and consequently EU) checklists

Appendix 5 — Individual Focal Point Sustainability Plan — NIB — Partner 30

Institute: National institute of Biology

Contact person: Davorin Tome

1. Please give a short description of your sustainability plan in view of your local/national situation

Considering all the facts, Focal Point will operate only "on demand" – if someone will ask for a help, which a Focal Point will be able to provide

2. Please describe in what way your Focal Point activities (see above key functions and roles) will be sustained at national level in the near future

- demonstrate PESI to potential users

- assist policy makers with threaten species

- translations, adding new vernacular names, new checklists, species data if and when they will become available

3. How has PESI contributed to:

– the sustainability of your national Focal Point status

– the connection with other European or national biodiversity initiatives

– the quality and use of biodiversity information both at a national and European scale

4. Which specific support from PESI would in your view improve the contacts with European and/or national biodiversity initiatives, including liaison with LifeWatch?

- financial support

5. What support would you require from PESI (in cooperation with LifeWatch) to ensure that your FP activities gain priority on the national political Agenda?

- financial and political support

6. Would you like to continue the national Focal Point activities after the project timespan?

- yes, but with limited effort (due to lack of funds)

7. Would you like to coordinate/manage on a more general basis the activities of grouped European Focal Points?

- No

8. What would in your view a continuity plan of the Focal Point Network comprise beyond PESI (establishment of a formal Focal Point Organisation, involvement in LifeWatch, association with GBIF, other)? - ?

Appendix 6 — Individual Focal Point Sustainability Plan — RBINS - Partner 00

Institute: Belgian Biodiversity Platform / Royal Belgian Institute of Natural Sciences - non-contracted Focal Point

Contact person: Hendrik Segers

1. Please give a short description of your sustainability plan in view of your local/national situation

The Belgian PESI NFP (non-funded) activities are integrated in the work of the Belgian Biodiversity Platform (the Platform) as Belgian GBIF node, collaborator in 4D4Life (CoL) project, and coordinator of the Freshwater Animal Diversity Assessment project (FADA).

2. Please describe in what way your Focal Point activities (see above key functions and roles) will be sustained at national level in the near future

Basic continuity can be ensured through the core activities of the Platform. However, any specific PESI-related activity will only be considered, if support can be found.

3. How has PESI contributed to:
– the sustainability of your national Focal Point status

Not.

– the connection with other European or national biodiversity initiatives

Not to our knowledge – the Platform is already well-linked to such initiatives.

– the quality and use of biodiversity information both at a national and European scale

Unknown.

4. Which specific support from PESI would in your view improve the contacts with European and/or national biodiversity initiatives, including liaison with LifeWatch?

As non-funded PESI partner we have not been involved with, or benefited from, any support from PESI regarding such contacts. The Platform, as science-policy interface, is already well liaised with initiatives as the ones listed. As far as LifeWatch is concerned, we await the announcement of (and hearing details about) a funded LifeWatch project in support of a European Taxonomic standard or backbone.

5. What support would you require from PESI (in cooperation with LifeWatch) to ensure that your FP activities gain priority on the national political Agenda?

See (4)

6. Would you like to continue the national Focal Point activities after the project timespan?

Yes

7. Would you like to coordinate/manage on a more general basis the activities of grouped European Focal Points?

We're open for discussion on this.

8. What would in your view a continuity plan of the Focal Point Network comprise beyond PESI

- establishment of a formal Focal Point Organisation: OK, but please avoid crowding the landscape any further. I suggest linking up with/becoming member of SMEBD
- involvement in LifeWatch: OK if this would mobilize resources to support the network. Otherwise the potential relevance of LifeWatch to PESI NFP network is not entirely clear (understatement).
- association with GBIF: doubt the relevance of this – (1) associated partners of GBIF have little influence in GBIF governance and (2) I fail to see what/how GBIF can contribute to the continuity of a PESI (regional!) NFP network beyond PESI.
- other)?

9. Please feel free to communicate any other ideas, suggestions, comments.

I suggest liaising more with international initiatives beyond Europe, in particular Catalogue of Life.

Appendix 7 — Individual Focal Point Sustainability Plan — NTNU - Partner 24

Institute: Norwegian University of Science and Technology (NTNU), Museum of Natural History and Archaeology until May 1. 2011 and thereafter Norwegian Biodiversity Information Centre (NBIC, located at NTNU)

Contact person: Kaare Aagaard until May 1. 2011 and Nils Valland after May 1. 2011

1. Please give a short description of your sustainability plan in view of your local/national situation

The Norwegian Biodiversity Information Centre (NBIC) was established by the Norwegian Government in 2005 with permanent funding. Further information on NBIC is available in the PESI Focal Point Handbook section 6.2.

2. Please describe in what way your Focal Point activities (see above key functions and roles) will be sustained at national level in the near future

NBIC has the role of being a national source of information on biodiversity. The organisations main function is to supply the public with updated and accessible information on Norwegian species and ecosystems. NBIC has the mandate of collecting and maintaining scientific and vernacular names for Norwegian species. NBIC has established 29 scientific expert committees with over 90 members with the task of collecting, assuring quality and revising Norwegian taxa. NBIC has established a national database (Artsnavnebase) for maintaining and distributing (webservices and downloading) the taxonomic thesauri to national and international users. To sustain these activities NBIC have regular governmental budgets.

3. How has PESI contributed to:

– the sustainability of your national Focal Point status

PESI has strongly increased the national awareness and usefulness of a harmonized European nomenclature. This awareness has stimulated and justified the importance of that NBIC continues and contributes to further development of a European taxonomy infrastructure

– the connection with other European or national biodiversity initiatives

International taxonomy services is needed both for Lifewatch, INSPIRE and regional (e.g. Nordic) cooperation on sharing data and information about species. Unified taxonomy is a crucial infrastructure to achieve interoperability in sharing biodiversity information.

– the quality and use of biodiversity information both at a national and European scale

The result will be visible when the www.eu-nomen.eu is fully operable. At national level the harvesting of names (scientific and vernacular) is an important possibility.

4. Which specific support from PESI would in your view improve the contacts with European and/or national biodiversity initiatives, including liaison with LifeWatch?

A PESI taxonomic portal service is a cornerstone in all cooperation with data sharing initiatives as LifeWatch, INSPIRE, GBIF and others. The technical infrastructure and the taxon linking facility needs to be dynamic to ensure that taxon concepts development both nationally and at European level is facilitated and thereby the overall taxonomy is updated. The taxonomic expert network will in addition make it easier to cooperate with other institutions.

5. What support would you require from PESI (in cooperation with LifeWatch) to ensure that your FP activities gain priority on the national political Agenda?
In Norway the Focal Point activities are a main and lasting task for NBIC. Functional PESI services and international visibility will strengthen the capability of distribute species name services and biodiversity informatics.

6. Would you like to continue the national Focal Point activities after the project timespan?
Yes, the national taxonomy task for NBIC is a continuous focus area. The
Norwegian Taxonomy Initiative will address the need for strengthening the education of new taxonomists.

7. Would you like to coordinate/manage on a more general basis the activities of grouped European Focal Points?
At present NBIC has no available resources for broadening the scope of working in grouped Focal Points. Nordic cooperation within LifeWatch may, if adequate funding, open other possibilities.

8. What would in your view a continuity plan of the Focal Point Network comprise beyond PESI
The network of responsible national Focal Points will be an essential contribution to update and maintain the European taxonomy dynamics. In a permanent taxonomy organisation, PESI follow-up, should maintain the PESI results and further develop a stable lasting European institution and infrastructure for taxonomy services. National Focal Points with sufficient funding should be an integrated part of this institutional network. The PESI-permanent institution could be an important node in a broader cooperation with other biodiversity sharing initiatives/organisations/institutions as LifeWatch, GBIF, INPIRE, EoL and numerous others.

9. Please feel free to communicate any other ideas, suggestions, comments.
N/A

Appendix 8 — Individual Focal Point Sustainability Plan — NRC - Partner 20

Institute: Nature Research Centre

Contact person: Eduardas Budrys

1. Please give a short description of your sustainability plan in view of your local/national situation

Nature Research Centre (NRC) will continue its activities, in collaboration with other institutions conducting taxonomic and other biodiversity-related research, thus sustaining the basic Focal Point functions after the end of PESI.

2. Please describe in what way your Focal Point activities (see above key functions and roles) will be sustained at national level in the near future

Currently the Nature Research Centre (NTC) is the largest biodiversity- and taxonomy-related research institution in Lithuania. It cooperates with the few other institutions (universities and museums), where taxonomic research is proceeded. NRC publishes a quarterly scientific journal in English, where taxonomic study papers are a part of the scope. These activities of NRC in the future will naturally sustain its function as the Focal Point.

Since Lithuania is a relatively small country with relatively limited taxonomic expertise, all available experts are easily found by the standard web search tools and contacted by the interested stakeholders directly. Therefore, for the national goals, a more sophisticated structure of the Focal Point or building a complex taxonomic expertise network is not needed at the moment.

3. How has PESI contributed to:

– the sustainability of your national Focal Point status

Despite a little direct funding of Focal Point activities, the PESI project stimulated such activities as integration of the NRC into the European Focal Point network, accumulation and dissemination of the national taxonomy-related metadata, involvement of new young taxonomists into these activities, etc.

4. Which specific support from PESI would in your view improve the contacts with European and/or national biodiversity initiatives, including liaison with LifeWatch?

At the moment, the NRC is not involved into activities within LifeWatch. Hopefully, the activities following the PESI project will stimulate such involvement in the future.

5. What support would you require from PESI (in cooperation with LifeWatch) to ensure that your FP activities gain priority on the national political Agenda?

Information on the ongoing European projects in the frame of LifeWatch submitted directly to the Ministry of Education and Science as well as the Ministry of Environment might be efficient way in shifting up the biodiversity and taxonomy in the list of the national political Agenda.

6. Would you like to continue the national Focal Point activities after the project timespan?
Yes

7. Would you like to coordinate/manage on a more general basis the activities of grouped European Focal Points?
No

8. What would in your view a continuity plan of the Focal Point Network comprise beyond PESI
Beyond PESI, the NRC will continue its activities in the field of taxonomic expertise, including publishing of national checklists and accumulation of other biodiversity-related information. Possible involvement of NRC into LifeWatch and GBIF in the future would strengthen such international Focal Point roles and functions like communication of national expertise to the European and wider taxonomic community, and the transfer of the international leading edge knowledge and tools to the national scale.

9. Please feel free to communicate any other ideas, suggestions, comments.
N/A

Appendix 9 — Individual Focal Point Sustainability Plan — SMNH — Partner 25

Institute: State Museum of Natural History, National Academy of Sciences of Ukraine

Contact person: Volodymyr Rizun

1. Please give a short description of your sustainability plan in view of your local/national situation

Practically all key functions (building the taxonomic expertise network; unlock local and regional species inventories; compilation of national species checklists; update and quality check of national species checklists; make these available to the PESI user community; building awareness of PESI at national level; demonstrate PESI tools to national users and contribution of the Focal Point partners to PESI) will be performed by FaEu Ukrainian Focal Point regardless of PESI timespan, because all these activities (key functions) closely correspond with the institutional (SMNH) long-term plans. The main aim of the FaEu Ukrainian Focal Point in the nearest future is the development and maintenance of "Biodiversity – Ukraine" web-portal and building the National Biodiversity Data Centre on this base.

The execution of the part of key functions (communicate with policy makers regarding country-specific prioritised species; assist in environmental policy-making regarding threatened species or pests) connected with the contacts with policy makers is complicated due to the difficult economic situation in the country and the existing opinion (point of view) that this thematic is not important or not at the first place now. Generally all attempts and promotion in these directions have been finished only by declarations.

According to the strategic roles of the PESI Focal Points, listed above, at the first place we can see the integration of the specialists joined to the national taxonomic expertise network by holding workshops (on key topics like: data management; validation national species checklists) with the crucial national environmental policy makers. The next steps will be: liaise with governmental bodies on implementation of European standards relevant to regulation and environmental monitoring; document local expertise and applied tools for the greater European taxonomic community.

2. Please describe in what way your Focal Point activities (see above key functions and roles) will be sustained at national level in the near future.

Taking into consideration our national realities we have greate doubts that Focal Point activities will be sustained at the national level in the nearest future.

3. How has PESI contributed to:

– the sustainability of your national Focal Point status.

Very important was the work on preparation of the PESI project, the information on the European biodiversity e-resources, clarifying of priorities and the experience obtained during the execution of PESI project, the financial support.

– the connection with other European or national biodiversity initiatives.
Understanding the role and the place of the Ukrainian Focal Point; trigger to gathering all possible information about biodiversity resources in Ukraine, the national experts web creation, encouraging further work in this direction.

– the quality and use of biodiversity information both at a national and European scale.
Understanding the needs of the creation of national standardized and unified taxonomic checklists of Ukrainian fauna (biota).

4. Which specific support from PESI would in your view improve the contacts with European and/or national biodiversity initiatives, including liaison with LifeWatch?
Exchanging by the information and informational support by the PESI web-portal and Biodiversity-Ukraine web-portal; joining Fauna Ukraine Focal Point to the European initiatives and projects (as well LifeWatch). Joining Fauna Ukraine Focal Point to the GBIF (contribution of collections data in particular) via PESI or via formal Focal Point Organisation associated with GBIF omitting the national Ministry level (because joining Ukraine to the GBIF is low-probable in the nearest future).

5. What support would you require from PESI (in cooperation with LifeWatch) to ensure that your FP activities gain priority on the national political Agenda?
As the “Biodiversity – Ukraine” web-portal and the National Biodiversity Data Centre built on this basis should be the main (national/international) tools, which ensure the liason between the users, scientists and policy-makers, the main support required from PESI (in cooperation with LifeWatch) is the minimal financial support for the development and maintaining the national “Biodiversity – Ukraine” web-portal, what will be the evidence of the importance and the interest from EU structures and will ensure the priority of the national FP activities on the national political Agenda.

6. Would you like to continue the national Focal Point activities after the project timespan?
Yes.

7. Would you like to coordinate/manage on a more general basis the activities of grouped European Focal Points?
No. We have much to do on the national level.

8. What would in your view a continuity plan of the Focal Point Network comprise beyond PESI (establishment of a formal Focal Point Organisation, involvement in LifeWatch, association with GBIF, other)?

All formal actions (listed above) will be useful and not spare, but the most reliable basis of the Focal Point Network vitality is the participation in the real work (projects etc.).

9. Please feel free to communicate any other ideas, suggestions, comments.

Appendix 10 — Individual Focal Point Sustainability Plan — NRM — Partner 22

Institute: Biodiversity Informatics Unit at the Swedish Museum of Natural History, Stockholm

Contact person: Anders Telenius -

1. Please give a short description of your sustainability plan in view of your local/national situation

In Sweden close cooperation between GBIF and PESI-officials exist, and this will probably be the best way to create sustainability in the operations of the latter organization (1). Since 1 Jan. 2011 the newly established Biodiversity Informatics Unit at the Swedish Museum of Natural History house both endeavours, and as GBIF is currently proposing prolonged engagement 2012-2016 I intend to include the PESI Focal Point work in this application. In particular the position at the museum – in the midst of a number of informatics initiatives – is very valuable, but we also have a fortunate position as partners of the Swedish Lifewatch initiative (otherwise posted at the Species Information Centre/Swedish University of Agricultural Science in Uppsala)

2. Please describe in what way your Focal Point activities (see above key functions and roles) will be sustained at national level in the near future.

(2, 3a+b). PESI, GBIF and Lifewatch have mutually contributed to the quality and use of biodiversity information (3c).

3. How has PESI contributed to:

– the sustainability of your national Focal Point status.

see 2

– the connection with other European or national biodiversity initiatives.

see 2.

– the quality and use of biodiversity information both at a national and European scale.

see 2

4. Which specific support from PESI would in your view improve the contacts with European and/or national biodiversity initiatives, including liaison with LifeWatch?

Left unanswered

5. What support would you require from PESI (in cooperation with LifeWatch) to ensure that your FP activities gain priority on the national political Agenda?

The support PESI would be able to supply me/us with, is that of becoming a solid and trustworthy European partner to any of the other biodiversity informatics initiatives

6. Would you like to continue the national Focal Point activities after the project timespan?

Yes, I would like to – and intend to - continue Focal Point activities after the project time span

7. Would you like to coordinate/manage on a more general basis the activities of grouped European Focal Points?
I would not at this stage have the guts to explicitly state that we would like to manage a group of European Focal Points – BUT… the application for funding mentioned includes organizing a Nordic/Baltic network of BIF:s hence also (in accordance with the above) PESI activities (7, 8).

8. What would in your view a continuity plan of the Focal Point Network comprise beyond PESI (establishment of a formal Focal Point Organisation, involvement in LifeWatch, association with GBIF, other)?
See 7.

9. Please feel free to communicate any other ideas, suggestions, comments.

Appendix 11 — Individual Focal Point Sustainability Plan — LU — Partner 33

Institute: University of Latvia

Contact person: Voldemārs Spuņģis

1. Please give a short description of your sustainability plan in view of your local/national situation
Focal point is planned to establish in the Faculty of Biology, University of Latvia as a centre of biodiversity research in Latvia. It is planned to establish Biodiversity laboratory under the governance of Department of zoology and animal ecology and Department of botany and ecology. Focal point activities will be a part of the functions of the laboratory.

2. Please describe in what way your Focal Point activities (see above key functions and roles) will be sustained at national level in the near future
The national focal point will serve as a node for data collecting, exchange, analysis and distribution. Available taxonomic experts will be involved in the newly established virtual network. Financial issues are not discussable now, but it is expected to attract resources in the future.

3. How has PESI contributed to:
– the sustainability of your national Focal Point status
– the connection with other European or national biodiversity initiatives
– the quality and use of biodiversity information both at a national and European scale
PESI should include national focal point in the network of focal points. PESI should provide with the current news in biodiversity related information.

4. Which specific support from PESI would in your view improve the contacts with European and/or national biodiversity initiatives, including liaison with LifeWatch?
Mostly by informing about biodiversity related activities and including the national focal point in the mailing lists.

5. What support would you require from PESI (in cooperation with LifeWatch) to ensure that your FP activities gain priority on the national political Agenda?
Support the activities of the national focal point.

6. Would you like to continue the national Focal Point activities after the project timespan?
Yes!

7. Would you like to coordinate/manage on a more general basis the activities of grouped European Focal Points?
No!

8. What would in your view a continuity plan of the Focal Point Network comprise beyond PESI (establishment of a formal Focal Point Organisation, involvement in LifeWatch, association with GBIF, other)?
The national focal point has to be a member of formal Focal Point Organisation.

9. Please feel free to communicate any other ideas, suggestions, comments.

Appendix 12 — Individual Focal Point Sustainability Plan — IBSAS — Partner 17

Institute: Institute of Botany, Slovak Academy of Sciences, Bratislava, Slovakia

Contact person: Karol Marhold, prof., dr.

1. Please give a short description of your sustainability plan in view of your local/national situation
The Institute of Botany SAS took part in the improvement of the Euro+Med PlantBase checklist of vascular plants. We had responsibility for numerous families of vascular plants. The institute can grant that we will continue the editorial work on these families (taking care about the input and comments from the national Euro+Med PlantBase centers) as well as to continue in serving as National node (focal point) of the Euro+Med PlantBase. Both these activities will be carried out by the permanent staff of the Institute.

2. Please describe in what way your Focal Point activities (see above key functions and roles) will be sustained at national level in the near future
The Institute of Botany SAS is the key institution in taxonomic research of vascular plants in the Slovak Republic. In this capacity we are leading the long-term project of the Flora of Slovakia and updating the Checklist on non-vascular and vascular plants of Slovakia that is available not only in a printed form, but also online.

3. How has PESI contributed to:
– the sustainability of your national Focal Point status
– the connection with other European or national biodiversity initiatives
– the quality and use of biodiversity information both at a national and European scale
The PESI project considerably contributed towards the improvement of the Euro+Med PlantBase checklist, which is now available via PESI portal. The project helped to establish regular workflow of the input of corrections into the database and its quality check. The project also helped to organise the workflow of the National Focal Point and improved its contacts with other National Focal Point. The established structure has full support of the Institute. It will help to improve the quality of the national checklists of non-vascular and vascular plants in Slovakia and cooperation in improving the European checklists.

4. Which specific support from PESI would in your view improve the contacts with European and/or national biodiversity initiatives, including liaison with LifeWatch?

5. What support would you require from PESI (in cooperation with LifeWatch) to ensure that your FP activities gain priority on the national political Agenda?

6. Would you like to continue the national Focal Point activities after the project timespan?
Yes, as described above.

7. Would you like to coordinate/manage on a more general basis the activities of grouped European Focal Points?
Yes, we will continue in the work on the improvement of the Euro+Med PlantBase (part of the PESI final product), keeping the responsibility for the selected families of vascular plants.

8. What would in your view a continuity plan of the Focal Point Network comprise beyond PESI (establishment of a formal Focal Point Organisation, involvement in LifeWatch, association with GBIF, other)?
We see here as important point of the continuity plan to keep close connections with the GBIF and with the Global Taxonomic Initiative of the CBD (both at national and European levels).

9. Please feel free to communicate any other ideas, suggestions, comments.

Appendix 13 — Individual Focal Point Sustainability Plan — NNM/NCB — Partner 19

Institute: NCB Naturalis

Contact person: Roy Kleukers

1. Please give a short description of your sustainability plan in view of your local/national situation
In the Netherlands we have the Dutch Species Register, which is well established as an taxonomic authority and (more and more) as on online species encyclopedia. NCB Naturalis and partners will continue to keep the database up-to-date. There is broad support to keep standardizing the lists with PESI.

2. Please describe in what way your Focal Point activities (see above key functions and roles) will be sustained at national level in the near future
All functions and roles mentioned will be sustained in the Netherlands in the future.

3. How has PESI contributed to:
– the sustainability of your national Focal Point status
– the connection with other European or national biodiversity initiatives
– the quality and use of biodiversity information both at a national and European scale
PESI has provided a standardized lists and trees which help greatly in standardizing taxonomic information at the national level. The network of focal points has provided a good basis for future initiatives.

4. Which specific support from PESI would in your view improve the contacts with European and/or national biodiversity initiatives, including liaison with LifeWatch?
Ongoing development of the PESI-portal.

5. What support would you require from PESI (in cooperation with LifeWatch) to ensure that your FP activities gain priority on the national political Agenda?
It would help if biodiversity issues would be put on the European agenda.

6. Would you like to continue the national Focal Point activities after the project timespan?
Yes

7. Would you like to coordinate/manage on a more general basis the activities of grouped European Focal Points?
That would surely be possible, depending on the activity.

8. What would in your view a continuity plan of the Focal Point Network comprise beyond PESI (establishment of a formal Focal Point Organisation, involvement in LifeWatch, association with GBIF, other)?

Any alignment with other global, European or taxonomically (e.g. Fishbase) organized biodiversity initiatives will help.

9. Please feel free to communicate any other ideas, suggestions, comments.

Appendix 14 — Individual Focal Point Sustainability Plan — MBA — Partner 39

Institute: The Marine Biological Association of the United Kingdom

Contact person: Daniel Lear

1. Please give a short description of your sustainability plan in view of your local/national situation

The Marine Biological Association (MBA) was formed in 1884 as a Learned Society to promote scientific research into life in the sea and to disseminate that knowledge. As such the aims of the PESI project and the focal point activities are clearly tied to the long history of the MBA and its mission. The MBA is a charity and whilst the MBA receives some core funding from UK Research Councils additional resources must be bid for from a variety of sources. The close alignment of the focal point activities with the MBA's mission means that activities that support the MBA's role as a marine focal point will continue to be written into funding bids. In the short-term the promotion of the developed national species checklist at the national level will be championed by 2 key MBA initiatives; the Marine Life Information Network (www.marlin.ac.uk) and the UK Archive for Marine Species and Habitats Data (www.dassh.ac.uk).

2. Please describe in what way your Focal Point activities (see above key functions and roles) will be sustained at national level in the near future.

The MBA is now working with UK Government and its agencies to promote taxonomic standards and tools throughout the marine biological community. There is now an established Standards Working Group for the wider marine community in the UK and the work undertaken by the MBA as a PESI focal point has been integrated into the suite of standards that this group recommends. Some national resources have been identified to maintain the marine species checklist that was initially developed with funds from PESI ensuring that the list does not quickly become outdated.

3. How has PESI contributed to:

– the sustainability of your national Focal Point status
– the connection with other European or national biodiversity initiatives
– the quality and use of biodiversity information both at a national and European scale

The work of PESI has allowed the MBA to identify and work with key partners in the UK to promote taxonomic standards to all members of the marine biological data community. PESI has ensured sufficient resources are available to allow the development of a standard checklist that will facilitate data exchange between organizations and has been adopted by the UK marine biodiversity data archive centre (DASSH) as the default species checklist for all its data holdings. By standardising UK species against the ERMS list we have enabled the interoperability of UK species holdings with those held by European and national groups.

4. Which specific support from PESI would in your view improve the contacts with European and/or national biodiversity initiatives, including liaison with LifeWatch?
Funding for EU-wide workshops to exchange tools and best-practice.

5. What support would you require from PESI (in cooperation with LifeWatch) to ensure that your FP activities gain priority on the national political Agenda?
Continued promotion and the development of EU-wide tools that can be adopted at the national level would encourage buy-in from UK government and its agents.

6. Would you like to continue the national Focal Point activities after the project timespan?
Yes, we see the role of a PESI Focal Point for ERMS and the associated activities as something that the MBA has a long history and expertise in, and will continue to operate in this role.

7. Would you like to coordinate/manage on a more general basis the activities of grouped European Focal Points?
The MBA can undertake to provide a wider co-ordination role, providing such a role is suitably resourced.

8. What would in your view a continuity plan of the Focal Point Network comprise beyond PESI (establishment of a formal Focal Point Organisation, involvement in LifeWatch, association with GBIF, other)?
It is important that the work of the Focal Point Network is integrated into other EU funded initiatives including LifeWatch, EMODNet, EurOBIS. A Focal Point Organization would allow for greater co-ordination with other initiatives and ensure that the current focal points retain a cohesive set of ideas, values and aims.

9. Please feel free to communicate any other ideas, suggestions, comments.

Appendix 15 — Individual Focal Point Sustainability Plan — TU — Partner 3

Institute: Trakya University, Faculty of Science, Dept. of Biology – Edirne/ TURKEY

Contact person: Prof.Dr. Nihat AKTAÇ

1. Please give a short description of your sustainability plan in view of your local/national situation

Turkey Focal Point is the Official Cooperation of TU Biology Department. Contact person is the Head of the Zoology Department. The staffs of the department are working mainly on Biodiversity of Turkey. The department has vertebrate and invertebrate collections (mainly insects). The academic staffs of the department have good relation and collaboration with the other Turkish Universities. The check lists of studied groups were obtained for PESI project directly from academic experts so the validity of the checklists has high percentage. The validation process of checklists for Turkey attained quite interest of the academic staff and they will continue to support PESI initiative.

2. Please describe in what way your Focal Point activities (see above key functions and roles) will be sustained at national level in the near future.

As it is mentioned above the Focal Point activities will be sustained at national level by: Unlock local and regional species inventories, compilation of national species checklists, and update and quality check of national species checklists, make these available to the PESI user community, building awareness of PESI at national level in the near future.

3. How has PESI contributed to:

– the sustainability of your national Focal Point status.
– the connection with other European or national biodiversity initiatives.
– the quality and use of biodiversity information both at a national and European scale.

PESI has contributed to the sustainability of our national Focal Points status by recognizing us as the National Focal Point for the terrestrial and fresh water fauna,Get connection with other European national Biodiversity initiatives,Use of reliable biodiversity information's both at a national and European scale.

4. Which specific support from PESI would in your view improve the contacts with European and/or national biodiversity initiatives, including liaison with LifeWatch?

This could be discussing with the academic staff and NGO.

5. What support would you require from PESI (in cooperation with LifeWatch) to ensure that your FP activities gain priority on the national political Agenda?

Turkey involved in the LifeWatch by Sabanci University, Istanbul. We expect more close collaboration with the representative of LifeWatch in Turkey.

6. Would you like to continue the national Focal Point activities after the project timespan?

We can not decide to continue the national focal point activities with the same team by the reason of the administrative position of contact person my change in the near future.

7. Would you like to coordinate/manage on a more general basis the activities of grouped European Focal Points?
We have to discuss with our colleagues.

8. What would in your view a continuity plan of the Focal Point Network comprise beyond PESI (establishment of a formal Focal Point Organisation, involvement in LifeWatch, association with GBIF, other)?
We could spent effort for the establishment of a formal Focal Point Organisation and association with GBIF.

9. Please feel free to communicate any other ideas, suggestions, comments.

Appendix 16 — Individual Focal Point Sustainability Plan — NMNHS — Partner 31

Institute: National Museum of Natural History (NMNHS), Bulgaria

Contact person: Alexi Popov, Pavel Stoev

1. Please give a short description of your sustainability plan in view of your local/national situation

The National Museum of Natural History (NMNHS) has a history of more than one hundred and twenty years. It is the oldest museum in Bulgaria and the oldest and richest among the natural history museums on the Balkan Peninsula. Currently the museum is part of the Bulgarian Academy of Sciences.

NMNHS is the only national institution directly engaged with the preservation of scientific collections of live and non-live nature from Bulgaria and the world. The study of biodiversity, environmental protection and the evolution of organisms are the museum's major priorities. Consequently, the main task of NMNHS is the all-around study of the fauna, flora, fossils, minerals and rocks of Bulgaria and other countries. It develops the following scientific areas: taxonomy, faunistics, floristics, mineralogy, zoogeography and ecology. Some of the areas have priority as a result of which NMNHS has become the national centre of biospeleology, archaeozoology and palaeontology of vertebrates and of bat studies. Insect studies are also highly developed.

2. Please describe in what way your Focal Point activities (see above key functions and roles) will be sustained at national level in the near future

NMNHS is one of the main taxonomic centers in the country. The museum will keep providing expertise to national and international institutions on issues related to biodiversity. With its numerous education activities (exhibitions, lectures, performances) the museum build awareness in the society on questions related to biodiversity and its conservation. We are trustful partner of national agencies and ministries and conduct long-term monitoring and inventories of selected groups of organisms.

3. How has PESI contributed to:

- the sustainability of your national Focal Point status
- the connection with other European or national biodiversity initiatives
- the quality and use of biodiversity information both at a national and European scale

PESI strengthened our position as a national taxonomic center. The project helped us to create contacts with numerous partners throughout Europe and to exchange ideas for future collaboration.

4. Which specific support from PESI would in your view improve the contacts with European and/or national biodiversity initiatives, including liaison with LifeWatch?

The national meeting participated by representatives from LifeWatch was the event that introduced us to LifeWatch and its activities.

5. What support would you require from PESI (in cooperation with LifeWatch) to ensure that your FP activities gain priority on the national political Agenda?
PESI and LifeWatch have to establish closer contacts with national authorities (Ministry of Environment, Ministry of Science, Youth and Education) via political lobbies through the European Parliament and EC DGs and try to promulgate the ideas of PESI and LifeWatch in a way to be given priority in the national plans for research and environmental protection.

6. Would you like to continue the national Focal Point activities after the project timespan?
Yes. We could serve as a coordination center for the region (Turkey, North Africa, Russia, Balkans) in the frames of another FP project.

7. Would you like to coordinate/manage on a more general basis the activities of grouped European Focal Points?
We could take part as partners in future PESI-related projects and even take a lead in WP. Currently we don’t have the capacity to serve as a coordination center for maintenance of the FP network outside specific project.

8. What would in your view a continuity plan of the Focal Point Network comprise beyond PESI (establishment of a formal Focal Point Organisation, involvement in LifeWatch, association with GBIF, other)?
Perhaps the most sustainable move will be if the Focal Point Network joint LifeWatch.

9. Please feel free to communicate any other ideas, suggestions, comments.

Appendix 17 — Individual Focal Point Sustainability Plan — UCPH — Partner 2

Institute: University of Copenhagen

Contact person: Phillip Bøgh

1. Please give a short description of your sustainability plan in view of your local/national situation

The Danish national node DanBif has at least 2½ more years, and the Nature Agency of Denmark has funded 1 month further adding species to the Allearter.dk checklist. No LifeWatch is yet funded.

2. Please describe in what way your Focal Point activities (see above key functions and roles) will be sustained at national level in the near future

No further sustainability is planned.

3. How has PESI contributed to:

– the sustainability of your national Focal Point status

The national information has been distributed (e.g.,to WP3), the Danish experts has been informed of the FP project, LifeWatch DK knows it exist. But a final PR – e.g., an email to all experts would improve the knowledge about interesting links.

– the connection with other European or national biodiversity initiatives

Please see above about DanBif, LifeWatch DK and Nature Agency of Denmark

– the quality and use of biodiversity information both at a national and European scale

PESI has started the process of collecting the date – except Allearter.dk that would have appeared anyway. The quality is, however, in a start-up level.

4. Which specific support from PESI would in your view improve the contacts with European and/or national biodiversity initiatives, including liaison with LifeWatch?

IF any funding comes up in DK it would be important to get:

→ an automatic mapping/CSS from Allearter.dk;

→ an automatic mapping/CSS redlists, blacklists, fugleognatur.dk...

→ a training facility and

→ a way to involve the national and EUtaxonomists;

5. What support would you require from PESI (in cooperation with LifeWatch) to ensure that your FP activities gain priority on the national political Agenda?

→ A mapping/CSS facility to EU databases

→ salary to a person to start national communication-skills and the training facility

6. Would you like to continue the national Focal Point activities after the project timespan?

→ Yes, but no funding is available and till now FP has only been extra labor.

7. Would you like to coordinate/manage on a more general basis the activities of grouped European Focal Points?
Yes

8. What would in your view a continuity plan of the Focal Point Network comprise beyond PESI (establishment of a formal Focal Point Organisation, involvement in LifeWatch, association with GBIF, other)?
→ Anyone must take this as the main area for a period.
→ Someone responsible to attract voluntary labour
→ Someone to combine Allearter.dk database and the Fugleognatur.dk communication site – e.g., through the training facility.

9. Please feel free to communicate any other ideas, suggestions, comments.

Appendix 18 — Individual Focal Point Sustainability Plan — IBSS — Partner 4

Institute: A.O. Kovalevsky Institute of Biology of the Southern Seas of National Academy of Sciences of Ukraine (IBSS NASU)

Contact person: Dr Volodymyr Vladymyrov

1. Please give a short description of your sustainability plan in view of your local/national situation

Ukrainian Focal Point for marine species that is not the official legal body is based at the Laboratory of Marine Information Systems of the Institute of Biology of the Southern Seas of National Academy of Sciences of Ukraine. This Laboratory hosts as well UNESCO IOC/IODE Ukrainian National Oceanographic Data Center. Recently the support of the staff dealing with the Focal point issues is being provided from the budget of the host institution. The IBSS, former the Marine biological station exist in Sevastopol, Ukraine, already 140 years and one can suppose that it will be supported by the country in future as well.

The checklists for the Black Sea created within the PESI project attained big interest of the regional marine biologist and they are going to support this initiative in future.

2. Please describe in what way your Focal Point activities (see above key functions and roles) will be sustained at national level in the near future

As it is mentioned above under the bullet 1, the Focal Point activities will be sustained at national level due to the expertise and resources of the host institution (IBSS NASU) and Ukrainian National Oceanographic Data Center – Biology.

3. How has PESI contributed to:

- the sustainability of your national Focal Point status
- the connection with other European or national biodiversity initiatives
- the quality and use of biodiversity information both at a national and European scale

PESI has contributed to the sustainability of our national Focal Point status recognizing us as the National Focal Point for the marine species.

Check lists created with the PESI support are in the basis of development of the quality control procedures for marine biological data in the Black Sea region.

4. Which specific support from PESI would in your view improve the contacts with European and/or national biodiversity initiatives, including liaison with LifeWatch?

This could be discussed probably as well with the Commission on the Protection of the Black Sea Against Pollution (the Black Sea Commission).

5. What support would you require from PESI (in cooperation with LifeWatch) to ensure that your FP activities gain priority on the national political Agenda?

First of all we need the involvement of Ukraine in the LifeWatch, that is not a case yet.

6. Would you like to continue the national Focal Point activities after the project timespan?
Yes, we would like to continue the national Focal Point activities after the project timespan

7. Would you like to coordinate/manage on a more general basis the activities of grouped European Focal Points?
Yes, we would like to coordinate/manage on a more general basis the activities of grouped European Focal Points for the Black Sea region.

8. What would in your view a continuity plan of the Focal Point Network comprise beyond PESI (establishment of a formal Focal Point Organisation, involvement in LifeWatch, association with GBIF, other)?
Involvement in LifeWatch would be in our mind the main point of a continuity plan of the Focal Point Network.

9. Please feel free to communicate any other ideas, suggestions, comments.
The network of the Focal points should be more user oriented including the public in large. Anybody should find the necessary biodiversity information, applying to this network. For example, user looking at the unknown species could find all information related to this species within the Focal points network. So the network should be useful as for house wife looking for the name of the unknown fish till scientist trying to recognise the unknown for him species of phytoplankton.

Appendix 19 — Individual Focal Point Sustainability Plan — NKUA — Partner 18

Institute: National & Kapodistrian University of Athens

Contact person: Anastasios Legakis

1. Please give a short description of your sustainability plan in view of your local/national situation

The team that has worked for the project will continue to collect data on species checklists and vernacular names, expertise, taxonomic resources and taxonomic projects. It will contribute towards projects that bring together taxonomists at the national level. It will disseminate information basically through the Internet as well as in local and national congresses, conferences, meetings or workshops. Finally it will rally for a greater share of funding of taxonomic activities.

2. Please describe in what way your Focal Point activities (see above key functions and roles) will be sustained at national level in the near future

The Focal Point activities will be sustained through the cooperation of the scientists that are involved in taxonomic, biogeographical, ecological or other kinds of work, as well as through the relevant scientific societies that exist in Greece.

3. How has PESI contributed to:

– the sustainability of your national Focal Point status
– the connection with other European or national biodiversity initiatives
– the quality and use of biodiversity information both at a national and European scale

The participation in PESI allowed the compilation for the first time, of data and information that had been scattered in various sources. This compilation can assist in the documentation of the status of biodiversity in Greece and therefore, in the taking of measures for its conservation and sustainable use. At the same time, PESI has helped in strengthening ties with institutions in other European countries that are carrying out similar tasks.

4. Which specific support from PESI would in your view improve the contacts with European and/or national biodiversity initiatives, including liaison with LifeWatch?

The creation of common European lists of species, projects, specialists, databases, tools etc. will certainly support and enhance cooperation of national and European scientists and initiatives.

5. What support would you require from PESI (in cooperation with LifeWatch) to ensure that your FP activities gain priority on the national political Agenda?

Taxonomy has a very low status in the eyes of the people that are involved in political agendas. The fact that the EU has funded a project on making taxonomic data available to the

public will in itself help raise awareness and will help linking this kind of data with the conservation and sustainable use of biodiversity.

6. Would you like to continue the national Focal Point activities after the project timespan?
The team that has worked for the project will continue its Focal Point activities after the end of the project. These activities were already taking place before the start of the project both at the national and the European level in projects such as Fauna Europaea and ERMS.

7. Would you like to coordinate/manage on a more general basis the activities of grouped European Focal Points?
We would be interested in managing or coordinating activities in thematic Focal Points if these could be established in cooperation with initiatives such as SMEBD.

8. What would in your view a continuity plan of the Focal Point Network comprise beyond PESI (establishment of a formal Focal Point Organisation, involvement in LifeWatch, association with GBIF, other)?
As a continuation of PESI I would envisage the creation of a thematic Focal Point Network based on taxonomic groups. Several European societies exist for a number of animal or plant groups such as Societas Europaea Mammalogica, Societas Europea Herpetologica, European Bird Census Council, European Invertebrate Survey etc. For other groups, there is no coordination and sometimes very little contact.
Involvement in LifeWatch and contributions to GBIF are essential in continuing the dissemination of taxonomic information.

Appendix 20 — Individual Focal Point Sustainability Plan — ABG — Partner 00

Institute: Azorean Biodiversity Group (University of Azores) - Non-contracted Focal Point

Contact person: Paulo A. V. Borges

1. Please give a short description of your sustainability plan in view of your local/national situation

There is a growing interest in academia to provide biodiversity data to both the scientific community and the public (see also the European Directive INSPIRE; http://inspire.jrc.ec.europa.eu/). Currently the total number of terrestrial *taxa* (species and subspecies) in the Azores is estimated of about 6,164 (about 6,112 species). The inclusion of an exhaustive listing of non breeding species and a preliminary list of potentially breeding species adds 332 species and subspecies of birds to the Azorean list of species. Most of these species are already mapped in a scale of 500x500 m using the Software ATLANTIS 2.0. Some of the data stored in Azorean ATLANTIS database is now being made universally available through an internet interface, the Azorean Biodiversity Portal (ABP,http://www.azoresbioportal.angra.uac.pt/), which presents a wealth of resources not only for each Azorean species, but also for Macaronesian biodiversityCurrently the total number of coastal marine taxa (species and subspecies) in the Azores is estimated of about 1,885 taxa belonging to 16 Phyla. However, these organisms are not adequately mapped.

The information currently available in the Azorean ATLANTIS database and in the Azorean Biodiversity Portal includes very few data on coastal and marine invertebrates and vertebrates. In addition now there is available a new platform, ATLANTIS 3.0 to manage the current database on Macaronesian biodiversity. The two main advantages of this new platform are the fact that i) both the feeding platform and the web platform are web-based and ii) unlike ATLANTIS 2.0, it can accommodate marine data.

Therefore, the main aims of the current project are:

1) To migrate the current Azorean terrestrial and coastal database ATLANTIS 2.0 to the new web-based platform ATLANTIS 3.0.
2) To gather , as extensively as possible, the biodiversity literature and unpublished reports on Azorean marine invertebrates and vertebrates and digitize it into the new ATLANTIS 3.0 database;
3) To create a completely new version of the Azorean Biodiversity Portal to include the marine habitats and new facilities of utility for the Azorean Government and general public.

2. Please describe in what way your Focal Point activities (see above key functions and roles) will be sustained at national level in the near future.

Our research will provide an important contribution to the selection and/or refinement of “in situ” Azorean coastal and marine protected areas through the comparison of diversity values. Our research program will generate comparative data on species and communities at both

intra- and inter-island scales. Our program of close liaison with policy and decision-makers will ensure that the information generated by the project can be implemented in the establishment of future research and conservation priorities.
The science community will benefit from our research program by access to our work through publication in high impact peer reviewed journals, and access to published data made available on Azorean Biodiversity Portal and other international Biodiversity public data bases (e.g. GBIF).
All the information on the final database will be available for the Azorean Government for management of the Azorean Marine Park.

3. How has PESI contributed to:
– the sustainability of your national Focal Point status.
– the connection with other European or national biodiversity initiatives.
– the quality and use of biodiversity information both at a national and European scale.
PESI has contributed to the sustainability of our regional Focal Point status recognizing us as the Azorean National Focal Point for the terrestrial species.

4. Which specific support from PESI would in your view improve the contacts with European and/or national biodiversity initiatives, including liaison with LifeWatch?
Have no idea at the moment

5. What support would you require from PESI (in cooperation with LifeWatch) to ensure that your FP activities gain priority on the national political Agenda?
First of all we need the involvement of Azores in the LifeWatch, that is not a case yet.

6. Would you like to continue the national Focal Point activities after the project timespan?
Yes, we will continue the regionalFocal Point activities after the project timespan. By providing unrestricted, detailed information on the distribution of Azorean species using the Azorean Biodiversity Portal, we will continue contributing to conservation efforts in the Azores. Government managers frequently consult the Azorean Biodiversity Group to obtain data on individual species distributions or maps of species richness for the Terrestrial realm, and such information has been used to reshape the boundaries of protected areas of the region, both terrestrial and coastal.

7. Would you like to coordinate/manage on a more general basis the activities of grouped European Focal Points?
Yes, we would like to coordinate/manage on a more general basis the activities of grouped European Focal Points for the Azores and even Portugal mainland.

8. What would in your view a continuity plan of the Focal Point Network comprise beyond PESI (establishment of a formal Focal Point Organisation, involvement in LifeWatch, association with GBIF, other)?
Presently, the ATLANTIS Azores database is not connected with GBIF. We intend to make this link, assuring that the distributional data already registered in the Azorean Biodiversity Portal and the Atlantis database, and the data gathered by this new project, is made available

to the world through the GBIF data portal. This will allow the internationalization and recognition of the regional databases at a global level. It will also allow completing the scarce information existing nowadays at GBIF regarding the Azorean species, especially the endemics, and to promote future scientific collaboration with different partners

9. Please feel free to communicate any other ideas, suggestions, comments.

Appendix 21 — Individual Focal Point Sustainability Plan — HCMR — Partner 34

Institute: Hellenic Centre for Marine Research

Contact person: Christos Arvanitidis

1. Short description of the sustainability plan:
The sustainability plan includes the following components: (a) the defragmentation of the Greek scientific community working on various aspects of marine biodiversity under a common framework of activities, (b) the further development and maintenance of the taxonomic observatories, (c) to assist the Zoological Museum of the National and Kapodestrian University of Athens to build the national GBIF node, (d) the creation and operation of e-Fora, e-Conferences and other means of communication, (e) the liaison with the relevant stakeholders at local, regional and national level, (f) the submission of marine biodiversity oriented proposals.

2. Sustainability of the Greek marine Focal Point activities:
The sustainability at national level has been discussed in a number of meetings with the national marine biodiversity community in the context of the preparation phase of the national LifeWatch node. The project manager, who heads the PESI activities for the Greek marine FP, visited the involved institutes and academic departments all over Greece and they agreed upon the plan presented above. All of the partners agreed to continuously support the species lists delivered so far and even to develop those for the currently missing taxa. HCMR agreed to undertake the task to maintain any website that cannot be sustained by any other partner. However, further building on the basis of PESI deliverables can only be achieved through the state funding in the years to come. This has been sited at the core of the Feasibility Study and of the proposal submitted to the Greek General Secretariat of Research and Technology (GSRT) for the development and implementation phase of the national node of LifeWatch ESFRI Platform.

3. How has PESI contributed to:
PESI gave the incentive to the Greek taxonomy and biodiversity community to communicate and share a vision. This is happening only for first time in the state. The members of this particular scientific community joined forces to deliver the lists and the latest classification status, along with the remainder deliverables. HCMR, communicated all the plan by these special visits to all other partner institutions.
PESI paved the way to apply standards (e.g. the implementation of the taxonmatch tool) for the deliverables to achieve a certain quality and to be compatible with those delivered by other EU member states and other partner countries. During the PESI FP Workshops HCMR had the opportunity to discuss emerging problems and details with the other partner institutions and to find solutions. Many of the marine FPs delivered their lists to HCMR for a final check before final submission.

PESI filled in a gap by creating a concept in the EU taxonomy community in terms of a set reference tools for the design, development and use of taxonomic information. It brought the members of this community together and made them concentrate and find solutions for the existing problems for the creation of pan-European taxonomy tools. Therefore, mutual understanding, solidarity, and complementarity developed and increased the connectivity and concept exchange between its members. Provided that the sustainability plan will work as anticipated this project will remain as a landmark in the history of taxonomy in Europe from now on.

4. Which specific support from PESI would in your view improve the contacts with European and/or national biodiversity initiatives, including liaison with LifeWatch?
There are two issues which PESI must shoulder responsibility for: (a) the maintenance of the tools developed, so that the national communities would use as reference tools and base their efforts for the development of new ones, (b) implement mechanisms to keep the European taxonomic community allied through other activities in the context of other projects such as the World Register of Marine Species (WoRMS) and LifeWatch. Plurality and complementarity are two of the emerging attributes of this community that may help it to survive in the years to come.

5. What support would you require from PESI (in cooperation with LifeWatch) to ensure that your FP activities gain priority on the national political Agenda?
PESI has already been involved in our FP activities to take priority on the national agenda through our LifeWatch national proposal. Political pressure was one basic step which has been already been taken through multiple meetings with the GSRT officers in Athens. We have proved to the officers that our convincing argument is that massive assemblage of the network of scientists from all over the state and which provides us with a good priority rank over the other proposals submitted. PESI was instrumental to provide the first common and solid basis for this community to start the dialogue.

6. Would you like to continue the national Focal Point activities after the project timespan?
Yes, this is our plan in HCMR.

7. Would you like to coordinate/manage on a more general basis the activities of grouped European Focal Points?
Yes, and we have already started to by collecting more datasets from all over the Mediterranean Sea Countries.

8. What would in your view a continuity plan of the Focal Point Network comprise beyond PESI (establishment of a formal Focal Point Organisation, involvement in LifeWatch, association with GBIF, other)?
The first steps have already taken. These are: (a) the EU project ViBRANT (e-Infrastructures), which will create the infrastructure for taxonomic work by following all necessary steps from the design and sampling all the way up to scientific publications, and

which encompasses much of the work done in PESI and brings it to the forefront of the technological development in e-infrastructures; (b) the ESFRI Platform LifeWatch, which is anticipated to be approved by at least a number of the participating EU states this year and so to start its development and implementation phase; (c) a number of other projects such as the EMODNET which can offer some resources to keep this community allied around the achievement made over PESI.

9. Please feel free to communicate any other ideas, suggestions, comments.
The overall scope of this large taxonomic Consortium is to keep it in functioning and this can only be through the communication in the context of following-up projects such as those mentioned in the above points. WoRMS can offer a solid basis for the marine taxa experts to continue to be active and to deliver reliable taxonomic information, which can subsequently be installed on the web tools assisting hundreds of other scientists not only in Europe but all over the world.

Appendix 22 — Individual Focal Point Sustainability Plan — CUB — Partner 23

Institute: Comenius University, Bratislava, Slovakia

Contact person: Eduard Stloukal

1. Please give a short description of your sustainability plan in view of your local/national situation

Comenius University maintains the information system on taxonomy and diversity of fauna in Western Carpathian and northern Pannonian regions of Central Europe. Supported by project of European structural funds, we build together with other institution in Slovakia, the information system on biodiversity on the national scale.

2. Please describe in what way your Focal Point activities (see above key functions and roles) will be sustained at national level in the near future

We do:

- Maintain the national taxonomic expertise network
- Collate and unveil local and regional species inventories
- Support, maintain and update the national species checklists
- Make checklists available to global community of potential users
- Develop and distribute and demonstrate tools (including the PESI tools) to national users
- Communicate with policy makers regarding country-specific prioritised species
- Assist in environmental policy-making regarding threatened species or pests
- Liaise with governmental bodies on implementation of European standards relevant to regulation and environmental monitoring
- Document local expertise and applied tools for the greater European taxonomic community
- Hold workshops on key topics like: data management; validation national species checklists; cooperation with national policy makers

We contribute to PESI (when the citation of data will be solved):

- Inventory and delivery of national prioritised species checklists
- Vernacular names of prioritised species in national and EU legislation
- Translations for a multilingual portal
- Application for national funds regarding Focal Point outreach
- Contribution to the Focal Point Handbook

We wish to contribute to PESI (when the citation of data will be solved):

- Inventory of major regional checklists and species data
- Inventory and delivery of national prioritised species checklists

- Vernacular names of prioritised species in national and EU legislation

3. How has PESI contributed to:
– the sustainability of your national Focal Point status
– the connection with other European or national biodiversity initiatives
– the quality and use of biodiversity information both at a national and European scale

4. Which specific support from PESI would in your view improve the contacts with European and/or national biodiversity initiatives, including liaison with LifeWatch?
No specific support

5. What support would you require from PESI (in cooperation with LifeWatch) to ensure that your FP activities gain priority on the national political Agenda?

6. Would you like to continue the national Focal Point activities after the project timespan?
We shall continue.

7. Would you like to coordinate/manage on a more general basis the activities of grouped European Focal Points?

8. What would in your view a continuity plan of the Focal Point Network comprise beyond PESI (establishment of a formal Focal Point Organisation, involvement in LifeWatch, association with GBIF, other)?

9. Please feel free to communicate any other ideas, suggestions, comments.

Appendix 23 — Individual Focal Point Sustainability Plan — CSIC — Partner 29

Institute: Museo Nacional de Ciencias Naturales (Fauna Ibérica)

Contact person: Dr. Marian Ramos

1. Please give a short description of your sustainability plan in view of your local/national situation

The sustainability plan for PESI is largely depending on the sustainability of Fauna Ibérica whose Leader has been the PESI Focal Point for the Ibero-balearic fauna. Fauna Ibérica is a research project funded since 1989 by the R+D+I Spanish National Plans (each project runs for 3 years). Dr. Ramos is the project Leader of Fauna Ibérica. The bi-directional collaboration between PESI and Fauna Ibérica has been very positive and could be enhanced in the future as follows: a) IBERFAUNA, the database of Fauna Ibérica increases the information compiled and b) if new tools are developed to facilitate bi-directional exchange of information.

2. Please describe in what way your Focal Point activities (see above key functions and roles) will be sustained at national level in the near future

The networking role of Fauna Ibérica has been largely proved, as well as its training capacity (many PhD on taxonomy).

Regional inventories exist in Spain compiled by Regional Governments although they deal with distributional data and has not been among the objectives of Fauna Ibérica. The movilization of such data would be more easily done within the framework of the LifeWatch ERIC.

The main contribution of Fauna Ibérica to PESI could be to update the current information on the PESI databases for the endemic taxa (near 50% of European endemics). A tool allowing this will be very necessary in order to update and to improve the PESI databases.

Regarding other key functions (building awareness, communicating and advising policy makers on prioritised species, pest species, etc.) will continue as Fauna Ibérica has always done.

3. How has PESI contributed to:

– the sustainability of your national Focal Point status

The international collaboration of Fauna Ibérica favours the sustainability of the project and supports its need at national level.

– the connection with other European or national biodiversity initiatives

PESI databases will be a key element in the data platform of LifeWatch

– the quality and use of biodiversity information both at a national and European scale

PESI databases are essential tool for curators of scientific collections and a reference for other users

4. Which specific support from PESI would in your view improve the contacts with European and/or national biodiversity initiatives, including liaison with LifeWatch?
As already said in 3, PESI has to be a key element in the data platform of LifeWatch. In addition, PESI could help to hightlight the need of high quality taxonomic databases and to lobby on the need of: 1) creation of new knowledge (research), 2) training new generation of taxonomists and 3) linking the community of the private taxonomists.

The role of PESI could be crucial to avoid a biass of LifeWatch towards its ecological level. The need to keep focus on genes, species in addition to ecosystems should be highlighted.

5. What support would you require from PESI (in cooperation with LifeWatch) to ensure that your FP activities gain priority on the national political Agenda?
The same that for point 4, but at national level. That is, keep focus on the 3 levels of biodiversity, not forgetting species nor considering this level as a "service" to the other 2.

6. Would you like to continue the national Focal Point activities after the project timespan?
Yes

7. Would you like to coordinate/manage on a more general basis the activities of grouped European Focal Points?
No objection

8. What would in your view a continuity plan of the Focal Point Network comprise beyond PESI (establishment of a formal Focal Point Organisation, involvement in LifeWatch, association with GBIF, other)?
I think that a combined approach could be interesting. That is, an organization of Focal Points, linking other national databases and very closely related to GBIF providing high quality data, new knowledge, expertise services (under demand) and within the framework of LifeWatch.

9. Please feel free to communicate any other ideas, suggestions, comments.
Already mentioned my concerns.
As suggestion: the case of countries with many endemics should be discussed in depth in the future because new rules and new tools will be necessary to secure that the information produced in this countries is included within the European databases.
I will be willing to contribute in the future as requested

Configuration History			
**Version No.**	**Date**	**Changes made**	**Author**
0.1	25 March 2011	Initial version	YdJ
0.2	31 March 2011	Inclusion of Focal Points contributions	JK
0.3	31 March 2011	Review JK	JK
0.4	8 April 2011	Review OB (LifeWatch)	OB
1.0	8 April 2011	Final preparation for submission	YdJ
