# Supplementary material for: PESI - a taxonomic backbone for Europe
Source: Biodivers Data J. 2015 Sep 28;(3):e5848. doi: 10.3897/BDJ.3.e5848 (PMC4609752; doi:10.3897/BDJ.3.e5848)
Supplement: Supplementary material 8 — The future of taxonomy – the role of national focal points networks in taxonomic information infrastructure networks [file biodiversity_data_journal-3-e5848-s008.pdf]

Project no. 018340

Project acronym: EDIT

Project title: Toward the European Distributed Institute of Taxonomy

Instrument: Network of Excellence

Thematic Priority: Sub-Priority 1.1.6.3: "Global Change and Ecosystems"

M3.2.3a The future of taxonomy – the role of national focal points networks in taxonomic information infrastructure networks

First scoping meeting on focal points involvement

Due date of component: Month 28
Actual submission date: Month 29

Start date of project: 01/03/2006 Duration: 5 years

Organisation name of lead contractor for this component: 5 UvA

Project co-funded by the European Commission within the Sixth Framework Programme (2002-2006)		
**Dissemination Level ("X" in the relevant box)**		
**PU**	Public	
**PP**	Restricted to other programme participants (including the Commission Services)	
**RE**	Restricted to a group specified by the consortium (including the Commission Services)	X
**CO**	Confidential, only for members of the consortium (including the Commission Services)	

Revision 1

Table of Content

Table of Content 2

Conference invitation 3

Conference agenda & guidelines 6

- Venue 6
- Attendance 6
- Programme (draft) 6
- Introduction 8
- Organising the national focal points networks 8
- Meeting Report Guidelines 11
  - Organisation: 11
  - Local resources: 11
  - Reviewing / validation: 11
  - Taxonomic standards: 11
  - Techniques: 12
  - E-taxonomy: 12

Conference discussion notes 13

- Network organisation / arrangements 13
- Cross-validation 14
- Facilitate local Focal Points set up 14
- Local expertise resource allocation 14
- Additional data types 14
- Establish and disseminate taxonomic standards 15
- Interoperability 15
- Networking 15
- Structure of the network: 16
- Non national members: 16
- Meeting presentations: 17

Configuration History 18

European Distributed Institute of Taxonomy (EDIT) (www.e-taxonomy.eu)
WP3.2 - taxonomic information infrastructure network

The future of taxonomy – the role of focal points in taxonomic information infrastructure networks

A Conference of the European Distributed Institute of Taxonomy

Congress Centre, Smolenice Castle, Slovakia
28-30 March 2007

Dear representatives of national focal points, Dear EDIT partners, Dear others,

We are delighted to invite you to partake in the conference "The future of taxonomy - the role of national focal point networks", March 28-30, 2007 in Smolenice, Slovakia.

The European Distributed Institute of Taxonomy (EDIT) is a network of leading European institutions in the area of research on the taxonomy of organisms[1].

One of the important activities within the EDIT workpackage on *the taxonomic information infrastructure network* (WP 3.2) is the set up of a Pan-European species-directories infrastructure to fulfil Europe's contribution to worldwide species-list initiatives by establishing a secure organisation and management for Europe's species databases and repositories, which include:

- ◇ developing an integrated pan-European species databases e-infrastructure,
- ◇ establishing improved research collaboration through the set up of a common management approach for the maintenance and updating of European species-lists by:
  - leading the expert community starting to think and work as one entity with respect to task division and research standards
  - improving the organisation and (efficient) access to the collective 'knowledge memory', research expertise and repositories content
  - arranging national or regional focal points into a partnership structure
- ◇ bringing the taxonomic (digital) data content to a next (higher) level of completeness, quality, consistency, integration and incorporation.

The aim of this EDIT conference is to provide a forum to discuss the involvement of national focal points into this taxonomic information infrastructure network to:

- ◇ support the European taxonomic standardisation efforts; the so-called 'reaching of excellence', including the updating and validations of the current major (authoritative) checklists FaEu, ERMS and EMP,

---

[1] For EDIT, "Taxonomy" is interpreted in a very broad sense, i.e., including not only description and classification of species, but also phylogeny, intraspecific variation, and the construction and use of identification tools (see http://www.e-taxonomy.eu/files/the_systematist.pdf)

◇ support the further institution of taxonomic standards within Europe; the so-called 'spreading of excellence', including their dissemination and implementation.

Examples of themes and topics to be discussed include:

- preparing a road map for a further institution of taxonomic standards for Europe, to solve the instability and inconsistency of species names (and concepts) among and within European countries; practically and principally,
- supporting the set up of taxonomic authority files on relevant additional data types (e.g. author names), also assisting the cybertaxonomy work process (EDIT WP 6&7) and backing the authorities of nomenclators like ZooBank,
- involving taxonomic societies within the process of taxonomic standardisations and the assembly of authority files,
- involving key-users (like IUCN, ICES, CBD, EU-directives and EPPO) to approve (e.g. by certification) on taxonomic standards,
- implementation of the (authoritative) EU taxonomic standards in local biodiversity systems, by means of the above efforts,
- validating the European checklists by cross-checking with regional lists and reviewing European lists with help of local experts (including the set up of validation routines and protocols),
- using the context of the European checklists programs to establish national lists,
- cross-referencing of the European lists to species details in local biodiversity systems,
- assembling details on local expertise as input for EDIT WP 2 on-line expert(ise) information service,
- discussing the role of molecular markers (like DNA barcodes) for taxonomic identification,
- providing or giving access to or supporting the collecting of additional info and data types on European species, for instance:
  - common names
  - detailed faunistics
  - conservation status.

Some practical focal point matters to be considered will include:

- focal point network organisation and management (for FaEu),
- focal point network criteria and identification (for ERMS and EMP),
- examples of working national focal points .

We would like to bring representatives of (potential) national or regional focal points in Europe, EDIT partners, and other affianced initiatives together for discussion. That is why we would like to invite you to take part in this conference.

We welcome your suggestions for further topics to be discussed at the meeting. Details on the programme will be circulated a few weeks before the meeting. Every participating focal point representative is requested to give a short overview of his/her focal point state of the affairs in a round table arrangement (guidelines will follow).

Further information on the conference and its venue, as well as the registration form, can be found at website http://zoology.fns.uniba.sk/edit (link "Meetings"). Please notice the deadline for registration is February 25, 2007.

At least part of the conference costs, including travel and accommodation, will be covered by EDIT (for details look at the website).

We would also like to call your attention to another meeting, to be held back-to-back with our conference, viz. "The future of taxonomy – the role of societies and networks", to be held in Smolenice 25-30 March (same web link); see also the EDIT WP 2 meeting documents.

Yours sincerely

Yde de Jong, EDIT WP 3.2 coordinator

Eduard Stloukal, EDIT WPs 2 and 3.2 activity leader & local organiser

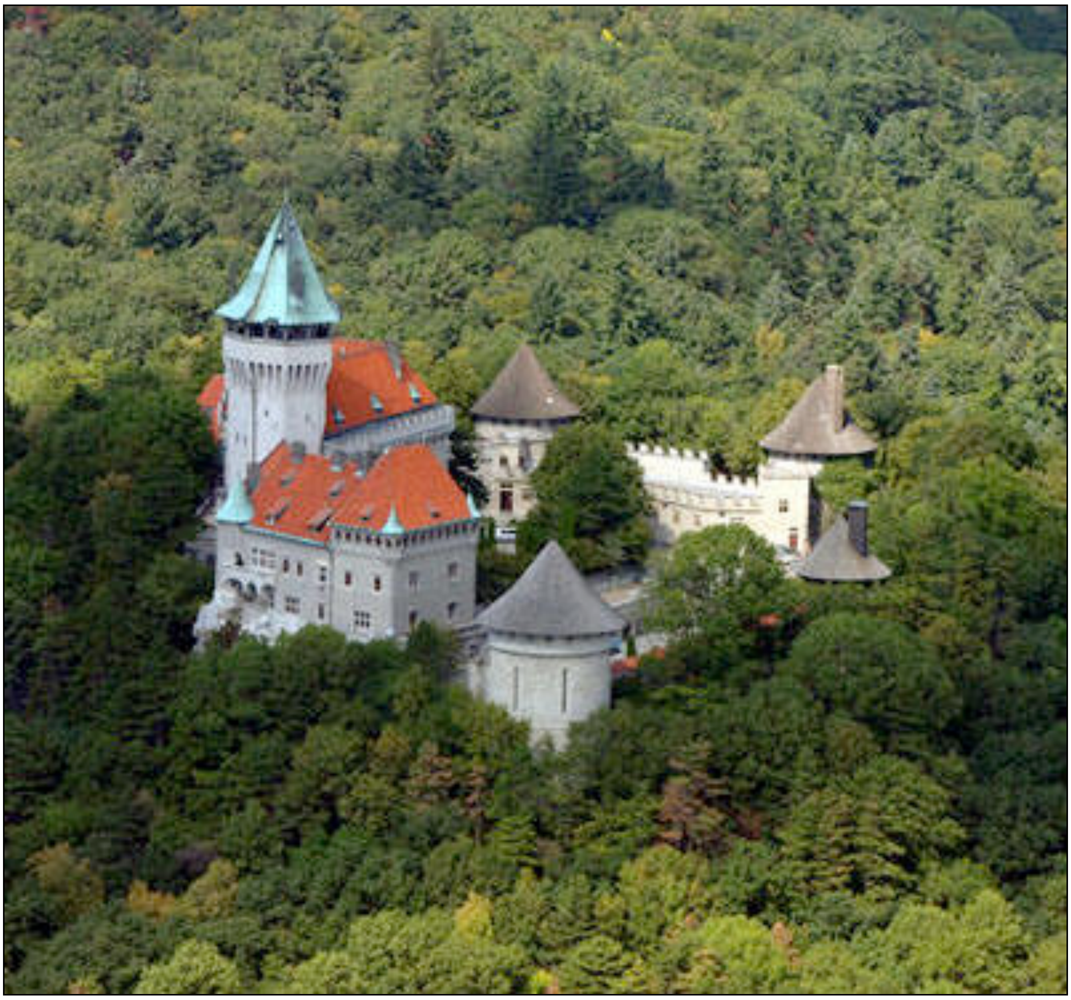

Eduard Stloukal
Department of Zoology, Comenius University, Bratislava, Slovakia
stloukal@fns.uniba.sk; mobile +421-905-570149; phone/fax +421-2-60296333

European Distributed Institute of Taxonomy (EDIT) (www.e-taxonomy.eu)
WP3.2 - taxonomic information infrastructure network

The future of taxonomy - the role of focal points in taxonomic information infrastructure networks

A Conference of the European Distributed Institute of Taxonomy

28-30 March 2007

Venue

Congress Centre – Smolenice Castle
Zámocká 18
919 04 Smolenice
Slovakia
http://www.kcsmolenice.sav.sk

Attendance

National Focal Point representatives: {final list in preparation}

Invited speakers: Benoît Fontaine, Otto Moog, Andrew Polaszek

EDIT project representatives: {final list in preparation}

Programme (draft)

Wednesday 28th March 2007: Arrival of participant

Thursday 29th March 2007: Preliminary agenda:

9.00	*Conference opening*	*{Slovak executive}*
9.05	*Welcome by host*	Ed Stloukal
	**Session 1 - EDIT & pan-European checklists**	Chair: ...
9.10	*Introduction to EDIT*	Wouter Los
9.30	*Introduction to EDIT WP 3.2*	Yde de Jong & Wouter Los
9.45	*Euro+Med PlantBase - State of affairs*	Eckhard von Raab-Straube
10.00	*ERMS - State of affairs*	Edward vanden Berghe & Ward Appeltans
10.15	*Fauna Europaea - State of affairs*	Yde de Jong
10.30	*Coffee break*	
	**Session 2 - Briefing of focal points**	Chair: ...
10.50	*National Focal Points reports*	NFP representatives
13.00	*Lunch*	
14.00	*National Focal Points reports (continued)*	NFP representatives
16.00	*Tea break*	
16.20	*National Focal Points reports (continued) and initial discussion*	NFP representatives
~ 19.30	*End of meeting day*	
20.00	*Evening program (dinner)*	

Friday 30th March 2007: Preliminary[2] agenda:

8.50	*Start of meetings second day*	
	**Session 1 - EU taxonomic standards**	Chair: ...
9.00	*Towards EU taxonomic standards - the example of AQEM-STAR*	Otto Moog
9.25	*Perspectives on taxonomic standards within Zoology - ZooBank*	Andrew Polaszek
9.50	*Discussion on EU taxonomic standards*	
10.15	*Coffee break*	
	**Session 2 - EU taxonomic gaps**	Chair: ...
10.45	*Gaps in EU taxonomic knowledge - Fauna Europaea gap analysis*	Benoît Fontaine
11.10	*Gaps in EU taxonomic expertise*	{not confirmed yet}
11.30	*Gaps in EU taxonomic knowledge and expertise - Fauna Palearctica* (optional talk)	Yde de Jong, Sergei Medvedev & Natalia Ananieva
11.50	*Discussion on taxonomic gaps*	
12.30	*Lunch*	
	**Session 3 - E-infrastructures perspectives**	Chair: ...
14.00	*Perspectives on e-infrastructures - Cybertaxonomy (EDIT WP 5)*	Malte Ebach & Walter Berensohn
14.30	*Perspectives on e-infrastructures - EU Framework 7 program* (optional talk)	Yde de Jong & Edward Vanden Berghe
	**Session 4 - General discussion**	Chair: ...
15.00	*Concluding discussion*	
16.00	*Tea break*	
16.20	*Concluding discussion (continued)*	
17.30	*End of meeting & participants (early) depart*	
20.00	*Evening program (dinner)*	

Saturday 31th March 2007: Remaining participants depart

For information on the optional social programme, visit the web site of the meeting.

Meeting website: http://zoology.fns.uniba.sk/edit

[2] The mentioned schedule and titles are only indicative. A final agenda will be distributed at the first meeting day. Most important parts of the meeting will be the briefing of the focal points and concluding discussions. To fullfill these objectives the timetable will be treated in a flexible way. Talks labeled as 'optional' could for instance be skipped when needed for time extension.

Introduction

A joint EDIT objective is to advance the set up of an infrastructure of European species databases to fulfil Europe's contribution to worldwide species list initiatives by establishing a secure organisation and management for European biodiversity information databases and repositories, including the founding of a pan-European checklist. This objective covers a range of EDIT ambitions in common with:

- ◇ developing an integrated pan-European species databases e-infrastructure,
- ◇ establishing improved research collaboration through the set up of a common management approach for the maintenance and updating of European species-lists,
- ◇ bringing the taxonomic (digital) data content to a next (higher) level of completeness, quality, standardisation, consistency, integration and incorporation.

In general the recognized working program moves along four lines:

- ◇ organising and using the expert(ise) networks,
- ◇ developing and advancing the e-infrastructure,
- ◇ enforcing data validation and (European) standardisation,
- ◇ arranging national or regional focal points into a partnership structure.

Organising the national focal points networks

The national focal point networks contain the local/regional expertise basis supporting certain tasks related to the European species databases infrastructure. The idea behind is a different organisation of taxonomic expertise compared to the framework of experts dealing with the primary data collation:

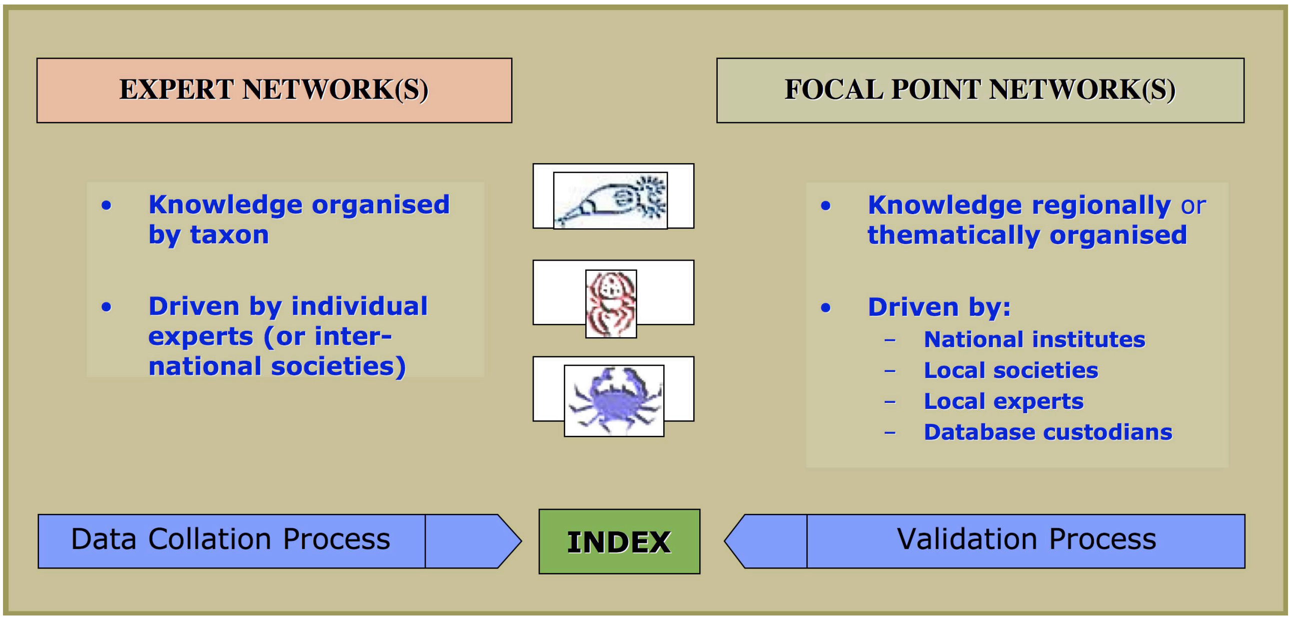

Figure 1: Expert networks and (left) and focal point networks (right); different responsibilities and organizational structure within the taxonomic framework.

Within the NAS-extension part of Fauna Europaea it was possible to identify in each European country a central institute that acted – and in most cases still acts – as a so-called 'Fauna Europaea focal point'[3]. Such focal points gather country specific information about species, species information (databases, literature, etc.), experts, professional societies and others. A dedicated website[4] supporting these focal point network activities became available as an output of the Fauna Europaea NAS extension and will continue as such in the future for subsequent updates. The focal points were also important in the validation process and for the institution of European standards on animal taxonomy (by implementing the results of Fauna Europaea).

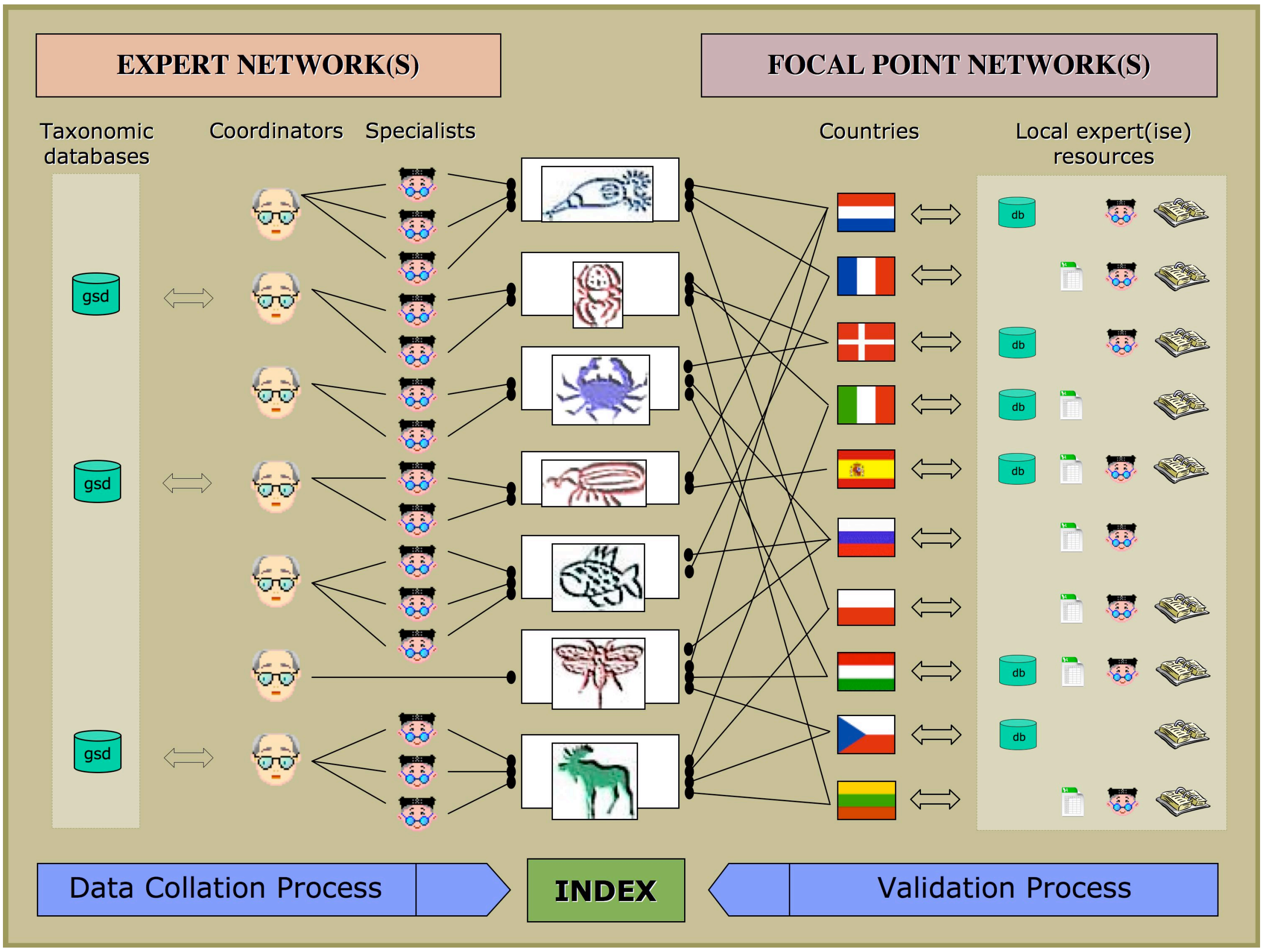

Figure 2: Classical representation of expertise organised in expert networks (left) and focal point networks (right) within Fauna Europaea.

At the moment the continuation and commitment of existing Fauna Europaea focal points is reconsidered and initial steps have been made on further community building, similarly supporting the sister projects *Euro+Med PlantBase* (EMP)[5] and the *European Register of Marine Species* (ERMS)[6]. Besides, as an initial step towards the

---

3 Fauna Europaea focal point network: http://www.faunaeur.org/focal_point.php

4 Fauna Europaea NAS metadata search service: http://zoology.fns.uniba.sk/faeu/search_service

5 Euro+Med PlantBase: http://www.euromed.org.uk

6 European Register of Marine Species: http://www.marbef.org/data/erms.php

enclosure of the Palearctic as a whole, the eastwards extension of the Fauna Europaea focal point network (starting with Georgia and the Ukraine) is anticipated.

The aim of this EDIT conference is to provide a forum to discuss the involvement of European focal points into the European taxonomic information infrastructure network to support the so-called 'reaching' (through validation) and 'spreading' (through dissemination and implementation) of 'excellence'. This also includes the further institution of taxonomic standards within Europe to solve the instability and inconsistency of species names (and concepts) among and within European countries, practically and principally. To reach these objectives, several potential focal point tasks (see figure 3) will be considered during the meeting.

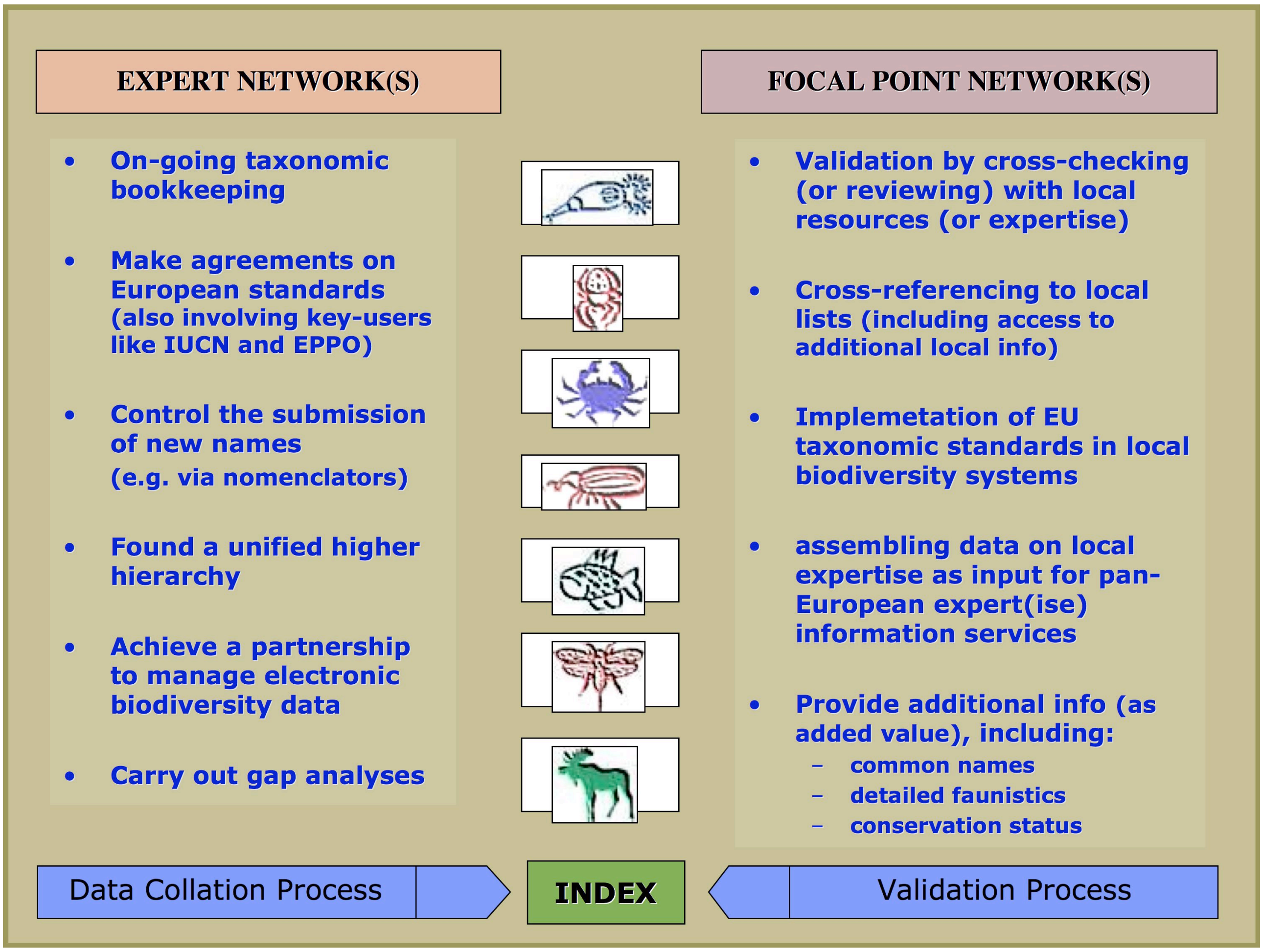

Figure 3: Comparison of potential task division between expert (left) and focal point (right) networks.

The Comenius University Bratislava[7] is in charge for EDIT WP3.2 to take care about the continuation and operation of the national focal point networks efforts within the EDIT context.

[7] Eduard Stloukal <stloukal@fns.uniba.sk>, Department of Zoology, Comenius University, Bratislava, Slovakia, http://zoology.fns.uniba.sk

Meeting Report Guidelines

After some introductions on the European Distributed Institute of Taxonomy (EDIT) and the position of the pan-European checklists within this framework, the first day of the meeting will be occupied by briefing the National (or Regional) Focal Points representatives. Every focal point is requested to give a short (15 to 20 minutes) presentation about the state of affairs within his country (or region). Below an outline of items preferably to be addressed in your presentation.

Organisation:

- What is the current state of affairs and level of organisation of your focal point?
- Does your home institute support the focal point activities?
- What are the future perspectives; could you —for instance— use your EDIT involvement to obtain financial (or otherwise) support?
- Does your country support GBIF and (if so) what is your relationship with your local GBIF-node?
- What is your relationship with other international (DIVERSITAS, CHM, EPBRS, etc.) and local biodiversity programs?

Local resources:

- Does your country have a national species checklist program and (if so) are you involved within this effort?
- What is (was/will) be the role of the European major checklists on establishing a national species list?
- Do you maintain an overview of local expertise resources? How do you assembling details on local expertise and could this information be used as input for other European expert(ise) information services?
- Do you maintain an information system and (if so) what kind of system?

Reviewing / validation:

- Did (or will) you review your local species list(s) by crosschecking it/them with the European checklists or vice verse?
- Did (or will) you involve local specialists in reviewing the European checklists and what (in general) is/was the involvement of local societies and taxonomic working groups within the validation process?
- Would you like to have (technical) support to validate your local checklists?

Taxonomic standards:

- How does your country deal with taxonomic standards for EU rules on environmental issues (ICES, CBD), EU-directives, conservation biology (IUCN),

pest organisms (EPPO), and so on? Does your country agree on certain taxonomic standards for EU regulations?

- Which additional formal steps (in your view) need to be taken to approve on European taxonomic standards?
- Does your country have standard (red) lists on endangered species?

Techniques:

- Did (or will) you implement the existing EU taxonomic standards (=the European authoritative checklists) within your local biodiversity services?
- Did (or will) you cross-reference your species details to the European checklists species details?
- Do your local biodiversity service allow cross-referencing from the European checklists giving access to additional local biodiversity details?
- Which additional info and data types could you provide (common names, detailed faunistics, conservation status, species images, identification keys, etc.)?

E-taxonomy:

- To what extent is your country involved within the ongoing e-taxonomy work process?

The (PowerPoint) presentations will be given in a round table arrangement. Please don't hesitate to include additional suggestions for discussion within your presentation. Final details on the programme will be circulated at the meeting. If we will not be able to end the briefing and discussion at the first day we will continue at the second day.

The second day of the meeting will consider the European taxonomic standardisation efforts from a broader perspective and highlight some future advances.

Looking forward to meet you at Smolenice!

Yde de Jong & Eduard Stloukal

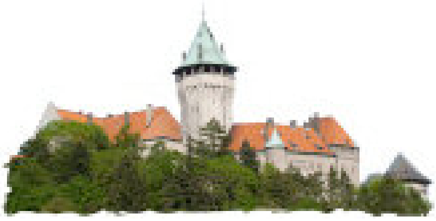

European Distributed Institute of Taxonomy (EDIT) (www.e-taxonomy.eu)
WP3.2 - taxonomic information infrastructure network

The future of taxonomy - the role of focal points in taxonomic information infrastructure networks

A Conference of the European Distributed Institute of Taxonomy

28-30 March 2007

Discussion notes on the work plan drafting

EDIT WP3.2 meeting final discussion notes on the European Focal Points Networks organisation.

Organising the European Focal Points Network

Network organisation / arrangements

- Focal Points Networks set up:
  - network arrangements
  - communication tools
- Partnership agreements of Focal Points Networks.
- Focal Point Networks interim board set up:
  - Olivier Gargominy, Roy Kleukers, Eckhard von Raab-Straube, Edo Stloukal, Chris Emblow, Henrik Enghof, Marian Ramos, Yde de Jong
- EDIT Focal Point Networks board meeting:
  - to be organised by Edo Stloukal from the remaining budget.
- Long-term network set up and maintenance.
  - support collaboration
  - coordinate funding: common businesses plan development.
- Expanding the framework by adding partners from the Palearctic region outside of the EU:
  - contribute to relevant FP7-proposals

Cross-validation

- Define procedures for establishing harmonised European taxonomic metadata standards:
    - contribute to the validation of pan-European checklists by cross-checking and reviewing against local resources and expertise
    - centre of attention validation on 'certified taxa'
    - Gap-analysis

Facilitate local Focal Points set up

- Support focal points with preparation of national lists:
    - use downloads of pan-European checklists
    - checklist tools
    - ensure updates
- Collaborate with GBIF-nodes or establish 'proto nodes' (clearing house):
    - supports GBIF with the set up of a national portals (as most easy way way to access to data in a country)
    - supports GBIF data-sharing activities
- Sharing best practise (Focal Point Handbook):
    - document experiences & best practises
    - output working plan
    - tools, policy, etc.
- Support focal points with preparation of local policy plan.

Local expertise resource allocation

- Set up and maintenance of national networks:
    - define the appropriate national network structure
- Local expertise resource allocation plan:
    - locate & register local taxonomic resources and registers
    - revitalize and upgrade FaEu NAS data management system
- Promotion of local taxonomic resources
- Contribute to GBIF-nodes program:
    - sharing local expertise resource inventory details
    - proto GBIF nodes functioning
- Contribute to expert network set up, allow expert exchange.

Additional data types

- Enriching the European biodiversity data with:

- common names / vernacular names
- detailed occurrence
- phenology data
- (local) conservation status

- Define procedures and tools for European biodiversity data enrichment:
- Contribute to GBIF-nodes program:
  - sharing local resource inventory details

Establish and disseminate taxonomic standards

- Define procedures to disseminate information on taxonomic standards to local databases owners:
  - standardisation of approach for taxonomic databases
- Identification of different opinions on taxonomic hierarchy, especially at the sub- and infra level.
- Agree on taxonomic standards for EU regulations and environmental control:
  - collate information on 'certified taxa' (having a protection status or play a role in habitat directives)
  - support common application of taxonomic information for nature protection
  - survey protocols to approve on European 'certified taxa' list

Interoperability

- On-line share of information between national focal points and central hub.
- Link pan-European checklists with national checklists or local GSDs.
- Include and apply standards and permanent identifieres.
- More detailed distribution map presentation (discrete geo-unit or geo-referencing).
- Localised versions of website(-s).
- English version of national websites.

Networking

- Contact with EDIT:
  - share expertise/excellence of EDIT
  - add local experts to European taskforce
  - contribute to FP7 proposal drafting
  - interaction with users of taxonomic information
  - Link to ATBI infrastructure (www.atbi.eu)
- Contact with legislation bodies (UICN, ICZN, ICBN)

- Contact with standardisation bodies (TDWG, GBIF)
- Contact with GBIF:
  - node committee
  - ECAT committee
- Contact with SYNTHESYS:
  - allow expert & research policy exchange
- Contact with CETAF:
  - allow national/institutional policy exchange

Structure of the network:

- Estonia
- Bulgaria
- UK (in concepts YES)
- Romania
- France
- Poland
- Austria
- Ireland
- Norway
- Spain + Portugal
- Denmark
- Latvia
- Lithuania
- Turkay
- Germany?
- The Netherlands
- Luxemburg?
- Belgium?
- Slovakia (NaTaF)

Non national members:

- SMEBD
- MarBEF

Meeting presentations:

- Aagaard – Organisation in Norway
- Aktac & Viran – European Turkey
- Ardelean – My Nature
- Barsevskis Pankjans – Taxonomy in Latvia: Quo Vadis?
- Budrys – Options on activities of the Lithuanian FaEu focal point
- de Jong – Introduction (Taxonomic Information Infrastructure (integration))
- de Jong – FaEu
- de Jong & Stloukal – Functions of the focal points network
- Ebach – Internet Platform for Cybertaxonomy
- Fontaine – Fauna Europaea What remains to be discovered, where and by whom?
- Gargominy – The use of reference taxonomic databases for the global inventory of French biodiversity
- Kleukers – Focal Point FE - The Netherlands
- Kullander – Managing Taxonomy in Sweden
- Los – EDIT - short presentation
- Martin – Report - Estonia
- Mergen, Adriaens, Verheyen et Liers – The Royal Belgian Zoological Society (RBZS)
- Polaszek – ZooBank
- Ramos – Coordinating Taxonomy in Spain The Fauna Ibérica project
- Schmidt-Kloiber & Moog – Towards EU taxonomic standards - the example of AQEM-STAR
- Tweddle – The National Biodiversity Network
- von Raab-Straube – Euro+Med PlantBase

Meeting presentations and documents can be downloaded at:

- http://zoology.fns.uniba.sk/edit/meetings/wp3-final.htm

  and/or
- http://www.eu-nomen.eu/pesi/index.php?option=com_remository&Itemid=56&func=select&id=77

Yde de Jong & Edo Stloukal

Configuration History			
Version No.	Date	Changes made	Author
0.8	16 February 2007	Invitation	YdJ
0.9	14 March 2007	Agenda & guidelines	YdJ
1.0	2 April 2008	Meeting notes	YdJ

Project no. 018340

Project acronym: EDIT

Project title: Toward the European Distributed Institute of Taxonomy

Instrument: Network of Excellence

Thematic Priority: Sub-Priority 1.1.6.3: "Global Change and Ecosystems"

M3.2.3b The future of taxonomy – the role of national focal points networks in taxonomic information infrastructure networks

Second scoping meeting on focal points involvement

Due date of component: Month 31
Actual submission date: Month 31

Start date of project: 01/03/2006 Duration: 5 years

Organisation name of lead contractor for this component: 5 UvA

Project co-funded by the European Commission within the Sixth Framework Programme (2002-2006)		
**Dissemination Level ("X" in the relevant box)**		
**PU**	Public	
**PP**	Restricted to other programme participants (including the Commission Services)	
**RE**	Restricted to a group specified by the consortium (including the Commission Services)	X
**CO**	Confidential, only for members of the consortium (including the Commission Services)	

Revision 1

Table of Content

Table of Content 2
Venue 3
Contact 3
Hotel 3
Attendance 3
Agenda 4
Circulated documents 4
Previous meeting(s): 5
Minutes / Results: 5
Configuration History 5

European Distributed Institute of Taxonomy (EDIT)

A pan-European Species-directories Infrastructure (PESI)

EDIT WP3.2: The future of taxonomy - the role of focal points in taxonomic information infrastructure networks - second meeting

PESI WP3: Focal Point working group - first meeting

Agenda

Venue

Department of Zoology
Faculty of Natural Sciences
Comenius University
Mlynská dolina B-1 {opposite to the Botanical garden}
842 15 - Bratislava
Slovakia

Contact

Eduard Stloukal (local organiser): + 421 905570149 (GSM)
Yde de Jong: + 31 6 51050565 (GSM)

Hotel

Hotel Ibis Bratislava Centrum {first night}
Zamocka 38
81101 – Bratislava
Slovakia

Hotel Nivy {second night}
Líščie nivy 3
82108 – Bratislava
Slovakia

Attendance

PESI WP3: Nihat Aktaç; Selcuk Yurtsever

FaEu & EDIT: Roy Kleukers; Eduard Stloukal

E+M & EDIT: Eckhard von Raab-Straube; Karol Marhold

ERMS: Christos Arvanitidis; Roisin Nash

PESI WP1 & EDIT: Julia Kouwenberg; Louis Boumans; Yde de Jong

Agenda

Monday 6th October 2008

Afternoon	*Participants arrivals*
17:00 – 18:30 hour	*Informal site seeing Bratislava city*
19:00 – 23:00 hour	*Diner at “Three Musketeers restaurant”* (http://www.trajamusketieri.sk)

Tuesday 7th October 2008

09:00 – 09:15 hour	*Welcome to participants*
09:15 – 12:00 hour	*Focal Points working plan presentation and discussion*
12:00 – 13:00 hour	*Lunch*
13:00 - 17:00 hour	*Continued discussion*
17:00 hour (at last)	*Meeting closure*
Evening	*Participants departures*

Wednesday 8th October 2008

Morning	*Remaining participants departures*

Circulated documents

- Focal Points Work Plan version 2:

http://www.eu-nomen.eu/pesi/index.php?option=com_remository&Itemid=56&func=fileinfo&id=109

- Legislation and national responsibility:

http://www.eu-nomen.eu/pesi/index.php?option=com_remository&Itemid=56&func=fileinfo&id=111

- Target species:

http://www.eu-nomen.eu/pesi/index.php?option=com_remository&Itemid=56&func=fileinfo&id=112

- See also the PESI WP3 folder for other relevant documents:

http://www.eu-nomen.eu/pesi/index.php?option=com_remository&Itemid=56&func=select&id=11

- ....and the Focal Points meeting folder:

http://www.eu-nomen.eu/pesi/index.php?option=com_remository&Itemid=56&func=select&id=77

Previous meeting(s):

- The future of taxonomy - the role of national focal point networks: first meeting, Smolenice Castle, Slovakia on March 29-30, 2007.

  http://www.e-taxonomy.eu/node/720

Minutes / Results:

- The results of this meeting has been integrated into the PESI Focal Point Workplan

  http://www.eu-nomen.eu/pesi/index.php?option=com_remository&Itemid=56&func=fileinfo&id=384

Configuration History			
Version No.	Date	Changes made	Author
0.9	3 October 2008	Draft agenda	YdJ
1.0	5 October 2008	Final agenda & documents	YdJ
