# Supplementary material for: PESI - a taxonomic backbone for Europe
Source: Biodivers Data J. 2015 Sep 28;(3):e5848. doi: 10.3897/BDJ.3.e5848 (PMC4609752; doi:10.3897/BDJ.3.e5848)
Supplement: Supplementary material 9 — PESI Focal Points Working Plan [file biodiversity_data_journal-3-e5848-s009.pdf]

Focal Points Working Plan

1 Introduction

Regional (often national) focal points become increasingly important for the integrated and synergistic promotion of taxonomic expertise and data standards throughout Europe. With their geographical, national and regional focus, focal points differ from traditional expert networks that are organised around a taxonomic group of organisms. The Focal Points contribute to the organisation and management of European biodiversity information in a different, complementary way. Their activities include (1) the liaison with national governmental bodies on the implementation of European standards relevant to, for instance, national and European regulations and environmental monitoring, (2) the collection and transfer of local expertise and applied tools, (3) lobbying and public policy input at the national and European level, and (4) support for closer collaboration of scientific contributor and user communities across Europe. Focal points contribute country/regional-specific information about species, relevant databases, local literature, experts, professional societies and major users such as government organisations.

PESI investigates models to establish a more formal, integrated infrastructure of pan-European Regional Focal Points to enable joint activities. PESI will give new energy to focal point activities, and search for mechanisms to secure resource commitments from national governments. PESI provides a forum to discuss the involvement of focal points into the European taxonomic scientific and information infrastructure, and to coordinate their integrative role in the assignment and confirmation of taxonomic knowledge. These tasks include the coordination of the provision of additional expertise to the pan-European checklists and the arrangement of local commitment to focal point tasks.

To this end, WP3 will investigate the possibility of establishing a more formal Focal Points Network (FPN). As a formal entity, the FPN could facilitate the acquisition of funds and participation in future infrastructural projects. A future FPN can proceed from groundwork carried out in the pan-European checklist programmes.

A Focal Point Handbook will be compiled documenting the methods and experiences of existing Focal Point organisations. The Handbook will facilitate the creation of new focal points, notably in the eastern and southern parts of the Western Palaearctic, as well as assist the organisations and persons that in some cases take over the tasks of an existing focal point.

In PESI WP3 a reservation is made for seed money for Focal Point participants that will be used to involve them in coordination activities, the establishment of the network organisation and infrastructural developments. Such activities, e.g. workshops on data management, cooperation with national policy makers or the application of tools for the cross-linking of web resources, will lead to the spread of best practices. The project allocated a part of the seed money to the partners a priori. In order to maintain transparency, the allocation of the remaining seed money will be done according to pre-established rules.

Because PESI builds on existing networks representing marine, zoological and botanical expert communities with different levels of geographic organisation (regional or national), parallel focal point networks for each of these communities are foreseen for the near future. However, the development of the work plan and performance of non community-specific tasks will be harmonised as necessary. The involvement of national focal points as providers of biodiversity data ties in with several upcoming FP7 initiatives like Life Watch. Therefore by organising the focal point networks and by establishing infrastructures to allow cross-European collaboration, PESI contributes substantially to the development of the European Research Area.

2 Description of Tasks

2.1 Establishment of a Focal Point organisation

Taxonomic Regional Focal Points (FPs) are the interface between the pan-European (and global) taxonomic infrastructure and national or local institutions. As discussed during the EDIT meeting in Smolenice in March 2007[1], it may be useful to organise the network of FPs in a legal form, e.g. an association or trust. This 'European Focal Point Organisation' would facilitate the participation of the FPs in European projects (in analogy with SMEBD) and constitute a central contact point for other organisations like GBIF, IUCN etcetera. The FP Organisation could help selling the expertise of the FPs and support their long-term sustenance. PESI can pave the way for this new organisation.

Discussion points for the PESI General Meeting:

- Purposes and tasks of the FP Organisation
- Its legal form
- Its governance and internal organisation
- Membership or representation of FPs (e.g. by councils)
- Relationship with other national biodiversity information nodes (e.g. GBIF national nodes) and data resource networks
- Its business plan
- Links to the international agenda

How can we profit from the different experiences in FaEu, E+M and ERMS so far? A small number of regional FPs, as in E+M, could be more effective from the perspective of work flow (although the accessibility of faunistic and floristic data is dissimilar), but connections with national database owners is important from the policy perspective and for cross-validation of national and pan-European checklists.

Deliverables: decision on desirability of a central, mediating organisation; if so: formation of a group of volunteers preparing its establishment; documents for the legal establishment. The FP Organisation, if established, could contribute to the preparation of future FP7 grant proposals, like the scheduled Flora & Fauna Palearctica proposal.

The programmed items listed below are preliminary and need further editing.

---

[1] http://zoology.fns.uniba.sk/edit/meetings/wp3-final.htm

2.2 Pan-European taxonomic standardisation

This task aims to reduce the discrepancies in taxonomic data among national databases and between the national and pan-European databases (e.g. ERMS, E+M, FaEu), by using the pan-European databases as a taxonomic reference to standardise concepts and spellings and to harmonise synonymy.

In principal all Focal Point members of the PESI consortium are expected to participate in this effort, in the interest of the quality of the national data resources, relevant for the proper implementation of environmental management systems related to for instance nature conservation and sustainable use of biological resources and to EC regulations.

Each Focal Point should develop a plan according to the local situation. This could parallel efforts on local capacity building and checklists set up (see 2.6) or include the organisation of local validation meetings.

Different methods for cross-validation can be considered. This could for instance include the distribution of data sets extracted from the pan-European checklists, organised per taxonomic group, towards local experts (as carried out during the FaEu NAS project) or the integral cross-referencing of national checklists against the pan-European checklists.

Provisionally the following subtasks can be distinguished:

Task1: *cross-validation of national and pan-European checklists*	
- Protocol development	WP3 & 5 management
- Tool development	WP3 & 4 and others
- Cross-validation of national and pan-European checklists	WP3 all partners
- Integration of results	WP3 management
- Implementation of the results in pan-European checklists	WP5 management
- Implementation of the results in national checklists	WP3 focal points

For the standardisation aims, taxa that figure in legislative texts must be prioritised in the time planning. It is desirable that their status in national or regional legislation be harmonised according to the European taxonomic standards. Taxonomists cooperating in PESI can also advise the respective legislative bodies on this topic.
This effort will counterpart similar strategies and action plans on prioritising the digitisation of parts of collections dealing with “target species” as outlined by for instance GBIF.
Possibly some additional data fields (see item 2.3) will be desirable for these taxa.

Task2: *data standards and enrichment for species figuring in legislation*	
- Protocol development	WP4 management
- Tool development	WP3&4 and others
- Liaison with legislation bodies (IUCN, EPPO, etc.)	WP1
- Cross-validation with local targeted species lists	WP3 all partners
- Report of results	WP4 management

To prevent confusion: because Euro+Med partners responsible for the updating of Euro+Med PlantBase are also playing a role as botanical FPs, these partners are formally included within PESI WP3 although part of their activities (and associated funding) actually contribute to WP2 and WP5.

Task3: *support updating of pan-European checklists* (only E+M)	
- Protocol development	WP5 management (E+M Editors)
- Tool development	WP5 (E+M)
- Set-up of an expertise network	WP2 (Eckhart)
- Updating Euro+Med PlantBase	WP3 USE, UNIPA, CUB
- Integration and implementation of results in pan-European checklists	WP5 management

2.3 Provision of additional data types

Common names

Task4: *provision of common names*	
- Protocol development	WP3, 4 & 5 management
- Tool development	{undecided}
- Local data collation	WP3 all partners
- Integration of results	WP3 management
- Implementation of the results	WP5 management

As a start PESI could focus on getting all vernacular names of all European main languages of a selected group of prioritised taxa, meaning those species playing a role in EU legislation on for instance nature conservation, habitat protection (Natura2000), and health care.

{Support translation because of multilingual portal}

{Support translation 'Informal Groups' ranking, example Nature Navigator site: http://www.nhm.ac.uk/nature-online/biodiversity/nature-navigator/}

This component also includes the guidance and provision of data standards on diverse data types when relevant within the process of data gathering (in collaboration with PESI WP4).

{Status in national legislation} {List of other data types…}

2.4 Update the inventory of local expertise

PESI WP2 provides the overview of taxonomic experts at the European or global level, ordered by taxon. WP3 provides the inventory of local and national experts and data resources.

Tasks: Revitalise and upgrade of the FaEu NAS data management system[2]; identify and register local taxonomic resources and registers; include metadata profile of potential regional/local taxonomic data resources, secure widest possible access to these resources.

---

[2] http://zoology.fns.uniba.sk/faeu/search_service/search_index.htm

This local expertise resource will be used by for instance PESI WP2 to cover potential gaps in taxonomic expertise on certain groups.

The FP taxonomic resource meta-database is planned to be linked to the GBIF metadata services. This will support the institution of PESI as a GBIF thematic node and establish a liaison of non-member countries towards GBIF. This 'proto-GBIF node' functioning could help focal points during the negotiation process with local governments on financially support (see 2.5).

2.5 Support Focal Points' activities and policy plans

PESI supports FPs in their organisation and functioning as national or regional clearinghouse for taxonomic data. Part of this is the documentation of experiences and best practice in a Focal Point Handbook:

- Documentation of technical procedures
- Information on the organisation of FPs in relation to various national institutions, GBIF nodes and the compilation of national checklists.
- Routes to financial resources
- Assistance in the application for national funds, preparation of policy plans and contacts with potential users (see also item 2.3)
- Coordination with developments in e.g. GBIF, EDIT, LifeWatch and LIFE+ projects.

This activity includes efforts to facilitate the acquisition of funds for regional focal points to assure a more permanent and constructive focal point participation, for instance by drafting supporting documents:

- Preparation of a document to convince local governments to financially support focal points to make their local legislation Natura2000-proof.
- Preparation of a document emphasizing the importance of "targeted species", together with organizations on health care, plant protection (EPPO), invasive species (Daisy), red lists (IUCN), habitat protection (EEA), etc.
- Preparation of Memorandums of Understanding (MoU's) with associated EDIT or CETAF institutes to assure long term support.

Deliverables: FP Handbook (D3.3, D3.4); a generic policy plan to be applied by individual FPs to support local funding.

2.6 Set-up and maintenance of national checklists

Building on the experience of several partners, PESI provides support for the establishment or updating of national checklists where needed. Part of this task follows from the results of the cross-validation effort.

Task5: *Preparation and maintenance of national checklists*	
- Technical infrastructure support	e.g. EDIT editor tool or GBIF checklist tool
- Data collation support (e.g. downloads of pan-European checklists)	WP5
- Local expert network support	
- Long-term maintenance, regular updates	

Procedures and tools must be documented in the Focal Point Handbook.

2.7 Publication plan for taxonomic notes

The validation and standardisation process (section 2.2) as well as the set-up of new national checklists will generate taxonomic information that needs to be documented in a sustainable way, e.g. new occurrence records, discussion of alternative taxonomies. One possible way to do this is to publish a series of PESI-related notes in an (online) journal. In this manner the taxonomists working on the topic can be credited for their efforts. The *Euro+Med Notulae*, which are published in *Willdenowia*, may serve as an example for the marine and zoological communities.

In addition a publication plan for local distributional details could be considered, like the reporting-facility of the Swedish http://artportalen.se, allowing amateurs to include faunistic details.

The possibilities should be discussed and worked out in consultation with WP6 and the Steering Committee.

2.8 Interoperability and integrated (PESI) web-portals

The taxonomic data in the future PESI portal (or other European or global portals) can be enriched with links to national data portals that provide distribution and other data (phenology, images, sounds, etc.). Conversely, PESI as the taxonomic backbone can link the same species across different national (and other) data portals.
Part of the WP3 seed money can be allocated to small projects in this realm. The previously mentioned tasks, however, have first priority.

3 Further work plan development

3.1 Time path

An initial draft WP3 Work Plan (based on DoW and Smolenice report) was presented to the SC in month 1 (kick-off meeting). The present second draft (based on input of SC-meeting) was prepared in month 3 and discussed in month 5 at a core group meeting. The fourth version of work plan will be presented to the SC in month 6. A fifth, SC-approved version should be ready in month 8 and sent to PESI partners for discussion at General Meeting in November 2008. The final version of the work plan will be established during this meeting.

3.2 Division of labour

TU staff will do the executive management of the WP3 Work Plan. An advisory group including representatives of other project WP3 participants will revise the Work Plan before the General Meeting: Suggested possible members are (most of them volunteered at the Smolenice meeting):

- WP3: Nihat Aktaç and Selçuk Yurtsever

- WP1: Yde de Jong, Julia Kouwenberg and Louis Boumans
- FaEu: Edouard Stloukal and Roy Kleukers
- ERMS: Roisin Nash and Christos Arvanitidis
- E+M: Eckhart von Raab-Straube and Karol Marhold

The Seed Money Allocation Plan (see below) organises the distribution of tasks and seed money among these project partners.

3.3 Seed money allocation

With respect to seed money allocation in general it is important to realise that PESI WP3 will primary contribute seed money to the development of infrastructural components to support PESI objectives on European collaboration on taxonomic standardisation. The local application of those infrastructures should be matched form local resources. If such local resources are lacking, PESI consortium partners could participate in the Focal Points' policy plan supporting program (item 2.5) to recover funding to ensure their functioning as national or regional taxonomic clearinghouse.

Based on the work plan, WP3 staff at TU prepares a spreadsheet with tasks and information on the task. This list is then distributed to the FP members of the PESI consortium, who are invited to indicate what tasks they would be interested in doing, and under what conditions (time planning and costs). The TU staff collects this information and prepares one or more alternative plans in consultation with the advisory group. The plan, which is supported by the advisory, is submitted to the SC for final decision.

For details on the time path for the seed money allocation plan see the PESI DoW.

**Configuration History**			
**Version No.**	**Date**	**Changes made**	**Author**
0.1	31 March 2008	First draft prepared for the kick-off meeting at Amsterdam, 19-20 May 2008.	YdJ / LB
0.2	12 June 2008	Second draft, including comment from the kick-off meeting.	YdJ / LB / NA
0.3	28 July 2008	Third draft prepared for the management meeting at Edirne, 6-9 September 2008.	YdJ / LB / NA
0.4	15 September 2008	Fourth draft prepared for first PESI Focal Point working group meeting at Bratislava, 7 October 2008.	YdJ / LB / / NA / JK
0.5	14 November 2008	Fifth draft prepared for the First General meeting at Sevilla, 28-29 November 2009.	YdJ / LB / / NA / JK
1.0	27 May 2009	Final first version, including additional Appendices.	WP1 / WP3

Appendix I — *Draft table of WP3 subtasks and efforts*

TASKS	CODE	EXPLANATORY TASK INFORMATION		WORK PROGRAM PRIORITY	SEED MONEY PRIORITY	PHASE	TIMEPATH	PERSON MONTHS	COST ESTIMATE	DESCRIPTION OF ACTIVITIES FOR TASK COMPLETION
			WP3 Management tasks							
				High (H)	High (H)	1 (Feb 09-Oct 09)			direct	
			Focal Points tasks	Medium (M)	Medium (M)	2 (Oct 09-Sep 10)			costs	
				Low (L)	Low (L)	3 (Sep 10 - Apr 11)				
			Other WPs tasks		Non-Eligible (NE)					
**Establishment of a Focal Point Organisation**	**2,1**									
Development plan for a legal FP establishment	T2.1A	Set up of an organisation (FP society / association) equivalent to for instance SMEBD (regarding experts), also important to ensure an easy participation of the focal point network in future projects (as one entity).	**WP3 - TU (with help of WP1 and other FPs)**	H	NE	1-2				
Connection, relationships with relevant national biodiversity resources (national checklists owners, etc.)	T2.1B	Also supporting other activities (e.g. 2.3 and 2.4)	**WP3 - FPs**	H	NE	1				
Build relationships with other EU or global oriented national biodiv. nodes (CBD, Natura2000, IUCN, WWF, WHO, GBIF, etc.) (with WP1)	T2.1C	Establish a position within national biodiversity platforms, also important to deliver the results of PESI WP3.	**WP3 - FPs**	H	NE	2-3				
Update overview of Smolonice questionnaire	T2.1D	Important management tool for FPs policy development.	**WP3 - FPs**	H	NE	1				
**Pan-European taxonomic standardisation**	**2,2**									
** Cross-validation of national checklists against pan-european checklists (FaEu + ERMS)*										
Set-up of PESI virtual data validation office	T2.2A	Including protocol development, download format definition, tool development (for cross-matching), validation (process monitoring) staff appointment	**WP3, WP4, WP5 & selected FPs**	H	NE	1				
Cross-validation of national checklists against pan-european checklists (FaEu) – first validation cycle	T2.2B	Self-validation by national focal points supported by WP3 management	**FaEu FPs**	H	L / NE	1-2				
Cross-validation of national checklists against pan-european checklists (FaEu) – second validation cycle (if relevant)	T2.2C	Self-validation by national focal points supported by WP3 management	**FaEu FPs**	M	NE	2 -3				
Compile country-based spp. inventories from region-based spp. inventories (ERMS-FP's)	T2.2D	According to ERMS WP3 working program	**ERMS FPs**	H	H	1-2				
Cross-check national inventories with EurOBIS, ERMS using ERMS matching tool (ERMS-FP's)	T2.2E	According to ERMS WP3 working program	**ERMS, TU**	H	H	2-3				
** Support updating of pan-European checklists (E+M)*										
Updating Euro + Med Plantbase	T2.2F	According to E+M WP3 working program	**E+M FPs**	H	H	1-3				
**Provision of additional data types**	**2,3**									
** Additional data types*										
Inventory of additional data types in national and major regional checklists	T2.3A	Data sheet: PESI_checklist data types_v1.xls	**All FPs**	H	H	1				
** Vernacular names*										
Protocol development (operation manual) for vernacular names compilation & integration	T2.3B		**WP3, WP4, WP5 & selected FPs**	H	NE	1				
Vernacular names of prioritised taxa obtained from national and major regional checklists	T2.3C	Provide vernacular names of prioritised taxa using existing national / regional checklists.	**All FPs**	H	H	2 (-3)				
Vernacular names of prioritised taxa obtained from other data resources	T2.3D	Define a program to obtain vernacular names of prioritised taxa when they can not be collated from existing national / regional checklists.	**All FPs**	H	H	2 (-3)				
Vernacular names, available in national, major regional checklists or other resources	T2.3E	Include all other vernacular names (also of non prioritised taxa) from all other local resources (national / regional checklists or else).	**All FPs**	M	L / NE	2-3				
** Prioritised species lists (species figuring in legislation)*										
Inventory and delivery of European priority species lists	T2.3F	WP1 liaison with IUCN, EU Topic Centre et cetera.	**WP1**	H	NE	1				
Cross-validation of European prioritised species lists against pan-european checklists	T2.3G		**WP3 - TU**	H	NE	2				
Inventory national prioritised species lists	T2.3H	Survey availability of national prioritised species lists focussing on standardised national lists of targeted/prioritised species (playing a role in national legislation on nature protection, etc.)	**All FPs**	M	M	1				

Delivery of national prioritised species lists	T2.3I	Use T2.3H to select relevant lists	All FPs	M	M	2				
Aligning (comparison matrix of) national prioritised species lists	T2.3J		WP3 - TU	M	NE	2				
Cross-validation of EU priority species lists against national priority species lists matrix	T2.3K	Comparison of EU priority species lists and national priority species lists to report potential incongruences	WP3 - TU	M	NE	3				
** Other additional data types (for data enrichment of the PESI portal)*										
Photos, sounds, legislation status, links to species records elsewhere, and so on	T2.3L	If relevant for the PESI web portal	All FPs	M/L	M/L	3				
Support with translations to establish a multilingual webportal	T2.3M	For all EU languages	All FPs	H	M	3				
Digitisation of available, non electronic spp lists	T2.3N	If relevant for the PESI web portal	All FPs	L	L	3				
**Update the inventory of local expertise**	**2,4**									
Revitalise and upgrade FaEu NAS data management system	T2.4A	Upgrade import sheets, adapt data management system (adding countries, adpat interface)	CUB	H	H	1				
Deliver metadata (profile) of national/local/regional taxonomic data resources and expertise	T2.4B	Data sheets: PESI_TaxExpertise_v1.xls, PESI_TaxRes_v1.xls	All FPs	M	M	1-3				
Secure widest possible access to these resources	T2.4C	Link to GBIF	CUB	M	M	2-3				
**Support Focal Points' activities and policy plans**	**2,5**									
Application for national funds regarding WP3 activities, FPoutreach, FP organisation planification	T2.5A		All FPs	M	M	1-3				
Contributions to FP handbook (best practices etc)	T2.5B		All FPs	H	H	1-3				
**Set-up and maintenance of national checklists**	**2,6**									
** Preparation and maintenance of national checklists*										
Local expert network support	T2.6A	Program to help FPs with the set up of a national checklist	All FPs	L	L / NE	2-3				
Long-term maintenance, regular updates	T2.6B	Program to help FPs with the set up of a national checklist	All FPs	L	L / NE	2-3				
**Other tasks**	**2,7**									
Index Fungorum (formulate task)	T2.7A	In collaboration with Paul Kirk	WP4		NE	1-3				
Desmidiales (formulate task)	T2.7B	In collaboration with Mike Guiry	WP4		NE	1-3				
**Other task suggestions**										
Cross-reference matrix for web portal function	T2.7C	Preparing a mechanism to link the pan-European checklist results to the national checklist results	Naturalis	M	M	2-3				

Appendix II — *Data Flow Plan*

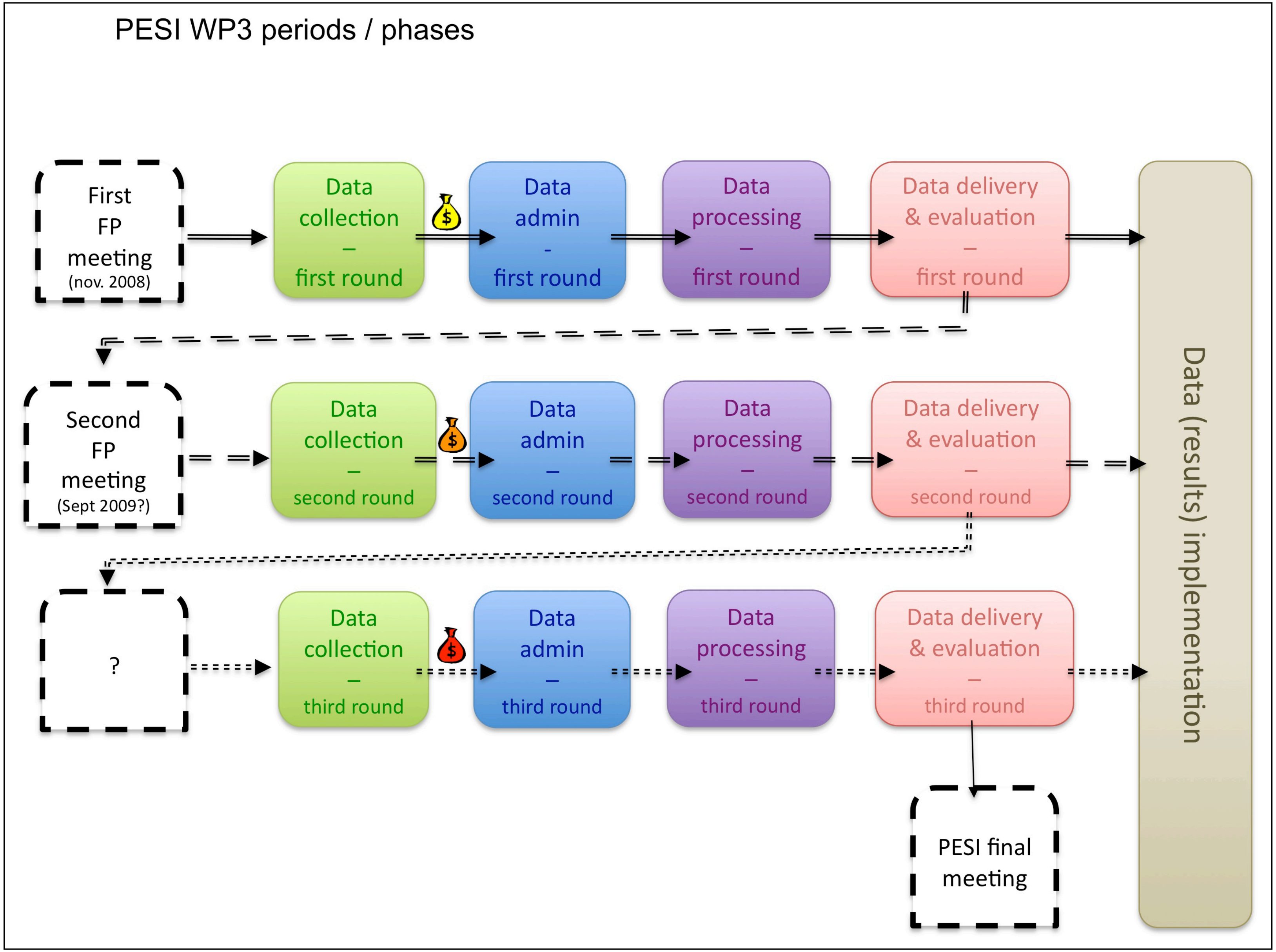

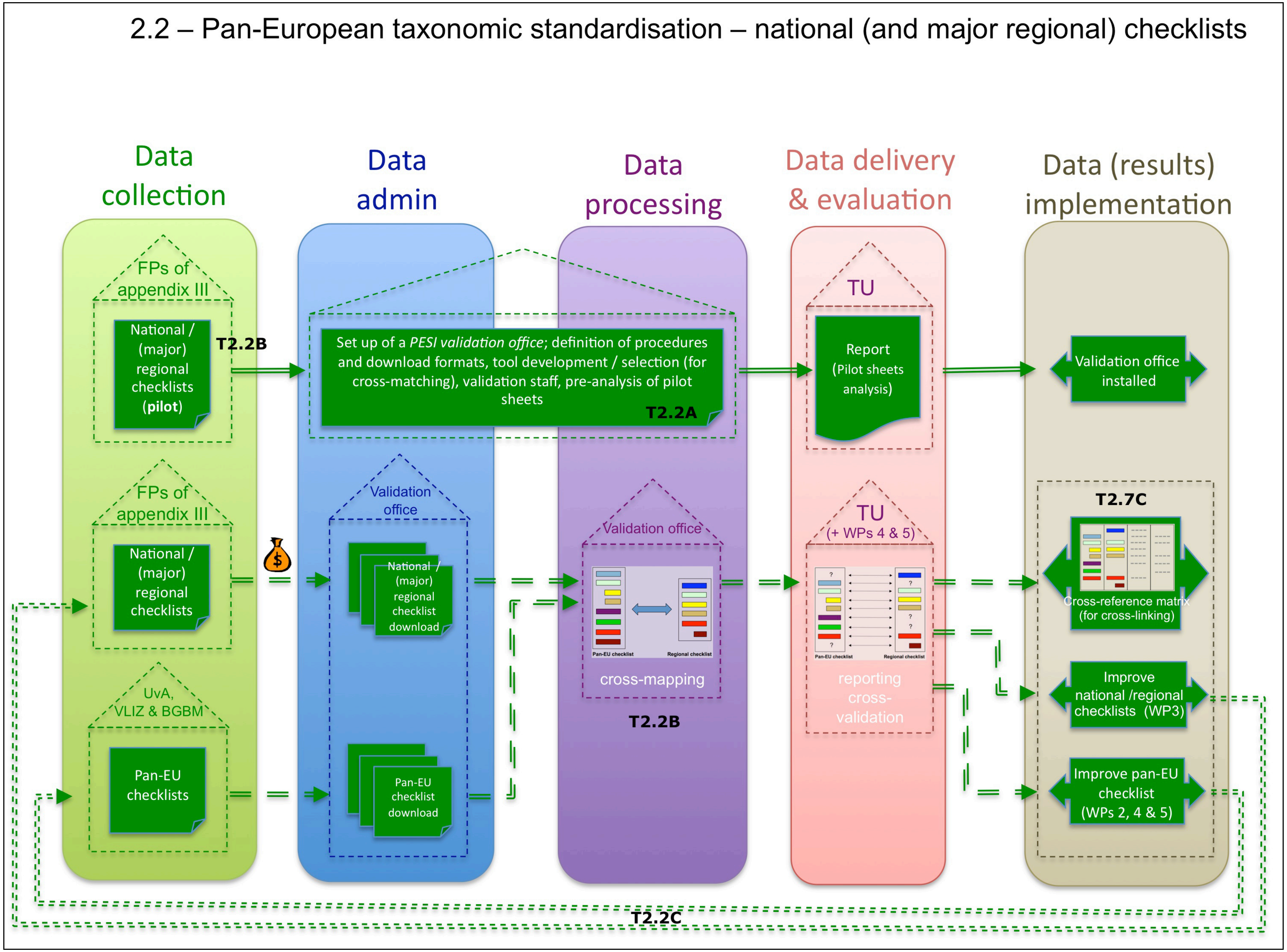

2.2 – Pan-European taxonomic standardisation – national (and major regional) checklists
Data collection
Data admin
Data processing
Data delivery & evaluation
Data (results) implementation
FPs of appendix III
National / (major) regional checklists (pilot)
T2.2B
Set up of a PESI validation office; definition of procedures and download formats, tool development / selection (for cross-matching), validation staff, pre-analysis of pilot sheets
T2.2A
TU
Report (Pilot sheets analysis)
Validation office installed
FPs of appendix III
National / (major) regional checklists
Validation office
National / (major) regional checklist download
Validation office
cross-mapping
T2.2B
TU (+ WPs 4 & 5)
reporting cross-validation
T2.7C
Cross-reference matrix (for cross-linking)
Improve national /regional checklists (WP3)
Improve pan-EU checklist (WPs 2, 4 & 5)
UvA, VLIZ & BGBM
Pan-EU checklists
Pan-EU checklist download
T2.2C

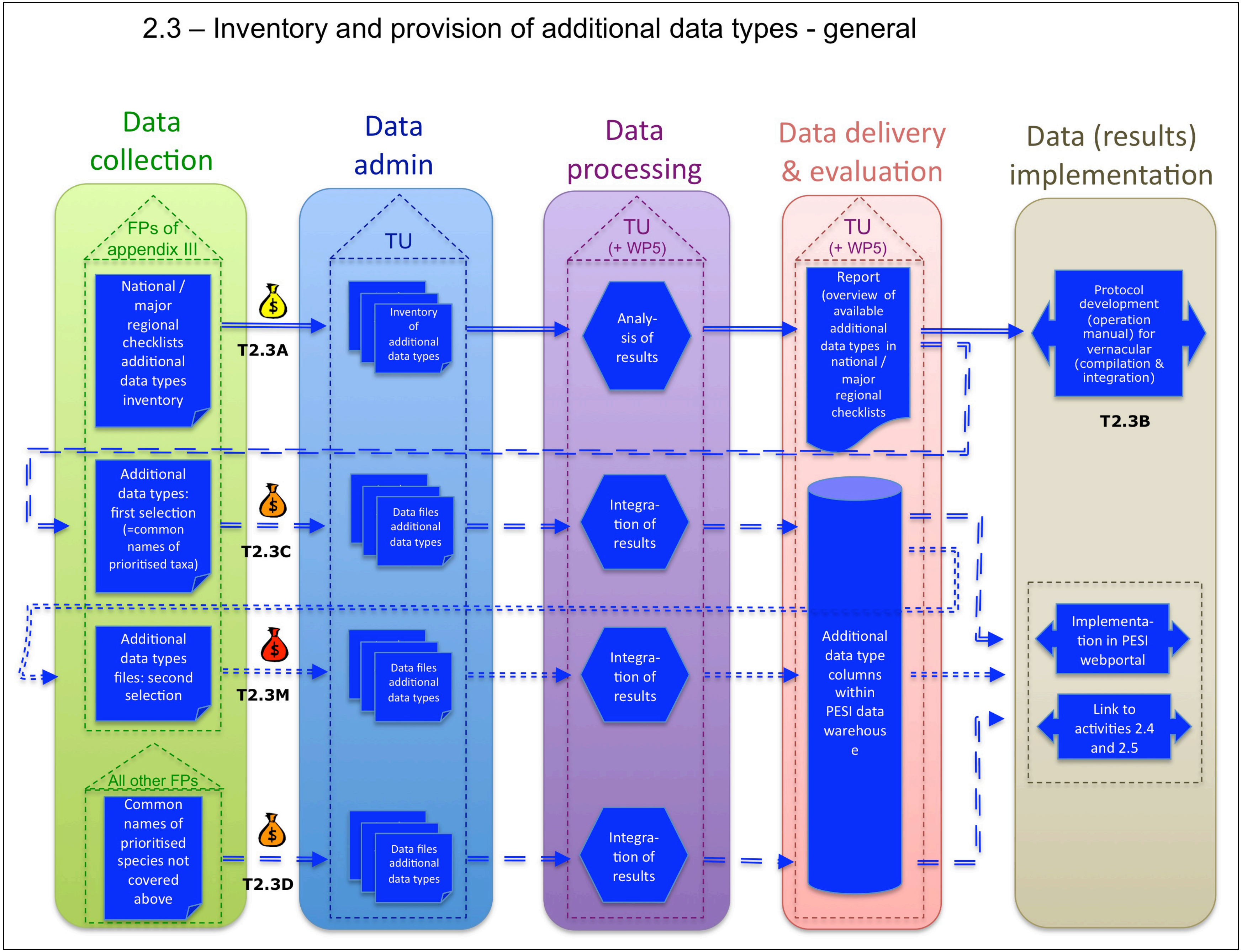
2.3 – Inventory and provision of additional data types - general
Data collection
Data admin
Data processing
Data delivery & evaluation
Data (results) implementation
FPs of appendix III
TU
TU (+ WP5)
TU (+ WP5)
National / major regional checklists additional data types inventory
Inventory of additional data types
Analy-sis of results
Report (overview of available additional data types in national / major regional checklists
Protocol development (operation manual) for vernacular (compilation & integration)
T2.3A
T2.3B
Additional data types: first selection (=common names of prioritised taxa)
Data files additional data types
Integra-tion of results
T2.3C
Additional data type columns within PESI data warehouse
Additional data types files: second selection
T2.3M
Implementa-tion in PESI webportal
Link to activities 2.4 and 2.5
All other FPs
Common names of prioritised species not covered above
T2.3D

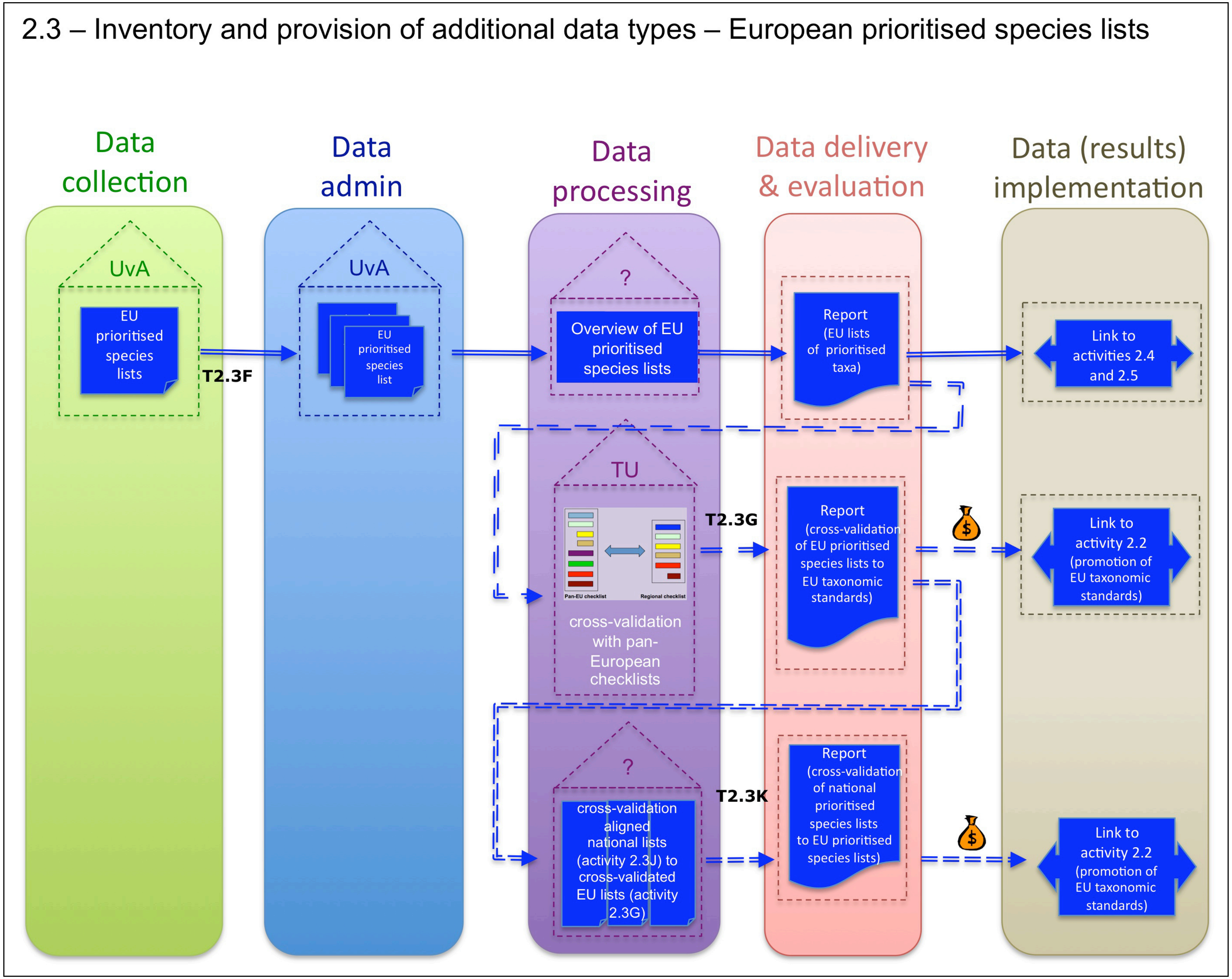
2.3 – Inventory and provision of additional data types – European prioritised species lists
Data collection
Data admin
Data processing
Data delivery & evaluation
Data (results) implementation
UvA
EU prioritised species lists
T2.3F
UvA
EU prioritised species list
?
Overview of EU prioritised species lists
Report (EU lists of prioritised taxa)
Link to activities 2.4 and 2.5
TU
Pan-EU checklist
Regional checklist
cross-validation with pan-European checklists
T2.3G
Report (cross-validation of EU prioritised species lists to EU taxonomic standards)
$
Link to activity 2.2 (promotion of EU taxonomic standards)
?
cross-validation aligned national lists (activity 2.3J) to cross-validated EU lists (activity 2.3G)
T2.3K
Report (cross-validation of national prioritised species lists to EU prioritised species lists)
$
Link to activity 2.2 (promotion of EU taxonomic standards)

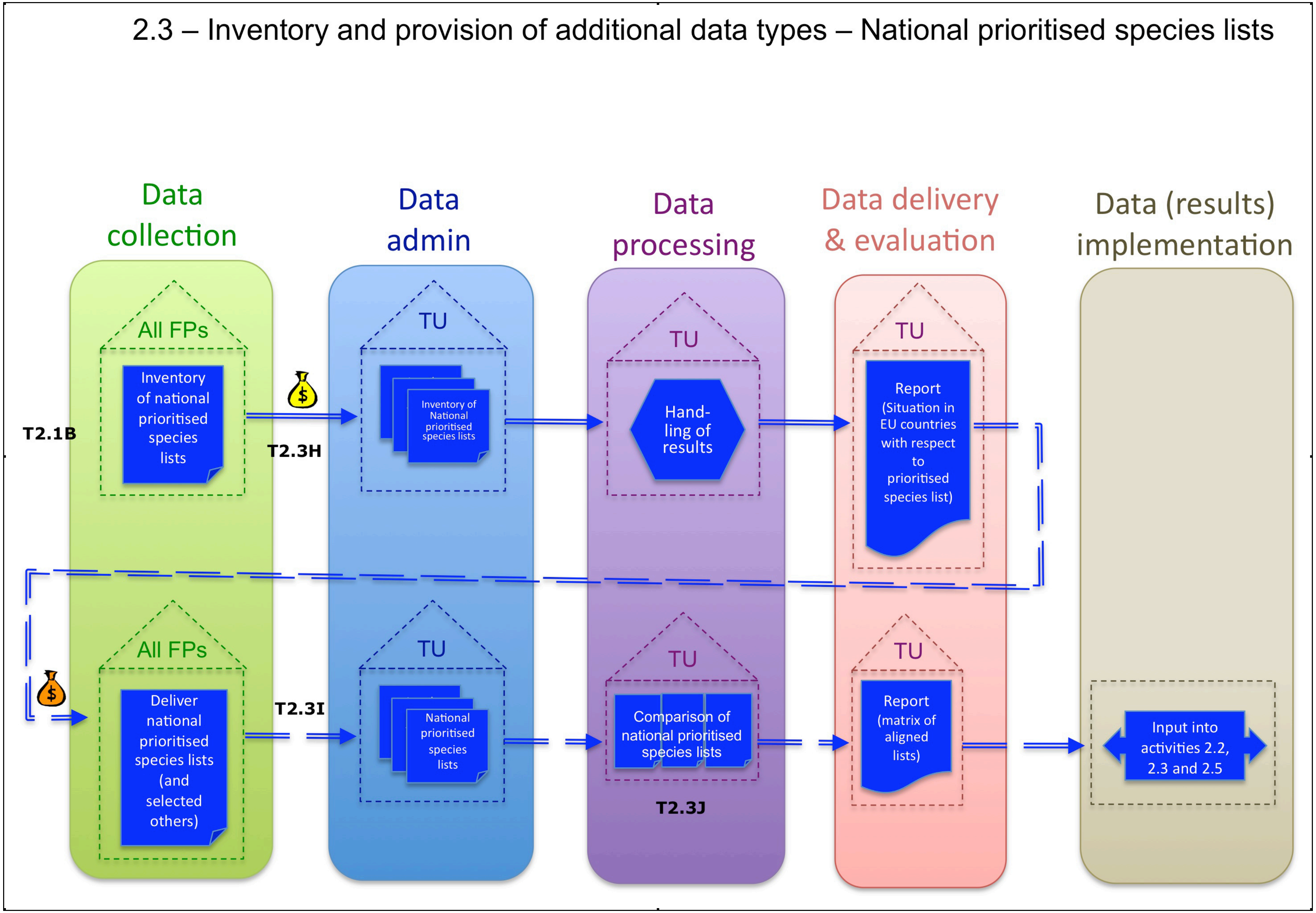
2.3 – Inventory and provision of additional data types – National prioritised species lists
Data collection
Data admin
Data processing
Data delivery & evaluation
Data (results) implementation
All FPs
Inventory of national prioritised species lists
T2.1B
T2.3H
TU
Inventory of National prioritised species lists
TU
Hand-ling of results
TU
Report (Situation in EU countries with respect to prioritised species list)
All FPs
Deliver national prioritised species lists (and selected others)
T2.3I
TU
National prioritised species lists
TU
Comparison of national prioritised species lists
T2.3J
TU
Report (matrix of aligned lists)
Input into activities 2.2, 2.3 and 2.5

2.4 – Inventory of meta data of local taxonomic data resources and expertise

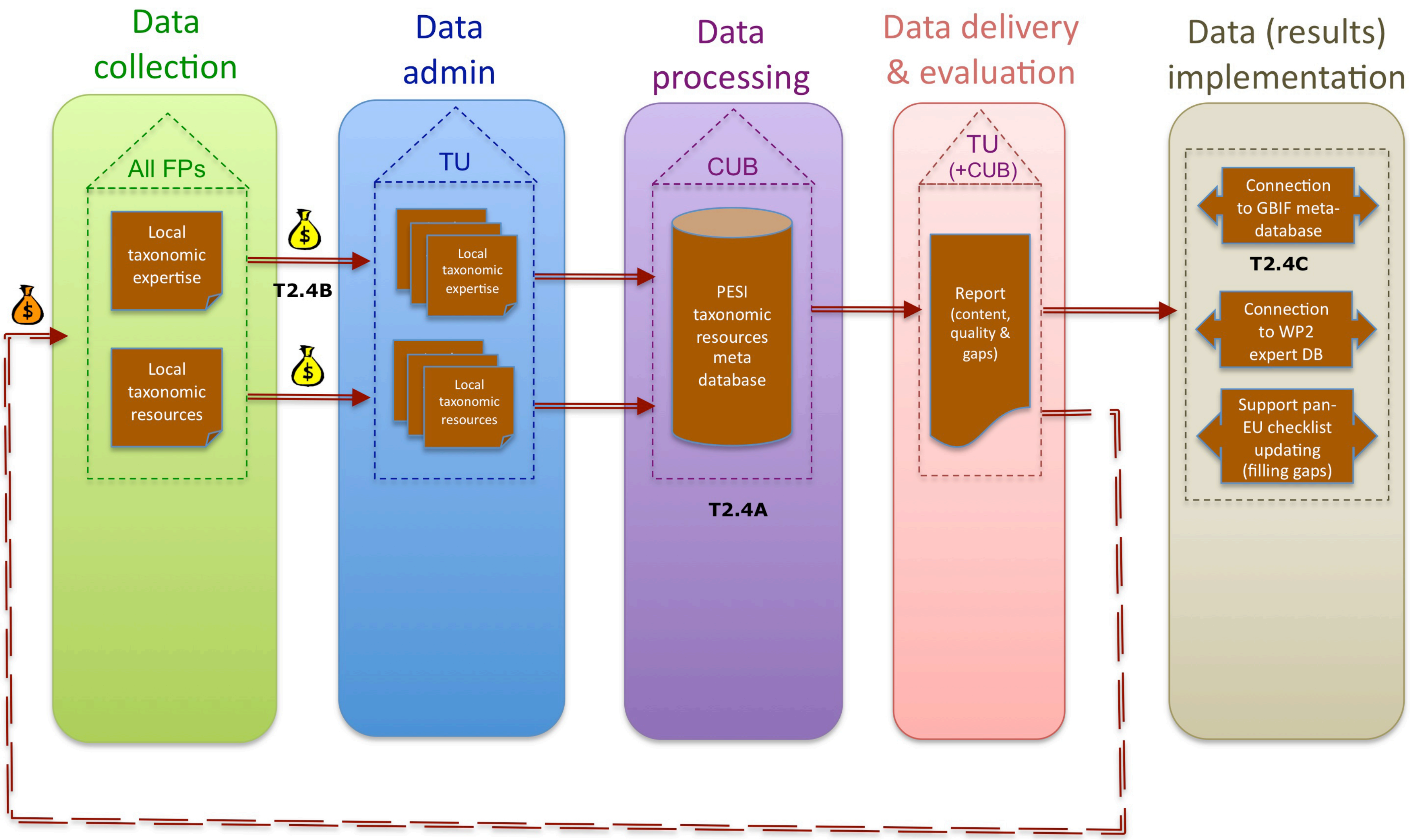

Appendix III — *Focal Points overview*

PESI Focal Points contracting partners overview:

Ptcp No.	Organisation	Contact	Country	Role
2	University of Copenhagen (UCPH)	Henrik Enghoff	Denmark	FaEu Focal Point
3	University of Trakya (TU)	Nihat Aktac	Turkey	FaEu Focal Point
4	Natural History Museum (NHM)	Charles Hussey	United Kingdom	FaEu Focal Point
5	Freie Un. Berlin, Botanic Garden, Botanic Museum (BGBM)	Walter Behrendsohn	Germany	Euro + Med Plantbase
6	Flanders Marine Institute (VLIZ)	Ward Appeltans	Belgium	ERMS Focal Point
7	Ecological Consultancy Services Ltd (Ecoserve)	Roisin Nash	Ireland	FaEu Focal Point, ERMS
9	Muséum National d'Histoire Naturelle (MNHN)	Olivier Gargominy	France	FaEu Focal Point
13	National University of Ireland (NUIG)	Michael Guiry	Ireland	ERMS Focal Point
15	University Palermo, Department Botanical Sciences (DPCE)	Francesco M. Raimondo	Italy	Euro + Med Plantbase
16	University of Seville, USE	Benito Valdés	Spain	Euro + Med Plantbase
17	Institute of Botany, Slovak Academy of Sciences (IBSAS)	Karol Marhold	Slovakia	Euro + Med Plantbase
18	National and Kapodistrian University of Athens (NKUA)	Anastasios Legakis	Greece	FaEu Focal Point
19	National Museum of Natural History Naturalis (NNM)	Roy Kleukers	The Netherland s	FaEu Focal Point
20	Institute of Ecology of Vilnius University (VUEI)	Eduardas Budrys	Lithuania	FaEu Focal Point
21	Scientific Committee for the Italian Fauna (CSFI)	Fabio Stoch	Italy	FaEu Focal Point
22	Swedish Museum of Natural History (NRM)	Sven Kullander	Sweden	FaEu Focal Point
23	Comenius University Bratislava (CUB)	Eduard Stloukal	Slovakia	FaEu Focal Point
24	Museum of Natural History and Archaeology - Un. Sci. and Techn. (NTNU)	Kaare Aagaard	Norway	FaEu Focal Point
25	State Museum of Natural History, Nat. Ac. of Sciences of Ukraine (SMNH)	Volodymyr Rizun	Ukraïne	FaEu Focal Point
26	Museum and Institute of Zoology - Polish Academy of Sciences (MIZPAN)	Wieslaw Bogdanowicz	Poland	FaEu Focal Point
27	Swiss Systematics Society	Jean Mariaux	Switzerland	FaEu Focal Point

	(SSS)			
28	Ilia Chavchavadze State University (ILIAUNI)	David Tarkhnishvili	Georgia	FaEu Focal Point
29	Consejo Superior de Investigaciones Cientificas (CSIC)	Marian Ramos	Spain	FaEu Focal Point
30	Slovenian National Institute of Biology (NIB)	Davorin Tome	Slovenia	FaEu Focal Point
31	National Museum of Natural History - Sofia (NMNHS)	Pavel Stoev	Bulgaria	FaEu Focal Point
32	myNature Association (myNA)	Adorian Ardelean	Romenia	FaEu Focal Point
33	University of Latvia (LU)	Voldemars Spungis	Latvia	FaEu Focal Point
34	Hellenic Centre for Marine Research (HCMR)	Christos Arvanitidis	Greece	ERMS Focal Point
35	Israel Oceanographic and Limnological Research (IOLR)	Bella Galil	Israel	ERMS Focal Point
36	Institute of Oceanology of Polish Academy of Sciences (IOPAN)	Jan Marcin Weslawski	Poland	ERMS Focal Point
37	Zoological Institute of Russian Academy of Sciences (ZIN RAS)	Sergei Medvedev	Russia	FaEu Focal Point
38	A.O.Kovalevsky Inst. of Biol. of the Southern Seas (IBSS)	Oleksandra Sergeyeva	Ukraine	ERMS Focal Point
39	Marine Biological Association of the United Kingdom (MBA)	Dan Lear	United Kingdom	ERMS Focal Point
40	University of Sciences and Technology of Lille (USTL)	Jean-Claude Dauvin	France	ERMS Focal Point

PESI non-contracting partners in addition to the project consortium:

**Organisation**	**Contact**	**Country**	**Role**
Musée national d'histoire naturelle – Luxembourg	Marc Meyer	Luxembour g	FaEu Focal Point
Malta Environment & Planning Authority – Floriana	Marie Therese Gambin	Malta	FaEu Focal Point
Senckenberg Museum – Frankfurt	Michael Tuerkay	Germany	FaEu Focal Point
Hungarian Natural History Museum – Budapest	László Peregovits	Hungary	FaEu Focal Point
Estação de Biologia Marinha do Funchal – Madeira	António Domingos Abreu	Portugal (Macarones ia)	FaEu Focal Point
Institute of Zoology and Hydrobiology (Univ. of Tartu) – Tartu	Mati Martin	Estonia	FaEu Focal Point
Austrian State Museum – Linz-Dornach	Michael Malicky	Austria	FaEu Focal Point
Institute of Oceanology (IO-Bas) – Varna	Snejana Moncheva	Bulgaria	ERMS Focal Point

Appendix IV — *Regional Focal Points local species information services*

Country	Name of resource	URL
Mediterranean Marine Fauna	MediFaune	http://nephi.unice.fr/Medifaune
Austria	Zoologisch - Botanische Datenbanken Österreich	http://www.zobodat.at
Britain	NBN Species Dictionary	http://www.nhm.ac.uk/nbn
Estonia	Estonian Species Register	http://unite.ut.ee/temp/natmus_in.php
France	Inventaire national du Patrimoine naturel	http://inpn.mnhn.fr
Georgia	Biodiversity-Georgia	http://www.biodiversity-georgia.net
Greek	Greek Biodiversity	http://greek-biodiversity.bio.auth.gr
Italy	Fauna Italia	http://www.faunaitalia.it
Netherlands	Nederlands Soortenregister	http://www.nederlandsesoorten.nl
Norway	Artdatabanken	http://www.biodiversity.no
Portugal & Spain	Fauna Iberica	http://www.fauna-iberica.mncn.csic.es
Romania	myNature	http://mybiosis.org
Russia	Biological Diversity of Russia	http://www.zin.ru/biodiv
Slovak	Slovak Taxonomic and Biodiversity Information Facility	http://zoology.fns.uniba.sk/dataportal
Sweden	ArtPortalen	http://www.artportalen.se
Sweden	ArtDatabanken	http://www.artdata.slu.se
Switzerland	CSCF & KARCH & CCO & KOF	http://lepus.unine.ch/carto
Turkey	BIOCES	http://bioces.tubitak.gov.tr

Appendix V — *Updated Smolenice Questionnaire*

Country	Participation	Local resources	Taxonomic standards	Additional data types	e-Infrastructure
**NORWAY**	FaEu, GBIF, Lifewatch, Species 2000	CHECKLIST PRESENT NBIC (artsdatabanken), National expertise resources maintained, Information system is developing by NBIC (can be accessed)	National checklists cross-checked by specialists using EU checklists (no detail)	VERNACULAR NAMES PRESENT EU nomenclature implemented (no detail), Maps, Images, Common names (Norvegian)	present (no detail)
**TURKEY**	FaEu	CHECKLIST PRESENT Expertise data (ARBIS). Can be accessed National checklists: BIOCES-TUBİTAK, TUBIVES, CLOMFOT, Turkish fishbase (marine), akyaka.org (fish),	FaEu checklists used for cross-checking	VERNACULAR NAMES PRESENT EU Taxonomic standards implemented. Common names, Conservation status, ID keys, Distribution	Partly
**ROMANIA**	FaEu, Royal Botanical Garden, LSF, Catalogue of Life	CHECKLIST PRESENT Checklist present (my nature), membership requred	FaEu, Royal Botanical Garden Edinburg, LSF, Catalogue of Life	VERNACULAR NAMES PRESENT Partly	Partly
**LATVIA**	FaEu, EDIT	CHECKLIST PRESENT National checklists present: Baltic beetles checklist & data base, Baltic molluscs checklist & data base, Data bases: DU, SBI, LNHM, other Information system (partly)	EU checklists	VERNACULAR NAMES PRESENT All Eu standards, Common names, Distribution	NOT present
**LITHUANIA**	EPBRS, FaEu	NO CHECKLIST NO Expert data base Experts:Villinius University and different societies on different taxa	FaEu used for cross-checking synonymy, Cross-checking details on local lists not allowed	VERNACULAR NAMES PRESENT EU standards implemented on checklists. Common names, Conservation status, Images	Many sites on different groups: Lithuania mycological society Vilnius University ; - Botany, - Zoology, Lithuaniaan Entomological society, Lithuanian Ornithological Society, - Lithuanian Botanical Society, - Lithuanian Dendrological Society, Lithuanian Theriological Society
**SLOVAKIA**	FaEu, GBIF, EDIT, Natura 2000, TDWG, Icpdr	CHECKLIST PRESENT Checklists present: Databank of Slovak Fauna, Fauna Slovenska. Zoology expertise data maintained.	EPBRS	? Distribution, other details?	DFS (Digital Library on Fauna Slovakia and adjacent countries)
**FRANCE**	FaEu, GBIF, EDIT, CHM (CBD), ERMS, Fishbase, Natura 2000	CHECKLIST PRESENT INPN (National Inventory of Natura Heritage, France), National plant list, MNHN; DIVERSITAS, Expert data: ??	Pan-European databases used, ERMS	VERNACULAR NAMES PRESENT Common names, Distribution, Endemism, Maps, Images, Valid names, Synonym, Unknown names, Conservation status	Present
**NETHERLANDS**	FaEu, GBIF, EDIT, Others?	CHECKLIST PRESENT Checklist: Dutch Species Register, Dutch experts >100, Expertise Data maintained and can be accessed	Cross-checking performed, using FaEu, mostly by non-professionals	VERNACULAR NAMES PRESENT Certain restrictions implemented (partly), Common names, Conservation status, Images, Maps, ID keys	Present (partly)
**SWEDEN**	GBIF, FISHBASE, FaEu, ENBI, EDIT	CHECKLIST PRESENT Many botanist and entomologist experts, National checklist program, Mapping expertise, periodic national survey	Specialists independently engaged in checklist review validation to be reviewed mostly voluntary	VERNACULAR NAMES PRESENT Common names, Conservation status, Images, Abundance, Tracking data, Morphology, Phenology	Not formallly
**SPAIN**	GBIF, EDIT, FaEu, Many other connections present, GTI	CHECKLIST PRESENT Internationally recognised experts Restricted access. Fauna Iberica, Flora Iberica: Flora Micologica, Flora Liquenologica Iberica, Flora Briofitica Iberica, Flora Ficologica Iberica	Yes (but no details)	VERNACULAR NAMES PRESENT Images, ID keys, Common names, Conservation status	Yes restricted, but open to experts
**UNITED KINGDOM**	FaEu, GBIF, EDIT, OBIS, BIOCASE, Species 2000	CHECKLIST PRESENT National Biodiversity Network (NBN), Checklists, , Nature navigator, Expert data resource	MOSt of them	VERNACULAR NAMES PRESENT Most EU standards implemented. Common names (English, Welsh, Gaelic), Images, Conservation status	e-taxonomy present
**UKRAINE**	NONE	NO CHECKLIST No national checklist program, some local : Animals of Ukraine, Flora of Ukraine, Conservation of Ukraine and more, Local expertise resourses maintained, information can be used by EU expertise information systems, Information system is maintained	Crosschecking is done by using EU checklists	VERNACULAR NAMES PRESENT EU standards implemented. EU checklists used, Local biodiversity service does not allow local details, Common names, Conservation status, Images, ID keys, others	Institutional level only
**ITALY**	FaEu, Not GBIF, No other programs	CHECKLIST PRESENT National checklist. Local expertise resource maintained, can be accessed for EU Information Services, Information system maintained	Checklists crosschecked on individual basis	VERNACULAR NAMES PRESENT EU taxonomic standards implemented, FaEu, ERMS, Geographic distribution , Common names, Conservation status	NO e-Taxonomy

Organizing taxonomic information – national and European contributions

Legal obligations

The set-up and maintenance of reliable taxonomic information systems follows from international agreements. Nearly all countries are parties of the United Nations *Convention on International Trade in Endangered Species of Wild Fauna and Flora* (CITES), concluded at Washington in 1973, and the *Convention on Biological Diversity* (CBD) concluded at Rio de Janeiro in 1992. Furthermore, the member States of the European Community are bound by the *Council Directive 79/409/EEC of 2 April 1979 on the conservation of wild birds* (Birds Directive), the *Council Directive 92/43/EEC of 21 May 1992 on the conservation of natural habitats and of wild fauna and flora* (Habitats Directive) and the *Directive 2000/60/EC of the European Parliament and of the Council of 23 October 2000 establishing a framework for Community action in the field of water policy* (Water Framework Directive). These conventions and directives form the basis of national legislations as well as a whole range of policy documents.

CITES, one of the largest conservation agreements in existence, regulates international trade in specimens of wild animals and plants and accords varying degrees of protection to more than 33,000 species of animals and plants. The CBD obliges parties, inter alia, to establish a system of protected areas, control the introduction of alien species, develop or maintain legislatory provisions for the protection of threatened species and adopt measures for the sustainable use of biological resources.

The Birds Directive contains measures for the protection of all species of naturally occurring birds in the wild state in the European territory of the EC member states (article 1). Annex I to the Birds Directive lists species that are subject to special conservation measures, while the species referred to in Annexes II and III enjoy lower levels of protection.

The Habitats Directive is intended to help maintain biodiversity in the EC Member States by defining a common framework for the conservation of wild plants and animals and habitats of Community interest. Its main instrument is the establishment of a coherent network of special areas of conservation, known as Natura 2000. Annex II (Animal and plant species of Community interest) to the Directive lists the species whose conservation requires the designation of special areas of conservation. Species of community interest are endangered, vulnerable, rare or endemic. Some of them are defined as 'priority' species (in danger of disappearing). The occurrence of species of community interest is the major criterion for the designation of protected areas under Natura 2000. Finally, the Habitats Directive obliges the EC Member States to regulate the introduction of non-native species (article 22-b).

The international conventions and European directives refer to species in a direct way. In addition, organisms in the taxonomical sense are a major constituent of the wider concepts of *biological diversity* and *biological resources*.
The Water Framework Directive commits European Union member states to achieve good qualitative and quantitative status of all water bodies (including marine waters along the shore) by 2015. The assessment of ecological water quality according to this directive involves the monitoring of the composition and abundance of aquatic flora and fauna, including plankton.

Checklists

Implicit in these legal texts is the obligation to set up and maintain standardized taxonomic checklists of species. After all, the identification of rare, endemic, threatened or alien species referred to requires thorough knowledge of biological taxa and their natural distribution. In addition, the practical realization of protective measures is feasible only when taxonomic reference to species is standardized at the international level.
Whereas the legal texts focus on rare, endangered or invasive species, it is obvious that taxonomic knowledge of all species is imperative. Rare or endemic species can only be identified in the context of not so rare and more widespread species, just like invasive species can only be identified once their original area of distribution is known.

Taxonomic research

Indeed, the CBD and the Birds and Habitats Directives contain provisions for taxonomic research. Thus, article 7 of the CBD prescribes the identification and monitoring of (habitats and) species important for conservation and sustainable use, as well as the organization and maintenance of data derived from these processes. Likewise, the Birds Directive encourages research and other work required for the protection and management of birds species, such as national lists of endangered species (article 10). Finally, the Habitats Directive obliges the EC and the member States to encourage necessary research and scientific work that contributes towards ensuring bio-diversity, inter alia through the surveillance of the conservation status of all species of wild flora and fauna (article 18).
In the same vein, the European Commission’s communication Halting the Loss of Biodiversity by 2010 — and Beyond advocates strengthening research infrastructures, the science–policy interface and data interoperability for biodiversity under FP7 and national research programmes. Standardized and authoritative taxonomic resources will enhance our understanding of biodiversity and ecosystem services and enable the refinement of policy responses in the future.

Problems arising from lack of standardization

The application of names to species and other biological taxa is not fully standardized due to a variety of reasons. Part of the incongruence results from variation in spelling or simple errors. Names may also change due to the advancement of taxonomic knowledge, and are not updated in all databases at the same time. Only in some cases different names reflect real disagreement of specialists.

If a protected species is mentioned as such in e.g. the Habitats Directive or a piece of national legislation, lack of international taxonomic standardization hampers the implementation of the regulations. This is best illustrated by the following examples:

The butterfly *Graellsia isabellae* (Graells, 1849) is mentioned in Annex II of the Habitats Directive, but its current valid name in Fauna Europaea is *Actias isabellae* (Graells, 1849). It occurs as *Actias* in the French national checklist INPN, and as *Graellsia* in Fauna Iberica.

In addition to the taxonomic data per se, data on occurrence and for instance legal status need regular updating.

Responsibilities on European and national level

Checklists of biological species are maintained at the European, national and sometimes regional level. In addition there exist specialized global checklists for certain groups of organisms (the Global Species Databases, GSDs). Over the past decades the European Union has supported the development of the major Europe-wide databases of plants, animals and marine organisms, and the organization of taxonomic specialists in the networks that are needed to maintain these databases. The EU also helped to set up a number of GSDs. The EU-funded PESI project now aims at integrating these knowledge resources at a higher level.

In the majority of European countries, national funding enabled the set-up of national and regional checklists, such as the Fauna Iberica or the Inventoire National de Patrimoine Naturel in France. However, continuous effort is required for their maintenance and also for the cross-validation of the different resources at national, regional and international level. In those cases where no national checklists exist as yet, the European databases and infrastructure will be helpful to produce first versions.

Accurate national checklists are indispensible for the application of international, European and national regulations concerning organisms occurring on the national territory, e.g. the protection of endemic species. While European project contribute greatly by providing infrastructural means like the organization of expert networks and development of software tools, the creation and maintenance of national checklists remains the responsibility of the individual countries.

Appendix VII — *Policy document: Target Species*

Prioritised Taxa for validation and Standardisation

State of the art in European Environmental policy

From 10 to 12 October 2007 the Sixth Ministerial Conference 'Environment for Europe' took place in Belgrade, Serbia. The Ministers adopted the so-called **Belgrade Biodiversity Statement**, via which they reconfirmed Europe's political commitment to the European biodiversity 2010 target to halt the decline of biodiversity by the year 2010. The Ministers **expressed their worries** about the continuing decline of Europe's biodiversity. **They will continue to invest in realising the target in time**.

Conservation of biological diversity **remains at the core of EU environment policy**. Implementing the existing legislation in the field entails a significant and **continuing investment of time and money**. By the end of 2008, a mid-term report on the implementation of the **Biodiversity Action Plan** will be produced. This report will measure progress towards the 2010 EU commitment to stop biodiversity loss within the EU and to significantly reduce loss worldwide.

PESI priorities regarding the cross-validation of local, regional and national species checklists

The PESI project aims at cross-validation of all European and national or regional taxonomic checklists, as well as the relevant Global Species Databases.
However, for practical and political reasons, priority will be given to those taxon names that are explicitly mentioned in legislative texts, with highest priority for international regulations.

Uncertainty of species identity is currently hampering the implementation of these regulations. For this reason, standardization of taxonomic reference to the species mentioned therein is a key deliverable to be delivered before all validation is finished. Validation and adjustment of these taxon names will be done in close cooperation with pertinent international, European and national agencies, such as the IUCN, EPPO, the European Topic Centre on Biodiversity, and others, who urgently need this information for the execution of their tasks.

Identification of relevant regulations referring to taxon names will be carried out in WP4. Validation and cross-checking with European and national checklists will be carried out by WP4 and WP3, where WP3 focuses on the coordination at the national level.

Below we provide a first inventory of potentially relevant resources. Some URL’s of relating websites are listed.

Categories of Target Species

1. *Endangered species*

When the last of a species dies out, the gene pool of the species is lost forever. To protect species, we must monitor their population levels. If one species dies out, those who rely on it in one way or another (i.e. protection or food) will also be affected. In light of this, humans aim to preserve genetic diversity and the diversity of species alive today. The **IUCN Red List of Threatened Species** provides taxonomic, conservation status and distribution information on taxa that have been globally evaluated using the IUCN Red List Categories and Criteria. This system is designed to determine the relative risk of extinction, and the main purpose of the IUCN Red List is to catalogue and highlight those taxa that are facing a higher risk of global extinction (i.e. those listed as Critically Endangered, Endangered and Vulnerable). The IUCN Red List also includes information on taxa that are categorized as Extinct or Extinct in the Wild; on taxa that cannot be evaluated because of insufficient information (i.e. are Data Deficient); and on taxa that are either close to meeting the threatened thresholds or that would be threatened were it not for an ongoing taxon-specific conservation programme. (IUCN red list, Europe's Endangered Species, EEC).

Thousands of plant- and animal species occur in Europe only. These endemic species can be considered as Europe's specific contribution to global biodiversity. Following the IUCN Red Data Books, hundreds of these European species are threatened. These species merit special nature conservation efforts in Europe. However, only several hundreds of species (not covering all species that are threatened according to IUCN-criteria) are protected under European regulations.

In 2005, Alterra Wageningen published a report, entitled: "***Target species – Species of European concern***":

The report proposes a Pan European Ecological Network and the establishment of a **"target species" database** of European concern on which European legislation should be based in the near future. A target species list is included in the document.

(www2.alterra.wur.nl/Internet////Modules/pub/PDFFiles/Alterrarapporten/AlterraRapport1119.pdf)

The **Convention on International Trade in Endangered Species (CITES)** lays down provisions for the protection of endangered species of flora and fauna. These provisions constitute controls on international trade in specimens of these species and are the basis of a worldwide policy on protection of endangered species.

Besides ratifying and implementing the CITES provisions, the EU has set additional import restrictions in Regulation (EC) 338/97 and Regulation (EC) 865/2006.

For Europe, the **Habitats Directive** lists in its Annex II all animal and plant species of community interest whose conservation requires the designation of special areas of conservation, and in Annex IV species that are in need of strict protection. The "priority" species are indicated with an *. This list that dates from 1992 was updated in 2006.

http://64.233.183.104/search?q=cache:o6Pj8Qvt8-4J:eurlex.europa.eu/LexUriServ/LexUriServ.do%3Furi%3DOJ:L:2006:363:0368:0408:EN:PDF+Directive+2006/105/EC&hl=nl&ct=clnk&cd=1&gl=nl&client=firefox-a

http://eur-lex.europa.eu/LexUriServ/LexUriServ.do?uri=CELEX:31992L0043:EN:HTML

http://www.earthsendangered.com/continent.asp?ID=6

http://www.iucnredlist.org/

http://www.petermaas.nl/extinct/EUmammals.htm

2. Invasive Alien Species

Invasive alien species threaten native biodiversity and cover all taxonomic groups from micro-organisms to animals and plants in all ecosystems. Biological invasions by non-native species are one of the greatest threats to the ecological and economic well being of the planet. Alien species can act as vectors for new diseases, alter ecosystem processes, change biodiversity, disrupt cultural landscapes, reduce the value of land and water for human activities and cause other socio-economic consequences. (DAISIE, GISD with 188 Invasive Species Databases, CIESM Atlas of Exotic Species). **Habitats Directive:** Article 22 of EC Directive 92/43/EEC (on the conservation of natural habitats and of wild flora and fauna) requires member states to "ensure that the deliberate introduction into the wild of any species which is not native to their territory is regulated so as not to prejudice natural habitats within their natural range or the wild native fauna and flora and, if they consider it necessary, prohibit such introduction."

Birds Directive: Article 11 of EC Directive 79/409/EEC states that "member states shall see that any introduction of species of bird which do not occur naturally in the wild state in the European territory of the member states does not prejudice the local flora and fauna."
The **European Strategy on Invasive Alien Species (2003)** (http://www.jncc.gov.uk/page-4013) addresses constraints, faced by many European States in their efforts to tackle the problem. These constraints include:
* low public awareness and opposition to government intervention;
* shortage and inaccessibility of scientific information (for species identification, risk analysis, detection and mitigation techniques etc.);
* absence of clear and agreed priorities for action;
* ease of introduction and movement (e.g. through the post), inadequate inspection and quarantine;
* inadequate monitoring capacity;
* lack of effective emergency response measures;
* outdated or inadequate legislation;
* poor coordination between government agencies, States and other stakeholders.

In 2008, measures will be proposed to tackle invasive 'alien' species, which threaten the survival of native species of fauna and flora. (Extract from the **2008 Annual Management Plan, DG Environment**).

http://www.europe-aliens.org/
http://www.issg.org/database/species/search.asp?st=100ss
http://www.issg.org/database/reference/index.asp
http://www.ciesm.org/online/atlas/intro.htm

3. Species related to human health

Human medicines, biomedical research, the emergence and spread of infectious diseases, and the production of food, both on land and in the oceans, depend on biodiversity. The **World Health Organization (WHO)** states in its report **"Biodiversity, Its Importance to Human Health", 2003**, that there is growing concern about the health consequences of biodiversity loss and change. An important consequence for humans is the disruption of ecosystems that provide nature's goods and services. Biodiversity loss also means that we are losing, before discovery, many of nature's chemicals and genes, of the kind that have already provided humankind with enormous health benefits. Some of the most endangered

organisms on Earth—sharks, bears, primates, amphibians, cone snails, gymnosperms, and horseshoe crabs—contributed already to human medicine, and others are expected to do so if we do not drive them to extinction.

In its report, the WHO emphasises the high value of plants, animals and microbes to medical research. Plant-based systems continue to play an essential role in health care. Approximately 80% of the world's population in developing countries rely mainly on traditional medicines, mostly derived from plants, for their primary health care. In addition to plants and microbes, there has been increasing attention paid to animals, both vertebrates and invertebrates, as sources for new medicines.

A very important area that is not usually considered is the use of natural compounds as agricultural agents or natural pesticides of many types that keep people healthy by maintaining adequate food supplies and preventing malnutrition. For example, one of the oldest and most successfully used plant products (from the 19th Century) is the powder from pyrethrum flowers, *Chrysanthemum cinerariaefolium*, originally native to the Dalmatian Mountains in Croatia.

Also, biodiversity can reduce pathogen transmission among hosts and therefore protect human health. Infection rates decrease as species numbers increase. So-called "reservoir" species can easily become infected with a disease. When these species have to compete with other species for resources, they encounter each other less often, infection rate remains low and disease does not spread widely. (Sustaining Life, 2008; Canary Database).

http://www.who.int/globalchange/ecosystems/biodiversity/en/index.html
http://chge.med.harvard.edu/programs/bio/index.html
http://canarydatabase.org/browse/species/?l=A
http://www.glopp.net/
http://www.loc.gov/rr/scitech/tracer-bullets/medicplantstb.html
http://www.pfaf.org/database/index.php

4 Indicator species for environmental (climate) change

Biological indicator species are unique environmental indicators as they offer a signal of the biological condition in an ecosystem and are a warning system that pollution has entered the food web or other environmental changes have occurred. The term indicator species is a bit misleading, as indicators are often whole groups of flora/fauna types, which can be used to assess environmental condition. However, the so-called "keystone" species can represent a community. These species are capable of expressing characteristics that can indicate the state of the ecosystem they currently occupy. Indicator species can leave clues about the state of the ecosystem; they "indicate" the state of the local environment. In the aquatic environment, most indicator species are fish, invertebrates, periphyton and macrophytes. Amphibians are also common indicator species, as they absorb substances easily. Frogs are a very good example of indicator species. When frogs show deformities or are in bad shape, there is certainly a problem in a nearby body of water.

A terrestrial example: *Biston betularia*, otherwise known as peppered moth, is a species that can adapt to polluted environments more suitably as a result of an adaptation changing the colour of them to suit their environment.

* A higher frequency of the light peppered moth would indicate that there is little pollution in the local environment

* A higher frequency of the dark peppered moth would indicated that there is high pollution in the local environment
* A decrease in light peppered moths' population may suggest that pollution is beginning to accumulate in the area.

In light of this, various species exhibit characteristics that give us insight into the local environment without having to study the local environment itself. In the case of the peppered moth being an indicator species, the presence of pollution (and dark moth) would indicate that additional abiotic stress is being placed on the organisms that live in that polluted (and usually less favourable) environment. Using bioindicators as an early warning of pollution or degradation in an ecosystem can help sustain critical resources. Available databases on Indicator Species:

http://www.biologie.uni-hamburg.de/b-online/bonn/Biodiv_mapping/biomaps.htm
http://herba.msu.ru/mirrors/www.helsinki.fi/kmus/botflor.html#feurope
