# Supplementary material for: PESI - a taxonomic backbone for Europe
Source: Biodivers Data J. 2015 Sep 28;(3):e5848. doi: 10.3897/BDJ.3.e5848 (PMC4609752; doi:10.3897/BDJ.3.e5848)
Supplement: Supplementary material 10 — PESI Focal Points Handbook [file biodiversity_data_journal-3-e5848-s010.pdf]

Focal Point Handbook

second edition

PESI Deliverable 3.4

March 2011

Acknowledgements

The WP3 management team wishes to thank those Focal Point partners that contributed to this second edition of the Handbook. We are indebted to the EcoServe staff, Dublin, Ireland for the initial coordination and the drafting of Chapter 7.

Feedback and suggestions

The handbook is a living document and will be regularly revised and updated according to Focal Point needs and requests. Your comments and suggestions are welcome for future editions. Please send feedback to the manager of Work Package 3: kouwenberg@uva.nl

Table of contents

Summary 5
1. Introduction 5
2. The PESI Project 6
3. The PESI Focal Point Network 7
   3.1 Description of the PESI Focal Point Partners 7
   3.2 Strategic roles of national Focal Points 10
4. How Focal Points contribute to the PESI e-infrastructure 11
   4.1 Results of Focal Point tasks and activities in PESI 11
   4.2 Visualisation of Focal Point efforts in PESI 14
   4.3 Results from Focal Point activities (proposals) 15
5. How Focal Points benefit from the PESI e-infrastructure and web portal services 17
   5.1 Expert-edited species databases in PESI 17
   5.2 Taxon match and national checklists 17
      5.2.1 Guidelines for using the Taxon match tool (validation national checklists) (prepared by Christos Arvanitidis, HCMR, Greece) 17
      5.2.2 Guidelines for the creation of national species checklists or national Data Portal, examples from the Azores and Ukraine 18
   5.3 Exchange tool between PESI and Focal Point websites (prepared by Roy Kleukers, NCB Naturalis, Leiden, NL; Ward Appeltans and IT team, VLIZ, Oostende, BE) 24
   5.4 E-science applications 25
   5.5 Multilingual portal pages 25
   5.6 EDIT Expert Net 25
   5.7 Focal Points communication: tools and data services 26
      5.7.1 The Focal Point mailing list 26
      5.7.2 The Focal Point discussion forum 26
      5.7.3 The Focal Point Handbook wiki pages 26
6. Establishing a Focal Point 27
   6.1 Organisation of Focal Points (Sweden, Lithuania, Poland) 27
   6.2 National best practices and lessons learned models (Ukraine, Norway) 29
7. Further information 32
   7.1 Legal issues and copyrights of Focal Point data in PESI 32
      a. Terms of use of data 32
      b. Termination of licence and rights of use 33
      c. Disclaimer 34
   7.2 Continuity plan (LifeWatch, GBIF, LIFE+ ambitions/organisation thematic topics/regional level) 34
   7.3 Preparation of joint proposals 34
   7.4 Focal Points influence in policy planning 35
      7.4.1 Policy plans - for national and international conservation purposes 35
      7.4.2 The potential of PESI and the FP Network as a tool in policy making 35
8. Feedback and suggestions 36
Please consult the Focal Point Handbook Wiki pages! 36
9. Contact Information 36
   9.1 PESI management contacts 36
   9.2 Focal Point data portals and useful websites per country 36
APPENDIX I 40
Summary of Recommendations for Controlled Vocabularies Use in the PESI Portal 40
   Occurrence Status 40

Taxon Status 40
Nomenclatural Status 41
Geographic Regions 43
APPENDIX II 45
The Darwin Core Archive Format (DwC-A) 45
What Does a DwC-A File Consists Of? 45
Simplest Authoring Scenario 45
Taxonomic Hierarchies 46
Multiple hierarchies. 46
Columns in the Spreadsheet 46
Common Columns 46
Normalised Taxonomy Columns 48
Flattened Taxonomy Columns 48
Names vs Taxa 48
Additional Nomenclatural Information 48
Default Values 49
Vernacular Names 49
Distributions 49
Taxon Identifiers 49
Provenance Data 49
RDF and the Semantic Web 50

Summary

The Focal Point Handbook deliverable is a living document that will serve as a guide to the existing PESI focal points (49) and new focal points to be included into the Focal Point network in the future. It first gives a short description of the PESI project, the PESI Focal Point partners and their strategic roles in a wide-European perspective. A short description of the tasks and activities for the contracted Focal Points is followed by their contributions to the PESI e-Infrastructure. The visualisation of these contributions is supported by screenshots of the Focal Point Expertise database with explanation how to search for provided data. The Handbook further provides guidelines for using the Taxon Match tool, the Focal Point communication tools and Wiki pages, finding taxonomic Experts and taxon-related literature. Guidelines are given for the establishment of a Focal Point and the development and creation of national species checklists through examples and lessons learned of existing Focal Points. The PESI portal appears in 19 languages. Further information addresses the legal issues and copyrights, continuity plan, influence in policy planning, how to feedback and make suggestions. Finally, a long list of useful websites per participating country completes the present version of the hard copy Handbook.

1. Introduction

This handbook is being developed for Focal Points within the PESI consortium (both established and non-contracted associate FPs) and future Focal Points for readily accessible guidance. The information and material in this handbook are provided as both a reference and a guide. The handbook may also be useful for the other PESI partners and everyone involved in the validation process of (national) species lists.

The handbook informs about Focal Point roles, functions and benefits as well as on the strategic coordination and integration process of biodiversity resources (checklist custodians) that PESI Focal Points facilitate at country level.

This material is presented in a way that users can retrieve information easily. The handbook is a dynamic document and will also be publicly accessible on the near-future “PESI Focal Point Wiki Pages” where it will be regularly revised and updated according to Focal Point needs and requests.

Organisation of the Focal Point handbook

This handbook is organised in chapters on the following topics

- The PESI project and Focal Point network
- Focal Point benefits from PESI
- Models for continuity beyond PESI

2. The PESI Project

The PESI Project: http://www.eu-nomen.eu/pesi/ seeks to integrate the European taxonomic e-infrastructures, with 25 European member countries, and several neighbouring countries (Russia, Ukraine, Caucasus region, Israel, Turkey) participating in these efforts. It will function as a taxonomic backbone for Europe and the western Palaearctic region.

The projects main objectives are:

a) The standardisation in taxonomic reference systems
b) Quality improvement and completion of the main European taxonomic, expert-edited data sets
c) The creation of integrated, user-friendly access to taxonomic information in all European languages (species, experts, classification and additional data)

The methods to accomplish these objectives are:

a) Upgrading the content and infrastructure of three main species databases registered in Europe, covering (almost) all wildlife forms: Fauna Europaea (FaEu); Euro + Med PlantBase (E+M); European Register of Marine Species (ERMS). Key-activities comprise increasing the compatibility between the databases in terms of content, enabling data integration using data standards, creating mirror sites.
b) Building a pan-European network of taxonomic expertise, coordinated through national Focal Points. The expertise comprises: 1) the experts in species taxonomy, who will quality control the taxonomy and national species inventories; 2) the Taxonomical Institutions; 3) Taxonomical Societies across Europe and (provisionally) the Western Palaearctic.
c) Setting up a common data-portal for integrated (a and b) taxonomic access: http://www.eu-nomen.eu/portal/ to serve a wider range of users than the present resources, providing the first synonymised up-to-date inventory of all species in Europe. Species of special importance (EU legislation lists, conservation, pests, invasive) are given special attendance with additional data, pictures, common names, links, importance on national levels, etc. The data portal also includes a taxon match tool for the quality check of species names, an exchange tool between PESI and Focal Point websites, and interoperability with scientific publications, including journals.
d) Developing sustainability strategies for securing the long-term sustainability and maintenance of the PESI databases.

The resulting data portal will allow for rapid retrieval of all authoritative, species-related information. This includes taxonomic history, alternative names and classification, vernacular names in most European languages and those from the participating neighbouring countries, relevance to legal provisions, literature references, contact details of specialists and many taxonomic metadata services.

3. The PESI Focal Point Network

The PESI Focal Point network holds a key function in the PESI project regarding the continuity of building the expertise network, the compilation, update and quality check of the national species checklists, the creation of awareness and usage of the PESI tools at a national level. The Focal Points also form an important link in the process of unlocking local and regional taxonomic species inventories and making those available to the PESI user community and much more.

3.1 Description of the PESI Focal Point Partners

Fig. … gives an overview of the PESI Focal Points in Europe and neighbouring countries. The white-dotted pointers indicate the established FaEu Focal Points, the green E+M Focal Points, the blue ERMS Focal Points. Pointers without dot indicate the associate, non-contracted Focal Points. For a clickable map, visit: http://www.eu-nomen.eu/pesi/index.php?option=com_content&view=article&id=93&Itemid=64

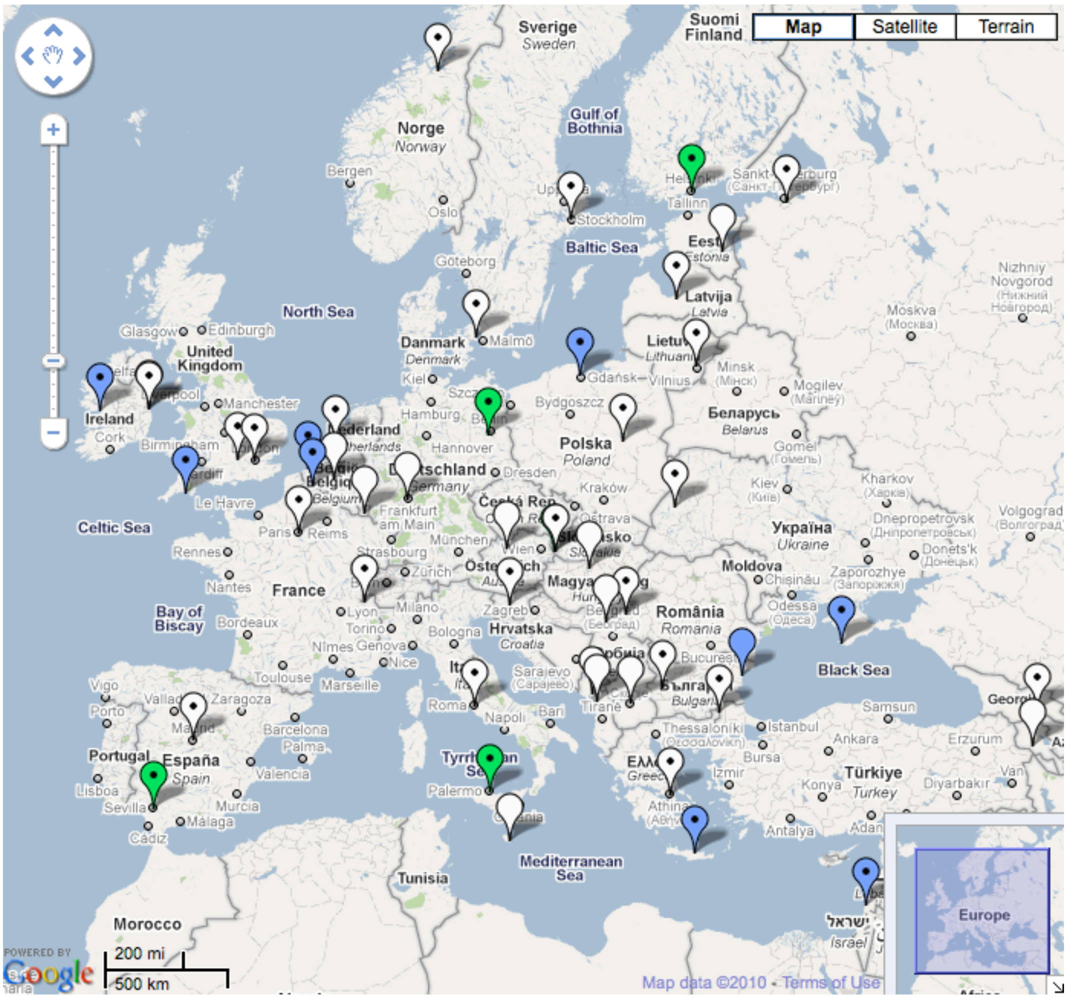

A total of 34 established and 15 non-contracted Focal Points from 38 countries are listed in the table below, including contact details.

Ptcp No.	Organisation	City, Country	Contact person	Email address	Focal Point
2	University of Copenhagen (UCPH)	Copenhagen , Denmark	Dr.Henrik Ærenlund Pedersen	henrikp@snm.ku.dk	FaEu
3	University of Trakya (TU)	Edirne, Turkey	Prof. Nihat Aktaç	nihata@trakya.edu.tr	FaEu
4	Natural History Museum (NHM)	London, United Kingdom	Dr. Charles Hiussey	c.hussey@nhm.ac.uk	FaEu
5	Freie Un. Berlin, Botanic Garden, Botanic Museum (BGBM)	Berlin, Germany	Dr. Eckhard von Raab-Straube	e.raab-straube@bgbm.org	E+M
6	Flanders Marine Institute (VLIZ)	Oostende, Belgium	Dr. Ward Appeltans	ward.appeltans@vliz.be	ERMS
7	Ecological Consultancy Services Ltd (Ecoserve)	Dublin, Ireland	Dr. Roisín Nash	roisin@ecoserve.ie	FaEu
9	Muséum National d'Histoire Naturelle (MNHN)	Paris, France	Dr. Olivier Gargominy	gargo@mnhn.fr	FaEu
13	National University of Ireland (NUIG)	Galway, Ireland	Dr. Roisín Nash	roisin@ecoserve.ie	ERMS
15	University Palermo, Department Botanical Sciences (DPCE)	Palermo, Italy	Dr. Gianniantonio Domina	gdomina@unipa.it	E+M
16	University of Seville, USE	Sevilla, Spain	Prof. Benito Valdès	bvaldes@us.es	E+M
17	Institute of Botany, Slovak Academy of Sciences (IBSAS)	Bratislava, Slovakia	Dr. Karol Marhold	karol.marhold@savba.sk	E+M
18	National and Kapodistrian University of Athens (NKUA)	Athens, Greece	Dr. Anastasios Legakis	alegakis@biol.uoa.gr	FaEu
19	National Museum of Natural History Naturalis (NNM)	Leiden, The Netherlands	Dr. Roy Kleukers	roy.kleukers@ncbnaturalis.nl	FaEu
20	Nature Research Centre (EKOI) (formerly Institute of Ecology of Vilnius University)	Vilnius, Lithuania	Dr. Eduardas Budrys	ebudrys@ekoi.lt	FaEu
21	Scientific Committee for the Italian Fauna (CSFI)	Rome, Italy	Dr. Fabio Stoch	fabio.stoch@gmail.com	FaEu
22	Swedish Museum of Natural History (NRM)	Stockholm, Sweden	Dr. Sven Kullander	Sven.Kullander@nrm.se	FaEu
23	Comenius University Bratislava (CUB)	Bratislava, Slovakia	Dr. Eduard Stloukal	stloukal@fns.uniba.sk	FaEu
24	Museum of Natural History and Archaeology - Un. Sci. and Techn. (NTNU)	Trontheim, Norway	Dr. Kaare Aagaard	kaare.aagaard@vm.ntnu.no	FaEu
25	State Museum of Natural History, Nat. Ac. of Sciences of Ukraine (SMNH)	Lviv, Ukraïne	Dr. Volodymyr Rizun	rizun@museum.lviv.net	FaEu
26	Museum and Institute of Zoology - Polish Academy of Sciences (MIZPAN)	Warsaw, Poland	Prof. Wieslaw Bogdanowicz	wieslawb@miiz.waw.pl	FaEu

27	Swiss Systematics Society (SSS)	Genève, Switzerland	Dr. Jean Mariaux	Jean.Mariaux@ville-ge.ch	FaEu
28	Ilia Chavchavadze State University (ILIAUNI)	Tbilishi, Georgia	Prof. David Tarkhnishvili	davitar@gmail.com	FaEu
29	Consejo Superior de Investigaciones Cientificas (CSIC)	Madrid, Spain	Dr. Marian Ramos	m.ramos@mncn.csic.es	FaEU, ERMS
30	Slovenian National Institute of Biology (NIB)	Ljubljana, Slovenia	Dr. Davorin Tome	Davorin.Tome@nib.si	FaEu
31	National Museum of Natural History - Sofia (NMNHS)	Sofia, Bulgaria	Prof. Alexi Popov	alpopov@bulinfo.net	FaEu
32	myNature Association (myNA)	Timisoara, Romania	Dr. Adorean Ardelean	mynature@gmail.com	FaEu
33	University of Latvia (LU)	Riga, Latvia	Dr. Voldemars Spungis	adalia@lanet.lv	FaEu
34	Hellenic Centre for Marine Research (HCMR)	Heraklion, Crete, Greece	Dr. Christos Arvanitidis	arvanitidis@her.hcmr.gr	ERMS
35	Israel Oceanographic and Limnological Research (IOLR)	Haifa, Israel	Prof. Bella Galil	galil@math.tau.ac.il	ERMS
36	Institute of Oceanology of Polish Academy of Sciences (IOPAN)	Sopot, Poland	Prof. Jan Marcin Weslawski	weslaw@iopan.gda.pl	ERMS
37	Zoological Institute of Russian Academy of Sciences (ZIN RAS)	St.-Petersburg, Russia	Dr. Sergei Medvedev	fleas@zin.ru	FaEu
38	A.O.Kovalevsky Inst. of Biol. of the Southern Seas (IBSS)	Sevastopol, Ukraine	Prof. Volodymyr Vladymyrov	v.vladymyrov@ibss.org.ua	ERMS
39	Marine Biological Association of the United Kingdom (MBA)	Plymouth, United Kingdom	Dr. Dan Lear	dble@mba.ac.uk	ERMS
40	University of Sciences and Technology of Lille (USTL)	Lille, France	Prof. Jean-Claude Dauvin	jean-claude.dauvin@univ-lille1.fr	ERMS
0	University of Tartu (UTEE)	Tartu, Estonia	Dr. Mati Martin	mati.martin@ut.ee	FaEu
0	Upper Austrian Provincial Museum (OOE.BZ)	Linz, Austria	Dr. Michael Malicky	m.malicky@landesmuseum.at	FaEu
0	Hungarian Natural History Museum (HNHM)	Budapest, Hungary	Dr. Laszlo Peregovits	laszlo.peregovits@gmail.com	FaEu
0	Senckenberg Naturmuseen und Forschungsinstitute (SNG)	Frankfurt, Germany	Dr. Michael Tuerkay	michael.tuerkay@senckenberg.de	FaEu
0	University of Madeira, Dept. of Biology (UMDB)	Funchal, Madeira, Portugal	Dr. Antonio Abreu	antoniodabreu@netmadeira.com	FaEu
0	Belgian Biodiversity Platform (BBPF)	Brussels, Belgium	Dr. Hendrik Segers	hendrik.segers@naturalsciences.be	FaEu

0	Musée National d'Histoire Naturelle (MNHN)	Luxembourg, Luxembourg	Dr. Mark Meyer	zool.invert1@mnhn.lu	FaEu
0	Malta Environment and Planning Authority (MEPA)	Floriana, Malta	Dr. Marie-Thérèse Gambin	marietherese.gambin@mepa.org.mt	FaEu
0	Montenegrin Academy of Sciences and Arts (MASA)	Podgorica, Monte Negro	Prof. Gordon Karaman	karaman@t-com.me	FaEu
0	University of Shkodra, Faculty of Natural Sciences (US)	Shkodra, Albania	Prof. Dhimiter Dhora	dh-dhora@unishk.edu.al	FaEu
0	Macedonian Museum of Natural History (MMNH)	Skopje, Macedonia	Dr. Vladimir Krpach	vkrpach@gmail.com	FaEu
0	Institute of Oceanology (IO-Bas)	Varna, Bulgaria	Dr. Snezhana Moncheva	snejm@varna.techno-link.com	ERMS
0	National Academy of Sciences of Armenia, Institute of Zoology (NASA)	Yerevan, Armenia	Prof. Hasmik Khachatryan	hasoika@yahoo.com	FaEu
0	Centre for the Balkan Biodiversity Conservation (CBBC)	Novi Sad, Serbia	Prof. Smiljka Simic	smiljka.simic@dbe.uns.ac.rs	FaEu
0	Azoran Biodiversity Group	Terra Chä, Azores, Portugal	Prof. Paulo Borges	pborges@uac.pt	FaEu

3.2 Strategic roles of national Focal Points

Operational Focal Points can be seen as strategic pillars between the local taxonomy/biodiversity expertise groups, the national governments, and the European taxonomic infrastructure. They liaise with governmental bodies regarding the implementation of European standards relevant to regulations and environmental monitoring. They document local expertise and applied tools for the greater European taxonomic community. They hold workshops on key topics, like 1) data management; 2) validation of national species checklists; 3) cooperation with national policymakers; 4) tool application for cross-linking of web-resources. Results of workshops will lead to the spread of best practices. These best practices will be documented in the Focal Point Handbook Wiki Pages < http://pesifocalpointhandbook.wikispaces.com/>.

4. How Focal Points contribute to the PESI e-infrastructure

4.1 Results of Focal Point tasks and activities in PESI

D3.1, the "Focal Points Working Plan": http://www.eu-nomen.eu/pesi/index.php?option=com_remository&Itemid=56&func=fileinfo&id=384 gives detailed descriptions of Focal Point tasks and activities, including dependencies with other work packages and the envisaged workflow.

The prioritised tasks for which seed money was allocated to the established focal points (D3.2, the "Focal Point Seed Money Allocation Plan") and resulting deliverables are summarised below:

Connection, relationships with relevant national biodiversity resources (national checklist owners, etc)

Two Focal Points (NL, UA) have submitted a short report regarding relevant connections at a national scale for PESI-related networking activities:

Netherlands. The PESI tasks for the Netherlands are based on two projects, in which almost all Dutch biodiversity specialists are involved.

1) Within the website Dutch Species Register (www.nederlandsesoorten.nl) the checklists on all Dutch multicellular species have been standardized and brought together. The project is co-ordinated by NCB Naturalis en the European Invertebrate Survey – the Netherlands, in co-operation with all major institutions dealing with Dutch biodiversity, such as natural history museums, volunteer recording organizations and research institutes.

2) In 2009 the initiative was taken to produce a book on Dutch biodiversity in 2010. For this cause the Dutch specialistst have critically evaluated their own species lists and written texts on their groups, together creating an up-to-date overview of Dutch biodiversity. The information will also be available through the Dutch Species Register and therefore for PESI.

In the coming years NCB Naturalis will continue to host and support the Dutch Species Register. Our focal point is interested in being involved in the future development of PESI.

Ukraine. Negotiation and relationships exist with owners and authors of the following: the checklist of animals of Ukraine (Dr. Zagorodniuk I., Luhansk Taras Shevchenko National University), the data base of Ukrainian bats (Dr. Godlevska O., Schmalhausen Institute of Zoology of National Academy of Sciences of Ukraine), the checklist "Birds of Ukraine" (Dr. Bokotey A., State Museum of Natural History, National Academy of Sciences of Ukraine (SMNH)), the rare and endangered species of the Ukrainian Carpathians data base (Dr. Mateleshko Yu., Uzhgorod National University), the rare and endangered species of the Western Ukraine data base (Dr. Yanytsky T., SMNH), the zoological collections of the SMNH (Lviv), the Flora Carpathica (Prof. Tasenkevych L., SMNH) were carried out. All of these persons are ready to cooperation without preconditions or, sometimes, in various contractual conditions.

Almost all biosphere reserves (Carpathian Biosphere Reserve), natural reserves (Rivnenskyi, Cheremskyi, Roztochchia, Gorgany, Medobory), national parks (Shackyi, Prypiat-Stokhid, Yavorivskyi, Halyckyi, Skolivski Beskydy, Carpathian, Huculshchyna, Vyzhnyckyi, Synevyr, Uzhanskyi, Podilski Tovtry, Mezynskyi) (the owners of faunistic information) we have contacted with, are ready to co-operate with the national FaEu Focal Point.

New edition of the Red Data Book of Ukraine (2009) at this moment is at editors' and will be published soon. From the previous edition of the Red Data Book of Ukraine (1994) 31 species of animals were excluded, and the list of animals in comparison with the older edition (352 species) increases up to 542 species.

Build relationships with other EU or globally oriented national biodiversity nodes (CBD, Natura 2000, IUCN, WWF, WHO, GBIF etc.)

Three Focal Points have reported their activities regarding the relationships with national biodiversity nodes:

Georgia. ILIAUNI has good working relations with CBD- national Focal Points, Georgian National Red List Commission, WWF Caucasus, Caucasus Biodiversity Council. Agreed joint activities:

ILIAUNI and Georgian National Red List Commission will cross - reference their databases: Georgian Biodiversity Database (www.biodiversity-georgia.net) will include red list categories for at least all species listed on Georgian National Red list Commission's website (www.red-list.ge). (Georgian Biodiversity Database already includes IUCN categories for some species, however it is not linked with Georgian National Red list Commission's website.)

Netherlands. National Database of Flora and Fauna (NDFF): In 2011 the species lists of the Dutch Species Register (NLSR) will be compared with, and synchronized with, the National Database of Flora and Fauna (NDFF). A tool will be developed which will keep the taxonomy of the NDFF up-to-date, with NLSR as leading database. Organisation: NCB Naturalis, European Invertebrate Survey – NL, VOFF (Foundation for the study of Flora and Fauna)

Orthoptera of Europa (www.ortheur.org): A website on European Orthoptera, based on the checklist in Fauna Europaea. We will investigate how we can keep the country distribution table updated and synchronised with Fauna Europaea / PESI.

Encyclopedia of Life (EOL): NCB Naturalis makes information and illustrations from NLSR available for EOL. The best way to present this information is being negotiated. NLBIF/GBIF: Information from NLSR is presented in NLBIF/GBIF. Closer co-operation is in discussion.

Ukraine. The biodiversity-related nodes in Ukraine have different subordination (are situated and are under influence of different institutions). Some of them are under a Ministry level, some under institutional level, activities of some nodes depend on a single responsible person. Therefore, PESI WP-3 FaEu NFP negotiations have different progress and level of co-operation. We will continue this process. Furthermore, these national nodes have the different significance (are the owners of the different kind of information on biodiversity) for cooperation with PESI. First of all our efforts were aimed at those who have greater importance in PESI activities.

The proposed joint activities for Focal Point network functioning could be as follows:

CBD - the main activities concerning this national node could be: the co-operation with Ministry of Environmental Protection of Ukraine and applying of PESI opportunities (national experts web service, biodiversity data and others) to the national policy in the protection areas, Global Taxonomy Initiative, biodiversity monitoring, target species monitoring.

WWF – the main activities concerning this national node could be: the applying of PESI opportunities (national experts web service, biodiversity data, data on target species and others) into WWF Conservation Projects.

Natura 2000 – the main activities concerning this national node could be: the using of the PESI national expert web service, the biodiversity data and data on the target species for elaboration of biotopes' classification.

IUCN – at present the directions of co-operation with PESI NFP are not clear, because activities of NECU (representative of IUCN in Ukraine) are very multilateral.

WHO – the main activities with this national node could be: the exchange with information on a part of the target species.

GBIF – the main task is to persuade a ministry servants of the importance of joining Ukraine to the GBIF.

CERI – the main activities concerning this national node could be: the participation of the PESI experts in different projects and exchanging with biodiversity information in the Carpathians' region.

USBP - the main activities concerning this national node could be: cooperation in ornithological investigations and exchanging with biodiversity information.

URNFP - the main activities concerning this national node could be: the participation of the PESI experts in different projects and exchanging with biodiversity information on wetlands conservation.

Cross-validation of national inventories and checklists against the PESI pan-european checklist using the Taxon Match tool, provided by WP6

At the moment of submission of this handbook a total of 49 national checklists were cross-validated with the PESI species database using the Taxon Match tool (B-version). These comprise lists from **Turkey, Ukraine, Georgia, United Kingdom, Greece, Israel, Bulgaria, Poland, and Spain**. The mismatches from these files will be forwarded to the taxonomic experts for study and updating. We expect that many focal points will have their national checklists validated after the official launching of the PESI portal.

Inventory of additional data types in national and major regional checklists

Nine Focal Points have sent an inventory of their national checklists and additional data types (**Spain, Denmark, France, Ireland, Italy, Netherlands, Ukraine, Lithuania, Bulgaria**). For a complete overview of present and missing data we will need more inventory information from the other focal points. Then, an analysis for gaps can be made and joint proposals developed for completion of standardised national checklists at a wide-European scale. The Focal Point Handbook Wiki pages will allow putting forward this process under proper coordination beyond PESI.

Vernacular names of prioritised taxa obtained from national and major regional checklists

Almost all focal points have submitted the vernacular names in their language for the European prioritised species (EU Habitat Directive, EU Birds Directive, IUCN red list, ORPAS, CITES, EPPO, HYPPZ Pest Species) and many more. These are all included in the PESI Data Portal.

Inventory and delivery of national prioritised species lists

Seven lists of national prioritised species were received from four focal points (**Turkey (2x), Ukraine (2x), Poland, UK, Bulgaria**). These species lists will be visible on the PESI Data portal Taxon Search page, provided that the listed species match with the PESI database.

Support with translations to establish a multilingual web portal

At the moment of submission of this Handbook, the PESI Data Portal exists in 18 languages from European and neighbouring countries: **Bulgarian, Dutch, English, German, Georgian, Hebrew, Irish, Italian, Latvian, Lithuanian, Norwegian, Romanian, Slovak, Slovenian, Spanish, Swedish, Turkish, Ukrainian.**

Deliver metadata (profile) of national/local/regional taxonomic data resources and expertise

A valuable collection of many regional and national taxonomic experts, societies and resources was received from most focal points, comprising more than 2000 experts, some 1000 institutions and journals, hundreds URLs. Uploading of all these data in the searchable Focal Point expertise database on the PESI Portal is in progress http://www.eu-nomen.eu/portal/imis.php?module=person&firstview=1.

Application for national funds regarding WP3 activities, FP outreach

Chapter 4.3 of this Handbook gives an overview of the various proposals, submitted by the focal points.

Plan development for a formal FP organisation or network

At the moment of submission of this Handbook a formal organisation of a Focal Point network is in discussion. It is proposed that the Focal Points officially form a joint Society or Alliance and in that form become a member of SMEBD. This can be done when SMEBD has become an independent society. Another possibility is involvement of the Network into the LifeWatch Programme.

Contributions to FP handbook (best practices etc)

A number of Focal Points have committed to contribute to the development and realisation of this Handbook. These Focal Points are co- authored. More Focal Points will contribute to future updates. One of them will take the lead to coordinate this process, as well as publication. The results from these activities will be available on the PESI Focal Point Handbook Wiki pages.

4.2 Visualisation of Focal Point efforts in PESI

The PESI Data Portal visualises and disseminates the Focal Point efforts by including the assembled data in the various sections of the PESI Data Portal, as highlighted in the screenshots below (yellow arrows refer to the data contributed by the Focal Points).

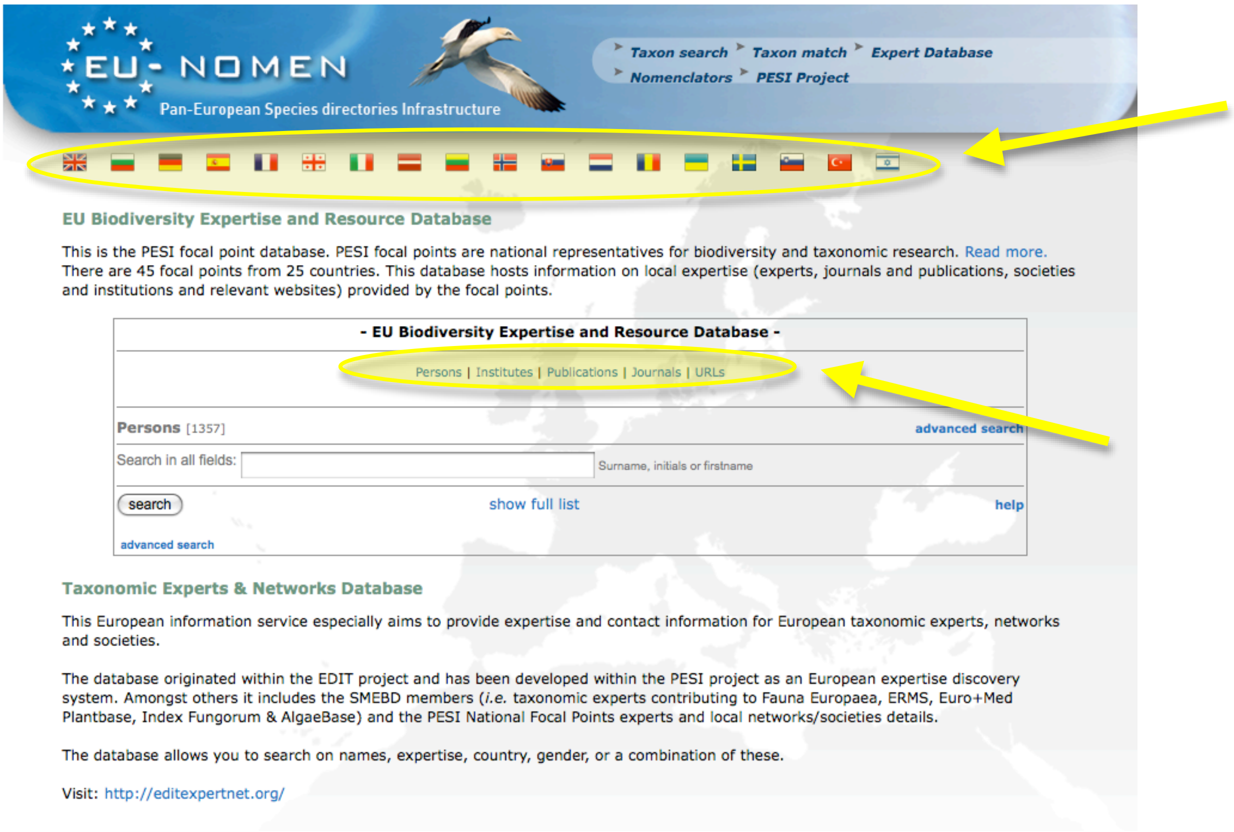

The data portal will be available in as many languages as there are national Focal Points. Below is an example of a protected species with vernacular names, submitted by the Focal Points. At the right side are links to the species at other websites with detailed information.

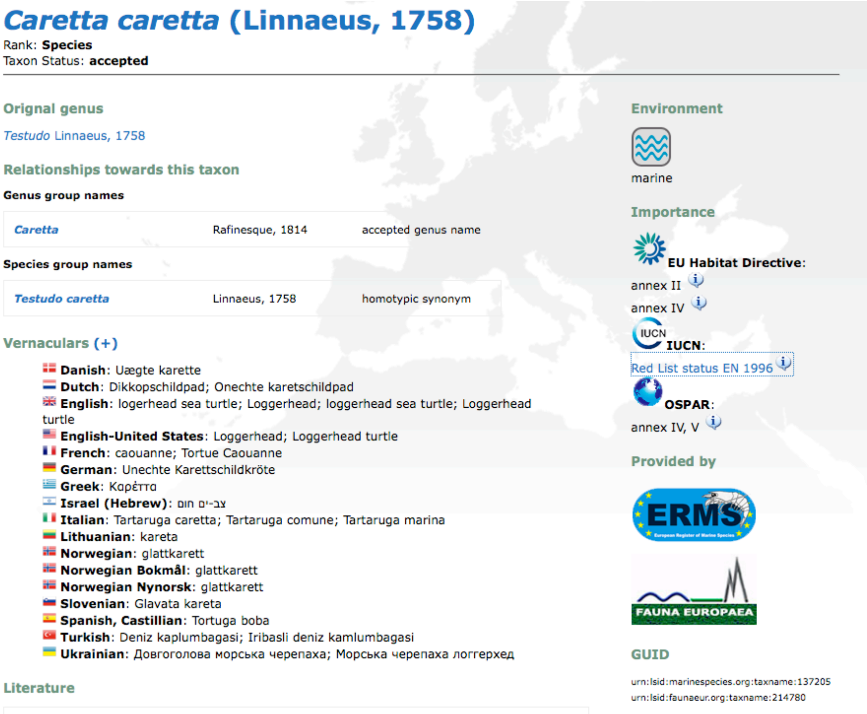

More Focal Point data are planned for the PESI web portal in the near future, like national distribution maps, overlaying the European one (tool in development WP1 and WP6). This will give information about the anomalies in presence/absence of species according to the European databases in comparison with the national species checklists. A pilot will comprise the national prioritised species lists in comparison with the European prioritised species lists.

4.3 Results from Focal Point activities (proposals)

One of the Focal Point tasks includes proposal development and submission. Some examples of submitted proposals are given below. Feedback (ideas, joining) from other focal points to the submitters is greatly appreciated.

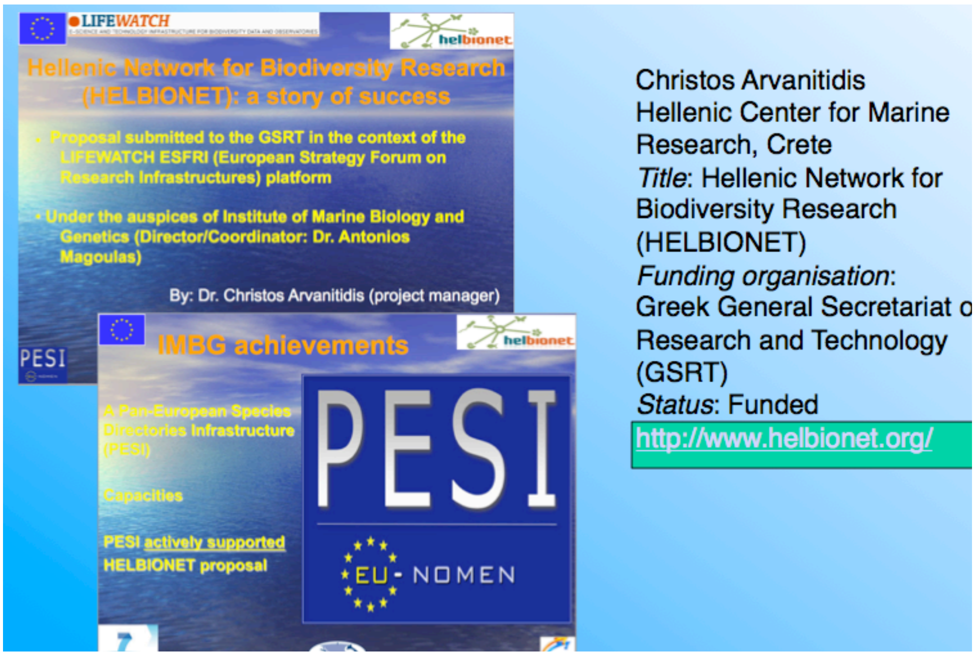

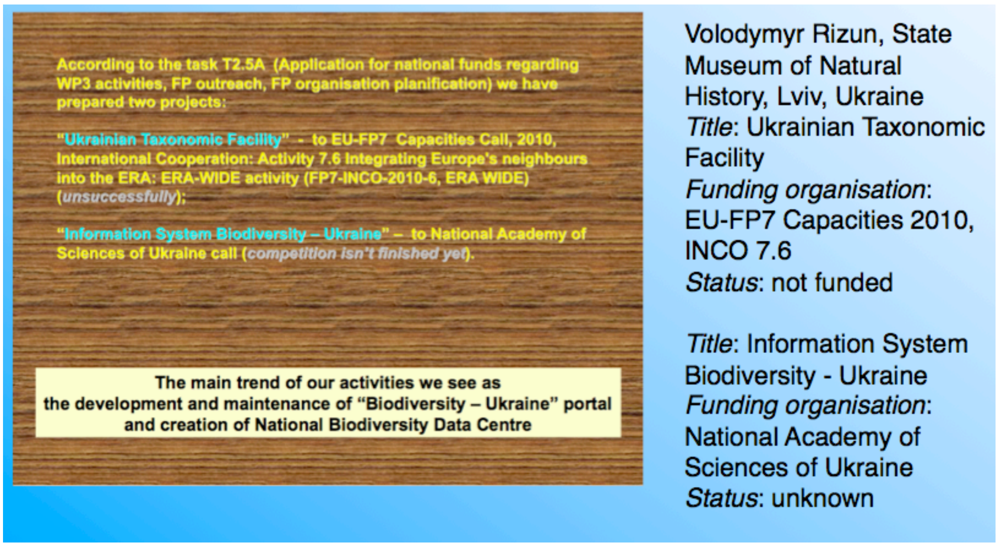

A proposal with a regional character, submitted within the framework of EU-FP7 was not funded. This proposal may be revised and resubmitted.

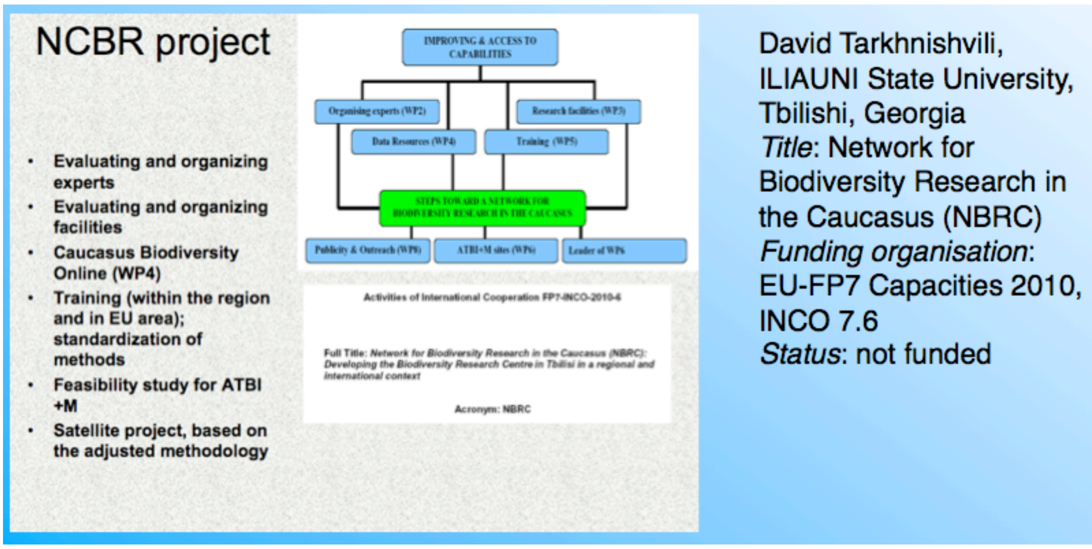

The Fauna Iberica X proposal below was granted:

http://www.fauna-iberica.mncn.csic.es/english/index.php

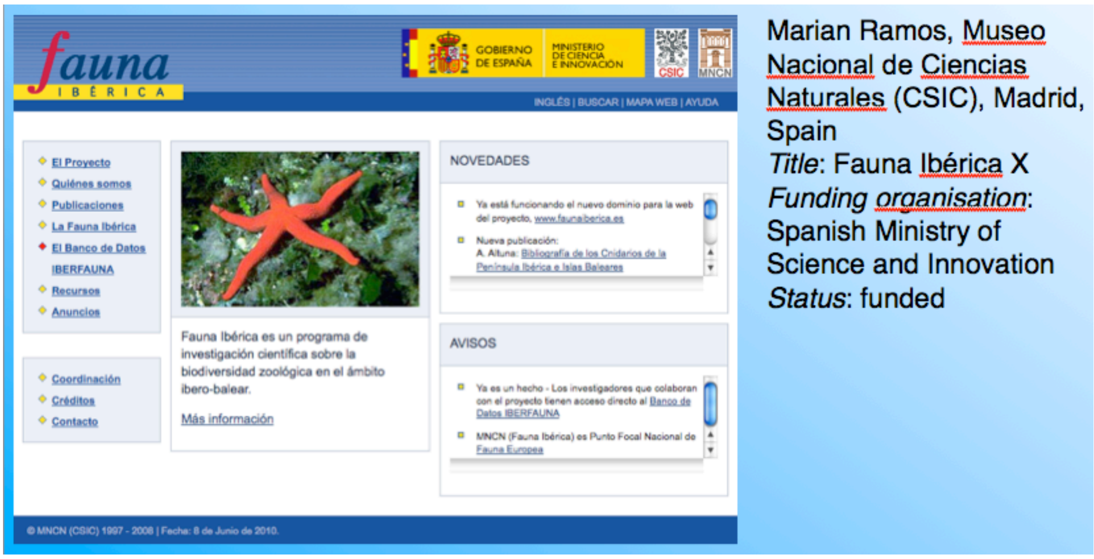

5. How Focal Points benefit from the PESI e-infrastructure and web portal services

5.1 Expert-edited species databases in PESI

The PESI web portal has published an integrated European species list, including the expert-edited species checklists and databases:
FaEu (http://www.faunaeur.org/about_fauna_intro.php),
E+M (http://www.emplantbase.org/home.html),
ERMS (http://www.marbef.org/data/erms.php),
Index Fungorum-PESI (http://pesi.indexfungorum.org/),
Desmids (from Algaebase) (http://www.algaebase.org/)

The portal allows users to search species by:

- Scientific name (any taxon rank)
- Common name (any taxon rank)
- Geography (country, region, seas)
- Environment (marine, freshwater, land)
- Other attributes (e.g. priority/protection status)

The PESI web portal also has an end-user forum for user input on functional requirements and as a feedback mechanism for data quality assurance.

5.2 Taxon match and national checklists

Focal Points can benefit using the "Taxon Match Tool" for validation of the national species checklists. The Taxon match applications include:

- spelling-check of taxa
- authority output for taxa
- full classification for taxa
- resolve all invalid names to valid ones
- match species lists
- resolve a common name to a scientific name
- get the sources/references for a taxon

5.2.1 Guidelines for using the Taxon match tool (validation national checklists) (prepared by Christos Arvanitidis, HCMR, Greece)

NOTE BEFORE YOU START: Your *.xls file to upload should have less than 1000 rows with data. Subspecies are not treated by the tool. Low matching percentage can have several reasons, including typos in authority names, wrong year etc.

1. Prepare an *.xls file with your taxa names in the first column (up to 999 names). You can have either taxa names only or the full name string including taxa names, author and year, but in the latter case you will have a lower matching rate.
2. Go to http://www.eu-nomen.eu/pesi/ and log in. If you have no username and password you can ask the PESI manager; if you have forgotten your username or

password you can click on the phrases: “forgot your username” and/or “forgot your password”, which are written in blue letters. Once registered, click on the box “remember me” (login will not be necessary once the Portal is launched)..
3. On the left menu click on “Pesi Data Portal”.
4. On the menu appearing on the new page click on the “taxon match” button.
5. A new page appears now that allows you to upload your *.xls file and to follow the taxon matching process.
6. Upload your *.xls file by clicking on the “Browse” button that allows you to browse your file in your own hard disk.
7. Click on the box “First row contains column names” only if your file has indications on the first row of the data sheet.
8. Choose the type of your file through the box “Column delimiter”; normally the first (default) choice (tab delimited) is fine.
9. Then click on the box “Match authority”.
10. Click on the box “Limit to taxa belonging to” only of you want to limit your search certain taxa with certain names.
11. Check all boxes you wish appearing on the last choice (Output): “GUID”; “Scientific name”; “Authority”; “Accepted name”; “Classification”; “Quality status”; “Taxon status”; “Citation”.
12. Click on “Next” and wait. It may take a few seconds if your file is large.
13. A new box is appearing with the species names of your list. Click on the button “Match” which is under the box.
14. Now two columns appear on your box: one is your submitted species list and the other is the column with matched names from the PESI species lists along with the status. Above the box the matching percentage is indicated.
15. By choosing the button “Excel sheet” (default) click on the “Download” button to save the taxon matching *.xls file on your disk. This is the end of your first taxon matching trial on the PESI web services. Click on the blue letters “new match”, next to the matching percentage, to start the process on a new file. If you wish at this point to quit the taxon match web page, then click on the “Pesi Project” button and log out the PESI EU-Nomen web portal.
16. Now, you have to review the names that did not match with the PESI species list. Reasons for non-matching can be numerous. Often it concerns mis-spellings, typos or other simple errors. This is when the cleaning process starts.
17. Clean the file, correct the obvious errors and repeat the matching. This cleaning may take several cycles. Where appropriate consult the experts in your country.
18. Once you have consulted the experts of your country and possibly solved all of the names mismatches, then save the new *.xls file with both matched and mismatched names.
19. Follow steps 2-17 once again. By the end you can download, again, a new *.xls file with some names potentially still mismatched. If you can consult your experts, once more, this will solve some of the remainder mismatches. Then, submit the final list to the PESI data coordinators.

5.2.2 Guidelines for the creation of national species checklists or national Data Portal, examples from the Azores and Ukraine

How to create a regional online biodiversity portal step by step

(**from publication by Borges et al., 2010, *Systematics and Biodiversity,* 8: 423-434**)

1. Team

(a) Promote a multidisciplinary network of specialists on: (i) the taxonomy of the target

groups, (ii) databasemanagement,
(iii) web design and (iv) knowledge organization systems (KOS).
(b) Manage the different kinds of expertise available on the team, based on the specific needs of each project phase.
Note: It is essential to select or train technicians in taxonomy and GIS and promote collaboration with image providers (professionals and amateurs), ethnologists and historians (for obtaining local species names).
2. Data sources
(a) Plan a comprehensive strategy of information gathering, selecting an array of data sources, including all published literature and reports coming from mapping projects, faunistic monitoring, animal collections, herbaria, letters, travel reports and journals and other private unpublished records. If not available, an important step is the creation of a checklist of all the species occurring in the target region and an exhaustive list of synonyms. This will allow an accurate interpretation of the old historical literature.
(b) Obtain all the possible environmental information for the target region (e.g. digital elevation maps, spatial location of roads, pathways and water courses, names of localities) to be added as layers in a GIS environment. This will allow a correct interpretation of the distribution of species.
(c) Guarantee that all the information is adequately stored and available for consultation.
3. Database
(a) Design a database for biodiversity data storing and management. So far we have been successfully using SQL. If possible, we suggest following the TWDG standards, such as Darwin Core.
(b) Choose software (e.g. Atlantis Tierra 2.0 or internet platforms) for an easy interface with the database allowing its proper feeding.
(c) Determine optimal solutions to minimize errors in feeding the database. In addition, the technicians should work in close connection with the taxonomic coordinators allowing an easy interpretation of the taxonomic literature.
Note: The software may use the SQL language to develop interrogation queries and should have an easy interface with all Geographic Information System (GIS) software. Ideally the feeding of data should be performed online facilitating the participation of experts of different regions or countries (see also Frazier *et al.*, 2008 for a detailed appraisal on how to initiate a digitization project).
4. Biodiversity portal
(a) Define the aims of the portal and the services to be provided.
(b) Together with a web designer, create its architecture.
Note: It is important to guarantee a pleasant and informative experience to the user. Therefore, all efforts should be taken to allow an easy access to the information, providing a clear, structured and intuitive navigation.
(c) Promote the sharing of information among users and data providers (e.g. allowing access to the original database; receiving and making available information collected by users such as pictures, new records of species or locations).
(d) Ensure the updating of all the available information and create new resources, as they are needed/requested, assuring that all the novelties are easy to see and access.
(e) Create a regulation system that allows the validation and monitoring of the comprehension, functionality and satisfaction of the users in all stages of the process, conjugating quantitative (e.g. number of visits, number of downloads) and qualitative (e.g. feedback on-line questionnaires, e-mail messages) data.
5. Funding and long-term management
(a) Last but not least, guarantee the interest of funding agencies, for the short- and long-term maintenance of both the database and the portal.

(b) If possible, maintain partnerships with conservation and education stakeholders that endorse the use of the portal as a resource to promote the biodiversity of the region.

Wiki-based tool for creation of marine checklists

Oleksandra SERGEYEVA, Volodymyr VLADYMYROV, Kseniia SKURATOVA

Institute of Biology of the Southern Seas, National Academy of Sciences of Ukraine, 2, Nakhimov av., Sevastopol, 99011, Crimea, Ukraine

During the FP-6 Black Sea Scientific Network Project the Institute of Biology of the Southern Seas (IBSS, Ukraine) designed a wiki-based tool for the creation and support of marine checklists (http://phyto.ibss.org.ua, http://zoo.bss.ibss.org.ua).

The checklists software was created on the basis of a wiki engine (http://en.wikipedia.org/wiki/Wiki). A wiki is a collection of web pages designed to enable anyone who accesses it to contribute or modify content, using a simplified mark-up language. Wikis are often used to create collaborative websites and to power community websites. Unlike most wiki projects the taxonomic checklists can only be edited by registered users (editors). Registration is made through an administrator and account information for access is sent via e-mail to the approved editors.

Generally speaking the checklist species page is the wiki page with text, tables, images, formulas, etc. that can be linked with other internal or external sources. But special features are developed that enable the checklist to be parsable by computers and thus to provide developers with the means by which it is possible to aggregate and make use of the content, and also it makes possible the use of simple online viewing of any related unstructured information - text, maps, pictures, tables, etc.

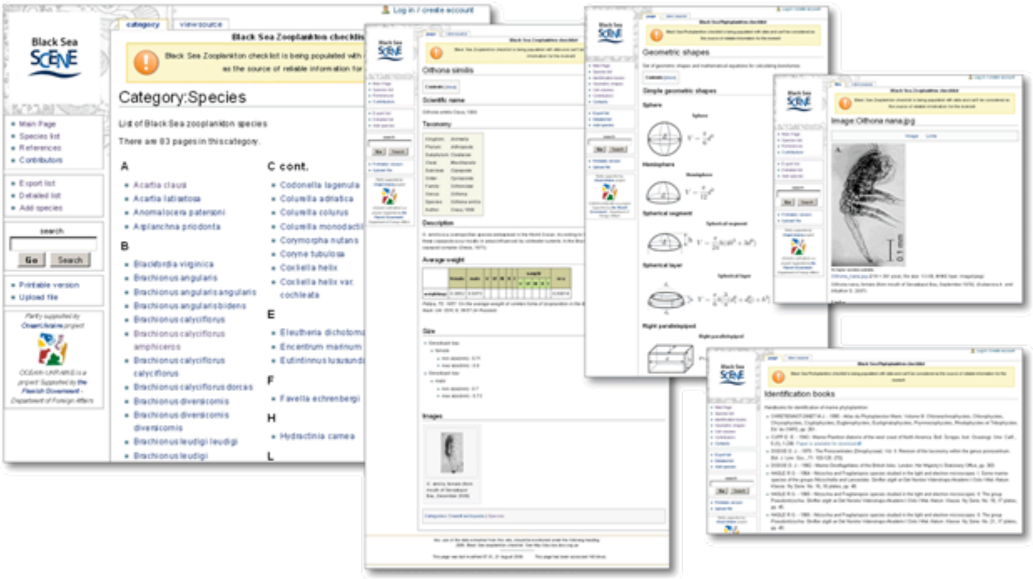

Checklists software system screen-shots

In order to store key species information in a structured form, suitable for parsing and use it in other data management systems, dynamic templates were developed. Templates are standard wiki pages whose content is designed to be embedded inside other pages.

Using templates forces the checklist editors to fill in the key species information in an online form with several parameters – value pairs (Figure below). Various templates can easily be added by the checklist administrator (10-20 minutes) if there is a demand for other structured information.

```
{{Taxonomy
| kingdom =
| phylum =
| subphylum =
| class =
| subclass =
| order =
| family =
| genus =
| species =
| subspecies =
| author =
| synonyms =
| spellvariations =
}}
```

```
{{Taxonomy
| kingdom = Animalia
| phylum = Arthropoda
| subphylum = Crustacea
| class = Maxillopoda
| subclass = Copepoda
| order = Cyclopoida
| family = Oithonidae
| genus = Oithona
| species = nana
| author =  Giesbrecht, 1893
| synonyms = ''Oithona
minuta'' Krichagin, 1873
}}
```

Kingdom:	Animalia
Phylum:	Arthropoda
Subphylum:	Crustacea
Class:	Maxillopoda
Subclass:	Copepoda
Order:	Cyclopoida
Family:	Oithonidae
Genus:	Oithona
Species:	*Oithona nana*
Author	Giesbrecht, 1893

Synonyms: *Oithona minuta* Krichagin, 1873

Taxonomy template (left), filled taxonomy template (middle) and appearance on species page for *Oithona nana* (right).

Applying 'parser functions' to template parameters make them powerful in presenting the structured information. Thus the appearance of rather simple templates can consequently be very sophisticated:

```
{{Average weight
|author = Petipa
|female = 0.0052
|male = 0.0073
|ova= 0.00014
}}
```

Average weight

	female	male	V	VI	III	II	I	nauplii					ova
								V	VI	III	II	I	
weight(mg)	0.0052	0.0073											0.00014

Petipa, TS. 1957. On the average weight of common forms of zooplankton in the Black Sea. Tr. Sevastopol Stn. Akad. Nauk. Ukr. SSR, 9, 39-57 (in Russian)

The average weight (zooplankton) template on a typical species page.

```
{{SpeciesLists
| list1 = x
| list2 = x
| list3 = x
| list9 = x
| list6 = x
| list4 = x
| list7 = x
| list8 = x
| list10 = x
| list11 = x
| list12 = x
| list13 = x
| list14 = x
| list18 = x
| list19 = x
| list20 = x
| list21 = x
| list22 = x
| list26 = x
| list27 = x
}}
```

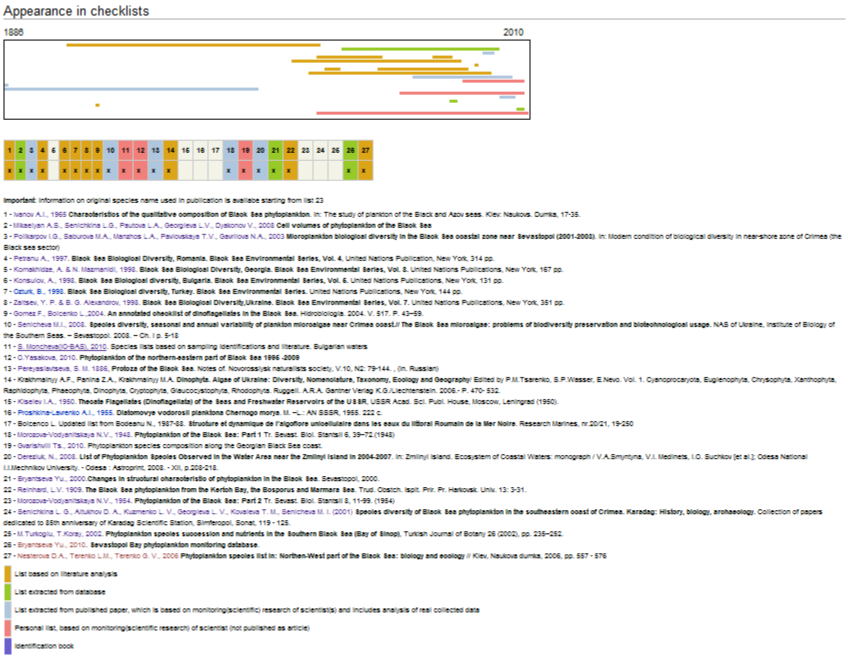

The species lists template and its representation on a page

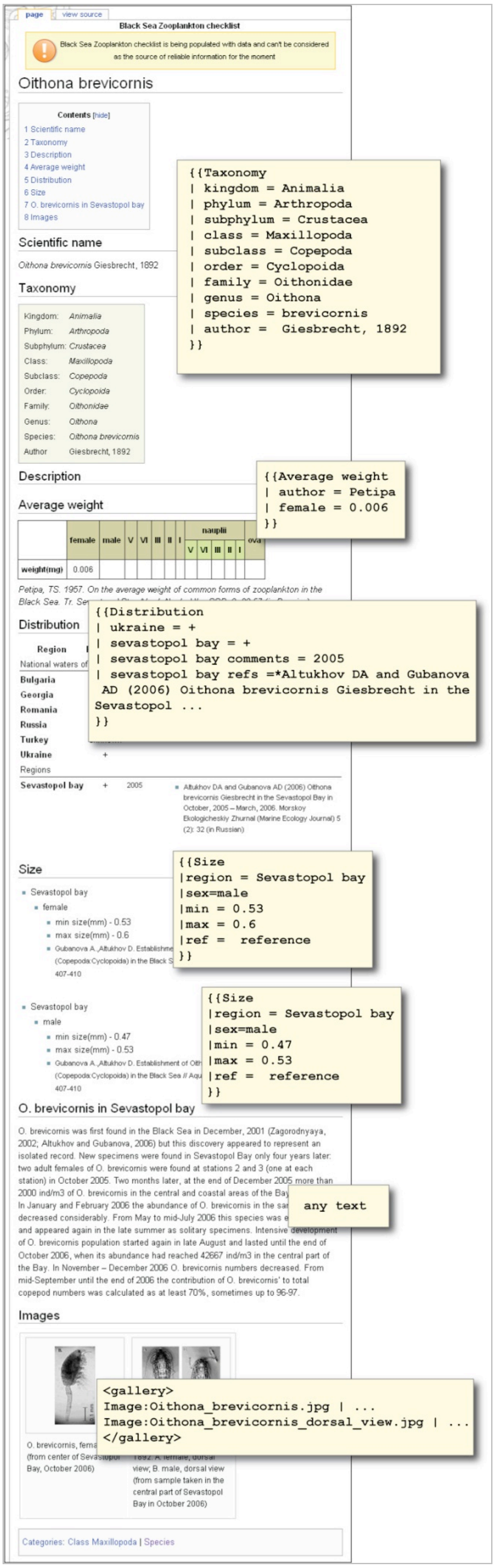

The species page and its source

The use of templates also makes the procedure of filling species information rather easy for species checklist editors.

When a new species is being added to the checklist, the system includes mandatory templates by default.

For example, for the Black Sea phytoplankton checklist the following templates were developed: taxonomy, appearance in checklists, cell volume, and formula; for the Black Sea zooplankton checklist this included: taxonomy, average weight, distribution, and size. In Figure (left) one of the species pages (zooplankton species *Oithona brevicornis*) and its source can be seen.

As mentioned above, all other necessary information such as text, images, etc. can be included in the page in order of the editor preference. The order of templates and free text sections is not strictly registered but it is recommended that editor follows a set species template to allow pages to be easily perceived by users.

The majority of species checklists existing on the web nowadays are developed as relational databases or simply a set of HTML pages. Both approaches have limitations on information which can be added to the species list and would require the full redevelopment of the checklist engine in some cases. The suggested wiki-based software tool doesn't set limitations on the type and amount of information that can be used to describe species in the species checklist.

Information on the ecology, morphology, biogeographically distribution, etc. can be and should be attached to each species. Concurrently the key information which is stored in the form of templates represents the backbone of the checklist and can be easily extracted from the pages.

In addition to the modification made to the wiki engine to fit the specific project objectives and to create dynamic templates, several additional features were developed. An export check list feature was created to enable users to export a full list of species, or part of it, in a form of a tab delimited text file for later use in publications or other data management systems and databases.

Developed software can be used to create regional (national) checklists of species with all information needed for the stakeholders and to provide the subset of information to global information system like WoRMS, etc.

A draft checklist of the Black Sea phytoplankton (http://phyto.ibss.org.ua) that is well developed and filled with big amount of data and information can be a good example of the possibilities of the developed system. All available Black Sea data bases, many publications, monographs, personal checklists, and manuals were used to compile this check-list. 1608 species are included in this check-list at the moment for 1886 – 2010.

5.3 Exchange tool between PESI and Focal Point websites (prepared by Roy Kleukers, NCB Naturalis, Leiden, NL; Ward Appeltans and IT team, VLIZ, Oostende, BE)

We had the special task to develop an exchange tool to facilitate the exchange of information between the focal point websites and the PESI website and between the focal points. As this can only be effective when the names are synchronized between the different systems, this project is thought to contribute to the standardization of the species lists throughout Europe. At the moment of submission of this handbook only a first step is taken, e.g. the linking of photos from the Dutch Species Register on the PESI-webportal. The technical implementation is as follows:
When displaying a PESI taxon page the PESI webserver contacts the NLSR webservice. It sends one input parameter (searchString) that corresponds to the name of the taxon. The webservice at NLSR responds with XML-structured data. This XML tells us whether there are images available for this particular taxon or not. If any references to images are present in the xml results, they contain absolute links to the image files at NLSR. When displaying the PESI taxon page these pictures are included in the HTML output by using the image URL's we retrieved from the webservice.

An expansion of this project will be developed with more partners and information types for exchange of information between selected Focal Point portals and the PESI portal. In the future (beyond PESI), it is also planned to adapt the tool to exchange information between the Focal Point portals as well.

The exchange possibilities from Focal Point to PESI and between Focal Points will concern mainly:
- Photos
- Status info
- Source info
- Vernacular names updates

The exchange possibilities from PESI to Focal Point include a.o. country maps

The conditions for the exchange tool include:
- Agreement on what to exchange and under which conditions (contract)

• Switchboard (technical elaboration)
• Technical adaptations at all portals involved.

The advantages comprise:
• Online sharing of biodiversity information
• Addition source information to country table in PESI
• Refining national status of species in PESI
• Dialog between focal points and taxonomic experts
• Contribution to standardisation of biodiversity data
• Support for start new FP-websites

5.4 E-science applications

PESI will establish links with publishers of scientific journals, e.g. display of RSS feeds linked to species, and will add deep links from species pages to publishers websites.
PESI will propose to publish data appendices from science journals in a standard electronic format that facilitate data integration and distribution towards GBIF and OBIS.

5.5 Multilingual portal pages

Supported by the translations from the Focal Points, each country will benefit from the PESI web portal in its own language and alphabet. As much text as possible will be included. A start has been made with the buttons, gradually extending to translations of all web portal services.

5.6 EDIT Expert Net

The EDIT Expert Net is a Taxonomic Experts and Networks database: http://editexpertnet.org/. This European information service especially aims to provide expertise and contact information of European taxonomic experts, networks and societies. The PESI web portal button "Expert db" gives a link to this service.

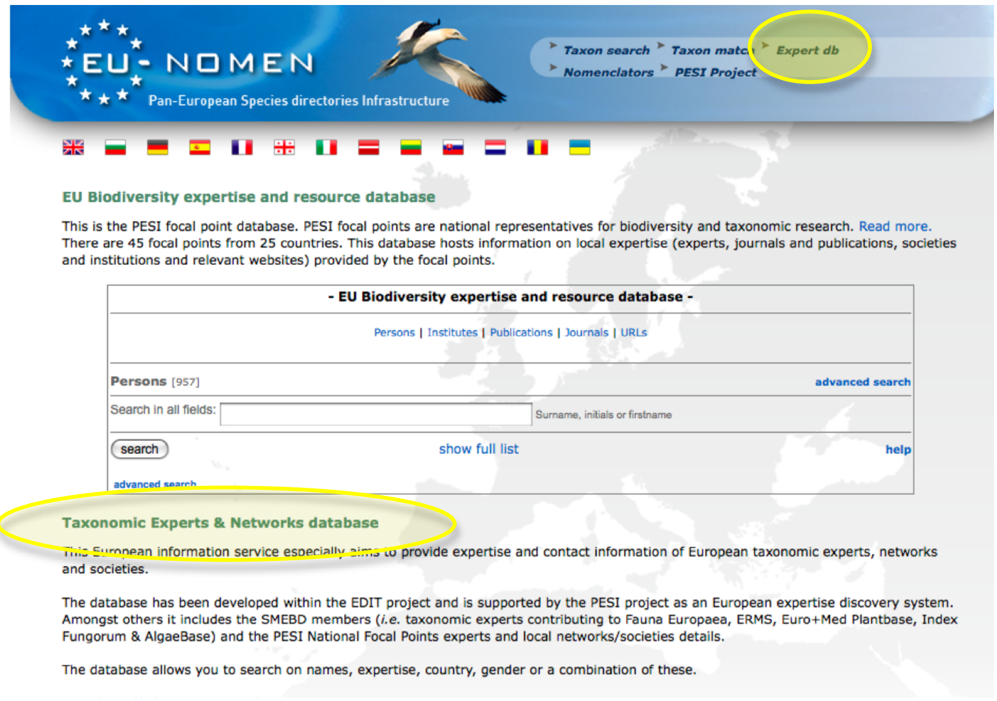

The database has been developed within the EDIT project and is supported by the PESI project as a "*European expertise discovery system*". Amongst others it includes the SMEBD members (*i.e.* taxonomic experts contributing to Fauna Europaea,

ERMS, Euro+Med Plantbase, Index Fungorum & AlgaeBase) and in the future the PESI National Focal Points experts and local networks/societies details, if agreed.

The database allows searching on names, expertise, country, gender or a combination of these.

5.7 Focal Points communication: tools and data services

5.7.1 The Focal Point mailing list

The list: fp@eu-nomen.eu includes all contracted (FaEu, ERMS, E+M) focal points. All the fp members of this list are allowed to post a message onto the list. The list: fpas@eu-nomen.eu includes all contracted (FaEu, ERMS, E+M) and associated focal points and all members of this list can post a message here.

5.7.2 The Focal Point discussion forum

All established and associate Focal Points are allowed to post discussion topics on the Focal Point forum at: http://www.eu-nomen.eu/pesi/.

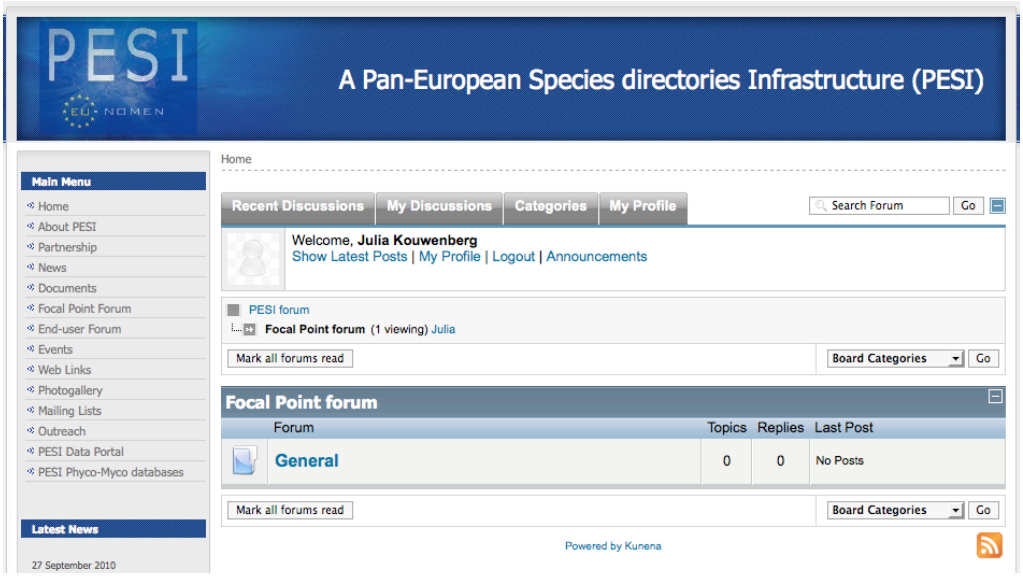

5.7.3 The Focal Point Handbook wiki pages

A Focal Point Handbook Wiki was set up for shared updating, communication, web-linking, etc. at: http://pesifocalpointhandbook.wikispaces.com/
If desired and appropriate, it will contain the evolving text of this handbook and much more useful information, which will be gathered by the contributing focal points and WP3/WP1 management staff.

6. Establishing a Focal Point

6.1 Organisation of Focal Points (Sweden, Lithuania, Poland)

Sweden

The PESI Focal Point in Sweden is fully integrated with the Swedish GBIF node (http://www.gbif.se - offering data on more than 25 million objects) located at the Swedish Museum of Natural History, Stockholm (SMNH - http://www.nrm.se). This museum is a major institute of taxonomic, systematic, ecological and environmental research in Sweden, gathering and harbouring a large part of the natural science collections and databases in the country. The collections comprise of almost ten million specimen, and the research staff at the museum collaborate with colleagues from all over the world in four fundamental research themes (The Diversity of Life; Ecosystems and Species History; Man and the Environment, and The Changing Earth).

The Swedish PESI Focal Point also collaborates closely with the Swedish Species Information Centre (http://www.artdata.slu.se/default.asp) which is a shared body of The Swedish University of Agricultural Sciences, Uppsala (http://www.slu.se) and the Swedish Environmental Protection Agency (http://www.naturvardsverket.se/sv) The major tasks of the former organization are to collect, evaluate and store the most important information about threatened and rare plant and animal species in Sweden, whereas the Swedish Environmental Protection Agency is the national agency for environmental protection and nature conservation as well as outdoor recreation and hunting issues. Its key tasks are to present proposals for environmental policy and legislation to the Swedish Government and ensure that environmental policy decisions are implemented.

A basic part in the work of the Swedish Species Information Centre is to assess degrees and types of threats and to prepare the national so called Red Lists and Red Data Books for which expert committees for each of the following groups of multicellular organisms are active: vascular plants, bryophytes, lichens, fungi, algae, vertebrates (excl. fishes), fishes and invertebrates. The committees consist of some of the leading specialists in Sweden. The Swedish Taxonomy Initiative (http://www.artdata.slu.se/svenskaartprojektet.asp) which forms part of the Swedish Species Information Centre in finding, treating and publishing in a series of identification handbooks with keys in Swedish and English to the Swedish plant and animal species – "The Encyclopedia of the Swedish Flora and Fauna" - http://www.nationalnyckeln.se/english/index.asp - taxonomic, systematic and ecological information, also constitute a collaborating part to the PESI Focal Point.

The amount of time allotted to work on the PESI project amounts to slightly less than 15% of one full time employee on a yearly basis, and so far the task have been fulfilled by assigning two staff members to the various tasks contained in the task description, consisting of altogether eight objectives: extracting information on national taxonomic resources and expertise, searching and cross-validating national checklists, supplying vernacular names and lists of prioritized taxa, translating international texts to Swedish, and planning for fund-raising.

Lithuania

The PESI Focal Point in Lithuania does not have permanent staff. It is maintained by volunteering researchers of the Nature Research Centre (http://www.gamtostyrimai.lt/en/) and Department of Zoology of Vilnius University (www.zool.gf.vu.lt) have been employed on part-time basis during the PESI project to fulfil particular tasks of it.

The number of research institutions of Lithuania with a scope covering taxonomy is very limited. The Nature Research Centre conducts scientific studies in the fields of botany, zoology and parasitology, including taxonomy of multiple groups of organisms. Vilnius University has long-lasting traditions of research in entomology, ornithology, ichthyology and parasitology. Collections and herbaria of Nature Research Centre include over 1 million specimens, with additional 150000 specimens stored at the Museum of Zoology of Vilnius University.
The Lithuanian PESI Focal Point maintains collaborative links with other scientific institutions conducting studies in the fields of biodiversity and taxonomy, such as Kaunas T.Ivanauskas Zoological museum (http://www.zoomuziejus.lt/index.php) and Vilnius Pedagogical University. Kaunas Zoological museum stocks over 220000 specimens of vertebrates and invertebrates. A team of entomologists in Vilnius Pedagogical University is carrying out research on biodiversity with particular focus on taxonomy of Microlepidoptera.
At the moment, there is no single national centre accumulating the data of all the species of Lithuanian Flora and Fauna. Such kind of data is accumulated by different scientists or scientific societies and published in scientific journals as *Acta Zoologica Lituanica*, *New and Rare for Lithuania Insect Species*, *Biologia*, *Botanica Lithuanica*, most of which are available online free of charge. National Red Data Book lists are compiled and supervised by a commission of experts at the Ministry of Environment of the Republic of Lithuania. An 'Information System of Protected Habitats, Animals and Plants' is under development by the Ministry of Environment. However, it is not yet functioning, and it is not clear when it will be available to the public. Online databases covering data on some taxa are available through the websites of societies like the Lithuanian Entomological Society (http://www.entomologai.lt) or Lithuanian Ornithological Society (http://www.birdlife.lt).
The tasks performed so far by the Lithuanian PESI Focal Point included extracting information on national taxonomic resources and expertise, supplying vernacular names and lists of prioritized taxa and providing other needed information from the sources in Lithuania. The future plans include promoting the tools developed by PESI project in Lithuanian scientific community, cross-validation the national checklists using the matching tool and, in the future, raising funds to establish a data portal with Lithuanian species register.

Poland

The PESI Focal Point in Poland for marine species is maintained by Department of Marine Ecology , Institute of Oceanology Polish Academy of Sciences (http://www.iopan.gda.pl). Marine Ecology Department is a major institute of taxonomic, ecological and environmental research, specifically in comparative field ecology in marine systems in Poland. The biodiversity and its relation to the ecosystem function and the climate change are in the main focus. Information on marine fauna and flora is gathered and harboured in open access databases (http://www.iopan.gda.pl/webpages.html). Also IOPAS is organising a central oceanographic data base that will include a biodiversity model for Polish Exclusive Economic Zone. Institute does not maintain species collections on a large scale but specimens collected in projects and newly described species are stored on site.
The Polish PESI Focal Point maintains collaborative links with other Polish scientific institutions conducting studies in the fields of biodiversity, taxonomy and ecology. This collaboration includes Sea Fisheries Institute in Gdynia (http://www.mir.gdynia.pl) which conducts research that includes fisheries biology, fisheries oceanography and marine ecology, fish processing technology and fishery economics; Faculty of Biology and Environmental Protection University of Łódź (http://www.biol.uni.lodz.pl/en/) which has long-lasting traditions in botany, zoology, hydrobiology and ecology of marine systems including Baltic Sea, and Institute of Oceanography University of Gdansk (http://www.ocean.ug.edu.pl/) which conducts research in Baltic Sea on all levels of taxonomic organisations, from phytoplankton to marine mammals and birds.

At the moment, there is no single national centre accumulating the data of all marine species of Polish Exclusive Economic Zone in the Baltic Sea. Such kind of data is accumulated by different scientists or scientific societies. The tasks performed so far by the Polish PESI Focal Point included extracting information on national taxonomic resources and expertise, supplying vernacular names and lists of prioritized taxa and providing other needed information from the sources in Poland.

6.2 National best practices and lessons learned models (Ukraine, Norway)

Ukraine

To develop the unified checklist service for the user community and to carry out the PESI tasks the Fauna Ukraine Focal Point with the State Museum of Natural History, National Academy of Sciences of Ukraine have made the first version of the interactive, bilingual (Ukrainian, English) web portal "Biodiversity Ukraine" <http://museum.lviv.net/pesi-ua/en/>. During the process of the web portal creation, the main biodiversity web portals were used (Italy: Fauna Italia http://www.faunaitalia.it, Portugal and Spain: Fauna Iberica http://www.fauna-iberica.mncn.csic.es, France: Inventaire National du Patrimoine Naturel http://inpn.mnhn.fr, Sweden: ArtPortalen http://www.artportalen.se, Netherlands: Nederlands soortenregister http://www.nederlandsesoorten.nl, Slovakia: Databank of Slovak fauna http://www.dfs.sk, etc.) and their best experience was taken into account.

"Biodiversity Ukraine" provides the main information trends, as follows: (1) biodiversity, (2) biota of Ukraine, (3) collections, (4) conservation, (5) literature, (6) photos, (7) links.

Biodiversity. Will include information on all aspects of biodiversity in Ukraine.

Biota of Ukraine. Will include the checklists of the Ukrainian flora and fauna, as full as possible, brought to conformity with EU taxonomic standards. Each species will be arranged on its own page with the main taxonomic, biological, ecological information and its distribution in Ukraine being mapped. Vernacular names of the species will be given as well, including the information on their importance in human culture, if available.

Collections. Will include the information on the collections of institutions, museums, reserves and private persons. While preparing this information the requirements of the existing global information system like GBIF will be taken into consideration.

Conservation. Will include the information about all Ukrainian laws and legislative documents on nature protection and others of a kind and their PDF copies. As well, here the information will be placed on all Ukrainian nature protected areas with the highest protection status (biosphere reserves, nature reserves, national nature parks).

Literature. Will include the list of monographs and papers on the Ukrainian flora and fauna with PDF copies.

Photos. For the user community each species page will be illustrated with the species photos.

Links. For better dissemination of the biodiversity information for user community all main useful links will be provided.

This web portal will be a useful tool that will help users in their education, research, and management in the environmental information. Standardization of the taxonomic information according to EU criteria will assure high quality of information.

Thus, free online tool will enable users to upload the national species lists and associated information and to receive back a file certifying names which are correct, stating their conservation (e.g. in EU Habitats and Birds Directives) or economic importance (e.g. invasive pests, parasites, commercial fish species).

Norway

The Norwegian Biodiversity Information Centre

Bridging gaps
The Norwegian Biodiversity Information Centre (NBIC) was established by the Norwegian Government in 2005, with the aim to:

- bridge the gaps between science and policy makers, other stakeholders and the public on biodiversity issues
- achieve more independence in relation to policy and political processes
- increase the relevance, credibility and legitimacy of biodiversity information
- establish an official access point for biodiversity information in Norway
- strengthen the work with threatened and vulnerable species, ecosystems and genes (populations)

Information from the Norwegian Biodiversity Information Centre can be accessed at www.biodiversity.no

The Mission
The NBIC's major role is to provide society with updated and easily accessible information on biodiversity. To achieve this, NBIC collates information from research institutions and other sources and makes this information accessible to society.
NBIC is the official national source for information on biodiversity in Norway.
The information conveyed by NBIC is of high quality and up-to-date. The organisation places emphasis on maintaining a neutral and independent approach, and on being an institution with the highest level of integrity.
Results thorugh Cooperation
Primary knowledge on biodiversity is acquired by natural history museums, research institutes, nature management institutions and NGO's. The Norwegian Biodiversity Information Centre cooperates closely with these organizations to collate information so that it becomes available to everyone. NBIC also seeks advice from the end users so that the presentation of information matches the user's requirements.
Increasing Public Awareness
The Norwegian Biodiversity Information Centre works to raise awareness of biodiversity issues in society. The objective is to provide the public with up-to-date, objective information, and to make the issue of biodiversity an important factor in decision-making processes. Putting biodiversity on the agenda implies that we place emphasis on active and current communication with governmental institutions, media and society as a whole.
Focus on International Cooperation
International cooperation is high on the NBIC agenda. This started with the development of a close collaboration with our sister organisation in Sweden, the Swedish Species Information Centre.
The Norwegian Biodiversity Information Centre also contributes to the work of international organizations operating regionally and globally to increase our knowledge of biodiversity and to improve the dissemination and exchange of such knowledge across national borders. At present NBIC is involved in the work of the following international institutions, initiatives and projects:
• Global Biodiversity Information Facility (GBIF)
• European Commission – The Life Watch Project
• European Commission – INSPIRE Directive
• Encyclopaedia of Life (EOL)
• European Environment Agency (EEA)
• International Union for Conservation of Nature (IUCN)
Information Services from NBIC

Information from the Norwegian Biodiversity Information Centre is available through our databases and map services on the Internet (www.biodiversity.no). Most of the services are only available in Norwegian at present. However, NBIC is currently working to increase the amount of information available in English.

The Species Red List Database

contains documentation on the 2010 Norwegian Red List with 4599 species.

The Alien Species Database

contains documentation on the 2007 Norwegian Black List. New lists of more than 2500 alien species will be available in 2012.

Species Map Service

Link updated species databases to web-based map interfaces, to produce maps with species distributions. Contains over 11 mill. objects from 30 source institutions/organisations and nearly 20 000 species.

Species Observations Service

Register your own species observations, upload pictures, and see yours and other's observations on a map. Over 5 mill. sightings is reported by 2010.

Species Names Database

Search the taxonomic database containing scientific and Norwegian names for species in Norway, with synonyms. Contains at present 57948 species, of this 35439 species found in Norway, with 82536 scientific and 22655 vernacular names.

Habitat Type Database

Browse the hierarchical system for typification of Norway's diverse habitat/ecosystem types.

The Ecosystem Red List Database

Contains Red Listed ecosystems in Norway, will be launched in May 2011

Publications from NBIC

Publications from the Norwegian Biodiversity Information Centre are free to download from our website (www.biodiversity.no).

The 2010 Norwegian Red List for Species.

The Norwegian Red List contains risk assessments for species extinction in Norway. Altogether 21000 species have been evaluated, resulting in 4599 red list species.

The 2007 Norwegian Black List

The Black List is the first official overview of ecological risk assessments for a selection of alien species in Norway.

The 2011 Norwegian Red List for Ecosystems

The Norwegian Red List for Ecosystems contains risk assessments for habitat/ecosystem degradation and disappearance in Norway.

Pages - Red list species

A collection of 150 species pages on selected red list species from the 2010 Norwegian Red List, mainly in the IUCN categories Critical endangered (CR) and Endangered (EN).

Pages - Black listed alien species

A collection of 35 pages on selected black list alien species from the 2007 Norwegian Black List, mainly in the category "High Risk".

The Norwegian Taxonomy Initiative

There are assumed to be around 55 000 species in Norway. So far, rather more than 40 000 of these are known. However, basic knowledge about very many of the species is poor and there is a great need to improve scientific expertise associated with our knowledge of species (biosystematics). The Norwegian Taxonomy Initiative has been set up to try to fill these gaps in our knowledge and to help to improve expertise and recruitment in the field of biosystematics.

The Norwegian Biodiversity Information Centre is located at the Norwegian University of Science and Technology in Trondheim, near the Museum of Natural History and Archaeology.
Visiting address:
Elvegata 17, Trondheim

Postal address:
Erling Skakkes gt. 47,
NO-7491 Trondheim, Norway
E-mail: postmottak@artsdatabanken.no
Phone: +47 73 59 21 45 Telefax: +47 73 59 22 40

7. Further information

7.1 Legal issues and copyrights of Focal Point data in PESI

Submitted focal point data will have the same legal status as other data in PESI, copyrighted under the SMEBD IPR model (see D2.2): http://www.eu-nomen.eu/pesi/index.php?option=com_remository&Itemid=56&func=startdown&id=597. Cited from this document:

a. Data ownership statement

The content of the PESI databases is vested in the Society for the Management of Electronic Biodiversity Data Ltd (www.smebd.eu). All scientists contributing to the databases are eligible for SMEBD membership, and thus share collective responsibility for ensuring the data are quality controlled, maintained, and hosted by appropriate institutions. Upon completion of the PESI project, SMEBD will continue to develop the databases in collaboration with their host organisations. Decisions on the management of the databases, such as: a) appointing and replacing experts to edit contents, and b) providing copies to third parties, are made by specific database committees under the authority of the SMEBD Council.

For new databases, contributors interested in becoming involved and providing information to PESI will have the option of allowing SMEBD to take over the management of the selection of data they provide and signing a SMEBD agreement form.

The EU-Nomen portal will have a common approach to citation and Creative Commons licensing for all databases, including databases outside the SMBED committee. The copyright used will follow the *Attribution-ShareAlike* scheme (for more information, view http://creativecommons.org/licenses/by-sa/3.0/). Ideally, all data providers should abide by the same licence requirements to avoid conflicting policy interactions if combined datasets from different sources are downloaded through the PESI facilities.

a. Terms of use of data

By downloading or consulting data from this website, the visitor acknowledges that he/she agrees with the PESI data policy, and agrees to the following:

- If data is extracted from the PESI website for secondary analysis resulting in a publication, the PESI website should be cited following the PESI citation scheme:

The citation system proposed for PESI generally follows that used in citing works published in hard-copy and so should be straightforward for researchers to use.

- [Author/s][Year]. [Chapter] *In* [Book]. [Publisher]. [Page]

The template to be used for PESI will be as follows when verified by expert/contributed by European Species Database (ESD) or other database outside SMEBD:

- Editors (Year). Taxon + Authority. In: ESD title or other Database or data manager. Accessed through the PESI at [URL] on [date].

For example:

- Reid, David G., Gofas, S. (2010). *Littorina littorea* (Linnaeus, 1758). *In*: Bouchet, P.; Gofas, S.; Rosenberg, G. (2010) World Marine Mollusca database. Accessed through the World Register of Marine Species at http://www. eu-nomen.eu/pesi/aphia.php?p=taxdetails&id=140262 on 2010-07-28.

Where the record has not been verified the database is the author, in which case the following template is used.

- PESI (Year). Taxon + Authority. Accessed through PESI at [URL] on [date].

For example:

- PESI (2010). *Salmo salar* Linnaeus, 1758. Accessed through PESI at http:// eu-nomen.eu/pesi/aphia.php?p=taxdetails&id=127186 on 2010-07-28.

- Users must acknowledge the contribution of the relevant Data Providers in any derived information product or publication, whether printed, electronic or broadcast, that is based wholly or in part on the material, data and/or information they make available to them. Where users make specific use of or reference to a particular biological dataset they must acknowledge the original data provider (where their name is made available) using the referencing format provided within the PESI database.

b. Termination of licence and rights of use

This License and the rights granted hereunder will terminate automatically upon any breach by the user of the terms of this License. Individuals or entities who have received Adaptations or Collections from the user under this License, however, will not have their licenses terminated provided such individuals or entities remain in full compliance with those licenses.

Subject to the above terms and conditions, the license granted here is perpetual (for the duration of the applicable copyright in the Work). Notwithstanding the above, PESI reserves the right to release the Work under different license terms or to stop distributing the Work at any time; provided, however that any such election will not serve to withdraw this License (or any other license that has been, or is required to be, granted under the terms of this License), and this License will continue in full force and effect unless terminated as stated above.

c. Disclaimer

General disclaimer

The PESI Data Administrators reserve their exclusive right in its sole discretion to alter, limit or discontinue the Site or any Materials in any respect. The PESI Data Administrators shall have no obligation to take the needs of any User into consideration in connection therewith. The PESI Data Administrators reserve the right to deny in their sole discretion any user access to this Site or any portion thereof without notice. No waiver by the Data Administrators of any provision of these Terms and Conditions shall be binding except as set forth in writing and signed by its duly authorized representative.

No Warranty

PESI and its data suppliers provide this database free of charge for the benefit of the public in an "as is" condition. Neither PESI nor its data suppliers warrants, guarantees, or makes any representation regarding the accuracy, completeness, correctness, reliability, currency or otherwise, of the databases or the use or results to be obtained from using the databases or the information contained therein, or any related documentation or written materials. Neither PESI nor its data suppliers make any representations or warranties whatsoever, express or implied, with respect to the database, and, in particular, PESI and its data suppliers disclaim all implied warranties including without limitation any warranties of merchantability, non-interference, non-infringement, informational content, or fitness for a particular purpose with regard to the database.

Limitations on liability

Except to the extent required by applicable law, in no event will PESI or its data suppliers be liable to you on any legal theory for any special, incidental, consequential, punitive or exemplary damages arising out of this license or the use of the work, even if licensor has been advised of the possibility of such damages.

7.2 Continuity plan (LifeWatch, GBIF, LIFE+ ambitions/organisation thematic topics/regional level)

Part of PESI business plan

7.3 Preparation of joint proposals

Part of PESI business plan

7.4 Focal Points influence in policy planning

7.4.1 Policy plans - for national and international conservation purposes

Every policy plan follows a number of basic steps, namely:

1.) Identify a problem and whether it can be solved.
2.) Devise a policy that addresses the problem.
3.) Adopt the policy while agreeing on a strategic approach.
4.) Implement the policy by putting a measure in place.
5.) Determine the impact of the policy and whether it solves the problem or creates a new problem.
6.) Revise the policy and make the necessary changes.

Communicating specialist knowledge on complex issues, such as biodiversity, to policy makers poses diverse challenges. It is important to devise ways to illustrate the importance of high quality taxonomic information, a fundamental resource, and its implications when integrated into policy-making.

7.4.2 The potential of PESI and the FP Network as a tool in policy making

The initial step in the policy process or indeed the last step in the process is where FPs can become involved. The science-policy interface is where FPs can help to communicate to their policy makers the present uncertainty related to the current country species lists and the implications for biodiversity plans. Better communication of uncertainty can help decision-makers integrate science into environmental policy[1]. Also, the possible implications of uncertainty provide policy makers with useful perspectives. FPs can also illustrate how they can be part of the solution to the problem.

Threatened species lists are designed primarily to provide an easily understood qualitative estimate of the risk of extinction of a species. Such lists are being incorporated by the taxon registers within PESI. Although these estimates of risk can be accurate, the lists have inevitably become linked to several policy-making processes. There are four ways in which such lists are commonly used:

- To set priorities for resource allocation for species recovery;
- To inform and help design networks of protected areas;
- To constrain development and exploitation;
- To report on the state of the environment.

The validated lists provided by PESI from the FPs could be designed accordingly to any of these purposes, and consequently perform at the highest integration and usage level. These lists will form the basis for future monitoring and potentially may highlight changes as rare or sensitive species become locally extinct or their distributions change.
The species identified, within PESI by FPs, as protected and rare highlight species can be found in ecological communities that have particular threats, are witnessing declining population trends, or restricted distributions have indicated that they require special attention. Some of these will be identified as included within the Habitat Directive annexes,

---

[1] Wardekker, J.A., van der Sluijs, J.P., Janssen, P.H.M. *et al*. (2008). Uncertainty communication in environmental assessments: views from the Dutch science-policy interface. *Environmental Science & Policy*. 11:627-641.

others will be country specific. These lists serve as a practical method to assist in making conservation and land-use decisions and in prioritising research, inventory, management, and protection activities.

8. Feedback and suggestions

Please consult the Focal Point Handbook Wiki pages!

9. Contact Information

9.1 PESI management contacts

Yde de Jong, PESI Project coordinator and coordinator FaEu validated checklists: yjong@uva.nl
Julia Kouwenberg, PESI Project manager, WP3 manager: kouwenberg@uva.nl
Nihat Aktaç, WP3 leader: nihata@trakya.edu.tr
Ward Appeltans, WP6 leader and coordinator national marine validated checklists: ward.appeltans@vliz.be
Eckhard von Raab-Straube, coordinator E+M validated checklists: E.Raab-Straube@BGBM.ORG

9.2 Focal Point data portals and useful websites per country

- **Albania**: Biodiversity in Albania <http://enrin.grida.no/biodiv/biodiv/national/albania/home.htm>; Fauna of Albania <http://www.unishk.edu.al/.../Register_of_Species_of_the_Fauna_of_Albania_2010.pdf>
- **Armenia**: Division of Natural Sciences <http://www.sci.am/resorgs.php?oid=24&langid=1>
- **Austria**: Zoologisch - Botanische Datenbank Österreich <http://www.zobodat.at/D/runD/D/cacheD/>; GBIF Austria <http://data.gbif.org/countries/AT>
- **Azores**: Azorean Biodiversity Portal <http://www.azoresbioportal.angra.uac.pt/index.php?lang=en>; Spiders of the Azores <http://www.jorgenlissner.dk/azoreanspiders.aspx>; Azorean Biodiversity Group <http://cita.angra.uac.pt/biodiversidade/>
- **Belgium**: Belgian Species List <http://www.species.be/en/index.php>; Belgian Biodiversity Platform <http://www.biodiversity.be/>; Lepidoptera of Belgium <http://webh01.ua.ac.be/vve/Checklists/Lepidoptera/Introduction.htm>
- **Bulgaria:** National Museum of Natural History, Sofia <http://www.nmnhs.com/>; BiodiversityBG <http://www.biodiversity.bg/index.htm>; Bulgarian fungi <http://www.fungi.biodiversity.bg/gallery-a.htm>; Prime Butterfly areas in Bulgaria <http://www.nmnhs.com/butterfly_areas_bg/>; Checklist of Bulgarian spiders <http://www.cl.bas.bg/bulgarianspiders/feedback.htm>; The Dragonflies of Bulgaria <http://www.odonata.biodiversity.bg/species_en.htm>; Bulgarian orchids <http://orhidei.biodiversity.bg/list.htm>;
- **Denmark**: Denmarks Naturdata <http://www.naturdata.dk/><http://www.fugleognatur.dk/wildaboutdenmark/>Checklist of phytoplankton in the Skagerak-Kattegat; DanBIF <http://www.dk.gbif.net/> <http://www.smhi.se/oceanografi/oce_info_data/plankton_checklist/ssshome.htm>

- **Estonia**: Estonian Species Register <http://unite.ut.ee/temp/natmus_in.php>; Estonian e-Biodiversity <http://elurikkus.ut.ee/index.php?lang=eng>; The Nature Observations Database <http://eelis.ic.envir.ee/lva/LVA.aspx?lang=eng>
- **Finland:** Atlas Flora Europaea <http://www.luomus.fi/english/botany/afe/index.htm>; GBIF Finland <http://data.gbif.org/countries/FI>
- **France**: Inventaire National du Patrimoine Naturel (Muséum National d'Histoire Naturelle) <http://inpn.mnhn.fr>; Tela Botanica <http://www.tela-botanica.org/site:accueil>; GBIF France <http://www.gbif.fr/>
- **Georgia:** Georgian Biodiversity Database <http://www.biodiversity-georgia.net/>
- **Germany**: <http://www.bgbm.org/>; Floraweb <http://www.floraweb.de/>; GBIF Germany <http://www.gbif.de/homeenglish>
- **Greece**: <http://greek-biodiversity.bio.auth.gr>; Helbionet <http://www.helbionet.org/>
- **Hungary**: Hungarian Natural History Museum <http://www.nhmus.hu/index.php?newlang=english>
- **Ireland**: The Irish Species Register <http://www.species.ie/>; National Biodiversity Data Center <http://www.biodiversityireland.ie/>; Invasive Species Ireland <http://invasivespeciesireland.com/>; Ground beetles of Ireland <http://www.habitas.org.uk/groundbeetles/index.html>
- **Israel**: Israel Biodiversity Information System <http://habitat.bot.huji.ac.il/biogis/static/en/index.html>
- **Italy**: Fauna Italia <http://www.faunaitalia.it>; Acta Plantarum <http://www.actaplantarum.org/>
- **Latvia**: Biodiversity in Latvia <http://enrin.grida.no/biodiv/biodiv/national/latvia/>
- **Lithuania**: Nature Research Centre <http://www.gamtostyrimai.lt/en/>; Lithuanian Ornithological Society <http://www.birdlife.lt/>; Lithuanian Entomological Society <http://www.entomologai.lt/>; Catalogue of Lithuanian molluscs; <http://www.macrogamta.lt/e107_plugins/content/content.php?cat.124>; Catalogue of Lithuanian spiders <http://vorai.mke.lt/>
- **Luxembourg**: Fondation Faune-Flore <http://mnhnl.lu/fff/>
- **Macedonia**: Macedonian Museum of Natural History <http://prirodonaucen.com.mk/english/vraboteni/vladimirkrpacen.html>
- **Malta**: Environment & Planning Authority <http://www.mepa.org.mt/home?l=1>
- **Montenegro**: Ministry of Sustainable Development and Tourism <http://www.mse.gov.me/en/ministry>; Montenegrin Academy of Sciences and arts <http://www.interacademies.net/Academies/ByRegion/EuropeCentralAsia/Montenegro/13024.aspx>
- **Netherlands**: Nederlands soortenregister <http://www.nederlandsesoorten.nl>; Waarneming.nl <http://waarneming.nl/index.php>; Telmee <http://www.telmee.nl/>; NLBif Biodiversity Data Portal <http://www.nlbif.nl/>
- **Norway**: Artdatabanken <http://www.biodiversity.no>; GBIF Norway <http://gbif.no/>; Norwegian Seaweeds <http://seaweeds.uib.no/?about=specieslist>
- **Poland**: Museum and Institute of Zoology: <http://www.miiz.waw.pl/index.php?lang=english>; Institute of Oceanology: <http://www.iopan.gda.pl/><http://www.iopan.gda.pl/webpages.html>; GBIF Poland <http://www.ksib.pl/?l=en>
- **Portugal**: Naturdata <http://naturdata.com/>; Azores Biodiversity Portal <http://www.azoresbioportal.angra.uac.pt/pesquisa.php?sstr=5&lang=en>; Madeira biodiversity <http://www.netbiome.org/index.php?option=com_content&view=article&id=58%3Amadeira&catid=51%3Amonographies&Itemid=30&limitstart=3>
- **Romania**: myNature <http://mybiosis.info/nature/portal.php?pagename=firstpage>

- **Russia:** Zoological Institute RAS <http://www.zin.ru/index_e.htm>
- **Serbia**: Centre for the Balkan Biodiversity Conservation <http://www.dbe.uns.ac.rs/CBBC/en/index.htm>; National Park Fruska Gora <http://www.npfruskagora.co.rs/eng/natural-values.html>; Nature Conservation Serbia <http://www.natureprotection.org.rs/>
- **Slovakia**: Databank of Slovak fauna, but also to the botanical datasets <http://zoology.fns.uniba.sk/dataportal>; <http://ibot.sav.sk/page/>; <http://www.dfs.sk>; Diptera of CZ and SK <http://zoology.fns.uniba.sk/diptera2009/>; GBIF Slovakia <http://data.gbif.org/countries/SK>
- **Slovenia**: Biodiversity of Slovenia <http://biodiversityslovenia.net/florafauna.htm>; GBIF Slovenia <http://data.gbif.org/countries/SI>
- **Spain**: Fauna Iberica <http://www.fauna-iberica.mncn.csic.es>; Flora Iberica <http://www.floraiberica.org/>; Biodiversity Spain Inventories <http://www.mma.es/portal/secciones/biodiversidad/inventarios/>; Anthos <http://www.anthos.es/dispatch.en.html>; GBIF Spain <http://www.gbif.es/>
- **Sweden**: ArtPortalen <http://www.artportalen.se>; ArtDatabanken <http://www.artdata.slu.se/english/>; GBIF Sweden <http://www.gbif.se/>; Lepidoptera from Sweden <http://www2.nrm.se/en/svenska_fjarilar/svenska_fjarilar.html - Micropterigidae>
- **Switzerland**: CSCF & KARCH & CCO & KOF<http://www.cscf.ch/>; Swiss Systematics Society: http://www.swiss-systematics.ch/e/PESI/>; <http://lepus.unine.ch/carto/>; <http://www.biodiversitymonitoring.ch/english/aktuell/portal.php> GBIF Switzerland <http://www2.unine.ch/gbif>
- **Turkey**: BIOCES <http://www.bioces.tubitak.gov.tr>; EDTU Herbarium <http://edtuherbarium.trakya.edu.tr/en/>
- **Ukraine:** Biodiversity Ukraine <http://museum.lviv.net/pesi-ua/en/index.php>; Red book of Ukraine <http://mail.menr.gov.ua/publ/redbook/redbook.php?lang=ukr>; Black Sea Phytoplankton checklist <http://phyto.bss.ibss.org.ua/wiki/Category:Checked>
- **United Kingdom**: NHM UK Biodiversity Portal <http://www.nhm.ac.uk/nature-online/british-natural-history/uk-biodiversity-portal/index.html>; NBN Gateway<<http://www.searchnbn.net/> <http://www.biodiversityislife.net/>; National Biodiversity Network <http://www.nbn.org.uk/>; Biological Records Center <http://www.brc.ac.uk/>; UK Biodiversity Action Plan <http://www.ukbap.org.uk/>; GB Non-native species secretariat <https://secure.fera.defra.gov.uk/nonnativespecies/home/index.cfm>; Natural England <http://www.naturalengland.org.uk/>; Scottish Natural Heritage's Information Service <http://www.snh.org.uk/snhi/>; Countryside Council for Wales <http://www.ccw.gov.uk/?lang=en>; Marine Life Information Network <http://www.marlin.ac.uk/>; Bees, wasps and Ants recording society <http://www.bwars.com/index.htm>; Hoverfly recording scheme <http://www.hoverfly.org.uk/portal.php>; UK Butterflies <http://www.ukbutterflies.co.uk/index.php>; UK moths <http://www.ukmoths.org.uk/>

Configuration History			
**Version No.**	**Date**	**Changes made**	**Author**
0.9	04 November 2010	First draft for circulation	JK
1.0	26 February 2011	Second draft for circulation	JK
1.1	23 March 2011	Third draft for circulation	JK
1.2	23 March 2011	Final preparation for submission	YdJ

APPENDIX I

Summary of Recommendations for Controlled Vocabularies Use in the PESI Portal

PESI is primarily annotated checklist i.e. a list of accepted taxa, their preferred names, synonymous names, conservation status and areas in which they occur. These are some useful controlled vocabularies that you can either map your data to or use as standards within your data.

Data in the PESI portal is decorated with other annotations, such as photos and literature citations, which require other controlled vocabularies. Liaise with the PESI Portal team to be sure you use the appropriate vocabularies in these areas.

Occurrence Status

The PESI source databases have very different occurrence vocabularies and an attempt was made to produce a formal mechanism to merge these vocabularies. The results of this exercise are published as Hyam, R. (2010) *The use of Web Ontology Language (OWL) to Combine Extant Controlled Vocabularies in Biodiversity Informatics Appears Redundant.* Available from Nature Precedings2 The conclusion of the application of this work in the portal was to adopt eight possible statuses.

**Occurrence Status**	**Definition**
Present	There is sufficient evidence to assert that the organism does occur in the region.
Absent	There is no (sufficient) evidence to assert that the organism does occur in the region.
Native	The organism either evolved in this region or arrived by non-anthropogenic means.
Introduced	The organism arrived in the region via an anthropogenic mechanism or mechanisms.
Naturalised	The organism reproduces naturally and forms part of the local ecology.
Invasive	The organism is having a deleterious impact on another organism, multiple organisms or the ecosystem as a whole.
Managed	The organism maintains its presence through intentional cultivation or husbandry.
Doubtful	Available evidence for the occurrence of an organism in the region is unverified or suspicious.

The terms should be thought of as tags that occurrence records are labelled with. Each record may have one or more, possibly contradictory tags. Each contributing database should use these terms internally or should map their existing terms to these agreed ones manually at export.

Taxon Status

Differentiation between the statuses that apply to names and those that apply to taxa can be confusing. Technically a 'synonym' is a designation for a **name** rather than a taxon. At some point there must have existed a classification in which this name was the name for an accepted taxon but under the currently recognised classification the type specimen is considered to belong in a different taxon for which another name has priority. By convention resources tagged with the now synonymous name (i.e. identified to the taxon it was the accepted name for) are treated as if they belong to the taxon accepted in this classification. This is an assumption that may not be appropriate in all

2 http://dx.doi.org/10.1038/npre.2010.5168.2

situations.

Taxon Status	Definition
Accepted	PESI recognises the existence of a taxon with the associated name and other information.
Synonym	PESI does not recognise a taxon of this name and refers information tagged with name to an accepted taxon.
Unaccepted	PESI does not recognise a taxon of this name and is unable to refer the information tagged with this name to any accepted taxon.

Nomenclatural Status

Non-taxonomist users of PESI are unlikely to have an interest in nomenclatural statuses as they will be primarily using the service to find the recommended names for accepted taxa. They don't need to know how the decisions were taken to arrive at the correct name string for a taxon. Expert taxonomists, for whom the nomenclatural details are important, are likely to be interacting directly with the source databases. Inclusion of nomenclatural statuses in PESI is therefore somewhat redundant but worthwhile for the sake of completeness.

Nomenclatural statuses are currently only given for 7,116 of the 567,047 names (i.e. 1.25%) and some of the statuses are only used for a handful of names. In the majority of cases names which bear a nomenclatural status are not the accepted names of taxa.

The table below includes the current number of names bearing this status. It also links to *Terms Used in Bionomenclature*. Compiled by David L. Hawksworth3 where appropriate.

Nomenclatural Status	#	Definition
alternate representation	601	An alternative spelling of the name
comb. ined.	43	A “com. nov.” prior to its formal publication.
nom. & orth. cons.	1	See “nom. cons.”
nom. altern.	40	An alternative name. http://bionomenclature-glossary.gbif.org/term/nomen+alternativum/1
nom. ambig.	2	A name used in different senses which has thus become a source of error and confusion; a name applied by different authors to different taxa. http://bionomenclature-glossary.gbif.org/term/nom.+ambig./1
nom. confus.	15	a name based on a type consisting of discordant elements from which it was considered impossible to select a satisfactory lectotype; see also parahomonym. http://bionomenclature-glossary.gbif.org/term/nomen+confusum/1
nom. cons.	126	A name otherwise unavailable or invalid that the Commission, by the use of its plenary power, has enabled to be used as a valid name by removal of the known obstacles to such use. See http://bionomenclature-glossary.gbif.org/term/nom.+cons./1
nom. cons. prop.	10	A “nom. cons.” prior to its formal publication.
nom. dub.	296	a name of unknown or doubtful application; e.g. because of a lack of original or type material and sufficient information about it to

3 http://bionomenclature-glossary.gbif.org/

		make a satisfactory typification possible, or because it is impossible to ascertain to which taxon its type should be refered. http://bionomenclature-glossary.gbif.org/term/nom.+dub./1
nom. illeg.	3025	a validly published name that is not in accordance with one or more rules in the Code (principally those on superfluity and homonymy); such names must be rejected unless conserved; illegitimate names are validly published but are not to be taken into consideration for the purposes of priority when the correct name of a taxon is being decided. http://bionomenclature-glossary.gbif.org/term/illegitimate+name/1
nom. inval.	2133	In Zoology: of an available name, one which is either (a) objectively invalid (i.e. either is a junior homonym or a junior objective synonym of a potentially valid name, or must be rejected under the provisions of the Code, or has been suppressed by the Commission), or (b) is subjectively invalid (because it is considered subjectively to be a junior synonym or to be inapplicable to a particular taxon).<br>In Botany: used colloquially of names that are not validly published according the Code, and hence, strictly speaking, not names. http://bionomenclature-glossary.gbif.org/?term=invalid
nom. nov.	248	In Zoology: a replacement name; see also avowed substitute, new clade name, new replacement name, new scientific name. http://bionomenclature-glossary.gbif.org/term/new+name/2<br>In Botany: a newly published name; this may be the name of a new taxon, a new combination, a name at a new rank (i.e. status novus), or an avowed substitute (nomen novum) for an existing name; most commonly used in this last sense. http://bionomenclature-glossary.gbif.org/term/new+name/1
nom. nov. ined.	1	A “nom. nov.” prior to its formal publication.
nom. nud.	148	In Zoology: a name published before 1931, which fails to conform to the Code, or if published before 1930 fails to conform to other provisions of the Code; such a naked name is not an available name, and therefore the same name may be made available later for the same or a different concept; in such a case it would take the authors name and date from that act of establishment, not from any publication where it was a nomen nudum. http://bionomenclature-glossary.gbif.org/term/naked+name/1<br>In Botany: a name of a new taxon published without a description or diagnosis or reference to a description or diagnosis, and so not validly published; a bare name (prok.); see also nomen subnudum. http://bionomenclature-glossary.gbif.org/term/naked+name/2
nom. provis.	30	a provisional name http://bionomenclature-glossary.gbif.org/term/nomen+provisorium/1
nom. rej.	64	a rejected name http://bionomenclature-glossary.gbif.org/term/nomen+rejiciendum/1
nom. rej. prop.	5	a name proposed for rejection under the Code but awaiting ratification either by a Committee or by a subsequent International Botanical Congress; see conservation, rejected name.

		uri: http://bionomenclature-glossary.gbif.org/term/nomen+rejiciendum+propositum/1
nom. superfl.	308	a superfluous name http://bionomenclature-glossary.gbif.org/term/nomen+superfluum/1
species inquirenda	13	species of doubtful identity, and needing further investigation; in monographic works, such names are often treated in a separate section. http://bionomenclature-glossary.gbif.org/term/species+inquirenda/1
temporary name	7	A temporary name that will be replaced.

Geographic Regions

VLIZ have produced a list of geographic regions and associated polygon definitions based on the TDWG "World Geographical Scheme for Recording Plant Distributions"4 extended to cover seas of the world. These regions are list below but also available as an OGC Web Feature Service – for details contact VLIZ.

Geographic regions should be seen as a reporting mechanism not a data gathering mechanism. Whenever possible data should be gathered and stored at the finest level of detail possible e.g. GPS coordinates or, failing this, location names.

199 Geographic Regions: Adriatic Sea, Aegean Sea, Afro-tropical region, Albania, Algeria, Andorra, Archipelago Sea, Armenia, Asiatic Turkey, Australian region, Austria, Austria with Liechtenstein, Azerbaijan, Azerbaijan including Nakhichevan, Azores, Balear Sea, Baleares, Baltic Proper, Baltic Sea, Baltic states (Estonia, Latvia, Lithuania) and Kaliningrad region, Barents Sea, Belarus, Belgian Exclusive Economic Zone, Belgium, Belgium with Luxembourg, Belt Sea, Biscay Bay, Black Sea, Bosnia-Herzegovina, Bothnian Sea, Bulgaria, Bulgarian Exclusive Economic Zone, Canary Islands, Caspian Sea, Caucasus region, Central European Russia, Channel Islands, Corse, Corvo, Crete, Crete with Karpathos, Kasos & Gavdhos, Crimea, Croatia, Croatian Exclusive Economic Zone, Cyclades Islands, Cyprus, Czech Republic, Danish Exclusive Economic Zone, Denmark with Bornholm, Desertas, Dodecanese Islands, Dutch Exclusive Economic Zone, East Aegean Islands, East Palaearctic, Eastern European Russia, Egypt, Egyptian Exclusive Economic Zone, English Channel, Estonia, Estonian Exclusive Economic Zone, European Marine Waters, European Turkey, Faial, Faroe Islands, Finland with Ahvenanmaa, Flores, Former Czechoslovakia, Former Jugoslavia, Former USSR, France, Franz Josef Land, French Exclusive Economic Zone, French mainland, Fuerteventura with Lobos, Georgia, German Exclusive Economic Zone, Germany, Gibraltar, Gomera, Graciosa, Gran Canaria, Great Britain, Grecian Exclusive Economic Zone, Greece with Cyclades and more islands, Greek East Aegean Islands, Greek mainland, Hierro, Hungary, Ibiza with Formentera, Iceland, Icelandic Exclusive Economic Zone, Ireland, Irish Exclusive economic Zone, Irish Sea, Israel, Israel-Jordan, Italian Exclusive Economic Zone, Italian mainland, Italy, Jordan, Kaliningrad, Kingdom of Spain, La Palma, Lanzarote with Graciosa, Latvia, Lebanese Exclusive Economic Zone, Lebanon, Lebanon-Syria, Libya, Liechtenstein, Lithuania, Luxembourg, Madeira, Madeira, Madeiran Exclusive Economic Zone, Mallorca, Malta, Marmara Sea, Mediterranean Sea, Menorca, Moldova, Monaco, Montenegro, Moroccan Exclusive Economic Zone, Morocco, Nakhichevan, Near East, Nearctic region, Neotropical region, Netherlands, North Aegean Islands, North Baltic proper, North Caucasus, North Sea, Northern Africa, Northern European Russia, Northern Ireland, Northwest European Russia, Norwegian Exclusive Economic Zone, Norwegian mainland, Norwegian Sea, Novaya Zemlya, Novaya Zemlya & Franz-Joseph Land, Oriental region, Pico, Poland, Porto Santo, Portuguese Exclusive Economic Zone, Portuguese mainland, Republic of Ireland, Romania, Russia Baltic, Russia

4 http://www.tdwg.org/standards/109/

Central, Russia Northern, Russia Southeast, Russia Southwest, San Marino, Santa Maria, São Jorge, São Miguel, Sardegna, Sea of Azov, Selvagens Islands, Serbia including Vojvodina and with Kosovo, Serbia with Montenegro, Sicily, Sicily with Malta, Sinai, Skagerrak, Slovakia, Slovenia, South Baltic proper, South European Russia, Spain, Spanish Exclusive Economic Zone, Spanish Exclusive Economic Zone [Mediterranean part], Svalbard with Björnöya and Jan Mayen, Sweden, Swedish Exclusive Economic Zone, Switzerland, Syria, Tenerife, Terceira, The former Jugoslav Republic of Makedonija, The Russian Federation, Tirreno Sea, Transcaucasus, Tunisia, Tunisian Exclusive Economic Zone, Turkey, Turkish East Aegean Islands, Turkish Exclusive Economic Zone, Ukraine, Ukraine including Crimea, Ukrainian Exclusive Economic Zone, United Kingdom Exclusive Economic Zone, Vatican City, Wadden Sea, White Sea.

APPENDIX II

The Darwin Core Archive Format (DwC-A)

The DwC-A is a derivative of the DwC TDWG standard developed by GBIF to facilitate the exchange of checklist data. It is primarily a mapping of the DwC fields into a CSV file format (as defined within DwC) with an extension mechanism to allow the addition of other fields for taxonomic data. (http://code.google.com/p/gbif-ecat/wiki/publishingChecklists). The DwC-A is supported by the GBIF IPT (Integrated Publishing Toolkit) but is simple enough to be created without specialist software, such as via a database dump or spreadsheet export.
This applicability statement provides a description of how the DwC-A format could be used to exchange basic taxonomic checklists under the PESI umbrella. There may be other fields that need discussion with the PESI Portal team.
If you can publish your checklist data in this format you should be able to submit it to PESI, GBIF and Encyclopaedia of Life.
This guide should give an outline of how to prepare your data but please check with these organisations that you are using the latest version of the standard. This is particularly important as regards the dataset metadata that may vary from project to project.

What Does a DwC-A File Consists Of?

A DwC-A file is a Zip compressed archive of files within a single directory. There are only two required files but here we recommend a minimum of three.

- Core Taxon File (taxa.txt) – a CSV file of the taxa with one row per taxon in the source data set.
- Descriptor File (meta.xml) – an XML file that describes the contents of the taxa.txt file plus any other files. The structure of this file is quite simple and consists of a basic list of the columns in the taxa.txt file with the separator and escape characters used.
- Dataset Provenance (provenance.xml) – a simple XML file that specifies the creator, created date, publisher, rights and licensing for the data in the archive.

A DwC-A may contain other files.

- Extension files – these are CSV files containing data that relates to the taxa defined in the taxa.txt file. They allow one to many relationships to be expressed from taxa to data in extension files such as listing type specimens or vernacular names in multiple languages.
- Dataset Information. A file containing information about the provenance of the data set may be included. The preferably format for this is Ecological Markup Language (EML). EML is an extensible language for describing the contents of datasets.

Simplest Authoring Scenario

In the simplest case a DwC-A **could** be produced following these steps.

- Create a spreadsheet with the correct columns in the correct order using a programme such as MS Excel or Open Office.
- Export the spreadsheet as a CSV file to a new directory.
- Add the supplied meta.xml and provenance.xml files.
- Edit the provenance.xml file to include creation and creator details.

- Zip up the directory by right clicking on it and selecting 'Send to ...' zip folder on a Windows machine or create 'Compress "folder name" on a Mac or equivalent command on a Linux.

In database driven environments this process could be automated or carried out on demand. The GBIF IPT also provides functionality to support publishing checklists in this format.

Taxonomic Hierarchies

There is no need for a DwC-A to contain any hierarchical data. It can contain a list of species without reference to a higher classification at all. This is probably a rarer case as most databases will contain some notion of higher taxonomic placement even if that is only to family or order level.
There are two ways that a taxonomic hierarchy can be represented in DwC-A.

- **Normalised**. In this form each taxon row in the file has a parentNameUsageID field that contains the taxonID of the next higher taxon in the classification. A species row may have the id of the genus row in the file for example. A consuming application can reconstruct the hierarchy by following all these child -> parent links and creating their **reverse** parent->child likes.
  A normalised approach has the advantage that it can handle any number of arbitrary ranks.
- **Flattened.** There are a series of fields available for the major ranks as well as a 'higherClassification' field for providing a semicolon separated list of higher taxa. These fields can be populated for each row with the names of the higher taxa that that particular row is placed in. This approach has the advantage of being simpler to implement but lacks the expressivity of the Normalised approach.

Recommendation: DwC-A files should only contain one approach to encoding the taxonomic hierarchy: either include the parentNameUsageID, or the kingdom, phylum, class, order, family, genus, subgenus, higherClassification fields but not both. This avoids the confusion of possible conflicting or ambiguous classifications.
The list of recommended fields given below is split into two. One for the Normalised appoach and one for the Flattened approach.

Multiple hierarchies.

It is possible for a single taxon.txt file to contain multiple hierarchies of accepted taxa i.e. multiple classifications. There are a minority of receiving applications that are likely to be able to understand and import multiple classifications.
Recommendation: DwC-A files should only contain a single accepted classification. Multiple classifications should be split between different archives to allow people to import a single classification at a time.

Columns in the Spreadsheet

This section defines the columns that should be included in taxonomic checklists. The first part lists the columns common to both Normalised hierarchical approach and the Flattened approach. The second and third sections are specific to the two approaches.

Common Columns

Name	Notes
dwc:taxonID	A unique ID for this taxon or name. At a minimum this should be unique within this particular DwC-A; ideally it should be a GUID

Name	Notes
	and resolvable URI. (See discussion below). Required.
dwc:scientificName	The full taxon name (with authorship and date information if applicable). Required.
dwc:scientificNameID	Exclusively used to reference an external and resolvable identifier that returns nomenclatural (not taxonomic) details of the name. This is a link to a nomenclator. Current examples are Index Fungorum, IPNI and ZooBank LSIDs. Optional.
dwc:genus	The full scientific name of the genus in which the taxon is classified. Optional.
dwc:subgenus	The full scientific name of the subgenus in which the taxon is classified. Optional.
dwc:specificEpithet	The name of the species epithet of the scientificName. Optional.
dwc:infraspecificEpithet	The name of the infra-species epithet of the scientificName. The name of the lowest or terminal infraspecific epithet of the scientificName, excluding any rank marker. Optional.
dwc:scientificNameAuthorship	The authorship information for the scientificName formatted according to the conventions of the applicable nomenclatural Code. Optional.
dwc:taxonRank	The taxonomic rank of the scientificName. This should be one of the ranks defined in http://rs.tdwg.org/ontology/voc/TaxonRank e.g. 'species'. Optional but recommended.
dwc:verbatimTaxonRank	The taxonomic rank of the scientificName as a free text string. Optional but recommended if dwc:taxonRank is absent.
dwc:nomenclaturalCode	The nomenclatural code under which the scientificName is constructed. Recommended best practice is to use a controlled vocabulary. One of ICBN; ICZN; BC; ICNCP. Required.
dwc:taxonomicStatus	The taxonomic status in the context of this classification in this DwC-A. This row represents a taxon that is either accepted, sunk into synonymy (in the broad sense) or should be ignored. i.e. if this were a printed list it would represent a list entry in bold or a sub-entry to another taxon. One of 'accepted', 'synonym' or 'rejected'. Required for taxon lists.
dwc:acceptedNameUsage	If the dwc:taxonomicStatus is 'synonym' then this field contains the name of the taxon that is accepted in its place. Optional.
dwc:acceptedNameUsageID	If the dwc:taxonomicStatus is 'synonym' then this field contains the ID of the taxon that is accepted in its place. The ID should either be the dwc:taxonID of a row in the file and/or a GUID. Optional.
dwc:nameAccordingTo	To use a name precisely an indication of which concept of that name one refers to is needed. Traditionally the Latin sensu or sec. (for secundum – according to) have been used. For taxa that result from identifications a reference to the keys or monographs used, online source or experts should be given. Could be a publication (identification key), institution or team of individuals. Optional.
dwc:nameAccordingToID	A unique identifier that returns the details of a taxonAccordingTo reference. Optional.

Name	Notes
dwc:originalNameUsage	The basionym (botany) or basonym (bacteriology) of the scientificName or the junior/later homonym for replaced names. Optional.
dwc:originalNameUsageID	The ID of the basionym (botany) or basonym (bacteriology) of the scientificName or the junior/later homonym for replaced names. Optional.

Normalised Taxonomy Columns

Name	Notes
dwc:parentNameUsageID	The ID of the parent taxon. The ID should either be the dwc:taxonID of a row in the file and/or a GUID. Required.

Flattened Taxonomy Columns

It is recommended that either the kingdom/phylum/class/order/family columns be used or the higherClassification column and not both as they may contain ambiguous information.

Name	Notes
dwc:kingdom	The full scientific name of the kingdom in which the taxon is classified. Optional.
dwc:phylum	The full scientific name of the phylum in which the taxon is classified. Optional.
dwc:class	The full scientific name of the class in which the taxon is classified. Optional.
dwc:order	The full scientific name of the order in which the taxon is classified. Optional.
dwc:family	The full scientific name of the family in which the taxon is classified. Optional.
dwc:higherClassification	A list (concatenated and separated) of taxa names terminating at the rank immediately superior to the taxon referenced in the taxon record. Order the list starting with the highest rank and separating the names for each rank with a semi-colon (";"). Optional.

Names vs Taxa

Each row is considered to be a taxon that is either accepted by the current classification or rejected (as a synonym or rejected name). Sometimes there may be a need to express purely nomenclatural information in DwC-A — particularly when dealing with nomenclators. In such cases no taxonomic hierarchy should be provided and no indication of taxonomic status given.

Additional Nomenclatural Information

The fields suggested here are not capable of expressing the full complexity of biological nomenclature as they are focussed instead on expressing the products of the nomenclatural process i.e. A list of correctly named taxa. DwC and the DwC-A format allows for expansion of the taxa.txt file with additional fields. The user is referred to the appropriate documentation on the GBIF ECAT site should additional fields be required. http://code.google.com/p/gbif-ecat/wiki/ChecklistFormat. Bear in mind that consumers of the DwC-A files may not make use of fields other than those listed here. It may be worth establishing how the data will be used before committing resources to curating and publishing it. Full nomenclatural details may be

better expressed in RDF or TCS XML as the result of a call to a URI as is done at the moment by IPNI and Index Fungorum.

Default Values

If you are publishing a classification of, for example, organisms who's names are only governed by plant or only animal codes of nomenclature, some of the columns listed above would contain the same value in every row. All the names would be governed by the ICBN or ICZN for example. In these situations it is possible to specify the default value for the field in the meta.xml file using the 'default' attribute of the field element. There are examples of how to do this on the GBIF ECAT Wiki (http://code.google.com/p/gbif-ecat/wiki/DwCArchive).

Vernacular Names

DwC-A offers two approaches to including vernacular names. The dwc:vernacularName field could be added directly to the taxa.txt file with the vernacular name in it or a separate extension file could be included and mapped to the taxon.txt file using the dwc:taxonID column.
Recommendation: Including the vernacular name directly in the taxa.txt does not allow for expressing the language or location of the vernacular usage. It is recommended, therefore, that this approach is not taken and that a separate file is included instead. This file should contain the language and location of the usage of the vernacular name either within the file itself or using the default values mechanism in the meta.xml file (see above). There are examples of how to do this on the GBIF ECAT Wiki (http://code.google.com/p/gbif-ecat/wiki/DwCArchive).

Distributions

If distribution data is to be included it should be included as an extension file as per the recommendations on the GBIF ECAT Wiki (http://code.google.com/p/gbif-ecat/wiki/DwCArchive). There is a direct mapping between recommendations in previous reports and the columns suggested by GBIF.

Taxon Identifiers

Each row in the taxa.txt file is identified by an index column — the dwc:taxonID. This should be a locally unique ID within the scope of the DwC-A. It is used to reference the row from the dwc:acceptedNameUsageID, dwc:parentNameUsageID and dwc:originalNameUsageID fields and also to link the extension files to the core taxa.txt file. There is no requirements for the identifier to be a GUID (out-with the scope of the file). Use of GUIDs in this field would make it easier for consuming applications to work on multiple data sets that cite each other and is encouraged.

Provenance Data

It is important to include provenance data in the archive as this permits users of the data to follow any rights restrictions and give appropriate attribution. This data should be included in a simple XML file (provenance.xml) and linked to from the meta.xml file's achive@metadata attribute.

```
<?xml version="1.0"?>
<metadata xmlns:dc="http://purl.org/dc/terms/">
  <dc:title> ... </dc:title>
  <dc:description>... </dc:description>
  <dc:subject>... </dc:subject>
```

```
    <dc:created>... </dc:created>
    <dc:creator>... </dc:creator>
    <dc:publisher>... </dc:publisher>
   <dc:license>... </dc:license>
</metadata>
```

The file should contain properties from the Dublin Core /terms/ name space as defined by Dublin Core (http://dublincore.org/documents/dcmi-terms/). Those terms illustrated above are recommended. It is also recommended that the dc:license property contain the URI of a Creative Commons license and the dc:publisher property contain the URI of a institution or project web site if possible.

RDF and the Semantic Web

The DwC-A format is highly amenable to being mapped to RDF so as to make the data available for use on the Semantic Web as Linked Data or used in OWL Ontologies for semantic reasoning. A normative mapping is not defined here as it is still an area of active research. Multiple mapping strategies may be used depending on the use envisaged for the resulting RDF.
