# Supplementary material for: PESI - a taxonomic backbone for Europe
Source: Biodivers Data J. 2015 Sep 28;(3):e5848. doi: 10.3897/BDJ.3.e5848 (PMC4609752; doi:10.3897/BDJ.3.e5848)
Supplement: Supplementary material 11 — Report on authoritative taxonomic standards from multiple sources suitable for deployment within European Research Area. [file biodiversity_data_journal-3-e5848-s011.pdf]

D4.1 — Report on authoritative taxonomic standards from multiple sources suitable for deployment within European Research Area.

This report constitutes deliverable D4.1 of Work Package 4 (WP4) of the Pan-European Species-directories Infrastructure (PESI). This document (and its successors) should be seen as critical to the success of the PESI project. It is a practical guide to aid the integration of the component PESI databases with each other and with external users and suppliers of taxonomic information.

Subsequent reports will deal with tighter nomenclator integration (D4.2: Report on procedures and mechanisms for the functioning of nomenclators within the intended European taxonomic e-infrastructure), practical integration with consumers of taxonomic data (D4.3: Report on possible beneficiaries of EU taxonomic e-infrastructure and potential impact) and contributions to the global taxon/name architecture (D4.4: Report on the contributions to the set up of a Global Name Architecture).

1. Why Standards are Needed

The internet has massively increased the importance of "distributed innovation" - something that has been supported on paper by the scientific publishing process for several centuries. Distributed innovation is the notion that in any given sphere of activity most of the pertinent knowledge will reside outside of the boundary of any one organisation. Development of knowledge therefore occurs across organisations and projects by re-cycling and building on the work of others who are not part of this particular endeavour.

Prior to the widespread adoption of internet technologies only the results of discrete scientific investigations were widely shared, in the form of journal and book publications. It is now possible to share data and the results of studies at much finer granularity, at the level of data sets or even individual data points. This granularity also extends to time slicing where data may be available in near real time from environmental sensors (e.g. sensor web).

Publishers and libraries have well established processes for handling the products of science at the level of individual publications. There are systems for cataloguing and data retrieval. There are social norms for citing and giving attribution to the work of others. These standards do not exist for data at finer granularity shared across the internet - but there is an active effort to establish such standards within the biodiversity informatics community.

A biologist carrying out a study that uses data from multiple sources needs to be able to compile that data into a single analysis. They need to maintain attribution, reproducibility and transparency. This can only be done if the suppliers of the data have

some level of uniformity in the way they present or publish data. It is particularly important that the suppliers tag their data with Globally Unique Identifiers (GUIDs) – see linked data below. Only when the major biodiversity initiatives embrace the shared standards development process will it be possible to build non-trivial biodiversity informatics applications that enable real science and informed decision making to take place.

2. Types of Standards

A technical standard is an established and documented norm or requirement of a system. WP4 recognises four categories of standards.

- **Standardised Biological Taxonomies** – As described below, PESI will become a supplier of a standardised biological taxonomy and will be involved in integrating with other standardised taxonomies. Because integration with other taxonomy providers will be largely at a nomenclatural level this is dealt with in the following report D4.2 "Report on Procedures and Mechanisms for the Functioning of Nomenclators within the e-Infrastructure". This report concentrates on standards used to build and maintain the standardised biological taxonomy.
- **Generic Information Technology Standards** – These are standards defined outside the biodiversity informatics community by organisations such as the Internet Engineering Task Force (IETF), World Wide Web Consortium (W3C) and International Standards Organisation (ISO). They include standards such as the Hypertext Transfer Protocol, XML, RDF and two letter country codes.
- **Community Specific Data Exchange Standards** – TDWG (Biodiversity Information Standards) is the standards organisation dedicated to the biodiversity informatics community. Since 2000 TDWG has acted as a forum for the development of a series of exchange standards including DiGIR, DarwinCore, BioCASe and ABCD. These standards are dealt with in the Appendix to this report.
- **Community Specific Controlled Vocabularies** – TDWG defines a series of controlled vocabularies for use in taxonomic databases. These included a geographic regions standard and a plant occurrence status schema (POSS). Both these standards are of relevance to PESI, they are mentioned below and will be dealt with further in separate application-specific documents.

PESI WP4 will be guided by two principles in recommending the adoption of standards:

- Adopt more widely used standards over those of limited applicability wherever possible.
- Adopt simpler standards over more complex ones.

3. Standards Development and Maintenance

PESI is not a standards development organisation but a user of established standards and a participant in the development of standards within standards bodies. TDWG is the standards body for the biodiversity informatics community. PESI will therefore work with other organisations and projects, such as GBIF and the Encyclopaedia of Life, under the umbrella of TDWG, to develop the standards needed to share data where these standards do not already exist.

Standards development and maintenance is a very time consuming (and therefore resource intensive) process. Even in the recently streamlined TDWG standardisation

process it may take several years to reach consensus around a new standard and marshal it through the standards process. WP4 will not sponsor the development of new standards but will contribute to existing standards development efforts.

4. Standards and PESI

The role of PESI is to accelerate the rate of distributed innovation around the European Biota. It aims to enable scientists and other users of biological data to carry out their work more efficiently.

To do this PESI must exploit data held by others, such as the Global Species Databases (GSDs) and make its own data available for use in harmony with other regional and global checklists such as ITIS (Integrated Taxonomic Information System), Australian Faunal Directory and Species2000. This can only be achieved if PESI adheres to the same standards as its 'suppliers' and 'customers'. Because many of these standards are immature PESI must actively collaborate with partner projects and organisations to establish these standards. As a significant focal point of taxonomic expertise PESI can play an active role in the development of some standards whilst supporting the acceptance of others through their use and endorsement. The vehicle for collaboration with others on standards development is likely to be Biodiversity Information Standards (TDWG), but this does not preclude participation in other standardisation efforts like the GBIF task groups and EDIT working groups.

5. Scope

> "*PESI provides standardised and authoritative taxonomic information by integrating and* securing Europe's taxonomically authoritative species name registers and nomenclators (name *databases) that underpin the management of biodiversity in Europe.*" - PESI Description of Work.

Biodiversity is a broad term encompassing habitat, species and genetic level heterogeneity of a region. In this case the region extends from Europe across the entire Western Palaearctic region. PESI is concerned with delivering an infrastructure for managing the taxonomic component of this diversity.

There exists a range of taxonomic products. In order of increasing detail these are:

1. **Names** (Check)**lists** – These are lists of pure names without any indication of whether the names represent accepted, real world taxa or not. Synonymy may be included in these lists but only homotypic (objective) synonymy concerned with name string construction.
2. **Nomenclatural Checklists** – These are lists of names including the nominal taxa, meaning the registry of published usages of scientific names representing nomenclatural acts as governed by the respective Codes of Nomenclature. Most of these acts are 'original descriptions' of new scientific names, but other acts may include emendations, lectotypifications, and other acts as governed by the Codes. Synonymy is not included in these lists as taxonomic concept, but only as newly established combination (for botanists) linked to a basionym.

   Other kinds of data objects linked to the registration of nomenclatural acts are may also be included, like:

   - Publications that contain Nomenclatural Acts (as defined above)
   - Names of Authors of the relevant publications

- Type specimens allocation

In general this is the kind of data provided by nomenclatural checklists or databases. When a nomenclatural database or a taxonomic database representing nomenclatural information is formally or functionally accepted by a community as common (single) reference point on controlled vocabulary (e.g. correct spelling of taxon names) and for regulating effective cross-linking this is usually called a **nomenclator**.

3. **Heterotypically Synonymised Checklists** – These lists build on nomenclatural checklists by adding taxonomic opinion. They are lists of the names of accepted taxa for a region with other names placed in synonymy or rejected. Although these lists imply the existence of accepted taxa they do not supply circumscriptions of those taxa.
4. **Annotated Checklists** – These lists build on heterotypically/subjectively synonymised checklists by adding other data. Information could include indications of distribution and threat status. Links could be included to preferred descriptions of the taxa in monographs.
5. **Fauna/Flora Accounts** – Typically these are books that provide circumscriptions suitable for the region covered but not necessarily global in scope. They are usually derived from monographic accounts or reflect the current state of knowledge for a taxonomic group.
6. **Monographs** – Detailed nomenclature, synonymy and taxon circumscription, phylogenetic and other data. Descriptions are typically global in scope.

In this spectrum of taxonomic knowledge **PESI is an Annotated Checklist.** Although it may combine its core taxonomic data with other data to provide rich 'Species Pages', in the first instance it is a single taxonomic list for Europe capable of producing compatible sub-lists for different regions.

Subject areas that are clearly **in scope** and for which relevant standards need to be considered are: Taxon Names, Taxon Concepts, Common Names, Authority Names, Geographic Regions, Regional Occurrence Status, Conservation status.

Subject areas that are **out of scope** for the core dataset in the first instance includes: specimen data and individual organisms occurrence data, descriptive data, abundance and other ecological data and molecular data.

6. Two Phases of PESI Standardisation

There are two clear phases in the exploitation of standards by PESI.

Phase I: Integration of Core databases. During this phase a mechanism will be established for integrating data from Euro+Med PlantBase (E+M), Fauna Europaea (FaEu) and European Register of Marine Species (ERMS) databases into a single PESI data store. This will involve moving E+M and FaEu into a single instance of the Common Data Model data store (CDM) - as developed by the EDIT project - and then merging an export from this store into an integrated, denormalised (star-scheme), somewhat condensed, PESI data warehouse that includes an export from ERMS. This PESI data warehouse will be used to drive the PESI data portal. This process is being engineered by teams at Vlaams Instituut voor de Zee (VLIZ) and Botanischer Garten und Botanisches Museum (BGBM) and is totally 'bespoke' – designed to solve only this problem. At the same time VLIZ will develop a database containing supplemental data that is held in parallel to the core data warehouse and that serves to enrich the species pages presented through the PESI

web portal.

The three databases being merged were developed independently and so have different internal controlled vocabularies for some key fields. Phase I of standardisation will deal with standardising the vocabularies used in the CDM and combined PESI data warehouse, so as to meet three needs:

1. Accurately represent the contents of the source databases in the PESI data warehouse - although with an acceptable loss of precision.
2. Allow source databases to continue using more precise terminology internally by improving the CDM information model.
3. Map to publicly available standard vocabularies for integration with other projects where appropriate. This may involve defining those standards in collaboration with other projects, starting with the Europe-based GSDs.

Phase I is largely inward looking and necessary for the initial development of the PESI infrastructure. Phase I will be based almost entirely on this report and should be complete by mid 2009.

Phase II: External integration. Once the core structure of PESI is in place and the synchronisation mechanism allows the three source databases to be exposed through the prototype portal then attention can move to how external projects will fully exploit the PESI data set and how PESI can benefit from other projects. The work programme for Phase II will be briefly outlined in this report. The second and third reports of WP4 (D4.2 and D4.3) will detail the implementation of Phase II with other projects.

What follows deals with the key fields that need to be agreed on as part of Phase I. Much of this comes out of discussions held at VLIZ on 18th and 19th March 2008. The final part of the report outlines the standards that will need to be implemented in Phase II to enable integration with others.

Appendix A gives a break down of current TDWG and related standards and their relationship to PESI.

7. Internationalisation and Localisation

The description of work states that the interface for the PESI web portal will be internationalised and localised into major official European languages. This presents a problem with regard to the localisation of data. If a user is confronted with an interface in their own language they will expect to access data in that language. It is not enough to only internationalise the interface - the data must also be internationalised to create an acceptable user experience. PESI will meet this challenge by minimising the use of free text strings within the data structure to minor comments. As much data as possible will be stored in controlled vocabularies that can be translated as the portal is localised to different languages. The nature of PESI data is particularly suited to this approach. This approach will also facilitate sharing data externally and the sharing of controlled vocabularies with other projects.

8. Taxonomic Ranks

Part of Phase II of standardisation will be submitting improvements to the TDWG TaxonName vocabulary and by implication the TaxonRank vocabulary. This is an extensive (but not comprehensive) list of rank terms. Although the International Code of Zoological Nomenclature (ICZN) stipulates the ranks that can be used, the International

Code for Botanical Nomenclature (ICBN) allows authors to establish their own ranks and so it is virtually impossible to track all possible taxonomic ranks. It is also undesirable from the point of view of interoperability to allow any arbitrary rank within the database. PESI will take a pragmatic approach to taxonomic rank and only recognise the 35 ranks listed below. These cover the ranks used in the three source databases and the vast majority of ranks used for taxon names externally. Phase II will ensure that these internal rank names are mapped to the external rank terms proposed by TDWG.

Kingdom	Order	Section (Botany)
Subkingdom	Suborder	Subsection (Botany)
Division	Infraorder	Aggregate
Subdivision	Section (Zoology)	Coll. Species
Phylum	Subsection (Zoology)	Species
Subphylum	Superfamily	Subspecies
Infraphylum	Family	Variety
Superclass	Subfamily	Subvariety
Class	Tribe	Forma
Subclass	Subtribe	Taxa infragen.
Infraclass	Genus	Taxa infraspec.
Superorder	Subgenus	

9. Taxon Name Author Strings

Taxon name author strings are used to disambiguate homonyms and near homonym names. Unfortunately the codes of nomenclature (ICBN and ICZN) do not stipulate in detail how these author names should be cited neither maintains a controlled vocabulary on author names, especially on the use of initials. Two full taxon name strings (that include author strings) for the same taxon name can therefore contain different sets of characters – sometimes radically so. PESI does not mandate a format for author string or author string abbreviations but defers to the source databases that will be presumed to follow best practice. In botany this may be to use the abbreviations as supplied by the IPNI Authors service. For zoology this could be a joint effort with the ZooBank community.

10. Character Encoding

To avoid character encoding errors all data passed in and out of PESI will be in UTF-8 encoded.

11. Common Names

There are two possible approaches PESI could take to common (vernacular) names. It could take a lexicographical approach of tracking the occurrence of all names used for biological organisms in the regions and languages of the (Western) Palaearctic, or it could take a more prescriptive approach, simply stating the preferred common name for each taxon in a particular language/region when there is one. It was agreed at the

March '09 meeting at VLIZ to take the latter approach. PESI will provide preferred common names for taxa. It will include common names already present in PESI source datasets, supplemented by common names gathered by PESI taxon experts and Focal Points. PESI will define a standardised approach to include common names, but not deal with common name standards.

Justification: PESI is primarily an annotated checklist of taxa for use in other studies. Developing a complex thesaurus of common names falls more within the scope of an ethnographic or linguistic project. This is the kind of study that PESI will enable by providing a taxonomic backbone, rather than carrying out itself.

Each taxon will have one or more common names associated with it. A common name will consist of a UTF-8 encoded string plus a language tag in accordance with the Internet Engineering Task Force (IETF) best current practice for the contents of language tags BCP 47. Typically this is the two-letter language code from ISO 639-1 followed by a hyphen followed by a two-letter country code from ISO3166-1 for example the familiar “en-US”. It also allows for more sophisticated use e.g. "sr-Latn-CS" represents Serbian ('sr') written using Latin script ('Latn') as used in Serbia and Montenegro ('CS').

Justification: Interoperability is promoted by adopting widely used standards where they are applicable. The IETF language tags are widely used and understood by many applications. They are the standard recommend for the contents of xml:lang attributes in XML and so facilitate the import/export of data in XML serialisations. They are also used in HTML and HTTP content negotiation. An alternative approach would have been to use entries from the geographic codes used for taxon distributions as the geographic component of the language tag but this would have prevented communication using standard internet protocols.

Many countries have authorities for the official use of words (e.g. Académie française) or widely accepted *de facto* standards such as the Oxford English Dictionary in the UK and the Van Dalen Dictionary in the Netherlands. Taxonomic experts should defer to these sources for common names where there is such an authority and where that authority records a name for a particular taxon.

12. Geographic Regions Vocabulary

PESI will build a vocabulary of geographic regions. This will act as a standard list of regions for which the occurrence status of taxa will be stored. It will be the level at which PESI will be able to offer regional checklists. The vocabulary will be based on the TDWG standard (World Geographical Scheme for Recording Plant Distributions) for land areas, the used geographic standards of FaEu and E+M (as referred in their project guidelines) plus additional areas for seas developed by VLIZ for ERMS. The vocabulary will consist of written descriptions of the regions and the nesting of regions into a hierarchy. The regions used in the three source databases will be mapped into this single vocabulary. VLIZ will develop a series of geospatial polygons for the regions. These polygons will be used as a data validation tool for point data produced by GBIF, BioCASE and others.

PESI will participate in the TDWG process to have the vocabulary ratified as an extension or formalisation of the existing TDWG standard. A decision will be taken at a later data on standardisation of polygons for the areas.

PESI may also maintain a more detailed gazetteer of place names for other purposes.

Justification: Use of standard country codes such as ISO3166-1 is not appropriate as administration/political regions do no always map to regions of biological significance. An example would be the island of Ireland, which includes part of both the United Kingdom (GB) and all of Ireland (IE), or the British Isles, which includes GB and IE as well as other jurisdictions. In addition to this the political divisions are not appropriate to maritime areas, which may be split between multiple national waters and international waters.

13. Taxon Occurrence Status Vocabulary

PESI will provide occurrence data on a regional basis, using the regions defined in the Geographic Regions Vocabulary (10 above) and occurrence statuses from a standardized Taxon Occurrence Status Vocabulary.

The three source databases have different ways of recording occurrence status. E+M has the most complex occurrence statuses based on a modified version of the TDWG (Plant Occurrence and Status Scheme) with more than twelve statuses. ERMS and FaEu have simpler systems based on presence or absence, with a qualifier for confidence.

A unified Occurrence Status Vocabulary will be developed that allows the complex status schema used by E+M to be mapped to a simpler vocabulary that will be exposed through PESI. This new vocabulary will be extensible, enabling complexity to be recorded in the source databases but not necessarily through the PESI portal. The new vocabulary will be offered up as a TDWG standard, possibly to replace the existing Plant Occurrence and Status Scheme (POSS) standard.

Justification: The current POSS TDWG standard does not meet the needs of the existing databases and there does not appear to be a standard that could be used in its place. Of primary importance is the ability to map between complex occurrence statuses such as 'Winter Breeding Migrant' and simple statuses such as 'Present' without loss of accuracy, as well as to relate currently used status codes into any new vocabulary.

14. Legal Protection and Conservation Status

The legal protection- and conservation- statuses of taxa will be considered to be supplemental data and will be harvested from EU Habitat and Bird Directive (European Environmental Agency, EEA) and International Union for Conservation of Nature (IUCN) once the taxonomy within PESI, EEA and IUCN has been reconciled. PESI will therefore adopt the Habitat Directive statuses of Conservation (Annex II), Strict protection (Annex IV), and Bird Directive statuses of Near Extinction (Annex I), Rare (Annex I) as well as the IUCN Red List statuses of Extinct (EX), Extinct in the wild (EW), Critically Endangered (CR), Endangered (EN), Vulnerable (VU), Near threatened (NT), Least Concern (LC), Data deficient (DD) and Not Evaluated (NE).

For the marine species PESI will adopt the OSPAR statuses of Threatened (TH) and Declined (DC) as well as the marine IUCN Red List statuses.

For completeness it may be necessary to collaborate on producing a TDWG vocabulary of these terms for use by semantic web technologies. Currently the statuses can't be used in the context where URIs are required.

Justification: The use of external vocabularies is always preferred over creating new ones.

15. Work Plan for Phase I

A pilot of the PESI portal is up and running since May 2009. It is therefore important that the standards necessary for the integration of the three source databases are in place before this date. They do not have to be integrated within the TDWG process but they do need to be clearly enough defined to enable practical mappings between the databases. The priority for development of standards is therefore to produce a Taxon Occurrence Status Vocabulary and a Geographic Regions Vocabulary first. During Phase II these vocabularies will be further developed and promoted as a means of integration with others. These activities will be led by WP4.

16. Outline of Phase II Standardisation – External integration

Phase II of the standardisation process will look at how PESI data is exposed to other users so as to maximise its utility – supporting the notion as **PESI as a service**. The same standardisation process will facilitate PESI's access to other data sources, such as those provided by the GSDs. To support the functional development of the PESI portal from a user perspective, an end-user forum has been established by at the PESI website.

17. Linked Data

The underlying paradigm that will be adopted is that of Linked Data. Linked Data is the term used to describe a recommended best practice for exposing, sharing, and connecting pieces of data, information, and knowledge on the Semantic Web using URIs and RDF. This paradigm will be adopted within PESI because it appears to address most of the issues in linking heterogeneous data across disciplines. If PESI is to be a taxonomic backbone it must facilitate cross-subject domain linking. Although this will be the underlying paradigm, a pragmatic approach will be taken to supplying data in whatever form clients require – but always with reference to the Linked Data behind it.

18. TDWG TaxonConcept and TaxonName Vocabularies.

PESI is a taxonomy provider and as such will publish its data using the requisite standards. The TDWG TaxonName/TaxonConcept vocabularies are ideal candidates for this kind of communication and are already in use by IPNI, Index Fungorum, ZooBank, MycoBank, Tropicos and Catalogue of Life. They need developing further and ratifying by a standards body for wider adoption. WP4 will engage with and take a leadership role in this standardisation process. Once the three core databases have been integrated into the prototype portal and the standard is stable, WP4 will deliver recommendations on how to expose PESI data in accordance with these standards.

19. Globally Unique Identifiers (GUIDs).

The term Globally Unique Identifier (GUID) is used in two slightly different ways. In computer science GUIDs are values that are complex strings of characters that are extremely likely to be unique in any context. In the biodiversity informatics community the term is used in a narrower sense. In this sense GUIDs have three related properties. They are not only globally unique they are also resolvable (or actionable) and identify a typed object.

Uniqueness: There are two principle ways of achieving global uniqueness. One is to

generate a long and complex number that is highly unlikely to be generated twice and so is functionally unique. This approach enables distributed systems to uniquely identify data without significant central co-ordination. The most common implementation of this approach is the Universally Unique Identifier (UUID) standard. UUIDs are widely used in lower level computing applications such as distributed file systems. Another way to establish uniqueness is the use of a central issuing authority. An example of this approach is the Domain Name System (DNS). DNS is a hierarchical naming system for resources on the Internet including websites and email servers. The Internet Corporation for Assigned Names and Numbers (ICANN) issues top level domain names such as .com to lower level issuing authorities who issue subdomain names (e.g. example.com) who then have authority over issuing subdomains of these subdomains. Theoretically this can carry on for up to 127 levels but practically rarely exceeds five or six. The domain names are then used in protocols such as the Hypertext Transfer Protocol (HTTP) which enable the addition of values after the domain name (e.g. http://example.com/123). DNS has been used to define what is termed a namespace. The locally unique identifier '123' is globally unique when it is combined with "http://example.com/".

Resolution: UUID type GUIDs are useful in distinguishing between, say, dots on a map when the location and names of those dots may change through time - tagging them with UUIDs provides an unambiguous identifier. What UUIDs do not help with is the provenance of the data behind the dots. If an application needs to know more than the information provided to plot the map (such as the licensing terms or whether the data has been modified since issue) then it must be able to do something with the GUID to access the original data source. The GUID needs to be resolvable to some meaningful information. The identifier contains information which refers to data stored elsewhere, as opposed to containing the data itself. Accessing the value referred to by a reference is called dereferencing. An analogy of this process would be the citing of references in published works. The citations act as identifiers that can be dereferenced to the original papers in the library. Resolution of identifiers is only possible with some form of centralised authority with which the identifiers have been registered. Simply searching for identifiers does not provide authoritative data about GUID tagged data as it may result in multiple hits which may contain different data.

Typing: When a researcher fetches a referenced work from the library they are usually certain how to handle it. It will be a paper or book of some form probably in a language they can read or recognise. Likewise when a machine dereferences an identifier the response it receives needs to be understandable both syntactically and semantically, so that it can display the response in an appropriate way or carry out further calculations. The GUID resolution therefore needs to be linked to some form of typing mechanism. If a machine is presented with a GUID it should, for example, be able to tell the users that the GUID represents a taxon from a particular taxonomic treatment.

Technologies: There is some debate over the use of GUID technologies in the biodiversity informatics community. This debate is on-going and some of the technologies involved are summarised here. Unfortunately it is not possible to avoid an 'alphabet soup' of acronyms when discussing these technologies.

The most widely used identifiers on the internet are HTTP Uniform Resource Identifiers (HTTP URI) these include the HTTP Uniform Resource Locators (HTTP URL) used as addresses for web pages. HTTP URIs on their own provide uniqueness and resolution but not response typing. Following the set of best practices proposed under the banner 'Linked Data' does provide response typing however.

Life Science Identifiers (LSID) were first proposed by Object Modelling Group and IBM. After several workshops TDWG adopted LSID as its preferred GUID technology. They provide uniqueness, resolution and response typing. The default resolution mechanism is based on DNS (as with HTTP URIs) but there are very few clients that exploit it. Most LSIDs are resolved by being appended to the HTTP URL of a proxy program that fetches the associated data and metadata.

The motivation for TDWG choosing LSID over HTTP URI was principally social. HTTP URIs are considered inherently unreliable by many users because of their experience with broken webpage links and their ease of creation. It takes a conscious action on the part of administrators to implement LSIDs and this instils a sense of importance to their maintenance. A similar motivation is given for the adoption of Digital Object Identifiers by the publishing community. DOIs have a similar resolution mechanisms to LSIDs using either an HTTP URI proxy or the Handle System.

In order to fully integrate with other data sets whilst providing provenance of data, PESI must tag its taxa with resolvable GUIDs. Applying the two principles for adoption of standards outlined above (widest used and simplest first) implies the use of HTTP URIs in the first instance. Appendix B is give a list of tasks required to implement use of these identifiers in the time-scale of the current project.

20. Interaction with Nomenclators.

An important part of integration is to ensure that PESI can make use of other people's identifiers - linking out. Primary amongst those are the GUIDs provided by the global nomenclators (IPNI, Index Fungorum, MycoBank, Tropicos and ZooBank) and those provided via the Global Names Architecture as proposed by GBIF.

In order for PESI to do this it needs a service to call that can provide a GUID in response to a name string. Although some suppliers have implemented prototype services along these lines there isn't a standard service definition. WP4 will work with the nomenclators to define such a service for the benefit of all. This will be the “Code Compliant Names Service”.

21. Managing Changing Taxonomies.

Taxonomy is a living science and taxa will continue to change, either being sunk into synonymy or added to. A policy needs to be developed for how the implications of any change are communicated to users through the use of TaxonName/TaxonConcept standard and appropriate GUIDs. WP4 will develop this policy in collaboration with other taxonomy providers.

22. Tool integration.

Concurrently with defining **PESI as a service,** we shall examine how these services can be used and promote their use. How will PESI integrate with biological recording packages for example? What niches do standardised PESI data open up for exploitation by new tools?

Appendix A: Existing TDWG and Related Standards

This appendix lists existing TDWG and other standards that may be of relevance to the PESI project.

TDWG Standards Documentation Specification

Specifies how standards should be documented under the new TDWG process introduced in 2006. Any standards submitted to TDWG process will have to follow this standard.

Access to Biological Collection Data - version 2.06 (ABCD)

An XML Schema based standard ratified in 2005 that facilitates the exchange of data between natural history collections. ABCD is the most widely used exchange format in Europe. ABCD is widely deployed in the BioCASE network using the BioCASe protocol. ABCD is a more complex equivalent to DarwinCore. ABCD is actively managed and updated.

Relevance to PESI: Users of ABCD are highly likely to want to exploit the PESI taxonomic backbone. PESI should make documentation available as to how to reference the PESI taxonomy from within ABCD for determinations of specimens and observations.

BioCASe

BioCASe is the protocol used to exchange ABCD documents in the BioCASE network. BioCASe has never been ratified as a TDWG standard and is likely to be replaced by TAPIR.

Relevance to PESI: This protocol is out of scope for PESI.

Darwin Core

There are several versions of the XML Schema based exchange standard. DwC is the most widely used format but it has never been ratified as a TDWG standard. A unified version, with extensions is ready to be submitted.

Relevance to PESI: Users of Darwin Core are likely to want to exploit the PESI taxonomic backbone but also likely to want to use other taxonomy providers such as ITIS. PESI should make documentation available as to how to reference the PESI taxonomy from within Darwin Core.

DiGIR

The exchange protocol used to serve DarwinCore and other federation schemas that have been bound to it. It has never been ratified as a TDWG standard. It is now being replaced by TAPIR.

Relevance to PESI: TAPIR should be used in preference to DiGIR in new networks. DiGIR is out of scope for PESI.

Structured Descriptive Data

SDD is an XML Schema based standard ratified in 2005 that facilitates the encoding of

taxonomic descriptive data and diagnostic keys. SDD is a replacement for the DELTA language. SDD is actively supported and there is discussion on producing RDF compatible SDD-Lite in the future.

Relevance to PESI: SDD is being used for the parallel EU project of Key To Nature.

Taxonomic Concept Transfer Schema

An XML Schema based standard ratified in 2005 that facilitates the transfer of nomenclatural and taxon concept data. TCS promotes the separation of nomenclature and taxonomy in data exchange and is represented almost entirely in the LSID Vocabularies of the TDWG Ontology. Most deployments of TCS use the ontology representation rather than one based on the XML Schema.

Relevance to PESI: PESI should not use the XML Schema version of TCS but work with others to standardise the RDF based version in the TDWG vocabularies.

Herbarium Information Standards and Protocols for Interchange of Data (HISPID3)

HISPID3 is non-XML, flat file based exchange standard ratified in 1996 that has been replaced by HISPID4 (which is not ratified by TDWG).

Relevance to PESI: HISPID is out of scope for PESI.

Economic Botany Data Collection Standard

A book ratified as a standard in 1995.

Relevance to PESI: This is an old standard and there is no active group within TDWG maintaining it. It is currently out of scope for PESI though may become of importance with regard to supplementary data. This standard should be examined in relation to other efforts to standardise species pages such as that coming out of EoL and GBIF.

Plant Occurrence and Status Scheme

POSS is a controlled vocabulary of terms ratified as a standard in 1995 that is used as a lookup tables in curation databases.

Relevance to PESI: These controlled vocabulary terms need to be made accessible as URIs as part of the TDWG Ontology for integration with legacy systems. PESI will undertake this work as part of standardising the occurrence vocabularies used internally.

Plant Names in Botanical Databases

Recommendations for storing botanical names in databases ratified as a standard in 1994. This work is largely superseded by Taxon Concept Transfer Schema and its associated documentation. It may form the basis of older curatorial database schemas.

Relevance to PESI: Out of scope for PESI as replace by modern standards.

Authors of Plant Names

A book of author abbreviations ratified as a standard in 1992 and maintained as a

database accessible through. http://www.ipni.org. The on-line version is not ratified by TDWG. This is a potential TDWG data standard.

Relevance to PESI: There is no official mechanism that standardises the IPNI authors database within TDWG so it is difficult to see how it could be adopted directly by PESI other than through best practise by the source databases.

World Geographical Scheme for Recording Plant Distributions

A list of geographic regions widely used in curation of databases and ratified as a standard in 1992. The boundaries of these regions have been defined as ESRI Shape files but these files are not ratified. The regions have been described as part of the TDWG Ontology http://rs.tdwg.org/ontology/voc/GeographicRegion.

Relevance to PESI: PESI will actively pursue the future development of this standard and its extension to cover European seas.

XDF - A Language for the Definition and Exchange of Biological Data Sets

An early XML based standard now deprecated.

Relevance to PESI: This is a 'dead' standard and out of scope for PESI.

Botanico-periodicum-huntianum and its supplement.

There is one standard for the original publication and one for the supplement. These are books that provide standard abbreviations for 12,000 journals dealing with plants and approximately 12,000 non-standard abbreviations for those same titles found in other works. Abbreviations appear to be freely available through the Harvard University Herbaria http://asaweb.huh.harvard.edu:8080/databases/publication_index.html.

Relevance to PESI: This is beyond the scope of PESI. It is assumed the source databases will follow best practise and use abbreviations from these publications where it is appropriate.

Index Herbariorum. Part I: The Herbaria of the World

A book giving standard abbreviations for herbaria and ratified in 1990. This work is on-line by New York Botanic Gardens at http://sweetgum.nybg.org/ih/.

Relevance to PESI: PESI is unlikely to need to reference herbaria directly at this stage. It is therefore out of scope.

International Transfer Format for Botanic Garden Plant Records

A non-XML based transfer format ratified in 1987 that has been widely used in botanic gardens community. Some of the controlled vocabularies within ITF2 have been adopted as lookup tables in curation databases.

Relevance to PESI: Out of scope for PESI.

Floristic Regions of the World

A book ratified in 1986 of floristic regions of the word. This work could be useful if it was made available in an electronic format.

Relevance to PESI: Out of scope.

User's Guide to the DELTA System

DELTA, ratified in 1986, is a description Language for Taxonomy that has been widely implemented. DELTA has largely been superseded by SDD and proprietary formats. It is very unlikely that this standard corresponds to any live system.

Relevance to PESI: Out of scope.

Taxonomic Literature, ed. 2 and its Supplements

A series of books containing "A selective guide to botanical publications and collections with dates, commentaries and types". Now available on-line (http://tl2.idcpublishers.info/) by subscription or free to IAPTA members.

Relevance to PESI: Out of scope.

Natural Collections Descriptions (NCD)

An emerging standard for the description of biological collections that resulted from a collaboration between the European Union SYNTHESIS and RAVNS. The standard is being developed as a XML Schema that is integrated with the LSID Vocabularies.

Relevance to PESI: Out of scope for PESI

TDWG Access Protocol for Information Retrieval (TAPIR)

TAPIR is a unification of the BioCASe and DiGIR protocols that is being rolled out across both the BioCASE and DiGIR networks. Full implementations of the protocol can support custom response formats. TAPIR is capable of serving RDF that uses the TDWG ontology. A specification will be submitted to the standards process in the near future.

Relevance to PESI: It may be appropriate to provide access to some PESI data via a TAPIR end point. This should only be done once consumers of the service and testing methods have been identified.

Life Science Identifiers (LSID)

Life Science Identifiers have been proposed as the preferred GUID technology for the key objects within the TDWG domain. A standard is in preparation that specifies how the LSIDs should be applied within the biodiversity informatics domain. As LSIDs are an OMG standard, TDWG’s recommendations will take the form of an Applicability Statement.

Relevance to PESI: There is currently some debate concerning the adoption of LSIDs across the community. This debate is likely to come to a head at the e-Biosphere conference in June 2009 after which it will be clear whether they are the preferred GUID technology for PESI. This does not detract from the necessity of PESI to provide Globally Unique Identifiers of some form.

Open Archives Initiative Protocol for Metadata Harvesting (OAI-PMH)

The primary use of DiGIR and BioCASe providers is to expose data sources for harvest by caching indexers such as GBIF. The same outcome can be achieved using a simpler protocol from the wider community such as OAI-PMH. This was accepted as a strategy in the original TAG1 meeting but did not appear in the 2006 Roadmap document. A test service has now been implemented using the TDWG Ontology as the metadata format and as part of the TAPIR.NET software.

Relevance to PESI: If PESI identifies users who would exploit an OAI-PHM service then one should be enabled. The rate of change of data may not justify this though.

Nexus/NeXML

Nexus is the main file format for exchanging data between phylogenetic analysis and display programmes. NeXML is propose as an XML based replacement for Nexus. Some work has been done on integrating NeXML with semantic technologies.

Relevance to PESI: As a taxonomy provider PESI should examine how users of phylogenetic data can semantically mark up NeXML files with taxonomic information from PESI. This should only done if NeXML appears to be gaining widespread adoption.

Appendix B: Plan for Implementation of GUIDs in PESI

PESI will take the Linked Data approach to sharing its content using GUIDs. Linked data has been defined as: "*a term used to describe a recommended best practice for exposing, sharing, and connecting pieces of data, information, and knowledge on the Semantic Web using URIs and RDF.*"

Specifically Tim Berners-Lee has articulated the linked data paradigm as involving four key principles (as paraphrased on wikipedia)

- Use URIs to identify things that you expose to the Web as resources.
- Use HTTP URIs so that people can locate and look up (dereference) these things.
- Provide useful information about the resource when its URI is dereferenced.
- Include links to other, related URIs in the exposed data as a means of improving information discovery on the Web.

Task 1: The primary resources that PESI will expose to the web are taxa, arranged into a hierarchical classification. PESI should therefore identify these taxa with persistent HTTP URIs that can be used by other projects to reference them unambiguously. These HTTP URIs should resolve to useful information that, after content negotiation, should be presented either as a web page or an RDF document.

Task 2: The useful data should contain links, in the form of other HTTP URIs, to other sources of data. There isn't a fixed list of links but the more links there are the better. Candidate data sets to link to are:

- TDWG controlled vocabularies for ranks
- Nomenclators holding name data
- Geographic regions repository (possibly holding PESI generated regions)

Implementation Burden: Production of RDF in response to HTTP URI calls is relatively trivial. Adopting a linked data approach does not imply supplying Semantic Web search services such as SparQL end points.

Financial Aspects: Support for linked data publishing is no more financially onerous than support for a minor feature on an existing website. Provided PESI is maintaining a web presence support for linked data should not add a significant additional burden.

Administrative Aspects: Everyone involved in decision making around PESI needs to appreciate that maintaining persistent HTTP URIs and linking to other data sources using them is core to enabling machine access to the data published by the system. Beyond this there is no increase in administrative burden.

Risks: The Linked Data movement is relatively new but is only an application of already widely used technologies (hence the low implementation burden). There are three main threats to this approach:

1. HTTP URIs are rejected as persistent identifiers in favour of LSIDs or some other technologies. This is mitigated by the fact that it would be very simple to layer LSIDs over HTTP URIs because LSIDs basically only offer services description mechanism that results in a call to and HTTP URI for metadata.
2. The metadata should be returned for a taxon is not clearly defined in the community and is therefore likely to change through time. Because of the

inherent flexibility of RDF it should be possible to modify the data that is returned as consensus is reached. This will result in some on-going maintenance burden up to this point.

3. The HTTP URIs used must be stable and persistent so that if they are embedded in other peoples' data they will work at a later data. If the HTTP URIs contain an element of branding, such as “PESI”, there is a danger that they will become out of data as far as the supporting project is concerned. Everyone concerned needs to be educated on the need for persistent HTTP URIs and the structure of these URIs needs to be chosen carefully so as to fit with future technologies.

There are a series of tutorials available covering some of these issues on the Linked Data site.

Configuration History			
Version No.	Date	Changes made	Author
0.1	03-04-2009	First draft for circulation within WP4	RDH
0.2	09-04-2009	Added appendix A and expanded Phase II	RDH
0.3	23-04-2009	Addressed minor comments from CGH and added more links	RDH
1.0	06-05-2009	Incorporated comments from YdJ and WA	RDH
1.1	20-05-2009	Minor changes after final circulation for comment	RDH
2.0	14-09-2009	Version for re-submission following 1st Year Review	RDH
2.1	22-09-2009	Minor editorial changes	YdJ & JK
