# Supplementary material for: PESI - a taxonomic backbone for Europe
Source: Biodivers Data J. 2015 Sep 28;(3):e5848. doi: 10.3897/BDJ.3.e5848 (PMC4609752; doi:10.3897/BDJ.3.e5848)
Supplement: Supplementary material 12 — Report on Procedures and Mechanisms for the functioning of Nomenclators within the e-Infrastructure [file biodiversity_data_journal-3-e5848-s012.pdf]

D4.2 — Report on Procedures and Mechanisms for the functioning of Nomenclators within the e-Infrastructure

Contents

1 Introduction 1
2 The Difference Between Nomenclature and Taxonomy 1
3 The Key Difference Between Nomenclatural Codes. 4
4 The Role of Nomenclators in General. 5
5 Existing Nomenclators and Taxonomic Databases 6
6 The Role of Nomenclators in PESI 8
7 Strategy for Linking Nomenclators and Taxonomic Databases within Europe and Globally 9
8 Glossary 10
9 References 11

1 Introduction

This report constitutes deliverable D4.2 of Work Package 4 (WP4) of the Pan-European Species-directories Infrastructure (PESI). It establishes the difference between nomenclature and taxonomy, discusses differences between the different nomenclatural codes, identifies a role for nomenclators within PESI and specifies a strategy for integration of nomenclators and taxonomic databases.

2 The Difference Between Nomenclature and Taxonomy

A name is a language unit (word or words) by which a “thing” is known. When two people communicate it is assumed that the names used by one person apply to the same things as when they are used by the other person. If the definition of the words used by two people are incompatible then communication breaks down. Dictionaries are compiled to facilitate unambiguous communication. They provide two key functions:

1. **A recognised word list.** This enables users to clearly establish that they are **using the same words** by clarifying issues such as spelling and homonyms.
2. **A mapping between words and their meanings.** In the case of names the dictionaries provide descriptions of the things for which the names are used. This enables users to clearly establish that they **mean the same things by using those words.**

Scientific names of organisms are like any other names - they are language units by which things are known. Because there are a large number of scientific names and because, for most groups of organisms, any scientist can invent a new name or redefine an existing one there is much complexity associated with their use and a series of nomenclatural codes have arisen as a codification of the accepted best practise. Major

advances were made around the turn of the twentieth century when the International Commission on Zoological Nomenclature was founded (1895) and the International Botanical Congress published the “International rules of Botanical Nomenclature” (1905). Today there are a number of nomenclatural codes and proposed replacement codes including the ICBN (McNeill et al. 2006), ICZN (Ride and International Commission on Zoological Nomenclature. 1999), ICNCP (Brickell and International Society for Horticultural Science.;International Commission for the Nomenclature of Cultivated Plants. 2004), ICNB (Lapage S and International Union of Microbiological Societies.;International Union of Microbiological Societies. 1992), PhyloCode (Queiroz 2006). Of these by far the most significant for the majority of scientists are the ICBN and the ICZN. Discussion here is restricted to these two codes because of their significance but it should be noted that these codes probably govern the names of a minority of organisms. This is because the majority of the organisms on earth are bacteria, whose names would be governed by the ICNB which is currently beyond the scope of PESI.

Under the ICBN and the ICZN every scientific name that is validly published at the rank of species and below is directly bound to a preserved type specimen (which, in some instances, may be substituted by an illustration) that is stored in a reference collection. Names above the rank of species are indirectly bound to type specimens via designated type species. The type specimens bind the names used in the literature into the biological reality found in the reference collections.

The correct name for any circumscribed taxon is calculated from the first published name whose type specimen is included within the circumscription of that taxon, taking into account the placement of the taxon within a particular classification and the names in use for other taxa in that classification (the nomenclatural rule of priority ICZN Article 23 & ICBN Article 11). If there are no suitable names bearing type specimens within the circumscription then a new name is published and a type specimen assigned to it.

There are many complex rules concerning matters such as binomial names (ICZN Article 5 and ICBN Article 23.1), missing type specimens (ICZN Article 74 and ICBN Article 9.9) and publication of duplicate names (ICZN Article 52 and ICBN Article 53.3). The fact that names are determined via an algorithm based on name-bearing types is particularly important to this discussion (ICZN Article 61 and ICBN Article 7).

Figure 1: Dissimilar taxa (from different classifications) can share a single unqualified name.

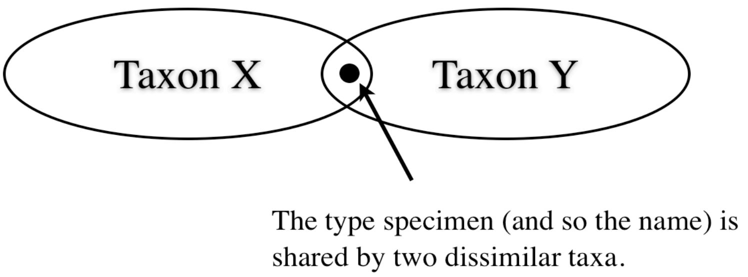

The nomenclatural codes are chiefly concerned with the first function of the dictionary above – to provide a recognised list of correctly spelled words. Unfortunately, because the names for organisms are linked to type specimens it is possible for radically different taxa to have the same name. Two taxa need only share a single specimen – the type – to have the same name (Figure 1). The converse is also possible – two taxa could share all specimens except for the name bearing types and minor changes in taxon circumscription between classifications can lead to disproportionate changes in

nomenclature (Figure 2). It is also possible for a taxon to change its name without changing its circumscription – for example, if it is discovered to include an older name's type. Scientists who cite names in their work therefore do not precisely cite the taxa to which their observations pertain. To precisely cite taxa they should include details of the taxonomic treatment they are employing as well as the name.

This separation between the notions of a name and the taxon to which that name refers is now widely accepted and referred to as the Taxon Concept or Potential Taxon problem (Pullan et al. 2000; Berendsohn 1995; Kennedy et al. 2006 and works cited therein).

In order to facilitate the accurate exchange of taxonomic information, both within the taxonomic community and more widely in the biological and environmental sciences, the e-infrastructure needs to provide the two dictionary functions described above for scientific names of organism i.e.

1. **A recognised list of the names used.** To establish that any two studies are actually using the same names whilst accounting for spelling variants and homonyms as well as to facilitate consistency in spelling and presentation. The term 'nomenclature' refers to this function.

A mapping between the names and descriptions of the taxa they are used for. To establish that any two studies are using the names in the same sense or compatible senses. The term 'taxonomy' refers to this function.

Figure 2: A slight change in circumscriptions moves the type of Taxon B into Taxon A. In the revised classification A has B's name and B has a new name.

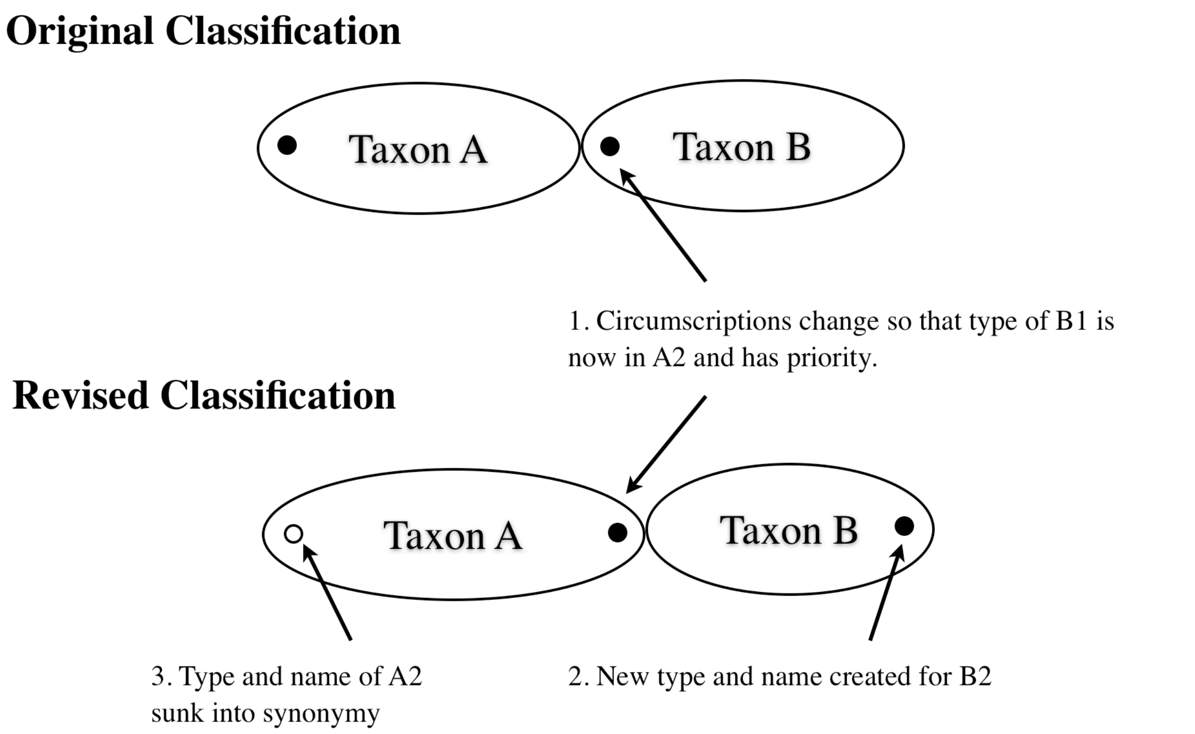

3 The Key Difference Between Nomenclatural Codes.

The current proposed scope of PESI is covered entirely by ICBN (plants, algae and fungi) and ICZN (all animals). The term 'algae' does not represent a natural group and some of the members of this group are biologically bacteria (Cyanobacteria or Cyanophyta) but for historical reasons their names are governed by the ICBN instead of the ICNB.

The ICBN and ICZN codes are very similar in many respects but have one key conceptual difference. When a botanist publishes a species name, they do so within a particular genus and thus create a binomial. If another botanist subsequently moves the species to a different genus as part of a revision they must publish the name as a new combination of specific epithet and new genus name.

If a zoologist publishes a species name it is placed within a genus and the genus name forms part of the initial binomial for that name just like in botany, but if another zoologist believes the species is better placed in a different genus they simply use the new combination of genus and species name. There is no requirement for formal publication of new combinations.

In both botany and zoology there is a convention to place the name of the original author of the species name in brackets after the name when it is used in combination with a genus other than the one it was first published in.

Botanists will always consider a species name to be a binomial whilst many zoologists will consider a species name to be only the specific epithet and author string. They consider the combination of the name with different generic names as merely 'usages' of the name. This can lead to confusion when discussing botanical and zoological names together. It is further confused by the fact that, although zoologists do not formally track the usages of specific epithets in different genera, should it be discovered that two epithets of different origin (having different types) are being used in the same genus, and thus causing homonymy, the one that was first used in the genus has priority over the second one. It is therefore necessary to retrospectively track down the first usage as if it had been formally published. A zoological nomenclatural system therefore has to act as if it were a botanical system in tracking all the unique combinations of genus and specific epithet names even though there is no requirement in the code for formal publication of these names.

4 A Case for Name Registration

If the ICBN and ICZN codes required all names to be registered in a single or limited number of places, then this would effectively fulfil the first function of a biological names dictionary for the e-infrastructure. Unfortunately neither the ICBN or ICZN codes require names to be registered. Neither do they require names to be published in a particular list of journals. They merely set out the conditions for effective publication. The publications in which new names appear could be published anywhere and deposited in any library. There is no requirement for them to be peer reviewed.

Both codes require that the oldest name be used for a taxon yet provide no mechanism for ascertaining if the oldest name has been found. This means that any revisionary treatment of a group has the potential to destabilise the names in current use by uncovering older publications.

Although continuously updated, the codes are still focused on a world of paper publication with a relatively small number of journals in each specialist field. The codes

are run on the basis of consensus building and, because the need for registration is not yet universally recognised, no such registers have been established. ICZN is in the process of attempting to establish one in the form of ZooBank. A register for ICBN was proposed (Tokyo 1994) but rejected (St Louis 2000).

In contrast to these attempts, the Bacteriological Code (ICNB) has an effective registration system that has been in place for nearly thirty years. All bacteria names have to either be in the Approved Lists of bacteriological names published in 1980 or published in the Journal of Systematic and Evolutionary Microbiology.

Unless some external body forces, and perhaps funds, the ICBN and ICZN to establish effective registers of names, the community must build and maintain separate databases of nomenclatural data. These are the nomenclators. One way in which a register of names may come about is via collaborative efforts that are now being proposed between nomenclators to build a Global Names Architecture (see below).

5 The Role of Nomenclators in General.

When a nomenclatural database or a taxonomic database representing nomenclatural information is formally or functionally accepted by a community as common (single) reference point or controlled vocabulary (e.g. correct spelling of taxon names) for regulating effective cross-linking this is usually called a nomenclator. It provides the primary function of a dictionary as defined above - **a recognised word list.**

Nomenclators are not intended to provide the secondary function of the dictionary - **a mapping between words and their meanings** – although they may provide some of this information incidentally.

Nomenclatural information consists of acts governed by the respective codes of nomenclature. Most of these acts are ‘original descriptions’ of new scientific names, but other acts may include emendations, lectotypifications, and other acts as governed by the codes. Synonymy is not included in these lists as taxonomic concept, but only as newly established combinations (for botanists) or usages (for zoologists) linked to a basionym.

Other kinds of data objects linked to the registration of nomenclatural acts may also be included, like:

1. Publications that contain Nomenclatural Acts (as defined above)
2. Names of Authors of the relevant publications
3. Type specimen allocations

Nomenclators are often referred to as sources of **objective** information in that they provide factual lists of what has been published and how this should be interpreted under the appropriate nomenclatural code. This is in contrast to synonymised checklists, monographs, floras, faunas and mycotas which all present expert opinions as to which taxa are real and therefore which names should be used and which names should be treated as synonyms and not used.

No nomenclatural database is an absolutely 'pure' nomenclator. They all contain or imply some information regarding taxonomy, if only for the sake of data navigation. It is useful, for example, to know whether a name is used for a monocot or a dicot when dealing with plant names even though this has no bearing on nomenclature.

6 Existing Nomenclators and Taxonomic Databases

There are a large number of taxonomic and nomenclatural databases available having a varying degree of nomenclatural detail or taxonomic content. They include:

- Index Fungorum (IF), an attempt to list the names of all fungi managed as a partnership between CABI, Centraalbureau voor Schimmelcultures (CBS) Fungal Biodiversity Centre and Landcare Research. IF includes links to subjective taxonomic synonymy (in Species Fungorum) as well as objective nomenclatural synonymy. Fungal names are governed by the ICBN. IF is acting as a key player in establishment of the Global Names Architecture (GNA) and is a PESI partner.
- International Plant Names Index (IPNI), an attempt to list all the names used for vascular plants in the world. The records in IPNI come from three principle sources: the Index Kewensis (IK), the Gray Card Index (GCI) and the Australian Plant Names Index (APNI) as well as Index Filicum. IPNI includes taxonomic placement of names at family level for management purposes but doesn't otherwise include taxonomic opinion. IPNI is a PESI partner.
- AlgaeBase, a database of information on algae that includes terrestrial, marine and freshwater organisms. AlgaeBase includes taxonomic placement of names along with images and other data. Most of the names in AlgaeBase are governed by the ICBN. AlgaeBase is a PESI partner.
- Index Nominum Genericorum (ING), a collaborative project of the International Association for Plant Taxonomy (IAPT) and the Smithsonian Institution, was initiated in 1954 as a compilation of generic names published for all organisms covered by the International Code of Botanical Nomenclature.
- uBio, is an initiative within the science library community to join international efforts to create and utilize a comprehensive and collaborative catalogue of known names of all living (and once-living) organisms. It is essentially an aggregator creating a highly organised index of organism names and classifications. uBio aims to reflect what is found in the literature rather than be an authoritative source.
- Index Animalium by Charles Davies Sherborn was a standard text for Zoology for a long time and has now been digitised and indexed by uBio.
- Index Nominum Algarum The INA is a part-digitized card file maintained by Paul Silva at the Herbarium of the University of California. It contains nearly 200,000 names of algae (in the broad sense).
- Index Nominum Supragenericorum Plantarum Vascularium a joint effort between the International Association for Plant Taxonomy, the University of Maryland, and now Cornell University. The purpose of the project is to capture all valid and legitimate extant vascular plants names, as defined by the International Code of Botanical Nomenclature, proposed above the rank of genus. At any one time, the listing of a name means only that it is the earliest, valid place of publication found to date.
- Mycobank MycoBank is an on-line database aimed as a service to the mycological and scientific society by documenting mycological nomenclatural novelties (new names and combinations) and associated data, for example descriptions and illustrations. The nomenclatural novelties are each allocated a unique MycoBank number that is cited in the publication where the nomenclatural novelty is introduced; MycoBank number are cited for 65% (and increasing) of nomenclatural novelties in the fungi. These numbers are also used by Index Fungorum, with which

MycoBank is associated, and serve as the terminal part of Life Science Identifiers (LSIDs) issued by Index Fungorum.

- Nomenclator Zoologicus is a continuous record of the bibliographical origins of the names of every genus and subgenus in zoology published since the 10th ed. of Linnaeus' Systema Naturae in 1758 up to 1994 in nine volumes. Names are listed alphabetically, with a bibliographic reference to the original description of each one and an indication of the animal group to which it belongs. There are an estimated 340,000 genera represented in the text as well as approximately 3000 supplemental corrections. In 2003, uBio received permission from the Zoological Society of London to undertake the digital conversion of Volumes 1-9 of Nomenclator Zoologicus. This has now been completed, following funding in 2004 to uBio from the Global Biodiversity Information Facility and the Andrew W. Mellon Foundation.

- ZooBank is intended as the official registry of Zoological Nomenclature, according to the International Commission on Zoological Nomenclature (ICZN). It is currently in prototype phase and contains a limited number of names. ZooBank is acting as a key player in establishment of the Global Names Architecture (GNA) and is being supported in its endeavours by PESI in collaboration with the ICZN. On way in which PESI will assist is through backwards uploading of taxonomic names from PESI data sources to Zoobank.

- Catalogue of Life **(CoL)** A partnership between Species 2000 & ITIS, Catalogue of Life is planned to become a comprehensive catalogue of all known species of organisms on Earth. The ninth edition of the Annual Checklist, contains 1,160,711 species. This is probably around two-thirds of the world's known species. This means that for many groups it continues to be deficient, and users will notice that many species are still missing. Catalogue of Life is not a nomenclator, but like PESI presents a consensus taxonomic checklist. However, in contrast to PESI, CoL is not a primary taxonomic resource, contains no (annotated) degree of nomenclatural details, and has a global scope (therefore is incomplete at the regional level). Nevertheless CoL contains in excess of two million names (including synonyms).

- NCBI Taxonomy The National Center for Biotechnology Information is a US national resource for molecular biology information. It maintains a biological taxonomy for data management purposes. Although the taxonomy contains a clear warning that it is not authoritative: "Disclaimer: The NCBI taxonomy database is not an authoritative source for nomenclature or classification - please consult the relevant scientific literature for the most reliable information." it is still frequently used as a reference taxonomy. It contains in excess of 330,000 taxa.

- Wikipedia is a successful collaboratively written web encyclopaedia. It contains entries for a growing number of taxa along with mechanisms to annotate taxonomic and nomenclatural details for these taxa. It is anecdotally reported to have the best coverage of any taxonomic resource in terms of descriptions, images and links. Taxon pages contain "taxobox" templates (example here) that help structure the data. A companion wiki based project, Wikispecies, has around two hundred thousand nomenclatural and taxonomic entries that lack descriptions.

- Encyclopedia of Life (EoL) aims to be an online reference and database on all 1.8 million species currently known to science and will stay current by capturing information on newly discovered and formally described species.

- Global Names Architecture (GNA) is a collaborative approach on developing a common solution for creating a complete and integrated taxonomic framework for all

names coordinated by GBIF-ECAT. GNA doesn't exist yet in a full shape, but some of the components are being developed, like the Global Names Usage Bank (GNUB) or are available in 'beta' version, like the Global Names Index (GNI). The GNA has been proposed as a unified global system of biological nomenclature. It will consist of both registers of names and indexes to usages of those names. Although a distributed system, it would provide a single contact point for all biological nomenclature.

- A Pan-European Species-directories Infrastructure (PESI). The PESI data set is built on the pan-European checklists Fauna Europaea (FaEu), Euro+Med PlantBase (E+M) and the European Register of Marine Species (ERMS), which contain complete, high quality, primary taxonomic resources of European species. The PESI data set is supplemented with additional taxonomic and nomenclatural resources, like Index Fungorum and AlgaeBase, to compensate for missing groups. Because of the high degree of nomenclatural detail within the used resources, the PESI data set could be considered a regional nomenclator in that by necessity it must contain accurate nomenclatural data for all the taxa it recognises (see below).

7 The Role of Nomenclators in PESI

PESI is envisaged as an annotated checklist of European organisms (see WP4.1 "Report on authoritative taxonomic standards from multiple sources suitable for deployment within European Research Area."). As such it is principally a taxonomic resource – providing a standard classification for the region rather than a nomenclatural resource, but any taxonomic resource of this significance must have the "correct" nomenclature for each taxon it recognises. It can therefore also act as nomenclator for the taxa that occur in the region.

If there were registers for taxonomic names, then ensuring nomenclatural correctness would simply be a matter of referring to the registers for each taxon name used – possibly by using the GUIDs they have issued. The registers would be authoritative. Unfortunately there are no registers and so there is no notion of a normative version of names data. The correct citation of every name has to be worked out manually using the rules of the appropriate codes. This comes with the danger that other databases with which PESI wishes to become integrated may interpret the rules of the codes differently and therefore have different name strings.

There is hope that the nascent Global Names Architecture (GNA) will provide such register-like services in the future and there is an opportunity for PESI to play a role in helping develop the GNA. There is also a risk. The GNA is a highly ambitious project, and currently unfunded, so may prove a distraction from PESI's core activity of building an annotated checklist.

Bearing in mind that names do not uniquely identify taxa, and that the primary purpose of PESI is to build a standard list of annotated **taxa**, the recommended approach to take is as follows.

- Identify all taxa within PESI using a unique opaque identifier that can be used externally as part of a GUID. This is the approach taken by Integrated Taxonomic Information System (ITIS) for North America with its Taxon Serial Numbers (TSN) and more widely by Catalogue of Life (CoL) with its LSIDs (CoL incorporates large parts of ITIS).

- Treat names as metadata on taxa and therefore as peripheral to the process of building the checklist. The name data must be correct but minor changes to

nomenclatural details are of no greater significance than minor changes to distribution maps - because it is the GUID, rather than the name, that identifies the taxon.

- All names that occur in the literature and in existing taxonomic and nomenclatural databases still have to be accounted for but this is outside the scope of PESI, which only has to map names coming from its data providers to currently accepted taxa.

In short, PESI should take a taxon-centric rather than a name-centric approach. There are therefore two roles that nomenclators can play in PESI:

- Provide new names that may have been used for taxa that occur in the PESI region.
- Provide additional nomenclatural data (correctly-formed authorities, place of publication, etc) about existing names in PESI.

PESI has an evolving relationship with the nomenclators as they move towards acting as authoritative registers within the GNA. The flow of data will be bidirectional with PESI contributing as much as it receives. This symbiosis is one of the valuable aspects of PESI; because this reciprocity allows for instance cross-validation and cross-referencing to be done in a single effort.

8 Strategy for Linking Nomenclators and Taxonomic Databases within Europe and Globally

The above discussion illustrates the complexity of the realm of nomenclators and taxonomic databases into which PESI is being launched. There is an opportunity here for PESI to act as a catalyst for integration, rather than adding to this complexity. By doing this PESI will facilitate the wider e-infrastructure.

If all taxonomic and nomenclatural services were to implement similar interfaces to their data and, where possible, link to each other, it would be possible to build generic applications that can exploit multiple resources.

PESI will be a significant resource within the global e-infrastructure and the PESI WP4 partners represent four influential data sources (AlgaeBase, Index Fungorum, IPNI and ZooBank). If the five data sources act in unison they may have enough critical mass to instigate adoption of a single interface across all taxonomic and nomenclatural data sources.

A six-stage strategy will therefore be adopted:

- **Design** a suitable interface to taxonomic data. This will be based on the proven TDWG Taxon Concept Schema in RDF and the use of HTTP URIs. Completed by the annual TDWG meeting in November 2009.
- **Document** the interface in a clear and accessible manner. Completed by the annual TDWG meeting in November 2009.
- **Implement** the interface within PESI WP4 partners. Completed during first half of 2010.
- **Promote** the implementation of the interface by other providers. An on-going process, to be begun at the TDWG conference November 2009.
- **Encourage** development of applications that exploit data published using the

interface; perhaps with demonstration applications, or by issuing a funded "challenge" to developers. Can be started once there are more than two sources implementing the interface and linking to each other. Estimated start date of second quarter 2010. Culminating at TDWG 2010 annual meeting in fourth quarter 2010.

- **Standardise** the interface across a web of resources. Begin after 2010 if/when interface has been demonstrated to work. May extend beyond PESI's initial funding and can be carried forward by the implementing databases.

Design and documentation of the interface are crucial to the success of this project. It must be:

- **Conceptually simple**. It has to be understood by all providers and potential consumers of data.
- **Non-restrictive.** Providers must be able to implement the interface without compromising their own systems, either technically or from the point of view of data integrity. Providers will need some flexibility and shouldn't be shoehorned into adopting the same world view.
- **Easy to implement**. Must use standard technologies understood by any web developer. As simple as publishing or altering an existing webpage.
- **Provide a solution to the specific problem** "How are the data in the different taxonomic and nomenclatural resources related?"
- **Able to facilitate** the development of tools, rather than providing a single fixed solution.

The Linked Data paradigm offers the current best practice approach to build such flexible webs of resources and will be the one adopted (see below). Adopting such an approach will effectively add all participating resources to the Semantic Web thus linking them into a far wider constituency than just the biodiversity informatics community.

The web of taxonomic and nomenclatural resources, provided by the PESI Portal and through its partners, will be PESI's technical contribution to the development of a Global Names Architecture. This will be reported on in the WP4 deliverable D4.4 "Report on the contributions to the set up of a Global Name Architecture".

9 Linked Data

"Linked Data" is a recommended best practice for exposing, sharing, and connecting pieces of data, information, and knowledge on the Semantic Web using URIs and RDF. Tim Berners-Lee gave a TED Talk as a very high level explanation of the what Linked Data is and how it fits with the Semantic Web.

The basic concept is that every data resource, and indeed every real world object of interest, should be given an HTTP URI as a web name. When these HTTP URIs are looked up on the web they give back useful information. If the user is human they get data back in the form of a web page or other human readable resource. If user is a machine they get data in RDF format. Importantly the data contains more links to other data objects thus building a global web of information objects just as the World Wide Web today is a web of documents.

The Linked Data uses well established standard technologies, principally HTTP and RDF. It is widely documented with tutorials and validation mechanisms.

From the point of view of PESI the advantages of the linked data approach are:

- It is easy to implement at a technical level because it uses standard web technologies all developers are familiar with.
- It should be easy to write client applications because the data resolution mechanisms are built into all web aware devices.
- If more complex technologies, such as LSIDs, are required in the future they can easily be layered on top of HTTP URIs.

10 Glossary

Basionym The first binomial (or trinomial) that is associated with a type specimen, the epithet of which is subsequently used in another genus as part of any published revision.

Circumscription In taxonomy, circumscription is the definition of the limits of a taxonomic group of organisms.

GUID (Globally Unique Identifier) The term GUID is used in two slightly different ways. In computer science GUIDs are values that are complex strings of characters that are extremely likely to be unique in any context. In the biodiversity informatics community the term is used in a narrower sense. In this sense GUIDs have three related properties. They are not only globally unique; they are also resolvable (or actionable) and identify a typed object. The addresses of web pages that are displayed in the location bar of web browsers are good examples of resolvable GUIDs.

ICBN International Code of Botanical Nomenclature

ICNB International Code of Nomenclature of Bacteria

ICNCP International Code of Botanical Nomenclature for Cultivated Plants

ICZN International Code of Zoological Nomenclature

ITZN International Trust for Zoological Nomenclature – the governing body of the ICZN

Lectotypification The process of nominating a new type specimen for a name when the original type has been lost or was not clearly specified.

Linked Data A term used to describe a recommended best practice for exposing, sharing, and connecting pieces of data, information, and knowledge on the Semantic Web using URIs and RDF.

LSID (Life Science Identifiers) A kind of GUID. LSIDs were first proposed by Object Modelling Group and IBM. After several workshops TDWG adopted LSID as its preferred GUID technology. They provide uniqueness, resolution and response typing. The default resolution mechanism is based on special DNS SRV entries but there are very few clients that exploit the mechanism.

Nomenclator(s) An inventory of (biological) names

Nomenclaturator A body that provides a nomenclator service

Nominal Taxon A concept of a taxon which is denoted by an available name (e.g. Mollusca, Diptera, Bovidae, Papilio, *Homo sapiens*).

PhyloCode Phylogenetic nomenclature is an alternative to rank-based nomenclature, applying definitions from cladistics (or phylogenetic systematics). The PhyloCode is a developing draft for a formal set of rules governing phylogenetic nomenclature.

RDF (Resource Description Framework) A World Wide Web Consortium specification for a metadata model and component in the proposed Semantic Web.

Taxon (pl. taxa) A taxonomic unit, whether named or not: i.e. a population, or group of populations of organisms which are usually inferred to be phylogenetically related and which have characters in common which differentiate the unit (e.g. a geographic population, a genus, a family, an order) from other such units. A taxon encompasses all included taxa of lower rank and individual organisms. (after ICZN definition)

TDWG Originally the Taxonomic Databases Working Group now known as "TDWG (Biodiversity Information Standards)" is the principle organisation for ratification of standards in biodiversity informatics.

URI (Uniform Resource Identifier) A string of characters used to identify or name a resource on the Internet.

11 References

Berendsohn, Walter G. 1995. "The concept of "potential taxa" in databases." *Taxon* 44:207-212.

Brickell, Christopher, and International Society for Horticultural Science.; International Commission for the Nomenclature of Cultivated Plants. 2004. *International code of nomenclature for cultivated plants : (I.C.N.C.P. or cultivated plant code) : incorporating the rules and recommendations for naming plants in cultivation*. 7th ed. Leuven Belgium: International Society for Horticultural Science.

Kennedy, J.B., R. Hyam, R. Kukla, and T. Paterson. 2006. "Standard Data Model Representation for Taxonomic Information." *OMICS: A Journal of Integrative Biology* 10:220-230.

Lapage S, P, and International Union of Microbiological Societies.; International Union of Microbiological Societies. 1992. *International code of nomenclature of bacteria, and Statutes of the International Committee on Systematic Bacteriology, and Statutes of the Bacteriology and Applied Microbiology Section of the*. 1990th ed. Washington D.C.: Published for the International Union of Microbiological Societies by American Society for Microbiology.

McNeill, J et al., eds. 2006. *International Code of Botanical Nomenclature (Vienna Code)*. International Association for Plant Taxonomy.

Pullan, M.R., M.F. Watson, J.B. Kennedy, C. Raguenaud, and R. Hyam. 2000. "The Prometheus Taxonomic Model: a practical approach to representing multiple classifications." *Taxon* 49:55-75.

Queiroz, K. 2006. "The PhyloCode and the distinction between taxonomy and nomenclature." *Syst Biol* 55:160-2.

Ride, W, and International Commission on Zoological Nomenclature. 1999. *International code of zoological nomenclature*. 4th ed. London: International Trust for Zoological Nomenclature c/o Natural History Museum.

Configuration History			
**Version No.**	**Date**	**Changes made**	**Author**
0.1	07-08-2009	Early draft between Charles Hussey and RDH	RDH
0.2	29-08-2009	Corrections by CGH	CGH
0.9	31-08-2009	Circulation within WP4	RDH
0.95	14-09-2009	Corrections by CGH, LB, YdJ and others	CGH
1.0	15-09-2009	Version for submission	RDH
1.1	22-09-2009	Minor editorial changes	YdJ & JK
