# Supplementary material for: PESI - a taxonomic backbone for Europe
Source: Biodivers Data J. 2015 Sep 28;(3):e5848. doi: 10.3897/BDJ.3.e5848 (PMC4609752; doi:10.3897/BDJ.3.e5848)
Supplement: Supplementary material 13 — The future of taxonomy – the role of GSD-networks and nomenclators in taxonomic information infrastructure networks (GNOMA). [file biodiversity_data_journal-3-e5848-s013.pdf]

Project no. 018340

Project acronym: EDIT

Project title: Toward the European Distributed Institute of Taxonomy

Instrument: Network of Excellence

Thematic Priority: Sub-Priority 1.1.6.3: "Global Change and Ecosystems"

M3.2.5c The future of taxonomy – the role of GSD-networks and nomenclators in taxonomic information infrastructure networks

Global Nomenclator Architecture (GNOMA) meeting on the contribution of nomenclators to the Global Names Architecture (GNA)

ZooBank provisioning meeting on defining strategies for the uploading of ZooBank, especially on the contribution of taxonomic (zoological) key-resources

Due date of component: Month 28
Actual submission date: Month 29

Start date of project: 01/03/2006 Duration: 5 years

Organisation name of lead contractor for this component: 5 UvA

**Project co-funded by the European Commission within the Sixth Framework Programme (2002-2006)**		
**Dissemination Level ("X" in the relevant box)**		
**PU**	Public	
**PP**	Restricted to other programme participants (including the Commission Services)	
**RE**	Restricted to a group specified by the consortium (including the Commission Services)	X
**CO**	Confidential, only for members of the consortium (including the Commission Services)	

Revision 1

Table of Content

Table of Content 2
GNOMA meeting 3
GNOMA meeting agenda 3
GNOMA meeting logistics 5
GNOMA meeting attendees 6
GNOMA meeting summary 7
GNOMA meeting actions 17
ZooBank meeting 19
ZooBank meeting agenda 19
ZooBank meeting attendess & minutes 21
Previous meeting(s) 47
Document & presentations 47
Configuration History 47

Global Nomenclators Architecture Meeting

Supported by the Pan European Species Directories Infrastructure (PESI), the Global Biodiversity Information Facility (GBIF), and the European Distributed Institute of Taxonomy (EDIT)

DRAFT

Global Nomenclators Architecture (GNOMA)
Date: August 28 2008
Location: Sale the Baleine, Pavillion de la Baleine at the Jardin des Plantes at the Muséum national d'Histoire naturelle, Paris, France
Chairpersons: Paul Kirk

See GNOMA meeting Logistics
See GNOMA meeting Attendees

We need a Global Nomenclator Architecture ("GNOMA" - J.A.Cooper) – "an agreed usage of TCS to expose nomenclator data via TAPIR and via LSID mediated resolution of the appropriate TCS vocabulary content". It then needs GBIF (or some other resource') to provide a Name Resolution Service – for example so potential LSIDs can be returned from just a bunch of names. It could also manage any overlap between the nomenclators.

Agenda

0900-1230 - Introduction and discussion (the What)

Introduction and Rationale: Why do we need a coordinated and global nomenclatural infrastructure?

Perspectives from within the nomenclators. Paul Kirk and Jerry Cooper
External perspectives from other initiatives. David Remsen, Yde de Jong, others

Discussion:

What do the nomenclators want to do for GBIF, EoL ... anyone else ... in terms of automated services?
What standard methods and data elements should be supported?
What are the use cases that should be supported?
How much should be code independent/dependent

Coordination and Visibility
Common index of Nomenclators (GNI) as an internal or external index.
What subset of metadata should be supported?
What reconciliation and de-aliasing should this index support?
Needs some thought on how GSD and Checklist data can 'migrate' back down to the Nomenclators where there are identified conflicts (e.g. different orthography, different authors etc) – perhaps this could be managed by the Names Resolution Service in reverse – a name annotation service which the Nomenclators could then use to updata/augment content as appropriate.
Governance
Needs: Tools/Protocols/Formats
Demo of current GNI by Dmitry Mozzherin

All Genera Index - Update and demo by David Remsen

1230-1330 - Lunch

1330-1630 - Discussions

Other GNOMA Outputs and Services (various useful additions)
Name lexicons
Official lists / groups of names could be very useful for various name-finding algorithms and applications.
Author abbreviation services

> IPNI partners have plant data. Zoological author names are available from multiple sources for a start. Should a Author Resolution Service be a practical addition to GNOMA. What are the use cases for it?

Resolution of citations

> Someone needs to get to grips with bibliography data; I want some standard lists of core systematics journals with dates of publication for each volume-part and standardized Full titles and Abbreviated forms – Roger Hyam tried to kickstart this last year – I'm sure most of this is

already available – it just needs harvesting and working in to some useful form.

Populating the nomenclators - discovery of names NOT within the current nomenclators

How should unverified or even verified names originating outside of the nomenclators (for example the BDWD fly genera) be incorporated & discoverable within for example, the AGI or a common index and subsequently made available to the nomenclators.

Comprehensive classification of names

The plant and fungal nomenclators use a management classification. ZooBank will need some means to provide access to names that fall within taxonomic domains.

Could the GNOMA include a common names management framework for easier disambiguation of inter-regnal names and enhanced interoperability? (both among nomenclators and with other data management initiatives like BHL/GBIF who could also employ such a management classification as one method for organizing names-annotated data). The issue arose at the PESI workshop.

Should it go forward and how?

What options are available now?

More than one classification for access?

1630-1730 - Summary and Wrap up (the How)

Recommendations

Technical Requirements

Social Barriers

Financial Needs

Short term vs longer term

Outputs

What documentation should we target for the meeting outcome?

1730 Adjourn

PESI / GBIF /EDIT Workshops (GNOMA AND ZOOBANK DATA PROVISION)

Supported by the Pan European Species Directories Infrastructure (PESI), the Global Biodiversity Information Facility (GBIF), and the European Distributed Institute of Taxonomy (EDIT)

Facilitators: Yde de Jong (PESI, EDIT) and David Remsen (GBIF)

Global Nomenclators Architecture (GNOMA) - Thursday August 28, 2008, 0900
Chairs: Paul Kirk, CABI

ZooBank Data Providers - Friday August 29 0900
Chairs: Ellinor Michel (ICZN secretariat) and Richard Pyle (ICZN & Bishop Museum)
Minutes recorded by (TBD).

Venue Location:

Pavillion de la Baleine at the Jardin des Plantes at the Muséum national d'Histoire naturelle, 43 rue Cuvier. Download location map.

Two meeting rooms: GNOMA
Thursday, Aug 28th - 'Sale the Baleine' (max 15 people)
Friday, Aug 29th - 'Amphitheatre Rouelle' (max 50 people)

Both rooms will be available for the 28-29 August.

Meals provided (for lunch):

Thursday 28 August (GNOMA):
Entry: Roman salad
Main dish: Sliced chicken, with Parmesan cheese accompanied with a Caesar sauce
Dessert: Caramel cream

Friday 29 August (ZooBank Data Providers):
Main dish: Escalope of salmon accompanied with potatoes crushed in olive oil
Dessert: Chocolate mousse

Places to Stay

Hotel Best Western Latin Quartier (no longer available).

Alternative suggestion:

FIAP-Paris (Monet) centre

Hotel Libertel Austerlitz
12 Bd de l\'Hôpital
75005 Paris, France
Tel: +33 1 43 37 60 80
Fax: +33 1 42 40 64 51
http://www.hotel-austerlitz.com

Systema Naturae and the ICZ

Registration for the ICZ or Systema Naturae meetings/symposia are not required to attend the meetings. Participants wishing to attend the ICZ meetings will need to cover their own costs for the symposia.
The ICZ organisers created a one-day congress ticket, which can be bought on the site, costing 70 Euros for August 26, 27 and 28 and the double for 29th which includes the banquet. For more information please contact Jean-Marc Jallon.

GNOMA Attendees : Sheet1

	Global Nomenclators Architecture Attendees				
	**First**	**Last**	**Affiliation**	**Role**	**Status**
1	Alan	Paton	IPNI	Participant	provisional (likely)
2	Jerry	Cooper	Landcare Research	Co-Chair?	**confirmed**
3	Yde	de Jong	PESI, EDIT	Facilitator	**confirmed**
4	Paul	Kirk	CABI	Chair	**confirmed**
5	Pier	Kuipers	Algaebase	Participant	provisional
6	Chris	Lyal	NHM	Participant	confirmed
7	Richard	Pyle	ZooBank	Participant	**confirmed**
8	David	Remsen	GBIF	Facilitator	**confirmed**
9	Brian	Tindall	DSMZ	Participant	**confirmed**
10	Dave	Roberts	EDIT	Participant	**confirmed**
11	James	Macklin	Harvard (IPNI)	Participant	**confirmed**
12	Nicky	Nicholson	IPNI	Participant	provisional (likely)
13	Ellinor	Michel	ICZN	Participant	**confirmed**
14	Dmitry	Mozzherin	EoL	Technical Obs.	provisional (likely)
15	Bart	Vanhoorne	MarBEF, WORMS, PESI	Technical Obs	**confirmed**
16	Ward	Appeltans	MarBEF, WORMS, PESI	Observer	**confirmed**
17	Kehan	Harman	NHM	Rapporeur	**confirmed**

Deze pagina bewerken (als u toestemming heeft) – Gepubliceerd door Google Documenten – Misbruik rapporteren – Automatisch elke vijf minuten bijgewerkt

Global Nomenclators Architecture (GNOMA) Meeting

Date: August 28 2008
Location: Sale de la Baleine, Pavillion de la Baleine at the Jardin des Plantes at the Muséum national d'Histoire naturelle, Paris, France
Chairpersons: Paul Kirk
Agenda: Hosted on Google Documents
Supported by the Pan European Species Directories Infrastructure (PESI), the Global Biodiversity Information Facility (GBIF), and the European Distributed Institute of Taxonomy (EDIT).

Executive Summary

Attendees

Full list at http://spreadsheets.google.com/ccc?key=pdGw-1ZvQrab0T_5v-OLzPg&hl=en_GB

Name	Initials	Institution / Role
Ward Appeltans <ward.appeltans_at_vliz.be>	WA	Flanders Marine Institute: Belgium. Manager of WORMS.
Philipe Bouchet <>	PB	ICZN: ERMS, WORMS, Molluscan Generic names: 23,000 validated names.
Jerry Cooper <cooperj_at_landcareresearch.co.nz>	JC	Landcare Research in NZ. Works with PK on Index Fungorum. On Global committee of sp2000. Working on Global Checklist of Composites & Checklist of NZ Plants
Yde de Jong <yjong_at_uva.nl>	YJ	Facilitator. Chair of ECAT subcommittee.
Vanessa Demanoff <edit_at_mnhn.fr>	VD	Edit Project manager
Olivier Gardonomie <>	OG	MHNH Paris: head of national inventory of natural heritage (French equiv of GBIF). Involved in data Repatriation: French overseas territories.
Kehan Harman <kehanharman_at_gmail.com>	KH	NHM / GBIF: Minutes

Charles Hussey <c.hussey_at_nhm.ac.uk>	CH	NHM PESI & UK Species Dictionary
Paul Kirk <p.kirk_at_cabi.org >	PK	CABI, UK. Managing Index Fungorum (IF): Chair of GNA steering committee
Chris Lyal <c.lyal_at_nhm.ac.uk >	CL	Natural History Museum London (NHM): PESI/ UK Species Dictionary
James Macklin <jmacklin_at_oeb.harvard.edu >	JM	Harvard University Herbarium (HUH) Informatics. IPNI & also
Ellinor Michel <e.michel_at_nhm.ac.uk>	EM	Natural History Museum, London / ICZN Secretary.
Dmitry Mozzherin <dmozzherin_at_eol.org >	DM	MBL/EOL. Implementation of GBA & EOL
Alan Paton <a.paton_at_kew.org>	AP	Royal Botanic Gardens, Kew managing IPNI. Concentrating on content, not technical.
Richard Pyle <deepreef_at_bishopmuseum.org>	RP	Bishop Museum, Hawaii. ICZN Commissioner – Implementation of ZooBank
David Remsen <dremsen_at_gbif.org>	DRE	Facilitator.ECAT/uBio: Taxonomic Names Component. ECAT needs more structure.
Dave Roberts <workpackage6_at_googlemail.com>	DRO	NHM, London. WP6 Edit Project: Scratchpads & Wiki like sites
Brian Tindall <bti_at_dsmz.de >	BT	Chair on Prokaryotes commissio.
Bart Van Hoorn <bart.vanhoorne_at_vliz.be >	BV	WORMS
Miguel <>	M	MHNH Paris

Morning Session

Simon Tillier opened meeting with welcome from Edit followed by a welcome from David Remsen on behalf of GBIF. At the 2004 GBIF Nomenclators Meeting – there were verbal agreements about Global Nomenclator interactions but no tangible followup. David Remsen will give presentation on needs for big initiatives and smaller. This was followed by attendees intro themselves – details captured in the attendees list above.
David Remsen – some coordination required to pull initiatives together.
Paul Kirk – Nomenclators governed by different codes but they should be in a position to provide a framework upon which others can build. Nomenclators possibly being left behind – Index Fungorum (IF) was first nomenclator to provide LSIDs. Very little takeup of LSIDs since this has been set up which makes comparing datasets a nightmare. For example, recently worked with 21 European fungi red data lists trying to pull them together, and this had to all be done manually, whereas if they were all referring to the

IF LSID's then it would have been easier. Need to be able to at least link homotypic (Objective|Nomenclatural) synonyms together.
Jerry Cooper – Has access to IF & is also a user of it. Mycologists have a single clean nomenclator so very lucky. An example project – The Checklist of British Basidiomycetes - editing tool was developed to build this off of IF rather than Word Processing the text from scratch. He also uses IF as basis of merging other datasets. This requires a set of services which ground truths every record through to a nomenclator.
Phillipe Bouchet- Clarified with Paul Kirk that IF is just a nomenclator – homotypic names are linked together (Links to basionym). – Species Fungorum (SF) is a checklist with a taxonomic opinion. Note on definition Homotypic Synonyms == Objective Synonyms == Synonyms which all have the same type.
Yde de Jong – how many other checklists are linking to the nomenclator?
Paul Kirk – only 2 or 3 databases using web services. People should take a local cache copy and then keep in sync using the web services.

A common terminology

Rich Pyle – if possible like to use a common vocabulary – e.g. Objective vs. Subjective == Homotypic vs. Heterotypic synonyms - some discussion followed:
Paul Kirk – Biocode has a glossary.
Brian Tindall – need identifier to link between the codes. This has been done for the Biocode work.
ACTION - Get Glossary of terms up on the wiki. Either TDWG or GBIF should be the host of these - possibly TDWG in the TDWG vocabulary.
David Remsen gave a presentation on the need for nomenclators to be the baseline on which all other taxon concepts reference their associated taxon names. Presentation online at http://docs.google.com/Presentation?id=dgmbb297_14fw3jshgw
Brian Tindall – Tools for journal editors being developed by the DSMZ to check spelling & species combinations to work off of the official list (spellcheck/vocabulary check).
Jerry Cooper – envisage these tools to be developed for other nomenclators
Brian Tindall – these corrections need to be fed back to databases.
Paul Kirk – many of the problems are in the literature, and you can't change that (DB index the literature).
Rich Pyle – Algorithms need to provide confidence of linking.
Paul Kirk – correct spelling should be linked to the Nomenclator's LSID.
James Macklin – Not simple to feed this information back to the other databases. People using big databases – are using 3rd hand data.
Brian Tindall – Even though microbiology has an authoritative list, there are serious problems in that so few people are using it. Can even go into older journals .
Alan Paton – There is no 1 stop shop to fix problems – need the sociological aspect.
Jerry Cooper – Need to market GNI / GNA.
Chris Lyal – Taxonomists have gone to great lengths to do this for each of their specific groups.
David Remsen – Need an index of what's represented within the Nomenclators.
Paul Kirk fungi as another e.g. Mycotaxon is major journal for mycological taxonomy. It enforces that all the names & authorities must link back to IF.
Jerry Cooper – Need a Catalogue of Life-type initiative for nomenclators.

Phillipe Bouchet- Need to clarify the role of a Nomenclator:

Zoology – Nomenclator Zoologicus(NZ). Index of names. Don't necessarily fill requirements of the Zoological Code.

Paul Kirk – this is the same for IF

Alan Paton – same for IPNI

Rich Pyle – ZooBank will have a flag as whether these have been validated.

Metrics need to be developed all of which add up to a threshold over which things are valid or not.

Group needs to find a baseline set of standards through which they can overlap.

Gaps in Zoology are mainly that they're not all collated – many disparate lists.

David Remsen – Indexers need to make links to nomenclators (assertions)

Wish 1. Single virtual nomenclator to explicitly provide correct spelling for all names.

ACTION – List of reasons for assertions.

Paul Kirk – IF only follows requirements rather than recommendations of the code.

If code is not specific then the original orthography is followed.

Chris Lyal – Many zoologists not following gender agreements (esp Lepidopterists).

Paul Kirk – the codes may one day become redundant as big initiatives are mobilising and usage will define.

Raw names fall into groups for which there are many orthographic variants and also

Original Combinations

New Combinations

Wish 2. For any combination I want all other nomenclatural combinations.

Generic homonyms

Oenanthe

6 distinct homonyms in Darwin Core

Infra and intra regnal homonyms.

But actually just 2

Wish 3 – I want a provisional classification for all names where names can be categorized to enable taxonomic treatment.

David Remsen – good to provisionally classify names for which there are no other ambiguous occurrences – e.g. *Stegomyia aegypti* – not in fly list, but is a mosquito (*Aedes aegypti*).

Place to latch unknowns too rather than just lumping them together.

Practical Application linking specimens to names

COL doesn't provide all birds

Use other checklists – e.g. Howard and Moore – Many more get pulled out.

Need to get palaeontologists on board as well.

3 Wishes to an Action →

Index of Major Nomenclators

- Super-index of nomenclators.
  - Rich Pyle – Clearing house per code of nomenclature?
    - How do you deal with overlap
      - Internally within the clearing house – e.g. animals should all be done in ZooBank.

- Discussion about ambiregnal names – e.g. Microsporidia & Cyanobacteria (Blue-green algae).
- Rich Pyle – is everybody using LSIDs?
  - Brian Tindall – Bacterial not using GUIDs at the moment but probably going to go with DOIs
    - Names for Life using DOI's
  - Inherent cost of DOI
    - but this leads to potential longevity

Index of Genera

- Knowledge-base
  - All Genus Index
  - Compiled dynamically from other persistent services

Summary

- Namestring → as many as possible are going to be anchored to Nomenclatural objects
- Nomenclature → as many as possible anchored to different Concepts.
- Taxonomy (Concept Layer)
  - Nomenclatural databases have an opportunity to play a keystone role of de-aliasing content.

Paul Kirk – 2 types of taxonomic opinion

- The opinion used to make the nomenclatural act (ie the new name or the new combination (although difference of opinions in Zoology as to the standing of new combinations)
- The opinion used in making subjective (heterotypic) synonyms.

Coordination between nomenclators and taxonomic opinions required – the latter should be treated separately.

Paul Kirk – how far do you go with specifics. Do you just store 2 orthographic variants – the first published and the valid combination? What about other later validations of that name. (Clarify this with Paul Kirk) (Assume it refers to new subsequent valid combinations – David Remsen)

David Remsen – Clarified

- BIG Index= a finite list of names that exist out there – conceptually exists
- GNI – place to put BIG.
-

Chris Lyal – Zoology – Name in sense of the zoological code can exist an multiple ranks since the name is not the combination (e.g, Vireo solitarius plumbeous and Vireo plumbeous are the same name in diff ranks). Therefore require information on Rank to be captured as well.

Paul Kirk - Botany not a problem as name endings (authorship and year??) required. Some work is necessary to separate these endings from the body of the name.

PK what do we define as a name?

- String of characters that's code compliant irrespective of rank?
- Species name?
- Concept?

Rich Pyle – Zoological Code – some articles do deal with how ranks are managed in the code.

Brian Tindall – Driving force in bacteriology = metagenomics, not red lists etc. In the absence of names still need a hierarchy.

Rich Pyle – Portal interrogates BIG, which then goes to nomenclators which feed back homotypic synonyms then goes to taxon concepts to find heterotypic synonyms. BIG also has pointers to where text strings come from as well as links to which is the accepted.

Rich Pyle – next step is to have a BIG for taxon concepts.

David Remsen – these can be developed independently, Rich Pyle felt that the BIG is a prerequisite.

Paul Kirk/Jerry Cooper – need to explicitly treat cases where there is inconsistency – e.g. ambiregnal.
Paul Kirk - What do the sponsors require?

- David Remsen responded:
    - Index of nomenclators
    - Develop consistency amongst nomenclators
        - Cardinality of records
            - e.g. IPNI will provide identifiers to a single name and from there reference multiple records
    - What services will come out
    - How will data be structured
        - Practical value of getting information.
    - Index nomenclators.

Discussion as to the role of the prototype AGI / GNI that was worked through at Nomina 2. Dmitry Mozzherin clarified that an XML dump file is required from the different nomenclators to be able to feed into the GNI. Paul Kirk queried whether this would be more difficult than the existing available web services.
David Remsen – TAPIR/DIGIR = designed for federated searching, but GBIF harvesting = very slow. Therefore need dump of relevant information. **David Remsen needs to circulate GNI report.** Discussion ensued as to how much extra work (or less work) this would involve.

Afternoon Session

Required Outcomes
Yde de Jong

Common interchangeable terminology.

- Sources
  - Biocode terminology exists – PDF available to be sourced
  - One of the TCS Wiki's has a glossary
  - Brian Tindall The BioCode is Table available
  - TCS
  - ICZN & ICBN Glossary
  - LinneanCore?
    - Rather use Roger Hyam's vocabularies
- Rich Pyle - Missing words
    - e.g. Basionym comes close. "Name of original intent".
    - Namestring – series of textual characters?
      - optionally Include/Exclude authorship
      - optionall include / exclude Date
      - GBIF Index - RawTaxonName
- DR – Possibility of a workshop for doing this.
  - The ultimate outcome would be to embed these in the TDWG vocabulary
- Rich Pyle – Best place to do this?
  - TDWG Subgroup? - **Action** for Rich Pyle to do this.
    - TCS Mailing list?
    - Require a TDWG Sessions
    - or do we just do it
- Codes
  - Viruses
  - Botanical
  - Cultivated
  - Bacterial
  - Zoological

Definition of the Nomenclatural Layer as a technical implementation

- GNI
  - Index of names attached to stuff
    - Name
    - Identifier/GUID
  - What's needed to make your stuff discoverable
- GNA
  - Pull information together.
  - Resolve Taxonomic Hierarchy
  - Homotypic & Heterotypic Synonyms
- GNI – lean 4 column table
- Use Cases
  - Objective set of synonyms retrieved for a submitted namestring
- Index of Genera = static
- Metadata Needed
  - Name of source
  - Name of contact

- Fields to go into the GNI
  - Source
  - Identifier
  - Raw taxon Name or Parsed:
    - Genus
    - Infraspecific
    - rank
    - Author
    - Year
  - Rank
  - DateLastChanged
- Discussion as to how to build the AGI out of what is being served to GNI.
- David Remsen produced a sample text document with what the index contains.
  - Nomenclator records e.g. IPNI/IF/ING
  - Data Records e.g. BHL
  - Taxonomy e.g. COL
- Charles Hussey & David Remsen clarified that the AGI would be the place to disambiguate the higher classification.
- Make LSID for implied parents resolved with the LSID for entry
- Need a way of resolving abbreviations for authors.
- James Macklin wanted to know whether parsed taxon name is in GNI.
  - David Remsen – algorithms break name up into many parts with good confidence
    - These parts are internally stored in a normalised form
  - Rich Pyle – Nomenclators can easily provide it parsed
    - Providers should provide either parsed OR concatenated
  - Resolution of Nomenclator LSID
  - Mandatory subset of nomenclator elements
- From the perspective of an AGI – GNI data elements are enough.
- David Remsen covered the metadata profile
  - Basic Profile
    - Provider Name
    - Contact
    - highest taxonomic scope
    - etc.
  - GBIF working on expanded metadata profile
- Index Fungorum resolution LSID
  - Doesn't
    - No LSID for Genus record (Nomenclatural Parent - typification)
    - Homotypic Synonyms cannot be resolved
    - Correct Name
    - Nomenclatural parents need to be added to Roger's vocabulary.
  - Does
    - Orthographic Variants
    - Wrong Names

Afternoon Tea break

Use Cases for GNA

- Dave Roberts - Scratchpads
  - BIG index of value
    - Use to build/expand nomenclator
    - Hierarchy needed
      - Management Hierarchy or
      - Explicit Parent Child Relationships.
    - Discover initial content.
  - Taxonomies pulled from uBio ClassificationBank
    - Jerry Cooper - Should link to nomenclators for editing taxonomies
  - Need initial position for taxonomists to fill out their taxonomic revisions.
- David Remsen – index serves as a seed.
- David Remsen – ZooBank should use seed to assign GUIDs to potential names.
- Rich Pyle – Will be more conservative about putting the gold standard stamp on a name.
- David Remsen - Index makes resolution pathways visible, and when these come on line they can be used.
- Dave Roberts – Scratchpads
  - Classification “Exploding” query service useful – also having nomenclator linking homotypic synonyms
- Rich Pyle
  - BIG/GNA – bridges a number of services
- Dave Roberts – Scratchpads
  - Not expecting resolution
  - Needs Parent:Child relationships
    - To go and find all possible names for a group.
    - Allow being able to complete the GNI
- Charles Hussey – Disambiguate names
  - Assign names to phylum/order – use the AGI
- David Remsen – suspects that if the info is available then many of the problems with management classifications will be solved.
- David Remsen – Management classification should be divorced from the nomenclatural synonyms.
- Dave Roberts – Scratchpads – Expansion of generic abbreviations
  - David Remsen – not done previously but value added service

Meeting outputs

- Coordinated statement from the nomenclators
  - Done in TCS Wiki?
  - Linked from nomenclators
  - intent to collaboration make information more available
  - integrate initiatives

        - ACTION – Jerry Cooper to put document together – Rich Pyle to put forward at TDWG
- Commitment from nomenclators for indexing
    - Index Fungorum – guinea pig for 2nd AGI
        - Dictionary of Fungi is the management hierarchy
    - IPNI
        - Alan Paton and James Macklin assume this will be done
    - ZooBank – Yes
        - Meeting on Friday to develop strategy
    - Brummits Genera
    - Prokaryotes
        - Brian Tindall to get permission from George Garrity
        - Names4Life
    - Index Nominum Algarum
        - ING for algae.
    - Spec to be sent out by David Remsen & DM to other partners.
- Management Hierarchy – try to get from COL.
    - Task Group – (Charles, Rich, Paul)
        - Use Cases
        - How low do you go
            - Minimum = Code
                - Kingdom
                    - Lowest consensus taxon group to resolve homonyms.
        - Don't make it visible.

AOB

None!

GNOMA Meeting Outcomes and Actions

Publish a coordinated statement from the primary nomenclatural databases that indicates an intent to integrate the different initiatives and collaborate on defining services and specifications for a unified Nomenclator network

Jerry Cooper	Circulate GNA/GNI concept documents to GNOMA participants. *Believed to be in progress. JC to verify*
Rich Pyle	To put forward at TDWG

Develop a common interchangeable terminology among the nomenclators. *Please note that GBIF is willing to support tasks groups to expedite the development of this terminology.*

Brian Tindall	Locate and circulate the BioCode terminology PDF
David Remsen	Assemble terminology resources into a common repository or list of pointers for review. See Google Docs (http://docs.google.com/Doc?id=dgmbb297_115g3hmx7g4)
Rich Pyle	Set up a TDWG subgroup to act as repository or should this live at GBIF?<br>*TBD by Pyle/Remsen*
Rich Pyle	To circulate email summary of discussion history

Circulate a GNA/GNI document

David Remsen	Circulate GNA/GNI concept documents to GNOMA participants<br>*In progress.*

Get commitments from the primary nomenclators for providing a common index output file for the GNI.

Brian Tindall	Will consult with George Garrity over the creation of a prokaryote index file via Names4Life.<br>*This has been done and requires additional consultation with George Garrity.*
IPNI	Alan Paton and James Macklin assume it will be done.<br>*IPNI Partners will be approached at Nomina III regarding the creation of an index file.*
Index Fungorum	Paul Kirk will serve as test case with Dmitry Mozzherin

**Anyone!!**	Index Nominum Algarum<br>*Does anyone have a contact there to determine if they are willing to provide an index of algal names?*
ZooBank	Rich Pyle says yes<br>*Dmitry to coordinate with Rich on the registration and output format.*
Dmitry Mozzherin	Will supply the basic specification and example file to participants.

Establish a task group for the development of a nomenclatural management hierarchy. *Please note that GBIF is willing to support tasks groups to expedite the development of a strategy to develop a management hierarchy. Recommend establishing such a group in concert with Catalogue of Life efforts and to include Zoological Record efforts in this area. PESI and CoL also have resources or strategies for this.*

Charles Hussey, Rich Pyle, Paul Kirk	Task group to develop use cases established.<br>*Please affirm agreement to pursue this.*
Charles Hussey, Rich Pyle, Paul Kirk	Determine the scope of the hierarchy (i.e., How far down the taxonomic hierarchy is required? Is it uniform for all taxa or relative to different groups?)<br><br>Lowest consensus group to resolve homonyms.

Additional
Data Model Analysis and Comparison
In preparation for Nomina III, Jerry Cooper is collecting information on current data models of MycoBank, Index Fungorum, ZooBank.

Alan Paton or James Macklin	Provide IPNI data model in preparation for Nomina III for evaluation in regard to identification of common data elements

ZooBank Data Provision Meeting

A meeting supporting the International Code of Zoological Nomenclature (ICZN) ambitions on establishing ZooBank facilitated by the Pan European Species Directories Infrastructure (PESI), the Global Biodiversity Information Facility (GBIF), and the European Distributed Institute of Taxonomy (EDIT)

Draft Agenda

Date: August 29 2008
Location: Amphitheatre Rouelle, Pavillion de la Baleine at the Jardin des Plantes at the Muséum national d'Histoire naturelle, Paris, France
Chairpersons: Richard Pyle and Ellinor Michel

See ZooBank meeting Logistics
See ZooBank Attendees List

Meeting Agenda

Welcome (09.00)

Introductions / lectures (09.15-12.15):

1. What is the rationale for ZooBank? Why is it needed at all?

ZooBank Scoping What ZooBank is NOT - Discussions perhaps led by Phillipe Bouchet
Differentiating between biodiversity information, nomenclators, and taxonomic authority lists.
Possible report of the outcomes of the Sunday/Monday ICZN meetings

Internal perspective - by Ellinor Michel
External perspective (Global Names Architecture) - David Remsen & Yde de Jong

2. How: Social, technical, practical, financial perceptual, and legal issues attached to the ZooBank development

Prospective: ZooBank and registration of new species names

ZooBank as (prospective) ICZN electronic name submission system, anticipated functioning and associated issues (modifications to the code, etc,) - by ICZN code commissioner associate {unconfirmed}
Proposal for ICZN-certified registration centers - by Nigel Robinson
ZooBank technical model - by Rich

Retrospective: Addressing zoological names from 1758 to present.

Processing retrospective registration (role of the existing nomenclators in populating ZooBank, data quality assessment, uploading models/procedures, tools/interfaces, scheduling, etc.) - by Chris Lyal

Building the resources network / data providers community (SMEBD example) - by Phillipe Bouchet

Workshop / discussion (13.30-17.00)

3. What conditions are required for ZooBank collaboration and what are the barriers to sharing nomenclatural data?

Data ownership / IPR

Technical aspects (tools for uploading / interfaces, cross-linking, GUIDs, etc.)

Acknowledgement models

Benefits (improved quality through validation process, improved visibility/access through GNA, development of impact factors, etc.)

What steps needed to reach consensus (road map, interim committee, etc.)

Additional requirements (financial support, IT support, etc.)

Desired Outcomes of the discussion

1. Scoping document and strategy for integrating/accounting for needs of internal and external partners.
A reconciled and coordinated plan that can be used to leverage support for the development of ZooBank for those parts of ZooBank that will benefit different user communities and use cases. A clear strategy for facilitating needs NOT within the scope of ZooBank that were raised within the meeting.

2. Business model for ZooBank, which can also be used for the PESI reporting and to obtain additional funding.
I think we are all willing to contribute to (parts of) that document.

Note: Several participants need to catch their flights/trains in the early evening and therefore will leave around tea time (15.30 hour). As a consequence the last part of the meeting will probably be somewhat less formal and continued at a suitable occasion (pub/restaurant) after the official meeting ending (at 17.00).

ZooBank Data Provision Meeting

A meeting supporting the International Code of Zoological Nomenclature (ICZN) ambitions on establishing ZooBank facilitated by the Pan European Species Directories Infrastructure (PESI), the Global Biodiversity Information Facility (GBIF), and the European Distributed Institute of Taxonomy (EDIT)

Date: August 29 2008

Location: Amphitheatre Rouelle, Pavillion de la Baleine at the Jardin des Plantes at the Muséum national d'Histoire naturelle, Paris, France

Chairpersons: Richard Pyle and Ellinor Michel

Attendees

For complete list see list on google docs.

Name	Initials	Institution / Role
Donat Agosti <agosti_at_amnh.org>	DA	Plazi / Antbase
Ward Appeltans <ward.appeltans_at_vliz.be>	WA	Flanders Marine Institute: Belgium. Manager of WORMS. Also involved in
Marilyn Beckman <>	MB	SFS,OSF,PSF
Philipe Bouchet <>	PB	ICZN: ERMS, WORMS, Molluscan Generic names: 23,000 validated names.
Thierry Bourgion <>	TB	FLOW / Species2000
Jerry Cooper <cooperj_at_landcareresearch.co.nz >	JC	Landcare Research in NZ. Works with PK on Index Fungorum. On Global committee of sp2000. Working on Global Checklist of Composites & Checklist of NZ Plants
Vanessa Demanoff <edit_at_mnhn.fr>	VD	Edit Project manager
Edward Dickinson <edward_at_asiaorn.org>	ED	Howard and Moore Checklist
Daphne Fautin <fautin_at_ku.edu>	DF	Hexacorals/ICZN
Olivier Gardonomie <>	OG	MHNH Paris: head of national inventory of natural heritage (French equiv of GBIF).

		Involved in data Repatriation: French overseas territories.
Kehan Harman <kehanharman_at_gmail.com>	KH	NHM / GBIF: Minutes
Bart Van Hoorn <bart.vanhoorne_at_vliz.be >	BV	WORMS
Charles Hussey <c.hussey_at_nhm.ac.uk>	CH	NHM PESI & UK Species Dictionary
Norman Johnson <njohnson_at_ent.umass.edu>	NJ	Hymenoptera Name Server/AntBase
Yde de Jong <yjong_at_uva.nl>	YJ	Facilitator. Chair of ECAT subcommittee.
Paul Kirk <p.kirk_at_cabi.org >	PK	CABI, UK. Managing Index Fungorum (IF): Chair of GNA steering committee
Chris Lyal <c.lyal_at_nhm.ac.uk >	CL	Natural History Museum London (NHM): PESI/ UK Species Dictionary
James Macklin <jmacklin_at_oeb.harvard.edu >	JM	Harvard University Herbarium (HUH) Informatics. IPNI & also
Ellinor Michel <e.michel_at_nhm.ac.uk>	EM	Natural History Museum, London / ICZN Secretary.
Sandro Minelli <>	SM	Chilobase
Dmitry Mozzherin <dmozzherin_at_eol.org >	DM	MBL/EOL. Implementation of GNA & EOL
Thomas Pape <TPape_at_snm.ku.dk>	TP	BDWD
Eric Pasquet <pasquet_at_mnhn.fr>	EP	MHNH
Jurate De Prins <>	JP	World Gracillariidae
Richard Pyle <deepreef_at_bishopmuseum.org>	RP	Bishop Museum, Hawaii. ICZN Commissioner – Implementation of ZooBank
David Remsen <dremsen_at_gbif.org>	DRE	Facilitator.ECAT/uBio: Taxonomic Names

		Component. ECAT needs more structure.
Nigel Robinson <>	NR	Zoological Record / ICZN Trustee
Michael Ruggiero <ruggierm_at_si.edu>	MR	ITIS/CoL
Paul Schoolmeesters <>	PS	Scarabs
Brian Tindall <bti_at_dsmz.de >	BT	Chair on Prokaryotes commission
Ed De Walt <>	EW	SFS, OSF, PSF
Zhi-Qiang Zhang <zhangz_at_LandcareResearch.co.nz>	ZZ	ICZN/Zootaxa
Miguel <>	M	MHNH Paris

Philipe Tiller – Head of EDIT opened meeting with welcome to all visitors. Covered the need for integration of all taxonomic activities, from collaboration, to datasets, to administrative to institutional.

EM – welcome from ICZN – covered the history of ZooBank and the process of changing the code which would facilitate registration as a methodology for zoological names. There is work in process to change the Zoological Code in order to make it possible to have a global registry of animals,with both prospective and retrospective data.

RP – Brief historical review of ZooBank

– Announced in Nature Sept 2005

2007 – July – LSIDs implemented

2008 – Jan – Official Launch

2008 – April – Grant to Zootaxa/Plazi -

2008 – May – publications started appearing incorporating ZooBank LSIDs in the publications

2008 – August – ICZN Meeting in Paris

Details of implementation overseen by a governing board

Prospective Registration

What:

Nomenclatural Acts.

- Governed by the Zoological Code
- Bringing the names into existence
    - Establishing new Species, genus & Family group names in Zoology
    - Lectotypification/Neotypification, Emendations, First reviser etc.
    - Higher rank names:
        - A few rules covering these
        - New Combinations
            - Not governed by the code, but can lead to secondary homonyms
            - Need to be kept track of.
            - Need to link new combinations back to their 'Basionyms' – term not used in Zoology – maybe need to find a term in zoology.
        - Published misspellings – others
    - Intellectual Architects of ZooBank
        - Commission, not RP
- Publications
    - as defined in article 8 of the ICZN Code
- Authors
    - Authors of registered publications
    - Registered users of ZooBank
    - Botanical world keeps track of authors
- Type Specimens
    - Integral to nomenclature; cross-linked to museums
- ZooBank should be fairly autonomous – not depend on other people's LSID's/GUIDs

Who

- Open to Anyone
    - Open access – welcoming & Friendly, but problem of hackers and others with malicious intent – need facility of roll back changes.

- Approval Processing
    - Tighter control over who can contribute
    - Problem as perception of Authoritarianism / Exclusion
- Self Policing
    - Start with robust list of pre-approved taxonomists
    - Allow any existing registered user to register others
    - Low-level entry point for being data contributor to ZooBank
    - Some responsibility for malicious / bogus content

How

- User interface
    - Appearance and Aesthetics
    - Searching Capabilities
    - Statistics Page
    - Help Pages
    - Workflow
        - for new entries
            - Human mitigated entries not the web service type entries that most of the current meeting will be about.
        - Editing entries
    - Other interface issues
- Data Quality
    - 3 domains:
        - Verified / Registered Content
        - Unverified/ Provisional/Pending Content – LSID – Prior to registration
            - Including other databases
        - External Content – Global Names Architecture – no ZooBank LSID

                - overlay ZooBank of names 'in the wild'

Questions:

DR asked for some definitions of terminology

- GUID – Globally Unique Identifier – unambiguously unique identification units.
    - Humanly readable – always leads to ambiguity issues
    - Forms of GUIDs:
        - DOI – Digital Object Identifier
            - used in publication industry
        - LSID
            - Self resolving identifier

EW – Data Quality – will the commission help?

- How will the group gather new content.
- RP – let anybody with good content into the door
- ZZ – Eg Zootaxa – registration may become part of the publication process – Authors themselves

PK – Voluntary Registration now in MycoBank – in 3 years 40% of nomenclatural novelties are registered in MycoBank. Journals sign up to the idea of registration and require it. Not universally accepted but some good process.

PB – Need to define what ZooBank is _not_.

- WORMS – World Register of Marine Species – subset of zoology but cuts across all taxonomic groups.
- Each initiative says they are different but a lot seem to be doing the same thing.
- Authoritative lists are required.
- Need to discuss the territory that every one wants to list on ZooBank
    - Recording
    - Registration
    - Code Compliance

EM – question for PB – proposed changes to ICZN

- PB – Commission considering electronic only publication of names

YJ – be good to require that new names are registered within publication process similar to sequences in GenBank

SM – There is a difference in that the names need to follow code compliance for example priority in publication of names.

Names in the global scope – DRE

- Biological data tied to Names
- GBIF – involved with mobilising biodiversity information.
- Other initiatives have many different sources of data
- GBIF has to make use of what information they have

Nomenclature needs for biodiversity indexers.

- Biological info has species names attached to it. GBIF 150 Million records – 5% / month.
- 100GB of text to build index.
- Taxon names link.
- 1 of many projects in the process of mobilising data.
    - BHL
    - NCBI
    - Zoological Record
    - AntWeb
    - MaNIS
    - GBIF
    - OBIS
        - BIG index of names (Biodiversity Indexing Group).

- In everybody's interest to know about names
- COL – conventionally used as an authority list. GBIF using it as a scaffolding.

        - Syntax
            - Correct spellings
            - Some misspellings
        - Semantics
            - Browse / organise
            - Synonymy
            - Some homonyms
        - Only a fraction of data
            - GBIF
                - 5 000 000 distinct namestrings with authorship
                - 3 500 000 without authorship
                - only 328 301 shared (even though COL has 1.1M
                - BHL/NameBank/NCBI – 10 M names
                - only 30 % overlap with GBIF.
            - Spelling variations *Actinobacillus actimomycetemcomitans* and 40 other spelled forms. Using semantic tech can specify why orthographic variants
    - Names as strings of characters
        - Many different ways of expressing names. - with / without authority – different combinations

3 Wishes

Wish 1. Single virtual nomenclator to explicitly provide correct spelling for all names.

BIG index of raw names needs to be able to checked against.

Wish 2. For any combination I want all other nomenclatural combinations.

Discussion as to whether other combinations of a name are synonyms (objective synonyms) or just new combinations.

Wish 3 – I want a provisional classification for all names where names can be categorized to enable taxonomic treatment.

Problems with intra and infra regnal homonyms – how many *Oenanthe* are there? How many *Gerrardia* are

there?

CoL – Birds – have very little overlap with other authoritative checklists / catalogues – eg Howard & Moore, Handbook of Birds, etc. Plus specimens with birds, and there is very little overlap.

Nomenclators do exist – subregnal & super regnal – would be ideal to unify them into an index of nomenclators.

We have the bits in place to make a 3 tier system with Nomenclature between Taxonomy and raw name strings.

Would like ZooBank to capture Objective Synonyms

Questions:

PB – Wish-list in reverse

- 3 - Wish to have a classification
    - No way that the classification can come from ZooBank
    - PK – Just need to have some kind of container of convenience for a large number of names
    - RP – Use a 'Management Hierarchy'
    - CH – The management hierarchy will be necessary to disambiguate homonyms
    - General agreement that some form of hierarchy would be necessary, but the term 'Classification' is loaded.
    - **ACTION** – DR to refer to Management Hierarchy not 'Classification'
- 2 - Wish for new combinations to be captured
    - PB – NameStrings – does this include new combinations?
        - Backlog of 1.4 million valid animal species
        - 1M Fossils
        - 4-5M additional species.
        - 2-5 nomenclatural combinations per nominal species
        - 4 orders of magnitude more names.
        - If ICZN says for e-only publications that registration is compulsory.
        - GBIF should prioritise
    - PK – NameStrings linked to a type.

- ED – does not think that other combinations can be kept track of.
- YJ – Fauna Europea – Tracks Subjective Synonyms of Genera & therefore obtain objective synonyms.
- DF – requirement of core data?
    - also have stuff which people might have which would be useful for taxonomists.
        - RP
            - Links to holotypes
            - New Combinations of names in other genera other than the original
            - Other misspellings
            - Two categories of what to do with these
                - Include optionally
                - Explicitly exclude
            - New combinations are useful because they preclude later homonyms
        - CL – Bank of names which Zoologists/Taxonomists cannot use.
- ED – Need to have the facility to keep track of changes that are being made continually with new technologies
- DR – where do we go to find information
- JP – constant process of moving names between different genera and new gender variations of names
    - Only way of tracing names esp with new epithet spellings
- RP – Secondary combinations will only be linked to original combinations.
- RP – Any increased workload for other nomenclators with increased rate of changes due to modern research techniques?
    - PK – Workload shifted but not increased
    - BT – any new name including new combinations are required to be entered in the Bacteriological Code
    - JM – Some increased workload for IPNI, but IPNI has dedicated staff to deal with this.
- DF – what do we do with misspellings?
    - Somebody has to take responsibility

        - DR – GNA will capture names but does need the names to be linked to some putative 'original/authoritative name' – therefore GNA will certainly have algorithms to try and identify misspellings and will explicitly document it when these links are made
    - SM – ZooBank not include misspellings but should include new combinations.
    - PK – Nomenclature is a service to taxonomy not the other way round. Taxonomists need to deal with all names of
    - PB – Alternative names are the domain of the taxonomic authority lists.
    - TP – Require the Code Compliant Names. Other databases are already capturing the new combinations.
        - RP – role of ZooBank is to maintain the 'Basionyms' sensu Botany, then all the other databases can provide these to the GNA
        - Many DB's will provide these, but there are also many other groups of animals for which nobody is doing it. Don't want these to fall through the cracks and somebody possibly ZooBank or sister thereof or ICZN should maintain.
    - RP – what will happen to orphan databases which have been
    - CL – Where when secondary combinations become nomenclatural acts (eg become secondary homonyms) they should be incorporated.
    - RP – Technically DB can capture all of this but need a political statement.
- 1 – Relates to nomenclature
    - PB – only link between biodiversity and nomenclature
- ED - Ornithology
    - Many people working on ornithology but very few are taxonomists, and those who are taxonomists do not have the time to capture the changes.
- NR – Zoological Record is capturing new combinations where these have been specified as such.
- JM – 6 person job for botanical names. ACTION will provide details of the costing.
- PK – 1 person 40% of the time capturing all new fungal names. 2000 *per annum*.

Nigel Robinson – Building ZooBank content with accredited registration centres

Zoological record in ZooBank.

Prospective Registration Problems

>15 000 – 25 000 new names going into ZooBank.

2300 journals and monographs

1300 Journals contain new species

Scale of task – 6 people full time - €250K PA

Proposal

- A series of ICZN accredited registration centres
    - Under direction of ICZN
        - Ensure quality standards
        - Relationship governed by agreement to protect supplied ZooBank data
    - Does not preclude registration
    - Trade of services for mutual benefit
- Benefits for ICZN & ZooBank
    - Builds ongoing ZooBank Content efficiently at low cost with guaranteed quality
    - Increased visibility for ZooBank through accreditation stamp and links on partner sites
    - Risk spread to cover gaps
- Benefits for Taxonomists
    - Automated registration of names exposes research in timely pre-validated manner
    - Exposes data from developing countries
    - Does not preclude registration
    - Commercial Interest
- Benefits of Registration Centre for ZR
    - Accreditation
    - Links back to their sites
    - Joint development of additional content
    - Feedback on data quality
    - In terms of Zoological Record
        - ZR provide these for

        - Coverage – 5000 Journals
        - High quality data
        - New names validated to >99 % Accuracy
        - High level of completeness
        - Sophisticated monitoring / data capture systems
            - Direct from original publications by experienced dedicated grad zoologists
        - Durable – 150 years old
            - Used to partnering with non profit organisations
        - **Index to Organism Names**
            - http://www.organismnames.com/
            - Free service available – quick and dirty list
    - Mechanism
        - PDFs / scanned entries – acquisition
        - Indexing analysis
            - Forms based on ICZN code – 200 data checks
                - Authority files
                    - Names
                    - Generic Index 350 K names4Life
        - Data Warehouse
            - unified data format for 1864 → Present
        - Feed into ZR, ION, ZooBank

Questions

- BT – Brings up Biocode and questions of where the problems come from
    - PB – Problems
        - Finding Names
        - Code Compliance

- Zoological Record = a record – will they always find things eg paleo names in geological literature? NR – would like to and they will certainly be able to as they're part of a larger organisation which is recording

RP – Architecture of ZooBank

Should we add a link to RP's presentation here as the diagram is more accurate.

Pending	Registry	External Content
HNS lsid<br><br>add ZB LSID	Name details<br><br>Status: Available/Unavailable<br><br>Rank<br><br>Epithet<br><br>OrigGen<br><br>Auth<br><br>Year<br><br>Page<br><br>Source urn:lsid<br><br>Links	Plugs in to Zooank – pulling info from other sources such as gbif, bisop museum etc

Protocol for bidirectional data exchange between eg HNS (Hymenoptera Name Server) & Catalogue of Fishes (CoF) and ZooBank

Be able to build cross links of registered names to external content.

Advantage to having access to external content is taxonomists will have convenient way of finding out what's 'in the wild'.

Model of how this might work. But not complete requirements.

- PB
    - Recording = Pending
    - Validated (Code Compliant) is not the same as 'Registration'
    - Discussion as to what constitutes Registration – Validation is the Gold Standard.
        - Change of diagram

- External → Pending → Registration → Code Compliant ← External overlay

- TP – Names have to be code compliant

Overview of workflow supplied by Chris Lyal:

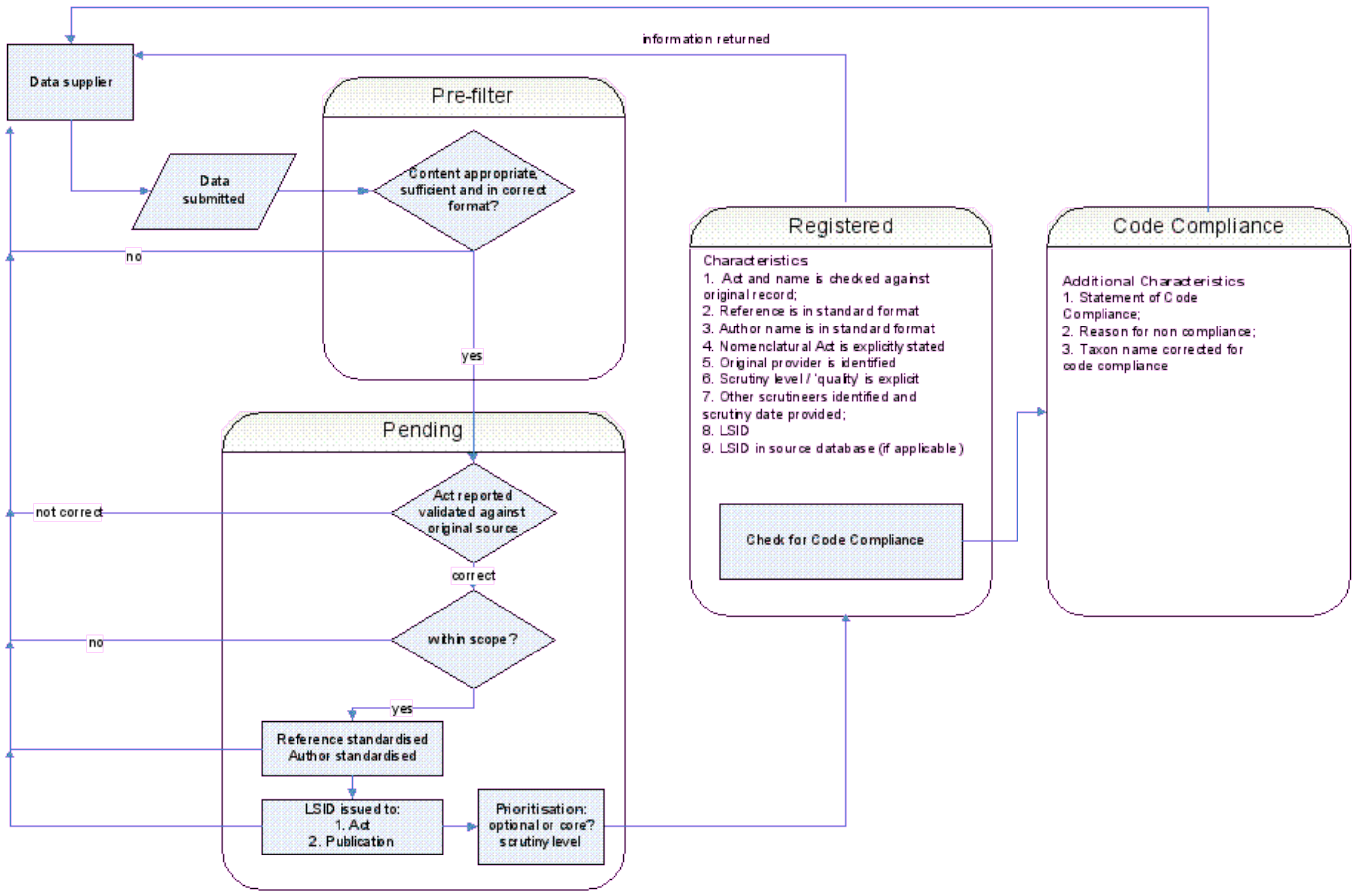

Lunch break

Afternoon session:

CL – How to deal with retrospective registration

Not about the weevil DB.

- Who does it:

- What is the content

- what are the quality / fitness for use criteria

- what are the quality controls

- Who Does it
  - Pre existing content
    - Index Animalium – nomenclator up to 1860
      - Good quality
      - lots of content
    - Official Lists
      - A lot that has been done/ don't always remember to look for it
    - already are gathering lots of data that can go into ZooBank
      - checklist compilers
      - Individuals as part of their taxonomic / nomenclatural research
  - Aim to capture what's already being done
    - Need incentives rather than additional burden
    - More consistency between initiatives
    - Consistency with ZooBank requirements including clarity of nomenclatural acts.
    - Eg need to capture explicit checks against the code – eg implicit if its in a list, but it needs to be explicit in the list.
  - Already discussed
    - Recording
    - Registration
    - Code Compliance
  - Data collected will vary between different providers so potential for refinement of records by different providers will be needed.
  - However ZooBank content may influence future checklist content.
    - Have some form of checklist best practice.
  - Edward – Validation on entry always required
    - RP – pre filtering will check things algorithmically
      - Humans then do the stuff that computers can't

      - Then corrections should still need to be possible, but target is that to have this on a case by case basis.
    - DF – DB's at different states themselves
      - e.g. if provider can certify that it's been checked by the original description
- Quality Criteria
  - Data are of different quality
  - Need agreed set of criteria to help both individual and machine users to assess content.
  - Eg WTAXA (Weevil DB) confidence levels recorded:
    - secondary data source
    - checked against original-
      - and available revisions (not necessarily the most revision)
      - and last modern revision
    - validated by taxonomic scrutinizer (with name and date of scrutiny)
  - The above may not be fully applicable to ZooBank content, so others may be needed, e.g.
    - checked ICZN Opinions / official lists
    - original orthography
    - correct according to code
  - final verification (c.f. ZooRec checks for accuracy?) and lock-down.
  - RP
    - some rules pertain to acts
    - some rules are just part of the code.
    - E.g. genus typification is part of an act.
      - Do we have an e.g. of a rule that's just part of the code?
  - CL – need advice from community.
- Quality controls & Tools
  - Format advice / requirements of ZooBank
    - Went through mock-up of a form for ICZN – a form to check for code compliance.

        - Feedback from this results in a data packet that can then go into own db (or ZooBank) rather than re-keying it all over again.
    - Ownership of provisional records needs to be decided – who can alter and when?
        - How often do you submit data
        - Do you just take the updated stuff
        - all sorts of other efficiency questions – dealt with by technical community.
- ACTIONS
    - Quality indicators identified and agreed
    - Guidelines that we can follow.
        - ED – Happy to contribute to these
        - RP – ZooBank space required on CalAcademy ICZN Wiki
            - ZooBank Mailing list.
    - What forms we can devise.
- EM – How many people are contributing
    - currently 2
- EM – Need a list of potential data providers and links to those that they link to.
    - Give credit for work that people have done
    - ZooBank becoming more clearly defined from taxonomic DB's, and much credit should go to the taxonomic DB as well as ZooBank if info is being pulled together there.
- RP – performance indicators for ZooBank
    - the quicker you get people off your site the better – click through rather than time on site.
- DA – Suggest facility at ZooBank to store all PDF's checked for your work. Link from record to page in PDF as to how the judgement was made.
    - PB
        - Many taxonomists will not check facts using electronic version rather use original
    - ED
        - Brings up copyright bugbear
    - RP

    - Require strong connection between ZooBank, PLAZI, BHL
  - PB
    - Want to outsource the important literature capture
    - As is the case with specimens
  - RP have the capacity to locally cache specimen / literature data but when these come available / are available, they will be redirected to automatically.
    - CL – need to develop mechanism for periodic updating of this information not just on entry / validation of names in ZooBank.

Floor open to audience

Impressions

Conditions for Participation

Doubts

Questions

WA – No objections to provide all WORMS names as long as individual editors are cited

- Store all new combinations & available names in ZooBank, but corrections to be made outside.
- Want new information to be fed back to WORMS
- Possibly registration come through WORMS and serve through ZooBank
- Discussion as to whether new combinations feed back into WORMS but opinion seemed to be that new combinations are required to be captured by WORMS but the requirement is that these new combinations have to point through to the
- Type information in Zoobank Mandatory?
  - RP – Unresolved as there are ambiguities in the code.

TP – Need structure that is strictly code compliant

- Need to cope with credit system
- Registration should be built in to the publication process of new names
- Dipterist community working reasonably well, but a long process to get this working.

DM – Registration great tool

- need facility for registration by proxy – snail mail to authority.
- Some kind of spreadsheet / document to send

TB

- Agrees with TP points
- Feedback from ZooBank = critical, but will also produce more work – need some form of resource to be able to incorporate this
- Very interesting as it will focus on custodians with small databases – give credit for work that is being done which is difficult to gather within institutions.
- Much diversity in different approaches in taxonomy
    - Very different specific activities that differ even within the discipline of entomology
    - Considerations as to the sociological model of how work is done
- Tools for validation of own databases against ZooBank

JP

- Not many people working on the Gracilliaridae DB. Create own index / catalogue built for research purposes – impossible to restrict to a particular region.
    - Make good quality database of one group globally.
    - Years spent on all original literature
    - locality of type
    - original descriptions
    - distribution
    - biology & parasites
    - in a book – published
        - but changed when published therefore Belgian Biodiversity Platform – constantly updated and therefore online DB
        - Contacted by SP2000 & CoL – years of work involved should she give?
            - But if it's not shared then the correct information can't spread
        - Therefore role of ZooBank = place for information to feed through.

        - LinkOut for ZooBank also facility.

PS (Paul Schoolmeesters – Scarabs)

- Question about ZooBank
    - Who are the users
    - What do they need
    - YJ – everybody will be – glue that links information
    - RP
        - Individual taxonomists
        - Database Managers
        - Aggregator Community – e.g. GBIF etc. (BIG)
- Sees all the information out on the web but there are problems regarding the fact that everybody is using the same primary sources e.g. errors in ITIS/CoL get carried through to GBIF etc.
    - General agreement that this was something that would be worked on.

SM

- Centred around acts, not around names.
    - Only information in the publication
        - Author.
        - Type in pub.
        - Original info. Only.
- Information should be checked against original publication.

MR – ITIS

- Will use ZooBank for validations
- ITIS already checks for code compliance for all names that go into it

EW & MB

- Illinois Orthoptera Species File
    - Willing to help out providing data
    - Scope broader than ZooBank

- YJ how do you deal copyright
    - Authorship to contributors and designers and a few other people
- YJ – share data?
    - SP2000
    - others who ask
    - fully subsidised & long-term viability

DF

- Hexacorralians
    - Sea Anemones well covered.
    - Other groups less well covered, but there has been some funding to allow for collaboration.
        - Would ask for funding for getting information in/available to ZooBank
        - Data fed out into a variety of resources – some way to disambiguate the resource is required.

ED

- Howard and Moore
- Ornithology a strange community
    - Originally species names only
        - no authors and publications
        - 2003 – started incorporating these
    - Other sources of info of birds coming out at the moment
    - 35 000 names being collected eg by ITIS, but others also out there. Over 100 000 more.
- ED is one of members of ornithological nomenclature committee. Feel that they can help validate names in ornithological area.
- Data ownership
    - All data contributing to database has agreements for nonexclusive permanent use
- Two databases
    - Literature in NHM server (Scratchpad)

    - Separate database containing information for the rest of the volume.

- Many of the works that he deals with affect disciplines not only ornithology, so it would be good to be able to aggregate these together.

DB – No Database but here as president of commission. Supportive of all the work being put into ZooBank.

NJ – Hymenoptera Name Server

- concern that gold standard stuff is locked down, but if it should be possible to change it if it's found to be wrong
- ZooBank needs to be flexible that others have their own standards when gathering data
- Concern about mission creep
- Different fields for retrospective registration vs. prospective registration

PB

- CLEMAM Checklist of European Marine Molluscs
    - Accepted 3 5000
    - Nomenclaturally Available 15 000
    - Then incorporated into ERMS
    - Much feedback which is not useful
- 21 years of work of nomenclator of molluscan suprageneric names
    - 21 000 names
    - 4000 family group names
        - No demand from other initiatives for this suprageneric information
        - Not available online
        - ZooBank should focus on this kind of information.

TB -

- Will information be available to feed back to taxonomists to work on unworked groups

NR

- If you go down the route of collecting Genera → in Nomenclator Zoologicus but also more information available.

ED & RP– positive experience & more to do and hope things go forward.

DF – What data format

Process different data structures to get information together. Could be handled on case by case basis, but also there could also be a template.

What do we do if Rich goes? Data model has been published but information is all out there.

EM – General enthusiasm about ZooBank and a lot of data available for retrospective infilling. Is there financial burden on doing it?

- Is there a budget to do it 1-2 M €
- More for reterospective infilling.

Teabreak

Final Issues

YJ – Looking at the next step

- Zoobank Mailing list will be used
- Wiki Pages and Google docs WRT meeting outcomes

PB -SMEBD – There should be some form of continuity of ERMS after the finish of the framework programme.

- Society for the Maintenance of Electronic (/European) Biodiversity Databases
- Provides some form of intellectual continuity of ERMS
- Only considered at the end of the framework programme
    - Sociology – people are happy to give away their rights as long as there is some formal agreement that they are giving them away and there is some moral agreement that their data will be maintained after the end of the programme

YJ Question to TB – Databases which are currently in sp2000 europa – what will happen if contributors stop building.

- DB in Career of Scientist

        - Institution has a role to maintain, as well as replacement of scientist
        - but in general institution will not manage this – this is where SMEDB come into play
    - SP2000 working on 2 new proposals
        - Proto-GSD's (Global Species Databases)
            - Gaps exist
            - Create quick and dirty DB with some information available
        - Possibly role of SMEDB to take custody of these

DR – 3 wishes not necessary what ZooBank should do but we need coordination between different initiatives to include them all.

YJ – to EM what resources are available to do this?

- EM
    - Not vast.
    - Office fit for purpose
    - Would need financial backing to do it
    - May cost too much to do more at the office in London
    - May be possible to develop in collaboration
- DF – Financial Plan of what will contribute to ZooBank?
    - Proposal for 1M to Gatsby foundation
        - Doesn't cater for
        - Gatsby
            - have ¼ M for it if there's matched funding and it can be done within a timeframe and for Max of 1M
        - therefore need to cost it first and then get funding
- Need to be able to find the foundations that have the money for this

JM – Botany – ICBN not driving it. Partnership of institutions with the resources in house.

- Longevity – need to look at institutions to support this.
- $.5-1M a year just on people & infrastructure

Previous meeting(s):

- The future of taxonomy – the role of GSD-networks and nomenclators in taxonomic information infrastructure networks: initial scoping meeting on GSDs and nomenclators involvement, London, Natural History Museum, UK on 18-19 March 2007.

  http://www.e-taxonomy.eu/node/720

- The future of taxonomy - the role of nomenclators in taxonomic information infrastructure networks: ZooBank involvement, Padova, Italy on 28-29 May 2008 (paralleling the Linnaean Society meeting: *Updating the Linnaean Heritage: Names as tools for thinking about animals and plants*).

  Meeting: http://www.linnean.org/index.php?id=243&tx_ttnews[tt_news]=185&tx_ttnews[backPid]=139&cHash=a228a1b905

  Abstracts: http://www.mapress.com/zootaxa/list/2008/zt01950.html

Document & presentations:

- The documents and presentations of these meetings can be found at:

  http://www.eu-nomen.eu/pesi/index.php?option=com_remository&Itemid=56&func=select&id=76

Configuration History			
Version No.	Date	Changes made	Author
0.8	23 March 2008	Draft agenda	YdJ
0.9	11 August 2008	Final agenda	YdJ
1.0	9 October 2008	Meeting minutes	YdJ
