# Supplementary material for: PESI - a taxonomic backbone for Europe
Source: Biodivers Data J. 2015 Sep 28;(3):e5848. doi: 10.3897/BDJ.3.e5848 (PMC4609752; doi:10.3897/BDJ.3.e5848)
Supplement: Supplementary material 14 — The future of taxonomy – the role of GSD-networks and nomenclators in taxonomic information infrastructure networks (ZooBank). [file biodiversity_data_journal-3-e5848-s014.pdf]

Project no. 018340

Project acronym: EDIT

Project title: Toward the European Distributed Institute of Taxonomy

Instrument: Network of Excellence

Thematic Priority: Sub-Priority 1.1.6.3: "Global Change and Ecosystems"

M3.2.5a The future of taxonomy – the role of GSD-networks and nomenclators in taxonomic information infrastructure networks

Initial scoping meeting on GSDs and nomenclators involvement

Due date of component: Month 24
Actual submission date: Month 24

Start date of project: 01/03/2006 Duration: 5 years

Organisation name of lead contractor for this component: 5 UvA

Project co-funded by the European Commission within the Sixth Framework Programme (2002-2006)		
**Dissemination Level ("X" in the relevant box)**		
**PU**	Public	
**PP**	Restricted to other programme participants (including the Commission Services)	
**RE**	Restricted to a group specified by the consortium (including the Commission Services)	X
**CO**	Confidential, only for members of the consortium (including the Commission Services)	

Revision 1

Table of Content

Table of Content 2
Logistics 3
Venue 3
Attendance 3
Programme 3
Rationale 3
Introduction 4
Organising the European GSDs network 4
GSDs network meeting guidelines 5
PESI admin: 5
Network organisation: 5
Additional local resources: 5
Taxonomic standards: 5
E-infrastructure: 6
Outreach: 6
Contribute to GNA: 6
Organising the European GSDs network 7
GSDs network meeting guidelines 7
PESI admin 7
Nomenclators establishment: 7
Taxonomic standards: 7
E-infrastructure: 8
Outreach: 8
Contribute to GNA: 8
Appendix 1 – Species 2000 europa – Draft list of GSDs 9

European Distributed Institute of Taxonomy (EDIT)

WP3.2 – taxonomic information infrastructure network

The future of taxonomy - the role of GSD-networks and nomenclators in taxonomic information infrastructure networks[1]

Logistics

Venue

Natural History Museum,
Cromwell Road,
London SW7 5BD
United Kingdom

Attendance

European GSDs network coordinator: Thierry Bourgin (MNHN).

European nomenclator representatives: Nicola Nicolson (IPNI), Ellinor Michel (ZooBank), Paul Kirk (Index Fungorum).

Observers/guests: David Remsen (GBIF-ECAT).

PESI project management: Charles Hussey (NHM), Ward Appeltans (VLIZ), Yde de Jong (ZMA).

Programme

Tuesday 18th March 2007: 09:30 – 11:00 hour: PESI management meeting

Tuesday 18th March 2007: 12:00 – 16:00 hour: Role of European GSDs-network

Wednesday 19th March 2007: 09:30 – 14:00 hour[2]: Role of nomenclators

Rationale

The purpose of this meeting will be to have a preliminary scoping of the PESI WP4 working plan, to evaluate the state of affairs of the European GSDs network and nomenclators (in general), to develop plans for deriving (European) taxonomic data standards, to plan for the long term sustainability of taxonomic resources, and to discuss the contribution of the respective networks to the Global Names Architecture (GNA).

[1] A PESI WP4 preparatory meeting: PESI WP4 fulfils components of the EDIT WP3.2 objectives.
[2] To be continued in the pub during/after lunch if necessary.

Introduction

A joint EDIT / PESI objective is to advance the set up of an infrastructure of European species databases to fulfil Europe's contribution to worldwide species list initiatives by establishing a secure organisation and management for European biodiversity information databases and repositories, including the founding of a pan-European checklist. This objective covers a range of EDIT ambitions in common with:

◇ developing an integrated pan-European species databases e-infrastructure,

◇ establishing improved research collaboration through the set up of a common management approach for the maintenance and updating of European species-lists,

◇ bringing the taxonomic (digital) data content to a next (higher) level of completeness, quality, standardisation, consistency, integration and incorporation.

In general the recognized working program moves along four lines:

◇ organising and using the expert(ise) networks,

◇ developing and advancing the e-infrastructure,

◇ enforcing data validation and promoting (European) standardisation,

◇ arranging national or regional focal points into a partnership structure.

Organising the European GSDs network

The European GSDs network contains taxon-based capacity related to the Species 2000 Global Species Databases infrastructure. The Species 2000 europa network has been invited to participate in PESI. Thierry Bourgin has agreed to coordinate the EU-based GSD networking organization. This includes supporting the main objectives of PESI on integrating and securing all Europe based GSDs by helping them to cooperate and to adopt common standards, by helping them to access to local expertise to enhance their content and to reach interoperable facilities. PESI will also help GSDs to follow best practice in their field and to participate into the Global Names Architecture for a more efficient and unambiguous data mining of names of organisms. It will also help the Society for the Management of European Biodiversity Data (SMEBD) to overcome the problem of lost and orphaned databases and to develop Integrated IPR and proper attribution and accreditation for electronic taxonomic data.

A draft list of European GSDs can be found in appendix 1.

GSDs network meeting guidelines

After an introduction (by Thierry) on the state of affairs of the European GSDs network we shall address a range of items related to the PESI working program, including:

PESI admin:

- Administrative arrangements (Consortium Agreements, budget, personnel, allowances for travel).
- Timetable and involvement in delivering WP4.

Network organisation:

- Long-term network set up and maintenance. Common businesses plan development on the sustainability of European GSDs (collaborate with PESI WP5).
- Set up of an EU GSDs member board/council to support working program drafting and networking activities.
- SMEBD membership of GSDs experts and custodians to support an integrated IPR policy for electronic taxonomic data acknowledgement and to develop additional 'models of recognition'.
- Establish working groups to support for instance (1) Common Data Model reviewing, (2) unified management of the classification progress, (3) EU FP7 proposal drafting.
- *EU-nomen* consortium membership.
- Explore the potential for using international expertise to support GSDs managed within the EU.

Additional local resources:

- Develop models to increase the number of GSDs by searching local expertise networks (collaborate with PESI WP3).
- Develop models to help GSDs to get access to local expertise to enhance their respective GSDs (collaborate with PESI WPs 2&3).

Taxonomic standards:

- Consider which additional steps need to be taken to derive authoritative European taxonomic standards.
- For instance: (1) common data (type/field) terminology, vocabulary & definition, (2) set up of authority files, (3) contribute to unified higher hierarchy. Collaborate with nomenclator and pan-EU checklists. Collaborate with PESI WP4 general program. Collaborate with TDWG.

- Harmonised (European) taxonomic meta-data: review/validate GSDs against nomenclators and pan-EU checklists. Collaborate with PESI WP4 general program.

E-infrastructure:

- Encourage GSDs to consider alternative information facilities, like the GBIF/EDIT Global Bee project (http://globalbees.editwebrevisions.info/), to organise their expertise communities.
- Develop a relevant e-infrastructure to support the implementation and long term sustain of small and middle-sized GSDs (for instance the 'data warehouse model').
- Define further e-Science network development for integrated access to GSD data. Should PESI give access to the GSDs via the public web portal?

Outreach:

- Promote the existence of GSD's to stakeholders and the wider community.

Contribute to GNA:

- Catalogue resources.
- Synchronised indexing /cross-index GUIDs.
- Identify priority / example / pilot data sets.
- Joint program on vernacular names.
- Provision of direct access by GBIF to GSDs

Organising the European GSDs network

PESI considers nomenclators to play a central role in the future of e-taxonomy and to function as an objective cross-reference layer in the 'Global Names Architecture' and associated systems (e.g. library systems). It will be a challenge for the systematic community to have nomenclator services installed as mandatory registration systems for nomenclatural acts, including complete, authoritative records of all scientific names. This system will prepare Europe for emerging virtual workbench developments, notably web-based taxonomy.

PESI will contribute to this ambition by enhancing the functionality of the respective nomenclators, supporting their integration within the 'Global Names Architecture', as well as supporting the standardisation and harmonisation of European taxonomic meta-data.

GSDs network meeting guidelines

After introductions by the nomenclator representatives on the state of affairs of their respective databases, we shall address a range of items related to the PESI working program, including:

PESI admin

- Administrative arrangements (Consortium Agreements, budget, personnel, allowances for travel).
- Timetable and involvement in delivering WP4.

Nomenclators establishment:

- Models for long-term sustainability (common businesses plan development).
- Expertise/community network (taxonomic societies, etc.) involvement.
- Uploading ZooBank.
- Contribute to future EU FP7 proposal drafting.
- SMEBD membership to support an integrated IPR on electronic taxonomic data acknowledgement'.
- *EU-nomen* consortium membership.

Taxonomic standards:

- Consider which additional steps need to be taken to derive authoritative European taxonomic standards.
- Contribute to PESI WP4 program on taxonomic standardisation (see also above).

- Harmonised (European) taxonomic meta-data: review/validate nomenclators against pan-EU checklists. Collaborate with PESI WP4 general program.

E-infrastructure:

- Define tools to facilitate data input (validation / uploading / submission of nomenclatural acts / fixation to type specimens / etc.) of expertise communities.
- Define further informatics and e-Science network developments to enhance the nomenclators functionality.

Outreach:

- Promote the existence of the nomenclators (especially the improvements derived through PESI) to stakeholders and the wider community.

Contribute to GNA:

- Synchronised indexing /cross-index GUIDs.
- Define nominal taxa as anchor points within GNA
- Identify priority / example / pilot data sets.
- Joint program on vernacular names.

Configuration History			
Version No.	Date	Changes made	Author
0.9	10 March 2008	Draft agenda	YdJ
1.0	11 March 2008	Final agenda	YdJ

Appendix 1 - Species 2000 europa - Draft list of GSDs

Institute / Organisation	Custodian	GSD Name
Zool. Stuttgart	Haeuser Steiner	• GART/GLOBIS
MNHN Paris	Bouchet, Bourgoin, Soulier-Perkins	• CLEMAM<br>• FLOW<br>• COOL
NHM London	Scoble, Robinson, Beccaloni, Noyes, Pitkin, Lyal	• Tineids<br>• LepIndex & BSF<br>• Chalcids<br>• WTAXA
Naturalis	Tol, van derLand	• Odonata • URMO
CABI	Kirk	• Sp Fungorum<br>• Glomeromycota<br>• Phyllochorales<br>• Trichomycetes<br>• Xylariaceae<br>• Zygomycetes<br>• Rhitismatales
Kew Garden	Paton, Govaerts	• Kew Global List
Univ Wageningen & Vienna	Chatrou, Rainer	• Annonbase
Utrecht (Veterinary)	Jongejan, Nijhof	• TicksBase
BGBM Berlin	Berendsohn (Gebhardt)	• IOPI
NUI Galway	Guiry	• Algaebase
Univ Paris 6	Vignes-Lebbe	• CIPA
IFM Geomar	Froese	• Fishbase
ILDIS/ Wageningen	Van der Maesen, Bisby, Roskov	• ILDIS
ITG, Karlsruhe	Uetz, Hallermann	• TIGR Reptiles
BSM Munich	Triebel	• LIAS
Zoology, Warsaw	Bogdanowicz, Proszynski	• Spider
Univ Linz	Malicky, Aubrecht	• ZOBODAT
DSMZ, Braunschweig	Kracht, Erko, Stakesbrandt	• DSMZ
Museum of Wales	Seddon	• MolluscaFW
IRD	Tavakilian, Peinaherraris-Leiva	• TITAN
CSIC Madrid	Zarazaga, Ramos, Lyal	• WTAXA
Univ Amsterdam	de Kluijver, S.S. Ingalsuo	• Euphausidae
CVS Utrecht	Stalpers, Crous	• Species Fungorum
Univ Amsterdam	Oosterboek	• CCW Craneflies
Private	Schoolmeesters	• Scarabs
ZSM, Munich	Spelda	• SysMyr
Univ Padova	Minelli	• ChiloBase
Univ Amsterdam	Van Soest	• Porifera
Individual	Farjon	• Conifer DB
RBINS, Brussels	Segers	• Rotifera
CSIC, RJB	Aedo	• RJB Geranium<br>• Eumycetozoa.com
Tervuren	De Prins	• World Gracillariidae

Project no. 018340

Project acronym: EDIT

Project title: Toward the European Distributed Institute of Taxonomy

Instrument: Network of Excellence

Thematic Priority: Sub-Priority 1.1.6.3: "Global Change and Ecosystems"

M3.2.5b The future of taxonomy – the role of GSD-networks and nomenclators in taxonomic information infrastructure networks

Scoping meeting on ZooBank involvement

Due date of component: Month 29
Actual submission date: Month 29

Start date of project: 01/03/2006 Duration: 5 years

Organisation name of lead contractor for this component: 5 UvA

Revision 1

Project co-funded by the European Commission within the Sixth Framework Programme (2002-2006)		
**Dissemination Level ("X" in the relevant box)**		
**PU**	Public	
**PP**	Restricted to other programme participants (including the Commission Services)	
**RE**	Restricted to a group specified by the consortium (including the Commission Services)	X
**CO**	Confidential, only for members of the consortium (including the Commission Services)	

Meeting details:

- The future of taxonomy - the role of nomenclators in taxonomic information infrastructure networks: scoping meeting on ZooBank involvement, Padova, Italy on 28-29 May 2008 (paralleling the Linnaean Society meeting: *Updating the Linnaean Heritage: Names as tools for thinking about animals and plants*).

Minutes / Results:

- Meeting:

  http://www.linnean.org/index.php?id=243&tx_ttnews[tt_news]=185&tx_ttnews[backPid]=139&cHash=a228a1b905

- Abstracts:

  http://www.mapress.com/zootaxa/list/2008/zt01950.html

- Relevant contributions:

  ZooBank: Developing a nomenclatural tool for unifying 250 years of biological information — RICHARD L. PYLE & ELLINOR MICHEL
  http://www.mapress.com/zootaxa/2008/f/zt01950p050.pdf (*Zootaxa* **1950**: 39-50)

  Actual usage of biological nomenclature and its implications for data integrators; a national, regional and global perspective — CHARLES HUSSEY , YDE DE JONG & DAVID REMSEN
  http://www.mapress.com/zootaxa/2008/f/zt01950p008.pdf (*Zootaxa* **1950**: 5-8)

Configuration History			
Version No.	Date	Changes made	Author
1.0	28 May 2008	Contributions submitted	YdJ
