# Supplementary material for: PESI - a taxonomic backbone for Europe
Source: Biodivers Data J. 2015 Sep 28;(3):e5848. doi: 10.3897/BDJ.3.e5848 (PMC4609752; doi:10.3897/BDJ.3.e5848)
Supplement: Supplementary material 15 — Application and Adoption of Taxonomic Standards [file biodiversity_data_journal-3-e5848-s015.pdf]

D4.3 — Application and Adoption of Taxonomic Standards

Table of Contents

Application and Adoption of Taxonomic Standards 1
1 Introduction 1
2 TDWG Montepellier Declaration 2
3 Globally Unique Identifiers 2
4 RDF and Semantic Technologies 3
5 Darwin Core Archive Format 3
6 Adoption 4
7 Application Outside the Taxonomic Community 4
8 Potential Impact on Smaller Data Providers 5
9 Combined Formal Classification 6
10 Informal Classification 7
Glossary 8
Appendix 1: Montpellier Taxonomic Declaration 11
Appendix 2: Applicability Statement for the Darwin Core Archive Format in Europe 12
1 What Does a DwC-A File Consists Of? 12
2 Simplest Authoring Scenario 12
3 Taxonomic Hierarchies 13
4 Multiple hierarchies 13
5 Columns in the Spreadsheet 13
6 Names vs Taxa 15
7 Additional Nomenclatural Information 16
8 Default Values 16
9 Vernacular Names 16
10 Distributions 16
11 Taxon Identifiers 17
12 Provenance Data 17
13 RDF and the Semantic Web 17

1 Introduction

This is the third report of Work Package 4 of the Pan European Species dictionaries Infrastructure (PESI). Its purpose is to report on how taxonomic standards will be adopted.

Previous reports have outlined the available standards and how they should ideally be adopted. In contrast this report can be seen as a 'reality check' – outlining what is actually likely to be achieved within the lifetime of this round of PESI funding but with and eye to the future.

There are two clearly differentiated types of taxonomic standard:

1. **Taxonomic data exchange standards.** These standards are concerned with how taxonomic data, such as classifications and checklists, are exchanged.
2. **Standardised taxonomies.** Classifications of accepted taxa and rejected or synonymised names.

PESI as a whole is concerned with producing a standardised taxonomy (2) in the form of a dynamic, annotated checklist of species for Europe. This report (and WP4 in general) is primarily concerned with data exchanged standards (1) i.e. how the taxonomists will exchanged data so as to build the standardised taxonomy and how this standardised taxonomy will be published for non-taxonomists to incorporate into their analyses. WP4 **does** have responsibility for facilitating the construction of a single formal and informal classification – and there are section in the report on this work.

Note: Extensive use of acronyms is made here. Expanding these acronyms frequently fails to communicate their meaning for non-experts whilst making the document harder to read. A glossary has therefore been provided as an alternative to expansion of acronyms even on first use.

2 TDWG Montepellier Declaration

Many of the key stake holders in the biodiversity informatics global community were present at the TDWG meeting in November 2009 in Montpellier. There was a general agreement on the way forward for sharing taxonomic data and so PESI WP4 took the opportunity to capture this by asking those involved to sign up to a loose agreement on the way forward. Signatories included representatives from major stakeholders including Atlas of Living Australia, CATE, the DwC maintainers, EDIT(CDM Team), EDIT(Scratchpads Team), ETI, GBIF, IPNI and ZooBank. The Encyclopaedia of Life also expressed and interest in being involved. The full text and signatories are given in Appendix 1.

The key agreement was that publishing data would involve a combination of the following components.

1. Lists of taxa in text files in accordance with the DwC archive format.
2. HTTP URIs for taxa that are "Linked Data" compliant and return RDF using an agreed vocabulary.
3. Inclusion of standardised HTTP URI for shared taxon names e.g. from nomenclators
4. Links to other classifications also published on the web.

3 Globally Unique Identifiers

Numbers 2 ,3 and 4 on the list of components in the Montpellier declaration require the implementation of resolvable, persistent, globally unique identifiers in the form of HTTP URIs. Number 1 does not require them although can exploit them if they are present.

Within PESI, the nomenclators (IPNI, Index Fungorum, ZooBank) have implemented globally unique identifiers in the form of LSIDs for names. The nascent GNA will make use of them but the main taxonomic databases (Fauna Europea, Plant Base and ERMS) do not currently publish taxon data tagged with them. There are discussions in progress as to how GUIDs will be implemented in the PESI portal and between the key databases.

These discussion are scheduled to continue in May 2010 at a meeting in Amsterdam of work packages 4, 5 and 6.

A major hurdle to adoption of GUIDs is the requirement to commit resources for the foreseeable future. Data must be hosted live on the internet for periods of time that are orders of magnitude greater than the length of funded projects. Even though many of the projects are hosted in institutions (such as museums, botanic gardens and universities) that have been curating data in the form of libraries and collections for hundreds of years there is not yet a will at the institutional level to commit to hosting data in perpetuity. Without this commitment from a significant proportion of institutions widespread adoption of GUID tagged data is not possible.

By the end of this round of PESI funding, in April 2011, the PESI partners may have come to an agreement for persistent hosting of data for the core databases and implemented it. It is unlikely though that the same approach will be suitable for integration of GSDs in smaller institutions around Europe. The potential tools that rely on the presence of these GUIDs can't take off until those taxon GUIDs are available. It is unlikely, therefore, that GUID based technologies will emerge within the next 12 months.

Following discussions the WP 4-5-6 meeting in Amsterdam in May 2010 the following course of action was developed:

- The existing internal identifiers in ERMS and PlantBase will be propagated to the portal data warehouse.
- Fauna Europaea will implement internal identifiers and propagate them to the data warehouse.
- The portal will provide URIs terminating with internal IDs from contributing databases that returns data. The format of this data is unspecified but will most likely be Linked Data compliant.
- A formal requirements gathering process is needed for "public" identifiers for taxa in the future. These could be similar to the Taxon Serial Numbers (TSNs) used by ITIS. This is needed to establish the behaviour required by non-taxonomy stakeholders. This process is out of scope for this round of funding but should be considered for future funding rounds.

4 RDF and Semantic Technologies

RDF, Linked Data and the Semantic web rely on the availability of data at stable GUIDs in the form of HTTP URIs. As it is unlikely that GUIDs for taxa will be available in the near future adoption of these technologies is likely to remain experimental over the next 12 months. This does not preclude the use of semantic technologies such as OWL for analysis of data but conversion of 'raw' (non-RDF) data to RDF or OWL ontologies is likely to occur when the data is consumed rather than as it is published. There are opportunities for third parties to take published taxonomic data and render is as RDF for further analysis. In the broader web community attempts at this have already occurred with dbpedia.org, freebase.com and sharednames.org although the success of these projects is debatable.

5 Darwin Core Archive Format

The lack of widespread adoption of GUIDs (and even web services) by taxonomic (as

opposed to nomenclatural) data providers precludes the use of technologies other than via the exchange of files. As the Montpellier Agreement highlights the DwC Archive format is the preferred exchange format for taxonomic data and its adoption is widely supported by different providers — notably the GBIF ECAT project. DwC-A is advantageous in that it can be produced and consumed without specialist software yet can facilitate automation in more complex environments such as portals. **DwC-A should therefore be adopted as the default data representation for exchange within PESI.** Appendix 2 is an applicability statement specifying how DwC-A should be used.

6 Adoption

With a little help anyone with a spreadsheet program (such as Excel or OpenOffice) and a text editor should be able to produce DwC-A files of taxonomic data and submit them to a repository. **There are therefore no technical barriers to adoption of this technology.** Taxonomic data suppliers merely need to:

1. Produce regular snapshots of their preferred classifications in DwC-A format.
2. Clearly label these with a widely sharable license, author and publication date.
3. Submit them to the PESI Portal.
4. Submit them to the GBIF ECAT repository and any other available repositories.

The appended applicability statement (Appendix 2) in combination with the documentation available from GBIF (http://code.google.com/p/gbif-ecat/wiki/publishingChecklists) should be sufficient for many data suppliers and consumers. It may be necessary to provide tailored training and documentation to some suppliers but these needs will have to be devised and resourced on a per supplier basis.

Larger taxonomic data suppliers or groups of suppliers may consider setting up an instance for the GBIF IPT to publish their checklists. It may be appropriate for PESI to establish such an instance to manage data publishing.

Remaining barriers to exchange of primary taxonomic data within the taxonomic community are likely to be social, particularly around licensing and attribution.

7 Application Outside the Taxonomic Community

Adoption of these standards outside of the taxonomic community, with stakeholders in the wider scientific and commercial world, is harder to predict. If taxonomists can put there own house in order as regards consistent representation of their research results (as suggested here) then this provides a firm foundation on which to build services for the wider community. This wider community is unlikely to be interested in details of taxonomy and nomenclatural and are more likely to be interested in task specific services. Supplying these services should be seen as a separate process from carrying out taxonomic research.

An example of interaction between taxonomy (particularly nomenclature) and the wider community is given by the European Water Vole. The preferred latin binomial for this is *Arvicola amphibius*. Until recently it was called *Arvicola terrestris*. In 1758 Linnaeus created two names that have for many years now been considered to apply to the same species. Because the interpretation of the intervening publications changed the name for the species changed. Note that the understanding of the biology did not change. The result is that all assets (legislation, publication, signage etc) that use *A. terrestris* is now

technically 'wrong'. If it is OK for everything that was called *A. terrestris* to now be called *A. amphibius* it is hard to justify to an outside world why they should dedicate resources to changing the name. If the words mean the same it doesn't matter which is used but if it does matter which is used it implies the words have different meanings and can't be arbitrarily changed (Catch-22). If the needs of the wider community had been considered greater than the need to maintain compliance with the zoological code of nomenclature then the name may not have been changed. An outsider may consider this a case of “the tale wagging the dog”. The English vernacular name “European Water Vole” could be considered more stable than the scientific name.

What is required is a user focussed (rather than taxonomist focussed) requirements gathering process. On the basis of the identified requirements services can be designed to act as an interface between taxonomic research and the wider user community. Without this interface volatility in taxonomic research will hamper the work of the wider community and the need for stability in the wider community will hamper research in taxonomy. This requirements gathering process is non-trivial.

8 Potential Impact on Smaller Data Providers

Some data providers have expressed concerns over the way larger projects exploit the data they produce when there isn't necessarily a mechanism to 'trickle down' resources. There is a fear that future funding efforts may be directed to large, high profile projects whilst the smaller projects they depend are starved of resources. The result would be an overall loss of data quality and expertise.

Examples of larger projects include PESI, GBIF and EoL. Smaller data providers are those that are typically run by one or two academic researchers on limited budgets and often not on a full time basis.

Even when data aggregators correctly attribute the sources of their data the latency times mean that corrections made at source are not propagated to the aggregator rapidly enough and the data providers feel misrepresented – thus further effecting their chances of attracting further funding. This could be further exasperated if the funding for the aggregator dries up and data is maintained on line but not updated at all.

It is not WP4's role to address these social and economic issues. The data standards can, however, have an impact in this area.

An analogy can be made with car manufacturers. A large company like the Volkswagen Group (VW) is highly dependent on having a healthy ecosystem of smaller parts suppliers. The public tend to deal directly with VW for purchase and servicing of their vehicles. A proportion of the purchase costs of the vehicle is passed down the supply chain to the parts suppliers because VW pay them for the parts. Even with this economic mechanism there is a danger that parts suppliers become dependent on a single large manufacturer who will then drive down their margins and possibly put them out of business. Parts suppliers can avoid this by making their products as generic as possible so that they can supply not only multiple car manufactures but also sell directly to independent garages and consumers.

In biodiversity informatics there is currently no financial trickle down of money from aggregators to data suppliers. Any funding that is supplied is typically for the implementation of data harvesting interfaces not production and maintenance of data. The free exchange of this kind of data is cultural and is unlikely to change.

Where taxonomic standards can have an impact is in allowing data suppliers to diversify. If they make their data available on the web in DwC-A format it can be used by multiple data aggregating projects as well as directly by consumers. The provenance information within the archive file can ensure that the provider is correctly acknowledged for the data. It can also make clear the version of this particular data set and let the end consume know where they can get the latest version i.e. directly from the data supplier. If there is latency between improvements in the data and those improvements appearing in aggregated data sources it is apparent to end consumer.

Taxonomic data exchange standards therefore have a potential empowering role for the smaller data providers.

9 Combined Formal Classification

The three main source databases (Plant Base, Fauna Europaea and ERMS) each have their own higher classifications (i.e. classifications above the rank of genus). When they are combined into a single database in the portal these classifications need to be reconciled to produce a single hierarchy. Because the databases cover very different groups of organisms there is little overlap at the species level. There is no overlap between PlantBase and Fauna Europaea. There is very little between PlantBase and ERMS. There **is** overlap between Fauna Europaea and ERMS as there are higher taxa of animals that have both marine and terrestrial or fresh water species. There are three main problems that arise.

- Duplicate higher taxa – taxa with the same names occurring in both databases.
- Taxa having different parents in the two databases.
- Missing ranks e.g. Genera in the two databases will be in the same family but in one of the databases a subfamily is interposed between the family and genus.

Some issues of producing a single hierarchy can be resolved heuristically by the merging algorithm. Other issues require human interaction and can only be resolved by taxonomic experts. At the meeting of WP 4-5-6 in Amsterdam in May 2010 the following course of action was developed.

- The results of the current automated merging is not visible in the portal. The next major update will removed duplicate entries. This process will favour ERMS in conflict resolutions and will be in place by June 2010.
- Around 400 known conflicts have been identified that will be manually reconciled in ERMS (without specialist input) so that they are not present in next data merge. This takes the data to the stage where purely technical fixes are no longer possible.
- A report (as a spreadsheet) will be generated as part of the merge process for circulation to experts for feedback. Suggested changes will be incorporated into source databases so that they propagate to the portal. They must be incorporated both relevant source database where there is an overlap. This will be on-going, long term process as the classifications will continue to develop but it should remove conflicts between source databases and result in a single European consensus taxonomy in the long run.

10 Informal Classification

Formal taxonomic hierarchies are informative for biologists but may be of little use to non-biologist or even biologists who specialise in different taxonomic groups. It is therefore important that species lists can be retrieved by commonly recognised groups with vernacular names. Examples are “Marine Invertebrates” or “Butterflies”.

Previous work, carried out as part of the UK National Biodiversity Networking (NBN), has arrange all UK higher taxa by commonly recognised groups. This is valuable work because it was based on an initial survey of group names in actual use.

As an outcome of the WP 4-5-6 in Amsterdam in May 2010 Charles Hussey agreed to make these groupings available for incorporation into the portal and to discuss them at the up coming Focal Points meeting in Turkey for their international applicability.

Glossary

Atlas of Living Australia – The Atlas of Living Australia is a five-year project funded under the Australian Government's National Collaborative Research Infrastructure Strategy. Its mission is to develop a biodiversity data management system which will link Australia's biological knowledge with its scientific and agricultural reference collections and other custodians of biological information. http://www.ala.org.au/

CATE - The Creating a Taxonomic e-Science project, funded by the United Kingdom's Natural Environment Research Council (NERC) under its e-science initiative. The particular goal of CATE is to test the feasibility of creating a web-based, consensus taxonomy using two model groups, one from the plant and the other from the animal kingdom. The wider aim is to explore practically the idea of 'unitary' taxonomy and promote web-based revisions as a source of authoritative information about groups of organisms for specialist and non-specialist users. http://www.cate-project.org/

CDM – see EDIT CDM

Drupal – An open source content management platform used to create websites.

DwC – Darwin Core. Darwin Core is a Biodiversity informatics data standard that consists of a vocabulary of terms to facilitate the discovery, retrieval, and integration of information about organisms, their spatiotemporal occurrence, and the supporting evidence housed in biological collections. DwC is a TDWG standard. http://en.wikipedia.org/wiki/Darwin_Core

DwC-A – Darwin Core Archive. DwC-A is a derivative of the DwC standard developed by GBIF to facilitate the exchange of checklist data.

EDIT – European Distributed Institute of Taxonomy. A network of excellence gathering 28 major institutions devoted to knowing the living world better with the support of the European Commission. http://www.e-taxonomy.eu/

EDIT CDM – The EDIT Common Data Model is the domain model for the core EDIT components. It has been instantiated as a Java base API over a relational data model for embedding within different taxonomy based projects. http://dev.e-taxonomy.eu/trac/wiki/CommonDataModel

EDIT Scratchpads - Scratchpads are an easy to use, social networking application that enable communities of researchers to manage, share and publish taxonomic data online. Sites are hosted at the Natural History Museum London, and offered free to any scientist that completes an online registration form. http://scratchpads.eu

Encyclopaedia of Life – An international, but largely USA based, project to create a web page for every species inspired by the ecologist E.O. Wilson.

ETI Bioinformatics – ETI develops and produces scientific and educational computer-aided information systems. http://www.eti.uva.nl/

GBIF – Global Biodiversity Information Facility. An international government-initiated and funded initiative focused on making biodiversity data available to all and anyone, for scientific research, conservation and sustainable development. The GBIF Secretariat is based in Copenhagen. The GBIF Data Portal indexes many

millions of data points per year. http://www.gbif.org/

GBIF ECAT – Electronic Catalogue of Names of Known Organisms

GBIF IPT – Integrated Publishing Toolkit. The GBIF IPT is an open source, Java (TM) based web application that connects and serves three types of biodiversity data: taxon primary occurrence data, taxon checklists and general resource metadata. The data registered in a GBIF IPT instance is connected to the GBIF distributed network and made available for public consultation and use. http://code.google.com/p/gbif-providertoolkit/

GSD – Global Species Databases are the building blocks of the Species2000/Catalogue of Life data set. They are typically taxon specific (occasional region specific) databases of names managed by experts or institutions.

GUID – Globally Unique Identifier. Within the biodiversity informatics domain GUID has been used to mean a resolvable or actionable identifier that has global scope. An HTTP URI is an example of this. In the wider computing community it is often used as a synonym for Universally Unique Identifier (UUID) which are essentially large random numbers. Anyone can create a UUID and use it to identify something with reasonable confidence that the identifier will never be unintentionally used by anyone for anything else but UUIDs don't have an associated dereferencing mechanism i.e. they can't be used as an address to look up normative information about what they identify.

HTTP URI – An HTTP URI is a web address or name starting with 'http://' See also HTTP and URI.

HTTP – Hypertext Transfer Protocol. An application layer protocol for distributed, collaborative, hypermedia information systems. It is the key technology that turns the Internet into the World Wide Web. http://en.wikipedia.org/wiki/HTTP

Index Fungorum – A fungal nomenclator (names and associated bibliographical data) currently co-ordinated and supported by CABI, CBS and LCR. http://www.indexfungorum.org/

IPNI – The International Plant Names Index (IPNI) is a database of the names and associated basic bibliographical details of seed plants, ferns and fern allies. http://www.ipni.org/

ITIS – Integrated Taxonomic Information System. A partnership of USA federal agencies formed to satisfy their mutual needs for scientifically credible taxonomic information. http://www.itis.gov/

Linked Data - The term Linked Data is used to describe a method of exposing, sharing, and connecting data via dereferenceable URIs on the Web. http://linkeddata.org/

OWL – The Web Ontology Language. OWL is a family of knowledge representation languages for authoring ontologies, and is endorsed by the World Wide Web Consortium http://en.wikipedia.org/wiki/Web_Ontology_Language

RDF – Resource Description Framework. A family of World Wide Web Consortium (W3C) specifications originally designed as a metadata data model. It has come to be used as a general method for conceptual description or modelling of information that is implemented in web resources, using a variety of syntax formats. http://en.wikipedia.org/wiki/Resource_Description_Framework

Semantic Technologies – The collections of technologies used to implement the Semantic Web. Core technologies are HTTP URIs and RDF.

Semantic Web - An evolving development of the World Wide Web in which the meaning (semantics) of information and services on the web is defined, making it possible for the web to ‘understand’ and satisfy the requests of people and machines to use the web content. http://en.wikipedia.org/wiki/Semantic_web

TDWG (Biodiversity Standards) – A not for profit scientific and educational association that is affiliated with the International Union of Biological Sciences. TDWG was formed to establish international collaboration among biological database projects. TDWG promotes the wider and more effective dissemination of information about the World's heritage of biological organisms for the benefit of the world at large. TDWG focuses on the development of standards for the exchange of biological/biodiversity data. http://www.tdwg.org/

URI – Uniform Resource Identifier. A string of characters used to identify a name or a resource on the Internet. http://en.wikipedia.org/wiki/Uniform_Resource_Identifier

ZooBank – ZooBank is intended as the official registry of Zoological Nomenclature, according to the International Commission on Zoological Nomenclature (ICZN). http://www.zoobank.org/

Appendix 1: Montpellier Taxonomic Declaration

We intend to promote the sharing of taxonomic data (lists of names and taxa) by attempting to publish them in a unified way.

Publishing a classification or taxonomic checklist on the web involves some or all of the following components

- Lists of taxa in text files in accordance with the DwC archive format.
- HTTP URIs for taxa that are "Linked Data" compliant and return RDF using an agreed vocabulary.
- Inclusion of standardised HTTP URI for shared taxon names e.g. from nomenclators
- Links to other classifications also published on the web.

How publication is achieved for any one dataset will depend on the abilities of the data provider. Various resources are needed to make progress.

- A set of guidelines or best practice documentation
- Validation services to check that published data meets the guidelines.
- Promotional material (docs, talks, challenges) to encourage the exploitation of published taxonomies.

We will cooperate in creating these resources.

Things that it would be nice to happen before the next TDWG meeting and that we will attempt to achieve are:

- Drupal plug-in to help the Drupal powered systems (Scratchpads, Lifedesks etc) meet the guidelines.
- Adaption of the CDM or CDM portal component to meet the guidelines.
- Adaption of the IPT to meet the guidelines.

Signatories

Andreas Kohlbecker <a.kohlbecker@BGBM.org> - EDIT/CDM
Clark, Benjamin R <b.clark@rbgkew.org.uk> - CATE/CDM
[Cynthia Parr <parrc@si.edu> - possible adoption by EoL]
David Remsen <dremsen@gbif.org>- GBIF
Donald Hobern <Donald.Hobern@csiro.au> - ALA
John Wieczorek <tuco@berkeley.edu> - IPT/DwC
Markus Döring (GBIF) <mdoering@gbif.org> - GBIF
Nicola Nicolson <n.nicolson@rbgkew.org.uk> - IPNI
Rich Pyle <deepreef@bishopmuseum.org> - ZooBank
Roger Hyam <roger@hyam.net> - PESI
Simon Rycroft <s.rycroft@nhm.ac.uk> - ScratchPads
Wouter Addink <wouter@eti.uva.nl> - ETI

Appendix 2: Applicability Statement for the Darwin Core Archive Format in Europe

The DwC-A is a derivative of the DwC TDWG standard developed by GBIF to facilitate the exchange of checklist data. It is primarily a mapping of the DwC fields into a CSV file format (as defined within DwC) with an extension mechanism to allow the addition of other fields for taxonomic data. (http://code.google.com/p/gbif-ecat/wiki/publishingChecklists). The DwC-A is supported by the GBIF IPT but is simple enough to be created without specialist software, such as via a database dump or spreadsheet export.

This applicability statement provides a description of how the DwC-A format could be used to exchange basic taxonomic checklists under the PESI umbrella. There may be other fields that need discussion and this will be examined at the work package meeting in Amsterdam.

1 What Does a DwC-A File Consists Of?

A DwC-A file is a Zip compressed archive of files within a single directory. There are only two required files but here we recommend a minimum of three.

- Core Taxon File (taxa.txt) – a CSV file of the taxa with one row per taxon in the source data set.
- Descriptor File (meta.xml) – an XML file that describes the contents of the taxa.txt file plus any other files. The structure of this file is quite simple and consists of a basic list of the columns in the taxa.txt file with the separator and escape characters used.
- Dataset Provenance (provenance.xml) – a simple XML file that specifies the creator, created date, publisher, rights and licensing for the data in the archive.

A DwC-A may contain other files.

- Extension files – these are CSV files containing data that relates to the taxa defined in the taxa.txt file. They allow one to many relationships to be expressed from taxa to data in extension files such as listing type specimens or vernacular names in multiple languages.
- Dataset Information. A file containing information about the provenance of the data set may be included. The preferably format for this is Ecological Markup Language (EML). EML is an extensible language for describing the contents of datasets.

2 Simplest Authoring Scenario

In the simplest case a DwC-A **could** be produced following these steps.

1. Create a spreadsheet with the correct columns in the correct order using a programme such as MS Excel or Open Office.
2. Export the spreadsheet as a CSV file to a new directory.
3. Add the supplied meta.xml and provenance.xml files.

4. Edit the provenance.xml file to include creation and creator details.
5. Zip up the directory by right clicking on it and selecting 'Send to ...' zip folder on a Windows machine or create 'Compress “folder name” on a Mac or equivalent command on a Linux.

In database driven environments this process could be automated or carried out on demand. The GBIF IPT also provides functionality to support publishing checklists in this format.

3 Taxonomic Hierarchies

There is no need for a DwC-A to contain any hierarchical data. It can contain a list of species without reference to a higher classification at all. This is probably a rarer case as most databases will contain some notion of higher taxonomic placement even if that is only to family or order level.

There are two ways that a taxonomic hierarchy can be represented in DwC-A.

- **Normalised**. In this form each taxon row in the file has a parentNameUsageID field that contains the taxonID of the next higher taxon in the classification. A species row may have the id of the genus row in the file for example. A consuming application can reconstruct the hierarchy by following all these child -> parent links and creating their **reverse** parent->child likes.
  A normalised approach has the advantage that it can handle any number of arbitrary ranks.
- **Flattened.** There are a series of fields available for the major ranks as well as a 'higherClassification' field for providing a semicolon separated list of higher taxa. These fields can be populated for each row with the names of the higher taxa that that particular row is placed in. This approach has the advantage of being simpler to implement but lacks the expressivity of the Normalised approach.

Recommendation: DwC-A files should only contain one approach to encoding the taxonomic hierarchy: either include the parentNameUsageID, or the kingdom, phylum, class, order, family, genus, subgenus, higherClassification fields but not both. This avoids the confusion of possible conflicting or ambiguous classifications.

The list of recommended fields given below is split into two. One for the Normalised appoach and one for the Flattened approach.

4 Multiple hierarchies.

It is possible for a single taxon.txt file to contain multiple hierarchies of accepted taxa i.e. multiple classifications. There are a minority of receiving applications that are likely to be able to understand and import multiple classifications.

Recommendation: DwC-A files should only contain a single accepted classification. Multiple classifications should be split between different archives to allow people to import a single classification at a time.

5 Columns in the Spreadsheet

This section defines the columns that should be included in taxonomic checklists. The first part lists the columns common to both Normalised hierarchical approach and the

Flattened approach. The second and third sections are specific to the two approaches.

Common Columns

Name	Notes
dwc:taxonID	A unique ID for this taxon or name. At a minimum this should be unique within this particular DwC-A; ideally it should be a GUID and resolvable URI. (See discussion below). Required.
dwc:scientificName	The full taxon name (with authorship and date information if applicable). Required.
dwc:scientificNameID	Exclusively used to reference an external and resolvable identifier that returns nomenclatural (not taxonomic) details of the name. This is a link to a nomenclator. Current examples are Index Fungorum, IPNI and ZooBank LSIDs. Optional.
dwc:genus	The full scientific name of the genus in which the taxon is classified. Optional.
dwc:subgenus	The full scientific name of the subgenus in which the taxon is classified. Optional.
dwc:specificEpithet	The name of the species epithet of the scientificName. Optional.
dwc:infraspecificEpithet	The name of the infra-species epithet of the scientificName. The name of the lowest or terminal infraspecific epithet of the scientificName, excluding any rank marker. Optional.
dwc:scientificNameAuthorship	The authorship information for the scientificName formatted according to the conventions of the applicable nomenclatural Code. Optional.
dwc:taxonRank	The taxonomic rank of the scientificName. This should be one of the ranks defined in http://rs.tdwg.org/ontology/voc/TaxonRank e.g. 'species'. Optional but recommended.
dwc:verbatimTaxonRank	The taxonomic rank of the scientificName as a free text string. Optional but recommended if dwc:taxonRank is absent.
dwc:nomenclaturalCode	The nomenclatural code under which the scientificName is constructed. Recommended best practice is to use a controlled vocabulary. One of ICBN; ICZN; BC; ICNCP. Required.
dwc:taxonomicStatus	The taxonomic status in the context of this classification in this DwC-A. This row represents a taxon that is either accepted, sunk into synonymy (in the broad sense) or should be ignored. i.e. if this were a printed list it would represent a list entry in bold or a sub-entry to another taxon. One of 'accepted', 'synonym' or 'rejected'. Required for taxon lists.
dwc:acceptedNameUsage	If the dwc:taxonomicStatus is 'synonym' then this field contains the name of the taxon that is accepted in its place. Optional.
dwc:acceptedNameUsageID	If the dwc:taxonomicStatus is 'synonym' then this field contains the ID of the taxon that is accepted in its place. The ID should either be the dwc:taxonID of a row in the file and/or a GUID. Optional.

Name	Notes
dwc:nameAccordingTo	To use a name precisely an indication of which concept of that name one refers to is needed. Traditionally the Latin sensu or sec. (for secundum – according to) have been used. For taxa that result from identifications a reference to the keys or monographs used, online source or experts should be given. Could be a publication (identification key), institution or team of individuals. Optional.
dwc:nameAccordingToID	A unique identifier that returns the details of a taxonAccordingTo reference. Optional.
dwc:originalNameUsage	The basionym (botany) or basonym (bacteriology) of the scientificName or the junior/later homonym for replaced names. Optional.
dwc:originalNameUsageID	The ID of the basionym (botany) or basonym (bacteriology) of the scientificName or the junior/later homonym for replaced names. Optional.

Normalised Taxonomy Columns

Name	Notes
dwc:parentNameUsageID	The ID of the parent taxon. The ID should either be the dwc:taxonID of a row in the file and/or a GUID. Required.

Flattened Taxonomy Columns

It is recommended that either the kingdom/phylum/class/order/family columns be used or the higherClassification column and not both as they may contain ambiguous information.

Name	Notes
dwc:kingdom	The full scientific name of the kingdom in which the taxon is classified. Optional.
dwc:phylum	The full scientific name of the phylum in which the taxon is classified. Optional.
dwc:class	The full scientific name of the class in which the taxon is classified. Optional.
dwc:order	The full scientific name of the order in which the taxon is classified. Optional.
dwc:family	The full scientific name of the family in which the taxon is classified. Optional.
dwc:higherClassification	A list (concatenated and separated) of taxa names terminating at the rank immediately superior to the taxon referenced in the taxon record. Order the list starting with the highest rank and separating the names for each rank with a semi-colon (";"). Optional.

6 Names vs Taxa

Each row is considered to be a taxon that is either accepted by the current classification or rejected (as a synonym or rejected name). Sometimes there may be a need to express

purely nomenclatural information in DwC-A — particularly when dealing with nomenclators. In such cases no taxonomic hierarchy should be provided and no indication of taxonomic status given.

7 Additional Nomenclatural Information

The fields suggested here are not capable of expressing the full complexity of biological nomenclature as they are focussed instead on expressing the products of the nomenclatural process i.e. A list of correctly named taxa. DwC and the DwC-A format allows for expansion of the taxa.txt file with additional fields. The user is referred to the appropriate documentation on the GBIF ECAT site should additional fields be required. http://code.google.com/p/gbif-ecat/wiki/ChecklistFormat. Bear in mind that consumers of the DwC-A files may not make use of fields other than those listed here. It may be worth establishing how the data will be used before committing resources to curating and publishing it. Full nomenclatural details may be better expressed in RDF or TCS XML as the result of a call to a URI as is done at the moment by IPNI and Index Fungorum.

8 Default Values

If you are publishing a classification of, for example, organisms who's names are only governed by plant or only animal codes of nomenclature, some of the columns listed above would contain the same value in every row. All the names would be governed by the ICBN or ICZN for example. In these situations it is possible to specify the default value for the field in the meta.xml file using the 'default' attribute of the field element. There are examples of how to do this on the GBIF ECAT Wiki (http://code.google.com/p/gbif-ecat/wiki/DwCArchive).

9 Vernacular Names

DwC-A offers two approaches to including vernacular names. The dwc:vernacularName field could be added directly to the taxa.txt file with the vernacular name in it or a separate extension file could be included and mapped to the taxon.txt file using the dwc:taxonID column.

Recommendation: Including the vernacular name directly in the taxa.txt does not allow for expressing the language or location of the vernacular usage. It is recommended, therefore, that this approach is not taken and that a separate file is included instead. This file should contain the language and location of the usage of the vernacular name either within the file itself or using the default values mechanism in the meta.xml file (see above). There are examples of how to do this on the GBIF ECAT Wiki (http://code.google.com/p/gbif-ecat/wiki/DwCArchive).

10 Distributions

If distribution data is to be included it should be included as an extension file as per the recommendations on the GBIF ECAT Wiki (http://code.google.com/p/gbif-ecat/wiki/DwCArchive). There is a direct mapping between recommendations in previous reports and the columns suggested by GBIF.

11 Taxon Identifiers

Each row in the taxa.txt file is identified by an index column – the dwc:taxonID. This should be a locally unique ID within the scope of the DwC-A. It is used to reference the row from the dwc:acceptedNameUsageID, dwc:parentNameUsageID and dwc:originalNameUsageID fields and also to link the extension files to the core taxa.txt file. There is no requirements for the identifier to be a GUID (out-with the scope of the file). Use of GUIDs in this field would make it easier for consuming applications to work on multiple data sets that cite each other and is encouraged.

12 Provenance Data

It is important to include provenance data in the archive as this permits users of the data to follow any rights restrictions and give appropriate attribution. This data should be included in a simple XML file (provenance.xml) and linked to from the meta.xml file's achive@metadata attribute.

```xml
<?xml version="1.0"?>
<metadata xmlns:dc="http://purl.org/dc/terms/">
   <dc:title> ... </dc:title>
   <dc:description>... </dc:description>
   <dc:subject>... </dc:subject>
    <dc:created>... </dc:created>
    <dc:creator>... </dc:creator>
    <dc:publisher>... </dc:publisher>
   <dc:license>... </dc:license>
</metadata>
```

The file should contain properties from the Dublin Core /terms/ name space as defined by Dublin Core (http://dublincore.org/documents/dcmi-terms/). Those terms illustrated above are recommended. It is also recommended that the dc:license property contain the URI of a Creative Commons license and the dc:publisher property contain the URI of a institution or project web site if possible.

13 RDF and the Semantic Web

The DwC-A format is highly amenable to being mapped to RDF so as to make the data available for use on the Semantic Web as Linked Data or used in OWL Ontologies for semantic reasoning. A normative mapping is not defined here as it is still an area of active research. Multiple mapping strategies may be used depending on the use envisaged for the resulting RDF.

Configuration History			
**Version No.**	**Date**	**Changes made**	**Author**
0.1	30/03/10	Initial version	Roger Hyam
0.5	13/03/10	Added Glossary	Roger Hyam
1	22/04/10	First complete text for discussion in Amsterdam	Roger Hyam
1.1	17/05/10	Additions from Amsterdam WP meeting	Roger Hyam
