# Supplementary material for: PESI - a taxonomic backbone for Europe
Source: Biodivers Data J. 2015 Sep 28;(3):e5848. doi: 10.3897/BDJ.3.e5848 (PMC4609752; doi:10.3897/BDJ.3.e5848)
Supplement: Supplementary material 16 — Report on the contributions to the set up of a Global Name Architecture [file biodiversity_data_journal-3-e5848-s016.pdf]

D4.4 — Report on the contributions to the set up of a Global Name Architecture

Table of Contents

Report on the contributions to the set up of a Global Name Architecture 1
Table of Contents 1
1 Introduction 1
2 Current Global Names Architecture 2
2.1 What is the Global Names Architecture? 2
2.2 Global Names Index 3
2.3 Checklist Bank 3
2.4 Global Names Usage Bank (GNUB) 4
2.5 GBIF Nub Taxonomy 5
3 Future of Global Names Architecture 5
3.1 Immediate Future – To 2013 5
3.2 Risk - Over Engineering of GNA 6
3.3 Risk – Underfunding of Primary Data Sources 6
3.4 Risk/Opportunity – Duplication of Infrastructures 6
3.5 Risk/Opportunity – IPR Improvements 7
Glossary 7
References 10

1 Introduction

This is the fourth and final report of Work Package 4 (WP4) of the Pan-European Species-directories Infrastructure (PESI).

The first report (WP4.1 “Report on authoritative taxonomic standards from multiple sources suitable for deployment within European Research Area.”) differentiated between standard taxonomies and standards used to exchange taxonomic information and build consensus biological classifications. It listed the range of different taxonomic products and defined PESI as an **annotated checklist**.

The second report (WP4.2 “Report on Procedures and Mechanisms for the Functioning of Nomenclators within the e-Infrastructure.”) differentiated between nomenclature and taxonomy and proposed a strategy for linking nomenclators to PESI.

The third report (WP4.3 “Application and Adoption of Taxonomic Standards”) outlined the success in reaching agreements between key players in the global community (The

Montpellier Declaration) and detailed specifically how standards would be used to exchange taxonomic data (specifically by using the Darwin Core Archive format).

This report aims to expand on how PESI is contributing to the set up of a global system for managing scientific names of organisms. It identifies opportunities and risks going forward.

As with the third report, a glossary is included covering not only acronyms mentioned in the text but also those likely to be encountered during background reading.

These reports have been compiled over a period of two years. During this time the landscape has changed. This has occurred as our understanding of the data and requirements has matured and as other projects have come on-line, or have failed to materialise in the expected time frame. PESI, and particularly WP4 partners, have been involved in the dialogues that have lead to this; notably by participation at Nomina, TDWG and other meetings.

2 Current Global Names Architecture

2.1 What is the Global Names Architecture?

> *The Global Names Architecture (GNA) is a system of databases, programs, and web services - a cyberinfrastructure - that can be used to discover, index, organize and interconnect on-line information about organisms and their names...[It] is a communal open environment that manages names so that we can manage information about organisms and serve the needs of biologists. (*http://www.globalnames.org/*).*

GNA is an evolving collection of databases and services. It is not an extant formal architecture but a vision supported by the major taxonomic projects. It is made up of a series of components as illustrated in the diagram below. Key amongst these is the Global Names Index (GNI), the Checklist Bank (Classification Repository in the diagram below) and the Global Names Usage Bank (GNUB). These are dealt with in separate sections below. A useful overview of the rationale and intention for a GNA is given in Patterson et. al 2010 [1], and also at http://www.globalnames.org.

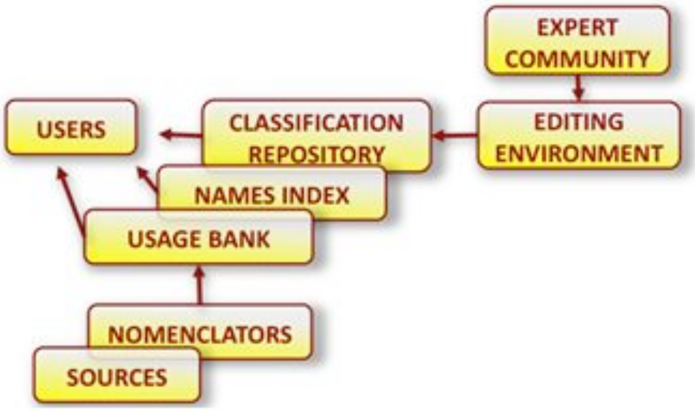

Fig. 1: GNA Components (from http://www.globalnames.org/Components)

PESI Relevance: As outlined in WP4.3, PESI feeds into this diagram at two points. As an annotated checklist it can publish its data directly as authoritative classifications to the Checklist Bank (Classification Repository). As a source of high quality nomenclatural data it can publish data directly to the GNUB (Usage Bank) - once GNUB is operational. In the future PESI will be able use the names index to find resources associated with names in its own index. PESI will be able to call on GNUB for usages of names within the taxonomic literature.

There is some difference of opinion on the nature of the GNA. Some view it as a single set of linked services whilst others view it as a set of protocols and standards with little or no central services. These two views both have shades of meaning and can overlap.

2.2 Global Names Index

> *The Global Names Index(GNI) is the first component of a semantic environment for biology called the Global Names Architecture GNA. GNI has been developed by the Global Biodiversity Information Facility and the Encyclopedia of Life. It has benefited from the ideas of an array of gifted and enthusiastic individuals who contributed through the Nomina workshops that they attended. (http://github.com/dimus/gni/wiki/global-names-index-help)*

GNI is primarily an indexed list of 20 million scientific name strings from multiple sources. By building a single index it is possible to use parsing and clustering algorithms to build lexical groups of names, so as to provide query expansion services. Given a query string, the GNI can return links to occurrences of this string in source databases as well as 'similar' strings that appear to represent alternate spellings of the same name as it appears in other sources. It is intended for machine, rather than human, use.

GNI gets its data from multiple sources but notably imports the names found in checklists in the Checklist Bank and GNACLR.

GNI has been jointly implemented by GBIF and EoL. It is operational and in version 0.9.28.

PESI Relevance: Names data from PESI will be harvested into the GNI from Checklist Bank. In the future they may also enter GNI via contributions to the GNUB. PESI can query GNI to find variants of names used in Europe.

2.3 Checklist Bank

GBIF has built Checklist Bank (http://www.gbif.org/informatics/name-services/checklist-bank/). This allows the deposition of synonymised, annotated checklists in Darwin Core Archive format. These checklists are indexed and published to the web. It also includes name strings found in occurrence records submitted to the GBIF data portal (http://data.gbif.org/) and a number of services around these resources. Some of these services duplicate those found in the GNI. Checklist Bank is currently

available as a Beta version (http://ecat-dev.gbif.org/).

GNACLR (http://gnaclr.globalnames.org/classifications) is a similar effort, also based on DwC Archive but hosted by EoL. The primary focus being on maintaining a versioned repository for classifications and to interact with the proposed GNA editing environment which is also an EoL initiative.

PESI Relevance: PESI will publish data to Checklist Bank in Darwin Core Archive format (see D4.3) effectively using Checklist Banks as a publishing mechanism. PESI could use Checklist Bank to help organise contributions of checklists from the focal points.

2.4 Global Names Usage Bank (GNUB)

> *Global Names Usage Bank – an environment to record the occurrence of names in documents, databases, notes or other records. A large and central component of GNA. The usages will include all nomenclatural acts, making GNUB critical to nomenclators [and existing] and emerging registration environments such as Zoobank. GNUB will index usages that help to clarify the meaning of each name and so contains resources that will be developed into taxonomic tools and services.* (http://www.globalnames.org/glossary/term/45)

Of the GNA components, the GNUB is the most ambitious and the least developed. It has been looking for funding over the past year but now has developer support. The Nomina VIII Meeting in Christchurch, New Zealand in November 2010 examined the GNUB component in detail (http://www.aa3sd.net/IPNI2/index.php?title=Main_Page). The proposed development time line (updated March 2011, pers. comm.) is:

- November 2010 – stabilisation of data model
- December 2010 – porting of ZooBank data to new data model
- December 2010 – Buy-in to GNUB concept by IPNI & Tropicos at a meeting early Christchurch, NZ.
- May 2011 – Multiple replicates of live GNUB database on 4-6 servers around the world
- Mid 2011 – Pipelining services between GNUB and GNI. Documentation of APIs

Report D4.2 expanded on the differences between nomenclature and taxonomy as well as the type specimen based conventions used to name taxa under the botanical and zoological codes. The GNUB builds on these notions. It records instances of name usages (recording also the source publication) which will include, but go beyond, nomenclatural acts.

There are complex rules of nomenclature layered upon taxonomic changes. Being able to track the type specimen based relationships between the usages of names for different taxa can help facilitate understanding the taxonomic relationships, for example revealing that two taxa with similar but different names are based on the same type specimen and may have a similar circumscription. More ambitious taxonomic databases, such as the GNUB, attempt to track these relationships between names, taxa and names

and taxa as they occur in the literature rather than just storing lists of accepted and synonymous names as is done by PESI and similar projects. The GNUB will attempt to catalogue each time a name is used as a new taxon circumscription.

PESI Relevance: The GNUB will not become fully operational until after the end of the current round of PESI funding. PESI data will be available for integration on the basis of it being supplied to Checklist Bank but there are other routes whereby partner data will be incorporated. Zoobank is now envisaged as service running on the GNUB. Index Fungorum is a key partner in the development of the GNUB and will contribute data right from the start. IPNI has also been closely involved and will contribute data directly. PESI will benefit from the GNUB by using it as an authoritative source of data on names usages globally.

2.5 GBIF Nub Taxonomy

> *The GBIF Backbone Taxonomy, usually called the Nub taxonomy, is a single synthetic management classification with the goal of covering all taxa GBIF is dealing with. It's the taxonomic backbone that allows GBIF to integrate name based information from different resources, no matter if these are occurrence datasets, species pages, names from nomenclators or external sources like EOL, Genbank or IUCN. This backbone allows taxonomic search, browse and reporting operations across all those resources in a consistent way and to provide means to crosswalk names from one source to another. (http://ecat-dev.gbif.org/about/nub/)*

This is a resource within Checklist Bank, and should not be confused with GBIF GNUB. It provides, not only a management classification, but also an automated process for merging data deriving from disparate sources. The Nub provides the taxonomic backbone needed for the GBIF Data Portal.

PESI Relevance: PESI is also engaged in integrating data from different sources (Fauna Europaea, ERMS and Euro+Med Plantbase). The management classification that PESI uses has been built from the existing classifications employed by the three sources with expert reconciliation for overlapping taxonomic groups. The management classification used by GBIF is based upon that used in Catalogue of Life. The Catalogue of Life Team is in the process of reviewing their classification and this will, most likely, impact on the classification used by GBIF. Because it was not possible to wait for these impending changes, PESI had to stay with its own classification. However, it would be advantageous to align PESI with GBIF's classification once this has stabilised.

3 Future of Global Names Architecture

3.1 Immediate Future – To 2013

Projects are underway and resources committed to complete and launch the main components of the GNA as currently envisaged. The GNI and Checklists Bank are more or less complete and will increase in influence. Implementation of the GNUB has begun and

is funded but success will require buy-in by major projects. The effort to populate the GNUB will be much greater than the effort to create the technical infrastructure. Unless contributors find it useful in the short to medium term it won't attract the commitment needed to reach critical mass and so provide the benefits of a fully populated database.

The ICZN are moving towards registration of names and it will be clear by 2013 if this is going to happen. The fungal community within ICBN is moving towards mandatory registration of names from 1st January 2013. Both these registration schemes could provide the "killer app" for the GNUB.

The first round of PESI funding will end in 2011 with PESI having made a significant contribution through participation of its partners in GNA and donation of its data.

3.2 Risk – Over Engineering of GNA

A biological classification can be thought of as a hierarchical thesaurus of terms with each taxon represented by a single term and names represented as either preferred labels or alternate labels for these terms. In this approach names are treated merely as labelling strings even if they are the product of the application of complex rules of nomenclature. A combination of lexical expansion of names and thesaurus based query expansion using thesauri derived from basic classification information provides a powerful resource discovery mechanism. This approach is not specific to biological systematics and so benefits from tools built for other domains.

If the primary use-case is resource discovery then the currently implemented GNI and Checklist Bank may be sufficient to meet the needs of the wider scientific community. Building more complex databases may not provide significant return on investment. On the other hand, if there are clear use-cases for tracking each usage of a name (each re-definition of a taxon) then building such a database is very worthwhile.

PESI relevance: From the perspective of PESI this is not a current risk. Data published in DwC Archive format (see WP4.3) will be sufficient to help populate currently proposed databases but care should be taken that further development is use-case driven.

3.3 Risk – Underfunding of Primary Data Sources

PESI, along with many of the other taxonomic projects mentioned in this report, rely on the gathering of biodiversity data both from the field and from the literature. This initial round of funding has established an infrastructure for data to flow from taxonomic experts and national focal points into an integrated system but does not supported the data sources themselves which remain voluntary. Without a continuous supply of data and expert editorial contributions the infrastructure will fail. Future rounds of funding should address this issue. WP5 has looked at this in more detail.

3.4 Risk - Co-ordination and Ownership of Activities

The GNA is a worthy concept and considerable effort is being directed at designing and building a number of its components. Various organisations and projects are involved but

progress is dependant on funding, which usually is only available in small amounts for focused activities. Thus there is a danger that progress on the various components cannot be effectively co-ordinated. Although GBIF is helping to drive forward the concept and process of the GNA, there does not seem to be a sustainable business plan, since the work of populating, validating and maintaining will require long-term commitments from a number of partners.

3.5 Risk/Opportunity – Duplication of Infrastructures

As has been outlined above there is overlap between the work of EoL and GBIF in their building of the components of the GNA. This can be viewed as a risk because of the duplication of implementation effort, although the duplication is actually inevitable because of the integrated nature of the tasks. Building any of the components of the GNA requires the implementation of some form of name parsing and indexing to keep track of multiple classifications.

This duplication can be viewed as an opportunity, as it encourages the use of open exchange standards such as Darwin Core. Once the data is 'chunked' into discrete units (currently Darwin Core Archive files) context-specific applications can be built. The current vision of a Global Names Architecture can enable the development of more local names architectures based on the needs of specific communities at specific times. PESI can play a leading role for communities with Europe in this vision of future GNA.

3.6 Risk/Opportunity – IPR Improvements

During the course of PESI there has been a general move towards the use of open licensing for data exchange. A strong facilitator of this has been the creation, by Creative Commons (http://creativecommons.org), of a series of understandable, customisable licenses that allow people to express how they would like to share their data, whether or not these licenses are legally enforceable in all jurisdictions or not. The Encyclopaedia of Life has enthusiastically adopted these licenses. The applicability of Creative Commons licensing within PESI was discussed within the WP2 Deliverable D2.2 *The Government of IPR of Electronic Biodiversity Data*. It is intended that the PESI Portal will follow the CC *Attribution-Share-Alike* scheme.

Glossary

Atlas of Living Australia – The Atlas of Living Australia is a five-year project funded under the Australian Government's National Collaborative Research Infrastructure Strategy. Its mission is to develop a biodiversity data management system which will link Australia's biological knowledge with its scientific and agricultural reference collections and other custodians of biological information. http://www.ala.org.au/

CATE - The Creating a Taxonomic e-Science project, funded by the United Kingdom's Natural Environment Research Council (NERC) under its e-science initiative. The particular goal of CATE is to test the feasibility of creating a web-based, consensus taxonomy using two model groups, one from the plant and the other from the

animal kingdom. The wider aim is to explore practically the idea of 'unitary' taxonomy and promote web-based revisions as a source of authoritative information about groups of organisms for specialist and non-specialist users. http://www.cate-project.org/

CDM – see EDIT CDM

Drupal – An open source content management platform used to create websites.

DwC – Darwin Core. Darwin Core is a Biodiversity informatics data standard that consists of a vocabulary of terms to facilitate the discovery, retrieval, and integration of information about organisms, their spatiotemporal occurrence, and the supporting evidence housed in biological collections. DwC is a TDWG standard. http://en.wikipedia.org/wiki/Darwin_Core

DwC-A – Darwin Core Archive. DwC-A is a derivative of the DwC standard developed by GBIF to facilitate the exchange of checklist data.

EDIT – European Distributed Institute of Taxonomy. A network of excellence gathering 28 major institutions devoted to knowing the living world better with the support of the European Commission. http://www.e-taxonomy.eu/

EDIT CDM – The EDIT Common Data Model is the domain model for the core EDIT components. It has been instantiated as a Java base API over a relational data model for embedding within different taxonomy based projects. http://dev.e-taxonomy.eu/trac/wiki/CommonDataModel

EDIT Scratchpads - Scratchpads are an easy to use, social networking application that enable communities of researchers to manage, share and publish taxonomic data online. Sites are hosted at the Natural History Museum London, and offered free to any scientist that completes an online registration form. http://scratchpads.eu

Encyclopaedia of Life (EoL) – An international, but largely USA based, project to create a web page for every species inspired by the ecologist E.O. Wilson.

ETI Bioinformatics – ETI develops and produces scientific and educational computer-aided information systems. http://www.eti.uva.nl/

GBIF – Global Biodiversity Information Facility. An international government-initiated and funded initiative focused on making biodiversity data available to all and anyone, for scientific research, conservation and sustainable development. The GBIF Secretariat is based in Copenhagen. The GBIF Data Portal indexes many millions of data points per year. http://www.gbif.org/

GBIF ECAT – Electronic Catalogue of Names of Known Organisms. A programme within GBIF.

GBIF IPT – Integrated Publishing Toolkit. The GBIF IPT is an open source, Java (TM) based web application that connects and serves three types of biodiversity data: taxon primary occurrence data, taxon checklists and general resource metadata. The data registered in a GBIF IPT instance is connected to the GBIF distributed network and made available for public consultation and use. http://code.google.com/p/gbif-providertoolkit/

GNACLR - is a similar effort to GBIF Checklist Bank also based on DwC Archive but hosted by EoL. The primary focus being on maintaining a versioned repository for classifications and to interact with the proposed GNA editing environment which is also an EoL initiative. http://gnaclr.globalnames.org/classifications

GNITE – An editing tool being developed by EoL for the Global Names Architecture. http://www.globalnames.org/GNITE

GSD – Global Species Databases are the building blocks of the Species2000/Catalogue of Life data set. They are typically taxon specific (occasional region specific) databases of names managed by experts or institutions.

GUID – Globally Unique Identifier. Within the biodiversity informatics domain GUID has been used to mean a resolvable or actionable identifier that has global scope. An HTTP URI is an example of this. In the wider computing community it is often used as a synonym for Universally Unique Identifier (UUID) which are essentially large random numbers. Anyone can create a UUID and use it to identify something with reasonable confidence that the identifier will never be unintentionally used by anyone for anything else but UUIDs don't have an associated dereferencing mechanism i.e. they can't be used as an address to look up normative information about what they identify.

HTTP URI – An HTTP URI is a web address or name starting with 'http://' See also HTTP and URI.

HTTP – Hypertext Transfer Protocol. An application layer protocol for distributed, collaborative, hypermedia information systems. It is the key technology that turns the Internet into the World Wide Web. http://en.wikipedia.org/wiki/HTTP

Index Fungorum – A fungal nomenclator (names and associated nomenclatural and bibliographical data) currently co-ordinated and supported by CABI, CBS and LCR. http://www.indexfungorum.org/

IPNI – The International Plant Names Index (IPNI) is a database of the names and associated basic bibliographical details of seed plants, ferns and fern allies. http://www.ipni.org/

ITIS – Integrated Taxonomic Information System. A partnership of Canadian, Mexican and USA federal agencies formed to satisfy their mutual needs for scientifically credible taxonomic information. http://www.itis.gov/

Linked Data - The term Linked Data is used to describe a method of exposing, sharing, and connecting data via dereferenceable URIs on the Web. http://linkeddata.org/

MycoBank – A voluntary name registration portal for fungal nomenclature. http://www.mycobank.org

OWL – The Web Ontology Language. OWL is a family of knowledge representation languages for authoring ontologies, and is endorsed by the World Wide Web Consortium http://en.wikipedia.org/wiki/Web_Ontology_Language

RDF – Resource Description Framework. A family of World Wide Web Consortium

(W3C) specifications originally designed as a metadata data model. It has come to be used as a general method for conceptual description or modelling of information that is implemented in web resources, using a variety of syntax formats. http://en.wikipedia.org/wiki/Resource_Description_Framework

Semantic Technologies – The collections of technologies used to implement the Semantic Web. Core technologies are HTTP URIs and RDF.

Semantic Web - An evolving development of the World Wide Web in which the meaning (semantics) of information and services on the web is defined, making it possible for the web to 'understand' and satisfy the requests of people and machines to use the web content. http://en.wikipedia.org/wiki/Semantic_web

TDWG (Biodiversity Standards) – A not for profit scientific and educational association that is affiliated with the International Union of Biological Sciences. TDWG was formed to establish international collaboration among biological database projects. TDWG promotes the wider and more effective dissemination of information about the World's heritage of biological organisms for the benefit of the world at large. TDWG focuses on the development of standards for the exchange of biological/biodiversity data. http://www.tdwg.org/

URI – Uniform Resource Identifier. A string of characters used to identify a name or a resource on the Internet. http://en.wikipedia.org/wiki/Uniform_Resource_Identifier

ZooBank – ZooBank is intended as the official registry of Zoological Nomenclature, according to the International Commission on Zoological Nomenclature (ICZN). http://www.zoobank.org/

References

1. Patterson, D.J., et al., 2010. Names are key to the big new biology. Trends in Ecology and Evolution, 25(12) : 686-691.
2. Pyle, R . and Michel, E., 2009. Unifying nomenclature: Zoobank and Global Names Usage Bank. Bull.Zool.Nomenclature, 66 : 298.

Configuration History			
**Version No.**	**Date**	**Changes made**	**Author**
0.1	01 November 2010	Initial version	Roger Hyam
0.4	16 November 2010	Final version before employment contract end	Roger Hyam
0.5	16 March 2011	Edited by Hussey prior to release	Hyam & Hussey
1.0	18 March 2011	Final preparation for submission	YdJ
