# Supplementary material for: PESI - a taxonomic backbone for Europe
Source: Biodivers Data J. 2015 Sep 28;(3):e5848. doi: 10.3897/BDJ.3.e5848 (PMC4609752; doi:10.3897/BDJ.3.e5848)
Supplement: Supplementary material 17 — Global Names Europe (GN-­EU) – a names based cyber-­‐infrastructure [file biodiversity_data_journal-3-e5848-s017.pdf]

ViBRANT
Virtual Biodiversity

Milestone M4.43

Global Names Europe (GN-EU)
a names based cyber-infrastructure

Contributing to the Global Names Architecture developments as a necessary component of Research Data e-Infrastructures: Framework for Action in H2020

(D4.3 - Design of robust services)

Leading partner: RBINS (Yde de Jong)

Other partners: Kew, Plazi, Pensoft, NHM, OU

Date: 14 October 2013

Abstract

Taxon names provide key ontologies on connecting biodiversity data. The absence of an appropriate global name-reference system[1] hinders an efficient and dynamic cross-referencing of taxon names, the functional re-use of biodiversity information and a single access to 'all names in use'. It also hinders the further development of a communal (virtual) research environment, supporting science as a community effort.

Global Names (Architecture) would like to create more stability, compatibility and community in names resolution, by including an objective (nomenclatural) layer, stabilising the cross-referencing of taxon names and by including advanced indexing services for name discovery, recognition and re-use, which will also optimise the use and uptake of all associated biodiversity information.

GN(A) will optimise the discovery and integration of biodiversity data by developing and improving authority files and names backbones (like CoL, PESI and WoRMS), checklist mapping routines and repositories (like the GBIF Checklist Bank), harmonise web portal APIs, build workflows to secure the proper resolution and data cleaning for e-Science application, and support the on-going virtualisation of the research domain. It will advance the names architecture set up towards a global names references system, including necessary components like the Global Names Usage Bank (GNUB) and Global Names Index (GNI).

Introduction & Background

Linnaeus' system of Latinized scientific names for organisms is one of the most enduring and universal standards in science and can serve as a near universal system of metadata for biodiversity information. The Global Names infrastructure (GN) is an internationally supported concept to build a names-based cyber-infrastructure that will act as a virtual layer that uses expert systems to interconnect distributed data, making it discoverable and actionable. It is generally regarded that a names-based cyber-infrastructure is a necessary component of the Big Data world for biology. Such an infrastructure must address problems with names as metadata (overcoming the one name for many species and many name for one species problems), transform names to bring them up to date with current taxonomic standards. The infrastructure can monitor and capture metadata from digital resources, taxonomically update and

1 Currently name resolution takes place at the conceptual level, which is due to change (never fixed ground), subjective (depending your classification and mapping algorithm), incomplete, unconnected and redundant (many local (distributed) implementations).

cross-link electronic datasets of relevance to biodiversity, and make discovery metadata available to the Linked Open Data Cloud. The infrastructure will transform multiple overlapping freestanding taxonomic databases, digital resources, and services into a virtual pool; and through it accelerate access to available information on any taxon. As such, GN will be a part of the processes that will transform life sciences into a more unified and data-centric "Big New Biology".

The Global Names Architecture is a multi layered architecture taking care about different aspects on creating and managing controlling taxonomic ontologies and developing the relevant strategies and (open) semantics for efficiently sharing and integrating biodiversity data.

The Encyclopedia of Life (EoL) and the Global Biodiversity Information Facility (GBIF) conceived the Global Names Architecture. In Europe the GNA efforts are extensively supported by the pan-European Species-directories Infrastructure (PESI) project, within the ViBRANT project and via the European contributions to GBIF-ECAT.

In 2011, the US National Science Foundation invested in the US-based Global Names project[2] attempting to provide an infrastructure for unifying taxonomic databases and services for managers of biological information, especially improving the imbedding of the nomenclators within the Global Names Architecture.

The here proposed European contribution to the Global Names Architecture, provisionally called GN-EU, is supposed to proceed from this US Global Names effort, but adapted to the European political and infrastructural landscape and requirements. It will transform the prototype developments into a persistent infrastructure that will improve visibility, integration and re-use of biodiversity information, providing a robust virtual environment for nomenclators, particularly profiling ZooBank as a registration service for names of animals, and an array of other names-based tools and services that have demonstrated the value of a names-based approach to biodiversity data management. This will be done in collaboration with the US-based Global Names project, sharing all software and content, which is openly available.

To illustrate the potential of a names-based infrastructure, one component would be tools and services that can be applied to a wide diversity of file types in a heterogeneous storage environments, capable of recognizing the file types, opening the files, and then applying algorithms that recognize names (because they occur in the Global Names infrastructure) or discovery names because of their distinctive format. Names can be extracted an associated with data centres, data files, or atoms of data and made available through User Interfaces,

[2] NSF grant: DBI-1062387 – http://globalnames.org

Application Interface Services, or to the Linked Open Data Cloud, where they can act as discovery-level metadata. Such tools are scalable, replacing very costly manual addition of metadata. Prototypes have already been developed and are being positioned as automated metadata discovery tools for the NSF Data Conservancy project, and now ready for improvement into scalable indexing services.

Progress in ViBRANT

During the ViBRANT project several contributions have been made supporting the further setup and development of Global Names Europe. These include:

(1) In collaboration with GN-US at 26 March 2013 a bid was prepared for the DG CONNECT call for "Consultation on Research Data Infrastructures: Framework for Action"[3].

(3) A brainstorm session was held at 2 August 2013 in the Natural History Museum in London on the relevance of Global Names Europe with partners from RBINS, NHM, Kew and OU.

(4) On the BIH2013 conference at 3-6 September 2013 in Rome [4], as part of the demand to support the formation of H2020 consortia, the PESI consortium launched 'PESI Plus' as an input to the H2020 preparations, including a contribution to a (global) names-based architecture for linking biodiversity data (Global Names)[5].

(5) GN-EU was invited at the COOPEUS[6] meeting at 18-20 September 2013 in Madrid, in a side-session of the EGI-Technical Forum[7], to highlight its collaboration with outer European partners on developing the Global Names Architecture.
As a result Global Names was successfully included at the COOPEUS agenda and GN-US was invited for the COOPEUS annual meeting (25-27 September 2013) in Colorado to join LifeWatch relevant discussions.
In addition, an interesting collaboration with EUDAT was kicked-off, a project that provides substantial support on building data infrastructures for

---

3 https://ec.europa.eu/digital-agenda/en/content/consultation-research-data-infrastructures-framework-action

4 http://conference.lifewatch.unisalento.it/index.php/EBIC/BIH2013

5 http://h2020.myspecies.info/content/pesi-plus-pesi-inspire-lifewatch-eu-bon

6 http://www.coopeus.eu

7 http://go.egi.eu/tf2013

researchers. LifeWatch is a partner in EUDAT[8] and PESI (incl. GN-EU) is on the LifeWatch roadmap. This could be a good starting point for the GNUB. It's proposed to fill-in a EUDAT survey[9] to express our interests and requirements on data infrastructures.

(4) A charter for a Global Names (Architecture) Interest Group[10] in RDA was positively received and is reviewed by the TAB and Council (see below).

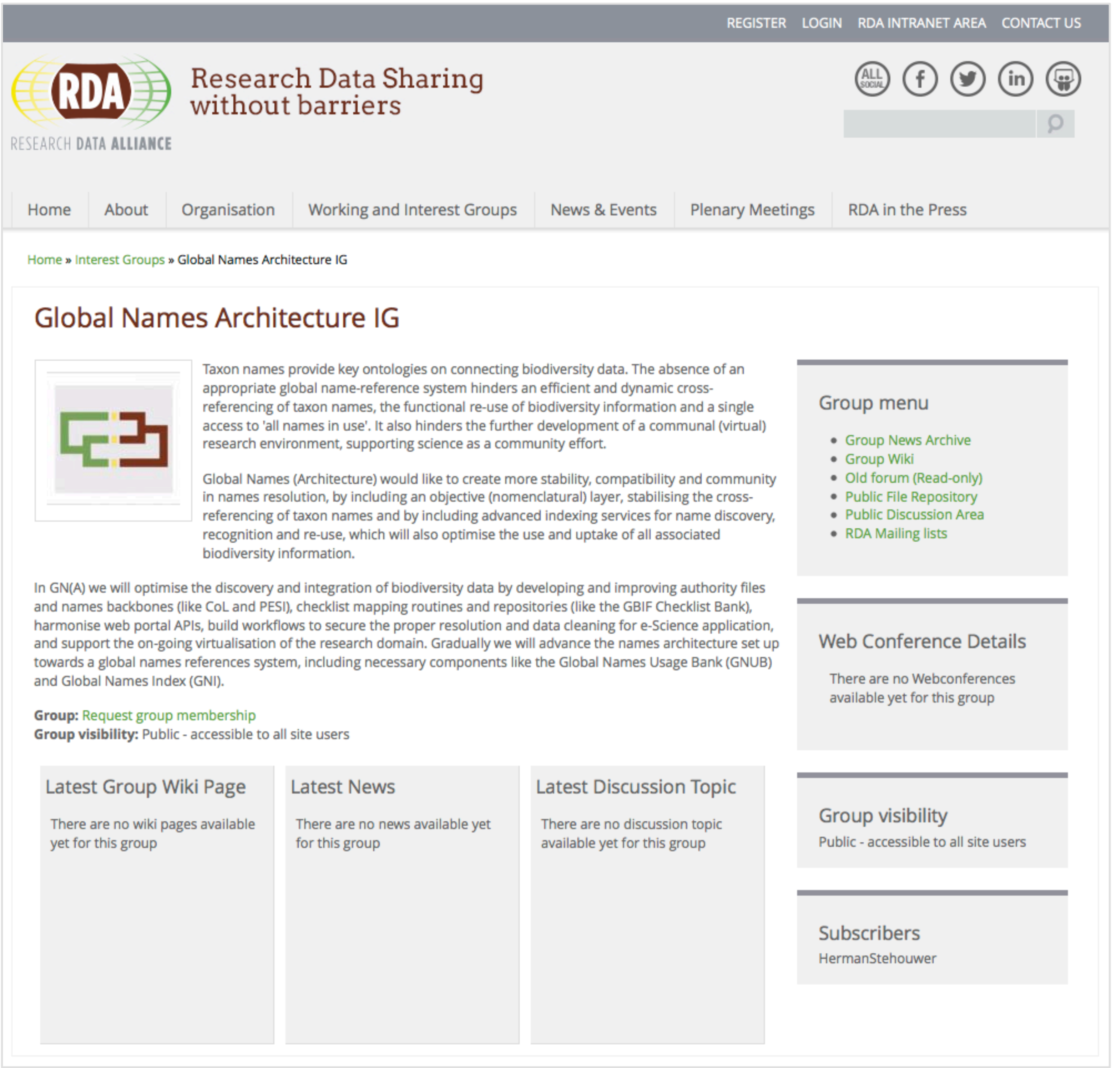

(5) At the upcoming TDWG conference in Florence (28 October - 1 November 2013)[11] a session will be held on "Developing a names-based architecture for linking biodiversity data"[12].

---

8 http://www.eudat.eu

9 http://www.eudat.eu/news/have-say-selection-and-development-next-set-eudat-services

10 https://www.rd-alliance.org/internal-groups/global-names-architecture-ig.html

11 http://www.tdwg.org/conference-2013

12 https://mbgserv18.mobot.org/ocs/index.php/tdwg/2013/schedConf/trackPolicies - track21

TDWG "Names session" details:

SYMPOSIUM: DEVELOPING A NAMES-BASED ARCHITECTURE FOR LINKING BIODIVERSITY DATA

We expect around 10 short presentations, some open discussion, and (hopefully) speed introductions to the posters.

This session will focus on the role of names infrastructures supporting the integration of biodiversity data for e-Science application and the progress on the sharing and linking of taxonomic information in the e-Taxonomy domain.

Contributions include the ongoing efforts on establishing a common names reference system (Global Names), including its components, like the Global Names Usage Bank (GNUB), relevant cross-mapping and annotation services, routines for names discovery, technical implementation models, and consortium set up. Presentations will highlight some best practices on name portal services, name resolution workflows, taxonomic concept modeling, backbone models, reference system requirements, and names licensing and attribution.

For e-Taxonomy the contributions will show recent developments on cross-platform integration and the further scoping of virtual workbenches to serve the taxonomic community and taxonomic indexing projects.

Everyone willing to contribute to this session by reporting progress on topics related to names infrastructures, name indexing, taxonomic management systems, taxonomic knowledge networks, and so on, is requested to contact the conveners. Since the number of talks will be limited, also poster presentations will be (explicitly) acknowledged as contributions to this session.

Directors

Yde de Jong, Universiteit of Finland (Joensuu) & Royal Belgian Institute of Natural Sciences (Brussels)
Richard Pyle, Bishop Museum 1525 Bernice Street Honolulu, HI 96817

☑ Open Submissions ☐ Peer Reviewed

SYMPOSIUM: DEVELOPING A NAMES-BASED ARCHITECTURE FOR LINKING BIODIVERSITY DATA

374. Towards a Global Names Architecture: A Names-Based Backbone for Integrating Global Biodiversity Data
Richard L. Pyle

439. A names backbone – a graph of taxonomy
Nicky Nicolson

370. Dynamics of taxon concepts: How to handle changes of taxonomic information, and why?
Oskar Kindvall

483. Concepts and Tools Needed to Increase Bottom-Up Taxonomic Expert Participation in a Global Names-Based Infrastructure
Nico Franz, David Patterson, Sudhir Kumar, Edward Gilbert

380. Design of "LODAC Species" as a Names-based Linked Open Data architecture
Akihiro Kameda, Fumihiro Kato, Utsugi Jimbo, Ikki Ohmukai, Hideaki Takeda

489. What Taxonomic information is 'Open Access'?
Donat Agosti, Gregor Hagedorn, Willi Egloff, David Patterson

389. BiOnym – a flexible workflow approach to taxon name matching
Edward Vanden Berghe, Nicolas Bailly, Caselyn Aldemita, Fabio Fiorellato, Gianpaolo Coro, Anton Ellenbroek, Pasquale Pagano

399. The EDIT Platform for Cybertaxonomy as an information broker in name infrastructures
Andreas Kohlbecker, Yde de Jong, Cherian Mathew, Lorna Morris, Andreas Müller, Anton Güntsch, Walter Berendsohn

361. Scratchpads: The virtual research environment for biodiversity data
Simon Rycroft, Dave Roberts, Vince Smith, Alice Heaton, Katherine Bouton, Laurence Livermore, Dimitris Koureas, Ed Baker

355. Development of a dataportal providing European taxonomic information: past activites and future plans
Simon Claus, Yde de Jong, Klaas Deneudt, Bart Vanhoorne, Francisco Hernandez

476. A pan-European Species-directories Infrastructure (PESI)
Yde de Jong, Florian Wetzel, Gregor Hagendorn, Falko Gloeckler, Christoph Haeuser, Marc Geoffroy, Eckhard von Raab-Straube, Anton Guentsch, Walter Berendsohn, Nicola Nicolson, Alan Paton, Paul Kirk, Bart Vanhoorne, Simon Claus, Francisco (Tjess) Hernandez, Jan Mees, Christos Arvanitidis, Hannu Saarenmaa, Valerio Sbordoni, Aaike De Wever, Hendrik Segers, Ulf Gärdenfors, David King

358. In the hot seat: managing data- and workflows between biologists and computers
Dmitry Schigel, Hanna Koivula, Esko Piirainen, Eija-Leena Laiho

551. Taxonomic Names-related Poster Introductions
Richard L. Pyle

Global Names Europe partnership

So far the Global Names Europe network consists of:

- Nicola Nicolson, Alan Paton & Paul Kirk – Kew Garden
- Ellinor Michel – NHM London
- Dauvit King – Open University
- Donat Agosti – Plazi
- Lyubo Penev – Pensoft
- Yde de Jong – PESI

. . . more to follow.

At global level the Global Names consortium so far exists of GN-US and GN-EU (proposed here). Also China (GN-CN), New Zealand (GN-NZ) and Korea (GN-KR) showed interest. GN-CN is supported by the Chinese Academy of Sciences, GN-NZ is supported by LandCare Research and has already a EU liaison project via PaceNet, GN-KR support is given via the Korean GBIF node.
