# Supplementary material for: PESI - a taxonomic backbone for Europe
Source: Biodivers Data J. 2015 Sep 28;(3):e5848. doi: 10.3897/BDJ.3.e5848 (PMC4609752; doi:10.3897/BDJ.3.e5848)
Supplement: Supplementary material 18 — GN-EU – a names based cyberinfrastructure contributing to the Global Names Architecture developments as a necessary component of Research Data e-Infrastructures : Framework for Action in H2020 [file biodiversity_data_journal-3-e5848-s018.pdf]

GN-EU – a names based cyberinfrastructure

contributing to the Global Names Architecture developments

as a necessary component of Research Data e-Infrastructures : Framework for Action in H2020

Linnaeus' system of Latinized scientific names for organisms is one of the most enduring and universal standards in science and can serve as a near universal system of metadata for biodiversity information. The Global Names infrastructure (GN) is an internationally supported concept to build a names-based cyberinfrastructure that will act as a virtual layer that uses expert systems to interconnect distributed data, making it discoverable and actionable. It is generally regarded that a names-based cyberinfrastructure is a necessary component of the Big Data world for biology. Such an infrastructure must address problems with names as metadata (overcoming the one name for many species and many name for one species problems), transform names to bring them up to date with current taxonomic standards. The infrastructure can monitor and capture metadata from digital resources, taxonomically update and cross-link electronic datasets of relevance to biodiversity, and make discovery metadata available to the Linked Open Data Cloud. The infrastructure will transform multiple overlapping free-standing taxonomic databases, digital resources, and services into a virtual pool; and through it accelerate access to available information on any taxon. As such, GN will be a part of the processes that will transform life sciences into a more unified and data-centric "Big New Biology".

The Global Names Architecture is a multi layered architecture taking care about different aspects on creating and managing controlling taxonomic ontologies and developing the relevant strategies and (open) semantics for efficiently sharing and integrating biodiversity data.

The Global Names Architecture was conceived by the Encyclopedia of Life (EoL) and the Global Biodiversity Information Facility (GBIF). In Europe the GNA efforts are extensively supported by the pan-European Species-directories Infrastructure (PESI) project[1] and via the European contributions to GBIF-ECAT.

In 2011, the US National Science Foundation invested in the US-based Global Names project[2] attempting to provide an infrastructure for unifying taxonomic databases and services for managers of biological information, especially improving the imbedding of the nomenclators within the Global Names Architecture.

The here proposed European contribution to the Global Names Architecture, provisionally called GN-EU, is supposed to proceed from this US Global Names efforts. It will transform the prototype developments into a persistent infrastructure that will improve visibility, integration and re-use of biodiversity information, providing a robust virtual environment for nomenclators, particularly profiling ZooBank as a registration service for names of animals, and an array of other names-based tools and services that have demonstrated the value of a names-based approach to biodiversity data management. This will be done in collaboration with the US-based Global Names project, sharing all software and content, which is openly available.

To illustrate the potential of a names-based infrastructure, one component would be tools and services that can be applied to a wide diversity of file types in a heterogeneous storage environments, capable of recognizing the file types, opening the files, and then applying algorithms that recognize names (because they occur in the Global Names infrastructure) or discovery names because of their distinctive format.

---

1 EC-FP7 contract: RI-223806 – http://www.eu-nomen.eu/pesi

2 NSF grant: DBI-1062387 – http://globalnames.org

Names can be extracted an associated with data centres, data files, or atoms of data and made available through User Interfaces, Application Interface Services, or to the Linked Open Data Cloud, where they can act as discovery-level metadata. Such tools are scalable, replacing very costly manual addition of metadata. Prototypes have already been developed and are being positioned as automated metadata discovery tools for the NSF Data Conservancy project, and now ready for improvement into scalable indexing services.

The value of such tools is evident because they will promote progress in the following Fiches:

Fiche 01, community support data services. Automated name recognition and discovery will make data visible through local and centralized indexing services supporting a wide range of science community services, including biodiversity research, environment management, and food protection. Furthermore a proper integration of controlled (authoritative/standardised) and open semantics, will accomplish the appropriate application of policy and science standards in the information domain.

PESI is formally adopted as a European standard as part of the INSPIRE directive. This means that EU countries and projects will have an obligation to contribute to the maintenance of the PESI infrastructure and the implementation of taxonomic standards at the European and local level. This could be an incentive for DG CONNECT to support associated e-Infrastructural developments.

Fiche 02, infrastructure for Open Access. PESI actively contributes on developing Open Access policies for biodiversity data.

Fiche 03, storing, managing and preserving research data. Automated name recognition and discovery will allow content to be indexed such that data centres better meet the needs of research communities by allowing them to find relevant data.

GNEU contributes to the sustainability of EU-based nomenclators, like IPNI, Index Fungorum, AlgaeBase and ZooBank, by further developing a common access and management layer (the so-called GNUB). Establishing ZooBank can be considered a collaborative US-EU endeavour.

Fiche 04, discovery and provenance of research data. PESI will feature SMEBD[3], representing the integrated European taxonomic workforce on practical data governance. In addition, PESI will facilitate the training of individual experts, experts groups and Focal Points expert networks on sharing best practises on GNA advancements. This training capacity will be increased, profiling SMEBD as a SME in the Horizon2020 program (also relevant for fiche 07).

Fiche 05, towards global data e-infrastructures. A standard indexing system coupled with UUIDs for names, with services that manage the problems of many names in many forms for one species and one name for many species, will provide a unifying standardised framework for access to biodiversity data. GNEU, situated within the Global Names Architecture developments and proposed as a US-EU collaboration, naturally secures a global consensus on data governance, exchange and interoperability issues. Further PESI is contributing to global standardising bodies like TDWG and is part of the LifeWatch construction plan.

Fiche 06, towards authentication and authorisation e-infrastructures. PESI has an extended Focal Point network and a considerable experience on coordinating distributed e-Infrastructural tasks within a federated European context. In addition, triggered by the GEANT project, PESI is selected by the divers EC bodies (DG-INFSO, DG-EAC, DG-RTD, DEVCO) to outreach European e-Infrastructures for the Eastern Partnership countries. This has different advantages, including the potential (distributed)

[3] http://www.smebd.eu/

outsourcing of e-Infrastructural developments and the efficient implementation of established infrastructures within a broad European sense.

Fiche 07, skills and new professions for research data. PESI will use both the network of Focal Points (outreaching to national educational programs), SMEBD (see finche 4) and the available European institutional networks represented by for instance CETAF/EDIT and SYNTHESIS on sharing knowledge and contributing to European curriculums for Biodiversity Informatics education.
