# Supplementary material for: PESI - a taxonomic backbone for Europe
Source: Biodivers Data J. 2015 Sep 28;(3):e5848. doi: 10.3897/BDJ.3.e5848 (PMC4609752; doi:10.3897/BDJ.3.e5848)
Supplement: Supplementary material 19 — PESI Report on the criteria, procedures and mechanisms for quality control [file biodiversity_data_journal-3-e5848-s019.pdf]

A pan-European Species-directories Infrastructure (PESI)

PESI WP5 — Deliverable D 5.2

Report on the criteria, procedures and mechanisms for quality control

Background

The Pan-European Species directories Infrastructure project (PESI) implements an integrated taxonomic information system for the major European checklist initiatives, starting with Euro+Med plantbase (E+M), Fauna Europaea (FaEu), and the European Register of Marine Species (ERMS). Apart from its role as a provider of standardized, harmonized, and authoritative taxonomic data, PESI will have a special focus on data quality.

This document summarizes the measures PESI will take to detect and address quality problems in the participating checklists and how this feedback will be reintegrated by information providers. The implementation of these measures will greatly improve the value of both PESI and contributing checklist information systems.

PESI information flow

The PESI information system will be set up using the Internet Platform for Cybertaxonomy implemented by the "European Distributed Institute of Taxonomy" (EDIT, http://wp5.e-taxonomy.eu/) and in particular a CDM store based on the EDIT Common Data Model.

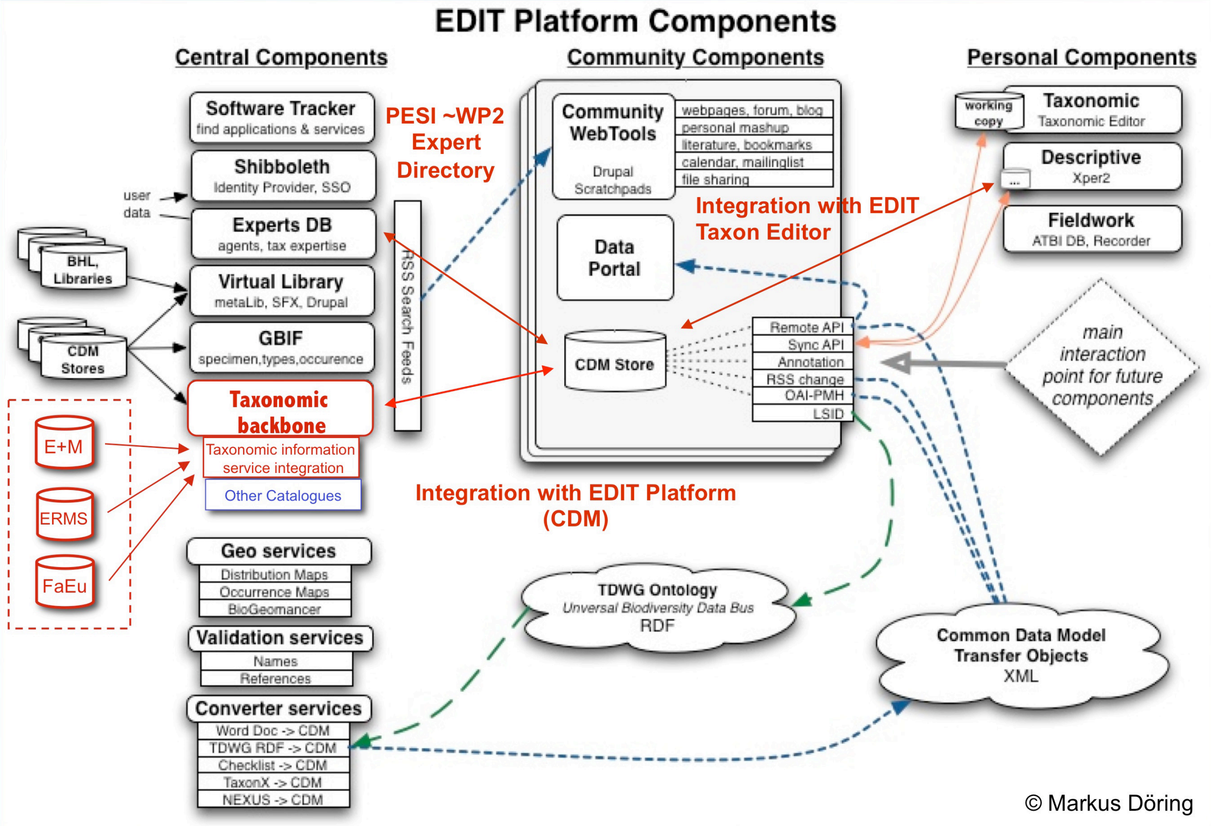

Fig. 1: Outline of the implementation of PESI components within the EDIT Cybertaxonomy Platform, including the CDM store.

The Common Data Model (CDM) is the domain model for core components of the EDIT Platform for Cybertaxonomy. The CDM, as an information model, is primarily based on the TDWG Ontology (http://wiki.tdwg.org/twiki/bin/view/TAG/TDWGOntology) and the information models from which it was derived, especially the Berlin Taxonomic Information Model (http://www.bgbm.org/biodivinf/Docs/bgbm-model/documentation.htm) and the BioCISE Model for Specimen and Observation Data (http://www.bgbm.org/biodivinf/docs/CollectionModel/). Other models and standards, such as taxonomic data standards, bibliographical data standards, and specimen or observation standards also influenced the modelling.

The CDM is a normalized object-based format developed in the Unified Modeling Language (UML, http://www.uml.org/). It covers the entire taxonomic information flow from fieldwork to printed and electronic publication. The CDM also acts as an information broker for existing biodiversity informatics applications such as descriptive tools, taxonomic database systems, and specimen and observation management systems. The CDM and its Java-library API offers version control for all important entities including names, taxa, references, and media for example. Interfacing between external applications and CDM data stores is done primarily using TDWG XML-based standards such as TCS, ABCD, and SDD (http://www.tdwg.org/standards/), as well a CDM/XML format. RDF exports and imports will be implemented at a later project stage; the import of TCS/RDF has already been tested successfully. The EDIT Cybergate (http://dev.e-taxonomy.eu/platform/) is a tool for gaining an overview of EDIT Platform components and demonstrating their interoperability and connectivity.

All participating checklists will either be maintained within a PESI CDM store instance, or maintained externally and regularly imported into the CDM store (fig. 2).

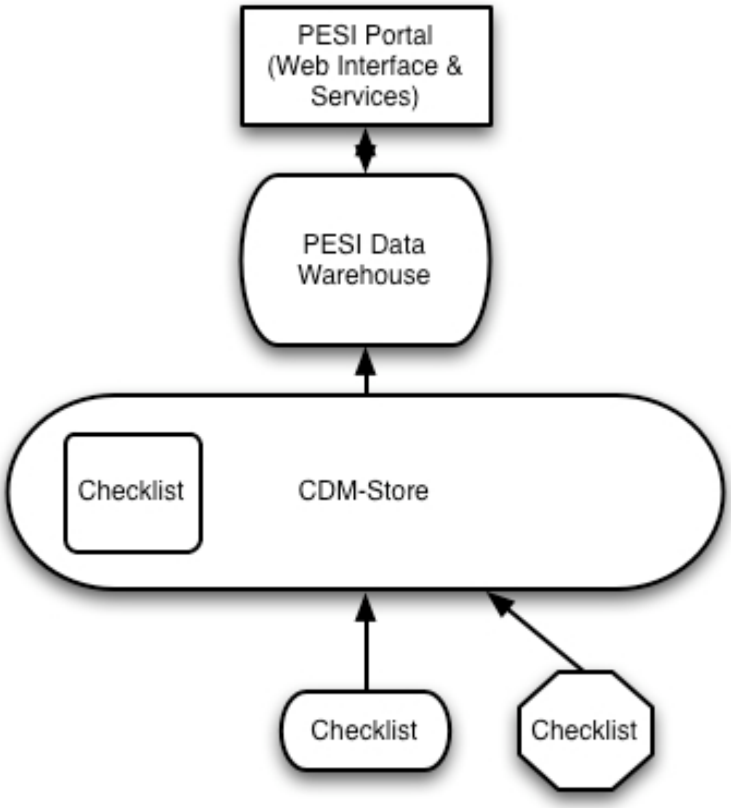

Fig. 2: Simplified PESI dataflow from checklist data providers through CDM store and PESI data warehouse to the PESI World Wide Web portal.

From the CDM store, PESI data will be exported into a denormalised relational database management system (the "PESI data warehouse" presently implemented with Microsoft SQL-Server) optimized for queries from the World Wide Web portal and PESI web-services (fig. 3).

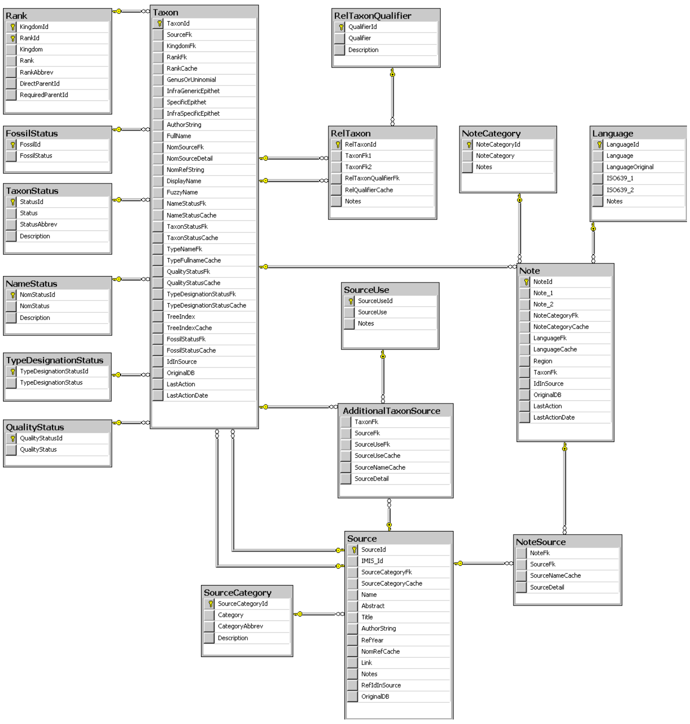

Fig. 3: E/R-diagram of the PESI data warehouse (version 0.7).

Within this information flow, data quality problems will be detected i) during the import of checklist data into the CDM, ii) by means of special data quality software used for data already stored in the PESI CDM store, and iii) through Portal User feedback to the participating checklist databases.

Integrity Checks

There are four levels of integrity to be checked during or after the import of source checklists into the CDM:

Level 1 - Syntax of terms: at the lowest level, the syntactical correctness of terms occurring in the data will be checked. Examples of syntax rules to be applied include:

- A genus-group or higher taxon name must start with an upper-case character followed by lower-case characters and can not contain any diacritics.
- A URL must follow the syntax defined at http://www.w3.org/Addressing/URL/5_BNF.html.
- Date and time information must follow the format specified in ISO8601.

Level 2 - Structural integrity: the second level of integrity checks will focus on the completeness and appropriateness of information belonging to individual objects. Examples of rules for detecting structural problems include:

- A taxonomic scientific species name must have a genus name and a species epithet.

- A scientific name should have an authority.
- A bibliographic reference must have a year of publication.

Level 3 – relational integrity: at the third level, the correctness of relations between objects will be analysed. Examples of rules enforcing relational integrity include:

- A synonym must be linked to an accepted taxon.
- A genus must have at least one species.
- A URL must refer to accessible content at the given address.

Level 4 – dataset integrity: the highest level of integrity checks detects contradictions between different datasets (checklists). The set of rules may include:

- The same name appearing in different checklists should not have different status values.
- Reference strings should not use different spellings when referring to identical references.

Integrity rules will not necessarily be enforced when a violation is recognized, for example, by rejecting data that do not conform to the given rule. Instead, in many cases the problem (or potential problem) will be highlighted and reported back to the data provider for further consideration.

The majority of syntax rules will be implemented by the CDM business logic so that malformed data will already be highlighted during import. Other problems will be detected by new CDM integrity checker software. A good basis for this software is the integrity checker implemented in the course of Berlin Model development, which comprises hundreds of integrity rules formulated as SQL statements. Fauna Europaea has also compiled a comprehensive list of 146 "Taxonomic Business Rules" which are used for several integrity checker tools (online and offline, http://www.faunaeur.org/about_fauna_data_entry.php).

PESI and EDIT maintain a joint compilation of taxonomic integrity rules on the EDIT WP5 developers Wiki at https://dev.e-taxonomy.eu/trac/wiki/IntegrityRulesEditPESI. The list of rules is constantly extended based on communications with taxonomists from all organism groups as well as the maintenance bodies of the contributing European checklist databases.

User feedback

Apart from automatic detection of problematic data by the import and integrity checker software, an important contribution to data quality issues will be user feedback, from contributing regional and taxonomic experts as well as from end users of the PESI portal, to debate and review the pan-European checklist data. Ideally, experts should have the opportunity to assess PESI output before publication for the wider public. The CDM data portal software is perfectly suited for this task and could provide a protected view of the data held in the PESI CDM store as an extra public layer. Problems in the data could than be fed back into the system using annotations. This common validation will also increase the acceptance of the pan-European checklist as a standard among a broad set of users.

The end user of the PESI portal should also be given the opportunity to report potential problems back to the checklist data holders. To this end, a feedback mechanism should be implemented into the portal to store all annotations transparently on a central server and send reports with all annotations and the information items they reference back to the information provider. A model for this mechanism could be the annotation system prototype for specimen and observational data implemented in the framework of SYNTHESYS (see http/::www.marinebiodiversity.ca:OBI07:sessions:metadata-developments:oral-guntsch). The system stores all annotations on a subversion server as a version of the original (xml) data record. Using this data, providers can compare the differences between the original record and the annotated record using standard subversion clients. Alternative models for review layers below the expert level have been implemented by the Swedish Artportalen initiative (http://www.artportalen.se) as well as GlobalTwitcher (http://www.globaltwitcher.com/). As long as a sophisticated feedback mechanism has not been implemented, the PESI portal will use a simple email report system to ensure that User feedbacks can be incorporated from the beginning.

Data quality control procedures

Data quality measures for the PESI information system will be carried out with three recurrent procedures (Fig. 4). Quality checks, which are part of the import process, will create a data quality report every time a data import has been conducted. The report will be fed back to the maintenance body, which is responsible for the maintenance of the respective checklist. Dataset integrity violations (level 4, e.g. disparities between identical taxonomic groups in different checklists) will be sent to taxonomic or regional experts for clearance. Level 1-3 integrity problems will not need taxonomic expertise in most cases and be resolved at checklist maintenance level.

Checklists, which will use the CDM as their central project database (e.g. Euro+Med), will benefit from integrity checks as part of the import procedure only once and rely on the CDM integrity checker henceforward. All other checklist will undergo an import quality check whenever a new version is imported into the PESI information system.

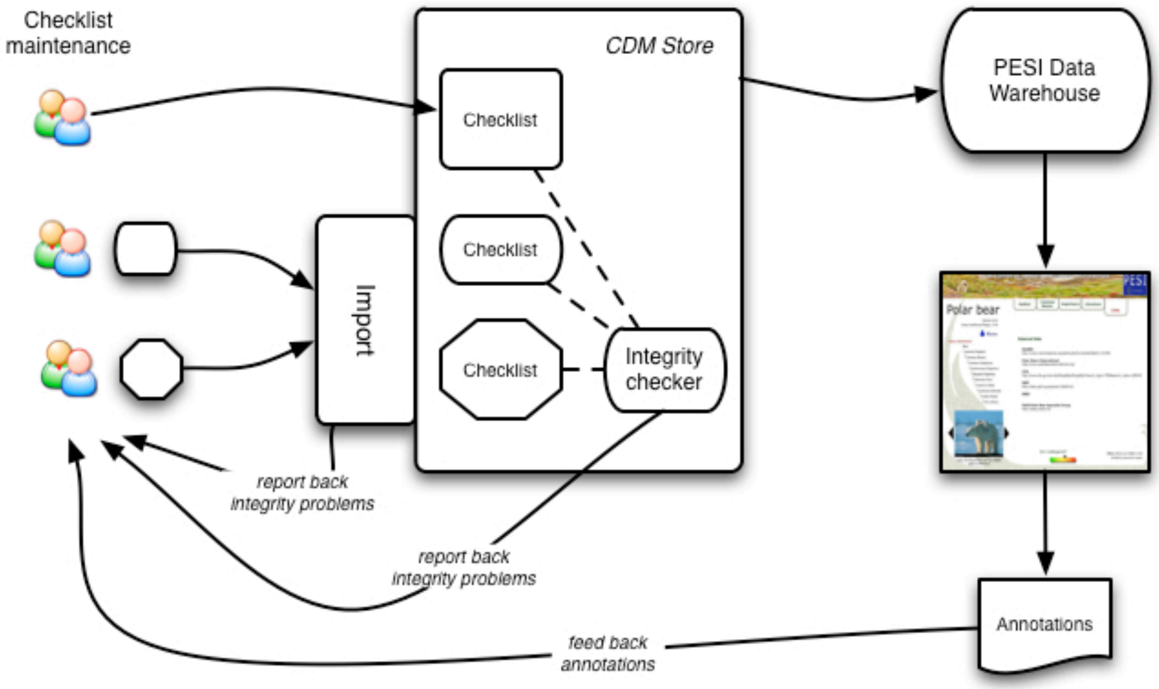

Fig. 4: Data quality procedures

The second data quality procedure is the CDM integrity checker, which is built into the PESI CDM store. This procedure will be carried out cyclically depending on the dynamics of changes within the PESI data warehouse. Experiences from the Euro+Med plantbase suggest to run the integrity check every other month and additionally after significant changes in the database (e.g. after import of larger amounts of data). Results of the integrity check will also compiled in a report, which is sent back to the checklist maintenance bodies.

User annotations will be the third instrument contributing to the improvement of PESI data and information within the participating checklist systems. As explained in the User feedback section, we envisage the deployment of an annotation server for this purpose, which will be based on an existing framework such as the BioCASE/SYNTHSYS annotation system. For the short term, the PESI User annotation system will be implemented following the GBIF-model. This means that the PESI portal will collect User annotations and cyclically transmit a report to the checklist maintenance bodies. In the same manner as the first two quality procedures, the respective checklist managers will decide which annotation leads to a correction which can be made directly in the checklist database and which annotation has to be fed back to a taxonomic or regional expert.

Configuration History			
**Version No.**	**Date**	**Changes made**	**Author**
0.1	18 April 2009	First draft for circulation within WP4, WP5, WP6	AG
0.2	29 April 2009	Updated version of D5.2 (including comments from WP4, WP5, WP6)	AG
1.0	26 May 2009	Final first version of D5.2	AG
2.0	15 September 2009	Version for re-submission following 1st Year Review	AG
