# Supplementary material for: PESI - a taxonomic backbone for Europe
Source: Biodivers Data J. 2015 Sep 28;(3):e5848. doi: 10.3897/BDJ.3.e5848 (PMC4609752; doi:10.3897/BDJ.3.e5848)
Supplement: Supplementary material 20 — PESI joint e-infrastructure disseminating pan-European checklists [file biodiversity_data_journal-3-e5848-s020.pdf]

Joint e-infrastructure disseminating Pan-European checklists

Summary

The Pan-European Species directories Infrastructure project (PESI) implements an integrated information flow for taxonomic data provided by the major European checklist initiatives starting with Euro+Med plantbase (E+M), Fauna Europaea (FaEu), and the European Register of Marine Species (ERMS). As a result, all checklists will use a unified infrastructure for propagating taxonomic information from the provider databases to their integrated publication in the PESI portal in human- and machine-readable form.

PESI has chosen to deploy the EDIT Platform for Cybertaxonomy (http://wp5.e-taxonomy.eu/) as its common technological backbone. The Platform is a collection of tools and services covering all aspects of the taxonomic workflow. Being generic with respect to organismic groups it provides the ideal ground for the integration of European checklists.

Deliverable D 5.3 is the prototype for this e-infrastructure which is now up and running at the BGBM Berlin. It will be further refined within the last project year based on experiences drawn from PESI publication cycles as well as User feedback and further standardization activities.

Components of the e-infrastructure

Data integration from the source checklists to the publication-ready data warehouse structure is a three-step process consisting of a data import, a data merge process, as well as an export into a structure optimized for data publication purposes (Fig. 1).

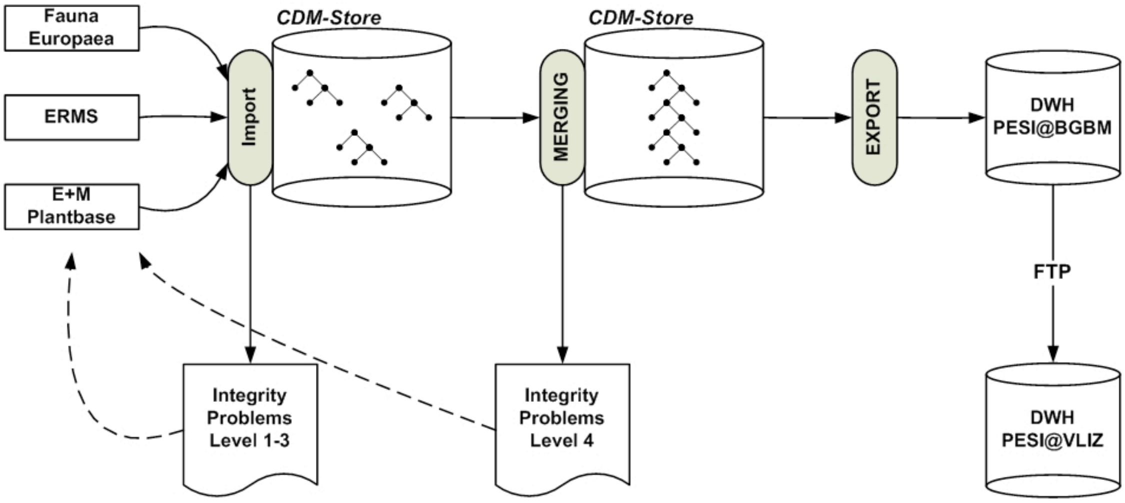

Fig. 1: Components of the PESI e-infrastructure

Import and Merging processes are associated with data quality measures producing reports which are fed back to the respective checklist data management bodies.

CDM Import modules

Imports of checklists into a CDM store belonging to the EDIT platform for Cybertaxonomy are carried out with import-software implemented for each of the major checklists Euro+Med, Fauna Europaea, and ERMS. The software uses the EDIT Platform CDM Java library (http://dev.e-taxonomy.eu/trac/wiki/CdmLibrary), which provides powerful methods for parsing and analysing taxonomic checklist data. In particular, quality problems at the following levels are detected:

- Level 1 (syntax of terms): syntactical correctness of individual terms such as a Genus epithet, which has to start with a capital character followed by lower-case characters.
- Level 2 (structural integrity): completeness and appropriateness of data belonging to a single object such as a species name which has to have species epithet and a genus epithet.
- Level 3 (relational integrity): correctness of relations between objects such as synonyms which have to be linked to an accepted taxon or URLs which have to refer to an existing web content.

```
Start import from (ERMS) ...
12:03:08,298  INFO LocalSessionFactoryBean:729 - Building new Hibernate SessionFactory
12:03:10,939  WARN BacterialNameDefaultCacheStrategy:32 - BacterialNameDefaultCacheStrategy not yet really implemented. Its
just a copy from BotanicalNameDefaultCacheStrategy right now !!
12:03:19,955  INFO HibernateTransactionManager:415 - Using DataSource
[eu.etaxonomy.cdm.database.NomenclaturalCodeAwareDataSource@1481b9a] of Hibernate SessionFactory for
HibernateTransactionManager
Start checking Source (ERMS) ...
12:03:48,159  WARN ErmsRankImportValidator:35 - Checking for ranks not yet fully implemented
12:03:48,159  WARN ErmsReferenceImportValidator:35 - Checking for references not yet fully implemented
12:03:48,174  WARN ErmsTaxonImportValidator:35 - Checking for Taxa not yet fully implemented
12:03:48,659  INFO Source:259 - Connected to ERMS
=========================================================
12:03:48,909  WARN ErmsTaxonImportValidator:66 - There are accepted taxa that have an unaccepted parent and also the parents
accepted taxon (tu_acctaxon) is not accepted.
=========================================================
ChildId:117123
   childName: Hydrichthys
  ParentId: 22802
   parentName: Hydrichthyidae
   parentStatus: unaccepted
  ParentAccId: 15029
   accParentName: Pandeidae
  accParentStatus: accepted
ChildId:137649
   childName: Ancistrocheirus
  ParentId: 22986
   parentName: Ancistrocheiridae
   parentStatus: unaccepted
  ParentAccId: 22987
   accParentName: Ancistrocheirinae
  accParentStatus: accepted
[ ... ]
```

Fig. 2: Snippet of the integrity problem report generated by CDM-PESI import modules.

The definition of levels follows the specification given by the "Report on the criteria, procedures and mechanisms for quality control" (PESI deliverable D 5.2). The import modules automatically generate a report containing a compilation of syntax and integrity problems found which are fed back to the responsible checklist managers for further consideration (Fig. 2).

The import software provides also the necessary mappings of individual data standards used by the source checklists to agreed standards within the PESI system. In particular, the projection of formats for distributions of organisms and the associated distribution statuses are performed during the import phase.

The result of the import process is a single CDM store containing independent taxonomic trees for all checklists which are ready for merging in the next step.

Checklist Merging

Once imported into an EDIT CDM store the checklists have to be merged into a single and consistent taxonomic tree which forms the basis for publication through the PESI portal. For this, merging software has been developed which detects overlaps and conflicts between the participating checklists (integrity level 4) and resolves them according to rules defined by the affected checklists. Presently, overlaps and integrity problems occur between Fauna Europaea and ERMS for a limited number of taxa. Both checklists decided that the ERMS taxonomy will be given priority within the PESI prototype system and a more sophisticated set of priority rules will be developed later in a joint effort.

Comparable to the import routines, the merging module produces a detailed (Microsoft Excel) report containing a compilation of taxonomic derivations between the checklists processed. This report is also fed back to the respective checklist management bodies, which either have to agree on a common taxonomy for the affected taxonomic groups or provide an implementable rule set as a basis for an automatic priority decision taken during the merging process.

The result of the merging process is a single CDM-store containing a consolidated taxonomy across the participating organism groups.

Export Module

Finally, the data have to be exported into the PESI data warehouse structure, which is a representation of PESI data optimized for efficient queries and output at portal level. Compared to the complex and highly atomized structure of the EDIT Common Data Model, the PESI data warehouse has only a few tables containing pre-processed data as it is required at presentation-level.

The data warehouse has two instances at BGBM (Berlin) and VLIZ (Oostende), both running on a Microsoft SQL-Server 2008. It was decided that data transfer from the central PESI backbone to the publication site at VLIZ will be implemented via FTP as long as the update frequency within the CDM is comparably low. New available versions of the consolidated checklist can be automatically detected by a naming convention on the BGBM FTP-server. If publication cycles become more frequent in the future VLIZ and the BGBM are ready to start a synchronous mirroring of the checklist data by using the SQL-Server synchronisation mechanisms.

Next steps

The PESI e-infrastructure provides the full information flow from the participating checklist projects, through the EDIT Platform for Cybertaxonomy to the data publication facilities provided by work package 6. We will further refine this approach in particular by implementing additional rules into the checklist merging module. This will help us to fine-tune the decision-process associated with conflicting taxa within the checklists that have to be processed.

In addition, we will work on the integration of additional checklists starting with Fungi and *Desmidiaceae*. For future imports we are aiming at a more standardized process which makes use of an agreed intermediate format supported by both the data provider side and the CDM data import layer. With this we hope to avoid the necessity to implement individual import modules for every new participating checklist. A promising candidate for such a data format is the "Darwin Core Archive" (http://www.gbif.org/informatics/standards-and-tools/publishing-data/data-standards/darwin-core-archives/), which has a relatively simple taxonomic core structure, which can be extended for specific purposes. Darwin Core Archive is also the data export format recommended for PESI by it work package 4.

Finally, further integration of related and external biodiversity data services will be investigated. This includes the development of a prototype for the integration of distribution data from the PESI system and specimen and observation data accessible through the Biological Collection Access Service for Europe (BioCASE, http://www.biocase.org/). The comparison of point occurrence data and polygon distribution data will provide a powerful measure for the assessment of data quality and gap analyses.

Configuration History			
**Version No.**	**Date**	**Changes made**	**Author**
1.0	11 May 2010	First version D5.3	AG
