# Supplementary material for: PESI - a taxonomic backbone for Europe
Source: Biodivers Data J. 2015 Sep 28;(3):e5848. doi: 10.3897/BDJ.3.e5848 (PMC4609752; doi:10.3897/BDJ.3.e5848)
Supplement: Supplementary material 21 — Versioning and the use of GUIDs for PESI [file biodiversity_data_journal-3-e5848-s021.pdf]

Versioning and the use of GUIDs for PESI

Anton Güntsch, Walter Berendsohn & Marc Geoffroy

Background

Over the last couple of years the Biodiversity Informatics community underwent a vivid debate on the implementation of persistent Globally Unique Identifiers (GUIDs) for the object types to be networked in the emerging biodiversity data infrastructure (see http://wiki.tdwg.org/GUID). The discussion has not been fully concluded but it seems that the LSID specification (http://wiki.tdwg.org/twiki/bin/view/GUID/LSID) is a promising candidate for common identifier technology for the community.

The choice of a common technical approach would obviously be a great step forward towards a truly networked and harmonized information space for biodiversity sciences. However, strategies and policies for the way how GUIDs will be assigned to objects by whom and which operations on objects will have to lead to the assignment of new GUIDs have never been sufficiently considered. The PESI infrastructure, approaching its operational prototype phase in May 2010 needs such a strategy and will have to find a pragmatic and implementable solution to the GUID problem which will not overburden the participating checklist databases as well as the work packages implementing the PESI infrastructure.

This paper summarizes a strategy for implementing GUIDs for PESI, which is relatively easy to implement and does not put risk at the present work plan. It is also an answer to the PESI mid-term review that asked for a more thorough analysis of the project aspects related to identifiers, versioning and proper archiving.

Which object types should have GUIDs?

Basically, all kinds of objects dealt within the biodiversity informatics domain (e.g. authors, author teams, references, citations, scientific names, vernacular names, taxa, collection sites, collections) are candidates for the assignment of GUIDs. However, for PESI we propose to restrict the assignment of GUIDs to scientific names (i.e. nomenclatural entities and not name strings) and taxa (a scientific name used in a certain context). We believe that PESI will primarily be used as authoritative resource for these two core object types and should offer an infrastructure as capable as possible to serve other networks. Following the example of the implementation of names and taxa GUIDs the approach can be extended to other object types at a latter PESI phase.

Who will create GUIDs in the PESI Network?

In the first PESI project year the question of who should assign GUIDs has already been discussed in an email conversation between Euro+Med plantbase, ERMS, and Fauna Europaea. The conclusion was, that

- each participating checklist will be responsible for assigning GUIDs to their objects.

- The GUIDs can be "raw" and do not have to follow a specific protocol (example: B85E62C3-DC56-40C0-852A-49F759AC68FB). ERMS and Euro+Med are running on a Microsoft SQL-Server and can use its ability to generate GUIDs. Fauna Europaea should check whether a comparable feature is available for Oracle as well. If not, existing web-services or relatively simple functions can be used to generate GUIDs using a combination of the server's MAC-Address and the time for example. Alternatively and even more pragmatic, the BGBM could fill a table with any number of pre-calculated GUIDs which can then be downloaded and used by Fauna Europaea.

- The GUIDs will not replace the checklist's internal identifier systems. They represent a parallel system exclusively used for proper interfacing and networking.

- Actionable GUIDs following a specific protocol (e.g. LSIDs, to be decided) are generated at PESI portal level using the GUIDs generated by the checklists as a component. With this, the protocol level can be decoupled from the GUID production so that protocol changes (which are quite likely to happen in the future) can easily be implemented at portal level without messing up the entire PESI network.

When do we assign a new GUID for a given object?

A critical question is: which operations to a given object turn it into a new object so that a new GUID has to be assigned. There are many philosophical aspects to this question, which should be discussed within the entire community. In particular, a grave problem is that taxonomic databases usually store the results of a taxonomic process and not the process itself and that databases cannot distinguish whether information added to a taxon is just a completion of an existing concept or an operation associated with a conscious concept change.

Given that we cannot expect an agreed answer to these and other questions for the near future and that PESI has to go forward with the construction of its infrastructure, we have to come up with a simple and implementable set of rules now. We propose the following strategy for taxa (accepted and synonym) and scientific names:

Accepted taxa

**Operation**	**New GUID?**
Change name/nomenclatural author (excluding orthographic corrections)	yes
Change (taxon) reference	yes
Change associated factual data (e.g. distribution data, threat status, uses, images, etc.)	no
Add/remove homotypic synonyms	no
Add/remove heterotypic synonyms	yes
Change status (from accepted to synonym)	yes
Move taxon to a different parent (not for taxa not yet having a parent)	yes

Change included taxa	no

Synonyms

**Operation**	**New GUID?**
Change name/nomenclatural author (excluding orthographic corrections)	yes
Change (taxon) reference	yes
Change status (from synonym to accepted)	yes
Move synonym to a different accepted taxon	yes

Scientific names

**Operation**	**New GUID?**
Change authorship (also year in zoology)	yes
Orthographic changes	no
Change nomenclatural Reference (excluding orthographical and numerical corrections)	yes

There have to be clear and preferably automatable rules defining, which syntactical changes to a name, authorstring, and nomenclatural reference are considered orthographic and which changes do lead to a new object. The rules do not have to be necessarily identical for the three checklist, but they should be well-defined and publicly accessible.

Assignment of new GUIDs

In each participating checklist the name and taxon tables/classes should be extended with two attributes "GUID" and "DerivedFromGuid". The GUID field receives a new value whenever one of the GUID-changing operations is performed on the object whereas the DerivedFromGUID field contains the object's GUID, which was valid the last time a proper version of the respective checklist has been published.

Obviously the two fields are not sufficient to store the entire derivation history of objects within the checklist. But they offer a simple way to maintain the association between objects at a very basic level. With this, a service could for example state that for a given taxon prior or later versions do exist and where to find them. This would be a big improvement compared to our existing systems, which publish always the latest checklist version and do not preserve the history of objects.

At the beginning it was said, that one of the major problems in the GUID arena is that taxonomic databases usually do not store taxonomic operations, which makes it practically impossible to calculate in a reliable way whether a taxonomic concept has been changed as a consequence of data changes belonging to a given taxon. We therefore

recommend that - in addition to the rules described in the last section - each checklist should provide a tool for taxonomists, which can be used to enforce the generation of a new GUID. This could be a simple button in the taxon-page of a taxonomic Editor saying "concept changed" or a tick-box in an Excel sheet for example.

How do we preserve versions in PESI?

The question was raised during the PESI mid-term review and needs an agreed answer. We propose the following strategy:

- PESI will support the publishing of versions at service level.
- The PESI web-portal (for human consumption) will only provide the latest version.
- The services will be based on defined versions (e.g. one per year) stored in parallel data warehouse installations at VLIZ and BGBM.
- Access/links to objects in PESI versions will be preserved "forever" so that external networks referring to PESI objects using the services can rely on their future existence.
- In addition to offering the metadata for a given GUID the services will offer information about the existence of later or prior versions of the object (if any) and provide their access points.

The PESI (data warehouse) versions will also be used for archiving purposes. A format has to be decided. We suggest creating an export into a simple txt- file to avoid SQL-Server Versioning problems. A documentation of the data warehouse tables, relations, and fields and their meaning could also be added to this file in text form.

**Configuration History**			
**Version No.**	**Date**	**Changes made**	**Author**
1.0	14 January 2010	First draft	AG, WB, MG
2.0	25 January 2010	Comments from Euro+Med included	AG, WB, MG
3.0	24 March 2010	Comments from Fauna Europaea and ERMS included	AG, WB, MG
