# Supplementary material for: PESI - a taxonomic backbone for Europe
Source: Biodivers Data J. 2015 Sep 28;(3):e5848. doi: 10.3897/BDJ.3.e5848 (PMC4609752; doi:10.3897/BDJ.3.e5848)
Supplement: Supplementary material 22 — Working plan to support European GSDs maintenance and updating [file biodiversity_data_journal-3-e5848-s022.pdf]

Working plan to support European GSDs maintenance and updating (PESI Deliverable D5.1)

Authors: Pascale Bezard-Falgas, David Ouvrard, Thierry Bourgoin

This report constitutes deliverable D5.1 of Work Package 5 (WP5) of the Pan-European Species-directories Infrastructure project (PESI). The document address mainly the issue of setting up a European infrastructural component in order to assist and defend existing Global or Regional Systematic Databases (GSDs/RSDs) dealing with European information for a particular taxonomic sector of the classification. It provides recommendations, missions and actions to set up in order to mobilise the data and information stored by these taxonomic databases and to make them available into the digital space of research. Accordingly, it will therefore serve also a future vision about the links and relationships that should be established between the future European PESI database/portal and these taxonomic databases.

Because of obvious close link, the last part of this report provides another deliverable of PESI (due for month 32) as draft version. It concerns the GSD Cost in view to quantify financial impact of GSD maintenance to include in a general financial plan.

1. The importance of GSDs

1.1. What is a GSD?
1.2. Role of GSDs / Why GSDs are needed
1.3. The GSD maintenance challenge
1.4. Credit and source lost: the multi-versioning problem

2. Infrastructural component: Organising the European GSD community

2.1. General objectives to consider
2.2. The need of an stable Infrastructural component
2.3. Actors in place: what already exists
- SMEBD
- PESI
- Species 2000

3. Organising the European GSD community: missions to cover and actions to take on

3.1. Foreseen missions
3.2. GSD website

4. Updating & speeding-up the taxonomic indexing activities with better quality control

4.1. Data interoperability
4.2. Data flow
4.3. Data quality control

5. Governance and financial issues

5.1. Infrastructure governance
5.2. Financial maintenance of such an infrastructure

6. Identify the Costs (PESI M.32) [draft 1.2]

6.1. Technical component (maintaining a GSD)
6.2. Data component (maintaining a GSD)
6.3. A formula to use to evaluate a GSD cost.
6.4. Financial plan to ensure long-term maintenance of European GSD network (M32)

1. The importance of GSDs

1.1. What is a GSD?

Global Systematic Databases or Regional Systematic Databases are *systematic expert databases* focussing on a particular sector of the classification of life forms at the global (GSDs) or regional level (RSDs). These include various taxonomic products (see WP4, D4.1), more or less structured (check-lists, relational databases, …) delivering taxonomic information with, or without, various associated biological information (as distribution, etho-ecological data, depositories, …). In this report we use GSD in a general sense including RSDs.

GSDs are systematic expert databases that maintain a structured access to a sector of specific taxonomic knowledge

1.2. Role of GSDs / Why GSDs are needed

- GSDs represent the first step in collecting data in a structured way and organising them at the appropriate place in a digital space of research. Accordingly, data need to be atomised at their unit of information or aggregated or linked at the conceptual level needed allowing their re-usage by the scientific community or by any other users and fields of interest.

- Roles of GSDs are *to gather and to validate nomenclatural and taxonomic information* and their associated primary biological data into a comprehensive classification system through a structured format (generally a database). These databasing actions placed into a network perspective allow circulation of taxonomic information/knowledge in the digital space of research.

- Part of this task begins to be electronically processed as for data capture in taxonomic literature with OCR and code compliance tools, or even for assemblage/partition of the information collected (ranking, competitive classifications, homonymy, synonymy, chresonymy, …). However if this task can be helped by IT tools in producing for instance Proto-GSDs (e.g. an electronically maintained and gathered set of taxonomic data for a specific taxonomic sector without specialist behind), the human expertise to manipulate these units of information is crucial for the quality of the information that needs to be validated before being delivered into the digital space for other usages and decision makings.

A GSD maintains and validates/certifies the link between the primary taxonomic knowledge production and the digital taxonomic knowledge usage

1.3. The GSD maintenance challenge

As any other scientific production, a GSD needs 1) to be funded, 2) to be based on a real scientific expertise that produces it, 3) to be published for recognition, and to be spread and used demonstrating its quality. But, also because *GSDs are evolving resources and services*, they need to be regularly updated which means that they need also 4) to address the challenging maintenance on the long term of the first three requirements (funding, expertise, delivering).

Funding and cost issues to secure GSD maintenance are specifically addressed in another task (WP5-Month 32), but first results (as a list of issues to be considered) are yet delivered at the end of this document (part 6). The scientific expertise issue based on the European GSD community together with the need of stable Infrastructural component (part 2) form the core of this report (parts 2, 3, 4 and 5). It does not address the European expert community which is specifically treated by the European network of Excellence on taxonomy (EDIT WP2) but the GSD community itself and its long-term maintenance. The specific problem of publication and recognition is addressed in part 1 in the following item.

1.4. Credit and source lost: the multi-versioning problem

Once entered into the digital sphere, taxonomic data begging to use used, amalgamated, partially or globally re-stored at numerous and un-controlled levels. In absence of accurate data tracing system and rules, the original source (GSD) which make the data available in the digital sphere is quickly no more recognisable, and their data are harvested by major initiatives also harvesting hits, credits and recognition to the detriment of GSD and their maintain.

Putting a name on a specimen is just a hypothesis and adding a specimen to a taxa enrich the species concept carried by the name. This is why concepts evolve and taxa and names change or are corrected. This is why GSD data change and why it is important to mention the data source and its version (= make available into the digital space).

> A recent example on the Taxacom network illustrates clearly the quick circulation of the data, the lost of the original source and the multi-versioning problem:
> R. Mesibov, Taxacom (30.VI.09, 07:20):
>
> "…My revision of the millipede genus Agathodesmus was published in the open access journal ZooKeys two weeks ago, on 18 June. A couple of days later I FTP'ed all the latest classificatory and other details on the two Australian species to the server hosting the Millipedes of Australia (MoA) website. ZooKeys is linked to ZooBank, so ZooBank also had the latest info.
> By Monday 22 June a Google search could find two up-to-date sources on Agathodesmus: ZooKeys and ZooBank. The MoA site, which topped the Google list, had 2 out-of-date pages listed (one no longer exists, the other lacks Agathodesmus), but you could find the new Agathodesmus info by going to the Checklist page once you were on the MoA site.
> A week later (yesterday) I dipped again into our gigantic external cultural memory to see how 'agathodesmus' was doing. Google still had the old MoA pages, not a problem. But it also showed many sites with out-of-date info, including an apparently authoritative Australian government site to which I added millipedes in 2002, and which is not regularly updated. EOL says the name Agathodesmus exists, as does ZipcodeZoo and the EDIT lists and some name-gathering sites I'd never heard of. Wikipedia seemed to be missing Agathodesmus.
> Today I tried searching Google on '2009 agathodesmus' instead of just 'agathodesmus'. Better: the top 6 listings are for MoA (2), ZooBank (2) and ZooKeys (2). Our Zipcode Zoo friends are no. 7 because their page has the copyright date "2004-2009". 8 and 9 are derivative of the Australian government site.
> The fascinating one is no. 10, from the Catalog of Life. This gives CoL LSIDs for the genus and its type species, which to me at least makes the site look up to date. However the taxonomy is incorrect…"

Paul Kirk's answer , Taxacom (2.VI.2006, 10:16):

There is nothing like a new product to attract customers - ask anyone in the real world. And in the case of the 'covert aggregators' (e.g. ZipCodeZoo, amongst others), who may add a link to the source web site ... thanks ... but ... taking traffic from the source has a detrimental effect on the profile of the source and thus the justification for maintaining it.

Recommendation:

- To investigate with TDWG a standard for the source recognition of the circulating data into the digital sphere. Any king of data should circulate with its original sources and successive change sources as for instance:

Taxonomic data / Taxonomic sources / Original database source // Change 1-n Sources

This report provides some ideas of what could be the missions and actions of an infrastructural component as a mechanism to set up to defend the GSD community.

2. Infrastructural component: Organising the European GSD community

2.1. General objectives to consider:

In organising the GSDs community, goals are to secure and extend their usability by better compatibility and, when needed or wished, to extend their functionality to support new projects and the up-coming new digital biology vision. The following objectives need to be considered:

- to secure Based-based GSDs.

GSDs are expert knowledge and expert taxonomic publication production based. This expertise can be acquired (a long term process) and can be passed on (in a few years), but it can also get lost (in a few months!) by lack of maintenance. The Taxonomic impediment, already formally recognized more than 20 years ago (Darwin Declaration, Feb. 1998), has clearly alerted on the vanishing of this taxonomist expertise. Recent review shows that the situation is still decreasing[1]. *Securing the expert knowledge assembled into a GSD should be considered as a top priority.*

- to support establishment of new GSDs.
- to increase value and usability of GSDs.
- to provide GSDs with their own, independent, professional body to advocate their interests
- to stimulate toward a common vision and program to ensure a proper acknowledgement and easier project participation.
- to help, supplement and re-enforce at the European level the role that plays Species 2000 at the international/global level (PESI as the European Hub of Species 2000).

2.2. The need of an Infrastructural component

In the frame of building a general European information infrastructure (e.g. Life*Watch*) a stable infrastructure component has been recommended to be organised. The following criteria should be taken into account:

- A distributed and organised network of smaller and diverse units (GSDs) interoperable between them is preferable rather than a centralized unique and hierarchic structure. The

[1] *(Bourgoin, Th., J.-F. Silvain. 2008. Systematics and Taxonomy. The scientific commission of the IFB. Strategic Study: 41-50. http://www.fondationbiodiversite.fr/Documents_files/Bilan%20CS%20IFB%20UK.pdf).*

diversity of the interoperable approaches allow progress and innovative solutions while a uniform constraining organisation will reduce its efficency at the short term.
- Granularity of the system is the expertise level that a GSD is able to efficiently cover at a given taxonomic level (in general a GSD covers at least several hundreds of taxa). For RSD it should be at least national. It means that flexibility is necessary to take into account the specificity of the various GSDs.

This component should be an independent and professional organisation that would become the chosen spokesman for the defence of network member rights and for the acknowledgement of their work.

Recommendations:
- A clear mandate of the roles of such an organisation acting as a lobby would have to be delivered with a clear distinction from the role of an academic European systematic association.
- At the European level, contact is recommended with the Consortium of European taxonomic Facility, CETAF, through a MoU for instance, recognizing this component as an operator for the CETAF in its fields of interest.
- Close discussions between SMEBD and Species 2000 are necessary as these consortiums already assure some of these roles for the GSDs.

2.3. Actors in place: what already exists

- **The role of SMEBD** (Society for the Management of Electronic Biodiversity Data):

http://www.smebd.eu/: "A society aiming to make biodiversity data available for the benefit of science and environmental management. This includes the archiving of electronic biodiversity data and encouraging and facilitating data being given an 'added value' by being combined and linked with other data.

SMEBD is currently funded by PESI.

- **The role of PESI** (A Pan-European Species directories Infrastructure):

PESI is a Project-project to organise a European taxonomic expertise network to ensure long-term updating of the GSDs and pan-European checklists. Particularly PESI provides models for interoperability with persistent land marks (standardised vocabularies) to build on for future biodiversity services. PESI will give access to local knowledge thanks to a national node network. PESI should provide the European component (European hub) of Species 2000.

http://www.eu-nomen.eu/pesi/: Expertise network(s) and e-Infrastructure(s) for pan-European species directories

"PESI provides standardised and authoritative taxonomic information by integrating and securing Europe's taxonomically authoritative species name registers and nomenclators (name databases) and associated exper(tise) networks that underpin the management of biodiversity in Europe.
PESI defines and coordinates strategies to enhance the quality and reliability of European biodiversity information by integrating the infrastructural components of four major community networks on taxonomic indexing into a joint work programme. This will result in functional knowledge networks of taxonomic experts and regional focal points, which will collaborate on the establishment of standardised and authoritative taxonomic (meta-) data.
PESI will coordinate the integration and synchronisation of the European taxonomic information systems into a joint e-infrastructure and the set up of a common user-interface disseminating the pan-European checklists and associated user-services results.

The organisation of national and regional focal point networks as projected not only assures the efficient access to local expertise, but is also important for the synergistic promotion of taxonomic standards throughout Europe, for instance to liaison with national governmental bodies on the implementation of European biodiversity legislations.
PESI will provide a robust infrastructure to support the nomenclatural needs of European users and stakeholders. PESI will specifically address the issues of: pooling resources, standardisation, sustainability, accessibility and international cooperation".

- The role of Species 2000 at the global/international level.

http://www.sp2000.org/: Species 2000 is a "federation" of database organisations working closely with users, taxonomists and sponsoring agencies.

"The goal of the Species 2000 project is to create a validated checklist of all the world's species (plants, animals, fungi and microbes). This is being achieved by bringing together an array of global species databases (GSDs) covering each of the major groups of organisms. Each database covers all known species in the group, using a consistent taxonomic system.
The participating databases are widely distributed throughout the world (…). The existing global species databases presently account for some 60% of the total known species, so substantial investment in new databases will be needed for full coverage of all taxa to be achieved.
The programme in partnership with the Integrated Taxonomic Information system (ITIS) of North America currently produces the Catalogue of Life. …/… In addition to the Species 2000 global project there are also four regional programmes concerned with the compilation of catalogues of all species in specified areas of the world, including Europe".

3. Organising the-GSD community: missions to cover and actions to take on

3.1. Foreseen Missions

Such a management structure would be put in charge of various missions that particularly will help in organising a distributed e-infrastructure (Life*Watch*) interoperable between the different levels of interest. We list here a series of them:

- Maintaining a GSD database: To collect metadata on databases in order to establish and maintain an over-view of who is doing what. To identify the gaps and anticipate orphan GSDs (by questionnaires, on-line registration, GSD community analysis…). Pertinent data will describe the content (title, subject, description, source, language(s),…), the intellectual property rights (owner, editor, contributors, rights…), the 'material' (date, type, format, identifier…), the risks of loosing data (custodian/expert retirement, stop of institutional funding/hosting, …).

  Recommendation:
  Part of such a metadatabase is already maintained by Species 2000 at the global level. It is currently up-dated via the 4D4Life EU-project (WP3). At the European level, PESI is also gathering different metadata (WP4). While different these data are complementary and should be maintained in a single database by the two partners (to be considered in a future new European project)

- Publicising and promoting the use of standards as recommended to be deployed in Europe by PESI (WP4-D4.1) following TDWG recommendations (Biodiversity Information Standards, a not-for-profit international group that develops standards and protocols for sharing biodiversity data, http://www.tdwg.org/about-tdwg/). It will allow easy access to and interoperability of all kind of data particularly to overcome

the problem of scaling from local to global actions and transdisciplinarity circulation of data outside of the single biodiversity field (economic, societal, …).

Recommendation:

- Minimize duplication of efforts by facilitating close discussions/participation into TDWG tasks and GBIF related actions
- Assist and help to adopt standards and make them usable for the GSDs (Task force)

- Alerting and defending GSDs about their rights and properties. Several databases have seen their data "stolen" by aggregator databases without mentioning their sources and not giving accurate credit. These small entities don't have the means to defend themselves alone while such an international recognised structure could do it for them.

  Recommendation:

  - Species 2000 and SMEDB have already worked conjointly successfully to solve such kind of problems. Weight of the European Community to help to solve them quickly should be looked for to secure this European expertise.

- Representing the Taxonomic-taxonomic database community in larger Initiatives-initiatives and organisations (Life*Watch*, CETAF, …).

  Recommendation:

  A clear role of SMEDB, PESI and Species 2000 needs to be sorted out at this level.

- Developing on a common agenda to trigger institutional involvement (at the directors level) to allow institutional experts to spend time on taxonomic indexing activities (e.g. via CETAF, EDIT BoD and at a global scale via the new OECD working group on collections, SciColl) and a plan for better databasing activities recognition;

  Recommendation:

  Develop a plan to publicise best practises how making a database citation and to encourage doing so.

- Developing a common plan with the institutions to cover the gaps of the missing GSDs and taking-on the proto-GSDs. Work on a common European policy program asking taxonomic institutes to take care about the long term updating of certain groups.

  Recommendation:

  This common European policy program should not target/distribute all taxonomic groups between the institutions but should only focus on the orphan ones/gaps: institutions have to only share the problems. Where there is no problem, competition is a guaranty for progress and quality and needs to be maintained.

- Providing/maintaining access, guarantee and support to an e-space to store physically orphan databases as well as proto-GSDs 'ready to take-on'.
  And providing access, guarantee and support to an e-space to archive active databases back-up.

- Developing and maintaining communication in the GSD community.

  Recommendations:

  - Develop and distribute rules and best practice documents to institutions developing GSDs.

- Particularly, organise and explain the LSIDs/GUIDs/UUIDs general rules, uses and best practises.
- Develop and maintain a high level of communication between GSDs and strengthen the social link in order each GSD remains aware of progresses and contents (e-bulletin, mailing alerts, …);
- Maintain the link with the EDIT cybertaxonomy platform and advertise on new development.

- …/…

3.2. GSD website

Establishing a network is relatively easy. To secure it on the long term is much more challenging. However the real challenge is not so much to provide a powerful infrastructure and even to maintain it in time, than the social/human dimension, so that GSD custodians feel themselves really part of the process. This social interaction can only be done by direct contact between people and not only through an e-structure that can only be the support of it.

However the objective to secure the long-term identity of the community needs also to be investigated through a specific **GSD Website**. At least, the following items need to be look for as they will help in securing an European taxonomic expertise network:

- to be simple to manage and do not consume too much time and human activity.

Recommendation.
The GSD metadatabase should look for a self-registered/maintained system (as in the TDCI project, http://rameau.snv.jussieu.fr/cgi-bin/TDCI/tdcisite.pl)

Recommendation. Looking for a common expertise management system (extending the EDIT Expert DB also used in PESI WP2 to GSDs)

- to be useful for each of the partners of the network.

Following the foreseen missions, several items should be investigated:

- delivering information:
  - platform of exchange for news and opportunities
  - storage place for copyright, important texts and advices
  - needs: gap analysis results / list orphan GSDs / proto-GSDs to take-on /
  - direct link with the CDM store to handle data and extend data to adjacent sectors

- delivering services:
  - storage place for tools ready to use related to the community (Cybertaxonomy platform);
  - GSD assessment tool with resulting guidelines for GSD upgrades (similar to the TDCI);
  - storage place for standards adopted, field definition (CDM), thesaurus ready-to-use (list of biogeographical areas, list of countries, list of world ecosystems, maps,...), …;
  - credit/recognition gain within the community
  - …

Recommendation.

Offer to the GSD custodians easy means to move their data into the CDM store. This migration will solve most of the sustainability problems.

- to be useful for the community:

- to allow better/greater impact
- to complete the expert community offer because of the integration effect (up-scaling effect). Therefore the infrastructure through the website should look after to offer:
  - one voice for the community
  - an anchor point to set-up collective/integrated community proposal
  - an anchor point to organise collective/integrated answers to calls

4. Updating & speeding-up the taxonomic indexing activities with better quality control

4.1. Data interoperability

Objective: better interoperability between expert GSDs and aggregator Databases

- help and endorse plans to agree ASAP on standards for taxonomic data at international level and adopt thesaurus (action item with GBIF, TDWG, …)
- make standards and thesaurus available on-line on the GSD web site. Probably an action item to look for with the new EDIT ISTC MoU about using 'of existing European and Global organism and classification services'
- make available tools (effective wrappers) for real dynamic links
- Set up a task group (with strong social skills) travelling around Europe, which can migrate in a short time an old database into modern standardised ones or educate a new custodian to take-on proto-GSD.

4.2. Data flow

Objectives:

- organising the data flow **with** the GSDs (not short-cutting them).
- speeding-up: one of the major time consuming task for the GSDs is to find/identify the data source and to implement it into its database. This process could be strongly speeded-up with adequate data, structured feedback and RSS procedures.
- organise the data collecting and the feedback process of new taxonomic and already structured information (e.g. from Zoological Record, IPNI, ZooBank,…) not only toward the major initiatives as currently planed (GBIF, Species 2000, PESI, …) but also down to expert GSDs. Going over GSDs head is counter-productive as it is where stand the taxonomic information expertise and where data quality and data validation take place.
- set-up automatic quality control procedures during data transfer processes between major data providers, expert databases (GSDs), aggregator databases, major international initiatives, (IPNI, ZooBank, GBIF ECAT, u-BIO, CoL, GNA, Zoological Record, ZooKeys, Zootaxa, …)…

Recommendation.

The most the data circulate between the different levels of the digital space, the best their quality could be because each initiative/partner of the network has some cleaning-up/alert procedures for non-compliant data. With an efficient

feedback between all network participants down to the GSDs, the data quality should be strongly increased.

- set-up a common versioning system for GSDs (set-up or adopt standards to solve at the same time questions as 'how to cite a database?', 'how numbering GSD versions?'…).
- set-up a mechanism of alert for new GSD version toward aggregator databases when no dynamic updating is in place
- …

4.3. Data quality control

- Data
Better organising the taxonomic knowledge production and collecting (name registration, taxonomic recognized journals (i.e. the TDCI, …) should be looked for.

- GSDs
Certification system for the GSDs: The peer review process as organized by Species 2000 before accepting contribution in the CoL should be extended to all databases.

- People (certification system)
At the human level, a "licence driver" system as certification has been recently proposed. This is a process to think about very carefully and has a very sensitive human dimension. For instance this proposal was publicly set up during the last e-biosphere 2009 meeting (while not agreed during the work group session) and was very badly received by the professionals themselves. The peer review system in science is the accepted system for people. Even if not perfect, adding a new system is a negative signal, as if taxonomists should not be real scientists!

The amatory community or "non-career taxonomists' people appear to be of crucial importance in many if not most taxonomic groups and most could feel offended by setting up such a system. A professional taxonomist is not 'as such' a guarantee of data quality delivering. The best validation of stored data is their use by their community (just a kind a peer review).

Many invertebrate groups for instance might be never validated/certified because of the absence of professional expert or validated by "non-true taxonomist" but just by a people in charge of the taxa in an institution.

<u>Recommendation</u>: Certification of taxonomists themselves is not recommended.
See for more discussion Boegh 's paper for pro-argumentation and all documentation inside at: http://www.wikigenes.org/e/art/e/32.html .

5. Governance and financial issues

5.1. Infrastructure governance

- needs to consult the GSDs themselves
- needs to be discussed with the two main actors in the field: Species 2000 and SMEBD.

- needs to place the infrastructure within the scene of the other major players, at least European ones and some global ones as cited through this report.

5.2. Financial maintenance of such an infrastructure

Several financing plans should be investigated but the answer is much probably a mix of all these possibilities:

- from the institution directly (but no control)
- via institution fees through CETAF that would fund this infrastructure as an operator for all major Institutions-Institutions
- by participation to Programmes-programmes as PESI funding SMEBD or 4D4Life funding GSDs and the Species 2000 association. The long-term sustainability of the Species 2000 model with continuous progresses clearly show that both GSDs and the community infrastructure need to be funded. Moreover this model that place GSDs as direct partners in European funded programmes is a greatly appreciated action providing support to them.
- the infrastructure should receive long-term support from major European and global initiatives (Life Watch, EDIT, CBoL, GBIF, EoL, IUCN, …) for which GSD data are their backbone on which they aggregate their own data. These do not have to see GSDs as only data providers for them, but as proper partner to which they should provide feedback for the data they access by other ways in the field of expertise of each GSD. This should be also the case for major aggregators as PESI or Species 2000.
- …/…

*[Should be discussed during the AGM of SMEDB
during the EDIT 2009 meeting in Faro and up-dated later]*

6. Identify the Costs (PESI M.32). [Draft 1.2]

To answer this question a list of all the items involved in maintaining a GSD is provided in order to identify the very specific costs of such a task. From this, we are elaborating a formula [12.XII.2009: currently in test] that will allow to give some figures on how much costs a GSD according to various criteria, as taking into account the taxonomic level covered by the GSD, the country where the GSD is maintained, and various technical issues used by the GSD.

Two main actions have been tracked. The first has been to identify the costs in the case of GSD is maintained with its own schema ("Maintaining an expert GSD/RSD") and the second if the GSD needs to migrate into some new schema to secure its maintenance as for instance looking for better interoperability, new technologies issues, lost of the expert maintaining it, …, ("Migration to a new system"). Also, technical and data components need to the respectively analysed.

6.1. Technical component

This part focuses on the technical part of maintaining a GSD up-to-date. The Cybertaxonomy Platform of EDIT should serve most of the following items at term. Until this Platform for Cybertaxonomy becomes ready to use, one still needs to enumerate all requirements.

> <u>Recommendation:</u>
> One principle is important to be maintained: diversity of databases is not a problem but a source of progress and it needs to be encouraged; in return higher and quicker development of database interoperability (via a distributed network) in a global sharing data space is an urgent necessity.

Two cases need to be taken into account: a) in order to maintain an up-to-date and interoperable already existing GSD that uses its own system ; b) in order to make the GSD evolving on a new and more efficient system as proposed by EDIT. In both cases it allows them to evolve and follows setting up of new standards.

> <u>Recommendation:</u>
> The following tasks could be devoted to a European taxonomic computer task-force (which could be managed by SMEBD). The missions of this group of bioinformaticians would be to assist GSDs with the implementation of new standards, various updates, wrappers or additional thesaurus, to help to move to schemas and tools developed for the EDIT Cyberplatform or to assist people starting to deal with proto-GSDs, …).

6.1.1. Maintaining An expert GSD/RSD

- ***Regular back-up copies.*** This can be an automated task. (+ see § Giving access, guarantee and support an e-space to archive back-up copies)
  (possible role for the Infrastructure component as proposed in 5.1 report)

- ***Material***
  - *Back up media* [Choice/price/number of media]
  - *New server (every 10 years)*
  [Choice, purchase or leasing, amortization, maintenance cost].

- *Hosting server* [Database hosted by the Information Technology Department of an Institute or use of a shared web hosting service (webhotel). Many parameters must be evaluated: Random-access memory, Central processing unit, disk space, web traffic, bandwidth, IP address, operating system… Information to be linked to a database of databases]

  (possible role for the Infrastructure component as proposed in 5.1 report)

- *Number of workstation*s [purchase, amortization / Minimum is one workstation per GSD].

- ***Technology watch/resources***

  - *Every 5 years, evolution of the back up media (hard disk, memory stick, DVD, CD, new media?)*
    - Last back up versions must be transferred on new media.
    - Guaranty and secure copy on a more suitable and secure media if it becomes available
  - *Every ten years, evolution of the hosting hardware (server)* [Keep informed about new available material for hosting the database]
  - *Be informed if a new version of particular software is available* [tools already exist on the web but a possible SMEBD role for specialized softwares and what is going on in databasing/bioinformatics]

(most of these issues with possible roles for the infrastructure component as proposed in 5.1 report)

- ***Staff and human resources necessary for:***

  - *Make transfers and back up on new media*
  - *Corrective maintenance: dysfunctions, errors…*
  - *Person-day cost* (varies with countries, institution's staff or people with a contract).
  - *Implementing general tools to be produced for any GSD often absent in expert GSDs/RSD*
    - Implement Versioning tool
    - Implement data-mining, RSS, feedbacks and wrapper modules

      [Automated tasks of data-mining and RSS links shall be implemented in order to speed up taxonomic indexing. The evaluation of extracted data and the final validation is devoted to the expert.

      Provide and organise feedback to the Expert GSDs from the major aggregator databases GBIF, CoL, EoL, GNA, particularly those which directly collecting data independently (Lifedesk in EoL for instance) to assure one up-to-date lecture everywhere on the web.

      Catalogue of Life and the GBIF Electronic Catalogue of Names of Known Organisms ECAT to fill gaps by cross-checking both names indexes (i.e. Adapt ITIS new powerful on-line Taxonomic Workbench (OTWB) tool to the cybertaxonomy platform and provide).]
    - Implement automatic data conformity/logic control procedures
    - Implement LSIDs, GUIDs, or UUIDs usage and take benefit of these technologies
    - Implement new standards/thesaurus in the GSD/RSD schemas
    - Implement data quality control items (punctual & workflow)

6.1.2. Migration to a new system

A plan of maintenance could include evolution of old GSD migrating on new schemas available as those provided by the cybertaxonomy platform. They might continue to go independently with assuring interoperability or "disappear/sunk" into major e-infrastructure as in SMEBD, PESI or in Species 2000 organisations. In any case this migration will include costs in terms of humans, time and financial resources that need to be take into account.

- ***Material***

- ***Cost of the development, test and production servers***
  Staff and human resources to:
  - *write out a new requirements/specifications document according to the users needs.*
  - *develop taxonomic editor and explorer for various Operating Systems [make news of the EDIT tools]*
  - *develop wrappers in order to ensure interoperability with global initiatives (GBIF, CoL,..)*
  - *elaborate a series of tests and run the tests*
  - *add any specific fields in the database proper to the taxonomic group (any etho-ecological specific data of interest according to the taxa)*
  - *adopt new standards*
  - *add/modify of a thesaurus* [Thesaurus are a good way of surrounding data edition and avoiding any multiple occurrence of similar data. Adopted as standards they are a mean of greater interoperability.]
  - *complete technical documents with comments inside programs (editor and explorer)* [Remarks concerning the program should be made by the original developer(s) in order to make the resumption of the work by someone else easier.
  - *provide users guides/documents* [These documents compile instructions for use dedicated to people in charge of maintenance of the programs and the database itself.]
  - *test/learn new softwares if a migration of the project to a new structure is considered.*

- ***Cost of the developing softwares***
  - Free when developed on PostgreSQL, MySQL or Firebird; License-based like Oracle, Sybase, DB2 or Microsoft SQL Server (SFS program for example). The BRAHMS software has been developed using the license-based 'Visual FoxPro' programming language. In the case of a license, cost for upgrading versions must be anticipated. [recommendation: to use open access tools]
  - In case of an upgrade, tests must be performed in order to avoid any inconsistency.

6.2. Data component

Concerns the database content.
- It is crucial for Europe to accelerate taxonomic data indexing, keeping in mind that the validation of captured data must follow well-established routines of quality control.
- Focus should not be given only to what has been already published/available but a pro-active policy in producing new primary data/knowledge need to be set up. A clear link should be established with any digitisation plan of specimen collections because this is where the

maximum biodiversity data is currently stored (and already partly organised) and therefore collectable at the best benefits/costs ratio.

6.2.1. Maintaining An expert GSD/RSD

Tasks concerned:

- **Scientific watch** (possible SMEBD role to rationalise globally this specific task)

  At the expert level, data validation is a major point, which can be only partly handled by automated procedures of logic validation (completeness, nomenclature code compliant tools, …). Indeed, precise data quality control needs a return to publication(s) and a more complete checking of the taxonomic data by the expert. Beside the current task of the GSD custodian, feedback of users is most often the best alert of data quality problems. In link with PESI (particularly using the focal points) automatisation of these feedbacks need to be organised.

  Maintenance of existing expert GSD requires scientific watch of:
  - Going through literature and electronic publications [Depending on the Institute's policy, some libraries have electronic access to taxonomic editors websites]
  - Managing automated feedback from the web through the RSS
  - Organising its data-mining and its network of data collecting
  - Answering feedback (from users or by cross-checking)

- **Addition/modification of a complete taxonomic information**

  This task depends on the structure of each GSD. For instance, updating a simple names checklist is an easy task without any complex treatment of the data, whereas updating a relational nomenclatural database may imply multiple modifications (a single change in the nomenclatural status of a taxa may imply many other adjustments in the database).

  WP4 of PESI is establishing rules for the Standardisation of Data Exchange. These standards determine which controls and validation steps are needed in order to enter/update properly the data in the database. Any tools following these standards that will prepare/provide these data in a structured way would help to speed-up the data entry process. [Standards established in PESI Work Package 4 will guarantee the reliability of the data (it may imply the capture of published nomenclatural acts, author(s) name, associated biological data…).]

  This concerns:
  - Updating (change of a status, modification from valid to synonym for instance)
  - Adding a taxa, a name, a publication, an usage or any other data of interest [recommendation: to use BHL tools]

6.2.2. Migration to a new system

Should be though in term of an evolutionary maintenance: evolution of the database depending on publication of new standards and according to users wishes. PESI can play a crucial role in guiding the migration to the cybertaxonomy family tools by providing ready to use tools.

In any cases, any change of a GSD to a new expert structure will require.

- *Upstream: redefining needs and model*. [Changes in the structure of the base (new structure's design), adding fields, using a thesaurus]
- *Downstream: Analysis, setting of the new version of the database (tables, relations…)*
- *Migration towards the Berlin CDM*
- *Adaptation and Implement standards (interoperability)*
- *Include other biological information (distribution, associated organisms, biotopes, genomic information, sounds, pictures, links towards other websites like Genbank, common names…)*
- *Implement new language(s)*
- This item should be addressed at the beginning of the migration process in line with users needs.
- *Search engine specification (simple, advanced…)*
- *Document the new database* [It is fundamental to provide a general presentation of the database and details on the biological data and taxa displayed]

6.3. A formula to use to evaluate a GSD cost.

To be delivered in M32.
…/…

6.4. Financial plan to ensure long-term maintenance of European GSD network (M32)

To be delivered in M32.
…/…

Configuration History			
**Version No.**	**Date**	**Changes made**	**Author**
1.1	03 July 2009	First draft for circulation to YdJ and MC	Paris PESI staff
1.2	24 November 2009	Revision of parts 2-5	YdJ
1.3	13 December 2009	Current version (for internal PESI circulating)	Paris PESI staff
