# Supplementary material for: PESI - a taxonomic backbone for Europe
Source: Biodivers Data J. 2015 Sep 28;(3):e5848. doi: 10.3897/BDJ.3.e5848 (PMC4609752; doi:10.3897/BDJ.3.e5848)
Supplement: Supplementary material 23 — Sustainability of European GSDs: Quantify financial and other resources to ensure long-term maintenance of European GSDs database systems [file biodiversity_data_journal-3-e5848-s023.pdf]

Sustainability of European GSDs: Quantify financial and other resources to ensure long-term maintenance of European GSDs database systems

Pascale Bezard-Falgas & Thierry Bourgoin

This report constitutes deliverable D5.4 of Work Package 5 (WP5) of the Pan-European Species directories Infrastructure project (PESI). It addresses: 1) possible issues for GSD sustainability and 2) evaluation of the cost of maintaining on the long-term a Global or a Regional Systematic Databases (GSDs/RSDs) for a particular taxonomic sector of the classification.

Together with some new ideas about feedback mechanisms between GSDs and major Taxonomic database initiatives, this report provides an easy-to-use tool allowing evaluating the financial costs of maintenance for any individual database through a formula to fill in with a few parameters.

It does not concern the cost of provisioning and validating the taxonomic data that concerns the taxonomic expertise. Several ideas and issues for a better organisation and management of the GSD community have already been addressed in a previous report (D5.1) for which this D5.4 report should be considered also as a continuation of the previous task.

This D5.4 task is delivered with two files:

- the present text document that addresses in its first part new ideas to help GSD sustainability and its second part provides mainly the user document to explain, manage and use the formula: *PESI D5.4 GSDmaintenanceCost v4.pdf*

- an Excel file that allows to calculate this cost: *PESI D5.4 GSDmaintenanceCost v4.xls*

Contents

1 GSD ACTIVITIES AND SUSTAINABILITY 3

1.1 DEFINITIONS AND KEY ITEMS 3

1.2 WHY SUSTAINABILITY 3

1.3 GSD ACTIVITY SUSTAINABILITY 3

1.3.1 GSDs Activities 4

1.3.2 GSD Activity sustainability 6

1.4 SUCCESSION PLANNING 9

1.5 CONCLUSION 11

2 GSD SUSTAINABILITY COST 12

2.1 INTRODUCTION 12

2.2 OVERVIEW 13

2.3 THE SERVER 14

2.3.1 Amortization of the purchase price 14

2.3.2 Cost of maintenance 14

2.3.3 Coefficient of use 15

2.3.4 Annual cost of the server 15

2.3.5 Annual cost of the server if rented 16

2.3.6 Summary 16

2.4 CUSTODIAN'S HARDWARE 16

2.4.1 Amortization of the purchase price 16

2.4.2 Cost of maintenance 17

2.4.3 Coefficient of use 17

2.4.4 Annual cost of the custodian's hardware 17

2.5 THE OPERATING SYSTEM OF THE SERVER AND THE CUSTODIAN'S HARDWARE 18

2.6 THE DATABASE MANAGER 19

2.7 ANTIVIRUS SOFTWARE 20

2.8 THE UPDATE OF THE DATA, THE DOCUMENTATION 20

2.8.1 Description of the database complexity 20

2.8.2 Data Entry 21

2.8.3 The tools for corrective maintenance 23

2.8.4 Management of the documentation 24

2.8.5 Annual cost of the data update, and the documentation update 24

2.9 MECHANISM OF BACKUP AND ASSOCIATED SUPPORT 24

2.9.1 Coefficient of use 25

2.9.2 Backup hardware 25

2.9.3 Frequency of backups 26

2.9.4 Annual cost of GSD's backup 26

2.10 GENERAL FORMULA 26

2.11 APPENDICES 27

2.11.1 List of the customizable variables 27

2.11.2 Summary of the calculation's formulae 27

2.12 INSTRUCTIONS FOR USE OF THE EXCEL FORM 30

2.12.1 Spreadsheet « Parameters Entry » 31

2.12.2 Spreadsheet «Calculation results » 33

2.12.3 Spreadsheet « Calculation details » 34

2.12.4 Spreadsheet «Choice List Information» 36

1 GSD ACTIVITIES AND SUSTAINABILITY

1.1 Definitions and key items

- Global Systematic Databases (GSD) or Regional Systematic Databases (RSD) are systematic expert databases **that maintain a structured access to provide information for a sector of specific taxonomic knowledge (fig. 1).**

- They represent the first step in collecting data in a structured way and their roles are to extract, gather and to validate nomenclatural and taxonomic information and their associated primary biological data into a comprehensive classification system.

- The human expertise to manipulate these units of information is crucial (species concept based) for the quality of the information that needs to be validated before being delivered into the digital space for other usages and decision-makings. In other terms:

GSDs maintain and validate/certify the link between
the primary taxonomic knowledge production (primary data)
and the digital taxonomic knowledge usage.

1.2 Why Sustainability

GSD is a scientific production. As any of them it needs 1) to be funded, 2) to be based on a real scientific expertise that produces it, 3) to be published for recognition and to be spread and used demonstrating its quality. However moreover these tasks, GSDs represent also **evolving resources and services** that follow progress of science and ideas. Therefore, in addition to other scientific productions, GSDs need also sustainability.

GSD sustainability has already been addressed (D.5.1) in terms of 1) data credit, data traceability and the multi-versioning problem and 2) infrastructure components to organise de the European GSD community in link with major European actors as SMEBD and Species 2000. In this document we address more specifically two others items: the funding sustainability and the evaluation of cost.

1.3 GSD Activity sustainability

Any plan to assure GSD funding for sustainability will have to ensure their activities. This is the first point we report below in order to evaluate their general cost. In a second step, we suggest solutions to lower the difficulties the GSDs are facing in these activities - even if before all "money matters!" -. In a third step we provide a succession plan and conclusion.

1.3.1 GSDs Activities

GSDs activities deal essentially with data plus some networking and informatics activities as summarised in the following figure (fig.1) that ensure the providing of a taxonomic data (primary data) to build information and knowledge to external users. According to the number of taxa covered by the GSD these activities are more or less time consuming.

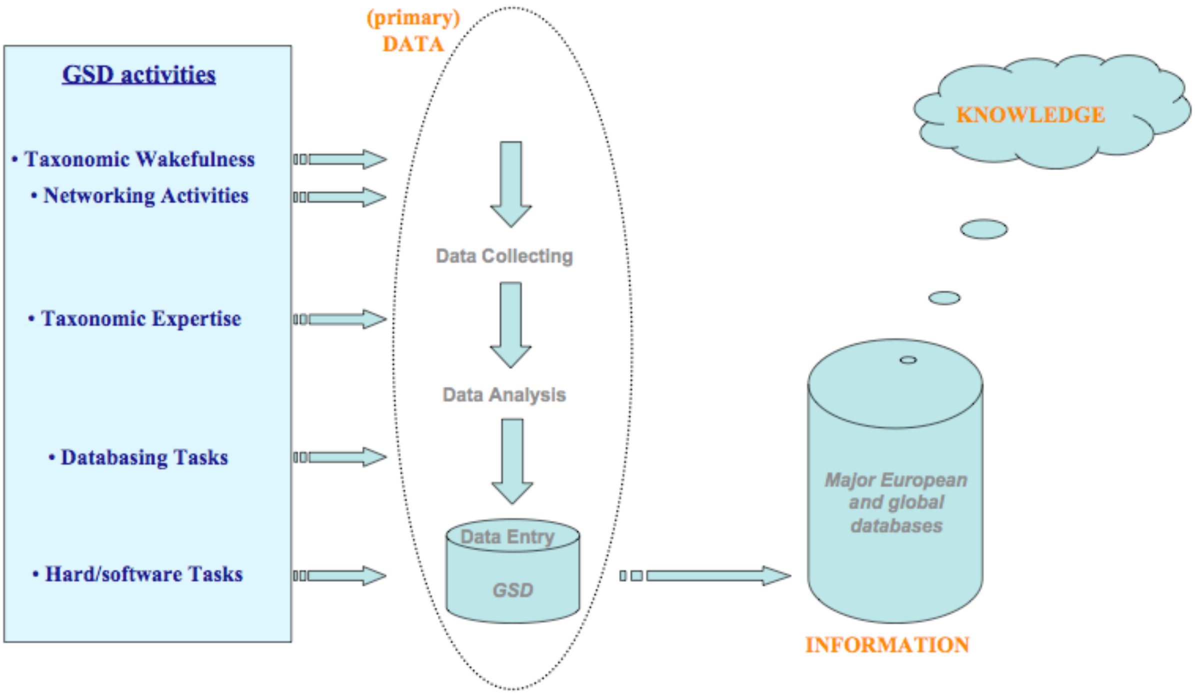

Fig. 1. GSD activities with their corresponding results in the data domain and their links to information and knowledge spaces

1.3.1.1 Taxonomic wakefulness: Data collecting

Collecting data is one of the most consuming time for a GSD custodian. It deals with:

- looking for new taxonomic (and distribution) publications in libraries,
- data-mining through the web,
- comparing data to those already databased

1.3.1.2 Taxonomic expertise: Data analysis

These involve the precise taxonomic expertise of the GSD custodian and concern:

- Data triage/selection,

- Data interpretation,
- Data correction and validation,

1.3.1.3 Databasing tasks: Data entry

If part of this task could be now automated thanks to parsing capabilities of taxonomic data into unit of information by very recent software devices (see EDIT Desktop Taxonomic Editor : http://wp5.e-taxonomy.eu/taxeditor/) all Eu-GSD still maintain a classical single field entry editor, even most often without a web edition. The support that these new tools could provide to GSDs still remain limited but it is foreseen that this will increase.

1.3.1.4 Networking issues

Most GSDs activate and maintain :

- their own expert network in order to be alerted and to get access to newly published publications (publication pdfs/scans directly sent to the GSD custodians).

This networking activity is also used to:

- Alert to authors on taxonomic problems in the field in which they have published. For several taxa the role of citizen scientists (amateurs) remains crucial regarding the number of species that remain to be described. However all these citizen scientists do not always have the full skills necessary to publish accurately their discoveries.

- Maintain access and link with major European and Global databasing taxonomic initiatives (GBIF, Species 2000 and the Catalogue of Life, Fauna Europaea, European Register of Marine Species, Euro+Med PlantBase, … and their ongoing programmes and projects like PESI, 4D4life, i4Life, …).

1.3.1.5 Hard- and software and other annexe activities

Most often GSD custodians ask to spend time in developing the database content more than just the taxonomic information with appropriately linking other associated biological data, adding photos, sounds, etc… to the data they manage. Accordingly and while this is not the main activity for GSD custodians, most of them have (self-) developed some basic skills in hardware, software, web access, archiving and back-ups, and database management in general.

GSD custodians moreover need to develop a clear Intellectual Property Rights, copyright and ownership for their data. They also need to be aware of standards and good practices in these domains.

1.3.2 GSD Activity sustainability

All these activities are more or less time consuming depending the size of the database. It is clear that all GSDs have started with large time consuming activities, particularly with data mining for old taxonomic and linked biological data information. This first phase of a GSD life is difficultly quantifiable in term of costs but it is obviously high.

The second phase is maintaining up-to-date the GSD. It is also depending of the size of the database but with a good self-organisation the custodian can manage a database of several thousands of taxa for a relatively low cost. We provide in part II of this document a tool that allows each GSD to get a first estimate of their maintenance costs.
GSD sustainability has to be addressed through three main issues (fig.2):

- the community item
- the funding item
- the data item

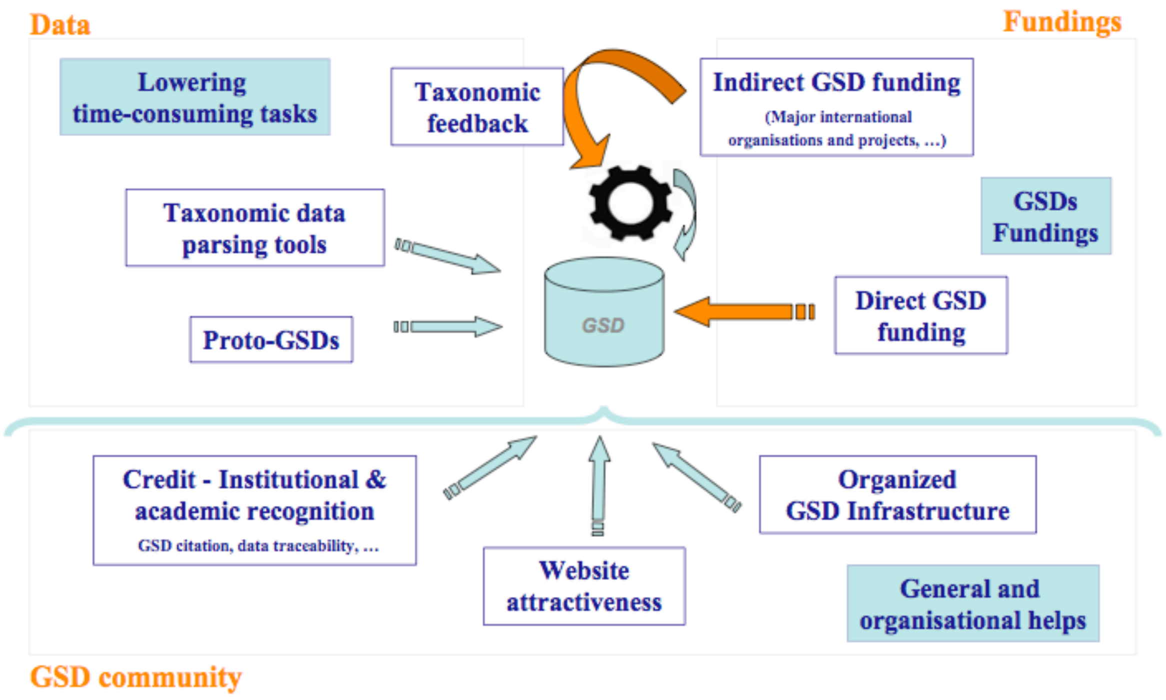

Fig. 2. Three main sustainability levers to action for GSD:
1- The ground component for organising and recognising the GSD community,
2- the direct or indirect mechanisms for funding and
3- a better data management for lowering time-consuming GSD tasks.

1.3.2.1 To develop and to strengthen the GSD community and its attractiveness

These aspects have been mainly addressed in D.5.1. and concern the GSD producers, their possible organisation through a recognised infrastructure, their website database attractiveness, the credit given to the GSD via the citation mechanisms and institutional (museums mainly, CETAF, …) and organisational (GBIS, Species 2000, CoL, EoL, major European databases, and many of these international initiatives around Name Indexing activities, …) recognition.

Obviously a better recognition of the GSD community is a lever to action to facilitate its members to attract funding. As already mentioned (D.5.1.) their is a clear role here for SMEDB and Species 2000 in organising and strengthening the GSD community in Europe and even globally.

1.3.2.2 To attract funding

Because 'time is money': **MONEY MATTERS!** Two ways, direct (GSDs are directly funded) and indirect mechanisms (GSDs are not directly funded but can benefit from funding given to third parties), are identified to fund GSDs.

- **Direct funding** to GSDs is of course the best situation for the custodian: he is funded to maintain its GSD and he feels at the same time recognised for his work. Several GSDs have started this way being funded as part of a research project. In general it is however the hard- and software components that are treated in priority and the cost of the taxonomic expertise not fully taken into account. Later, punctual financial resources are sometimes offered to GSDs through research projects but one cannot speak about sustainability in such cases.

It is however important to mention the Species 2000 politics that through its distributed organisation has chosen to systematically associate all European GSDs with its European Commission funded projects. The first one in 2003-2006, through FP5, was Species 2000 Europa and the second one in 2009-2011, through FP7, is 4D4life (Distributed Dynamic Diversity Databases for Life). With these two programmes, more than 20 European GSDs have received direct funding and sustainability support for better completeness and better interoperability of their data.

At another level, the European project PESI has adopted is similar mechanism by integrating three major European databases in his project: FaEu (Fauna Europaea) ERMS (European Register of Marine Species), Euro+Med PlantBase.

In the current situation, it however remains very difficult to foresee any other direct funding mechanisms for European GSD sustainability.

- **Indirect GSD funding** mechanisms have not been addressed until now. We thought however that it could become a major lever to support GSD sustainability as explained below.

1.3.2.3 To lower time-consuming GSD tasks

Because European GSD custodians lack of recognition, they have great difficulties to attract funding to pay for their activities as they have been listed in this document. More visible, major organisations as already mentioned have however less difficulties to be funded to update their data while they have greater difficulties to find taxonomic expertise for their validation.

Until now and very frustratingly for them, the data flow has been unidirectional: from GSD to global initiatives. It is suggested here to organise a structured feedback of collected data from these global organisations to GSDs, for data validation. Indeed, this large time-consuming task will be accordingly lowered for GSDs: 1. will no more spent huge amount of time to track newly or old published data, 2. they will receive data in a parsed and structured flow, ready to integrate their GSDs. Once integrated and examined by the taxonomic experts, the data will return back, validated, to the global organisation. In other terms if the collect cannot be funded at the GSD level it can be more easily done at a more global/European scale.

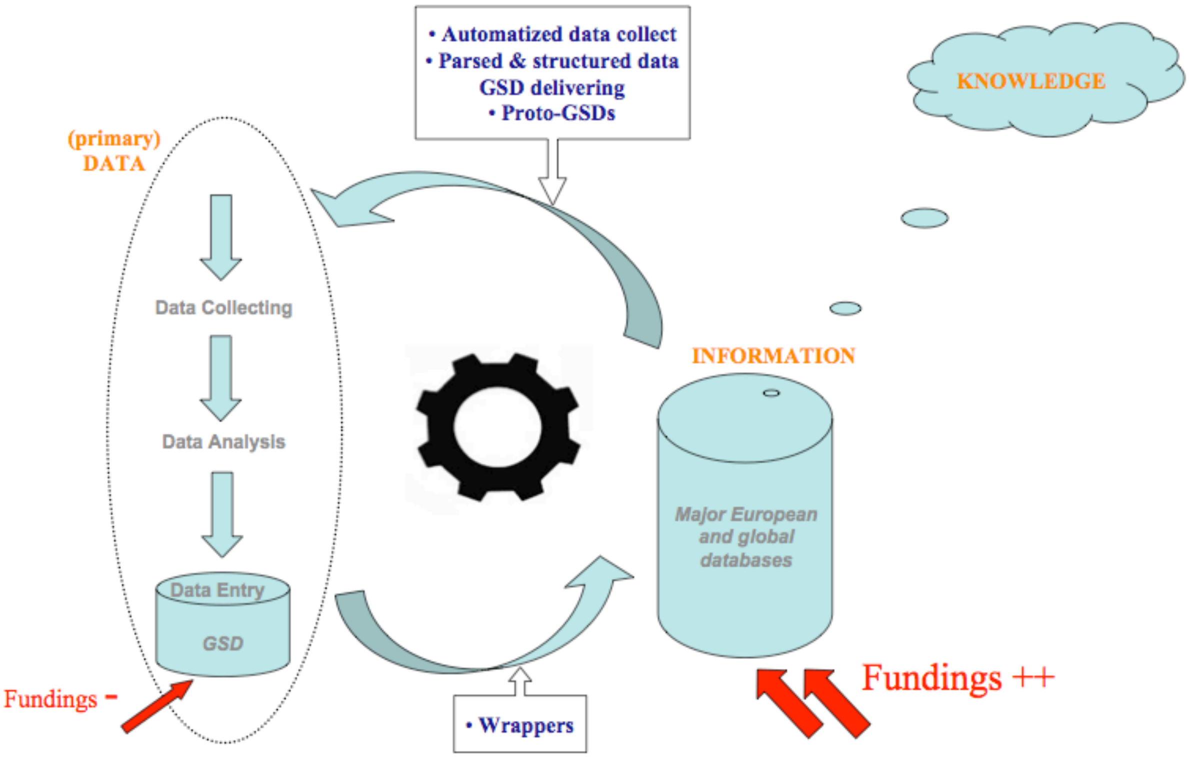

Fig. 3. A feedback mechanism between GSDs and Major European or global databases will allow findings to irrigate both parties and will strengthen data completeness, data quality, taxonomic databases visibility and will reinforce their sustainability.

Such an indirect GSD funding mechanism is seen as a **win-win situation**: global organisations gain data quality and visibility, interactions with GSD are supported and valorised, GSDs gain easier management of the new data (collect and ready-to-entry data) and can concentrate on their real task and expertise (data analysis and validation) reaching also better completeness of their GSD.

Proto-GSDs projects (see D.5.1.) are based on a similar idea: major organisation organise the first collect of the data for a given taxon where gaps are observed. These data are structured for ready-to-validate database. It is delivered in a second step to a specialized taxonomic community for validation and eventually sustainability of this new GSD.

1.4 Succession planning

A taxonomic database may have been developed by one scientist or several who work for a university, museum or other science organisation; and may also involve self-employed and retired scientists, and graduate students. It may have been created to satisfy the needs of a funding agency for research results to be made publicly available, and its creators may or may not wish to continue to develop it. The scientist(s) may be very successful in winning regular funding to develop the database over some years, and perhaps a decade or more. However, ultimately, somebody else will need to take over its leadership. Having several collaborators involved of different ages will facilitate a smooth transition to new leadership.

Should the resource be recognised by other scientists and organisations as being sufficiently unique, large, and authoritative, the following options are available for its succession:

1. One or more organizations agree to host the database from within their own budget;
2. One or more institutes commit to the long-term maintenance of the database combined with institutional applications for external funding;
3. One or more scientists agree to take responsibility for its content, quality and development on their own time;
4. Other scientists continue to find funding on a project by project basis;
5. A sponsor provides annual funding, or an endowment that provides annual funding;
6. Users pay for the operational costs, which may include hardware, informatics, and personnel time;
7. The resource raises funds through donations, advertising, publications, CD sales, or other products;
8. Funds are raised through special services built on the resources (e.g. data analysis, reports);
9. More content and services are available to users who pay a subscription fee;
10. Mixture of above.

It may be useful to distinguish the resource into four components, namely (1) overall management, (2) the experts who contribute and validate its content, (3) the software, including the web interface and services, and (4) the hardware, including archiving, back-ups, 24-7 online access, and response time. These components may be managed by different

people and have different funding streams. If the resource is a stand-alone facility, with its own hardware, software, IT support and scientific staff, it will have a significant budget, probably in hundreds of thousands of euro per year. However, most taxonomic databases are modest in their demands for resources and may be more cost-effectively maintained if they are integrated into larger computer systems. If this is planned at an early stage, and the database follows common standards, the difficulties in extracting data due to idiosyncratic formats will be minimised.

The funding sources may be grouped into host organisations, funding agencies, individual scientists, and users. Ideally, it is desirable to have a portfolio of funds from several sources in case some are unavailable at some time. For example, the European Register of Marine Species was initiated by a €385,000 research project in 1997 (Costello 2000), moved to a new host institution in 2000 which had €250,000 of projects that built upon it, received six small grants from projects funded by EU and USA sources totalling €110,000, and then €300,000 and €400,000 projects in 2004 and 2008 respectively to develop the content, editorial board, and infrastructure further. The incremental extensions of the content resulted in a new goal to produce a World Register of Marine Species, which doubled the size of its editorial board while making it a more prestigious and valuable resource (Appeltans et al. 2010). Between projects, the host institution can maintain the online services and address user needs, while the editors can keep it updated with modest time input.

A challenge in establishing biodiversity databases is that they are often little used until they reach some critical size where they become the first place users will look for particular information and data. For example, over the past decade GBIF and OBIS have published tens of millions of distribution records of hundreds of thousands of species. However, the unique scientific insights possible from such massive global databases are only beginning to emerge. Resource development should plan on its use for scientific research from the onset so demonstrations of the data use can emerge as soon as possible.

A larger and more widely used resource will be easier to obtain funding because (a) it will be more prestigious and useful for an organisation to sponsor or host, and attractive to scientists to be its editors or authors, and (b) it will have more potential funding sources, perhaps globally, including users and project funding. Frequent engagement with users is desirable to ensure their needs can be planned for. This will involve email correspondence and interactions at scientific meetings. Special workshops and web based tools and services can also aid user engagement. The resource will need to provide a service that is unique in terms of quality and/or comprehensiveness than alternatives. For example, GenBank is now an integral part of the world science e-infrastructure with host institutions in three different countries, and a large global network of scientists who use it daily in their research. This has been aided by the large resources for human, animal, plant and microbial genetic sciences, and the fact that genetic data is more amenable to data management than text based information. Other examples of well-established databases focused on biodiversity content shows they are all significant in size and have a large international user community (Table 1). Other species biodiversity databases should consider how they can achieve such critical-mass of users and consequent interest from national funding sources.

1.5 Conclusion

GSD sustainability needs to be improved and addressed at 3 levels:
- Structuring of the GSD community to improve GSD credit, citation and recognition,
- Lowering time-consuming tasks by accelerating and facilitating data management through new computational tools (data mining, data parsing, … proto-GSDs, …),
- Organise feedback mechanisms between GSDs and major global/European databases (GBIF, Species 2000, EOL, ERMS, FaEu, Euro PlantBase + Med, …) in order to reverberate the funding effects at all levels of the taxonomic knowledge chain, from the producers and experts to the users.

The ideal approach is for (a) taxonomic databases to become integrated into larger databases with a consequently larger user community and pool of funding opportunities, (b) be owned by a science organisation or fully committed institute with a suitable mandate that is governed by the scientists who have been contributors. In this regard, bigger is better because the resource will have more content, more potential uses of its content, more users, more contributors, be more prestigious to contribute to, and have more funding options. While developing in this way, it is important to maintain the collegiality and team spirit that is often key to the success of such initiatives. This may be achieved through good governance, including transparency of management, democracy and meritocracy, and proactive communication with contributors. Of course other models can also work and the ultimate measure of success is their longevity. The organisational model should be designed to ensure sufficient resources for its development, in terms of both money and people's time.

2 GSD SUSTAINABILITY COST

2.1 Introduction

- In this document we refer to the normative terminology of 'maintenance' as approved in June, 2001: *all the technical, administrative and management actions during the life cycle of a good, intended to maintain it or to restore it in a state in which he can carry out the required function* (NF IN 13306: in June, 2001)

The purpose of the task is to provide a mean to evaluate the cost of the preservation and the maintenance of existing databases. This document explains the use of the tool that has been built.

This task being an experiment, the different requested parameters will be entered in an Excel file. Each user must enter data carefully, with attention and precision, because Excel is far from being software allowing the checking of data entry coherence in real-time. The default value will be shown only at the first opening of this file but will not necessarily been shown again during the following uses of this Excel file. The last used figures will be shown again during the following uses.

We therefore recommend you to save a copy of the excel file before beginning to play with it.

- This formula attempts to define the costs of maintenance of the existing GSDs. It absolutely not delivers the cost of the creation of a new GSD. Accordingly it mainly concerns up-dating tasks such as:

- Importing new data sets, new associated biological data, new checklists (gap filling)Ö
- Updating existing checklists
- Managing synonymy (keeping abreast of changes in taxonomy)
- Managing classification (keeping abreast of changes in taxonomy)
- Corresponding with data providers and users.
- Quality checking

- New in Version 2 and 3:
Comments received from several GSD custodians have led to modify two points in the formula: costs of data capture and corrective maintenance.

2.2 Overview

Types of GSDs are very diverse: from simple lists of taxa and names (checklist) to better-structured database and/or with more associated biological data.

With the help of the questionnaire sent to the custodians in November 2009, we observed different types of GSDs. Some of them were Excel files (all databases can be converted in a simple table), others were relational databases. Other types of lists, such as simple Word file lists were also maintained. The formula can be considered by any of them.

Before any general standardization of taxonomic databases, we shall try to determine the essential points to be listed and to be estimated for this calculation of the costs. Everyone will adjust the following list according to his own data.

The cost of maintenance of a database depends on several factors that are linked directly or indirectly to the use of the base:

We shall consider in our calculation only the preservation of what exists in every GSD:

- The **server** that stores the database
- The **custodian's hardware** that manages the GSD
- The **operating system** of the **server** and of the **custodian's hardware**
- The **database manager** who stores and organizes the data on the server
- The **antivirus** that protects the system
- The **update** of the data
- The management of the **documentation**
- The **data entry tools** which allow to extends the database
- The **tools of corrective maintenance** which allow to correct the database
- The **tools of maintenance** which allow to develop the database
- The **search and consultation tools** which allow to consult the database
- The **mechanisms of backup** and the **associated equipment**

We did not take into account the cost of the internet access for non-institutional GSD (internet **subscription** for private people). This will have to be included to the calculated cost by these GSD.

Legend of colours

**SER, SER_amo**	Results of a calculation or a predetermined coefficient	(Principle of
**SER_ach**	Parameter to be entered by the user before the calculation	(the reserved
**800 Ä**	Default value being subject to be adjusted before the calculation	(colours

We shall have several parameters to be entered to obtain the global amount of this cost of maintenance for a given GSD at a given moment. We shall group these parameters together by category to facilitate the entry of the parameters by the custodian.

2.3 The Server

The server is the computer that hosts the database. Only the hardware issue is concerned in this chapter. Its annual cost of maintenance, noted **SER**, is calculated with the elements described below.

Paragraphs 3.1, 3.2, 3.3 and 3.4 describe the calculation of the cost if the server has been purchased ; and the item 3.5 if the server is rented.

2.3.1 Amortization of the purchase price

The amortization of the computer hardware is generally made over 3 years (Source: tax authorities). The hardware will be amortized using the digressive depreciation: 40 % first year and 30 % on the next two years.
The rule of calculation of this element is as follows:

SER_age : age of the server in **months**
SER_ach : cost price excl. Tax of the server in **euros**
800 Ä default value of **SER_ach** which can be adjusted before the calculation

We ask for the age of the server in months, because it returns a more precise calculation. Calculating the amortization, we round off **SER_age** to the following year. For example, if the user of the formula enters **SER_age** = 6 months, then in our calculations, **SER_age** = 12 months

If **SER_age** > 36 months
    SER_amo = 0
Otherwise
    If **SER_age** <= 12 months
    SER_amo = **SER_ach** *** 40%**
        Otherwise
        SER_amo = **SER_ach** *** 30%**

When the cost price is not known, the default value of **800 Ä** will thus be used and indicated by the user of the "formula" during the parameters entry.

2.3.2 Cost of maintenance

The cost of maintenance of a computer hardware increases with years: 1 -A too low cost of maintenance for a small price of purchase 2 -A too important cost of maintenance for a high price of purchase

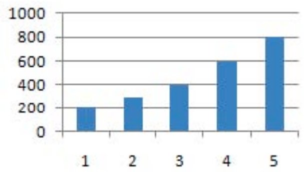

The formula is established so as to avoid:

1 - A too low cost of maintenance for a small price of purchase
2 - A too important cost of maintenance for a high price of purchase

Chart of the cost of the maintenance when compared to the cost price:

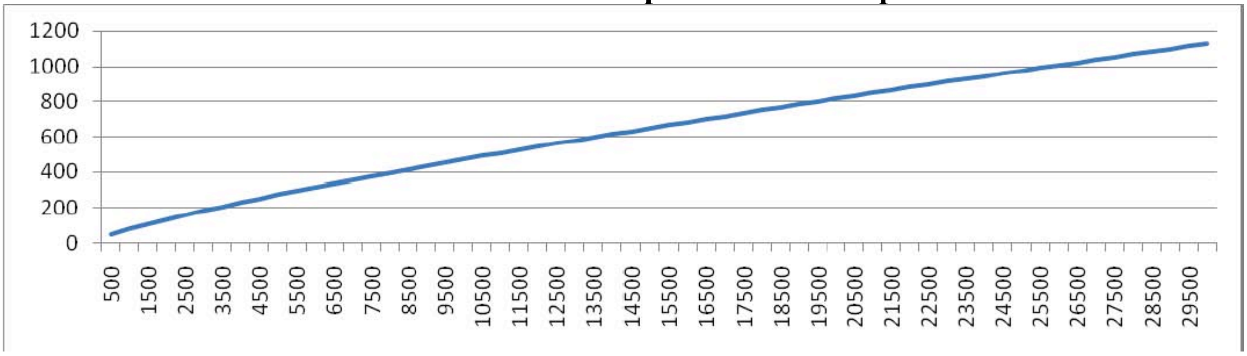

On the x-axis the cost of the material and the cost of maintenance on the y-axis.

Chart of the rate of the cost of the maintenance:

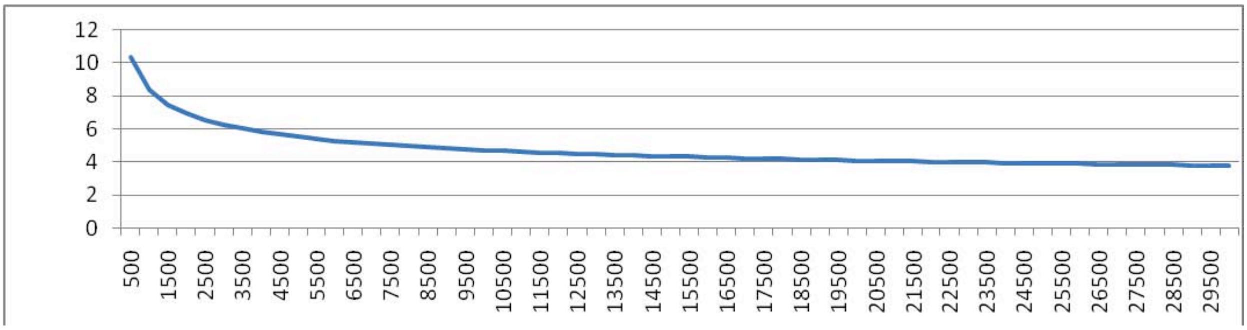

On the x-axis the cost of the material and the percentage of the cost of maintenance on the y-axis.

The chosen rule to calculate the costs of maintenance of the server is the following one:

SER_mnt =(SER_ach * 0,1 * (1+ (0,2 * ((SER_age/12)-1)))/(LN(SER_ach))^2) * 40

2.3.3 Coefficient of use

The coefficient **SER_coe** reflects the rate of use of the server by the database under study:

0 Insignificant part of the server is used to run the database
1 Only a small part of the server is used to run the database
2 Server is used equally to run the database and to run other devices
3 The majority of the server is used to run the database
4 The server is dedicated fully to the database

2.3.4 Annual cost of the server

The annual cost of the server is the result of the following calculation:

SER = (SER_amo + SER_mnt) * SER_coe / 4

2.3.5 Annual cost of the server if rented

Taking the example of the MNHN of Paris, the server hosting the GSDs (CIPA, FLOW, COOL, Psyl'list, Aradidae & MBB) is a space rented to the server of the University of Jussieu. In that case, the custodian will enter the annual cost excl. Tax of the rent. This example was true till the end of June 2010!!

SER_loc : the annual price of the rent excl. Tax of the server in **euros**

This data is a mandatory. No answer can be obtained in case of blank for the rental cost. On the other hand, the value zero is accepted.

IND_loc : Server rented or not. 1 if yes or 0 if no.

In that case the annual cost of the server will be:

SER = SER_loc * SER_coe / 4

2.3.6 Summary

So, in brief:

If **IND_loc = 1**
SER = SER_loc * SER_coe / 4
otherwise
SER = (SER_amo + SER_mnt) * SER_coe / 4

2.4 Custodian's Hardware

The custodian, in charge of the GSD under study, needs a minimum hardware requirement. A computer with its operating system is needed to manage the database maintenance.

It is clear that the cost of custodian's hardware will have no impact on the cost of maintenance if the custodian uses only sporadically its computer for the GSD.

Again only the material issue is concerned with in this chapter.
Its annual cost of maintenance, noted **MAT**, is calculated using the elements described below.

2.4.1 Amortization of the purchase price

The amortization of the computer hardware's price is generally made over 3 years. (Source: tax authorities). The hardware will be amortized using the digressive depreciation: 40 % on first year and 30 % on the following two years.

The rule of calculation of this element is as follows:

MAT_age : age of the hardware in **months**
MAT_ach : cost price excl. Tax of the hardware in **euros**
400 Ä default value of **MAT_ach** which can be adjusted before the calculation. The default value will be displayed at first use and users can put the real price if known or 0 if necessary.

If **MAT_age** > 36 months
MAT_amo = 0
Otherwise
If MAT_age <= 12 months
MAT_amo = **MAT_ach * 40%**
Otherwise
MAT_amo = **MAT_ach * 30%**

2.4.2 Cost of maintenance

The chosen rule to calculate the costs of maintenance of the hardware is the same that for the server:

$$MAT\_mnt = (MAT\_ach * 0{,}1 * (1+(0{,}2*((MAT\_age/12)-1)))/(LN(MAT\_ach))^2) * 40$$

2.4.3 Coefficient of use

The coefficient **MAT_coe** reflects the rate of use of the custodian's hardware by the database under study:

0	Insignificant part of the computer is used to manage the database
1	Only a small part of the computer is used to manage the database
2	Computer is used equally to manage the database and to run other devices
3	The majority of the computer is used to manage the database
4	The computer is dedicated fully to manage the database

2.4.4 Annual cost of the custodian's hardware

The annual cost of the hardware is the result of the following calculation:

$$MAT = (MAT\_amo + MAT\_mnt) * MAT\_coe / 4$$

2.5 The operating system of the server and the custodian's hardware

Its annual global cost of maintenance, noted **SYG**, is calculated using the elements described below.

SYG = SES + SEC with:

The cost of the operating system of the server, noted **SES**, is only considered in case of it purchase and not if it is rented. This cost includes the amount of the license and the price of its maintenance; e.g. Windows is an operating system under license.

The cost of the operating system of the custodian, noted **SEC**, must be entered in any cases. Most often, the price of the OS license is included in the purchasing price of the hardware of the custodian.

However, maintaining an operating system up-to-date has a cost. This is the system manager’s task. This cost will be impacted by the coefficients of use of the server and the custodian’s hardware.

With :

**HOR_ing**	Average labour cost of a system manager/IT engineer (in Euros per hour)
**50 Ä**	Default value of **HOR_ing** which can be adjusted before the calculation.
**HOR**	Average labour cost of the custodian/person in charge of entering data (in Ä/h)
**25 Ä**	Default value of **HOR_ing** which can be adjusted before the calculation.
**SER_coe**	Coefficient reflecting the rate of use of the server by the database under study
**MAT_coe**	Part of the computer’s resources used to enter data and manage the database (coefficient)
**SER_lic**	Licence cost for the server’s operating system (in Euros per year)
**IND_loc**	Indicator of server’s renting. 1 if rent or 0 otherwise.

To be as realistic as possible, variables **HOR_ing** and **HOR** must include the salary, the various social contribution costs, the paid leaves, the fringe benefits, etcÖ These variables should be communicated by the financial department of the institution.

SES =(**HOR_ing** * 16 + **SER_lic**) *(**SER_coe**/4)

SEC = **HOR** * 8 * (**MAT_coe**/4)

So in brief :

If IND_loc = 1
SES = 0
SYG = SEC
Otherwise
SYG = SES + SEC

2.6 The Database Manager

A database can be either local, being only available on one computer by a user, or distributed, the stored information being available on a distant computer and reachable via a network.

The main advantage of databases is the opportunity of being reached by several users simultaneously.

´ Early on, the need for a management system in order to control both data and users quickly arose. Database management is done using a system called a ***DBMS*** *(Database management system). The DBMS is a suite of services (software applications) for managing databases, which involves:*

> ** enabling simple access to data*
> ** allowing multiple users access to the information*
> ** manipulating the data found in the database (inserting, deleting, editing)*

The DBMS can be broken down into three subsystems:

> ** The file management system: for storing information in a physical medium*
> ** the internal DBMS: for placing information in order*
> ** the external DBMS: represents the user interface* [al]

The database manager is a software like Oracle for example that is under license.
Some GSDs use freeware, or free software, like PostgreSQL. These GSDs are not concerned by the cost of a license but only by the cost of maintenance.

Thus, whatever the type of manager system, it is necessary to take its maintenance – like the version upgrading – into account.

This cost will be impacted by the coefficient of use of the server for the GSD under study.

Its annual cost of maintenance, noted **GBD**, is calculated using the elements described below.

GBD = (**HOR_ing** * 16 + **GBD_lic**)* (**SER_coe**/4)

With :

> **HOR_ing** Average labour cost of a system manager/IT engineer (in Euros per hour)
> **50 Ä** Default value of **HOR_ing** which can be adjusted before the calculation.
> **SER_coe** Part of the server used to run the database (coefficient)
> **GBD_lic** Licence cost for the database management software (in Ä/year excl. Tax)
> **IND_loc** Indicator of server's renting. 1 if rent or 0 otherwise.

So in brief :

If **IND_loc** = 1

---

1 http://www.commentcamarche.net/contents/bdd/bddintro.php3

GBD = **HOR_ing** * 16* (**SER_coe**/4)
Otherwise
GBD = (**HOR_ing** * 16 + **GBD_lic**)* (**SER_coe**/4)

2.7 Antivirus software

Antivirus software is developed to identify, neutralize and eliminate any hostile element of your computer.

If the hardware under study is without antivirus program, put zero as amount for the cost of this software. We considered that the software is to be renewed every two year.

It is clear that this cost has to be shared between the various applications involving this software, so that the coefficient **MAT_coe** has to be taken into account in our formula for the calculation of this cost.

The cost of the annual antivirus software, noted **AVI**, is calculated using the elements described below.

AVI = **AVI_ach** * **MAT_coe** **/ 8**

With

AVI_ach Purchasing price of the antivirus (in **Euros** excl. Tax)
MAT_coe Part of the computer's resources used to enter data and manage the database (coefficient)
50 Ä Default value of **AVI_ach** which can be adjusted before the calculation.

Several GSDs have free antivirus software. Users should be aware that the default-displayed value is 50 euros but that they can put 0 if necessary.

2.8 The update of the Data, the Documentation

Updating data is the main factor of the cost of maintenance of a database.

Its annual cost of maintenance, noted **MAJ**, is calculated using the elements described below.

2.8.1 Description of the database complexity

The cost of the different actions linked to the update of a database depends on the database's complexity, which can be estimated according to, various factors to which are associated various coefficients:

- **Database type** **BD_typ** :

- Text file *Associated coefficient : 1*
- Excel spreadsheet *Associated coefficient : 2*

- Indexed sequential file — *Associated coefficient : 5*
- Evolved database — *Associated coefficient : 8*

- **Average number of fields to fill per species in the database BD_nbr** :

- Low (up to 10) — *Associated coefficient : 1*
- Medium (up to 50) — *Associated coefficient : 2*
- High (> 50) — *Associated coefficient : 3*

- **Database size BD_vol** :

- Small (up to 1 Giga) — *Associated coefficient : 1*
- Medium (up to 10 Giga) — *Associated coefficient : 1,2*
- High (> 10 Giga) — *Associated coefficient : 1,3*

- **Update rate BD_frq** :

- Full time — *Associated coefficient : 450*
- Daily — *Associated coefficient : 200*
- Weekly — *Associated coefficient : 50*
- Monthly — *Associated coefficient : 12*
- Sporadically — *Associated coefficient : 5*
- Never — *Associated coefficient : 1*

Concerning this project, one should remember that it deals only with the preservation of the existing databases (GSD). Thus, the entry tools, which allow to enrich the database, as well as tools of search and consultation, which allow to consult the database, are considered as existing and do not enter in the calculation of the cost of maintenance of the GSD.

2.8.2 Data Entry

Fifteen GSDs have kindly accepted to test the first version of the formula. Their comments and remarks have been incorporated in the new formula (v. 2) and one new coefficient has been added.

The type of database (text file, Excel file, indexed sequential file or evolved database) has no influence on the calculation of the cost of the data capture. Indeed the entry of the data will take the same time, only the size of the data entered will be different, but this will not be taken into account.

The cost of the update (creation or modification of data in the GSD), noted **MAJ_don** is the result of the following formula :

$$\mathbf{MAJ\_don = HOR * (1+(0,2 * BD\_nbr))*((LN(BD\_frq))^2 * TYP)}$$

With :

HOR Average labour cost of the custodian/person in charge of entering data (in **Euros** per hour)

BD_frq Update rate (coefficient)
BD_nbr Average number of fields per species in the database (coefficient)
TYP Coefficients resulting from values of **BD_frq**.

Coherence of obtained figures:

Here is a detailed example of calculation of data entry cost:
HOR = 50 euros per hour
In this example, the GSD is an evolved database, with an average number of fields to fill per species **BD_nbr** between 10 and 50.

•Update is made on a **full-time** basis.
In this case: MAJ_don = 78 378 euros. This amount could appear overestimated, but when calculated monthly, we get 7 125 euros (based on 11 months a year due to holidays). One-month comprising approximately 20 working days, we get a daily cost of about 326 euros. Or a little more than 7 hours a day of data entry in our GSD (50 euros per hour). It seems coherent.

•Update is made on a **daily** basis.
In this case: MAJ_don = 39 300 euros. This amount could appear overestimated, but when calculated monthly, we get 3 573 euros (based on 11 months a year due to holidays). One-month comprising approximately 20 working days, we get a daily cost of about 179 euros. Or 3h30mn a day of data entry in our GSD (50 euros per hour). It seems coherent.

•Update is made on a **weekly** basis.
In this case: MAJ_don = 16 070 euros. When calculated monthly, we get 1461 euros (based on 11 months a year due to holidays). One month comprising approximately 4,5 weeks, we get a weekly cost of about 325 euros. Or 6h30mn a week of data entry in our GSD (50 euros per hour). It seems coherent.

•Update is made on a **monthly** basis.
In this case: MAJ_don = 4 322 euros. When calculated monthly, we get 393 euros (based on 11 months a year due to holidays). Or 8h a month of data entry in our GSD (50 euros per hour). It seems coherent.

•Update is made on a **sporadic** basis.
In this case: MAJ_don = 1 088 euros. It is equivalent to 22 hours a year of data entry or two hours a month (50 euros per hour). It seems coherent.

•Database **never** up dated.
In this case: MAJ_don = 0 euro, which is of course seems coherent!!

We have compiled results of this test in the table below together with additional tests.

**CASE 1 : HOR= 50 €**	Full time	Daily	Weekly	Monthly	Sporadically	Never
Average number of fields <= 10	67181 (6h/d)	33687 (3h/d)	13774 (5h5/w)	3705 (7h/m)	933 (1h72/m)	0
Average number of fields > 10 et <=50	78378 (7h/d)	39300 (3h5/d)	16070 (6h5/w)	4322 (8h/m)	1088 (2h/m)	0
Average number of fields > 50	89575 (8h/d)	44915 (4h/d)	18365 (7h45/w)	4940 (9h/m)	1243 (2h27/m)	0
**CASE 2 : HOR= 25 €**	Full time	Daily	Weekly	Monthly	Sporadically	Never
Average number of fields < =10	33591 (6h/d)	16843 (3h/d)	6887 (5h5/w)	1852 (7h/m)	466 (1h72/m)	0
Average number of fields > 10 et <=50	39189 (7h/d)	19651 (3h5/d)	8035 (6h5/w)	2161 (8h/m)	544 (2h/m)	0
Average number of fields > 50	44787 (8h/d)	22458 (4h/d)	9182 (7h4/w)	2470 (9h/m)	622 (2h27/m)	0

d for day, **w** for week, **m** for month. Hours are **decimal** hours. The costs are given in **euros**.

2.8.3 The tools for corrective maintenance

Concerning this project, we remind that only the preservation of the existing databases (GSD) is concerned. Thus, the tools of maintenance allowing the development of the database are not involved in the current calculation of the cost of maintenance of the GSD. Here, the corrective maintenance is only considered in the case of data entry error, treatment error, and bad utilisation of the database.

During the testing period, we have noticed that the corrective maintenance also depends on the frequency of the data capture. Indeed, the more we use the editor for data entry, the more we have a chance to use unexplored roads and to detect possible errors.

That is why we used the stabilizing coefficient **SAU_frq** initially used for the GSD back up cost.
The frequency of updates is indicated by the parameter **BD_frq** (cf. 8.1) with **BD_frq** having the following values

▫ Full time	*Associated Coefficient : 450*	**SAU_frq = 4**
▫ Daily	*Associated Coefficient : 200*	**SAU_frq = 3**
▫ Weekly	*Associated Coefficient : 50*	**SAU_frq = 2**
▫ Monthly	*Associated Coefficient : 12*	**SAU_frq = 1**
▫ Sporadically	*Associated Coefficient : 5*	**SAU_frq = 1/2**
▫ Never	*Associated Coefficient : 1*	**SAU_frq = 0**

The cost of the corrective maintenance, noted **MAJ_cor** is the result of the following formula:

MAJ_cor = (**SAU_frq** /4) * (**HOR** * (**BD_nbr** + **BD_vol**) * **BD_typ**/2)

With :

HOR Average labour cost of the custodian/person in charge of entering data (in **Euros** per hour)
BD_nbr Average number of fields per species in the database (coefficient)
BD_vol Size of the database (coefficient)
BD_typ Database's type (coefficient)

2.8.4 Management of the documentation

During the development of an IT project, whatever it is, a technical documentation and an up-to-date and complete user documentation guarantee continuity and durability of the project. So that the project can be resumed by another person without difficulties.

The cost of the documentation's update of the GSD, noted **MAJ_doc**, is the result of the following formula:

MAJ_doc = **MAJ_cor** / 2

2.8.5 Annual cost of the data update, and the documentation update

The annual cost of the data update, the corrective maintenance and the update of the documentation is the result of the following calculation:

MAJ = **MAJ_don** + **MAJ_cor** + **MAJ_doc**

2.9 Mechanism of backup and associated support

As for any IT application, it is imperative to proceed regularly to the backup of the GSD. This cost, noted **SAU** is dependent on the used support, itself depending on the type and on the size of the database. The frequency of the backups is directly connected to the frequency of the updates of the GSD under study.

2.9.1 Coefficient of use

According to the first replies to the questionnaire sent in November 2009, we understood that the investment in the backup equipment was very disparate, which led us to introduce a new coefficient, the coefficient of use of the backup equipment, **SAU_coe**. This coefficient is based on the same logic as the coefficients of use of the server and the custodian's hardware.

The coefficient **SAU_coe** reflects the rate of use of the backup equipment concerning the database under study:

0 Insignificant part of the equipment is used to backup the database
1 Only a small part of the server is used to backup the database
2 The backup equipment is used equally by the database and other resources
3 The majority of the equipment is used to backup the database
4 The backup hardware is dedicated fully to the database

2.9.2 Backup hardware

The **amortization** of the computer hardware's price is generally made over 3 years. (Source: tax authorities). The hardware will be amortized using the digressive depreciation: 40 % on first year and 30 % on the following two years.

If SAU_ach (Purchasing price excl. Tax of the backup system, in Euros) **is equal to zero then the annual cost of the back-ups is also equal to zero**. **SAU = 0**.

This can appear, for example, when the backup system is part of the server or when it is rented together with the server.

We use the same calculation formula as for the server to estimate the amortization of the computer hardware's price noted **SAU_amo**. The rule of calculation of this element is as follows:

SAU_age : age of the backup system in months
SAU_ach : Purchasing price of the backup system (in Euros excl. Tax)

If **SAU_age** > 36 months
SAU_amo = 0

Otherwise

If **SAU_age** <= 12 months

SAU_amo = **SAU_ach * 40%**
otherwise
SAU_amo = **SAU_ach * 30%**

The annual cost of the support of maintenance, noted **SAU_sup**, will be :

SAU_sup = (SAU_amo + SAU_mnt) * SAU_coe / 4

with

If **SAU_ach** = 0

SAU_amo = **SAU_mnt** = **SAU_sup** = **SAU =0**

otherwise

SAU_amo Cf above

SAU_mnt=(SAU_ach*0,1*(1+(0,2*((SAU_age/12)-1)))/(LN(SAU_ach))^2)*40

2.9.3 Frequency of backups

According to the frequency of the backups which is directly linked to the frequency of the updates **BD_frq**, we shall use in our calculation a stabilizing coefficient which will be the following one **SAU_frq** and which will have the following value:

The frequency of the updates of the GSD under study is indicated by the parameter **BD_frq** that is the frequency of update of the database (cf. 8.1) with **BD_frq** having the following values:

▫ Several times a day	*Associated coefficient : 450*	**SAU_frq = 4**
▫ Daily	*Associated coefficient : 200*	**SAU_frq = 3**
▫ Weekly	*Associated coefficient : 50*	**SAU_frq = 2**
▫ Monthly	*Associated coefficient : 12*	**SAU_frq = 1**
▫ Sporadically	*Associated coefficient : 5*	**SAU_frq = 1/2**
▫ Never	*Associated coefficient : 1*	**SAU_frq = 0**

2.9.4 Annual cost of GSD's backup

The annual cost is the result of the following calculation:

SAU = (SAU_frq + 1) * SAU_sup / 4

2.10 General formula

According to the elements described earlier, the calculation of the cost of maintenance of a database can be formalized as follows:

COU = SER + MAT + SYG + GBD + AVI + MAJ + SAU

This formula is programmed in the Excel spreadsheet joined to this document.

We draw your attention on the fact that the result reflects only an order of magnitude of the cost of maintenance of the studied GSD.

2.11 Appendices

2.11.1 List of the customizable variables

Variable	Description	Value by default
**SER_age**	Server age in months. Necessarily different from zero	
**SER_ach**	Purchasing price excl. Tax of the server in euros	800
**SER_coe**	Part of the server used to run the database (coefficient: Cf. 3.3)	
**SER_loc**	Rental price excl. Tax for the server in euros	
**IND_loc**	Rented server? 1 if rent or 0 otherwise	
**MAT_age**	Age of the computer used to enter the data and manage the database in months. Necessarily different from zero	
**MAT_ach**	Purchasing price excl. Tax of the computer used to enter the data and manage the database in euros	400
**MAT_coe**	Part of the computer's resource used to enter data and manage the database (coefficient: Cf. 4.3)	
**HOR_ing**	Average labour cost of a system manager/IT engineer in Euros per hour (*)	50
**SER_lic**	Licence cost excl. Tax for the server's operating system (in Euros per year)	
**HOR**	Average labour cost of the custodian/person in charge of entering data in Euros (*) per hour	25
**GBD_lic**	Licence cost excl. Tax for the database management software (in Euros per year)	
**AVI_ach**	Purchasing price excl. Tax of the antivirus software (in Euros)	50
**BD_typ**	Database's type (coefficient: cf. 8.1)	
**BD_nbr**	Average number of fields per species in the database (coefficient: cf. 8.1)	
**BD_vol**	Size of the database (coefficient: cf. 8.1)	
**BD_frq**	Update rate of the database (coefficient: cf. 8.1)	
**SAU_ach**	Purchasing price excl. Tax of the back up system in Euros	
**SAU_age**	Back up system age (in months). Necessarily different from zero	
**SAU_coe**	Rate of use of the backup equipment (Coefficient: Cf. 9.1)	

(*)To be as realistic as possible, variables **HOR_ing** and **HOR** must include the salary, the various social contribution costs, the paid leaves, the fringe benefits, etc… These variables should be communicated by the accounting department of the institution.

2.11.2 Summary of the calculation's formulae

2.11.2.1 The annual cost of the server

The annual cost of the server is the result of the following calculation :

SER = (SER_amo + SER_mnt) * SER_coe / 4

Detailed formula :

If **SER_age** > 36

$$\text{SER} =((\text{SER\_ach}*0,1*(1+(0,2*((\text{SER\_age}/12)-1)))/(\text{LN}(\text{SER\_ach}))^{2})*40)*\text{SER\_coe}/4$$

Otherwise

If **SER_age** <= 12

$$\text{SER} =(((\text{SER\_ach}*40\%)+(\text{SER\_ach}*0,1*(1+(0,2*((\text{SER\_age}/12)-1)))/(\text{LN}(\text{SER\_ach}))^{2})*40))*\text{SER\_coe}/4$$

Otherwise

$$\text{SER} =(((\text{SER\_ach}*30\%)+(\text{SER\_ach}*0,1*(1+(0,2*((\text{SER\_age}/12)-1)))/(\text{LN}(\text{SER\_ach}))^{2})*40))*\text{SER\_coe}/4$$

2.11.2.2 The annual cost of the custodian's hardware

The annual cost of the custodian's hardware is the result of the following calculation:

$$\text{MAT} = (\text{MAT\_amo} + \text{MAT\_mnt}) * \text{MAT\_coe} / 4$$

Detailed formula :

If **MAT_age** > 36 months

$$\text{MAT} =((\text{MAT\_ach}*0,1*(1+(0,2*((\text{MAT\_age}/12)-1)))/(\text{LN}(\text{MAT\_ach}))^{2})*40)*\text{MAT\_coe}/4$$

Otherwise

If **MAT_age** <= 12 months

$$\text{MAT} =(((\text{MAT\_ach}*40\%)+(\text{MAT\_ach}*0,1*(1+(0,2*((\text{MAT\_age}/12)-1)))/(\text{LN}(\text{MAT\_ach}))^{2})*40))*\text{MAT\_coe}/4$$

Otherwise

$$\text{MAT} =(((\text{MAT\_ach}*30\%)+(\text{MAT\_ach}*0,1*(1+(0,2*((\text{MAT\_age}/12)-1)))/(\text{LN}(\text{MAT\_ach}))^{2})*40))*\text{MAT\_coe}/4$$

2.11.2.3 The annual cost of the operating system of the server and the custodian’s hardware

The annual cost of the operating system of the server and the custodian’s hardware is the result of the following calculation:

$$\text{SYG} = \text{SES} + \text{SEC}$$

Detailed formula :

If **IND_loc** = 1

SYG = HOR * 8 * (MAT_coe/4)

Otherwise

SYG = =(HOR_ing * 16 + SER_lic) * (SER_coe/4) + HOR * 8 * (MAT_coe/4)

2.11.2.4 The annual cost of the database manager

The annual cost of the database manager is the result of the following calculation :

If **IND_loc** = 1

GBD = HOR_ing * 16* (SER_coe/4)

Otherwise

GBD = (HOR_ing * 16 + GBD_lic)* (SER_coe/4)

2.11.2.5 The annual cost of the antivirus software

The annual cost of the antivirus software is the result of the following calculation :

AVI = **AVI_ach** * **MAT_coe** / **8**

2.11.2.6 The annual cost of the update of the data and the documentation

The annual cost of the update of the data and the documentation is the result of the following calculation :

MAJ = **MAJ_don** + **MAJ_cor** + **MAJ_doc**

Detailed formula :

MAJ =(HOR*(1+(0,2*BD_nbr))*(LN(BD_frq))^2*TYP))+3/4*((SAU_frq/4)*
(HOR* (BD_nbr+BD_vol)*BD_typ/2))

2.11.2.7 The annual cost of the GSD's backup

The annual cost of the GSD backup is the result of the following calculation :

SAU = (**SAU_frq** + 1) * **SAU_sup** / 4

SAU_frq being directly deducted from BD_frq.

Detailed formula :

If SAU_ach = 0

SAU =0

Otherwise

If SAU_age > 36 months

SAU=1/4*(SAU_frq+1)*(((SAU_ach*0,1*(1+(0,2*((SAU_age/12)-1))) /(LN(SAU_ach))^2)*40)* SAU_coe/4)

Otherwise

If SAU_age <= 12 months

SAU = 1/4*(SAU_frq+1)*((SAU_ach * 40%)+((SAU_ach*0,1* (1+(0,2*((SAU_age/12)-1)))/(LN(SAU_ach))^2)*40)*SAU_coe/4)

Otherwise

SAU = 1/4*(SAU_frq+1)*((SAU_ach * 30%)+ ((SAU_ach*0,1* (1+(0,2*((SAU_age/12)-1)))/(LN(SAU_ach))^2)*40)*SAU_coe/4)

2.12 Instructions for use of the excel form

The Excel file allowing to calculate the cost of maintenance of your database at a given moment must be used carefully. Excel is not dedicated to the development of an editor and offers no validity check when entering the data, nor display of the default values at every use... This brief chapter presents how using and the functioning of this Excel file for your GSD.

The file consists of 4 spreadsheets: Parameters entry, Calculation Results, Calculation Details and Choice List Information. **The only spreadsheet which you have to fill in with data is the first one: Parameters entry.** The two others give you the results, and the last one is a reminder of the possible values of the coefficients.

After entering the various parameters on the spreadsheet entitled " Parameters entry ", the global cost will be displayed automatically. On the two other spreadsheets, you will have the details of the results sorted by major items like server, custodian's hardware, backup system or in a even more detailed way.

The fourth spreadsheet is only displayed to facilitate the entry of the coefficients by the user, in a totally transparent way for the user by means of drop-down menu. This fourth spreadsheets allows to use these drop-down menus.

2.12.1 Spreadsheet « Parameters Entry »

Name of the GSD under study.
Name enter by the user of this Excel file.

Cost of maintenance calculated automatically according to the entry of the parameters.

**DATABASE**		**XXXX**	
**ANNUAL COST**	**1 248,00**		
**PARAMETERS ENTRY**			
**Human resources**			
Labour cost of the custodian or person in charge of entering data (in €/hour includ. charges)			
Labour cost of the system manager/IT engineer (in €/hour includ. charges)			
**General information about the database**			
Database type (select right item)		**Text file**	
Average number of fields per species (select right item)		**Low (up to 10)**	
Size of the database (select right item)		**Low (up to 1 Giga)**	
Update rate (select right item)		**Never**	
**Server**			
Purchasing price of the server (in Euros excl. Tax)		**800**	
Age (in months)		**12**	
Part of the server used to run the database (select right item)		**Equal part**	
Rented server ((select right item)		**No**	
Rental price for the server (in Euros excl. Tax)		**0,00**	
Annual cost of the Server Operating System (in Euros excl. Tax)		**0,00**	
Annual licence cost of the Database Management System (in Euros excl. Tax)		**0,00**	
**Custodian's Hardware**			
Purchasing price (in Euros excl. Tax)		**400**	
Age (in months)		**12**	
Part of the custodian's hardware used to enter data and run the database (select right item)		**Equal part**	
**Antivirus software**			
Purchasing price (in Euros excl. Tax)		**50,00**	
**Back up Hardware**			
Purchasing price (in Euros excl. Tax)		**0**	Warning: is it really the price ?
Age (in months)		**0**	
Part of the Hardware used to back up the database (select right item)		**Equal part**	

This sign means that a drop-down menu is available. By clicking on the sign the list appears.

Message displayed when the user enters the value 0 for the cost price of the server, the custodian's hardware or the hardware for backup. It does not prevent the entry of 0 but draws the attention of the user on this fact.

Identification of cells

Spreadsheet " Parameters Entry "

Value entered by the user

PARAMETERS ENTRY	
**Human resources**	
Labour cost of the custodian or person in charge of entering data (in €/hour includ. charges)	25,00
Labour cost of the system manager/IT engineer (in €/hour includ. charges)	50,00
**General information about the database**	
Database type (select right item)	Text file
Average number of fields per species (select right item)	Low (up to 10)
Size of the database (select right item)	Low (up to 1 Giga)
Update rate (select right item)	Never

Cell with drop-down list:
→ *Corresponds to the value of the spreadsheet " Choice List Information"*

Spreadsheet " Choice List Information "

BD : Database's type	1	Text file
Text file	1	
Excel spreadsheet	2	
Indexed sequential file	5	
Evolved database	8	

Value corresponding to choices made in " Parameters Entry " . This value will be used in the calculations.

2.12.2 Spreadsheet «Calculation results »

DATABASE	XXXX
**ANNUAL COST OF MAINTENANCE**	
Server	196
Custodian's Hardware	102
Operating System of the Server and the Custodian's Hardware	500
Database Management Software	400
Antivirus	13
Data and Documentation Update	38
Back-up	0
**Total**	**1 248**

The name of the GSD under study is repeated on each spreadsheet of the Excel file.

2.12.3 Spreadsheet « Calculation details »

DATABASE	XXXX
**Server**	
Amortization	320
Cost of Maintenance	72
**Annual Cost of the Server**	**196**
**Custodian's Hardware**	
Amortization	160
Cost of Maintenance	45
**Annual Cost of the Custodian's Hardware**	**102**
**Operating System of the Server and the Custodian's Hardware**	
Server Cost	400
Custodian's Hardware Cost	100
**Total**	**500**
**Database Management Software**	
**Total**	**400**
**Antivirus**	
**Total**	**13**
**Data and Documentation Update**	
Data Entry Cost	0
Cost of the Corrective Maintenance	25
Cost of the Documentation Update	13
**Total**	**38**
**Back Up**	
Amortization	0
Non-weighted Cost of Back-up Hardware Maintenance	0
Partially Weighted Cost of Back-up Hardware Maintenance	0
**Annual Cost of Back up**	**0**
**Global Cost of the GSD maintenance**	**1 248**

This spreadsheet details the intermediate results to point out the items with high cost for this GSD.

Furthermore, in this period of test of the formula, it allows the custodian to see if the formula is close to the reality. If it is not the case, this spreadsheet allows to see where the formula must be modified.

Let us see now the link between the results of the " Calculation detail " and the " Calculation results " spreadsheets.

Spreadsheet" Calculation Details "

**Server**	
Amortization	320
Cost of Maintenance	72
**Annual Cost of the Server**	**196**
**Custodian's Hardware**	
Amortization	160
Cost of Maintenance	45
**Annual Cost of the Custodian's Hardware**	**102**

The annual cost of the server is not necessarily the sum of both lines because the coefficient of use of the server balances the global cost of the server

Total of the item "Server"

Spreadsheet " Calculation Results "

➔ Summary of the calculations made in the spreadsheet " Detail of the calculations "

DATABASE	XXXX
**ANNUAL COST OF MAINTENANCE**	
Server	196
Custodian's Hardware	102
Operating System of the Server and the Custodian's Hardware	500
Database Management Software	400
Antivirus	13
Data and Documentation Update	38
Back-up	0
**Total**	**1 248**

Total of the item "Server" of the spreadsheet " Calculation Details"

Total of the items = global cost of maintenance of the GSD under study

2.12.4 Spreadsheet «Choice List Information»

This spreadsheet lists all the drop-down menus of the spreadsheet " Parameters entry" with the correspondence between the user's choice and the value of the associated coefficient.

Furthermore, for every parameter is shown the last choice made by the user.

Choice list	Value	Your Choice in page 1
**BD : Database's type**	8	Evolved database
Text file	1	
Excel spreadsheet	2	
Indexed sequential file	5	
Evolved database	8	
**BD : Average number of fields per species in the database**	3	High (>50)
Low (up to 10)	1	
Medium (up to 50)	2	
High (>50)	3	
**BD : Database size**	1	Low (up to 1 Giga)
Low (up to 1 Giga)	1	
Medium (up to 10 Giga)	1,2	
High (>10 Giga)	1,3	
**Update rate**	5	Sporadically
Full time	450	
Daily	200	
Weekly	50	
Monthly	12	
Sporadically	5	
Never	1	
**Part of the server used to run the database (coefficient)**	2	Equal part
Insignificant part	0	
Small part	1	
Equal part	2	
Major part	3	
Fully dedicated	4	
**Rented server (Yes/No)**	0	No
Yes	1	
No	0	
**Part of the custodian's hardware used to enter data and run the database (coefficient)**	2	Equal part
Insignificant part	0	
Small part	1	
Equal part	2	
Major part	3	
Fully dedicated	4	
**Part of the Hardware used to back up the database (coefficient)**	2	Equal part
Insignificant part	0	
Small part	1	
Equal part	2	
Major part	3	
Fully dedicated	4	

Last choice made by the user

Value associated with this choice

Configuration History			
**Version No.**	**Date**	**Changes made**	**Author**
1.0	20 September 2010	First draft for internal circulation and testing	PBF/ThB
1.1	12 October 2010	Second draft for circulation (12 GSDs custodians, Y. de Jong, Ch. Hussey, A. Guentsch)	PBF/ThB/JK
1.2	6 December 2010	Revised document (new tests)	PBF/ThB/JK
2	14 December 2010	Revised document (circulating document)	PBF/ThB/JK
3	15 February 2011	Final version delivered to WP1	PBF/ThB/JK
4.4	16 March 2011	Extended introduction (Ch 1)	JK/ThB
4.5	17 March 2011	Final version for submission	YdJ
