# Supplementary material for: PESI - a taxonomic backbone for Europe
Source: Biodivers Data J. 2015 Sep 28;(3):e5848. doi: 10.3897/BDJ.3.e5848 (PMC4609752; doi:10.3897/BDJ.3.e5848)
Supplement: Supplementary material 24 — PESI web portal [file biodiversity_data_journal-3-e5848-s024.pdf]

A pan-European Species-directories Infrastructure (PESI)

WP6 – Integrated e-Services for users and dissemination

D6.5 - M6.5 PESI web portal

Follow-us on twitter: @EU_nomen

211,638 valid/accepted species and 442,383 taxon names
149,763 invalid/unaccepted taxon names
132,616 common names in 88 languages
268,355 sources (literature references)
6,294,659 distribution records

Official launch of the portal: 18 April 2011, in Amsterdam

Summary

PESI is Europe’s e-infrastructure for taxonomic information on species occurring in Europe. It successfully integrates four major European checklists into a single portal at www.eu-nomen.eu/portal. Among the nearly half a million scientific names in this database, it contains 211,000 valid species and 150,000 synonyms. PESI has also become a major resource for non-scientific names of species with 132,000 common names in not less than 88 languages.

The portal provides information pages for each of these species and serves permanent unique identifiers, which enables clients to use, besides names (which are subject to change), also these (persistent) identifiers that are linked to the species concepts from the different component databases.

PESI has been networking with 45 focal points in 25 countries. They gathered information on local/national taxonomic expertise. The database contains contact and expertise details of nearly 2,000 experts and more than 500 organizations (from professional institutions to amateur societies) in Europe.

The PESI portal has been major step forward towards standardization of species names in Europe.

Introduction

PESI is Europe’s e-infrastructure for taxonomic information on species occurring in Europe.

The online portal integrates several major European check-lists: Fauna Europaea (FaEu), Euro+Med Plantbase (E+M), European Register of Marine Species (ERMS, the EU component of the World Register of Marine Species), and the EU component of Index Fungorum (IF). These registers still operate on their own (different host institutions, committees, experts and servers). However, the

data is merged in the PESI Data Warehouse, on a regular (yearly) interval, and is available through a single portal at: www.eu-nomen.eu/portal. An EU component of AlgaeBase is in preparation for a next version of the PESI Data Warehouse.

In addition to taxonomic information, PESI harvests information on species (common names, images (through web services, e.g. Nederlands Soortenregister (NLSR), distributions (through web services, e.g. Atlas Florae Europaeae (AFE), literature, conservation status and provides links to other portals (e.g. national check-lists, red species lists) and other bioinformatics databases such as the GenBank sequence database, the Barcode of Life database (BOLD) and the Biodiversity Heritage Library (BHL) for literature data.

Besides an attractive portal with a web page on every species, the portal provides an intelligent name validation service and web services to cross-match external species lists against names in PESI. This enables the end-users to standardize species names and promotes the use the persistent globally unique identifiers (GUIDs) generated by the different checklists.

Fig. 1. Screenshot of the homepage of the PESI web portal.

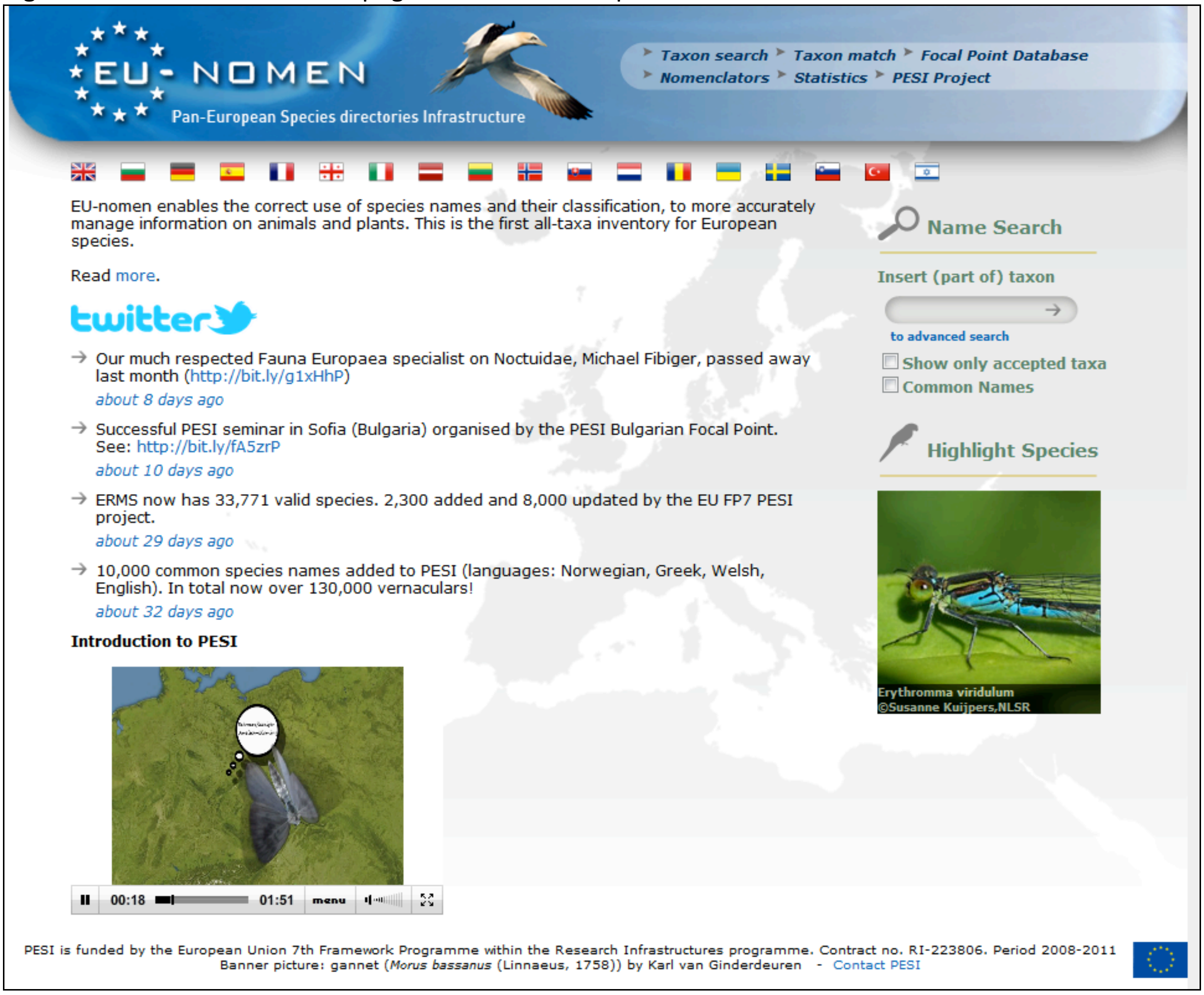

Statistics

The PESI species database contains:

- 211,638 valid/accepted species
- 442,383 taxon names
- 149,763 invalid/unaccepted taxon names
- 132,616 common names in 88 languages
- 6,294,659 distribution records
- 268,355 sources (literature references)

Table 1. Overall overview of number of taxa, species, infraspecies and all ranks per checklist (dataset).

Dataset	Above species	Species	Below species	All Ranks
FaEu all (accepted / valid & not accepted / valid)	42018	172466	19807	234291
FaEu all + orginal combinations	42018	233192	26184	301394
FaEu only accepted / valid	34300	131672	14234	180206
Euro+Med PlantBase all	5173	75934	41450	122557
Euro+Med PlantBase only accepted / valid	2371	25830	10116	38317
ERMS all	19058	43236	1322	63616
ERMS only accepted / valid	17565	33685	769	52019
Europe Index of Fungi all	3969	22384	0	26353
Europe Index of Fungi only accepted / valid	3969	22384	0	26353
FaEu-ERMS overlap all	1923	1806	20	3749
FaEu-ERMS overlap only accepted / valid	1859	1664	20	3543
IF-ERMS overlap all	389	217	0	606
IF-ERMS overlap only accepted / valid	386	207	0	593
E+M-ERMS overlap all	45	32	2	79
E+M-ERMS overlap only accepted / valid	43	27	1	71
PESI-DWH all	67861	311965	62557	442383
PESI-DWH only accepted / valid	55885	211638	25097	292620

The PESI portal also provides information on species of societal importance. The species in PESI are linked to priority species lists (see overview below). However, not every species from these lists could be linked to a name in PESI. Were possible, PESI provides deep links to the species pages on their web sites (e.g. to IUCN red list and HYPPZ).

Table 2. Overview of the number of species in priority lists vs those that match a name in PESI.

priority_list	total	matching
CITES (global)	1,174	147
EPPO alert	45	15
EPPO invasive plants	40	26
EU Birds Directive	308	305
EU Habitat Directive	2,244	1,570
HYPPZ	371	222
IUCN	2,896	2,196
OSPAR	43	41

How to search information on the PESI portal?

The user can search for a particular taxon by entering (part of) the scientific name, name authority or common name. One can also create a list of taxa that correspond to a number of search criteria (e.g. on the basis of a higher taxon, the occurrence status in a country, be listed in one or any of the priority species lists).

If there is no exact match, the search tool performs a number of consecutive queries until matches are found:

1. fuzzy match (Tony Rees' TAXAMATCH algorithm)
2. other potential genus-species combinations (FaEu model*: checks for reverse synonyms, e.g. if the species epithet occurs in other genera that are synonym to the genus that is entered in the search box)
3. other potential genus-species combinations (WoRMS model: checks if the species epithet occurs in other genera within the same *Classis*)
4. checks if the name is present in WoRMS
5. checks if the name is present in CoL
6. checks if the name is present in GNI.

* FaEu model for potential combinations, e.g.:

genus *Euphydryas* = valid
genus *Eurodryas* = syn of *Euphydryas*
genus *Hypodryas* = syn of *Euphydryas*
genus *Occidryas* = syn of *Euphydryas*

species *Euphydryas aurinia* = valid
species *Eurodryas aurinia* = potential combination of *Euphydryas aurinia*
species *Hypodryas aurinia* = potential combination of *Euphydryas aurinia*
species *Occidryas aurinia* = potential combination of *Euphydryas aurinia*

species *Melitaea beckeri* = syn of *Euphydryas aurinia*
species *Euphydryas beckeri* = potential combination of *Melitaea beckeri*
species *Eurodryas beckeri* = potential combination of *Melitaea beckeri*

The web portal also makes it possible to create a list of matching results after setting a number of taxonomic, geographical or other criteria. For example: create a list of taxa (any rank) based on:

- a higher parent taxon and/or
- geographical zones and/or
- additional data types such as conservation status

For example, this allows the web visitor to generate a list of all protected bird species occurring in Belgium. You can use wildcards in the taxon name field or leave it blank to obtain a full list.

Fig. 2. Screenshot of the search page with the various selection boxes.

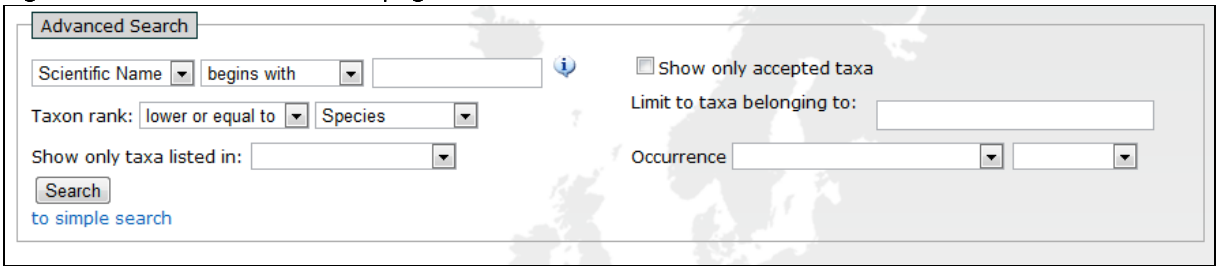

Taxon match, an ABC tool for species names

The correct spelling of a species name is not always trivial (which one is correct: Cirrhitichthys, Cirrhitychthys or Cirritichthys?) and it is very difficult for non-taxonomists to keep up with the valid status of species names. PESI has developed a powerful online name matching tool to standardize your names with the PESI database.

The tool returns standard PESI taxonomic information in a user-friendly format (e.g. MS Excel or tab-delimited text file). The user needs to upload a list of species names, match the columns with the fields in the PESI data warehouse and the system will return the file with valid names (notifies when the name is an unaccepted synonym), the authority and publication date, the hierarchical classification, quality status (expert validated or not) and the check-list's Globally Unique Identifiers. When there are multiple matches the system provides a pick-list. To avoid matching with homonyms, you can limit the query to a specific higher rank (e.g. Aves, Mollusca, etc). If your species list is restricted to a particular area, you can also check if this corresponds to the occurrences in the PESI database (via the "limit taxa belonging to a particular country" box).

The tool is an implementation of the fuzzy matching algorithm written by Tony Rees (CSIRO, Australia), which comprises a suite of custom filters and tests used in succession on genus, species epithet, plus authority where supplied. We also used the Scientific Names Parser written by Dmitry Mozzherin.

For more information on the taxon match algorithm, visit
http://www.cmar.csiro.au/datacentre/taxamatch.htm.

Fig. 3. Screenshot of the first page of the taxon match tool.

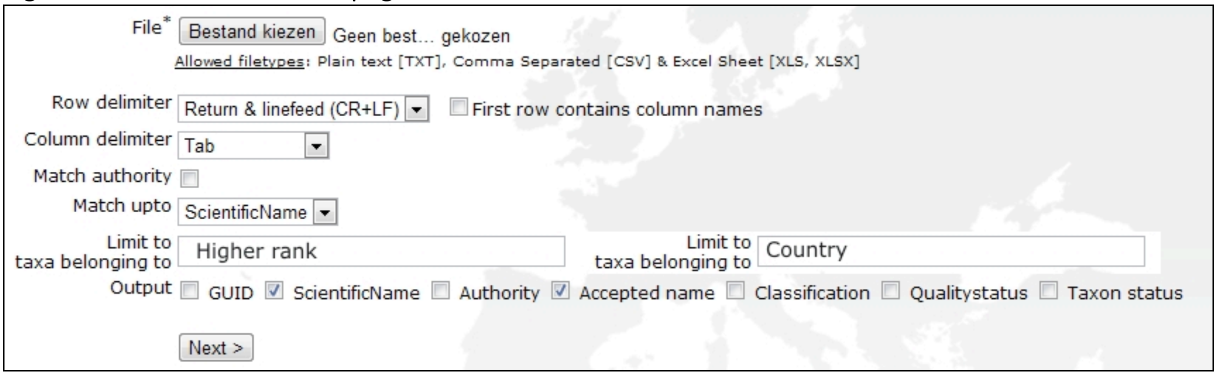

Species occurrence data

PESI has two types of occurrence data on the portal.

The first type is provided by the component databases and directly included in the PESI datawarehouse. The areas used have been standardized to TDWG areas (http://www.kew.org/gis/tdwg/) for terrestrial areas and for marine areas to the IHO/EEZ Intersect standard proposed by VLIZ: (http://www.vliz.be/vmdcdata/vlimar/downloads.php#Intersect%20IHO%20and%20EEZ).

These occurrences are shown on the map as polygons and colored according to the Occurrence Status.

The second type of occurrence data are those provided by Atlas Florae Europaeae (more info in 'how to contribute data').

The map is built on OpenLayers (www.openlayers.org), an open source javascript library to display dynamic maps in any web page. The backend of both occurrence types is GeoServer (www.geoserver.org), an open source implementation of WMS that implements the Open Geospatial Consortium (OGC) standards.

Fig. 4. Two screenshots of occurrence maps, on the left those served by the PESI component databases, on the right those served by Atlas Florae Europaeae, for species *Atriplex patula*. Both occurrence types can be shown/hidden on the map.

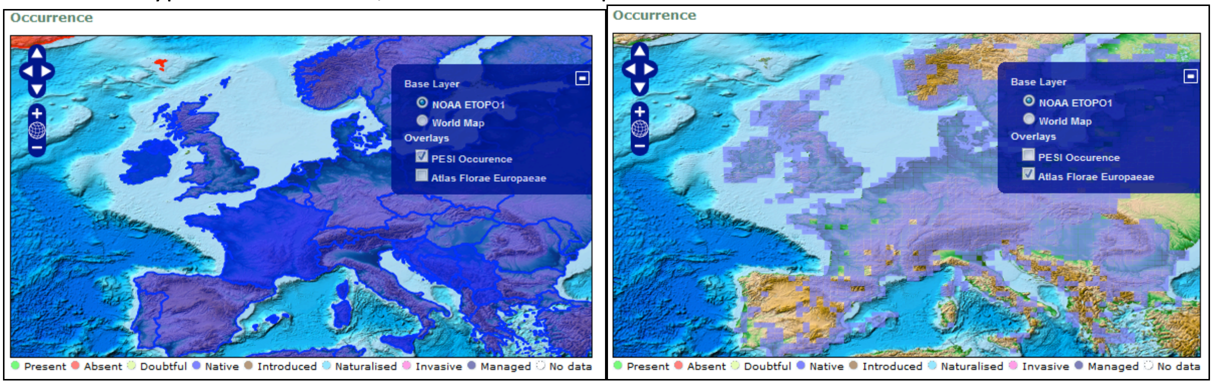

PESI web services

The PESI portal provides a web service that allows users to dynamically link their own applications to the PESI database and will allow them to match a locally stored species list with the PESI check list and add taxonomic and additional information derived from PESI.

We currently support the platform-independent SOAP/WSDL standard.

Web Service Definition Language File: WSDL file
Brief description: PESINameService description

A few examples of possible applications:

- getGUID: Get the first exact matching GUID for a given name.

- getPESIRecords: Get one or more matching (max. 50) PESIRecords for a given name.
- getPESINameByGUID: Get the correct name for a given GUID.
- getPESIRecordByGUID: Get the complete PESI Record for a given GUID.

Globally Unique Identifiers (GUIDs)

Over the last couple of years the Biodiversity Informatics community underwent a vivid debate on the implementation of persistent Globally Unique Identifiers (GUIDs) for the object types to be networked in the emerging biodiversity data infrastructure (see http://wiki.tdwg.org/GUID).

For PESI, the assignment of GUIDs is restricted to scientific names (i.e. nomenclatural entities and not name strings) and taxa (a scientific name used in a certain context). PESI will primarily be used as an authoritative resource for these two core object types and hence should offer persistent GUIDs to serve other infrastructures and networks.

Who will create GUIDs in the PESI Network?

Each participating checklist is responsible for assigning GUIDs to their objects. The GUIDs can be "raw" and do not have to follow a specific protocol (example: B85E62C3-DC56-40C0-852A-49F759AC68FB, used by E+M) or can be based on the checklist's internal identifier systems and have the format of Life Science Identifiers (LSIDs). ERMS and FaEu has implemented LSIDs for all its taxonomic names.

For example, the LSID for *Solea solea* is: urn:lsid:marinespecies.org:taxname:127160

You can resolve an LSID via the various services available. Example for Solea solea:
http://lsid.tdwg.org/summary/urn:lsid:marinespecies.org:taxname:127160

The returned model is RDF (XML) with metadata elements from Darwin Core and Dublin Core.

The checklist GUIDs are listed on every taxon page, and is part of the URL. You can use the taxon match or the web service to add these GUIDs to your list of names.

Contribute data to PESI

The taxonomic information comes from four European checklists (ERMS, FaEu, E+M and IF). If you wish to contribute taxonomic information, you should contact the managers of these checklists.

If you have comments/remarks on a particular taxon, you can use the online feedback form. We will forward your message to the responsible person.

The PESI portal can establish links with other (national) species portals and exchange data and information in an automated manner. There are currently several successful cases:

1. Distribution data from the Atlas Florae Europaeae (AFE).

Atlas Florae Europaeae (AFE) provides its occurrence data via a Web Mapping Service (WMS) server set up at the University of Helsinki. They have been extracted from the AFE volumes

(http://www.luomus.fi/english/botany/afe/index.htm) and displayed as a grid colored by the PESI Occurrence Statuses. These occurrences are restricted to a number of vascular plants described in AFE.

Fig. 5. Screenshot of the distribution map, showing the occurrences of *Atriplex patula*, as served by the Atlas Florae Europaeae.

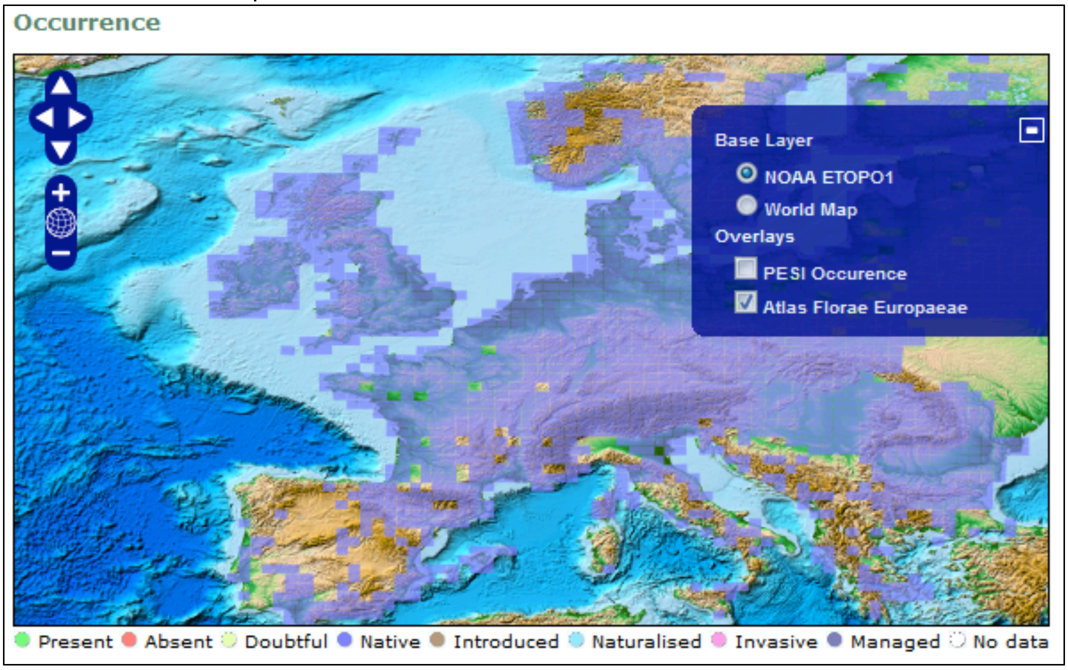

2. Images from the Dutch Species Register (NLSR).

We have established a dynamic connection with the Dutch Species Register (NLSR). The NLSR portal serves the information of species images in an RDF XML format, which we harvest and display on the portal via a POST/GET request. The species pages show thumbnail images and the metadata of each image (author + source). When clicking on the thumbnail you are directed to the full image URL on the source website.

It works as follows: when displaying a PESI taxon page the PESI web server contacts the NLSR web service at http://www.nederlandsesoorten.nl/get?site=nlsr&view=nlsr&id=i000091&action=search&searchString=Taxon. It sends one input parameter (searchString) which corresponds to the name of the taxon. The web service at NLSR responds with XML-structured data. This XML tells us whether there are images available for this particular taxon or not. If any references to images are present in the XML results, they contain absolute links to the image files at NLSR. When displaying the PESI taxon page these pictures are included in the HTML output by using the image URL's we retrieved from the web service.

For example:

- NLSR: http://www.nederlandsesoorten.nl/get?site=nlsr&view=nlsr&id=i000091&action=search&searchString=passer montanus

- PESI: http://www.eu-nomen.eu/portal/taxon.php?GUID=urn:lsid:faunaeur.org:taxname:97441

Fig. 6. Screenshot of the thumbnail images served by the Nederlands SoortenRegister.

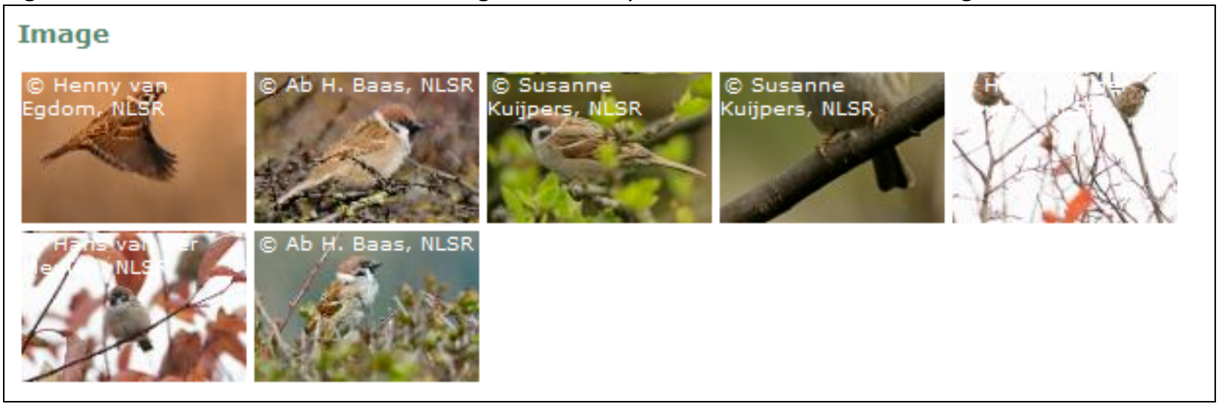

3. Deep links to the Biodiversity Heritage Library (BHL), the Barcode of Life Database (BOLD) en GenBank.

The biodiversity heritage library (BHL) website is queried at http://www.biodiversitylibrary.org/services/pagesummaryservice.ashx?op=PageNameSearchForTitles&name=taxon name. The PESI web portal performs a regular expression match on the keys 'TitleCount' and 'PageCount'. When matches are found, the information is cached in our database for one month. The next time someone loads that particular taxon page within the same month, the cached information is displayed. If the request time compared to the last update in the database is above one month, the information is refreshed by re-querying the BHL web service.

Genbank provides a standard method for linking to their pages. We retrieve the information from http://www.ncbi.nlm.nih.gov/Taxonomy/Browser/wwwtax.cgi?name=taxon name. When the link exists, it is displayed on the species page.

BOLD results are fetched from http://www.boldsystems.org/views/taxbrowser.php?taxon=taxon name. Only when the 'specimens with barcodes' parameter is set, link is visible on the species page.

Fig. 7. Screenshot of the deeplinks to BHL, BOLD and GenBank, for *Passer montanus.*

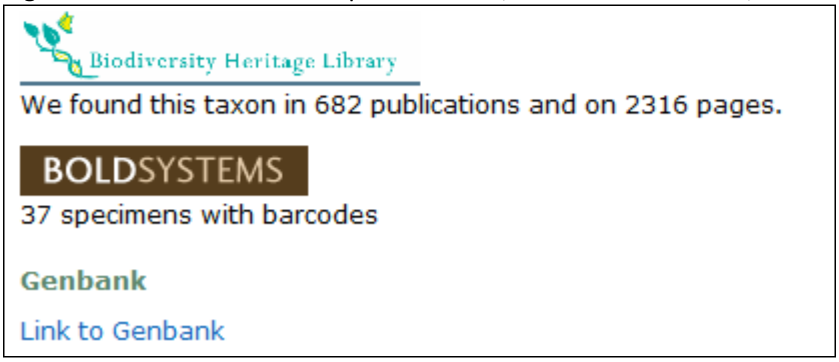

Please contact us at info@eu-nomen.eu if you wish to contribute to PESI and/or wish to set up similar dynamic connections.

EU Biodiversity Expertise and Resource Database

This is the PESI focal point database. PESI focal points are national representatives for biodiversity and taxonomic research. There are 45 focal points from 25 countries. This database hosts information on local/national taxonomic expertise. The different modules are experts, journals and publications, societies and institutions and relevant websites. The database contains contact details and expertise details of nearly 2,000 experts and more than 500 organisations (from professional institutions to amateur societies).
The modules are interlinked, and each item is recorded only once. You can query the database via the portal at: http://www.eu-nomen.eu/portal/imis.php?module=person&firstview=1

Fig. 8. Screenshot of the search interface of the PESI focal point database.

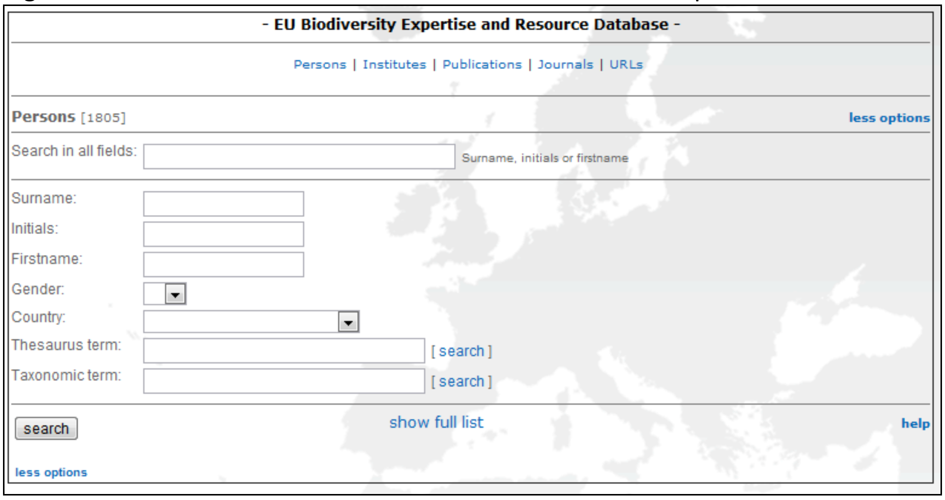

Disclaimer

General disclaimer
The PESI Data Administrators reserves their exclusive right in its sole discretion to alter, limit or discontinue the Site or any Materials in any respect. The PESI Data Administrators shall have no obligation to take the needs of any User into consideration in connection therewith. The PESI Data Administrators reserves the right to deny in their sole discretion any user access to this Site or any portion thereof without notice. No waiver by the Data Administrators of any provision of these Terms and Conditions shall be binding except as set forth in writing and signed by its duly authorized representative.

No Warranty
PESI and its data suppliers provide this database free of charge for the benefit of the public in an "as is" condition. Neither PESI nor its data suppliers warrants, guarantees, or makes any representation regarding the accuracy, completeness, correctness, reliability, currency or otherwise, of the databases or the use or results to be obtained from using the databases or the information contained therein, or any related documentation or written materials. Neither PESI nor its data suppliers makes any representations or warranties whatsoever, express or implied, with respect to the database, and, in particular, PESI and its data suppliers disclaim all implied warranties including without limitation any warranties of merchantability, non-interference, non-infringement, informational content, or fitness for a particular purpose with regard to the database.

Limitations on liability
Except to the extent required by applicable law, in no event will PESI or its data suppliers be liable to you on any legal theory for any special, incidental, consequential, punitive or exemplary damages arising out of this license or the use of the work, even if licensor has been advised of the possibility of such damages.

Governance and Copyright

The content of the PESI databases is vested in the Society for the Management of Electronic Biodiversity Data Ltd (www.smebd.eu). All scientists contributing to the databases are eligible for SMEBD membership, and thus share collective responsibility for ensuring the data are quality controlled, maintained, and hosted by appropriate institutions. Upon completion of the PESI project, SMEBD continues to develop the databases in collaboration with their host organisations. Decisions on the management of the databases, such as appointing and replacing experts to edit their content, and providing copies to third parties, are made by specific database committees under the authority of the SMEBD Council.

For new databases, contributors interested in becoming involved and providing information to PESI will have the option of allowing SMEBD to take over the management of the selection of data they provide and signing a SMEBD agreement form (download from SMEBD -> documents -> Member Agreement Forms or go directly to
http://www.smebd.eu/index.php?option=com_remository&Itemid=2&func=fileinfo&id=83).

The PESI portal will have a common approach to citation and Creative Commons licensing for all databases, including databases outside the SMBED committee. The copyright used will follow the Attribution-Share Alike scheme (for more information, view
http://creativecommons.org/licenses/bysa/
3.0/). Ideally, all data providers should abide by the same license requirements to avoid conflicting policy interactions if combined datasets from different sources are downloaded through the PESI facilities.

Terms of Use

By downloading or consulting data from this website, the visitor acknowledges that he/she agrees with the PESI data policy, and agrees to the following:

• If data is extracted from the PESI website for secondary analysis resulting in a publication, the PESI website should be cited following the citation scheme given below.

Users must acknowledge the contribution of the relevant Data Providers in any derived information product or publication, whether printed, electronic or broadcast, that is based wholly or in part on the material, data and/or information they make available to them. Where users make specific use of or reference to a particular biological dataset they must acknowledge the original data provider (where their name is made available) using the referencing format provided within the PESI database.

The citation system proposed for PESI generally follows that used in citing works published in hardcopy and so should be straightforward for researchers to use.

- [Author/s][Year]. [Chapter] In [Book]. [Publisher]. [Page]

The template to be used for PESI will be as follows when verified by expert/contributed by European Species Database (ESD) or other database outside SMEBD:

- Editors (Year). Taxon + Authority. In: ESD title or other Database or data manager. Accessed through the PESI at [URL] on [date].

For example: Reid, David G., Gofas, S. (2010). *Littorina littorea* (Linnaeus, 1758). In: Bouchet, P.; Gofas, S.; Rosenberg, G. (2010) European Register of Marine Species, Accessed through the PESI at http://www.eu-nomen.eu/portal/taxon.php?GUID=urn:lsid:marinespecies.org:taxname:140262 on 2010-07-28.

Where the record has not been verified the database is the author, in which case the following template is used.

- PESI (Year). Taxon + Authority. Accessed through PESI at [URL] on [date].

For example:

- PESI (2010). *Salmo salar* Linnaeus, 1758. Accessed through PESI at http://www.eu-nomen.eu/portal/taxon.php?GUID=urn:lsid:marinespecies.org:taxname:127186 on 2010-07-28.

To cite the entire database, use the following citation:

PESI (Year). Pan-European Species directories Infrastructure. Accessed through www.eu-nomen.eu/portal, at [date]

Archive

The PESI portal only shows the latest version of the PESI datawarehouse. Previous versions are archived in Miscrosoft SQL server 2008 format and are stored at the VLIZ Marine Data Archive (MDA; http://mda.vliz.be). VLIZ is an official national data centre and the data on these servers are also stored on back-up servers and on tapes that are physically stored on a remote location.

Fig. 9. Screenshot of the Marine Data Archive at VLIZ, where the entire PESI database is backed-up, and versions are saved.

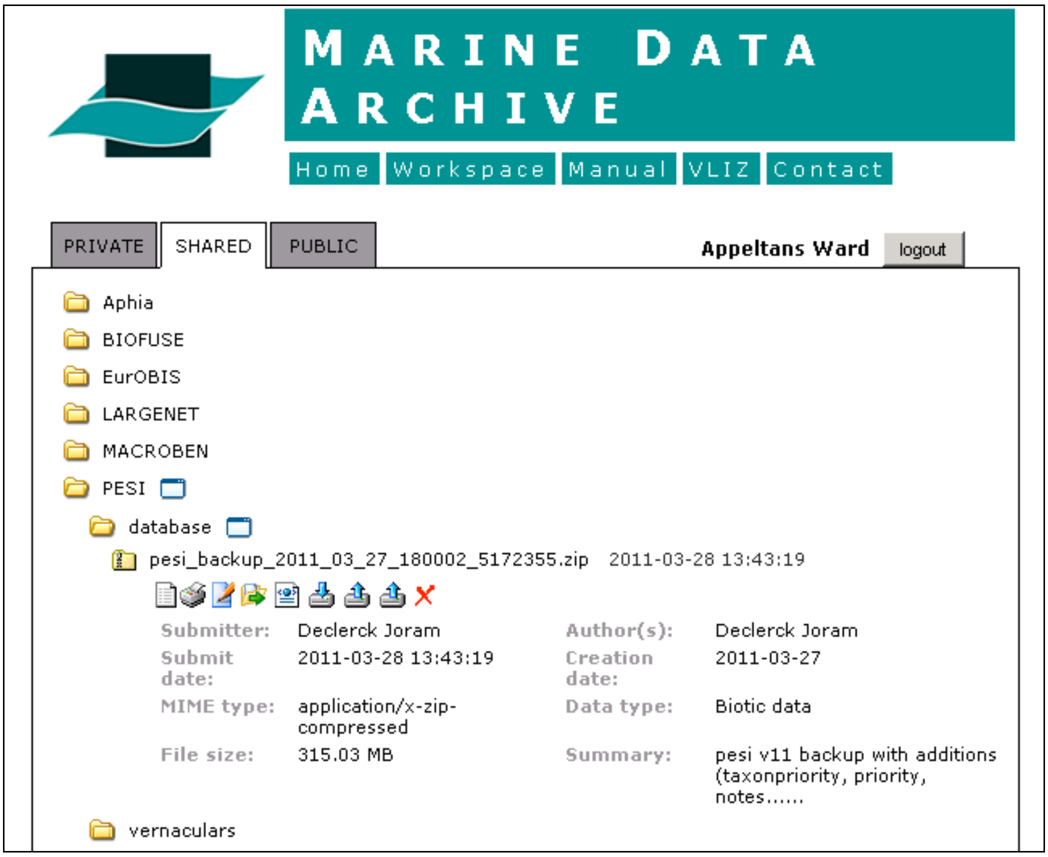

Technological Implementation Plan

For more detailed information on the technical implementations of the portal we would like to refer to our Technological Implementation Plan at:

http://www.eu-nomen.eu/pesi/index.php?option=com_remository&Itemid=56&func=startdown&id=362.

Configuration History			
**Version No.**	**Date**	**Changes made**	**Author**
0.1	31 March 2011	First version	WA
1.0	1 April 2011	Final version	WA, BV, JD, JK
1.1	4 April 2011	Final preparation for submission	YdJ
