# Supplementary material for: PESI - a taxonomic backbone for Europe
Source: Biodivers Data J. 2015 Sep 28;(3):e5848. doi: 10.3897/BDJ.3.e5848 (PMC4609752; doi:10.3897/BDJ.3.e5848)
Supplement: Supplementary material 25 — Towards a Common Agenda on Prioritised Taxa [file biodiversity_data_journal-3-e5848-s025.pdf]

Project no. 018340

Project acronym: EDIT

Project title: Toward the European Distributed Institute of Taxonomy

Instrument: Network of Excellence

Thematic Priority: Sub-Priority 1.1.6.3: "Global Change and Ecosystems"

C3.2.10 Towards a Common Agenda on Prioritised Taxa

Due date of component: Month 52
Actual submission date: Month 60

Start date of project: 01/03/2006 Duration: 5 years

Organisation name of lead contractor for this component: 5 UvA

Revision 1

Project co-funded by the European Commission within the Sixth Framework Programme (2002-2006)		
**Dissemination Level ("X" in the relevant box)**		
**PU**	Public	
**PP**	Restricted to other programme participants (including the Commission Services)	
**RE**	Restricted to a group specified by the consortium (including the Commission Services)	X
**CO**	Confidential, only for members of the consortium (including the Commission Services)	

Table of Content

INTRODUCTION **3**
EDIT'S PARTICIPATION IN THE SET UP OF A COMMON AGENDA ON PRIORITISED SPECIES 3
STATE OF THE ART IN EUROPEAN ENVIRONMENTAL POLICY 3
CATEGORIES OF TARGET SPECIES **4**
ENDANGERED SPECIES 4
INVASIVE ALIEN SPECIES 6
SPECIES RELATED TO HUMAN HEALTH 6
INDICATOR SPECIES FOR ENVIRONMENTAL (CLIMATE) CHANGE 7
TAXONOMIC RESEARCH & AUTHORITATIVE CHECKLISTS **8**
TAXONOMIC STANDARDS 8
TAXONOMIC RESEARCH 8
PROBLEMS ON TAXONOMIC (IN)CONGRUENCY 9
WORK PLAN AND RESULTS **10**
VALIDATION & POLICY AGREEMENTS 10
VALIDATION TOOLS & EXPERT EVALUATION 10
PROCESSING VALIDATION RESULTS 11
EXTENDED PESI WEB-PORTAL FUNCTIONS 13
FUTURE PLANS 14
CONFIGURATION HISTORY **15**
APPENDIX I: PRIORITISED SPECIES, DATA TYPES AND DATA VALUES **16**

Introduction

EDIT's participation in the set up of a common agenda on prioritised species

In the third project year EDIT joined the advancements on the development of a common agenda on managing prioritised taxa for Europe, meaning the set up of a common policy on those targeted species that play some kind of role within Europe's legislations on for instance habitat protection, nature conservation, pest control and health care. This not only includes activities on implementing authoritative taxonomic standards across Europe via EDIT WP3.2 (in close collaboration with PESI), but also prepares for the set up of agreements on preferencing the digitising of certain parts of European natural history collections (WP3.1), on involving the national Focal Point on implementing the European standards at the local level (WP3.2), on validating the CDM on the presence of relevant legislation annotation fields (WP5), on instructing the taxonomic work force how to anticipate on potential disagreements from a scientific perspective (WP2), and so on.

Especially the integrated approach showing a cross-collaboration between the EDIT initiated infrastructures and facilities, would be a nice proof of concept of EDIT's integrating actions and an important selling point towards the European biodiversity stakeholders' community.

State of the art in European Environmental policy

From 10 to 12 October 2007 the Sixth Ministerial Conference 'Environment for Europe' took place in Belgrade, Serbia. The Ministers adopted the so-called ***Belgrade Biodiversity Statement***, via which they reconfirmed Europe's political commitment to the European biodiversity 2010 target to halt the decline of biodiversity by the year 2010. The Ministers expressed their ***worries*** about the continuing decline of Europe's biodiversity and their willing to ***continue*** investing in realising the target in time.

Most relevant for this report is that the conservation of biological diversity remains at the core of EU environment policy, meaning that implementing the existing legislation in the field entails a significant and continuing investment of time and money. By the end of 2008 a mid-term report on the implementation of a so-called ***Biodiversity Action Plan*** will be produced. This report will measure progress towards the 2010 EU commitment to stop biodiversity loss within the EU and to significantly reduce loss worldwide.

The set-up and maintenance of reliable taxonomic information systems follows from international agreements. Nearly all countries are parties of the United Nations *Convention on International Trade in Endangered Species of Wild Fauna and Flora* (CITES), concluded at Washington in 1973, and the *Convention on Biological Diversity* (CBD) concluded at Rio de Janeiro in 1992. Furthermore, the member States of the European Community are bound by the *Council Directive 79/409/EEC of 2 April 1979 on the conservation of wild birds* (Birds Directive), the *Council Directive 92/43/EEC of 21 May 1992 on the conservation of natural habitats and of wild fauna and flora* (Habitats Directive) and the *Directive 2000/60/EC of the European Parliament and of*

the Council of 23 October 2000 establishing a framework for Community action in the field of water policy (Water Framework Directive). These conventions and directives form the basis of national legislations as well as a whole range of policy documents. Below an inventory of relevant prioritised species groupings and a link to their resources.

Categories of Target Species

Endangered species

When the last of a species dies out, the gene pool of the species is lost forever. To protect species, we must monitor their population levels. If one species dies out, those who rely on it in one way or another (i.e. protection or food) will also be affected. In light of this, humans aim to preserve genetic diversity and the diversity of species alive today. The ***IUCN Red List of Threatened Species*** provides taxonomic, conservation status and distribution information on taxa that have been globally evaluated using the IUCN Red List Categories and Criteria. This system is designed to determine the relative risk of extinction, and the main purpose of the IUCN Red List is to catalogue and highlight those taxa that are facing a higher risk of global extinction (i.e. those listed as Critically Endangered, Endangered and Vulnerable). The IUCN Red List[1], and its European equivalent EEC (*Europe's Endangered Species*)[2], also includes information on taxa that are categorized as Extinct or Extinct in the Wild; on taxa that cannot be evaluated because of insufficient information (i.e. are Data Deficient); and on taxa that are either close to meeting the threatened thresholds or that would be threatened were it not for an ongoing taxon-specific conservation programme.

Thousands of plant- and animal species occur in Europe only. These endemic species can be considered as Europe's specific contribution to global biodiversity. Following the IUCN Red Data Books, hundreds of these European species are threatened. These species merit special nature conservation efforts in Europe. However, only several hundreds of species (not covering all species that are threatened according to IUCN-criteria) are protected under European regulations.

In 2005, Alterra Wageningen published a report, entitled: "***Target species – Species of European concern***". The report proposes a Pan European Ecological Network and the establishment of a "target species" database[3] of European concern on which European legislation should be based in the near future. A target species list is included in the document.

The ***Convention on International Trade in Endangered Species*** (CITES)[4] one of the largest conservation agreements in existence regulates international trade in specimens of wild animals and plants and accords varying degrees of protection to more than 33,000 species of animals and

1 http://www.iucnredlist.org/
2 http://eur-lex.europa.eu/LexUriServ/LexUriServ.do?uri=CELEX:31992L0043:EN:HTML
3 http://www2.alterra.wur.nl/Internet////Modules/pub/PDFFiles/Alterrarapporten/AlterraRapport1119.pdf
4 http://www.cites.org

plants. These provisions not only constitute controls on international trade in specimens of these species, but are also the basis of a worldwide policy on protection of endangered species. The CBD[5] obliges parties, inter alia, to establish a system of protected areas, control the introduction of alien species, develop or maintain legislatory provisions for the protection of threatened species and adopt measures for the sustainable use of biological resources.

Besides ratifying and implementing the CITES provisions, the EU has set additional import restrictions in Regulation (EC) 338/97 and Regulation (EC) 865/2006.

For Europe the ***Habitats Directive***[6] is intended to help maintain biodiversity in the EC Member States by defining a common framework for the conservation of wild plants and animals and habitats of Community interest. Its main instrument is the establishment of a coherent network of special areas of conservation, known as *Natura 2000*. Annex II (Animal and plant species of Community interest) to the Directive lists the species whose conservation requires the designation of special areas of conservation. Species of community interest are endangered, vulnerable, rare or endemic. Some of them are defined as ‘priority’ species (in danger of disappearing). The occurrence of species of community interest is the major criterion for the designation of protected areas under Natura 2000. Finally, the Habitats Directive obliges the EC Member States to regulate the introduction of non-native species (article 22-b). Annex IV lists species that are in need of strict protection. The “priority” species are indicated with an *. This list that dates from 1992 was updated in 2006[7].

The ***Water Framework Directive***[8] commits European Union member states to achieve good qualitative and quantitative status of all water bodies (including marine waters along the shore) by 2015. The assessment of ecological water quality according to this directive involves the monitoring of the composition and abundance of aquatic flora and fauna, including plankton.

The ***Birds Directive***[9] contains measures for the protection of all species of naturally occurring birds in the wild state in the European territory of the EC member states (article 1). Annex I to the Birds Directive lists species that are subject to special conservation measures, while the species referred to in Annexes II and III enjoy lower levels of protection.

The international conventions and European directives refer to species in a direct way. In addition, organisms in the taxonomical sense are a major constituent of the wider concepts of *biological diversity* and *biological resources*.

5 http://www.cbd.int/
6 http://europa.eu/legislation_summaries/environment/nature_and_biodiversity/l28076_en.htm
7 http://eur-lex.europa.eu/LexUriServ/LexUriServ.do?uri=OJ:L:2006:363:0368:0408:EN:PDF
8 http://eur-lex.europa.eu/LexUriServ/LexUriServ.do?uri=CELEX:32000L0060:EN:NOT
9 http://eur-lex.europa.eu/LexUriServ/LexUriServ.do?uri=CELEX:31979L0409:EN:NOT

Invasive Alien Species

Invasive alien species threaten native biodiversity and cover all taxonomic groups from micro-organisms to animals and plants in all ecosystems. Biological invasions by non-native species are one of the greatest threats to the ecological and economic well being of the planet. Alien species can act as vectors for new diseases, alter ecosystem processes, change biodiversity, disrupt cultural landscapes, reduce the value of land and water for human activities and cause other socio-economic consequences. (DAISIE[10], GISD with 188 Invasive Species Databases[11], CIESM Atlas of Exotic Species[12]). ***Habitats Directive***: Article 22 of EC Directive 92/43/EEC (on the conservation of natural habitats and of wild flora and fauna) requires member states to "ensure that the deliberate introduction into the wild of any species which is not native to their territory is regulated so as not to prejudice natural habitats within their natural range or the wild native fauna and flora and, if they consider it necessary, prohibit such introduction."

Birds Directive: Article 11 of EC Directive 79/409/EEC states that "member states shall see that any introduction of species of bird which do not occur naturally in the wild state in the European territory of the member states does not prejudice the local flora and fauna."

The ***European Strategy on Invasive Alien Species*** (2003)[13] addresses constraints, faced by many European States in their efforts to tackle the problem. These constraints include:

- low public awareness and opposition to government intervention;
- shortage and inaccessibility of scientific information (for species identification, risk analysis, detection and mitigation techniques etc.);
- absence of clear and agreed priorities for action;
- ease of introduction and movement (e.g. through the post), inadequate inspection and quarantine;
- inadequate monitoring capacity;
- lack of effective emergency response measures;
- outdated or inadequate legislation;
- poor coordination between government agencies, States and other stakeholders.

In 2008, measures will be proposed to tackle invasive 'alien' species, which threaten the survival of native species of fauna and flora.

Species related to human health

Human medicines, biomedical research, the emergence and spread of infectious diseases, and the production of food, both on land and in the oceans, depend on biodiversity. The ***World Health Organization*** (WHO) mentioned in its report "*Biodiversity, Its Importance to Human Health*" (2003)[14] the growing concern about the health consequences of biodiversity loss and change. An important consequence for humans is the disruption of ecosystems that provide nature's goods and

10 http://www.europe-aliens.org/
11 http://www.issg.org/database/reference/index.asp
12 http://www.ciesm.org/online/atlas/intro.htm
13 http://www.jncc.gov.uk/page-4013
14 http://www.who.int/globalchange/ecosystems/biodiversity/en/index.html

services. Biodiversity loss also means that we are losing, before discovery, many of nature's chemicals and genes, of the kind that have already provided humankind with enormous health benefits. Some of the most endangered organisms on Earth—sharks, bears, primates, amphibians, cone snails, gymnosperms, and horseshoe crabs—contributed already to human medicine, and others are expected to do so if we do not drive them to extinction.

In its report, the WHO emphasises the high value of plants, animals and microbes to medical research. Plant-based systems continue to play an essential role in health care. Approximately 80% of the world's population in developing countries rely mainly on traditional medicines, mostly derived from plants, for their primary health care[15][16]. In addition to plants and microbes, there has been increasing attention paid to animals, both vertebrates and invertebrates, as sources for new medicines.

A very important area that is not usually considered is the use of natural compounds as agricultural agents or natural pesticides of many types that keep people healthy by maintaining adequate food supplies and preventing malnutrition. For example, one of the oldest and most successfully used plant products (from the 19th Century) is the powder from pyrethrum flowers, *Chrysanthemum cinerariaefolium*, originally native to the Dalmatian Mountains in Croatia.

Also, biodiversity can reduce pathogen transmission among hosts and therefore protect human health[17]. Infection rates decrease as species numbers increase. So-called “reservoir” species can easily become infected with a disease. When these species have to compete with other species for resources, they encounter each other less often, infection rate remains low and disease does not spread widely. (Sustaining Life, 2008[18]; Canary Database[19]).

Indicator species for environmental (climate) change

Biological indicator species are unique environmental indicators as they offer a signal of the biological condition in an ecosystem and are a warning system that pollution has entered the food web or other environmental changes have occurred. The term indicator species is a bit misleading, as indicators are often whole groups of flora/fauna types, which can be used to assess environmental condition. However, the so-called “keystone” species can represent a community. These species are capable of expressing characteristics that can indicate the state of the ecosystem they currently occupy. Indicator species can leave clues about the state of the ecosystem; they “indicate” the state of the local environment. In the aquatic environment, most indicator species are fish, invertebrates, periphyton and macrophytes. Amphibians are also common indicator species, as they absorb substances easily. Frogs are a very good example of indicator species. When frogs show deformities or are in bad shape, there is certainly a problem in a nearby body of water.

---

15 http://www.pfaf.org/
16 http://www.loc.gov/rr/scitech/tracer-bullets/medicplantstb.html
17 http://www.glopp.net/
18 http://chge.med.harvard.edu/programs/bio/index.html
19 http://canarydatabase.org/

A terrestrial example: *Biston betularia*, otherwise known as peppered moth, is a species that can adapt to polluted environments more suitably as a result of an adaptation changing the colour of them to suit their environment.

- A higher frequency of the light peppered moth would indicate that there is little pollution in the local environment
- A higher frequency of the dark peppered moth would indicated that there is high pollution in the local environment
- A decrease in light peppered moths' population may suggest that pollution is beginning to accumulate in the area.

In light of this, various species exhibit characteristics that give us insight into the local environment without having to study the local environment itself. In the case of the peppered moth being an indicator species, the presence of pollution (and dark moth) would indicate that additional a-biotic stress is being placed on the organisms that live in that polluted (and usually less favourable) environment. Using bio-indicators as an early warning of pollution or degradation in an ecosystem can help sustain critical resources. An example of a database on *Indicator Species* is BIOMAPS[20].

Taxonomic Research & Authoritative Checklists

Taxonomic standards

Implicit in these legal texts is the obligation to set up and maintain standardised taxonomic checklists of species. After all, the identification of rare, endemic, threatened or alien species referred to requires thorough knowledge of biological taxa and their natural distribution. In addition, the practical realization of protective measures is feasible only when taxonomic reference to species is standardized at the international level.

Whereas the legal texts focus on rare, endangered or invasive species, it is obvious that taxonomic knowledge of all species is imperative. Rare or endemic species can only be identified in the context of not so rare and more widespread species, just like invasive species can only be identified once their original area of distribution is known.

Taxonomic research

Indeed, the CBD and the Birds and Habitats Directives contain provisions for taxonomic research. Thus, article 7 of the CBD prescribes the identification and monitoring of (habitats and) species important for conservation and sustainable use, as well as the organization and maintenance of data derived from these processes. Likewise, the Birds Directive encourages research and other work required for the protection and management of bird species, such as national lists of endangered species (article 10). Finally, the Habitats Directive obliges the EC and the member States to encourage necessary research and scientific work that contributes towards ensuring bio-

[20] http://www.biologie.uni-hamburg.de/b-online/bonn/Biodiv_mapping/biomaps.htm

diversity, inter alia through the surveillance of the conservation status of all species of wild flora and fauna (article 18).

In the same vein, the European Commission's communication *Halting the Loss of Biodiversity by 2010 — and Beyond* [21] advocates strengthening research infrastructures, the science–policy interface and data interoperability for biodiversity under FP7 and national research programmes. Standardized and authoritative taxonomic resources will enhance our understanding of biodiversity and ecosystem services and enable the refinement of policy responses in the future.

Problems on taxonomic (in)congruency

The application of names to species and other biological taxa is not fully standardised due to a variety of reasons. Part of the incongruence results form variation in spelling or simple errors. Names may also change due to the advancement of taxonomic knowledge, and are not updated in all databases at the same time. Only in some cases different names reflect real disagreement of specialists. In addition to the taxonomic data per se, also data on occurrence and for instance legal status need regular updating.

When a protected species is mentioned as such in for instance the Habitats Directive or a piece of national legislation, lack of international taxonomic standardisation hampers the implementation of the regulations. This is best illustrated by the following examples:

> The butterfly *Graellsia isabellae* (Graells, 1849) is mentioned in Annex II of the Habitats Directive, but its current valid name in Fauna Europaea is *Actias isabellae* (Graells, 1849). It occurs as *Actias* in the French national checklist INPN, and as *Graellsia* in Fauna Iberica.

Such incongruencies highlight the need for a collaborative effort on scrutinising the European prioritised species lists compared to the European authoritative taxonomic standards provided by the pan-European checklists *Fauna Europaea*[22], ERMS[23] and *Euro+Med PlantBase*[24] joined in the PESI project[25].

21 http://eur-lex.europa.eu/smartapi/cgi/sga_doc?smartapi!celexplus!prod!DocNumber&lg=en&type_doc=COMfinal&an_doc=2006&nu_doc=216
22 http://www.faunaeur.org
23 http://www.marbef.org/data/erms.php
24 http://www.emplantbase.org
25 http://www.eu-nomen.eu

Work plan and Results

Validation & Policy agreements

Uncertainty of species identity is currently hampering the accurate implementation of the above mentioned regulations. EDIT WP3.2 in collaboration with the PESI project aims at cross-validation of all European and national or regional taxonomic checklists, as well as the relevant Global Species Databases. To support the proper operation of European regulations on biodiversity, priority will be given to those taxon names that are explicitly mentioned in legislative texts, with highest priority for international regulations. For this reason, standardisation of taxonomic reference to the species mentioned therein is a key deliverable to be delivered before all validation is finished. Validation and adjustment of these taxon names will be done in close cooperation with pertinent international, European and national agencies, such as the IUCN, EPPO, the European Topic Centre on Biodiversity (EEA-ETC), and others stakeholders, who urgently need this information for the execution of their tasks. Appendix I provide an overview of prioritised species lists and values taken into account in the validation process.

Validation tools & expert evaluation

The validation of prioritised species lists compared to the pan-European checklists is taking care about using the PESI web-portal validation services, including the Taxon Match Tool (Figs 1 & 2).

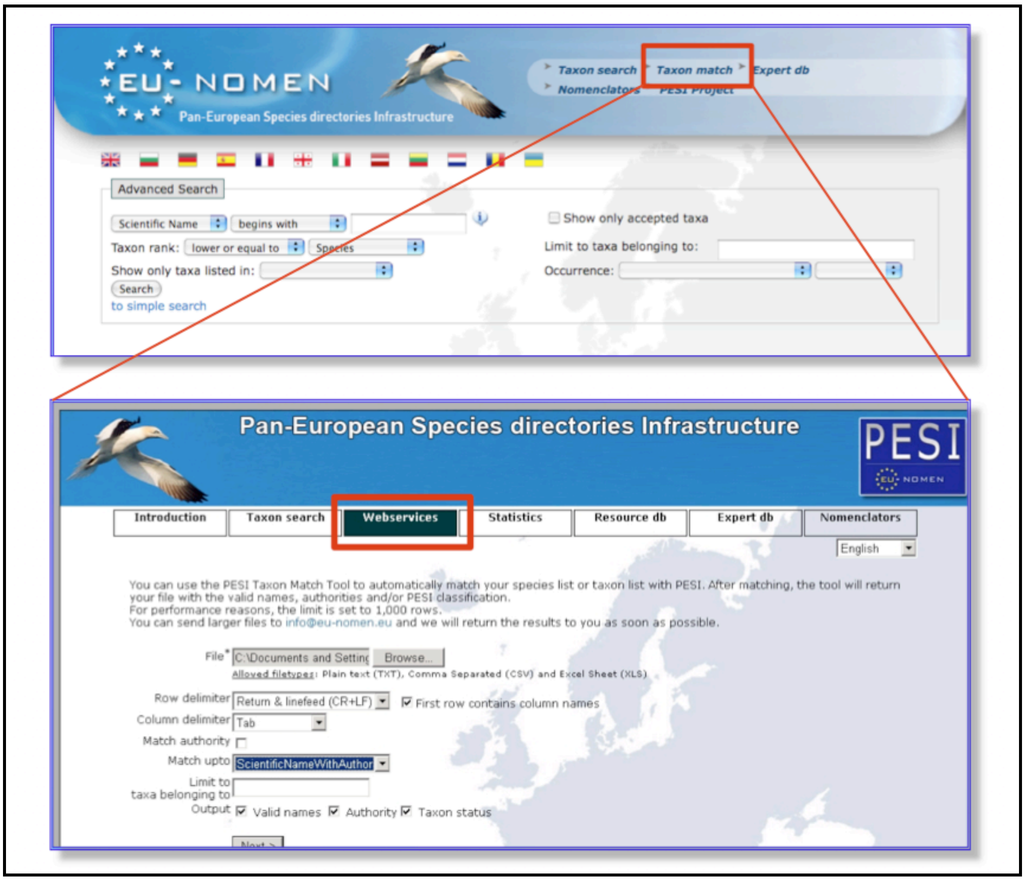

Figure 1: The PESI Taxon Match Tool as one of the web-portal services.

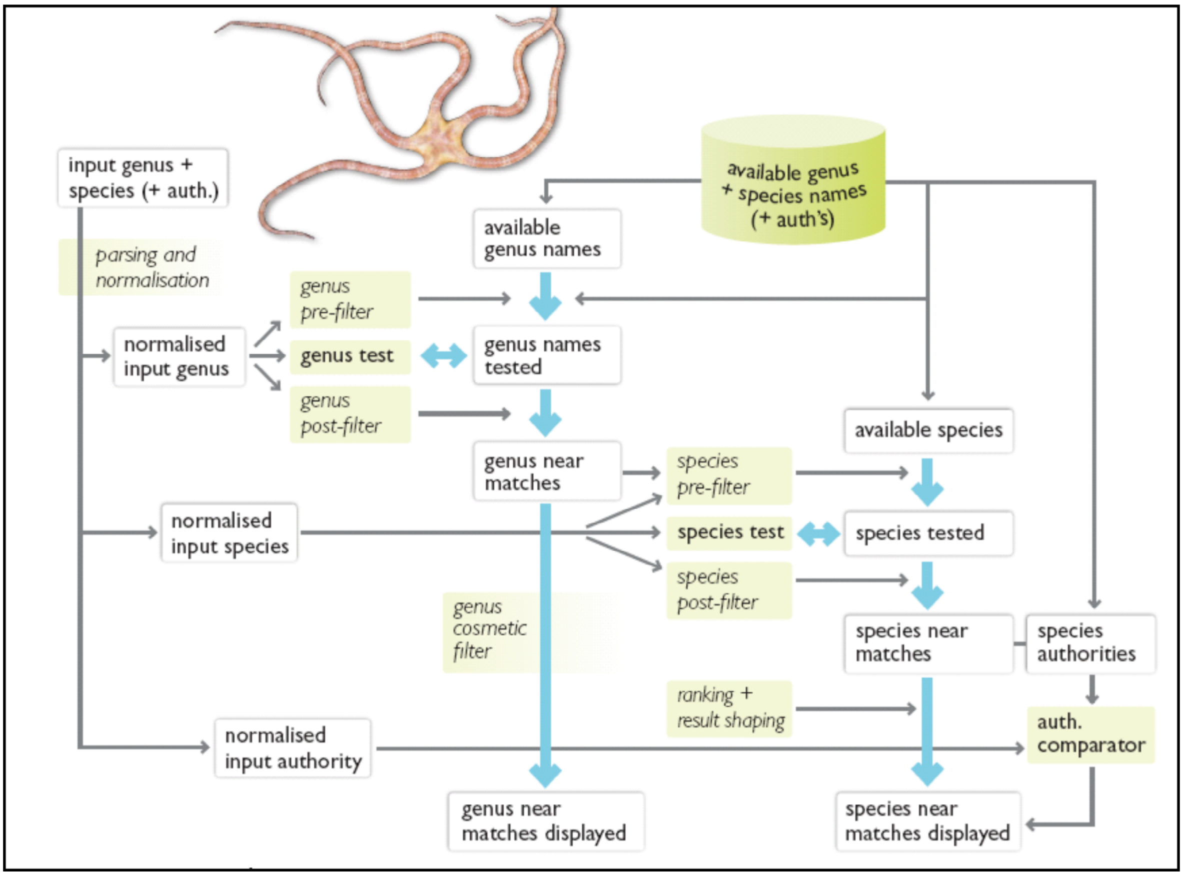

Figure 2: A schematic overview of the Taxon Match Tool operation work flow (©VLIZ).

Processing validation results

The resulting output files of the validation process, including a mapping of both lists showing the found overlap and discrepancies (see Figs 3 & 4) are distributed to the SMEBD[26] experts for further evaluation. Commentary of the SMEBD experts is processed advancing the pan-European checklist species entries to optimise the cross-referencing with relevant biodiversity information services and support the further taxonomic standardisation of prioritised species names in Europe. In addition relevant annotations are made to inform pan-European checklist users about the status of species names within the EC legislation documents (Fig. 5).

	Exact match	Different concept (synoym/infra rank)	No match / gap
Arthropods	105	12	5
Amphibians	45	10	0
Fish	56	4	15
Mammals	72	1	7
Molluscs	36	0	8
Reptiles	74	10	9
Others	3	0	0

Figure 3: Results of the comparative analysis of the European Habitat directives and Fauna Europaea version 1.3.

26 http://www.smebd.eu/

	A	B	C
1	Directive	Birds_Directive_Species_name	remarks
4	Birds Directive	Accipiter gentilis arrigonii	subspecies of a species listed in FaEu
5	Birds Directive	Accipiter gentilis strigonii	wrong spelling of arrigonii
7	Birds Directive	Accipiter nisus granti	subspecies of a species listed in FaEu
25	Birds Directive	Alectoris graeca graeca	subspecies of a species listed in FaEu
26	Birds Directive	Alectoris graeca saxatilis	subspecies of a species listed in FaEu
27	Birds Directive	Alectoris graeca whitakeri	subspecies of a species listed in FaEu
40	Birds Directive	Anser albifrons albifrons	subspecies of a species listed in FaEu
41	Birds Directive	Anser albifrons flavirostris	subspecies of a species listed in FaEu
48	Birds Directive	Anthus berthelotti	wrong spelling of Anthus berthelotii
51	Birds Directive	Anthus novaeseelandiae	now Anthus richardi, split off from Anthus novaeseelandiae
58	Birds Directive	Apus melba	Genus differs at present: in FaEu Tachymarptis melba
64	Birds Directive	Aquila heliaca adalberti	now Aquila adalberti, split off from Aquila heliaca
103	Birds Directive	Calidris alpina schinzii	subspecies of a species listed in FaEu
117	Birds Directive	Carduelis chloris	Genus differs at present: in FaEu Chloris chloris
126	Birds Directive	Certhia brachydactyla dorotheae	subspecies of a species listed in FaEu
160	Birds Directive	Columba palumbus azorica	subspecies of a species listed in FaEu
171	Birds Directive	Cyanopica cyana	correct gender: Cyanopica cyanus
172	Birds Directive	Cygnus columbianus bewickii	now Cygnus bewickii, split off from Cygnus columbianus
178	Birds Directive	Dendrocopos major canariensis	subspecies of a species listed in FaEu
179	Birds Directive	Dendrocopos major thanneri	subspecies of a species listed in FaEu
184	Birds Directive	Egretta alba	Genus differs at present: in FaEu Casmerodius albus
194	Birds Directive	Emberiza leucocephala	correct gender: Emberiza leucocephalos
220	Birds Directive	Fringilla coelebs ombriosa	subspecies of a species listed in FaEu
227	Birds Directive	Galerida malabarica	now Galerida theklae, split off from Galerida malabarica
245	Birds Directive	Haematopus moquini	now Haematopus meadewaldoi, split off from Haematopus r
254	Birds Directive	Hippolais pallida	Genus differs at present: in FaEu Iduna pallida
264	Birds Directive	Ketupa zeylonensis	Restricted to SE Turkey and thus not in FaEu
266	Birds Directive	Lagopus lagopus hibernicus	subspecies of a species listed in FaEu
267	Birds Directive	Lagopus lagopus lagopus	subspecies of a species listed in FaEu
268	Birds Directive	Lagopus lagopus scoticus	subspecies of a species listed in FaEu
270	Birds Directive	Lagopus mutus helveticus	subspecies of a species listed in FaEu
271	Birds Directive	Lagopus mutus pyrenaicus	subspecies of a species listed in FaEu
314	Birds Directive	Meleagris gallopavo	Introduced, population not self-sustaining, should not be lis
319	Birds Directive	Miliaria calandra	Genus differs at present: in FaEu Emberiza calandra
327	Birds Directive	Motacilla finere	wrong spelling of Motacilla cinerea
329	Birds Directive	Muscicapa latirostris	name latirostris pre-occupied, correct name Muscicapa dau
337	Birds Directive	Nyctea scandiaca	Genus differs at present: in FaEu Bubo scandiaca
342	Birds Directive	Oenanthe finischii	Wring spelling of Oenanthe finschii
351	Birds Directive	Otus brucei	Restricted to SE Turkey and thus not in FaEu
358	Birds Directive	Parus ater cypriotes	subspecies of a species listed in FaEu
374	Birds Directive	Perdix perdix hispaniensis	subspecies of a species listed in FaEu
375	Birds Directive	Perdix perdix italica	subspecies of a species listed in FaEu
380	Birds Directive	Phalacrocorax aristotelis desmarestii	subspecies of a species listed in FaEu
382	Birds Directive	Phalacrocorax carbo sinensis	subspecies of a species listed in FaEu
386	Birds Directive	Phalaropus tricolor	Genus differs at present: in FaEu Steganopus tricolor
389	Birds Directive	Phoenicopterus ruber	now Phoenicopterus roseus, split off from Phoenicopterus r
392	Birds Directive	Phoenicurus torquata	Unidentified, unknown combination
410	Birds Directive	Podiceps caspicus	name caspicus pre-occupied, correct name Podiceps auritus
449	Birds Directive	Rhodopechys githaginea	Genus differs at present: in FaEu Bucanetes githagineus
454	Birds Directive	Saxicola torquata	now Saxicola rubicola and Saxicola maurus, both split off fro
457	Birds Directive	Serinus citrinella	Genus differs at present: in FaEu Carduelis citrinella
501	Birds Directive	Sylvia sarda balearica	subspecies of a species listed in FaEu

Figure 4: Exemplar of a validation file, comparing the Bird Directive and Fauna Europaea data, showing overlap and discrepancies.

At a later stage the results of the comparative analyses will be shared with the relevant stakeholders for further assessment. For instance the European Habitat/Bird directives will be evaluated, discussing the scientific and political complications regarding the different taxonomic concepts used, in collaboration with the European Topic Center in Paris.

As an example, nearly all mismatching found in the comparative analysis of the EU Bird directive and Fauna Europaea concerned subspecies (Fig. 4). So far, subspecies have principally been excluded from Fauna Europaea for birds, because in most cases their scientific status is ambiguous or doubtful, reflecting the overemphasis of certain local variations and a political prejudice. However, as an exception, to allow an optimal mapping of both lists, subspecies have been included in later versions of Fauna Europaea for these particular cases.

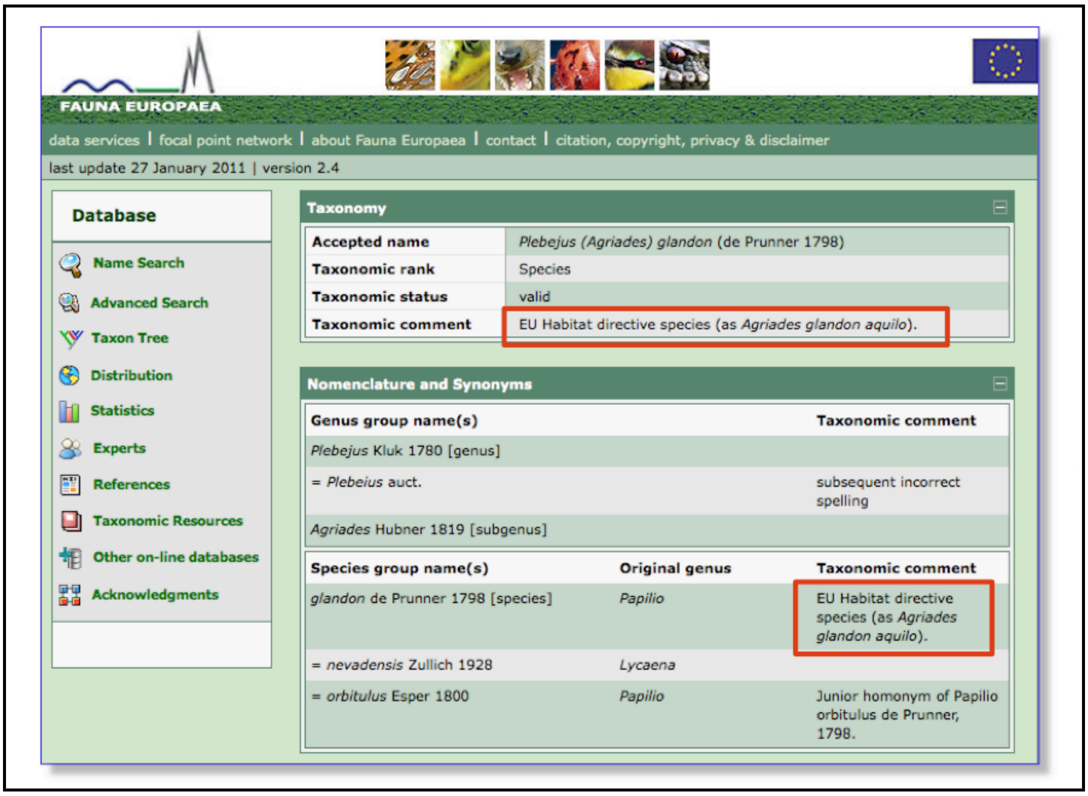

Figure 5: Exemplar of species details in Fauna Europaea, showing EU Habitat directive status annotations.

Extended PESI web-portal functions

The cross-referencing of prioritised species lists with the pan-European checklists resulted in a set of advanced PESI web portal functions. This consists of (1) search routines on selected prioritised species lists, for instance "all bird species listed on the IUCN redlist and present in the Netherlands" (see Fig. 6) and (2) annotations on the legislation status in the species details, including direct links to the relevant prioritised species web-pages (see Fig. 7).

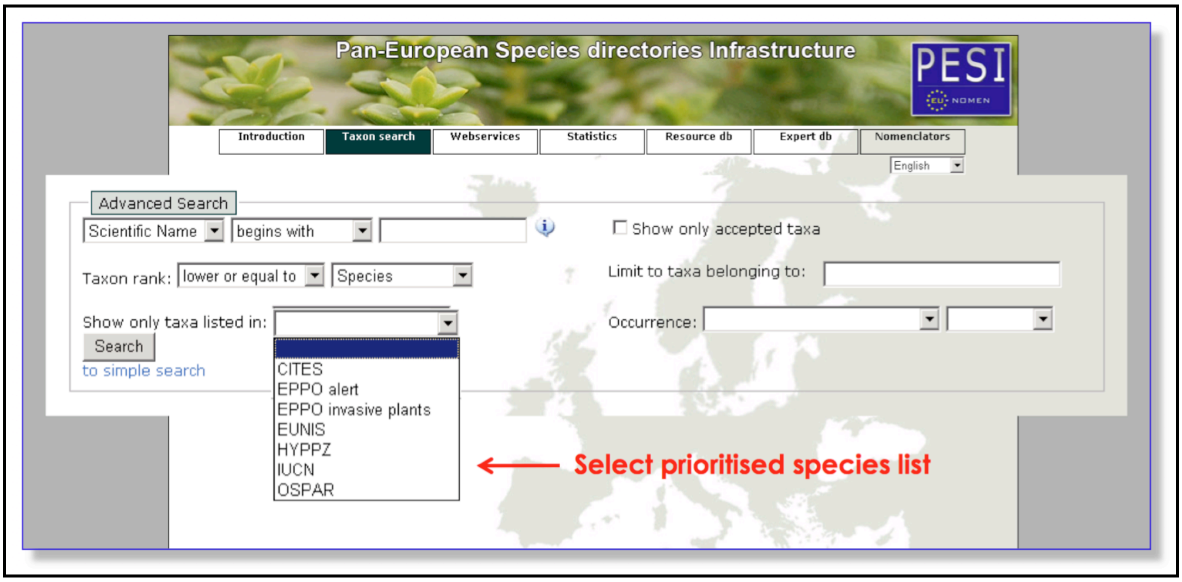

Figure 6: Search routines on selected prioritised species lists in the PESI web-portal

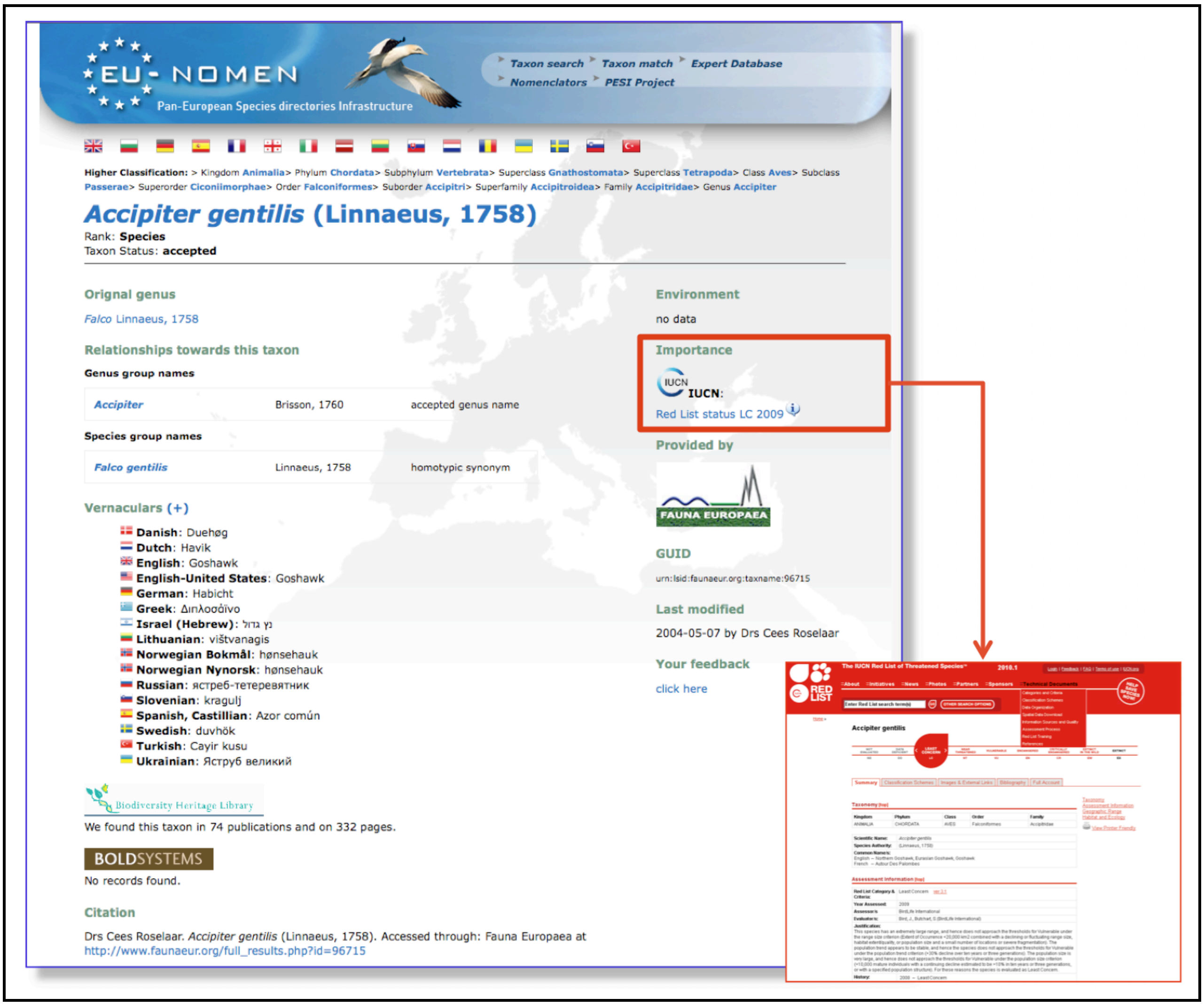

Figure 7: Exemplar of species details in the PESI web-portal, showing IUCN status annotation and cross-reference.

Future plans

Accurate national checklists are indispensible for the application of international, European and national regulations concerning organisms occurring on the national territory, like the protection of endemic species. Therefore, apart from sustaining the established routines on validating the European prioritised species lists, the implementation of the results within the PESI web-portal and the liaison with the respective legislation bodies, a further outreach towards the individual European countries to allow a proper application at the national level, is projected. For this purpose strong emphasis will be put on the collaboration with the network of National Focal Points.

Configuration History			
Version No.	Date	Changes made	Author
0.1	3 October 2008	Initial draft introduction	LB
0.2	15 December 2008	Collecting prioritised species lists for cross-validation	JK
0.3	13 September 2010	Performing cross-validation	WA
0.4	26 November 2010	Evaluating validation results by SMEBD experts	YdJ
0.5	19 February 2011	Draft sent to WP leader	YdJ
1.0	26 February 2011	Final version submitted	YdJ

Appendix I: Prioritised Species, Data types and Data values

Species lists	URL SPECIES LISTS	No. of species	Data Type	Value 1	Value 2	Value 3
Species in EU-Habitat Directive	http://eunis.eea.europa.eu/species-threat-international-result.jsp?&pageSize=300&idGroup=-1&idCountry=80&idConservation=-1&showGroup=true&showVernacularNames=true&sort=1&ascendency=1 SEE ALSO FILE: eunis_hd_spec _Unique_recs.xls	ca. 1400	Legal protection		Strict protection (Annex IV)	
Species in EU-Birds Directive	http://eunis.eea.europa.eu/species-threat-international-result.jsp?&pageSize=300&idGroup=-1&idCountry=80&idConservation=-1&showGroup=true&showVernacularNames=true&sort=1&ascendency=1	ca. 300	Legal protection	Vulnerable to habitat changes (Annex I)	In danger of extinction (Annex I)	Rare (small populations or restricted distributions, Annex I)
IUCN Red List for Europe	http://www.iucnredlist.org/apps/redlist/search/link/4b012499-c2161c66	ca. 2400	Threatened	not evaluated (NE)	Data Deficient (DD)	Least concern (LC)
IUCN Red List for European marine species	http://www.iucnredlist.org/apps/redlist/search/link/4b012aa8-8ab8e83d	ca. 650	Threatened	not evaluated (NE)	Data Deficient (DD)	Least concern (LC)
Marine species from OSPAR Convention	SEE EXCEL FILE OSPAR SPECIES	ca. 50	Legal protection	threatened	declined	
IUCN Red List for European Native species	http://www.iucnredlist.org/apps/redlist/search/link/4b01380b-c2dc15b8	2273	Endemism (Species restricted to, or characteristic for, Europe)	Endangered (EN)	Critically endangered (CR)	Extinct in the wild (EW)
EPPO Pest species alert lists	http://www.eppo.org/QUARANTINE/Alert_List/alert_list.htm	ca. 300	Regulated Pests	A1 (still absent in Europe)	A2 (locally present in Europe)	
EPPO Invasive Alien Plants list	http://www.eppo.org/QUARANTINE/ias_plants.htm	60	Threat to biodiversity	important threat	risky1 (limited EU distribution)	risky2 (still absent in Europe)
HYPPZ (INRA) Animal Pest Species Western Europe	http://www.inra.fr/internet/Produits/HYPPZ/species.htm	376	Crop devastating animals			
Species in EU Wildlife Trade Regulation (from CITES)	http://www.cites.org/eng/app/appendices.shtml	ca 700	Legal trade suspension (introduction in EU by trade)	I (species threatened with extinction, no trade allowed)	II (controlled trade)	III (protected in at least one country, controlled trade)
