# Supplementary material for: PESI - a taxonomic backbone for Europe
Source: Biodivers Data J. 2015 Sep 28;(3):e5848. doi: 10.3897/BDJ.3.e5848 (PMC4609752; doi:10.3897/BDJ.3.e5848)
Supplement: Supplementary material 26 — Applying taxonomy to organise and deliver publications to biologists [file biodiversity_data_journal-3-e5848-s026.pdf]

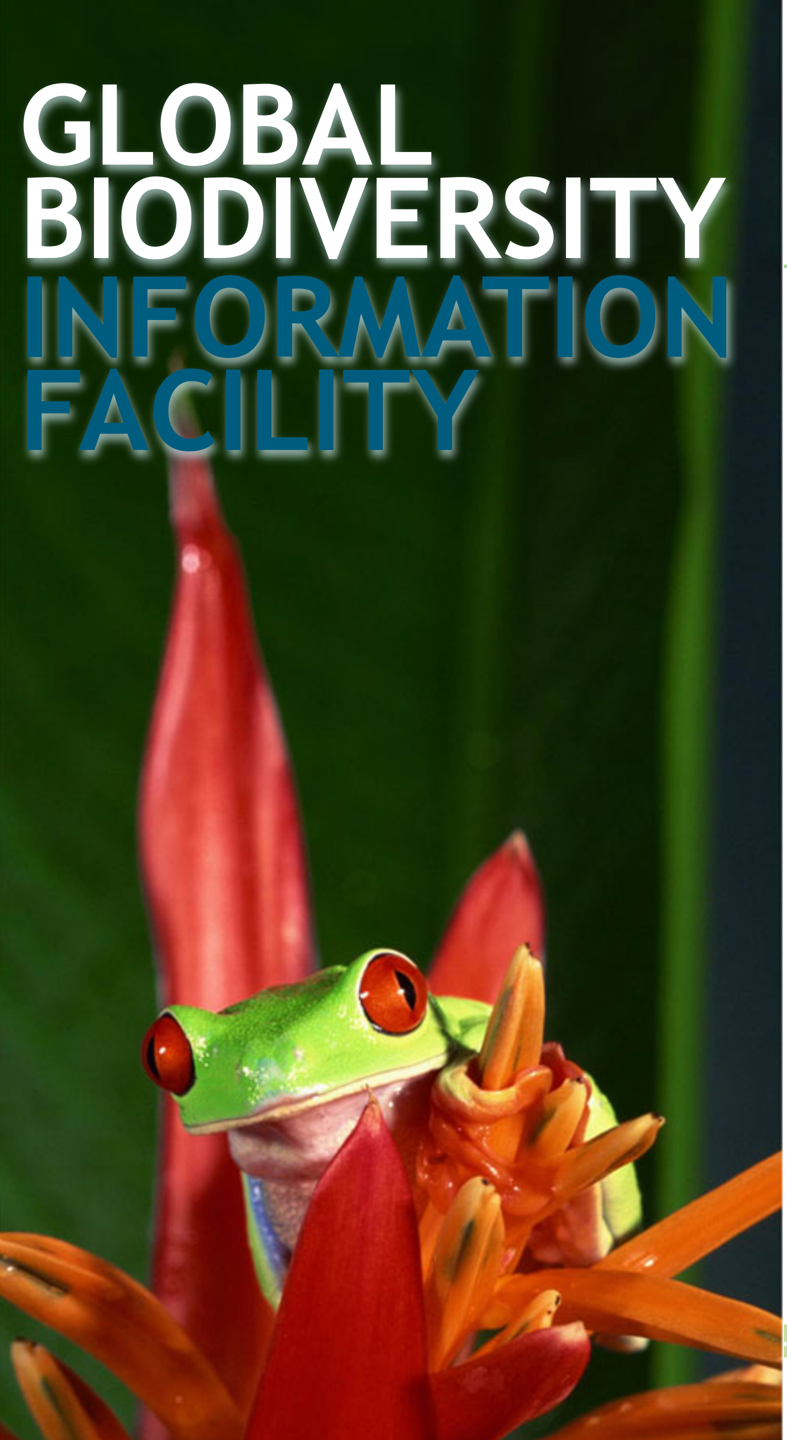

GBIF

PESI Publishers Meeting

Applying taxonomy to organise and deliver publications to biologists

David Remsen, Senior Programme Officer, GBIF
15 July Amsterdam

Scope of this presentation

- Promote dissemination and accessibility of scholarly publications
- Increase impact through integrated activity

RSS: Really Simple Syndication

http://www.bioone.org/toc/cope/current

http://www.bioone.org/action/showFeed?type=etoc&feed=rss&jc=cope

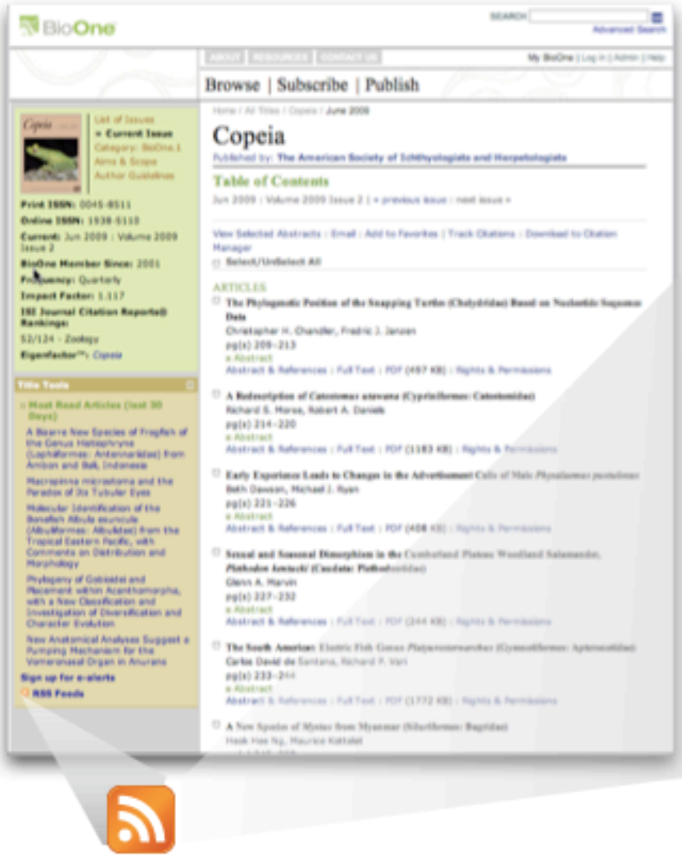

RSS Feed

<Channel>

Metadata about the feed
Journal Title
Description
Links Image (Current Issue)

Item (Article) **Article Title** **Link**
Description (Abstract) **GUID**

Item (Article)

Item (Article)

Item (Article)

Item (Article)

Item (Article)

</Channel>

Discovery of articles by publication

Really Simple

GBIF


```
<?xml version="1.0" encoding="UTF-8"?>
<rss version="2.0">
  <channel>
    <title>Animal Conservation - Current Issue</title>
    <link>http://journals.cambridge.org/action/displayJournal?jid=ANI</link>
    <description>Animal Conservation, Volume 8 Issue 04&lt;br /&gt;&lt;br /&gt;&lt;br /&gt;&lt;table border='0'&gt;&lt;tr&gt;&lt;td&gt;
    <image>
      <title>Journals Cambridge Online</title>
      <url>http://journals.cambridge.org/images/logo_6699CC_large.gif</url>
      <link>http://journals.cambridge.org</link>
      <description>Journals Cambridge Online</description>
    </image>
    <item>
      <title>Volume 8 Issue 04</title>
      <link>http://journals.cambridge.org/action/displayIssue?jid=ANI&amp;volumeId=8&amp;issueId=04</link>
      <description>Animal Conservation, Volume 8 Issue 04&lt;br /&gt;&lt;br /&gt;&lt;br /&gt;&lt;table border='0'&gt;&lt;tr&gt;&lt;td&
      <pubDate>Tue, 01 Nov 2005 00:00:00 GMT</pubDate>
      <guid>http://journals.cambridge.org/action/displayIssue?jid=ANI&amp;volumeId=8&amp;issueId=04</guid>
    </item>
    <item>
      <title>Translocation as a conservation tool: site fidelity and movement of repatriated gopher tortoises ( Gopherus polyphemus )</
      <link>http://journals.cambridge.org/action/displayAbstract?fromPage=online&amp;aid=349289</link>
      <description>Research Articles&lt;br /&gt;Tracey D. Tuberville, Erin E. Clark, Kurt A. Buhlmann, J. Whitfield Gibbons,  &lt;br /&
      <guid>http://journals.cambridge.org/action/displayAbstract?fromPage=online&amp;aid=349289</guid>
    </item>
    <item>
      <title>Modelling impacts of long-line fishing: what are the effects of pair-bond disruption and sex-biased mortality on albatross
      <link>http://journals.cambridge.org/action/displayAbstract?fromPage=online&amp;aid=349287</link>
      <description>Research Articles&lt;br /&gt;Michael S. L. Mills, Peter G. Ryan,  &lt;br /&gt;&lt;a href='http://journals.cambridge.
      <guid>http://journals.cambridge.org/action/displayAbstract?fromPage=online&amp;aid=349287</guid>
    </item>
    <item>
      <title>The response of tree squirrels to fragmentation: a review and synthesis</title>
      <link>http://journals.cambridge.org/action/displayAbstract?fromPage=online&amp;aid=349293</link>
      <description>Research Articles&lt;br /&gt;John L. Koprowski,  &lt;br /&gt;&lt;a href='http://journals.cambridge.org/jid_ANI'&gt;
      <guid>http://journals.cambridge.org/action/displayAbstract?fromPage=online&amp;aid=349293</guid>
    </item>
    <item>
      <title>Population genetic structure and conservation of Asian elephants ( Elephas maximus ) across India</title>
      <link>http://journals.cambridge.org/action/displayAbstract?fromPage=online&amp;aid=349295</link>
      <description>Research Articles&lt;br /&gt;T. N. C. Vidya, P. Fernando, D. J. Melnick, R. Sukumar,  &lt;br /&gt;&lt;a href='http:/
      <guid>http://journals.cambridge.org/action/displayAbstract?fromPage=online&amp;aid=349295</guid>
    </item>
    <item>
```


JOURNAL

VOLUME

ARTICLE

Index of 1000+ RSS feeds

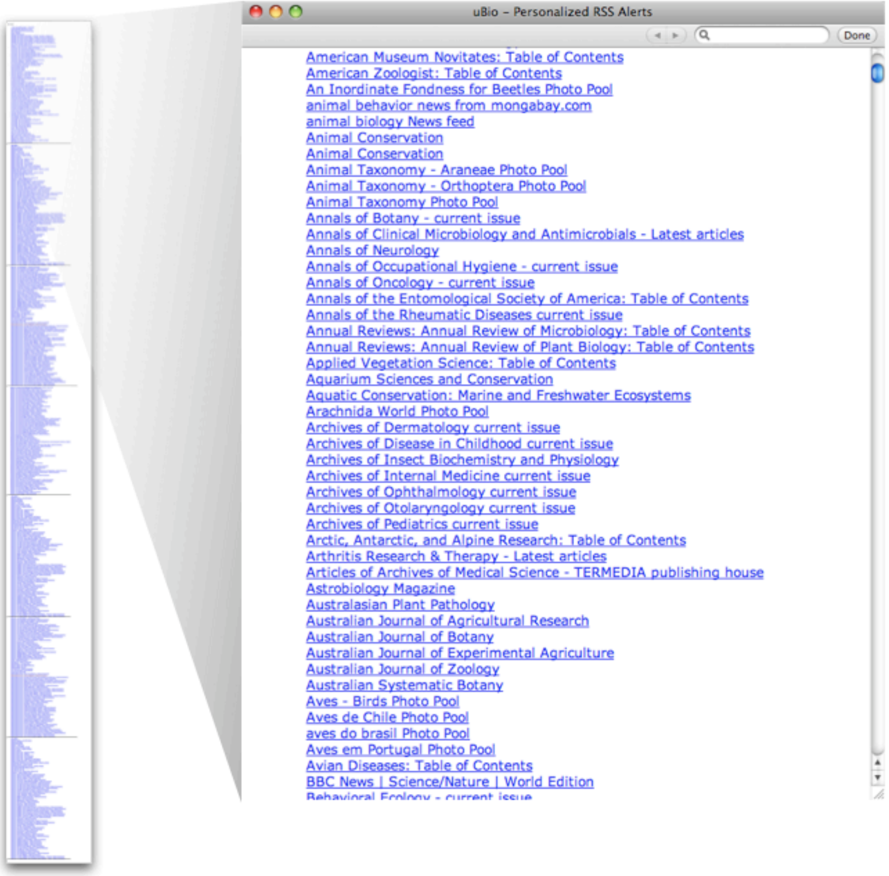

- uBio Work catalogued RSS feeds
- Scholarly Publications
- Few Scholarly Blogs

Scientific Names embedded in RSS Text

GBIF

Revised diagnosis and redescription of *Apistobuthus susanae* (Scorpiones, Buthidae)
Shahrokh Navidpour, Graeme Lowe
pg(s) 45–59
⊞ Abstract
Abstract & References : Full Text : PDF (3791 KB) : Rights & Permissions

On the endemic Sri Lankan genus *Pettalus* (Opiliones, Cyphophthalmi, Pettalidae) with a description of a new species and a discussion of its diversity
Prashant Sharma, Indika Karunarathna, Gonzalo Giribet
pg(s) 60–67
⊞ Abstract
Abstract & References : Full Text : PDF (2957 KB) : Rights & Permissions

Characterization of the green iridescence on the chelicerae of the tube web spider, *Segestria florentina* (Rossi 1790) (Araneae, Segestriidae)
A. L. Ingram, A. D. Ball, A. R. Parker, O. Deparis, J. Boulenguez, S. Berthier
pg(s) 68–71
⊞ Abstract
Abstract & References : Full Text : PDF (542 KB) : Rights & Permissions

Post-reproductive changes in female crab spiders (*Misumena vatia*) exposed to a rich prey source

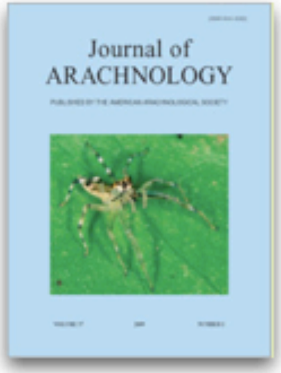

Scientific Names are links to taxa

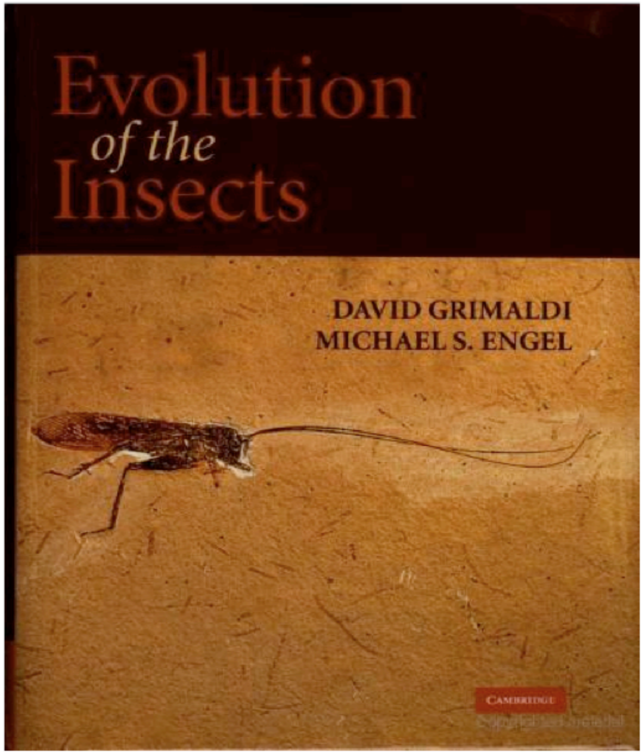

All *accumulated information* of a species is tied to a scientific name, a name that serves as a link between what has been learned in the past and what we today add to the body of knowledge.

- Grimaldi & Engel, 2005, Evolution of the Insects

Nomenclature is what makes literature taxonomic literature

TaxonFinder: Finds scientific names in RSS Text

GBIF

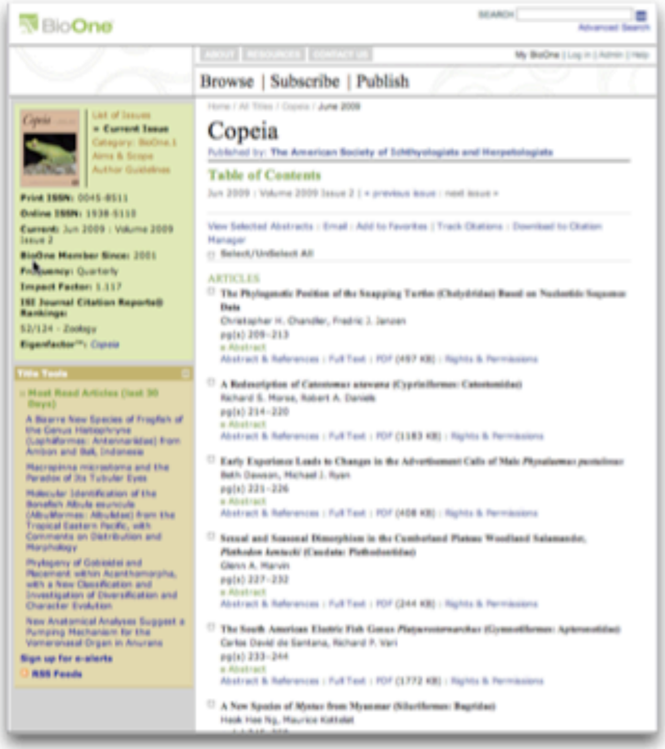

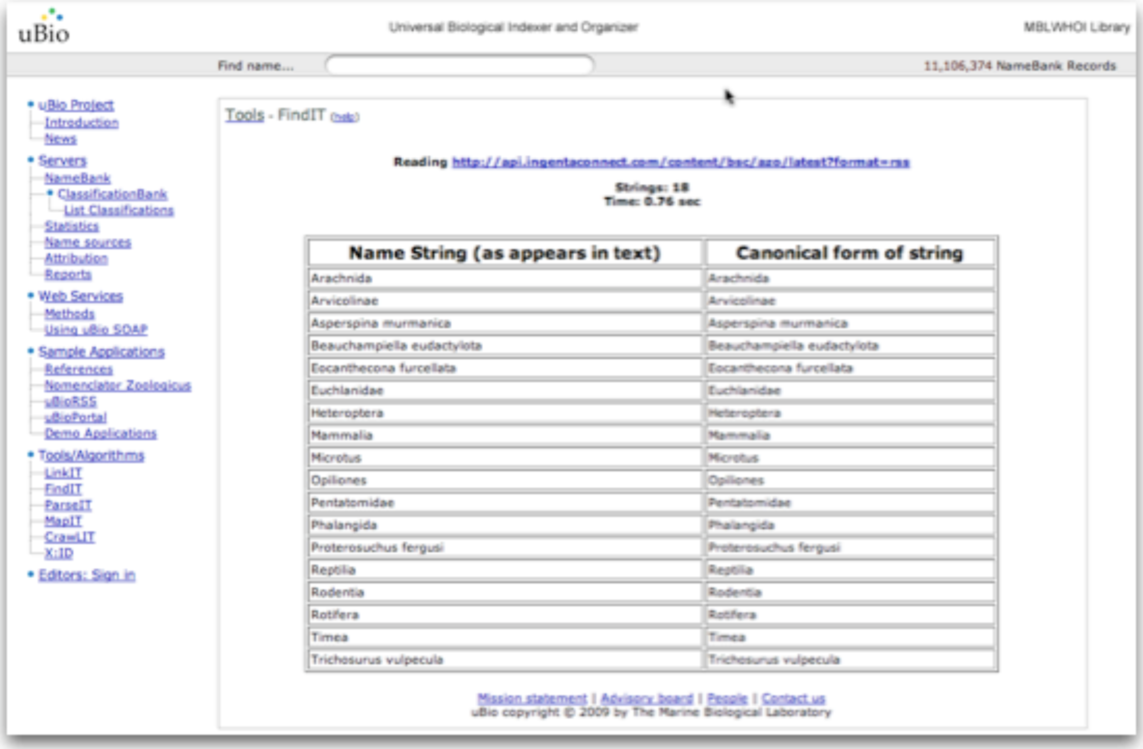

Name String (as appears in text)	Canonical form of string
Arachnida	Arachnida
Arvicolinae	Arvicolinae
Asperspina murmanica	Asperspina murmanica
Beauchampiella eudactylota	Beauchampiella eudactylota
Eocanthecona furcellata	Eocanthecona furcellata
Euchlanidae	Euchlanidae
Heteroptera	Heteroptera
Mammalia	Mammalia
Microtus	Microtus
Opiliones	Opiliones
Pentatomidae	Pentatomidae
Phalangida	Phalangida
Proterosuchus fergusi	Proterosuchus fergusi
Reptilia	Reptilia
Rodentia	Rodentia
Rotifera	Rotifera
Timea	Timea
Trichosurus vulpecula	Trichosurus vulpecula

uBioRSS : Process Feeds

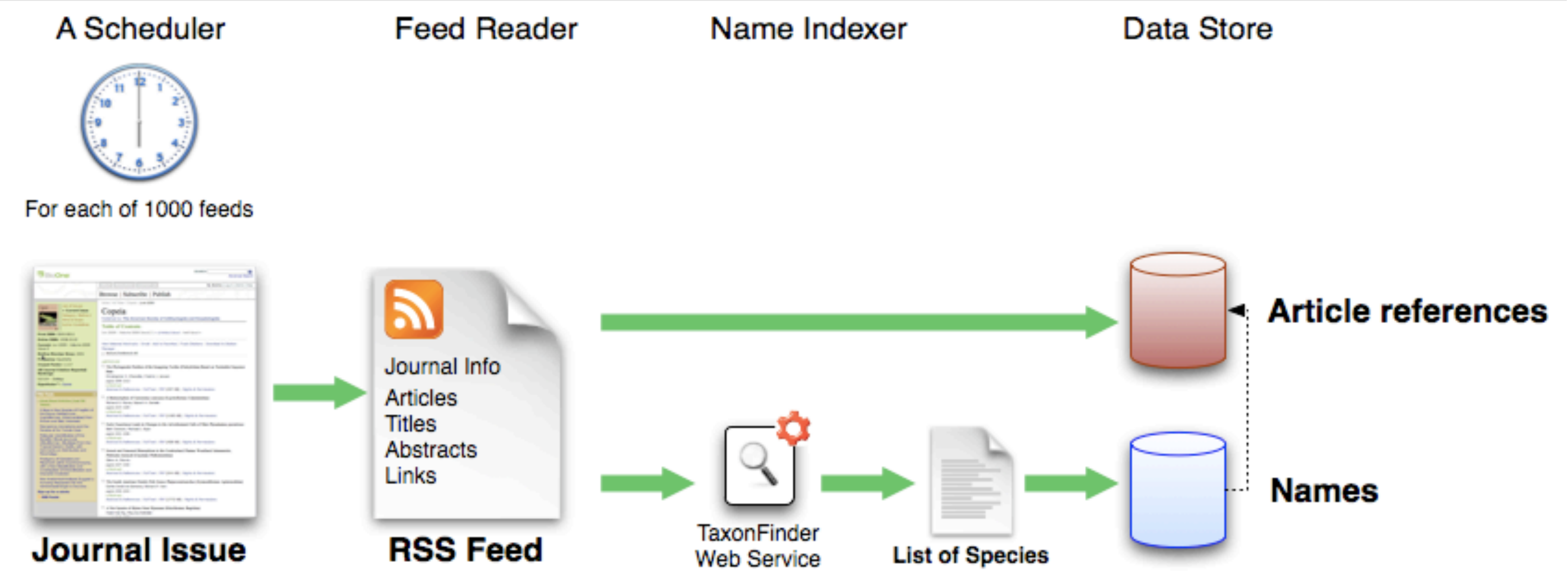

uBioRSS : Access Taxonomic Checklists

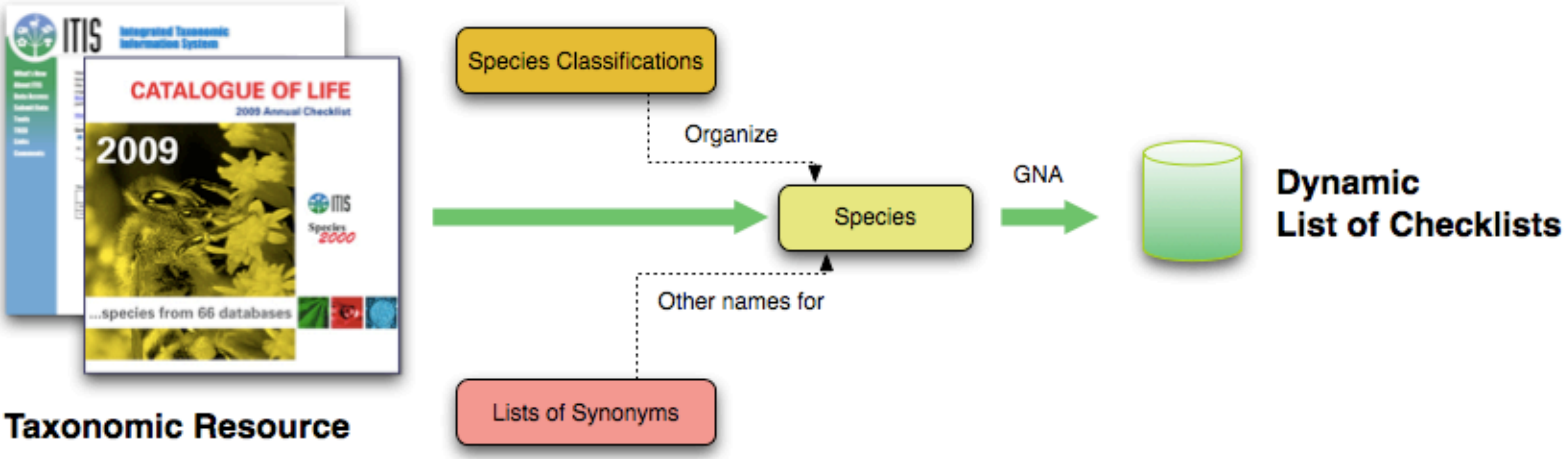

Taxonomic Resources provide the dictionaries that make TaxonFinder work

uBioRSS : Access Taxonomic Checklists

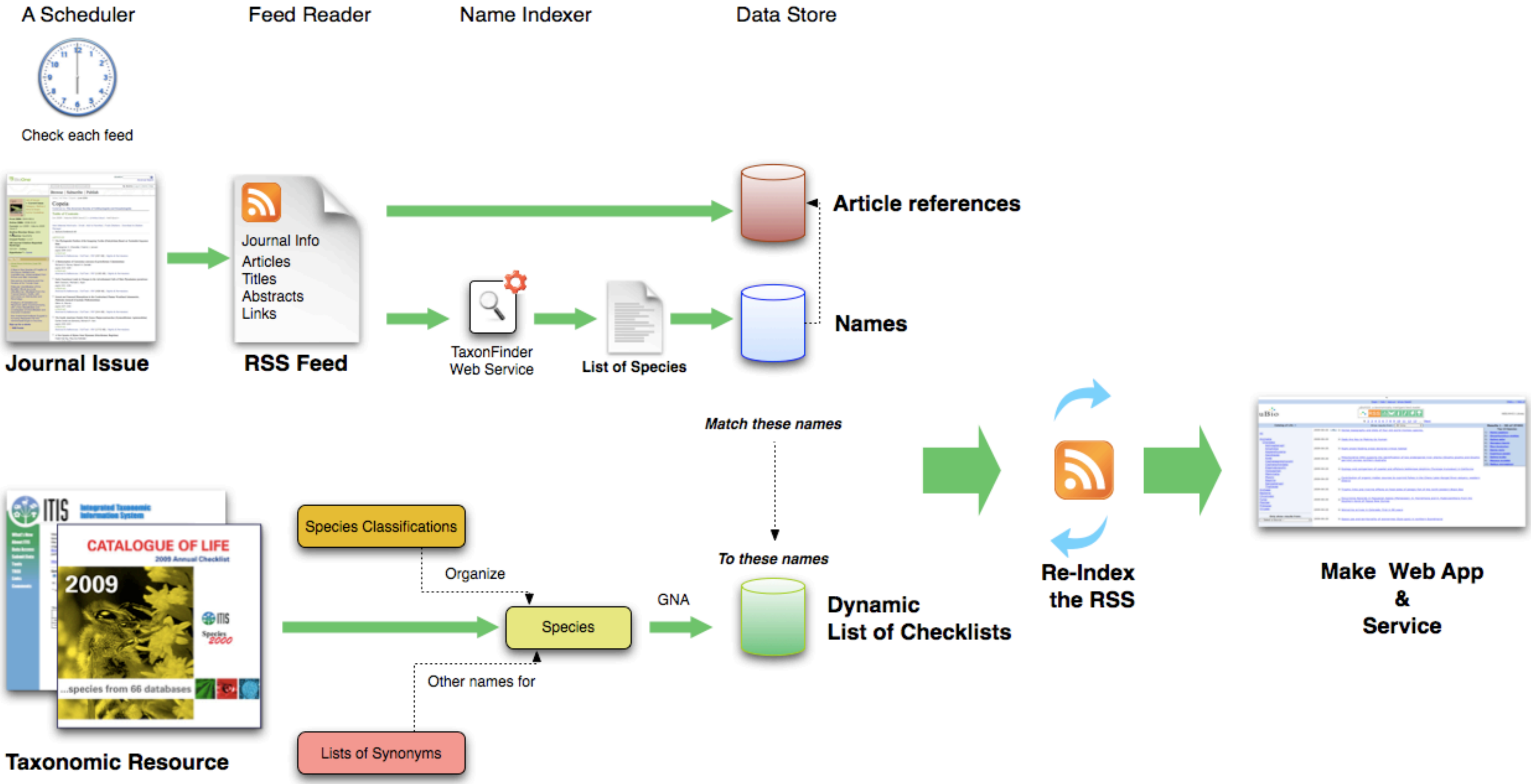

uBioRSS : Publications re-organised by taxonomy

GBIF

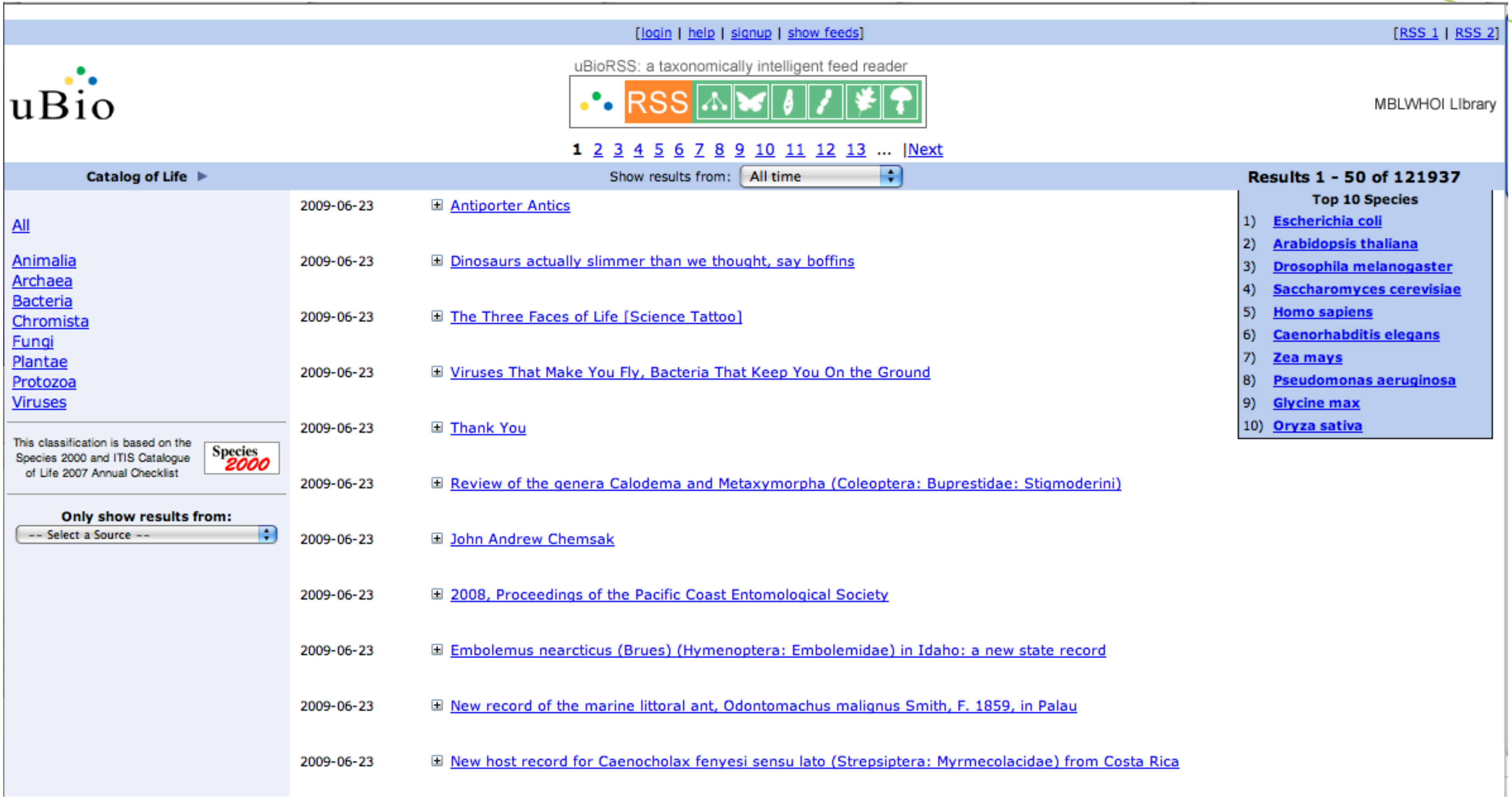

uBioRSS : RSS details available with matched names

GBIF

[login | help | signup | show feeds] [RSS 1 | RSS 2]

uBio

uBioRSS: a taxonomically intelligent feed reader

RSS

MBLWHOI Library

1 2 3 4 5 6 7 8 9 10 11 12 13 ... |Next

Catalog of Life ▶

Show results from: All time

Results 1 - 50 of 5079

All

Animalia
- Chordata
  - Aves
    - Anseriformes
    - Apodiformes
    - Bucerotiformes
    - Ciconiiformes
    - Coliiformes
    - Columbiformes
    - Coraciiformes
    - Craciformes
    - Cuculiformes
    - Galbuliformes
    - Galliformes
    - Gruiformes
    - Musophagiformes
    - Passeriformes
    - Piciformes
    - Psittaciformes
    - Strigiformes
    - Struthioniformes
    - Tinamiformes
    - Trogoniformes
    - Turniciformes
    - Upupiformes

Archaea
Bacteria
Chromista

2009-06-20 Occurrence Records in Papuasian Aglaia (Meliaceae): A. Pannelliana and A. Puberulanthera from the Southern Karst of Papua New Guinea

2009-06-19 Expectant parents groom adult sons according to previous alloparenting in a biparental cooperatively breeding primate

ScienceDirect Publication: Animal Behaviour

ScienceDirect

Publication year: 2009
Source: Animal Behaviour, In Press, Corrected Proof, Available online 18 June 2009
Anita J., Ginther , Charles T., Snowdon

In the biparental, cooperatively breeding cottontop tamarin, Saguinus oedipus oedipus, adult sons provide an energetically and reproductively valuable service to their breeding parents by helping to carry infants. Other species may use aggressive coercion or punishment to enforce alloparenting. Yet, affiliation in primates is important in negotiating social relationships and may be exchanged for services. We hypothesized that the rewards of grooming might be used by expectant parents to cultivate strong relationships with and maintain the services of adult son alloparents. We tested for an interchange trade of aggression or grooming for infant carrying according to a biological market. Individual...

Keywords: Acipenser transmontanus, Callimico goeldii, Callithrix jacchus, Callithrix jacchus jacchus, Callithrix kuhli, Euplectes orix, Haliotis asinina, Heterocephalus glaber, Leontopithecus rosalia, Macaca fascicularis, Macaca mulatta, Macaca sylvanus, Malurus cyaneus, Microcebus murinus, Morone chrysops, Morone saxatilis, Mungos mungo, Neolamprologus pulcher, Saguinus fuscicollis, Saguinus labiatus, Saguinus mystax, Saguinus oedipus, Saguinus oedipus oedipus, Suricata suricatta

2009-06-19 Use of landscape metrics to predict avian nest survival in a fragmented midwestern forest landscape

2009-06-17 Migratory songbird use of shade coffee in the Venezuelan Andes with implications for conservation of cerulean warbler

Top 10 Species

1) **Parus major**
2) **Gallus gallus**
3) **Sturnus vulgaris**
4) **Columba livia**
5) **Taeniopygia guttata**
6) **Anas platyrhynchos**
7) **Passer domesticus**
8) **Ficedula hypoleuca**
8) **Hirundo rustica**
10) **Uria aalge**

Publications: Taxonomically organised

1000s of RSS feeds

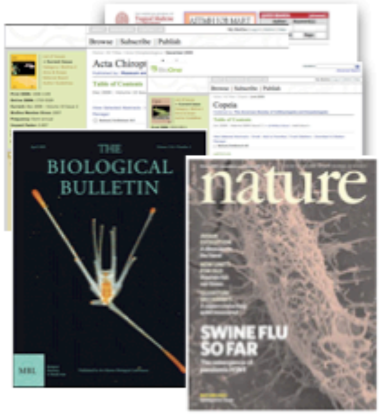

100,000s of articles

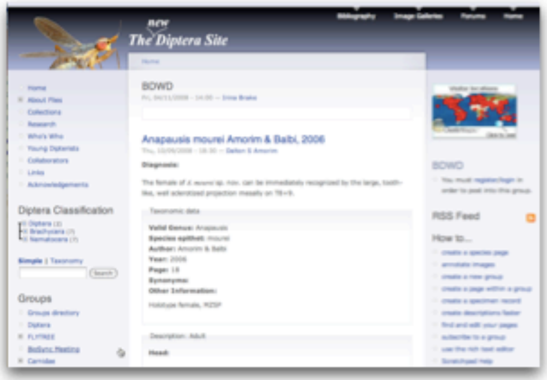

125,000 species of Diptera

Re-Organized by Taxonomic Group

Diptera (Flies) (125,000)

Culicidae (Mosquitoes) 3000+

Aedes (Yellow fever mosquitoes) 961

Aedes aegypti
Stegomyia aegypti
Culex aegypti

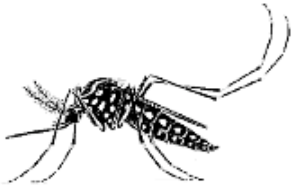

Publications: Thematically organised

GBIF

1000s of RSS feeds

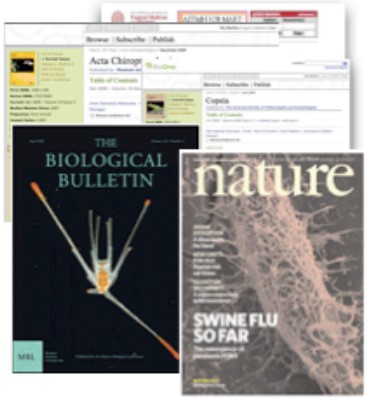

100,000s of articles

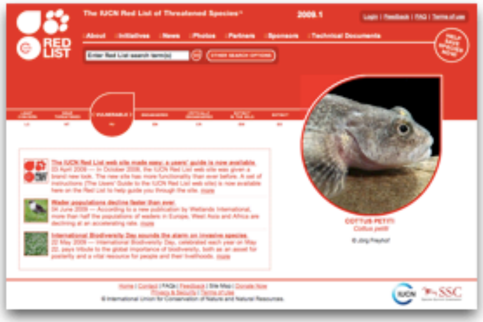

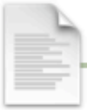

8241 species

Organised by Thematic Species groups

Endangered Vertebrates 2366 articles

Endangered/Threatened Birds 961 articles

Red-listed Hummingbirds 4 articles

Eulidea yarrelli *Chilean Woodstar* 2 articles
Myrtis yarrelli

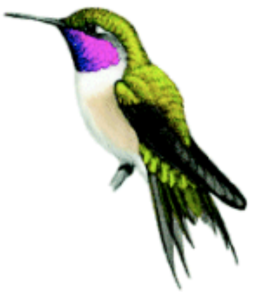

Publications: User-defined species lists

1000s of RSS feeds

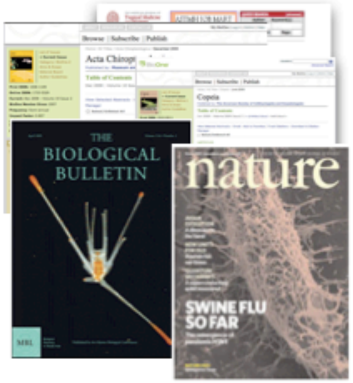

100,000s of articles

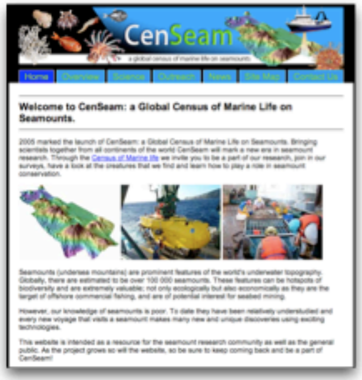

Species that live on Seamounts

7,014 species

User-submitted species lists

- Embassichthys bathybius
- Systellaspis cristata
- Eupasiphae gilesii
- Plesionika rossignoli
- Sergia maximia
- Glyptidotea lichtensteini
- Paridotea ungulata
- Cymodoce unguiculata
- Jaeropsis monsmarinus
- Leontocaris bulba

Page 1 of 700

Consider RSS Integration for PESI

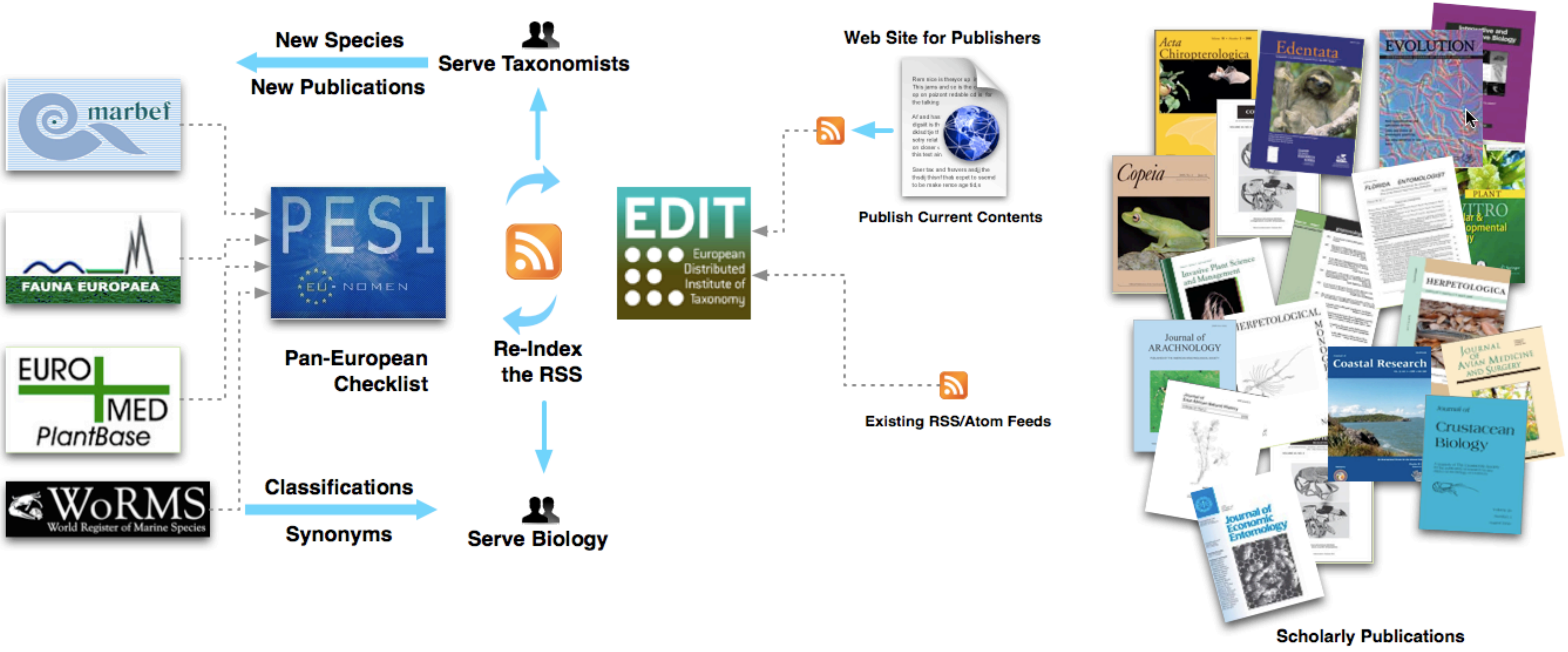

2009 Developments

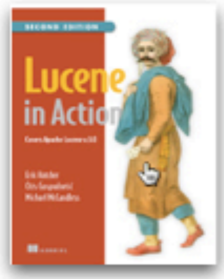

Distributed processing

Indexing full journal runs

Open Source technologies

Massive pre-processing

Community Standard APIs

1 Improve the Technology

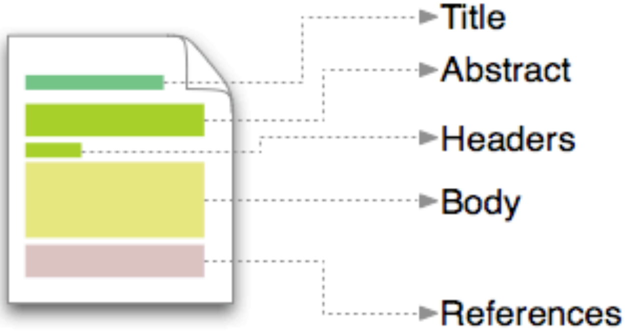

2 Increased granularity in Name Indexing

JOURNAL CITATION REPORTS

3 Better management /filtering of journals

Taxonomic Names Processing Group

Quercus bycolor

0 1 2 3 4 5 6 7 8 9 10

http://code.google.com/p/taxon-name-processing/

4 Improve community coordination

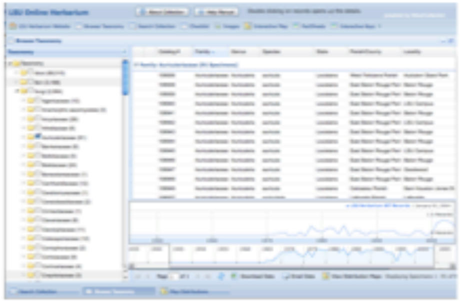

5 Better interfaces and services

Thank you

dremsen@gbif.org
Skype:dremsen

From Publications to Taxonomic databases

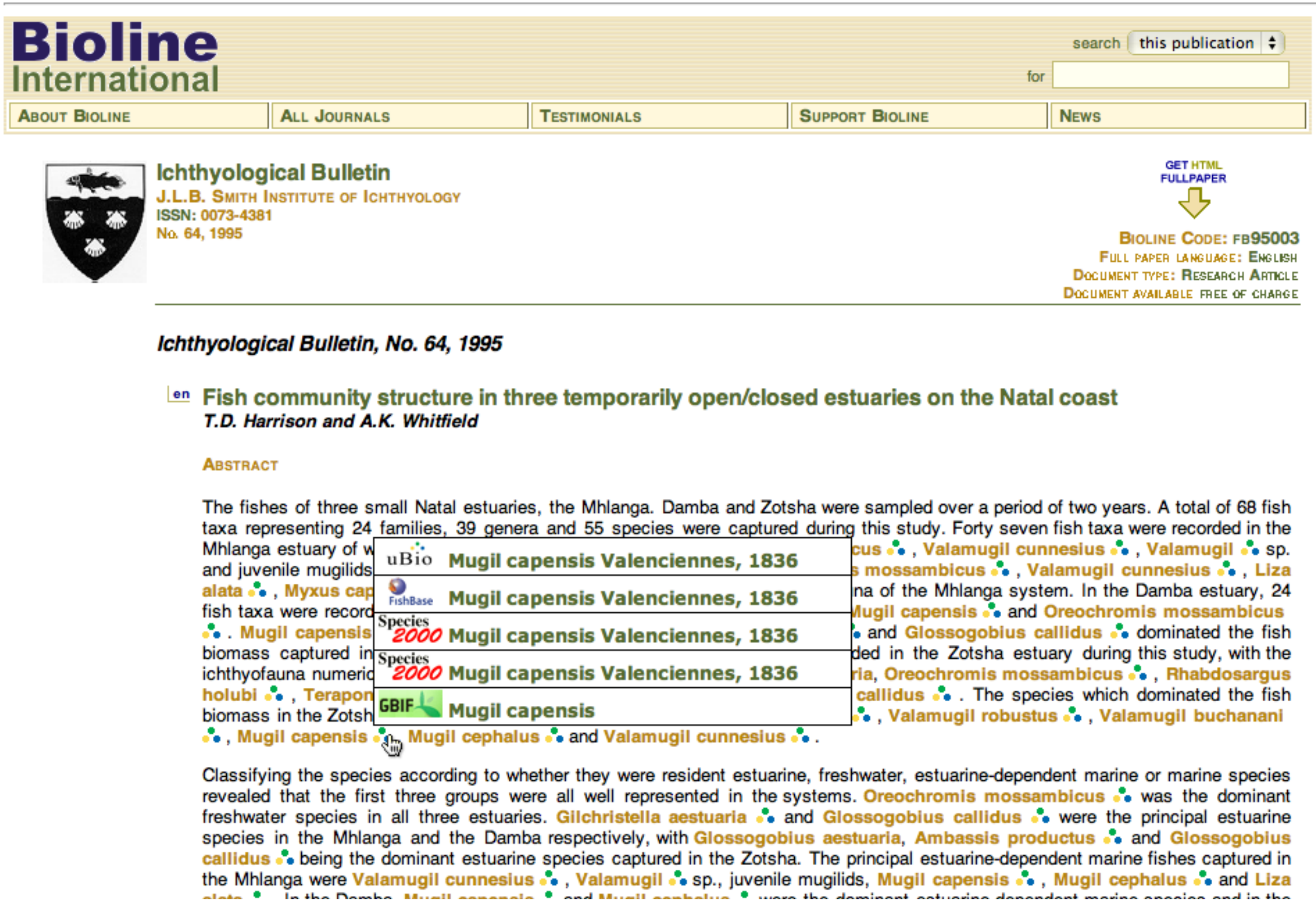

- on-the-fly
- up-to-date

From Publications to other similar publications

GBIF

Bioline International

search this publication
for

ABOUT BIOLINE | ALL JOURNALS | TESTIMONIALS | SUPPORT BIOLINE | NEWS

Ichthyological Bulletin
J.L.B. SMITH INSTITUTE OF ICHTHYOLOGY
ISSN: 0073-4381
No. 64, 1995

GET HTML FULLPAPER

BIOLINE CODE: FB95003
FULL PAPER LANGUAGE: ENGLISH
DOCUMENT TYPE: RESEARCH ARTICLE
DOCUMENT AVAILABLE FREE OF CHARGE

Ichthyological Bulletin, No. 64, 1995

en **Fish community structure in three temporarily open/closed estuaries on the Natal coast**

T.D. Harrison and A.K. Whitfield

ABSTRACT

The fishes of three small Natal estuaries, the Mhlanga. Damba and Zotsha were sampled over a period of two years. A total of 68 fish taxa representing 24 families, 39 genera and 55 species were captured during this study. Forty seven fish taxa were recorded in the Mhlanga estuary of which **Gilchristella aestuaria**, **Oreochromis mossambicus**, **Valamugil cunnesius**, **Valamugil** sp. and juvenile mugilids numerically dominated. In terms of biomass, **Oreochromis mossambicus**, **Valamugil cunnesius**, **Liza alata**, **Myxus capensis** and **Mugil cephalus** dominated the ichthyofauna of the Mhlanga system. In the Damba estuary, 24 fish taxa were recorded, the most abundant being **Glossogobius callidus**, M. capensis and O. mossambicus. M. capensis, M. cephalus, O. mossambicus and G. callidus dominated the fish biomass captured in the Damba system. A total of 56 fish taxa were recorded in the Zotsha estuary during this study, with the ichthyofauna numerically dominated by juvenile mugilids, G. aestuaria, O. mossambicus, **Rhabdosargus holubi**, **Terapon jarbua**, **Ambassis productus** and G. callidus. The species which dominated the fish

(2009-02-27) ScienceDirect Search: species
Artificial mouth opening fosters anoxic conditions that kill small estuarin...

Classifying the species according to whether they were resident estuarine, freshwater, estuarine-dependent marine or marine species revealed that the first three groups were all well represented in the systems. **Oreochromis mossambicus** was the dominant freshwater species in all three estuaries. **Gilchristella aestuaria** and **Glossogobius callidus** were the principal estuarine species in the Mhlanga and the Damba respectively, with **Glossogobius aestuaria**, A. productus and **Glossogobius callidus** being the dominant estuarine species captured in the Zotsha. The principal estuarine-dependent marine fishes captured in the Mhlanga were V. cunnesius, **Valamugil** sp., juvenile mugilids, M. capensis, M. cephalus and L. alata. In the Damba, M. capensis and M. cephalus were the dominant estuarine-dependent marine species and in the Zotsha juvenile mugilids, R. holubi, T. jarbua, M. capensis, **Valamugil cunnesius**, **Valamugil robustus**, M. cephalus, L. alata and **Valamugil buchanani** were the principal estuarine-dependent marine species.

- on-the-fly
- up-to-date
