# Supplementary material for: PESI - a taxonomic backbone for Europe
Source: Biodivers Data J. 2015 Sep 28;(3):e5848. doi: 10.3897/BDJ.3.e5848 (PMC4609752; doi:10.3897/BDJ.3.e5848)
Supplement: Supplementary material 27 — EDIT Scientific Publishing in Natural History Institutions 3nd meeting [file biodiversity_data_journal-3-e5848-s027.pdf]

Project no. 018340

Project acronym: EDIT

Project title: Toward the European Distributed Institute of Taxonomy

C3.6.4
Report on

3rd meeting
Scientific Publishing in Natural History Institutions

Meeting held

October 7-8, 2010

Copenhagen

version 1

Project co-funded by the European Commission within the Sixth Framework Programme (2002-2006)		
**PU**	Public	X
**PP**	Restricted to other programme participants (including the Commission Services)	
**RE**	Restricted to a group specified by the consortium (including the Commission Services)	
**CO**	Confidential, only for members of the consortium (including the Commission Services)	

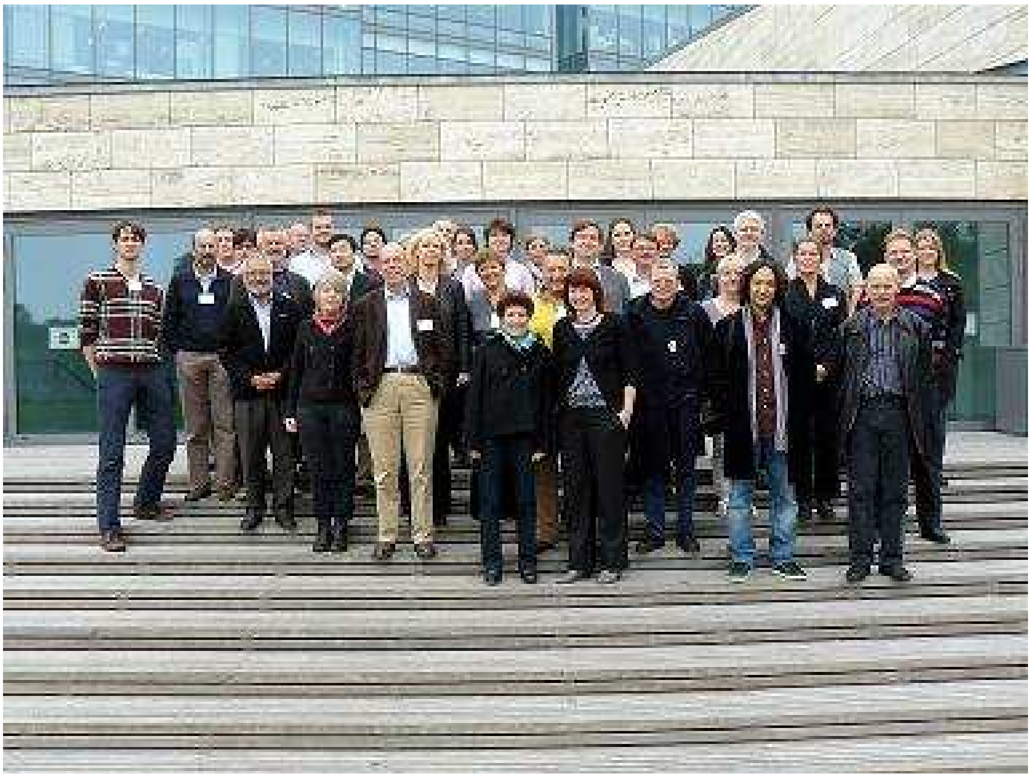

Acknowledgements

This third EDIT meeting on Scientific Publishing in NHIs came into being with help of a number of people. We would like to thank all of those who provided us with input and feedback during the preparation of the meeting, we would like to name Isabelle Gerard, Michele Ballinger and Graham Higley, but there were many others. Furthermore, we thank the EDIT colleagues from the Natural History Museum of Denmark Katrine Mohr, Jesper Birkedal Schmidt and Danny Eibye-Jacobsen for their help with the logistics and their kind hospitality hosting the meeting. Also we thank our sponsors the EDIT and PESI projects for their financial support. Lastly, we thank all the publishing representatives and speakers that came to Copenhagen to share their views with us.

For questions on the meeting or the meeting report, please contact the organisers:

Laurence Bénichou
Editorial manager, Muséum National d'Histoire Naturelle
benichou@mnhn.fr

Daphne Duin
EDIT – Stakeholder Liaison Officer
duin@mnhn.fr

From March 1, 2011
User Liaison Officer ViBRANT
Research associate
VU-University Amsterdam
d.duin@vu.nl

Table of contents

Introduction 4

Participation and format 4

1 Trends and Developments 4

The new article 4

The new journal 5

The new research library 6

2 Collaborative solutions 8

Moving together to Open Access 8

Enhancing the access, visibility and impact of taxonomic publications 9

Literature digitalisation projects 10

3 Conclusions and recommendations from discussion 10

Annex 1 - Acronyms 12

Annex 2 - Agenda 13

Annex 3 - Abstracts 15

Annex 4 - break-out groups 17

Annex 5 - Participant list 19

Introduction

The EDIT scientific publishing meetings were held in the light of the project's objectives as European virtual centre of excellence, aiming to increase the scientific basis and capacity for biodiversity conservation. The meeting series[1] discussed the enhancement of access and dissemination of taxonomic publications will contribute to both of the previous[2].

Natural history institutions have been publishers of scholarly publications (journals and monographs) since they were founded[3]. Over time specific publishing traditions in natural history got established. In the digital era of today the field of scholarly publishing is rapidly changing. The move towards the web of online journals and use of extensive digital services will greatly help enhance access and dissemination of publications. However some of the recent trends clash with long existing publishing traditions in the field of natural history. During the 1st and the 2nd meeting on Scientific Publishing in Natural History Institutions (NHIs) (2008, 2009) several barriers of change were indentified and discussed. The third meeting, which discussion is reflected, in this report aimed to look over the barriers and focused on what is ahead of us in the (near) future and on at what these developments have to offer to taxonomy and to taxonomic institutions.

This third meeting, in the series of EDIT Scientific Publishing meetings was organised in collaboration with PESI and is an example of how both projects liaise with stakeholders from the field of scholarly publishing.

Participation and format

In the 3rd meeting on Scientific Publishing in NHIs 38 people, from 21 institutions among which were 14 EDIT member institutions, participated. The meeting had plenary talks and one break-out session. Slides of the presentations and other meeting documents can we found on the website [http://www.e-taxonomy.eu/publishing then click: 3rd meeting Scientific Publishing in Natural History Institutions].

1 Trends and Developments

Publishing scholarly information online has many interesting advantages to offer to authors and readers. The majority of online publishing journals make pdf files available on their website. These electronic files look a lot like the traditional hard copies and offer a similar experience (service) to the reader. However during the last years or so some journals have switched to publishing their content in .html or .xml format. These formats allow the integration of hyperlinks in the reference lists which provide: direct access to cited papers; RSS feeds; geographical information with zoom options for greater details; access to relevant grey literature etc. Examples of these Web 2.0 publications can be found in BiomedCentral, PloSOne, PubMed, in some extent in Zookeys. These developments have an impact on scientific publishing business at different levels. In the workshop in Copenhagen we discussed developments at the level of the article, the journal and the library

The new article

From the presentations and discussion in Copenhagen we learnt that the examples above are only the beginning of what Web 2.0 features in scholarly publications have to offer. The prevailing model for

---

[1] In total 3 meetings were held, December 2008, June 2009 and October 2010 see also http://www.e-taxonomy.eu/publishing

[2] The EDIT project ran from March 2006 until February 2011.

[3] Among EDIT member institutions around 65 journals are published

online publishing is still pdf based and the majority of the online publications still resemble the traditional print publications. However some publishing services are experimenting with linking to the original data material that have been used by the author, such as databases, video and interview material, 3-d images of collection objects etc. These types of publications are often drawn up in XML format (machine readable format) and are sometimes called "enhanced publications" [see presentation John Doove, SURFfoundation]. An enhanced publication generates all kinds of new uses for publication and at the same time raises questions about the role of the journal and why and under which conditions linking to original data can be an interesting feature and a valuable addition to pdf formatted papers. Some advantages and considerations of an enhanced publication are mentioned in textbox below[4].

Supporters of the Open Science approach[5] applaud this development because the author can demonstrate in much greater detail how he came to his conclusions and makes it possible to the reader to repeat more accurately scientific experiments, either to use the original dataset for new research (of course with giving recognition to the data contributor).

Enhanced publications are:

Pro's
• Simplification of accessibility and exchange of research data
• Increases visibility, discoverability
• Increases transparency of the data used
• Facilitates the reproduction of (lab) experiments, surveys etc.
• Facilitates quality control of the data used

Considerations
•Copy rights (some data sets are behind a subscription or were purchased with a copy right licence and cannot made available)
• Sustainability of links and data repositories, this increases the complexity and IT competencies of publishers (and libraries?)
• Increases complexity of peer review

SURFfoundation

The new article makes use of article-level impact measurement ("tracks": citations, web usages, expert rating, social bookmarking, community rating, media/blog coverage, commenting activity) in contrast to measuring the number of citations per journal. The traditional disciplinary academic journal as forum for the presentation and scrutiny of new research is losing its dominant position.

The new journal

Although things are changing, the prevailing model today is still that articles get submitted to a journal (where the name of journal is important), the article goes trough different rounds of peer-review before it gets accepted or rejected. It can take months or years from the findings being obtained to being published. Organisations like PLoS are questioning this model and ask: 1) "What do we need to do before research is published? 2) What can we leave until after publication? [See presentation Mark Patterson, PLoS].

New web technologies in scholarly publishing have increased the speed and amounts of information that we have access to. They bring information right at the desk of users in research and policy makers

[4] SurfFoundation has been involved several pilot project on enhanced publications see http://www.surffoundation.nl/en/themas/openonderzoek/verrijktepublicaties/Pages/default.aspx

[5] Open Science" encompasses the ideals of transparent working practices across all of the (life) science domains, to share and further scientific knowledge. It can also be thought of to include the complete and persistent access to the original data from which knowledge and conclusions have been extracted. From the initial observations recorded in a lab-book to the peer-reviewed conclusions of a journal article".

and other practitioners. New models of scholarly communication are tested in for instance PLoS One and PloS Currents, publishing examples which are demonstrating that the journal landscape is changing. In PLoS One the prevailing model is continued but part of the peer review takes place after publication. Before publication a pre-publication peer review process is followed but any article that is rigorous science will pass. Articles are not excluded on the basis of lack of perceived importance or adherence to a scientific field, this can be done after publication. The PLoS One work flow is traditional in the sense that the peer review process and the production process still takes about 100 days. PLoS Currents instead has a complete different work flow and its main objective is to accelerate the publication of new ideas and findings while preserving the functions of a journal as a communication platform. PLoS Currents is organised around subject sections, each section has a small group of scientific experts, the board of reviewers who make a rapid determination if the content is appropriate or not (do the conception, structure and presentation of the submission indicate that it is a legitimate work of science and does not contain any obvious methodological, ethical or legal violations). There is no external review beyond that core group. If the article passes the test it gets published immediately and is achieved in PubMed Central 6. Authors submit their articles trough Google Knol, saving resources on typesetting and desk editing.

As said before, the new trends in Web 2.0 technologies have triggered a debate around the role of the journal. In the digital era research articles are "discovered" trough search portals (e.g. PubMed Central, Google Scholar, BioOne) where the portal is the first point of access, not the journal. The digitalisation has boosted Open Access publications resulting in today, 5795 journals and 485659 scientific articles are only one click away from every one with internet access (see http://www.doaj.org/ (2/12/10). Furthermore a whole new set of tools has been developed that can visualise networks of literature and of literature and data which offers new ways of looking at relationships and connections and with a new level of innovation and community collaboration.

Publishers are aware of the changing role of the journal and are reinventing part of their business models. Online publications and the use of machine readable publication formats make it possible not only to focus on the article any more but to zoom in at the data level and easily subtract part of the article. "Community publishing" initiatives such as PLoS Biodiversity Hub and ViBRANT will extend these type of services further in the near future in the field for biodiversity research, offering data-community building services. The Biodiversity Hubs of PLoS (like ViBRANT) is exploring new grounds for the scholarly publications by asking "how to organise content otherwise than by journals?" The Hub's are meta journals, added on top of the literature, essentially they aggregate Open Access content, around a particular theme, in this case biodiversity. The Hubs add value to the content to themes by linking data and by building expert communities of curators around the content.

The new research library

The developments discussed above also have their impact on the role of research libraries [see presentation Lars Björnshauge]. Today scientists have access to literature 24/7 without having to leave their desk. This does not mean that libraries will become obsolete, if they are willing to face the necessary changes. As Lars Björnshauge put it : "During the last decade libraries have changed more than during the last century" but "libraries are no longer islands in institutions". The research libraries of today are expected to:

---

6 PubMed and PubMed Central are two different systems. PubMed is a citation database. PubMed Central is a free digital database of full-text scientific literature in biomedical and life sciences.)

- be involved in the development of teaching and support to learning
- Handle the institute's output (publishing)
- Engage in scholarly communication
- be involved in research evaluation support

Although researchers visit the library less and less, students on the other hand are using the library more and more for self study, group work and learning.

> Trends at the Lund University teach us that there is a clear need for digital information resources among researchers. Their library expenses of e-books quadrupled between 2002 and 2008 and that the download of e-books rose from 6k to 4141k.
>
> *Lars Björnshauge, SPARC Europe*

Today, in the digital era researcher's work with electronic publications not with paper. The libraries' responsibility is to look after the back files and institutional repositories. Libraries face that their library information system is not longer the key system for literature access – these systems are often too complicated and obsolete to use and above all do not adequate access to digital resources. Other portals such as Google Scholar are more used.

Different needs of the users of research libraries need to be followed by a shift in budget priorities and perhaps even the adoption of a new economic model (see for example *Hahn, 2008*[7]). An example of a traditional paper-era agreement are the library exchange programmes (exchanges of free hard copies between befriended institutions). These programs still exists between natural history institutions, but in most other fields they have been abandoned since a long time. This exchange of paper does not fit in the responsibility and profile of the new research library. As was discussed during the 2nd meeting on Scientific Publishing in NHI (Bratislava meeting June 2009), there is no savings argument to keep the programs in the air, on the contrary the programs are expensive

> Yesterday users had to come to the library and use our system. Tomorrow libraries have to be where the users are library services have to be embedded in the user services, platforms, devices and gadgets…Or become obsolete!
>
> *Lars Björnshauge, SPARC Europe*

The Scientific Publishing meetings in 2008 and 2009 showed that adopting to change is not always easy, sometimes even painful. This meeting in Copenhagen highlighted the opportunities of new scholarly publishing trends. It was stressed that if institutions inform themselves and participate in the debates on trends and pilot projects they can influence the developments in the best interests of their field. However, informed participation demands for an investment at the level of institutions and individual staff members.

[7] Hahn, K. (2008). Research library publishing services: new options for university publishing and new roles for libraries. *Association for research libraries,* 2 April, p. 8-9. URL: http://www.arl.org/bm~doc/research-library-publishing-services.pdf

2 Collaborative solutions

At different levels and on different subjects, individuals, institutions and initiatives are seeking to improve the access and dissemination of their publications by initiating or following the trends mentioned before. As described in previous reports new trends come with new challenges. Below we list some solutions for different challenges. Many of them are based on a collaborative approach. The main reasons to opt for a collaborative solution are: to increases expertise and resources and to avoid duplication of effort. During the meeting in Copenhagen we focused on examples on how joint efforts generate solutions to some of the barriers mentioned in the text boxes below.

How are taxonomic publications "different"?

- Taxonomic publication with new taxa are governed by nomenclature *codes* which for now inhibits e-only publications
- Taxonomic publications are historical records, they are timeless, and they are referred no matter how old they are.
- In taxonomic literature priority is important, so the date of publication is extremely important because it provides the base to determine which is the valid name.
- Artificial restrictions on pages numbers in each issue cannot handle the demand for taxonomic publications
- "The taxonomic impediment"

Barriers faced by taxonomic publications

- Low ISI impact factor
  - There are few publication outlets for monographs which results often in isolated book publications that do not go into the citation cycle
  - Small expert communities therefore few potential citations
- Technological expertise and rapid changes make it difficult to keep up as a small journal

Moving together to Open Access

An example of a collaborative approach to new developments in scholar publishing is the NOAP project that run from 2007 to 2010. Within the project Nordic libraries worked together to move their journals to an OA publishing model. Through knowledge exchange the project aimed to contribute to a broader understanding of Open Access and consequences of e-publishing as well as contribute to making Nordic research visible. The partners were successful in:

- Translating the Open Journal Systems to Danish, Swedish, Norwegian, Finnish
- The start of a Nordic Open Journal Systems User Group (Nordic OJS Users http://www.ub.uit.no/wiki/noap/index.php/Nordic_Journals_using_OJS)
- Investigation and questionnaire on journal business models and Nordic journal hosting/publishing possibilities (journal hosting/publishing)
- Making a start among traditional humanities journals to move towards electronic publishing
- Creating a network of stakeholders

Changing a publication model has consequences for a journals business model, work flow and technical requirements have to be met. The experiences from NOAP are that together such a step is easier to make

and will be more effective and make services user friendly

Enhancing the access, visibility and impact of taxonomic publications

In order to enhance the access, dissemination, visibility and impact of taxonomic journals two compatible ways forward were presented in the Copenhagen meeting.

The first project focuses on the integration of expertise and resources. The project is run by a group of 5 natural history institutions who decided collectively to launch a journal in taxonomy under the name European Journal of Taxonomy (EJT). Traditionally NHIs have their own journals sometimes several. Unfortunately many of these have little visibility. The aim of EJT is that by bringing these institutions together expertise and resources become available to publishing a journal according the latest (technological) standards. Some of the journals will merge their existing title(s), other consortium members will run the journal next to their existing bundle of publications. The new journal will work with a distributed team of technicians employed by different consortium members.

The rational behind the launch of EJT is that next to the (some successful) commercial journals, there is also a need for NHIs to act as public producers of taxonomic information. EJT will be Open Access and will not charge author fees and articles will be stored for perennial access in the BHL. EJT's public business model offers to taxonomy: 1) an organisational mean to publish a truly Open Access journal; 2) a facility to continue (and enhance) decades of publishing expertise developed within NHI institutions; 3) ease of collaboration at technical and scientific level; 4) empowerment of institutions and their staff to act as experts in the international scholarly publishing debate and to look after the interests of their field of work. The increased scale, use of new technology and "open archiving" and the participation of several large institutions EJT will give taxonomic publications an enhanced visibility and impact. The publication of the first article is scheduled for 2011.[8]

Another way forward is to focus on journal aggregation. Zhi-Qiang Zhang, founder and editor-in-chief of Zootaxa presented a proposal of a common Web portal for taxonomic journals which has integrated several publishing facilities such as a publishing system to manage manuscripts and peer review, the possibility to register for DOIs, and an achieving facility for perennial access. A common archive has many advantages, of which one is that this will remove the key objection of the Commission for Zoological Nomenclature to allow e-only publications. The portal would use a model similar to BioOne or JSTOR but will only store taxonomic publications and offer the services that are relevant to taxonomy. Besides one contract for electronic services the portal will offer to journals an easy way to track the impact for their publications. Citations within the portal can be easily linked and tracked, the number of citations and other functions can generate article metrics and rank article based impact. Because taxonomic papers mainly cite taxonomic work - having a large bundle of taxonomic journals in one portal will make a more accurate measure of its real importance. Another very important thing what can be done in the portal relates to taxonomic names. The names are the key to information the portal will provide mark up items that will help to link and search more easily all the information about biodiversity. Finally, within the portal journals keep their own identity and have the flexibility to choose to go Open Access or use a subscription based access model. Museums, universities and societies who join the portal, will pay a membership fee. The membership fee will be used for the maintenance and costs of for the content provider.

Besides the above projects on integrations and aggregation of taxonomic journals, two other collaborative examples were presented in Copenhagen which are dealing with literature digitalization.

---

[8] The project is also discussed in the report of 2009 http://www.e-taxonomy.eu/files/Report_Scientific_Publishing_NHIs_2009.pdf . Institutions involved are: Natural History Museum, London, Muséum national d'Histoire naturelle, National Botanic Garden of Belgium, Royal Belgian Institute of Natural Sciences, Brussels, Royal Museum for Central Africa, Tervuren

Literature digitalisation projects

The Global References Index to Biodiversity (GRIB) is a scanning management tool for librarians and scientists. At the time of writing the GRIB tool was still in the developing phase. The purpose of GRIB is to support the creation of an index for bibliographic references, based on taxonomic literature catalogues. It is a bibliographic index of EDIT and BHL-Europe partner libraries developed jointly with the BHL-Europe Project. It links to the index of references and provides information on subscribed digital literature resources available at the partners. GRIB links through to these resources where access is permitted, it links freely to available digital resources (e.g. BHL) to the index of references and makes the search capabilities for the index of references accessible as (i) a web service and (ii) within a web page (ViTaL web portal). GRIB allows users of the index of references to nominate literature items for digitisation. At the time of writing a GBIB demonstrator was available via http://grib.gbv.de/. The demonstrator is a joint effort of EDIT/ViTaL and BHL-Europe, built by the Common Library Network GBV (Gemeinsamer Bibliotheksverbund) and allows users to browse and search titles held by EDIT, BHL-Europe and BHL member institutions (test sets at the time of writing are from BGBM, MfN, and NHM) using advanced filtering. It will be a tool to manage taxonomic literature that is (a) already available in digital form, (b) in the process of being digitised, and (c) for which plans have been created for digitisation.

A second digitalization partnership presented concentrates on sharing human and material resources. The three institutions in the Belgian Centre for Taxonomic Facilities (BETAF) collaborate in several digitisation projects around online access of taxonomic publications these projects are among others BHL, STERNA and EUROPEANA. Digitisation of literature requires specific knowledge regarding Intellectual Property Right and risk managements around IPR questions. Collectively the three institutions contribute to technical documents and a wiki. They share material such as servers and scanners and they apply a common IPR policy. This collaboration allows them to make efficient use of human and material resources, will avoid a duplication of efforts of which taxonomic publications will benefit.

3 Conclusions and recommendations from discussion

The presentations and discussion during the meeting have brought many issues at light relevant to the natural history institutions' scientific publishing agenda for 2011 and beyond. The conclusions and recommendations are relevant for technical and management staff in publishing and library departments of Natural History institutions.

Trends in digital publishing will change the function of the scientific paper and the research journal. The enhanced publication and the new journal offer many new opportunities for researchers to share data, research findings, to explore new forms of collaboration, new audiences and alternative ways to measure impact. Online (data) publishing offers for taxonomists the possibility to make their work available and usable to the biodiversity community at large and at the same time a possibility to measure (track) the use of their data more accurately than is the case in a traditional publishing environment. "Enhanced" publications, data publication and community publications as discussed in this report will take down the information silos that are so prominent in taxonomy where publications are explicitly organised around sub-disciplines (marine, terrestrial, botany, zoology ect) and specific species groups. This trend will help to further embed taxonomy in the landscape of biodiversity research and to make its contribution to other disciplines more visible.

In natural history institutions the libraries are no longer islands in institutions, but a central node in the organisation for scholarly information management. They are responsible for training researchers and students to access information and have an important role in providing information to the institutions' management and for research evaluation purposes. The new library manages the virtual information environment of the institution and offers a physical space where researchers and students come for individual and group work. It was said that library exchange programmes will be severely impacted by

changes in publication business models within the next 3-5 years.

Modern scholarly publishing management requires cross institutional collaborations in order to keep up with the rapid changes in use of technological standards, workflows and business models. Collaboration is also important to be able to address barriers to digital publication, such as the library exchange programmes and to enhance the visibility of publications trough Web portals and the efficient and safe application of IPR policies. Projects such as the launch EJT or a journal Web portal require a collaborative approach so to have access to the necessary human and material resources and to avoid a duplication of efforts.

In order to make the above possible the following recommendations to EDIT institutions and their staff were formulated:

- Publish Open Access and use reliable archiving solutions for perennial access to publications and data
- Explore the possibilities to introduce enhanced publications in taxonomy. Make use of the experiences from the "enhanced publication projects" of SURFfoundation and similar initiatives.
- Encourage researchers to participate in data publishing and community publications
- Bring the limitations of the ISI impact factor for taxonomy to the attention of your university and national science boards.
- Contribute to the discussion on how we can better measure the impact of taxonomic publications
- Set up a working group to establish an elegant process to wind-down most exchange activity in a consistent way across the EDIT/CETAF organisations. This will enable consistent and helpful support for exchange partners in the developing and biodiversity-rich countries who may be reliant on these sources of information.
- Libraries should index meta data and incorporate them in the offers from aggregators subscription agents, link resolvers, discovery services. If not their information will be forgotten and ignored
- Integration at the journal level as well as aggregation of taxonomic journals in a common Web portal will enhance the visibility and access to taxonomic publications and is essential in order to have the right human and financial resources at hand.
- Set up collaborations (meetings, mailings lists, wikis) at all levels within the publishing chain to collectively solve day-to-day /technical issues and to have well informed staff members, able to participate at an international level in scholarly publishing debates on taxonomic publications and to look after the interest of taxonomic publications at a (national) science policy level.

Annex 1 - Acronyms

**BETAF**	Belgian Centre for Taxonomic Facilities
**BGBM**	Botanischer Garten und Botanisches Museum
**BHL**	Biodiversity Heritage Library
**CETAF**	Consortium of European Taxonomic Facilities
**DOIs**	Digital Object Identifier
**EDIT**	European Distributed Institute of Taxonomy
**EJT**	European Journal of Taxonomy
**GBIB**	Global References Index to Biodiversity
**IPR**	Intellectual Property Right
**ISI**	Institute for Scientific Information
**JSTOR**	Journal Storage
**MfN**	Museum für Naturkunde
**NHI**	Natural History Institution
**NHM**	Natural History Museum
**NOAP**	Nordic Open Access Publishing
**OA**	Open Access
**PESI**	A Pan-European Species directories Infrastructure
**PLoS**	Public Library of Science
**ViBRANT**	Virtual Biodiversity Research and Access Network for Taxonomy
**ViTaL**	Virtual Taxonomic Library

Annex 2 - Agenda

TIME	MIN	ITEM	DAY 1	
11:00 until 13:00	120	0	**REGISTRATION OPEN**<br>**In front of meeting room**	
12:00 until 13:00	60	0	**LUNCH (packs)**<br>**Canteen Biocentret seminarrum**	

			**START 13:00 h**<br>**Meeting room:**<br>**Biocentret seminarrum 1-2-03**	
13:00	**SESSION 1**		**INTRODUCTION**<br>**Chair: Graham Higley**	
13:00	5	1	Welcome	Danny Eibye-Jacobsen, Natural History Museum of Denmark
13:05	25+5	2	EDIT & Scientific Publishing in Natural History Institutions	Laurence Bénichou, MNHN
13:35	**SESSION 2**		**TRENDS & DEVELOPMENTS**<br>**Chair: Ruth Linklater**	
13:35	25+5	3	Enhanced publications	John Doove, SurfFoundation
14:05	25+5	4	Open Science and scientific publishing	Daphne Duin, MNHN
14:35	25	5	**COFFEE/TEA BREAK**	
15:00	30+5	6	Rethinking the functions of a journal - some case studies from PLoS	Mark Patterson - PLoS
15:35	25+5	7	Acquisition policy and business models of research libraries in a digital era	Lars Björnshauge – SPARC Europe
	**SESSION 3**		**BREAK-OUT SESSION**	Moderators
16:05	55	8	1) Economic models	Laurence Bénichou, MNHN
			2) Library exchange programme	Graham Higley, NHML
			3) Copyright	Ruth Linklater, RBGK
			4) Dissemination of natural history books	Nigel Massen, NHBS
			5) Fast track publishing	Connie Baak, NCBNaturalis
			6) Co-publishing	Isabelle Gerard, RMCA
17:00	35	9	Presentation break-out groups (5 min each)	
17:35	10	10	**Summary DAY 1: Graham Higley**<br>**Day 1 closes 17:45**	
			**Dinner city centre - 20:00h**	

TIME	MIN	ITEM	DAY 2	
			**START 09:00h**<br>**Meeting room:**<br>**Biocentret seminarrum 1-2-03**	
09:00	**SESSION 4**		**COLLABORATIVE SOLUTIONS**<br>**Chair: Graham Higley**	
09:00	25+5	11	A joint Nordic approach to Open Access and research distribution	Jörgen Eriksson, Lund University
09:30	25+5	12	EJT a new journal	Patrick Grootaert, RBINS
10:00	25+5	13	Journal aggregation and integration for enhancing the access and impact of taxonomic publications	Zhi-Qiang Zhang, Landcare Research, Zootaxa
10:30	20	14	**COFFEE/TEA BREAK**	
10:50	25+5	15	Global References index to Biodiversity (GRIB), a bibliographic index of EDIT and BHL-Europe partner libraries and a scanning management tool for librarians and scientists	Boris Jacobs – MFN
11 :20	25+5	16	A digitalization partnership: Sharing human and material resources	Larissa Smirnova, RMCA, & Régine Fabri, NBGB
	**SESSION 5**		**DISCUSSION + RECOMMENDATIONS**<br>Chair: Danny Eibye-Jacobsen	
11:50	15+5	17	Scientific publishing network after EDIT	Isabelle Gérard, RMCA<br>Michele Ballinger, CNRS
12:10	45	18	Plenary discussion<br>Recommendations to EDIT partners	
			**Meeting Closes 13:00**<br><br>**LUNCH (packs): 13:00 -14:00 h**<br>**Canteen Biocentret seminarrum,**<br>**Ground floor**	

Acronyms:

CNRS	Centre National de la Recherche Scientifique
MfN	Museum für Naturkunde
MNHN	Museum national d'Histoire naturelle
NBGB	National Botanic Garden of Belgium
NHML	Natural History Museum, London
PLoS	Public library of Science
RBGK	Royal Botanic Gardens Kew
RMCA	Royal Museum for Central Africa

Annex 3 - Abstracts

Item 2: 3rd Scientific publishing in Natural History Institutions
Laurence Bénichou

Over the last 3 years a group of publishing and library personnel from NHIs in Europe and the EDIT consortium have identified a combination of traditions and developments in scientific publishing natural history sciences that seriously affect the work of natural history institutions as publishers. These current developments created a need for yearly meetings between technical and management staff to exchange know-how and to draft a common work agenda. Previous meetings Scientific publishing in Natural History Institutions were held in Paris, 2008 and in Bratislava, 2009.

Item 3: abstract is missing

Item 4: abstract is missing

Item 6: Rethinking the functions of a journal - some case studies from PLoS
Mark Patterson
The use of online tools is allowing the processes of scholarly communication to be rethought and re-engineered. At PLoS, the concept of open access has been central to our publishing efforts so far, because open access is a first step towards maximizing the impact of published research findings. More recently we have been exploring new ways to enhance scholarly communication through online publications that publish new findings more rapidly, and new products that facilitate the evaluation and organization of content.

Item 7: Acquisition policy and business models of research libraries in a digital era
Lars Björnshauge
The presentation will focus on the dramatic shift from print to digital information resources - both in terms of what research libraries purchase and in terms of how access to information resources is or will be provided to users within and outside research libraries. Keyword is visibility: what is not on the web does not exist! Special emphasis will be on the emerging shift from subscription based information resources to open access and how research libraries can facilitate this and work with content and service providers.

Item 11: A joint Nordic approach to Open Access and research distribution
Jörgen Eriksson
"The NordBib sponsored project "Aiding Scientific Journals Towards Open Access Publishing" was active from 2008 until spring 2010. Participants where libraries, publishers and scholarly societies with an interest in supporting open access publishing. Participating organizations came from Denmark, Finland, Norway and Sweden. In my presentation I will describe the project and its aims. I will also go into some of the practical results and results of the surveys and overviews of the changing landscape of journal publishing that also where part of the project. In the project we mainly looked at ways to support small journals, published by a scholarly society or an institution."

Item 12: EJT a new journal
Patrick Grootaert, RBINS
Five NHIs plan to launch an electronic, fast-track, Open Access journal in descriptive taxonomy, covering subjects in zoology, botany, and palaeontology under the title EJT, *a European Journal in Taxonomy*. The rational behind the journal is to increase visibility and renew the appreciation of taxonomic information while suiting a long term business model that is important for taxonomy. Our view is that taxonomic institutions should collectively create/claim a centre stage on the Web by offering visibility to authors; make data easily accessible for users of the information and the publishing institutions and guarantee long term access. This means no technical, legal, financial, or time barriers in place that may hinder publishing, access, or reuse of taxonomic data, while respecting the norms of credit and peer-review.

Item 13: Journal aggregation and integration for enhancing the efficiency, access and impact of taxonomic publications
Zhi-Qiang Zhang
Taxonomic publishing faces both challenges and opportunities in this cyber era. To meet the challenges

of describing world's rapidly disappearing biodiversity, it is essential to enhance the efficiency, access and impact of taxonomic publications. One way this can be achieved is by taxonomic journal integration and aggregation. Sharing of resources and collaboration among partners are keys to success.

Item 15: abstract is missing

Item 15: A digitalization partnership: Sharing human and material resources
Larissa Smirnova, & Régine Fabri
The three Belgian institutions participating in BHL-Europe project present their experience in management of digitalisation and related activities. Examples of cooperation between institutions and relations with other digitalisation projects are given to show an efficient way to minimize the labour effort and to reduce the costs. The IPR policy of BHL-Europe is briefly explained and concrete use cases are discussed.

Item 17: Scientific publishing network after EDIT
Michèle Ballinger & Isabelle Gérard, RMCA
By bringing publishing representatives together (Paris, December 2008), the EDIT and the MNHN created the basis of our Network for exchanging knowledge and explore opportunities for collaboration in the domain of scientific publishing. Our second meeting held at Bratislava in June 2009 and strengthened the rising network. This meeting in Copenhagen follows the same goals and aims to build our future. We will explore several possible tracks in order to continue the actions of the network after 2011, year of the achievement of the EDIT consortium.

Annex 4 - break-out groups

Introduction of break-out groups: As part of the meeting, the audience has been split up into six small groups to brainstorm about different topics identified beforehand by the organisers. This session aimed to produce checklists about topics, summarising the state of the knowledge and providing a rapid overview for each subject. Attendees were asked to pick the subject they were most familiar with. The objective was to indentify key issues and short answers for publishing and library staff in NHIs which they can use in their day-to-day work. Each group appointed a rapporteur to present the group output in a plenary setting. Below follows a brief summary of the discussion for some of the subjects.

1. Economic models for scholarly journals –Moderator: *Laurence Bénichou*.

Participants: Mark Costello, András Gubyányi, Boris Jacob, Mark Patterson, Simon Tillier

1) the group identified the publishing costs then describe 3 different business models and gave, for each model their pros and cons.
2) Four different steps were identified as part of the publishing costs: the intellectual production of the article (its research, basically done by the researcher); the referee process; the editing; the dissemination.
3) In the traditional subscription model the library buys the journal from the publisher (most of the time commercial publisher), this is a pay-to-read model. In the pay-to-publish model, the author pays the publisher in order to make the article publicly available (OA). Finally, there is the in the institution-pay model. The producer (institutional publisher in that case) gives the journal for free in an exchange program or makes it available for free to all o the Web.

2. Library Exchange Programme – Moderator: *Graham Higley*.

Participants: Rudy Jocqué, Karina Bekhoei, Lars Björnshauge, HanneEspersen

The members of the breakout group compared experiences and agreed on the fact that all institutions were seeing a decline in the number of exchanges, but that rates were varying. Most of the institutions represented would probably need to stop exchanges in the foreseeable future, for cost reasons and because these would become meaningless in an e-publishing environment. The group recommend that the institutions form a working group to decide on a date when most institutions would stop most (90%?) exchanges. Giving partners a deadline would enable all to plan effectively and to enable a graceful end to exchanges. The end of 2013 was suggested as a possible end-date.
The group has also expressed its concern that a couple of physical copies of any title should be kept available within Europe for preservation and nomenclatural reasons (at least for a while). They therefore propose that the institutions look at the opportunity to develop an EU-wide stock-holding policy, such that an individual library (or libraries) would commit to housing the last physical copy of a title. This could be done in conjunction with US and other institutions to produce a global physical stock policy as the overwhelming majority of current titles become e-publications.

3. Dissemination of natural history books - Moderator: *Nigel Massen.*

Participants: Daphne Duin, Michiel Thijssen, Marine Danek-Gontard, Xavier Eekhout, Ole Karsholt

This group studied how to increase the dissemination of books published by natural history institutions and increase their (international) sales. First of all, they agreed on a list of prepublication decisions that influence the dissemination strategy. The potential market would differ considering the choice of the language of the publication, and an international market would only be suitable for a book published in English. The format (print offset, print on demand, or ebook) would also strongly influence the dissemination strategy. A book printed to a high print-run would imply to deal with a wholesaler whereas an ebook would imply a different channel for instance an ebook vendor. The core mission of dissemination of a natural history institutes also requires paying special attention to the archiving options of book publications. The publishing institute needs to guarantee that the book would still be available in the future.

4. Co-publishing – Moderator: *Isabelle Gérard*

Participants: Gwenaëlle Chavassieu, Danny Eibye-Jacobsen, YuriRoskov

The group has explored the main reasons for an institution to co-publish a title: increase its visibility in other (geographical or other) areas, share the risks and the production's cost (printing, photo's copyrights, etc.). The group then produced a checklist of what should any agreement include: description of the role of each partner in terms of copyediting, layout, copyrights managements, logos, legal deposit, dissemination, etc.

5. Copyrights and scientific publications – **Moderator:** *Ruth Linklater*.

Participants: Regine Weidner, Emmanuel Côtez, Wioletta Tomaszewska, Larissa Smirnova

The group aimed to define copyrights in scientific publishing context: What are different copyright models used (i.e. what rights are held by the author, by the distributor or publisher, and by the institution where the author is employed) in your publications? Give some examples of the obstacles of current copyright use in your publications. What is necessary to prevent copyright becoming an obstacle to the re-use of material for teaching and research?

6. Fast track publications – Moderator: *Zhi-Qiang Zhang*

Participants: Michèle Ballinger, Laszlo Peregovits, Andreas Zwick, Henrick E. Pedersen, Patrick Grootaert

The group questioned the editorial process of fast tract publications: What is a fast track publication? What are the benefits and for whom? Disadvantages and for whom? What do you need in terms of skills and tools to offer a fast track publishing option? Describe the workflow for the preparation of a fast track publication from a desk editor's perspective.

Annex 5 - Participant list

First name	Name	Institution
Henrik	Ærenlund Pedersen	National Museum of Natural History, Denmark
Connie	Baak	NCB Naturalis
Michele	Ballinger	Centre National de la Recherche Scientifique (CNRS)
Karina	Bekhoei	National Museum of Natural History, Denmark
Laurence	Bénichou	Muséum national d'Histoire naturelle, Paris
Lars	Björnshauge	SPARC-Europe
Gwenaelle	Chavassieu	Muséum national d'Histoire naturelle, Paris
Mark	Costello	Society for management of European Biodiversity Data (SMEBD)
Emmanuel	Cotez	Muséum national d'Histoire naturelle, Paris
Michael	Day	UKOLN, University of Bath
John	Doove	SurfFoundation, The Netherlands
Daphne	Duin	Muséum national d'Histoire naturelle, Paris
Xavier	Eekhout	Museo Nacional de Ciencias Naturales, Madrid
Danny	Eibye-Jacobsen	National Museum of Natural History, Denmark
Hanne	Espersen	National Museum of Natural History, Denmark
Regine	Fabri	National Botanical Garden of Belgium
Isabelle	Gerard	Royal Museum for Central Africa, Tervuren
Marie-Christine	Gontard	Muséum national d'Histoire naturelle, Paris
Patrick	Grootaert	Royal Belgian Institute of Natural Sciences, Brussels
András	Gubányi	Hungarian Natural History Museum
Kees	Hendriks	NCB Naturalis
Graham	Higley	Natural History Museum, London
Boris	Jacob	Museum für Naturkunde, Berlin
Alice	Jacobs	Royal Botanic Garden Edinburgh
Rudy	Jocqué	Royal Museum for Central Africa, Tervuren
Jörgen	Eriksson	University Lund, Nordbid
Ole	Karsholt	National Museum of Natural History, Denmark
Ruth	Linklater	Royal Botanic Gardens Kew
Nigel	Massen	NHBS Environment Bookstore
Mark	Patterson	Public library of Science (PLoS)
Laszlo	Peregovits	Hungarian Natural History Museum
Yuri	Roskov	Species 2000, Catalogue of Life
Larissa	Smirnova	Royal Museum for Central Africa, Tervuren
Michiel	Thijssen	BRILL
Simon	Tillier	Muséum national d'Histoire naturelle, Paris
Wioletta	Tomaszewska	Museum and Institute of Zoology, Poland
Regine	Weidner	Botanic Garden and Botanical Museum, Berlin
Zhi-Qiang	Zhang	Landcare Research, Auckland
Andreas	Zwick	Staatliches Museum for Naturkunde Stuttgart
