# Supplementary material for: PESI - a taxonomic backbone for Europe
Source: Biodivers Data J. 2015 Sep 28;(3):e5848. doi: 10.3897/BDJ.3.e5848 (PMC4609752; doi:10.3897/BDJ.3.e5848)
Supplement: Supplementary material 28 — A recommended design for “BiodiversityKnowledge”, a Network of Knowledge to support decision making on biodiversity and ecosystem services in Europe [file biodiversity_data_journal-3-e5848-s028.pdf]

A recommended design for "BiodiversityKnowledge", a Network of Knowledge to support decision making on biodiversity and ecosystem services in Europe

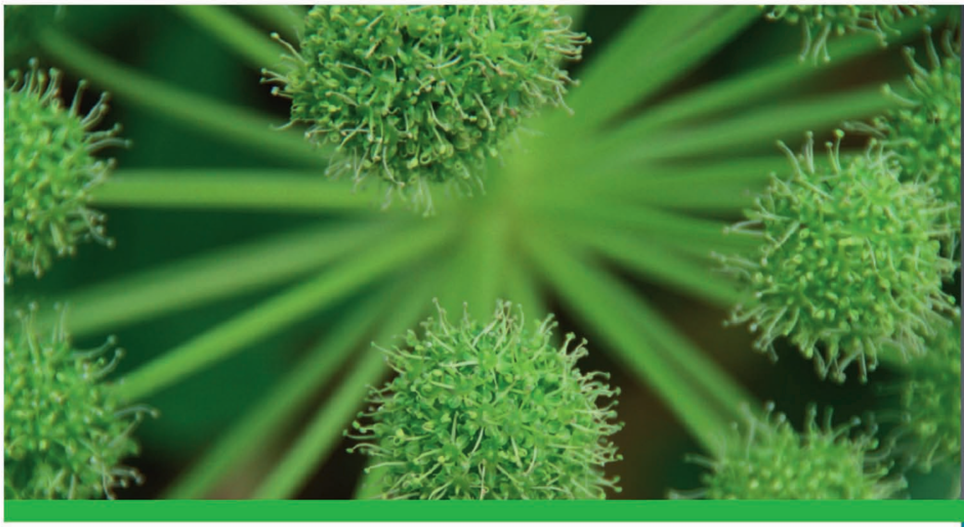
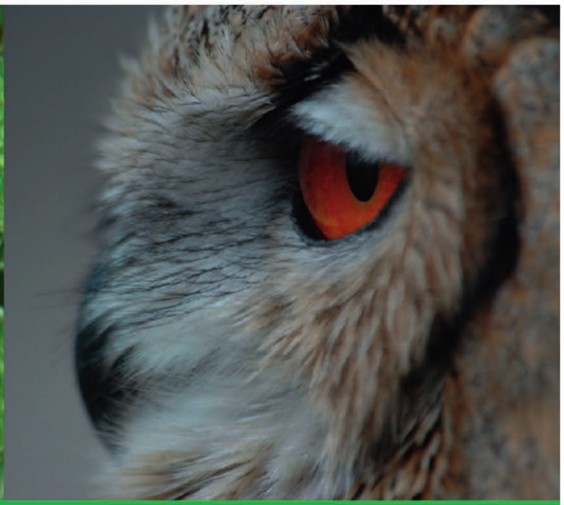

Prepared by the consortium of
the KNEU project, based on a broad
European consultation

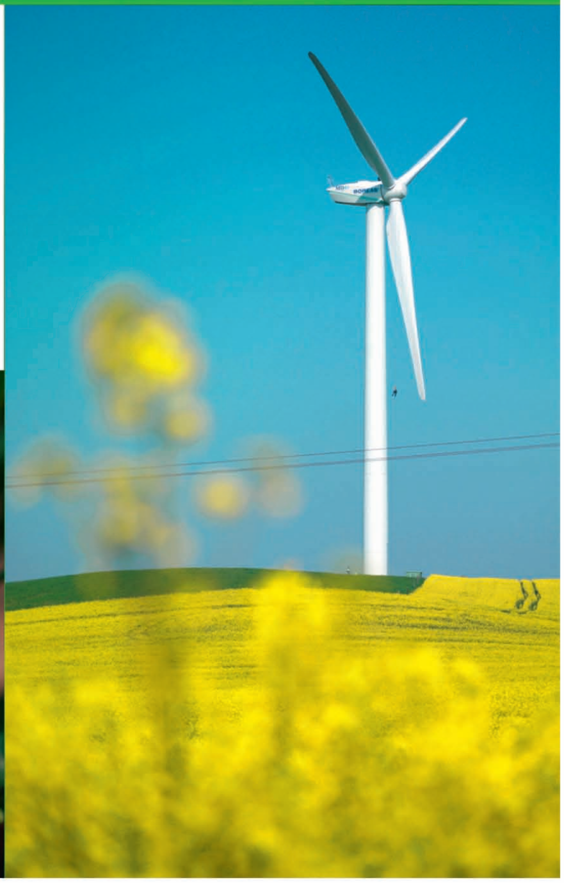
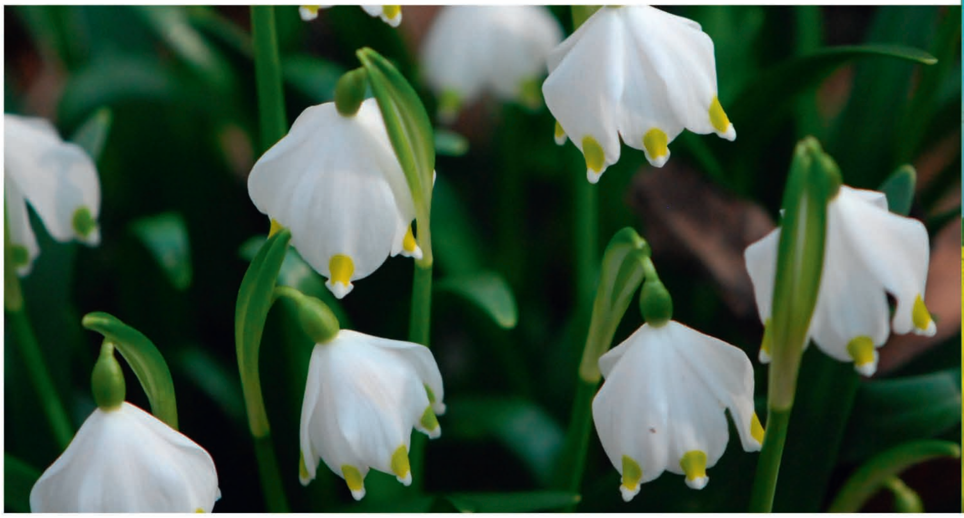

Creating a Network of Knowledge
for biodiversity and ecosystem services

www.biodiversityknowledge.eu

Biodiversity
Knowledge

ACKNOWLEDGEMENTS:

The EU funded Coordination Action KNEU (Grant No. 265299), which has the main aim to develop a European Scientific biodiversity Network of Knowledge to inform policy-making and economic sectors (ENV.2010.2.1.4.3-3) and whose consortium (see Annex 9) wrote the present document, would like to greatly thank all the participants to the project's processes, who attended the workshops in Budapest, Copenhagen, Aix-en Provence, Brussels, Wageningen, Glasgow, Ghent, Paris, Peterborough, Frankfurt/Main as well as to the BiodiversityKnowledge conferences in Brussels in May 2012 and Berlin, September 2013. Also, we thank those who have answered questionnaires, surveys, got involved in the project's demonstration cases, and provided support and feedback at different stages, including often detailed comments on earlier versions of this paper. Without their input, this paper would not have been able to supply so many details. This document has been continuously enriched through the exchanges between person from various backgrounds, expertise, visions, and different cultures.

Legal disclaimer:

The views expressed in this discussion paper, as well as the information included in it, do not necessarily reflect the opinion or position of the European Commission or its officials – who provided feedback and comments on earlier drafts – and, they in no way commit the Institution.

Preamble – some guiding words of explanation on this document

This paper is the **final draft version** of a "*recommended design for a Network of Knowledge to support decision making on biodiversity and ecosystem services in Europe"* – a potential instrument for improving the science-policy interface on biodiversity in Europe in the near future.

The final version of this paper, as a "white paper"[1], is the main deliverable of the EU funded Coordination Action KNEU (Grant No. 265299), whose main aim was to develop a European Scientific biodiversity Network of Knowledge to inform policy-making and economic sectors (ENV.2010.2.1.4.3-3).

An executive summary can be downloaded under this link:
http://biodiversityknowledge.eu/images/PDF/2013-08-01-WhitePaper-Summary.pdf

Definition of main terms and concepts can be found under this link and in Annex 2:
http://biodiversityknowledge.eu/faq

[1] Document intended to help readers understand the issue of a Network of knowledge on biodiversity and ecosystem services. It is not an official or legally binding document as the term 'white paper' might suggest.

Short explanation of context

For the sake of clarity, we briefly introduce the three levels of activities representing the context of this document: 1) The FP7 funded project to develop a possible design of a Network of Knowledge (NoK) on biodiversity in Europe, i.e. the KNEU project, 2) the product of the KNEU project; the recommended design of a Network of Knowledge (NoK) named BiodiversityKnowledge and finally 3) the wider context justifying the design of BiodiversityKnowledge; i.e. a potential EU mechanism on biodiversity expertise[2]. **The main focus of the paper is to discuss, the NoK BiodiversityKnowledge** but the other levels will be mentioned in this paper – KNEU as it delivered many additional inputs for this paper by its case studies and workshops, and the EU mechanism context as it frames the role of the NoK on the policy side:

Project: KNEU

The FP7 funded KNEU project (2010-2014) is our working environment to develop the lessons learned and a recommended design for a Network of Knowledge on Biodiversity and Ecosystem Services. Eventhough comprising 18 European institutions, the project is designed to interact more broadly with the whole community of knowledge holders on biodiversity and ecosystem services. To facilitate this discussion, the KNEU project set up the website www.biodiversityknowledge.eu and conducted two conferences and other meetings involving a high number of experts.

↓

Product: BiodiversityKnowledge

The Network of Knowledge (NoK) we name *BiodiversityKnowledge* and its recommended design and operationalisation for the future is the main product of the KNEU project, embedded in the specific European context of biodiversity science and policy. The BiodiversityKnowledge recommended design and operationalisation, documented in this paper, is based on a prototype that was developed, broadly discussed and applied in demonstration cases within the KNEU project in 2011/2012[3].

↓

Context: an EU mechanism for biodiversity expertise

The wider policy context of the NoK approach is linked to the process towards an **"EU mechanism on Biodiversity Expertise",** as officially referred to in the Biodiversity 2006 Communication and Action Plan[4] and the building of the biodiversity knowledge base as in the EU Biodiversity Strategy to 2020. In addition, current initiative related to the target 2 Action 5 of the Biodiversity strategy, namely the MAES process[5] on Mapping and Assessing Ecosystem Services, also provided insights throughout the project work. As demonstrated by the high level of interest and current related activities, the organization of European expertise is expected to be valuable in the context of the IPBES[6] development.

---

[2] http://ec.europa.eu/environment/nature/biodiversity/comm2006/index_en.htm

[3] See deliverable 3.1 of the KNEU project (downloadable from www.biodiversityknowledge.eu)

[4] http://ec.europa.eu/environment/nature/biodiversity/comm2006/bap_2006.htm

[5] The EU Commission jointly with the Member States started a major process towards Mapping and Assessing Ecosystems and their Services (MAES), see http://biodiversity.europa.eu/ecosystem-assessments

[6] Intergovernmental Platform on Biodiversity and Ecosystem Services, see http://www.ipbes.net

A recommended design for "BiodiversityKnowledge", a Network of Knowledge to support decision making on biodiversity and ecosystem services in Europe

Draft prepared by the consortium of the KNEU project (See Annex 9), based on a broad European consultation[7].

In order to develop and discuss the concept for BiodiversityKnowledge as open and transparent as possible, this paper is issued from a series of consultations at a larger scale than the KNEU consortium itself:

- September 2012: first document draft prepared by the team of WP5 & 2 of the KNEU project, based on the work done in WP1, WP2 (Deliverable D.2.1), including the discussions at the first project conference in May 2012 and numerous workshops with experts and their specific feedback via the evaluation work package (WP4)
- October 2012: First draft discussed within KNEU consortium
- November 2012: revised first draft discussed with stakeholders in Dialogue Group,
- April 2013: Development of second draft, completely revised and more focused to key functions of the NoK
- April 2013: Consultation on second draft with Dialogue Group and with WP2, 3 and 5 of the KNEU team
- July 2013: revision second draft and consultation within the whole KNEU consortium
- August 2013: Development of third draft and launch for open consultation, including direct feedback from institutions and use of workshops to discuss specific elements of the Biodiversity Knowledge structure
- September 24-26, 2013 Berlin: 2nd BiodiversityKnowledge conference for final discussions
- January 2014: specific additional workshops to specify issues on methods & questions in policy support function (see chapter 3.3) and governance models (see chapter 5), wrap-up workshop of core team to derive NoK recommended design (chapter 5.6)
- March 2014: final consultation
- April 1, 2014 Brussels: European parliament Science Policy Society conference "Towards a consolidated Network of Knowledge on biodiversity and ecosystem services in Europe"
- April 2014: Final concept and communication of results

7 The KNEU project consulted more than 300 individuals and organisations through the organisation of workshops, focus groups and conference side events as well as through interviews with stakeholders.

Content

1 Aim and approach of the concept paper 9

1.1 Aim of the concept paper 9
1.2 Approach to serve different needs 9

2 Background 11

2.1 The European biodiversity knowledge landscape – Needs assessment 11
2.2 The global context – IPBES 14
2.3 The potential functions of a knowledge-policy interface on Biodiversity for Europe – The 'EU mechanism' discussion 15

3 "BiodiversityKnowledge": A proposal to address networking and policy support 19

3.1 Introduction 19
3.2 Networking and capacity building function (-NET): Building a responsive community 20
3.3 Answering-decision-making-needs function (ADN-function) 24
3.4 Showcasing the pathways for decision support through the NoK 31
3.5 Conclusions: The Network of Knowledge as science-driven part of the wider science-policy interfaces 34

4 Ability of a Network of Knowledge to deliver relevant products while ensuring credibility and legitimacy 35

4.1 Quality assurance 36
4.1.1 Challenges 36
4.1.2 Lessons learned 38
4.1.3 Added values of a NoK 39

4.2 Data standards, data sharing & exchange and methods to analyse them 40
4.2.1 Challenges 40
4.2.2 Lessons learned 40
4.2.3 Added value of a NoK 41

4.3 Connecting, motivating and acknowledging the actors 43
4.3.1 Challenges 43
4.3.2 Lessons learned 44
4.3.3 Added value of a NoK 46

4.4 Communication 47
4.4.1 Challenges 47
4.4.2 Lessons learned 47
4.4.3 Added values of a NoK 49

4.5 Capacity building 49
4.5.1 Challenges 49
4.5.2 Lessons learned 50
4.5.3 Added value 51

5 BiodiversityKnowledge in more detail: guiding principles, potential models and a recommended governance design ... 53

5.1 Key principles of BiodiversityKnowledge ... 53
5.1.1 The mission of BiodiversityKnowledge: ... 53
5.1.2 Principles of BiodiversityKnowledge ... 54

5.2 Gradients for the operationalization of the NoK ... 54

5.3 Roles of knowledge providers and policy ... 56
5.3.1 Overview of roles of knowledge providers ... 56
5.3.2 Link to European policy and decision making: mandated but independent ... 58

5.4 Potential building blocks for a governance structure of the NoK ... 59
5.4.1 Minimum set of building blocks ... 59
5.4.2 Additional building blocks: different options for operationalising ... 61

5.5 Options for the NoK design ... 63
5.5.1 NETWORK MODEL: based on individual commitment and self-organisation ... 63
5.5.2 PLATFORM MODEL: institutional commitment and clear allocation of roles ... 67

5.6 Recommended NoK design: balancing structural reliability and engagement ... 71
5.6.1 Assumptions used when developing the recommended design: ... 71
5.6.2 Governance of the recommended design for the NoK ... 72
5.6.3 Links to decision-making ... 74

5.7 Finances ... 75
5.7.1 Possible models of financing ... 75
5.7.2 Estimation of financial needs of the recommended NoK design ... 77

5.8 Road map for NoK implementation: timeline, actors and stepwise approach ... 79

A final note by the KNEU team ... 81

ANNEXES ... 83

Annex 1: Acronyms used ... 84
Annex 2: Glossary ... 87
Annex 3: Detailed outline of the prototype to answer decision making needs ... 89
Annex 4: Elements and criteria for initial request form for the NoK ... 89
Annex 5: Matrix of potential types of requests posed to the NoK and suitable of different methods to address them ... 90
Annex 6: Elements for expert selection criteria ... 93
Annex 7: List of potential contributions and benefits of institutions, as outlined at the 2nd BiodiversityKnowledge conference ... 93
Annex 8: Comparison table of use of building blocks in different models and recommended NoK design ... 94
Annex 9: Partners of the KNEU project ... 98

1 Aim and approach of the concept paper

1.1 Aim of the concept paper

The aim of this paper is to present a recommended design of a Network of Knowledge (NoK) for European expertise on biodiversity and ecosystem services (*BiodiversityKnowledge*) to inform *decision making*[8], including policy making and economic sectors.

In this paper we successively tackle the following aspects:

→ outline the **background and context** for the NoK (chapter 2)

→ discuss **potential functions for a NoK** (chapter 3)

→ outline the **challenges, lessons learned and added values of a NoK** (chapter 4)

→ derive main options for the design of the NoK and its **potential governance structure, rules and procedures** for operating it (chapter 5)

The contents of this paper are based on the work and analysis undertaken in the KNEU project, complemented with a literature review and the findings from the SPIRAL project[9] . It also benefits from suggestions collected via interviews and workshops with scientists and policy makers during the KNEU project, with a special emphasis on those issued from the demonstration cases of KNEU carried out during May 2012 to May 2013.

> The ideas presented are thus the result of the broad engagement of more than 300 individuals and organisations into earlier discussions on the prototype NoK concept, interviews with stakeholders and the demonstration cases participants. In order to make those contributions visible, summaries of them are included in boxes throughout the document.

For shortness of the paper, we are using acronyms after their first introduction (for easy reference, see Annex 1). Annex 2 in addition gives a glossary of major terms used in this document, yet these definitions can also be found in the document.

1.2 Approach to serve different needs

In compiling this paper, the project faced a major challenge in serving the various points of view of stakeholders involved in the knowledge-policy interface[10] on biodiversity and ecosystem services in Europe. Science policy interfaces, their functions, and the cost-benefits of different SPI-models are still subjects of research (e.g. SPIRAL project results[6]). The KNEU project took into account the latest findings on these issues, but also acknowledges that the matter is evolving and that the related understanding is non-exhaustive. In this context and as the Network of Knowledge is per essence building on a large variety of organisations, the project aimed to:

---

[8] See glossary (Annex 2) for explanation of main terms in the context of this white paper

[9] SPIRAL: Science-Policy Interface on biodiversity – Research, Action and Learning (contract No. 244035), see www.spiral-project.eu (2010-2013)

[10] Today, the commonly used term is still "science-policy-interface", although the term "knowledge-policy-interface" is more adequate to the objectives and approaches of a Network of Knowledge, as it recognizes that different forms of knowledge (including science) are relevant for policy and decision-making. As we didn't want to introduce such a new term explicitly, we keep using the term "science-policy-interface", but will stress the role of different forms of knowledge in the interface regularly.

- identify the *facts* on science-policy interfaces and potential options for their organization/governance
- identify the *interests* of all potential knowledge holders and requesters and their institutions, in order to acknowledge their role in the biodiversity SPI for the purpose of being as inclusive as possible.

Finally, being at the cross-road of policy, science and other stakeholders implies that many various values are at stake and require compromises. The project then also aimed to:

- take into account the *values of* potential knowledge holders and requesters regarding the science-policy interface (e.g., a policy maker may focus on relevance and the "added-value" to the current situation, a scientist may focus on credibility), but also when addressing the topic of biodiversity and ecosystem services and the different values assigned to them by different actors in the field (see Box 01)

The proposal for BiodiversityKnowledge is an attempt to address this complex set of expectations, needs and values but of course represents a compromise with potential strengths and challenges. The KNEU project team nonetheless is confident that the paper successfully demonstrates that BiodiversityKnowledge has clear added-values to improve the way knowledge and decision-making interact in the multi-level governance of biodiversity and ecosystem services in Europe for us all, the "Biodiversity knowledge community".

2 Background

2.1 The European biodiversity knowledge landscape – Needs assessment

The need for better informed decision making, especially in the environmental sector has gained increased recognition over the last decade, and has recently been outlined again in the proposal for the 7th Environmental Action Programme[11]. With increasing complexities in the sector, the risks of making inadequate and/or contested decisions increases as do the risks of not properly implementing policies and thus not achieving their targets. This calls for a more reflexive involvement of the evidence-base into the design and the implementation of decisions, and consequently for more credible, relevant and easily accessible knowledge. The field of biodiversity and ecosystem services and its development over the last decades is especially challenging in this respect (See Box 01).

Discussions with policy makers and other stakeholders suggest that three concrete needs exist where decision making could profit directly from an improved scientific input:

- The joint formulation of questions building on an integral and more holistic understanding of all relevant factors should identify distinct policy-relevant questions that science and other forms of relevant knowledge[12] is able to address and provide concrete answers to;
- A better understanding of concrete policy impacts on the ground, to allow for the development of implementation-oriented concrete proposals for tools and options to bring about desired change in practice;
- Coherent and independent analysis able to inform, raise awareness and trigger action beyond the environmental sector, in all relevant policy domains.

Parts of these needs are addressed by EU institutions from a policy as well as research policy perspective. On the policy side, for example, the role of the European Environment Agency was strengthened, including its leading role in setting up and further developing the Biodiversity Information System Europe (BISE).

---

[11] See http://ec.europa.eu/environment/newprg/proposal.htm

[12] When talking about "knowledge", this document often refers to "science and other forms of knowledge" in order to stress the fact, that often, scientific knowledge is not sufficient to address specific questions. If the document sometimes refers to "science" alone, this doesn't mean that other forms of knowledge are excluded in that specific context, see also definitions in Annex 2.

Box 01: Challenges in tackling the field of conservation, biodiversity, ecosystem services and natural capital at the interface between policy and science

The area of nature conservation has undergone major changes in its conceptual basis, in science as well as society over the last 20 years. With the success of "biodiversity" as major concept and its political implementation in the Convention on Biological Diversity (CBD), classical conservation concepts (and their underlying values) have been opening up and now include sustainable use of natural resources, which are tightly linked with the concept of ecosystem services and, more recently the concept of natural capital (for a reflection, see Sharman & Mlambo 2012).

The CBD itself shows this development with including use-perspectives and the terms of ecosystem services and natural capital very strongly into its recent Strategic Plan for 2020. So does the European Biodiversity Strategy for 2020.

As this shift changes the underlying rationale for environmental policy (see for example Spierenburg 2012, Jax et al. 2013, Turnhout et al. 2013), it holds some dangers for classical approaches, while at the same time allowing for a stronger mainstreaming of environmental policies in other sectors.

When further developing the science-policy interface in this field, as proposed in this paper, this holds the challenge of supporting both strains of rationale for policies – the classical ones focusing on nature conservation and biodiversity, which is more based on intrinsic values and the new services-centred one, using a utilitarian point of view. This means that questions to be tackled at the interface always need to reflect on both these perspectives. Thus the approach of the interface must really be multidisciplinary, reaching out to scientific and other forms of knowledge which normally serve other policies (e.g., economics, agricultural research and many more) and are based on other forms of experiences, e.g. in practical management of natural resources.

The following questions illustrate some of the questions a NoK could address:

- How do changes in the diversity and abundance of pollinators in Europe relate to factors such as use of pesticides, landscape attributes, parasites and other factors?
- What is the relationship between public health and aspects of global change (including changes in biodiversity) in Europe?
- What are the potential consequences of climate change in Europe on the current legislation in nature conservation (Birds and Habitats Directive)?
- Ecosystem restoration: How to balance the goals of service provision and nature conservation in restoration efforts across European ecosystems?

<u>Further reading:</u>

Sharman, M. & Mlambo, M.C. (2012): Wicked: The problem of biodiversity. Gaia 21: 274-277.

Spierenburg, M. (2012): Getting the Message Across Biodiversity Science and Policy Interfaces A Review. GAIA 21: 125-134.

Jax, K.; Barton, D.N.; Chan, K.M.A.; de Groot, R.; Doyle, U.; Eser, U.; Görg, C.; Gómez-Baggethun, E.; Griewald, Y.; Haber, W.; Haines-Young, R.; Heink, U.; Jahn, T.; Joosten, H.; Kerschbaumer, L.; Korn, H.; Luck, G.W.; Matzdorf, B.; Muraca, B.; Neßhöver, C.; Norton, B.; Ott, K.; Potschin, M.; Rauschmayer, F.; von Haaren, C. & Wichmann, S. (2013): Ecosystem services and ethics.- Ecological Economics 93: 260-268.

Turnhout, E., Waterton, C., Neves, K. and Buizer, M. (2013): Rethinking biodiversity: from goods and services to "living with". Conservation Letters, 6: 154–161.

On the research policy side, several initiatives were launched to stimulate research and research infrastructures. Biodiversity research funded through the EU's Framework Programmes, which include more than 80 projects in the last 10-15 years has become increasingly linked to policy needs, *for example*[13] :

- The Networks of Excellence ALTER-Net (terrestrial biodiversity), MARBEF (marine biodiversity, now EuroMarine) and EDIT (taxonomy)
- LifeWATCH as a joint Infrastructure supported by these networks
- The ERA-Net BiodivERsA as programme for integration of funding activities for research among member states
- The EU-funded project on Building the European Biodiversity Observation Network (EUBON) and its predecessor, the European Biodiversity Observation Network (EBONE), that inter alia are aiming to contribute to the GEO BON initiative,
- large scale EU projects like ALARM, BIOFRESH, SCALES, TESS, OpenNESS and OPERAs

to name just the few large initiatives and projects over the last years.

Besides, many other stakeholders are increasingly engaging in an active exchange with policy on issues of biodiversity and ecosystem services: e.g. learned societies (e.g. European Ecological Federation (EEF), the Society for Conservation Biology (SCB)), NGOs (e.g. BirdLife, WWF, EEB), the private sector, etc. On the international level, the Future Earth programme set up by the International Council for Science (ICSU) might support and link up with these activities and institutions.

Despite these efforts, concise and consolidated knowledge often remains difficult to access for potential users. To facilitate this access for all the mentioned players is the main purpose of a Network of Knowledge. It needs to build on these different kinds of contributions and ensure that the potential benefits for institutions and individuals can be achieved when they engage with the NoK (See Annex 7 for a list of potential contributions and benefits of institutions presented at the second BiodiversityKnowledge conference).

Although a general overview exists, access points to knowledge are still scattered and poorly organised across disciplines and institutions (see Box 02). For many biodiversity topics, scientific knowledge alone is not always sufficient to provide answers to specific policy and management questions. Practical and local place-based knowledge, including 'Traditional Ecological Knowledge' (TEK) may also need to be included, especially when it comes to implementation and management decisions on the regional and local scale[14]. How to access and integrate practical and local knowledge remains a challenge, as re-

[13] For a complete list of relevant projects, see
http://ec.europa.eu/research/environment/index_en.cfm?pg=projects&area=bio&fptab=fp7&fp7page=all
http://www.edinburgh.ceh.ac.uk/biota/

[14] According to Tengö et al. there are at least three ways of connecting knowledge systems: integration, where components of one knowledge systems incorporated into another through a validation process; parallel approaches placing knowledge systems next to each other, using separate validation mechanisms and emphasizing complementarity; and co-production of knowledge, where representatives from different knowledge systems are engaging in mutual processes of knowledge generation, see Tengö, M.; Malmer, P.; Brodizio, E.; Elmqvist, T.; Spierenburg, M. (2013): Discussion paper: The Multiple Evidence Base as a framework for connecting diverse knowledge systems in IPBES.- http://bit.ly/1cFkMP1

cently outline by a review on TEK in Europe[15]. However, as the KNEU project and its demonstration cases have shown, this challenge might be easier to address in Europe, with its broad networks of practitioners, NGOs and expertise in administrations compared to the global scale.

BOX 02: Where is knowledge on Biodiversity in Europe? How does this knowledge flow?

Within KNEU, the complex task of mapping the knowledge landscape on biodiversity in Europe was undertaken to create an overview of expertise and stakeholders on biodiversity and ecosystem services knowledge in Europe. The original aim was to identify candidates for permanent knowledge hubs for a NoK that can provide timely evidence-based answers to topical questions. However, the exercise inevitably demonstrated that in order to build a network of knowledge in Europe, we need to first understand the flows of knowledge within Europe, i.e. where is knowledge coming from, where does it go, where it might be hidden and who is playing a key role in this knowledge landscape. In order to establish the flows while highlighting biodiversity knowledge hubs we have interviewed persons working with biodiversity issues, using the interview-based mapping tool called Net-Map (Schiffer and Hauck, 2010) as a directive. In total 44 persons were interviewed, working in a very broad range of disciplines all related to biodiversity; e.g. practitioners, researchers, environmental lawyers, policy makers, etc. The resulting map of knowledge flows shows actors which are key providers of knowledge, actors which are key requesters as well as actors which are playing a key role in the knowledge flow paths, i.e. they are relaying knowledge. Those latter connecting actors for example include IUCN, the European Commission, the EEA or the recently released and quite influential TEEB initiative. This continuous Net-Mapping exercise contributes to the building of a responsive community as developed later in this document in chapter 3.2 as it highlights not only the key players in the knowledge landscape but also connection gaps hindering the knowledge flow.

2.2 The global context – IPBES

At the global level, efforts by the international community to operationalize the Intergovernmental science-policy Platform on Biodiversity and Ecosystem Services (IPBES) have led to its official launch in April 2012[16]. The first meeting of the plenary in January 2013 took first steps in developing the work programme and set up its bodies, including a Bureau to guide the work in between plenary session and a Multidisciplinary Expert Panel to guide the scientific work. The second plenary in December 2013 in Antalya adopted the work programme including a number of assessments to start already in 2014. With the secretariat of IPBES being located in Bonn, Germany, Europe will be expected to bring its broad expertise on biodiversity and ecosystem services into this process (see Box 03).

There is a common agreement that many topics related to biodiversity and ecosystem services need to be tackled on the regional[17], national and even local level and that these scales need to be taken into account in global efforts. Accordingly, a support of IPBES work from the regional level (namely the EU or from a pan-European perspective) could ensure higher regional relevance and implementation of the outcomes. Currently, no specific structure is available to serve such a support function and how this

[15] Hernández-Morcillo M., Hoberg, J., Oteros-Rozas, E., Plieninger, T., Gómez-Baggethun, E. & Reyes-García, V. (2014): Traditional Ecological Knowledge in Europe: Status Quo and Insights for the Environmental Policy Agenda. *Environment: Science and Policy for Sustainable Development* 56, 3-17, DOI: 10.1080/00139157.2014.861673

[16] For more details on IPBES, please visit www.ipbes.net

[17] Please note, that in the context of global UN-related activities, “regional” addresses the scale of continents or biomes, so that “national” is below this level. In the EU context, “regions” refer to the sub-national level.

challenge of a regional support to IPBES can be tackled is currently an open issue in Europe. Chapter 5.4 describes the potential role of the NoK as a regional support body for IPBES.

BOX 03: The functions of IPBES and the objectives of its work programme 2014-2018

The multi-stakeholder-conference in Busan 2011, preparing the launch of IPBES, decided that IPBES should serve four different functions*:

1. perform regular and timely assessments of knowledge on biodiversity and ecosystem services and their inter-linkages, at appropriate scales and including thematic issues;
2. Promote access to, and development of policy-relevant tools and methodologies;
3. Prioritize and enable key capacity-building needs to improve the science-policy interface at appropriate levels;
4. identify and prioritize key scientific information needed for policymakers, and catalyse efforts to generate new knowledge

These functions align in parts with the functions 1-3 introduced for BiodiversityKnowledge in chapter 2.3, where 'regular assessments' and 'promoting access to relevant tools and methodologies' are included in the "Answering-decision-making-needs" function.

To translate these functions into concrete activities, the first IPBES work programme derives four main objectives from these functions:

1. enhance the enabling environment for the knowledge-policy interface for biodiversity and ecosystem services;
2. strengthen the knowledge-policy interface on biodiversity and ecosystem services at and across subregional, regional and global levels;
3. strengthen the knowledge-policy interface on biodiversity and ecosystem services with regards to thematic and methodological issues;
4. communicate and evaluate IPBES activities, deliverables and findings.

** These functions are defined in paragraph 1 in Appendix I to Annex I in UNEP/IPBES.MI/2/9, specifically in sub-paragraphs (b) to (e)*

*** see first work programme of IPBES UNEP/IPBES/2/4, available at www.ipbes.net*

2.3 The potential functions of a knowledge-policy interface on Biodiversity for Europe – The 'EU mechanism' discussion

There are a lot of organisations, institutions and working groups that already support the exchange of knowledge between science and policy, and with IPBES developing on the global scale, it is important to identify potential gaps and avoid overlap when establishing further institutions for the science-policy interface in Europe. In the 2006 EU Biodiversity Action Plan, under the heading "*To substantially strengthen the knowledge base for conservation and sustainable use of biodiversity, in the EU and globally*", the need was stressed for an "*EU mechanism for independent, authoritative research-based*

advice to inform implementation and further policy development"[18] which should be able to deliver a consolidated view from science (and other knowledge) to inform policy making.

Since then, analyses have been carried out at an international scale for IPBES[19], and at a European scale on what the concrete functions of such a mechanism could be, and how science and all knowledge holders could best contribute to it.

Potential functions at the science-policy interface in Europe: Four main functions have been identified[20] by the work of KNEU that would serve different purposes and also would need to complement each other as well as complement the existing institutions, thus adding "oil in the system" for an improved functionality in the science-policy landscape:

1. **a Networking and capacity building function (NET)**, to better network existing knowledge holders and their knowledge as basis to improve access to this knowledge. Networking here is understood in its broad sense and includes a strong element of capacity building activities to strengthen the community of knowledge holders and their ability to participate in the processes of the following functions.
2. **an Answering-Decision-making-Needs function (ADN),** to improve the support of decision making through the provision of relevant knowledge on a request driven basis with tested methods and protocols. The objective is to provide consolidated views on specific topics and to make use of all relevant types of knowledge including practical and local knowledge.
3. **a Research Strategy function (RS),** to identify policy-relevant research gaps and how the research landscape could be used to address them (see Box 04)
4. **an International Collaboration function (IC),** to use and feed the European knowledge into international science-policy processes like IPBES or SBSTTA-CBD, as well as foster European links to global research efforts (see Chapter 2.2 for a short introduction)

Several institutions in Europe are already contributing to each of these functions to some extent or at least working in such a direction, like the European Platform for Biodiversity Research Strategy (EPBRS) and BiodivERsA for the research strategy function (see Box 04). This implies that any concrete operational model for the EU Mechanism will need to be based on or linking with these existing initiatives to avoid duplication and to streamline efforts.

What is currently lacking nonetheless is an enabling environment of better structured interactions acknowledging the roles of existing knowledge holders and organizing the knowledge flow between actors by a targeted, integrative approach, bringing today's possibilities of networking and up-to-date methodologies on knowledge assessments together and aligning them with the needs from the different actors.

---

[18] See http://eur-lex.europa.eu/LexUriServ/LexUriServ.do?uri=COM:2006:0216:FIN:EN:PDF COM (2006) 216 final, p.13.

[19] See "Gap analysis Gap analysis for the purpose of facilitating the discussions on how to improve and strengthen the science-policy interface on biodiversity and ecosystem services" (UNEP/IPBES/2/INF/1)

[20] See for example the discussions and presentations of the first BiodiversityKnowledge conference: http://biodiversityknowledge.eu/index.php?option=com_content&view=article&id=32

BOX 04: Developing the research strategy in Europe: EPBRS and BiodivERsA

The research strategy function has mainly been facilitated since 1999 by the EPBRS (European Platform for Biodiversity Research Strategy), with support from the EU projects BIOPLATFORM and BIOSTRAT. The effectiveness of EPBRS to bring together scientists as well as policy makers and other stakeholders from many Member States at focused meetings and via electronic conferences has helped considerably to derive the European research agenda on biodiversity. This included a significant number of specific recommendations on different issues, with some of them resulting in concrete project calls over the last ten years. Also, EPBRS developed a framework document for a European Biodiversity Research Strategy for 2020 and laid the foundation for the concept of a network of knowledge on biodiversity, which is explored in the KNEU project.

Such an integrative function for identifying knowledge needs from a broader policy perspective will still be needed in the future, for example in serving the forthcoming Horizon 2020 programme and other funding schemes for implementing the research strategy. In this context, also other networks play an important role, e.g. in linking the European and the national funding perspectives, as done for example in the ERA-Net BiodivERsA. The proper identification of knowledge gaps and needs is of major importance for BiodivERsA (and other ERA-Nets) when updating its strategic agenda to further integrate national research programmes on biodiversity and ecosystem services across European countries. Also, ERA-Nets can make links to other areas and networks in science, for example, BiodivERsA organized a joint call together with the Joint Programme Initiative FACCE on Agriculture, food security and climate change.

For more information on EPBRS: www.epbrs.org

For more information on BiodvERsA: www.biodiversa.org

In the scope of this document, we focus on the first two functions, the networking and the answering-decision-making-needs function, that were identified as the main functions needed to strengthen the knowledge flow and address the basic needs identified in chapter 2.1. In addition, the link to the research strategy function is discussed and included in the discussion of potential design options, as a close link between the functions has been identified as major need by policy-making.

3 “BiodiversityKnowledge”: A proposal to address networking and policy support

3.1 Introduction

Processes at the interface of science and policy can have very different structures and approaches. Very generally speaking, these processes can be mainly driven by policy (e.g. expert panels set up on a specific topic) or by science (e.g. policy support work via applied research projects or via learned societies), accordingly, the diversity of approaches is high[21]. All science-policy interfaces (SPIs) face a joint problem: the challenge of finding the right balance between ensuring credibility, legitimacy and relevance. Box 05 summarizes this challenge and chapter 4 further elaborates it.

BiodiversityKnowledge is an attempt from the science community to self-organize and better integrate other forms of knowledge (including the private sector) in order to improve the capacity to respond to knowledge demands from decision-making. It is driven by science and other knowledge holder institutions and aims to ensure the credibility and legitimacy of the knowledge used and the contributing knowledge holders. In order to make it relevant for policy and other decision-making processes, it nevertheless needs to include elements that link up directly with policy – both thematically as well as within its governance structure (see chapter 5 for details).

BOX 05: The balance between credibility, relevance and legitimacy in SPIs – insights from the SPIRAL project

Credibility, relevance and legitimacy (CRELE) are attributes which can explain the influence and impact of SPIs.

- Credibility is the perceived quality, validity and scientific adequacy of the people, processes and knowledge exchanged at the interface;
- Relevance is the salience and the responsiveness of the SPI to policy and societal needs;
- Legitimacy includes the perceived transparency and the balance of perspectives within SPI processes.

These CRELE attributes are widely accepted and used, and can explain an SPI’s influence. The Intergovernmental Science-Policy Platform on Biodiversity and Ecosystem Services (IPBES, http://www.ipbes.net/) considers the CRELE attributes as important. The Intergovernmental Panel on Climate Change (IPCC) uses CRELE to evaluate scenarios, draw lessons from past experiences and explain assessments’ influence.

Building credibility, relevance and legitimacy into SPI design is key to ensuring impact. But SPIs have to work with numerous constraints (resources, time, policy cycle and so on), and it is not always possible to enhance all aspects of CRELE. Though it may be tempting to focus on the immediate policy challenges, it is important to consider not just short-term improvements in CRELE, but also the long-term prognosis. CRELE takes time to build, but can be lost very quickly. SPIs need to make strategic choices regarding what dimension of CRELE to emphasize and what specific features to prioritise to ensure high impact over the long term. There is no ‘one size fits all’ recipe: the right balance of features will vary according to the context.

Sources: SPIRAL briefs: Keep in CRELE: credibility, relevance and legitimacy for SPIs: http://www.spiral-project.eu/sites/default/files/07_Keep-it-CRELE.pdf and CRELE Choices: trade-offs in SPI Design: http://www.spiral-project.eu/sites/default/files/13_Brief_CRELE-choices.pdf

[21] For more details on the different forms of SPIs, see SPIRAL (2012): a study on the landscape of science-policy interfaces: http://www.spiral-project.eu/sites/default/files/SPIRAL_1-2.pdf

The Network of Knowledge approach, as proposed here, takes advantage and acknowledges the situation described above in offering an open and transparent process for better interlinking knowledge provision and knowledge needs. Today, consolidated views from science (and other forms as knowledge) are often lacking in discussions, and it is not clear, where knowledge comes from, what its uncertainties are, and whether the process compiling it has been credible and inclusive. Knowledge holders, especially from science want to ensure that the credibility of science (and of the persons and institutions involved) is ensured when getting engaged and that their efforts result in relevant outcomes.

These challenges and concerns call for a participatory, transparent approach which not only identifies a credible way to conduct assessments of knowledge, but also acknowledges the challenge of transparency in its processes and an engagement strategy not restricted to specific institutions, disciplines or forms of knowledge.

Accordingly, BiodiversityKnowledge puts emphasis on the NET-function for the benefit of the knowledge community as well as the ADN-function to concretely address the needs from decision-making, using the community brought together by the NET-function as foundation.

The following sections outline what these two functions need, which building blocks already exist, and how BiodiversityKnowledge could complement them.

3.2 Networking and capacity building function (-NET): Building a responsive community

Reliable and rapid access to existing information, knowledge, and expertise is not always available and/or sufficient for some of the needs expressed by decision-makers from different levels and institutions. Also, depending on their position, they require different kinds of information and knowledge (see Box 06). Interviews on knowledge needs conducted within KNEU show, that an **internet-based "one-stop-shop" or portal as entry point** to this always evolving knowledge is considered very helpful but is not available so far (see Box 06).

On the other hand, building a responsive community, a **Community of Interest**[22], goes far beyond simply setting up a platform. Needs for knowledge as well as the benefits of getting involved should be actively communicated. These include a better understanding of the policy relevance of many research topics, facilitated networking, and the possibility of gaining acknowledgment for providing personal expertise. The KNEU project has shown that in order to build and activate such a community, a need for **Capacity Building** on the science-policy-interface was expressed to enable experts from different regions and backgrounds to actively participate in the activities of a NoK.

With regard to a central entry point, the Biodiversity Information System Europe (BISE), established in 2010, is an important starting point for such a portal, but it currently lacks an explicit link to the knowledge holder community, as the links to science still need to be developed (e.g., towards EU projects, see Box 07) and similarly links to practice are not explicitly foreseen yet. Accordingly, a concept on how to engage the knowledge holder community in a continuous exchange via BISE is not available. A workshop of the SPIRAL project in September 2012, bringing together researchers from 20 EU pro-

[22] Community of Interest: A (virtual) gathering of people assembled around a topic of common interest. Its members take part in the community to exchange information, to obtain answers to (personal) questions or problems, to improve their understanding of a subject, to share common passions. [Definition based on Wikipedia entry]. On a topic like biodiversity and ecosystem services, the community will include a broad diversity of potential stakeholders, which we call "the NoK community". See glossary Annex 1

jects and experts from EEA, DG Environment and DG Research and Innovation developed a set of ideas and recommendations on how to improve this link (see Box 07). They show the enormous potential that was discovered in better linking BISE and knowledge holders. Some of the recommendations could be implemented via the NET-function of a NoK.

BOX 06: Knowledge required – but in different ways: Different needs of "Briefers", "Digesters" and "Implementers" and different ways to address them

In the KNEU project, we conducted a number of interviews with potential knowledge requesters for a NoK on biodiversity and ecosystem services. As first results it needs to be outlined that there are different needs from different groups of requesters, across policy and society, depending on the way they are working with knowledge in their daily work:

- **Briefers**, who as a group are most actively engaged in the policy agenda: They have short, concise knowledge needs depending on the most recent agenda topic they are tackling at a certain point of time;
- **Digesters**, who, while they may have some limited active engagement in the policy process, (and there is indeed a level of overlap with the Briefers), tend to be mainly involved in "creating and collating" knowledge for the specific needs of their institutions;
- **Implementers**, who are more likely to be involved in the direct implementation (at various levels: regional, national, international, etc) of specific policy areas and thus have quite concrete knowledge needs as well, but on a more thorough level compared to briefers

Although the knowledge needs of these groups differ, the general barriers of accessing the right knowledge and the potential solutions where similar across these groups. These barriers include **an information overload in general, but on the other hand a lack of specific knowledge tailored to needs**. This includes as problem the fragmentation of relevant and poorly signposted knowledge and a lack of time to access it. Also the restricted access to some knowledge (e.g. in scientific journals) was an obstacle as well as the lack and availability of relevant data. All in all, a lack of coordination and collaboration in the field was recognized.

As solution, knowledge requesters asked for a **centralisation/streamlining of information and knowledge** for exchange with knowledge holders (acknowledging the role that BISE, the Biodiversity Information System Europe, might play in this, a thematic presentation of information and knowledge, more digests and briefings with filtered information and in general tools or mechanisms (e.g. via IT/ social media approaches). Nonetheless, information and knowledge accesses should be easy to validate.

Clearly the solutions to the barriers provided an important reference for the expressed preferences for, and expectations of the NoK. Thus the system should be Internet-based and it should be open access (and pertinent to civil society). Linked to this it should have no login or registration requirements or password protection and should be available to external stakeholders in order that everybody should have the same level of information.

Source: KNEU Deliverable 1.1: Overview of experts and requesters of a potential NoK: Mapping knowledge holders, identifying requesters and barriers on how to link them.

Independently of BISE, the networking within the knowledge community is still poorly developed in terms of its capacity to actively engage in policy processes. Most often, this is done on the project level only, where the duration of interaction is limited. The projects OpenNESS and OPERAs are currently working on a common platform linking European stakeholder on ecosystem services to best-practice

knowledge and experience on ecosystem services and natural capital. At a recent meeting of biodiversity-related EU projects in Brussels, organised by DG RTD, the need for more coordinated efforts in this respect, reaching out beyond single projects was outlined (see also Box 07).

BOX 07: Recommendations of the SPIRAL workshop to better link scientific results and knowledge with BISE (shortened with respect to NoK relevant tasks)

The Biodiversity Information System for Europe (BISE) is a single entry point for data and information on biodiversity in Europe. It is a partnership between the European Commission (DG Environment, Joint Research Centre and Eurostat) and the European Environment Agency. Bringing together facts and figures on biodiversity and ecosystem services, it links to related policies, environmental data centres, assessments and research findings from various sources. Research is one of the 5 foci of BISE portal. That part of BISE is still in its infancy, though.

The following recommendations to further develop BISE were discussed at the SPIRAL workshop organized in September 2012:

- **BISE as a standard entry point:** With its general approach, BISE has the best potential to become the starting place for all biodiversity-related information and knowledge
- **Networking beyond BISE:** Although BISE should be an entry point for research information and knowledge, further networking in research will be needed outside BISE to strengthen science-policy activities.
- **Sharing data from projects:** Beyond the formal data flows managed by the EEA and available via BISE, BISE could also make use of data and knowledge from research projects as an additional resource for long-term availability.
- **Further develop the database of research projects in BISE.** The recently established database in BISE on research projects related to biodiversity, hosted by the EEA Biodiversity Data centre, is a good starting point to promote further projects results.
- **Managing and opening the project section of BISE**. The project section of the BISE website could furthermore be opened by a guided content management system for projects to post their material.
- **Long-term archiving of project knowledge.** After the lifetime of projects, their knowledge often gets lost. It should be explored whether BISE could become a long-term archive of the results, products and website contents after completion of projects.
- **Promote BISE in the research community.** The research community should be made more aware of BISE.
- **BISE as provider of research-relevant information on policy**. A function in BISE that could be developed is the provision of an entry point for researchers to better understand the policy context of their research.

Source: text copied and shortened from SPIRAL brief "Tools for Science-Policy Interfaces: Recommendations on BISE and Eye on Earth", which was developed in a joint workshop of researchers and policy makers from DG RTD, DG ENV and the EEA in October 2012, available at: http://www.spiral-project.eu/sites/default/files/18_WS%20recs_BISE%20EoE_3.pdf

Nonetheless, based on the networks existing between research institutions, e.g. ALTER-Net and Marbef; the PEER Network and the network of BiodivERsA projects, a core community of Interest has been developing over the last decade that should serve as basis for an interface on Biodiversity and Ecosystem Services. What is currently lacking is a common web-platform that could be managed by the NoK. These different communities could then be actively connected via a tailored web-platform and could jointly develop communication pathways and capacity building opportunities.

Such a "network of networks of existing institutions and individuals" would form the basis for a broad engagement strategy of the knowledge community for the biodiversity science-policy dialogue in general, and would form the basis for the more formally organised answering decision-making needs function (ADN) and also the research strategy function (RS).

As such (social) network approaches may be time and labour intensive in setting them up and keeping them active, as shown by the examples of the Networks of Excellence, incentives have to be given to encourage participation. However, numerous examples illustrate how such communities were successfully established, for example the Cochrane or Campbell Collaborations and the Collaboration for Environmental Evidence.

Major elements of an online platform that would form the basis for the Community of Interest (and would be the basis for the "one-stop-shop" asked for by decision makers) would include[23]:

- An **overview of finalised and on-going knowledge generation activities on the European level**, including direct links, sorted by themes to existing information and knowledge and expert networks, see also Box 07
- A **'knowledge holder' area** where single experts and/or knowledge hubs are registered and able to present themselves and the area they work in to make them easily accessible for direct contact
- A '**thematic knowledge' area** that allows for thematic access to knowledge from different policy relevant areas. It could include digests of knowledge, such as policy briefs as entry points and then link to both the 'project' and the 'knowledge holder' area for further information and detail. Using thematic areas as main building blocks would also allow to build up the platform step by step[24]
- A **'forum'** which allows knowledge requesters to pose questions to the community of knowledge holders and projects. It could be either completely open, or it could be restricted to allow requesters to even pose conflicting or 'simple' questions or communicate directly with selected knowledge holders.

For all of these elements, an analysis should be conducted on i) how they relate to BISE, ii) if they can be taken over (in parts) by existing networks[25] or iii) if they should be complementary with clear links for easy access.

[23] A platform with similar elements is currently under development for the projects OPENESS and OPERAs and it will be checked if this could be used as sarting points.

[24] As a specific example, see the databases of evidence-base conservation: www.conservationevidence.com

[25] On the European level, the first major networks are the former Networks of Excellence (www.alter-net.info; http://www.euromarineconsortium.eu/fp6networks/marbef; www.e-taxonomy.eu) and their common infrastructure LIFEwatch (www.lifewatch.eu). On the global level, linkages will need to be explored to the potential BES-NET web portal aiming to support the work of IPBES, which is currently under discussion.

! **The added value of actively developing a community of interest via the Network function**:

- **Knowing who is who:** by helping the knowledge holders to organize themselves, including strengthening and uniting existing network, the possibility to identify right addressees for requests will be strongly increased. Similar approaches on the national scale have shown that this is an essential ingredient for success at the SPI.
- **Enhance collaboration and encourage openness:** bringing together different disciplines and expertise across countries on a specific topic, will bring together disparate groups, strengthen collaborative work, and foster greater cross-disciplinary understanding. Knowledge holders will be able to have easier access to the work of others and build on it, thus contributing to tangible progress in biodiversity knowledge and policy.
- **Making the link between knowledge forms (including science) and policy more explicit** will help to build the Community of Interest and enhance the exchange between science and other knowledge holders, e.g. from practical biodiversity management via the thematic knowledge areas. Further developing this link is crucial for a better integration of knowledge.
- **Enhanced responsiveness**: in complement to existing platforms, a more diverse and mutual exchange of knowledge holders and requesters will increase awareness on both sides on "what is out there" and a rapid response mechanism to informal questions is created.
- **Enhance cost-effectiveness** of money invested in European research: The Network-function will enhance the ability to use and reuse knowledge gathered in European projects and beyond.
- **Establish a European wide platform for capacity building and institutional learning on the science-society interface,** as today, such knowledge is often restricted to the duration of projects and to single institutions.
- **Enhance a reflexive process** on knowledge generation and exchange of values and interests in the field of nature preservation.

3.3 Answering-decision-making-needs function (ADN-function)

The second and main function of the BiodiversityKnowledge NoK is to explicitly support European policy in different areas of the policy cycle – in the development, design, implementation, monitoring, evaluation and reporting of policy and management strategies.

Whenever a topic requires an in-depth analysis and a consolidated view from science, specific activities to synthesize and analyse existing knowledge will be needed. To serve this second function, BiodiversityKnowledge would provide an interface where knowledge holders are identified and invited to jointly synthesize available knowledge on a given topic. The prototype of this interface (Livoreil et al. 2012) is a request-driven knowledge-policy interface process. Such a process has three phases. The steps for handling a request would include a preparation, a conducting and a finalising phase (see Figure 3.1)[26].

[26] The general process presented here is roughly similar to the one currently under discussion for conducting the work of the Intergovernmental Platform for Biodiversity and Ecosystem Services (IPBES). With the explicit focus on openness, transparency and an elaborate process of selecting from different methodological approaches, we aim at further strengthening the credibility and legitimacy of the process and at enhancing the methodological basis of interface work.

Different types of actors will be involved in this interface: the knowledge requesters, the knowledge holders, organised in ad-hoc working groups or acting as peer reviewers and a knowledge coordination body (KCB) to coordinate the entire process (see chapter 5.4 and 5.6 for details). Different stakeholders, especially the requesters, will be involved in all phases of the process. A small secretariat would be helpful as well, especially for ensuring openness and transparency of the process and that it follows the agreed protocol (see chapter 5 and the narrative of the NoK prototype in Annex 3).

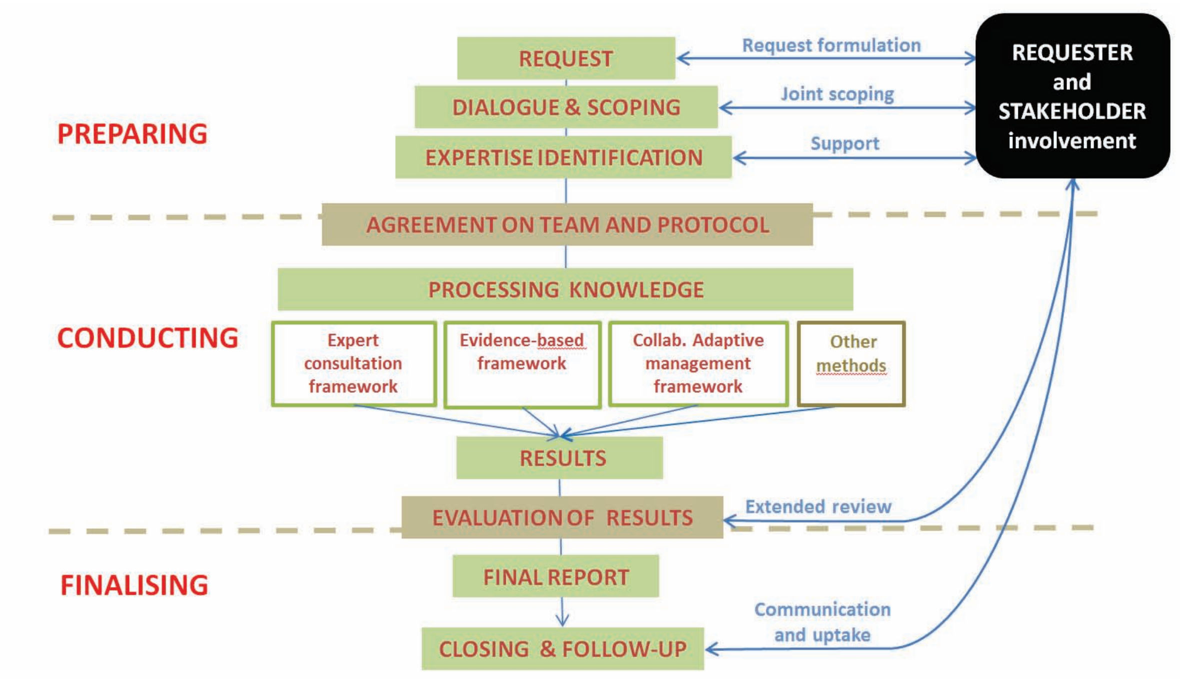

Figure 3.1: *Phases to conduct a detailed knowledge analysis for a policy request via the Policy Support function of an EU mechanism (Source: Livoreil et al 2012, KNEU Deliverable 2.1, for details see the narrative of the NoK prototype in Annex 3 at www.biodiversityknowledge.eu)*

For the **preparation phase**, a dialogue and scoping process between requesters, knowledge holders and other stakeholders will be the central element in order to properly identify the requesters' needs and how these can be framed in order to be answered. At the same time, the scoping will gain a first overview of the quantity and type of knowledge available on the topic.

The preparation phase starts with a request posed to the NoK. A request form may help outlining the major elements of the requests (see Annex 4 for initial format) and will help to check for the basic criteria of selection a request to the NoK should contain (see Box 08). In the beginning of the NoK, the authorisation to pose requests might be restricted to policy bodies, depending also on how the NoK is linked to the wider EU Mechanism.

To further expand the selection process, the request form will be analysed by the NoK which might get into a first exchange with the requester to clarify general questions, and the selected requests will then be proposed to and discussed with the KCB. This stepwise selection will help increase the quality and relevance of the request, as it will be in the interest of the requester to prepare their request as thoroughly and precisely as possible to benefit from an efficient process in the preparation phase. A set of

guidelines for submitting requests will be made available[27]. Any interested person or institution will be able to check the whole process of selection of requests on the web-platform.

Box 08: Examples of selection criteria for accepting to process a request

The decision whether a request should be checked further would be subject to three filters; 1) the request form including basic selection criteria, 2) the secretariat will further check the forms and 3) the Knowledge Coordination Body (KCB) will mainly check the feasibility of the request.

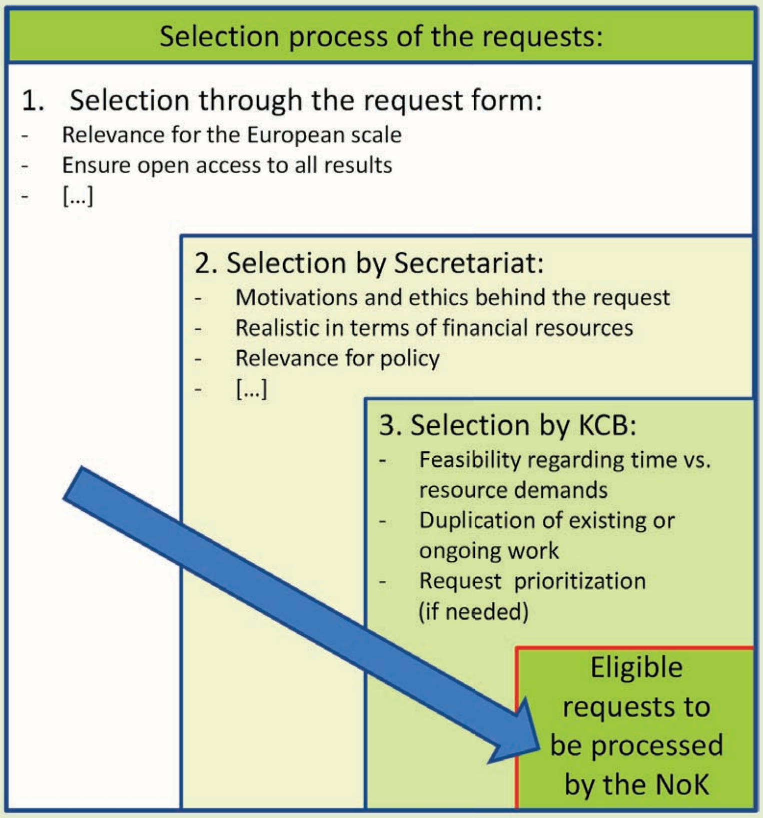

To develop relevant requests, that are answerable with the available knowledge and methods to assess it, it is crucial that requests are jointly developed between the requester(s) and the NoK. Within the KNEU project, potential requests were classified in different types, including requests that are:

- **Seeking greater understanding or predictive power** (e.g. What is the role of biodiversity in maintaining specific ecosystem functions (e.g. biogeochemical cycles)?)
- **Seeking measures of anthropogenic impact** (e.g. what is the impact of wind farm installations on bird populations?)
- **Seeking measures of effectiveness of interventions** (e.g. How effective are marine protected areas at enhancing commercial fish populations?)
- **Seeking appropriate methodologies** (e.g. what is the most reliable method for monitoring changes in carbon stocks in forest ecosystems?)

[27] Similar to this process, the IPBES plenary recently decided on the request-process within IPBES: in IPBES, governments and international agreements are invited to submit requests to a given date, with a detailed list of issues to be tackled in a form, to the MEP for consideration in the IPBES work programme.

- **Seeking optimal management options** (e.g. what is the optimal grazing regime for maximizing plant diversity in upland meadows?)
- **Socio-economic impacts of specific developments in biodiversity** (e.g. what are the anticipated costs of the spread of the invasive species xyz on health or agriculture?)
- **What is a desirable state for….?** (e.g. What is the desirable state of forest in terms of deadwood and other biodiversity-relevant structures?)
- **Scenario building** (e.g. How will the risk of flooding change under current climate scenarios up to 2050?)
- **Horizon scanning** (e.g. what will be the biggest novel threats to biodiversity in 2050?)
- **Public opinion/ perception issues** (e.g. is there public support for badger culling in the UK?)
- **Distribution of species, diseases and other elements of biodiversity** (e.g. How has the distribution and abundance of rabies in fox populations changed in the last 10 years?)
- **Clarification of definitions** (e.g. how do different people/groups define ecosystem services?)

In order to properly analyse and select requests, it will be important to identify which types of questions are included within a request, and which methods serve best to address it. Even if a request cannot be conducted, for example because of resource limitations, the preliminary stage should always be a win-win situation as its outcomes can be used as benchmarks and guidelines for future requests, or could be used again when the resources are made available.

Once a request is accepted, a scoping group will be formed, acting independently from single institutions and covering a suitable range of stakeholders and knowledge holders for the given topic. The group will retrieve an overview of the knowledge available to assess its quantity and quality according to a list of criteria. This will include the disciplines needed to provide input, the potential role of other forms of knowledge, the type and quantity of data and information needed (e.g. from experimental studies, models…), and the potential methods to be used for compiling the knowledge.

The scoping group may also launch a call to the NoK, its members and other knowledge hubs to identify experts on the topic and consult them about (1) the importance of the request for biodiversity & ecosystem services, (2) their perception of current challenges and state of knowledge on the topic, (3) whether they would like to get involved in processing the topic (Figure 3.2). The joint scoping with requesters (and other potential stakeholders) is a major step in ensuring mutual understanding of the question and topic to be tackled, understand the needs of requesters, but also possible restrictions in the ability to answer the requests by knowledge holders, including the selection of appropriate methods and their potential limits in terms of quality, effort and other criteria (see Annex 4).

Often, the scoping process might lead to a refinement of questions, breaking them down into sub questions, and even prioritizing these from a requester perspective, depending on the means available to conduct the work. This might lead to an agreement between the NoK and the requester on the future process regarding procedure, timeline and also financial issues (see chapter 5 on finances).

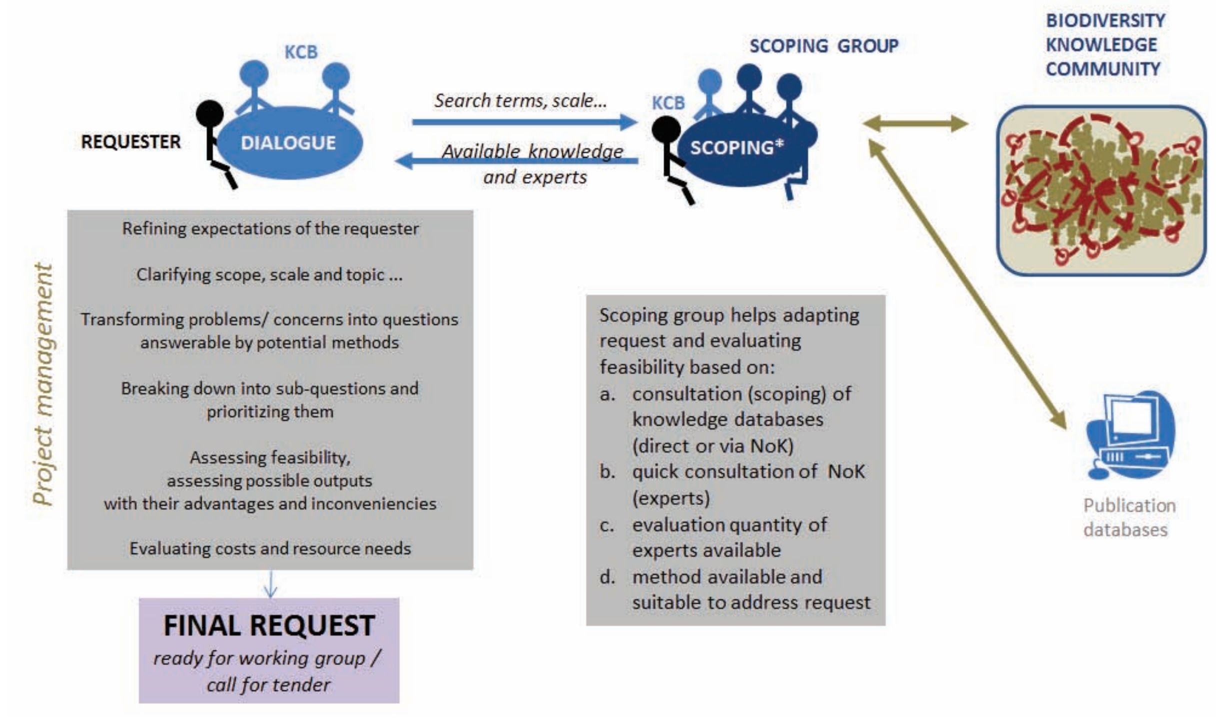

Figure 3.2: *Dialogue and scoping process to finalize the request (see also Annex 3, slide 22 for more details)*

Following the final acceptance and refinement of the request, detailed guidelines (via a general protocol) will be developed to synthesize the knowledge, using appropriate methods in the **Conduction phase** (see Annex 5). For the process of conducting the synthesis, the first step is to set up an ad-hoc working group which includes experts from the scoping group, but most probably additional ones based on the methods chosen and the needs for expertise identified. The first task of this working group is to specify the general protocol regarding the methodological details. This methodological protocol should give a maximum of details about how the knowledge will be gathered, examined, compiled, the scope and scale, and about the methods that will be used for synthesizing the knowledge. Thus, the knowledge about the according methods is crucial in the working groups and has been a challenge in the demonstration cases of KNEU and other activities. According capacity building is needed in the NET—function (see Box 09). Within the project, methods from the expert consultation, evidence-based and collaborative adaptive management frameworks have been applied; these methods are not mutually exclusive but can be combined, which has proven helpful in the demonstration cases of KNEU[28]. It can be concluded that the applicability of these and related approaches depends on the types of questions to be addressed, the knowledge needs and the availability of time and information. Often, complex biodiversity and ecosystem-service-related questions require a combination of different methods. Especially with regard to societal and economic questions a broader set of methods will be relevant (see Annex 5).

[28] See KNEU Deliverable 3.1. For another example, see Dicks, L.V., Hodge, I.; Randall, N.P.; Scharlemann, J.P.W.; Siriwardena, G.M.; Smith, H.;G.; Smith, R.K. & Sutherland, W.J. (2013): A transparent process for “evidence-informed” policy making.- Conservation Letters. DOI: 10.1111/conl.12046

Box 09: Lessons learned on capacity building needs in methods for synthesizing knowledge

Methodologies used in knowledge synthesis require specialist skills within a team of people that would conduct the process and report the findings. Although some specialist skills are already widespread in academia (e.g. meta-analysis), significant capacity building will be required in many other skills in the environment sector. As an example, lessons learned from both this project and the work of the Collaboration for Environmental Evidence (CEE) has highlighted the need for capacity building in the conduct of systematic reviews. All three case studies in this project attempted to conduct a systematic review and all found the process challenging from the beginning. The processes of question setting, stakeholder engagement and protocol formation were time consuming and resulted in fundamental rethinking of the approach. These challenges are commonly recorded by the CEE and it has embarked on a series of training workshops across Europe to increase capacity to conduct systematic reviews and systematic mapping and form a community to promote these methodologies. A key finding of this training program is that the standard of planning and conduct of searching for knowledge, screening of articles and other sources for relevance to the question and assessment of study quality is new even to most academic scientists. A lesson learned for BiodiversityKnowledge is that organisations that co-ordinate development of methodologies and provide standards of conduct for knowledge synthesis will be important contributors to the NoK and can help to build capacity, provide independence and avoid duplication of effort.

The same challenge applies for other methodological approaches, including different kinds of expert consultations and collaborative adaptive management approaches (see also Annex 5).

The main constraint will be the availability of resources, the type of knowledge available and the time and funds from the requester perspective (or the resources available via other means, see chapter 5 on finances). In all approaches, the NoK will gather, evaluate and use the best available knowledge, including where relevant field, local and indigenous knowledge, grey literature and knowledge available only in languages other than English (for details, see the narrative of the NoK prototype in Annex 3). For each case to be conducted, different challenges will arise. For example, it remains challenging to integrate Traditional Ecological Knowledge in knowledge reviews (see Box 10 for discussion).

The draft protocol, once refined with all relevant methodological details by the working group, should be made available to any interested party (i.e. open-access) and peer-reviewed as appropriate, as it has been successfully conducted in all KNEU demonstration cases for systematic reviews. This ensures that all stakeholders had a chance to highlight flaws or possible biases, lack of clarity or inappropriate semantics, gaps in relevance or scope before the work is conducted.

Box 10: Involving Traditional Ecological Knowledge (TEK) into Knowledge interface processes

TEK is recognized as providing substantial benefits for implementing sustainable management practices and adaptive capacities to deal with change. Therefore, the issue of better acknowledging and including TEK into decision making has gained continuous attention over the last decades, especially since the adoption and implementation of the CBD in 1993 where the role of indigenous and traditional communities and their knowledge in conservation and sustainable use of natural resources has been a major item of discussions. With the inception of IPBES, this issue becomes even more prominent, as IPBES wants to ensure that TEK is properly included in all relevant activities of the platform. In its first work programme, a task force to develop according guidelines is already foreseen.

Mostly, the issues of TEK have been discussed in the context of developing countries. However, the first state of the art of TEK in Europe was recently performed by Hernández-Morcillo et al. through an in depth review of about 40 scientific case studies. The cases show how nowadays TEK in Europe is habitually used in forestry, agriculture, fisheries management and nature conservation.

The paper concludes that there is wide evidence of the benefits of TEK for implementing sustainable management practices across environmental sectors contributing to increased socio-ecological resilience and adaptive capacity to deal with change in Europe. Substantial bodies of TEK in Europe exist mainly researched in peripheral areas. However, they show declining trend levels attributed to a variety of factors, including transition from subsistence-oriented economies to market economies, rural abandonment and associated demographic changes in remote regions of Europe. Besides the study suggests that the classical understanding of TEK needs to be revised within the European context to create a collaborative TEK concept that could definitely enrich the NoK and positively contribute to global environmental governance processes such as IPBES.

Source: Hernández-Morcillo M., Hoberg, J., Oteros-Rozas, E., Plieninger, T., Gómez-Baggethun, E. & Reyes-García, V. (2014): Traditional Ecological Knowledge in Europe: Status Quo and Insights for the Environmental Policy Agenda. Environment: Science and Policy for Sustainable Development *56, 3-17, DOI: 10.1080/00139157.2014.861673*

The main and core step of the NoK will then be to conduct the synthesis on the request, based on the finalised protocol agreed with the requester. The working group built for the specific request will be responsible for overseeing and leading the process, based on the protocol. The final products will be highly variable (e.g. reports, briefs, scenarios…) and depending on the request and the requester's needs and the resources available. In any case, the set-up of products should also take into account as far as possible the interest of the experts involved, e.g. in eventually allowing for producing peer-reviewed publications in scientific journals or making input into future research needs identified in the conduct of the synthesis.

For the **Finalisation phase**, the involvement of experts in a broad review process (including scientific and other knowledge providers as well as stakeholder review elements as appropriate) is essential. This will help to ensure that results are of adequate quality, relevance and well understood by all concerned. The quality of the process and work in progress should also be reviewed at various stages during the conducting phase, to ensure that the protocol is adequately followed. For more information, see Annex 3.

The entire request-driven process requires a set of rules and procedures, including the identification of a number of different groups (scoping group, working group, review group) where experts need to get involved. Further details on this process can be found in Livoreil et al. (2012) and in chapter 5 of this paper, where the procedural aspects are further outlined.

The added values of establishing a clear process for answering decision-making needs on biodiversity-related issues in Europe are the following:

!

- **One entry point for requests:** The need for an entry point for requests from decision-making (across scales) to science (and beyond) has been articulated clearly across the KNEU project. The questions to be addressed may be limited in number and only be addressed if they go beyond the scope of existing mechanisms like consultancy contracts and the work of responsible agencies and other bodies.
- **Ensuring broad and updated coverage of the available knowledge:** Knowledge synthesis within the NoK enables broad participation and includes an iterative process with several review loops and opportunities to provide feedback at all stages of the process as well as other means for controlling and increasing quality.
- **Ability to access knowledge at appropriate scales and forms:** The direct link to the open network of knowledge hubs enables to target expertise at the appropriate scales from local to global. It will also enable to include knowledge from other sources than science in its strict sense, for example traditional ecological knowledge
- **Using tested methodological approaches:** Although flexibility will be needed, a high level of credibility can only be achieved by sound methodological approaches. The methodological "toolbox" proposed and tested in the NoK will be crucial to achieve this credibility and explicitly adds a new dimension of quality and transparency but also enables the NoK to address different kinds of questions and thus goes beyond a standard assessment process
- **Transparency of processes:** In addition to using tested methods, the NoK process will clearly document every step in addressing a given request. It thus allows a broad participation and opens up to different perspectives in science and beyond.
- **Reducing reaction time** to policy needs and shortening the timeframes for information to reach policy makers. It will also facilitate rapid updating of synthesis by easy exchange of knowledge.

Thus, the Network of Knowledge will be able to provide a consolidated view from science, and include other forms of knowledge as necessary.

3.4 Showcasing the pathways for decision support through the NoK

How could a NoK, with the functions outlined above, work to support policy making?

Figure 3.3 outlines the potential general "pathway for requests": A request arises in decision making (top of figure) and if the requesters are not able to answer it via their usual ways (e.g., by addressing colleagues, experts or knowledge sources they know, by using consultation contracts), they may use ❶ BISE (or other specific sources/platforms) as an entry point to look for the according knowledge. If this is not sufficient, a next step or pathway could lead them into ❷ the operation space of NoK which first provides them with additional sources as outlined in section 3.2 via the NoK community and its shared web platform, which may provide links to existing projects and other sources (if this is not embedded in BISE) and give the opportunity to contact experts with the requests. For many requests, this may yield

sufficient knowledge, e.g. if relevant studies for the question can be identified or quick responses from a limited number of experts seems sufficient.

Whenever a topic requires specific activities to synthesize and analyse existing knowledge to ensure a consolidated view from science, it will be transferred to ❸ the ADN-function, where the process outlined in section 3.3 would apply.

This process could also be directly addressed whenever a request has a strategic or long-term perspective where the need for a detailed analysis with direct involvement of science and other forms of knowledge is evident from the outset. Also the ADN-function can be applied to a "new topic" that could come from horizon-scanning processes, or when diverging approaches and opinions, e.g. from different disciplines have led to inconclusive results. The methods offered in the NoK processes can be applied to establish overviews of agreements and disagreements and outlining different degrees of certainty in such cases (see chapter 3.3. and Annex 5).

If an analysis in the ADN processes identifies gaps in knowledge, which can only be tackled by additional research, these gaps would be communicated ❹ to the processes of the research strategy function to develop according research needs with research funding mechanisms, member states and research institutions explicitly. Additionally, the research strategy function could be directly activated by requests (e.g., from DG RTD or an ERA-Net like BiodivERsA), to discuss research needs for broader and more strategic issues which don't relate directly to another request from decision-making.

Although this flowchart is rather mechanistic and leaves out the challenging activities within the different functions, it highlights the demand of time and resources which each step may take, especially each request processed in the ADN function (❸). The more demanding the method is (see also section 3.3), the more time and resources-consuming it will be and thus may conflict with the timelines of the policy process to be informed. This is neither specific to nor amplified by the NoK. Such time constraints need to be carefully considered and stress the importance for ensuring a well-organised and resourced process. The demonstration cases[29] have shown, that especially relying strongly on voluntary contributions from experts limits the possibility for short term reaction, an according funding to support major working steps (*e.g.* in conducting systematic reviews) may help shortening the timelines considerably, but several months will need to be calculated in most cases.

Importantly, all steps will require continuous interactions between the knowledge holders (indicated by blue colours) and knowledge requesters and other stakeholders (indicated by green colours and especially the green arrows). This will be especially important in the ADN-function for scoping and review processes.

Communicating these different pathways will help requesters consciously decide the type of knowledge review they require: Pathway ❶ and ❷ may be sufficient for certain requests but are surely limited with respect to achieving consolidated views from knowledge holders especially on contested issues or areas of high uncertainty. Using the ADN-function ❸ will enable to develop concerted and validated views from science (either inner-, multidisciplinary or transdisciplinary) on a given topic, a deficit often cited by decision makers in consultations. Uncertainties and gaps in knowledge, which are identified during the process, can even lead to identifying additional research needs❹.

[29] See deliverable 3.1 of the KNEU project (downloadable from www.biodiversityknowledge.eu)

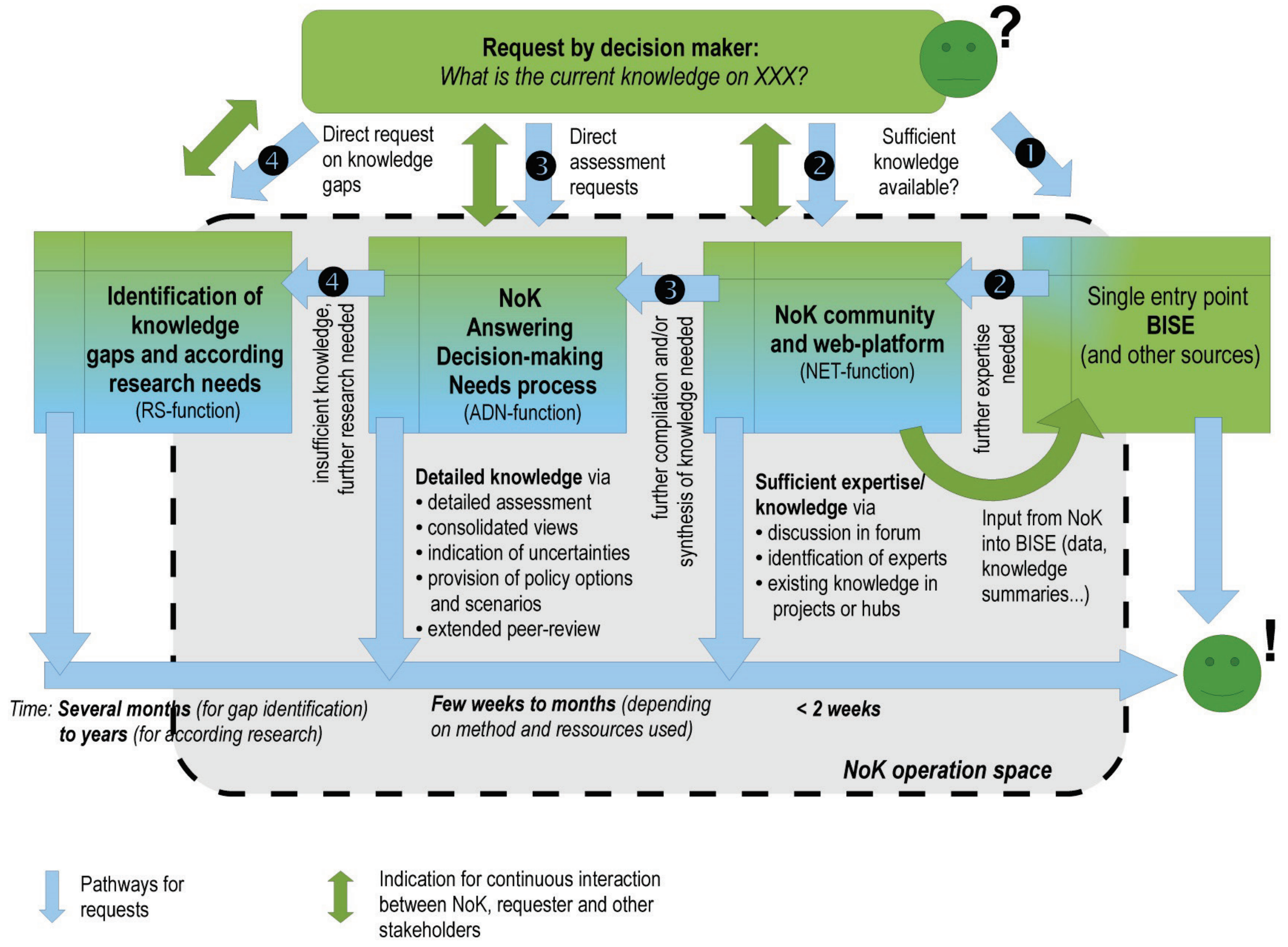

Figure 3.3: *Flowchart of the entry points where the NoK is supposed to support decision making in identifying and collating relevant knowledge*

3.5 Conclusions: The Network of Knowledge as science-driven part of the wider science-policy interfaces

As the word ‘interface’ indicates what is needed is an operating space at which the two systems ‘knowledge’ and ‘policy’ interact. To create and enhance this interaction both complex systems have to be organized accordingly. If both sides are properly organized facilitating their interaction may be enough as interface, avoiding the need for a third complex system in between. As highlighted with the grey-shaded ‘operation space’ in Figure 3.3, BiodiversityKnowledge would support existing interface processes by facilitating ‘networking the networks’ and enhancing knowledge flow to support decision making. Within this context, the importance and also ambition of the network function cannot be underestimated, as its success will make a difference in how the policy support function is perceived in terms of legitimacy, but also in terms of credibility. Activating a large range of knowledge holders from science and beyond will enable a much broader engagement in Science Policy Interface processes.

Operationalizing this in a crowded space of existing institutions and avoiding duplication of efforts, raises a number of challenges. These will be discussed in the following chapter 4, as basis for the potential design options that are then discussed in chapter 5.

4 Ability of a Network of Knowledge to deliver relevant products while ensuring credibility and legitimacy

A broad consultation was undertaken to identify recommendations on the design of the draft NoK and what it would take to significantly improve the capacity of the scientific community to respond to knowledge requests from policy.

Three regional workshops, two conference by the KNEU project as well as four sessions organized at external conferences (ESP 2011, ECCB 2012, IPBES-1 2013 and ALTER-Net 2013) were carried out to collect feedback and discuss the NoK structure (See details list of events on www.biodiversityknowledge.eu). Together with further meetings (e.g. with the project's client dialogue group), interviews and spontaneous feedback, approx. 300 individuals have commented on the draft NoK; approx. 10% were policy makers, 15% practitioners and about 75% scientists. Through this process, key challenges were identified, which BiodiversityKnowledge has either since tackled in an updated NoK prototype or still needs to address in the setup of the NoK.

The broad consultation led to the identification of five essential ingredients for developing a NoK (each one is further detailed below, list doesn't indicate ranking):

- Quality assurance;
- Data sharing, standards and data exchange
- Connecting, motivating and acknowledging the knowledge holders and requesters ;
- Communication
- Capacity building

In addition to those ingredients, further challenges lie in the more process-oriented elements of the NoK – its governance and its financial support. These issues will be addressed in chapter 5 directly with the options presented for the NoK design.

To integrate the four ingredients mentioned, while keeping the work of the NoK open, accessible and transparent, the following analysis uses the CRELE attributes, already introduced in section 3 and Box 05 in order to serve as baseline and guide the procedures[30]:

- **Credibility** of the NoK which is the perceived quality, validity and expertise of the people, processes and knowledge exchanged at the interface. Credibility should be ensured by e.g. the rigour of the process and the skills of the participants and by transparency of all processes and decisions
- **Relevance** or saliency, which represent the responsiveness of the NoK to policy and societal needs, i.e. to the users of the NoK
- **Legitimacy** is the perceived fairness and balance of perspectives within the SPI processes, including inclusiveness of all relevant stakeholders, transparency, fairness in treatment of diverging values, beliefs, and interests.

These attributes are widely accepted and used, and can explain an SPI's set-up and outcomes, and have been explicitly considered for example in the creation of IPBES. The Intergovernmental Panel on

[30] See also according briefs of the SPIRAL project: http://www.spiral-project.eu/content/documents#jump2briefs

Climate Change (IPCC) uses them to evaluate scenarios, draw lessons from past experiences and explain assessments' influence.

Particularly to achieve credibility and legitimacy, **independence**, i.e. avoiding influence of specific groups e.g. from donors, political parties and vested interest groups, will be important. As many contributors in the discussions stressed the importance of this attribute, we added it as a fourth interlinked attribute.

Both practical application and scientific analysis using the CRELE attributes have shown that major trade-offs can arise when designing and conducting SPIs[31]. For example, a strong legitimacy, e.g. via a mandate by governments, might reduce credibility on the science-side, as political control of results might be a part of the SPI process (e.g., the negotiations by governments on the "summary for policy-makers" of IPCC reports). On the other hand, a strong focus on scientific credibility might reduce the relevance of the work, as issues tackled might get reduced to those where scientific knowledge is available and consolidated in terms of clear results[32].

For each of the five ingredients mentioned above, the following sections outline the *challenges* faced in terms of credibility, relevance, legitimacy and independence, then draw *lessons learned* so far from a general perspective on science-policy interactions as well as from the KNEU project and how the NoK approach can in fact *add value* to science-policy interactions by creating new solutions for the challenges identified.

4.1 Quality assurance

Quality assurance in SPIs covers a broad range of issues, some of them directly tied to scientific work (see also next chapter on data), and some of them related to the SPI process itself, where quality stands for effective procedures. Thus, "quality assurance" is an overall challenge affecting all four attributes from credibility to independence.

4.1.1 Challenges

Over the past decades trust in the ability of the scientific community to speak with "one voice" and in the quality of scientific knowledge has decreased in both public opinion and among policy makers. For almost any position you can find scientific arguments and evidence[33]. Besides some work that does not comply with scientific standards in many cases seemingly contradictory results or conclusions are due to the fact that different often very narrowly defined questions are analyzed or different methodologies are applied.

Hence, in a science-policy context at least two challenges arise with regard to quality assurance: the quality of the knowledge used must be assessed and an adequate framing is essential. This means that policy questions need to be translated and often broken down in such a way that they can be addressed with the available knowledge (whether scientific and/ or beyond) and results need to be integrated in

[31] See for example the work of SPIRAL, www.spiral-project.eu, or the paper of Cash, D. W. et al. (2003): Knowledge systems for sustainable development.- Proceedings of the National Academy of Sciences of the United States of America 100: 8086-8094.

[32] For details, see mentioned SPIRAL briefs on the CRELE concept, see www.spiral-project.eu/documents

[33] For a brief discussion, see Pielke Jr., R. (2007). The Honest Broker: Making sense of science in Policy and Politics.- Cambridge University Press

adequate ways to provide answers to the policy question, taking the available knowledge and the ways they were achieved into account.

But it is not sufficient to ensure quality internally it also has to be communicated so that results are considered of high quality by requesters and relevant stakeholders (see chapter 4.4).

Quality of knowledge input into a decision-making process has several dimensions. It will be considered of high quality if the criticism against it has been lowered to a minimum, i.e. the outcomes cannot be easily (and honestly) disputed/debated. Or it can be said as of high quality because it is useful, understandable and relevant to the current context. "Quality" will always be perceived with a variety of meanings by different stakeholders. Nevertheless, high quality science relies on principles that are valid for all disciplines and make the scientific endeavour as rigorous and objective as possible. Explaining and using these principles can provide an explicit basis to give an indication of the level of confidence or risk associated to each result.

Adding to this, a co-production of knowledge from different forms beyond purely scientific ones, as for example in some methods of expert consultation or in collaborative adaptive management approaches can also lead to a high quality of knowledge as well as the processes leading to them.

Keeping this complexity in mind, a few – more general – challenges can be highlighted and will need to be tackled to enhance quality assurance in any science-policy process:

- **Accuracy of information and quality assurance**: identifying and controlling for biases and confounding variables, and differences in methods in original work, confidence, level of transparency and replicability for provision of data, peer reviewing, ensuring appropriateness and consistency of parameters.
- **Limitations:** comprehensiveness of knowledge taken into account, its validity, applicability of the evidence and uncertainties of findings; adequacy of the information and relevance to real-world conditions; measurable indicators of performance.
- **Uncertainty:** Communicating overall uncertainty by clearly highlighting the limitations of the knowledge presented and distinguishing between knowledge gaps and missing data.
- **Alternative options**: Identifying multiple perspectives on a topic and presenting different options for action and the potential trade-offs associated with the options identified.
- **Expected barriers to the use of results**, including time pressure, perceived threats to autonomy, preference for tacit knowledge, resources required.
- **Lifespan of the answer**: Anticipated needs for future updating of findings due to expected new results, especially in the context of existing uncertainties. Ability to update knowledge when new knowledge is produced.

Although many of these issues may appear complex, suitable ways of accounting for them are available from assessments and other evaluation processes[34]. As one major cornerstone, this would include, besides review processes, an approach to assign certainty terms to key findings, as it has been devel-

[34] For a recent example on issues related to the CAP reform, see for example Dicks, L.V., Hodge, I.; Randall, N.P.; Scharlemann, J.P.W.; Siriwardena, G.M.; Smith, H.;G.; Smith, R.K. & Sutherland, W.J. (2013): A transparent process for "evidence-informed" policy making.- Conservation Letters. DOI: 10.1111/conl.12046

oped for the MA and IPCC. This includes an indication of the level of expert agreement on a given statement and type, amount, quality and consistency of evidence[35].

4.1.2 Lessons learned

General lessons: Learning from experiences like the IPCC and the MA, quality assurance of the process and the output are of crucial importance. For the NoK and through its limited experience so far, many lessons learned could be used to improve the quality of *both,* process and products, while ensuring a balance in addressing the four attributes.

The credibility of the process and products of the NoK are highly dependent on a broad and balanced participation of experts, bringing in a diversity of backgrounds (disciplines, geographic, etc.), experiences and approaches, thus enhancing relevance and building legitimacy (see also section 4.3).

The quality of the process and product, as well as the quality of the methodologies used can only be judged if they are understood by all those concerned. To achieve this, it is important to ensure transparency of processes, to use understandable language within and outside the working groups (see also section 4.4.) and ensure broad review procedures, using extended peer-review.

Lessons from the work of KNEU: In order to ensure quality throughout the entire NoK process, quality control on the expertise involved in the process should be built-in as early as possible. A combination of structured search for adequate experts through the existing hubs that can help identify relevant experts and an open call for expertise should be used to ensure broad participation. The experiences from the KNEU test cases show that it is important to use a combination of open calls for expertise and specific identification of hubs and experts, and thus directly acknowledge their expertise. In order to avoid bias, it is also important to consciously address knowledge hubs outside the usual (biodiversity) network, which could be supported by an early stakeholder analysis during the scoping process[36]. In addition, a transparent open recruiting/nomination process to select the participating knowledge holders for working groups and evaluation should be based on a priori defined criteria, which should include inter alia:

- Scientific/technical expertise in the topic, based on according publications and other relevant activities in the field
- Demonstrable expertise on the topic from other relevant knowledge systems (e.g., private sector, natural resource management, TEK)
- Experience in collaboration in synthesis/ assessment processes
- Experience in European (and/or international) collaborations
- Experience in communicating results and reaching out to relevant organisations and networks (e.g., NGOs, policy institutions in the field)

Other important lessons (from KNEU and other processes) are that adequate framing can be achieved in joint scoping procedures of the requesters and the relevant working groups, ensuring transparency

[35] See for example the according document of IPCC: Mastrandrea, M.D., C.B. Field, T.F. Stocker, O. Edenhofer, K.L. Ebi, D.J. Frame, H. Held, E. Kriegler, K.J. Mach, P.R. Matschoss, G.-K. Plattner, G.W. Yohe, and F.W. Zwiers, 2010: Guidance Note for Lead Authors of the IPCC Fifth Assessment Report on Consistent Treatment of Uncertainties. Intergovernmental Panel on Climate Change (IPCC). Available at www.ipcc.ch

[36] As outlined by Dicks et al. (2013), the influence of group composition on outcomes in science-policy processes in environmental issues has rarely been analysed in studies, so that a broad approach trying to involve multiple disciplines and key stakeholders should be used from a "precautionary" perspective

and adequate stakeholder involvement can further increase both relevance and legitimacy. For all products clear review procedures will have to be established and the final products should not only be reviewed by different scientists but also by different stakeholders (e.g., NGOs, land user's associations) with different professional backgrounds (e.g., engineering, economics, law, policy making etc…), thus using an extended peer-review approach.

Finally, a system for quality evaluation and improvement of both process and outcomes will need to be developed, including for example integration of feedback, screening for more advanced methodologies for knowledge assessment, or a build-in, but independent regular evaluation procedure.

4.1.3 Added values of a NoK

The broad consultation and the demonstration cases have clearly indicated some added values of the NoK prototype in enhancing quality.

The NoK approach aims at a broad participation and thus enables independent internal and external feedback loops and other means for controlling and increasing quality. Particularly for conflicting issues bringing the different perspectives into a common process can help to bring more evidence into the decision-making process, but also make the underlying conflicts and interest visible.

The NoK approach includes an explicit choice of the best available methodologies to compile and assess the available evidence. This ranges from evidence-based methodologies such as systematic reviews to different forms of moderated expert consultations to transdisciplinary approaches such as collaborative adaptive management, with the possibility to combine these approaches depending on the needs identified. Combinations of different methods are also possible. This choice process will be made transparent explaining what each method means for accuracy of information used, its limitations etc.to ensure credibility (see Annex 5 on methods).

When using evidence-based methodologies to assess knowledge, the extensive and comprehensive literature search (including "grey literature" to minimize publication bias), the critical appraisal approach and the goal of transparency and objectivity in reporting aim at minimizing bias and selectivity to particular sources unlike any other review process[37].

Where more applied forms of experience-based knowledge are relevant, other forms of knowledge can be included. Here again, the process used to acquire information sources and their basis will need to be documented and made available, so that transparency and traceability regarding the origins of knowledge and outputs contribute to enhancing credibility.

Where there is not sufficient evidence available different forms of expert consultation will be used. Here the process of selecting relevant experts is particularly important and again making the selection criteria explicit can help to increase credibility. Different forms of triangulation can be applied to ensure acceptable levels of validity. Possible approaches include stakeholder dialogues preceded by stakeholder mapping and analysis, more structured joint-fact finding processes, different forms of Delphi processes and Bayesian networks method for situations characterized by high levels of uncertainties and low levels of knowledge available[38].

---

[37] See for example the approaches used by the Collaboration of Environmental Evidence, www.environmentalevidence.org

[38] For decription and discussion of these methods, see Bergmann, M.; Jahn, T.; Knobloch, T.; Krohn, W.; Pohl, C.; & Schramm, E. (2012): Methods for Transdisciplinary Research. A Primer for Practice. Campus Verlag

To sum up, the NoK will use an explicit, transparent and traceable procedure to ensure quality throughout the entire process: from selection of experts, scoping of available knowledge, choice of methodologies, conduction of the synthesis, and extended peer review. This will increase credibility and legitimacy. Similarly, accounting for and communicating uncertainty will increase credibility.

4.2 Data standards, data sharing & exchange and methods to analyse them

4.2.1 Challenges

Answering questions and producing knowledge that require interpretation of biodiversity data is still hampered by lack of harmonization of protocols, taxonomy and accessible, common databases, although major improvements have been achieved or are on their way, for example via GBIF and EUBON/ GEOBON for core areas of biodiversity data. The lack of agreement and use of standardized protocols and species' names can result in multiple experts seemingly disagreeing with each other already on the data integration level. This does not contribute to transparent and easy-to-understand communication with requesters at a later stage of knowledge compilation, nor does it contribute to the credibility of the scientific community. Standards and data harmonization have to be developed to allow research institutes and agencies to communicate and exchange findings.[39] As one underlying reason data sharing is often problematic due to issues like confidentiality and ownership which hinder a timely and constant integration of new data into shared databases[40].

This underlying challenge for a Network of Knowledge cannot be tackled directly by the NoK, but rather by specialized processes in science or between science and agencies or other implementation bodies. It is, however, seen as a major obstacle for better informed policy-making in both science as well as in policy, as it may hinder the use of certain methods in analyzing existing knowledge. It therefore needs to be taken into account when designing a NoK, making sure that organisations dealing with data harmonization and data sharing are involved and informed on identified needs.

4.2.2 Lessons learned

General lessons: On the policy level, harmonization is being pursued and stimulated by the reporting obligations for International Conventions such as the Convention on Biological Diversity (CBD) but also by the European reporting on the Birds Directive and the Habitats Directive. These require integrated assessments on status and trends of species, habitats and ecosystems, to name just a few[41]. Similarly making data accessible and providing metadata as pursued e.g. by the INSPIRE directive contribute to a better availability of relevant data[42].

The three domains of biodiversity, marine, freshwater and terrestrial, have achieved standardization and common database development in decreasing degree: In the marine domain, data sharing is common practice as cooperation is of utmost importance when collecting data e.g. for monitoring fish stocks.

---

[39] It should be noted that in first place, the interest in better data harmonisation and sharing lies in science itself, as it is needed to better answer scientific questions across scales, taxa, and for complex interactions, to name just a few.

[40] For an according analysis, see for example Enke, N.; Thessen, A.; Bach, K.; Bendix, J.; Seeger, B. & Gemeinhölzer, B. (2012): The user's view on biodiversity data sharing — Investigating facts of acceptance and requirements to realize a sustainable use of research data.- Ecological Informatics 11: 25-33

[41] See for example the GEO BON concept document: GEO BON (2008): The GEO Biodiversity Observation Network – Concept document, online at http://www.earthobservations.org/documents/cop/bi_geobon/200811_geobon_concept_document.pdf (accessed 30-5-2013)

[42] See http://inspire.ec.europa.eu/

Hence, standards and data harmonization had to be developed to allow research institutes and agencies to communicate and exchange findings. In freshwater ecology the standardization of protocols and existence of common databases is much less developed[43]. In terrestrial ecology, only species like birds and butterflies have standard procedures for collecting data, for other species and for ecosystems, such standards are only developing[44].

The work of earlier EU-projects like EUMON and EBONE, and the work carried out currently in the EU-BON project[45], and the continuation of LifeWatch will bring this integration of data and standards further and this work would need to be used and further linked to the aspects of knowledge generation from such data.

Lessons from the work of KNEU: Depending on the methods used in processing requests in the NoK, there is a strong need for data in a processed and readily accessible format. In any case, a broader accessibility of available data and information based on broad datasets on a sound scientific basis increase credibility of knowledge derived from such data and information. Often the availability of information is a major criterion for decisions in the NoK process on whether certain methods can be used and may often restrict the work to expert consultation approaches.

The NoK thus highlights the need for data and information integration and availability and supports their further development in initiatives like LifeWatch, EUBON and GBIF.

4.2.3 Added value of a NoK

As outlined, the integration of data and information towards accessible and relevant knowledge is important for scientific work, but also for the broader evidence base, that a NoK would need to build upon to gain credibility.

Accordingly, the added value of a NoK is to facilitate, speed up and demonstrate the usefulness of data integration and sharing, and its potential links to the general needs of policy, *e.g.* when it comes to regular reporting, developing monitoring approaches, but also for science, as many high level studies from integrated datasets show (e.g. from the U.K National Biodiversity Network[46]).

The NoK will need to establish a close collaboration with existing data sharing initiatives, but it can also significantly contribute to their promotion and use. The NoK can thus support the dissemination and use of many databases which until now have been less well known or poorly used. Such a work could be developed in close collaboration or directly with the Biodiversity Information System Europe (BISE).

On the operational level, when trying to collate existing knowledge to answer a request, finding the scientific literature is a relatively easy yet time-consuming task, thanks to today's tools and databases. Yet, a part of science is hidden from these sources if the data is not accessible via these sources or when a programme or study is currently conducted, it is not referenced yet and could be easily omitted. By locating relevant ongoing activities in knowledge generation, NoK contributes to their integration into the

---

43 but see the EU-funded project BioFresh www.freshwaterbiodiversity.eu, which takes major steps forward in this respect

44 but see ongoing approaches like the European Long Term Ecosystem Research Network (LTER-Europe) www.lter-europe.net, the work of the LIFEWATCH infrastructure (www.lifewatch.eu) and the work of GBIF (www.gbif.org) on the global scale

45 For further information, see websites of the projects: EUMON: http://eumon.ckff.si/, EBONE: http://www.wageningenur.nl/en/Expertise-Services/Research-Institutes/alterra/Projects/EBONE-2/About-EBONE.htm; EUBON: eubon.eu

46 See https://data.nbn.org.uk/

current request and thus supports its (further) use, as shown by the integration of the work of NERC Cambridge on issues of the agricultural case study.

In bringing together different disciplines and expertise across countries on a specific topic, the NoK also strengthens the collaborative work across disciplines and knowledge domains, to answer a request which can play an important role in evaluating existing databases and highlighting potential quality gaps. The central added value of collaborative work consists in enabling contributors to have access to and work on the work of other scientists and enables others to build on their own efforts, all in all contributing to tangible progress in biodiversity knowledge and policy. Many European projects, including the collaboration of funding agencies via BiodivERsA show that this approach is successful and could be further facilitated by a NoK.

A NoK operating as a 'network of networks' showcases the relevance of integrating data and speeds up ongoing processes and dissemination across countries, it will also contribute strongly to a higher legitimacy of SPI processes, as country-specific results and views can be incorporated especially in expert consultations, thus being able to add value not only at the European, but also at the country level (see for example the Conservation demonstration case in deliverable 3.1 of the KNEU project (downloadable from www.biodiversityknowledge.eu). Box 11 summarizes some lessons learned from the agriculture demonstration case.

Box 11: Some lessons learned from the Agriculture Demonstration Case

The demonstration case presented here analyzed the question "Which types of landscape management are effective at maintaining or increasing natural pest regulation in a context of decreased use of pesticides?" A combination of methods was used to address this question and subsets of it (for details, see KNEU Deliverable 3.1). The following lessons learned can be drawn from it:

- Systematic reviews showed to be a useful tool not only to get a clear picture of a knowledge field for policymaker information, but also to get a comprehensive overview of a subject for designing research or monitoring, answer open questions, identify key knowledge gaps, spot traditional approach flaws (e. g. recurring design setups doing the same kind of research over and over again and expecting different outcomes) and summarize the state of a particular art for whatever purpose.
- Working with professional librarians and information managers to conduct the search for systematic review approaches in the case was very interesting because they know the search engines, tools, and have experience in designing searches.
- Even if information on indigenous and practical knowledge was exchanged and methods to access such knowledge were presented and discussed during the conduction of the case, a balanced representation of such knowledge in decision-making processes might be difficult to achieve due to the different nature of knowledge forms.
- Workshops are important ways of networking and they are more effective in bringing together people of various backgrounds and exchanging knowledge at various levels than other forms of networking, such as e-mailed information. However, they also require much more resources and they are facilitated if financial support for travel and subsistence is provided.
- The workshop created a positive atmosphere but was not enough to maintain a traceable level of exchanges afterwards.

(Extracted from the deliverable KNEU WP3 (3.1) see http://biodiversityknowledge.eu/documents)

4.3 Connecting, motivating and acknowledging the actors

4.3.1 Challenges

There are a large number of ongoing formal and informal interactions which can provide the majority of knowledge needed. Thus a first important step when setting up a NoK consists in identifying, connecting, committing and acknowledging the knowledge holders on the one hand and the potential requesters on the other. Since a NoK should be able to connect with as many relevant networks, organizations and individuals as possible in different regions and member states, within Europe and worldwide it will need a basic coordinating structure to do so, but has to be flexible in order to address the right stakeholders for each potential topic, often reaching out beyond the 'classical' disciplines and stakeholders of the biodiversity and environmental sector. This is maybe the most crucial challenge in setting up a NoK.

Most stakeholders today, in policy, in science, or from any other area, face a high workload in their specific context, limiting the investment of time (and potentially the motivation) to engage in interface processes. Even if the general interest in the work of a NoK is high, as it has been stated by many participants in the various events of the KNEU project, the challenge is to keep them informed as for many thematic requests, they (as individuals and/or institutions) might not be the right experts to involve, but might be for later requests. So the design of the NoK (as a community of interest, and as answering-decision-making-needs process) needs to provide incentives not only for getting actively involved, but also for "staying tuned" into the overall process.

Another challenge in this context is that different entry points are required for different stakeholders, as they will have different interests and the benefits of staying tuned will differ as well (see for example Box 12 on the motivations of experts to get engaged).

The community of knowledge holders which needs to be addressed is a dynamic entity: knowledge from some sources (e.g. from research projects) quickly loses accessibility, people change affiliations and/or belong to various hubs, for example to a university, a European research project and a learned society. Some of these knowledge hubs have encountered ways of dealing with the dynamic community of interest by establishing their own ways of interacting internally and with the area of policy, for example some learned societies have established "policy committees". For example, the British Ecological Society has a "Public and Policy Committee" to develop public and policy outreach activities.

Nonetheless, major challenges remain in better connecting all these hubs and pathways into policy and bring them together for a common input into policy discussions, whenever this is needed and desired.

The main challenge here is then to connect and commit this dynamic and diverse community of different groups and to connect enough knowledge holders for a comprehensive representation of the existing and interdisciplinary knowledge on a topic. To enhance credibility and legitimacy, the NoK will have to work in a complementary process of networking excellent people, skills and the latest knowledge as well as integrating different types of knowledge. Involving well-known and respected contributors for example will improve visibility and credibility, but also processes need to be open to new, not so experienced contributors also to ensure a certain level of capacity building (see also 4.5). In addition, continuity in the commitment from the Community of Interest should ensure long-term functioning of the mechanism.

Box 12: Motivations of experts for getting involved in policy support activities

The motivations of individual experts to get involved in policy support activities vary from individual to individual, between the individual and institutional level, as well as between disciplines. They include (in no specific order of importance)

- Demand-driven process by policy
- Technical learning and new ideas from other countries
- Networking and future collaborations
- Working together in focused technical groups
- Personal contacts with coordinators (trust)
- Personal contacts with other participants (spread the word and trust)
- Interdisciplinary process
- Contributing knowledge and data
- Career development (scientific publishing for early career)
- Institutional agreement (scientific publishing)
- Knowledge exchange ideas/ techniques
- Prestige of being involvement in European projects
- Sharing information and feedback/ dialogue with peers
- Learning about methodologies
- Information on the project progress and wider context
- Meeting location
- Non-scientists increasing scientific knowledge
- Expenses paid

Taken together, these all help to justify time away from other workload. Accordingly, it is important to communicate this range of potential benefits to support involvement.

(Based on KNEU WP4 interviews and focus groups with participants from demonstration cases)

4.3.2 Lessons learned

Lessons from the work of KNEU: The NoK would need to maintain and improve the mapping of knowledge holders of directly-linked actors at different scales but also including scientific, legal, social and technical actors beyond core areas, which is a specific challenge. In addition, it would be important to leave enough flexibility in the mapping to cope with the dynamics of the knowledge landscape and to include new actors.

Through the broad consultation and the mapping exercise of KNEU (see Box 02), obstacles to commit work time to the NoK were highlighted, such as (1) the resource limitation of the knowledge holders and (2) motivations from the knowledge holders to get involved in any policy support activities (see concrete examples in Box 10). Reasons to increase the willingness to participate include:

- **Confidence in usefulness:** if participants feel confident that their engagement will help to make a difference in comparison to the current situation of decision support. This requires ensuring that the NoK is useful for policy development and practical management (although this is a “chicken and egg” situation especially at the very early stages of NoK,

when this confidence has no explicit proofs yet). This confidence can be enhanced by ensuring a well-functioning, supported and well-communicated process that tries to maintain the focus on decision-making and is transparent internally as well as externally.

- **Mandate:** A clear mandate from the policy side balanced to ensure independence of the NoK would enhance interest and dedication from knowledge holders, as it ensures an interest in and relevance of the results from the requester's side. Also an acceptance (or even official mandate) of the process from institutional knowledge holders would increase its legitimacy

- **Easy-to-use:** The NoK should limit the time the participants spend with understanding the engagement process and the technology involved in the process. NoK should provide clear guidelines for the different actors on how to get engaged (e.g. explicit instructions, documentation, user support or even video demonstrations). If involvement is too complicated few people will bother to try it out. Additionally, the way to get involved should be tailored to the users, (e.g. through a topical approach where participants can contribute their expertise) and the outputs should be adapted to the different categories of users. The work performed within the project also highlighted the additional importance of creating and supporting a "Community of interest", via a web portal, which would require further exploring the possible technological approaches to achieve an active use of such a tool. A suggestion was to provide a friendly and free-access web-interface to facilitate exchange of information, enable communication with the community and allow for commenting on the different products.

- **Credits and outputs:** Participation in the NoK and any contributions to its outputs need to be acknowledged through making them explicit. Status, financial or scientific rewards (including scientific papers) should be pursued wherever possible, over time a certain prestige in taking part should be built up.[47]

- **Learning environment:** The NoK creates a learning environment where participants feel that their time investment is rewarded with learning new methods, new knowledge and increasing their network (see also 4.5).

- **Collaboration:** The NoK can create an open and pro-collaborative working environment, where involved experts might also benefit from in later collaborations. Knowledge holders with similar or related research interests who want to help answering a request should find that they have more to gain from collaboration than from competing for decision-maker's attention. Being transparent during the whole process will help contributors/followers to build on the work of others.

- **Independence:** The NoK should ensure that the whole process is independent from external control and from vested interests, contributing to its credibility. The NoK should be both cautious and transparent regarding links to other organizations and interests, in particular where significant funding is involved. According procedures for dealing with conflicts of interest need to be set up.

[47] The issue of prestige shouldn't be underestimated, as can be seen from the high prestige in science today that experts gain when becoming lead authors in the IPCC Assessment reports.

- **Link to international activities:** Although the NoK would focus on the European level, it will get additional acceptance and support by linking up with international activities. Here, the link to IPBES is of specific importance. For example, the NoK could provide European synthesis work on topics tackled by IPBES, and thus create a “win-win” situation where the work in the NoK is used on the European as well as on the global level and thus the input of experts is acknowledged twice.

Box 13 gives some examples on how challenges, lessons learned and added values are linked, derived from the case studies.

Box 13: Some examples from the case studies on challenges and lessons learned regarding connecting and committing actors

- The NoK worked well to reach knowledge hubs and experts and to inform them about the development of the case studies, but it was not enough to get them strongly involved or to achieve that collaborators dedicated significant efforts to the work. Personal contacting worked much better, and face-to-face meetings (e.g. workshops) are one important way to get people more involved and to really exchange knowledge of various kind.
- The heterogeneity of the knowledge holders and users is a challenge in terms of achieving an efficient knowledge exchange and synthesis. In each community, there are people who have skills and the mind-set which favours bridging the gap between knowledge-oriented community (e.g. scientists) and the task-oriented stakeholders (managers, decision-makers including policy makers). They should be identified as they are very effective members of working groups. The challenge is to make sure they are acknowledged by their own community as representatives with integrity and authorisation to speak on behalf of their groups (no conflict of interest, not only promoting their own vision).
- Scientific tools (search engines, libraries, databases, analysis...) are in place to implement scientific synthesis and assessment. However, nothing comparable is available for traditional, practical and technical knowledge, here the involvement of knowledge holders is even more important but also more time consuming.

(Extracted from the deliverable KNEU WP3 (3.1) on demonstration cases)

4.3.3 Added value of a NoK

Within KNEU, the current biodiversity landscape of experts, networks and knowledge holders in Europe has been mapped (see KNEU deliverable 1.1 on www.biodiversityknowledge.eu). The mapping shows that the broad community of individual knowledge holders is diverse and includes among others research organizations, cross-institutional projects and networks, learned societies, and NGOs. The most efficient way to access and connect the knowledge on biodiversity is to use existing hubs and organizations, reaching multiple individuals simultaneously as the NoK approach proposes. It will never be possible to address a complete community, but hubs act as multipliers and also as a first implicit level of quality control on the expertise involved in the process. Addressing experts and hubs from Eastern and in parts southern Europe and supporting them to participate in the NoK activities will be of specific importance; a NoK could in fact effectively support this by continuously building up links to national networks. Similarly, a NoK, by working continuously could also help in bridging the divide between disciplines.

When taking this aspect, and the lessons learned into account, a NoK approach – through explicitly reaching out to the whole community – has a clear added value with regard to credibility, relevance and legitimacy as it enables a broad participation. This is rarely the case in science-policy approaches that restrict the input to certain groups, institutions or individuals, like (many but not all) consultancy contracts, work by single research projects or institutions.

This is especially important in the area of biodiversity and ecosystem services, with its diverse community of knowledge holders' that need to be activated differently for each topic to be addressed, depending on the knowledge needs. The demonstration cases have shown, that this can be achieved for different communities (see Box 11), but it will always require dedication and continuity in the processes of the NoK.

4.4 Communication

4.4.1 Challenges

As outlined in the three chapters before, there are challenges concerning quality control, data harmonization and involvement but they can be tackled by a NoK approach. For achieving each of them, a high level of professional communication – on policy needs, processes of the NoK, data and methodologies, to name just a few– is required. This is especially true as the NoK approach is, at least in parts, new to many actors in the field and requires a high level of understanding why the processes of the NoK are designed in a certain way and do not always follow "classical" approaches of science-policy interactions.

Thus, communications in the NoK – with those involved as well as to the outside – will need to balance the need of communicating results and engaging people, but also achieve a level of capacity building to raise understanding of the processes and thus the ability and willingness of actors to get involved.

This holds especially true, as many biodiversity and ecosystem services related issues are mainstreaming issues, so a continuous broad outreach is needed to engage and make aware the relevant knowledge holders and requesters from all areas, including other policy sectors (e.g., agriculture, forestry and fisheries, climate and transport) and their stakeholders as well as different scientific disciplines. Here a major challenge lies in the translation of problems to be tackled and the results achieved into the language and mindset of those sectors and disciplines. This will require dialogue with the policy 'requesters' to understand their needs in terms of process and outputs from the NoK.

Connecting and motivating the different actors to be involved in the NoK, requires broad and clear communication on (1) the added values of involvement for both knowledge holders and requesters, and (2) current and future projects tackled by NoK.

4.4.2 Lessons learned

General lessons: Many experiences over the last years have shown that internal and external communication in science-policy interfaces are of major importance to ensure credibility, legitimacy and relevance of a process. The problems of the "Climategate" discussions of IPCC for example showed that a professional communication about the processes of getting to specific results, and the way potential mistakes are tackled are important for the perceived credibility of a process[48]. The lessons from the

[48] See for example: Hajer, M.A. (2012): A media storm in the world risk society: enacting scientific authority in the IPCC controversy (2009–10).- Critical Policy Studies 6: 452-464, and Beck, S. (2012): Between Tribalism and Trust: The IPCC under the "Public Microscope".- Nature and Culture 7(2): 151-173

TEEB process, on the other hand, show how helpful a joint framing from policy and science in combination with a suitable outreach campaign can be to promote findings from science-policy processes effectively and engage more than 500 experts in different sets of reports and other activities within a relatively short timeframe[49].

Lessons learned in the KNEU project: Within the first phase of the project, which organized general discussions on the NoK approach and specifically the prototype for answering-decision-making-needs, it became obvious that the implementation of such an approach requires a very reflexive process that sometimes seems to be contradicting approaches used in the more linear model of policy advice that many experts are familiar with. Accordingly, explanation and joint framing of the process, goals and activities is very important to gain internal acceptance and motivation to participate in a NoK process.

Wherever the processes had been discussed and understood, many experts (and decision makers involved) where supportive. Accordingly this internal communication has to be taken very seriously and cannot just be a sideline in the work of the NoK in order to achieve legitimacy and general acceptance of the process. Only then a broad engagement (see chapter 4.3) can be achieved.

Putting communications into the centre of the work will also be relevant as the conduct of knowledge synthesis will necessarily need to focus on relevant protocols in order to ensure credibility, but also needs to ensure that it stays relevant as the work develops. An essential step will be to clearly identify shared goals, expectations, roles and responsibilities and working practice at the start of collaborative working with other initiatives and ensure regular two-way communication throughout the process.

Accordingly, the NoK's strategy should plan to present results not only from a scientific point of view, but adapt the language for the requesters and provide help with the interpretation of the results in the context of their work. This problem can very often be found in research projects[50], but also the demonstration cases of KNEU faced this challenge of bringing back the scientific findings of the synthesis phase into the relevant context of decision-making. To improve the policy usability of the outputs the evaluation suggests that the NoK seeks information on needs at the start of the process to help frame the question with the target audiences (see scoping process, chapter 3.3), consistently use language which is relevant and understandable in the policy community and selects and prepares appropriate tools to disseminate this information to the target audiences and wider.

In addition, and linked to the quality control challenge (see chapter 4.1), communication needs to be clear about the quality of results and their level of certainty.

The NoK will also have to maximize on innovative, creative and dynamic tools to improve interaction with knowledge holders and within its working groups, i.e. regular meetings, but also to propose tools and technology solution to facilitate the communication between knowledge holders (for example social media, interactive website platform, wiki, e-conference). However, when feedback and timely contributions are needed, using targeted communication with individuals will be more effective that group communication. Finally, in order to keep attracting actors, the NoK needs to develop and maintain a strong position in the international context.

---

[49] Ring, I., Hansjürgens, B., Elmqvist, T., Wittmer, H. & Sukhdev, P. (2010): Challenges in Framing the Economics of Ecosystems and Biodiversity: The TEEB Initiative.- Current Opinion in Environmental Sustainability 2: 15-26

[50] See for example Neßhöver, C.; Timaeus, J.; Wittmer,H.; Krieg, A.; Geamana, N.; van den Hove, S.; Young, J.; Watt, A. (2013): Improving the Science-Policy Interface of Biodiversity Research Projects.- Gaia 22: 99-103

A communication strategy will also need to address how to reach out to new actors beyond the "usual suspects", which proved difficult in the KNEU project so far. As EU coordination action, KNEU maybe did not have enough traction to attract new groups (e.g. the private sector, different DGs of the European Commission) as those actors already working at the interfaces between sectors might currently be overloaded by similar processes and KNEU as project appeared as lower priority.

Finally, a communication strategy will also need to include the usual PR elements including marketing approaches, press releases, an informative, focused and dynamic website, conference presentations etc. to raise awareness on the NoK, and communicate the approach and results of the NoK. For this, the NoK will need the involvement of communication specialist in its development and implementation.

4.4.3 Added values of a NoK

The main added value of a NoK in terms of communications would be that the complexities arising from the broad topics to be discussed, and the needs to do this in a sound process described here, would be streamlined into one pro-active strategy of communications; an approach that can hardly be done by individual processes like projects or institutions. In terms of credibility, this is a major issue that require dedicated resources, e.g. via a person in the secretariat working full-time on this subject.

Today, communications about processes, the methodologies used to derive recommendations for policy, and the uncertainties behind these recommendations is rarely done explicitly, and this again would serve the credibility of the process if the NoK does so.

Finally, a coherent communication strategy including all functions of the NoK also enables stakeholders from all sides to find different entry points into the knowledge landscape and thus contribute to building the "Community of Interest" that is the backbone of the work the NoK could conduct.

4.5 Capacity building

4.5.1 Challenges

Building capacity involves developing understanding and fostering trust across groups, creating new links and applying new skills and knowledge which may influence attitudes, behaviors and actions of individuals, institutions and across the system as a whole.

In the context of strengthening the science-policy interface, key challenges include the need to ensure sufficient capacity and skills in the coordinating team in addition to scientific understanding. Skills such as facilitation and negotiation may be key to implementing the NoK processes and building capacity. These skills will be vital to coordinating the interactions between scientists, practitioners and policy makers in the process.

Another important challenge is the need to better understand the policy decision-making process. For example the influences on decision making and how knowledge is used.

Capacity building in the expert groups will require the inclusion of a wide range of perspectives, skills, expertise and knowledge sources from the start, including sufficient expertise and understanding on social science methods and theories. For everyone involved, capacity building will be needed to ensure that the understanding of the processes carried out is on the same level.

A final challenge is to increase understanding on methods to integrate different knowledge types by biodiversity science groups within the NoK and across Europe. For example, skills and capacity to conduct systematic reviews in the environmental sector is very low.

4.5.2 Lessons learned

General lessons: Much of the earlier work on the science-policy interface (on biodiversity and beyond) has highlighted the challenge of ensuring a 'level playing field' of understanding, awareness and trust among participants in science-policy-interface activities (e.g., SPIRAL Handbook 2013). Also, a lack of continuity in activities and in the persons involved, both from science and from decision making appear to be a challenge and need to be tackled (see for example Neßhöver et al. 2013).

Lessons learned in the KNEU project: The project highlighted a number of successes in developing new knowledge and skills to those directly involved in the NoK process as well as contributing knowledge to the wider scientific community. The process of gathering scientific knowledge from different sources contributed new knowledge to the scientific community, for example gathering and integrating data from across Europe on trends in kelp beds in the marine case study. In addition, the identification of knowledge gaps within the case studies, for example by developing a systematic map in the agriculture cases to show what knowledge is available, also contributed valuable information to the scientific community. Furthermore many scientists involved in the case studies highlighted that as a result of their involvement in the case studies they had developed their knowledge on processes and how better to manage them in the future by observing how the coordinators managed the case study experts and workload. There was also an increased understanding about previously unfamiliar methodologies, particularly relating to systematic reviews.

There were also benefits of group working within the expert group to foster knowledge exchange, particularly across different geographic areas of Europe and between scientists and practitioners. The strong interactions between scientists and practitioners in the case study were also perceived positively. Group working with those beyond usual networks, in new collaborative projects and expanded networks, is therefore a key aspect in the learning participants can gain from the NoK. This strengthening of links with existing networks and organisations in the future could therefore also be beneficial to better exchange knowledge and skills at an organisational level and with wider society.

A useful capacity building activity within a NoK will need substantial ressources to especially address the imbalances in terms of engagement into science-policy processes across Europe. Capacity building and also involvement from Eastern and in parts Southern Europe needs to be supported proactively via the NoK or accordingly funded processes working closely together with it.

On a general level, the discussions on the development of the NoK, especially in the regional workshops and the two conferences already had a strong element of mutual capacity building between many different actors on experiences and challenges with science-policy interactions – between SPI experts, natural and social scientists, practitioners and policy-makers. So, a capacity building function is to some extent built in all activities of a NoK. This is important to keep in mind when designing its processes and for example accepting that different levels of knowledge about the processes will lead to different paces in which those might be conducted.

4.5.3 Added value

Capacity-building will be an important aspect of any future NoK, requiring support not only from funders, but through strengthening links with all kinds of knowledge hubs – organisations, networks and initiatives at both the European and to a lesser extent national levels. A process of reflection and learning must be central to the NoK to help build bridges and reduce gaps between groups and move ever closer to collaborative working and information sharing. With that, a capacity building would work on the individual expert level, but also on the level of organisations.

An important aspect is to build skills to co-design processes in terms of delivering outputs as well as allowing to some extent for according capacity building "on-the-fly". These skills need to be embedded in the bodies of the NoK and its coordination team. Here, a major added-value could be achieved by creating a new environment via the NoK community that enables a continuous process of capacity building on SPIs, and allows an improved networking on SPIs and other skills needed in the NoK, e.g. on common methodologies.

5 BiodiversityKnowledge in more detail: guiding principles, potential models and a recommended governance design

The aim of chapter 5 is to derive a recommended design for a governance structure of BiodiversityKnowledge, the Network of Knowledge (NoK) proposed to support decision making on biodiversity and ecosystem services in Europe. During the development and testing of the prototype, it became quite clear that some decisions, related to the operationalization of BiodiversityKnowledge, needed to be further discussed or at least the range of choices needed to be highlighted to guide future decisions. Those elements of decisions were mostly related to governance, and were often very inter-dependent. To decrease the complexity and derive appropriate structures three main items help to frame decisions on governance:

- Key principles of BiodiversityKnowledge (see 5.1)
- Gradients of operationalization (see 5.2)
- Potential building blocks (see 5.4)

Next, the potential role of different actors is discussed (5.3), before three models of governance structures are presented (5.5); two extreme models – a 'network' and a 'platform' model (5.5.1-5.5.2), and a recommended NoK design (5.6), which was derived from the strength and weaknesses of the two extreme models. This recommended design is then complemented by discussions of the finances (5.7) and a roadmap for its implementation (5.8.). This recommended design is a proposal from the KNEU project: it is meant to trigger discussion on the wider process of improving the science-policy interface on biodiversity and ecosystem services.

5.1 Key principles of BiodiversityKnowledge

The key principles[51] serve as orientation in the choice of governance model and in deriving operating procedures.

5.1.1 The mission of BiodiversityKnowledge:

BiodiversityKnowledge is an initiative by researchers and practitioners to set up and operationalize a Network of Knowledge to improve the knowledge flow between biodiversity knowledge holders and users in Europe.

The goals of BiodiversityKnowledge are to answer questions from decision making, to improve the evidence base, to contribute to developing a research strategy, and to enable societal actors to make better informed decisions concerning biodiversity and ecosystem services.

The approach of BiodiversityKnowledge strives to integrate all relevant forms of knowledge to answer questions jointly formulated with decision makers using transparent and rigorous procedures. Throughout this approach, BiodiversityKnowledge relies on and provides networking, actively builds capacity and engages in learning on all aspects of knowledge interfacing. Accordingly, the processes of BiodiversityKnowledge matter as much as topics and outputs to ensure a coherent and credible approach.

---

[51] During the course of the KNEU project, the principles, initially inspired by the principles of the Cochrane Collaboration, were revisited several times to ensure they cover relevant issues for achieving the objectives of the NoK. The mission statement has also been reworked in order to ensure coherence with the whole set of principles and the results of WP4 (evaluation of the process).

5.1.2 Principles of BiodiversityKnowledge

BiodiversityKnowledge will…

1. enable **OPENNESS** by wide participation from all potential actors, including relevant experts and knowledge holders, through open invitations for participation, building on participants' enthusiasm and diversity, and ensuring open access to the NoK products.
2. **ENSURE QUALITY**, by applying established and tailored methodologies, developing systems for quality assurance including extended peer-review, and responding to feedback.
3. **MINIMISE BIAS and ENSURE FAIR and TRANSPARENT PROCESSES**, by ensuring scientific rigour, broad participation, and by avoiding conflicts of interest, through clear rules and procedures.
4. **AVOID DUPLICATION** by collaborating with relevant established institutions to maximize efficiency and minimize costs in science-policy interactions.
5. integrate **CAPACITY BUILDING** as essential component to improve collaborative working and information sharing.
6. ensure strong internal and external **COMMUNICATION.**
7. integrate **REFLEXIVITY and LEARNING**, by ensuring that processes and results are continuously and formatively evaluated.

To put these principles into practice the NoK requires a clearly structured and transparent, but flexible process, which defines roles of different actors and ensures acknowledgement of involvement. The governance structure and the rules and procedures should be clearly defined internally (following processes, protocols and defined roles) as well as externally (transparent selection of experts, including the whole spectrum of expertise available).

5.2 Gradients for the operationalization of the NoK

A large range of different governance structures is possible for operationalizing the NoK. Based on the principles above and the challenges outlined in Chapter 4, we have derived relevant aspects. We realized the different options for governance structure can be conceptualized as gradients rather than alternate options (Table 5.1). These gradients help orient the decision on a model of governance.

The first gradient deals with **duplication** and the **level of overlap with existing institutions** and it takes into account that there is always a certain level of competition between institutions in the science-policy-landscape. The **investment of additional resources** is the second major gradient. There was general agreement across the discussions in KNEU that additional resources need to be invested, but that the level of in-kind-contributions vs. additional resources can vary considerably between different models of funding (see also 5.6.) and governance. As in-kind-contributions also create commitment, a NoK might mainly look for a mixture of resources to be invested. Also, a high level of "new funding" might increase dependency on funding bodies. A high level of **openness** is crucial for the NoK, as outlined in the first principle. Accordingly, entry barriers to get engaged into NoK activities should be low. **Inclusiveness of knowledge forms** into the NoK is also essential but will strongly depend on the nature of single requests, as sometimes only a restricted set of very specific knowledge might be needed or valid.

Policy dependency, or in other words: independence from policy (and other vested interests) is also crucial, but needs to be balanced with the issues of legitimacy and relevance of the work of the NoK. Consequently, processes to establish a partnership between the NoK and policy, e.g. via the joint scoping of requests, is important, a potential membership of policy or other decision makers in the NoK decision bodies would need critical reflection. For the **types of requests** a NoK would respond to, it is difficult to foresee which area will be most prominent: With many smaller (and short-term) requests, the NoK might improve its relevance, but might also compromise a high quality standard of its outputs and thus endanger its credibility. Accordingly, for different kinds of requests, the operational criteria for accepting requests (see chapter 3.5) need to be explicit and acknowledge possible trade-offs.

The **timeframe** for the NoK will need to take into account time for constitution and consolidation where a mid-term perspective beyond the usual project-bound timeframe of a few years is crucial. Whether it needs to become a permanent institution remains to be seen.

Table 5.1: *Gradients to be considered while deciding upon a governance structure with indication (blue boxes) of the realistic range based on the principles and the identified challenges (see chapter 4).*

**Level of overlap with existing institutions (duplication)**	
Competition	Complete complementarity
**Investment of additional resources** (the „cheaper“ the more relying on in-kind-work)	
Cheap	Expensive
**Openness** (level of ability for “everybody” to get engaged in major elements of NoK work) [> legitimacy]	
Restricted Core group	Broad participation
**Inclusiveness of knowledge forms** [> relevance & legitimacy]	
“Purely” biodiversity science driven	Integration of all knowledge forms
**Policy dependency** (level of influence of policy in shaping work plan, including methods etc.) [> rel. & leg.]	
Completely independent	Strong influence
**Types of request in focus (including individual timeframe per request)** [> credibility]	
Many small ones (with high level of informality)	Few big ones (IPBES-like assessments)
**Timeframe for whole**	
Timebound project structure	Permanent institutions

5.3 Roles of knowledge providers and policy

5.3.1 Overview of roles of knowledge providers

A proper acknowledgment of contributions from institutions and individuals is at the core of a NoK. This will make it functional and able to deliver timely, credible, relevant and legitimate results.

A) Individuals' engagement

In general any individual with relevant disciplinary, policy or practitioner-based expertise can be a contributor to the NoK. This scientific, practical or policy expertise would be verified using a record of scientific outputs (papers, projects etc.), grey literature (reports, written contributions to various science-policy or science-practitioner interfaces) and/or by recorded experiences in management and policy (See Annex 6 on expert selection). Involving different types of expertise and experience will be crucial to ensure that the outputs will fit into the relevant processes in science, management and policy.

Individuals will be able to be active in the NoK on several levels (see also Figure 3.3):

1. via the networking function (NET function) in registering as knowledge provider and thus as *individual member* for the NoK. This will allow individuals to provide input to ad-hoc requests, discussions and other fora on the e-platform and have easier access to notifications in the NoK on other activities[52]
2. by becoming an *active individual member* (for a given time) in ad-hoc scoping or working groups or other activities[53]
3. by becoming an active member of regular bodies of the NoK for a given time[54]

While the first activity will follow a social network approach, where activity will heavily rely on self-initiative, the second will include an active identification of potential contributors by the Knowledge Coordinating Body (KCB) and via knowledge hubs.

In terms of acknowledgement, it will be important to highlight contributions from individuals on all levels, but especially when they become active contributor to activities linked to the Answering-Decision-making-Needs-function (ADN) (see chapter 3.3).

One major challenge here is to keep potential experts on 'continuous standby'. This will require a dedicated communication strategy from the NoK to enable knowledge holders to become actively engaged when their knowledge is needed[55].

B) Institutions as core members and knowledge hubs

Although it will mainly be individuals that become engaged in NoK activities, the involvement of institutions plays a decisive role in accessing and activating these experts. Research institutions, projects, research networks and learned societies are increasingly linked and communicate via electronic means so that information and requests can be easily distributed among them, thus they can easily serve as

[52] see chapter 3.2 for further explanations on the idea of creating a Community of Interest

[53] e.g., in conferences and other activities identifying research needs on the research strategy function

[54] e.g, the KCB, see below page 59

[55] One pitfall here is that most experts (and even most knowledge hubs) are highly specialized and most topics under discussion in the NET-function activities and especially the cases addressed in the ADN- or the RS-function will be outside of their specific scope of interest. Thus, there are few incentives to regularly check whether their knowledge might be needed.

knowledge hubs to provide knowledge but also to forward knowledge requests into their respective communities. Often, individual experts will even be linked to different hubs, e.g. via their own institute, a collaborative project, and a learned society.

As many institutions have the support of policy or societal processes in their overall mission, there is often an intrinsic interest to get involved in a broader process, but nonetheless, as many discussions in KNEU have shown, their visibility in the process needs to be ensured and other incentives need to be available. For a number of partners, especially in Eastern/Central and Southern Europe, financial support will be needed to become engaged in the NoK[56].

The challenge is to attract such institutions as core supporters or partners in the NoK, to ensure visibility of leading partners, motivate their experts to contribute, but at the same time keep the NoK and its bodies open for other actors on the institutional as well as the individual level (see previous section and gradient discussion).

As Europe has a multitude of networks and institutions, "networking the network" is of specific importance and plays a crucial role when outlining potential design options. For different kinds of institutions, different roles can be identified in terms of their contribution to the NoK to align their interests and expertise with the principles of the NoK. Those main roles are[57]:

- **Provision or expertise role**: all institutions, networks and projects can play a role in providing expertise via their individual members, employees etc., and by motivating and allowing them to contribute, will provide the biggest support for a NoK
- **Knowledge hub role:** most institutions, networks and projects, but especially learned societies can provide an important *internal* communication role for the NoK community by inviting and motivating their individual members to get engaged in the NoK, especially by highlighting NoK activities of special relevance for their members (e.g., learned societies such as EEF, IALE-Europe, SCB, INNGE; Networks: ALTER-Net, MARBEF; large national research institutes, major research projects: EUBON, projects funded by BiodivERsA, national biodiversity hubs: FRB, Belgian Biodiversity Platform, NeFo; NGOs: IUCN, WWF; and many more)
- **Management support role**: Some institutions with experience in interface work, might directly support the NoK with their management expertise in this field (e.g. ECNC, CEH, ALTERRA, UFZ, Ecologic, FRB and others)
- **Data, information provision role**: Major players working on the integration of biodiversity and other data to provide data and baseline information for requests where this might be needed (e.g., EUBON, GBIF, Lifewatch, PESI)
- **Tools and infrastructure provision role**: Institutions that work on providing relevant tools and the according infrastructure for specific work on integrating knowledge, e.g. via systematic review and meta-analyses (CEE) and similar activities, but also via provision of potential research sites (e.g., Lifewatch, LTER-Europe, ALTER-Net, MARS, CESAB)

---

[56] see recommendations from the according regional workshops: http://biodiversityknowledge.eu/index.php?option=com_content&view=article&id=42&Itemid=142

[57] The reference made to specific institutions and networks here is based on a survey among them about their potential contributions to and benefits from the NoK during the 2nd BiodiversityKnowledge conference, September 2013

- **Capacity building role**: Many networks are active in capacity building, especially for junior experts, and could thus support the capacity building on engagement into a NoK (e.g., IN-NGE, ALTER-Net, ECNC)
- **Communication support role**: Many institutions have well established routes for *external* communication with society and stakeholders, which could be used for broadcasting results and other relevant news from the NoK (e.g., institutes, NGOs, long-term projects)
- **Society and policy interface role**: Many institutions actively engage in the science-society interface already, and could thus act as partners for the NoK for specific activities, by providing access to their specific communities as well as bringing in their own processes, if in line with the NoK principles (e.g., Networks of excellence, "applied" learned societies: SCB, IALE-Europe; ECNC, ENCA, IUCN etc.)

This diversity of roles outlines the difficulty in subsuming institutions and networks in one category of actors in a NoK. What will be needed are individual Memorandums of Understanding between the NoK and such institutions to build on their specific expertise for one or more roles, rather than asking them to support all of them. Accordingly, the benefits they would derive from contributing to the NoK would be much better tailored to their interests.

5.3.2 Link to European policy and decision making: mandated but independent

As outlined earlier, a successful NoK will need to have close connections to policy to ensure that it is part of an overall EU mechanism on biodiversity expertise (see chapter 2.3). For this connection, it might be relevant for the NoK to receive an explicit mandate from one or more policy bodies to ensure that its work and results are relevant and acknowledged as an important input to decision-making processes. Also, according to interviews with knowledge holders, a mandate would increase their motivation to participate in NoK working groups and activities.

At the EU level, different policy institutions exist that could play a role in connecting (and probably explicitly mandating) a NoK. In general, this could be

- The European Parliament or a sub-body of it
- The European Commission via one or more of its DGs (in the case of biodiversity and ecosystem services, for example DG Environment and DG Research, DG Agri and DG Mare)
- Expert groups of the member states in the EU contexts (e.g., the Working Party on International Environmental Issues (WPIEI) for international activities or the Coordination Group on Biodiversity and Nature (CGBN) on EU related issues) – these groups would reach out to the Member States and thus ensure their involvement
- The Nature Directors meeting or relevant body of the Member States
- The Programme Committee and the newly formed Advisory and Expert Groups in Horizon 2020 which may be interested in linking up for the research strategy function
- Chief scientific advisors of the Commission or other relevant institutions

Which of these bodies (or another or a combination of them, e.g. working together in a forum) could serve as main contact point for a NoK once it has been set up and is running, will need to be further discussed between the EU and the Member States.

5.4 Potential building blocks for a governance structure of the NoK

Building blocks are interchangeable units of decision of a governance structure, i.e. several versions of a same unit, to make the NoK operational. They can be complementary or dependent on each other. Each building block should be evaluated against the established principles of the NoK and against the implications for the gradients, e.g. gradient of cost, independence, see Table 5.1.

From designing the prototype for the ADN-function (see the detailed narrative in Annex 3 and at www.biodiversityknowledge.eu) and taking the analyses of challenges in chapter 4 into account, a general and a minimum set of governance bodies can be identified to ensure the coherence and added value of a NoK. The following sections shortly introduce these bodies as basis for discussion of the design options, beginning with the minimum set of governance bodies needed to ensure the NoK primary functions.

Different types of governance issues will need to be tackled by a set of building blocks. Different combinations of these blocks open an option space for the recommended governance structure, and are explored in the next section with the following questions in mind:

- What will be the role of knowledge/science? What should/could the NoK bodies decide?
- What should be the role of policy? What should policy decide?
- What are the roles and intentions of the different bodies involved?
- Who takes decisions on nomination and selection of important bodies (KCB, Assembly, Steering committee, etc…)?
- What are the criteria for nomination and selection of the KCB….?
- Who is going to publicly explain and "defend" the work of the NoK in case results or processes get challenged?
- Who can make early decisions on the governance issue in initial stages of the implementation?

5.4.1 Minimum set of building blocks

Some building blocks are a minimum requirement for a NoK to ensure the delivery of its main tasks – knowledge coordination, administration, working groups and a link to policy and decision-making.

A) "Knowledge coordinating" building block

To ensure continuity and efficiency in the work, the minimum setting for this building block will need to be "institutionalized". In the prototype of the NoK, this was already referred to as the Knowledge Coordinating Body (KCB).

The KCB is the central decision body of the NoK (comparable to a steering committee in many institutions), it decides in collaboration with knowledge requesters which and how requests to take up in the ADN-function, ensures responsiveness of the community, i.e. activates the NET function and orchestrates the whole process to answer the request. For each request, the KCB maintains and facilitates dialogue across actors, which it may have identified jointly with requesters in the first place, or which may enter the process at later stages (e.g., in review processes).

The composition and selection of the KCB will be subject to detailed discussion in the next section, as possible roles of the KCB may vary according to different governance structures of the NoK. Depending

on the level of political linkage, the KCB could be composed solely of knowledge holders (networking option) or a combination of knowledge holders and ex-officio decision-makers (delegated by the mandating policy bodies). In any case, as the central body of the NoK it will require a set of dedicated and skilled experts not only on biodiversity and ecosystem services, but also on interface processes, interdisciplinary work, communication, methods for assessment processes and research strategy, depending on the functions fulfilled by the KCB.

B) Administration building block

The KCB should be supported by a secretariat, responsible for the administrative functions of the NoK. The secretariat would schedule and handle the day-to-day work and budget, and support the work of the KCB and the NoK in general by linking with knowledge hubs.

The secretariat will oversee and guide the processes to answer requests from decision makers. Experiences from the KNEU test cases and from other processes have shown that going through the procedures, particularly of the ADN-function, requires a thorough overview of the process and constant follow-up.

KCB and secretariat should be seen more like a conductor of an orchestra composed of experts: they are responsible for linking requests with individuals or hubs of experts to answer requests (governments to civil society).

The basic tasks to be fulfilled by KCB and secretariat, if no other bodies are added to complement their work, is therefore managing daily business, coordinating the knowledge holders and requesters and conducting and communicating the NoK processes and products.

C) Working groups

In order to organise the work of the NoK on specific requests (ADN-function), *scoping groups* as well as *thematic ad-hoc working groups* will need to be set up. The scoping groups will be responsible for a detailed scoping of a topic and will include high level experts, information managers/expert-librarians able to scope literature in an efficient way, the requester(s) and additional stakeholders on the given topic. From this group, the thematic working group will be set up, most likely including additional experts following the identified needs during the scoping and the methods to be used. The requesters will be able to follow the work directly, but will not be members of the working groups themselves.

While in general it was agreed that working groups should be *ad-hoc* and only be installed for specific requests, it might also be relevant to install *open-ended working groups*, especially in the further development of methodological approaches to be used in the ADN-function and on the work to develop the NET-function, which is the basis for identifying experts for the thematic *ad-hoc* working groups.

Also, for the research strategy function a working group could be envisaged, which would serve a similar role as currently the EPBRS Steering Committee, which organises relevant research strategy processes through broad involvement and facilitated discussions.

Tasks and responsibilities of working groups would need to be further defined. As a baseline, guidelines for authors and reviewers as used in IPCC, MA, the Cochrane Collaboration, and similar processes could be used[58]. Also the composition of working groups should reflect a range of expertise and views

[58] e.g., see IPCC principles and procedures Appendix A, Annex 1

from different disciplines, forms of expertise, and geographical representation[59] (see Box 14 for first experiences on this process in KNEU).

BOX 14: Experience in identifying and involving experts for working groups from the KNEU demonstration cases

The KNEU test cases identified and involved designated experts into request-driven thematic working groups. All three test cases were able to involve a significant number of experts but also experienced some general challenges, which includes:

- General calls for involvement (via email) most often don't yield much feedback. Identifying and addressing experts personally was more successful. [An established, renowned structure like a NoK may improve this situation]
- Willingness and ability to participate is generally higher in member states from northern and central Europe. Colleagues from eastern and southern European countries face strong constraints in terms of resources, which hamper their contributions
- Open calls in general will most often attract experts that are already active in science-policy activities. Colleagues not familiar with such processes but with an important expertise might not get involved
- The limitations in time resources of most experts willing to get involved are a strong barrier. Accordingly the most appropriate way of involvement and method to be applied might not be used because of these constraints.

As a consequence working groups will need (a) a dedicated person responsible for the management and communications within the group and (b) additional financial resources to support travel and in some case also working time of working group members.

D) Policy-Link building block

Considering the approach of the NoK "to answer questions jointly formulated with decision makers using transparent and rigorous procedures" and the added value this mechanism needs to reach, an efficient policy link is needed.

This policy link building block will need to interact regularly with the NoK and its KCB. This could either be done by nominating policy delegates for the KCB, and/or regularly inviting a representative of the secretariat and/or KCB to report on the activities of the NoK in the meetings of the policy body.

For further background, please refer to section 5.3.2.

5.4.2 Additional building blocks: different options for operationalising

A minimum set to ensure the functioning of the NoK has been outlined above. However, these are not the only ones which might be needed to optimize the NoK process, which can be structured by identifying key roles that should be fulfilled in a NoK. It should be recognized that the NoK is a dynamic learning process with an evolving structure. Therefore, some aspects will be decided and adapted along the way. Nevertheless, some roles must be addressed early on and should be included in the recommended design.

[59] Compare IPCC principles and procedures, Appendix A, 4.2.2.

In addition to the essential roles which have been summarized in the section above, i.e.

- Networking of the different actors and networks
- Handling requests for the ADN function and setting up relevant working groups
- Communication within and outside the NoK

Many other roles/ functions will add value and ensure the success of the NoK. This may involve more resources but would set the NoK better apart from other processes, resulting in less competition:

- Evaluation of the NoK process, in order to form an iterative process, continuously integrating the lessons learned from the NoK work and improving it over time
- Capacity building for both the knowledge holder community and the policy side
- Potential link to international processes such as IPBES and CBD (as required)
- Visibility of the products and process ensuring the prestige of the NoK
- Explaining or publicly "defending"[60] the NoK products and processes in case of controversy or someone challenging results or procedures.

Table 5.2 provides an overview of potential building blocks to address these roles. From left to right there is an increasing degree of formalization and institutionalization of the body envisaged to fulfill the respective role.

Table 5.2: *List of key building blocks that could potentially be used in a NoK. Boxes which are crossed over are unrealistic building blocks, as they cannot fulfill the NoK principles.*

**Categories**	**Potential Building blocks**			
**Policy-link**	~~No Policy Link Body~~	Dialogue restricted with policy requesters	Policy-people involved in the NoK process (either part of KCB or Steering committee, etc.)	Policy link body (e.g. SPI forum)
**Membership**	No membership	Only Individual-based membership	Mix of Institutions/ networks/and individuals memberships	Only institutions/ networks based membership
**High level advice**	No advisory board	Non-permanent advisory board (ad-hoc group)	Advisory board composed of knowledge holders	Advisory board with representation of broader society
**Steering**	No steering committee	Non-permanent steering committee	Small permanent steering committee	Big permanent steering committee
**Secretariat**	No secretariat	Small secretariat incorporated in the KCB	Medium size secretariat	Big secretariat including IT, administrative and communication persons
**Knowledge Coordination**	~~No Knowledge Coordination Body (KCB)~~	Ad-hoc Knowledge Coordination	Decentralized Knowledge Coordination Body, part time staff	Centralized KCB, full time staff

[60] Evaluation of the NoK is in charge of providing feedback and ensuring procedures are duely followed and improved where needed. However, even if procedures are carefully adhered to, it is to be expected that particularly in controversial cases results and/or the procedures leading to the results will be challenged. In such an event, the role of "defending" or explaining the NoK is required. Its importance to maintain the credibility and traction of the NoK should not be underestimated.

<table>
<tr><th>Categories</th><th colspan="5">Potential Building blocks</th></tr>
<tr><td>Research Strategy</td><td>No Research Strategy Body</td><td colspan="2">Research Strategy Body (RSB) independent from NoK but strong link</td><td>RSB included in KCB (Research prioritization as requests to the NoK)</td><td>Separate RSB body within the NoK</td></tr>
<tr><td>Evaluation</td><td>only internal evaluation</td><td colspan="2">Non-permanent evaluation group (ad-hoc group)</td><td>External ‘formative evaluation’ body (mix between evaluation and advisory board)</td><td>External evaluation</td></tr>
<tr><td>Financing (see also chapter 5.7)</td><td>No funding</td><td>A bottom-up approach using crowd-funding</td><td>Project funding</td><td>A bottom-up approach mainly driven by members/knowledge holder institutions</td><td>Complete funding of activities by one major donor or fund</td></tr>
</table>

5.5 Options for the NoK design

After outlining different versions of more network or more platform-oriented models in previous versions of this concept paper, two extreme models of governance for the NoK were outlined in order to compare strengths and weaknesses and to be able to propose an adequate design for initially setting up the NoK. A workshop organized in Frankfurt with social scientists and other experts in January 2014 has considerably helped to flesh out the two extreme governance models (referred to as the network model and the platform model) for the NoK. In this chapter, we outline these two models for illustration.

An additional meeting with a subgroup from the KNEU consortium has then analyzed both extremes against the principles and the gradients presented earlier in this chapter and derived a recommended design, which is presented in the following chapter section (5.6).

5.5.1 Network model: based on individual commitment and self-organisation

Assumptions used when developing the model

This model is designed to be as light and additional to existing structures as possible and is thus also the least expensive (see Table 5.3). The network approach should be as open as possible and benefit from as many contributions as possible at all levels.

The assumption is that the network model should be light on bureaucracy and institutionalization, should be easy to engage with, not prone to corruption, lobbying, power imbalances or vested interests.

Illustration of the Network model of the NoK

This model (see Figure 5.1) consists of two permanent bodies:

- A combined KCB + Secretariat: KCB members being decentralized, part-time staff seconded in kind and based at their home institutions, and 2-3 permanent full-time positions covering the secretariat function, based in Brussels to create visibility and facilitate interaction with policy. The secretariat’s main role would be to provide the entry point to receive requests or suggestions by the members and to organize day to day work.
- A formative external evaluation[61] body, very closely accompanying the process, particularly in the initial years. This body would ensure that procedures are carefully followed and

[61] Evaluation that is used to modify or improve products, programs, or activities and is based on feedback obtained during their planning and development. A periodically repeated assessment of efforts prior to their completion for the purpose of improving the efforts. See Glossary.

principles are not breached. It provides an outside view combined with a full understanding of the background and the challenges involved. In addition another fully external review should be conducted e.g. after 2 and after 5 years of functioning, as the formative evaluation is too close to provide an outside view.

Characteristics of the network model are that membership would be set-up at the individual level, members would self-register, membership fees would be suggested and voluntary, donations being possible, crowd funding would be used to cover requests that do not come from policy, and project money or EC funding would have to cover basic operations. Members would vote on KCB representatives. For a start, types of requests for the Network model would be smaller first, but could become larger with increasing experiences in the NoK.

The model assumes that policy gets organized in a policy coordinating body (PCB), which would be the discussion partner for the KCB/secretariat within the ADN function.

Table 5.3: *Approximate position/evaluation of the Network model on the different gradients.*

**Level of overlap with existing institutions (duplication)**		
	Competition	Complete complem[illegible]arity
**Investment of additional resources** (the „cheaper" the more relying on in-kind-work)		
	Cheap	Expensive
**Openness** (level of ability for "everybody" to get engaged in major elements of NoK work) [> legitimacy]		
	Restricted Core group	Broad participation
**Inclusiveness of knowledge forms** [> relevance & legitimacy]		
	"Purely" biodiversity science driven	Integration of all knowledge forms
**Policy dependency** (level of influence of policy in shaping work plan, including methods etc.) [> rel. & leg.]		
	Completely independent	Strong influence
**Types of request in focus (including individual timeframe per request)** [> credibility]		
	Many small ones (with high level of informality)	Few big ones (IPBES-like assessments)
**Timeframe for whole**		
	Timebound project structure	Permanent institutions

The KCB could facilitate the setting up of additional working groups for communication, capacity building, or other specific topics of interest to the members. Research strategy development would be treated like any other policy request on a demand basis following the usual procedures envisaged for the ADN function. Table 5.4 provides further details on the composition and functioning of the model.

One suggestion for selecting KCB members is to let institutions nominate interested individuals by allowing them to contribute some of their time, these would then be elected by members (e.g. requiring a minimum vote) to establish a short list. The Formative Evaluation body could then select and thereby ensure disciplinary, gender and geographic representation.

Table 5.4: Overview of key building blocks to be used in the Network model (highlighted in red). If two or more boxes in a row are highlighted, they showcase potential options for the model.

<table>
<tr><th>Categories</th><th colspan="6">Potential Building blocks</th></tr>
<tr><td>Policy-link</td><td>No Policy Link Body</td><td colspan="2">Dialogue restricted with policy requesters</td><td colspan="2">Policy-people involved in the NoK process (either part of KCB or Steering committee, etc..)</td><td>Policy link body (SPI forum)</td></tr>
<tr><td>Membership</td><td>No membership</td><td colspan="2">Only Individual-based membership</td><td colspan="2">Mix of Institutions/ networks/and individuals memberships</td><td>Only institutions/ networks based membership</td></tr>
<tr><td>High level advice</td><td>No advisory board</td><td colspan="2">Non-permanent advisory board (ad-hoc group)</td><td colspan="2">Advisory board composed of knowledge holders</td><td>Advisory board with representation of broader society</td></tr>
<tr><td>Steering</td><td>No steering committee</td><td colspan="2">Non-permanent steering committee</td><td colspan="2">Small permanent steering committee</td><td>Big permanent steering committee</td></tr>
<tr><td>Secretariat</td><td>No secretariat</td><td colspan="2">Small secretariat incorporated in the KCB</td><td colspan="2">Medium size secretariat</td><td>Big secretariat including IT, administrative and communication persons</td></tr>
<tr><td>Knowledge Coordination</td><td>No Knowledge Coordination Body (KCB)</td><td colspan="2">Ad-hoc Knowledge Coordination</td><td colspan="2">Decentralized Knowledge Coordination Body, part time staff</td><td>Centralized KCB, full time staff</td></tr>
<tr><td>Research Strategy</td><td>No Research Strategy Body</td><td colspan="2">Research Strategy Body (RSB) independent from NoK but strong link</td><td colspan="2">RSB included in KCB (Research prioritization as requests to the NoK)</td><td>Separate RSB body within the NoK</td></tr>
<tr><td>Evaluation</td><td>only internal evaluation</td><td colspan="2">Non-permanent evaluation group (ad-hoc group)</td><td colspan="2">Formative evaluation body (mix between evaluation and advisory board)</td><td>External evaluation</td></tr>
<tr><td>Financing (see section 5.7 for further details)</td><td>No funding</td><td>A bottom-up approach using crowd-funding</td><td>Project funding</td><td colspan="2">A bottom-up approach mainly driven by members/knowledge holder institutions</td><td>Complete funding of activities by one major donor or fund</td></tr>
</table>

Strength and weaknesses of the Network model of the NoK

Strengths (see also Table 5.4):

- The structure and process are light and cheap, as mainly based on in-kind contributions, having no institutional membership will bring openness into the whole structure.
- External evaluation body: performing formative evaluation by accompanying and back-stopping the process, a combination of evaluation and advisory board.

Weaknesses

- Depends nearly entirely on good will and dedication of individuals
- KCB could be dominated by large institutions (with vested interests)
- Limited link with the policy world, and little capacity to be proactive in establishing contacts

- Practically very hard to manage and to ensure delivery and effectiveness

The fact that the **Research strategy** (RS) function is not treated separately may imply loss of visibility and independence of RS function – which should not "wait" for a RS request, but should ideally be pro-active in horizon scanning etc.

Remaining questions

- **External evaluation body**: Who will be in this body? How would they be chosen? Size of the evaluation group? How to ensure a realm of expertise in the evaluation group? Would this body be based on in-kind contributions (e.g. researchers interested in evaluation of processes).
- **Data ownership**: What about data ownership – if a member leaves an institution, they may not be able to bring their data with them, but their expertise will follow. MoUs with institutions could bridge the gap with individual members (for example encouraging in-kind contributions), and keep the institutions on board.
- **Funding & ressources**: Needs dedication and ressources of partners to support the NoK and KCB to be running continuously but how to safeguard against influence of institutions? Three year terms for KCB members (max two terms) and turnover from institutions might be able to counteract influence. Funding via in-kind contributions and support of institutions as baseline, but further support needed, e.g. for conducting requests.
- **KCB** would need a "bundle of competencies" – what could be handled by the part time members of KCB and which skills would be needed in the Secretariat?

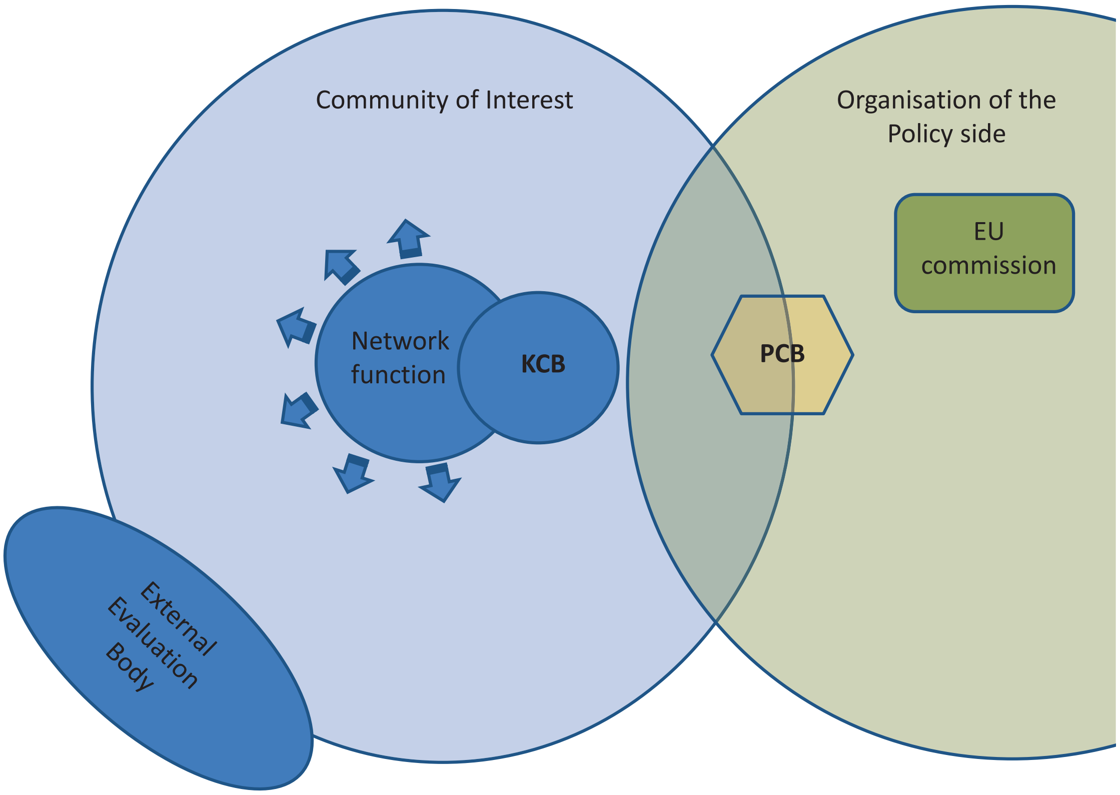

Figure 5.1: Illustration of Network model (see text for details)

5.5.2 Platform model: institutional commitment and clear allocation of roles

5.5.2.1 Assumptions used when developing the model

This model commits organisations to contribute in order to reduce dependence on outside funding and help mobilizing sufficient commitment from the outset. Different bodies will fulfil different roles and can thus divide tasks among them.

5.5.2.2 Illustration of the platform model

Membership at the institution level is the main element constituting the NoK in this model. Institutions becoming members acquire rights but also obligations to contribute. All members form an assembly which selects/elects the steering committee (see Figure 5.2). The steering committee takes all strategic decisions and is supported by a secretariat to do the work and decide on operational issues. The KCB would focus mainly on deciding on which requests the NoK can handle and on organizing the procedures required for the ADN-function. The research strategy function would be conducted by a separate body in the NoK, but linked to KCB and the other bodies.

Such a model would set up strong processes, and thus would be able to conduct different types of request, allow for a broad inclusion of knowledge forms, but would also be strongly linked to policy (see Table. 5.5.).

***Table 5.5:** Approximate position/evaluation of the platform model on the different gradients.*

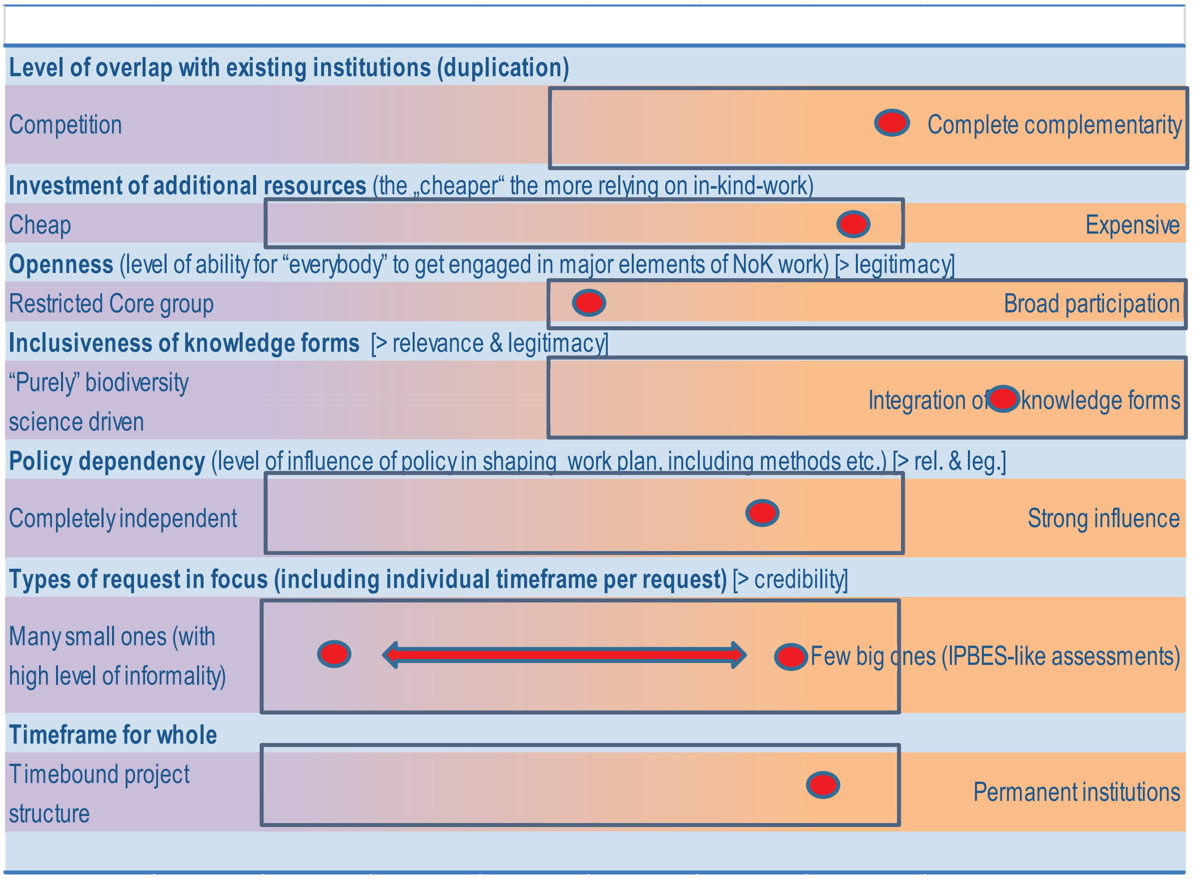

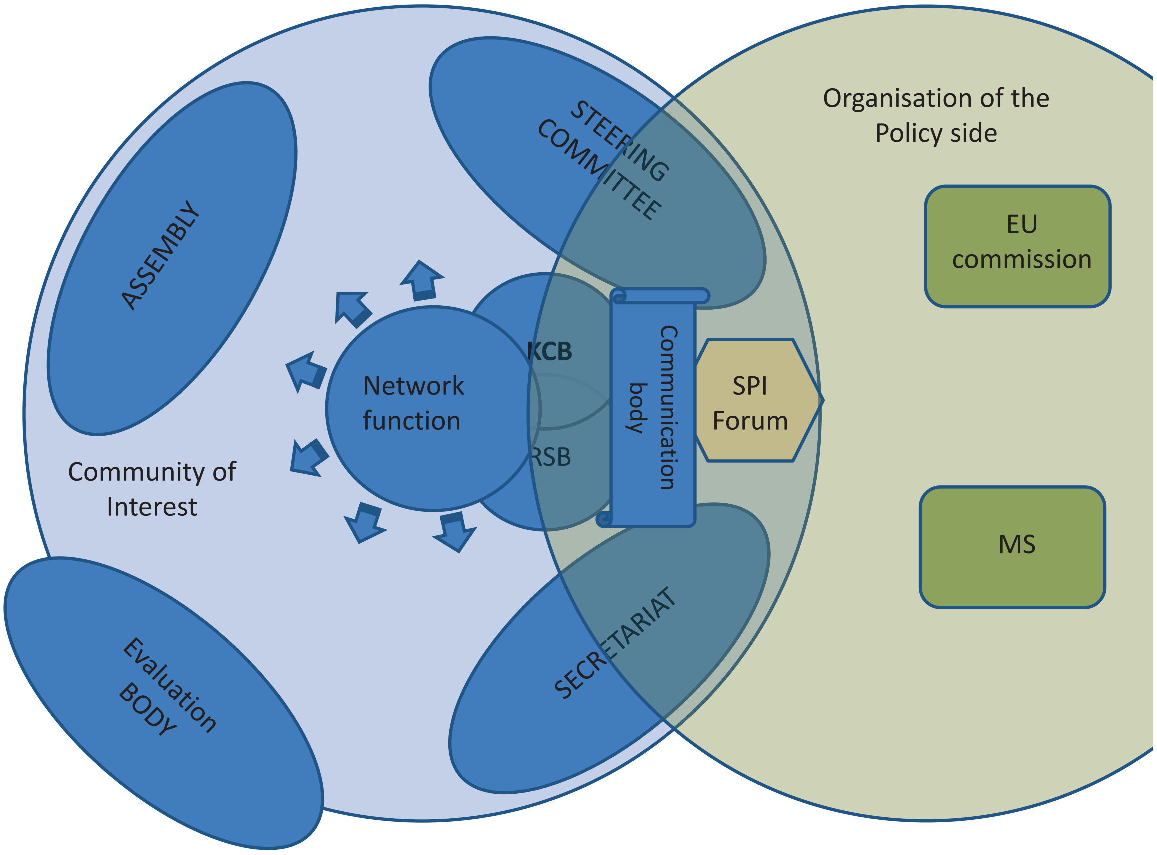

Figure 5.2: *Illustration of the Platform model (see text for details)*

The secretariat will have an important role but having three different bodies with different responsibilities will facilitate clear division of work. Membership by institutions or projects and assemblies will also help members become active and may help to attract new members. A challenge to solve is that in the network, members can very easily join, whereas a platform involving the membership of large organisations and networks can look like a closed club and well-established members might occupy leading roles over extended periods of time.

An additional evaluation body composed of both internal and external reviewers/evaluators (potentially some from outside Europe) would support the NoK in its operations and identify potential adaptations of processes.

See Table 5.6 for an analysis of building blocks needed for this model.

Table 5.6: Overview of key building blocks to be used in the Platform model (highlighted in red). If two or more boxes in a row are highlighted, they showcase potential options for the model.

Categories	Potential Building blocks				
**Policy-link**	No Policy Link Body	Dialogue restricted with policy requesters		Policy-people involved in the NoK process (either part of KCB or Steering committee, etc..)	Policy link body (SPI forum)
**Membership**	No membership	Only Individual-based membership		Mix of Institutions/ networks/and individuals memberships	Only institutions/ networks based membership
**High level advice**	No advisory board	Non-permanent advisory board (ad-hoc group)		Advisory board composed of knowledge holders	Advisory board with representation of broader society
**Steering**	No steering committee	Non-permanent steering committee		Small permanent steering committee	Big permanent steering committee
**Secretariat**	No secretariat	Small secretariat incorporated in the KCB		Medium size secretariat	Big secretariat including IT, administrative and communication persons
**Knowledge Coordination**	No Knowledge Coordination Body (KCB)	Ad-hoc Knowledge Coordination		Decentralized Knowledge Coordination Body, part time staff	Centralized KCB, full time staff
**Research Strategy**	No Research Strategy Body	Research Strategy Body (RSB) independent from NoK but strong link		RSB included in KCB (Research prioritization as requests to the NoK)	Separate RSB body within the NoK
**Evaluation**	only internal evaluation	Non-permanent evaluation group (ad-hoc group)		Formative evaluation body (mix between evaluation and advisory board)	External evaluation
**Financing (see also chapter 5.7)**	No funding	A bottom-up approach using crowd-funding	Project funding	A bottom-up approach mainly driven by members/knowledge holder institutions	Complete funding of activities by one major donor or fund

Strengths and weaknesses for the Platform model of the NoK

Strengths (see also Table 5.6)

- Institutional membership will bring explicit rights, obligations, duties and incentives for the members to invest in the process to make it work. The model thus looks more stable as it has more institutional support, while at the same time providing more visibility to institutions involved.
- The chairperson of the steering committee can "defend" the NoK/ KCB in case outcomes and/or procedures of KCB's work on organizing the ADN function are contested.
- A separate secretariat will have a strong communication role, as well as being a visible entry point and being able to approach the different bodies as needed.

- Members would be able to cover at least basic funding which would guarantee basic functioning and a certain level of independence from external funding.
- Regarding the link to the policy process, as a platform it will be more likely to be seen by EU public bodies as a representative body to provide expertise. So it is expected that the Commission will be more likely to produce a statement of support/relevance for the platform and will be more likely to engage in communication with the platform.
- The platform model is expected to allow for better management of processes because of commitments made by members.

Weaknesses of the Platform model

- Members: Membership of strong organisations and networks might weaken the openness in this model, or at least lead to a perception of a closed club with more bureaucratic procedures where individuals are less inclined to join.
- The Platform could be influenced by the institutions' vested interest or a sort of "elitist club". It is unclear how to best limit this influence and how to communicate this issue, because even without influence of vested interests, the platform might still be perceived as influenced by single members or specific stakeholders. This could be mitigated by having clear rules of engagement, which might include different levels of membership; e.g. associate memberships, individual membership…
- The Platform, with its many bodies might be too formalized, thus a lot of effort would be needed to keep it running and everyone engaged and thus would have a high need of ressources.
- As the platform is more visible, and IPCC and IPBES involve governments it might be associated as being part of the Brussels politics and not perceived as independent.
- The term "platform" might have a connotation of being "heavy, inflexible and expensive", but this could be avoided by using a different name.

Remaining questions

- Appointment procedures such as: Should the steering committee nominate people in the KCB? Should that be the secretariat? Who appoints the secretariat? Who appoints the KCB?
- Involvement of the Member States in the Research Strategy Body for this model?
- How to avoid/minimize the influence of vested interests?
- In the Platform there should not only be members, or maybe different levels of membership. A newcomer should feel welcome. What are the procedures for becoming a member? What would be the costs of a membership?

5.6 Recommended NoK design: balancing structural reliability and engagement

Both general NoK models presented in the previous chapter have clear strengths and weaknesses with respect to the current landscape of knowledge holders and requesters on biodiversity in Europe Against this background a recommended design for the NoK on Biodiversity was developed that combines the strengths of both models: keeping it manageable and less costly than a fully-fledged platform model while making use of clear divisions of tasks. A main issue when developing the recommended design was to strongly consider what this would mean in practice. To derive a realistic model, the suggested building blocks were continuously evaluated according to the principles, the pros and cons of the two models and the positioning along the proposed gradients (see table 5.7).

Table 5.7: *Approximate position/ evaluation of the recommended NoK design on the different gradients.*

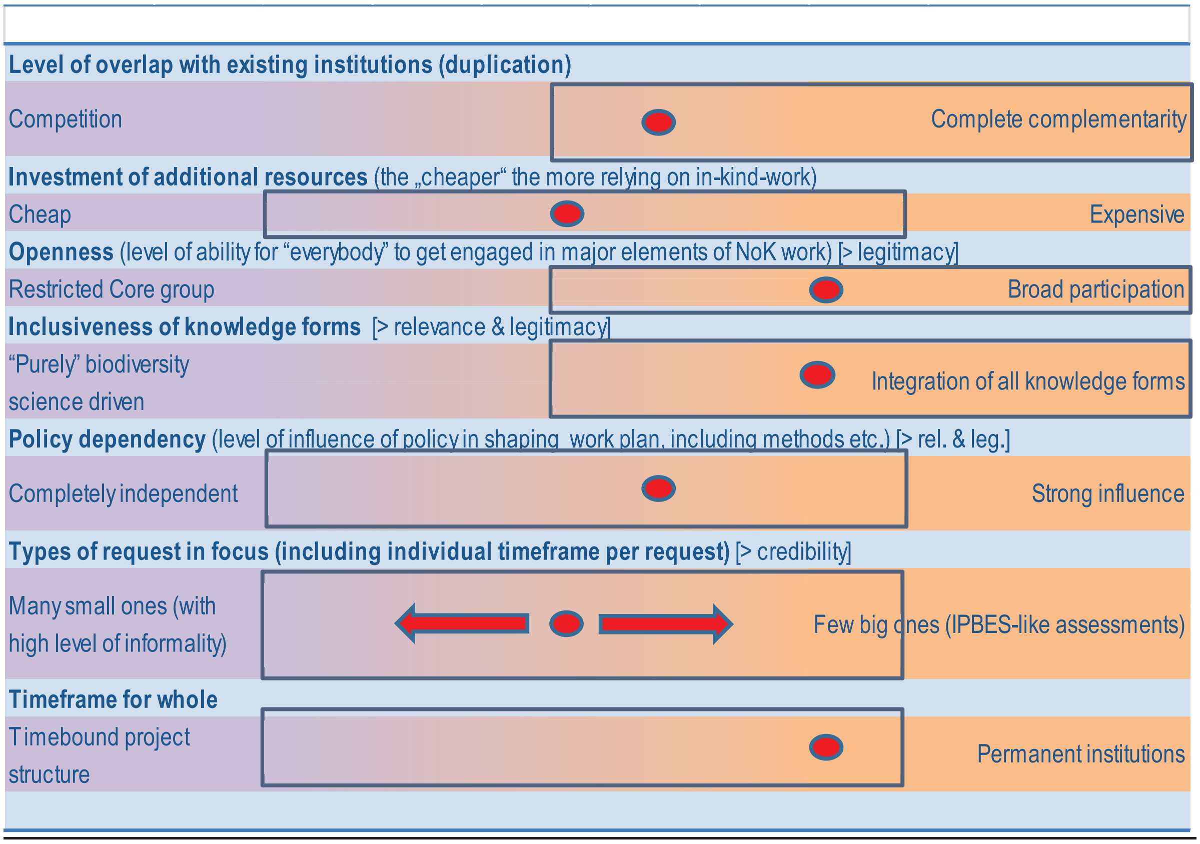

5.6.1 Assumptions used when developing the recommended design:

The following assumptions were made for shaping the recommended design. They try to connect the insights from the two models with the current situation and needs in the science-society knowledge landscape, and the challenge to balance potential trade-offs between the principles of a NoK and this situation (See also gradient table 5.7 for this model):

- The NoK should be open for active engagement and link up with existing institutions in order to ensure mutual benefits
- At the same time, it should enable openness and inclusiveness in all its aspects of operations

- The number and size of bodies should be as small as possible, but still ensure efficient work as well as high-quality processes
- Independence from vested or political interests is assured by principles and key procedures that are transparent and organized according to scientific standards
- The types of requests to be addressed would be flexible in size and ambition, probably starting with few medium sized ones allowing for a good implementation and traceability of approaches and process
- With respect to funding and support, the model should allow in-kind- as well as financial contribution via different pathways

5.6.2 Governance of the recommended design for the NoK

The recommended NoK design has four main bodies (see Figure 5.3):

A relatively large **part time decentralized Knowledge Coordination Body (KCB)**, which meets regularly (6-8 times/year) responsible for the external engagement and strategic development in addressing the three main functions: networking (2 dedicated members), research strategy (2 dedicated members) and ADN (6 dedicated). The ADN function would need more experts, as it will need a mix of experts on methodologies and processes, as well as some with a broad thematic overview and diverse disciplinary background. For each function, the dedicated experts would serve for a term of 3 years (and could be reappointed once) one for each function would take up a leading role. A spokesperson would be elected out of these experts. Members of KCB are in charge of their specific tasks to ensure division of labour and clear responsibilities, but decisions are taken jointly, e.g. decisions of which requests are tackled in the ADN function, and which other activities are taken up based on available resources and overall development (for more details, see table in Annex 8).

A **separate Secretariat**, as entry point for all communications, coordinating and supporting the overall work flow processes in the different activities of the three functions jointly with the according experts in the KCB, and conductor of day to day work. The secretariat would consist of a coordinator, two additional scientific process managers, a communication expert, and a part time administrative assistant as a minimum. It should ideally be located in Brussels and host the meetings of the KCB.

An **Advisory Board** that will allow involvement of a broad set of additional expertise, to follow the work of the NoK and serve as ambassadors of the NoK and provide advice on strategic decisions. The Board would consist of high-level experts from all areas of expertise (including policy and society), mainly invited due to the distinct roles they have in their institutions and their experience in the field of environmental and science policy. The chair of the advisory board would be able to publicly explain and defend the work of the KCB in case (correctly followed) procedures are being challenged. The advisory board thus has a strong facilitative role between different actors; a function, that hasn't been in focus on the Network or Platform model.

The **formative Evaluation Body** is kept to ensure that procedures and principles are followed and to provide outside advice for improving processes of the NoK and ensure flexibility and learning. In the evaluation body, an ombudsman could also be included that gets active in case of challenging situations during the NoK's work. In addition there should be an external evaluation after 2 and 5 years.

See table 5.8 below for details on the composition and functioning of the model.

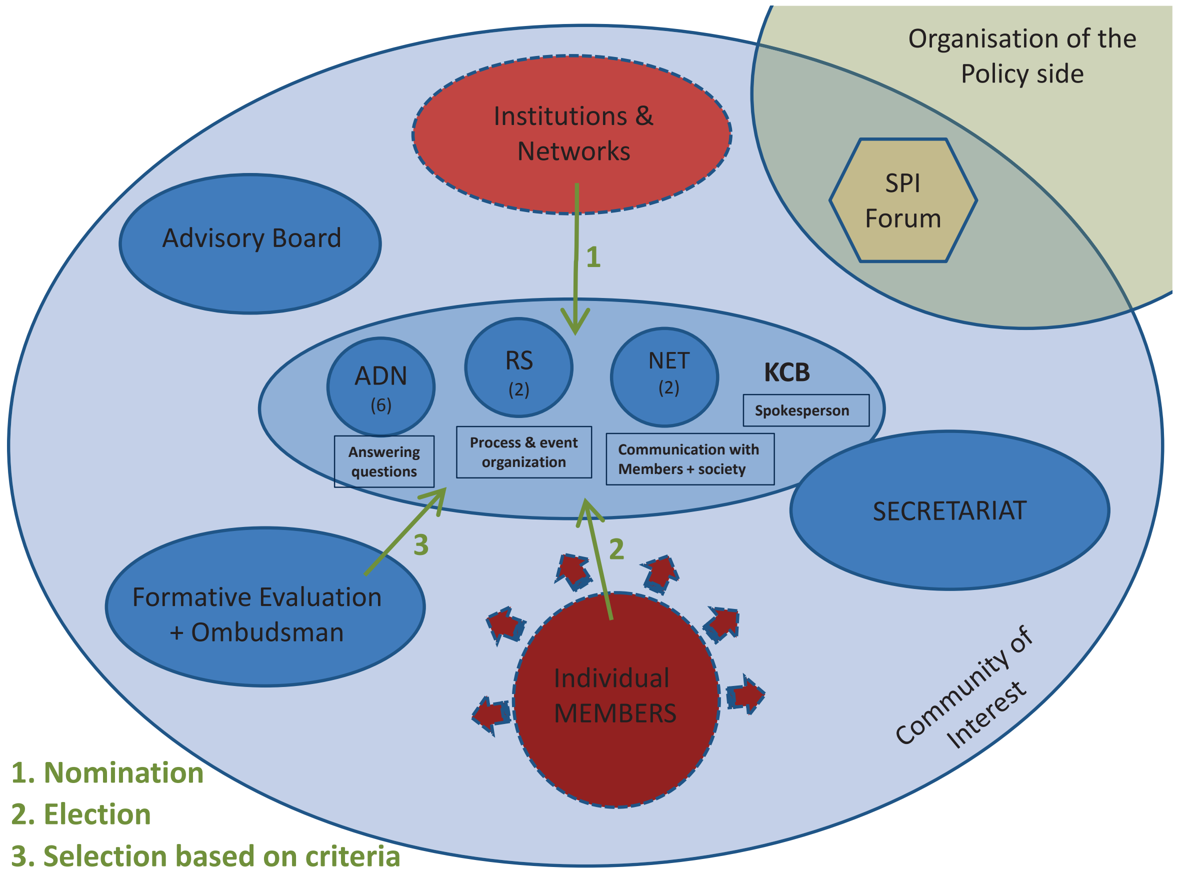

Figure 5.3.: *Illustration of the recommended NoK design, including roles for selection of members for the KCB*

Members can be individual experts in the broad fields of biodiversity, ecosystem services and natural resource management, and any field that might prove relevant for the topics tackled in the NoK, including methodologies for synthesis. As outlined in section 5.3, experts will be able to register via a simple online procedure and need to announce their willingness to contribute to the NoKs work in its different functions. They will be able to elect the potential members of the KCB, once the NoK is properly established (see Roadmap, chapter 5.8) and become engaged in working groups.

Institutions and Networks would get involved via their individual strengths and interests, thus ensuring targeted contributions according to their main areas of expertise (as outlined in the different roles in chapter 5.3), thus also committing themselves to support the NoK for the whole community of interest. They will be allowed (as all members) to nominate experts for the KCB, bringing in explicitly (and transparently) their expertise for the different functions served by the NoK. In the early phase of setting up the NoK (see 5.8), they could actively support other bodies of it, e.g. by supporting the secretariat by in-kind management capacity, but also by helping to identify preliminary actors for the Advisory Board and the Evaluation body.

Table 5.8: *Overview of key building blocks to be used in the recommended NoK design (highlighted in red). If two or more boxes in a row are highlighted, they showcase potential sub-options for the model.*

Categories	Potential Building blocks				
**Policy-link**	No Policy Link Body	Dialogue restricted with policy requesters	Policy-people involved in the NoK process (either part of KCB or Steering committee, etc.)	Policy link body (SPI forum)	
**Membership**	No membership	Only Individual-based membership	Mix of Institutions/ networks/and individuals memberships	Only institutions/ networks based membership	
**High level advice**	No advisory board	Non-permanent advisory board (ad-hoc group)	Advisory board composed of knowledge holders	Advisory board with representation of broader society	
**Steering**	No steering committee	Non-permanent steering committee	Small permanent steering committee	Big permanent steering committee	
**Secretariat**	No secretariat	Small secretariat incorporated in the KCB	Medium size secretariat	Big secretariat including IT, administrative and communication persons	
**Knowledge Coordination**	No Knowledge Coordination Body (KCB)	Ad-hoc Knowledge Coordination	Decentralized Knowledge Coordination Body, part time staff	Centralized KCB, full time staff	
**Research Strategy**	No Research Strategy Body	Research Strategy Body (RSB) independent from NoK but strong link	RSB included in KCB (Research prioritization as requests to the NoK)	Separate RSB body within the NoK	
**Evaluation**	only internal evaluation	Non-permanent evaluation group (ad-hoc group)	Formative evaluation body (mix between evaluation and advisory board)	External evaluation	
**Financing (see also chapter 5.7)**	No funding	A bottom-up approach using crowd-funding	Project funding	A bottom-up approach mainly driven by members/knowledge holder institutions	Complete funding of activities by one major donor or fund

A strength of this design is that although there are only a few bodies, a good division of labour is possible and a complementary set of people can speak on behalf of the NoK in different situations and put "faces" to the Network. These include the coordinator of the secretariat for receiving requests and easy interaction with policy, the spokesperson of the KCB, particularly on processes and outcomes from the ongoing work on requests, research strategy and building the network. In addition both the chair of the advisory board and ombudsman would be able to provide an informed outside perspective, especially in case of controversies. Co-ordination among them will of course be necessary.

5.6.3 Links to decision-making

An effective link of the NoK to decision-making, and especially to EU and Member State level policy is crucial to ensure the relevance and legitimacy of the NoK. With the recommended design, this link is not explicitly defined, as the way in which this link is organised is the responsibility of policy. The following

elements will need to be clarified in order to ensure a proper balance between policy and science in the process, which also ensures the engagement of all relevant stakeholders and knowledge forms:

- Regular exchange between main policy actors and the NoK: this could be achieved by regular meetings
  - on the working level between the NoK secretariat and assigned contact points in policy institutions (e.g., in relevant DGs and the EEA)
  - direct interactions with scientific advisors of DGs and Bureau European Policy Advisors (BEPA)
  - participation of ex-officio policy delegates in KCB meetings
  - active roles of policy makers and other stakeholders in the Advisory Board
- Ensuring a broad input into scoping processes of the ADN-function from decision-makers beyond the first requester on a task (e.g., different DGs, organisations of land managers, NGOs, etc.), e.g. through
  - The link of a NoK to a broader Stakeholder / Policy Forum of an EU mechanism
  - Joint open calls for interests from policy and the NoK to ensure awareness and participation in a topic
- Involvement of Member State representatives in strategic discussions, especially on the Research Strategy. While the NoK will ensure a high level of specific expertise in its processes on research strategy, it will be important to ensure representation of Member States (funding agencies, ministries and others) in such processes.

5.7 Finances

5.7.1 Possible models of financing

As in every science-policy interface, a cross-institutional and cross-thematic process like a NoK as the knowledge assessment part of the SPI is a major challenge in terms of finances. Looking at existing SPIs, at least four models of operation and financing could be identified:

A. **Complete funding of activities by one major donor** (e.g. a governmental body): In such cases, the SPI is most often linked, also in terms of legitimacy, to the funding institution (e.g. as advisory boards/bodies). For example, DG Environment supports several boards on different policies (e.g., on Biodiversity and Nature), or DG RTD support the Standing Committee on Agricultural Research (SCAR)[62]. There is currently no clear model on the European level that such a funding model could follow.

B. **A core funding via a fund/new institution** which is supported by one or several parties (governmental or others), and additional financing by requesters (or via crowdfunding, see E) to conduct work on their request: This option is common at the global level (e.g., via charity foundations or international NGOs), but has not been common practice in Europe. It would assume that some countries or ministries (and/ or DGs) would agree on a memorandum of understanding with according rules and guidelines on setting up such a fund, as

[62] See http://ec.europa.eu/research/agriculture/scar/index_en.html

it has just been set up for IPBES. Alternatively, or as add-on, a new institution could be founded that is supported by the fund or directly by the different donors.

C. **Time-restricted project-based funding**: Over the last decade, particularly in FP 6 and 7 several research networks and similar projects have been funded not only to improve scientific coordination, but also to better link up for the exchange with policy and society. For example, the FP funded Networks of Excellence such as ALTER-Net, MARBEF (now integrated in EuroMarine) and EDIT have developed means also to engage with society. A similar approach is followed by the ESFRI project LifeWatch. Until now, these networking activities, although gaining impact in improving communication to policy, have not been able to ensure a critical mass of joint funding beyond the initial lifetime to support the development of high-profile science-policy interface work. Project funding is restricted in time, even if some networks continue their work afterwards[63]. A similar lesson can be drawn from the European Platform for Biodiversity Research Strategy (EPBRS). With funding via two EU projects (BIOPLATFORM 2002-2004 and BIOSTRAT 2006-2009), a broad involvement of participants across Europe was achieved. Without such support, participation in the EPBRS processes has been more restricted to institutions and partners able to fund themselves. Nonetheless, project funding for specific activities of the NoK could particularly well support its overall performance. For example, the challenges identified for EU-funded projects to ensure an effective science-policy-interface[64] could be tackled in part by using the NoK to support them. The NoK could facilitate effective networking with policy makers and stakeholders and make it easier for projects to provide expertise for requests from policy on the project's topics. Accordingly, projects could use the NoK as a specialised partner for SPI tasks[65].

D. **A bottom-up funding by knowledge-holder institutions**: Some examples given under C tried to maintain core activities via a joint network format, especially ALTER-Net and MARBEF, including a core funding by the member institutes. Such an approach for the NoK would need a number of dedicated national institutions or regional networks (e.g., the mentioned ALTER-Net, MARBEF and others) to provide a core funding for NoK activities. Additional support could come via in-kind-contributions of person-months and logistical support by the institutes, but over the last years, even such support has not increased, but rather decreased in many networks due to the financial situations and pressures to apply for more third party funding, so that such a purely bottom-up approach may be risky in terms of continuity and resources.

The most realistic model for setting up a NoK and making it operational in the short term would be to fund the costs of its basic operation. The costs directly attributable to specific requests could be

[63] results of the SPIRAL project, see SPIRAL-briefs on EU projects and SPIs: http://www.spiral-project.eu/sites/default/files/16_recomm_2research%20projects.pdf

[64] See results and recommendations of the according SPIRAL workshop:
for funders: http://www.spiral-project.eu/sites/default/files/17_recomm_2fundinginstitutions.pdf;
for projects: http://www.spiral-project.eu/sites/default/files/16_recomm_2research%20projects.pdf;
for policy makers: http://www.spiral-project.eu/sites/default/files/15_recommendations_policy-makers.pdf

[65] A similar model has been developed over the last 20 years with for the marine sector with the founding and establishment of AquaTT, a non-for-profit SME that provides services for EU project on disseminations, knowledge transfer and stakeholder involvement, see http://aquatt.ie/

charged. This would also help maintain independence from funders. Requesters, who are not able to pay for their requests could be supported via add-on funds or via bottom-up approaches.

E. **A bottom-up approach using crowdfunding**: As an add-on option for open and transparent funding processes, the option of crowdfunding, a funding approach based on the ideas of a collective effort of individuals who network and pool their money to support specific efforts of people or organisations, like for example a network of knowledge answering a specific request. Today, crowdfunding, mainly driven by platforms on the internet is used to finance start-ups, software developments, creative activities such as movies and music production but also scientific projects. Crowdfunding without an explicit revenue to the funders (such as for example in many start-ups), rely heavily on the reputation of the endeavour, its transparency and the trust in the organization and activities being funded – all attributes of major importance in a NoK. Accordingly, once the NoK is established, a crowdfunding approach could be used to fund specific requests for the ADN-function that are then presented and discussed via the NoK community and proposed for a crowdfunding. Like in other crowdfunding approaches, a request would only be tackled if the required threshold for funding is reached. Crowdfunding would only be used for concrete activities, and not for the basic funding ensuring the operation of the NoK.

Given that the proposed NoK is clearly defined in its roles and includes in the first place tasks of European wide relevance, combinations of all options would be possible. A proposal for funding needs to be developed jointly between science and policy. Option E could become an additional source of funding once a NoK is established and perceived as credibly conducting knowledge assessments. The following section gives a rough indicative budget and shortly discusses the possibility of in-kind contributions of scientific institutions which could support a combination of options A or B with C.

5.7.2 Estimation of financial needs of the recommended NoK design

The NoK design as outlined in section 5.6 will require substantial funding from different sources such as the ones described above to become operational and provide the added value expected. As the recommended NoK design is a medium approach between the Network and the Platform model, it is also reasonably flexible and modular in its funding scheme, giving possibilities to set up bodies and functions step by step and have them supported by different donors or institutions, depending on their specific interests.

As central element, the **secretariat** will play a crucial role in keeping the processes of the NoK running and following the principles and guidelines developed for its processes. Accordingly, the secretariat will need to be equipped with several knowledge broker professionals (3-5 persons, plus assistance), which should be situated in one central location, ideally in Brussels, to ensure direct contact with key knowledge requesters. In addition, the secretariat should include explicit communications expertise by (at least) one dedicated communications expert, being able to design and adapt products and communications of the NoK for different audiences.

The meetings of the bodies, and the potential work in between by its members, especially the KCB, are generally expected to be delivered in-kind regarding the work time. Only travel costs for the **KCB**, the **Advisory Board** and the **formative evaluation body** should be funded by the NoK. For the evaluation, it might be reasonable to co-fund (potentially via third-party funding) a part time position to conduct interviews and analyses for the evaluation.

The different functions of the NoK will also need funding and the funding sources for them could vary.

For the NET-function, setting up a web-platform will be crucial and should be developed either jointly with other projects with a more narrow focus (e.g., the current development of a common platform for OpenNESS and OPERAs), or via a specific project for developing such interaction platform, e.g. via DG Connect. Capacity building is a crucial element for the overall success of the NoK, and needs to be included, for example capacity building workshops, which could be held in the context of other events. Also, specific web-learning products could be developed, e.g. inspired by the handbook on SPIs provided by the SPIRAL-project[66].

For the ADN-function, funding will be needed to conduct the work of the scoping and working groups for assessment requests. Besides meetings of the working groups themselves, additional funding will be needed depending on the methods used: evidence based approaches (e.g. systematic reviews) may need support for librarians and review experts; collaborative adaptive management or expert consultation approaches will need support for additional meetings and communication, etc. (see Annex 5).

Funding of the ADN-activities may come from different sources:

- from individual funding by the requester, *if* the independence of the NoK operations is maintained and requesters only get involved via the set pathways of scoping and review (see chapter 3.3).
- from a core funding, which would enable the KCB to freely decide upon which requests to take up (options A and B in chapter 5.7.1)
- via the funding of research projects (e.g. in H2020), which offer their specific expertise in the field of their work in the context of the NoK for relevant policy requests (option C in chapter 5.7.1), and thus increase the expected policy impact of their projects
- via a crowdfunding approach once the NoK, including the NET-function and an active community of interest is established. Here, requests would be tabled in a crowdfunding platform including an estimate of funding to see monitor whether necessary support is achieved (option E in chapter 5.7.1)

For the RS-function, e-conferences and meetings (with potential follow-up e-consultations) should be foreseen as main activities, based on the experiences from EPBRS. Such activities could be funded either by the core funding, a specific funding by a sponsor or a requesters (e.g., by ERA-Nets and similar funding bodies), or, at least as co-funding, by countries taking responsibility of further developing the research agenda during their successive EU presidencies (as already established by EPBRS).

Based on these considerations, an indicative budget for an effective NoK would, depending on the level of activities envisaged per year and the costs especially for employed knowledge broker experts (depending on country and institution of employment) vary between € 500.000 per year (with a core staff of about 3 persons and requests being funded by requesters) and € 1.500.000 per year (with a core staff of 7, a core budget for requests and setting up a web-platform).

[66] See for example http://www.spiral-project.eu/content/documents#jump2briefs

5.8 Road map for NoK implementation: timeline, actors and stepwise approach

Establishing a Network of Knowledge as described in this document requires a new way of action and interaction from the current interface between science, society and policy. It requires, as described as one main message in the output of the SPIRAL-project a change of thought in how we perceive and act in science and policy, changing from the idea of an interface between two distinct “silos” (or more, if other actors are taken into account) towards alliances which enable on-going opportunities for and a process of exchange and learning.

Accordingly, setting up the NoK needs to be a stepwise approach, which acknowledges the different roles of different actors from the start and only gradually can evolve into a new model of interaction that fully serves the principles laid out in the beginning of this chapter.

For example, the idea of an active, individuals-based membership which actively responds to calls for engagement and ensures a broad representation of the knowledge holder community able to elect the KCB will only be achieved gradually in the first 3-5 years of a NoK. Until then, a number of dedicated institutions, networks and also project-driven partnerships will be needed to set up and act in a first KCB and an according Advisory Board, implementing the NoK principles, setting up the main elements for the main functions, and ensuring an open and transparent process towards a broad individual membership.

For this, a detailed business model will be developed jointly by key partners. This will establish the NoK in a stepwise approach outlined below. Table 5.9 gives a provisional timeline for this process after the end of the KNEU project.

Firstly, based on this paper, the general design of the NoK and its guiding principles should be agreed on by core partners for its implementation. Many proposals made in the paper already try to digest a baseline consensus from earlier discussions from the KNEU project, but of course details need to be worked out jointly.

Secondly, an interim KCB and interim Advisory Board should be set up by core partners to jointly further develop the business plan for the NoK, taking into account the political support at the EU level as well as the potential role of the NoK in supporting international processes.

This business plan would include, as a third and parallel step, working groups to further detail the set-up of the NET- , the ADN-, and the RS-function. For all three functions, it will be important to involve core partners to make the steps for establishment as relevant for them and as complementary as possible to ongoing activities and then agree a MoU between them, taking into account the different potential roles and contributions as outlined in chapter 5.3.

For the NET-function, this would include setting up pilot areas for the web platform to build and activate the community. Here, special emphasis will need to be put on the link to existing platforms such as BISE and to use existing initiatives like the one from OpenNESS/OPERAs effectively. Also, specific funding opportunities, e.g. via grants from DG CONNECT, will need to be explored[67].

For the ADN-function this will mean to further develop the rules for using the different methodological approaches, clarify the interaction with policy and other decision-makers (with the support of the interim Advisory Board). Jointly with policy potential first requests for thematic knowledge assessments to be carried out in a first pilot phase will be identified. Also, this work will include a scoping exercise on how

[67] See for example http://ec.europa.eu/dgs/connect/en/content/digital-science-and-ict-enabled-science-society-interaction

future Horizon 2020 projects in the area of biodiversity and ecosystem services can actively contribute with their activities on the SPI to implementing the function and thus also ensure their expected impact to society.

For the RS-function, it will be important to build on the work of EPBRS so far and further clarify the role of the NoK for serving potential requesters, especially from DG RTD and from ERA-Nets.

Special attention will need to be paid to collaboration with existing interface institutions, especially the European Environment Agency and the Joint Research Centre. On the international level, the potential link to ICSU & Future Earth (e.g., for the Research Strategy function), and to IPBES with respect to the international collaboration will need to be further explored. Good coordination with these institutions and groups will avoid duplication of work and ensure that the NoK only takes action when this is complementary to other ongoing activities.

Table 5.9: *Steps to be taken to further develop and implement the NoK*

Step		Timeline
**1**	Agreement on recommended design and its principles	End of KNEU project, April 2014
**2**	Set up of Interim KCB and Interim Advisory Board with core partners (and interim secretariat); joint development of "business plan" including in-kind contributions and financial needs	Constituting towards end of 2014<br>Business plan accepted beginning of 2015
**3a**	Implementation of NET-function: set up of joint (interim) working group, identify concrete needs/interests from BISE and relevant science networks; set up pilot areas of interaction and according e-infrastructure	Early 2015: main lines set<br>Mid 2015: first implementation activities started, e.g. using existing platform to be further developed, or project outline for funding developed
**3b**	Implementation of ADN-function: Further development of general protocol and how to engage experts; identification of first tasks to be addressed (with requesters), organise first scoping processes and working groups	Early 2015: main lines set<br>Mid-end 2015: first implementation actions started, e.g. specific request
**3c**	Implementation of RS-function: workplan and potential activities for 2015/2016 identified jointly with EPBRS and potential stakeholders; implementation of first activities in 2015	End 2014: first activities for 2015/16 outlined<br>Mid-end 2015: first implementation actions started, e.g. planning for e-conference and meeting in late 2015
**4**	Official start of NoK with a set of MoUs between different partners, set up of interim web platform and first activities in ADN- and RS-function; set up of interim formative Evaluation Body	End of 2015

A final note by the KNEU team

The NoK recommended design and its future implementation as laid out here are based on the work of the KNEU project over the last 3 years, incorporating different views, interests and expertise from several hundred knowledge holders with various backgrounds from science and beyond as well as from decision-makers. It attempts to find a balance between an ambitious and innovative transdisciplinary approach for a dialogue-driven science-policy-society interface, and the feasibility of such an approach in the current landscape of knowledge holders and users on biodiversity and ecosystem services. Although we think that the recommended design shows a clear and balanced way forward, major challenges remain for the implementation BiodiversityKnowledge. Implementation will rely on the willingness of key institutional actors to take an active role. This community should explicitly include actors from other knowledge fields, also relevant for biodiversity and ecosystem services, especially in the rapidly growing field of natural capital discussions and implementation activities or infrastructure development.

With this roadmap, the KNEU team believes that the pathway for substantially improving the evidence base, and enabling societal actors to make better informed decisions concerning biodiversity and ecosystem services is set. Europe plays a major role at the international level. It is the “old continent”, rich in history and experience, but not always taking care of its own biodiversity unfortunately. It also holds responsibility for the future of biodiversity in many overseas territories. Europe is an innovative laboratory building upon its broad diversity of culture and structures. BiodiversityKnowledge is an attempt to promote this model in organizing better knowledge flow and brokerage in order to contribute to the conservation of biodiversity, sustainable development and innovative decision-making to face the challenges of climate change, ecosystem services and well-being, in Europe and elsewhere.

ANNEXES

Annex 1: Acronyms used

Acronym	Name
ADN-function	Answering-Decision-making-Needs function. One of the four potential functions at the science-policy interface in Europe and identified by the work of KNEU
ALARM	Assessing Large Scale Risks for biodiversity with tested Methods (www.alarmproject.net/alarm)
ALTER-NET	Europe's biodiversity research network – A Long-Term Biodiversity, Ecosystem and Awareness Research Network, Network of Excellence FP6 (www.alter-net.info)
BEPA	Bureau of European Policy Advisers (ec.europa.eu/bepa)
BISE	Biodiversity Information System Europe (biodiversity.europa.eu)
BiodivERsA	Network of 21 research-funding agencies across 15 European countries. It is a second-generation ERA-Net, funded under the EU's 7th Framework Programme for Research. (www.biodiversa.org)
BIOFRESH	Biodiversity of Freshwater Ecosystems: Status, Trends, Pressures, and Conservation Priorities (www.freshwaterbiodiversity.eu)
Bioplatform	European platform for biodiversity (www.edinburgh.ceh.ac.uk/projectpages/bioplatform_page.htm)
Biostrat	Developing EU Biodiversity Research Strategy (www.biostrat.org)
CAP	Common Agricultural Policy
CBD	Convention on Biological Diversity
CEE	Collaboration for. Environmental Evidence (www.environmentalevidence.org)
CEH	Centre for Ecology & Hydrology (www.ceh.ac.uk)
CESAB	Center for Synthesis and Analysis of Biodiversity (cesab.org)
CGBN	Coordination Group for Biodiversity and Nature Conservation (Replacing the BEG, Habitats scientific working group and the ORNIS scientific working group)
CIF	Common Implementation Framework (of the EU 2020 Biodiversity Strategy)
CRELE	Credibility, RElevance and Legitimacy. Attributes suggested to evaluate the effective-ness of an SPI
CSA	Chief Scientific Adviser (to the president of the European Commission)
DG	Directorate-general of the European Commission; e.g. DG Environment, DG RTD (Re-search and Innovation),DG Connect, etc.
EASAC	European Academies Science Advisory Councils
EBONE	European Biodiversity Observation Network
EC	European Commission (ec.europa.eu)
ECNC	European Centre for Nature Conservation (www.ecnc.org)
EDIT	European Distributed Institute of Taxonomy (www.e-taxonomy.eu)
EEA	European Environment Agency (eea.europa.eu)
EEB	European Environment Bureau
EEF	European Ecological Federation
EHF	The European Habitats Forum
EIONET	European Environment Information and Observation Network

ENCA	Heads of European Nature Conservation Agencies (www.encanetwork.eu)
EPBRS	European Platform for Biodiversity Research Strategy (www.epbrs.org)
EPRG	Environment Policy Review Group (Commission Expert Group)
ERANet	European Research Area Network (ec.europa.eu/research/era/index_en.htm)
ESFRI	The European Strategy Forum on Research Infrastructures (ec.europa.eu/research/infrastructures/index_en.cfm?pg=esfri)
ESP	The Ecosystem Services Partnership (www.es-partnership.org)
ETC/BD	European Topic Centre on Biological Diversity (bd.eionet.europa.eu)
EUBON	Building the European Biodiversity Observation Network (www.eubon.eu)
EuMon	EU-wide monitoring methods and systems of surveillance for species and habitats of Community interest (eumon.ckff.si/index1.php)
EUNIS	European Nature Information System (eunis.eea.europa.eu)
EuroMarine	Integration of European Marine Research Networks of Excellence (MarBEF, Eur-Oceans, Marine Genomics Europe), FP7 project (www.euromarineconsortium.eu)
FACCE	Joint Programming Initiative on Agriculture, Food Security and Climate Change (FAC-CE-JPI) (www.faccejpi.com)
FP7	Seventh Framework Programme of the European Union for the funding of research and technological development in Europe
FRB	Fondation pour la Recherche sur la Biodiversité (www.fondationbiodiversite.fr)
Future Earth	10-year international research initiative to develop the knowledge for responding effectively to the risks and opportunities of global environmental change and for supporting transformation towards global sustainability (www.futureearth.info)
GBIF	Global Biodiversity Information Facility (www.gbif.de)
GEOBON	Group on Earth Observations Biodiversity Observation Network (www.earthobservations.org/geobon.shtml)
H2020	Horizon 2020. The EU Framework Programme for Research and Innovation (ec.europa.eu/programmes/horizon2020)
IALE-Europe	The European chapter of the International Association for Landscape Ecology (www.iale-europe.eu)
IC-function	International Collaboration function. One of the four potential functions at the science-policy interface in Europe and identified by the work of KNEU
ICSU	International Council for Science (www.icsu.org)
INNGE	International Network of Next Generation Ecologists (www.innge.net)
INSPIRE	The INSPIRE directive of the European Commission aims to create a European Union (EU) spatial data infrastructure (inspire.jrc.ec.europa.eu)
IPBES	Intergovernmental science-policy Platform on Biodiversity and Ecosystem Services (www.IPBES.net)
IPCC	Intergovernmental Panel on Climate Change (www.ipcc.ch)
IUCN	International Union for Conservation of Nature (www.iucn.org)
JRC	Joint Research Centre (ec.europa.eu/dgs/jrc)
KNEU	Developing a Knowledge Network for EUropean expertise on biodiversity and ecosystem services to inform policy making economic sectors. EU FP7 project funded as co-ordination action (2010-2014 – Grant No.265299). KNEU consortium wrote the present document. (www.biodiversityknowledge.eu)
KCB	Knowledge Coordinating Body. Actor/building block of the NoK

LifeWatch	European e-Science infrastructure for biodiversity and ecosystem research (www.lifewatch.eu)
LTER-Europe	European Long-Term Ecosystem Research Network (www.lter-europe.net)
MA	Millennium Ecosystem Assessment (www.maweb.org)
MAES	Mapping and Assessment of Ecosystem Services (Target 2, action 5 of the EU 2020 Biodiversity strategy)
MARBEF	Marine Biodiversity and Ecosystem functioning Network of Excellence, FP6 (www.marbef.org)
MARS	European Network of Marine Research Institutes and Stations (www.marsnetwork.org)
NeFo	Netzwerk-Forum zur Biodiversitätsforschung. German science-policy interface for biodiversity research (www.biodiversity.de)
NGO	Non-governmental organization
NERC	UK's main agency for funding and managing research, training and knowledge exchange in the environmental sciences (www.nerc.ac.uk)
NET-function	Networking and capacity building function. One of the four potential functions at the science-policy interface in Europe and identified by the work of KNEU
NoK	Network of Knowledge
OpenNESS	Operationalisation of natural capital and ecosystem services (www.openness-project.eu)
OPERAs	Operational Potential of Ecosystem Research Applications (www.operas-project.eu)
PEER	Partnership on European Environmental Research (www.peer.eu)
PESI	Pan-European Species directories Infrastructure (www.eu-nomen.eu)
PR	Public Relations
RS-function	Research Strategy function. One of the four potential functions at the science-policy interface in Europe and identified by the work of KNEU
SCALES	Securing the Conservation of biodiversity across Administrative Levels and spatial, temporal and Ecological Scales (www.scales-project.net/)
SCAR	Standing Committee on Agricultural Research
SCB	Society for Conservation Biology (www.conservationbiology.org)
SEBI2010	Streamlining European 2010 Biodiversity Indicators (biodiversity.europa.eu/topics/sebi-indicators)
SPI	Science-Policy Interface
SPIRAL	Science-Policy Interfaces for Biodiversity: Research, Action, and Learning (www.spiral-project.eu)
TEEB	The Economics of Ecosystems and Biodiversity (www.teebweb.org/)
TEK	Traditional Ecological Knowledge
TESS	Transactional Environmental Support System (www.tess-project.eu)
UFZ	Helmholtz Centre for Environmental Research (www.ufz.de)
WPIEI	Working Party on International Environmental Issue
WWF	WorldWide Fund for Nature (www.wwf.org)

Annex 2: Glossary

Community of Interest: A (virtual) gathering of people assembled around a topic of common interest. Its members take part in the community to exchange information, to obtain answers to (personal) questions or problems, to improve their understanding of a subject, to share common passions. [Definition based on Wikipedia entry]. On a topic like biodiversity and ecosystem services, the community will include a broad diversity of potential stakeholders, which we call "the NoK community".

Credibility: The perceived quality, validity and scientific adequacy of the people, processes and knowledge exchanged at the interface. Part of the CRELE concept on evaluating SPIs, see Box 5.

Data: Known facts or measurements collected, which are often stored or exchanged in a digital form. Biological data are commonly stored in files or databases (adapted from Wikipedia)

Decision making: In the context of BiodiversityKnowledge, decision making is understood broadly: not only the political decision making on new laws and regulations, but also the decision making in the context of managing biodiversity and natural resources, including the decisions taken in the private sector.

Formative Evaluation: Evaluation that is used to modify or improve products, programs, or activities and is based on feedback obtained during their planning and development. A periodically repeated assessment of efforts prior to their completion for the purpose of improving the efforts.

Knowledge: Biodiversity management and policy require information gathered from a wide range of knowledge types. This includes scientific knowledge in the strict sense (mainly backed by peer-reviewed literature), but also knowledge from practical (management) experience and other evidence-based sources.

Knowledge holder: People and institutions (see also knowledge hubs) that possess relevant knowledge in various areas of expertise, including scientists from different fields, practitioners in biodiversity management, administrative bodies, companies, NGOs and indigenous and local people.

Knowledge hub: a collective term we use in the NoK context for any organisation, network or project that has an internal structure enabling it to address its members and identify potential experts for a topic raised by the NoK.

Knowledge requester (also called clients in earlier phase of KNEU): People and institutions responsible for the management and policy strategies on biodiversity and ecosystem services which approach the network with questions related to their responsibility. These clients include political institutions like DGs, ministries, European and national agencies as well as European or international conventions. They may also include the private sector and its umbrella organisations and NGOs in the field of biodiversity and ecosystem services.

Legitimacy: Legitimacy includes the perceived transparency and the balance of perspectives within SPI processes. Part of the CRELE concept on evaluating SPIs, see Box 5.

Regional: Depending on the level of discussion, "regional" might refer to different scales: In the context of global UN-related activities, "regional" addresses the scale of continents or biomes, thus "national" being below this level. In the EU context, "regions" refer to the sub-national level, which we normally use in the context of this paper.

Relevance: The salience and responsiveness of the SPI to policy and societal needs. Part of the CRELE concept on evaluating SPIs, see Box 5.

Stakeholders: In the context of a NoK stakeholders includes >knowledge holders and >knowledge requesters.

Traditional Ecological Knowledge (TEK): Defined as a body of knowledge and beliefs about the relations of specific human societies to the local environments in which they live, as well as their local practices for ecosystem use and stewardship (adapted from Hernández-Morcillo et al. 2014).

Annex 3: Detailed outline of the prototype to answer decision making needs

This annex can be found at http://www.biodiversityknowledge.eu/

Annex 4: Elements and criteria for initial request form for the NoK

I) Requester

- Institution requesting
- Contact person

II) Request

- What is your request?
- What is the political background and need for the request?
- What is the timeframe an answer needs to be provided in?

III) Key elements and details

- Species, habitats, ecosystem service or other resources concerned
- Geographical scope (European – regional – national – local)
- Timeframe addressed (e.g., one-off management activities, short- to long term effects, forward looking exercises)
- Sectors of actions, management and interventions concerned (e.g., agricultural activities, forest management, mobility, health…)
- Policy fields and instruments concerned (e.g., sectoral policies, specific directives)

IV) Background information

- Resources available to conduct assessment
- Type of product/reporting preferred
- Potential knowledge holders/ stakeholders to get involved in scoping and/or conduction
- Additional sources of information and existing knowledge (as attachments)

Annex 5: Matrix of potential types of requests posed to the NoK and suitable methods to address them

	Expert consultation framework			Evidence Based framework			Collaborative Adaptive management framework *		
	Focus group	Delphi process	Expert review	Systematic map	Systematic review	Rapid evidence review	Governance & Institutional Assessment (1)	Participatory Decision Making(2)	Adaptive Planning(3)
**Seeking greater understanding or predictive power**	√	√	√	√	√√ (strong base of published evidence)	√	√	√√ (conflicting views or interests exist)	√; in combination with other methods)
**Scenario building**	√	?	√; in combination with other methods	√; in combination with other methods	√; in combination with other methods	√; in combination with other methods	?	√√ (CL and PSM, supported by IT models)	?
**Horizon scanning**	√√	√√	√; in combination with other methods	√; in combination with other methods	X	X	?	√; in combination with other methods	√√ (addressing uncertainty & knowledge gaps)
**Seeking measures of anthropogenic impact**	√	√	√	√	√√ (strong base of published evidence)	√	√; in combination with other methods	√√ (conflicting views or interests exist)	√; in combination with other methods

Seeking measures of effectiveness of interventions	√	√	√	√	√√ (strong base of published evidence)	√	√	√	√√ (decision making constrained by complexity, uncertainty or knowledge gaps)
Seeking appropriate methodologies	√√	√√	√√	√	√	√	?	√	√√ (decision making constrained by complexity, uncertainty or knowledge gaps)
Seeking optimal management	√	√	√	√	√√ (strong base of published evidence)	√	√√	√√ (conflicting views or interests exist)	√√ (decision making constrained by complexity, uncertainty or knowledge gaps)
Public opinion/ perception	√	√	√	√	√	√	√	√	X
Distribution of species /disease	√; specific location	X	√√	√	√; suitable for establishing the abundance\ densities of an organism in ecosystems	√	?	√√ (CL and PSM, supported by IT models)	√√ (decision making constrained by complexity, uncertainty or knowledge gaps)
Definitions	√√	√	√	X	X	X	X	√	X

	Expert consultation framework			Evidence Based framework			Collaborative Adaptive management framework *		
	Focus group	Delphi process	Expert review	Systematic map	Systematic review	Rapid evidence review	Governance & Institutional Assessment [(1)]	Participatory Decision Making[(2)]	Adaptive Planning[(3)]
**What is a desirable state for….?**	√	√	√	X	X	X	√	**√√ (conflicting views or interests exist)**	X
**Socio-economic impacts**	√	√	√	√	√	√	√	**√√ (modeling socio-natural systems)**	**√√ (decision making constrained by complexity, uncertainty or knowledge gaps)**

Legend of Annex 5:

Most suitable methods   Not first choice method but can be used   Non suitable methods

* Methods under this heading are complementary, rather than alternative.

(1) Including, but not restricted to, a selection of methods such as: governance assessment, institutional analysis, discourse analysis, policy analysis.

(2) Including, but not restricted to, a selection of methods such as: collaborative learning, problem and solution trees, back-casting, participatory scenario building, (fuzzy) cognitive mapping, conceptual modelling, Bayesian belief networks, and double-sided critic.

(3) Including, but not restricted to, a selection of methods such as: collaborative learning, structured decision making, adaptive management, adaptive governance, transition management.

Further details on method choice can be found on www.biodiversityknowledge.eu

Annex 6: Elements for expert selection criteria

This annex can be found at www.biodiversityknowledge.eu

Annex 7: List of potential contributions and benefits of institutions, as outlined at the 2nd BiodiversityKnowledge conference

Potential contributions and benefits as suggested by key networks and other participants invited at the 2nd BiodiversityKnowledge conference. The table outlines *potential* roles, so it shouldn't be understood as a sort of commitments by the listed partners. The table will also be subject for continued revision and discussions with additional knowledge hubs.

This annex can be found at www.biodiversityknowledge.eu

Annex 8: Comparison table of use of building blocks in different models and recommended NoK design

Building blocks	Network model	Platform model	NoK Recommended design
**ASSEMBLY**	Not envisaged	• Composed of key institutions, knowledge hubs, networks…<br>• maybe 30-50 full members<br>• Assembly might meet twice a year (meeting of the members).<br>• Meetings could be linked to other conferences	Possible (following approach in platform model), but not required
**ADVISORY BOARD**	Not envisaged	Not envisaged	• Ambassadors<br>• Roles would be to provide general advice (e.g. possible pro-active research strategy topics),<br>• Role in making the process and the outputs of the NoK known<br>• Could have some people from the institutes, and some from the wider community (e.g. business, NGOs etc).
**STEERING COMMITTEE**	Fulfilled by KCB	• This body needs to have representativeness of important people = visibility.<br>• Its role would be to provide guidelines, and design the strategy of the platform.<br>• This body could have a chair person, responsible to talk to the media about the scientific outcomes and if someone contest outcomes and/or processes	Fulfilled by KCB
**SECRETARIAT**	Function included in the Knowledge Coordinating Body	• 3-4 people (including a communication specialist) in a centralised (Brussels) and full-time capacity.<br>• Communication, especially active role in raising awareness of the platform, not only in the EU commission but also in the EU parliament and with other stakeholders<br>• Receiving and collecting requests<br>• Support of KCB and working groups with processes of the work<br>• Appointments of staff by Steering Committee	• Overall day-to-day support and filtering of requests<br>• 3-4 people (including a communication specialist) in a centralised (Brussels) and full-time capacity.<br>• Overall coordination of the KCB; administrative function; receives all requests: deals with small requests (in/out; reformulation, contact with experts and putting requests on website), give larger requests to KCB;<br>• Communication to members/society (latter role shared with KCB).<br>• Secretariat will need to include an overall coordinator,<br>• Appointments of staff by KCB jointly with chairs of Advisory Board and Evaluation Board

Building blocks	Network model	Platform model	NoK Recommended design
**KNOWLEDGE COORDINATION BODY**	• Main permanent body<br>• Orchestrates the whole process.<br>○ Responsible for keeping track of all processes and products and documenting them in a transparent manner ("database" of the NoK).<br>○ Sets up working groups on specific requests, and engage working groups on any issue of relevance and interest such as communications, methods, capacity building and so on….<br>• composed of 7-10 people; some full-time and long-term positions, able to manage the process and including technical support people, with a secretary, accountant, IT person + 3-year-term members that would work maybe 6h per week.	• Body which will organize the whole process of provision of advice/giving answers.<br>• Composed of 5 to 10 people.<br>• They should represent a range and variety of disciplines.<br>• Part-time roles, in-kind-contributions based on nominations by institutional members	• Roles relating to networking function, research strategy function, ADN function.<br>• Main decision-making body comprising<br>• 1 spokesperson responsible for communication with the outside, especially in case of contested issues<br>• 2 persons on networking function, one of them as main contact point for institutions<br>• 2 people on RS function with virtual meeting organisation and conference organisation roles;<br>• up to 6 people in the ADN function with clear roles and responsibilities and is representative – this should be the case for all members of the KCB.<br>• Part-time, decentralised in kind contribution basis.<br>• In the first year 6-10 meetings.<br>• KCB selection: nomination through in-kind contribution by the institutes; election by the members; selection based on long-list from members by the formative evaluation body. KCB: 3 year terms, one re-election possible. Continuity needed so that not all drop out at the same time.<br>• Ad hoc meetings between the KCB and other people for relevant questions etc, also possible to invite people to specific KCB meetings.<br>• Who manages the KCB? Role for Secretariat who needs to ensure the KCB is working/managed.
**RESEARCH STRATEGY BODY**	• No specific body<br>• Requests for research prioritization are treated like other requests.<br>• Advantage of true inclusiveness, true independence, compared to old EPBRS.	• Selfstanding body ("working group") which will look at the gaps in research<br>• Request driven, but also taken a more strategic knowledge-driven role ("horizon scanning")	• RS function embedded in KCB work, thus linked to other functions<br>• more stable than EPBRS due to support from the Secretariat, and more flexibility in terms of both responding to requests (and jointly formulating requests) and proactive in terms of developing potential issues (e.g, based on advice from advisory board)<br>• Role to identify appropriate experts for a topic, engagement of policy (e.g., form member states) would need to be ensured via policy activities

Building blocks	Network model	Platform model	NoK Recommended design
**EXTERNAL EVALUATION BODY**	• Permanent body accompanying the process in terms of formative evaluation, giving reflections and an outsider view.<br>• Composed of external evaluators<br>• Additional mechanism to get feedback from the entire community, where anyone could comment and provide inputs.<br>• Interim audit of the process after 2 years, and then after 5 years with a diversity of people coming from different institutions (Science, policy, etc.)	• Mix between internal reviewers and external reviewers<br>• The review should focus on how the secretariat, the steering committee and KCB work together and process requests<br>• External reviewers maybe from outside Europe. Probably people out of IPBES.context	• Responsible for selecting the members of the KCB based on criteria of expertise and balance between needs, expertise etc.<br>• Formative evaluation body (including ombudsman), meaning an active role in co-developing the NoK<br>• Members should be experts and practitioners on SPI and other interface activities
**POLICY COORDINATING BODY**	• Body responsible of organizing the policy side (main requester to the NoK).<br>• This will be composed of representatives from DGs, EEA, JRC, etc…<br>• They meet regularly with the KCB (every 3 months)<br>• They identify forthcoming issues, forthcoming policy agenda and formulate preliminary requests, discuss NoK outputs and ensure they are distributed among policy and stakeholders	• Science Policy Forum; a broad forum engaging policy representatives from EU and MS level<br>• Possible: also involvement/ link to relevant stakeholder groups<br>• Identifies potential request issues for the NoK, supports the according scoping processes	• Link to Member states to be defined<br>• Link with a possible Science Policy Forum (or another format for link to policy) to be defined
**MEMBERS**	• Only individual membership from persons<br>• Links to all relevant institutions with individual MoU, in order to limit problem of influence from institutions, no problem of hierarchy…<br>• Members can become the experts working in the scoping, working groups and evaluation.<br>• Institutions only as requesters not members. They will have MoU as requesters.	• Institutional/ networks membership in the assembly (See ASSEMBLY)	• Self-declared individual membership (e.g. short questionnaire on what people can contribute in terms of knowledge or expertise, not money contribution).<br>• Institutional membership via individual MoUs or LoIs highlighting roles and contributions of specific insitutions

Building blocks	Network model	Platform model	NoK Recommended design
**FUNDING**	• The main idea is that all positions are funded through in-kind contributions from institutions.<br>• Ground funding, at least for some part:<br>○ E.g. Commission could fund permanent positions in the KCB.<br>○ Possible funding from ICSU, Future Earth for core funding (not further assessed).	• Funding will be (partly) through the membership of insitutions (fee most probably adapted to the institution) and policy would need to put money for the basic like the secretariat and on organizing meetings<br>• a formalized funding might mean that there is more pressure that the results are taken up by policy.<br>• In-kind contribution could play an important role.<br>• Membership should pay fee for travelling cost, organizing workshop…plus 1-2 persons in the secretariat.	• Need a diversity of funding sources.<br>• Need a combination of time-bound projects and permanent institutional funding (to maintain procedural knowledge and networking)<br>• Long-term perspective: use of crowdfunding-approach for requests
**SELECTION OF WORK FORCES**	**KCB**:<br>• KCB people would be nominated by their institutions to work within the KCB.<br>• Selection of the KCB people: combination of voting by all members of the network and then the external evaluation body will choose from this list of nomination, to ensure diversity, representativeness... Nomination will come with a letter from institution as support of the NoK.<br><br>**EEB**<br>• Selection?<br><br>**PCB**<br>• Selection?	**EXPERTS for ADN**:<br>• Strong procedures for expert selection to mitigate against dominance of some members (as an incentive for being a members might be to ensure that its research is influencing in the answering of the question). Strong rules will contribute to transparency of the expert selection.	• Need clear criteria for expert selection by the KCB<br>• See illustration for selection of KCB people

Annex 9: Partners of the KNEU project

Project Partners	Country
Helmholtz-Zentrum für Umweltforschung, UFZ (coordination)	Germany
Natural Environment Research Council – Centre for Ecology and Hydrology, CEH	U.K.
Institut Royal des Sciences Naturelles de Belgique, RBINS	Belgium
Centro Interdisciplinar de Investigação Marinha e Ambiental, CIIMAR	Portugal
Stichting Koninklijk Nederlands Instituut voor Zeeonderzoek, NIOZ	Netherlands
Foundations Francaise pour la Recherche sur la Biodiversité, FRB	France
Stichting Dienst Landbouwkundig Onderzoek, ALTERRA	Netherlands
Universität Wien	Austria
Stiftelsen Norsk Institutt for Naturforskning, NINA	Norway
Agencia Estatal Consejo Superior de Investigaciones Cientificas, CSIC	Spain
MTA Okologiai es Botanikai Kutatointezete	Hungary
Stichting Europees Centrum Voor Natuurbescherming, ECNC	Netherlands
Bangor University	U.K.
Eigen Vermogen van Het Instituut voor Natuur- en Bosonderzoek, INBO	Belgium
Umweltbundesamt GmbH, EA	Austria
Suomen Ymparistokesus, SYKE	Finland
Botanical, Envirionmental & Conservation Consultants Lmt.	Ireland
Vlaams Instituut vor de Zee, VLIZ	Belgium

Mission & Principles of BiodiversityKnowledge

BiodiversityKnowledge is an initiative by researchers and practitioners to set up and operationalize a Network of Knowledge to improve the knowledge flow between biodiversity knowledge holders and users in Europe.

The goals of BiodiversityKnowledge are to answer questions from decision making, to improve the evidence base, to contribute to developing a research strategy, and to enable societal actors to make better informed decisions concerning biodiversity and ecosystem services.

The approach of BiodiversityKnowledge strives to integrate all relevant forms of knowledge to answer questions jointly formulated with decision makers using transparent and rigorous procedures. Throughout this approach, BiodiversityKnowledge relies on and provides networking, actively builds capacity and engages in learning on all aspects of knowledge interfacing. Accordingly, the processes of BiodiversityKnowledge matter as much as topics and outputs to ensure a coherent and credible approach.

BiodiversityKnowledge will…

(1) enable **OPENNESS** by wide participation from all potential actors, including relevant experts and knowledge holders, through open invitations for participation, building on participants' enthusiasm and diversity, and ensuring open access to the NoK products.

(2) **ENSURE QUALITY**, by applying established and tailored methodologies, developing systems for quality assurance including extended peer-review, and responding to feedback.

(3) **MINIMISE BIAS and ENSURE FAIR and TRANSPARENT PROCESSES**, by ensuring scientific rigour, broad participation, and by avoiding conflicts of interest, through clear rules and procedures.

(4) **AVOID DUPLICATION** by collaborating with relevant established institutions to maximize efficiency and minimize costs in science-policy interactions.

(5) integrate **CAPACITY BUILDING** as essential component to improve collaborative working and information sharing.

(6) ensure strong internal and external **COMMUNICATION.**

(7) integrate **REFLEXIVITY and LEARNING**, by ensuring that processes and results are continuously and formatively evaluated.

www.biodiversityknowledge.eu

ACKNOWLEDGEMENTS: The EU funded Coordination Action KNEU (Grant No. 265299), that aims at developing a European Scientific biodiversity Network of Knowledge to inform policy-making and economic sectors (ENV.2010.2.1.4.3-3) and whose consortium wrote the present document, would like to extend many thanks to all the participants: those who attended the workshops in Budapest, Copenhagen, Aix-en Provence, Brussels, Wageningen, Glasgow, Ghent, Paris, Peterborough, Frankfurt/Main as well as to the BiodiversityKnowledge conferences in Brussels in May 2012 and Berlin, September 2013, and also those who have answered questionnaires, surveys, and provided support and feedback at different stages. Without their input, this paper would have never gone so far into detail. This document has been continuously enriched through the exchanges between various backgrounds, expertise, visions, and different cultures.

This project has received funding from the European Union's Seventh Programme for research, technological development and demonstration under grant agreement No 265299 (KNEU-Project)
