# Supplementary material for: PESI - a taxonomic backbone for Europe
Source: Biodivers Data J. 2015 Sep 28;(3):e5848. doi: 10.3897/BDJ.3.e5848 (PMC4609752; doi:10.3897/BDJ.3.e5848)
Supplement: Supplementary material 29 — ViBRANT: Design of robust services [file biodiversity_data_journal-3-e5848-s029.pdf]

Deliverable D4.3

Design of robust services

(D1.1 Overall management)

ViBRANT status report

BGBM: Lorna Morris, Sybille Bürs, Andreas Müller, David Fichtmüller, Cherian Mathew, Andreas Kohlbecker, Anton Güntsch, Walter Berendsohn
GBIF: Dag Endresen, Éamonn O Tuama, David Remsen, Burke Chih-Jen Ko
MfN: Falko Glöckler, Alexander Kroupa, Gregor Hagedorn
Pensoft: Lyubomir Penev, Pavel Stoev, Teodor Georgiev, Jordan Biserkov
UPMC: Regine Vignes-Lebbe, Thomas Burguiere, Nils Paulhe
JKI: Gregor Hagedorn, Andreas Plank
OU: Dauvit King, David Morse
NHM: Simon Rycroft, Alice Heaton, Ed Baker
UvA/LW: Wouter Los
RBINS: Yde de Jong (lead)

Associate partner (Harvard)**:** Robert (Bob) Morris, Steve Baskauf

Compiled by: Yde de Jong

Date: 25 November 2013 (v.1), 27 November 2013 (v.2), 24 January 2014 (v.3)

1 Introduction

This deliverable reports the status and enhancements made of the prototype tools produced in workpackage 4 under the earlier D4.1[1] and D4.2[2] workplans and later extensions, including the final implementation and integrated usage of tools and services within the ViBRANT framework and some future outlooks.

2 Description

ViBRANT workpackage 4 is devoted to guaranteeing that all data within ViBRANT are compatible with, and available to, other research and publishing infrastructures and services within and outside the project. This includes ensuring cross-platform interoperability and the development and sharing of externally standardised ontologies throughout the ViBRANT framework (see Fig. 1).

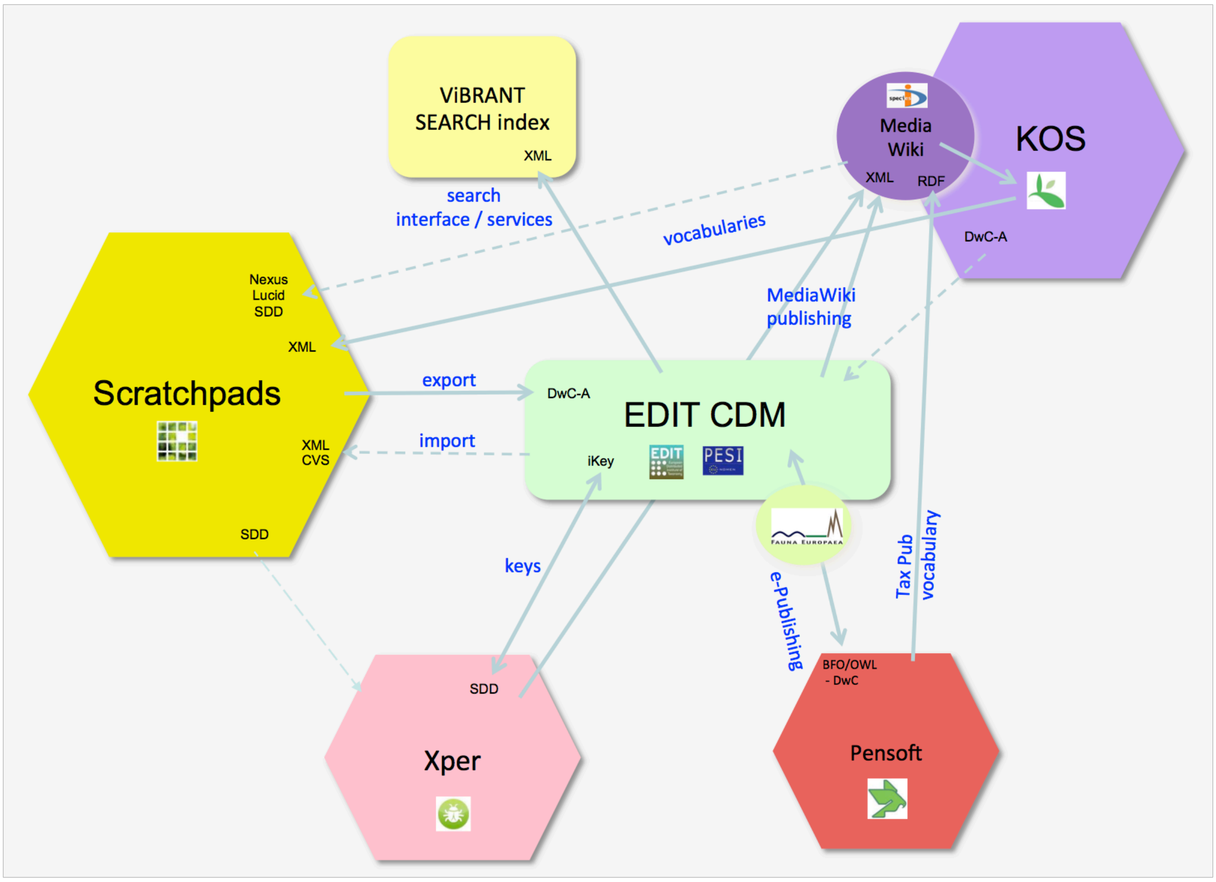

Figure 1: Outline of the ViBRANT framework showing the relevant WP4 core platforms and components.

In this context, within this report, several ViBRANT infrastructural features and functions will be highlighted, including:

- The usage of the GBIF Vocabulary Platform with other platforms, addressing best practices for maintaining a federated Knowledge Organization System (KOS) with a common vocabulary of terms.

[1] **D4.1** – http://vbrant.eu/content/d41-scratchpad-common-access-point
[2] **D4.2** – http://vbrant.eu/content/d42-ontology-tools

- The capacities of the EDIT Platform for Cybertaxonomy to interoperate with the Scratchpads, KOS, Pensoft and Xper platforms, providing a virtual common access point, optimising data exchange for taxonomists as a virtual community and supporting the generalisation of descriptive data.
- The implementation of the ViBRANT Index as a common searching index and service of ViBRANT and CDM data.
- The potential of the MediaWiki as a collaborative community interface to facilitate the (bottom-up) definition and management of vocabularies and as a sustainable, versioned publication tool for the online publishing of content from Scratchpads, CDM, and Xper2 resources.
- Contributions on the hosting and publishing of taxonomic authority files using the particular platforms capacities, particular on Fauna Europaea.

3 GBIF Vocabulary Platform

3.1 Introduction

Vocabularie[3] are an essential foundation for data interoperability within the biodiversity domain, enabling the convergence on shared terminology for integrating biodiversity informatics resources. During the ViBRANT project routines and mechanisms to build, manage and exchange vocabularies have been developed.

3.2 Semantic Media Wiki

Vocabulary management systems support a community to develop and manage terminology for their domain. Such tools range from generic spreadsheet templates to customised Content Managements systems. After investigation and evaluation[4], Semantic MediaWiki (SMW)[5] was selected and implemented[6] [7] as the application of choice for promotion within the GBIF/ViBRANT community.

For the development of the Semantic MediaWiki as a vocabulary management system, additional content providers and collaborators were sought; canvassing (existing) glossaries and engaging interested communities [8]. As examples, the TaxPub vocabularies as well as the Audubon Core standard[9] were integrated into the MediaWiki, later followed by the BiolFlor Data[10].

Semantic MediaWiki was identified as a complementary component of the GBIF KOS system (Fig. 2) and later adopted as the TDWG Terminology Platform, which is being

---

3 "Controlled vocabularies" typically refers to content value vocabs, i.e., the values taken by "property" vocabularies like DwC. We addressed both property and content value vocabs in ViBRANT and so the general term "vocabularies" is more accurate here instead of 'controlled vocabularies'.

4 **M4.11** – http://vbrant.eu/content/m411-prototype-collaborative-community-interface

5 **GBIF MediaWiki** – http://terms.tdwg.org/wiki/

6 **M4.11** – http://vbrant.eu/content/m411-prototype-collaborative-community-interface

7 **M4.13** – http://vbrant.eu/content/m413-release-api-catalogue-resources

8 **M4.19** – http://vbrant.eu/content/m419-canvassing-glossary-oriented-potential-contributors-0

9 **M4.27** – http://vbrant.eu/content/m427-audubon-core-standard-mediawiki

10 **M4.22** – http://vbrant.eu/content/m422-biolflor-data-semantic-web

hosted at Biowikifarm. Its features are extensively discussed in D4.2[11], including the comparison and relationship with the GBIF vocabulary services.

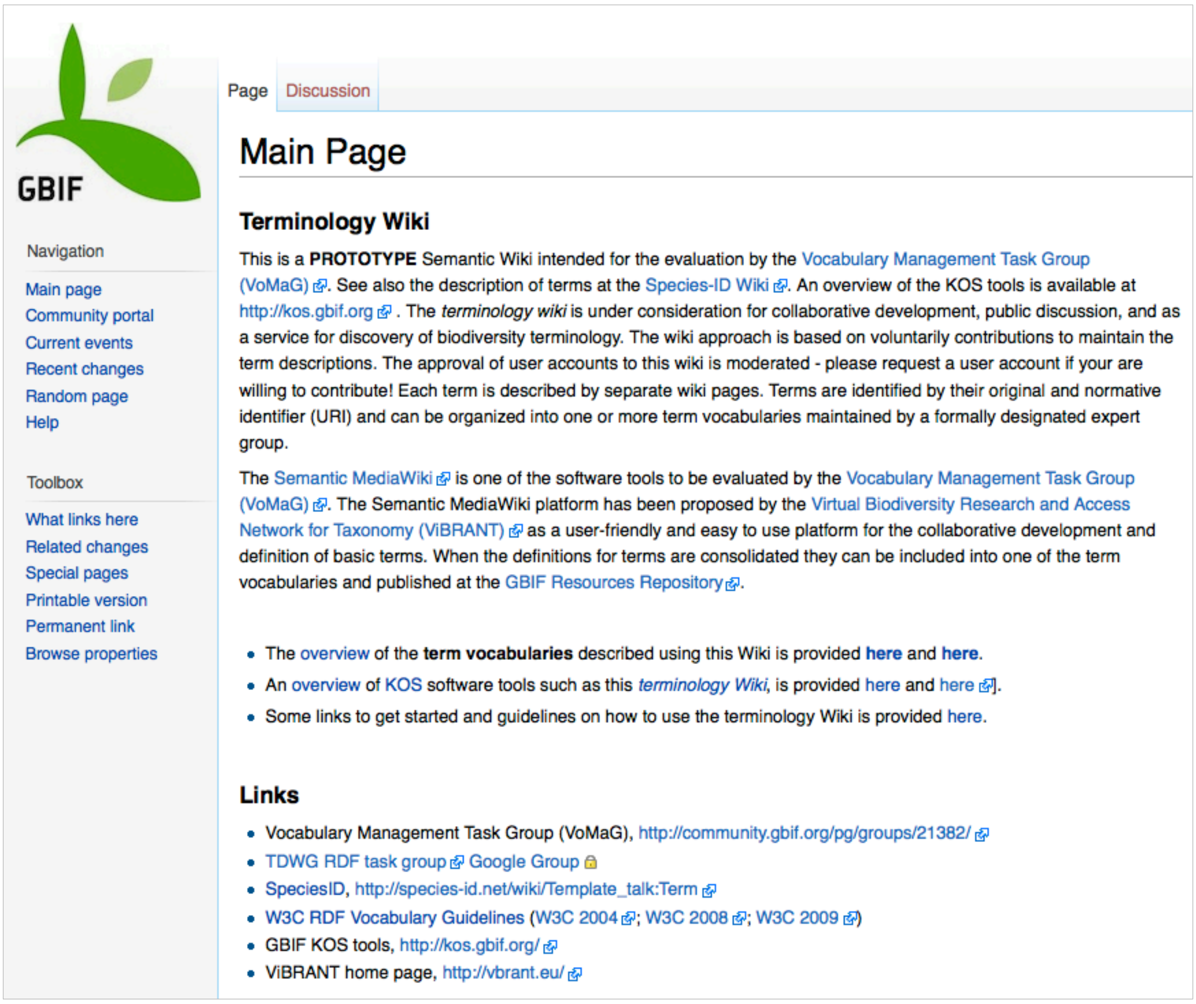

Figure 2: Main page of GBIF-Terms-Wiki.

3.3 GBIF KOS Infrastructure

During the ViBRANT project, alternative vocabulary management systems were examined by GBIF, e.g., BioPortal and IsoCAT, and participation in meeting and workshops undertaken to understand best practices for supporting the use of vocabularies and ontologies in the biodiversity community[12]. This resulted in the establishment of the TDWG VoMaG task group[13] and in the uptake of some widely used vocabularies such as Darwin Core, Dublin Core, FOAF.

Once a vocabulary is ready, it will be published in one or more registries for discovery and accessed in a standard format. An example of such a vocabulary registry used in ViBRANT is the GBIF Vocabularies[14], an example of a vocabulary access service is the Resources Repository[15], enabling the (automated) consumption of vocabularies by other

---

11 **D4.2** – http://vbrant.eu/content/d42-ontology-tools

12 **M4.12** – http://vbrant.eu/content/m412-liaison-and-networking-ontology-experts-and-existing-ontology-providers

13 **TDWG VoMaG** – http://www.gbif.org/resources/2246

14 **GBIF Vocabularies** – http://vocabularies.gbif.org/

15 **GBIF Resource Repository** – http://rs.gbif.org/

systems, including Scratchpads.

Both vocabulary management and discovery systems are part of the Knowledge Organization System (KOS)[16], proposed as a new architecture for biodiversity information resources, available for any user to develop or publish vocabularies, supported by ViBRANT[17] and hosted at the TDWG Terminology Platform (Fig. 3).

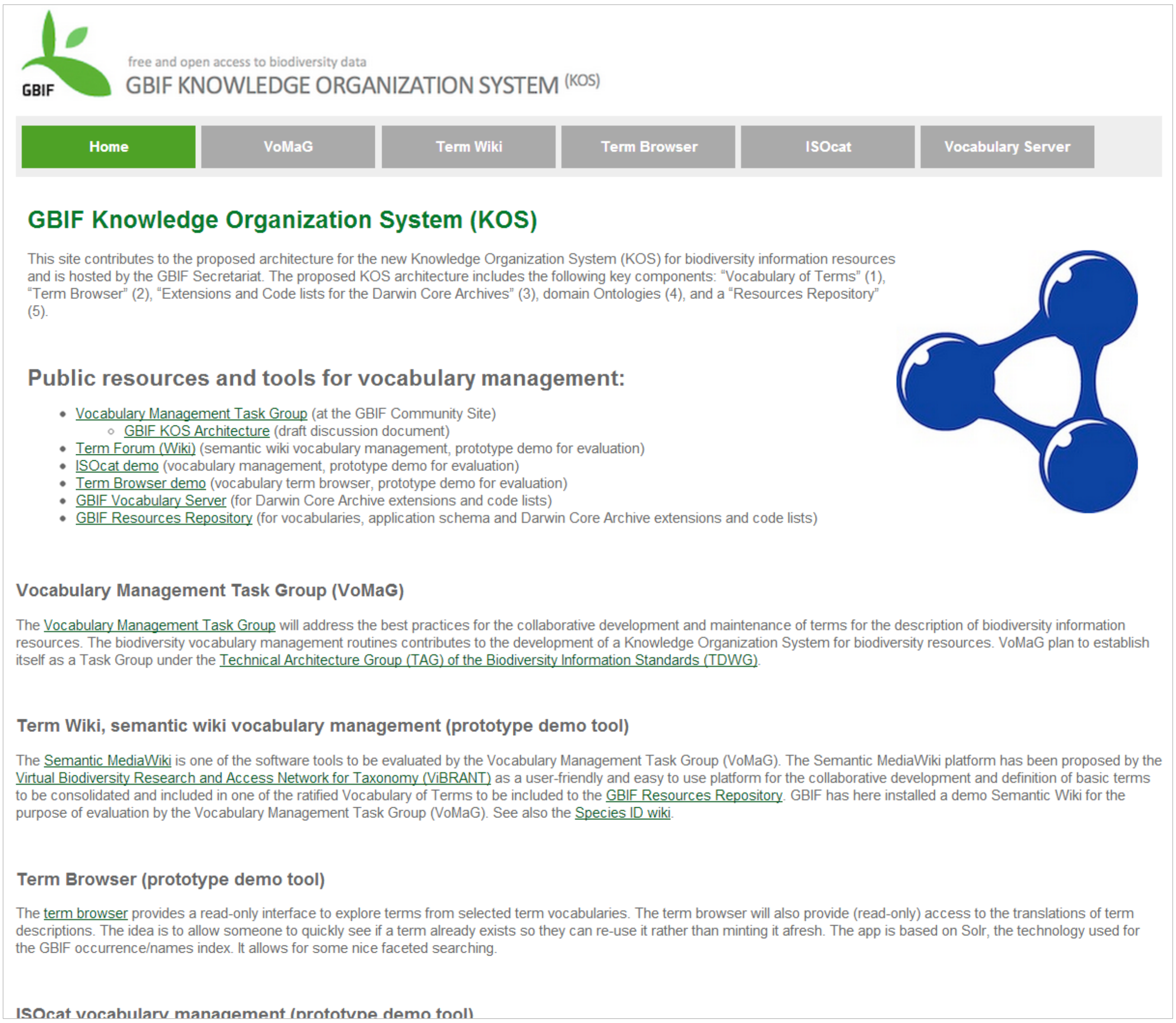

Figure 3: The GBIF Knowledge Organization System (KOS).

3.4 Integration of KOS vocabularies into Scratchpads

In ViBRANT some milestones contribute to the objective on establishing the interoperability between the GBIF Vocabulary Platform (KOS) and the Scratchpads, connecting both internet resources using well-defined APIs and XML exchange routines, allowing the sharing of standardised vocabularies –developed by the scientific community– by Scratchpads users within the GBIF KOS infrastructure. The initial concepts on developing a direct exchange routine between MediaWiki and Scratchpads[18], where modified in a later stage of the project in favour of a direct

16 **GBIF KOS** – http://kos.gbif.org/

17 **D4.2** – http://vbrant.eu/content/d42-ontology-tools

18 **M4.13** – http://vbrant.eu/content/m413-release-api-catalogue-resources

connectivity of the Scratchpads to the GBIF vocabulary tools[19] (see Fig. 4).

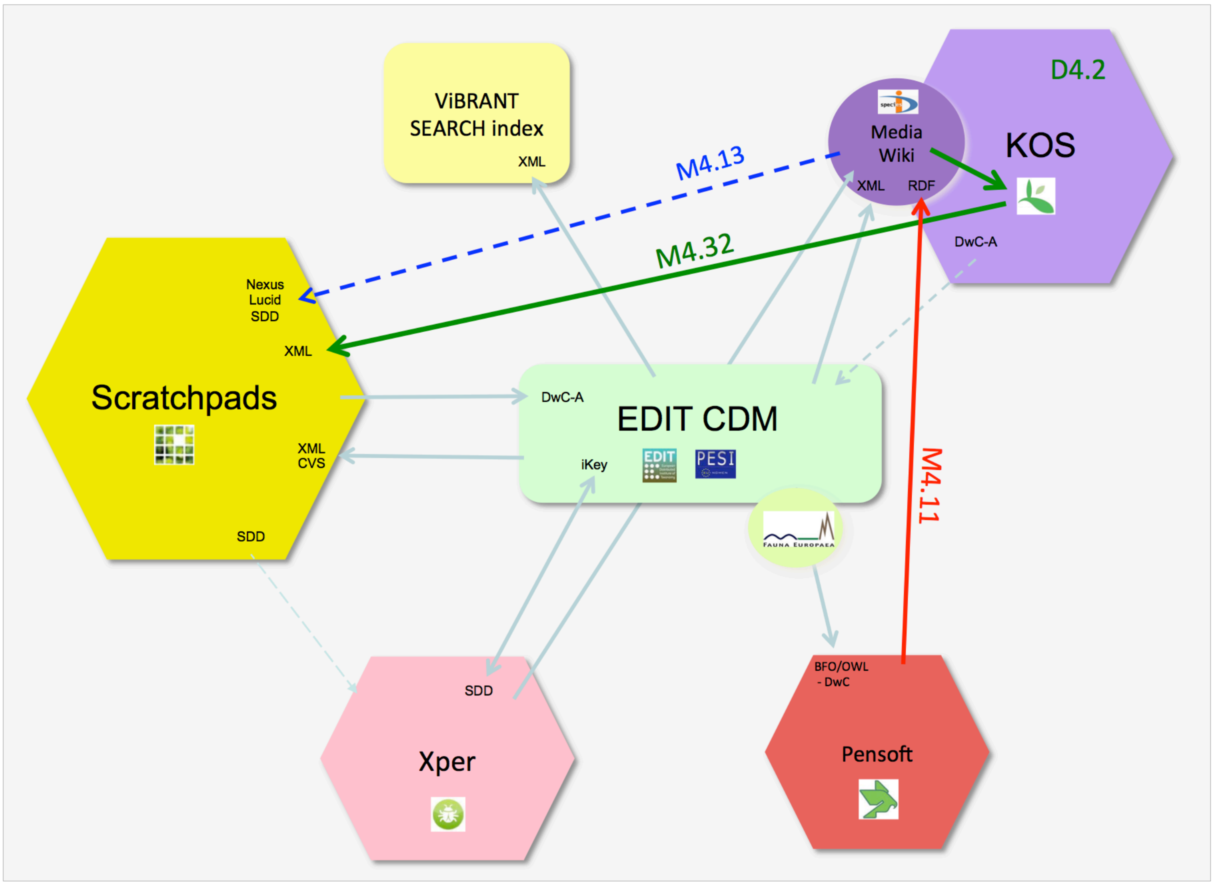

Figure 4: The position of the *Ontology Platform* milestones within the ViBRANT architecture.

A feature is installed replacing controlled vocabularies in Scratchpads by vocabularies from the GBIF Resources Repository[20] (Fig. 5). Updates of GBIF terms will be applied on the Scratchpads sites, giving the maintainers the option to decide how the changes are applied when there is no obvious way.

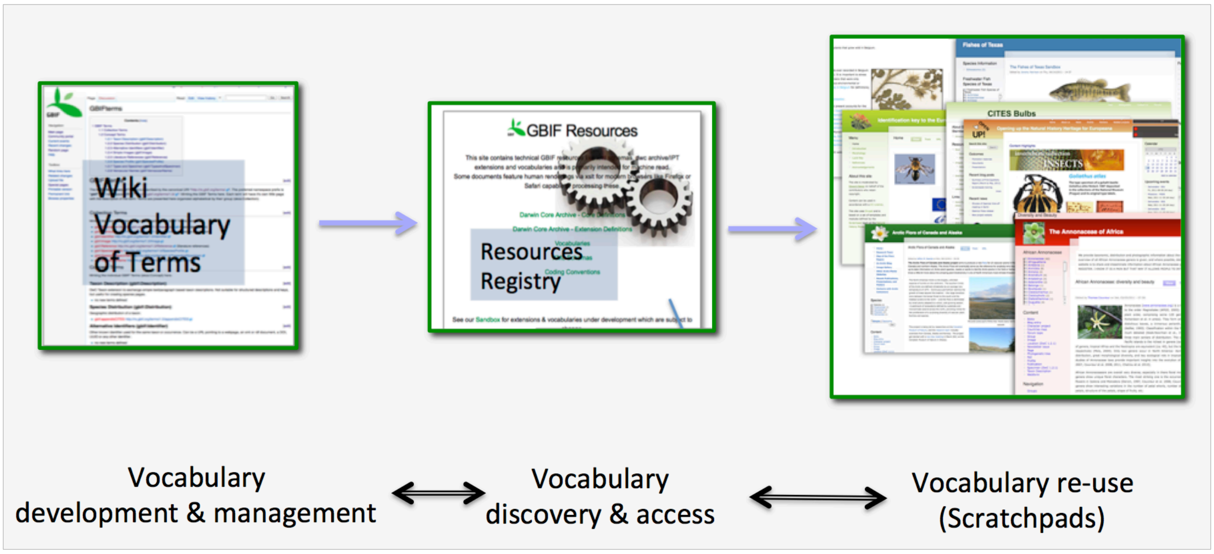

[19] **M4.32 –** http://vbrant.eu/content/m432-integrating-wiki-glossary-scratchpad

[20] **GBIF Resource Repository –** http://rs.gbif.org/

Figure 5: Proposed data-flow showing the integration of MediaWiki terms into the Scratchpads.

For the initial release of the vocabulary replacement feature, the 'Type status'[21] and 'Basis of record'[22] terms are used, although users could create their own fields and map them to other vocabularies (Fig. 6).

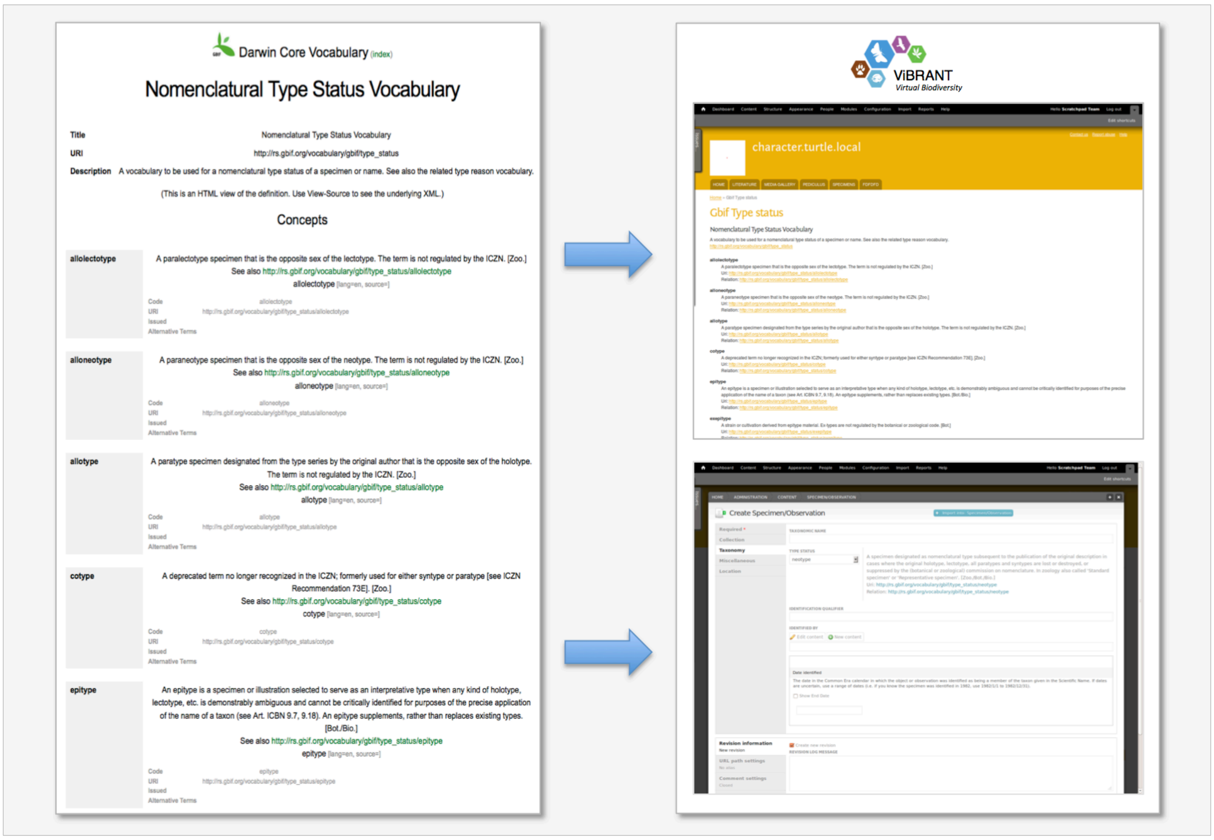

Figure 6: Example showing the uptake of GBIF 'Type status' terms into two Scratchpads.

Updates of GBIF terms will be applied on the Scratchpads sites - giving the maintainers the option to decide how the changes are applied when there is no obvious way (for instance when a term is deleted, maintainers will be given the option to re-map the deleted term).

On the Scratchpads side users can't add or edit the terms. However, with the full description of the vocabulary and each term within it; as well as per-term links to the GBIF wiki, when relevant they can propose any changes.

On usage: The above-described feature is awaiting testing in a formal Scratchpads release. To further ensure efficient cross-platform operability, the relevance of RDF as a potential important future exchange format is recognised [23], which should require the adaptation of some pipelines. BioWikiFarm statistics can be found in figure 7.

21 **Darwin Core Vocabulary (index)** – http://rs.gbif.org/vocabulary/gbif/type_status.xml

22 **Darwin Core Vocabulary (index)** – http://rs.gbif.org/vocabulary/dwc/basis_of_record.xml

23 At present the GBIF vocabularies are tied to the requirements of the IPT/DwC archive system and use propriety XML format. They can be seen as a kind of application schema. For real interoperability, the terms used in the DwC extensions (whether property or value terms) need to be expressed in a standard like RDF or SKOS for wide re-use.

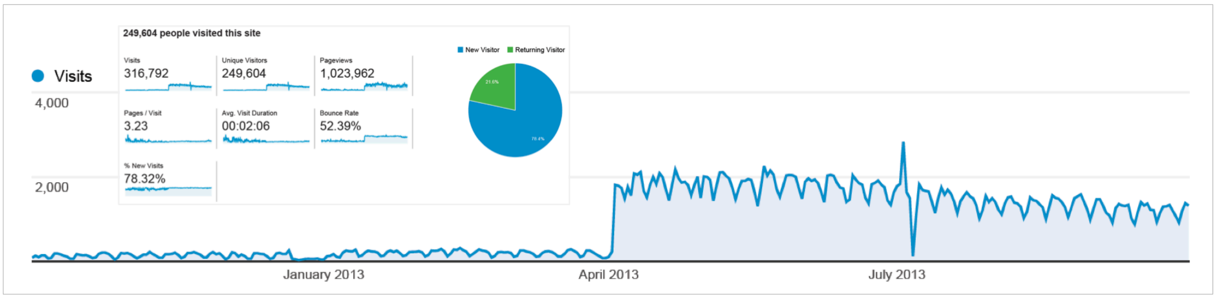

Figure 7: BioWikiFarm statistics up to 1 October 2013.

4 Cybertaxonomy Platform

4.1 Introduction

The EDIT Cybertaxonomy Platform[24] supports the full taxonomic workflow from data acquisition to publication. It consists of software for advanced data processing and capture (like the Taxonomic Editor - Fig. 7) and a series of web portals for viewing and querying the data. In the ViBRANT project the emphasis is on interoperability – taxonomists using the EDIT platform can share their data with users of other platforms (for example Scratchpads) by means of agreed international data standards.

The Cybertaxonomy Platform benefits from extensible and flexible data import and export facilities (for example publishing PDF documents of Flora treatments). During the ViBRANT project additional exchange routines have been developed, importing data from Scratchpads, creating a single web service to (integrally) search all Scratchpads data and routines for exporting data, for example publishing Mediawiki Taxon pages of taxonomic hierarchies from CDM resources.

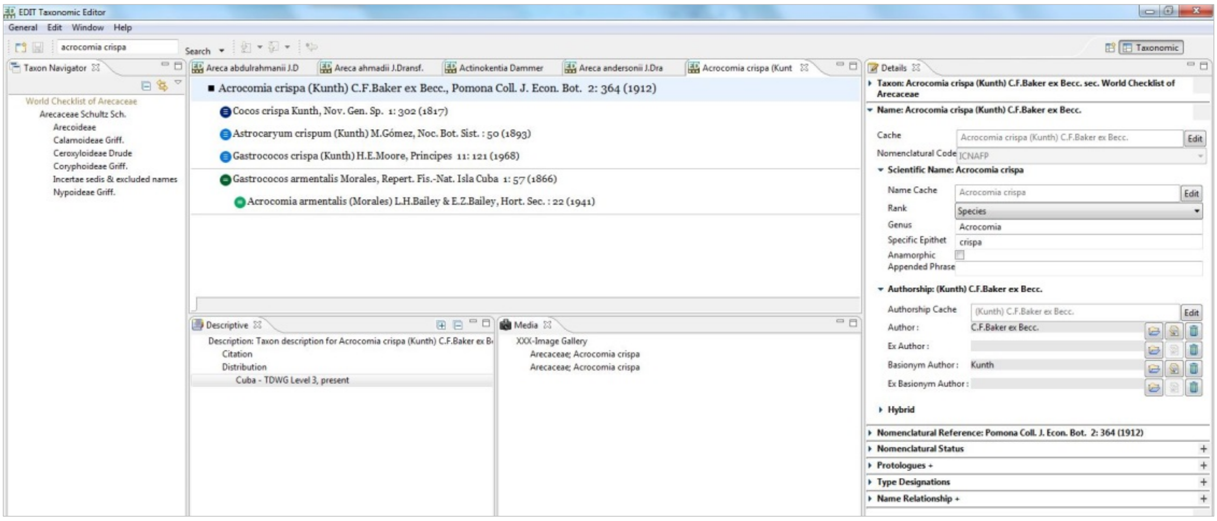

Figure 7: The EDIT Taxonomic Editor interface.

4.2 Integration of Scratchpads data into CDM = ViBRANT Export Module

A Scratchpads module is implemented to export data from the Scratchpads2 database for DwC-A extension, allowing users to specify the Scratchpads data they need in their DwC-A export file via a web form[25]. Each of the 5 DwC-A extensions in the default export module could be mapped to a Scratchpads view defined by the user. A static meta.xml file is provided in the export, which contained the default field mapping between fields in the Scratchpads view and terms in each extension[26]. The mapping has been described in detail in D4.1[27].

The mapping relies on users specifying an appropriate view for the extension, with the correct field types in the correct order. Moreover some data consumers intending to

[24] **EDIT Cybertaxonomy Platform** – http://cybertaxonomy.eu/

[25] **M4.10** – http://vbrant.eu/content/m410-define-methods-use-scratchpad-service-layer-deliverable-within-cdm-store

[26] **M4.17** – http://vbrant.eu/content/m417-extensions-dwc-export-functionality

[27] **D4.1** – http://vbrant.eu/content/d41-scratchpad-common-access-point

export data from Scratchpads (e.g. Encyclopedia of Life) are interested in different subsets of the data and simply providing a default set of extensions and fields to export is not sufficient. Therefore a flexible mapping solution was required[28]. The DwC-A mapping module is available from the Scratchpads git repository[29].

The user-interface can access the module by requesting an URL[30]. The module provides an interface for the user to create a Scratchpad view for the taxon core and extension data they would like to export (or uses default views that are provided with module). Then a user configures the DwC-A term mapping to each Scratchpad field they want to export (multi-step form). Finally DwC-A zip files are generated (the DwC-A is updated automatically whenever the data are changed), containing the meta.xml (configured by the user) and the text files for each of the views. Figures 9 shows the user interface for the mapping.

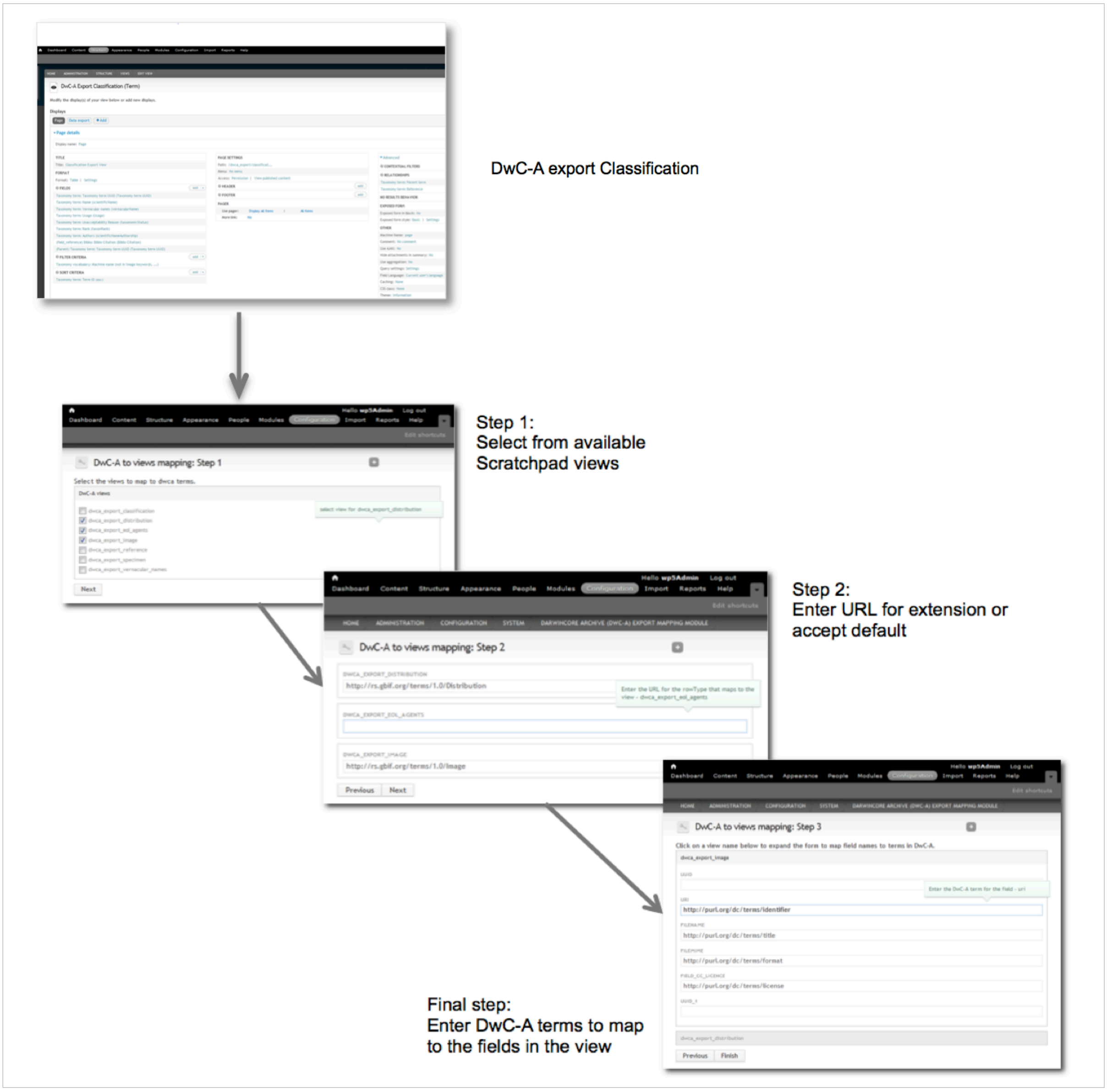

Figure 9: Scratchpad to DwC-A Mapping and Export Module.

28 **M4.23** – http://vbrant.eu/content/m423-scratchpad-dwc-mapping-module

29 **Scratchpads git repository** – https://git.scratchpads.eu/v/scratchpads-2.0.git

30 **DwC Export Module** – http://<scratchpads-base-url>/admin/conifg/system/dwca_export_views

Further discussion on using the generated DwC-A in the EDIT Common Data Model and the ViBRANT Index database, including new extensions and DwC-A terms required for import of the data into the CDM, was done in the context of the ViBRANT Index developments[31].

4.3 Integration of CDM data into Scratchpads = ViBRANT Export Module

A (reciprocal) functionality to export a Darwin Core Archive (DwC-A) from a CDM database has been implemented in an earlier release of the CDM.

The CDM already has a functionality to export a Darwin Core Archive (DwC-A) from a CDM database. The export procedure creates a zip file containing multiple csv based text files for different data types and a meta.xml describing the data in each text file.

Within ViBRANT, filter functionality was added to csv exports from the CDM. This filter functionality will facilitate the export of specific sub-trees, for instance a selected Taxon node and all its children from a CDM database.

Scratchpads do not currently support importing of DwC-A files. To test and improve the interoperability between Scratchpads and the CDM, an import of the CSV data into Scratchpads was made[32] using the Scratchpads interface for importing Excel files[33].

4.4 Common Scratchpads - CDM search interface = ViBRANT Index

For publishing content the MediaWiki (see Chapter 5 below) has a minimal and very flexible structure, it is good at adding relatively ad-hoc new requirements and at preserving content in the light of change (i.e. very old modelled data and new data can survive side by side). The MediaWiki is, however, less prepared for giving a fixed export/import interface for a very highly defined system with many consistency requirements, like the CDM (or GBIF vocabulary services). Therefore the role and application of the ViBRANT Index as an additional publishing platform, facilitating an integrated access and alternative views on ViBRANT and other resource data was investigated in the ViBRANT project.

The ViBRANT Index database is a Common Data Model (CDM) based data warehouse consisting of taxonomic data imported from a variety of sources from the Scratchpads and CDM. Starting with a simple web-based user-interface[34] it was extended enabling full text searching of data[35]. Later a new service for generating statistical summary data from the CDM was implemented[36]. The ViBRANT Index database makes use of the specially adapted web-service, service and persistence layers in the CDM[37].

The ViBRANT Index is supposed to provide a functional taxonomic Clearing House, simplifying the access and use of taxon names, including the production of regional

---

31 **M4.24** – http://vbrant.eu/content/m424-full-text-search-cdm-vibrant-index

32 **M4.35** – http://vbrant.eu/content/m435-filter-functionality-cdm-export

33 **Scratchpads Import tool** – http://help.scratchpads.eu/w/Import

34 **M4.18** – http://vbrant.eu/content/m418-human-interface-cdm-vibrant-index

35 **M4.24** – http://vbrant.eu/content/m424-full-text-search-cdm-vibrant-index

36 **M4.28** – http://vbrant.eu/content/m428-statistical-output-provided-cdm-vibrant-index-query-interface

37 **M4.31** – http://vbrant.eu/content/m431-webservice-layer-adapted-vibrant

checklists and taxonomic catalogues (e.g. relevant for CoL and PESI). In this function the ViBRANT Index could notably contribute to global efforts on developing smart and practical taxonomic name resolution services and could play an important role as an interface towards e-Science projects (like BioVel[38]).

The below screenshot (Fig. 10) of the user-interface[39] describes a simple query scenario.

Taxonomic data from all available Scratchpads 2.0 sources have been imported into the CDM ViBRANT index database (using the Export Module). The ViBRANT index user interface displays the link back to the Scratchpad source for each individual Taxon in the query results. The user interface could be extended to enable the user to filter the results e.g. on description type (Scratchpads description type data include for example: general description, distribution, conservation).

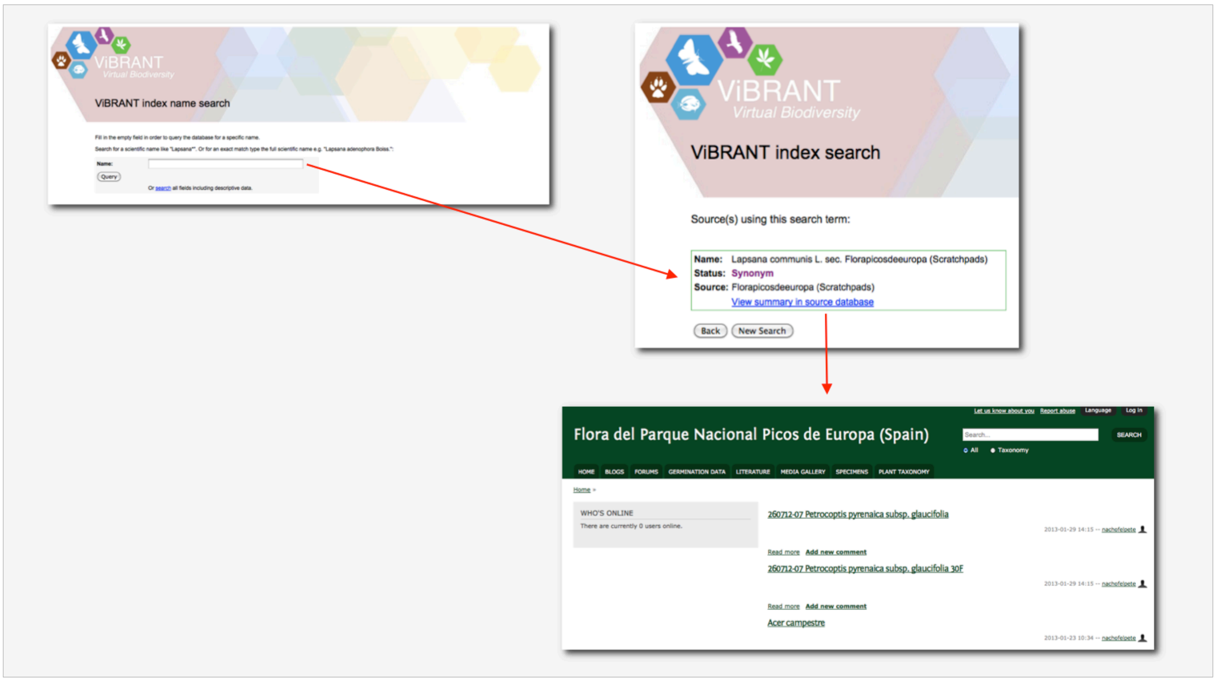

Figure 10: Example of querying the ViBRANT index for a full text search.

In the final stage of the ViBRANT project the ViBRANT Index web service statistics has been advanced[40] and the ViBRANT Index services been optimised for e-Science workflow application, using the new "workflow-enabled" CDM web service layer[41] developed for i4Life[42].

On usage: currently 67 Scratchpads are harvested (via DwC-A) into a single CDM database. 262,664 taxa (125,845 accepted taxa, 136,819 Synonyms) and 154,963 references (of which 105,362 nomenclatural references) are included.

38 **BioVel** – http://www.biovel.eu/
39 **ViBRANT Index** – http://dev.e-taxonomy.eu/vibrant_index/search
40 **M4.40** – http://vbrant.eu/content/m440-advanced-statistics-web-services-vibrant-index
41 **M4.41** – http://vbrant.eu/content/m441-workflow-optimized-services-vibrant-index
42 **i4Life** – http://www.i4life.eu/

4.5 Integrating CDM and Xper data = ViBRANT Single Access Key Service

Xper2[43] is a platform that utilises structured descriptive data to annotate taxonomic data, for the purpose of taxonomic identification developed by UPMC[44]. The CDM is able to handle character data. A web service is implemented for producing single access keys from the exchange format for structured descriptive data (SDD), helping the CDM to build a fully consistent descriptive system to be used in multi-access keys and to add very fine granular descriptive information items.

To organise the integrated access of descriptive features across the different platforms, a pilot implementation of the web service into the EDIT platform was developed in ViBRANT[45]. As an initial test a TDWG-SDD file containing descriptive character of 300 Cichorieae taxa was exported from the CDM to Xper2 to generate a key and get the SDD data right into the CDM, transformed into a relevant CDM iKey-Plus single access key. The Cichorieae test portal results, showing the generated key, have been visualised in the CDM data portal[46] (see Fig. 11 left).

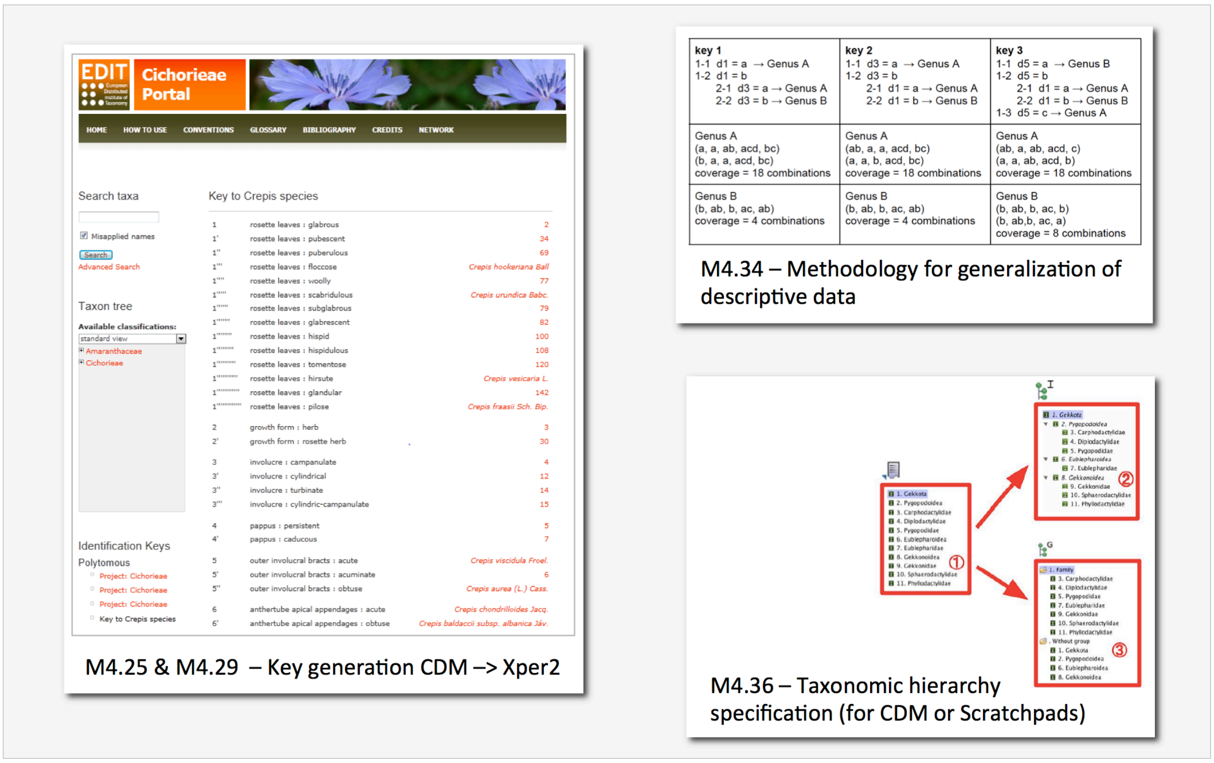

Figure 11: CDM - Xper2 import & export features.

CDM-SDD output was further optimised for Xper2 Applet[47] and a specification of the methodology for the generalisation of descriptive data (e.g. from specimens to taxon) for taxonomic generalisation was implemented in a prototype in Xper2[48] (Fig. 11 upper right). In addition, a mechanism was added to manage taxonomic hierarchy items in

---

43 **XPER** – http://lis-upmc.snv.jussieu.fr/lis/?q=en/resources/software/xper2

44 **UPMC** – http://www.identificationkey.fr/

45 **CDM iKey-Plus** – http://dev.e-taxonomy.eu/svn/branches/cdmlib/ikey-plus-import/

46 **M4.25** – http://vbrant.eu/content/m425-pilot-implementation-webservice-bgbm

47 **M4.29** – http://vbrant.eu/content/m429-cdm-sdd-output-optimised-xper2

48 **M4.34** – http://vbrant.eu/content/m434-prototype-taxonomic-generalisation

Xper2, similarly like in CDM and Scratchpads[49] (Fig. 11 down right). A further Xper2 integration into the CDM Taxonomic Editor is in progress; a beta version will be ready by the end of the project[50].

4.6 Conclusions

A *Common Access Point* was created for the Scratchpads, allowing a standardised cross-platform integration of taxonomic data, joining different infrastructural components and tools, using the CDM as a central hub and route of access to the Scratchpads and Xper platforms and using the ViBRANT Index as a (common) publishing platform (see Fig. 12). As a result, data can be exported from Scratchpads and imported into the CDM where it could be processed, edited and queried using the CDM toolset.

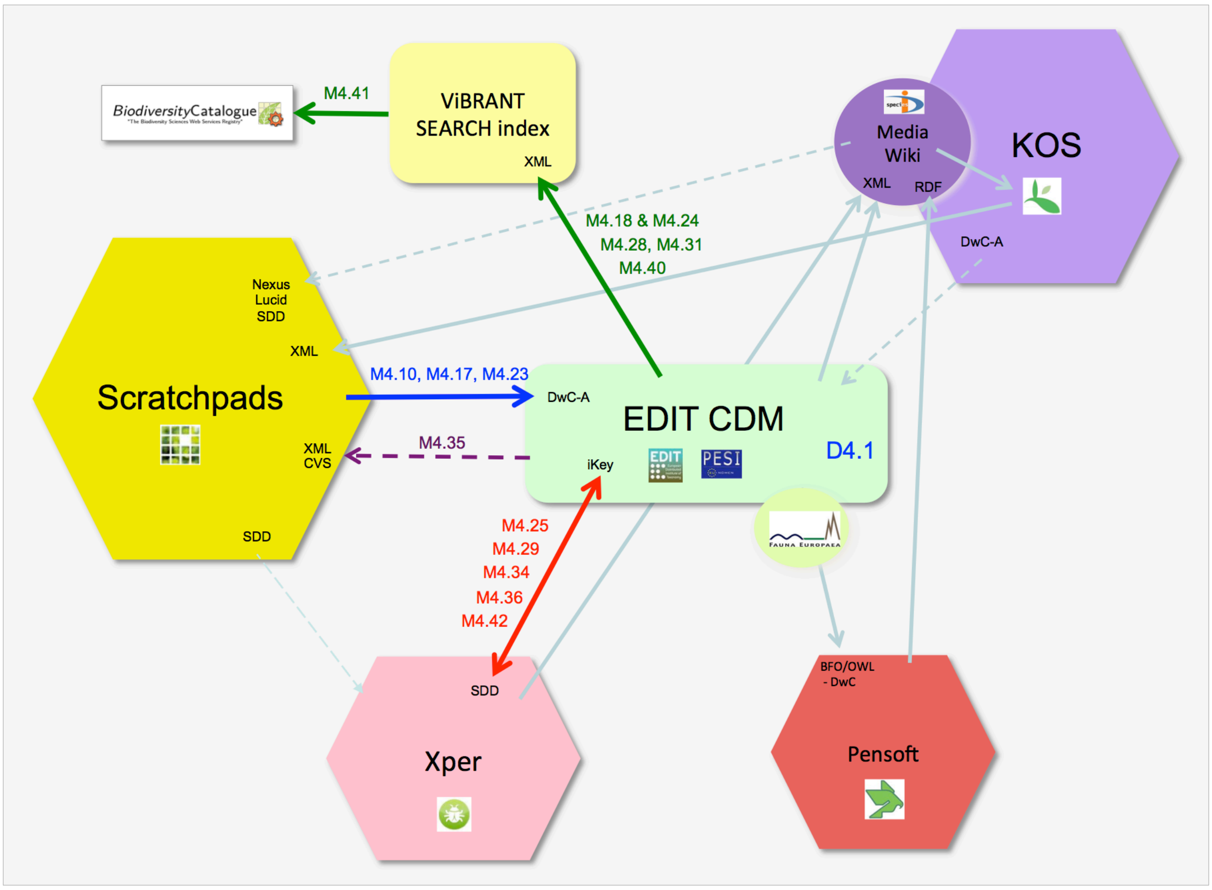

Figure 12: The position of the *Scratchpads Common Access Point* milestones within the ViBRANT architecture.

5 MediaWiki Platform

5.1 Introduction

ViBRANT prioritised the development of Application Program Interface (API) data delivery services to support an open data strategy to enable ViBRANT users to deliver and expose their data to external users and services, including researchers and major global biodiversity informatics initiatives. In this context in ViBRANT WP4 both the

---

49 **M4.36 –** http://vbrant.eu/content/m436-xper2-cdm-relation-taxonomic-hierarchy-specification

50 **M4.42 –** http://vbrant.eu/content/m442-xper2-integration-cdm-taxonomic-editor

MediaWiki and ViBRANT index (see earlier) are investigated as publishing platforms for biodiversity data.

5.2 MediaWiki as common publishing platform in ViBRANT

The use of the MediaWiki in KOS, because of representing the most stable solution to introducing KOS services to the broader community, was already discussed. Despite the sociological challenge, even if this does not generate an immediate product, it represents a significant step forward.

Also for Xper2 and the Cybertaxonomy platforms the publishing capability of MediaWiki was investigated as an online publication tool, providing long-term stable versions of content from the respective (Scratchpads, CDM, Xper2) platforms.

As a prologue, the structure for the taxonomic content of the wiki pages was designed, including an XML representation of parts of a CDM database (e.g. taxon family or genus), and transformed as XSLT, producing the wikitext sources for the MediaWiki pages[51].

As a next step the XML export of data from the CDM was further optimised and the entire workflow from the export of a selected CDM data source, the transformation into to wikitext format and the import of the files into a MediaWiki further automated. Comparable XML harvesting and exporting routines are developed for Xper, creating a hierarchised and cross-linked pool of pages in a MediaWiki server[52] (see Fig. 13).

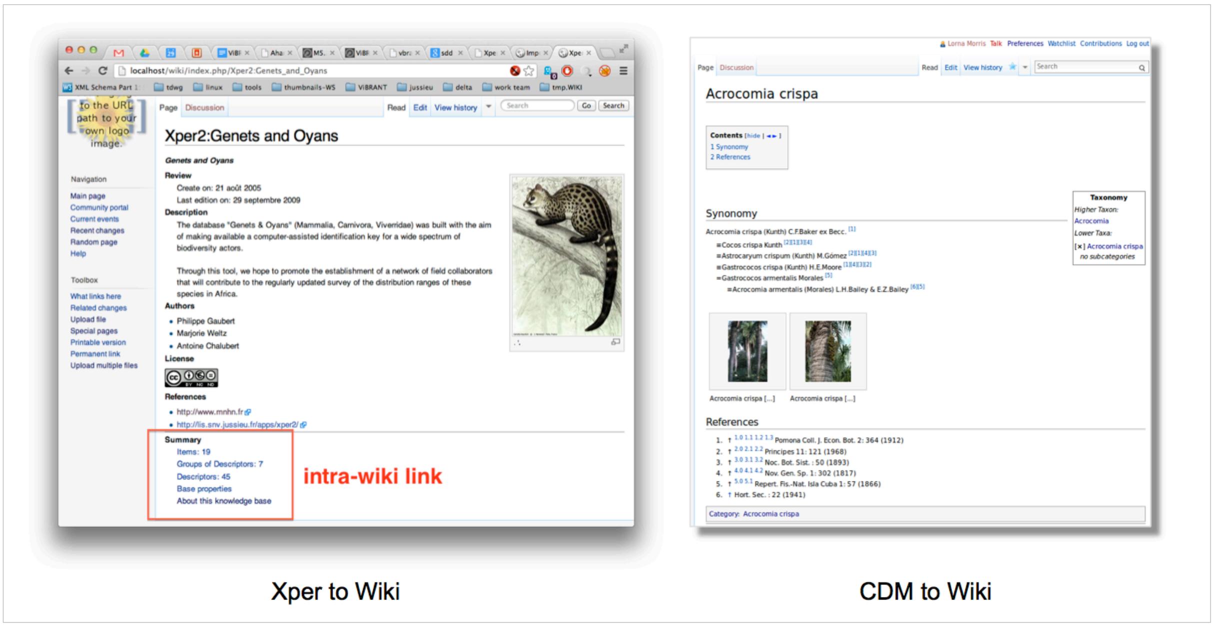

Figure 13: Xper2 and EDIT Platform publishing capability on Mediawiki.

5.3 Conclusions

The suitability of the MediaWiki, as *standard data interfaces and APIs* in ViBRANT has been effectively exploited (Fig. 14), including the implementation of the relevant technical workflows for exporting XML, transforming the data into wikitext format and the import of the files into structured Wiki pages.

---

51 **M4.33** – http://vbrant.eu/content/m433-xml-tranformations-cdm-export

52 **M4.37** – http://vbrant.eu/content/m437-xper2-and-edit-platform-publishing-capability-mediawiki

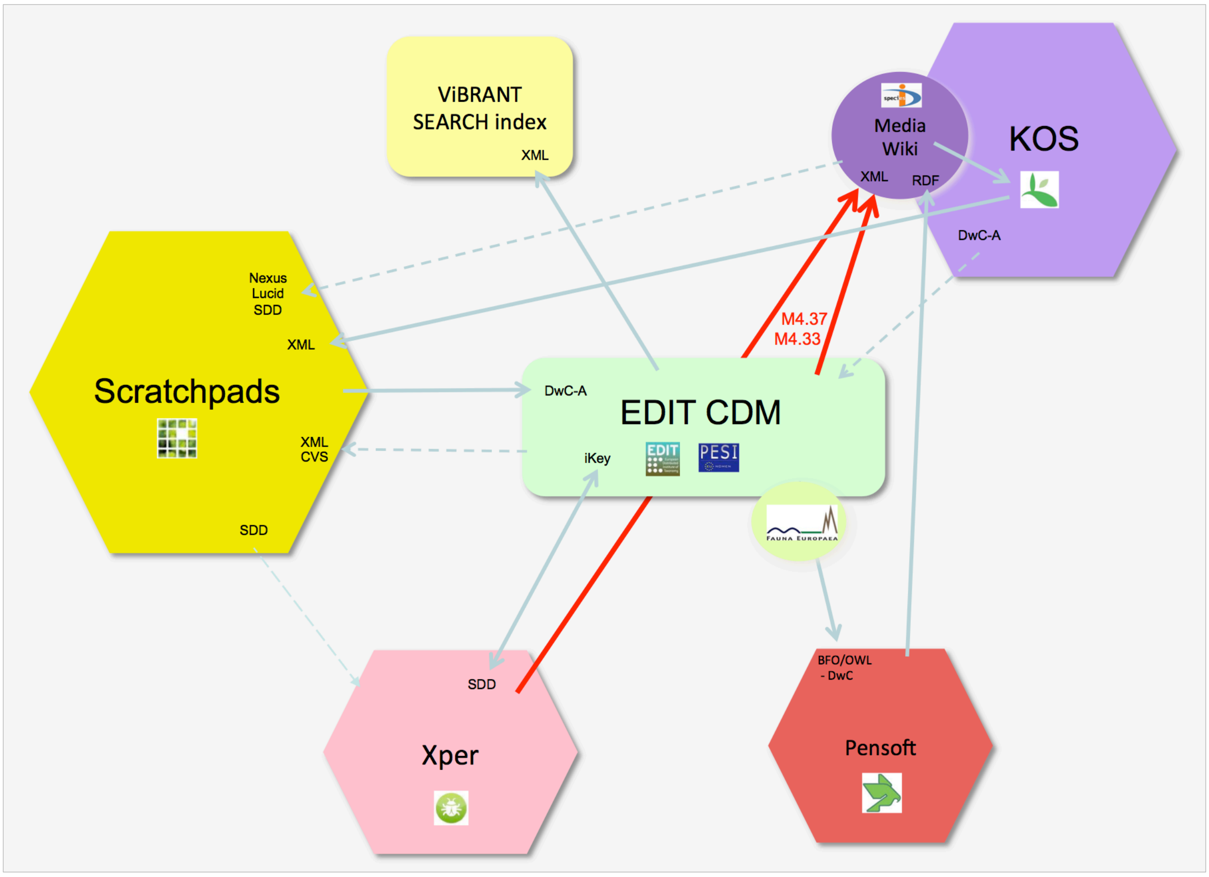

Figure 14: The position of the *Standard data interfaces and APIs* milestones within the ViBRANT architecture.

6 Global Names Architecture

6.1 Introduction

Taxon names provide key ontologies on connecting biodiversity data. The absence of an appropriate global name-reference system hinders an efficient and dynamic cross-referencing of taxon names, the functional re-use of biodiversity information and a single access to 'all names in use'. It also hinders the further development of a communal (virtual) research environment, supporting science as a community effort.

Global Names Architecture (GNA) would like to create more stability, compatibility and community in names resolution, by including an objective (nomenclatural) layer, stabilising the cross-referencing of taxon names by including advanced indexing services for name discovery, recognition and re-use, and by supporting the development and publishing of taxonomic authority files. ViBRANT contributes to these ambitions by supporting further setups and developments of a collaborative taxonomic roadmap for Europe, as necessary components of Research Data e-Infrastructures in the Framework for Action of H2020[53] [54] [55] [56].

---

53 **M4.43** – http://vbrant.eu/content/m443-global-names-europe

54 *BMC Ecology* 2013, **13**:16 – http://www.biomedcentral.com/1472-6785/13/16/abstract

55 **BIH**orizons – http://h2020.myspecies.info/

56 **BIH**2013 – http://conference.lifewatch.unisalento.it/index.php/EBIC/BIH2013

6.2 Development and publishing of taxonomic reference lists

Fauna Europaea[57] is a unique taxonomic (standard) reference, serving as a scientific baseline for a wide range of users, providing an index of scientific names (including important synonyms) of all living European land and freshwater animals, their geographical distribution at country level (up to Ural, excluding Caucasus region), and some additional optional information. As part of the ViBRANT project several changes and enhancements have been taken care of in Fauna Europaea, including the migration of the Fauna Europaea services from the University of Amsterdam (UvA) to the Museum für Naturkunde (MfN), the release of several new versions, and the publication of a sequence of data papers using the Pensoft publishing platform[58].

These activities contribute to several objectives in ViBRANT workpackage 4; it shows the interoperability between the Cybertaxonomy platform (the supposed future host of Fauna Europaea) and the Pensoft publishing platform (Fig. 15), it highlights the relevance of taxonomic authority files (as controlled vocabularies) for biodiversity research, it shows the potential of innovative publishing tools (like the Biodiversity Data Journal) on serving the demands of virtual biodiversity communities and it contributes to an integrated access of Scratchpads and CDM data (Fauna Europaea –as a PESI component– is available for use within the ViBRANT Index) for common use and outreach.

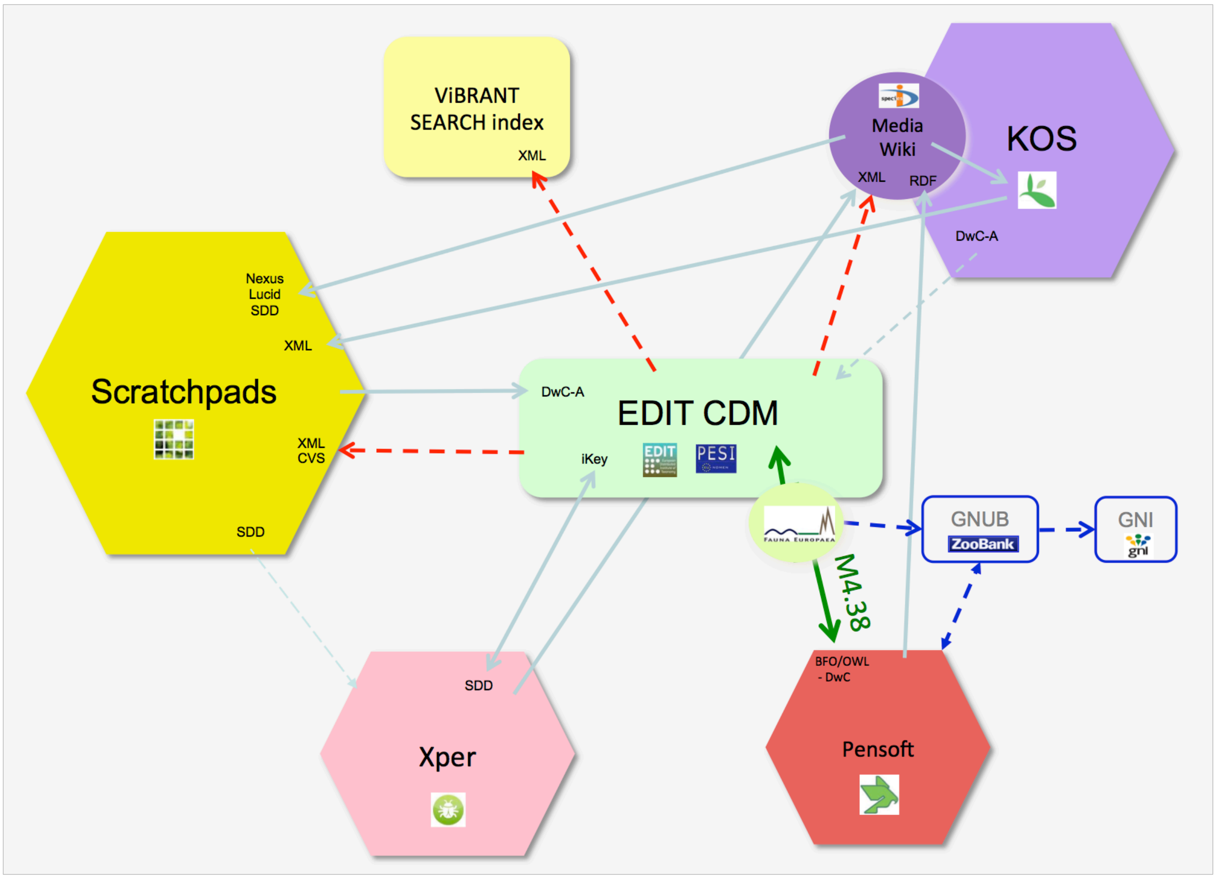

Figure 15: The position of *Fauna Europaea* milestone within the ViBRANT architecture, prospective data flows (red lines) and GNA outreach (blue lines).

57 **Fauna Europa** – http://www.faunaeur.org

58 **M4.38** – http://vbrant.eu/content/m438-zookeys-special-issue-fauna-europaea-data-papers

On usage: Fauna Europaea covers about 240,000 taxon names, including 145,000 accepted (sub)species, brought together by a network of more then 400 specialists. The Fauna Europaea portal is a recognised website on taxonomic information considering the increasing web statistics (Table 1).

Total pageviews – 2013 (extrapolated)	c. 5 million
Total pageviews – 2012	4,317,894
Total pageviews – 2011	3,584,484
Total pageviews – 2010	3,306,436
Total visitors – 2013 (extrapolated)	c. 1 million
Total visitors – 2012	892,966
Total visitors – 2011	515,490
Total visitors – 2010	383.001
Yearly unique visitors – 2013 (extrapolated)	c. 800k
Yearly unique visitors – 2012	703,420
Yearly unique visitors – 2011	333,402
Yearly unique visitors – 2010	209.655

Table 1: Fauna Europaea web statistics (period 2010 - 2013).

Of all 57 data papers (representing the main Fauna Europaea taxonomic sectors) prepared in the Pensoft Writing Tool[59] for publication in the Biodiversity Data Journal[60], five data papers are designed to be ready for publication at the end of the ViBRANT project lifetime, including the data papers on Helminths, Coleoptera 2, Nematoda, Diptera – Brachycera, and Hymenoptera – Apocrita (Fig. 16).

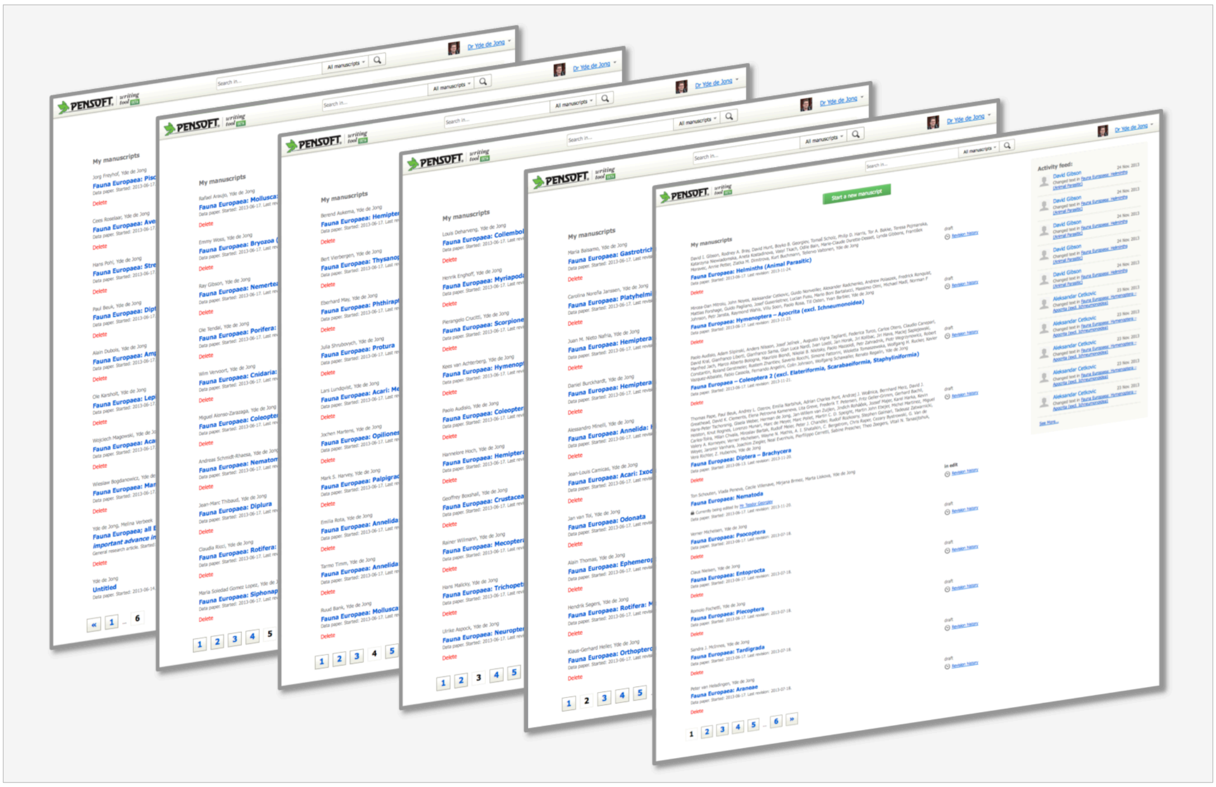

Figure 16: Draft Fauna Europaea data papers in preparation in the Pensoft Writing Tool.

59 **Pensoft Writing Tool** – pwt.pensoft.net

60 **Biodiversity Data Journal** – http://www.pensoft.net/journals/bdj
