# Supplementary material for: PESI - a taxonomic backbone for Europe
Source: Biodivers Data J. 2015 Sep 28;(3):e5848. doi: 10.3897/BDJ.3.e5848 (PMC4609752; doi:10.3897/BDJ.3.e5848)
Supplement: Supplementary material 30 — Taxonomic backbone databases integrated with EDIT platform and EU BON portal [EU BON MS121] [file biodiversity_data_journal-3-e5848-s030.pdf]

**Milestone MS121**

Version: 1
Date: 2014-07-23
Author: Andreas Kohlbecker
Document reference: MS121

# Taxonomic backbone databases integrated with EDIT platform and EU BON portal (M12/20)

STATUS: Final

| | |
|---|---|
| Project acronym: | EU BON |
| Project name: | EU BON: Building the European Biodiversity Observation Network |
| Call: | ENV.2012.6.2-2 |
| Grant agreement: | 308454 |
| Project Duration: | 01/12/2012 – 31.05.2017 (54 months) |
| Co-ordinator: | MfN, Museum für Naturkunde - Leibniz Institute for Research on Evolution and Biodiversity, Germany |
| Partners: | UTARTU, University of Tartu, Natural History Museum , Estonia<br>UEF, University of Eastern Finland, Digitisation Centre, Finland<br>GBIF, Global Biodiversity Information Facility, Denmark<br>UniLeeds, University of Leeds, School of Biology, UK<br>UFZ, Helmholtz Centre for Environmental Research, Germany<br>CSIC, The Spanish National Research Council, Doñana Biological Station, Spain<br>UCAM, University of Cambridge, Centre for Science and Policy, UK<br>CNRS-IMBE, Mediterranean Institute of marine and terrestrial Biodiversity and Ecology, France<br>Pensoft, Pensoft Publishers Ltd, Bulgaria<br>SGN, Senckenberg Gesellschaft für Naturforschung, Germany<br>VIZZUALITY, Vizzuality S.L., Spain<br>FIN, FishBase Information and Research Group, Inc., Philippines<br>HCMR, Hellenic Centre for Marine Research, Greece<br>NHM, The Natural History Museum, London<br>BGBM, Botanic Garden and Botanical Museum Berlin-Dahlem, Germany<br>UCPH, University of Copenhagen: Natural History Museum of Denmark, Denmark<br>RMCA, Royal Museum of Central Africa, Belgium<br>PLAZI, Plazi GmbH, Switzerland<br>GlueCAD, GlueCAD Ltd. – Engineering IT, Israel<br>IEEP, Institute for European Environmental Policy, UK<br>INPA, National Institute of Amazonian Research, Brazil<br>NRM, Swedish Museum of Natural History, Sweden<br>IBSAS, Slovak Academy of Sciences, Institute of Botany, Slovakia<br>EBCC-CTFC, Forest Technology Centre of Catalonia, Spain<br>NBIC, Norwegian Biodiversity Information Centre, Norway<br>FEM, Fondazione Edmund Mach, Italy<br>TerraData, TerraData environmetrics, Monterotondo Marittimo, Italy<br>EURAC, European Academy of Bozen/Bolzano, Italy<br>WCMC, UNEP World Conservation Monitoring Centre, UK |

This project has received funding from the European Union's Seventh Programme for research, technological development and demonstration under grant agreement No 308454.

# EU BON

EU BON: Building the European Biodiversity Observation Network
Project no. 308454

Large scale collaborative project

# MS121
# Taxonomic backbone databases integrated with EDIT platform and EU BON portal

| | |
|---|---|
| Milestone number | MS121 |
| Milestone name | Taxonomic backbone databases integrated with EDIT platform and EU BON portal |
| WP no. | 1 |
| Lead Beneficiary (full name and Acronym) | BGBM, Botanic Garden and Botanical Museum Berlin-Dahlem, Germany |
| Nature | Written report |
| Delivery date from Annex I (proj. month) | 30 November 2013 (M12) |
| Delivered | yes |
| Actual forecast delivery date | 2014-07-31 |
| Comments | Postponed to M20 |

| Name of the Authors | Name of the Partner | Logo of the Partner |
|---|---|---|
| Andreas Kohlbecker, Anton Güntsch | BGBM (main author) | BGBM |
| Florian Wetzel, Günther Korb | MfN (contribution to Fauna Europaea migration) | museum für naturkunde berlin |
| Karol Marhold, Matus Kempa | IBSAS (data provision and cleaning) | Institute of Botany SAS Bratislava |
| Kathleen Kesner-Reyes | FIN (data provision and cleaning) | FiN |
| Aaike De Wever | RBINS (data provision and cleaning) / MRAC | Africa TERVUREN |

In case the report consists of the delivery of materials (guidelines, manuscripts, etc)

| Delivery name | Delivery name | From Partner | To Partner |
|---|---|---|---|
| | | | |
| | | | |
| | | | |
| | | | |
| | | | |

## Summary of the Milestone

The main objective of this milestone is the improvement of merging processes of the Pan-European Species directories Infrastructure (PESI) into an instance of the EDIT Platform for Cybertaxonomy (hereafter called EDIT Platform ) running at the BGBM. In order to streamline this process and to guarantee the sustainability of the PESI backbone databases Fauna Europaea and the Euro+Med PlantBase have been migrated to the EDIT Platform. An “Advanced Validation Framework” has been specified which will allow more sophisticated validation checks to be run on PESI in the future. The PESI web service has been registered at GEOSS (http://www.earthobservations.org). The fitness of the PESI web services for use in the context of the EU BON taxonomic backbone has been tested; performance problems have been identified and solved, so that the PESI web service now can be a reliable primary source for the EU BON taxonomic backbone.

## Introduction

The EU BON taxonomic backbone will primarily connect and unify the checklists of the three data providers recommended by the INSPIRE directive[1]. These are the Pan-European Species directories Infrastructure (PESI, www.eu-nomen.eu), EUNIS, and Natura2000. PESI aims to provide an integrated view on nomenclatural and taxonomic information across all organism groups in Europe. At present, PESI integrates information from Euro+Med PlantBase, Fauna Europaea, ERMS, and Index Fungorum, databases which always have been managed in different heterogeneous systems. Since the last PESI merge in December 2012 the data models of the source databases have been changed so that an adaptation of the merging process has become necessary. This adaptation offers at the same time the opportunity to optimize and streamline the PESI merging process.

Other potential candidates to be connected to the EU BON taxonomic backbone are WoRMS (http://www.marinespecies.org) and the Catalogue of Life (CoL, http://www.catalogueoflife.org ).

The main objectives of this milestone are the improvement of merging processes of the PESI (www.eu-nomen.eu/pesi/) taxonomic database running on an instance of the EDIT Platform at the BGBM and to guarantee the sustainability of Fauna Europaea and the Euro+Med PlantBase, which are source databases of PESI. PESI will become a major source to the taxonomic backbone of EU BON once deployed and registered in the EU BON Portal. This divides into the following sub tasks:

- Full migration of Fauna Europaea and the Euro+Med PlantBase databases to the EDIT Platform for Cybertaxonomy.
- Manual check and data cleaning in the PESI source databases and reporting back detected problems to the project maintainers.
- Optimization and streamlining of the PESI backbone databases merging process (this is basically the PESI information flow).
- Improvement of the overall data quality in PESI by early detection of inconsistency problems in the source databases as well as in the merged PESI database. Automatic detection of inconsistencies by a “Advanced Validation Framework”
- Registration and hardening of PESI web services and deployment in the EU BON infrastructure (portal, workflows, etc.).

1 INSPIRE – Data Specification on Species Distribution, Draft Technical Guidelines, Recommendation 7. http://inspire.ec.europa.eu/documents/Data_Specifications/INSPIRE_DataSpecification_SD_v3.0rc3.pdf

# Progress towards objectives, achievements and current status

**Migration of Fauna Europaea into the EDIT Platform for Cybertaxonomy**
In the past Fauna Europaea (FaEu) was co-ordinated and maintained by the University of Amsterdam. Since this has become no longer possible the MfN has agreed to take over the co-ordination of FaEu and maintenance of the database. The subsequent move of the project to the MfN is not only organisational but also technical. To guarantee sustainability and the possibility to upload new data, to edit existing data and to simplify technical maintenance, it was necessary to migrate the entire project workflow to the EDIT Platform. The Fauna Europaea data has been successfully imported into the EDIT Platform and the system has been installed on a virtual server at the MfN hosting Fauna Europaea. This installation includes the CDM Server and service layer, the editorial system to access and modify the data, as well as the generic EDIT Platform Portal. In several online and face-to-face meetings between the MfN and the BGBM, Fauna Europaea workflows, data formats as well as rights and roles were analysed and specified. The implementation of Fauna Europaea specific functionalities started and will be continued and completed in the next phase of EU BON. Next steps will be to establish a working webportal and import functionalities for the database.

**Updating and data cleaning of PESI backbone database contents**
During the production of the past version of PESI import and merging of occurrence data was problematic and did produce incomplete and incorrect data. This situation has now been improved at various levels of the whole PESI process for the current version. The Euro+Med database has been updated, missing records have been added using corrected status values (native, introduced, cultivated) extracted from occurrence strings in MedChecklist. The occurrences in MedChecklist are stored as a string in specific format containing area abbreviations. For each missing area, a single row had to be added with single occurrence area in Euro+Med PlantBase format. Also status checks were performed on existing records. In total, more than 40000 occurrence records have been corrected. In addition more than 100000 common name records have been completed with missing information. Further improvements at the level of the source databases include the following. The global fish taxonomy maintained by FIN (FISHBASE INFORMATION & RESEARCH GROUP INC) has been updated with new published species and revisions and compared with the Catalogue of Fishes (species: 530 encoded / 3250 updated; synonyms: 1675 / 2590). An update of the list for WoRMS (World Register of Marine Species) is being prepared to be sent to VLIZ (Flanders Marine Institute, Belgium) in June.
An update for the European waters (ERMS) is being prepared for end of July. An Update for the FADA list was delivered to RBINS (16 May 2013). Corrupt data in ERMS has been found and resolved and reported back to VLIZ. In Fauna Europaea problematic data has been found and resolved for one taxon.

**Specification of an Advanced Validation Framework**
In preparation for the next step forward in terms of improving the data quality in PESI a sophisticated rule based data validation mechanism will be implemented in the EDIT Platform. This “Advanced Validation Framework” will continuously perform data quality and integrity checks on the PESI, Euro+Med PlantBase and Fauna-Europaea databases. The final specification for this system has been adopted and the system is presently implemented by the BGBM in cooperation with ETI/Naturalis (www.eti.uva.nl/about/index.html) as open source software. In its first version it will consist of 10 selected integrity rules based on a joint EDIT/PESI data quality specification list (see http://dev.e-taxonomy.eu/trac/wiki/IntegrityRulesEditPESI). Further rules will be implemented successively during EU BON project and based on requirements set by the project.

**Preparing the final 2015 merge of the PESI database**
The contributing European Checklists (Euro+Med PlantBase, Fauna Europaea, ERMS, Index Fungorum) have been contacted and exports of their data have been received. Content and structural changes have been checked and an initial trial import into the EDIT Platform has been performed (see also chapter ‘Updating and data cleaning of PESI backbone database contents’ for further details).

The import and merging process has been adapted to support the concept of building tree -indexes for hierarchies which have been recently introduced into the EDIT Platform components as a performance improvement. The final PESI merge into the EDIT Platform is planned for July 2015. The result will be exported into an agreed data warehouse structure and published by VLIZ via the PESI portal and service layer.

**Registration of PESI web services for EU BON**

Since the EU BON Portal (EBP) will only become available in a later phase of the EU BON project, it is not yet possible to register the PESI web services at EBP now. During one of the periodic EU BON WP1/WP2 calls it has been decided that the services should provisionally be registered at the BiodiversityCatalogue and at the GEOSS CSR Component Registry System.

The PESI web services have been contributed to the CSR and are registered with the following resource details:

> Resource Id: urn: geoss:csr:resource:urn:uuid:be5e6bea-e3f6-8adc-9f28-3536058e4bbd
> Resource Name: PESINameService
> Resource Url:
>
> http://geossregistries.info/geosspub/resource_details_ns.jsp?compId=urn:geoss:csr:resource:urn:uuid:be5e6bea-e3f6-8adc-9f28-3536058e4bbd

The PESI web services have found to be already registered at the BiodiversityCatalogue (https://www.biodiversitycatalogue.org/services/21), so it was not necessary to take any action in this case.

**Performance and stress testing of the PESI web services**

The PESI web services maintained and hosted by VLIZ will be the primary source of taxonomic information for EU BON, therefore it must be assured that the service endpoints are robust enough to handle frequent and parallel requests from the EU-BON taxonomic backbone. The taxonomic backbone will send different requests to the PESI web service making use of four different SOAP actions:

- search for taxa by an exact scientific name string (SOAP action: getPESIRecords, like=false)
- search for taxa which start with a scientific name string, also covers search with wildcard characters (SOAP action: getPESIRecords, like=true)
- fuzzy search for taxa by a scientific name (SOAP action: matchTaxon)
- search for taxa by an vernacular name (SOAP action: getPESIRecordsByVernacular)

Two assertions have been tested for each of the responses

- HTTP status header is 200
- Response body contains the string <item xsi:type="tns:PESIRecord"> (makes sure that records are returned)

Two fixed lists of names have been used to generate the test requests, one for scientific names and another for vernacular names whereas the tests have been run with the top 500 entries of the respective list.

- http://dev.e-taxonomy.eu/gitweb/jmeter-tests.git/blob/HEAD:/pesi/PESI-scientific-names.txt
- http://dev.e-taxonomy.eu/gitweb/jmeter-tests.git/blob/HEAD:/pesi/PESI-vernacular-names.txt

The Tests have been implemented on base of the JMeter test framework (https://jmeter.apache.org/). The complete test plans are available from the BGBM git repository: http://dev.e-taxonomy.eu/gitweb/jmeter-tests.git. (This repository also contains additional tests for EDIT Platform driven CoL name catalogue services which have been developed in the context of the BioVel project.)

Three test scenarios have been run so far with this setup. In each scenario 500 different SOAP requests for each of the above named actions have been send to the service. During the first scenario

all requests have been run sequentially one after another. The other scenarios made use of multiple threads (5 and 20) to send parallel requests to the service. The tables below show the results of these tests from the first test run:

| Label | # Samples | Average | Min | Max | Std. Dev. | Error % | Throughput | KB/sec | Avg. Bytes |
|---|---|---|---|---|---|---|---|---|---|
| getPESIRecords [SOAP] | 500 | 2238 | 130 | 11158 | 1147.43 | 0.00% | 26.5/min | 6.04 | 13994.5 |
| matchTaxon [SOAP] | 500 | 2167 | 216 | 13627 | 1300.78 | 4.40% | 27.4/min | 0.85 | 1910.9 |
| getPESIRecordsByVernacular [SOAP] | 500 | 2227 | 95 | 18238 | 1242.23 | 0.80% | 26.7/min | 1.07 | 2471.5 |
| TOTAL | 1500 | 2211 | 95 | 18238 | 1232.16 | 1.73% | 26.9/min | 2.68 | 6125.6 |

*Table 1: Summary report - 500 requests run in a single thread. The average response time for a single request was 2.2 seconds.*

| Label | # Samples | Average | Min | Max | Std. Dev. | Error % | Throughput | KB/sec | Avg. Bytes |
|---|---|---|---|---|---|---|---|---|---|
| getPESIRecords [SOAP] | 504 | 2732 | 143 | 6740 | 1199.44 | 0.00% | 1.8/sec | 24.41 | 13942.9 |
| matchTaxon [SOAP] | 504 | 3538 | 257 | 15013 | 2237.37 | 4.37% | 1.4/sec | 2.59 | 1917.8 |
| getPESIRecordsByVernacular [SOAP] | 504 | 2899 | 173 | 5358 | 1183.68 | 0.79% | 1.7/sec | 4.09 | 2471.2 |
| TOTAL | 1512 | 3056 | 143 | 15013 | 1653.99 | 1.72% | 1.6/sec | 9.56 | 6110.6 |

*Table 2: Summary report - 500 requests run 5 parallel threads. The average response time for a single request was 3 seconds.*

| Label | # Samples | Average | Min | Max | Std. Dev. | Error % | Throughput | KB/sec | Avg. Bytes |
|---|---|---|---|---|---|---|---|---|---|
| getPESIRecords [SOAP] | 519 | 2081 | 133 | 5384 | 1074.97 | 0.00% | 9.0/sec | 124.40 | 14167.0 |
| matchTaxon [SOAP] | 519 | 2448 | 206 | 12282 | 1802.46 | 4.05% | 7.1/sec | 13.31 | 1928.4 |
| getPESIRecordsByVernacular [SOAP] | 519 | 2334 | 300 | 4710 | 911.83 | 0.77% | 8.0/sec | 19.25 | 2472.5 |
| TOTAL | 1557 | 2288 | 133 | 12282 | 1329.97 | 1.61% | 7.9/sec | 47.93 | 6189.3 |

*Table 1: Summary report - 500 requests run in 20 parallel threads. The average response time for a single request was 2.3 seconds.*

The results of this first run clearly showed that the PESI SOAP web service is scaling well under the chosen test conditions. The error rate shown in the summary report tables stayed constant; the increased load by parallel requests is not causing any response drop outs or errors. Even though the average response time stays more or less constant in all three test scenarios (2.1 to 3 seconds) it is to slow for to be used as source for the EU BON taxonomic backbone. This response time would directly affect the responsiveness of EU BON taxonomic backbone, since the backbone cannot respond faster than its primary source of data. These findings haven been reported to VLIZ together with the recommendation to improve the PESI web service to improve the response time to less than one second. VLIZ was able to solve this problem and to improve the performance of the web services. Another run of the JMeter performance tests after the bug fix showed that the PESI web services are now about six times faster than before. Figure 1 shows the response time for a single thread with 500 requests over time. Table 4 shows once more the average response time for all request types with 0.3 seconds response time. Figure xxx, xxx, and table xx and xx show the corresponding values for parallel processing of requests.

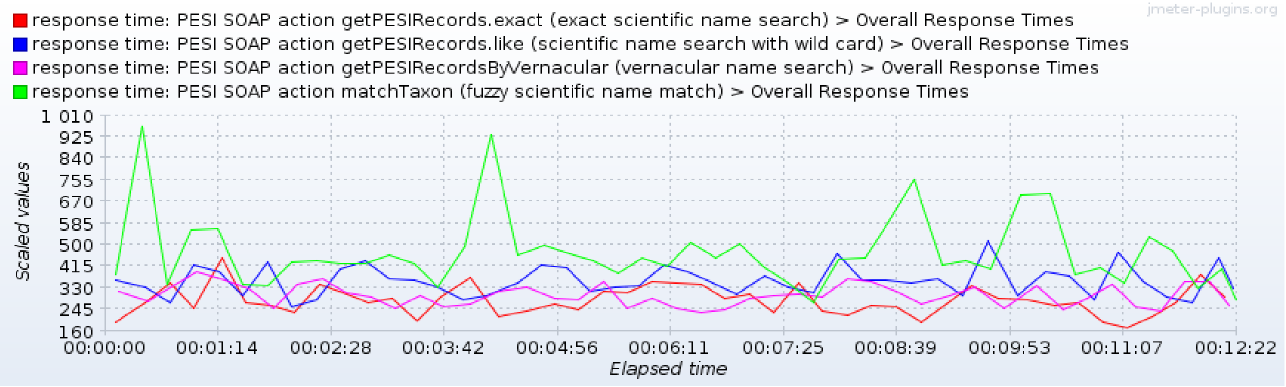


*Figure 1: Response time graph 1 thread 500 requests*

| Label | # Samples | Average | Min | Max | Std. Dev. | Error % | Throughput | KB/sec | Avg. Bytes |
|---|---|---|---|---|---|---|---|---|---|
| getPESIRecords.exact[SOAP] | 500 | 276 | 120 | 1713 | 161.20 | 2.80% | 40.4/min | 1.30 | 1976.8 |
| getPESIRecords.like [SOAP] | 500 | 347 | 129 | 2118 | 197.43 | 0.00% | 40.4/min | 10.07 | 15310.1 |
| matchTaxon [SOAP] | 500 | 462 | 200 | 4940 | 406.00 | 3.80% | 40.4/min | 1.27 | 1934.4 |
| getPESIRecordsByVernacular [SOAP] | 500 | 301 | 120 | 828 | 140.73 | 0.80% | 40.4/min | 1.63 | 2471.5 |
| TOTAL | 2000 | 347 | 120 | 4940 | 259.75 | 1.85% | 2.7/sec | 14.25 | 5423.2 |

*Table 2: Summary report 1 thread 500 requests*

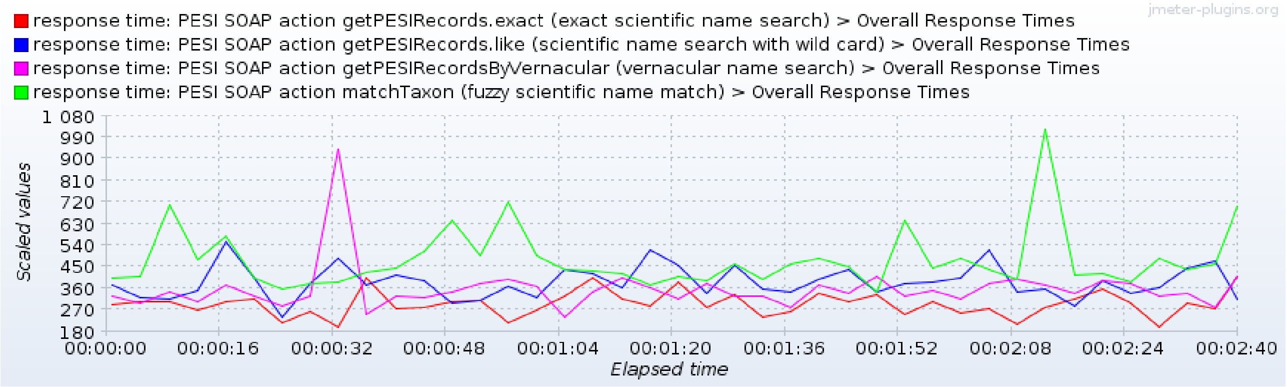


*Figure 2: Response time graph 5 threads 500 requests*

| Label | # Samples | Average | Min | Max | Std. Dev. | Error % | Throughput | KB/sec | Avg. Bytes |
|---|---|---|---|---|---|---|---|---|---|
| getPESIRecords.exact[SOAP] | 504 | 291 | 133 | 930 | 156.30 | 2.78% | 3.2/sec | 6.10 | 1978.0 |
| getPESIRecords.like [SOAP] | 504 | 385 | 150 | 1577 | 233.84 | 0.00% | 3.2/sec | 46.91 | 15227.1 |
| matchTaxon [SOAP] | 504 | 475 | 177 | 5617 | 418.30 | 3.77% | 3.2/sec | 5.97 | 1935.9 |
| getPESIRecordsByVernacular [SOAP] | 504 | 353 | 123 | 3346 | 219.57 | 0.79% | 3.2/sec | 7.62 | 2471.2 |
| TOTAL | 2016 | 376 | 123 | 5617 | 282.80 | 1.84% | 12.6/sec | 66.22 | 5403.1 |

*Table 3: Summary report 5 threads 500 requests*

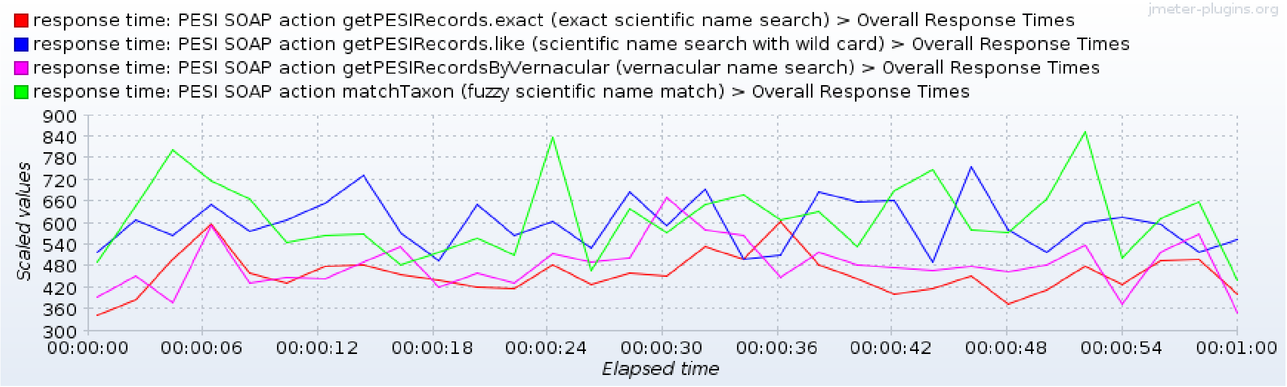


*Figure 3: Response time graph 20 threads 500 requests*

| Label | # Samples | Average | Min | Max | Std. Dev. | Error % | Throughput | KB/sec | Avg. Bytes |
|---|---|---|---|---|---|---|---|---|---|
| getPESIRecords.exact[SOAP] | 519 | 459 | 163 | 3953 | 242.05 | 2.70% | 8.6/sec | 16.78 | 1987.3 |
| getPESIRecords.like [SOAP] | 519 | 603 | 176 | 3377 | 330.63 | 0.00% | 8.6/sec | 128.23 | 15257.2 |
| matchTaxon [SOAP] | 519 | 620 | 226 | 5860 | 453.63 | 3.66% | 8.6/sec | 16.34 | 1946.0 |
| getPESIRecordsByVernacular [SOAP] | 519 | 486 | 143 | 1819 | 200.02 | 0.77% | 8.6/sec | 20.87 | 2472.5 |
| TOTAL | 2076 | 542 | 143 | 5860 | 329.24 | 1.78% | 33.7/sec | 178.40 | 5415.8 |

*Table 4: Summary report 20 threads 500 requests*

# Challenges and further/future developments

A major task for the next project phase is the completion of the Fauna Europaea migration to the EDIT Platform for Cybertaxonomy. After the final merge in 2015 the new version of PESI will be published to the data warehouse structure and will be made public by VLIZ via the Eu-Nomen portal and via the PESI web services.
Once the EBP (EU BON Portal) is released the PESI web services will also be registered directly for EU BON.
