# Supplementary material for: PESI - a taxonomic backbone for Europe
Source: Biodivers Data J. 2015 Sep 28;(3):e5848. doi: 10.3897/BDJ.3.e5848 (PMC4609752; doi:10.3897/BDJ.3.e5848)
Supplement: Supplementary material 31 — EU BON taxonomic backbone and services prototype integrated in EU BON portal [EU BON MS122] [file biodiversity_data_journal-3-e5848-s031.pdf]

MS122

Version: Final-1
Date: 2014-07-23
Author:Andreas Kohlbecker
Document reference: [MS122]

EU BON taxonomic backbone and services prototype integrated in EU BON portal

STATUS: FINAL

Project acronym:	EU BON
Project name:	EU BON: Building the European Biodiversity Observation Network
Call:	ENV.2012.6.2-2
Grant agreement:	308454
Project Duration:	01/12/2012 – 31.05.2017 (54 months)
Co-ordinator:	MfN, Museum für Naturkunde - Leibniz Institute for Research on Evolution and Biodiversity, Germany
Partners:	UTARTU, University of Tartu, Natural History Museum , Estonia<br>UEF, University of Eastern Finland, Digitisation Centre, Finland<br>GBIF, Global Biodiversity Information Facility, Denmark<br>UniLeeds, University of Leeds, School of Biology, UK<br>UFZ, Helmholtz Centre for Environmental Research, Germany<br>CSIC, The Spanish National Research Council, Doñana Biological Station, Spain<br>UCAM, University of Cambridge, Centre for Science and Policy, UK<br>CNRS-IMBE, Mediterranean Institute of marine and terrestrial Biodiversity and Ecology, France<br>Pensoft, Pensoft Publishers Ltd, Bulgaria<br>SGN, Senckenberg Gesellschaft für Naturforschung, Germany<br>VIZZUALITY, Vizzuality S.L., Spain<br>FIN, FishBase Information and Research Group, Inc., Philippines<br>HCMR, Hellenic Centre for Marine Research, Greece<br>NHM, The Natural History Museum, London<br>BGBM, Botanic Garden and Botanical Museum Berlin-Dahlem, Germany<br>UCPH, University of Copenhagen: Natural History Museum of Denmark, Denmark<br>RMCA, Royal Museum of Central Africa, Belgium<br>PLAZI, Plazi GmbH, Switzerland<br>GlueCAD, GlueCAD Ltd. – Engineering IT, Israel<br>IEEP, Institute for European Environmental Policy, UK<br>INPA, National Institute of Amazonian Research, Brazil<br>NRM, Swedish Museum of Natural History, Sweden<br>IBSAS, Slovak Academy of Sciences, Institute of Botany, Slovakia<br>EBCC-CTFC, Forest Technology Centre of Catalonia, Spain<br>NBIC, Norwegian Biodiversity Information Centre, Norway<br>FEM, Fondazione Edmund Mach, Italy<br>TerraData, TerraData environmetrics, Monterotondo Marittimo, Italy<br>EURAC, European Academy of Bozen/Bolzano, Italy<br>WCMC, UNEP World Conservation Monitoring Centre, UK

This project has received funding from the European Union’s Seventh Programme for research, technological development and demonstration under grant agreement No 308454.

EU BON

EU BON: Building the European Biodiversity Observation Network

Project no. 308454

Large scale collaborative project

MS122 – EU BON taxonomic backbone and services prototype integrated in EU BON portal

**Milestone number**	MS122
**Milestone name**	EU BON taxonomic backbone and services prototype integrated in EU BON portal
**WP no.**	1
**Lead Beneficiary (full name and Acronym)**	BGBM, Botanic Garden and Botanical Museum Berlin-Dahlem, Germany
**Nature**	Written report
**Delivery date from Annex I (proj. month)**	20
**Delivered**	[yes]
**Actual forecast delivery date**	[2014-07-31]
**Comments**	

Name of the Authors	Name of the Partner	Logo of the Partner
Andreas Kohlbecker	BGBM	
Antonio Garcia Camacho	CSIC	

In case the report consists of the delivery of materials (guidelines, manuscripts, etc)

Delivery name	Delivery name	From Partner	To Partner

Summary of the Milestone

A prototype of the EU BON taxonomic backbone has been developed and deployed to http://cybertaxonomy.eu/eu-bon/utis. It has been developed as a generic RESTful web service application for federated searches for taxon information named 'Unified Taxonomic Information Service' (UTIS). Currently the web services of the Pan-European Species directories Infrastructure (PESI, www.eu-nomen.eu) and of the CoL (Catalogue of Life) have been connected to the prototype. The EU BON taxonomic backbone is registered as a service at GEOSS Component and Service Registry (CSR) (http://geossregistries.info/) and at the BiodiversityCatalogue (https://www.biodiversitycatalogue.org/).

Introduction

The EU BON taxonomic backbone will primarily connect and unify the checklists of three data providers recommended by the INSPIRE directive[1]. It will also connect the Catalogue of Life (CoL, http://www.catalogueoflife.org) and a connection established to WoRMS is also being discussed (http://www.marinespecies.org). The EBP (EU-BON Portal) will use the taxonomic backbone as the main source of taxonomic information either in the context of its own search facility or to provide reference taxa with which other data can be associated. This second role is especially important in the context of reusability and comparability of data. More details on this interaction can be found in the MS251 - Specification for the EU BON portal which will be due in month 27.

Both aspects, to act as a search engine and to provide access to reference taxa must be covered by the EU BON taxonomic backbone. It thus will be able to perform a federated search on the connected check-list catalogues and will respond with unified taxonomic information hiding the heterogeneity of the connected catalogues.

The prototype of the taxonomic backbone initially connects to the PESI web service and to the Catalogue of Life (CoL, http://www.catalogueoflife.org) name catalogue service running on an instance of the EDIT Platform for Cybertaxonomy at the BGBM.

This milestone subdivides into the tasks:

- Development of a taxonomic backbone prototype for integration of taxonomic data into the overall EU BON framework. (Assure GEO BON and LifeWatch compliance)
- Registration of the taxonomic backbone Services

Progress towards objectives, achievements and current status

Development of a taxonomic backbone prototype

The EU-BON taxonomic backbone must be able to perform federated searches on multiple checklists. The individual result sets must be mapped into a unified data model. Finally the combined result set of all checklist providers to which the request has been sent should be returned to the client.
The INSPIRE directive requests that "*EU-Nomen is the preferred reference list to be used. If a taxon is listed in EU-Nomen, this reference must be used as first choice. If it is not listed in EU-Nomen, the second choice is EUNIS, if not in EUNIS, Natura2000 can be used."* A defined order of preference is therefore requested, the taxonomic backbone will thus retain this order in the set of results which are returned in response to a query. The results of the checklist with the highest preference are returned at first, followed by the results of the checklist with the next preference, and so on. The taxonomic backbone actually has been designed to allow flexible definition of the order of preference and of the checklist providers which should be taken into account during a federated search. The first prototype is able to connect to PESI (EU-Nomen) and to the CoL.
The results always contain the full taxonomic information as returned by the individual checklist together with information on the check-list which returned the data. Each result-set entry can thus always be tracked back to its source.

1 INSPIRE – Data Specification on Species Distribution, Draft Technical Guidelines, Recommendation 7. http://inspire.ec.europa.eu/documents/Data_Specifications/INSPIRE_DataSpecification_SD_v3.0rc3.pdf

The implementation of the EU-BON taxonomic backbone makes use of a federated search library, the drf-workflow, which has been developed for the BioVel Data Refinement Workflow (http://www.biovel.eu/workflows/available-workflow/data-refinement-wf). The source code of this library is available at GitHub (https://github.com/BioVeL/drf-workflow.git). BioVeL's aims to provide an e-infrastructure for analysis of biodiversity data. BioVel contributes to LifeWatch and it will eventually hand over the results of its work to LifeWatch for continued operation. BioVeL also expects to contribute to GEO BON's endeavour. Choosing the drf-workflow library as a federated search framework for the EU-BON taxonomic backbone assures compatibility to LifeWatch and to GEO BON.

The EU-BON taxonomic backbone prototype has been implemented as a generic service for federated searches in taxon information, the Unified Taxonomic Information Service (UTIS). The UTIS instance which is actually acting as the EU-BON taxonomic backbone prototype can be accessed via http://cybertaxonomy.eu/eu-bon/utis. When accessed with a web browser it offers a web page which links to the documentation of the web service api:

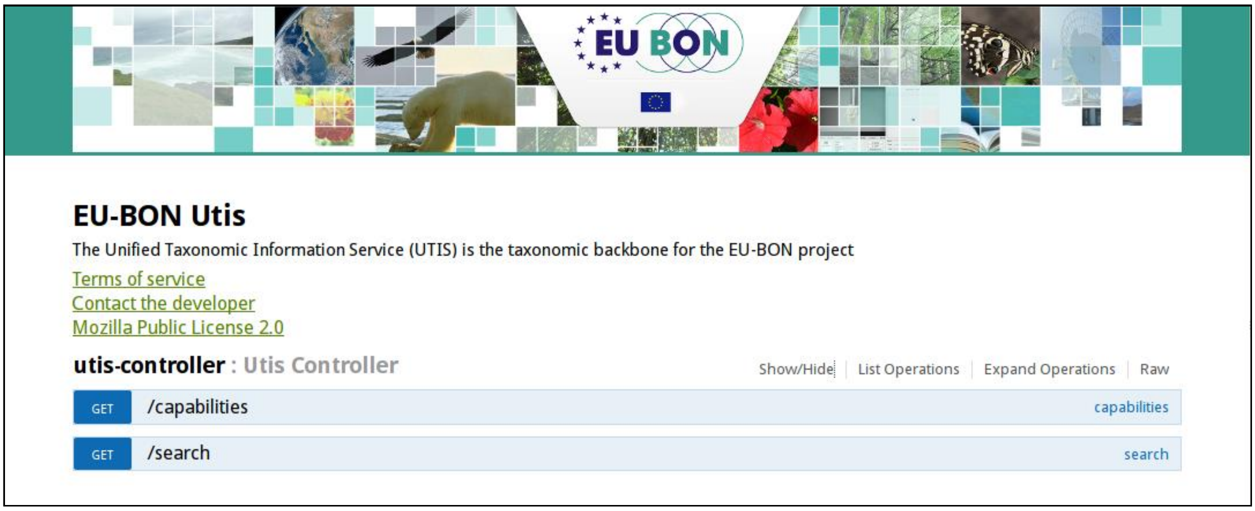

Figure 1: Front-end of the EU-BON taxonomic backbone

Technically the UTIS is a RESTful web-service which currently offers two operations:

- /capabilities : list the available service provider information and will in future also cover individual capabilities like for example fuzzy search and search by vernacular name.
- /search : execute a federated search for taxonomic information. The query string is a scientific name, like for example: "Bellis perennis" or "Prionus". This is an exact search and wildcard characters are not supported.

The response data is returned as JSON (http://json.org/) where each of the above listed operations returns data conforming to a different schema. The diagram below shows an overview on the tnr(Taxon Name Resolution)-schema, which is the return type of the search operation. For more detailed information on the schemata, please refer to the online REST service documentation (http://cybertaxonomy.eu/eu-bon/utis/doc.html#!/utis-controller). A new revision of the tnr-schema is planned for the next period of EU-BON in 2015. In this new version the terms used for class and field names will be more compliant to commonly accepted data exchange standards like DarwinCore, TCS and ABCD and thus should be intuitively comprehensible.

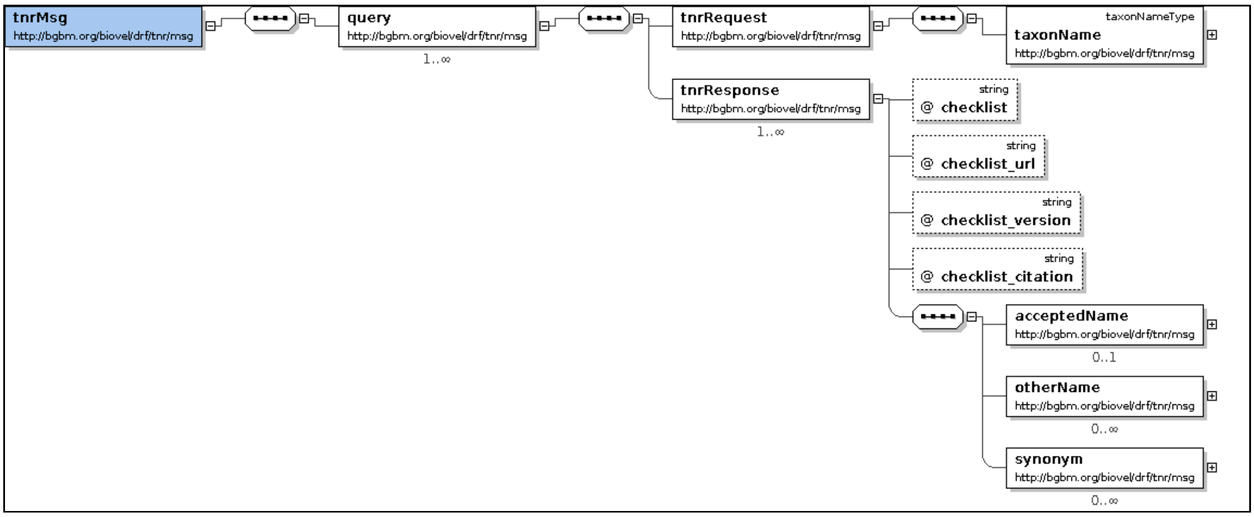

Figure 2: overview on the tnr-schema, the return type of the search operation

This prototype implementation currently only supports searching for taxa by an exact scientific name string. Other search modes using wild-cards, fuzzy search, searching by vernacular names are not yet supported. These features will be implemented in the next phase of EU BON.

Registration of the taxonomic backbone Services

The DoW demands that the taxonomic backbone is registered at the EU BON Portal (EBP), this however can only be done after the release of the EBP which is due for M5.3, therefore the registration has meanwhile been done at the GEOSS Component and Service Registry (CSR) (http://geossregistries.info) directly and also at the BiodiversityCatalogue (https://www.biodiversitycatalogue.org/):

- BiodiversityCatalogue: https://www.biodiversitycatalogue.org/services/79/
- GEOSS Component and Service Registry (CSR):
  Resource Id: urn:geoss:csr:resource:urn:uuid:45fc8fb3-d81b-99ee-d0a1-7845101ba42d
  Resource Name: EU-BON taxonomic backbone
  ResourceURL:
  http://geossregistries.info/geosspub/resource_details_ns.jsp?compId=urn:geoss:csr:resource:urn:uuid:45fc8fb3-d81b-99ee-d0a1-7845101ba42d

Challenges and further/future developments

A major task during the next period of EU-BON will be to add more checklist providers to the EU-BON taxonomic backbone with priority given to Natura2000 and EUNIS. The taxonomic information of EUNIS and Natura2000 will be available once this is accomplished. Another major step towards a fully functional taxonomic backbone will be the implementation of further search modes like: Search for taxa by vernacular names and a fuzzy search by scientific names and support for using wildcard characters in query strings. In this context the information returned by the capabilities operation must be extended to show the additional search types (vernacular name search, wildcard search, fuzzy search) supported by a certain service.

In addition to the implementation of new features it is also important to test if the connected services are fit for use in the context of the taxonomic backbone. Therefore performance testing of EUNIS, Natura2000 and of the EU-BON taxonomic backbone itself will be done. The CoL name catalogue has been already tested in the context of the BioVeL project and the PESI services have been tested for EU-BON, details on the test results regarding PESI can be obtained from the MS121 report. Finally a new revision of the tnr-schema will be developed and released (see above). Once the EBP has become available the EU BON taxonomic backbone will be registered there directly.
