# Supplementary material for: PESI - a taxonomic backbone for Europe
Source: Biodivers Data J. 2015 Sep 28;(3):e5848. doi: 10.3897/BDJ.3.e5848 (PMC4609752; doi:10.3897/BDJ.3.e5848)
Supplement: Supplementary material 32 — PESI Brochure [file biodiversity_data_journal-3-e5848-s032.pdf]

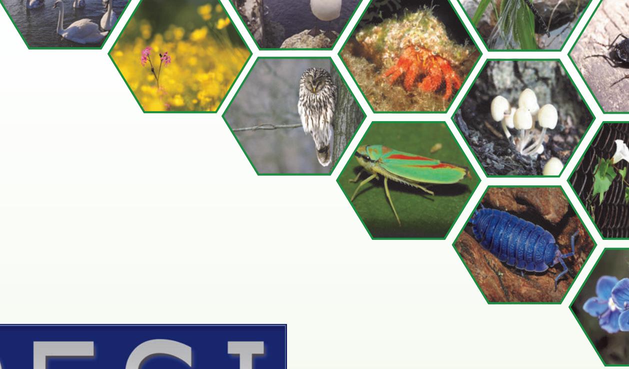

PESI

EU-NOMEN

A Pan-European
Species-directories Infrastructure

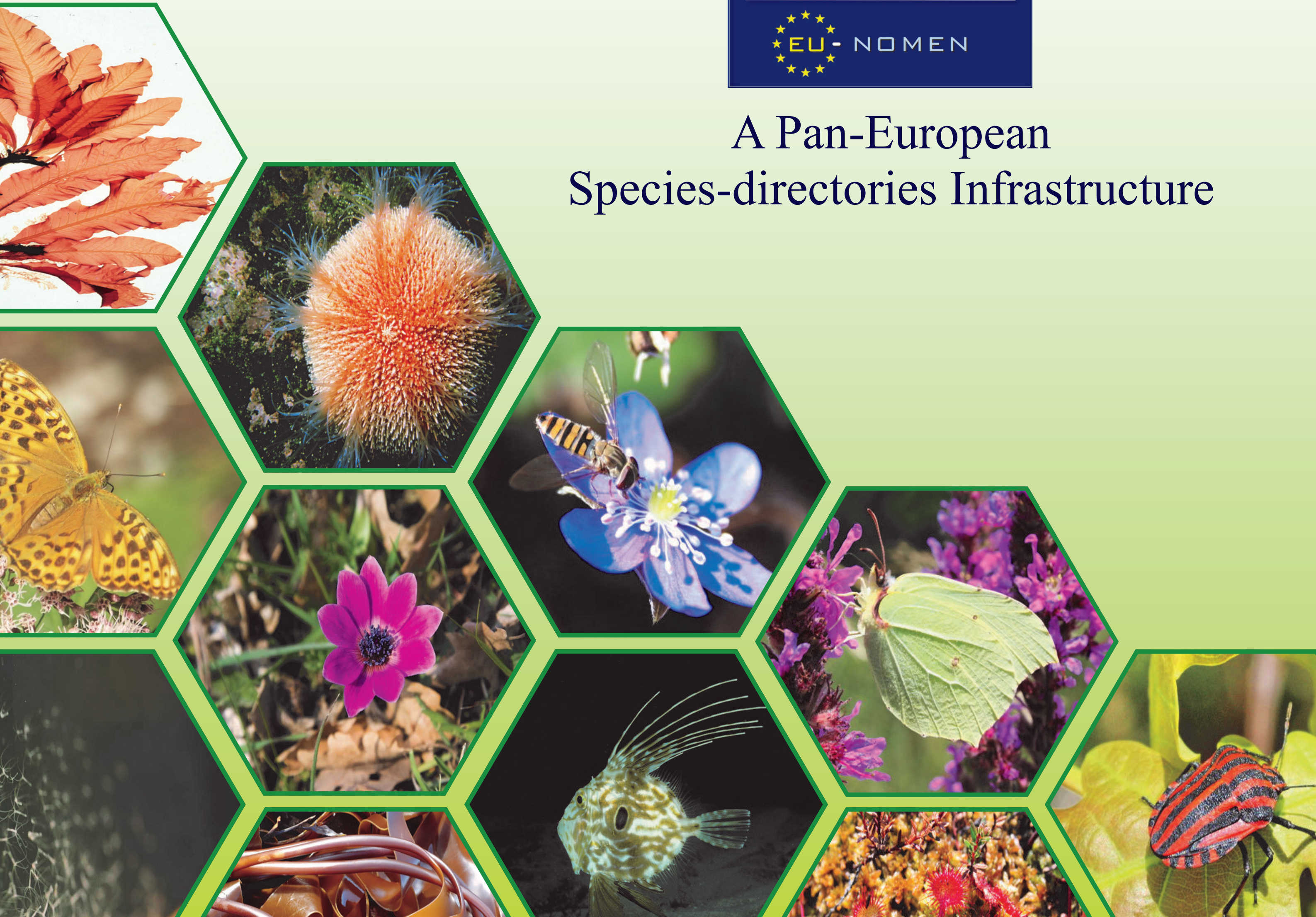

PESI

www.eu-nomen.eu/pesi

Photographs courtesy of Roy Kleukers, Marcin Penk, Eduard Stloukal, Davorin Tome, Mike Guiry, Fabio Rindi, Volodymyr Rizun, English Nature, ImagDOP, JG Marmelin

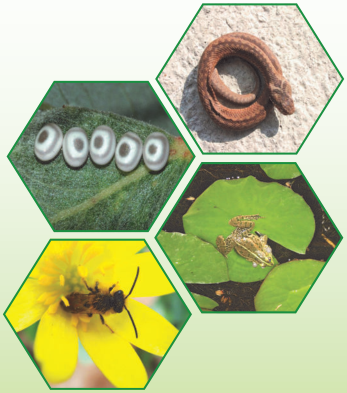

Rationale

Species names are critical data for biodiversity management and for most branches of biodiversity related applied and fundamental research. Correct species names are likewise important for pest and disease control, agriculture, aquaculture, forestry, fisheries, habitat protection, environmental control, and nature conservation.

Therefore the availability of high quality taxonomic name services, including valid species names and names relationships, functioning as authoritative taxonomic reference system, is essential for biological data management.

PESI is addressing weaknesses in taxonomic data infrastructures and is aiming for:

- ♦ standardisation of taxonomic data and database systems
- ♦ completion of high quality taxonomic data sets
- ♦ improving integration and accessibility of taxonomic reference systems

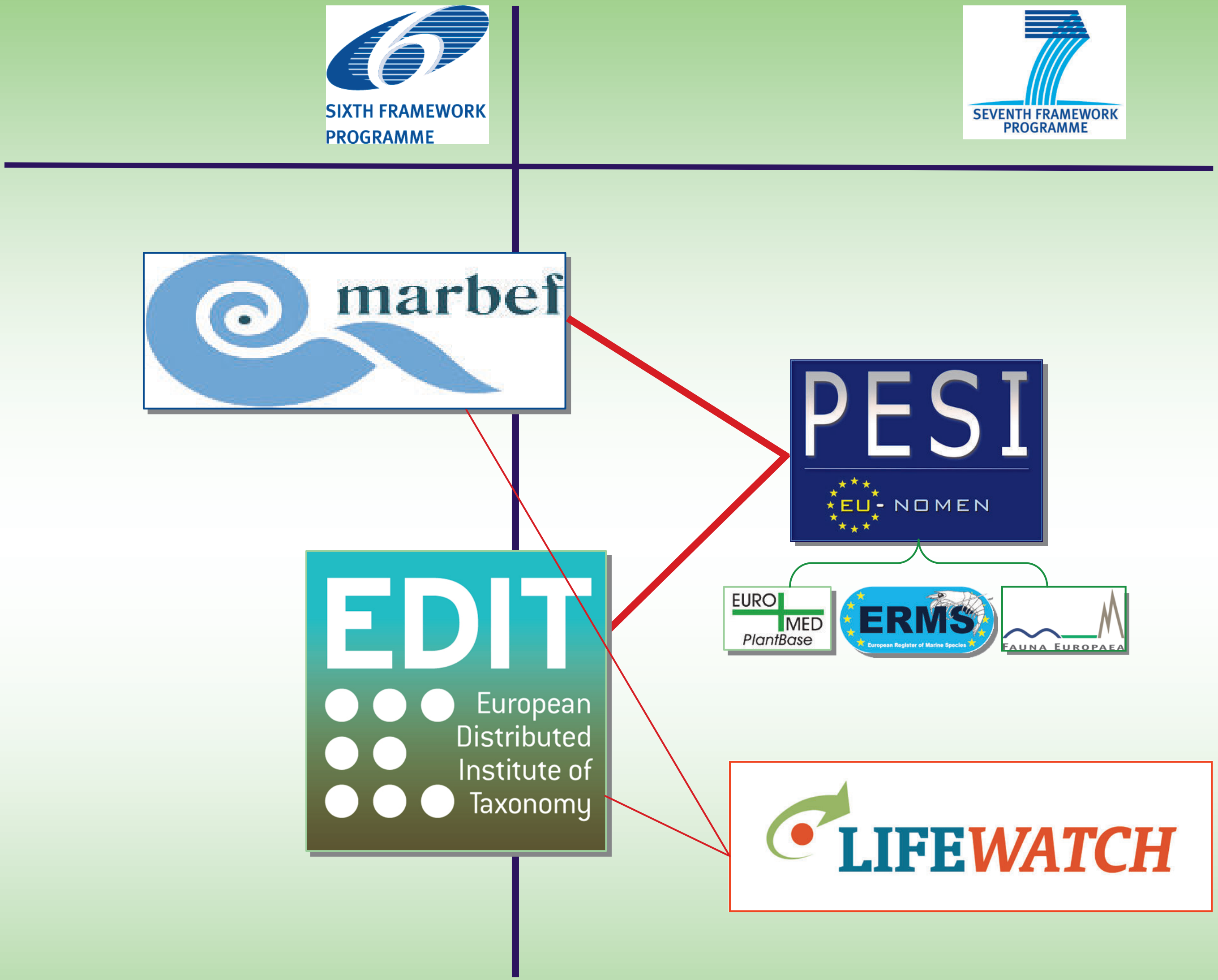
SIXTH FRAMEWORK PROGRAMME
SEVENTH FRAMEWORK PROGRAMME
marbef
PESI
EU - NOMEN
EURO MED PlantBase
ERMS
European Register of Marine Species
FAUNA EUROPAEA
EDIT
European Distributed Institute of Taxonomy
LIFEWATCH

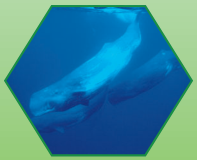

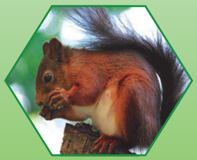

PESI Overview

PESI contributes to the **Global Names Architecture** development, co-ordinated by **GBIF** and **EoL,** including the implementation of the PESI taxonomic backbone as an authoritative reference for Europe, a common strategy on connecting Europe-based nomenclators to GNA (via the Global Names Usage Bank), a shared approach on the management and development of relevant standards and ontologies, and a joint program on data

PESI is scheduled to become the **'Euro-Hub'** of the **Catalogue of Life** (a shared **Species2000/ITIS** initiative)

PESI will be the European taxonomic backbone for **LifeWatch**

Cross Referencing

Nomenclators

PESI applies existing **Biodiversity Information Standards** (TDWG)

Tools and Services

Service to publishers
Taxon match tool
Expert database

EDIT - Platform for Cybertaxonomy

PESI plays an important role in the internationalisation of EDIT's Cybertaxonomy Platform

PESI contributes to the availability of a global unified cross-reference system for species names and associated information, by developing taxonomic standards, integrating European databases, international collaboration, and facilitating the efforts of taxonomic experts.

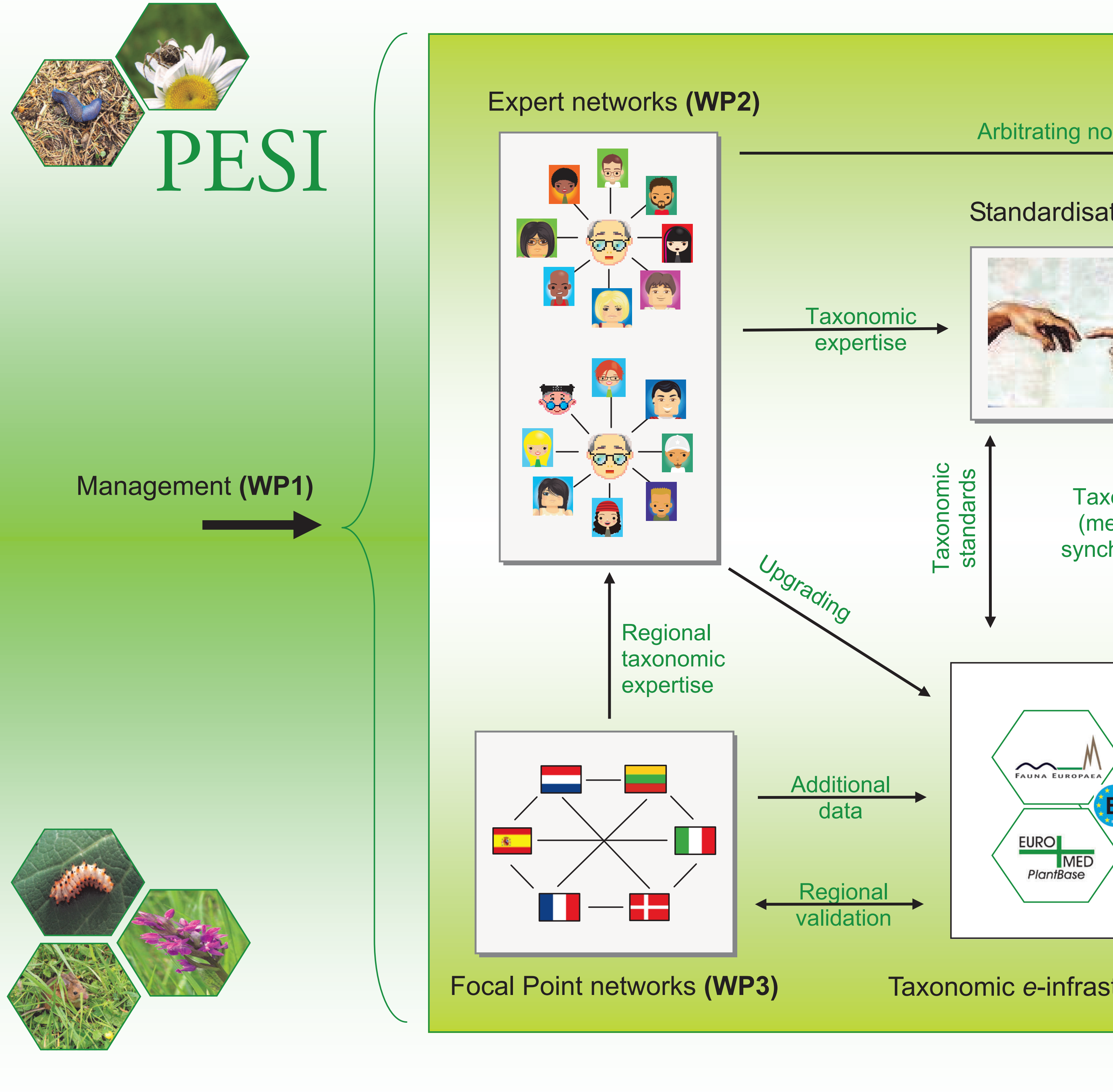

PESI
Management (WP1)
Expert networks (WP2)
Arbitrating no
Standardisat
Taxonomic
expertise
Taxonomic
standards
Upgrading
Regional
taxonomic
expertise
Additional
data
Regional
validation
FAUNA EUROPAEA
EURO
MED
PlantBase
Focal Point networks (WP3)
Taxonomic *e*-infras

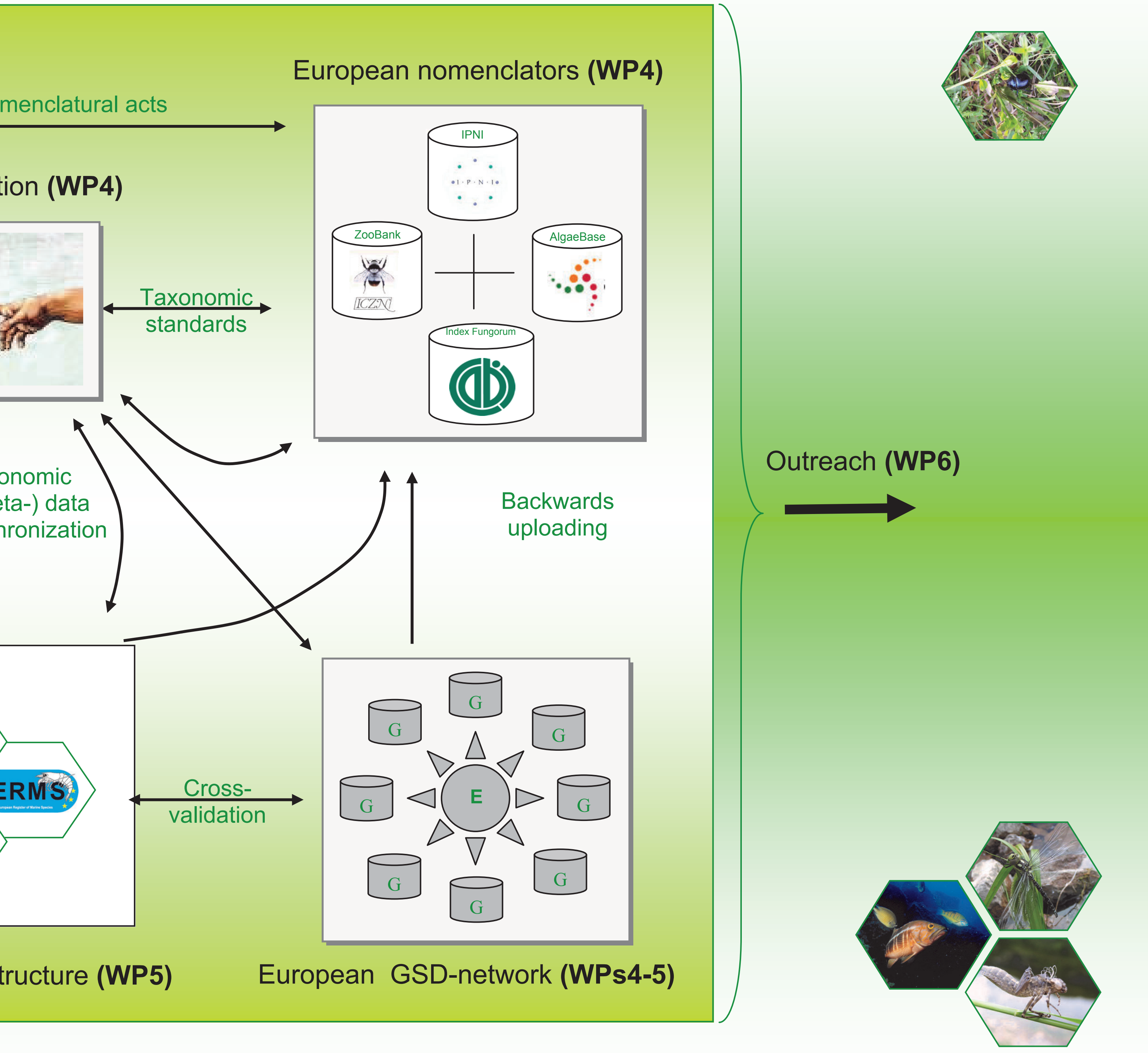

European nomenclators (WP4)
menclatural acts
IPNI
ZooBank
AlgaeBase
Index Fungorum
tion (WP4)
Taxonomic standards
onomic
eta-) data
hronization
Backwards uploading
Outreach (WP6)
Cross-validation
E
G
tructure (WP5)
European GSD-network (WPs4-5)

Enhancing the quality and reliability of information.

The foundation of biodiversity research is correctly identifying and naming species.

Although there has been a standard approach to naming species for the last 250 years, there has been no inventory of what species have been described. This results in multiple descriptions of the same species and a continuous effort to unravel confusion regarding species names.

"Probably one fifth of all recently described species names are synonyms" (Bouchet 2006)

There is an obvious need for standardisation…..

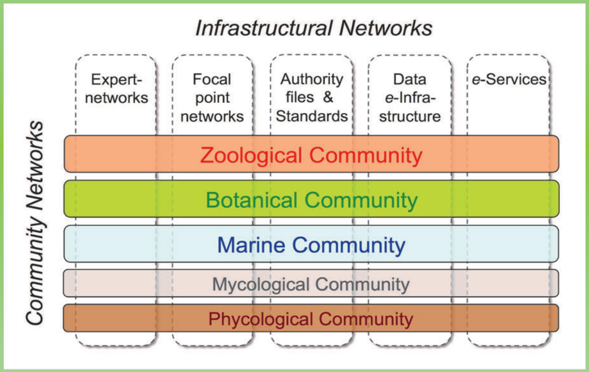

PESI is integrating the infrastructural components of five major community networks (horizontal) on taxonomic indexing and their respective knowledge infrastructures, i.e. marine life, terrestrial plants, fungi and animals.

Taxon registers are a standard reference for biodiversity training, research and management in Europe. In most cases these lists are, and will be compiled by experts in the various taxonomic groups.

PESI is not only integrating the taxon registers of ERMS, Fauna Europaea and Euro+Med PlantBase, but also expanding these networks by linking with Global Species Databases such as the World Register of Marine Species, Index Fungorum for fungi, and through increasing the number of 'Focal Points' in Europe and neighbouring countries.

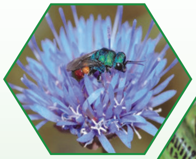

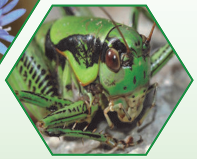

The Focal Point (FP) Network

- PESI Focal Points are national representatives for biodiversity and taxonomic research .
- This Focal Point network is crucial for the information exchange and data standards for the Pan-European (sensu latu) taxonomic infrastructure, forming the communication channel between the local level knowledge resources and the Pan-European integrated Taxonomic community network.
- The data contribution, leading to the Portal data-enrichment include: vernacular names in all European languages (sensu latu), species occurrence, cross-validation of species names, legal instruments and local expertise (e.g. experts, journals, societies, collections).

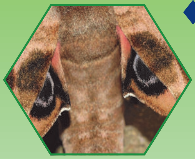

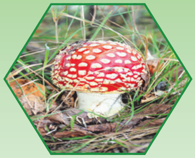

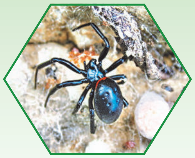

- FPs promote taxonomic standards throughout Europe. This is of direct importance for both European and national government bodies concerned with the implementation of legislation which refers to species and habitats.

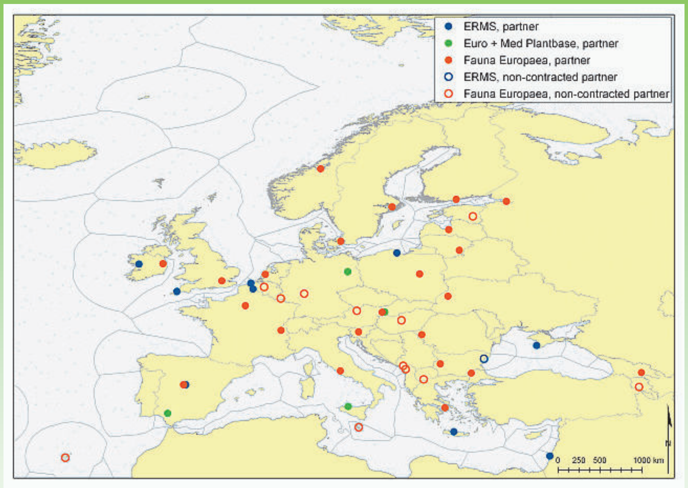

There are 45 Focal Points located in 25 countries.
Coordinate system: GCS ETRS 1989 or ETRS89

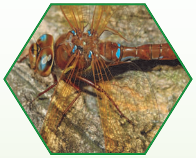

What to expect in the near future?

The Expert Network

Currently PESI has 11 new non contracted focal points participating in the Focal Point work programme (e.g. Albania, Armenia, Hungary, Malta, Ukraine). A further expansion of the geographic European networks of experts to cover the entire western Palaearctic biogeographic region is foreseen (see map below).

An expansion of EDIT's expert DataBase at www.EditExpertNet.org to include the expert communities within the PESI network.

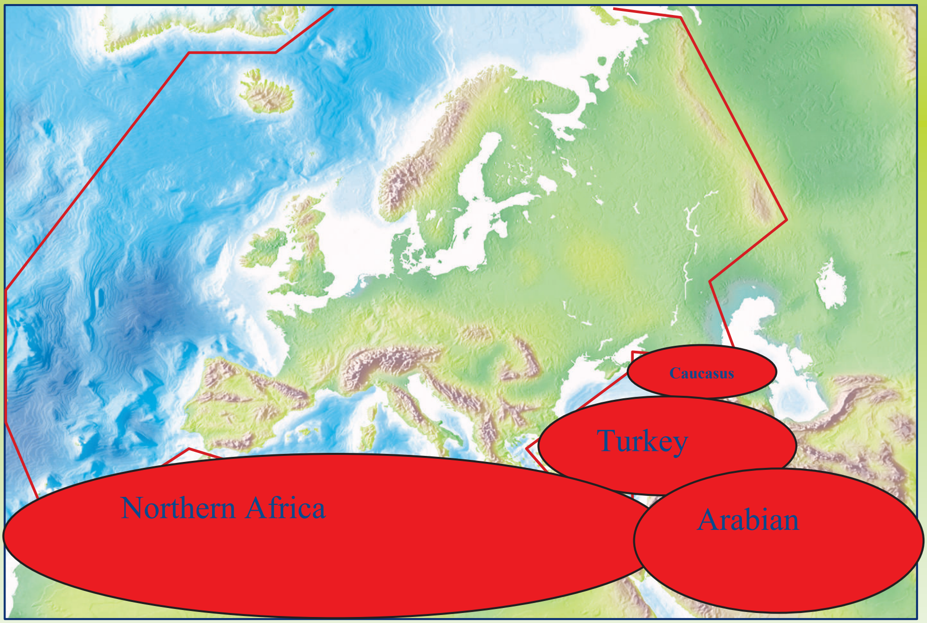

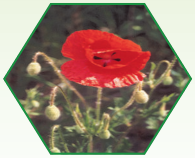

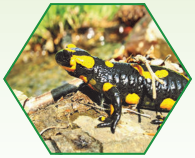

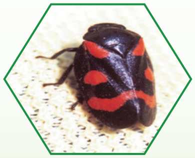

Data Integration

In co-operation with EDIT, PESI is presently defining a strategy for assigning persistent Globally Unique Identifiers (GUIDs) to names and taxa networked by PESI. The GUIDs together with services provided by WP6 will build a solid ground for the integration of PESI into the emerging worldwide biodiversity informatics infrastructures. GUIDs are also an important component of the versioning and archiving strategy being developed.

Integrated PESI data portal

The PESI portal is an interactive portal which will allow users to search not only by scientific names but also by common names of species (in most European languages) and higher taxonomic levels (e.g. *Thunnus thynnus*, blue-fin tuna, fish), browse down the classification tree and look up the conservation status of species and occurrences per country or sea region.

The portal will be a valuable tool that will help users in their research, teaching, and management of environmental information. (online May 2010 at http://www.eu-nomen.eu/portal).

Validation tool

PESI has implemented the 2nd version of the TAXAMATCH tool written by Tony Rees, CSIRO. This tool allows cross-checking long species lists against the PESI database.

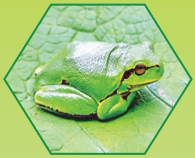

Project

PESI

www.eu-nomen.eu/pesi

a new initiative, funded by the
European Union under the Framework 7
Capacities Work Programme:
Research Infrastructures (RI-223806)
May 2008 –2011

Coordinator: Yde de Jong
University of Amsterdam

info@eu-nomen.eu

40 partner organisations
from 26 countries.

Partners

Universiteit van Amsterdam (NL)
University of Copenhagen (DK)
Trakya University (TR)
Natural History Museum (GB)
Flanders Marine Institute (BE)
Cab International (GB)
Natural History Museum (FR)
Royal Botanical Gardens (GB)
Society of the Management of Electronic Biodiversity Data (IE)
International Commission for Zoological Nomenclature (GB)
Botanic Garden and Botanical Museum Berlin-Dahlem, Freie Universität Berlin (DE)
Ecological Consultancy Services Ltd. (IE)
Università degli studi di Palermo (IT)
Slovak Academy of Sciences (SK)
National University Athens (GR)
Museum of Natural History (NL)
University of Helsinki (FI)
University of Sevilla (ES)
Vilnius University (LT)
AlgaeBase (IE)

Comitato Scientifico per la Fauna d'Italia(IT)
Museum of Natural History (SE)
Comenius University in Bratislava (SK)
University of Science & Technology (NO)
National Academy of Sciences (UA)
Polish Academy of Sciences (PL)
Swiss Systematics Society (CH)
Ilia Chavchavadze State University (GE)
Consejo Superior de Investigaciones Científicas (ES)
Israel Oceanographic & Limnological Research Ltd. (IL)
Polish Academy of Sciences Institute of Oceanology (PL)
Museum of Natural History (BG)
Asociatia Mynature (RO)
University of Latvia (LV)
Hellenic Centre for Marine Research (GR)
Université des Sciences et Technologies de Lille (FR)
National Institute of Biology (SL)
Russian Academy of Sciences (RU)
National Academy of Sciences (UA)

SEVENTH FRAMEWORK PROGRAMME
