# Supplementary material for: PESI - a taxonomic backbone for Europe
Source: Biodivers Data J. 2015 Sep 28;(3):e5848. doi: 10.3897/BDJ.3.e5848 (PMC4609752; doi:10.3897/BDJ.3.e5848)

Further highlights

Through its broad dissemination model PESI expands the network of end-users adopting the pan-European validated checklists as their taxonomic reference points.

PESI provides species occurrence details at the country level.

Co-development of a common policy to manage prioritised taxa under European legislation, by taxonomic standardising and disseminating the species data with deep-links to special features through the PESI Portal.

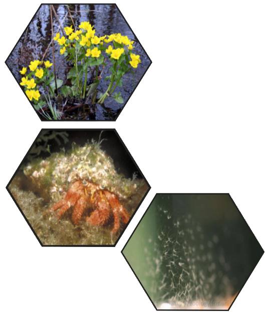

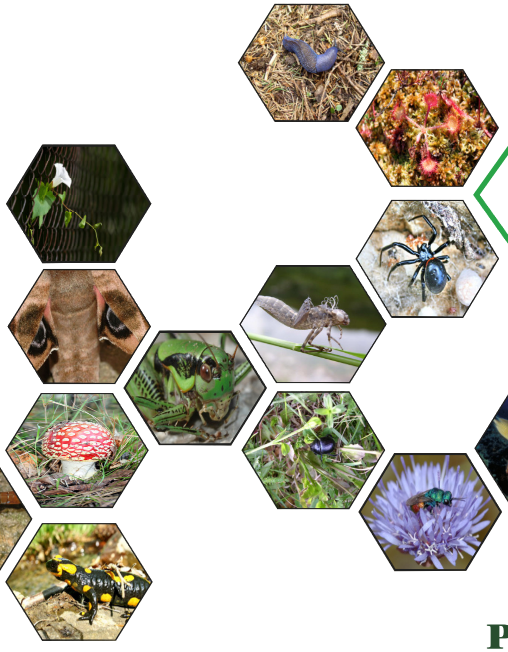

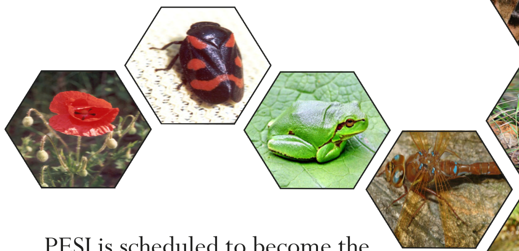

PESI is scheduled to become the ‘Euro-Hub’ of the Catalogue of Life. (a collective Species2000/ITIS initiative)

PESI will be the European taxonomic back-bone for LifeWatch.

PESI contributes to the Global Names Architecture development.

PESI will be further linked to ViBRANT’s the Ontology Platform and to Europe’s Bio-diversity virtual e-Laboratory (BioVel).

Photographs courtesy of Roy Kleukers, Marcin Penk, Eduard Stloukal, Davorin Tome, Mike Guiry, Fabio Rindi, Volodymyr Rizun, English Nature, ImagDOP, JG Marmelin.

Partners

Universiteit van Amsterdam (NL)
University of Copenhagen (DK)
Trakya University (TR)
Natural History Museum (GB)
Flanders Marine Institute (BE)
Cab International (GB)
Natural History Museum (FR)
Royal Botanical Gardens (GB)
Society of the Management of Electronic Biodiversity Data (IE)
International Commission for Zoological Nomenclature (GB)
Botanic Garden and Botanical Museum Berlin-Dahlem, Freie Universität Berlin (DE)
Ecological Consultancy Services Ltd. (IE)
Università degli studi di Palermo (IT)
Slovak Academy of Sciences (SK)
National University Athens (GR)
Museum of Natural History (NL)
University of Helsinki (FI)
University of Sevilla (ES)
Vilnius University (LT)

Comitato Scientifico per la Fauna d’Italia(IT)
Museum of Natural History (SE)
Comenius University in Bratislava (SK)
University of Science & Technology (NO)
National Academy of Sciences (UA)
Polish Academy of Sciences (PL)
Swiss Systematics Society (CH)
Ilia Chavchavadze State University (GE)
Consejo Superior de Investigaciones Cientificas (ES)
Israel Oceanographic & Limnological Research Ltd. (IL)
Polish Academy of Sciences Institute of Oceanology (PL)
Museum of Natural History (BG)
Asociatia Mynature (RO)
University of Latvia (LV)
Hellenic Centre for Marine Research (GR)
Université des Sciences et Technologies de Lille (FR)
National Institute of Biology (SL)
Russian Academy of Sciences (RU)
National Academy of Sciences (UA)
Marine Biological Association (UK)

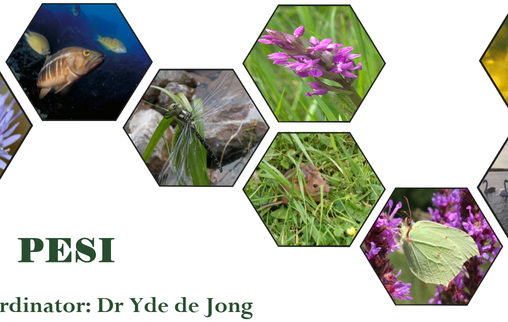

PESI

General Coordinator: Dr Yde de Jong

Netherlands Centre for Biodiversity Naturalis
(Zoological Museum Amsterdam)
University of Amsterdam
PO Box 94766
1090 GT Amsterdam, The Netherlands

www.eu-nomen.eu/portal.

Phone: +31 20 525 7191
Fax: +31 20 525 7780

Contract no. RI-223806
Period 2008-2011

CAPACITIES

Funded by the European Union 7th Framework Programme, Activity Area Capacities, Research Infrastructures programme.

PESI
EU-NOMEN

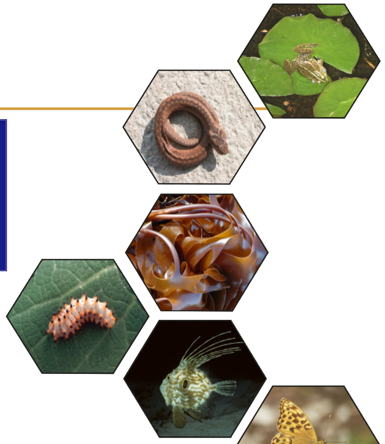

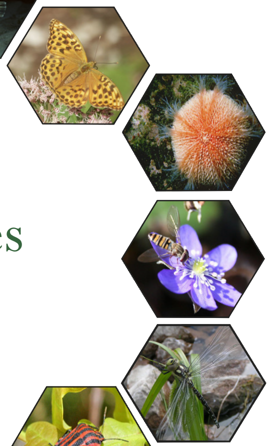

A Pan-European Species-directories Infrastructure

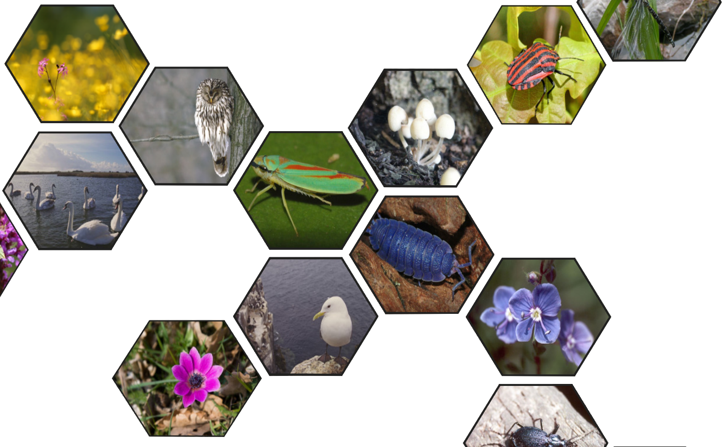

THE BACKBONE OF EUROPE’S BIODIVERSITY MANAGEMENT

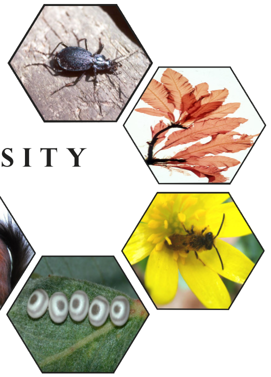

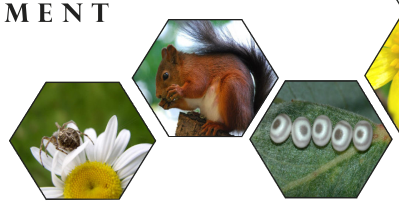

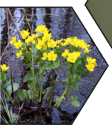
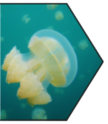
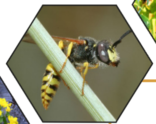
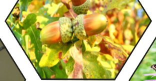
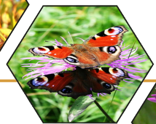
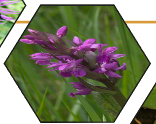
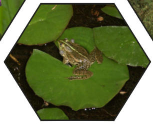
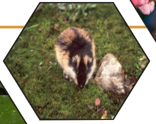
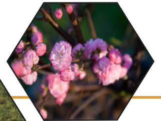

Europe's taxonomic e-infrastructure

PESI is Europe's e-infrastructure for taxonomic information on species occurring in Europe. PESI has successfully integrated the infrastructural components of five major community networks on taxonomic indexing and their respective knowledge infrastructures, i.e. marine life, terrestrial plants, fungi and animals.

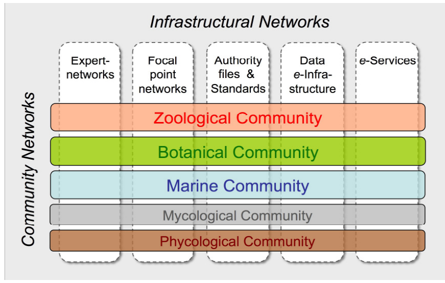

PESI successfully integrated the four main European authoritative all-taxon name registers in coordination with EU based nomenclators and major Species Databases into a standards-based, quality-controlled, expertly-validated, open-access infrastructure for research, education and data management.

PESI allows cross-linking to major biodiversity information services (IUCN, GBIF, CITES, EPPO, EU Habitat Directive/Birds Directive) and contains persistent identifiers; standardised vocabularies and exchange formats.

Data integration

In co-operation with other biodiversity informatics initiatives (TDWG, GBIF), PESI has defined a strategy for assigning persistent identifiers to names and taxa networked by PESI.

PESI Web Portal

The portal integrates major European check-lists: Fauna Europaea (FaEu), Euro+Med Plantbase (E+M), European Register of Marine Species (ERMS) and the EU component of Index Fungorum (IF). Still operating separately the register's data however is merged yearly in the PESI Data Warehouse and is available through a single portal at www.eu-nomen.eu/portal.

Almost a half a million scientific names are contained in this database:211,638 valid species; 150,000 synonyms; and 6,294,659 distribution records . PESI has also become a major resource for non-scientific names of species with 132,616 common names (88 languages).

In addition to taxonomic information, PESI harvests information on species (images, literature, conservation status and provides links to other portals (e.g. national check-lists, red species lists) and other bioinformatics databases such as GenBank sequence database.

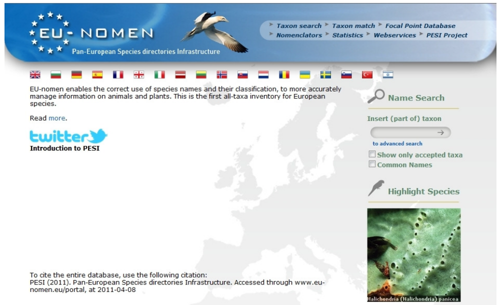

Data validation

The PESI portal provides an intelligent name validation service and web services to cross-match external species lists against names in PESI (of the so called TAXAMATCH tool). This enables the end user to standardize species names and promotes the use of the persistent identifiers generated by the different checklists.

The Focal Point Network

PESI Focal Points (FP) are national- representatives for biodiversity and taxonomic research. There are 49 Focal Points from 38 countries.

The focal point network is crucial for the information exchange and data standards for the Pan-European (sensu latu) taxonomic infrastructure, forming the communication channel between the local level knowledge resources and the Pan-European integrated Taxonomic community network.

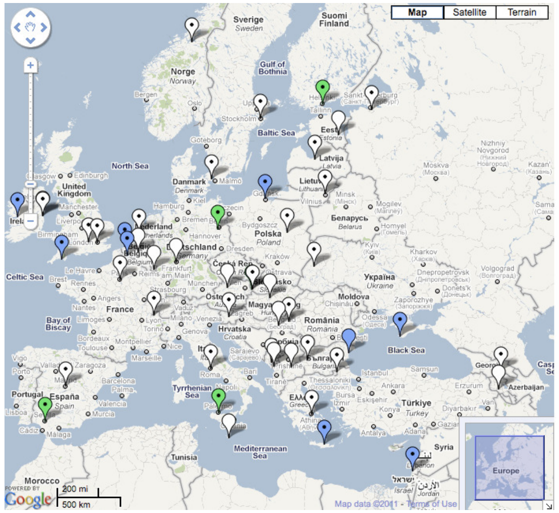

Focal Points promote taxonomic standards throughout Europe. This is of direct importance for both European and national government bodies concerned with the implementation of legislation which refers to species and habitats.

Translations by Focal Points has resulted in multilingual portal pages being developed to support a wider audience and user base.

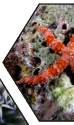
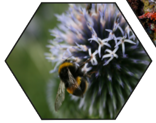
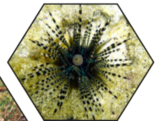
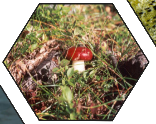
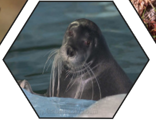
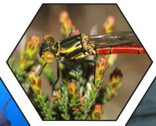
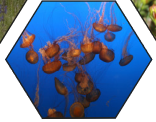
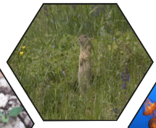
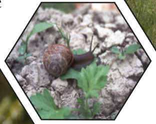
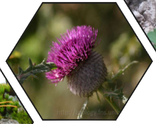
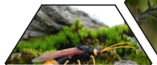

Supplement: Supplementary material 33 — PESI Flyer [file biodiversity_data_journal-3-e5848-s033.pdf]
