# Supplementary material for: PESI - a taxonomic backbone for Europe
Source: Biodivers Data J. 2015 Sep 28;(3):e5848. doi: 10.3897/BDJ.3.e5848 (PMC4609752; doi:10.3897/BDJ.3.e5848)
Supplement: Supplementary material 34 — PESI Focal Point Network description [file biodiversity_data_journal-3-e5848-s034.pdf]

PESI Focal Points Network

Country / Region	Organisation	Contact	Role
Albania	University of Shkodra, Faculty of Natural Sciences (US)	Prof. Dhimiter Dhora	FAUNA EUROPAEA
Armenia	National Academy of Sciences of Armenia, Institute of Zoology (NASA)	Prof. Hasmik Khachatryan	FAUNA EUROPAEA
Austria	Oberösterreichisches Landesmuseum (OOE.BZ)	Dr. Michael Malicky	FAUNA EUROPAEA
Azerbaijan	Institute of Zoology - Baku	Prof. Shaig Ibrahimov	FAUNA EUROPAEA
Belarus	United Institute of Informatics Problems National Academy of Sciences of Belarus	Prof. Alexander Tuzikov	FAUNA EUROPAEA
Belgium	Belgian Biodiversity Platform (BBPF)	Dr. Hendrik Segers (past), Dr. Aaike de Wever (now)	FAUNA EUROPAEA
Belgium	Flanders Marine Institute (VLIZ)	Dr. Ward Appeltans (past), Dr. Leen Vandepitte (now)	ERMS
Bulgaria	Institute of Oceanology (IO-Bas) – Varna	Dr. Snejana Moncheva	ERMS
Bulgaria	National Museum of Natural History - Sofia (NMNHS)	Prof. Zlatozar Boev & Prof. Alexi Popov (past), prof. Nikolai Spassov (now)	FAUNA EUROPAEA
Croatia	State Institute for Nature Protection (SINP)	Dr. Igor Boršić	FAUNA EUROPAEA
Cyprus	Department of Biological Sciences, University of Cyprus	Dr. Nicos Seraphides (past), Prof. Spyros Sfendourakis (now)	FAUNA EUROPAEA
Czech	Nature Conservation Agency CZ	Dr. Tomás Soldán (past),Mgr. Karel Chobot (now)	FAUNA EUROPAEA
Denmark	University of Copenhagen (UCPH)	Dr. Isabel Calabuig (past), vacant (now)	FAUNA EUROPAEA
Estonia	University of Tartu – Tartu	Dr. Marti Martin (past), Prof. Urmas Kõljalg (now)	FAUNA EUROPAEA
Finland	Zoological Museum of the Finnish Museum of Natural History	Juhani Lokki (past), vacant (now)	FAUNA EUROPAEA
Finland	Finnish Museum of Natural History, University of Helsinki	Prof. Pertti Uotila	EURO+MED PlantBase
France	Muséum National d'Histoire Naturelle (MNHN)	Dr. Olivier Gargominy	FAUNA EUROPAEA
France	University of Sciences and Technology of Lille (USTL)	Prof. Jean-Claude Dauvin	ERMS
Georgia	Ilia Chavchavadze State University (ILIAUNI)	Prof. David Tarkhnishvili & Dr. Giorgi Chaladze	FAUNA EUROPAEA
Germany	Botanic Garden and Botanical Museum Berlin-Dahlem, Freie Universität Berlin	Prof. Walter Berendsohn	EURO+MED PlantBase
Germany	Senckenberg Museum – Frankfurt	Dr. Michael Tuerkay	FAUNA EUROPAEA
Greece	National and Kapodistrian University of Athens (NKUA)	Dr. Anastasios Legakis	FAUNA EUROPAEA
Greece	Hellenic Centre for Marine Research (HCMR)	Dr. Christos Arvanitidis	ERMS
Hungary	Hungarian Natural History Museum – Budapest	Dr. László Peregovits	FAUNA EUROPAEA
Iceland	Icelandic Institute of Natural History	Dr. Snorri Baldursson (past), Dr. Gudmundur Gudmundsson & Dr. Erling Ólafsson (now)	FAUNA EUROPAEA
Ireland	National University of Ireland (NUIG)	Dr. Róisín Nash (past), Dr. Liam Lysaght & Dr. Eugenie Regan (now)	FAUNA EUROPAEA
Ireland	National University of Ireland (NUIG)	Prof. Michael Guiry	ERMS
Israel	Israel Oceanographic and Limnological Research (IOLR)	Prof. Bella Sarah Galil	ERMS
Italy	University Palermo, Department Botanical Sciences (DPCE)	Dr. Francesco M. Raimondo & Gianniantonio Domina	EURO+MED PlantBase
Italy	Scientific Committee for the Italian Fauna (CSFI)	Dr. Fabio Stoch	FAUNA EUROPAEA

Latvia	University of Latvia (LU)	Dr. Voldemars Spungis & Dr. Arvids Barsevskis	FAUNA EUROPAEA
Lithuania	Nature Research Centre (EKOI)	Prof. Eduardas Budrys & Prof. Sergej Olenin	FAUNA EUROPAEA
Luxembourg	Musée national d'histoire naturelle – Luxembourg	Dr. Marc Meyer & Dr. Edmée Engel (past), Dr. Armand Turpel & Dr. Tania Walisch (now)	FAUNA EUROPAEA
Macedonia (FYROM)	Macedonian Museum of Natural History (MMNH)	Prof. Vladimir Krpach	FAUNA EUROPAEA
Malta	Malta Environment & Planning Authority – Floriana	Prof. Patrick J. Schembri (past), Dr. Marie Therese Gambin (now)	FAUNA EUROPAEA
Moldavia	Institute of Zoology of the Academy of Sciences of Moldova	Dr. Laurentia Ungureanu	FAUNA EUROPAEA
Montenegrin	Montenegrin Academy of Sciences and Arts (MASA)	Prof. Gordan Karaman	FAUNA EUROPAEA
Netherlands	Naturalis Biodiversity Center (NBC)	Dr. Roy Kleukers	FAUNA EUROPAEA
Norway	Norwegian Biodiversity Information Centre (Artsdatabanken)	Dr. Kaare Aagaard & Elisabeth Stur (past), Dr. Nils Valland & Dr. Toril Loennechen Moen (now)	FAUNA EUROPAEA
Poland	University of Warsaw, Faculty of Biology, Dept. of Ecology	Prof. Wieslaw Bogdanowicz (past), Dr. Piotr Tykarski (now)	FAUNA EUROPAEA
Poland	Institute of Oceanology of Polish Academy of Sciences (IOPAN)	Prof. Jan Marcin Węsławski & Dr. Monika Kędra	ERMS
Portugal - Azores	Azorean Biodiversity Group and Universidade dos Açores	Prof. Paulo Borges & Dr. António Manuel de Frias Martins	FAUNA EUROPAEA
Portugal - Macarones	Estação de Biologia Marinha do Funchal – Madeira	Prof. António Domingos Abreu	FAUNA EUROPAEA
Portugal - mainland	Naturdata	Dr. Pedro Cardoso (past), Dr. Ricardo Silva (now)	FAUNA EUROPAEA
Romania	myNature Association (myNA)	Dr. Oana Moldovan (past), Dr. Adorian Ardelean (now)	FAUNA EUROPAEA
Russia	Zoological Institute of Russian Academy of Sciences (ZIN RAS)	Dr. Sergei Medvedev & Dr. Alexander Ryss	FAUNA EUROPAEA
Serbia	Centre for the Balkan Biodiversity Conservation (CBBC)	Prof. Smiljka Šimić	EURO MED PlantBase
Slovakia	Institute of Botany, Slovak Academy of Sciences (IBSAS)	Prof. Karol Marhold	EURO MED PlantBase
Slovakia	Comenius University Bratislava (CUB)	Dr. Eduard Stloukal	FAUNA EUROPAEA
Slovenia	Slovenian National Institute of Biology (NIB)	Dr. Davorin Tome	FAUNA EUROPAEA
Spain	Consejo Superior de Investigaciones Cientificas (CSIC)	Dr. Marian Ramos	FAUNA EUROPAEA
Spain	University of Seville (USE)	Prof. Benito Valdés & Dr. Francisco Pina	EURO MED PlantBase
Sweden	Swedish Museum of Natural History (NRM)	Dr. Sven Kullander (past), Dr. Anders Telenius (now)	FAUNA EUROPAEA
Switzerland	Swiss Systematics Society (past), Centre Suisse de Cartographie de la Faune (now)	Dr. Jean Mariaux (past), Dr. Yves Gonseth & Dr. Pascal Tschudin (now)	FAUNA EUROPAEA
Turkey	University of Trakya (TU)	Prof. Nihat Aktac	FAUNA EUROPAEA
Ukraine	A.O.Kovalevsky Institute of Biology of the Southern Seas (IBSS)	Prof. Volodymyr Vladymyrov & Dr. Oleksandra Sergeyeva	ERMS
Ukraine	State Museum of Natural History, Nat. Ac. of Sciences of Ukraine (SMNH)	Dr. Volodymyr Rizun	FAUNA EUROPAEA
United Kingdom	Natural History Museum (NHM)	Dr. Charles Hussey (past), Dr. Chris Rapper (now)	FAUNA EUROPAEA
United Kingdom	Marine Biological Association of the United Kingdom (MBA)	Dr. Dan Lear	ERMS

PESI project partners as well as associated partners are included in this list. For details about the Focal Points information services, please check the relevant Focal Points deliverables (D3.1 – FP Work-plan and D3.4 – FP Handbook).
