# Supplementary figures and images for: PESI - a taxonomic backbone for Europe
Source: Biodivers Data J. 2015 Sep 28;(3):e5848. doi: 10.3897/BDJ.3.e5848 (PMC4609752; doi:10.3897/BDJ.3.e5848)

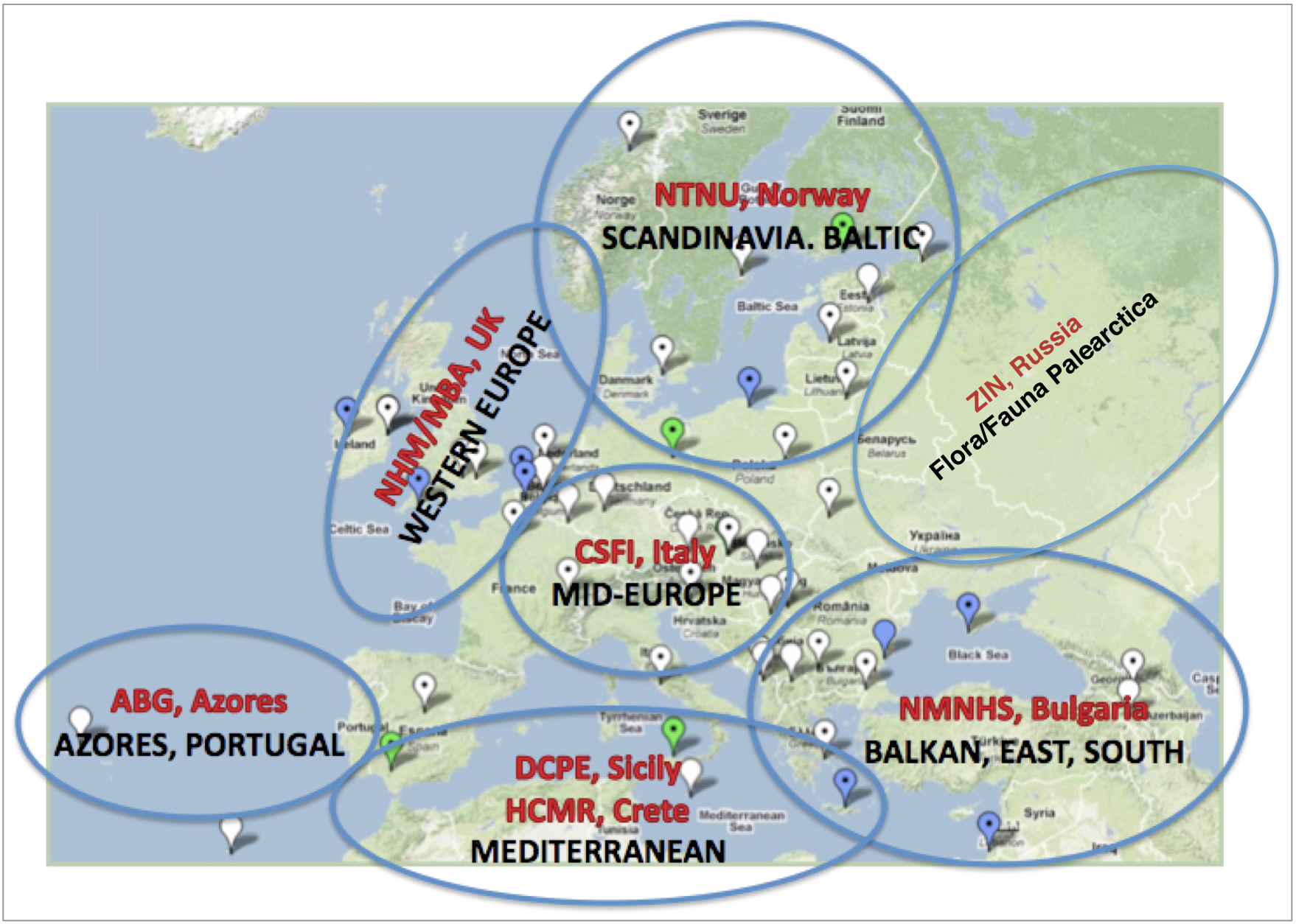

Supplement: Supplementary material 37 — Coordination framework for grouped PESI focal points [file biodiversity_data_journal-3-e5848-s037.png]
